# Associations between the probabilities of frequency-specific hearing loss and unaided APHAB scores

**DOI:** 10.1007/s00405-016-4385-7

**Published:** 2016-11-17

**Authors:** J. Löhler, B. Wollenberg, P. Schlattmann, N. Hoang, R. Schönweiler

**Affiliations:** 1 Scientific Institute for Applied ENT-Research of the German Professional Association of ENT-Surgeons, Maienbeeck 1, 24576 Bad Bramstedt, Germany; 2 Department of ENT-Surgery, Universital Hospital Schleswig–Holstein, Campus Luebeck, Lübeck, Germany; 3 Institute for Medical Statistics, Informatics and Documentations, Universital Hospital Jena, Campus Luebeck, Jena, Germany; 4 Section of Phoniatrics and Pedaudiology in the Department of ENT-Surgery, Universital Hospital of Schleswig-Holstein, Campus Luebeck, Lübeck, Germany

**Keywords:** Hearing loss, APHAB, Unaided APHAB, Hearing aid fitting, Frequency-specific hearing loss, Probability of hearing loss

## Abstract

The Abbreviated Profile of Hearing Aid Benefit (APHAB) questionnaire reports subjective hearing impairments in four typical conditions. We investigated the association between the frequency-specific probability of hearing loss and scores from the unaided APHAB (APHAB_u_) to determine whether the APHAB_u_ could be useful in primary diagnoses of hearing loss, in addition to pure tone and speech audiometry. This retrospective study included database records from 6558 patients (average age 69.0 years). We employed a multivariate generalised linear mixed model to analyse the probabilities of hearing losses (severity range 20–75 dB, evaluated in 5-dB steps), measured at different frequencies (0.5, 1.0, 2.0, 4.0, and 8.0 kHz), for nearly all combinations of APHAB_u_ subscale scores (subscale scores from 20 to 80%, evaluated in steps of 5%). We calculated the probability of hearing loss for 28,561 different combinations of APHAB_u_ subscale scores (results available online). In general, the probability of hearing loss was positively associated with the combined APHAB_u_ score (i.e. increasing probability with increasing scores). However, this association was negative at one frequency (8 kHz). The highest probabilities were for a hearing loss of 45 dB at test frequency 2.0 kHz, but with a wide spreading. We showed that the APHAB_u_ subscale scores were associated with the probability of hearing loss measured with audiometry. This information could enrich the expert’s evaluation of the subject’s hearing loss, and it might help resolve suspicious cases of aggravation. The 0.5 and 8.0 kHz frequencies influenced hearing loss less than the frequencies in-between, and 2.0 kHz was most influential on intermediate degree hearing loss (around 45 dB), which corresponded to the frequency-dependence of speech intelligibility measured with speech audiometry.

**Electronic supplementary material:**

The online version of this article (doi:10.1007/s00405-016-4385-7) contains supplementary material, which is available to authorized users.

## Introduction

In audiology, primary diagnoses of hearing loss are based on pure tone and speech audiometry and on the results of self-reporting questionnaires. Audiometry provides a more or less objective measurement, and questionnaire scores give a subjective evaluation of impairments in hearing.

The Abbreviated Profile of Hearing Aid Benefit (APHAB), developed by Cox and Alexander in 1995, consists of 24 questions grouped into four subscales that represent everyday hearing situations [1, 2], as follows. First, the ease of communication (EC) scale represents basic hearing situations in a quiet environment without ambient noise; second, the background noise (BN) scale represents hearing situations with background noises; third, the reverberation (RV) scale represents hearing situations in large spaces with echoes; and fourth, the aversiveness of sounds (AV) scale measures the perception of loud events. The EC, BN, and RV subscales evaluate difficulty in understanding sounds in the situations described. In contrast, the AV subscale measures the recruitment phenomenon or hyperacusis. In 2010 and 2012, investigations on the APHAB were carried out with German-speaking patients; those studies showed no difference in the distribution from the specified US norms [3, 4]. Starting in April 2012, the APHAB became an integral part of the resources policy in Germany [5, 6]; therefore, it the most commonly used inventory in the context of diagnostics for patients with state-required insurance, which includes about 90% of the German population. Accordingly, the APHAB has the most important role under all hearing loss measuring inventories in Germany. The APHAB actually exists in 21 different languages. Differences in interpretations are not known.

The first part of the APHAB is used for primary diagnoses of hearing loss; it is designated the unaided APHAB (APHAB_u_), because hearing problems are assessed without hearing aids. Hearing aids can be subsequently fitted, but there is no need for a second examination. That is, it is not necessary to evaluate differences in scoring between aided and unaided self-perception. Therefore, the APHAB_u_ must be given to patients with impaired hearing at an early stage on the diagnostic pathway, along with pure tone and speech audiometry.

Recently, it was demonstrated that there was no dependency on hearing loss represented by seven standard types of audiograms and the APHAB_u_ [7]. One the other hand, a second investigation showed that frequency-dependent hearing losses were related to some of the APHAB_u_ subscale scores [8]. Significant correlations between hearing loss and APHAB_u_ scores were found at frequencies above 0.5 kHz for the EC subscale, at all frequencies for the RV subscale, and at 1.0 and 2.0 kHz for the AV subscale. At these frequencies, for each decibel (dB) of hearing loss, the EC and RV APHAB_u_ scores increased by approximately 0.2 percentage points, and the AV subscale scores decreased by 0.1 percentage point. No correlation was found between the degree of hearing loss and the BN subscale score. This can be explained by the individual ability for hearing loss compensation. Hearing losses at defined single frequencies can influence the APHAB_u_ scores. Neither of those studies demonstrated any relationship between gender, hearing loss, and the APHAB_u_ score.

These findings due to the opposite question: is the frequency-specific hearing loss dependent on APHAB_u_ scores and how are the belonging probabilities for each subscale? The aim of this study is to provide a detailed investigation of correlations between the APHAB scores and the probability of having a frequency-dependent hearing loss. In addition, we investigated the probabilities of frequency-dependent hearing loss (with severity grouped in steps of 5 dB) at all octave frequencies, between 0.5 and 8 kHz.

## Methods

An APHAB database was established in Germany several years ago [9]. At present, this database contains APHAB records and associated audiograms from thousands of individuals that were examined at more than 90 ENT clinics and practices. This database contained 7199 records of patients with impaired hearing on 23 January 2016. Records were collected, both with an online method and with paper-and-pencil records, which were later entered into the database through internet-based access. All data were stored on a central server. In all cases of subsequent hearing aid fitting, the first part of the APHAB (i.e. the APHAB_u_) was given to the subject before fitting the hearing aid. Thus, the APHAB was used as a primary diagnostic tool in evaluating hearing loss, as described previously [7, 8]. In addition to individual APHAB results, the database also contained the associated pure-tone audiogram data (octave frequencies between 0.5 and 8.0 kHz). The database did not include records for patients with a difference in hearing loss greater than 60 dB between the right and left ears, evaluated with the air conduction testing at frequencies at 0.5, 1.0, and 2.0 kHz, based on the three-frequency table (Table 1, [3, 4]). This exclusion avoided any influence of compensating effects, in cases of severe hearing loss asymmetry [7, 8]. Furthermore, we eliminated records from the study, when the data were incomplete for the calculations involved in this study.

**Table 1:** Three-frequency table used to define the degree of hearing impairment [3, 4]

	Hearing loss at 2.0 kHz (dB)				
	<20	20–35	40–55	60–80	>80
Total hearing loss at 0.5 and 1.0 kHz (dB)					
0–35	None	Slight	Moderate	Moderate–profound	Profound
40–75	Slight	Slight	Moderate	Moderate–profound	Profound
80–115	Moderate	Moderate	Moderate	Moderate–profound	Profound
120–160	Moderate–profound	Moderate–profound	Moderate–profound	Moderate–profound	Profound
>160	Profound	Profound	Profound	Profound	Profound

Findings are from the sound audiogram of the ear with worse hearing, measured in 5-dB steps. Subjects with a difference >60 dB hearing loss between the left and the right ears were initially excluded from the database

We employed a multivariate generalised linear mixed model (i.e. logistic regression with random effects [10]) to investigate the probabilities of frequency-specific hearing losses (20–75 dB hearing loss, divided into groups of 5-dB steps), within groups with different average APHAB_u_ scores for the four subscales (EC, BN, RV, and AV). The APHAB_u_ was administered with air conduction tests at sound frequencies of 0.5, 1.0, 2.0, 4.0, and 8.0 kHz. The average APHAB_u_ scores for each subscale were sorted into groups that increased in steps of 5 percentage points. Thus, the APHAB_u_ scores were the independent variables, and the frequency-specific audiogram results were the dependent variables. Another independent variable was gender. Calculations were performed with SAS software, version 9.4, proc glimmix. All results are presented in tables with four dimensions (or levels), as follows: first level, the average scores for each of the four APHAB_u_ subscales (EC, BN, RV, AV); second level, fixed combinations of the four APHAB_u_ subscale values; third level, sound frequency (values from 0.5 to 8 kHz); and fourth level, the probability that hearing loss was associated with a given APHAB_u_ subscale combination, at each sound frequency. Because the APHAB yields a vast number of possible results (each subscale contains six questions, each question is scored 1–99%), we presented the data in tables that show the average score for each APHAB_u_ subscale, grouped in steps of 5%, for average scores of 20–80%. For better understanding, we created a series of graphs showing the probabilities that a given level of hearing loss will occur at each frequency for all combinations of the APHAB_u_ subscales (Figs. 1a–l). In addition, these figures were linked together in an animated slide show (Film 1, Online Resource 25).

Permission to store data was given voluntarily by all subjects included. The study was approved by the Ethics Committee of the Schleswig–Holstein Medical Association and the State Data Protection Officer.

## Results

### General data

We evaluated records of 7199 subjects, with an average age of 69.6 years [standard deviation (SD) ± 17.0 years]. Of these, 3081 were men (42.8%, average age 69.0 ± 16.4 years) and 4118 were women (57.2%, average age 70.1 ± 17.4 years). Some cases (*n* = 641) were not assigned to any hearing loss group, based on the three-frequency table (Table 1, [3, 4]), because the required data were incomplete for frequencies of 0.5, 1.0, and/or 2.0 kHz. These cases were not included in further assessments; thus, at least 6558 cases were available for analysis. For this study, the number of subjects that were subsequently fitted with hearing aids was irrelevant; therefore, it was not documented.

We investigated 6558 cases with a multivariate generalised linear mixed model to determine the probabilities that frequency-specific hearing losses measured at frequencies of 0.5, 1.0, 2.0, 4.0, and 8.0 kHz were associated with different combinations of APHAB_u_ values (combinations of all four APHAB subscales, EC, BN, RV, and AV, grouped in 5-percentage point steps, hearing loss severity: 20–75 dB, analysed in 5-dB steps). We found no differences in the probabilities of hearing loss between men and women. The distribution of records among different classes of hearing loss based on the three-frequency table (Table 1) is presented in Table 2. Our analyses generated 12 tables (Tables 3a–l), each with 28,561 different combinations of APHAB_u_ subscale values. We have presented the results online (Online Resource 1–12).

**Table 2:** Distribution of hearing loss categories, based on the definitions in Table 1, among subjects in this study (*n* = 7199)

Degree of hearing loss	Percent (%)	Number
None	15.9	1146
Slight	9.5	682
Moderate	32.3	2325
Moderate-profound	27.5	1978
Profound	5.9	427
Non-attributable^a^	8.9	641

^a^641 subjects had data that were non-attributable or incomplete, and these patients were excluded from further investigation

### Associations between the probability of frequency-specific hearing loss and unaided APHAB scores

We found that, in general, higher scores in the EC, BN, and RV subscales of the APHAB_u_ were associated with higher probabilities of hearing loss (Tables 3a–l). However, increasing hearing loss severity corresponded to decreasing values of probability. The probability of hearing loss associated with an APHAB_u_ score depended on the specific dB-value. However, the probability was lower for the lower frequencies and higher for the higher frequencies, except at 8.0 kHz, for all APHAB subscales in general.

Some interesting details emerged from this analysis. We found that the APHAB_u_-dependent probability of a 20-dB hearing loss was higher for 4.0 than for 8.0 kHz sounds. Up to a 35-dB hearing loss, increasing EC values were associated with lower probabilities of a hearing loss at 4.0 and 8.0 kHz, but at a 45-dB hearing loss that negative association was only observed for 8.0 kHz sounds. For hearing losses above 60 dB, the probability of a hearing loss was higher for 8.0 kHz than for 4.0 kHz sounds. Above 70 dB, except at 8.0 kHz, the APHAB_u_ scores had no detectable influence on the probability of a hearing loss. In the second step of this evaluation (Figs. 1a–l, Online Resource 13–24), we plotted the probabilities of hearing losses of 20–75 dB (in increasing steps of 5 dB) against the corresponding combinations of all APHAB_u_ subscale scores. In the third step of this evaluation, we presented the alterations in probabilities as the dB values increased, by connecting these figures in an animated slide show (Animation 1, Online Resource 25).

The statistical dispersion of the probabilities for hearing loss at test frequencies 4.0 and 8.0 kHz is decreasing for a hearing loss from 20 dB to a value about 35–40 dB. The probabilities for hearing loss at the test frequency 2.0 kHz have a wide spreading for a possible middle-degree hearing loss around 45 dB. The probability of a high-degree hearing loss is highest at test frequencies 4.0 and 8.0 kHz. But the belonging probabilities found in our study are on a very low level in these cases.

## Discussion

Although investigations of the relationships between audiometric and self-reported measures began decades ago, some questions in this field remain unanswered. The association between the probability of hearing loss and questionnaire scores has only been investigated in evaluations of screening-instruments, like the HHIS-E or MAT [11, 12], to determine their specificity and sensitivity. The APHAB is a very detailed questionnaire, too large for use in screening approaches. However, the APHAB is considered highly important in the context of diagnostics for patients with state-required insurance in Germany, a population roughly comparable to patients that receive nationally funded health services in other countries.

In this study, we did not discover any significant differences between genders in how the probability of hearing loss was associated with APHAB_u_ scores. The similarity between genders, both in distribution and average age, was consistent with findings in previous studies [7, 8].

The tables generated by this study give the probabilities that certain frequency-specific hearing losses might occur in patients that show a specific range of APHAB_u_ values. Thus, the reader might use these original data to predict pure-tone thresholds in all octave frequencies between 0.5 and 8 kHz. In addition, Tables 3a–l (Online resource 1–12) might allow the reader to introduce the APHAB_u_ in diagnoses of subjects that receive statutory accident insurance. The original data could be used further to compare the results of the APHAB_u_ scores for each subscale to the probability of the corresponding hearing loss, measured with audiometry. This information could enrich the expert’s evaluation of the subject’s hearing loss, and it might help resolve suspicious cases of aggravation and simulation.

Moreover, comparing APHAB_u_ scores of the AV subscale and the probabilities of hearing loss, based on pure-tone thresholds, could be a method for measurement of subjective hyperacusis or recruitment phenomenon. High scores in the AV_u_ should correspond to a severe hearing loss, particularly at high frequencies, and our tables can provide the associated probabilities of hearing loss. Our results have demonstrated more or less stringent associations between a possible frequency-specific hearing loss and all APHAB_u_ subscale scores. However, these probabilities are not equal at all frequencies, and the frequency-specific probability of hearing loss varies for different degrees of hearing loss. The probabilities we presented (Tables 3a–l, Online Resource 1–12) might enhance our understanding of the association between subjective speech intelligibility and hearing loss. Clearly, hearing losses in 0.5 and 8.0 kHz sounds are less possible than for sounds at the frequencies in-between, and the importance of 2.0 kHz sound was highest for a probability of an intermediate degree of hearing loss (around 45 dB). These findings corresponded to the frequency-specific dependency of speech intelligibility measured with speech audiometry (reviewed in [13]).

We found that high frequencies had limited influence, and frequencies below 4.0 kHz completely lacked influence on the probability of hearing losses of 65 dB and above. This finding could be explained by two components. First, only a relatively few number of subjects (5.9%) had profound hearing loss (Table 2). Thus, in general, the probability of a severe hearing loss was low in the investigated population. Moreover, clearly, some patients reported a severe subjective hearing impairment on the APHAB_u_, and a sharp threshold slope (dead region) was observed at high frequencies. This result pointed out the importance of high frequencies for speech intelligibility. In these cases, the score for the EC_u_ subscale had little or no influence on the probability of the associated hearing loss. The widespread dispersion of the influences of 4.0 and 8.0 kHz on a mild hearing loss and the influence of 2.0 kHz on a moderate hearing loss could also be explained this effect.

This study provided additional information on how the APHAB_u_ could be used by ENT surgeons and audiologists in primary measurements. In addition, the APHAB_u_ could be used as a method for measuring the benefit of hearing aids after fitting and for diagnosing subjects that receive statutory accident insurance. Due to the wide use of the APHAB in over 90% of all severe cases of hearing loss in Germany, future studies should supplement these results with determinations of the sensitivity and specificity of the APHAB.

In a broader view, a future fourth step of evaluation could be developed. The data we provided could be compiled in a computer program or a smart phone application that could calculate the predictions of different types and degrees of hearing loss, based on the patient’s answers on the APHAB questionnaire. An app like this would provide general practitioners a forecast of their patients’ hearing loss and indicate the urgency of scheduling an examination by an ENT specialist and/or audiologist.

## Electronic supplementary material

Below is the link to the electronic supplementary material.

Supplementary material 1 (PDF 24,536 kb)

Supplementary material 2 (PDF 3029 kb)

Supplementary material 3 (PDF 3035 kb)

Supplementary material 4 (PDF 3033 kb)

Supplementary material 5 (PDF 3045 kb)

Supplementary material 6 (PDF 3022 kb)

Supplementary material 7 (PDF 3009 kb)

Supplementary material 8 (PDF 3032 kb)

Supplementary material 9 (PDF 3030 kb)

Supplementary material 10 (PDF 3017 kb)

Supplementary material 11 (PDF 2969 kb)

Supplementary material 12 (PDF 2946 kb)

Supplementary material 13 (JPEG 149 kb)

Supplementary material 14 (JPEG 170 kb)

Supplementary material 15 (JPEG 179 kb)

Supplementary material 16 (JPEG 186 kb)

Supplementary material 17 (JPEG 177 kb)

Supplementary material 18 (JPEG 174 kb)

Supplementary material 19 (JPEG 179 kb)

Supplementary material 20 (JPEG 179 kb)

Supplementary material 21 (JPEG 162 kb)

Supplementary material 22 (JPEG 150 kb)

Supplementary material 23 (JPEG 149 kb)

Supplementary material 24 (JPEG 149 kb)

Supplementary material 25 (MP4 30,120 kb)
