# Supplementary material for: Associations between the probabilities of frequency-specific hearing loss and unaided APHAB scores
Source: Eur Arch Otorhinolaryngol. 2016 Nov 17;274(3):1345–9. doi: 10.1007/s00405-016-4385-7 (PMC5309283; doi:10.1007/s00405-016-4385-7)
Supplement: Supplementary file 1 — Supplementary material 1 (PDF 24,536 kb) [file 405_2016_4385_MOESM1_ESM.pdf]

Number	EC	BN	RV	AV	0.5 kHz	1.0 kHz	2.0 kHz	4.0 kHz	8.0 kHz
1	20	20	20	20	60,3%	66,3%	76,6%	79,3%	71,7%
2	20	20	20	25	60,7%	66,0%	77,2%	80,5%	73,1%
3	20	20	20	30	61,2%	65,7%	77,8%	81,5%	74,4%
4	20	20	20	35	61,6%	65,4%	78,4%	82,5%	75,7%
5	20	20	20	40	62,0%	65,1%	79,0%	83,5%	76,9%
6	20	20	20	45	62,4%	64,8%	79,5%	84,4%	78,1%
7	20	20	20	50	62,8%	64,5%	80,1%	85,3%	79,2%
8	20	20	20	55	63,2%	64,2%	80,6%	86,2%	80,3%
9	20	20	20	60	63,6%	63,9%	81,1%	87,0%	81,4%
10	20	20	20	65	64,0%	63,6%	81,7%	87,7%	82,4%
11	20	20	20	70	64,5%	63,3%	82,2%	88,4%	83,4%
12	20	20	20	75	64,9%	63,0%	82,7%	89,1%	84,3%
13	20	20	20	80	65,3%	62,7%	83,1%	89,8%	85,2%
14	20	20	25	20	54,3%	61,0%	74,3%	78,3%	70,8%
15	20	20	25	25	54,7%	60,6%	75,0%	79,5%	72,2%
16	20	20	25	30	55,2%	60,3%	75,6%	80,6%	73,6%
17	20	20	25	35	55,6%	60,0%	76,2%	81,6%	74,9%
18	20	20	25	40	56,0%	59,7%	76,8%	82,6%	76,2%
19	20	20	25	45	56,5%	59,4%	77,4%	83,6%	77,4%
20	20	20	25	50	56,9%	59,1%	78,0%	84,5%	78,6%
21	20	20	25	55	57,3%	58,8%	78,6%	85,4%	79,7%
22	20	20	25	60	57,8%	58,4%	79,2%	86,3%	80,8%
23	20	20	25	65	58,2%	58,1%	79,7%	87,1%	81,8%
24	20	20	25	70	58,6%	57,8%	80,3%	87,8%	82,8%
25	20	20	25	75	59,0%	57,5%	80,8%	88,5%	83,8%
26	20	20	25	80	59,5%	57,2%	81,3%	89,2%	84,7%
27	20	20	30	20	48,1%	55,3%	71,9%	77,3%	70,0%
28	20	20	30	25	48,6%	55,0%	72,6%	78,5%	71,4%
29	20	20	30	30	49,0%	54,7%	73,2%	79,6%	72,8%
30	20	20	30	35	49,4%	54,4%	73,9%	80,7%	74,1%
31	20	20	30	40	49,9%	54,0%	74,6%	81,8%	75,4%
32	20	20	30	45	50,3%	53,7%	75,2%	82,8%	76,7%

33	20	20	30	50	50,8%	53,4%	75,8%	83,7%	77,9%
34	20	20	30	55	51,2%	53,1%	76,4%	84,6%	79,0%
35	20	20	30	60	51,6%	52,7%	77,0%	85,5%	80,1%
36	20	20	30	65	52,1%	52,4%	77,6%	86,4%	81,2%
37	20	20	30	70	52,5%	52,1%	78,2%	87,1%	82,2%
38	20	20	30	75	53,0%	51,8%	78,8%	87,9%	83,2%
39	20	20	30	80	53,4%	51,4%	79,4%	88,6%	84,1%
40	20	20	35	20	42,0%	49,6%	69,3%	76,2%	69,1%
41	20	20	35	25	42,5%	49,3%	70,0%	77,4%	70,5%
42	20	20	35	30	42,9%	48,9%	70,7%	78,6%	72,0%
43	20	20	35	35	43,3%	48,6%	71,4%	79,7%	73,3%
44	20	20	35	40	43,7%	48,3%	72,1%	80,8%	74,6%
45	20	20	35	45	44,2%	48,0%	72,8%	81,9%	75,9%
46	20	20	35	50	44,6%	47,6%	73,5%	82,9%	77,1%
47	20	20	35	55	45,1%	47,3%	74,1%	83,8%	78,3%
48	20	20	35	60	45,5%	47,0%	74,8%	84,7%	79,5%
49	20	20	35	65	45,9%	46,7%	75,4%	85,6%	80,6%
50	20	20	35	70	46,4%	46,3%	76,0%	86,4%	81,6%
51	20	20	35	75	46,8%	46,0%	76,6%	87,2%	82,6%
52	20	20	35	80	47,2%	45,7%	77,2%	88,0%	83,6%
53	20	20	40	20	36,2%	43,8%	66,6%	75,1%	68,2%
54	20	20	40	25	36,6%	43,5%	67,4%	76,3%	69,7%
55	20	20	40	30	37,0%	43,2%	68,1%	77,6%	71,1%
56	20	20	40	35	37,4%	42,9%	68,8%	78,7%	72,5%
57	20	20	40	40	37,8%	42,6%	69,6%	79,9%	73,8%
58	20	20	40	45	38,2%	42,2%	70,3%	81,0%	75,1%
59	20	20	40	50	38,6%	41,9%	71,0%	82,0%	76,4%
60	20	20	40	55	39,0%	41,6%	71,7%	83,0%	77,6%
61	20	20	40	60	39,5%	41,3%	72,4%	83,9%	78,8%
62	20	20	40	65	39,9%	41,0%	73,0%	84,8%	79,9%
63	20	20	40	70	40,3%	40,7%	73,7%	85,7%	81,0%
64	20	20	40	75	40,7%	40,3%	74,3%	86,5%	82,0%
65	20	20	40	80	41,2%	40,0%	75,0%	87,3%	83,0%

66	20	20	45	20	30,7%	38,3%	63,8%	73,9%	67,3%
67	20	20	45	25	31,0%	38,0%	64,6%	75,2%	68,8%
68	20	20	45	30	31,4%	37,7%	65,3%	76,5%	70,3%
69	20	20	45	35	31,8%	37,3%	66,1%	77,7%	71,7%
70	20	20	45	40	32,2%	37,0%	66,9%	78,9%	73,0%
71	20	20	45	45	32,6%	36,7%	67,6%	80,0%	74,4%
72	20	20	45	50	33,0%	36,4%	68,4%	81,1%	75,6%
73	20	20	45	55	33,4%	36,1%	69,1%	82,1%	76,9%
74	20	20	45	60	33,7%	35,8%	69,8%	83,1%	78,1%
75	20	20	45	65	34,1%	35,5%	70,5%	84,0%	79,2%
76	20	20	45	70	34,5%	35,2%	71,2%	85,0%	80,3%
77	20	20	45	75	34,9%	34,9%	71,9%	85,8%	81,4%
78	20	20	45	80	35,3%	34,6%	72,6%	86,6%	82,4%
79	20	20	50	20	25,7%	33,0%	60,9%	72,7%	66,4%
80	20	20	50	25	26,0%	32,7%	61,7%	74,1%	67,9%
81	20	20	50	30	26,4%	32,4%	62,5%	75,4%	69,4%
82	20	20	50	35	26,7%	32,1%	63,3%	76,6%	70,8%
83	20	20	50	40	27,0%	31,8%	64,1%	77,8%	72,2%
84	20	20	50	45	27,4%	31,5%	64,8%	79,0%	73,6%
85	20	20	50	50	27,8%	31,3%	65,6%	80,1%	74,9%
86	20	20	50	55	28,1%	31,0%	66,4%	81,2%	76,1%
87	20	20	50	60	28,5%	30,7%	67,1%	82,2%	77,4%
88	20	20	50	65	28,8%	30,4%	67,9%	83,2%	78,5%
89	20	20	50	70	29,2%	30,2%	68,6%	84,2%	79,7%
90	20	20	50	75	29,6%	29,9%	69,3%	85,1%	80,8%
91	20	20	50	80	29,9%	29,6%	70,0%	85,9%	81,8%
92	20	20	55	20	21,3%	28,1%	57,9%	71,5%	65,5%
93	20	20	55	25	21,6%	27,8%	58,7%	72,9%	67,0%
94	20	20	55	30	21,9%	27,6%	59,5%	74,2%	68,5%
95	20	20	55	35	22,2%	27,3%	60,3%	75,5%	70,0%
96	20	20	55	40	22,5%	27,0%	61,2%	76,8%	71,4%
97	20	20	55	45	22,8%	26,8%	62,0%	78,0%	72,8%
98	20	20	55	50	23,1%	26,5%	62,8%	79,1%	74,1%

99	20	20	55	55	23,4%	26,3%	63,5%	80,2%	75,4%
100	20	20	55	60	23,7%	26,0%	64,3%	81,3%	76,6%
101	20	20	55	65	24,0%	25,8%	65,1%	82,3%	77,8%
102	20	20	55	70	24,4%	25,5%	65,9%	83,3%	79,0%
103	20	20	55	75	24,7%	25,3%	66,6%	84,3%	80,1%
104	20	20	55	80	25,0%	25,0%	67,4%	85,2%	81,2%
105	20	20	60	20	17,4%	23,7%	54,8%	70,2%	64,5%
106	20	20	60	25	17,7%	23,4%	55,7%	71,6%	66,1%
107	20	20	60	30	17,9%	23,2%	56,5%	73,0%	67,6%
108	20	20	60	35	18,2%	23,0%	57,3%	74,4%	69,1%
109	20	20	60	40	18,5%	22,7%	58,2%	75,7%	70,5%
110	20	20	60	45	18,7%	22,5%	59,0%	76,9%	71,9%
111	20	20	60	50	19,0%	22,3%	59,8%	78,1%	73,3%
112	20	20	60	55	19,3%	22,1%	60,6%	79,3%	74,6%
113	20	20	60	60	19,5%	21,8%	61,4%	80,4%	75,9%
114	20	20	60	65	19,8%	21,6%	62,2%	81,4%	77,1%
115	20	20	60	70	20,1%	21,4%	63,0%	82,5%	78,3%
116	20	20	60	75	20,4%	21,2%	63,8%	83,4%	79,5%
117	20	20	60	80	20,7%	20,9%	64,6%	84,4%	80,5%
118	20	20	65	20	14,1%	19,8%	51,7%	68,9%	63,6%
119	20	20	65	25	14,4%	19,5%	52,6%	70,4%	65,2%
120	20	20	65	30	14,6%	19,3%	53,4%	71,8%	66,7%
121	20	20	65	35	14,8%	19,1%	54,3%	73,2%	68,2%
122	20	20	65	40	15,0%	18,9%	55,1%	74,5%	69,7%
123	20	20	65	45	15,2%	18,7%	56,0%	75,8%	71,1%
124	20	20	65	50	15,5%	18,5%	56,8%	77,0%	72,5%
125	20	20	65	55	15,7%	18,3%	57,6%	78,2%	73,8%
126	20	20	65	60	15,9%	18,1%	58,4%	79,4%	75,1%
127	20	20	65	65	16,2%	18,0%	59,3%	80,5%	76,4%
128	20	20	65	70	16,4%	17,8%	60,1%	81,6%	77,6%
129	20	20	65	75	16,7%	17,6%	60,9%	82,6%	78,8%
130	20	20	65	80	16,9%	17,4%	61,7%	83,5%	79,9%
131	20	20	70	20	11,4%	16,3%	48,6%	67,6%	62,6%

132	20	20	70	25	11,6%	16,2%	49,5%	69,1%	64,2%
133	20	20	70	30	11,8%	16,0%	50,3%	70,6%	65,8%
134	20	20	70	35	11,9%	15,8%	51,2%	72,0%	67,3%
135	20	20	70	40	12,1%	15,6%	52,0%	73,3%	68,8%
136	20	20	70	45	12,3%	15,5%	52,9%	74,7%	70,2%
137	20	20	70	50	12,5%	15,3%	53,7%	76,0%	71,7%
138	20	20	70	55	12,7%	15,1%	54,6%	77,2%	73,0%
139	20	20	70	60	12,9%	15,0%	55,4%	78,4%	74,4%
140	20	20	70	65	13,1%	14,8%	56,2%	79,5%	75,6%
141	20	20	70	70	13,3%	14,6%	57,1%	80,6%	76,9%
142	20	20	70	75	13,5%	14,5%	57,9%	81,7%	78,1%
143	20	20	70	80	13,7%	14,3%	58,7%	82,7%	79,2%
144	20	20	75	20	9,1%	13,4%	45,5%	66,3%	61,7%
145	20	20	75	25	9,3%	13,3%	46,4%	67,8%	63,3%
146	20	20	75	30	9,4%	13,1%	47,2%	69,3%	64,8%
147	20	20	75	35	9,6%	13,0%	48,1%	70,7%	66,4%
148	20	20	75	40	9,7%	12,8%	48,9%	72,1%	67,9%
149	20	20	75	45	9,9%	12,7%	49,8%	73,5%	69,4%
150	20	20	75	50	10,1%	12,5%	50,6%	74,8%	70,8%
151	20	20	75	55	10,2%	12,4%	51,5%	76,1%	72,2%
152	20	20	75	60	10,4%	12,3%	52,3%	77,3%	73,6%
153	20	20	75	65	10,5%	12,1%	53,2%	78,5%	74,9%
154	20	20	75	70	10,7%	12,0%	54,0%	79,7%	76,1%
155	20	20	75	75	10,9%	11,8%	54,8%	80,8%	77,4%
156	20	20	75	80	11,1%	11,7%	55,7%	81,8%	78,5%
157	20	20	80	20	7,3%	11,0%	42,5%	64,9%	60,7%
158	20	20	80	25	7,4%	10,8%	43,3%	66,4%	62,3%
159	20	20	80	30	7,5%	10,7%	44,1%	68,0%	63,9%
160	20	20	80	35	7,6%	10,6%	45,0%	69,4%	65,5%
161	20	20	80	40	7,8%	10,5%	45,8%	70,9%	67,0%
162	20	20	80	45	7,9%	10,3%	46,7%	72,3%	68,5%
163	20	20	80	50	8,0%	10,2%	47,5%	73,7%	69,9%
164	20	20	80	55	8,2%	10,1%	48,4%	75,0%	71,4%

165	20	20	80	60	8,3%	10,0%	49,2%	76,2%	72,7%
166	20	20	80	65	8,4%	9,9%	50,1%	77,5%	74,1%
167	20	20	80	70	8,6%	9,7%	50,9%	78,7%	75,4%
168	20	20	80	75	8,7%	9,6%	51,8%	79,8%	76,6%
169	20	20	80	80	8,8%	9,5%	52,6%	80,9%	77,8%
170	20	25	20	20	62,4%	68,0%	76,1%	78,1%	69,7%
171	20	25	20	25	62,8%	67,7%	76,7%	79,3%	71,1%
172	20	25	20	30	63,2%	67,5%	77,3%	80,4%	72,5%
173	20	25	20	35	63,7%	67,2%	77,9%	81,4%	73,9%
174	20	25	20	40	64,1%	66,9%	78,4%	82,5%	75,2%
175	20	25	20	45	64,5%	66,6%	79,0%	83,4%	76,4%
176	20	25	20	50	64,9%	66,3%	79,6%	84,4%	77,6%
177	20	25	20	55	65,3%	66,0%	80,1%	85,3%	78,8%
178	20	25	20	60	65,7%	65,7%	80,7%	86,1%	79,9%
179	20	25	20	65	66,1%	65,4%	81,2%	86,9%	81,0%
180	20	25	20	70	66,5%	65,1%	81,7%	87,7%	82,0%
181	20	25	20	75	66,8%	64,8%	82,2%	88,4%	83,0%
182	20	25	20	80	67,2%	64,5%	82,7%	89,1%	84,0%
183	20	25	25	20	56,5%	62,8%	73,7%	77,0%	68,8%
184	20	25	25	25	56,9%	62,5%	74,4%	78,2%	70,3%
185	20	25	25	30	57,3%	62,2%	75,0%	79,4%	71,7%
186	20	25	25	35	57,8%	61,9%	75,6%	80,5%	73,1%
187	20	25	25	40	58,2%	61,6%	76,3%	81,6%	74,4%
188	20	25	25	45	58,6%	61,3%	76,9%	82,6%	75,7%
189	20	25	25	50	59,1%	61,0%	77,5%	83,5%	76,9%
190	20	25	25	55	59,5%	60,7%	78,1%	84,5%	78,1%
191	20	25	25	60	59,9%	60,3%	78,6%	85,4%	79,2%
192	20	25	25	65	60,3%	60,0%	79,2%	86,2%	80,4%
193	20	25	25	70	60,8%	59,7%	79,8%	87,0%	81,4%
194	20	25	25	75	61,2%	59,4%	80,3%	87,8%	82,4%
195	20	25	25	80	61,6%	59,1%	80,8%	88,5%	83,4%
196	20	25	30	20	50,3%	57,3%	71,2%	75,9%	67,9%
197	20	25	30	25	50,8%	57,0%	71,9%	77,2%	69,4%

198	20	25	30	30	51,2%	56,6%	72,6%	78,4%	70,8%
199	20	25	30	35	51,7%	56,3%	73,3%	79,5%	72,2%
200	20	25	30	40	52,1%	56,0%	73,9%	80,6%	73,6%
201	20	25	30	45	52,5%	55,7%	74,6%	81,7%	74,9%
202	20	25	30	50	53,0%	55,4%	75,2%	82,7%	76,2%
203	20	25	30	55	53,4%	55,0%	75,9%	83,7%	77,4%
204	20	25	30	60	53,9%	54,7%	76,5%	84,6%	78,6%
205	20	25	30	65	54,3%	54,4%	77,1%	85,5%	79,7%
206	20	25	30	70	54,7%	54,1%	77,7%	86,3%	80,8%
207	20	25	30	75	55,2%	53,7%	78,3%	87,1%	81,8%
208	20	25	30	80	55,6%	53,4%	78,8%	87,8%	82,8%
209	20	25	35	20	44,2%	51,6%	68,6%	74,8%	67,0%
210	20	25	35	25	44,6%	51,2%	69,4%	76,1%	68,5%
211	20	25	35	30	45,1%	50,9%	70,1%	77,3%	70,0%
212	20	25	35	35	45,5%	50,6%	70,8%	78,5%	71,4%
213	20	25	35	40	45,9%	50,3%	71,5%	79,7%	72,8%
214	20	25	35	45	46,4%	49,9%	72,2%	80,7%	74,1%
215	20	25	35	50	46,8%	49,6%	72,8%	81,8%	75,4%
216	20	25	35	55	47,3%	49,3%	73,5%	82,8%	76,7%
217	20	25	35	60	47,7%	48,9%	74,2%	83,8%	77,9%
218	20	25	35	65	48,1%	48,6%	74,8%	84,7%	79,0%
219	20	25	35	70	48,6%	48,3%	75,4%	85,6%	80,1%
220	20	25	35	75	49,0%	48,0%	76,1%	86,4%	81,2%
221	20	25	35	80	49,5%	47,6%	76,7%	87,2%	82,2%
222	20	25	40	20	38,2%	45,8%	65,9%	73,7%	66,1%
223	20	25	40	25	38,6%	45,5%	66,7%	75,0%	67,6%
224	20	25	40	30	39,1%	45,1%	67,4%	76,2%	69,1%
225	20	25	40	35	39,5%	44,8%	68,2%	77,5%	70,6%
226	20	25	40	40	39,9%	44,5%	68,9%	78,6%	72,0%
227	20	25	40	45	40,3%	44,2%	69,6%	79,8%	73,3%
228	20	25	40	50	40,7%	43,9%	70,3%	80,9%	74,6%
229	20	25	40	55	41,2%	43,5%	71,0%	81,9%	75,9%
230	20	25	40	60	41,6%	43,2%	71,7%	82,9%	77,1%

231	20	25	40	65	42,0%	42,9%	72,4%	83,9%	78,3%
232	20	25	40	70	42,5%	42,6%	73,1%	84,8%	79,5%
233	20	25	40	75	42,9%	42,3%	73,7%	85,7%	80,6%
234	20	25	40	80	43,3%	41,9%	74,4%	86,5%	81,6%
235	20	25	45	20	32,6%	40,1%	63,1%	72,5%	65,2%
236	20	25	45	25	33,0%	39,8%	63,8%	73,8%	66,7%
237	20	25	45	30	33,4%	39,5%	64,6%	75,1%	68,2%
238	20	25	45	35	33,8%	39,2%	65,4%	76,4%	69,7%
239	20	25	45	40	34,2%	38,9%	66,2%	77,6%	71,1%
240	20	25	45	45	34,5%	38,6%	66,9%	78,8%	72,5%
241	20	25	45	50	34,9%	38,3%	67,7%	79,9%	73,8%
242	20	25	45	55	35,4%	38,0%	68,4%	81,0%	75,1%
243	20	25	45	60	35,8%	37,7%	69,1%	82,0%	76,4%
244	20	25	45	65	36,2%	37,4%	69,9%	83,0%	77,6%
245	20	25	45	70	36,6%	37,0%	70,6%	84,0%	78,8%
246	20	25	45	75	37,0%	36,7%	71,3%	84,9%	79,9%
247	20	25	45	80	37,4%	36,4%	72,0%	85,7%	81,0%
248	20	25	50	20	27,4%	34,7%	60,1%	71,2%	64,2%
249	20	25	50	25	27,8%	34,4%	60,9%	72,6%	65,8%
250	20	25	50	30	28,1%	34,2%	61,7%	74,0%	67,3%
251	20	25	50	35	28,5%	33,9%	62,5%	75,3%	68,8%
252	20	25	50	40	28,8%	33,6%	63,3%	76,5%	70,3%
253	20	25	50	45	29,2%	33,3%	64,1%	77,7%	71,7%
254	20	25	50	50	29,6%	33,0%	64,9%	78,9%	73,0%
255	20	25	50	55	29,9%	32,7%	65,7%	80,0%	74,4%
256	20	25	50	60	30,3%	32,4%	66,4%	81,1%	75,7%
257	20	25	50	65	30,7%	32,1%	67,2%	82,2%	76,9%
258	20	25	50	70	31,1%	31,8%	67,9%	83,1%	78,1%
259	20	25	50	75	31,4%	31,6%	68,7%	84,1%	79,2%
260	20	25	50	80	31,8%	31,3%	69,4%	85,0%	80,3%
261	20	25	55	20	22,8%	29,7%	57,1%	70,0%	63,3%
262	20	25	55	25	23,1%	29,4%	57,9%	71,4%	64,9%
263	20	25	55	30	23,4%	29,2%	58,8%	72,8%	66,4%

264	20	25	55	35	23,7%	28,9%	59,6%	74,1%	67,9%
265	20	25	55	40	24,0%	28,6%	60,4%	75,4%	69,4%
266	20	25	55	45	24,4%	28,4%	61,2%	76,7%	70,8%
267	20	25	55	50	24,7%	28,1%	62,0%	77,9%	72,2%
268	20	25	55	55	25,0%	27,8%	62,8%	79,0%	73,6%
269	20	25	55	60	25,4%	27,6%	63,6%	80,2%	74,9%
270	20	25	55	65	25,7%	27,3%	64,4%	81,2%	76,2%
271	20	25	55	70	26,0%	27,0%	65,2%	82,3%	77,4%
272	20	25	55	75	26,4%	26,8%	65,9%	83,3%	78,6%
273	20	25	55	80	26,7%	26,5%	66,7%	84,2%	79,7%
274	20	25	60	20	18,7%	25,1%	54,0%	68,7%	62,3%
275	20	25	60	25	19,0%	24,9%	54,9%	70,1%	63,9%
276	20	25	60	30	19,3%	24,6%	55,7%	71,5%	65,5%
277	20	25	60	35	19,5%	24,4%	56,6%	72,9%	67,0%
278	20	25	60	40	19,8%	24,1%	57,4%	74,3%	68,5%
279	20	25	60	45	20,1%	23,9%	58,2%	75,6%	70,0%
280	20	25	60	50	20,4%	23,7%	59,0%	76,8%	71,4%
281	20	25	60	55	20,7%	23,4%	59,9%	78,0%	72,8%
282	20	25	60	60	21,0%	23,2%	60,7%	79,2%	74,1%
283	20	25	60	65	21,3%	23,0%	61,5%	80,3%	75,4%
284	20	25	60	70	21,6%	22,7%	62,3%	81,4%	76,6%
285	20	25	60	75	21,9%	22,5%	63,1%	82,4%	77,8%
286	20	25	60	80	22,2%	22,3%	63,9%	83,4%	79,0%
287	20	25	65	20	15,3%	21,0%	50,9%	67,3%	61,4%
288	20	25	65	25	15,5%	20,8%	51,8%	68,8%	63,0%
289	20	25	65	30	15,7%	20,6%	52,6%	70,3%	64,5%
290	20	25	65	35	16,0%	20,4%	53,5%	71,7%	66,1%
291	20	25	65	40	16,2%	20,2%	54,3%	73,1%	67,6%
292	20	25	65	45	16,4%	20,0%	55,2%	74,4%	69,1%
293	20	25	65	50	16,7%	19,8%	56,0%	75,7%	70,5%
294	20	25	65	55	16,9%	19,5%	56,8%	77,0%	71,9%
295	20	25	65	60	17,2%	19,3%	57,7%	78,2%	73,3%
296	20	25	65	65	17,4%	19,1%	58,5%	79,3%	74,6%

297	20	25	65	70	17,7%	18,9%	59,3%	80,4%	75,9%
298	20	25	65	75	17,9%	18,7%	60,1%	81,5%	77,1%
299	20	25	65	80	18,2%	18,5%	60,9%	82,5%	78,3%
300	20	25	70	20	12,3%	17,4%	47,8%	66,0%	60,4%
301	20	25	70	25	12,5%	17,3%	48,7%	67,5%	62,0%
302	20	25	70	30	12,7%	17,1%	49,5%	69,0%	63,6%
303	20	25	70	35	12,9%	16,9%	50,4%	70,5%	65,2%
304	20	25	70	40	13,1%	16,7%	51,2%	71,9%	66,7%
305	20	25	70	45	13,3%	16,5%	52,1%	73,2%	68,2%
306	20	25	70	50	13,5%	16,3%	52,9%	74,6%	69,7%
307	20	25	70	55	13,7%	16,2%	53,8%	75,9%	71,1%
308	20	25	70	60	13,9%	16,0%	54,6%	77,1%	72,5%
309	20	25	70	65	14,2%	15,8%	55,5%	78,3%	73,8%
310	20	25	70	70	14,4%	15,6%	56,3%	79,4%	75,1%
311	20	25	70	75	14,6%	15,5%	57,1%	80,5%	76,4%
312	20	25	70	80	14,8%	15,3%	58,0%	81,6%	77,6%
313	20	25	75	20	9,9%	14,4%	44,7%	64,6%	59,4%
314	20	25	75	25	10,1%	14,2%	45,6%	66,2%	61,0%
315	20	25	75	30	10,2%	14,0%	46,4%	67,7%	62,6%
316	20	25	75	35	10,4%	13,9%	47,3%	69,2%	64,2%
317	20	25	75	40	10,5%	13,7%	48,1%	70,6%	65,8%
318	20	25	75	45	10,7%	13,6%	49,0%	72,0%	67,3%
319	20	25	75	50	10,9%	13,4%	49,8%	73,4%	68,8%
320	20	25	75	55	11,1%	13,3%	50,7%	74,7%	70,2%
321	20	25	75	60	11,2%	13,1%	51,5%	76,0%	71,7%
322	20	25	75	65	11,4%	13,0%	52,4%	77,2%	73,0%
323	20	25	75	70	11,6%	12,8%	53,2%	78,4%	74,4%
324	20	25	75	75	11,8%	12,7%	54,1%	79,6%	75,6%
325	20	25	75	80	12,0%	12,5%	54,9%	80,7%	76,9%
326	20	25	80	20	7,9%	11,8%	41,7%	63,2%	58,4%
327	20	25	80	25	8,0%	11,6%	42,5%	64,8%	60,0%
328	20	25	80	30	8,2%	11,5%	43,4%	66,3%	61,7%
329	20	25	80	35	8,3%	11,4%	44,2%	67,9%	63,3%

330	20	25	80	40	8,4%	11,2%	45,0%	69,3%	64,8%
331	20	25	80	45	8,6%	11,1%	45,9%	70,8%	66,4%
332	20	25	80	50	8,7%	11,0%	46,7%	72,2%	67,9%
333	20	25	80	55	8,9%	10,8%	47,6%	73,6%	69,4%
334	20	25	80	60	9,0%	10,7%	48,4%	74,9%	70,8%
335	20	25	80	65	9,1%	10,6%	49,3%	76,2%	72,2%
336	20	25	80	70	9,3%	10,5%	50,1%	77,4%	73,6%
337	20	25	80	75	9,4%	10,3%	51,0%	78,6%	74,9%
338	20	25	80	80	9,6%	10,2%	51,8%	79,7%	76,1%
339	20	30	20	20	64,5%	69,7%	75,5%	76,8%	67,7%
340	20	30	20	25	64,9%	69,4%	76,1%	78,0%	69,1%
341	20	30	20	30	65,3%	69,2%	76,7%	79,2%	70,6%
342	20	30	20	35	65,7%	68,9%	77,3%	80,3%	72,0%
343	20	30	20	40	66,1%	68,6%	77,9%	81,4%	73,3%
344	20	30	20	45	66,5%	68,3%	78,5%	82,4%	74,7%
345	20	30	20	50	66,9%	68,0%	79,0%	83,4%	75,9%
346	20	30	20	55	67,3%	67,8%	79,6%	84,3%	77,2%
347	20	30	20	60	67,6%	67,5%	80,1%	85,2%	78,3%
348	20	30	20	65	68,0%	67,2%	80,7%	86,0%	79,5%
349	20	30	20	70	68,4%	66,9%	81,2%	86,8%	80,6%
350	20	30	20	75	68,8%	66,6%	81,7%	87,6%	81,6%
351	20	30	20	80	69,2%	66,3%	82,2%	88,3%	82,6%
352	20	30	25	20	58,6%	64,6%	73,1%	75,7%	66,7%
353	20	30	25	25	59,1%	64,3%	73,8%	77,0%	68,2%
354	20	30	25	30	59,5%	64,0%	74,4%	78,2%	69,7%
355	20	30	25	35	59,9%	63,7%	75,1%	79,3%	71,1%
356	20	30	25	40	60,3%	63,4%	75,7%	80,4%	72,5%
357	20	30	25	45	60,8%	63,1%	76,3%	81,5%	73,9%
358	20	30	25	50	61,2%	62,8%	76,9%	82,5%	75,2%
359	20	30	25	55	61,6%	62,5%	77,5%	83,5%	76,4%
360	20	30	25	60	62,0%	62,2%	78,1%	84,4%	77,6%
361	20	30	25	65	62,4%	61,9%	78,7%	85,3%	78,8%
362	20	30	25	70	62,8%	61,6%	79,2%	86,1%	79,9%

363	20	30	25	75	63,3%	61,3%	79,8%	86,9%	81,0%
364	20	30	25	80	63,7%	61,0%	80,3%	87,7%	82,0%
365	20	30	30	20	52,6%	59,2%	70,6%	74,6%	65,8%
366	20	30	30	25	53,0%	58,9%	71,3%	75,9%	67,3%
367	20	30	30	30	53,4%	58,6%	72,0%	77,1%	68,8%
368	20	30	30	35	53,9%	58,2%	72,7%	78,3%	70,3%
369	20	30	30	40	54,3%	57,9%	73,3%	79,4%	71,7%
370	20	30	30	45	54,7%	57,6%	74,0%	80,5%	73,1%
371	20	30	30	50	55,2%	57,3%	74,6%	81,6%	74,4%
372	20	30	30	55	55,6%	57,0%	75,3%	82,6%	75,7%
373	20	30	30	60	56,1%	56,6%	75,9%	83,6%	76,9%
374	20	30	30	65	56,5%	56,3%	76,5%	84,5%	78,1%
375	20	30	30	70	56,9%	56,0%	77,1%	85,4%	79,3%
376	20	30	30	75	57,4%	55,7%	77,7%	86,2%	80,4%
377	20	30	30	80	57,8%	55,4%	78,3%	87,0%	81,4%
378	20	30	35	20	46,4%	53,5%	67,9%	73,4%	64,9%
379	20	30	35	25	46,8%	53,2%	68,7%	74,7%	66,4%
380	20	30	35	30	47,3%	52,9%	69,4%	76,0%	67,9%
381	20	30	35	35	47,7%	52,5%	70,1%	77,2%	69,4%
382	20	30	35	40	48,2%	52,2%	70,8%	78,4%	70,8%
383	20	30	35	45	48,6%	51,9%	71,5%	79,6%	72,2%
384	20	30	35	50	49,0%	51,6%	72,2%	80,7%	73,6%
385	20	30	35	55	49,5%	51,2%	72,9%	81,7%	74,9%
386	20	30	35	60	49,9%	50,9%	73,6%	82,7%	76,2%
387	20	30	35	65	50,4%	50,6%	74,2%	83,7%	77,4%
388	20	30	35	70	50,8%	50,3%	74,9%	84,6%	78,6%
389	20	30	35	75	51,2%	49,9%	75,5%	85,5%	79,7%
390	20	30	35	80	51,7%	49,6%	76,1%	86,3%	80,8%
391	20	30	40	20	40,3%	47,8%	65,2%	72,2%	63,9%
392	20	30	40	25	40,8%	47,4%	65,9%	73,6%	65,5%
393	20	30	40	30	41,2%	47,1%	66,7%	74,9%	67,0%
394	20	30	40	35	41,6%	46,8%	67,5%	76,1%	68,5%
395	20	30	40	40	42,0%	46,5%	68,2%	77,4%	70,0%

396	20	30	40	45	42,5%	46,1%	68,9%	78,6%	71,4%
397	20	30	40	50	42,9%	45,8%	69,7%	79,7%	72,8%
398	20	30	40	55	43,3%	45,5%	70,4%	80,8%	74,1%
399	20	30	40	60	43,8%	45,2%	71,1%	81,8%	75,4%
400	20	30	40	65	44,2%	44,8%	71,8%	82,8%	76,7%
401	20	30	40	70	44,6%	44,5%	72,4%	83,8%	77,9%
402	20	30	40	75	45,1%	44,2%	73,1%	84,7%	79,0%
403	20	30	40	80	45,5%	43,9%	73,8%	85,6%	80,1%
404	20	30	45	20	34,6%	42,1%	62,3%	70,9%	63,0%
405	20	30	45	25	35,0%	41,7%	63,1%	72,3%	64,6%
406	20	30	45	30	35,4%	41,4%	63,9%	73,7%	66,1%
407	20	30	45	35	35,8%	41,1%	64,7%	75,0%	67,6%
408	20	30	45	40	36,2%	40,8%	65,4%	76,3%	69,1%
409	20	30	45	45	36,6%	40,5%	66,2%	77,5%	70,6%
410	20	30	45	50	37,0%	40,2%	67,0%	78,7%	72,0%
411	20	30	45	55	37,4%	39,8%	67,7%	79,8%	73,3%
412	20	30	45	60	37,8%	39,5%	68,4%	80,9%	74,6%
413	20	30	45	65	38,2%	39,2%	69,2%	82,0%	75,9%
414	20	30	45	70	38,7%	38,9%	69,9%	83,0%	77,1%
415	20	30	45	75	39,1%	38,6%	70,6%	83,9%	78,3%
416	20	30	45	80	39,5%	38,3%	71,3%	84,8%	79,5%
417	20	30	50	20	29,2%	36,5%	59,4%	69,7%	62,0%
418	20	30	50	25	29,6%	36,2%	60,2%	71,1%	63,6%
419	20	30	50	30	29,9%	35,9%	61,0%	72,5%	65,2%
420	20	30	50	35	30,3%	35,6%	61,8%	73,9%	66,7%
421	20	30	50	40	30,7%	35,3%	62,6%	75,2%	68,2%
422	20	30	50	45	31,1%	35,0%	63,4%	76,4%	69,7%
423	20	30	50	50	31,4%	34,7%	64,2%	77,7%	71,1%
424	20	30	50	55	31,8%	34,5%	64,9%	78,8%	72,5%
425	20	30	50	60	32,2%	34,2%	65,7%	80,0%	73,9%
426	20	30	50	65	32,6%	33,9%	66,5%	81,0%	75,2%
427	20	30	50	70	33,0%	33,6%	67,2%	82,1%	76,4%
428	20	30	50	75	33,4%	33,3%	68,0%	83,1%	77,6%

429	20	30	50	80	33,8%	33,0%	68,7%	84,0%	78,8%
430	20	30	55	20	24,4%	31,4%	56,3%	68,4%	61,0%
431	20	30	55	25	24,7%	31,1%	57,2%	69,8%	62,7%
432	20	30	55	30	25,0%	30,8%	58,0%	71,3%	64,2%
433	20	30	55	35	25,4%	30,5%	58,8%	72,7%	65,8%
434	20	30	55	40	25,7%	30,3%	59,6%	74,0%	67,3%
435	20	30	55	45	26,0%	30,0%	60,4%	75,3%	68,8%
436	20	30	55	50	26,4%	29,7%	61,3%	76,6%	70,3%
437	20	30	55	55	26,7%	29,4%	62,1%	77,8%	71,7%
438	20	30	55	60	27,1%	29,2%	62,9%	79,0%	73,0%
439	20	30	55	65	27,4%	28,9%	63,6%	80,1%	74,4%
440	20	30	55	70	27,8%	28,6%	64,4%	81,2%	75,7%
441	20	30	55	75	28,1%	28,4%	65,2%	82,2%	76,9%
442	20	30	55	80	28,5%	28,1%	66,0%	83,2%	78,1%
443	20	30	60	20	20,1%	26,6%	53,2%	67,0%	60,1%
444	20	30	60	25	20,4%	26,4%	54,1%	68,5%	61,7%
445	20	30	60	30	20,7%	26,1%	54,9%	70,0%	63,3%
446	20	30	60	35	21,0%	25,9%	55,8%	71,4%	64,9%
447	20	30	60	40	21,3%	25,6%	56,6%	72,8%	66,4%
448	20	30	60	45	21,6%	25,4%	57,4%	74,2%	67,9%
449	20	30	60	50	21,9%	25,1%	58,3%	75,5%	69,4%
450	20	30	60	55	22,2%	24,9%	59,1%	76,7%	70,8%
451	20	30	60	60	22,5%	24,6%	59,9%	77,9%	72,2%
452	20	30	60	65	22,8%	24,4%	60,7%	79,1%	73,6%
453	20	30	60	70	23,1%	24,2%	61,5%	80,2%	74,9%
454	20	30	60	75	23,4%	23,9%	62,3%	81,3%	76,2%
455	20	30	60	80	23,7%	23,7%	63,1%	82,3%	77,4%
456	20	30	65	20	16,4%	22,4%	50,1%	65,7%	59,1%
457	20	30	65	25	16,7%	22,1%	51,0%	67,2%	60,7%
458	20	30	65	30	16,9%	21,9%	51,8%	68,7%	62,3%
459	20	30	65	35	17,2%	21,7%	52,7%	70,2%	63,9%
460	20	30	65	40	17,4%	21,5%	53,5%	71,6%	65,5%
461	20	30	65	45	17,7%	21,3%	54,4%	73,0%	67,0%

462	20	30	65	50	17,9%	21,0%	55,2%	74,3%	68,5%
463	20	30	65	55	18,2%	20,8%	56,1%	75,6%	70,0%
464	20	30	65	60	18,5%	20,6%	56,9%	76,9%	71,4%
465	20	30	65	65	18,7%	20,4%	57,7%	78,1%	72,8%
466	20	30	65	70	19,0%	20,2%	58,6%	79,2%	74,1%
467	20	30	65	75	19,3%	20,0%	59,4%	80,3%	75,4%
468	20	30	65	80	19,6%	19,8%	60,2%	81,4%	76,6%
469	20	30	70	20	13,3%	18,6%	47,0%	64,3%	58,1%
470	20	30	70	25	13,5%	18,4%	47,9%	65,9%	59,7%
471	20	30	70	30	13,7%	18,2%	48,7%	67,4%	61,4%
472	20	30	70	35	13,9%	18,0%	49,6%	68,9%	63,0%
473	20	30	70	40	14,2%	17,8%	50,4%	70,3%	64,6%
474	20	30	70	45	14,4%	17,6%	51,3%	71,8%	66,1%
475	20	30	70	50	14,6%	17,5%	52,1%	73,1%	67,6%
476	20	30	70	55	14,8%	17,3%	53,0%	74,5%	69,1%
477	20	30	70	60	15,0%	17,1%	53,8%	75,8%	70,5%
478	20	30	70	65	15,3%	16,9%	54,7%	77,0%	71,9%
479	20	30	70	70	15,5%	16,7%	55,5%	78,2%	73,3%
480	20	30	70	75	15,7%	16,5%	56,3%	79,4%	74,6%
481	20	30	70	80	16,0%	16,4%	57,2%	80,5%	75,9%
482	20	30	75	20	10,7%	15,4%	44,0%	62,9%	57,1%
483	20	30	75	25	10,9%	15,2%	44,8%	64,5%	58,7%
484	20	30	75	30	11,1%	15,0%	45,6%	66,0%	60,4%
485	20	30	75	35	11,2%	14,9%	46,5%	67,6%	62,0%
486	20	30	75	40	11,4%	14,7%	47,3%	69,1%	63,6%
487	20	30	75	45	11,6%	14,5%	48,2%	70,5%	65,2%
488	20	30	75	50	11,8%	14,4%	49,0%	71,9%	66,7%
489	20	30	75	55	12,0%	14,2%	49,9%	73,3%	68,2%
490	20	30	75	60	12,1%	14,1%	50,7%	74,6%	69,7%
491	20	30	75	65	12,3%	13,9%	51,6%	75,9%	71,1%
492	20	30	75	70	12,5%	13,7%	52,4%	77,1%	72,5%
493	20	30	75	75	12,7%	13,6%	53,3%	78,3%	73,8%
494	20	30	75	80	12,9%	13,4%	54,1%	79,5%	75,1%

495	20	30	80	20	8,6%	12,6%	40,9%	61,4%	56,0%
496	20	30	80	25	8,7%	12,5%	41,8%	63,1%	57,7%
497	20	30	80	30	8,9%	12,3%	42,6%	64,7%	59,4%
498	20	30	80	35	9,0%	12,2%	43,4%	66,2%	61,0%
499	20	30	80	40	9,1%	12,0%	44,3%	67,7%	62,6%
500	20	30	80	45	9,3%	11,9%	45,1%	69,2%	64,2%
501	20	30	80	50	9,4%	11,8%	45,9%	70,7%	65,8%
502	20	30	80	55	9,6%	11,6%	46,8%	72,1%	67,3%
503	20	30	80	60	9,7%	11,5%	47,6%	73,5%	68,8%
504	20	30	80	65	9,9%	11,4%	48,5%	74,8%	70,3%
505	20	30	80	70	10,1%	11,2%	49,3%	76,1%	71,7%
506	20	30	80	75	10,2%	11,1%	50,2%	77,3%	73,0%
507	20	30	80	80	10,4%	11,0%	51,0%	78,5%	74,4%
508	20	35	20	20	66,5%	71,4%	74,9%	75,5%	65,5%
509	20	35	20	25	66,9%	71,1%	75,5%	76,7%	67,1%
510	20	35	20	30	67,3%	70,8%	76,1%	77,9%	68,6%
511	20	35	20	35	67,7%	70,5%	76,7%	79,1%	70,0%
512	20	35	20	40	68,0%	70,3%	77,3%	80,2%	71,4%
513	20	35	20	45	68,4%	70,0%	77,9%	81,3%	72,8%
514	20	35	20	50	68,8%	69,7%	78,5%	82,3%	74,1%
515	20	35	20	55	69,2%	69,4%	79,1%	83,3%	75,4%
516	20	35	20	60	69,5%	69,2%	79,6%	84,2%	76,7%
517	20	35	20	65	69,9%	68,9%	80,2%	85,1%	77,9%
518	20	35	20	70	70,3%	68,6%	80,7%	86,0%	79,0%
519	20	35	20	75	70,7%	68,3%	81,2%	86,8%	80,2%
520	20	35	20	80	71,0%	68,0%	81,8%	87,6%	81,2%
521	20	35	25	20	60,8%	66,4%	72,5%	74,3%	64,6%
522	20	35	25	25	61,2%	66,1%	73,1%	75,6%	66,1%
523	20	35	25	30	61,6%	65,8%	73,8%	76,9%	67,7%
524	20	35	25	35	62,0%	65,5%	74,5%	78,1%	69,1%
525	20	35	25	40	62,4%	65,2%	75,1%	79,2%	70,6%
526	20	35	25	45	62,9%	64,9%	75,7%	80,3%	72,0%
527	20	35	25	50	63,3%	64,6%	76,3%	81,4%	73,3%

528	20	35	25	55	63,7%	64,3%	77,0%	82,4%	74,7%
529	20	35	25	60	64,1%	64,0%	77,6%	83,4%	75,9%
530	20	35	25	65	64,5%	63,7%	78,1%	84,3%	77,2%
531	20	35	25	70	64,9%	63,4%	78,7%	85,2%	78,3%
532	20	35	25	75	65,3%	63,1%	79,3%	86,1%	79,5%
533	20	35	25	80	65,7%	62,8%	79,8%	86,9%	80,6%
534	20	35	30	20	54,8%	61,1%	69,9%	73,1%	63,6%
535	20	35	30	25	55,2%	60,8%	70,6%	74,5%	65,2%
536	20	35	30	30	55,6%	60,5%	71,3%	75,8%	66,7%
537	20	35	30	35	56,1%	60,1%	72,0%	77,0%	68,3%
538	20	35	30	40	56,5%	59,8%	72,7%	78,2%	69,7%
539	20	35	30	45	56,9%	59,5%	73,4%	79,4%	71,1%
540	20	35	30	50	57,4%	59,2%	74,0%	80,5%	72,5%
541	20	35	30	55	57,8%	58,9%	74,7%	81,5%	73,9%
542	20	35	30	60	58,2%	58,6%	75,3%	82,5%	75,2%
543	20	35	30	65	58,7%	58,3%	75,9%	83,5%	76,4%
544	20	35	30	70	59,1%	57,9%	76,6%	84,4%	77,6%
545	20	35	30	75	59,5%	57,6%	77,2%	85,3%	78,8%
546	20	35	30	80	59,9%	57,3%	77,8%	86,2%	79,9%
547	20	35	35	20	48,6%	55,5%	67,2%	71,9%	62,7%
548	20	35	35	25	49,0%	55,2%	68,0%	73,3%	64,3%
549	20	35	35	30	49,5%	54,8%	68,7%	74,6%	65,8%
550	20	35	35	35	49,9%	54,5%	69,5%	75,9%	67,4%
551	20	35	35	40	50,4%	54,2%	70,2%	77,1%	68,8%
552	20	35	35	45	50,8%	53,9%	70,9%	78,3%	70,3%
553	20	35	35	50	51,2%	53,5%	71,6%	79,5%	71,7%
554	20	35	35	55	51,7%	53,2%	72,3%	80,6%	73,1%
555	20	35	35	60	52,1%	52,9%	72,9%	81,6%	74,4%
556	20	35	35	65	52,6%	52,6%	73,6%	82,7%	75,7%
557	20	35	35	70	53,0%	52,2%	74,3%	83,6%	76,9%
558	20	35	35	75	53,4%	51,9%	74,9%	84,5%	78,1%
559	20	35	35	80	53,9%	51,6%	75,5%	85,4%	79,3%
560	20	35	40	20	42,5%	49,7%	64,5%	70,7%	61,7%

561	20	35	40	25	42,9%	49,4%	65,2%	72,1%	63,3%
562	20	35	40	30	43,4%	49,1%	66,0%	73,5%	64,9%
563	20	35	40	35	43,8%	48,7%	66,8%	74,8%	66,4%
564	20	35	40	40	44,2%	48,4%	67,5%	76,1%	67,9%
565	20	35	40	45	44,7%	48,1%	68,2%	77,3%	69,4%
566	20	35	40	50	45,1%	47,8%	69,0%	78,5%	70,9%
567	20	35	40	55	45,5%	47,4%	69,7%	79,6%	72,2%
568	20	35	40	60	46,0%	47,1%	70,4%	80,7%	73,6%
569	20	35	40	65	46,4%	46,8%	71,1%	81,8%	74,9%
570	20	35	40	70	46,8%	46,5%	71,8%	82,8%	76,2%
571	20	35	40	75	47,3%	46,1%	72,5%	83,7%	77,4%
572	20	35	40	80	47,7%	45,8%	73,2%	84,6%	78,6%
573	20	35	45	20	36,6%	44,0%	61,6%	69,4%	60,7%
574	20	35	45	25	37,0%	43,7%	62,4%	70,8%	62,4%
575	20	35	45	30	37,4%	43,3%	63,2%	72,2%	63,9%
576	20	35	45	35	37,8%	43,0%	63,9%	73,6%	65,5%
577	20	35	45	40	38,2%	42,7%	64,7%	74,9%	67,0%
578	20	35	45	45	38,7%	42,4%	65,5%	76,2%	68,5%
579	20	35	45	50	39,1%	42,1%	66,3%	77,4%	70,0%
580	20	35	45	55	39,5%	41,7%	67,0%	78,6%	71,4%
581	20	35	45	60	39,9%	41,4%	67,8%	79,7%	72,8%
582	20	35	45	65	40,3%	41,1%	68,5%	80,8%	74,1%
583	20	35	45	70	40,8%	40,8%	69,2%	81,9%	75,4%
584	20	35	45	75	41,2%	40,5%	69,9%	82,9%	76,7%
585	20	35	45	80	41,6%	40,2%	70,7%	83,8%	77,9%
586	20	35	50	20	31,1%	38,4%	58,6%	68,1%	59,7%
587	20	35	50	25	31,5%	38,1%	59,4%	69,6%	61,4%
588	20	35	50	30	31,8%	37,8%	60,2%	71,0%	63,0%
589	20	35	50	35	32,2%	37,5%	61,0%	72,4%	64,6%
590	20	35	50	40	32,6%	37,2%	61,8%	73,8%	66,1%
591	20	35	50	45	33,0%	36,9%	62,6%	75,1%	67,6%
592	20	35	50	50	33,4%	36,6%	63,4%	76,3%	69,1%
593	20	35	50	55	33,8%	36,3%	64,2%	77,6%	70,6%

594	20	35	50	60	34,2%	36,0%	65,0%	78,7%	72,0%
595	20	35	50	65	34,6%	35,6%	65,8%	79,9%	73,3%
596	20	35	50	70	35,0%	35,4%	66,5%	81,0%	74,6%
597	20	35	50	75	35,4%	35,1%	67,3%	82,0%	75,9%
598	20	35	50	80	35,8%	34,8%	68,0%	83,0%	77,2%
599	20	35	55	20	26,0%	33,1%	55,5%	66,7%	58,7%
600	20	35	55	25	26,4%	32,8%	56,4%	68,3%	60,4%
601	20	35	55	30	26,7%	32,5%	57,2%	69,7%	62,0%
602	20	35	55	35	27,1%	32,2%	58,0%	71,2%	63,6%
603	20	35	55	40	27,4%	31,9%	58,9%	72,6%	65,2%
604	20	35	55	45	27,8%	31,7%	59,7%	73,9%	66,7%
605	20	35	55	50	28,1%	31,4%	60,5%	75,2%	68,2%
606	20	35	55	55	28,5%	31,1%	61,3%	76,5%	69,7%
607	20	35	55	60	28,9%	30,8%	62,1%	77,7%	71,1%
608	20	35	55	65	29,2%	30,5%	62,9%	78,9%	72,5%
609	20	35	55	70	29,6%	30,3%	63,7%	80,0%	73,9%
610	20	35	55	75	30,0%	30,0%	64,5%	81,1%	75,2%
611	20	35	55	80	30,3%	29,7%	65,3%	82,1%	76,4%
612	20	35	60	20	21,6%	28,2%	52,5%	65,4%	57,7%
613	20	35	60	25	21,9%	27,9%	53,3%	66,9%	59,4%
614	20	35	60	30	22,2%	27,7%	54,1%	68,4%	61,0%
615	20	35	60	35	22,5%	27,4%	55,0%	69,9%	62,7%
616	20	35	60	40	22,8%	27,1%	55,8%	71,3%	64,3%
617	20	35	60	45	23,1%	26,9%	56,7%	72,7%	65,8%
618	20	35	60	50	23,4%	26,6%	57,5%	74,1%	67,3%
619	20	35	60	55	23,7%	26,4%	58,3%	75,4%	68,8%
620	20	35	60	60	24,1%	26,1%	59,1%	76,6%	70,3%
621	20	35	60	65	24,4%	25,9%	60,0%	77,8%	71,7%
622	20	35	60	70	24,7%	25,6%	60,8%	79,0%	73,1%
623	20	35	60	75	25,0%	25,4%	61,6%	80,1%	74,4%
624	20	35	60	80	25,4%	25,1%	62,4%	81,2%	75,7%
625	20	35	65	20	17,7%	23,8%	49,4%	64,0%	56,7%
626	20	35	65	25	18,0%	23,5%	50,2%	65,6%	58,4%

627	20	35	65	30	18,2%	23,3%	51,1%	67,1%	60,1%
628	20	35	65	35	18,5%	23,1%	51,9%	68,6%	61,7%
629	20	35	65	40	18,7%	22,8%	52,7%	70,1%	63,3%
630	20	35	65	45	19,0%	22,6%	53,6%	71,5%	64,9%
631	20	35	65	50	19,3%	22,4%	54,4%	72,9%	66,4%
632	20	35	65	55	19,6%	22,1%	55,3%	74,2%	67,9%
633	20	35	65	60	19,8%	21,9%	56,1%	75,5%	69,4%
634	20	35	65	65	20,1%	21,7%	56,9%	76,8%	70,8%
635	20	35	65	70	20,4%	21,5%	57,8%	78,0%	72,2%
636	20	35	65	75	20,7%	21,3%	58,6%	79,1%	73,6%
637	20	35	65	80	21,0%	21,0%	59,4%	80,3%	74,9%
638	20	35	70	20	14,4%	19,8%	46,3%	62,6%	55,7%
639	20	35	70	25	14,6%	19,6%	47,1%	64,2%	57,4%
640	20	35	70	30	14,8%	19,4%	47,9%	65,7%	59,1%
641	20	35	70	35	15,0%	19,2%	48,8%	67,3%	60,7%
642	20	35	70	40	15,3%	19,0%	49,6%	68,8%	62,3%
643	20	35	70	45	15,5%	18,8%	50,5%	70,2%	63,9%
644	20	35	70	50	15,7%	18,6%	51,3%	71,7%	65,5%
645	20	35	70	55	16,0%	18,4%	52,2%	73,0%	67,0%
646	20	35	70	60	16,2%	18,2%	53,0%	74,4%	68,5%
647	20	35	70	65	16,4%	18,0%	53,9%	75,7%	70,0%
648	20	35	70	70	16,7%	17,8%	54,7%	76,9%	71,4%
649	20	35	70	75	16,9%	17,6%	55,6%	78,1%	72,8%
650	20	35	70	80	17,2%	17,5%	56,4%	79,3%	74,1%
651	20	35	75	20	11,6%	16,4%	43,2%	61,1%	54,7%
652	20	35	75	25	11,8%	16,2%	44,0%	62,8%	56,4%
653	20	35	75	30	12,0%	16,1%	44,9%	64,4%	58,1%
654	20	35	75	35	12,2%	15,9%	45,7%	65,9%	59,7%
655	20	35	75	40	12,3%	15,7%	46,5%	67,5%	61,4%
656	20	35	75	45	12,5%	15,5%	47,4%	69,0%	63,0%
657	20	35	75	50	12,7%	15,4%	48,2%	70,4%	64,6%
658	20	35	75	55	12,9%	15,2%	49,1%	71,8%	66,1%
659	20	35	75	60	13,1%	15,0%	49,9%	73,2%	67,6%

660	20	35	75	65	13,3%	14,9%	50,8%	74,5%	69,1%
661	20	35	75	70	13,5%	14,7%	51,6%	75,8%	70,5%
662	20	35	75	75	13,7%	14,5%	52,5%	77,1%	72,0%
663	20	35	75	80	13,9%	14,4%	53,3%	78,3%	73,3%
664	20	35	80	20	9,3%	13,5%	40,2%	59,7%	53,7%
665	20	35	80	25	9,4%	13,3%	41,0%	61,3%	55,4%
666	20	35	80	30	9,6%	13,2%	41,8%	62,9%	57,1%
667	20	35	80	35	9,8%	13,0%	42,6%	64,5%	58,7%
668	20	35	80	40	9,9%	12,9%	43,5%	66,1%	60,4%
669	20	35	80	45	10,1%	12,7%	44,3%	67,6%	62,0%
670	20	35	80	50	10,2%	12,6%	45,1%	69,1%	63,6%
671	20	35	80	55	10,4%	12,5%	46,0%	70,6%	65,2%
672	20	35	80	60	10,6%	12,3%	46,8%	72,0%	66,7%
673	20	35	80	65	10,7%	12,2%	47,7%	73,4%	68,2%
674	20	35	80	70	10,9%	12,0%	48,5%	74,7%	69,7%
675	20	35	80	75	11,1%	11,9%	49,4%	76,0%	71,1%
676	20	35	80	80	11,2%	11,8%	50,2%	77,2%	72,5%
677	20	40	20	20	68,4%	72,9%	74,3%	74,1%	63,3%
678	20	40	20	25	68,8%	72,7%	74,9%	75,4%	64,9%
679	20	40	20	30	69,2%	72,4%	75,6%	76,6%	66,5%
680	20	40	20	35	69,6%	72,2%	76,2%	77,8%	68,0%
681	20	40	20	40	69,9%	71,9%	76,8%	79,0%	69,4%
682	20	40	20	45	70,3%	71,6%	77,4%	80,1%	70,9%
683	20	40	20	50	70,7%	71,4%	78,0%	81,2%	72,3%
684	20	40	20	55	71,0%	71,1%	78,6%	82,2%	73,6%
685	20	40	20	60	71,4%	70,8%	79,1%	83,2%	74,9%
686	20	40	20	65	71,8%	70,6%	79,7%	84,2%	76,2%
687	20	40	20	70	72,1%	70,3%	80,2%	85,1%	77,4%
688	20	40	20	75	72,5%	70,0%	80,8%	85,9%	78,6%
689	20	40	20	80	72,8%	69,7%	81,3%	86,7%	79,7%
690	20	40	25	20	62,9%	68,1%	71,8%	72,9%	62,4%
691	20	40	25	25	63,3%	67,9%	72,5%	74,2%	64,0%
692	20	40	25	30	63,7%	67,6%	73,2%	75,5%	65,5%

693	20	40	25	35	64,1%	67,3%	73,8%	76,8%	67,1%
694	20	40	25	40	64,5%	67,0%	74,5%	78,0%	68,6%
695	20	40	25	45	64,9%	66,7%	75,1%	79,1%	70,0%
696	20	40	25	50	65,3%	66,4%	75,8%	80,3%	71,4%
697	20	40	25	55	65,7%	66,1%	76,4%	81,3%	72,8%
698	20	40	25	60	66,1%	65,8%	77,0%	82,4%	74,1%
699	20	40	25	65	66,5%	65,5%	77,6%	83,3%	75,4%
700	20	40	25	70	66,9%	65,2%	78,2%	84,3%	76,7%
701	20	40	25	75	67,3%	64,9%	78,7%	85,2%	77,9%
702	20	40	25	80	67,7%	64,6%	79,3%	86,0%	79,0%
703	20	40	30	20	57,0%	62,9%	69,3%	71,7%	61,4%
704	20	40	30	25	57,4%	62,6%	70,0%	73,0%	63,0%
705	20	40	30	30	57,8%	62,3%	70,7%	74,4%	64,6%
706	20	40	30	35	58,2%	62,0%	71,4%	75,7%	66,1%
707	20	40	30	40	58,7%	61,7%	72,1%	76,9%	67,7%
708	20	40	30	45	59,1%	61,4%	72,7%	78,1%	69,1%
709	20	40	30	50	59,5%	61,1%	73,4%	79,3%	70,6%
710	20	40	30	55	59,9%	60,8%	74,1%	80,4%	72,0%
711	20	40	30	60	60,4%	60,5%	74,7%	81,4%	73,3%
712	20	40	30	65	60,8%	60,2%	75,4%	82,5%	74,7%
713	20	40	30	70	61,2%	59,8%	76,0%	83,4%	75,9%
714	20	40	30	75	61,6%	59,5%	76,6%	84,4%	77,2%
715	20	40	30	80	62,0%	59,2%	77,2%	85,3%	78,4%
716	20	40	35	20	50,8%	57,4%	66,5%	70,4%	60,4%
717	20	40	35	25	51,3%	57,1%	67,3%	71,8%	62,0%
718	20	40	35	30	51,7%	56,8%	68,0%	73,2%	63,6%
719	20	40	35	35	52,1%	56,5%	68,8%	74,5%	65,2%
720	20	40	35	40	52,6%	56,1%	69,5%	75,8%	66,8%
721	20	40	35	45	53,0%	55,8%	70,2%	77,1%	68,3%
722	20	40	35	50	53,5%	55,5%	70,9%	78,3%	69,7%
723	20	40	35	55	53,9%	55,2%	71,6%	79,4%	71,1%
724	20	40	35	60	54,3%	54,8%	72,3%	80,5%	72,5%
725	20	40	35	65	54,8%	54,5%	73,0%	81,6%	73,9%

726	20	40	35	70	55,2%	54,2%	73,6%	82,6%	75,2%
727	20	40	35	75	55,6%	53,9%	74,3%	83,6%	76,4%
728	20	40	35	80	56,1%	53,5%	74,9%	84,5%	77,6%
729	20	40	40	20	44,7%	51,7%	63,7%	69,1%	59,4%
730	20	40	40	25	45,1%	51,4%	64,5%	70,6%	61,1%
731	20	40	40	30	45,5%	51,0%	65,3%	72,0%	62,7%
732	20	40	40	35	46,0%	50,7%	66,0%	73,4%	64,3%
733	20	40	40	40	46,4%	50,4%	66,8%	74,7%	65,8%
734	20	40	40	45	46,9%	50,1%	67,6%	76,0%	67,4%
735	20	40	40	50	47,3%	49,7%	68,3%	77,2%	68,8%
736	20	40	40	55	47,7%	49,4%	69,0%	78,4%	70,3%
737	20	40	40	60	48,2%	49,1%	69,7%	79,5%	71,7%
738	20	40	40	65	48,6%	48,8%	70,5%	80,6%	73,1%
739	20	40	40	70	49,1%	48,4%	71,2%	81,7%	74,4%
740	20	40	40	75	49,5%	48,1%	71,9%	82,7%	75,7%
741	20	40	40	80	49,9%	47,8%	72,5%	83,7%	76,9%
742	20	40	45	20	38,7%	45,9%	60,8%	67,8%	58,4%
743	20	40	45	25	39,1%	45,6%	61,6%	69,3%	60,1%
744	20	40	45	30	39,5%	45,3%	62,4%	70,7%	61,7%
745	20	40	45	35	39,9%	45,0%	63,2%	72,1%	63,3%
746	20	40	45	40	40,4%	44,6%	64,0%	73,5%	64,9%
747	20	40	45	45	40,8%	44,3%	64,8%	74,8%	66,4%
748	20	40	45	50	41,2%	44,0%	65,5%	76,1%	68,0%
749	20	40	45	55	41,6%	43,7%	66,3%	77,3%	69,4%
750	20	40	45	60	42,1%	43,3%	67,1%	78,5%	70,9%
751	20	40	45	65	42,5%	43,0%	67,8%	79,7%	72,3%
752	20	40	45	70	42,9%	42,7%	68,5%	80,8%	73,6%
753	20	40	45	75	43,4%	42,4%	69,3%	81,8%	74,9%
754	20	40	45	80	43,8%	42,1%	70,0%	82,8%	76,2%
755	20	40	50	20	33,0%	40,3%	57,8%	66,5%	57,4%
756	20	40	50	25	33,4%	40,0%	58,6%	68,0%	59,1%
757	20	40	50	30	33,8%	39,6%	59,5%	69,5%	60,7%
758	20	40	50	35	34,2%	39,3%	60,3%	70,9%	62,4%

759	20	40	50	40	34,6%	39,0%	61,1%	72,3%	64,0%
760	20	40	50	45	35,0%	38,7%	61,9%	73,7%	65,5%
761	20	40	50	50	35,4%	38,4%	62,7%	75,0%	67,0%
762	20	40	50	55	35,8%	38,1%	63,5%	76,3%	68,5%
763	20	40	50	60	36,2%	37,8%	64,3%	77,5%	70,0%
764	20	40	50	65	36,6%	37,5%	65,0%	78,7%	71,4%
765	20	40	50	70	37,0%	37,2%	65,8%	79,8%	72,8%
766	20	40	50	75	37,4%	36,9%	66,6%	80,9%	74,1%
767	20	40	50	80	37,8%	36,6%	67,3%	81,9%	75,4%
768	20	40	55	20	27,8%	34,9%	54,8%	65,1%	56,4%
769	20	40	55	25	28,1%	34,6%	55,6%	66,6%	58,1%
770	20	40	55	30	28,5%	34,3%	56,4%	68,2%	59,8%
771	20	40	55	35	28,9%	34,0%	57,3%	69,6%	61,4%
772	20	40	55	40	29,2%	33,7%	58,1%	71,1%	63,0%
773	20	40	55	45	29,6%	33,4%	58,9%	72,5%	64,6%
774	20	40	55	50	30,0%	33,1%	59,7%	73,8%	66,1%
775	20	40	55	55	30,3%	32,8%	60,6%	75,1%	67,6%
776	20	40	55	60	30,7%	32,5%	61,4%	76,4%	69,1%
777	20	40	55	65	31,1%	32,2%	62,2%	77,6%	70,6%
778	20	40	55	70	31,5%	32,0%	63,0%	78,8%	72,0%
779	20	40	55	75	31,8%	31,7%	63,7%	79,9%	73,3%
780	20	40	55	80	32,2%	31,4%	64,5%	81,0%	74,7%
781	20	40	60	20	23,1%	29,8%	51,7%	63,7%	55,4%
782	20	40	60	25	23,4%	29,5%	52,5%	65,3%	57,1%
783	20	40	60	30	23,8%	29,3%	53,4%	66,8%	58,8%
784	20	40	60	35	24,1%	29,0%	54,2%	68,3%	60,4%
785	20	40	60	40	24,4%	28,7%	55,0%	69,8%	62,0%
786	20	40	60	45	24,7%	28,5%	55,9%	71,2%	63,6%
787	20	40	60	50	25,1%	28,2%	56,7%	72,6%	65,2%
788	20	40	60	55	25,4%	27,9%	57,5%	74,0%	66,7%
789	20	40	60	60	25,7%	27,7%	58,4%	75,3%	68,2%
790	20	40	60	65	26,1%	27,4%	59,2%	76,5%	69,7%
791	20	40	60	70	26,4%	27,2%	60,0%	77,8%	71,1%

792	20	40	60	75	26,7%	26,9%	60,8%	78,9%	72,5%
793	20	40	60	80	27,1%	26,6%	61,6%	80,1%	73,9%
794	20	40	65	20	19,0%	25,2%	48,6%	62,3%	54,4%
795	20	40	65	25	19,3%	25,0%	49,4%	63,9%	56,1%
796	20	40	65	30	19,6%	24,7%	50,3%	65,4%	57,8%
797	20	40	65	35	19,9%	24,5%	51,1%	67,0%	59,4%
798	20	40	65	40	20,1%	24,2%	52,0%	68,5%	61,1%
799	20	40	65	45	20,4%	24,0%	52,8%	70,0%	62,7%
800	20	40	65	50	20,7%	23,8%	53,6%	71,4%	64,3%
801	20	40	65	55	21,0%	23,5%	54,5%	72,8%	65,8%
802	20	40	65	60	21,3%	23,3%	55,3%	74,1%	67,3%
803	20	40	65	65	21,6%	23,1%	56,2%	75,4%	68,8%
804	20	40	65	70	21,9%	22,8%	57,0%	76,7%	70,3%
805	20	40	65	75	22,2%	22,6%	57,8%	77,9%	71,7%
806	20	40	65	80	22,5%	22,4%	58,7%	79,1%	73,1%
807	20	40	70	20	15,5%	21,1%	45,5%	60,8%	53,4%
808	20	40	70	25	15,7%	20,9%	46,3%	62,5%	55,1%
809	20	40	70	30	16,0%	20,7%	47,2%	64,1%	56,7%
810	20	40	70	35	16,2%	20,5%	48,0%	65,6%	58,4%
811	20	40	70	40	16,5%	20,3%	48,8%	67,2%	60,1%
812	20	40	70	45	16,7%	20,0%	49,7%	68,7%	61,7%
813	20	40	70	50	16,9%	19,8%	50,5%	70,1%	63,3%
814	20	40	70	55	17,2%	19,6%	51,4%	71,6%	64,9%
815	20	40	70	60	17,4%	19,4%	52,2%	72,9%	66,4%
816	20	40	70	65	17,7%	19,2%	53,1%	74,3%	67,9%
817	20	40	70	70	18,0%	19,0%	53,9%	75,6%	69,4%
818	20	40	70	75	18,2%	18,8%	54,8%	76,8%	70,8%
819	20	40	70	80	18,5%	18,6%	55,6%	78,0%	72,2%
820	20	40	75	20	12,5%	17,5%	42,4%	59,4%	52,3%
821	20	40	75	25	12,7%	17,3%	43,2%	61,0%	54,0%
822	20	40	75	30	12,9%	17,2%	44,1%	62,6%	55,7%
823	20	40	75	35	13,1%	17,0%	44,9%	64,2%	57,4%
824	20	40	75	40	13,3%	16,8%	45,8%	65,8%	59,1%

825	20	40	75	45	13,5%	16,6%	46,6%	67,3%	60,7%
826	20	40	75	50	13,7%	16,4%	47,4%	68,8%	62,3%
827	20	40	75	55	14,0%	16,2%	48,3%	70,3%	63,9%
828	20	40	75	60	14,2%	16,1%	49,1%	71,7%	65,5%
829	20	40	75	65	14,4%	15,9%	50,0%	73,1%	67,0%
830	20	40	75	70	14,6%	15,7%	50,8%	74,4%	68,5%
831	20	40	75	75	14,8%	15,5%	51,7%	75,7%	70,0%
832	20	40	75	80	15,0%	15,4%	52,5%	77,0%	71,4%
833	20	40	80	20	10,1%	14,4%	39,4%	57,9%	51,3%
834	20	40	80	25	10,2%	14,3%	40,2%	59,6%	53,0%
835	20	40	80	30	10,4%	14,1%	41,0%	61,2%	54,7%
836	20	40	80	35	10,6%	14,0%	41,9%	62,8%	56,4%
837	20	40	80	40	10,7%	13,8%	42,7%	64,4%	58,1%
838	20	40	80	45	10,9%	13,6%	43,5%	66,0%	59,7%
839	20	40	80	50	11,1%	13,5%	44,4%	67,5%	61,4%
840	20	40	80	55	11,2%	13,3%	45,2%	69,0%	63,0%
841	20	40	80	60	11,4%	13,2%	46,0%	70,5%	64,6%
842	20	40	80	65	11,6%	13,0%	46,9%	71,9%	66,1%
843	20	40	80	70	11,8%	12,9%	47,7%	73,3%	67,6%
844	20	40	80	75	12,0%	12,7%	48,6%	74,6%	69,1%
845	20	40	80	80	12,2%	12,6%	49,4%	75,9%	70,6%
846	20	45	20	20	70,3%	74,5%	73,7%	72,6%	61,1%
847	20	45	20	25	70,7%	74,2%	74,3%	74,0%	62,7%
848	20	45	20	30	71,0%	74,0%	75,0%	75,3%	64,3%
849	20	45	20	35	71,4%	73,7%	75,6%	76,5%	65,9%
850	20	45	20	40	71,8%	73,5%	76,2%	77,8%	67,4%
851	20	45	20	45	72,1%	73,2%	76,8%	78,9%	68,9%
852	20	45	20	50	72,5%	72,9%	77,4%	80,0%	70,3%
853	20	45	20	55	72,8%	72,7%	78,0%	81,1%	71,7%
854	20	45	20	60	73,2%	72,4%	78,6%	82,2%	73,1%
855	20	45	20	65	73,5%	72,2%	79,2%	83,1%	74,4%
856	20	45	20	70	73,9%	71,9%	79,7%	84,1%	75,7%
857	20	45	20	75	74,2%	71,6%	80,3%	85,0%	76,9%

858	20	45	20	80	74,5%	71,4%	80,8%	85,9%	78,1%
859	20	45	25	20	64,9%	69,8%	71,2%	71,4%	60,1%
860	20	45	25	25	65,3%	69,6%	71,9%	72,8%	61,7%
861	20	45	25	30	65,7%	69,3%	72,6%	74,1%	63,3%
862	20	45	25	35	66,1%	69,0%	73,2%	75,4%	64,9%
863	20	45	25	40	66,5%	68,7%	73,9%	76,7%	66,5%
864	20	45	25	45	66,9%	68,4%	74,5%	77,9%	68,0%
865	20	45	25	50	67,3%	68,2%	75,2%	79,1%	69,4%
866	20	45	25	55	67,7%	67,9%	75,8%	80,2%	70,9%
867	20	45	25	60	68,1%	67,6%	76,4%	81,2%	72,3%
868	20	45	25	65	68,4%	67,3%	77,0%	82,3%	73,6%
869	20	45	25	70	68,8%	67,0%	77,6%	83,3%	74,9%
870	20	45	25	75	69,2%	66,7%	78,2%	84,2%	76,2%
871	20	45	25	80	69,6%	66,4%	78,8%	85,1%	77,4%
872	20	45	30	20	59,1%	64,8%	68,6%	70,1%	59,1%
873	20	45	30	25	59,5%	64,5%	69,3%	71,6%	60,8%
874	20	45	30	30	60,0%	64,2%	70,0%	72,9%	62,4%
875	20	45	30	35	60,4%	63,9%	70,7%	74,3%	64,0%
876	20	45	30	40	60,8%	63,6%	71,4%	75,6%	65,5%
877	20	45	30	45	61,2%	63,3%	72,1%	76,8%	67,1%
878	20	45	30	50	61,6%	62,9%	72,8%	78,0%	68,6%
879	20	45	30	55	62,1%	62,6%	73,5%	79,2%	70,0%
880	20	45	30	60	62,5%	62,3%	74,1%	80,3%	71,4%
881	20	45	30	65	62,9%	62,0%	74,8%	81,4%	72,8%
882	20	45	30	70	63,3%	61,7%	75,4%	82,4%	74,2%
883	20	45	30	75	63,7%	61,4%	76,0%	83,4%	75,4%
884	20	45	30	80	64,1%	61,1%	76,6%	84,3%	76,7%
885	20	45	35	20	53,0%	59,3%	65,8%	68,8%	58,1%
886	20	45	35	25	53,5%	59,0%	66,6%	70,3%	59,8%
887	20	45	35	30	53,9%	58,7%	67,3%	71,7%	61,4%
888	20	45	35	35	54,4%	58,4%	68,1%	73,1%	63,0%
889	20	45	35	40	54,8%	58,1%	68,8%	74,4%	64,6%
890	20	45	35	45	55,2%	57,7%	69,5%	75,7%	66,2%

891	20	45	35	50	55,7%	57,4%	70,3%	77,0%	67,7%
892	20	45	35	55	56,1%	57,1%	71,0%	78,2%	69,1%
893	20	45	35	60	56,5%	56,8%	71,7%	79,3%	70,6%
894	20	45	35	65	57,0%	56,5%	72,3%	80,4%	72,0%
895	20	45	35	70	57,4%	56,1%	73,0%	81,5%	73,4%
896	20	45	35	75	57,8%	55,8%	73,7%	82,5%	74,7%
897	20	45	35	80	58,3%	55,5%	74,3%	83,5%	75,9%
898	20	45	40	20	46,9%	53,7%	63,0%	67,5%	57,1%
899	20	45	40	25	47,3%	53,3%	63,8%	69,0%	58,8%
900	20	45	40	30	47,8%	53,0%	64,6%	70,5%	60,4%
901	20	45	40	35	48,2%	52,7%	65,3%	71,9%	62,1%
902	20	45	40	40	48,6%	52,4%	66,1%	73,3%	63,7%
903	20	45	40	45	49,1%	52,0%	66,9%	74,6%	65,2%
904	20	45	40	50	49,5%	51,7%	67,6%	75,9%	66,8%
905	20	45	40	55	50,0%	51,4%	68,3%	77,1%	68,3%
906	20	45	40	60	50,4%	51,0%	69,1%	78,3%	69,7%
907	20	45	40	65	50,8%	50,7%	69,8%	79,5%	71,2%
908	20	45	40	70	51,3%	50,4%	70,5%	80,6%	72,5%
909	20	45	40	75	51,7%	50,1%	71,2%	81,6%	73,9%
910	20	45	40	80	52,2%	49,7%	71,9%	82,6%	75,2%
911	20	45	45	20	40,8%	47,9%	60,0%	66,2%	56,1%
912	20	45	45	25	41,2%	47,6%	60,9%	67,7%	57,8%
913	20	45	45	30	41,7%	47,2%	61,7%	69,2%	59,4%
914	20	45	45	35	42,1%	46,9%	62,5%	70,6%	61,1%
915	20	45	45	40	42,5%	46,6%	63,3%	72,0%	62,7%
916	20	45	45	45	42,9%	46,3%	64,0%	73,4%	64,3%
917	20	45	45	50	43,4%	45,9%	64,8%	74,7%	65,8%
918	20	45	45	55	43,8%	45,6%	65,6%	76,0%	67,4%
919	20	45	45	60	44,2%	45,3%	66,4%	77,2%	68,8%
920	20	45	45	65	44,7%	45,0%	67,1%	78,4%	70,3%
921	20	45	45	70	45,1%	44,6%	67,9%	79,6%	71,7%
922	20	45	45	75	45,6%	44,3%	68,6%	80,7%	73,1%
923	20	45	45	80	46,0%	44,0%	69,3%	81,7%	74,4%

924	20	45	50	20	35,0%	42,2%	57,0%	64,8%	55,1%
925	20	45	50	25	35,4%	41,9%	57,9%	66,3%	56,8%
926	20	45	50	30	35,8%	41,5%	58,7%	67,9%	58,4%
927	20	45	50	35	36,2%	41,2%	59,5%	69,3%	60,1%
928	20	45	50	40	36,6%	40,9%	60,3%	70,8%	61,7%
929	20	45	50	45	37,0%	40,6%	61,1%	72,2%	63,3%
930	20	45	50	50	37,4%	40,3%	61,9%	73,6%	64,9%
931	20	45	50	55	37,9%	40,0%	62,7%	74,9%	66,4%
932	20	45	50	60	38,3%	39,7%	63,5%	76,2%	68,0%
933	20	45	50	65	38,7%	39,3%	64,3%	77,4%	69,4%
934	20	45	50	70	39,1%	39,0%	65,1%	78,6%	70,9%
935	20	45	50	75	39,5%	38,7%	65,9%	79,7%	72,3%
936	20	45	50	80	40,0%	38,4%	66,6%	80,8%	73,6%
937	20	45	55	20	29,6%	36,7%	54,0%	63,4%	54,1%
938	20	45	55	25	30,0%	36,4%	54,8%	65,0%	55,8%
939	20	45	55	30	30,4%	36,1%	55,6%	66,5%	57,4%
940	20	45	55	35	30,7%	35,8%	56,5%	68,0%	59,1%
941	20	45	55	40	31,1%	35,5%	57,3%	69,5%	60,7%
942	20	45	55	45	31,5%	35,2%	58,1%	71,0%	62,4%
943	20	45	55	50	31,9%	34,9%	59,0%	72,4%	64,0%
944	20	45	55	55	32,2%	34,6%	59,8%	73,7%	65,5%
945	20	45	55	60	32,6%	34,3%	60,6%	75,0%	67,1%
946	20	45	55	65	33,0%	34,0%	61,4%	76,3%	68,5%
947	20	45	55	70	33,4%	33,7%	62,2%	77,5%	70,0%
948	20	45	55	75	33,8%	33,4%	63,0%	78,7%	71,4%
949	20	45	55	80	34,2%	33,1%	63,8%	79,8%	72,8%
950	20	45	60	20	24,7%	31,5%	50,9%	62,0%	53,0%
951	20	45	60	25	25,1%	31,2%	51,7%	63,6%	54,7%
952	20	45	60	30	25,4%	30,9%	52,6%	65,1%	56,4%
953	20	45	60	35	25,7%	30,7%	53,4%	66,7%	58,1%
954	20	45	60	40	26,1%	30,4%	54,3%	68,2%	59,8%
955	20	45	60	45	26,4%	30,1%	55,1%	69,7%	61,4%
956	20	45	60	50	26,8%	29,8%	55,9%	71,1%	63,0%

957	20	45	60	55	27,1%	29,6%	56,8%	72,5%	64,6%
958	20	45	60	60	27,5%	29,3%	57,6%	73,9%	66,1%
959	20	45	60	65	27,8%	29,0%	58,4%	75,2%	67,7%
960	20	45	60	70	28,2%	28,7%	59,3%	76,4%	69,1%
961	20	45	60	75	28,5%	28,5%	60,1%	77,7%	70,6%
962	20	45	60	80	28,9%	28,2%	60,9%	78,8%	72,0%
963	20	45	65	20	20,4%	26,7%	47,8%	60,5%	52,0%
964	20	45	65	25	20,7%	26,5%	48,6%	62,1%	53,7%
965	20	45	65	30	21,0%	26,2%	49,5%	63,8%	55,4%
966	20	45	65	35	21,3%	26,0%	50,3%	65,3%	57,1%
967	20	45	65	40	21,6%	25,7%	51,2%	66,9%	58,8%
968	20	45	65	45	21,9%	25,5%	52,0%	68,4%	60,4%
969	20	45	65	50	22,2%	25,2%	52,9%	69,9%	62,0%
970	20	45	65	55	22,5%	25,0%	53,7%	71,3%	63,6%
971	20	45	65	60	22,8%	24,7%	54,5%	72,7%	65,2%
972	20	45	65	65	23,1%	24,5%	55,4%	74,0%	66,7%
973	20	45	65	70	23,4%	24,3%	56,2%	75,3%	68,2%
974	20	45	65	75	23,8%	24,0%	57,1%	76,6%	69,7%
975	20	45	65	80	24,1%	23,8%	57,9%	77,8%	71,1%
976	20	45	70	20	16,7%	22,5%	44,7%	59,0%	51,0%
977	20	45	70	25	17,0%	22,2%	45,5%	60,7%	52,7%
978	20	45	70	30	17,2%	22,0%	46,4%	62,3%	54,4%
979	20	45	70	35	17,5%	21,8%	47,2%	63,9%	56,1%
980	20	45	70	40	17,7%	21,6%	48,1%	65,5%	57,8%
981	20	45	70	45	18,0%	21,3%	48,9%	67,1%	59,4%
982	20	45	70	50	18,2%	21,1%	49,8%	68,6%	61,1%
983	20	45	70	55	18,5%	20,9%	50,6%	70,0%	62,7%
984	20	45	70	60	18,8%	20,7%	51,4%	71,5%	64,3%
985	20	45	70	65	19,0%	20,5%	52,3%	72,8%	65,8%
986	20	45	70	70	19,3%	20,3%	53,1%	74,2%	67,3%
987	20	45	70	75	19,6%	20,1%	54,0%	75,5%	68,8%
988	20	45	70	80	19,9%	19,8%	54,8%	76,7%	70,3%
989	20	45	75	20	13,5%	18,7%	41,6%	57,6%	49,9%

990	20	45	75	25	13,8%	18,5%	42,5%	59,2%	51,7%
991	20	45	75	30	14,0%	18,3%	43,3%	60,9%	53,4%
992	20	45	75	35	14,2%	18,1%	44,1%	62,5%	55,1%
993	20	45	75	40	14,4%	17,9%	45,0%	64,1%	56,7%
994	20	45	75	45	14,6%	17,7%	45,8%	65,7%	58,4%
995	20	45	75	50	14,8%	17,5%	46,7%	67,2%	60,1%
996	20	45	75	55	15,1%	17,3%	47,5%	68,7%	61,7%
997	20	45	75	60	15,3%	17,2%	48,3%	70,2%	63,3%
998	20	45	75	65	15,5%	17,0%	49,2%	71,6%	64,9%
999	20	45	75	70	15,7%	16,8%	50,0%	73,0%	66,4%
1000	20	45	75	75	16,0%	16,6%	50,9%	74,3%	67,9%
1001	20	45	75	80	16,2%	16,4%	51,7%	75,6%	69,4%
1002	20	45	80	20	10,9%	15,4%	38,6%	56,1%	48,9%
1003	20	45	80	25	11,1%	15,3%	39,5%	57,8%	50,6%
1004	20	45	80	30	11,3%	15,1%	40,3%	59,4%	52,3%
1005	20	45	80	35	11,4%	14,9%	41,1%	61,1%	54,0%
1006	20	45	80	40	11,6%	14,8%	41,9%	62,7%	55,7%
1007	20	45	80	45	11,8%	14,6%	42,7%	64,3%	57,4%
1008	20	45	80	50	12,0%	14,4%	43,6%	65,9%	59,1%
1009	20	45	80	55	12,2%	14,3%	44,4%	67,4%	60,7%
1010	20	45	80	60	12,4%	14,1%	45,3%	68,9%	62,3%
1011	20	45	80	65	12,5%	14,0%	46,1%	70,4%	63,9%
1012	20	45	80	70	12,7%	13,8%	46,9%	71,8%	65,5%
1013	20	45	80	75	12,9%	13,6%	47,8%	73,2%	67,0%
1014	20	45	80	80	13,1%	13,5%	48,6%	74,5%	68,5%
1015	20	50	20	20	72,1%	75,9%	73,0%	71,1%	58,8%
1016	20	50	20	25	72,5%	75,7%	73,7%	72,5%	60,5%
1017	20	50	20	30	72,8%	75,5%	74,4%	73,9%	62,1%
1018	20	50	20	35	73,2%	75,2%	75,0%	75,2%	63,7%
1019	20	50	20	40	73,5%	75,0%	75,6%	76,4%	65,2%
1020	20	50	20	45	73,9%	74,7%	76,3%	77,7%	66,8%
1021	20	50	20	50	74,2%	74,5%	76,9%	78,8%	68,3%
1022	20	50	20	55	74,5%	74,2%	77,5%	80,0%	69,7%

1023	20	50	20	60	74,9%	74,0%	78,0%	81,0%	71,2%
1024	20	50	20	65	75,2%	73,7%	78,6%	82,1%	72,6%
1025	20	50	20	70	75,5%	73,5%	79,2%	83,1%	73,9%
1026	20	50	20	75	75,9%	73,2%	79,7%	84,0%	75,2%
1027	20	50	20	80	76,2%	72,9%	80,3%	84,9%	76,5%
1028	20	50	25	20	66,9%	71,5%	70,5%	69,9%	57,8%
1029	20	50	25	25	67,3%	71,2%	71,2%	71,3%	59,5%
1030	20	50	25	30	67,7%	70,9%	71,9%	72,7%	61,1%
1031	20	50	25	35	68,1%	70,7%	72,6%	74,0%	62,7%
1032	20	50	25	40	68,5%	70,4%	73,3%	75,3%	64,3%
1033	20	50	25	45	68,8%	70,1%	73,9%	76,6%	65,9%
1034	20	50	25	50	69,2%	69,8%	74,6%	77,8%	67,4%
1035	20	50	25	55	69,6%	69,6%	75,2%	79,0%	68,9%
1036	20	50	25	60	70,0%	69,3%	75,8%	80,1%	70,3%
1037	20	50	25	65	70,3%	69,0%	76,5%	81,2%	71,7%
1038	20	50	25	70	70,7%	68,7%	77,1%	82,2%	73,1%
1039	20	50	25	75	71,1%	68,4%	77,7%	83,2%	74,4%
1040	20	50	25	80	71,4%	68,2%	78,2%	84,1%	75,7%
1041	20	50	30	20	61,2%	66,5%	67,9%	68,6%	56,8%
1042	20	50	30	25	61,7%	66,2%	68,6%	70,0%	58,5%
1043	20	50	30	30	62,1%	65,9%	69,3%	71,4%	60,1%
1044	20	50	30	35	62,5%	65,7%	70,1%	72,8%	61,7%
1045	20	50	30	40	62,9%	65,4%	70,8%	74,2%	63,4%
1046	20	50	30	45	63,3%	65,1%	71,5%	75,5%	64,9%
1047	20	50	30	50	63,7%	64,8%	72,2%	76,7%	66,5%
1048	20	50	30	55	64,1%	64,5%	72,8%	77,9%	68,0%
1049	20	50	30	60	64,5%	64,2%	73,5%	79,1%	69,5%
1050	20	50	30	65	64,9%	63,9%	74,2%	80,2%	70,9%
1051	20	50	30	70	65,3%	63,6%	74,8%	81,3%	72,3%
1052	20	50	30	75	65,7%	63,3%	75,4%	82,3%	73,6%
1053	20	50	30	80	66,1%	63,0%	76,1%	83,3%	74,9%
1054	20	50	35	20	55,2%	61,2%	65,1%	67,2%	55,8%
1055	20	50	35	25	55,7%	60,9%	65,9%	68,7%	57,5%

1056	20	50	35	30	56,1%	60,6%	66,6%	70,2%	59,1%
1057	20	50	35	35	56,5%	60,3%	67,4%	71,6%	60,8%
1058	20	50	35	40	57,0%	60,0%	68,1%	73,0%	62,4%
1059	20	50	35	45	57,4%	59,6%	68,9%	74,3%	64,0%
1060	20	50	35	50	57,8%	59,3%	69,6%	75,6%	65,5%
1061	20	50	35	55	58,3%	59,0%	70,3%	76,9%	67,1%
1062	20	50	35	60	58,7%	58,7%	71,0%	78,1%	68,6%
1063	20	50	35	65	59,1%	58,4%	71,7%	79,2%	70,0%
1064	20	50	35	70	59,5%	58,1%	72,4%	80,3%	71,4%
1065	20	50	35	75	60,0%	57,7%	73,1%	81,4%	72,8%
1066	20	50	35	80	60,4%	57,4%	73,7%	82,4%	74,2%
1067	20	50	40	20	49,1%	55,6%	62,2%	65,9%	54,8%
1068	20	50	40	25	49,5%	55,3%	63,0%	67,4%	56,4%
1069	20	50	40	30	50,0%	55,0%	63,8%	68,9%	58,1%
1070	20	50	40	35	50,4%	54,6%	64,6%	70,4%	59,8%
1071	20	50	40	40	50,8%	54,3%	65,4%	71,8%	61,4%
1072	20	50	40	45	51,3%	54,0%	66,1%	73,2%	63,0%
1073	20	50	40	50	51,7%	53,7%	66,9%	74,5%	64,6%
1074	20	50	40	55	52,2%	53,3%	67,6%	75,8%	66,2%
1075	20	50	40	60	52,6%	53,0%	68,4%	77,0%	67,7%
1076	20	50	40	65	53,0%	52,7%	69,1%	78,2%	69,2%
1077	20	50	40	70	53,5%	52,4%	69,8%	79,4%	70,6%
1078	20	50	40	75	53,9%	52,0%	70,5%	80,5%	72,0%
1079	20	50	40	80	54,4%	51,7%	71,2%	81,5%	73,4%
1080	20	50	45	20	43,0%	49,9%	59,3%	64,5%	53,7%
1081	20	50	45	25	43,4%	49,5%	60,1%	66,0%	55,4%
1082	20	50	45	30	43,8%	49,2%	60,9%	67,6%	57,1%
1083	20	50	45	35	44,3%	48,9%	61,7%	69,1%	58,8%
1084	20	50	45	40	44,7%	48,6%	62,5%	70,5%	60,4%
1085	20	50	45	45	45,1%	48,2%	63,3%	71,9%	62,1%
1086	20	50	45	50	45,6%	47,9%	64,1%	73,3%	63,7%
1087	20	50	45	55	46,0%	47,6%	64,9%	74,6%	65,2%
1088	20	50	45	60	46,4%	47,2%	65,6%	75,9%	66,8%

1089	20	50	45	65	46,9%	46,9%	66,4%	77,2%	68,3%
1090	20	50	45	70	47,3%	46,6%	67,2%	78,4%	69,7%
1091	20	50	45	75	47,8%	46,3%	67,9%	79,5%	71,2%
1092	20	50	45	80	48,2%	45,9%	68,6%	80,6%	72,5%
1093	20	50	50	20	37,0%	44,1%	56,3%	63,1%	52,7%
1094	20	50	50	25	37,5%	43,8%	57,1%	64,7%	54,4%
1095	20	50	50	30	37,9%	43,5%	57,9%	66,2%	56,1%
1096	20	50	50	35	38,3%	43,1%	58,7%	67,8%	57,8%
1097	20	50	50	40	38,7%	42,8%	59,6%	69,2%	59,4%
1098	20	50	50	45	39,1%	42,5%	60,4%	70,7%	61,1%
1099	20	50	50	50	39,5%	42,2%	61,2%	72,1%	62,7%
1100	20	50	50	55	40,0%	41,9%	62,0%	73,5%	64,3%
1101	20	50	50	60	40,4%	41,6%	62,8%	74,8%	65,8%
1102	20	50	50	65	40,8%	41,2%	63,6%	76,1%	67,4%
1103	20	50	50	70	41,2%	40,9%	64,4%	77,3%	68,9%
1104	20	50	50	75	41,7%	40,6%	65,1%	78,5%	70,3%
1105	20	50	50	80	42,1%	40,3%	65,9%	79,6%	71,7%
1106	20	50	55	20	31,5%	38,5%	53,2%	61,6%	51,7%
1107	20	50	55	25	31,9%	38,2%	54,0%	63,3%	53,4%
1108	20	50	55	30	32,3%	37,9%	54,9%	64,9%	55,1%
1109	20	50	55	35	32,6%	37,6%	55,7%	66,4%	56,8%
1110	20	50	55	40	33,0%	37,3%	56,5%	67,9%	58,4%
1111	20	50	55	45	33,4%	37,0%	57,4%	69,4%	60,1%
1112	20	50	55	50	33,8%	36,7%	58,2%	70,9%	61,7%
1113	20	50	55	55	34,2%	36,4%	59,0%	72,3%	63,3%
1114	20	50	55	60	34,6%	36,1%	59,8%	73,6%	64,9%
1115	20	50	55	65	35,0%	35,8%	60,7%	74,9%	66,5%
1116	20	50	55	70	35,4%	35,5%	61,5%	76,2%	68,0%
1117	20	50	55	75	35,8%	35,2%	62,3%	77,4%	69,4%
1118	20	50	55	80	36,2%	34,9%	63,1%	78,6%	70,9%
1119	20	50	60	20	26,4%	33,2%	50,1%	60,2%	50,6%
1120	20	50	60	25	26,8%	32,9%	50,9%	61,8%	52,4%
1121	20	50	60	30	27,1%	32,6%	51,8%	63,4%	54,1%

1122	20	50	60	35	27,5%	32,4%	52,6%	65,0%	55,8%
1123	20	50	60	40	27,8%	32,1%	53,5%	66,6%	57,4%
1124	20	50	60	45	28,2%	31,8%	54,3%	68,1%	59,1%
1125	20	50	60	50	28,5%	31,5%	55,2%	69,6%	60,8%
1126	20	50	60	55	28,9%	31,2%	56,0%	71,0%	62,4%
1127	20	50	60	60	29,3%	30,9%	56,8%	72,4%	64,0%
1128	20	50	60	65	29,6%	30,7%	57,7%	73,8%	65,5%
1129	20	50	60	70	30,0%	30,4%	58,5%	75,1%	67,1%
1130	20	50	60	75	30,4%	30,1%	59,3%	76,4%	68,6%
1131	20	50	60	80	30,7%	29,8%	60,1%	77,6%	70,0%
1132	20	50	65	20	21,9%	28,3%	47,0%	58,7%	49,6%
1133	20	50	65	25	22,2%	28,0%	47,8%	60,4%	51,3%
1134	20	50	65	30	22,5%	27,8%	48,7%	62,0%	53,0%
1135	20	50	65	35	22,8%	27,5%	49,5%	63,6%	54,7%
1136	20	50	65	40	23,1%	27,3%	50,4%	65,2%	56,4%
1137	20	50	65	45	23,5%	27,0%	51,2%	66,8%	58,1%
1138	20	50	65	50	23,8%	26,7%	52,1%	68,3%	59,8%
1139	20	50	65	55	24,1%	26,5%	52,9%	69,7%	61,4%
1140	20	50	65	60	24,4%	26,2%	53,8%	71,2%	63,0%
1141	20	50	65	65	24,7%	26,0%	54,6%	72,6%	64,6%
1142	20	50	65	70	25,1%	25,7%	55,4%	73,9%	66,1%
1143	20	50	65	75	25,4%	25,5%	56,3%	75,2%	67,7%
1144	20	50	65	80	25,7%	25,2%	57,1%	76,5%	69,1%
1145	20	50	70	20	18,0%	23,9%	43,9%	57,2%	48,6%
1146	20	50	70	25	18,2%	23,6%	44,7%	58,9%	50,3%
1147	20	50	70	30	18,5%	23,4%	45,6%	60,6%	52,0%
1148	20	50	70	35	18,8%	23,2%	46,4%	62,2%	53,7%
1149	20	50	70	40	19,0%	22,9%	47,3%	63,8%	55,4%
1150	20	50	70	45	19,3%	22,7%	48,1%	65,4%	57,1%
1151	20	50	70	50	19,6%	22,5%	49,0%	66,9%	58,8%
1152	20	50	70	55	19,9%	22,2%	49,8%	68,4%	60,4%
1153	20	50	70	60	20,2%	22,0%	50,7%	69,9%	62,0%
1154	20	50	70	65	20,4%	21,8%	51,5%	71,3%	63,6%

1155	20	50	70	70	20,7%	21,6%	52,4%	72,7%	65,2%
1156	20	50	70	75	21,0%	21,3%	53,2%	74,1%	66,7%
1157	20	50	70	80	21,3%	21,1%	54,0%	75,4%	68,3%
1158	20	50	75	20	14,6%	19,9%	40,9%	55,7%	47,6%
1159	20	50	75	25	14,8%	19,7%	41,7%	57,4%	49,3%
1160	20	50	75	30	15,1%	19,5%	42,5%	59,1%	51,0%
1161	20	50	75	35	15,3%	19,3%	43,3%	60,8%	52,7%
1162	20	50	75	40	15,5%	19,1%	44,2%	62,4%	54,4%
1163	20	50	75	45	15,8%	18,9%	45,0%	64,0%	56,1%
1164	20	50	75	50	16,0%	18,7%	45,9%	65,6%	57,8%
1165	20	50	75	55	16,2%	18,5%	46,7%	67,1%	59,4%
1166	20	50	75	60	16,5%	18,3%	47,6%	68,6%	61,1%
1167	20	50	75	65	16,7%	18,1%	48,4%	70,1%	62,7%
1168	20	50	75	70	17,0%	17,9%	49,2%	71,5%	64,3%
1169	20	50	75	75	17,2%	17,7%	50,1%	72,9%	65,8%
1170	20	50	75	80	17,5%	17,5%	50,9%	74,2%	67,4%
1171	20	50	80	20	11,8%	16,5%	37,9%	54,2%	46,5%
1172	20	50	80	25	12,0%	16,3%	38,7%	55,9%	48,2%
1173	20	50	80	30	12,2%	16,1%	39,5%	57,6%	49,9%
1174	20	50	80	35	12,4%	16,0%	40,3%	59,3%	51,7%
1175	20	50	80	40	12,6%	15,8%	41,1%	61,0%	53,4%
1176	20	50	80	45	12,7%	15,6%	42,0%	62,6%	55,1%
1177	20	50	80	50	12,9%	15,4%	42,8%	64,2%	56,8%
1178	20	50	80	55	13,1%	15,3%	43,6%	65,8%	58,4%
1179	20	50	80	60	13,3%	15,1%	44,5%	67,3%	60,1%
1180	20	50	80	65	13,6%	14,9%	45,3%	68,8%	61,7%
1181	20	50	80	70	13,8%	14,8%	46,1%	70,3%	63,3%
1182	20	50	80	75	14,0%	14,6%	47,0%	71,7%	64,9%
1183	20	50	80	80	14,2%	14,4%	47,8%	73,1%	66,4%
1184	20	55	20	20	73,9%	77,3%	72,4%	69,6%	56,5%
1185	20	55	20	25	74,2%	77,1%	73,1%	71,0%	58,1%
1186	20	55	20	30	74,6%	76,9%	73,7%	72,4%	59,8%
1187	20	55	20	35	74,9%	76,6%	74,4%	73,8%	61,4%

1188	20	55	20	40	75,2%	76,4%	75,0%	75,1%	63,0%
1189	20	55	20	45	75,5%	76,2%	75,7%	76,4%	64,6%
1190	20	55	20	50	75,9%	75,9%	76,3%	77,6%	66,2%
1191	20	55	20	55	76,2%	75,7%	76,9%	78,8%	67,7%
1192	20	55	20	60	76,5%	75,5%	77,5%	79,9%	69,2%
1193	20	55	20	65	76,8%	75,2%	78,1%	81,0%	70,6%
1194	20	55	20	70	77,1%	75,0%	78,7%	82,0%	72,0%
1195	20	55	20	75	77,4%	74,7%	79,2%	83,0%	73,4%
1196	20	55	20	80	77,8%	74,5%	79,8%	84,0%	74,7%
1197	20	55	25	20	68,8%	73,0%	69,9%	68,3%	55,5%
1198	20	55	25	25	69,2%	72,8%	70,6%	69,7%	57,1%
1199	20	55	25	30	69,6%	72,5%	71,3%	71,2%	58,8%
1200	20	55	25	35	70,0%	72,3%	72,0%	72,6%	60,5%
1201	20	55	25	40	70,3%	72,0%	72,6%	73,9%	62,1%
1202	20	55	25	45	70,7%	71,7%	73,3%	75,2%	63,7%
1203	20	55	25	50	71,1%	71,5%	74,0%	76,5%	65,3%
1204	20	55	25	55	71,4%	71,2%	74,6%	77,7%	66,8%
1205	20	55	25	60	71,8%	70,9%	75,3%	78,9%	68,3%
1206	20	55	25	65	72,1%	70,7%	75,9%	80,0%	69,8%
1207	20	55	25	70	72,5%	70,4%	76,5%	81,1%	71,2%
1208	20	55	25	75	72,8%	70,1%	77,1%	82,1%	72,6%
1209	20	55	25	80	73,2%	69,8%	77,7%	83,1%	73,9%
1210	20	55	30	20	63,3%	68,3%	67,2%	66,9%	54,4%
1211	20	55	30	25	63,7%	68,0%	67,9%	68,4%	56,1%
1212	20	55	30	30	64,1%	67,7%	68,7%	69,9%	57,8%
1213	20	55	30	35	64,5%	67,4%	69,4%	71,3%	59,5%
1214	20	55	30	40	64,9%	67,1%	70,1%	72,7%	61,1%
1215	20	55	30	45	65,3%	66,8%	70,8%	74,1%	62,7%
1216	20	55	30	50	65,7%	66,5%	71,5%	75,4%	64,3%
1217	20	55	30	55	66,1%	66,2%	72,2%	76,6%	65,9%
1218	20	55	30	60	66,5%	66,0%	72,9%	77,9%	67,4%
1219	20	55	30	65	66,9%	65,7%	73,5%	79,0%	68,9%
1220	20	55	30	70	67,3%	65,4%	74,2%	80,1%	70,3%

1221	20	55	30	75	67,7%	65,1%	74,8%	81,2%	71,7%
1222	20	55	30	80	68,1%	64,8%	75,5%	82,2%	73,1%
1223	20	55	35	20	57,4%	63,1%	64,4%	65,6%	53,4%
1224	20	55	35	25	57,9%	62,8%	65,2%	67,1%	55,1%
1225	20	55	35	30	58,3%	62,5%	65,9%	68,6%	56,8%
1226	20	55	35	35	58,7%	62,1%	66,7%	70,1%	58,5%
1227	20	55	35	40	59,1%	61,8%	67,4%	71,5%	60,1%
1228	20	55	35	45	59,6%	61,5%	68,2%	72,9%	61,8%
1229	20	55	35	50	60,0%	61,2%	68,9%	74,2%	63,4%
1230	20	55	35	55	60,4%	60,9%	69,6%	75,5%	64,9%
1231	20	55	35	60	60,8%	60,6%	70,4%	76,8%	66,5%
1232	20	55	35	65	61,2%	60,3%	71,1%	78,0%	68,0%
1233	20	55	35	70	61,7%	60,0%	71,7%	79,2%	69,5%
1234	20	55	35	75	62,1%	59,7%	72,4%	80,3%	70,9%
1235	20	55	35	80	62,5%	59,3%	73,1%	81,3%	72,3%
1236	20	55	40	20	51,3%	57,5%	61,5%	64,2%	52,4%
1237	20	55	40	25	51,7%	57,2%	62,3%	65,8%	54,1%
1238	20	55	40	30	52,2%	56,9%	63,1%	67,3%	55,8%
1239	20	55	40	35	52,6%	56,6%	63,9%	68,8%	57,5%
1240	20	55	40	40	53,1%	56,3%	64,7%	70,2%	59,1%
1241	20	55	40	45	53,5%	55,9%	65,4%	71,7%	60,8%
1242	20	55	40	50	53,9%	55,6%	66,2%	73,0%	62,4%
1243	20	55	40	55	54,4%	55,3%	66,9%	74,4%	64,0%
1244	20	55	40	60	54,8%	55,0%	67,7%	75,7%	65,6%
1245	20	55	40	65	55,3%	54,6%	68,4%	76,9%	67,1%
1246	20	55	40	70	55,7%	54,3%	69,2%	78,1%	68,6%
1247	20	55	40	75	56,1%	54,0%	69,9%	79,3%	70,0%
1248	20	55	40	80	56,6%	53,7%	70,6%	80,4%	71,4%
1249	20	55	45	20	45,1%	51,8%	58,5%	62,8%	51,4%
1250	20	55	45	25	45,6%	51,5%	59,3%	64,4%	53,1%
1251	20	55	45	30	46,0%	51,2%	60,2%	65,9%	54,8%
1252	20	55	45	35	46,5%	50,8%	61,0%	67,5%	56,5%
1253	20	55	45	40	46,9%	50,5%	61,8%	69,0%	58,1%

1254	20	55	45	45	47,3%	50,2%	62,6%	70,4%	59,8%
1255	20	55	45	50	47,8%	49,9%	63,4%	71,8%	61,4%
1256	20	55	45	55	48,2%	49,5%	64,1%	73,2%	63,0%
1257	20	55	45	60	48,7%	49,2%	64,9%	74,5%	64,6%
1258	20	55	45	65	49,1%	48,9%	65,7%	75,8%	66,2%
1259	20	55	45	70	49,5%	48,6%	66,5%	77,1%	67,7%
1260	20	55	45	75	50,0%	48,2%	67,2%	78,3%	69,2%
1261	20	55	45	80	50,4%	47,9%	67,9%	79,4%	70,6%
1262	20	55	50	20	39,1%	46,1%	55,5%	61,3%	50,3%
1263	20	55	50	25	39,6%	45,7%	56,3%	63,0%	52,0%
1264	20	55	50	30	40,0%	45,4%	57,1%	64,6%	53,7%
1265	20	55	50	35	40,4%	45,1%	58,0%	66,1%	55,4%
1266	20	55	50	40	40,8%	44,8%	58,8%	67,6%	57,1%
1267	20	55	50	45	41,3%	44,4%	59,6%	69,1%	58,8%
1268	20	55	50	50	41,7%	44,1%	60,4%	70,6%	60,4%
1269	20	55	50	55	42,1%	43,8%	61,2%	72,0%	62,1%
1270	20	55	50	60	42,5%	43,5%	62,0%	73,4%	63,7%
1271	20	55	50	65	43,0%	43,2%	62,8%	74,7%	65,2%
1272	20	55	50	70	43,4%	42,8%	63,6%	76,0%	66,8%
1273	20	55	50	75	43,8%	42,5%	64,4%	77,2%	68,3%
1274	20	55	50	80	44,3%	42,2%	65,2%	78,4%	69,7%
1275	20	55	55	20	33,4%	40,4%	52,4%	59,9%	49,3%
1276	20	55	55	25	33,8%	40,1%	53,2%	61,5%	51,0%
1277	20	55	55	30	34,2%	39,8%	54,1%	63,1%	52,7%
1278	20	55	55	35	34,6%	39,5%	54,9%	64,7%	54,4%
1279	20	55	55	40	35,0%	39,1%	55,8%	66,3%	56,1%
1280	20	55	55	45	35,4%	38,8%	56,6%	67,8%	57,8%
1281	20	55	55	50	35,8%	38,5%	57,4%	69,3%	59,5%
1282	20	55	55	55	36,2%	38,2%	58,3%	70,7%	61,1%
1283	20	55	55	60	36,6%	37,9%	59,1%	72,2%	62,7%
1284	20	55	55	65	37,1%	37,6%	59,9%	73,5%	64,3%
1285	20	55	55	70	37,5%	37,3%	60,7%	74,8%	65,8%
1286	20	55	55	75	37,9%	37,0%	61,5%	76,1%	67,4%

1287	20	55	55	80	38,3%	36,7%	62,3%	77,3%	68,9%
1288	20	55	60	20	28,2%	35,0%	49,3%	58,4%	48,3%
1289	20	55	60	25	28,5%	34,7%	50,1%	60,1%	50,0%
1290	20	55	60	30	28,9%	34,4%	51,0%	61,7%	51,7%
1291	20	55	60	35	29,3%	34,1%	51,8%	63,3%	53,4%
1292	20	55	60	40	29,6%	33,8%	52,7%	64,9%	55,1%
1293	20	55	60	45	30,0%	33,5%	53,5%	66,5%	56,8%
1294	20	55	60	50	30,4%	33,2%	54,4%	68,0%	58,5%
1295	20	55	60	55	30,7%	32,9%	55,2%	69,5%	60,1%
1296	20	55	60	60	31,1%	32,6%	56,0%	70,9%	61,7%
1297	20	55	60	65	31,5%	32,4%	56,9%	72,3%	63,3%
1298	20	55	60	70	31,9%	32,1%	57,7%	73,7%	64,9%
1299	20	55	60	75	32,3%	31,8%	58,5%	75,0%	66,5%
1300	20	55	60	80	32,7%	31,5%	59,4%	76,3%	68,0%
1301	20	55	65	20	23,5%	29,9%	46,2%	56,9%	47,2%
1302	20	55	65	25	23,8%	29,7%	47,0%	58,6%	48,9%
1303	20	55	65	30	24,1%	29,4%	47,9%	60,3%	50,7%
1304	20	55	65	35	24,4%	29,1%	48,7%	61,9%	52,4%
1305	20	55	65	40	24,8%	28,8%	49,6%	63,5%	54,1%
1306	20	55	65	45	25,1%	28,6%	50,4%	65,1%	55,8%
1307	20	55	65	50	25,4%	28,3%	51,3%	66,6%	57,4%
1308	20	55	65	55	25,8%	28,0%	52,1%	68,2%	59,1%
1309	20	55	65	60	26,1%	27,8%	53,0%	69,6%	60,8%
1310	20	55	65	65	26,4%	27,5%	53,8%	71,1%	62,4%
1311	20	55	65	70	26,8%	27,3%	54,7%	72,5%	64,0%
1312	20	55	65	75	27,1%	27,0%	55,5%	73,8%	65,5%
1313	20	55	65	80	27,5%	26,7%	56,3%	75,1%	67,1%
1314	20	55	70	20	19,3%	25,3%	43,1%	55,4%	46,2%
1315	20	55	70	25	19,6%	25,1%	43,9%	57,1%	47,9%
1316	20	55	70	30	19,9%	24,8%	44,8%	58,8%	49,6%
1317	20	55	70	35	20,2%	24,6%	45,6%	60,5%	51,3%
1318	20	55	70	40	20,4%	24,3%	46,5%	62,1%	53,0%
1319	20	55	70	45	20,7%	24,1%	47,3%	63,7%	54,7%

1320	20	55	70	50	21,0%	23,9%	48,2%	65,3%	56,4%
1321	20	55	70	55	21,3%	23,6%	49,0%	66,8%	58,1%
1322	20	55	70	60	21,6%	23,4%	49,9%	68,3%	59,8%
1323	20	55	70	65	21,9%	23,2%	50,7%	69,8%	61,4%
1324	20	55	70	70	22,2%	22,9%	51,6%	71,2%	63,0%
1325	20	55	70	75	22,5%	22,7%	52,4%	72,6%	64,6%
1326	20	55	70	80	22,8%	22,5%	53,3%	74,0%	66,1%
1327	20	55	75	20	15,8%	21,2%	40,1%	53,9%	45,2%
1328	20	55	75	25	16,0%	21,0%	40,9%	55,6%	46,9%
1329	20	55	75	30	16,2%	20,8%	41,7%	57,3%	48,6%
1330	20	55	75	35	16,5%	20,6%	42,6%	59,0%	50,3%
1331	20	55	75	40	16,7%	20,3%	43,4%	60,6%	52,0%
1332	20	55	75	45	17,0%	20,1%	44,2%	62,3%	53,7%
1333	20	55	75	50	17,2%	19,9%	45,1%	63,9%	55,4%
1334	20	55	75	55	17,5%	19,7%	45,9%	65,5%	57,1%
1335	20	55	75	60	17,7%	19,5%	46,8%	67,0%	58,8%
1336	20	55	75	65	18,0%	19,3%	47,6%	68,5%	60,4%
1337	20	55	75	70	18,2%	19,1%	48,5%	70,0%	62,0%
1338	20	55	75	75	18,5%	18,9%	49,3%	71,4%	63,6%
1339	20	55	75	80	18,8%	18,7%	50,2%	72,8%	65,2%
1340	20	55	80	20	12,8%	17,6%	37,2%	52,4%	44,2%
1341	20	55	80	25	13,0%	17,4%	37,9%	54,1%	45,9%
1342	20	55	80	30	13,2%	17,2%	38,8%	55,8%	47,6%
1343	20	55	80	35	13,4%	17,0%	39,6%	57,5%	49,3%
1344	20	55	80	40	13,6%	16,9%	40,4%	59,2%	51,0%
1345	20	55	80	45	13,8%	16,7%	41,2%	60,8%	52,7%
1346	20	55	80	50	14,0%	16,5%	42,0%	62,5%	54,4%
1347	20	55	80	55	14,2%	16,3%	42,8%	64,1%	56,1%
1348	20	55	80	60	14,4%	16,1%	43,7%	65,6%	57,8%
1349	20	55	80	65	14,6%	16,0%	44,5%	67,2%	59,4%
1350	20	55	80	70	14,8%	15,8%	45,4%	68,7%	61,1%
1351	20	55	80	75	15,1%	15,6%	46,2%	70,1%	62,7%
1352	20	55	80	80	15,3%	15,4%	47,0%	71,6%	64,3%

1353	20	60	20	20	75,6%	78,7%	71,8%	68,0%	54,1%
1354	20	60	20	25	75,9%	78,5%	72,5%	69,5%	55,8%
1355	20	60	20	30	76,2%	78,3%	73,1%	70,9%	57,5%
1356	20	60	20	35	76,5%	78,0%	73,8%	72,3%	59,2%
1357	20	60	20	40	76,8%	77,8%	74,4%	73,7%	60,8%
1358	20	60	20	45	77,1%	77,6%	75,1%	75,0%	62,4%
1359	20	60	20	50	77,5%	77,3%	75,7%	76,3%	64,0%
1360	20	60	20	55	77,8%	77,1%	76,3%	77,5%	65,6%
1361	20	60	20	60	78,1%	76,9%	76,9%	78,7%	67,1%
1362	20	60	20	65	78,4%	76,7%	77,5%	79,8%	68,6%
1363	20	60	20	70	78,7%	76,4%	78,1%	80,9%	70,1%
1364	20	60	20	75	79,0%	76,2%	78,7%	81,9%	71,5%
1365	20	60	20	80	79,3%	75,9%	79,3%	82,9%	72,8%
1366	20	60	25	20	70,7%	74,6%	69,2%	66,6%	53,1%
1367	20	60	25	25	71,1%	74,3%	69,9%	68,2%	54,8%
1368	20	60	25	30	71,4%	74,1%	70,6%	69,6%	56,5%
1369	20	60	25	35	71,8%	73,8%	71,3%	71,1%	58,2%
1370	20	60	25	40	72,2%	73,6%	72,0%	72,5%	59,8%
1371	20	60	25	45	72,5%	73,3%	72,7%	73,8%	61,4%
1372	20	60	25	50	72,9%	73,0%	73,4%	75,1%	63,1%
1373	20	60	25	55	73,2%	72,8%	74,0%	76,4%	64,6%
1374	20	60	25	60	73,5%	72,5%	74,7%	77,6%	66,2%
1375	20	60	25	65	73,9%	72,3%	75,3%	78,8%	67,7%
1376	20	60	25	70	74,2%	72,0%	75,9%	79,9%	69,2%
1377	20	60	25	75	74,6%	71,7%	76,5%	81,0%	70,6%
1378	20	60	25	80	74,9%	71,5%	77,1%	82,1%	72,0%
1379	20	60	30	20	65,4%	69,9%	66,5%	65,3%	52,1%
1380	20	60	30	25	65,8%	69,7%	67,2%	66,8%	53,8%
1381	20	60	30	30	66,2%	69,4%	68,0%	68,3%	55,5%
1382	20	60	30	35	66,5%	69,1%	68,7%	69,8%	57,1%
1383	20	60	30	40	66,9%	68,8%	69,4%	71,2%	58,8%
1384	20	60	30	45	67,3%	68,6%	70,2%	72,6%	60,5%
1385	20	60	30	50	67,7%	68,3%	70,9%	74,0%	62,1%

1386	20	60	30	55	68,1%	68,0%	71,6%	75,3%	63,7%
1387	20	60	30	60	68,5%	67,7%	72,2%	76,5%	65,3%
1388	20	60	30	65	68,9%	67,4%	72,9%	77,8%	66,8%
1389	20	60	30	70	69,2%	67,1%	73,6%	78,9%	68,3%
1390	20	60	30	75	69,6%	66,8%	74,2%	80,1%	69,8%
1391	20	60	30	80	70,0%	66,5%	74,9%	81,1%	71,2%
1392	20	60	35	20	59,6%	64,9%	63,7%	63,9%	51,0%
1393	20	60	35	25	60,0%	64,6%	64,4%	65,5%	52,7%
1394	20	60	35	30	60,4%	64,3%	65,2%	67,0%	54,4%
1395	20	60	35	35	60,8%	64,0%	66,0%	68,5%	56,1%
1396	20	60	35	40	61,3%	63,7%	66,7%	70,0%	57,8%
1397	20	60	35	45	61,7%	63,4%	67,5%	71,4%	59,5%
1398	20	60	35	50	62,1%	63,1%	68,2%	72,8%	61,1%
1399	20	60	35	55	62,5%	62,8%	69,0%	74,1%	62,7%
1400	20	60	35	60	62,9%	62,5%	69,7%	75,4%	64,3%
1401	20	60	35	65	63,3%	62,2%	70,4%	76,7%	65,9%
1402	20	60	35	70	63,7%	61,8%	71,1%	77,9%	67,4%
1403	20	60	35	75	64,1%	61,5%	71,8%	79,1%	68,9%
1404	20	60	35	80	64,6%	61,2%	72,5%	80,2%	70,3%
1405	20	60	40	20	53,5%	59,5%	60,7%	62,5%	50,0%
1406	20	60	40	25	54,0%	59,1%	61,5%	64,1%	51,7%
1407	20	60	40	30	54,4%	58,8%	62,3%	65,6%	53,4%
1408	20	60	40	35	54,8%	58,5%	63,1%	67,2%	55,1%
1409	20	60	40	40	55,3%	58,2%	63,9%	68,7%	56,8%
1410	20	60	40	45	55,7%	57,9%	64,7%	70,1%	58,5%
1411	20	60	40	50	56,1%	57,6%	65,5%	71,6%	60,1%
1412	20	60	40	55	56,6%	57,2%	66,2%	72,9%	61,8%
1413	20	60	40	60	57,0%	56,9%	67,0%	74,3%	63,4%
1414	20	60	40	65	57,4%	56,6%	67,7%	75,6%	64,9%
1415	20	60	40	70	57,9%	56,3%	68,5%	76,8%	66,5%
1416	20	60	40	75	58,3%	55,9%	69,2%	78,0%	68,0%
1417	20	60	40	80	58,7%	55,6%	69,9%	79,2%	69,5%
1418	20	60	45	20	47,4%	53,8%	57,7%	61,0%	49,0%

1419	20	60	45	25	47,8%	53,5%	58,6%	62,7%	50,7%
1420	20	60	45	30	48,2%	53,1%	59,4%	64,2%	52,4%
1421	20	60	45	35	48,7%	52,8%	60,2%	65,8%	54,1%
1422	20	60	45	40	49,1%	52,5%	61,0%	67,4%	55,8%
1423	20	60	45	45	49,6%	52,2%	61,8%	68,9%	57,5%
1424	20	60	45	50	50,0%	51,8%	62,6%	70,3%	59,1%
1425	20	60	45	55	50,4%	51,5%	63,4%	71,7%	60,8%
1426	20	60	45	60	50,9%	51,2%	64,2%	73,1%	62,4%
1427	20	60	45	65	51,3%	50,9%	65,0%	74,4%	64,0%
1428	20	60	45	70	51,8%	50,5%	65,7%	75,7%	65,6%
1429	20	60	45	75	52,2%	50,2%	66,5%	77,0%	67,1%
1430	20	60	45	80	52,6%	49,9%	67,3%	78,2%	68,6%
1431	20	60	50	20	41,3%	48,0%	54,7%	59,6%	47,9%
1432	20	60	50	25	41,7%	47,7%	55,5%	61,2%	49,6%
1433	20	60	50	30	42,1%	47,4%	56,4%	62,8%	51,4%
1434	20	60	50	35	42,6%	47,0%	57,2%	64,4%	53,1%
1435	20	60	50	40	43,0%	46,7%	58,0%	66,0%	54,8%
1436	20	60	50	45	43,4%	46,4%	58,8%	67,5%	56,5%
1437	20	60	50	50	43,9%	46,1%	59,7%	69,0%	58,1%
1438	20	60	50	55	44,3%	45,7%	60,5%	70,5%	59,8%
1439	20	60	50	60	44,7%	45,4%	61,3%	71,9%	61,4%
1440	20	60	50	65	45,2%	45,1%	62,1%	73,3%	63,0%
1441	20	60	50	70	45,6%	44,8%	62,9%	74,6%	64,6%
1442	20	60	50	75	46,0%	44,4%	63,7%	75,9%	66,2%
1443	20	60	50	80	46,5%	44,1%	64,5%	77,1%	67,7%
1444	20	60	55	20	35,4%	42,3%	51,6%	58,1%	46,9%
1445	20	60	55	25	35,8%	42,0%	52,4%	59,8%	48,6%
1446	20	60	55	30	36,3%	41,7%	53,3%	61,4%	50,3%
1447	20	60	55	35	36,7%	41,4%	54,1%	63,0%	52,0%
1448	20	60	55	40	37,1%	41,0%	55,0%	64,6%	53,7%
1449	20	60	55	45	37,5%	40,7%	55,8%	66,2%	55,4%
1450	20	60	55	50	37,9%	40,4%	56,6%	67,7%	57,1%
1451	20	60	55	55	38,3%	40,1%	57,5%	69,2%	58,8%

1452	20	60	55	60	38,7%	39,8%	58,3%	70,6%	60,4%
1453	20	60	55	65	39,1%	39,5%	59,1%	72,1%	62,1%
1454	20	60	55	70	39,6%	39,2%	59,9%	73,4%	63,7%
1455	20	60	55	75	40,0%	38,8%	60,8%	74,7%	65,2%
1456	20	60	55	80	40,4%	38,5%	61,6%	76,0%	66,8%
1457	20	60	60	20	30,0%	36,8%	48,5%	56,6%	45,9%
1458	20	60	60	25	30,4%	36,5%	49,3%	58,3%	47,6%
1459	20	60	60	30	30,8%	36,2%	50,2%	59,9%	49,3%
1460	20	60	60	35	31,1%	35,9%	51,0%	61,6%	51,0%
1461	20	60	60	40	31,5%	35,6%	51,9%	63,2%	52,7%
1462	20	60	60	45	31,9%	35,3%	52,7%	64,8%	54,4%
1463	20	60	60	50	32,3%	35,0%	53,6%	66,4%	56,1%
1464	20	60	60	55	32,7%	34,7%	54,4%	67,9%	57,8%
1465	20	60	60	60	33,1%	34,4%	55,3%	69,4%	59,5%
1466	20	60	60	65	33,4%	34,1%	56,1%	70,8%	61,1%
1467	20	60	60	70	33,8%	33,8%	56,9%	72,2%	62,7%
1468	20	60	60	75	34,2%	33,5%	57,8%	73,6%	64,3%
1469	20	60	60	80	34,6%	33,2%	58,6%	74,9%	65,9%
1470	20	60	65	20	25,1%	31,6%	45,4%	55,1%	44,9%
1471	20	60	65	25	25,4%	31,3%	46,2%	56,8%	46,6%
1472	20	60	65	30	25,8%	31,0%	47,1%	58,5%	48,3%
1473	20	60	65	35	26,1%	30,8%	47,9%	60,1%	50,0%
1474	20	60	65	40	26,4%	30,5%	48,8%	61,8%	51,7%
1475	20	60	65	45	26,8%	30,2%	49,6%	63,4%	53,4%
1476	20	60	65	50	27,1%	29,9%	50,5%	65,0%	55,1%
1477	20	60	65	55	27,5%	29,7%	51,3%	66,5%	56,8%
1478	20	60	65	60	27,8%	29,4%	52,2%	68,1%	58,5%
1479	20	60	65	65	28,2%	29,1%	53,0%	69,5%	60,1%
1480	20	60	65	70	28,6%	28,8%	53,9%	71,0%	61,7%
1481	20	60	65	75	28,9%	28,6%	54,7%	72,4%	63,3%
1482	20	60	65	80	29,3%	28,3%	55,5%	73,7%	64,9%
1483	20	60	70	20	20,7%	26,8%	42,3%	53,6%	43,8%
1484	20	60	70	25	21,0%	26,6%	43,2%	55,3%	45,5%

1485	20	60	70	30	21,3%	26,3%	44,0%	57,0%	47,2%
1486	20	60	70	35	21,6%	26,1%	44,8%	58,7%	48,9%
1487	20	60	70	40	21,9%	25,8%	45,7%	60,3%	50,7%
1488	20	60	70	45	22,2%	25,6%	46,5%	62,0%	52,4%
1489	20	60	70	50	22,5%	25,3%	47,4%	63,6%	54,1%
1490	20	60	70	55	22,8%	25,1%	48,2%	65,2%	55,8%
1491	20	60	70	60	23,2%	24,8%	49,1%	66,7%	57,5%
1492	20	60	70	65	23,5%	24,6%	49,9%	68,2%	59,1%
1493	20	60	70	70	23,8%	24,3%	50,8%	69,7%	60,8%
1494	20	60	70	75	24,1%	24,1%	51,6%	71,1%	62,4%
1495	20	60	70	80	24,4%	23,9%	52,5%	72,5%	64,0%
1496	20	60	75	20	17,0%	22,6%	39,3%	52,1%	42,8%
1497	20	60	75	25	17,2%	22,3%	40,1%	53,8%	44,5%
1498	20	60	75	30	17,5%	22,1%	41,0%	55,5%	46,2%
1499	20	60	75	35	17,7%	21,9%	41,8%	57,2%	47,9%
1500	20	60	75	40	18,0%	21,7%	42,6%	58,9%	49,6%
1501	20	60	75	45	18,3%	21,4%	43,5%	60,5%	51,3%
1502	20	60	75	50	18,5%	21,2%	44,3%	62,2%	53,0%
1503	20	60	75	55	18,8%	21,0%	45,1%	63,8%	54,8%
1504	20	60	75	60	19,1%	20,8%	46,0%	65,3%	56,4%
1505	20	60	75	65	19,3%	20,6%	46,8%	66,9%	58,1%
1506	20	60	75	70	19,6%	20,4%	47,7%	68,4%	59,8%
1507	20	60	75	75	19,9%	20,1%	48,5%	69,9%	61,4%
1508	20	60	75	80	20,2%	19,9%	49,4%	71,3%	63,0%
1509	20	60	80	20	13,8%	18,8%	36,4%	50,5%	41,8%
1510	20	60	80	25	14,0%	18,6%	37,2%	52,3%	43,5%
1511	20	60	80	30	14,2%	18,4%	38,0%	54,0%	45,2%
1512	20	60	80	35	14,4%	18,2%	38,8%	55,7%	46,9%
1513	20	60	80	40	14,6%	18,0%	39,6%	57,4%	48,6%
1514	20	60	80	45	14,9%	17,8%	40,4%	59,1%	50,3%
1515	20	60	80	50	15,1%	17,6%	41,2%	60,7%	52,0%
1516	20	60	80	55	15,3%	17,4%	42,1%	62,3%	53,7%
1517	20	60	80	60	15,5%	17,2%	42,9%	64,0%	55,4%

1518	20	60	80	65	15,8%	17,0%	43,7%	65,5%	57,1%
1519	20	60	80	70	16,0%	16,9%	44,6%	67,1%	58,8%
1520	20	60	80	75	16,2%	16,7%	45,4%	68,6%	60,4%
1521	20	60	80	80	16,5%	16,5%	46,3%	70,0%	62,1%
1522	20	65	20	20	77,2%	80,0%	71,1%	66,4%	51,7%
1523	20	65	20	25	77,5%	79,8%	71,8%	67,9%	53,4%
1524	20	65	20	30	77,8%	79,6%	72,5%	69,4%	55,1%
1525	20	65	20	35	78,1%	79,3%	73,2%	70,8%	56,8%
1526	20	65	20	40	78,4%	79,1%	73,8%	72,2%	58,5%
1527	20	65	20	45	78,7%	78,9%	74,5%	73,6%	60,2%
1528	20	65	20	50	79,0%	78,7%	75,1%	74,9%	61,8%
1529	20	65	20	55	79,3%	78,5%	75,8%	76,2%	63,4%
1530	20	65	20	60	79,5%	78,3%	76,4%	77,4%	65,0%
1531	20	65	20	65	79,8%	78,0%	77,0%	78,6%	66,5%
1532	20	65	20	70	80,1%	77,8%	77,6%	79,7%	68,0%
1533	20	65	20	75	80,4%	77,6%	78,2%	80,8%	69,5%
1534	20	65	20	80	80,7%	77,4%	78,7%	81,9%	70,9%
1535	20	65	25	20	72,5%	76,0%	68,5%	65,0%	50,7%
1536	20	65	25	25	72,9%	75,8%	69,2%	66,5%	52,4%
1537	20	65	25	30	73,2%	75,6%	70,0%	68,0%	54,1%
1538	20	65	25	35	73,6%	75,3%	70,7%	69,5%	55,8%
1539	20	65	25	40	73,9%	75,1%	71,4%	71,0%	57,5%
1540	20	65	25	45	74,2%	74,8%	72,1%	72,4%	59,2%
1541	20	65	25	50	74,6%	74,6%	72,7%	73,7%	60,8%
1542	20	65	25	55	74,9%	74,3%	73,4%	75,0%	62,4%
1543	20	65	25	60	75,2%	74,1%	74,1%	76,3%	64,0%
1544	20	65	25	65	75,6%	73,8%	74,7%	77,5%	65,6%
1545	20	65	25	70	75,9%	73,6%	75,3%	78,7%	67,1%
1546	20	65	25	75	76,2%	73,3%	76,0%	79,8%	68,6%
1547	20	65	25	80	76,5%	73,1%	76,6%	80,9%	70,1%
1548	20	65	30	20	67,3%	71,6%	65,8%	63,6%	49,7%
1549	20	65	30	25	67,7%	71,3%	66,5%	65,2%	51,4%
1550	20	65	30	30	68,1%	71,0%	67,3%	66,7%	53,1%

1551	20	65	30	35	68,5%	70,8%	68,0%	68,2%	54,8%
1552	20	65	30	40	68,9%	70,5%	68,8%	69,7%	56,5%
1553	20	65	30	45	69,2%	70,2%	69,5%	71,1%	58,2%
1554	20	65	30	50	69,6%	70,0%	70,2%	72,5%	59,8%
1555	20	65	30	55	70,0%	69,7%	70,9%	73,9%	61,5%
1556	20	65	30	60	70,4%	69,4%	71,6%	75,2%	63,1%
1557	20	65	30	65	70,7%	69,1%	72,3%	76,5%	64,6%
1558	20	65	30	70	71,1%	68,8%	73,0%	77,7%	66,2%
1559	20	65	30	75	71,5%	68,6%	73,6%	78,8%	67,7%
1560	20	65	30	80	71,8%	68,3%	74,3%	80,0%	69,2%
1561	20	65	35	20	61,7%	66,7%	62,9%	62,2%	48,6%
1562	20	65	35	25	62,1%	66,4%	63,7%	63,8%	50,4%
1563	20	65	35	30	62,5%	66,1%	64,5%	65,3%	52,1%
1564	20	65	35	35	62,9%	65,8%	65,3%	66,9%	53,8%
1565	20	65	35	40	63,3%	65,5%	66,0%	68,4%	55,5%
1566	20	65	35	45	63,8%	65,2%	66,8%	69,9%	57,2%
1567	20	65	35	50	64,2%	64,9%	67,5%	71,3%	58,8%
1568	20	65	35	55	64,6%	64,6%	68,3%	72,7%	60,5%
1569	20	65	35	60	65,0%	64,3%	69,0%	74,0%	62,1%
1570	20	65	35	65	65,4%	64,0%	69,7%	75,3%	63,7%
1571	20	65	35	70	65,8%	63,7%	70,4%	76,6%	65,3%
1572	20	65	35	75	66,2%	63,4%	71,1%	77,8%	66,8%
1573	20	65	35	80	66,6%	63,1%	71,8%	79,0%	68,3%
1574	20	65	40	20	55,7%	61,3%	60,0%	60,7%	47,6%
1575	20	65	40	25	56,2%	61,0%	60,8%	62,3%	49,3%
1576	20	65	40	30	56,6%	60,7%	61,6%	63,9%	51,0%
1577	20	65	40	35	57,0%	60,4%	62,4%	65,5%	52,7%
1578	20	65	40	40	57,4%	60,1%	63,2%	67,1%	54,4%
1579	20	65	40	45	57,9%	59,8%	64,0%	68,6%	56,1%
1580	20	65	40	50	58,3%	59,5%	64,8%	70,0%	57,8%
1581	20	65	40	55	58,7%	59,1%	65,5%	71,5%	59,5%
1582	20	65	40	60	59,2%	58,8%	66,3%	72,8%	61,1%
1583	20	65	40	65	59,6%	58,5%	67,0%	74,2%	62,7%

1584	20	65	40	70	60,0%	58,2%	67,8%	75,5%	64,3%
1585	20	65	40	75	60,4%	57,9%	68,5%	76,7%	65,9%
1586	20	65	40	80	60,9%	57,6%	69,3%	78,0%	67,4%
1587	20	65	45	20	49,6%	55,7%	57,0%	59,2%	46,6%
1588	20	65	45	25	50,0%	55,4%	57,8%	60,9%	48,3%
1589	20	65	45	30	50,5%	55,1%	58,6%	62,5%	50,0%
1590	20	65	45	35	50,9%	54,8%	59,4%	64,1%	51,7%
1591	20	65	45	40	51,3%	54,4%	60,3%	65,7%	53,4%
1592	20	65	45	45	51,8%	54,1%	61,1%	67,2%	55,1%
1593	20	65	45	50	52,2%	53,8%	61,9%	68,7%	56,8%
1594	20	65	45	55	52,7%	53,5%	62,7%	70,2%	58,5%
1595	20	65	45	60	53,1%	53,1%	63,5%	71,6%	60,1%
1596	20	65	45	65	53,5%	52,8%	64,2%	73,0%	61,8%
1597	20	65	45	70	54,0%	52,5%	65,0%	74,3%	63,4%
1598	20	65	45	75	54,4%	52,2%	65,8%	75,6%	64,9%
1599	20	65	45	80	54,8%	51,8%	66,5%	76,9%	66,5%
1600	20	65	50	20	43,4%	50,0%	53,9%	57,8%	45,6%
1601	20	65	50	25	43,9%	49,7%	54,7%	59,4%	47,3%
1602	20	65	50	30	44,3%	49,3%	55,6%	61,1%	49,0%
1603	20	65	50	35	44,7%	49,0%	56,4%	62,7%	50,7%
1604	20	65	50	40	45,2%	48,7%	57,2%	64,3%	52,4%
1605	20	65	50	45	45,6%	48,4%	58,1%	65,9%	54,1%
1606	20	65	50	50	46,0%	48,0%	58,9%	67,4%	55,8%
1607	20	65	50	55	46,5%	47,7%	59,7%	68,9%	57,5%
1608	20	65	50	60	46,9%	47,4%	60,5%	70,4%	59,1%
1609	20	65	50	65	47,4%	47,1%	61,3%	71,8%	60,8%
1610	20	65	50	70	47,8%	46,7%	62,1%	73,2%	62,4%
1611	20	65	50	75	48,2%	46,4%	62,9%	74,5%	64,0%
1612	20	65	50	80	48,7%	46,1%	63,7%	75,8%	65,6%
1613	20	65	55	20	37,5%	44,2%	50,8%	56,3%	44,5%
1614	20	65	55	25	37,9%	43,9%	51,6%	58,0%	46,2%
1615	20	65	55	30	38,3%	43,6%	52,5%	59,6%	47,9%
1616	20	65	55	35	38,7%	43,3%	53,3%	61,3%	49,7%

1617	20	65	55	40	39,2%	43,0%	54,2%	62,9%	51,4%
1618	20	65	55	45	39,6%	42,6%	55,0%	64,5%	53,1%
1619	20	65	55	50	40,0%	42,3%	55,9%	66,1%	54,8%
1620	20	65	55	55	40,4%	42,0%	56,7%	67,6%	56,5%
1621	20	65	55	60	40,9%	41,7%	57,5%	69,1%	58,1%
1622	20	65	55	65	41,3%	41,4%	58,4%	70,5%	59,8%
1623	20	65	55	70	41,7%	41,0%	59,2%	71,9%	61,4%
1624	20	65	55	75	42,1%	40,7%	60,0%	73,3%	63,0%
1625	20	65	55	80	42,6%	40,4%	60,8%	74,6%	64,6%
1626	20	65	60	20	31,9%	38,6%	47,7%	54,8%	43,5%
1627	20	65	60	25	32,3%	38,3%	48,5%	56,5%	45,2%
1628	20	65	60	30	32,7%	38,0%	49,4%	58,2%	46,9%
1629	20	65	60	35	33,1%	37,7%	50,2%	59,8%	48,6%
1630	20	65	60	40	33,5%	37,4%	51,1%	61,5%	50,3%
1631	20	65	60	45	33,9%	37,1%	51,9%	63,1%	52,0%
1632	20	65	60	50	34,2%	36,8%	52,8%	64,7%	53,8%
1633	20	65	60	55	34,6%	36,5%	53,6%	66,2%	55,5%
1634	20	65	60	60	35,0%	36,2%	54,5%	67,8%	57,1%
1635	20	65	60	65	35,5%	35,9%	55,3%	69,3%	58,8%
1636	20	65	60	70	35,9%	35,6%	56,2%	70,7%	60,5%
1637	20	65	60	75	36,3%	35,3%	57,0%	72,1%	62,1%
1638	20	65	60	80	36,7%	35,0%	57,8%	73,5%	63,7%
1639	20	65	65	20	26,8%	33,3%	44,6%	53,3%	42,5%
1640	20	65	65	25	27,1%	33,0%	45,4%	55,0%	44,2%
1641	20	65	65	30	27,5%	32,8%	46,3%	56,7%	45,9%
1642	20	65	65	35	27,8%	32,5%	47,1%	58,4%	47,6%
1643	20	65	65	40	28,2%	32,2%	48,0%	60,0%	49,3%
1644	20	65	65	45	28,6%	31,9%	48,8%	61,7%	51,0%
1645	20	65	65	50	28,9%	31,6%	49,7%	63,3%	52,7%
1646	20	65	65	55	29,3%	31,3%	50,5%	64,9%	54,4%
1647	20	65	65	60	29,7%	31,1%	51,4%	66,4%	56,1%
1648	20	65	65	65	30,0%	30,8%	52,2%	67,9%	57,8%
1649	20	65	65	70	30,4%	30,5%	53,1%	69,4%	59,5%

1650	20	65	65	75	30,8%	30,2%	53,9%	70,9%	61,1%
1651	20	65	65	80	31,1%	29,9%	54,8%	72,3%	62,7%
1652	20	65	70	20	22,2%	28,4%	41,6%	51,7%	41,5%
1653	20	65	70	25	22,5%	28,1%	42,4%	53,5%	43,2%
1654	20	65	70	30	22,9%	27,9%	43,2%	55,2%	44,9%
1655	20	65	70	35	23,2%	27,6%	44,1%	56,9%	46,6%
1656	20	65	70	40	23,5%	27,4%	44,9%	58,5%	48,3%
1657	20	65	70	45	23,8%	27,1%	45,7%	60,2%	50,0%
1658	20	65	70	50	24,1%	26,8%	46,6%	61,8%	51,7%
1659	20	65	70	55	24,4%	26,6%	47,4%	63,5%	53,4%
1660	20	65	70	60	24,8%	26,3%	48,3%	65,0%	55,1%
1661	20	65	70	65	25,1%	26,1%	49,1%	66,6%	56,8%
1662	20	65	70	70	25,4%	25,8%	50,0%	68,1%	58,5%
1663	20	65	70	75	25,8%	25,6%	50,8%	69,6%	60,1%
1664	20	65	70	80	26,1%	25,3%	51,7%	71,0%	61,7%
1665	20	65	75	20	18,3%	24,0%	38,6%	50,2%	40,5%
1666	20	65	75	25	18,5%	23,7%	39,4%	51,9%	42,2%
1667	20	65	75	30	18,8%	23,5%	40,2%	53,7%	43,8%
1668	20	65	75	35	19,1%	23,3%	41,0%	55,4%	45,5%
1669	20	65	75	40	19,3%	23,0%	41,8%	57,1%	47,2%
1670	20	65	75	45	19,6%	22,8%	42,7%	58,7%	49,0%
1671	20	65	75	50	19,9%	22,6%	43,5%	60,4%	50,7%
1672	20	65	75	55	20,2%	22,3%	44,3%	62,0%	52,4%
1673	20	65	75	60	20,5%	22,1%	45,2%	63,6%	54,1%
1674	20	65	75	65	20,8%	21,9%	46,0%	65,2%	55,8%
1675	20	65	75	70	21,0%	21,7%	46,9%	66,8%	57,5%
1676	20	65	75	75	21,3%	21,4%	47,7%	68,3%	59,1%
1677	20	65	75	80	21,6%	21,2%	48,6%	69,8%	60,8%
1678	20	65	80	20	14,9%	20,0%	35,7%	48,7%	39,5%
1679	20	65	80	25	15,1%	19,8%	36,5%	50,4%	41,2%
1680	20	65	80	30	15,3%	19,6%	37,3%	52,1%	42,8%
1681	20	65	80	35	15,5%	19,4%	38,1%	53,9%	44,5%
1682	20	65	80	40	15,8%	19,2%	38,9%	55,6%	46,2%

1683	20	65	80	45	16,0%	19,0%	39,7%	57,3%	47,9%
1684	20	65	80	50	16,2%	18,8%	40,5%	58,9%	49,6%
1685	20	65	80	55	16,5%	18,6%	41,3%	60,6%	51,3%
1686	20	65	80	60	16,7%	18,4%	42,1%	62,2%	53,1%
1687	20	65	80	65	17,0%	18,2%	43,0%	63,8%	54,8%
1688	20	65	80	70	17,2%	18,0%	43,8%	65,4%	56,4%
1689	20	65	80	75	17,5%	17,8%	44,6%	67,0%	58,1%
1690	20	65	80	80	17,7%	17,6%	45,5%	68,5%	59,8%
1691	20	70	20	20	78,7%	81,2%	70,5%	64,7%	49,3%
1692	20	70	20	25	79,0%	81,0%	71,2%	66,2%	51,1%
1693	20	70	20	30	79,3%	80,8%	71,9%	67,8%	52,8%
1694	20	70	20	35	79,6%	80,6%	72,5%	69,2%	54,5%
1695	20	70	20	40	79,8%	80,4%	73,2%	70,7%	56,2%
1696	20	70	20	45	80,1%	80,2%	73,9%	72,1%	57,8%
1697	20	70	20	50	80,4%	80,0%	74,5%	73,5%	59,5%
1698	20	70	20	55	80,7%	79,8%	75,2%	74,8%	61,1%
1699	20	70	20	60	81,0%	79,6%	75,8%	76,1%	62,8%
1700	20	70	20	65	81,2%	79,4%	76,4%	77,3%	64,3%
1701	20	70	20	70	81,5%	79,1%	77,0%	78,5%	65,9%
1702	20	70	20	75	81,8%	78,9%	77,6%	79,6%	67,4%
1703	20	70	20	80	82,0%	78,7%	78,2%	80,7%	68,9%
1704	20	70	25	20	74,3%	77,4%	67,8%	63,3%	48,3%
1705	20	70	25	25	74,6%	77,2%	68,6%	64,9%	50,0%
1706	20	70	25	30	74,9%	77,0%	69,3%	66,4%	51,7%
1707	20	70	25	35	75,2%	76,7%	70,0%	67,9%	53,4%
1708	20	70	25	40	75,6%	76,5%	70,7%	69,4%	55,1%
1709	20	70	25	45	75,9%	76,3%	71,4%	70,9%	56,8%
1710	20	70	25	50	76,2%	76,0%	72,1%	72,3%	58,5%
1711	20	70	25	55	76,5%	75,8%	72,8%	73,6%	60,2%
1712	20	70	25	60	76,9%	75,6%	73,4%	74,9%	61,8%
1713	20	70	25	65	77,2%	75,3%	74,1%	76,2%	63,4%
1714	20	70	25	70	77,5%	75,1%	74,7%	77,4%	65,0%
1715	20	70	25	75	77,8%	74,8%	75,4%	78,6%	66,5%

1716	20	70	25	80	78,1%	74,6%	76,0%	79,8%	68,0%
1717	20	70	30	20	69,3%	73,1%	65,1%	61,8%	47,3%
1718	20	70	30	25	69,6%	72,9%	65,8%	63,5%	49,0%
1719	20	70	30	30	70,0%	72,6%	66,6%	65,0%	50,7%
1720	20	70	30	35	70,4%	72,4%	67,3%	66,6%	52,4%
1721	20	70	30	40	70,7%	72,1%	68,1%	68,1%	54,1%
1722	20	70	30	45	71,1%	71,8%	68,8%	69,6%	55,8%
1723	20	70	30	50	71,5%	71,6%	69,5%	71,0%	57,5%
1724	20	70	30	55	71,8%	71,3%	70,2%	72,4%	59,2%
1725	20	70	30	60	72,2%	71,0%	71,0%	73,8%	60,8%
1726	20	70	30	65	72,5%	70,8%	71,6%	75,1%	62,4%
1727	20	70	30	70	72,9%	70,5%	72,3%	76,4%	64,0%
1728	20	70	30	75	73,2%	70,2%	73,0%	77,6%	65,6%
1729	20	70	30	80	73,6%	70,0%	73,7%	78,8%	67,1%
1730	20	70	35	20	63,8%	68,4%	62,2%	60,4%	46,3%
1731	20	70	35	25	64,2%	68,1%	63,0%	62,0%	48,0%
1732	20	70	35	30	64,6%	67,8%	63,8%	63,6%	49,7%
1733	20	70	35	35	65,0%	67,5%	64,5%	65,2%	51,4%
1734	20	70	35	40	65,4%	67,2%	65,3%	66,8%	53,1%
1735	20	70	35	45	65,8%	66,9%	66,1%	68,3%	54,8%
1736	20	70	35	50	66,2%	66,7%	66,8%	69,8%	56,5%
1737	20	70	35	55	66,6%	66,4%	67,6%	71,2%	58,2%
1738	20	70	35	60	67,0%	66,1%	68,3%	72,6%	59,8%
1739	20	70	35	65	67,3%	65,8%	69,1%	73,9%	61,5%
1740	20	70	35	70	67,7%	65,5%	69,8%	75,2%	63,1%
1741	20	70	35	75	68,1%	65,2%	70,5%	76,5%	64,6%
1742	20	70	35	80	68,5%	64,9%	71,2%	77,7%	66,2%
1743	20	70	40	20	57,9%	63,2%	59,2%	58,9%	45,2%
1744	20	70	40	25	58,3%	62,9%	60,0%	60,6%	46,9%
1745	20	70	40	30	58,7%	62,6%	60,8%	62,2%	48,6%
1746	20	70	40	35	59,2%	62,3%	61,6%	63,8%	50,4%
1747	20	70	40	40	59,6%	62,0%	62,4%	65,4%	52,1%
1748	20	70	40	45	60,0%	61,7%	63,2%	66,9%	53,8%

1749	20	70	40	50	60,4%	61,3%	64,0%	68,5%	55,5%
1750	20	70	40	55	60,9%	61,0%	64,8%	69,9%	57,2%
1751	20	70	40	60	61,3%	60,7%	65,6%	71,4%	58,8%
1752	20	70	40	65	61,7%	60,4%	66,3%	72,7%	60,5%
1753	20	70	40	70	62,1%	60,1%	67,1%	74,1%	62,1%
1754	20	70	40	75	62,5%	59,8%	67,8%	75,4%	63,7%
1755	20	70	40	80	62,9%	59,5%	68,6%	76,7%	65,3%
1756	20	70	45	20	51,8%	57,7%	56,2%	57,4%	44,2%
1757	20	70	45	25	52,2%	57,4%	57,0%	59,1%	45,9%
1758	20	70	45	30	52,7%	57,0%	57,8%	60,8%	47,6%
1759	20	70	45	35	53,1%	56,7%	58,7%	62,4%	49,3%
1760	20	70	45	40	53,5%	56,4%	59,5%	64,0%	51,0%
1761	20	70	45	45	54,0%	56,1%	60,3%	65,6%	52,8%
1762	20	70	45	50	54,4%	55,7%	61,1%	67,1%	54,5%
1763	20	70	45	55	54,9%	55,4%	61,9%	68,6%	56,1%
1764	20	70	45	60	55,3%	55,1%	62,7%	70,1%	57,8%
1765	20	70	45	65	55,7%	54,8%	63,5%	71,5%	59,5%
1766	20	70	45	70	56,2%	54,5%	64,3%	72,9%	61,1%
1767	20	70	45	75	56,6%	54,1%	65,1%	74,2%	62,7%
1768	20	70	45	80	57,0%	53,8%	65,8%	75,5%	64,3%
1769	20	70	50	20	45,6%	52,0%	53,1%	55,9%	43,2%
1770	20	70	50	25	46,1%	51,6%	53,9%	57,6%	44,9%
1771	20	70	50	30	46,5%	51,3%	54,8%	59,3%	46,6%
1772	20	70	50	35	46,9%	51,0%	55,6%	61,0%	48,3%
1773	20	70	50	40	47,4%	50,7%	56,5%	62,6%	50,0%
1774	20	70	50	45	47,8%	50,3%	57,3%	64,2%	51,7%
1775	20	70	50	50	48,3%	50,0%	58,1%	65,8%	53,4%
1776	20	70	50	55	48,7%	49,7%	59,0%	67,3%	55,1%
1777	20	70	50	60	49,1%	49,3%	59,8%	68,8%	56,8%
1778	20	70	50	65	49,6%	49,0%	60,6%	70,3%	58,5%
1779	20	70	50	70	50,0%	48,7%	61,4%	71,7%	60,1%
1780	20	70	50	75	50,5%	48,4%	62,2%	73,1%	61,8%
1781	20	70	50	80	50,9%	48,0%	63,0%	74,4%	63,4%

1782	20	70	55	20	39,6%	46,2%	50,0%	54,4%	42,2%
1783	20	70	55	25	40,0%	45,9%	50,9%	56,1%	43,9%
1784	20	70	55	30	40,4%	45,5%	51,7%	57,8%	45,6%
1785	20	70	55	35	40,9%	45,2%	52,5%	59,5%	47,3%
1786	20	70	55	40	41,3%	44,9%	53,4%	61,2%	49,0%
1787	20	70	55	45	41,7%	44,6%	54,2%	62,8%	50,7%
1788	20	70	55	50	42,2%	44,3%	55,1%	64,4%	52,4%
1789	20	70	55	55	42,6%	43,9%	55,9%	65,9%	54,1%
1790	20	70	55	60	43,0%	43,6%	56,8%	67,5%	55,8%
1791	20	70	55	65	43,4%	43,3%	57,6%	69,0%	57,5%
1792	20	70	55	70	43,9%	43,0%	58,4%	70,4%	59,1%
1793	20	70	55	75	44,3%	42,6%	59,2%	71,8%	60,8%
1794	20	70	55	80	44,7%	42,3%	60,1%	73,2%	62,4%
1795	20	70	60	20	33,9%	40,5%	46,9%	52,9%	41,2%
1796	20	70	60	25	34,3%	40,2%	47,7%	54,6%	42,9%
1797	20	70	60	30	34,7%	39,9%	48,6%	56,3%	44,5%
1798	20	70	60	35	35,1%	39,6%	49,4%	58,0%	46,2%
1799	20	70	60	40	35,5%	39,3%	50,3%	59,7%	47,9%
1800	20	70	60	45	35,9%	39,0%	51,1%	61,3%	49,7%
1801	20	70	60	50	36,3%	38,7%	52,0%	63,0%	51,4%
1802	20	70	60	55	36,7%	38,3%	52,8%	64,6%	53,1%
1803	20	70	60	60	37,1%	38,0%	53,7%	66,1%	54,8%
1804	20	70	60	65	37,5%	37,7%	54,5%	67,7%	56,5%
1805	20	70	60	70	37,9%	37,4%	55,4%	69,1%	58,1%
1806	20	70	60	75	38,3%	37,1%	56,2%	70,6%	59,8%
1807	20	70	60	80	38,8%	36,8%	57,0%	72,0%	61,4%
1808	20	70	65	20	28,6%	35,1%	43,8%	51,4%	40,2%
1809	20	70	65	25	28,9%	34,8%	44,7%	53,1%	41,9%
1810	20	70	65	30	29,3%	34,5%	45,5%	54,8%	43,5%
1811	20	70	65	35	29,7%	34,2%	46,3%	56,5%	45,2%
1812	20	70	65	40	30,0%	33,9%	47,2%	58,2%	46,9%
1813	20	70	65	45	30,4%	33,6%	48,0%	59,9%	48,6%
1814	20	70	65	50	30,8%	33,3%	48,9%	61,5%	50,3%

1815	20	70	65	55	31,2%	33,1%	49,7%	63,2%	52,1%
1816	20	70	65	60	31,5%	32,8%	50,6%	64,7%	53,8%
1817	20	70	65	65	31,9%	32,5%	51,4%	66,3%	55,5%
1818	20	70	65	70	32,3%	32,2%	52,3%	67,8%	57,1%
1819	20	70	65	75	32,7%	31,9%	53,1%	69,3%	58,8%
1820	20	70	65	80	33,1%	31,6%	54,0%	70,8%	60,5%
1821	20	70	70	20	23,8%	30,0%	40,8%	49,9%	39,2%
1822	20	70	70	25	24,1%	29,8%	41,6%	51,6%	40,9%
1823	20	70	70	30	24,5%	29,5%	42,4%	53,3%	42,5%
1824	20	70	70	35	24,8%	29,2%	43,3%	55,0%	44,2%
1825	20	70	70	40	25,1%	29,0%	44,1%	56,7%	45,9%
1826	20	70	70	45	25,4%	28,7%	44,9%	58,4%	47,6%
1827	20	70	70	50	25,8%	28,4%	45,8%	60,1%	49,3%
1828	20	70	70	55	26,1%	28,2%	46,6%	61,7%	51,0%
1829	20	70	70	60	26,5%	27,9%	47,5%	63,3%	52,7%
1830	20	70	70	65	26,8%	27,6%	48,3%	64,9%	54,4%
1831	20	70	70	70	27,2%	27,4%	49,2%	66,5%	56,1%
1832	20	70	70	75	27,5%	27,1%	50,0%	68,0%	57,8%
1833	20	70	70	80	27,9%	26,8%	50,9%	69,5%	59,5%
1834	20	70	75	20	19,6%	25,4%	37,8%	48,4%	38,2%
1835	20	70	75	25	19,9%	25,2%	38,6%	50,1%	39,9%
1836	20	70	75	30	20,2%	24,9%	39,4%	51,8%	41,5%
1837	20	70	75	35	20,5%	24,7%	40,3%	53,5%	43,2%
1838	20	70	75	40	20,8%	24,4%	41,1%	55,2%	44,9%
1839	20	70	75	45	21,1%	24,2%	41,9%	56,9%	46,6%
1840	20	70	75	50	21,3%	24,0%	42,7%	58,6%	48,3%
1841	20	70	75	55	21,6%	23,7%	43,6%	60,3%	50,0%
1842	20	70	75	60	21,9%	23,5%	44,4%	61,9%	51,7%
1843	20	70	75	65	22,2%	23,3%	45,2%	63,5%	53,4%
1844	20	70	75	70	22,6%	23,0%	46,1%	65,1%	55,1%
1845	20	70	75	75	22,9%	22,8%	46,9%	66,7%	56,8%
1846	20	70	75	80	23,2%	22,6%	47,8%	68,2%	58,5%
1847	20	70	80	20	16,0%	21,3%	35,0%	46,8%	37,3%

1848	20	70	80	25	16,3%	21,1%	35,7%	48,6%	38,9%
1849	20	70	80	30	16,5%	20,9%	36,5%	50,3%	40,5%
1850	20	70	80	35	16,7%	20,6%	37,3%	52,0%	42,2%
1851	20	70	80	40	17,0%	20,4%	38,1%	53,7%	43,9%
1852	20	70	80	45	17,2%	20,2%	38,9%	55,4%	45,5%
1853	20	70	80	50	17,5%	20,0%	39,7%	57,1%	47,3%
1854	20	70	80	55	17,7%	19,8%	40,5%	58,8%	49,0%
1855	20	70	80	60	18,0%	19,6%	41,4%	60,5%	50,7%
1856	20	70	80	65	18,3%	19,4%	42,2%	62,1%	52,4%
1857	20	70	80	70	18,5%	19,2%	43,0%	63,7%	54,1%
1858	20	70	80	75	18,8%	19,0%	43,8%	65,3%	55,8%
1859	20	70	80	80	19,1%	18,8%	44,7%	66,8%	57,5%
1860	20	75	20	20	80,1%	82,4%	69,8%	63,0%	47,0%
1861	20	75	20	25	80,4%	82,2%	70,5%	64,6%	48,7%
1862	20	75	20	30	80,7%	82,0%	71,2%	66,1%	50,4%
1863	20	75	20	35	81,0%	81,8%	71,9%	67,6%	52,1%
1864	20	75	20	40	81,2%	81,6%	72,6%	69,1%	53,8%
1865	20	75	20	45	81,5%	81,4%	73,3%	70,6%	55,5%
1866	20	75	20	50	81,8%	81,2%	73,9%	72,0%	57,2%
1867	20	75	20	55	82,0%	81,0%	74,6%	73,4%	58,9%
1868	20	75	20	60	82,3%	80,8%	75,2%	74,7%	60,5%
1869	20	75	20	65	82,5%	80,6%	75,8%	76,0%	62,1%
1870	20	75	20	70	82,8%	80,4%	76,5%	77,2%	63,7%
1871	20	75	20	75	83,0%	80,2%	77,1%	78,4%	65,3%
1872	20	75	20	80	83,3%	80,0%	77,7%	79,6%	66,8%
1873	20	75	25	20	75,9%	78,8%	67,1%	61,5%	45,9%
1874	20	75	25	25	76,2%	78,6%	67,9%	63,2%	47,6%
1875	20	75	25	30	76,5%	78,3%	68,6%	64,7%	49,4%
1876	20	75	25	35	76,9%	78,1%	69,3%	66,3%	51,1%
1877	20	75	25	40	77,2%	77,9%	70,0%	67,8%	52,8%
1878	20	75	25	45	77,5%	77,7%	70,8%	69,3%	54,5%
1879	20	75	25	50	77,8%	77,4%	71,5%	70,8%	56,2%
1880	20	75	25	55	78,1%	77,2%	72,1%	72,2%	57,8%

1881	20	75	25	60	78,4%	77,0%	72,8%	73,5%	59,5%
1882	20	75	25	65	78,7%	76,7%	73,5%	74,8%	61,1%
1883	20	75	25	70	79,0%	76,5%	74,1%	76,1%	62,8%
1884	20	75	25	75	79,3%	76,3%	74,8%	77,4%	64,3%
1885	20	75	25	80	79,6%	76,0%	75,4%	78,5%	65,9%
1886	20	75	30	20	71,1%	74,7%	64,3%	60,1%	44,9%
1887	20	75	30	25	71,5%	74,4%	65,1%	61,7%	46,6%
1888	20	75	30	30	71,8%	74,2%	65,9%	63,3%	48,3%
1889	20	75	30	35	72,2%	73,9%	66,6%	64,9%	50,0%
1890	20	75	30	40	72,5%	73,7%	67,4%	66,5%	51,7%
1891	20	75	30	45	72,9%	73,4%	68,1%	68,0%	53,5%
1892	20	75	30	50	73,2%	73,2%	68,9%	69,5%	55,2%
1893	20	75	30	55	73,6%	72,9%	69,6%	70,9%	56,8%
1894	20	75	30	60	73,9%	72,6%	70,3%	72,3%	58,5%
1895	20	75	30	65	74,3%	72,4%	71,0%	73,7%	60,2%
1896	20	75	30	70	74,6%	72,1%	71,7%	75,0%	61,8%
1897	20	75	30	75	74,9%	71,9%	72,4%	76,3%	63,4%
1898	20	75	30	80	75,3%	71,6%	73,0%	77,5%	65,0%
1899	20	75	35	20	65,8%	70,1%	61,4%	58,6%	43,9%
1900	20	75	35	25	66,2%	69,8%	62,2%	60,3%	45,6%
1901	20	75	35	30	66,6%	69,5%	63,0%	61,9%	47,3%
1902	20	75	35	35	67,0%	69,2%	63,8%	63,5%	49,0%
1903	20	75	35	40	67,4%	68,9%	64,6%	65,1%	50,7%
1904	20	75	35	45	67,7%	68,7%	65,4%	66,7%	52,4%
1905	20	75	35	50	68,1%	68,4%	66,1%	68,2%	54,1%
1906	20	75	35	55	68,5%	68,1%	66,9%	69,7%	55,8%
1907	20	75	35	60	68,9%	67,8%	67,6%	71,1%	57,5%
1908	20	75	35	65	69,3%	67,5%	68,4%	72,5%	59,2%
1909	20	75	35	70	69,6%	67,2%	69,1%	73,8%	60,8%
1910	20	75	35	75	70,0%	67,0%	69,8%	75,1%	62,4%
1911	20	75	35	80	70,4%	66,7%	70,5%	76,4%	64,0%
1912	20	75	40	20	60,0%	65,0%	58,4%	57,1%	42,9%
1913	20	75	40	25	60,5%	64,7%	59,3%	58,8%	44,6%

1914	20	75	40	30	60,9%	64,4%	60,1%	60,5%	46,3%
1915	20	75	40	35	61,3%	64,1%	60,9%	62,1%	48,0%
1916	20	75	40	40	61,7%	63,8%	61,7%	63,7%	49,7%
1917	20	75	40	45	62,1%	63,5%	62,5%	65,3%	51,4%
1918	20	75	40	50	62,5%	63,2%	63,3%	66,8%	53,1%
1919	20	75	40	55	63,0%	62,9%	64,1%	68,3%	54,8%
1920	20	75	40	60	63,4%	62,6%	64,9%	69,8%	56,5%
1921	20	75	40	65	63,8%	62,3%	65,6%	71,3%	58,2%
1922	20	75	40	70	64,2%	62,0%	66,4%	72,6%	59,8%
1923	20	75	40	75	64,6%	61,7%	67,1%	74,0%	61,5%
1924	20	75	40	80	65,0%	61,4%	67,9%	75,3%	63,1%
1925	20	75	45	20	54,0%	59,6%	55,4%	55,6%	41,9%
1926	20	75	45	25	54,4%	59,3%	56,2%	57,3%	43,6%
1927	20	75	45	30	54,9%	59,0%	57,1%	59,0%	45,2%
1928	20	75	45	35	55,3%	58,6%	57,9%	60,7%	46,9%
1929	20	75	45	40	55,7%	58,3%	58,7%	62,3%	48,7%
1930	20	75	45	45	56,2%	58,0%	59,5%	63,9%	50,4%
1931	20	75	45	50	56,6%	57,7%	60,4%	65,5%	52,1%
1932	20	75	45	55	57,0%	57,4%	61,2%	67,0%	53,8%
1933	20	75	45	60	57,5%	57,0%	62,0%	68,5%	55,5%
1934	20	75	45	65	57,9%	56,7%	62,8%	70,0%	57,2%
1935	20	75	45	70	58,3%	56,4%	63,6%	71,4%	58,8%
1936	20	75	45	75	58,8%	56,1%	64,3%	72,8%	60,5%
1937	20	75	45	80	59,2%	55,8%	65,1%	74,1%	62,1%
1938	20	75	50	20	47,8%	53,9%	52,3%	54,1%	40,9%
1939	20	75	50	25	48,3%	53,6%	53,2%	55,8%	42,5%
1940	20	75	50	30	48,7%	53,3%	54,0%	57,5%	44,2%
1941	20	75	50	35	49,2%	52,9%	54,8%	59,2%	45,9%
1942	20	75	50	40	49,6%	52,6%	55,7%	60,8%	47,6%
1943	20	75	50	45	50,0%	52,3%	56,5%	62,5%	49,3%
1944	20	75	50	50	50,5%	52,0%	57,4%	64,1%	51,0%
1945	20	75	50	55	50,9%	51,6%	58,2%	65,7%	52,8%
1946	20	75	50	60	51,4%	51,3%	59,0%	67,2%	54,5%

1947	20	75	50	65	51,8%	51,0%	59,8%	68,7%	56,2%
1948	20	75	50	70	52,2%	50,7%	60,6%	70,2%	57,8%
1949	20	75	50	75	52,7%	50,3%	61,4%	71,6%	59,5%
1950	20	75	50	80	53,1%	50,0%	62,2%	73,0%	61,1%
1951	20	75	55	20	41,7%	48,2%	49,2%	52,6%	39,9%
1952	20	75	55	25	42,2%	47,8%	50,1%	54,3%	41,5%
1953	20	75	55	30	42,6%	47,5%	50,9%	56,0%	43,2%
1954	20	75	55	35	43,0%	47,2%	51,8%	57,7%	44,9%
1955	20	75	55	40	43,5%	46,9%	52,6%	59,4%	46,6%
1956	20	75	55	45	43,9%	46,5%	53,4%	61,0%	48,3%
1957	20	75	55	50	44,3%	46,2%	54,3%	62,7%	50,0%
1958	20	75	55	55	44,8%	45,9%	55,1%	64,3%	51,7%
1959	20	75	55	60	45,2%	45,6%	56,0%	65,8%	53,4%
1960	20	75	55	65	45,6%	45,2%	56,8%	67,4%	55,1%
1961	20	75	55	70	46,1%	44,9%	57,6%	68,9%	56,8%
1962	20	75	55	75	46,5%	44,6%	58,5%	70,3%	58,5%
1963	20	75	55	80	47,0%	44,3%	59,3%	71,7%	60,1%
1964	20	75	60	20	35,9%	42,4%	46,1%	51,1%	38,9%
1965	20	75	60	25	36,3%	42,1%	47,0%	52,8%	40,5%
1966	20	75	60	30	36,7%	41,8%	47,8%	54,5%	42,2%
1967	20	75	60	35	37,1%	41,5%	48,7%	56,2%	43,9%
1968	20	75	60	40	37,5%	41,2%	49,5%	57,9%	45,6%
1969	20	75	60	45	37,9%	40,9%	50,3%	59,6%	47,3%
1970	20	75	60	50	38,3%	40,5%	51,2%	61,2%	49,0%
1971	20	75	60	55	38,8%	40,2%	52,0%	62,9%	50,7%
1972	20	75	60	60	39,2%	39,9%	52,9%	64,4%	52,4%
1973	20	75	60	65	39,6%	39,6%	53,7%	66,0%	54,1%
1974	20	75	60	70	40,0%	39,3%	54,6%	67,5%	55,8%
1975	20	75	60	75	40,5%	39,0%	55,4%	69,0%	57,5%
1976	20	75	60	80	40,9%	38,7%	56,3%	70,5%	59,2%
1977	20	75	65	20	30,4%	36,9%	43,0%	49,6%	37,9%
1978	20	75	65	25	30,8%	36,6%	43,9%	51,3%	39,5%
1979	20	75	65	30	31,2%	36,3%	44,7%	53,0%	41,2%

1980	20	75	65	35	31,5%	36,0%	45,6%	54,7%	42,9%
1981	20	75	65	40	31,9%	35,7%	46,4%	56,4%	44,6%
1982	20	75	65	45	32,3%	35,4%	47,2%	58,1%	46,2%
1983	20	75	65	50	32,7%	35,1%	48,1%	59,8%	48,0%
1984	20	75	65	55	33,1%	34,8%	48,9%	61,4%	49,7%
1985	20	75	65	60	33,5%	34,5%	49,8%	63,0%	51,4%
1986	20	75	65	65	33,9%	34,2%	50,6%	64,6%	53,1%
1987	20	75	65	70	34,3%	33,9%	51,5%	66,2%	54,8%
1988	20	75	65	75	34,7%	33,6%	52,3%	67,7%	56,5%
1989	20	75	65	80	35,1%	33,3%	53,2%	69,2%	58,2%
1990	20	75	70	20	25,5%	31,7%	40,0%	48,0%	37,0%
1991	20	75	70	25	25,8%	31,4%	40,8%	49,8%	38,6%
1992	20	75	70	30	26,1%	31,2%	41,7%	51,5%	40,2%
1993	20	75	70	35	26,5%	30,9%	42,5%	53,2%	41,9%
1994	20	75	70	40	26,8%	30,6%	43,3%	54,9%	43,5%
1995	20	75	70	45	27,2%	30,3%	44,2%	56,6%	45,2%
1996	20	75	70	50	27,5%	30,0%	45,0%	58,3%	46,9%
1997	20	75	70	55	27,9%	29,8%	45,8%	60,0%	48,6%
1998	20	75	70	60	28,2%	29,5%	46,7%	61,6%	50,3%
1999	20	75	70	65	28,6%	29,2%	47,5%	63,2%	52,1%
2000	20	75	70	70	28,9%	29,0%	48,4%	64,8%	53,8%
2001	20	75	70	75	29,3%	28,7%	49,2%	66,4%	55,5%
2002	20	75	70	80	29,7%	28,4%	50,1%	67,9%	57,1%
2003	20	75	75	20	21,1%	26,9%	37,1%	46,5%	36,0%
2004	20	75	75	25	21,4%	26,7%	37,9%	48,2%	37,6%
2005	20	75	75	30	21,7%	26,4%	38,7%	50,0%	39,2%
2006	20	75	75	35	22,0%	26,2%	39,5%	51,7%	40,9%
2007	20	75	75	40	22,3%	25,9%	40,3%	53,4%	42,5%
2008	20	75	75	45	22,6%	25,7%	41,1%	55,1%	44,2%
2009	20	75	75	50	22,9%	25,4%	42,0%	56,8%	45,9%
2010	20	75	75	55	23,2%	25,2%	42,8%	58,5%	47,6%
2011	20	75	75	60	23,5%	24,9%	43,6%	60,2%	49,3%
2012	20	75	75	65	23,8%	24,7%	44,4%	61,8%	51,0%

2013	20	75	75	70	24,1%	24,4%	45,3%	63,4%	52,7%
2014	20	75	75	75	24,5%	24,2%	46,1%	65,0%	54,4%
2015	20	75	75	80	24,8%	24,0%	47,0%	66,5%	56,1%
2016	20	75	80	20	17,2%	22,6%	34,2%	45,0%	35,1%
2017	20	75	80	25	17,5%	22,4%	35,0%	46,7%	36,6%
2018	20	75	80	30	17,8%	22,2%	35,8%	48,4%	38,2%
2019	20	75	80	35	18,0%	22,0%	36,6%	50,2%	39,9%
2020	20	75	80	40	18,3%	21,7%	37,4%	51,9%	41,5%
2021	20	75	80	45	18,5%	21,5%	38,2%	53,6%	43,2%
2022	20	75	80	50	18,8%	21,3%	39,0%	55,3%	44,9%
2023	20	75	80	55	19,1%	21,1%	39,8%	57,0%	46,6%
2024	20	75	80	60	19,4%	20,9%	40,6%	58,7%	48,3%
2025	20	75	80	65	19,6%	20,7%	41,4%	60,3%	50,0%
2026	20	75	80	70	19,9%	20,4%	42,2%	62,0%	51,7%
2027	20	75	80	75	20,2%	20,2%	43,1%	63,6%	53,4%
2028	20	75	80	80	20,5%	20,0%	43,9%	65,2%	55,1%
2029	20	80	20	20	81,5%	83,5%	69,1%	61,2%	44,6%
2030	20	80	20	25	81,8%	83,3%	69,8%	62,8%	46,3%
2031	20	80	20	30	82,0%	83,1%	70,6%	64,4%	48,0%
2032	20	80	20	35	82,3%	83,0%	71,3%	66,0%	49,7%
2033	20	80	20	40	82,5%	82,8%	72,0%	67,5%	51,4%
2034	20	80	20	45	82,8%	82,6%	72,6%	69,0%	53,1%
2035	20	80	20	50	83,1%	82,4%	73,3%	70,5%	54,8%
2036	20	80	20	55	83,3%	82,2%	74,0%	71,9%	56,5%
2037	20	80	20	60	83,5%	82,0%	74,6%	73,3%	58,2%
2038	20	80	20	65	83,8%	81,8%	75,2%	74,6%	59,9%
2039	20	80	20	70	84,0%	81,6%	75,9%	75,9%	61,5%
2040	20	80	20	75	84,3%	81,4%	76,5%	77,1%	63,1%
2041	20	80	20	80	84,5%	81,2%	77,1%	78,3%	64,7%
2042	20	80	25	20	77,5%	80,1%	66,4%	59,8%	43,6%
2043	20	80	25	25	77,8%	79,9%	67,2%	61,4%	45,3%
2044	20	80	25	30	78,1%	79,6%	67,9%	63,0%	47,0%
2045	20	80	25	35	78,4%	79,4%	68,7%	64,6%	48,7%

2046	20	80	25	40	78,7%	79,2%	69,4%	66,2%	50,4%
2047	20	80	25	45	79,0%	79,0%	70,1%	67,7%	52,1%
2048	20	80	25	50	79,3%	78,8%	70,8%	69,2%	53,8%
2049	20	80	25	55	79,6%	78,6%	71,5%	70,7%	55,5%
2050	20	80	25	60	79,9%	78,3%	72,2%	72,1%	57,2%
2051	20	80	25	65	80,1%	78,1%	72,9%	73,4%	58,9%
2052	20	80	25	70	80,4%	77,9%	73,5%	74,8%	60,5%
2053	20	80	25	75	80,7%	77,7%	74,2%	76,0%	62,1%
2054	20	80	25	80	81,0%	77,4%	74,8%	77,3%	63,7%
2055	20	80	30	20	72,9%	76,1%	63,6%	58,3%	42,6%
2056	20	80	30	25	73,2%	75,9%	64,4%	60,0%	44,2%
2057	20	80	30	30	73,6%	75,6%	65,2%	61,6%	45,9%
2058	20	80	30	35	73,9%	75,4%	65,9%	63,2%	47,6%
2059	20	80	30	40	74,3%	75,2%	66,7%	64,8%	49,4%
2060	20	80	30	45	74,6%	74,9%	67,4%	66,4%	51,1%
2061	20	80	30	50	74,9%	74,7%	68,2%	67,9%	52,8%
2062	20	80	30	55	75,3%	74,4%	68,9%	69,4%	54,5%
2063	20	80	30	60	75,6%	74,2%	69,6%	70,8%	56,2%
2064	20	80	30	65	75,9%	73,9%	70,3%	72,2%	57,9%
2065	20	80	30	70	76,2%	73,7%	71,0%	73,6%	59,5%
2066	20	80	30	75	76,6%	73,4%	71,7%	74,9%	61,2%
2067	20	80	30	80	76,9%	73,2%	72,4%	76,2%	62,8%
2068	20	80	35	20	67,8%	71,7%	60,7%	56,8%	41,6%
2069	20	80	35	25	68,1%	71,4%	61,5%	58,5%	43,2%
2070	20	80	35	30	68,5%	71,1%	62,3%	60,2%	44,9%
2071	20	80	35	35	68,9%	70,9%	63,1%	61,8%	46,6%
2072	20	80	35	40	69,3%	70,6%	63,9%	63,4%	48,3%
2073	20	80	35	45	69,7%	70,3%	64,6%	65,0%	50,0%
2074	20	80	35	50	70,0%	70,1%	65,4%	66,5%	51,8%
2075	20	80	35	55	70,4%	69,8%	66,2%	68,1%	53,5%
2076	20	80	35	60	70,8%	69,5%	66,9%	69,5%	55,2%
2077	20	80	35	65	71,1%	69,2%	67,7%	71,0%	56,8%
2078	20	80	35	70	71,5%	69,0%	68,4%	72,4%	58,5%

2079	20	80	35	75	71,8%	68,7%	69,1%	73,7%	60,2%
2080	20	80	35	80	72,2%	68,4%	69,9%	75,1%	61,8%
2081	20	80	40	20	62,1%	66,8%	57,7%	55,3%	40,6%
2082	20	80	40	25	62,6%	66,5%	58,5%	57,0%	42,2%
2083	20	80	40	30	63,0%	66,2%	59,3%	58,7%	43,9%
2084	20	80	40	35	63,4%	65,9%	60,1%	60,3%	45,6%
2085	20	80	40	40	63,8%	65,6%	60,9%	62,0%	47,3%
2086	20	80	40	45	64,2%	65,3%	61,8%	63,6%	49,0%
2087	20	80	40	50	64,6%	65,0%	62,6%	65,2%	50,7%
2088	20	80	40	55	65,0%	64,7%	63,3%	66,7%	52,4%
2089	20	80	40	60	65,4%	64,4%	64,1%	68,2%	54,1%
2090	20	80	40	65	65,8%	64,1%	64,9%	69,7%	55,8%
2091	20	80	40	70	66,2%	63,8%	65,7%	71,1%	57,5%
2092	20	80	40	75	66,6%	63,5%	66,4%	72,5%	59,2%
2093	20	80	40	80	67,0%	63,2%	67,2%	73,9%	60,8%
2094	20	80	45	20	56,2%	61,5%	54,6%	53,8%	39,6%
2095	20	80	45	25	56,6%	61,2%	55,5%	55,5%	41,2%
2096	20	80	45	30	57,1%	60,8%	56,3%	57,2%	42,9%
2097	20	80	45	35	57,5%	60,5%	57,1%	58,9%	44,6%
2098	20	80	45	40	57,9%	60,2%	58,0%	60,5%	46,3%
2099	20	80	45	45	58,3%	59,9%	58,8%	62,2%	48,0%
2100	20	80	45	50	58,8%	59,6%	59,6%	63,8%	49,7%
2101	20	80	45	55	59,2%	59,3%	60,4%	65,4%	51,4%
2102	20	80	45	60	59,6%	59,0%	61,2%	66,9%	53,1%
2103	20	80	45	65	60,1%	58,6%	62,0%	68,4%	54,8%
2104	20	80	45	70	60,5%	58,3%	62,8%	69,9%	56,5%
2105	20	80	45	75	60,9%	58,0%	63,6%	71,3%	58,2%
2106	20	80	45	80	61,3%	57,7%	64,4%	72,7%	59,8%
2107	20	80	50	20	50,1%	55,9%	51,5%	52,3%	38,6%
2108	20	80	50	25	50,5%	55,5%	52,4%	54,0%	40,2%
2109	20	80	50	30	50,9%	55,2%	53,2%	55,7%	41,9%
2110	20	80	50	35	51,4%	54,9%	54,1%	57,4%	43,6%
2111	20	80	50	40	51,8%	54,6%	54,9%	59,1%	45,2%

2112	20	80	50	45	52,3%	54,3%	55,7%	60,7%	47,0%
2113	20	80	50	50	52,7%	53,9%	56,6%	62,4%	48,7%
2114	20	80	50	55	53,1%	53,6%	57,4%	64,0%	50,4%
2115	20	80	50	60	53,6%	53,3%	58,2%	65,5%	52,1%
2116	20	80	50	65	54,0%	53,0%	59,1%	67,1%	53,8%
2117	20	80	50	70	54,4%	52,6%	59,9%	68,6%	55,5%
2118	20	80	50	75	54,9%	52,3%	60,7%	70,0%	57,2%
2119	20	80	50	80	55,3%	52,0%	61,5%	71,5%	58,8%
2120	20	80	55	20	43,9%	50,1%	48,4%	50,7%	37,6%
2121	20	80	55	25	44,3%	49,8%	49,3%	52,5%	39,2%
2122	20	80	55	30	44,8%	49,5%	50,1%	54,2%	40,9%
2123	20	80	55	35	45,2%	49,1%	51,0%	55,9%	42,5%
2124	20	80	55	40	45,7%	48,8%	51,8%	57,6%	44,2%
2125	20	80	55	45	46,1%	48,5%	52,7%	59,3%	45,9%
2126	20	80	55	50	46,5%	48,2%	53,5%	60,9%	47,6%
2127	20	80	55	55	47,0%	47,8%	54,3%	62,5%	49,3%
2128	20	80	55	60	47,4%	47,5%	55,2%	64,1%	51,1%
2129	20	80	55	65	47,8%	47,2%	56,0%	65,7%	52,8%
2130	20	80	55	70	48,3%	46,9%	56,9%	67,3%	54,5%
2131	20	80	55	75	48,7%	46,5%	57,7%	68,8%	56,2%
2132	20	80	55	80	49,2%	46,2%	58,5%	70,2%	57,8%
2133	20	80	60	20	37,9%	44,4%	45,3%	49,2%	36,7%
2134	20	80	60	25	38,4%	44,1%	46,2%	50,9%	38,3%
2135	20	80	60	30	38,8%	43,7%	47,0%	52,7%	39,9%
2136	20	80	60	35	39,2%	43,4%	47,9%	54,4%	41,5%
2137	20	80	60	40	39,6%	43,1%	48,7%	56,1%	43,2%
2138	20	80	60	45	40,0%	42,8%	49,6%	57,8%	44,9%
2139	20	80	60	50	40,5%	42,4%	50,4%	59,5%	46,6%
2140	20	80	60	55	40,9%	42,1%	51,3%	61,1%	48,3%
2141	20	80	60	60	41,3%	41,8%	52,1%	62,7%	50,0%
2142	20	80	60	65	41,7%	41,5%	52,9%	64,3%	51,7%
2143	20	80	60	70	42,2%	41,2%	53,8%	65,9%	53,4%
2144	20	80	60	75	42,6%	40,9%	54,6%	67,4%	55,1%

2145	20	80	60	80	43,0%	40,5%	55,5%	68,9%	56,8%
2146	20	80	65	20	32,3%	38,8%	42,3%	47,7%	35,7%
2147	20	80	65	25	32,7%	38,5%	43,1%	49,4%	37,3%
2148	20	80	65	30	33,1%	38,2%	43,9%	51,1%	38,9%
2149	20	80	65	35	33,5%	37,8%	44,8%	52,9%	40,5%
2150	20	80	65	40	33,9%	37,5%	45,6%	54,6%	42,2%
2151	20	80	65	45	34,3%	37,2%	46,5%	56,3%	43,9%
2152	20	80	65	50	34,7%	36,9%	47,3%	58,0%	45,6%
2153	20	80	65	55	35,1%	36,6%	48,1%	59,6%	47,3%
2154	20	80	65	60	35,5%	36,3%	49,0%	61,3%	49,0%
2155	20	80	65	65	35,9%	36,0%	49,8%	62,9%	50,7%
2156	20	80	65	70	36,3%	35,7%	50,7%	64,5%	52,4%
2157	20	80	65	75	36,7%	35,4%	51,5%	66,1%	54,1%
2158	20	80	65	80	37,1%	35,1%	52,4%	67,6%	55,8%
2159	20	80	70	20	27,2%	33,5%	39,3%	46,2%	34,8%
2160	20	80	70	25	27,5%	33,2%	40,1%	47,9%	36,3%
2161	20	80	70	30	27,9%	32,9%	40,9%	49,6%	37,9%
2162	20	80	70	35	28,2%	32,6%	41,7%	51,3%	39,6%
2163	20	80	70	40	28,6%	32,3%	42,5%	53,1%	41,2%
2164	20	80	70	45	29,0%	32,0%	43,4%	54,8%	42,9%
2165	20	80	70	50	29,3%	31,7%	44,2%	56,5%	44,6%
2166	20	80	70	55	29,7%	31,4%	45,1%	58,2%	46,3%
2167	20	80	70	60	30,1%	31,2%	45,9%	59,8%	48,0%
2168	20	80	70	65	30,4%	30,9%	46,7%	61,5%	49,7%
2169	20	80	70	70	30,8%	30,6%	47,6%	63,1%	51,4%
2170	20	80	70	75	31,2%	30,3%	48,4%	64,7%	53,1%
2171	20	80	70	80	31,6%	30,1%	49,3%	66,3%	54,8%
2172	20	80	75	20	22,6%	28,5%	36,3%	44,7%	33,8%
2173	20	80	75	25	22,9%	28,3%	37,1%	46,4%	35,4%
2174	20	80	75	30	23,2%	28,0%	37,9%	48,1%	37,0%
2175	20	80	75	35	23,5%	27,7%	38,7%	49,8%	38,6%
2176	20	80	75	40	23,8%	27,5%	39,5%	51,5%	40,2%
2177	20	80	75	45	24,2%	27,2%	40,4%	53,3%	41,9%

2178	20	80	75	50	24,5%	26,9%	41,2%	55,0%	43,5%
2179	20	80	75	55	24,8%	26,7%	42,0%	56,7%	45,2%
2180	20	80	75	60	25,1%	26,4%	42,8%	58,4%	46,9%
2181	20	80	75	65	25,5%	26,2%	43,7%	60,0%	48,6%
2182	20	80	75	70	25,8%	25,9%	44,5%	61,7%	50,4%
2183	20	80	75	75	26,1%	25,7%	45,3%	63,3%	52,1%
2184	20	80	75	80	26,5%	25,4%	46,2%	64,9%	53,8%
2185	20	80	80	20	18,6%	24,1%	33,5%	43,2%	32,9%
2186	20	80	80	25	18,8%	23,8%	34,3%	44,9%	34,4%
2187	20	80	80	30	19,1%	23,6%	35,1%	46,6%	36,0%
2188	20	80	80	35	19,4%	23,3%	35,8%	48,3%	37,6%
2189	20	80	80	40	19,6%	23,1%	36,6%	50,0%	39,2%
2190	20	80	80	45	19,9%	22,9%	37,4%	51,7%	40,9%
2191	20	80	80	50	20,2%	22,7%	38,2%	53,5%	42,5%
2192	20	80	80	55	20,5%	22,4%	39,0%	55,2%	44,2%
2193	20	80	80	60	20,8%	22,2%	39,8%	56,9%	45,9%
2194	20	80	80	65	21,1%	22,0%	40,6%	58,6%	47,6%
2195	20	80	80	70	21,4%	21,7%	41,5%	60,2%	49,3%
2196	20	80	80	75	21,7%	21,5%	42,3%	61,9%	51,0%
2197	20	80	80	80	22,0%	21,3%	43,1%	63,5%	52,7%
2198	25	20	20	20	66,0%	73,3%	83,3%	85,2%	77,4%
2199	25	20	20	25	66,4%	73,1%	83,8%	86,0%	78,5%
2200	25	20	20	30	66,8%	72,8%	84,2%	86,8%	79,7%
2201	25	20	20	35	67,2%	72,5%	84,7%	87,6%	80,8%
2202	25	20	20	40	67,6%	72,3%	85,1%	88,3%	81,8%
2203	25	20	20	45	68,0%	72,0%	85,5%	89,0%	82,8%
2204	25	20	20	50	68,3%	71,8%	85,9%	89,7%	83,8%
2205	25	20	20	55	68,7%	71,5%	86,3%	90,3%	84,7%
2206	25	20	20	60	69,1%	71,2%	86,7%	90,9%	85,5%
2207	25	20	20	65	69,5%	71,0%	87,1%	91,4%	86,4%
2208	25	20	20	70	69,9%	70,7%	87,5%	92,0%	87,1%
2209	25	20	20	75	70,2%	70,4%	87,9%	92,5%	87,9%
2210	25	20	20	80	70,6%	70,1%	88,2%	92,9%	88,6%

2211	25	20	25	20	60,3%	68,6%	81,5%	84,4%	76,6%
2212	25	20	25	25	60,7%	68,3%	82,0%	85,3%	77,8%
2213	25	20	25	30	61,1%	68,0%	82,5%	86,1%	79,0%
2214	25	20	25	35	61,5%	67,7%	83,0%	86,9%	80,1%
2215	25	20	25	40	62,0%	67,4%	83,5%	87,7%	81,2%
2216	25	20	25	45	62,4%	67,1%	83,9%	88,4%	82,2%
2217	25	20	25	50	62,8%	66,9%	84,4%	89,1%	83,2%
2218	25	20	25	55	63,2%	66,6%	84,8%	89,8%	84,1%
2219	25	20	25	60	63,6%	66,3%	85,2%	90,4%	85,0%
2220	25	20	25	65	64,0%	66,0%	85,7%	91,0%	85,9%
2221	25	20	25	70	64,4%	65,7%	86,1%	91,5%	86,7%
2222	25	20	25	75	64,8%	65,4%	86,5%	92,0%	87,5%
2223	25	20	25	80	65,2%	65,1%	86,9%	92,5%	88,2%
2224	25	20	30	20	54,2%	63,4%	79,5%	83,6%	75,9%
2225	25	20	30	25	54,7%	63,1%	80,1%	84,5%	77,1%
2226	25	20	30	30	55,1%	62,8%	80,6%	85,4%	78,3%
2227	25	20	30	35	55,6%	62,5%	81,2%	86,2%	79,4%
2228	25	20	30	40	56,0%	62,2%	81,7%	87,0%	80,5%
2229	25	20	30	45	56,4%	61,9%	82,2%	87,8%	81,6%
2230	25	20	30	50	56,9%	61,6%	82,7%	88,5%	82,6%
2231	25	20	30	55	57,3%	61,2%	83,1%	89,2%	83,6%
2232	25	20	30	60	57,7%	60,9%	83,6%	89,8%	84,5%
2233	25	20	30	65	58,1%	60,6%	84,1%	90,4%	85,4%
2234	25	20	30	70	58,6%	60,3%	84,5%	91,0%	86,2%
2235	25	20	30	75	59,0%	60,0%	85,0%	91,6%	87,0%
2236	25	20	30	80	59,4%	59,7%	85,4%	92,1%	87,7%
2237	25	20	35	20	48,1%	57,9%	77,4%	82,7%	75,1%
2238	25	20	35	25	48,5%	57,6%	78,0%	83,7%	76,4%
2239	25	20	35	30	49,0%	57,2%	78,6%	84,6%	77,6%
2240	25	20	35	35	49,4%	56,9%	79,2%	85,5%	78,8%
2241	25	20	35	40	49,8%	56,6%	79,7%	86,3%	79,9%
2242	25	20	35	45	50,3%	56,3%	80,3%	87,1%	81,0%
2243	25	20	35	50	50,7%	56,0%	80,8%	87,9%	82,0%

2244	25	20	35	55	51,2%	55,6%	81,3%	88,6%	83,0%
2245	25	20	35	60	51,6%	55,3%	81,8%	89,3%	83,9%
2246	25	20	35	65	52,0%	55,0%	82,3%	89,9%	84,8%
2247	25	20	35	70	52,5%	54,7%	82,8%	90,5%	85,7%
2248	25	20	35	75	52,9%	54,3%	83,3%	91,1%	86,5%
2249	25	20	35	80	53,4%	54,0%	83,8%	91,6%	87,3%
2250	25	20	40	20	42,0%	52,2%	75,2%	81,8%	74,4%
2251	25	20	40	25	42,4%	51,9%	75,8%	82,8%	75,6%
2252	25	20	40	30	42,8%	51,5%	76,4%	83,8%	76,9%
2253	25	20	40	35	43,3%	51,2%	77,1%	84,7%	78,1%
2254	25	20	40	40	43,7%	50,9%	77,6%	85,6%	79,2%
2255	25	20	40	45	44,1%	50,5%	78,2%	86,4%	80,3%
2256	25	20	40	50	44,6%	50,2%	78,8%	87,2%	81,4%
2257	25	20	40	55	45,0%	49,9%	79,4%	87,9%	82,4%
2258	25	20	40	60	45,4%	49,6%	79,9%	88,7%	83,4%
2259	25	20	40	65	45,9%	49,2%	80,5%	89,3%	84,3%
2260	25	20	40	70	46,3%	48,9%	81,0%	90,0%	85,2%
2261	25	20	40	75	46,8%	48,6%	81,5%	90,6%	86,0%
2262	25	20	40	80	47,2%	48,3%	82,0%	91,2%	86,8%
2263	25	20	45	20	36,1%	46,4%	72,8%	80,9%	73,6%
2264	25	20	45	25	36,5%	46,1%	73,5%	81,9%	74,9%
2265	25	20	45	30	36,9%	45,8%	74,1%	82,9%	76,1%
2266	25	20	45	35	37,3%	45,4%	74,8%	83,9%	77,4%
2267	25	20	45	40	37,8%	45,1%	75,4%	84,8%	78,5%
2268	25	20	45	45	38,2%	44,8%	76,0%	85,7%	79,7%
2269	25	20	45	50	38,6%	44,5%	76,7%	86,5%	80,8%
2270	25	20	45	55	39,0%	44,1%	77,3%	87,3%	81,8%
2271	25	20	45	60	39,4%	43,8%	77,9%	88,0%	82,8%
2272	25	20	45	65	39,8%	43,5%	78,4%	88,7%	83,7%
2273	25	20	45	70	40,3%	43,2%	79,0%	89,4%	84,7%
2274	25	20	45	75	40,7%	42,9%	79,6%	90,0%	85,5%
2275	25	20	45	80	41,1%	42,5%	80,1%	90,6%	86,4%
2276	25	20	50	20	30,6%	40,7%	70,3%	79,9%	72,7%

2277	25	20	50	25	31,0%	40,4%	71,0%	81,0%	74,1%
2278	25	20	50	30	31,4%	40,1%	71,7%	82,1%	75,4%
2279	25	20	50	35	31,8%	39,8%	72,4%	83,1%	76,6%
2280	25	20	50	40	32,2%	39,5%	73,0%	84,0%	77,8%
2281	25	20	50	45	32,5%	39,2%	73,7%	84,9%	79,0%
2282	25	20	50	50	32,9%	38,9%	74,4%	85,8%	80,1%
2283	25	20	50	55	33,3%	38,5%	75,0%	86,6%	81,2%
2284	25	20	50	60	33,7%	38,2%	75,6%	87,4%	82,2%
2285	25	20	50	65	34,1%	37,9%	76,3%	88,1%	83,2%
2286	25	20	50	70	34,5%	37,6%	76,9%	88,8%	84,1%
2287	25	20	50	75	34,9%	37,3%	77,5%	89,5%	85,0%
2288	25	20	50	80	35,3%	37,0%	78,1%	90,1%	85,9%
2289	25	20	55	20	25,6%	35,3%	67,6%	78,9%	71,9%
2290	25	20	55	25	26,0%	35,0%	68,4%	80,1%	73,3%
2291	25	20	55	30	26,3%	34,7%	69,1%	81,1%	74,6%
2292	25	20	55	35	26,7%	34,4%	69,8%	82,2%	75,9%
2293	25	20	55	40	27,0%	34,1%	70,5%	83,2%	77,1%
2294	25	20	55	45	27,4%	33,8%	71,2%	84,1%	78,3%
2295	25	20	55	50	27,7%	33,5%	71,9%	85,0%	79,4%
2296	25	20	55	55	28,1%	33,2%	72,6%	85,9%	80,5%
2297	25	20	55	60	28,4%	33,0%	73,3%	86,7%	81,6%
2298	25	20	55	65	28,8%	32,7%	73,9%	87,5%	82,6%
2299	25	20	55	70	29,2%	32,4%	74,6%	88,2%	83,6%
2300	25	20	55	75	29,5%	32,1%	75,2%	88,9%	84,5%
2301	25	20	55	80	29,9%	31,8%	75,8%	89,6%	85,4%
2302	25	20	60	20	21,2%	30,2%	64,9%	77,9%	71,1%
2303	25	20	60	25	21,5%	29,9%	65,6%	79,1%	72,5%
2304	25	20	60	30	21,8%	29,7%	66,4%	80,2%	73,8%
2305	25	20	60	35	22,1%	29,4%	67,1%	81,3%	75,1%
2306	25	20	60	40	22,4%	29,1%	67,9%	82,3%	76,4%
2307	25	20	60	45	22,7%	28,9%	68,6%	83,3%	77,6%
2308	25	20	60	50	23,1%	28,6%	69,3%	84,2%	78,8%
2309	25	20	60	55	23,4%	28,3%	70,1%	85,1%	79,9%

2310	25	20	60	60	23,7%	28,1%	70,8%	86,0%	81,0%
2311	25	20	60	65	24,0%	27,8%	71,5%	86,8%	82,0%
2312	25	20	60	70	24,3%	27,5%	72,2%	87,5%	83,0%
2313	25	20	60	75	24,7%	27,3%	72,8%	88,3%	83,9%
2314	25	20	60	80	25,0%	27,0%	73,5%	89,0%	84,8%
2315	25	20	65	20	17,4%	25,6%	62,0%	76,8%	70,2%
2316	25	20	65	25	17,6%	25,3%	62,8%	78,1%	71,6%
2317	25	20	65	30	17,9%	25,1%	63,6%	79,2%	73,0%
2318	25	20	65	35	18,2%	24,8%	64,3%	80,3%	74,3%
2319	25	20	65	40	18,4%	24,6%	65,1%	81,4%	75,6%
2320	25	20	65	45	18,7%	24,4%	65,9%	82,4%	76,9%
2321	25	20	65	50	19,0%	24,1%	66,6%	83,4%	78,1%
2322	25	20	65	55	19,2%	23,9%	67,4%	84,3%	79,2%
2323	25	20	65	60	19,5%	23,6%	68,1%	85,2%	80,3%
2324	25	20	65	65	19,8%	23,4%	68,9%	86,1%	81,4%
2325	25	20	65	70	20,1%	23,2%	69,6%	86,9%	82,4%
2326	25	20	65	75	20,4%	22,9%	70,3%	87,6%	83,4%
2327	25	20	65	80	20,6%	22,7%	71,0%	88,4%	84,3%
2328	25	20	70	20	14,1%	21,4%	59,0%	75,7%	69,4%
2329	25	20	70	25	14,3%	21,2%	59,8%	77,0%	70,8%
2330	25	20	70	30	14,6%	21,0%	60,6%	78,2%	72,2%
2331	25	20	70	35	14,8%	20,8%	61,4%	79,3%	73,5%
2332	25	20	70	40	15,0%	20,6%	62,2%	80,5%	74,9%
2333	25	20	70	45	15,2%	20,4%	63,0%	81,5%	76,1%
2334	25	20	70	50	15,5%	20,1%	63,8%	82,5%	77,3%
2335	25	20	70	55	15,7%	19,9%	64,6%	83,5%	78,5%
2336	25	20	70	60	15,9%	19,7%	65,4%	84,4%	79,7%
2337	25	20	70	65	16,2%	19,5%	66,1%	85,3%	80,7%
2338	25	20	70	70	16,4%	19,3%	66,9%	86,2%	81,8%
2339	25	20	70	75	16,6%	19,1%	67,6%	87,0%	82,8%
2340	25	20	70	80	16,9%	18,9%	68,4%	87,7%	83,7%
2341	25	20	75	20	11,4%	17,8%	56,0%	74,6%	68,5%
2342	25	20	75	25	11,6%	17,6%	56,8%	75,9%	69,9%

2343	25	20	75	30	11,7%	17,4%	57,6%	77,1%	71,4%
2344	25	20	75	35	11,9%	17,2%	58,5%	78,3%	72,7%
2345	25	20	75	40	12,1%	17,1%	59,3%	79,5%	74,1%
2346	25	20	75	45	12,3%	16,9%	60,1%	80,6%	75,4%
2347	25	20	75	50	12,5%	16,7%	60,9%	81,6%	76,6%
2348	25	20	75	55	12,7%	16,5%	61,7%	82,6%	77,8%
2349	25	20	75	60	12,9%	16,3%	62,5%	83,6%	79,0%
2350	25	20	75	65	13,1%	16,1%	63,3%	84,5%	80,1%
2351	25	20	75	70	13,3%	16,0%	64,1%	85,4%	81,2%
2352	25	20	75	75	13,5%	15,8%	64,9%	86,3%	82,2%
2353	25	20	75	80	13,7%	15,6%	65,6%	87,1%	83,2%
2354	25	20	80	20	9,1%	14,7%	52,9%	73,4%	67,6%
2355	25	20	80	25	9,3%	14,5%	53,7%	74,8%	69,1%
2356	25	20	80	30	9,4%	14,3%	54,6%	76,0%	70,5%
2357	25	20	80	35	9,6%	14,2%	55,4%	77,3%	71,9%
2358	25	20	80	40	9,7%	14,0%	56,3%	78,5%	73,3%
2359	25	20	80	45	9,9%	13,9%	57,1%	79,6%	74,6%
2360	25	20	80	50	10,0%	13,7%	57,9%	80,7%	75,9%
2361	25	20	80	55	10,2%	13,6%	58,7%	81,8%	77,1%
2362	25	20	80	60	10,4%	13,4%	59,6%	82,8%	78,3%
2363	25	20	80	65	10,5%	13,3%	60,4%	83,7%	79,4%
2364	25	20	80	70	10,7%	13,1%	61,2%	84,6%	80,5%
2365	25	20	80	75	10,9%	13,0%	62,0%	85,5%	81,6%
2366	25	20	80	80	11,0%	12,8%	62,8%	86,3%	82,6%
2367	25	25	20	20	68,0%	74,8%	82,8%	84,2%	75,7%
2368	25	25	20	25	68,4%	74,6%	83,3%	85,1%	76,9%
2369	25	25	20	30	68,7%	74,3%	83,8%	86,0%	78,1%
2370	25	25	20	35	69,1%	74,1%	84,2%	86,8%	79,2%
2371	25	25	20	40	69,5%	73,8%	84,7%	87,5%	80,3%
2372	25	25	20	45	69,9%	73,6%	85,1%	88,3%	81,4%
2373	25	25	20	50	70,2%	73,3%	85,6%	89,0%	82,4%
2374	25	25	20	55	70,6%	73,1%	86,0%	89,6%	83,4%
2375	25	25	20	60	71,0%	72,8%	86,4%	90,3%	84,3%

2376	25	25	20	65	71,3%	72,5%	86,8%	90,8%	85,2%
2377	25	25	20	70	71,7%	72,3%	87,1%	91,4%	86,0%
2378	25	25	20	75	72,0%	72,0%	87,5%	91,9%	86,8%
2379	25	25	20	80	72,4%	71,8%	87,9%	92,4%	87,6%
2380	25	25	25	20	62,4%	70,2%	81,0%	83,4%	74,9%
2381	25	25	25	25	62,8%	70,0%	81,5%	84,3%	76,2%
2382	25	25	25	30	63,2%	69,7%	82,0%	85,2%	77,4%
2383	25	25	25	35	63,6%	69,4%	82,5%	86,1%	78,5%
2384	25	25	25	40	64,0%	69,1%	83,0%	86,9%	79,7%
2385	25	25	25	45	64,4%	68,9%	83,5%	87,6%	80,8%
2386	25	25	25	50	64,8%	68,6%	83,9%	88,4%	81,8%
2387	25	25	25	55	65,2%	68,3%	84,4%	89,1%	82,8%
2388	25	25	25	60	65,6%	68,0%	84,8%	89,7%	83,8%
2389	25	25	25	65	66,0%	67,7%	85,3%	90,3%	84,7%
2390	25	25	25	70	66,4%	67,4%	85,7%	90,9%	85,5%
2391	25	25	25	75	66,8%	67,1%	86,1%	91,5%	86,4%
2392	25	25	25	80	67,2%	66,9%	86,5%	92,0%	87,2%
2393	25	25	30	20	56,4%	65,2%	79,0%	82,5%	74,1%
2394	25	25	30	25	56,9%	64,9%	79,6%	83,5%	75,4%
2395	25	25	30	30	57,3%	64,6%	80,1%	84,4%	76,6%
2396	25	25	30	35	57,7%	64,3%	80,7%	85,3%	77,8%
2397	25	25	30	40	58,2%	64,0%	81,2%	86,2%	79,0%
2398	25	25	30	45	58,6%	63,7%	81,7%	87,0%	80,1%
2399	25	25	30	50	59,0%	63,4%	82,2%	87,7%	81,2%
2400	25	25	30	55	59,4%	63,1%	82,7%	88,4%	82,2%
2401	25	25	30	60	59,9%	62,8%	83,2%	89,1%	83,2%
2402	25	25	30	65	60,3%	62,5%	83,6%	89,8%	84,1%
2403	25	25	30	70	60,7%	62,2%	84,1%	90,4%	85,0%
2404	25	25	30	75	61,1%	61,9%	84,6%	91,0%	85,9%
2405	25	25	30	80	61,5%	61,6%	85,0%	91,5%	86,7%
2406	25	25	35	20	50,3%	59,8%	76,9%	81,6%	73,3%
2407	25	25	35	25	50,7%	59,5%	77,5%	82,6%	74,6%
2408	25	25	35	30	51,2%	59,2%	78,1%	83,6%	75,9%

2409	25	25	35	35	51,6%	58,8%	78,6%	84,5%	77,1%
2410	25	25	35	40	52,1%	58,5%	79,2%	85,4%	78,3%
2411	25	25	35	45	52,5%	58,2%	79,8%	86,3%	79,5%
2412	25	25	35	50	52,9%	57,9%	80,3%	87,0%	80,5%
2413	25	25	35	55	53,4%	57,6%	80,8%	87,8%	81,6%
2414	25	25	35	60	53,8%	57,3%	81,4%	88,5%	82,6%
2415	25	25	35	65	54,3%	56,9%	81,9%	89,2%	83,6%
2416	25	25	35	70	54,7%	56,6%	82,4%	89,9%	84,5%
2417	25	25	35	75	55,1%	56,3%	82,9%	90,5%	85,4%
2418	25	25	35	80	55,6%	56,0%	83,3%	91,0%	86,2%
2419	25	25	40	20	44,2%	54,1%	74,6%	80,7%	72,5%
2420	25	25	40	25	44,6%	53,8%	75,2%	81,7%	73,8%
2421	25	25	40	30	45,0%	53,5%	75,9%	82,8%	75,1%
2422	25	25	40	35	45,5%	53,2%	76,5%	83,7%	76,4%
2423	25	25	40	40	45,9%	52,8%	77,1%	84,6%	77,6%
2424	25	25	40	45	46,3%	52,5%	77,7%	85,5%	78,8%
2425	25	25	40	50	46,8%	52,2%	78,3%	86,3%	79,9%
2426	25	25	40	55	47,2%	51,9%	78,8%	87,1%	81,0%
2427	25	25	40	60	47,7%	51,5%	79,4%	87,9%	82,0%
2428	25	25	40	65	48,1%	51,2%	80,0%	88,6%	83,0%
2429	25	25	40	70	48,5%	50,9%	80,5%	89,3%	83,9%
2430	25	25	40	75	49,0%	50,5%	81,0%	89,9%	84,8%
2431	25	25	40	80	49,4%	50,2%	81,5%	90,5%	85,7%
2432	25	25	45	20	38,2%	48,4%	72,2%	79,7%	71,7%
2433	25	25	45	25	38,6%	48,0%	72,9%	80,8%	73,0%
2434	25	25	45	30	39,0%	47,7%	73,5%	81,9%	74,4%
2435	25	25	45	35	39,4%	47,4%	74,2%	82,9%	75,6%
2436	25	25	45	40	39,9%	47,1%	74,8%	83,8%	76,9%
2437	25	25	45	45	40,3%	46,7%	75,5%	84,7%	78,1%
2438	25	25	45	50	40,7%	46,4%	76,1%	85,6%	79,2%
2439	25	25	45	55	41,1%	46,1%	76,7%	86,4%	80,3%
2440	25	25	45	60	41,6%	45,8%	77,3%	87,2%	81,4%
2441	25	25	45	65	42,0%	45,4%	77,9%	88,0%	82,4%

2442	25	25	45	70	42,4%	45,1%	78,5%	88,7%	83,4%
2443	25	25	45	75	42,9%	44,8%	79,0%	89,4%	84,3%
2444	25	25	45	80	43,3%	44,5%	79,6%	90,0%	85,2%
2445	25	25	50	20	32,5%	42,7%	69,6%	78,7%	70,8%
2446	25	25	50	25	32,9%	42,3%	70,3%	79,9%	72,2%
2447	25	25	50	30	33,3%	42,0%	71,0%	80,9%	73,6%
2448	25	25	50	35	33,7%	41,7%	71,7%	82,0%	74,9%
2449	25	25	50	40	34,1%	41,4%	72,4%	83,0%	76,1%
2450	25	25	50	45	34,5%	41,1%	73,1%	83,9%	77,4%
2451	25	25	50	50	34,9%	40,7%	73,8%	84,8%	78,5%
2452	25	25	50	55	35,3%	40,4%	74,4%	85,7%	79,7%
2453	25	25	50	60	35,7%	40,1%	75,0%	86,5%	80,8%
2454	25	25	50	65	36,1%	39,8%	75,7%	87,3%	81,8%
2455	25	25	50	70	36,5%	39,5%	76,3%	88,1%	82,8%
2456	25	25	50	75	36,9%	39,2%	76,9%	88,8%	83,8%
2457	25	25	50	80	37,4%	38,9%	77,5%	89,4%	84,7%
2458	25	25	55	20	27,4%	37,1%	66,9%	77,7%	70,0%
2459	25	25	55	25	27,7%	36,8%	67,7%	78,9%	71,4%
2460	25	25	55	30	28,1%	36,5%	68,4%	80,0%	72,8%
2461	25	25	55	35	28,4%	36,2%	69,1%	81,1%	74,1%
2462	25	25	55	40	28,8%	35,9%	69,9%	82,1%	75,4%
2463	25	25	55	45	29,2%	35,6%	70,6%	83,1%	76,6%
2464	25	25	55	50	29,5%	35,3%	71,3%	84,0%	77,8%
2465	25	25	55	55	29,9%	35,0%	72,0%	84,9%	79,0%
2466	25	25	55	60	30,3%	34,7%	72,6%	85,8%	80,1%
2467	25	25	55	65	30,6%	34,4%	73,3%	86,6%	81,2%
2468	25	25	55	70	31,0%	34,1%	74,0%	87,4%	82,2%
2469	25	25	55	75	31,4%	33,8%	74,6%	88,1%	83,2%
2470	25	25	55	80	31,8%	33,5%	75,3%	88,8%	84,1%
2471	25	25	60	20	22,8%	31,9%	64,1%	76,6%	69,1%
2472	25	25	60	25	23,1%	31,6%	64,9%	77,8%	70,5%
2473	25	25	60	30	23,4%	31,3%	65,7%	79,0%	71,9%
2474	25	25	60	35	23,7%	31,1%	66,4%	80,1%	73,3%

2475	25	25	60	40	24,0%	30,8%	67,2%	81,2%	74,6%
2476	25	25	60	45	24,3%	30,5%	67,9%	82,2%	75,9%
2477	25	25	60	50	24,7%	30,2%	68,7%	83,2%	77,1%
2478	25	25	60	55	25,0%	30,0%	69,4%	84,2%	78,3%
2479	25	25	60	60	25,3%	29,7%	70,1%	85,0%	79,4%
2480	25	25	60	65	25,7%	29,4%	70,8%	85,9%	80,5%
2481	25	25	60	70	26,0%	29,1%	71,5%	86,7%	81,6%
2482	25	25	60	75	26,3%	28,9%	72,2%	87,5%	82,6%
2483	25	25	60	80	26,7%	28,6%	72,9%	88,2%	83,6%
2484	25	25	65	20	18,7%	27,1%	61,2%	75,5%	68,2%
2485	25	25	65	25	19,0%	26,9%	62,0%	76,8%	69,7%
2486	25	25	65	30	19,2%	26,6%	62,8%	78,0%	71,1%
2487	25	25	65	35	19,5%	26,3%	63,6%	79,1%	72,5%
2488	25	25	65	40	19,8%	26,1%	64,4%	80,2%	73,8%
2489	25	25	65	45	20,1%	25,8%	65,2%	81,3%	75,1%
2490	25	25	65	50	20,4%	25,6%	65,9%	82,3%	76,4%
2491	25	25	65	55	20,7%	25,3%	66,7%	83,3%	77,6%
2492	25	25	65	60	20,9%	25,1%	67,4%	84,3%	78,8%
2493	25	25	65	65	21,2%	24,9%	68,2%	85,1%	79,9%
2494	25	25	65	70	21,5%	24,6%	68,9%	86,0%	81,0%
2495	25	25	65	75	21,8%	24,4%	69,6%	86,8%	82,0%
2496	25	25	65	80	22,1%	24,1%	70,4%	87,6%	83,0%
2497	25	25	70	20	15,2%	22,8%	58,2%	74,4%	67,3%
2498	25	25	70	25	15,5%	22,6%	59,1%	75,7%	68,8%
2499	25	25	70	30	15,7%	22,3%	59,9%	76,9%	70,2%
2500	25	25	70	35	15,9%	22,1%	60,7%	78,1%	71,6%
2501	25	25	70	40	16,2%	21,9%	61,5%	79,3%	73,0%
2502	25	25	70	45	16,4%	21,7%	62,3%	80,4%	74,3%
2503	25	25	70	50	16,7%	21,4%	63,1%	81,4%	75,6%
2504	25	25	70	55	16,9%	21,2%	63,9%	82,5%	76,9%
2505	25	25	70	60	17,1%	21,0%	64,7%	83,4%	78,1%
2506	25	25	70	65	17,4%	20,8%	65,4%	84,4%	79,2%
2507	25	25	70	70	17,7%	20,6%	66,2%	85,3%	80,3%

2508	25	25	70	75	17,9%	20,4%	66,9%	86,1%	81,4%
2509	25	25	70	80	18,2%	20,2%	67,7%	86,9%	82,4%
2510	25	25	75	20	12,3%	19,0%	55,2%	73,2%	66,4%
2511	25	25	75	25	12,5%	18,8%	56,0%	74,5%	67,9%
2512	25	25	75	30	12,7%	18,6%	56,9%	75,8%	69,4%
2513	25	25	75	35	12,9%	18,4%	57,7%	77,0%	70,8%
2514	25	25	75	40	13,1%	18,2%	58,5%	78,2%	72,2%
2515	25	25	75	45	13,3%	18,0%	59,3%	79,4%	73,5%
2516	25	25	75	50	13,5%	17,8%	60,2%	80,5%	74,9%
2517	25	25	75	55	13,7%	17,6%	61,0%	81,6%	76,1%
2518	25	25	75	60	13,9%	17,4%	61,8%	82,6%	77,3%
2519	25	25	75	65	14,1%	17,2%	62,6%	83,5%	78,5%
2520	25	25	75	70	14,3%	17,1%	63,4%	84,5%	79,7%
2521	25	25	75	75	14,6%	16,9%	64,1%	85,4%	80,7%
2522	25	25	75	80	14,8%	16,7%	64,9%	86,2%	81,8%
2523	25	25	80	20	9,9%	15,7%	52,1%	72,0%	65,4%
2524	25	25	80	25	10,0%	15,5%	52,9%	73,3%	67,0%
2525	25	25	80	30	10,2%	15,3%	53,8%	74,7%	68,5%
2526	25	25	80	35	10,4%	15,2%	54,6%	75,9%	69,9%
2527	25	25	80	40	10,5%	15,0%	55,5%	77,2%	71,4%
2528	25	25	80	45	10,7%	14,8%	56,3%	78,4%	72,7%
2529	25	25	80	50	10,9%	14,7%	57,1%	79,5%	74,1%
2530	25	25	80	55	11,0%	14,5%	58,0%	80,6%	75,4%
2531	25	25	80	60	11,2%	14,4%	58,8%	81,7%	76,6%
2532	25	25	80	65	11,4%	14,2%	59,6%	82,7%	77,8%
2533	25	25	80	70	11,6%	14,0%	60,4%	83,7%	79,0%
2534	25	25	80	75	11,8%	13,9%	61,2%	84,6%	80,1%
2535	25	25	80	80	11,9%	13,7%	62,0%	85,5%	81,2%
2536	25	30	20	20	69,9%	76,3%	82,4%	83,2%	73,9%
2537	25	30	20	25	70,2%	76,0%	82,9%	84,1%	75,2%
2538	25	30	20	30	70,6%	75,8%	83,4%	85,0%	76,4%
2539	25	30	20	35	71,0%	75,6%	83,8%	85,9%	77,6%
2540	25	30	20	40	71,3%	75,3%	84,3%	86,7%	78,8%

2541	25	30	20	45	71,7%	75,1%	84,7%	87,5%	79,9%
2542	25	30	20	50	72,1%	74,8%	85,2%	88,2%	81,0%
2543	25	30	20	55	72,4%	74,6%	85,6%	88,9%	82,0%
2544	25	30	20	60	72,8%	74,3%	86,0%	89,6%	83,0%
2545	25	30	20	65	73,1%	74,1%	86,4%	90,2%	84,0%
2546	25	30	20	70	73,5%	73,8%	86,8%	90,8%	84,9%
2547	25	30	20	75	73,8%	73,6%	87,2%	91,4%	85,7%
2548	25	30	20	80	74,1%	73,3%	87,5%	91,9%	86,5%
2549	25	30	25	20	64,4%	71,9%	80,5%	82,3%	73,0%
2550	25	30	25	25	64,8%	71,6%	81,0%	83,3%	74,4%
2551	25	30	25	30	65,2%	71,3%	81,6%	84,3%	75,7%
2552	25	30	25	35	65,6%	71,1%	82,1%	85,1%	76,9%
2553	25	30	25	40	66,0%	70,8%	82,6%	86,0%	78,1%
2554	25	30	25	45	66,4%	70,5%	83,0%	86,8%	79,2%
2555	25	30	25	50	66,8%	70,2%	83,5%	87,6%	80,3%
2556	25	30	25	55	67,2%	70,0%	84,0%	88,3%	81,4%
2557	25	30	25	60	67,6%	69,7%	84,4%	89,0%	82,4%
2558	25	30	25	65	68,0%	69,4%	84,9%	89,7%	83,4%
2559	25	30	25	70	68,4%	69,1%	85,3%	90,3%	84,3%
2560	25	30	25	75	68,8%	68,9%	85,7%	90,9%	85,2%
2561	25	30	25	80	69,1%	68,6%	86,1%	91,4%	86,0%
2562	25	30	30	20	58,6%	67,0%	78,5%	81,4%	72,2%
2563	25	30	30	25	59,0%	66,7%	79,1%	82,5%	73,6%
2564	25	30	30	30	59,5%	66,4%	79,6%	83,4%	74,9%
2565	25	30	30	35	59,9%	66,1%	80,2%	84,4%	76,2%
2566	25	30	30	40	60,3%	65,8%	80,7%	85,2%	77,4%
2567	25	30	30	45	60,7%	65,5%	81,2%	86,1%	78,6%
2568	25	30	30	50	61,1%	65,2%	81,7%	86,9%	79,7%
2569	25	30	30	55	61,6%	64,9%	82,2%	87,7%	80,8%
2570	25	30	30	60	62,0%	64,6%	82,7%	88,4%	81,8%
2571	25	30	30	65	62,4%	64,3%	83,2%	89,1%	82,8%
2572	25	30	30	70	62,8%	64,0%	83,7%	89,7%	83,8%
2573	25	30	30	75	63,2%	63,7%	84,1%	90,4%	84,7%

2574	25	30	30	80	63,6%	63,4%	84,6%	90,9%	85,5%
2575	25	30	35	20	52,5%	61,7%	76,3%	80,5%	71,4%
2576	25	30	35	25	53,0%	61,4%	76,9%	81,6%	72,8%
2577	25	30	35	30	53,4%	61,1%	77,5%	82,6%	74,1%
2578	25	30	35	35	53,8%	60,7%	78,1%	83,5%	75,4%
2579	25	30	35	40	54,3%	60,4%	78,7%	84,5%	76,6%
2580	25	30	35	45	54,7%	60,1%	79,2%	85,3%	77,9%
2581	25	30	35	50	55,1%	59,8%	79,8%	86,2%	79,0%
2582	25	30	35	55	55,6%	59,5%	80,3%	87,0%	80,1%
2583	25	30	35	60	56,0%	59,2%	80,9%	87,8%	81,2%
2584	25	30	35	65	56,4%	58,9%	81,4%	88,5%	82,2%
2585	25	30	35	70	56,9%	58,5%	81,9%	89,2%	83,2%
2586	25	30	35	75	57,3%	58,2%	82,4%	89,8%	84,1%
2587	25	30	35	80	57,7%	57,9%	82,9%	90,4%	85,0%
2588	25	30	40	20	46,4%	56,1%	74,0%	79,5%	70,5%
2589	25	30	40	25	46,8%	55,8%	74,6%	80,6%	71,9%
2590	25	30	40	30	47,2%	55,4%	75,3%	81,7%	73,3%
2591	25	30	40	35	47,7%	55,1%	75,9%	82,7%	74,6%
2592	25	30	40	40	48,1%	54,8%	76,5%	83,6%	75,9%
2593	25	30	40	45	48,6%	54,5%	77,1%	84,6%	77,1%
2594	25	30	40	50	49,0%	54,1%	77,7%	85,4%	78,3%
2595	25	30	40	55	49,4%	53,8%	78,3%	86,3%	79,5%
2596	25	30	40	60	49,9%	53,5%	78,9%	87,1%	80,6%
2597	25	30	40	65	50,3%	53,2%	79,4%	87,8%	81,6%
2598	25	30	40	70	50,8%	52,8%	80,0%	88,6%	82,6%
2599	25	30	40	75	51,2%	52,5%	80,5%	89,2%	83,6%
2600	25	30	40	80	51,6%	52,2%	81,1%	89,9%	84,5%
2601	25	30	45	20	40,3%	50,3%	71,5%	78,5%	69,7%
2602	25	30	45	25	40,7%	50,0%	72,2%	79,6%	71,1%
2603	25	30	45	30	41,1%	49,7%	72,9%	80,7%	72,5%
2604	25	30	45	35	41,6%	49,4%	73,6%	81,8%	73,8%
2605	25	30	45	40	42,0%	49,0%	74,2%	82,8%	75,1%
2606	25	30	45	45	42,4%	48,7%	74,9%	83,8%	76,4%

2607	25	30	45	50	42,9%	48,4%	75,5%	84,7%	77,6%
2608	25	30	45	55	43,3%	48,1%	76,1%	85,5%	78,8%
2609	25	30	45	60	43,7%	47,7%	76,7%	86,4%	79,9%
2610	25	30	45	65	44,2%	47,4%	77,3%	87,2%	81,0%
2611	25	30	45	70	44,6%	47,1%	77,9%	87,9%	82,0%
2612	25	30	45	75	45,0%	46,7%	78,5%	88,6%	83,0%
2613	25	30	45	80	45,5%	46,4%	79,1%	89,3%	83,9%
2614	25	30	50	20	34,5%	44,6%	68,9%	77,5%	68,8%
2615	25	30	50	25	34,9%	44,3%	69,7%	78,6%	70,3%
2616	25	30	50	30	35,3%	43,9%	70,4%	79,8%	71,7%
2617	25	30	50	35	35,7%	43,6%	71,1%	80,9%	73,0%
2618	25	30	50	40	36,1%	43,3%	71,8%	81,9%	74,4%
2619	25	30	50	45	36,5%	43,0%	72,5%	82,9%	75,6%
2620	25	30	50	50	37,0%	42,7%	73,1%	83,9%	76,9%
2621	25	30	50	55	37,4%	42,3%	73,8%	84,8%	78,1%
2622	25	30	50	60	37,8%	42,0%	74,4%	85,6%	79,2%
2623	25	30	50	65	38,2%	41,7%	75,1%	86,5%	80,3%
2624	25	30	50	70	38,6%	41,4%	75,7%	87,3%	81,4%
2625	25	30	50	75	39,0%	41,1%	76,3%	88,0%	82,4%
2626	25	30	50	80	39,5%	40,8%	76,9%	88,7%	83,4%
2627	25	30	55	20	29,2%	39,0%	66,2%	76,4%	67,9%
2628	25	30	55	25	29,5%	38,7%	67,0%	77,6%	69,4%
2629	25	30	55	30	29,9%	38,4%	67,7%	78,8%	70,8%
2630	25	30	55	35	30,3%	38,1%	68,5%	79,9%	72,2%
2631	25	30	55	40	30,7%	37,7%	69,2%	81,0%	73,6%
2632	25	30	55	45	31,0%	37,4%	69,9%	82,0%	74,9%
2633	25	30	55	50	31,4%	37,1%	70,6%	83,0%	76,1%
2634	25	30	55	55	31,8%	36,8%	71,3%	84,0%	77,4%
2635	25	30	55	60	32,2%	36,5%	72,0%	84,9%	78,5%
2636	25	30	55	65	32,6%	36,2%	72,7%	85,7%	79,7%
2637	25	30	55	70	32,9%	35,9%	73,4%	86,6%	80,8%
2638	25	30	55	75	33,3%	35,6%	74,0%	87,3%	81,8%
2639	25	30	55	80	33,7%	35,3%	74,7%	88,1%	82,8%

2640	25	30	60	20	24,3%	33,6%	63,4%	75,3%	67,0%
2641	25	30	60	25	24,7%	33,4%	64,2%	76,5%	68,5%
2642	25	30	60	30	25,0%	33,1%	65,0%	77,7%	70,0%
2643	25	30	60	35	25,3%	32,8%	65,7%	78,9%	71,4%
2644	25	30	60	40	25,7%	32,5%	66,5%	80,0%	72,8%
2645	25	30	60	45	26,0%	32,2%	67,2%	81,1%	74,1%
2646	25	30	60	50	26,3%	31,9%	68,0%	82,1%	75,4%
2647	25	30	60	55	26,7%	31,6%	68,7%	83,1%	76,6%
2648	25	30	60	60	27,0%	31,4%	69,4%	84,1%	77,8%
2649	25	30	60	65	27,4%	31,1%	70,2%	85,0%	79,0%
2650	25	30	60	70	27,7%	30,8%	70,9%	85,8%	80,1%
2651	25	30	60	75	28,1%	30,5%	71,6%	86,7%	81,2%
2652	25	30	60	80	28,5%	30,2%	72,2%	87,4%	82,2%
2653	25	30	65	20	20,1%	28,7%	60,5%	74,1%	66,1%
2654	25	30	65	25	20,4%	28,4%	61,3%	75,4%	67,6%
2655	25	30	65	30	20,7%	28,2%	62,1%	76,7%	69,1%
2656	25	30	65	35	21,0%	27,9%	62,9%	77,9%	70,5%
2657	25	30	65	40	21,2%	27,6%	63,7%	79,0%	71,9%
2658	25	30	65	45	21,5%	27,4%	64,4%	80,2%	73,3%
2659	25	30	65	50	21,8%	27,1%	65,2%	81,2%	74,6%
2660	25	30	65	55	22,1%	26,9%	66,0%	82,3%	75,9%
2661	25	30	65	60	22,4%	26,6%	66,7%	83,2%	77,1%
2662	25	30	65	65	22,8%	26,4%	67,5%	84,2%	78,3%
2663	25	30	65	70	23,1%	26,1%	68,2%	85,1%	79,4%
2664	25	30	65	75	23,4%	25,8%	69,0%	85,9%	80,5%
2665	25	30	65	80	23,7%	25,6%	69,7%	86,8%	81,6%
2666	25	30	70	20	16,4%	24,2%	57,5%	72,9%	65,2%
2667	25	30	70	25	16,7%	24,0%	58,3%	74,3%	66,7%
2668	25	30	70	30	16,9%	23,7%	59,1%	75,6%	68,2%
2669	25	30	70	35	17,2%	23,5%	59,9%	76,8%	69,7%
2670	25	30	70	40	17,4%	23,3%	60,7%	78,0%	71,1%
2671	25	30	70	45	17,7%	23,0%	61,5%	79,2%	72,5%
2672	25	30	70	50	17,9%	22,8%	62,3%	80,3%	73,8%

2673	25	30	70	55	18,2%	22,6%	63,1%	81,4%	75,1%
2674	25	30	70	60	18,4%	22,3%	63,9%	82,4%	76,4%
2675	25	30	70	65	18,7%	22,1%	64,7%	83,4%	77,6%
2676	25	30	70	70	19,0%	21,9%	65,5%	84,3%	78,8%
2677	25	30	70	75	19,3%	21,7%	66,2%	85,2%	79,9%
2678	25	30	70	80	19,5%	21,5%	67,0%	86,0%	81,0%
2679	25	30	75	20	13,3%	20,2%	54,4%	71,7%	64,2%
2680	25	30	75	25	13,5%	20,0%	55,2%	73,1%	65,8%
2681	25	30	75	30	13,7%	19,8%	56,1%	74,4%	67,3%
2682	25	30	75	35	13,9%	19,6%	56,9%	75,7%	68,8%
2683	25	30	75	40	14,1%	19,4%	57,7%	77,0%	70,2%
2684	25	30	75	45	14,4%	19,2%	58,6%	78,2%	71,6%
2685	25	30	75	50	14,6%	19,0%	59,4%	79,3%	73,0%
2686	25	30	75	55	14,8%	18,8%	60,2%	80,4%	74,3%
2687	25	30	75	60	15,0%	18,6%	61,0%	81,5%	75,6%
2688	25	30	75	65	15,2%	18,4%	61,8%	82,5%	76,9%
2689	25	30	75	70	15,5%	18,2%	62,6%	83,5%	78,1%
2690	25	30	75	75	15,7%	18,0%	63,4%	84,4%	79,2%
2691	25	30	75	80	15,9%	17,8%	64,2%	85,3%	80,3%
2692	25	30	80	20	10,7%	16,8%	51,3%	70,4%	63,3%
2693	25	30	80	25	10,9%	16,6%	52,2%	71,9%	64,8%
2694	25	30	80	30	11,0%	16,4%	53,0%	73,2%	66,4%
2695	25	30	80	35	11,2%	16,2%	53,8%	74,6%	67,9%
2696	25	30	80	40	11,4%	16,0%	54,7%	75,9%	69,4%
2697	25	30	80	45	11,6%	15,9%	55,5%	77,1%	70,8%
2698	25	30	80	50	11,8%	15,7%	56,4%	78,3%	72,2%
2699	25	30	80	55	11,9%	15,5%	57,2%	79,4%	73,6%
2700	25	30	80	60	12,1%	15,3%	58,0%	80,5%	74,9%
2701	25	30	80	65	12,3%	15,2%	58,8%	81,6%	76,1%
2702	25	30	80	70	12,5%	15,0%	59,7%	82,6%	77,4%
2703	25	30	80	75	12,7%	14,8%	60,5%	83,6%	78,5%
2704	25	30	80	80	12,9%	14,7%	61,3%	84,5%	79,7%
2705	25	35	20	20	71,7%	77,7%	81,9%	82,1%	72,0%

2706	25	35	20	25	72,1%	77,5%	82,4%	83,1%	73,3%
2707	25	35	20	30	72,4%	77,2%	82,9%	84,1%	74,6%
2708	25	35	20	35	72,8%	77,0%	83,4%	85,0%	75,9%
2709	25	35	20	40	73,1%	76,8%	83,8%	85,8%	77,1%
2710	25	35	20	45	73,5%	76,5%	84,3%	86,7%	78,3%
2711	25	35	20	50	73,8%	76,3%	84,7%	87,4%	79,5%
2712	25	35	20	55	74,1%	76,1%	85,2%	88,2%	80,6%
2713	25	35	20	60	74,5%	75,8%	85,6%	88,9%	81,6%
2714	25	35	20	65	74,8%	75,6%	86,0%	89,5%	82,6%
2715	25	35	20	70	75,1%	75,3%	86,4%	90,2%	83,6%
2716	25	35	20	75	75,5%	75,1%	86,8%	90,8%	84,5%
2717	25	35	20	80	75,8%	74,8%	87,2%	91,3%	85,4%
2718	25	35	25	20	66,4%	73,4%	80,0%	81,2%	71,1%
2719	25	35	25	25	66,8%	73,2%	80,5%	82,3%	72,5%
2720	25	35	25	30	67,2%	72,9%	81,1%	83,2%	73,9%
2721	25	35	25	35	67,6%	72,7%	81,6%	84,2%	75,2%
2722	25	35	25	40	68,0%	72,4%	82,1%	85,1%	76,4%
2723	25	35	25	45	68,4%	72,1%	82,6%	85,9%	77,6%
2724	25	35	25	50	68,8%	71,9%	83,1%	86,7%	78,8%
2725	25	35	25	55	69,1%	71,6%	83,5%	87,5%	79,9%
2726	25	35	25	60	69,5%	71,3%	84,0%	88,3%	81,0%
2727	25	35	25	65	69,9%	71,1%	84,5%	89,0%	82,0%
2728	25	35	25	70	70,3%	70,8%	84,9%	89,6%	83,0%
2729	25	35	25	75	70,6%	70,5%	85,3%	90,2%	84,0%
2730	25	35	25	80	71,0%	70,2%	85,7%	90,8%	84,9%
2731	25	35	30	20	60,7%	68,7%	77,9%	80,3%	70,3%
2732	25	35	30	25	61,2%	68,4%	78,5%	81,4%	71,7%
2733	25	35	30	30	61,6%	68,1%	79,1%	82,4%	73,1%
2734	25	35	30	35	62,0%	67,8%	79,6%	83,4%	74,4%
2735	25	35	30	40	62,4%	67,5%	80,2%	84,3%	75,7%
2736	25	35	30	45	62,8%	67,3%	80,7%	85,2%	76,9%
2737	25	35	30	50	63,2%	67,0%	81,3%	86,0%	78,1%
2738	25	35	30	55	63,6%	66,7%	81,8%	86,8%	79,2%

2739	25	35	30	60	64,0%	66,4%	82,3%	87,6%	80,3%
2740	25	35	30	65	64,5%	66,1%	82,8%	88,3%	81,4%
2741	25	35	30	70	64,9%	65,8%	83,2%	89,0%	82,4%
2742	25	35	30	75	65,3%	65,5%	83,7%	89,7%	83,4%
2743	25	35	30	80	65,7%	65,2%	84,2%	90,3%	84,3%
2744	25	35	35	20	54,7%	63,5%	75,7%	79,3%	69,4%
2745	25	35	35	25	55,2%	63,2%	76,4%	80,4%	70,8%
2746	25	35	35	30	55,6%	62,9%	77,0%	81,5%	72,2%
2747	25	35	35	35	56,0%	62,6%	77,6%	82,5%	73,6%
2748	25	35	35	40	56,5%	62,3%	78,1%	83,5%	74,9%
2749	25	35	35	45	56,9%	62,0%	78,7%	84,4%	76,2%
2750	25	35	35	50	57,3%	61,7%	79,3%	85,3%	77,4%
2751	25	35	35	55	57,8%	61,4%	79,8%	86,1%	78,6%
2752	25	35	35	60	58,2%	61,1%	80,4%	86,9%	79,7%
2753	25	35	35	65	58,6%	60,7%	80,9%	87,7%	80,8%
2754	25	35	35	70	59,0%	60,4%	81,4%	88,4%	81,8%
2755	25	35	35	75	59,5%	60,1%	81,9%	89,1%	82,8%
2756	25	35	35	80	59,9%	59,8%	82,4%	89,8%	83,8%
2757	25	35	40	20	48,6%	58,0%	73,4%	78,3%	68,5%
2758	25	35	40	25	49,0%	57,7%	74,0%	79,4%	70,0%
2759	25	35	40	30	49,4%	57,4%	74,7%	80,5%	71,4%
2760	25	35	40	35	49,9%	57,1%	75,3%	81,6%	72,8%
2761	25	35	40	40	50,3%	56,7%	76,0%	82,6%	74,1%
2762	25	35	40	45	50,8%	56,4%	76,6%	83,6%	75,4%
2763	25	35	40	50	51,2%	56,1%	77,2%	84,5%	76,7%
2764	25	35	40	55	51,6%	55,8%	77,8%	85,4%	77,9%
2765	25	35	40	60	52,1%	55,4%	78,3%	86,2%	79,0%
2766	25	35	40	65	52,5%	55,1%	78,9%	87,0%	80,1%
2767	25	35	40	70	53,0%	54,8%	79,5%	87,8%	81,2%
2768	25	35	40	75	53,4%	54,5%	80,0%	88,5%	82,2%
2769	25	35	40	80	53,8%	54,2%	80,6%	89,2%	83,2%
2770	25	35	45	20	42,4%	52,3%	70,9%	77,2%	67,6%
2771	25	35	45	25	42,9%	52,0%	71,6%	78,4%	69,1%

2772	25	35	45	30	43,3%	51,7%	72,3%	79,6%	70,5%
2773	25	35	45	35	43,7%	51,3%	72,9%	80,7%	72,0%
2774	25	35	45	40	44,2%	51,0%	73,6%	81,7%	73,3%
2775	25	35	45	45	44,6%	50,7%	74,3%	82,7%	74,6%
2776	25	35	45	50	45,1%	50,3%	74,9%	83,7%	75,9%
2777	25	35	45	55	45,5%	50,0%	75,5%	84,6%	77,1%
2778	25	35	45	60	45,9%	49,7%	76,2%	85,5%	78,3%
2779	25	35	45	65	46,4%	49,4%	76,8%	86,3%	79,5%
2780	25	35	45	70	46,8%	49,0%	77,4%	87,1%	80,6%
2781	25	35	45	75	47,2%	48,7%	78,0%	87,9%	81,6%
2782	25	35	45	80	47,7%	48,4%	78,5%	88,6%	82,6%
2783	25	35	50	20	36,6%	46,5%	68,3%	76,1%	66,7%
2784	25	35	50	25	37,0%	46,2%	69,0%	77,4%	68,2%
2785	25	35	50	30	37,4%	45,9%	69,7%	78,6%	69,7%
2786	25	35	50	35	37,8%	45,6%	70,4%	79,7%	71,1%
2787	25	35	50	40	38,2%	45,2%	71,1%	80,8%	72,5%
2788	25	35	50	45	38,6%	44,9%	71,8%	81,8%	73,8%
2789	25	35	50	50	39,0%	44,6%	72,5%	82,8%	75,1%
2790	25	35	50	55	39,5%	44,3%	73,2%	83,8%	76,4%
2791	25	35	50	60	39,9%	44,0%	73,8%	84,7%	77,6%
2792	25	35	50	65	40,3%	43,6%	74,5%	85,6%	78,8%
2793	25	35	50	70	40,7%	43,3%	75,1%	86,4%	79,9%
2794	25	35	50	75	41,2%	43,0%	75,8%	87,2%	81,0%
2795	25	35	50	80	41,6%	42,7%	76,4%	88,0%	82,0%
2796	25	35	55	20	31,0%	40,9%	65,5%	75,0%	65,8%
2797	25	35	55	25	31,4%	40,6%	66,3%	76,3%	67,3%
2798	25	35	55	30	31,8%	40,2%	67,0%	77,5%	68,8%
2799	25	35	55	35	32,2%	39,9%	67,8%	78,7%	70,3%
2800	25	35	55	40	32,6%	39,6%	68,5%	79,8%	71,7%
2801	25	35	55	45	33,0%	39,3%	69,2%	80,9%	73,0%
2802	25	35	55	50	33,4%	39,0%	70,0%	82,0%	74,4%
2803	25	35	55	55	33,7%	38,7%	70,7%	83,0%	75,6%
2804	25	35	55	60	34,1%	38,4%	71,4%	83,9%	76,9%

2805	25	35	55	65	34,5%	38,1%	72,1%	84,8%	78,1%
2806	25	35	55	70	34,9%	37,7%	72,7%	85,7%	79,2%
2807	25	35	55	75	35,3%	37,4%	73,4%	86,5%	80,3%
2808	25	35	55	80	35,7%	37,1%	74,1%	87,3%	81,4%
2809	25	35	60	20	26,0%	35,4%	62,7%	73,9%	64,9%
2810	25	35	60	25	26,4%	35,1%	63,4%	75,2%	66,4%
2811	25	35	60	30	26,7%	34,8%	64,2%	76,4%	67,9%
2812	25	35	60	35	27,0%	34,5%	65,0%	77,6%	69,4%
2813	25	35	60	40	27,4%	34,2%	65,8%	78,8%	70,8%
2814	25	35	60	45	27,7%	33,9%	66,5%	80,0%	72,2%
2815	25	35	60	50	28,1%	33,7%	67,3%	81,0%	73,6%
2816	25	35	60	55	28,5%	33,4%	68,0%	82,1%	74,9%
2817	25	35	60	60	28,8%	33,1%	68,8%	83,1%	76,1%
2818	25	35	60	65	29,2%	32,8%	69,5%	84,0%	77,4%
2819	25	35	60	70	29,6%	32,5%	70,2%	84,9%	78,5%
2820	25	35	60	75	29,9%	32,2%	70,9%	85,8%	79,7%
2821	25	35	60	80	30,3%	31,9%	71,6%	86,6%	80,8%
2822	25	35	65	20	21,6%	30,3%	59,7%	72,7%	63,9%
2823	25	35	65	25	21,9%	30,1%	60,5%	74,0%	65,5%
2824	25	35	65	30	22,2%	29,8%	61,3%	75,3%	67,0%
2825	25	35	65	35	22,5%	29,5%	62,1%	76,6%	68,5%
2826	25	35	65	40	22,8%	29,2%	62,9%	77,8%	70,0%
2827	25	35	65	45	23,1%	29,0%	63,7%	79,0%	71,4%
2828	25	35	65	50	23,4%	28,7%	64,5%	80,1%	72,8%
2829	25	35	65	55	23,7%	28,4%	65,3%	81,2%	74,1%
2830	25	35	65	60	24,0%	28,2%	66,0%	82,2%	75,4%
2831	25	35	65	65	24,4%	27,9%	66,8%	83,2%	76,6%
2832	25	35	65	70	24,7%	27,6%	67,5%	84,1%	77,8%
2833	25	35	65	75	25,0%	27,4%	68,3%	85,0%	79,0%
2834	25	35	65	80	25,3%	27,1%	69,0%	85,9%	80,1%
2835	25	35	70	20	17,7%	25,7%	56,7%	71,4%	63,0%
2836	25	35	70	25	17,9%	25,4%	57,5%	72,8%	64,5%
2837	25	35	70	30	18,2%	25,2%	58,3%	74,2%	66,1%

2838	25	35	70	35	18,5%	24,9%	59,2%	75,5%	67,6%
2839	25	35	70	40	18,7%	24,7%	60,0%	76,7%	69,1%
2840	25	35	70	45	19,0%	24,5%	60,8%	77,9%	70,5%
2841	25	35	70	50	19,3%	24,2%	61,6%	79,1%	71,9%
2842	25	35	70	55	19,5%	24,0%	62,4%	80,2%	73,3%
2843	25	35	70	60	19,8%	23,7%	63,2%	81,3%	74,6%
2844	25	35	70	65	20,1%	23,5%	64,0%	82,3%	75,9%
2845	25	35	70	70	20,4%	23,3%	64,8%	83,3%	77,1%
2846	25	35	70	75	20,7%	23,0%	65,5%	84,2%	78,3%
2847	25	35	70	80	21,0%	22,8%	66,3%	85,1%	79,5%
2848	25	35	75	20	14,4%	21,5%	53,6%	70,2%	62,0%
2849	25	35	75	25	14,6%	21,3%	54,5%	71,6%	63,6%
2850	25	35	75	30	14,8%	21,1%	55,3%	73,0%	65,2%
2851	25	35	75	35	15,0%	20,9%	56,1%	74,3%	66,7%
2852	25	35	75	40	15,2%	20,7%	57,0%	75,6%	68,2%
2853	25	35	75	45	15,5%	20,4%	57,8%	76,9%	69,7%
2854	25	35	75	50	15,7%	20,2%	58,6%	78,1%	71,1%
2855	25	35	75	55	15,9%	20,0%	59,4%	79,2%	72,5%
2856	25	35	75	60	16,2%	19,8%	60,3%	80,3%	73,8%
2857	25	35	75	65	16,4%	19,6%	61,1%	81,4%	75,1%
2858	25	35	75	70	16,7%	19,4%	61,9%	82,4%	76,4%
2859	25	35	75	75	16,9%	19,2%	62,7%	83,4%	77,6%
2860	25	35	75	80	17,2%	19,0%	63,5%	84,3%	78,8%
2861	25	35	80	20	11,6%	17,9%	50,5%	68,9%	61,0%
2862	25	35	80	25	11,8%	17,7%	51,4%	70,3%	62,6%
2863	25	35	80	30	11,9%	17,5%	52,2%	71,8%	64,2%
2864	25	35	80	35	12,1%	17,3%	53,1%	73,1%	65,8%
2865	25	35	80	40	12,3%	17,1%	53,9%	74,5%	67,3%
2866	25	35	80	45	12,5%	16,9%	54,7%	75,8%	68,8%
2867	25	35	80	50	12,7%	16,8%	55,6%	77,0%	70,2%
2868	25	35	80	55	12,9%	16,6%	56,4%	78,2%	71,7%
2869	25	35	80	60	13,1%	16,4%	57,2%	79,4%	73,0%
2870	25	35	80	65	13,3%	16,2%	58,1%	80,5%	74,4%

2871	25	35	80	70	13,5%	16,0%	58,9%	81,5%	75,6%
2872	25	35	80	75	13,7%	15,9%	59,7%	82,5%	76,9%
2873	25	35	80	80	13,9%	15,7%	60,5%	83,5%	78,1%
2874	25	40	20	20	73,5%	79,0%	81,4%	81,0%	70,0%
2875	25	40	20	25	73,8%	78,8%	82,0%	82,1%	71,4%
2876	25	40	20	30	74,2%	78,6%	82,5%	83,1%	72,8%
2877	25	40	20	35	74,5%	78,4%	82,9%	84,0%	74,1%
2878	25	40	20	40	74,8%	78,1%	83,4%	84,9%	75,4%
2879	25	40	20	45	75,2%	77,9%	83,9%	85,8%	76,7%
2880	25	40	20	50	75,5%	77,7%	84,3%	86,6%	77,9%
2881	25	40	20	55	75,8%	77,5%	84,8%	87,4%	79,0%
2882	25	40	20	60	76,1%	77,2%	85,2%	88,1%	80,1%
2883	25	40	20	65	76,5%	77,0%	85,6%	88,8%	81,2%
2884	25	40	20	70	76,8%	76,8%	86,0%	89,5%	82,2%
2885	25	40	20	75	77,1%	76,5%	86,4%	90,1%	83,2%
2886	25	40	20	80	77,4%	76,3%	86,8%	90,7%	84,1%
2887	25	40	25	20	68,4%	74,9%	79,5%	80,1%	69,1%
2888	25	40	25	25	68,8%	74,7%	80,0%	81,2%	70,6%
2889	25	40	25	30	69,2%	74,4%	80,6%	82,2%	72,0%
2890	25	40	25	35	69,5%	74,2%	81,1%	83,2%	73,3%
2891	25	40	25	40	69,9%	73,9%	81,6%	84,1%	74,6%
2892	25	40	25	45	70,3%	73,7%	82,1%	85,0%	75,9%
2893	25	40	25	50	70,6%	73,4%	82,6%	85,9%	77,2%
2894	25	40	25	55	71,0%	73,2%	83,1%	86,7%	78,3%
2895	25	40	25	60	71,4%	72,9%	83,6%	87,5%	79,5%
2896	25	40	25	65	71,7%	72,7%	84,0%	88,2%	80,6%
2897	25	40	25	70	72,1%	72,4%	84,5%	88,9%	81,6%
2898	25	40	25	75	72,4%	72,1%	84,9%	89,6%	82,6%
2899	25	40	25	80	72,8%	71,9%	85,4%	90,2%	83,6%
2900	25	40	30	20	62,8%	70,4%	77,4%	79,1%	68,2%
2901	25	40	30	25	63,2%	70,1%	78,0%	80,2%	69,7%
2902	25	40	30	30	63,7%	69,8%	78,6%	81,3%	71,1%
2903	25	40	30	35	64,1%	69,5%	79,1%	82,3%	72,5%

2904	25	40	30	40	64,5%	69,2%	79,7%	83,3%	73,9%
2905	25	40	30	45	64,9%	69,0%	80,2%	84,2%	75,2%
2906	25	40	30	50	65,3%	68,7%	80,8%	85,1%	76,4%
2907	25	40	30	55	65,7%	68,4%	81,3%	86,0%	77,6%
2908	25	40	30	60	66,1%	68,1%	81,8%	86,8%	78,8%
2909	25	40	30	65	66,5%	67,8%	82,3%	87,6%	79,9%
2910	25	40	30	70	66,8%	67,6%	82,8%	88,3%	81,0%
2911	25	40	30	75	67,2%	67,3%	83,3%	89,0%	82,0%
2912	25	40	30	80	67,6%	67,0%	83,7%	89,6%	83,0%
2913	25	40	35	20	56,9%	65,3%	75,2%	78,1%	67,3%
2914	25	40	35	25	57,3%	65,0%	75,8%	79,2%	68,8%
2915	25	40	35	30	57,8%	64,7%	76,4%	80,3%	70,3%
2916	25	40	35	35	58,2%	64,4%	77,0%	81,4%	71,7%
2917	25	40	35	40	58,6%	64,1%	77,6%	82,4%	73,1%
2918	25	40	35	45	59,1%	63,8%	78,2%	83,4%	74,4%
2919	25	40	35	50	59,5%	63,5%	78,8%	84,3%	75,7%
2920	25	40	35	55	59,9%	63,2%	79,3%	85,2%	76,9%
2921	25	40	35	60	60,3%	62,9%	79,9%	86,1%	78,1%
2922	25	40	35	65	60,8%	62,6%	80,4%	86,9%	79,2%
2923	25	40	35	70	61,2%	62,3%	80,9%	87,6%	80,4%
2924	25	40	35	75	61,6%	62,0%	81,5%	88,4%	81,4%
2925	25	40	35	80	62,0%	61,7%	82,0%	89,1%	82,4%
2926	25	40	40	20	50,8%	59,9%	72,8%	77,0%	66,4%
2927	25	40	40	25	51,2%	59,6%	73,4%	78,2%	67,9%
2928	25	40	40	30	51,7%	59,3%	74,1%	79,3%	69,4%
2929	25	40	40	35	52,1%	59,0%	74,7%	80,5%	70,8%
2930	25	40	40	40	52,5%	58,7%	75,4%	81,5%	72,2%
2931	25	40	40	45	53,0%	58,3%	76,0%	82,5%	73,6%
2932	25	40	40	50	53,4%	58,0%	76,6%	83,5%	74,9%
2933	25	40	40	55	53,9%	57,7%	77,2%	84,4%	76,2%
2934	25	40	40	60	54,3%	57,4%	77,8%	85,3%	77,4%
2935	25	40	40	65	54,7%	57,1%	78,4%	86,2%	78,6%
2936	25	40	40	70	55,2%	56,7%	79,0%	87,0%	79,7%

2937	25	40	40	75	55,6%	56,4%	79,5%	87,7%	80,8%
2938	25	40	40	80	56,0%	56,1%	80,1%	88,5%	81,8%
2939	25	40	45	20	44,6%	54,3%	70,2%	75,9%	65,5%
2940	25	40	45	25	45,1%	53,9%	70,9%	77,1%	67,0%
2941	25	40	45	30	45,5%	53,6%	71,6%	78,3%	68,5%
2942	25	40	45	35	45,9%	53,3%	72,3%	79,5%	70,0%
2943	25	40	45	40	46,4%	53,0%	73,0%	80,6%	71,4%
2944	25	40	45	45	46,8%	52,6%	73,7%	81,6%	72,8%
2945	25	40	45	50	47,3%	52,3%	74,3%	82,7%	74,1%
2946	25	40	45	55	47,7%	52,0%	74,9%	83,6%	75,4%
2947	25	40	45	60	48,1%	51,7%	75,6%	84,5%	76,7%
2948	25	40	45	65	48,6%	51,3%	76,2%	85,4%	77,9%
2949	25	40	45	70	49,0%	51,0%	76,8%	86,3%	79,0%
2950	25	40	45	75	49,5%	50,7%	77,4%	87,1%	80,1%
2951	25	40	45	80	49,9%	50,4%	78,0%	87,8%	81,2%
2952	25	40	50	20	38,6%	48,5%	67,6%	74,8%	64,6%
2953	25	40	50	25	39,1%	48,2%	68,3%	76,0%	66,1%
2954	25	40	50	30	39,5%	47,9%	69,0%	77,3%	67,6%
2955	25	40	50	35	39,9%	47,5%	69,8%	78,5%	69,1%
2956	25	40	50	40	40,3%	47,2%	70,5%	79,6%	70,6%
2957	25	40	50	45	40,7%	46,9%	71,2%	80,7%	72,0%
2958	25	40	50	50	41,2%	46,6%	71,9%	81,8%	73,3%
2959	25	40	50	55	41,6%	46,2%	72,5%	82,8%	74,6%
2960	25	40	50	60	42,0%	45,9%	73,2%	83,7%	75,9%
2961	25	40	50	65	42,5%	45,6%	73,9%	84,6%	77,1%
2962	25	40	50	70	42,9%	45,3%	74,5%	85,5%	78,3%
2963	25	40	50	75	43,3%	44,9%	75,2%	86,4%	79,5%
2964	25	40	50	80	43,8%	44,6%	75,8%	87,1%	80,6%
2965	25	40	55	20	33,0%	42,8%	64,8%	73,6%	63,6%
2966	25	40	55	25	33,4%	42,5%	65,6%	74,9%	65,2%
2967	25	40	55	30	33,8%	42,1%	66,3%	76,2%	66,7%
2968	25	40	55	35	34,2%	41,8%	67,1%	77,4%	68,2%
2969	25	40	55	40	34,5%	41,5%	67,8%	78,6%	69,7%

2970	25	40	55	45	34,9%	41,2%	68,6%	79,7%	71,1%
2971	25	40	55	50	35,4%	40,9%	69,3%	80,8%	72,5%
2972	25	40	55	55	35,8%	40,6%	70,0%	81,9%	73,8%
2973	25	40	55	60	36,2%	40,2%	70,7%	82,9%	75,1%
2974	25	40	55	65	36,6%	39,9%	71,4%	83,8%	76,4%
2975	25	40	55	70	37,0%	39,6%	72,1%	84,7%	77,6%
2976	25	40	55	75	37,4%	39,3%	72,8%	85,6%	78,8%
2977	25	40	55	80	37,8%	39,0%	73,4%	86,4%	79,9%
2978	25	40	60	20	27,8%	37,2%	61,9%	72,4%	62,7%
2979	25	40	60	25	28,1%	36,9%	62,7%	73,8%	64,2%
2980	25	40	60	30	28,5%	36,6%	63,5%	75,1%	65,8%
2981	25	40	60	35	28,8%	36,3%	64,3%	76,3%	67,3%
2982	25	40	60	40	29,2%	36,0%	65,1%	77,6%	68,8%
2983	25	40	60	45	29,6%	35,7%	65,8%	78,7%	70,3%
2984	25	40	60	50	29,9%	35,4%	66,6%	79,9%	71,7%
2985	25	40	60	55	30,3%	35,1%	67,3%	81,0%	73,0%
2986	25	40	60	60	30,7%	34,8%	68,1%	82,0%	74,4%
2987	25	40	60	65	31,1%	34,5%	68,8%	83,0%	75,7%
2988	25	40	60	70	31,4%	34,2%	69,5%	83,9%	76,9%
2989	25	40	60	75	31,8%	34,0%	70,2%	84,9%	78,1%
2990	25	40	60	80	32,2%	33,7%	71,0%	85,7%	79,2%
2991	25	40	65	20	23,1%	32,0%	58,9%	71,2%	61,7%
2992	25	40	65	25	23,4%	31,7%	59,8%	72,6%	63,3%
2993	25	40	65	30	23,7%	31,5%	60,6%	73,9%	64,9%
2994	25	40	65	35	24,0%	31,2%	61,4%	75,2%	66,4%
2995	25	40	65	40	24,4%	30,9%	62,2%	76,5%	67,9%
2996	25	40	65	45	24,7%	30,6%	63,0%	77,7%	69,4%
2997	25	40	65	50	25,0%	30,3%	63,8%	78,9%	70,8%
2998	25	40	65	55	25,4%	30,1%	64,5%	80,0%	72,2%
2999	25	40	65	60	25,7%	29,8%	65,3%	81,1%	73,6%
3000	25	40	65	65	26,0%	29,5%	66,1%	82,1%	74,9%
3001	25	40	65	70	26,4%	29,2%	66,8%	83,1%	76,2%
3002	25	40	65	75	26,7%	29,0%	67,6%	84,1%	77,4%

3003	25	40	65	80	27,1%	28,7%	68,3%	85,0%	78,6%
3004	25	40	70	20	19,0%	27,2%	55,9%	69,9%	60,7%
3005	25	40	70	25	19,3%	27,0%	56,7%	71,3%	62,3%
3006	25	40	70	30	19,5%	26,7%	57,6%	72,7%	63,9%
3007	25	40	70	35	19,8%	26,5%	58,4%	74,1%	65,5%
3008	25	40	70	40	20,1%	26,2%	59,2%	75,4%	67,0%
3009	25	40	70	45	20,4%	25,9%	60,0%	76,6%	68,5%
3010	25	40	70	50	20,7%	25,7%	60,8%	77,8%	70,0%
3011	25	40	70	55	21,0%	25,4%	61,7%	79,0%	71,4%
3012	25	40	70	60	21,3%	25,2%	62,5%	80,1%	72,8%
3013	25	40	70	65	21,6%	25,0%	63,2%	81,2%	74,1%
3014	25	40	70	70	21,9%	24,7%	64,0%	82,2%	75,4%
3015	25	40	70	75	22,2%	24,5%	64,8%	83,2%	76,6%
3016	25	40	70	80	22,5%	24,2%	65,6%	84,2%	77,8%
3017	25	40	75	20	15,5%	22,9%	52,8%	68,6%	59,7%
3018	25	40	75	25	15,7%	22,7%	53,7%	70,1%	61,4%
3019	25	40	75	30	16,0%	22,4%	54,5%	71,5%	63,0%
3020	25	40	75	35	16,2%	22,2%	55,3%	72,9%	64,5%
3021	25	40	75	40	16,4%	22,0%	56,2%	74,2%	66,1%
3022	25	40	75	45	16,7%	21,8%	57,0%	75,5%	67,6%
3023	25	40	75	50	16,9%	21,5%	57,8%	76,8%	69,1%
3024	25	40	75	55	17,2%	21,3%	58,7%	78,0%	70,5%
3025	25	40	75	60	17,4%	21,1%	59,5%	79,1%	71,9%
3026	25	40	75	65	17,7%	20,9%	60,3%	80,3%	73,3%
3027	25	40	75	70	17,9%	20,7%	61,1%	81,3%	74,6%
3028	25	40	75	75	18,2%	20,5%	61,9%	82,3%	75,9%
3029	25	40	75	80	18,5%	20,2%	62,7%	83,3%	77,1%
3030	25	40	80	20	12,5%	19,1%	49,7%	67,3%	58,7%
3031	25	40	80	25	12,7%	18,9%	50,6%	68,8%	60,4%
3032	25	40	80	30	12,9%	18,7%	51,4%	70,2%	62,0%
3033	25	40	80	35	13,1%	18,5%	52,3%	71,7%	63,6%
3034	25	40	80	40	13,3%	18,3%	53,1%	73,0%	65,2%
3035	25	40	80	45	13,5%	18,1%	54,0%	74,4%	66,7%

3036	25	40	80	50	13,7%	17,9%	54,8%	75,7%	68,2%
3037	25	40	80	55	13,9%	17,7%	55,6%	76,9%	69,7%
3038	25	40	80	60	14,1%	17,5%	56,5%	78,1%	71,1%
3039	25	40	80	65	14,4%	17,3%	57,3%	79,3%	72,5%
3040	25	40	80	70	14,6%	17,1%	58,1%	80,4%	73,8%
3041	25	40	80	75	14,8%	16,9%	59,0%	81,4%	75,1%
3042	25	40	80	80	15,0%	16,8%	59,8%	82,5%	76,4%
3043	25	45	20	20	75,2%	80,3%	81,0%	79,9%	68,0%
3044	25	45	20	25	75,5%	80,1%	81,5%	81,0%	69,4%
3045	25	45	20	30	75,8%	79,9%	82,0%	82,0%	70,9%
3046	25	45	20	35	76,1%	79,7%	82,5%	83,0%	72,3%
3047	25	45	20	40	76,5%	79,5%	83,0%	83,9%	73,6%
3048	25	45	20	45	76,8%	79,2%	83,4%	84,8%	74,9%
3049	25	45	20	50	77,1%	79,0%	83,9%	85,7%	76,2%
3050	25	45	20	55	77,4%	78,8%	84,4%	86,5%	77,4%
3051	25	45	20	60	77,7%	78,6%	84,8%	87,3%	78,6%
3052	25	45	20	65	78,0%	78,4%	85,2%	88,1%	79,7%
3053	25	45	20	70	78,3%	78,1%	85,7%	88,8%	80,8%
3054	25	45	20	75	78,6%	77,9%	86,1%	89,4%	81,8%
3055	25	45	20	80	78,9%	77,7%	86,5%	90,1%	82,8%
3056	25	45	25	20	70,3%	76,4%	79,0%	78,9%	67,1%
3057	25	45	25	25	70,6%	76,1%	79,5%	80,0%	68,5%
3058	25	45	25	30	71,0%	75,9%	80,1%	81,1%	70,0%
3059	25	45	25	35	71,4%	75,7%	80,6%	82,1%	71,4%
3060	25	45	25	40	71,7%	75,4%	81,1%	83,1%	72,8%
3061	25	45	25	45	72,1%	75,2%	81,7%	84,0%	74,1%
3062	25	45	25	50	72,4%	74,9%	82,2%	85,0%	75,4%
3063	25	45	25	55	72,8%	74,7%	82,7%	85,8%	76,7%
3064	25	45	25	60	73,1%	74,4%	83,1%	86,6%	77,9%
3065	25	45	25	65	73,5%	74,2%	83,6%	87,4%	79,0%
3066	25	45	25	70	73,8%	73,9%	84,1%	88,1%	80,1%
3067	25	45	25	75	74,2%	73,7%	84,5%	88,9%	81,2%
3068	25	45	25	80	74,5%	73,4%	85,0%	89,5%	82,2%

3069	25	45	30	20	64,9%	72,0%	76,8%	77,8%	66,1%
3070	25	45	30	25	65,3%	71,7%	77,4%	79,0%	67,7%
3071	25	45	30	30	65,7%	71,4%	78,0%	80,1%	69,1%
3072	25	45	30	35	66,1%	71,2%	78,6%	81,2%	70,6%
3073	25	45	30	40	66,5%	70,9%	79,2%	82,2%	72,0%
3074	25	45	30	45	66,9%	70,6%	79,7%	83,2%	73,3%
3075	25	45	30	50	67,3%	70,4%	80,3%	84,2%	74,7%
3076	25	45	30	55	67,6%	70,1%	80,8%	85,1%	75,9%
3077	25	45	30	60	68,0%	69,8%	81,3%	85,9%	77,2%
3078	25	45	30	65	68,4%	69,5%	81,8%	86,7%	78,3%
3079	25	45	30	70	68,8%	69,3%	82,3%	87,5%	79,5%
3080	25	45	30	75	69,2%	69,0%	82,8%	88,2%	80,6%
3081	25	45	30	80	69,5%	68,7%	83,3%	88,9%	81,6%
3082	25	45	35	20	59,1%	67,1%	74,6%	76,8%	65,2%
3083	25	45	35	25	59,5%	66,8%	75,2%	78,0%	66,7%
3084	25	45	35	30	59,9%	66,5%	75,8%	79,1%	68,2%
3085	25	45	35	35	60,3%	66,2%	76,4%	80,2%	69,7%
3086	25	45	35	40	60,8%	65,9%	77,0%	81,3%	71,1%
3087	25	45	35	45	61,2%	65,6%	77,6%	82,3%	72,5%
3088	25	45	35	50	61,6%	65,3%	78,2%	83,3%	73,9%
3089	25	45	35	55	62,0%	65,0%	78,8%	84,3%	75,2%
3090	25	45	35	60	62,4%	64,7%	79,4%	85,2%	76,4%
3091	25	45	35	65	62,8%	64,4%	79,9%	86,0%	77,6%
3092	25	45	35	70	63,3%	64,1%	80,4%	86,8%	78,8%
3093	25	45	35	75	63,7%	63,8%	81,0%	87,6%	79,9%
3094	25	45	35	80	64,1%	63,5%	81,5%	88,3%	81,0%
3095	25	45	40	20	53,0%	61,8%	72,1%	75,7%	64,3%
3096	25	45	40	25	53,4%	61,5%	72,8%	76,9%	65,8%
3097	25	45	40	30	53,9%	61,2%	73,5%	78,1%	67,3%
3098	25	45	40	35	54,3%	60,9%	74,1%	79,3%	68,8%
3099	25	45	40	40	54,7%	60,6%	74,8%	80,4%	70,3%
3100	25	45	40	45	55,2%	60,2%	75,4%	81,4%	71,7%
3101	25	45	40	50	55,6%	59,9%	76,0%	82,5%	73,1%

3102	25	45	40	55	56,1%	59,6%	76,6%	83,4%	74,4%
3103	25	45	40	60	56,5%	59,3%	77,2%	84,4%	75,7%
3104	25	45	40	65	56,9%	59,0%	77,8%	85,3%	76,9%
3105	25	45	40	70	57,4%	58,7%	78,4%	86,1%	78,1%
3106	25	45	40	75	57,8%	58,3%	79,0%	86,9%	79,3%
3107	25	45	40	80	58,2%	58,0%	79,5%	87,7%	80,4%
3108	25	45	45	20	46,8%	56,2%	69,6%	74,5%	63,3%
3109	25	45	45	25	47,3%	55,9%	70,3%	75,8%	64,9%
3110	25	45	45	30	47,7%	55,6%	71,0%	77,0%	66,4%
3111	25	45	45	35	48,2%	55,2%	71,7%	78,2%	67,9%
3112	25	45	45	40	48,6%	54,9%	72,4%	79,4%	69,4%
3113	25	45	45	45	49,0%	54,6%	73,0%	80,5%	70,8%
3114	25	45	45	50	49,5%	54,3%	73,7%	81,6%	72,2%
3115	25	45	45	55	49,9%	54,0%	74,3%	82,6%	73,6%
3116	25	45	45	60	50,4%	53,6%	75,0%	83,5%	74,9%
3117	25	45	45	65	50,8%	53,3%	75,6%	84,5%	76,2%
3118	25	45	45	70	51,2%	53,0%	76,2%	85,4%	77,4%
3119	25	45	45	75	51,7%	52,6%	76,9%	86,2%	78,6%
3120	25	45	45	80	52,1%	52,3%	77,5%	87,0%	79,7%
3121	25	45	50	20	40,8%	50,5%	66,9%	73,3%	62,3%
3122	25	45	50	25	41,2%	50,1%	67,6%	74,7%	63,9%
3123	25	45	50	30	41,6%	49,8%	68,4%	76,0%	65,5%
3124	25	45	50	35	42,0%	49,5%	69,1%	77,2%	67,0%
3125	25	45	50	40	42,5%	49,2%	69,8%	78,4%	68,5%
3126	25	45	50	45	42,9%	48,8%	70,5%	79,5%	70,0%
3127	25	45	50	50	43,3%	48,5%	71,2%	80,6%	71,4%
3128	25	45	50	55	43,8%	48,2%	71,9%	81,7%	72,8%
3129	25	45	50	60	44,2%	47,9%	72,6%	82,7%	74,1%
3130	25	45	50	65	44,6%	47,5%	73,3%	83,7%	75,4%
3131	25	45	50	70	45,1%	47,2%	73,9%	84,6%	76,7%
3132	25	45	50	75	45,5%	46,9%	74,6%	85,5%	77,9%
3133	25	45	50	80	46,0%	46,6%	75,2%	86,3%	79,0%
3134	25	45	55	20	35,0%	44,7%	64,1%	72,1%	61,4%

3135	25	45	55	25	35,4%	44,4%	64,8%	73,5%	63,0%
3136	25	45	55	30	35,8%	44,1%	65,6%	74,8%	64,6%
3137	25	45	55	35	36,2%	43,8%	66,4%	76,1%	66,1%
3138	25	45	55	40	36,6%	43,4%	67,1%	77,3%	67,6%
3139	25	45	55	45	37,0%	43,1%	67,9%	78,5%	69,1%
3140	25	45	55	50	37,4%	42,8%	68,6%	79,7%	70,6%
3141	25	45	55	55	37,8%	42,5%	69,3%	80,8%	72,0%
3142	25	45	55	60	38,2%	42,2%	70,0%	81,8%	73,3%
3143	25	45	55	65	38,7%	41,8%	70,8%	82,8%	74,6%
3144	25	45	55	70	39,1%	41,5%	71,5%	83,8%	75,9%
3145	25	45	55	75	39,5%	41,2%	72,1%	84,7%	77,1%
3146	25	45	55	80	39,9%	40,9%	72,8%	85,6%	78,3%
3147	25	45	60	20	29,6%	39,1%	61,2%	70,9%	60,4%
3148	25	45	60	25	29,9%	38,8%	62,0%	72,3%	62,0%
3149	25	45	60	30	30,3%	38,5%	62,8%	73,7%	63,6%
3150	25	45	60	35	30,7%	38,2%	63,5%	75,0%	65,2%
3151	25	45	60	40	31,1%	37,9%	64,3%	76,2%	66,7%
3152	25	45	60	45	31,4%	37,6%	65,1%	77,5%	68,2%
3153	25	45	60	50	31,8%	37,3%	65,9%	78,7%	69,7%
3154	25	45	60	55	32,2%	36,9%	66,6%	79,8%	71,1%
3155	25	45	60	60	32,6%	36,6%	67,4%	80,9%	72,5%
3156	25	45	60	65	33,0%	36,3%	68,1%	81,9%	73,9%
3157	25	45	60	70	33,4%	36,0%	68,9%	82,9%	75,2%
3158	25	45	60	75	33,8%	35,7%	69,6%	83,9%	76,4%
3159	25	45	60	80	34,2%	35,4%	70,3%	84,8%	77,6%
3160	25	45	65	20	24,7%	33,8%	58,2%	69,6%	59,4%
3161	25	45	65	25	25,0%	33,5%	59,0%	71,1%	61,0%
3162	25	45	65	30	25,4%	33,2%	59,8%	72,5%	62,7%
3163	25	45	65	35	25,7%	32,9%	60,6%	73,8%	64,2%
3164	25	45	65	40	26,0%	32,6%	61,4%	75,1%	65,8%
3165	25	45	65	45	26,4%	32,3%	62,2%	76,4%	67,3%
3166	25	45	65	50	26,7%	32,0%	63,0%	77,6%	68,8%
3167	25	45	65	55	27,1%	31,7%	63,8%	78,8%	70,3%

3168	25	45	65	60	27,4%	31,5%	64,6%	79,9%	71,7%
3169	25	45	65	65	27,8%	31,2%	65,4%	81,0%	73,0%
3170	25	45	65	70	28,1%	30,9%	66,1%	82,0%	74,4%
3171	25	45	65	75	28,5%	30,6%	66,9%	83,0%	75,7%
3172	25	45	65	80	28,8%	30,3%	67,6%	84,0%	76,9%
3173	25	45	70	20	20,4%	28,8%	55,1%	68,3%	58,4%
3174	25	45	70	25	20,7%	28,5%	56,0%	69,8%	60,1%
3175	25	45	70	30	21,0%	28,3%	56,8%	71,2%	61,7%
3176	25	45	70	35	21,3%	28,0%	57,6%	72,6%	63,3%
3177	25	45	70	40	21,6%	27,7%	58,4%	74,0%	64,9%
3178	25	45	70	45	21,9%	27,5%	59,3%	75,3%	66,4%
3179	25	45	70	50	22,2%	27,2%	60,1%	76,5%	67,9%
3180	25	45	70	55	22,5%	27,0%	60,9%	77,7%	69,4%
3181	25	45	70	60	22,8%	26,7%	61,7%	78,9%	70,8%
3182	25	45	70	65	23,1%	26,5%	62,5%	80,0%	72,2%
3183	25	45	70	70	23,4%	26,2%	63,3%	81,1%	73,6%
3184	25	45	70	75	23,7%	25,9%	64,1%	82,2%	74,9%
3185	25	45	70	80	24,1%	25,7%	64,9%	83,1%	76,2%
3186	25	45	75	20	16,7%	24,3%	52,0%	67,0%	57,4%
3187	25	45	75	25	16,9%	24,1%	52,9%	68,5%	59,1%
3188	25	45	75	30	17,2%	23,8%	53,7%	70,0%	60,7%
3189	25	45	75	35	17,4%	23,6%	54,6%	71,4%	62,3%
3190	25	45	75	40	17,7%	23,4%	55,4%	72,8%	63,9%
3191	25	45	75	45	17,9%	23,1%	56,2%	74,1%	65,5%
3192	25	45	75	50	18,2%	22,9%	57,1%	75,4%	67,0%
3193	25	45	75	55	18,5%	22,7%	57,9%	76,7%	68,5%
3194	25	45	75	60	18,7%	22,4%	58,7%	77,9%	70,0%
3195	25	45	75	65	19,0%	22,2%	59,5%	79,1%	71,4%
3196	25	45	75	70	19,3%	22,0%	60,4%	80,2%	72,8%
3197	25	45	75	75	19,6%	21,8%	61,2%	81,2%	74,1%
3198	25	45	75	80	19,8%	21,5%	62,0%	82,3%	75,4%
3199	25	45	80	20	13,5%	20,3%	48,9%	65,6%	56,4%
3200	25	45	80	25	13,7%	20,1%	49,8%	67,2%	58,1%

3201	25	45	80	30	13,9%	19,9%	50,6%	68,7%	59,7%
3202	25	45	80	35	14,2%	19,7%	51,5%	70,1%	61,4%
3203	25	45	80	40	14,4%	19,5%	52,3%	71,6%	63,0%
3204	25	45	80	45	14,6%	19,3%	53,2%	72,9%	64,5%
3205	25	45	80	50	14,8%	19,1%	54,0%	74,3%	66,1%
3206	25	45	80	55	15,0%	18,9%	54,8%	75,6%	67,6%
3207	25	45	80	60	15,3%	18,7%	55,7%	76,8%	69,1%
3208	25	45	80	65	15,5%	18,5%	56,5%	78,0%	70,5%
3209	25	45	80	70	15,7%	18,3%	57,4%	79,2%	71,9%
3210	25	45	80	75	16,0%	18,1%	58,2%	80,3%	73,3%
3211	25	45	80	80	16,2%	17,9%	59,0%	81,4%	74,6%
3212	25	50	20	20	76,8%	81,5%	80,5%	78,6%	65,8%
3213	25	50	20	25	77,1%	81,3%	81,0%	79,8%	67,4%
3214	25	50	20	30	77,4%	81,1%	81,5%	80,9%	68,9%
3215	25	50	20	35	77,7%	80,9%	82,0%	81,9%	70,3%
3216	25	50	20	40	78,0%	80,7%	82,5%	82,9%	71,7%
3217	25	50	20	45	78,3%	80,5%	83,0%	83,9%	73,1%
3218	25	50	20	50	78,6%	80,3%	83,5%	84,8%	74,4%
3219	25	50	20	55	78,9%	80,1%	83,9%	85,7%	75,7%
3220	25	50	20	60	79,2%	79,9%	84,4%	86,5%	76,9%
3221	25	50	20	65	79,5%	79,7%	84,8%	87,3%	78,1%
3222	25	50	20	70	79,8%	79,5%	85,3%	88,0%	79,3%
3223	25	50	20	75	80,1%	79,2%	85,7%	88,7%	80,4%
3224	25	50	20	80	80,3%	79,0%	86,1%	89,4%	81,4%
3225	25	50	25	20	72,1%	77,8%	78,4%	77,6%	64,9%
3226	25	50	25	25	72,5%	77,5%	79,0%	78,8%	66,5%
3227	25	50	25	30	72,8%	77,3%	79,6%	79,9%	68,0%
3228	25	50	25	35	73,2%	77,1%	80,1%	81,0%	69,4%
3229	25	50	25	40	73,5%	76,9%	80,6%	82,0%	70,9%
3230	25	50	25	45	73,8%	76,6%	81,2%	83,0%	72,3%
3231	25	50	25	50	74,2%	76,4%	81,7%	84,0%	73,6%
3232	25	50	25	55	74,5%	76,1%	82,2%	84,9%	74,9%
3233	25	50	25	60	74,8%	75,9%	82,7%	85,7%	76,2%

3234	25	50	25	65	75,2%	75,7%	83,2%	86,6%	77,4%
3235	25	50	25	70	75,5%	75,4%	83,6%	87,4%	78,6%
3236	25	50	25	75	75,8%	75,2%	84,1%	88,1%	79,7%
3237	25	50	25	80	76,2%	74,9%	84,5%	88,8%	80,8%
3238	25	50	30	20	66,9%	73,5%	76,3%	76,5%	64,0%
3239	25	50	30	25	67,3%	73,3%	76,9%	77,7%	65,5%
3240	25	50	30	30	67,7%	73,0%	77,5%	78,9%	67,1%
3241	25	50	30	35	68,0%	72,8%	78,1%	80,0%	68,6%
3242	25	50	30	40	68,4%	72,5%	78,6%	81,1%	70,0%
3243	25	50	30	45	68,8%	72,2%	79,2%	82,2%	71,4%
3244	25	50	30	50	69,2%	72,0%	79,8%	83,1%	72,8%
3245	25	50	30	55	69,5%	71,7%	80,3%	84,1%	74,1%
3246	25	50	30	60	69,9%	71,4%	80,8%	85,0%	75,4%
3247	25	50	30	65	70,3%	71,2%	81,4%	85,8%	76,7%
3248	25	50	30	70	70,7%	70,9%	81,9%	86,7%	77,9%
3249	25	50	30	75	71,0%	70,6%	82,4%	87,4%	79,0%
3250	25	50	30	80	71,4%	70,4%	82,8%	88,2%	80,2%
3251	25	50	35	20	61,2%	68,8%	73,9%	75,4%	63,0%
3252	25	50	35	25	61,6%	68,5%	74,6%	76,7%	64,6%
3253	25	50	35	30	62,0%	68,2%	75,2%	77,9%	66,1%
3254	25	50	35	35	62,4%	67,9%	75,9%	79,0%	67,7%
3255	25	50	35	40	62,9%	67,7%	76,5%	80,2%	69,1%
3256	25	50	35	45	63,3%	67,4%	77,1%	81,2%	70,6%
3257	25	50	35	50	63,7%	67,1%	77,7%	82,3%	72,0%
3258	25	50	35	55	64,1%	66,8%	78,3%	83,3%	73,3%
3259	25	50	35	60	64,5%	66,5%	78,8%	84,2%	74,7%
3260	25	50	35	65	64,9%	66,2%	79,4%	85,1%	75,9%
3261	25	50	35	70	65,3%	65,9%	79,9%	85,9%	77,2%
3262	25	50	35	75	65,7%	65,6%	80,5%	86,8%	78,3%
3263	25	50	35	80	66,1%	65,3%	81,0%	87,5%	79,5%
3264	25	50	40	20	55,2%	63,6%	71,5%	74,3%	62,0%
3265	25	50	40	25	55,6%	63,3%	72,2%	75,6%	63,6%
3266	25	50	40	30	56,1%	63,0%	72,8%	76,8%	65,2%

3267	25	50	40	35	56,5%	62,7%	73,5%	78,0%	66,7%
3268	25	50	40	40	56,9%	62,4%	74,2%	79,2%	68,2%
3269	25	50	40	45	57,4%	62,1%	74,8%	80,3%	69,7%
3270	25	50	40	50	57,8%	61,8%	75,4%	81,4%	71,1%
3271	25	50	40	55	58,2%	61,5%	76,1%	82,4%	72,5%
3272	25	50	40	60	58,7%	61,2%	76,7%	83,4%	73,9%
3273	25	50	40	65	59,1%	60,9%	77,3%	84,3%	75,2%
3274	25	50	40	70	59,5%	60,6%	77,9%	85,2%	76,4%
3275	25	50	40	75	59,9%	60,2%	78,5%	86,0%	77,6%
3276	25	50	40	80	60,4%	59,9%	79,0%	86,8%	78,8%
3277	25	50	45	20	49,0%	58,1%	68,9%	73,1%	61,1%
3278	25	50	45	25	49,5%	57,8%	69,6%	74,4%	62,7%
3279	25	50	45	30	49,9%	57,5%	70,3%	75,7%	64,3%
3280	25	50	45	35	50,4%	57,2%	71,0%	77,0%	65,8%
3281	25	50	45	40	50,8%	56,9%	71,7%	78,2%	67,3%
3282	25	50	45	45	51,2%	56,5%	72,4%	79,3%	68,8%
3283	25	50	45	50	51,7%	56,2%	73,1%	80,4%	70,3%
3284	25	50	45	55	52,1%	55,9%	73,7%	81,5%	71,7%
3285	25	50	45	60	52,6%	55,6%	74,4%	82,5%	73,1%
3286	25	50	45	65	53,0%	55,3%	75,0%	83,5%	74,4%
3287	25	50	45	70	53,4%	54,9%	75,7%	84,4%	75,7%
3288	25	50	45	75	53,9%	54,6%	76,3%	85,3%	76,9%
3289	25	50	45	80	54,3%	54,3%	76,9%	86,1%	78,1%
3290	25	50	50	20	42,9%	52,4%	66,2%	71,9%	60,1%
3291	25	50	50	25	43,4%	52,1%	66,9%	73,2%	61,7%
3292	25	50	50	30	43,8%	51,8%	67,7%	74,6%	63,3%
3293	25	50	50	35	44,2%	51,5%	68,4%	75,9%	64,9%
3294	25	50	50	40	44,7%	51,1%	69,1%	77,1%	66,4%
3295	25	50	50	45	45,1%	50,8%	69,9%	78,3%	67,9%
3296	25	50	50	50	45,5%	50,5%	70,6%	79,4%	69,4%
3297	25	50	50	55	46,0%	50,2%	71,3%	80,5%	70,9%
3298	25	50	50	60	46,4%	49,8%	72,0%	81,6%	72,2%
3299	25	50	50	65	46,8%	49,5%	72,6%	82,6%	73,6%

3300	25	50	50	70	47,3%	49,2%	73,3%	83,6%	74,9%
3301	25	50	50	75	47,7%	48,8%	74,0%	84,5%	76,2%
3302	25	50	50	80	48,2%	48,5%	74,6%	85,4%	77,4%
3303	25	50	55	20	37,0%	46,7%	63,3%	70,6%	59,1%
3304	25	50	55	25	37,4%	46,4%	64,1%	72,0%	60,7%
3305	25	50	55	30	37,8%	46,0%	64,9%	73,4%	62,4%
3306	25	50	55	35	38,2%	45,7%	65,7%	74,7%	63,9%
3307	25	50	55	40	38,7%	45,4%	66,4%	76,0%	65,5%
3308	25	50	55	45	39,1%	45,1%	67,2%	77,2%	67,0%
3309	25	50	55	50	39,5%	44,7%	67,9%	78,4%	68,5%
3310	25	50	55	55	39,9%	44,4%	68,7%	79,6%	70,0%
3311	25	50	55	60	40,3%	44,1%	69,4%	80,7%	71,4%
3312	25	50	55	65	40,8%	43,8%	70,1%	81,7%	72,8%
3313	25	50	55	70	41,2%	43,4%	70,8%	82,7%	74,1%
3314	25	50	55	75	41,6%	43,1%	71,5%	83,7%	75,4%
3315	25	50	55	80	42,1%	42,8%	72,2%	84,6%	76,7%
3316	25	50	60	20	31,5%	41,0%	60,4%	69,3%	58,1%
3317	25	50	60	25	31,8%	40,7%	61,2%	70,8%	59,7%
3318	25	50	60	30	32,2%	40,4%	62,0%	72,2%	61,4%
3319	25	50	60	35	32,6%	40,1%	62,8%	73,6%	63,0%
3320	25	50	60	40	33,0%	39,7%	63,6%	74,9%	64,6%
3321	25	50	60	45	33,4%	39,4%	64,4%	76,2%	66,1%
3322	25	50	60	50	33,8%	39,1%	65,2%	77,4%	67,6%
3323	25	50	60	55	34,2%	38,8%	65,9%	78,6%	69,1%
3324	25	50	60	60	34,6%	38,5%	66,7%	79,7%	70,6%
3325	25	50	60	65	35,0%	38,2%	67,4%	80,8%	72,0%
3326	25	50	60	70	35,4%	37,9%	68,2%	81,8%	73,3%
3327	25	50	60	75	35,8%	37,6%	68,9%	82,8%	74,6%
3328	25	50	60	80	36,2%	37,3%	69,6%	83,8%	75,9%
3329	25	50	65	20	26,4%	35,5%	57,4%	68,0%	57,1%
3330	25	50	65	25	26,7%	35,2%	58,2%	69,5%	58,7%
3331	25	50	65	30	27,1%	35,0%	59,0%	71,0%	60,4%
3332	25	50	65	35	27,4%	34,7%	59,9%	72,4%	62,0%

3333	25	50	65	40	27,8%	34,4%	60,7%	73,7%	63,6%
3334	25	50	65	45	28,1%	34,1%	61,5%	75,0%	65,2%
3335	25	50	65	50	28,5%	33,8%	62,3%	76,3%	66,7%
3336	25	50	65	55	28,9%	33,5%	63,1%	77,5%	68,2%
3337	25	50	65	60	29,2%	33,2%	63,9%	78,7%	69,7%
3338	25	50	65	65	29,6%	32,9%	64,6%	79,8%	71,1%
3339	25	50	65	70	30,0%	32,6%	65,4%	80,9%	72,5%
3340	25	50	65	75	30,3%	32,3%	66,2%	82,0%	73,9%
3341	25	50	65	80	30,7%	32,0%	66,9%	83,0%	75,2%
3342	25	50	70	20	21,9%	30,5%	54,3%	66,7%	56,1%
3343	25	50	70	25	22,2%	30,2%	55,2%	68,2%	57,7%
3344	25	50	70	30	22,5%	29,9%	56,0%	69,7%	59,4%
3345	25	50	70	35	22,8%	29,6%	56,8%	71,1%	61,0%
3346	25	50	70	40	23,1%	29,4%	57,7%	72,5%	62,7%
3347	25	50	70	45	23,4%	29,1%	58,5%	73,9%	64,3%
3348	25	50	70	50	23,7%	28,8%	59,3%	75,2%	65,8%
3349	25	50	70	55	24,1%	28,5%	60,1%	76,4%	67,3%
3350	25	50	70	60	24,4%	28,3%	60,9%	77,7%	68,8%
3351	25	50	70	65	24,7%	28,0%	61,8%	78,8%	70,3%
3352	25	50	70	70	25,0%	27,8%	62,6%	80,0%	71,7%
3353	25	50	70	75	25,4%	27,5%	63,3%	81,0%	73,1%
3354	25	50	70	80	25,7%	27,2%	64,1%	82,1%	74,4%
3355	25	50	75	20	18,0%	25,8%	51,2%	65,3%	55,0%
3356	25	50	75	25	18,2%	25,5%	52,1%	66,9%	56,7%
3357	25	50	75	30	18,5%	25,3%	52,9%	68,4%	58,4%
3358	25	50	75	35	18,7%	25,0%	53,8%	69,8%	60,1%
3359	25	50	75	40	19,0%	24,8%	54,6%	71,3%	61,7%
3360	25	50	75	45	19,3%	24,6%	55,5%	72,7%	63,3%
3361	25	50	75	50	19,6%	24,3%	56,3%	74,0%	64,9%
3362	25	50	75	55	19,8%	24,1%	57,1%	75,3%	66,4%
3363	25	50	75	60	20,1%	23,8%	58,0%	76,6%	67,9%
3364	25	50	75	65	20,4%	23,6%	58,8%	77,8%	69,4%
3365	25	50	75	70	20,7%	23,4%	59,6%	79,0%	70,8%

3366	25	50	75	75	21,0%	23,1%	60,4%	80,1%	72,2%
3367	25	50	75	80	21,3%	22,9%	61,2%	81,2%	73,6%
3368	25	50	80	20	14,6%	21,6%	48,1%	63,9%	54,0%
3369	25	50	80	25	14,8%	21,4%	49,0%	65,5%	55,7%
3370	25	50	80	30	15,0%	21,2%	49,8%	67,0%	57,4%
3371	25	50	80	35	15,3%	21,0%	50,7%	68,6%	59,1%
3372	25	50	80	40	15,5%	20,7%	51,5%	70,0%	60,7%
3373	25	50	80	45	15,7%	20,5%	52,4%	71,4%	62,3%
3374	25	50	80	50	16,0%	20,3%	53,2%	72,8%	63,9%
3375	25	50	80	55	16,2%	20,1%	54,1%	74,2%	65,5%
3376	25	50	80	60	16,4%	19,9%	54,9%	75,5%	67,0%
3377	25	50	80	65	16,7%	19,7%	55,7%	76,7%	68,5%
3378	25	50	80	70	16,9%	19,5%	56,6%	77,9%	70,0%
3379	25	50	80	75	17,2%	19,3%	57,4%	79,1%	71,4%
3380	25	50	80	80	17,4%	19,1%	58,2%	80,2%	72,8%
3381	25	55	20	20	78,3%	82,7%	80,0%	77,4%	63,7%
3382	25	55	20	25	78,6%	82,5%	80,5%	78,6%	65,2%
3383	25	55	20	30	78,9%	82,3%	81,0%	79,7%	66,8%
3384	25	55	20	35	79,2%	82,1%	81,5%	80,8%	68,3%
3385	25	55	20	40	79,5%	81,9%	82,1%	81,8%	69,7%
3386	25	55	20	45	79,8%	81,7%	82,5%	82,8%	71,2%
3387	25	55	20	50	80,1%	81,5%	83,0%	83,8%	72,5%
3388	25	55	20	55	80,4%	81,3%	83,5%	84,7%	73,9%
3389	25	55	20	60	80,6%	81,1%	84,0%	85,6%	75,2%
3390	25	55	20	65	80,9%	80,9%	84,4%	86,4%	76,4%
3391	25	55	20	70	81,2%	80,7%	84,9%	87,2%	77,7%
3392	25	55	20	75	81,4%	80,5%	85,3%	88,0%	78,8%
3393	25	55	20	80	81,7%	80,3%	85,7%	88,7%	79,9%
3394	25	55	25	20	73,8%	79,1%	77,9%	76,3%	62,7%
3395	25	55	25	25	74,2%	78,9%	78,5%	77,5%	64,3%
3396	25	55	25	30	74,5%	78,7%	79,0%	78,7%	65,8%
3397	25	55	25	35	74,9%	78,4%	79,6%	79,8%	67,4%
3398	25	55	25	40	75,2%	78,2%	80,1%	80,9%	68,9%

3399	25	55	25	45	75,5%	78,0%	80,7%	82,0%	70,3%
3400	25	55	25	50	75,8%	77,8%	81,2%	83,0%	71,7%
3401	25	55	25	55	76,2%	77,6%	81,7%	83,9%	73,1%
3402	25	55	25	60	76,5%	77,3%	82,2%	84,8%	74,4%
3403	25	55	25	65	76,8%	77,1%	82,7%	85,7%	75,7%
3404	25	55	25	70	77,1%	76,9%	83,2%	86,5%	76,9%
3405	25	55	25	75	77,4%	76,6%	83,7%	87,3%	78,1%
3406	25	55	25	80	77,7%	76,4%	84,1%	88,0%	79,3%
3407	25	55	30	20	68,8%	75,0%	75,7%	75,2%	61,7%
3408	25	55	30	25	69,2%	74,8%	76,3%	76,4%	63,3%
3409	25	55	30	30	69,6%	74,5%	76,9%	77,7%	64,9%
3410	25	55	30	35	69,9%	74,3%	77,5%	78,8%	66,5%
3411	25	55	30	40	70,3%	74,0%	78,1%	80,0%	68,0%
3412	25	55	30	45	70,7%	73,8%	78,7%	81,0%	69,4%
3413	25	55	30	50	71,0%	73,5%	79,2%	82,1%	70,9%
3414	25	55	30	55	71,4%	73,3%	79,8%	83,1%	72,3%
3415	25	55	30	60	71,8%	73,0%	80,3%	84,0%	73,6%
3416	25	55	30	65	72,1%	72,8%	80,9%	84,9%	74,9%
3417	25	55	30	70	72,5%	72,5%	81,4%	85,8%	76,2%
3418	25	55	30	75	72,8%	72,2%	81,9%	86,6%	77,4%
3419	25	55	30	80	73,2%	72,0%	82,4%	87,4%	78,6%
3420	25	55	35	20	63,3%	70,5%	73,3%	74,0%	60,8%
3421	25	55	35	25	63,7%	70,2%	74,0%	75,3%	62,4%
3422	25	55	35	30	64,1%	69,9%	74,6%	76,6%	64,0%
3423	25	55	35	35	64,5%	69,6%	75,3%	77,8%	65,5%
3424	25	55	35	40	64,9%	69,4%	75,9%	79,0%	67,1%
3425	25	55	35	45	65,3%	69,1%	76,5%	80,1%	68,6%
3426	25	55	35	50	65,7%	68,8%	77,1%	81,2%	70,0%
3427	25	55	35	55	66,1%	68,5%	77,7%	82,2%	71,4%
3428	25	55	35	60	66,5%	68,2%	78,3%	83,2%	72,8%
3429	25	55	35	65	66,9%	68,0%	78,9%	84,1%	74,1%
3430	25	55	35	70	67,3%	67,7%	79,4%	85,0%	75,4%
3431	25	55	35	75	67,7%	67,4%	80,0%	85,9%	76,7%

3432	25	55	35	80	68,0%	67,1%	80,5%	86,7%	77,9%
3433	25	55	40	20	57,4%	65,4%	70,8%	72,8%	59,8%
3434	25	55	40	25	57,8%	65,1%	71,5%	74,2%	61,4%
3435	25	55	40	30	58,2%	64,8%	72,2%	75,5%	63,0%
3436	25	55	40	35	58,7%	64,5%	72,9%	76,7%	64,6%
3437	25	55	40	40	59,1%	64,2%	73,6%	77,9%	66,1%
3438	25	55	40	45	59,5%	63,9%	74,2%	79,1%	67,7%
3439	25	55	40	50	59,9%	63,6%	74,9%	80,2%	69,1%
3440	25	55	40	55	60,4%	63,3%	75,5%	81,3%	70,6%
3441	25	55	40	60	60,8%	63,0%	76,1%	82,3%	72,0%
3442	25	55	40	65	61,2%	62,7%	76,7%	83,3%	73,3%
3443	25	55	40	70	61,6%	62,4%	77,3%	84,2%	74,7%
3444	25	55	40	75	62,0%	62,1%	77,9%	85,1%	75,9%
3445	25	55	40	80	62,5%	61,8%	78,5%	86,0%	77,2%
3446	25	55	45	20	51,3%	60,0%	68,2%	71,6%	58,8%
3447	25	55	45	25	51,7%	59,7%	68,9%	73,0%	60,4%
3448	25	55	45	30	52,1%	59,4%	69,7%	74,3%	62,0%
3449	25	55	45	35	52,6%	59,1%	70,4%	75,6%	63,6%
3450	25	55	45	40	53,0%	58,8%	71,1%	76,9%	65,2%
3451	25	55	45	45	53,5%	58,5%	71,8%	78,1%	66,8%
3452	25	55	45	50	53,9%	58,2%	72,4%	79,2%	68,3%
3453	25	55	45	55	54,3%	57,8%	73,1%	80,3%	69,7%
3454	25	55	45	60	54,8%	57,5%	73,8%	81,4%	71,1%
3455	25	55	45	65	55,2%	57,2%	74,4%	82,4%	72,5%
3456	25	55	45	70	55,6%	56,9%	75,1%	83,4%	73,9%
3457	25	55	45	75	56,1%	56,6%	75,7%	84,3%	75,2%
3458	25	55	45	80	56,5%	56,2%	76,3%	85,2%	76,4%
3459	25	55	50	20	45,1%	54,4%	65,4%	70,3%	57,8%
3460	25	55	50	25	45,5%	54,1%	66,2%	71,8%	59,4%
3461	25	55	50	30	46,0%	53,8%	67,0%	73,1%	61,1%
3462	25	55	50	35	46,4%	53,4%	67,7%	74,5%	62,7%
3463	25	55	50	40	46,9%	53,1%	68,4%	75,8%	64,3%
3464	25	55	50	45	47,3%	52,8%	69,2%	77,0%	65,8%

3465	25	55	50	50	47,7%	52,4%	69,9%	78,2%	67,4%
3466	25	55	50	55	48,2%	52,1%	70,6%	79,4%	68,8%
3467	25	55	50	60	48,6%	51,8%	71,3%	80,5%	70,3%
3468	25	55	50	65	49,1%	51,5%	72,0%	81,5%	71,7%
3469	25	55	50	70	49,5%	51,1%	72,7%	82,5%	73,1%
3470	25	55	50	75	49,9%	50,8%	73,3%	83,5%	74,4%
3471	25	55	50	80	50,4%	50,5%	74,0%	84,4%	75,7%
3472	25	55	55	20	39,1%	48,6%	62,6%	69,1%	56,8%
3473	25	55	55	25	39,5%	48,3%	63,4%	70,5%	58,4%
3474	25	55	55	30	39,9%	48,0%	64,2%	71,9%	60,1%
3475	25	55	55	35	40,4%	47,7%	64,9%	73,3%	61,7%
3476	25	55	55	40	40,8%	47,3%	65,7%	74,6%	63,3%
3477	25	55	55	45	41,2%	47,0%	66,5%	75,9%	64,9%
3478	25	55	55	50	41,6%	46,7%	67,2%	77,2%	66,4%
3479	25	55	55	55	42,1%	46,4%	68,0%	78,3%	68,0%
3480	25	55	55	60	42,5%	46,0%	68,7%	79,5%	69,4%
3481	25	55	55	65	42,9%	45,7%	69,4%	80,6%	70,9%
3482	25	55	55	70	43,4%	45,4%	70,1%	81,6%	72,3%
3483	25	55	55	75	43,8%	45,1%	70,8%	82,7%	73,6%
3484	25	55	55	80	44,2%	44,7%	71,5%	83,6%	74,9%
3485	25	55	60	20	33,4%	42,9%	59,6%	67,7%	55,7%
3486	25	55	60	25	33,8%	42,6%	60,4%	69,2%	57,4%
3487	25	55	60	30	34,2%	42,3%	61,3%	70,7%	59,1%
3488	25	55	60	35	34,6%	42,0%	62,1%	72,1%	60,7%
3489	25	55	60	40	35,0%	41,6%	62,9%	73,5%	62,4%
3490	25	55	60	45	35,4%	41,3%	63,6%	74,8%	64,0%
3491	25	55	60	50	35,8%	41,0%	64,4%	76,1%	65,5%
3492	25	55	60	55	36,2%	40,7%	65,2%	77,3%	67,0%
3493	25	55	60	60	36,6%	40,4%	66,0%	78,5%	68,5%
3494	25	55	60	65	37,0%	40,1%	66,7%	79,6%	70,0%
3495	25	55	60	70	37,4%	39,7%	67,5%	80,7%	71,4%
3496	25	55	60	75	37,8%	39,4%	68,2%	81,8%	72,8%
3497	25	55	60	80	38,3%	39,1%	68,9%	82,8%	74,1%

3498	25	55	65	20	28,1%	37,4%	56,6%	66,4%	54,7%
3499	25	55	65	25	28,5%	37,1%	57,4%	67,9%	56,4%
3500	25	55	65	30	28,9%	36,8%	58,3%	69,4%	58,1%
3501	25	55	65	35	29,2%	36,5%	59,1%	70,8%	59,8%
3502	25	55	65	40	29,6%	36,2%	59,9%	72,3%	61,4%
3503	25	55	65	45	30,0%	35,9%	60,7%	73,6%	63,0%
3504	25	55	65	50	30,3%	35,6%	61,5%	74,9%	64,6%
3505	25	55	65	55	30,7%	35,3%	62,3%	76,2%	66,1%
3506	25	55	65	60	31,1%	35,0%	63,1%	77,4%	67,6%
3507	25	55	65	65	31,5%	34,7%	63,9%	78,6%	69,1%
3508	25	55	65	70	31,8%	34,4%	64,7%	79,8%	70,6%
3509	25	55	65	75	32,2%	34,1%	65,5%	80,8%	72,0%
3510	25	55	65	80	32,6%	33,8%	66,2%	81,9%	73,3%
3511	25	55	70	20	23,4%	32,1%	53,5%	65,0%	53,7%
3512	25	55	70	25	23,8%	31,9%	54,4%	66,6%	55,4%
3513	25	55	70	30	24,1%	31,6%	55,2%	68,1%	57,1%
3514	25	55	70	35	24,4%	31,3%	56,1%	69,6%	58,8%
3515	25	55	70	40	24,7%	31,0%	56,9%	71,0%	60,4%
3516	25	55	70	45	25,1%	30,7%	57,7%	72,4%	62,0%
3517	25	55	70	50	25,4%	30,5%	58,6%	73,8%	63,6%
3518	25	55	70	55	25,7%	30,2%	59,4%	75,1%	65,2%
3519	25	55	70	60	26,1%	29,9%	60,2%	76,3%	66,7%
3520	25	55	70	65	26,4%	29,6%	61,0%	77,6%	68,2%
3521	25	55	70	70	26,7%	29,4%	61,8%	78,7%	69,7%
3522	25	55	70	75	27,1%	29,1%	62,6%	79,9%	71,1%
3523	25	55	70	80	27,4%	28,8%	63,4%	81,0%	72,5%
3524	25	55	75	20	19,3%	27,3%	50,4%	63,6%	52,7%
3525	25	55	75	25	19,6%	27,1%	51,3%	65,2%	54,4%
3526	25	55	75	30	19,9%	26,8%	52,1%	66,8%	56,1%
3527	25	55	75	35	20,1%	26,6%	53,0%	68,3%	57,8%
3528	25	55	75	40	20,4%	26,3%	53,8%	69,7%	59,4%
3529	25	55	75	45	20,7%	26,0%	54,7%	71,2%	61,1%
3530	25	55	75	50	21,0%	25,8%	55,5%	72,6%	62,7%

3531	25	55	75	55	21,3%	25,5%	56,3%	73,9%	64,3%
3532	25	55	75	60	21,6%	25,3%	57,2%	75,2%	65,8%
3533	25	55	75	65	21,9%	25,1%	58,0%	76,5%	67,3%
3534	25	55	75	70	22,2%	24,8%	58,8%	77,7%	68,8%
3535	25	55	75	75	22,5%	24,6%	59,7%	78,9%	70,3%
3536	25	55	75	80	22,8%	24,3%	60,5%	80,0%	71,7%
3537	25	55	80	20	15,7%	23,0%	47,3%	62,2%	51,6%
3538	25	55	80	25	16,0%	22,8%	48,2%	63,8%	53,4%
3539	25	55	80	30	16,2%	22,5%	49,0%	65,4%	55,1%
3540	25	55	80	35	16,5%	22,3%	49,9%	66,9%	56,7%
3541	25	55	80	40	16,7%	22,1%	50,7%	68,4%	58,4%
3542	25	55	80	45	16,9%	21,8%	51,6%	69,9%	60,1%
3543	25	55	80	50	17,2%	21,6%	52,4%	71,3%	61,7%
3544	25	55	80	55	17,4%	21,4%	53,3%	72,7%	63,3%
3545	25	55	80	60	17,7%	21,2%	54,1%	74,1%	64,9%
3546	25	55	80	65	18,0%	21,0%	55,0%	75,4%	66,4%
3547	25	55	80	70	18,2%	20,8%	55,8%	76,6%	67,9%
3548	25	55	80	75	18,5%	20,5%	56,6%	77,8%	69,4%
3549	25	55	80	80	18,8%	20,3%	57,5%	79,0%	70,8%
3550	25	60	20	20	79,8%	83,8%	79,4%	76,1%	61,4%
3551	25	60	20	25	80,1%	83,6%	80,0%	77,3%	63,0%
3552	25	60	20	30	80,4%	83,4%	80,5%	78,5%	64,6%
3553	25	60	20	35	80,6%	83,2%	81,1%	79,6%	66,2%
3554	25	60	20	40	80,9%	83,0%	81,6%	80,7%	67,7%
3555	25	60	20	45	81,2%	82,9%	82,1%	81,8%	69,2%
3556	25	60	20	50	81,5%	82,7%	82,6%	82,8%	70,6%
3557	25	60	20	55	81,7%	82,5%	83,1%	83,7%	72,0%
3558	25	60	20	60	82,0%	82,3%	83,5%	84,7%	73,4%
3559	25	60	20	65	82,2%	82,1%	84,0%	85,5%	74,7%
3560	25	60	20	70	82,5%	81,9%	84,4%	86,4%	76,0%
3561	25	60	20	75	82,7%	81,7%	84,9%	87,2%	77,2%
3562	25	60	20	80	83,0%	81,5%	85,3%	87,9%	78,4%
3563	25	60	25	20	75,5%	80,4%	77,3%	74,9%	60,4%

3564	25	60	25	25	75,9%	80,2%	77,9%	76,2%	62,1%
3565	25	60	25	30	76,2%	80,0%	78,5%	77,4%	63,7%
3566	25	60	25	35	76,5%	79,8%	79,1%	78,6%	65,2%
3567	25	60	25	40	76,8%	79,5%	79,6%	79,7%	66,8%
3568	25	60	25	45	77,1%	79,3%	80,2%	80,8%	68,3%
3569	25	60	25	50	77,4%	79,1%	80,7%	81,9%	69,7%
3570	25	60	25	55	77,7%	78,9%	81,2%	82,9%	71,2%
3571	25	60	25	60	78,0%	78,7%	81,8%	83,8%	72,6%
3572	25	60	25	65	78,3%	78,5%	82,3%	84,8%	73,9%
3573	25	60	25	70	78,6%	78,2%	82,7%	85,6%	75,2%
3574	25	60	25	75	78,9%	78,0%	83,2%	86,5%	76,4%
3575	25	60	25	80	79,2%	77,8%	83,7%	87,2%	77,7%
3576	25	60	30	20	70,7%	76,5%	75,1%	73,8%	59,5%
3577	25	60	30	25	71,0%	76,2%	75,7%	75,1%	61,1%
3578	25	60	30	30	71,4%	76,0%	76,3%	76,3%	62,7%
3579	25	60	30	35	71,8%	75,8%	77,0%	77,6%	64,3%
3580	25	60	30	40	72,1%	75,5%	77,5%	78,7%	65,9%
3581	25	60	30	45	72,5%	75,3%	78,1%	79,9%	67,4%
3582	25	60	30	50	72,8%	75,0%	78,7%	81,0%	68,9%
3583	25	60	30	55	73,2%	74,8%	79,3%	82,0%	70,3%
3584	25	60	30	60	73,5%	74,5%	79,8%	83,0%	71,7%
3585	25	60	30	65	73,9%	74,3%	80,4%	83,9%	73,1%
3586	25	60	30	70	74,2%	74,0%	80,9%	84,9%	74,4%
3587	25	60	30	75	74,5%	73,8%	81,4%	85,7%	75,7%
3588	25	60	30	80	74,9%	73,5%	81,9%	86,5%	76,9%
3589	25	60	35	20	65,3%	72,1%	72,7%	72,6%	58,5%
3590	25	60	35	25	65,7%	71,8%	73,4%	73,9%	60,1%
3591	25	60	35	30	66,1%	71,5%	74,0%	75,2%	61,7%
3592	25	60	35	35	66,5%	71,3%	74,7%	76,5%	63,3%
3593	25	60	35	40	66,9%	71,0%	75,3%	77,7%	64,9%
3594	25	60	35	45	67,3%	70,7%	75,9%	78,9%	66,5%
3595	25	60	35	50	67,7%	70,5%	76,6%	80,0%	68,0%
3596	25	60	35	55	68,1%	70,2%	77,2%	81,1%	69,4%

3597	25	60	35	60	68,4%	69,9%	77,8%	82,1%	70,9%
3598	25	60	35	65	68,8%	69,6%	78,3%	83,1%	72,3%
3599	25	60	35	70	69,2%	69,4%	78,9%	84,1%	73,6%
3600	25	60	35	75	69,6%	69,1%	79,5%	85,0%	74,9%
3601	25	60	35	80	69,9%	68,8%	80,0%	85,8%	76,2%
3602	25	60	40	20	59,5%	67,2%	70,2%	71,3%	57,5%
3603	25	60	40	25	60,0%	66,9%	70,9%	72,7%	59,1%
3604	25	60	40	30	60,4%	66,6%	71,6%	74,1%	60,8%
3605	25	60	40	35	60,8%	66,3%	72,3%	75,4%	62,4%
3606	25	60	40	40	61,2%	66,0%	72,9%	76,6%	64,0%
3607	25	60	40	45	61,6%	65,7%	73,6%	77,8%	65,5%
3608	25	60	40	50	62,1%	65,4%	74,3%	79,0%	67,1%
3609	25	60	40	55	62,5%	65,1%	74,9%	80,1%	68,6%
3610	25	60	40	60	62,9%	64,9%	75,5%	81,2%	70,0%
3611	25	60	40	65	63,3%	64,6%	76,2%	82,2%	71,4%
3612	25	60	40	70	63,7%	64,3%	76,8%	83,2%	72,8%
3613	25	60	40	75	64,1%	64,0%	77,4%	84,2%	74,1%
3614	25	60	40	80	64,5%	63,7%	78,0%	85,1%	75,4%
3615	25	60	45	20	53,5%	61,9%	67,5%	70,1%	56,4%
3616	25	60	45	25	53,9%	61,6%	68,2%	71,5%	58,1%
3617	25	60	45	30	54,4%	61,3%	69,0%	72,9%	59,8%
3618	25	60	45	35	54,8%	61,0%	69,7%	74,2%	61,4%
3619	25	60	45	40	55,2%	60,7%	70,4%	75,5%	63,0%
3620	25	60	45	45	55,7%	60,4%	71,1%	76,8%	64,6%
3621	25	60	45	50	56,1%	60,1%	71,8%	78,0%	66,2%
3622	25	60	45	55	56,5%	59,7%	72,5%	79,1%	67,7%
3623	25	60	45	60	57,0%	59,4%	73,2%	80,3%	69,1%
3624	25	60	45	65	57,4%	59,1%	73,8%	81,3%	70,6%
3625	25	60	45	70	57,8%	58,8%	74,5%	82,4%	72,0%
3626	25	60	45	75	58,3%	58,5%	75,1%	83,3%	73,4%
3627	25	60	45	80	58,7%	58,2%	75,7%	84,3%	74,7%
3628	25	60	50	20	47,3%	56,3%	64,7%	68,8%	55,4%
3629	25	60	50	25	47,8%	56,0%	65,5%	70,2%	57,1%

3630	25	60	50	30	48,2%	55,7%	66,3%	71,7%	58,8%
3631	25	60	50	35	48,6%	55,4%	67,0%	73,0%	60,4%
3632	25	60	50	40	49,1%	55,1%	67,8%	74,4%	62,1%
3633	25	60	50	45	49,5%	54,7%	68,5%	75,7%	63,7%
3634	25	60	50	50	50,0%	54,4%	69,2%	76,9%	65,2%
3635	25	60	50	55	50,4%	54,1%	69,9%	78,1%	66,8%
3636	25	60	50	60	50,8%	53,8%	70,7%	79,3%	68,3%
3637	25	60	50	65	51,3%	53,4%	71,4%	80,4%	69,7%
3638	25	60	50	70	51,7%	53,1%	72,0%	81,5%	71,2%
3639	25	60	50	75	52,2%	52,8%	72,7%	82,5%	72,5%
3640	25	60	50	80	52,6%	52,5%	73,4%	83,4%	73,9%
3641	25	60	55	20	41,2%	50,6%	61,8%	67,5%	54,4%
3642	25	60	55	25	41,7%	50,3%	62,6%	69,0%	56,1%
3643	25	60	55	30	42,1%	50,0%	63,4%	70,4%	57,8%
3644	25	60	55	35	42,5%	49,6%	64,2%	71,8%	59,4%
3645	25	60	55	40	42,9%	49,3%	65,0%	73,2%	61,1%
3646	25	60	55	45	43,4%	49,0%	65,8%	74,5%	62,7%
3647	25	60	55	50	43,8%	48,6%	66,5%	75,8%	64,3%
3648	25	60	55	55	44,2%	48,3%	67,3%	77,1%	65,8%
3649	25	60	55	60	44,7%	48,0%	68,0%	78,3%	67,4%
3650	25	60	55	65	45,1%	47,7%	68,7%	79,4%	68,8%
3651	25	60	55	70	45,6%	47,3%	69,5%	80,5%	70,3%
3652	25	60	55	75	46,0%	47,0%	70,2%	81,6%	71,7%
3653	25	60	55	80	46,4%	46,7%	70,9%	82,6%	73,1%
3654	25	60	60	20	35,4%	44,9%	58,9%	66,1%	53,4%
3655	25	60	60	25	35,8%	44,5%	59,7%	67,6%	55,1%
3656	25	60	60	30	36,2%	44,2%	60,5%	69,1%	56,8%
3657	25	60	60	35	36,6%	43,9%	61,3%	70,6%	58,4%
3658	25	60	60	40	37,0%	43,6%	62,1%	72,0%	60,1%
3659	25	60	60	45	37,4%	43,2%	62,9%	73,4%	61,7%
3660	25	60	60	50	37,9%	42,9%	63,7%	74,7%	63,3%
3661	25	60	60	55	38,3%	42,6%	64,5%	76,0%	64,9%
3662	25	60	60	60	38,7%	42,3%	65,3%	77,2%	66,4%

3663	25	60	60	65	39,1%	42,0%	66,0%	78,4%	68,0%
3664	25	60	60	70	39,5%	41,6%	66,8%	79,5%	69,4%
3665	25	60	60	75	40,0%	41,3%	67,5%	80,6%	70,9%
3666	25	60	60	80	40,4%	41,0%	68,3%	81,7%	72,3%
3667	25	60	65	20	30,0%	39,2%	55,8%	64,7%	52,4%
3668	25	60	65	25	30,3%	38,9%	56,7%	66,3%	54,1%
3669	25	60	65	30	30,7%	38,6%	57,5%	67,8%	55,8%
3670	25	60	65	35	31,1%	38,3%	58,3%	69,3%	57,4%
3671	25	60	65	40	31,5%	38,0%	59,1%	70,7%	59,1%
3672	25	60	65	45	31,9%	37,7%	60,0%	72,1%	60,7%
3673	25	60	65	50	32,2%	37,4%	60,8%	73,5%	62,4%
3674	25	60	65	55	32,6%	37,1%	61,6%	74,8%	64,0%
3675	25	60	65	60	33,0%	36,8%	62,4%	76,1%	65,5%
3676	25	60	65	65	33,4%	36,5%	63,2%	77,3%	67,1%
3677	25	60	65	70	33,8%	36,2%	64,0%	78,5%	68,5%
3678	25	60	65	75	34,2%	35,9%	64,7%	79,7%	70,0%
3679	25	60	65	80	34,6%	35,6%	65,5%	80,8%	71,4%
3680	25	60	70	20	25,1%	33,9%	52,7%	63,3%	51,3%
3681	25	60	70	25	25,4%	33,6%	53,6%	64,9%	53,0%
3682	25	60	70	30	25,7%	33,3%	54,4%	66,5%	54,7%
3683	25	60	70	35	26,1%	33,0%	55,3%	68,0%	56,4%
3684	25	60	70	40	26,4%	32,7%	56,1%	69,5%	58,1%
3685	25	60	70	45	26,8%	32,4%	56,9%	70,9%	59,8%
3686	25	60	70	50	27,1%	32,1%	57,8%	72,3%	61,4%
3687	25	60	70	55	27,5%	31,9%	58,6%	73,7%	63,0%
3688	25	60	70	60	27,8%	31,6%	59,4%	75,0%	64,6%
3689	25	60	70	65	28,2%	31,3%	60,2%	76,3%	66,1%
3690	25	60	70	70	28,5%	31,0%	61,1%	77,5%	67,7%
3691	25	60	70	75	28,9%	30,7%	61,9%	78,7%	69,1%
3692	25	60	70	80	29,2%	30,5%	62,7%	79,8%	70,6%
3693	25	60	75	20	20,7%	28,9%	49,6%	61,9%	50,3%
3694	25	60	75	25	21,0%	28,7%	50,5%	63,5%	52,0%
3695	25	60	75	30	21,3%	28,4%	51,3%	65,1%	53,7%

3696	25	60	75	35	21,6%	28,1%	52,2%	66,6%	55,4%
3697	25	60	75	40	21,9%	27,9%	53,0%	68,2%	57,1%
3698	25	60	75	45	22,2%	27,6%	53,9%	69,6%	58,8%
3699	25	60	75	50	22,5%	27,3%	54,7%	71,1%	60,4%
3700	25	60	75	55	22,8%	27,1%	55,6%	72,5%	62,0%
3701	25	60	75	60	23,1%	26,8%	56,4%	73,8%	63,6%
3702	25	60	75	65	23,4%	26,6%	57,2%	75,1%	65,2%
3703	25	60	75	70	23,8%	26,3%	58,1%	76,4%	66,7%
3704	25	60	75	75	24,1%	26,1%	58,9%	77,6%	68,2%
3705	25	60	75	80	24,4%	25,8%	59,7%	78,8%	69,7%
3706	25	60	80	20	17,0%	24,4%	46,5%	60,4%	49,3%
3707	25	60	80	25	17,2%	24,2%	47,4%	62,1%	51,0%
3708	25	60	80	30	17,5%	23,9%	48,2%	63,7%	52,7%
3709	25	60	80	35	17,7%	23,7%	49,1%	65,3%	54,4%
3710	25	60	80	40	18,0%	23,5%	49,9%	66,8%	56,1%
3711	25	60	80	45	18,2%	23,2%	50,8%	68,3%	57,8%
3712	25	60	80	50	18,5%	23,0%	51,6%	69,8%	59,4%
3713	25	60	80	55	18,8%	22,8%	52,5%	71,2%	61,1%
3714	25	60	80	60	19,0%	22,5%	53,3%	72,6%	62,7%
3715	25	60	80	65	19,3%	22,3%	54,2%	74,0%	64,3%
3716	25	60	80	70	19,6%	22,1%	55,0%	75,3%	65,8%
3717	25	60	80	75	19,9%	21,9%	55,8%	76,5%	67,3%
3718	25	60	80	80	20,1%	21,6%	56,7%	77,8%	68,8%
3719	25	65	20	20	81,2%	84,8%	78,9%	74,7%	59,1%
3720	25	65	20	25	81,5%	84,6%	79,5%	76,0%	60,8%
3721	25	65	20	30	81,7%	84,5%	80,0%	77,2%	62,4%
3722	25	65	20	35	82,0%	84,3%	80,6%	78,4%	64,0%
3723	25	65	20	40	82,2%	84,1%	81,1%	79,5%	65,6%
3724	25	65	20	45	82,5%	83,9%	81,6%	80,6%	67,1%
3725	25	65	20	50	82,8%	83,8%	82,1%	81,7%	68,6%
3726	25	65	20	55	83,0%	83,6%	82,6%	82,7%	70,0%
3727	25	65	20	60	83,3%	83,4%	83,1%	83,7%	71,5%
3728	25	65	20	65	83,5%	83,2%	83,6%	84,6%	72,8%

3729	25	65	20	70	83,7%	83,0%	84,0%	85,5%	74,2%
3730	25	65	20	75	84,0%	82,9%	84,5%	86,3%	75,5%
3731	25	65	20	80	84,2%	82,7%	84,9%	87,1%	76,7%
3732	25	65	25	20	77,1%	81,6%	76,8%	73,5%	58,1%
3733	25	65	25	25	77,4%	81,4%	77,4%	74,8%	59,8%
3734	25	65	25	30	77,7%	81,2%	78,0%	76,1%	61,4%
3735	25	65	25	35	78,0%	81,0%	78,6%	77,3%	63,0%
3736	25	65	25	40	78,3%	80,8%	79,1%	78,5%	64,6%
3737	25	65	25	45	78,6%	80,6%	79,7%	79,7%	66,2%
3738	25	65	25	50	78,9%	80,4%	80,2%	80,8%	67,7%
3739	25	65	25	55	79,2%	80,2%	80,8%	81,8%	69,2%
3740	25	65	25	60	79,5%	80,0%	81,3%	82,8%	70,6%
3741	25	65	25	65	79,8%	79,8%	81,8%	83,8%	72,0%
3742	25	65	25	70	80,1%	79,5%	82,3%	84,7%	73,4%
3743	25	65	25	75	80,4%	79,3%	82,8%	85,6%	74,7%
3744	25	65	25	80	80,6%	79,1%	83,3%	86,4%	76,0%
3745	25	65	30	20	72,5%	77,9%	74,5%	72,3%	57,1%
3746	25	65	30	25	72,8%	77,6%	75,1%	73,7%	58,8%
3747	25	65	30	30	73,2%	77,4%	75,8%	75,0%	60,5%
3748	25	65	30	35	73,5%	77,2%	76,4%	76,3%	62,1%
3749	25	65	30	40	73,9%	77,0%	77,0%	77,5%	63,7%
3750	25	65	30	45	74,2%	76,7%	77,6%	78,7%	65,2%
3751	25	65	30	50	74,5%	76,5%	78,2%	79,8%	66,8%
3752	25	65	30	55	74,9%	76,2%	78,7%	80,9%	68,3%
3753	25	65	30	60	75,2%	76,0%	79,3%	81,9%	69,7%
3754	25	65	30	65	75,5%	75,8%	79,9%	82,9%	71,2%
3755	25	65	30	70	75,9%	75,5%	80,4%	83,9%	72,6%
3756	25	65	30	75	76,2%	75,3%	80,9%	84,8%	73,9%
3757	25	65	30	80	76,5%	75,0%	81,5%	85,7%	75,2%
3758	25	65	35	20	67,3%	73,6%	72,1%	71,1%	56,1%
3759	25	65	35	25	67,7%	73,4%	72,7%	72,5%	57,8%
3760	25	65	35	30	68,1%	73,1%	73,4%	73,8%	59,5%
3761	25	65	35	35	68,5%	72,9%	74,1%	75,1%	61,1%

3762	25	65	35	40	68,8%	72,6%	74,7%	76,4%	62,7%
3763	25	65	35	45	69,2%	72,3%	75,4%	77,6%	64,3%
3764	25	65	35	50	69,6%	72,1%	76,0%	78,8%	65,9%
3765	25	65	35	55	70,0%	71,8%	76,6%	79,9%	67,4%
3766	25	65	35	60	70,3%	71,5%	77,2%	81,0%	68,9%
3767	25	65	35	65	70,7%	71,3%	77,8%	82,0%	70,3%
3768	25	65	35	70	71,1%	71,0%	78,4%	83,0%	71,7%
3769	25	65	35	75	71,4%	70,7%	78,9%	84,0%	73,1%
3770	25	65	35	80	71,8%	70,5%	79,5%	84,9%	74,4%
3771	25	65	40	20	61,7%	68,9%	69,5%	69,8%	55,1%
3772	25	65	40	25	62,1%	68,6%	70,2%	71,2%	56,8%
3773	25	65	40	30	62,5%	68,3%	70,9%	72,6%	58,5%
3774	25	65	40	35	62,9%	68,1%	71,6%	74,0%	60,1%
3775	25	65	40	40	63,3%	67,8%	72,3%	75,3%	61,7%
3776	25	65	40	45	63,7%	67,5%	73,0%	76,5%	63,4%
3777	25	65	40	50	64,1%	67,2%	73,6%	77,8%	64,9%
3778	25	65	40	55	64,5%	66,9%	74,3%	78,9%	66,5%
3779	25	65	40	60	64,9%	66,6%	74,9%	80,1%	68,0%
3780	25	65	40	65	65,3%	66,3%	75,6%	81,1%	69,5%
3781	25	65	40	70	65,7%	66,0%	76,2%	82,2%	70,9%
3782	25	65	40	75	66,1%	65,7%	76,8%	83,2%	72,3%
3783	25	65	40	80	66,5%	65,5%	77,4%	84,1%	73,6%
3784	25	65	45	20	55,7%	63,8%	66,8%	68,5%	54,1%
3785	25	65	45	25	56,1%	63,5%	67,6%	70,0%	55,8%
3786	25	65	45	30	56,5%	63,2%	68,3%	71,4%	57,5%
3787	25	65	45	35	57,0%	62,8%	69,0%	72,8%	59,1%
3788	25	65	45	40	57,4%	62,5%	69,7%	74,1%	60,8%
3789	25	65	45	45	57,8%	62,2%	70,5%	75,4%	62,4%
3790	25	65	45	50	58,3%	61,9%	71,2%	76,7%	64,0%
3791	25	65	45	55	58,7%	61,6%	71,8%	77,9%	65,5%
3792	25	65	45	60	59,1%	61,3%	72,5%	79,1%	67,1%
3793	25	65	45	65	59,5%	61,0%	73,2%	80,2%	68,6%
3794	25	65	45	70	60,0%	60,7%	73,9%	81,3%	70,0%

3795	25	65	45	75	60,4%	60,4%	74,5%	82,3%	71,4%
3796	25	65	45	80	60,8%	60,1%	75,2%	83,3%	72,8%
3797	25	65	50	20	49,5%	58,3%	64,0%	67,2%	53,1%
3798	25	65	50	25	50,0%	58,0%	64,8%	68,7%	54,8%
3799	25	65	50	30	50,4%	57,6%	65,5%	70,1%	56,4%
3800	25	65	50	35	50,8%	57,3%	66,3%	71,6%	58,1%
3801	25	65	50	40	51,3%	57,0%	67,1%	72,9%	59,8%
3802	25	65	50	45	51,7%	56,7%	67,8%	74,3%	61,4%
3803	25	65	50	50	52,2%	56,4%	68,5%	75,6%	63,0%
3804	25	65	50	55	52,6%	56,0%	69,3%	76,8%	64,6%
3805	25	65	50	60	53,0%	55,7%	70,0%	78,0%	66,2%
3806	25	65	50	65	53,5%	55,4%	70,7%	79,2%	67,7%
3807	25	65	50	70	53,9%	55,1%	71,4%	80,3%	69,2%
3808	25	65	50	75	54,4%	54,7%	72,1%	81,4%	70,6%
3809	25	65	50	80	54,8%	54,4%	72,8%	82,4%	72,0%
3810	25	65	55	20	43,4%	52,6%	61,1%	65,8%	52,0%
3811	25	65	55	25	43,8%	52,3%	61,9%	67,3%	53,7%
3812	25	65	55	30	44,3%	51,9%	62,7%	68,8%	55,4%
3813	25	65	55	35	44,7%	51,6%	63,5%	70,3%	57,1%
3814	25	65	55	40	45,1%	51,3%	64,3%	71,7%	58,8%
3815	25	65	55	45	45,6%	50,9%	65,0%	73,1%	60,4%
3816	25	65	55	50	46,0%	50,6%	65,8%	74,4%	62,1%
3817	25	65	55	55	46,4%	50,3%	66,6%	75,7%	63,7%
3818	25	65	55	60	46,9%	50,0%	67,3%	77,0%	65,2%
3819	25	65	55	65	47,3%	49,6%	68,1%	78,2%	66,8%
3820	25	65	55	70	47,8%	49,3%	68,8%	79,3%	68,3%
3821	25	65	55	75	48,2%	49,0%	69,5%	80,4%	69,7%
3822	25	65	55	80	48,6%	48,7%	70,2%	81,5%	71,2%
3823	25	65	60	20	37,5%	46,8%	58,1%	64,4%	51,0%
3824	25	65	60	25	37,9%	46,5%	58,9%	66,0%	52,7%
3825	25	65	60	30	38,3%	46,2%	59,7%	67,5%	54,4%
3826	25	65	60	35	38,7%	45,8%	60,6%	69,0%	56,1%
3827	25	65	60	40	39,1%	45,5%	61,4%	70,5%	57,8%

3828	25	65	60	45	39,5%	45,2%	62,2%	71,9%	59,4%
3829	25	65	60	50	40,0%	44,9%	63,0%	73,3%	61,1%
3830	25	65	60	55	40,4%	44,5%	63,7%	74,6%	62,7%
3831	25	65	60	60	40,8%	44,2%	64,5%	75,9%	64,3%
3832	25	65	60	65	41,2%	43,9%	65,3%	77,1%	65,8%
3833	25	65	60	70	41,7%	43,6%	66,1%	78,3%	67,4%
3834	25	65	60	75	42,1%	43,2%	66,8%	79,5%	68,9%
3835	25	65	60	80	42,5%	42,9%	67,6%	80,6%	70,3%
3836	25	65	65	20	31,9%	41,1%	55,0%	63,0%	50,0%
3837	25	65	65	25	32,3%	40,8%	55,9%	64,6%	51,7%
3838	25	65	65	30	32,6%	40,5%	56,7%	66,2%	53,4%
3839	25	65	65	35	33,0%	40,2%	57,5%	67,7%	55,1%
3840	25	65	65	40	33,4%	39,9%	58,4%	69,2%	56,8%
3841	25	65	65	45	33,8%	39,6%	59,2%	70,6%	58,4%
3842	25	65	65	50	34,2%	39,2%	60,0%	72,0%	60,1%
3843	25	65	65	55	34,6%	38,9%	60,8%	73,4%	61,7%
3844	25	65	65	60	35,0%	38,6%	61,6%	74,7%	63,3%
3845	25	65	65	65	35,4%	38,3%	62,4%	76,0%	64,9%
3846	25	65	65	70	35,8%	38,0%	63,2%	77,3%	66,5%
3847	25	65	65	75	36,2%	37,7%	64,0%	78,4%	68,0%
3848	25	65	65	80	36,6%	37,4%	64,8%	79,6%	69,4%
3849	25	65	70	20	26,8%	35,7%	52,0%	61,6%	48,9%
3850	25	65	70	25	27,1%	35,4%	52,8%	63,2%	50,6%
3851	25	65	70	30	27,5%	35,1%	53,6%	64,8%	52,4%
3852	25	65	70	35	27,8%	34,8%	54,5%	66,3%	54,1%
3853	25	65	70	40	28,2%	34,5%	55,3%	67,9%	55,8%
3854	25	65	70	45	28,5%	34,2%	56,2%	69,4%	57,4%
3855	25	65	70	50	28,9%	33,9%	57,0%	70,8%	59,1%
3856	25	65	70	55	29,3%	33,6%	57,8%	72,2%	60,8%
3857	25	65	70	60	29,6%	33,3%	58,7%	73,6%	62,4%
3858	25	65	70	65	30,0%	33,0%	59,5%	74,9%	64,0%
3859	25	65	70	70	30,4%	32,7%	60,3%	76,2%	65,5%
3860	25	65	70	75	30,7%	32,4%	61,1%	77,4%	67,1%

3861	25	65	70	80	31,1%	32,2%	61,9%	78,6%	68,6%
3862	25	65	75	20	22,2%	30,6%	48,8%	60,1%	47,9%
3863	25	65	75	25	22,5%	30,3%	49,7%	61,8%	49,6%
3864	25	65	75	30	22,8%	30,0%	50,5%	63,4%	51,3%
3865	25	65	75	35	23,1%	29,7%	51,4%	65,0%	53,0%
3866	25	65	75	40	23,5%	29,5%	52,2%	66,5%	54,7%
3867	25	65	75	45	23,8%	29,2%	53,1%	68,0%	56,4%
3868	25	65	75	50	24,1%	28,9%	53,9%	69,5%	58,1%
3869	25	65	75	55	24,4%	28,7%	54,8%	71,0%	59,8%
3870	25	65	75	60	24,7%	28,4%	55,6%	72,4%	61,4%
3871	25	65	75	65	25,1%	28,1%	56,5%	73,7%	63,0%
3872	25	65	75	70	25,4%	27,9%	57,3%	75,0%	64,6%
3873	25	65	75	75	25,7%	27,6%	58,1%	76,3%	66,1%
3874	25	65	75	80	26,1%	27,3%	58,9%	77,5%	67,7%
3875	25	65	80	20	18,2%	25,9%	45,8%	58,7%	46,9%
3876	25	65	80	25	18,5%	25,6%	46,6%	60,3%	48,6%
3877	25	65	80	30	18,8%	25,4%	47,4%	62,0%	50,3%
3878	25	65	80	35	19,0%	25,1%	48,3%	63,6%	52,0%
3879	25	65	80	40	19,3%	24,9%	49,1%	65,2%	53,7%
3880	25	65	80	45	19,6%	24,7%	50,0%	66,7%	55,4%
3881	25	65	80	50	19,9%	24,4%	50,8%	68,2%	57,1%
3882	25	65	80	55	20,2%	24,2%	51,7%	69,7%	58,8%
3883	25	65	80	60	20,4%	23,9%	52,5%	71,1%	60,4%
3884	25	65	80	65	20,7%	23,7%	53,4%	72,5%	62,0%
3885	25	65	80	70	21,0%	23,5%	54,2%	73,9%	63,6%
3886	25	65	80	75	21,3%	23,2%	55,1%	75,2%	65,2%
3887	25	65	80	80	21,6%	23,0%	55,9%	76,5%	66,7%
3888	25	70	20	20	82,5%	85,8%	78,4%	73,3%	56,8%
3889	25	70	20	25	82,8%	85,6%	79,0%	74,6%	58,5%
3890	25	70	20	30	83,0%	85,5%	79,5%	75,9%	60,1%
3891	25	70	20	35	83,3%	85,3%	80,1%	77,1%	61,8%
3892	25	70	20	40	83,5%	85,1%	80,6%	78,3%	63,4%
3893	25	70	20	45	83,7%	85,0%	81,1%	79,5%	64,9%

3894	25	70	20	50	84,0%	84,8%	81,6%	80,6%	66,5%
3895	25	70	20	55	84,2%	84,6%	82,1%	81,6%	68,0%
3896	25	70	20	60	84,5%	84,5%	82,6%	82,6%	69,5%
3897	25	70	20	65	84,7%	84,3%	83,1%	83,6%	70,9%
3898	25	70	20	70	84,9%	84,1%	83,6%	84,5%	72,3%
3899	25	70	20	75	85,1%	84,0%	84,1%	85,4%	73,6%
3900	25	70	20	80	85,4%	83,8%	84,5%	86,2%	75,0%
3901	25	70	25	20	78,7%	82,7%	76,2%	72,0%	55,8%
3902	25	70	25	25	79,0%	82,6%	76,8%	73,4%	57,5%
3903	25	70	25	30	79,2%	82,4%	77,4%	74,7%	59,1%
3904	25	70	25	35	79,5%	82,2%	78,0%	76,0%	60,8%
3905	25	70	25	40	79,8%	82,0%	78,6%	77,2%	62,4%
3906	25	70	25	45	80,1%	81,8%	79,2%	78,4%	64,0%
3907	25	70	25	50	80,4%	81,6%	79,7%	79,6%	65,6%
3908	25	70	25	55	80,7%	81,4%	80,3%	80,7%	67,1%
3909	25	70	25	60	80,9%	81,2%	80,8%	81,7%	68,6%
3910	25	70	25	65	81,2%	81,0%	81,3%	82,7%	70,0%
3911	25	70	25	70	81,5%	80,8%	81,8%	83,7%	71,5%
3912	25	70	25	75	81,7%	80,6%	82,3%	84,6%	72,8%
3913	25	70	25	80	82,0%	80,4%	82,8%	85,5%	74,2%
3914	25	70	30	20	74,2%	79,2%	73,9%	70,8%	54,8%
3915	25	70	30	25	74,6%	79,0%	74,5%	72,2%	56,5%
3916	25	70	30	30	74,9%	78,8%	75,2%	73,6%	58,1%
3917	25	70	30	35	75,2%	78,5%	75,8%	74,9%	59,8%
3918	25	70	30	40	75,5%	78,3%	76,4%	76,2%	61,4%
3919	25	70	30	45	75,9%	78,1%	77,0%	77,4%	63,0%
3920	25	70	30	50	76,2%	77,9%	77,6%	78,6%	64,6%
3921	25	70	30	55	76,5%	77,6%	78,2%	79,7%	66,2%
3922	25	70	30	60	76,8%	77,4%	78,8%	80,8%	67,7%
3923	25	70	30	65	77,1%	77,2%	79,3%	81,9%	69,2%
3924	25	70	30	70	77,4%	77,0%	79,9%	82,9%	70,6%
3925	25	70	30	75	77,8%	76,7%	80,4%	83,8%	72,0%
3926	25	70	30	80	78,1%	76,5%	81,0%	84,7%	73,4%

3927	25	70	35	20	69,2%	75,1%	71,4%	69,5%	53,8%
3928	25	70	35	25	69,6%	74,9%	72,1%	71,0%	55,5%
3929	25	70	35	30	70,0%	74,6%	72,8%	72,4%	57,1%
3930	25	70	35	35	70,3%	74,4%	73,5%	73,7%	58,8%
3931	25	70	35	40	70,7%	74,1%	74,1%	75,0%	60,5%
3932	25	70	35	45	71,1%	73,9%	74,8%	76,3%	62,1%
3933	25	70	35	50	71,4%	73,6%	75,4%	77,5%	63,7%
3934	25	70	35	55	71,8%	73,4%	76,0%	78,7%	65,2%
3935	25	70	35	60	72,1%	73,1%	76,6%	79,8%	66,8%
3936	25	70	35	65	72,5%	72,9%	77,2%	80,9%	68,3%
3937	25	70	35	70	72,8%	72,6%	77,8%	82,0%	69,8%
3938	25	70	35	75	73,2%	72,3%	78,4%	83,0%	71,2%
3939	25	70	35	80	73,5%	72,1%	79,0%	83,9%	72,6%
3940	25	70	40	20	63,7%	70,6%	68,8%	68,2%	52,7%
3941	25	70	40	25	64,1%	70,3%	69,5%	69,7%	54,4%
3942	25	70	40	30	64,5%	70,0%	70,3%	71,1%	56,1%
3943	25	70	40	35	64,9%	69,8%	71,0%	72,5%	57,8%
3944	25	70	40	40	65,3%	69,5%	71,7%	73,9%	59,5%
3945	25	70	40	45	65,7%	69,2%	72,3%	75,2%	61,1%
3946	25	70	40	50	66,1%	68,9%	73,0%	76,4%	62,7%
3947	25	70	40	55	66,5%	68,6%	73,7%	77,7%	64,3%
3948	25	70	40	60	66,9%	68,4%	74,3%	78,8%	65,9%
3949	25	70	40	65	67,3%	68,1%	75,0%	80,0%	67,4%
3950	25	70	40	70	67,7%	67,8%	75,6%	81,1%	68,9%
3951	25	70	40	75	68,1%	67,5%	76,2%	82,1%	70,3%
3952	25	70	40	80	68,5%	67,2%	76,8%	83,1%	71,7%
3953	25	70	45	20	57,9%	65,6%	66,1%	66,9%	51,7%
3954	25	70	45	25	58,3%	65,3%	66,9%	68,4%	53,4%
3955	25	70	45	30	58,7%	65,0%	67,6%	69,9%	55,1%
3956	25	70	45	35	59,1%	64,7%	68,3%	71,3%	56,8%
3957	25	70	45	40	59,6%	64,4%	69,1%	72,7%	58,5%
3958	25	70	45	45	60,0%	64,1%	69,8%	74,0%	60,1%
3959	25	70	45	50	60,4%	63,8%	70,5%	75,3%	61,8%

3960	25	70	45	55	60,8%	63,5%	71,2%	76,6%	63,4%
3961	25	70	45	60	61,2%	63,2%	71,9%	77,8%	64,9%
3962	25	70	45	65	61,7%	62,9%	72,6%	79,0%	66,5%
3963	25	70	45	70	62,1%	62,6%	73,2%	80,1%	68,0%
3964	25	70	45	75	62,5%	62,2%	73,9%	81,2%	69,5%
3965	25	70	45	80	62,9%	61,9%	74,6%	82,2%	70,9%
3966	25	70	50	20	51,7%	60,2%	63,3%	65,5%	50,7%
3967	25	70	50	25	52,2%	59,9%	64,0%	67,1%	52,4%
3968	25	70	50	30	52,6%	59,5%	64,8%	68,6%	54,1%
3969	25	70	50	35	53,1%	59,2%	65,6%	70,0%	55,8%
3970	25	70	50	40	53,5%	58,9%	66,4%	71,5%	57,5%
3971	25	70	50	45	53,9%	58,6%	67,1%	72,8%	59,1%
3972	25	70	50	50	54,4%	58,3%	67,9%	74,2%	60,8%
3973	25	70	50	55	54,8%	58,0%	68,6%	75,5%	62,4%
3974	25	70	50	60	55,3%	57,6%	69,3%	76,7%	64,0%
3975	25	70	50	65	55,7%	57,3%	70,0%	77,9%	65,5%
3976	25	70	50	70	56,1%	57,0%	70,7%	79,1%	67,1%
3977	25	70	50	75	56,6%	56,7%	71,4%	80,2%	68,6%
3978	25	70	50	80	57,0%	56,4%	72,1%	81,3%	70,0%
3979	25	70	55	20	45,6%	54,5%	60,3%	64,1%	49,6%
3980	25	70	55	25	46,0%	54,2%	61,1%	65,7%	51,4%
3981	25	70	55	30	46,5%	53,9%	61,9%	67,2%	53,1%
3982	25	70	55	35	46,9%	53,6%	62,7%	68,7%	54,8%
3983	25	70	55	40	47,3%	53,2%	63,5%	70,2%	56,5%
3984	25	70	55	45	47,8%	52,9%	64,3%	71,6%	58,1%
3985	25	70	55	50	48,2%	52,6%	65,1%	73,0%	59,8%
3986	25	70	55	55	48,7%	52,3%	65,9%	74,3%	61,4%
3987	25	70	55	60	49,1%	51,9%	66,6%	75,6%	63,0%
3988	25	70	55	65	49,5%	51,6%	67,4%	76,9%	64,6%
3989	25	70	55	70	50,0%	51,3%	68,1%	78,1%	66,2%
3990	25	70	55	75	50,4%	51,0%	68,8%	79,2%	67,7%
3991	25	70	55	80	50,9%	50,6%	69,6%	80,4%	69,2%
3992	25	70	60	20	39,6%	48,8%	57,3%	62,7%	48,6%

3993	25	70	60	25	40,0%	48,4%	58,1%	64,3%	50,3%
3994	25	70	60	30	40,4%	48,1%	59,0%	65,9%	52,0%
3995	25	70	60	35	40,8%	47,8%	59,8%	67,4%	53,7%
3996	25	70	60	40	41,3%	47,5%	60,6%	68,9%	55,4%
3997	25	70	60	45	41,7%	47,1%	61,4%	70,4%	57,1%
3998	25	70	60	50	42,1%	46,8%	62,2%	71,8%	58,8%
3999	25	70	60	55	42,5%	46,5%	63,0%	73,2%	60,4%
4000	25	70	60	60	43,0%	46,2%	63,8%	74,5%	62,1%
4001	25	70	60	65	43,4%	45,8%	64,6%	75,8%	63,7%
4002	25	70	60	70	43,8%	45,5%	65,3%	77,0%	65,2%
4003	25	70	60	75	44,3%	45,2%	66,1%	78,2%	66,8%
4004	25	70	60	80	44,7%	44,9%	66,9%	79,4%	68,3%
4005	25	70	65	20	33,8%	43,0%	54,3%	61,3%	47,6%
4006	25	70	65	25	34,2%	42,7%	55,1%	62,9%	49,3%
4007	25	70	65	30	34,6%	42,4%	55,9%	64,5%	51,0%
4008	25	70	65	35	35,0%	42,1%	56,8%	66,1%	52,7%
4009	25	70	65	40	35,4%	41,8%	57,6%	67,6%	54,4%
4010	25	70	65	45	35,8%	41,5%	58,4%	69,1%	56,1%
4011	25	70	65	50	36,2%	41,1%	59,3%	70,5%	57,8%
4012	25	70	65	55	36,6%	40,8%	60,1%	71,9%	59,4%
4013	25	70	65	60	37,1%	40,5%	60,9%	73,3%	61,1%
4014	25	70	65	65	37,5%	40,2%	61,7%	74,6%	62,7%
4015	25	70	65	70	37,9%	39,9%	62,5%	75,9%	64,3%
4016	25	70	65	75	38,3%	39,6%	63,3%	77,2%	65,8%
4017	25	70	65	80	38,7%	39,2%	64,1%	78,4%	67,4%
4018	25	70	70	20	28,5%	37,5%	51,2%	59,8%	46,6%
4019	25	70	70	25	28,9%	37,2%	52,0%	61,5%	48,3%
4020	25	70	70	30	29,3%	36,9%	52,9%	63,1%	50,0%
4021	25	70	70	35	29,6%	36,6%	53,7%	64,7%	51,7%
4022	25	70	70	40	30,0%	36,3%	54,5%	66,2%	53,4%
4023	25	70	70	45	30,4%	36,0%	55,4%	67,8%	55,1%
4024	25	70	70	50	30,7%	35,7%	56,2%	69,2%	56,8%
4025	25	70	70	55	31,1%	35,4%	57,1%	70,7%	58,5%

4026	25	70	70	60	31,5%	35,1%	57,9%	72,1%	60,1%
4027	25	70	70	65	31,9%	34,8%	58,7%	73,5%	61,7%
4028	25	70	70	70	32,3%	34,5%	59,5%	74,8%	63,3%
4029	25	70	70	75	32,7%	34,2%	60,3%	76,1%	64,9%
4030	25	70	70	80	33,0%	33,9%	61,2%	77,3%	66,5%
4031	25	70	75	20	23,8%	32,3%	48,1%	58,3%	45,5%
4032	25	70	75	25	24,1%	32,0%	48,9%	60,0%	47,2%
4033	25	70	75	30	24,4%	31,7%	49,8%	61,7%	48,9%
4034	25	70	75	35	24,8%	31,4%	50,6%	63,3%	50,7%
4035	25	70	75	40	25,1%	31,1%	51,4%	64,9%	52,4%
4036	25	70	75	45	25,4%	30,8%	52,3%	66,4%	54,1%
4037	25	70	75	50	25,8%	30,6%	53,1%	67,9%	55,8%
4038	25	70	75	55	26,1%	30,3%	54,0%	69,4%	57,4%
4039	25	70	75	60	26,4%	30,0%	54,8%	70,9%	59,1%
4040	25	70	75	65	26,8%	29,7%	55,7%	72,3%	60,8%
4041	25	70	75	70	27,1%	29,5%	56,5%	73,6%	62,4%
4042	25	70	75	75	27,5%	29,2%	57,3%	74,9%	64,0%
4043	25	70	75	80	27,8%	28,9%	58,2%	76,2%	65,5%
4044	25	70	80	20	19,6%	27,4%	45,0%	56,9%	44,5%
4045	25	70	80	25	19,9%	27,2%	45,8%	58,5%	46,2%
4046	25	70	80	30	20,2%	26,9%	46,7%	60,2%	47,9%
4047	25	70	80	35	20,4%	26,7%	47,5%	61,8%	49,6%
4048	25	70	80	40	20,7%	26,4%	48,3%	63,5%	51,3%
4049	25	70	80	45	21,0%	26,2%	49,2%	65,0%	53,0%
4050	25	70	80	50	21,3%	25,9%	50,0%	66,6%	54,7%
4051	25	70	80	55	21,6%	25,6%	50,9%	68,1%	56,4%
4052	25	70	80	60	21,9%	25,4%	51,7%	69,6%	58,1%
4053	25	70	80	65	22,2%	25,2%	52,6%	71,0%	59,8%
4054	25	70	80	70	22,5%	24,9%	53,4%	72,4%	61,4%
4055	25	70	80	75	22,8%	24,7%	54,3%	73,8%	63,0%
4056	25	70	80	80	23,1%	24,4%	55,1%	75,1%	64,6%
4057	25	75	20	20	83,8%	86,7%	77,8%	71,8%	54,5%
4058	25	75	20	25	84,0%	86,6%	78,4%	73,2%	56,2%

4059	25	75	20	30	84,2%	86,4%	79,0%	74,5%	57,8%
4060	25	75	20	35	84,5%	86,3%	79,6%	75,8%	59,5%
4061	25	75	20	40	84,7%	86,1%	80,1%	77,0%	61,1%
4062	25	75	20	45	84,9%	86,0%	80,6%	78,2%	62,7%
4063	25	75	20	50	85,1%	85,8%	81,2%	79,4%	64,3%
4064	25	75	20	55	85,4%	85,6%	81,7%	80,5%	65,9%
4065	25	75	20	60	85,6%	85,5%	82,2%	81,5%	67,4%
4066	25	75	20	65	85,8%	85,3%	82,7%	82,6%	68,9%
4067	25	75	20	70	86,0%	85,1%	83,2%	83,5%	70,3%
4068	25	75	20	75	86,2%	85,0%	83,6%	84,4%	71,8%
4069	25	75	20	80	86,4%	84,8%	84,1%	85,3%	73,1%
4070	25	75	25	20	80,1%	83,8%	75,6%	70,5%	53,4%
4071	25	75	25	25	80,4%	83,7%	76,3%	71,9%	55,1%
4072	25	75	25	30	80,7%	83,5%	76,9%	73,3%	56,8%
4073	25	75	25	35	80,9%	83,3%	77,5%	74,6%	58,5%
4074	25	75	25	40	81,2%	83,1%	78,0%	75,9%	60,1%
4075	25	75	25	45	81,5%	82,9%	78,6%	77,2%	61,8%
4076	25	75	25	50	81,7%	82,7%	79,2%	78,4%	63,4%
4077	25	75	25	55	82,0%	82,6%	79,7%	79,5%	65,0%
4078	25	75	25	60	82,3%	82,4%	80,3%	80,6%	66,5%
4079	25	75	25	65	82,5%	82,2%	80,8%	81,7%	68,0%
4080	25	75	25	70	82,8%	82,0%	81,3%	82,7%	69,5%
4081	25	75	25	75	83,0%	81,8%	81,9%	83,6%	70,9%
4082	25	75	25	80	83,3%	81,6%	82,3%	84,6%	72,3%
4083	25	75	30	20	75,9%	80,5%	73,3%	69,2%	52,4%
4084	25	75	30	25	76,2%	80,3%	73,9%	70,7%	54,1%
4085	25	75	30	30	76,5%	80,0%	74,6%	72,1%	55,8%
4086	25	75	30	35	76,8%	79,8%	75,2%	73,5%	57,5%
4087	25	75	30	40	77,1%	79,6%	75,8%	74,8%	59,2%
4088	25	75	30	45	77,5%	79,4%	76,5%	76,1%	60,8%
4089	25	75	30	50	77,8%	79,2%	77,1%	77,3%	62,4%
4090	25	75	30	55	78,1%	79,0%	77,7%	78,5%	64,0%
4091	25	75	30	60	78,4%	78,8%	78,2%	79,6%	65,6%

4092	25	75	30	65	78,7%	78,5%	78,8%	80,7%	67,1%
4093	25	75	30	70	79,0%	78,3%	79,4%	81,8%	68,6%
4094	25	75	30	75	79,2%	78,1%	79,9%	82,8%	70,1%
4095	25	75	30	80	79,5%	77,9%	80,5%	83,7%	71,5%
4096	25	75	35	20	71,1%	76,6%	70,8%	67,9%	51,4%
4097	25	75	35	25	71,4%	76,3%	71,5%	69,4%	53,1%
4098	25	75	35	30	71,8%	76,1%	72,2%	70,9%	54,8%
4099	25	75	35	35	72,2%	75,9%	72,8%	72,3%	56,5%
4100	25	75	35	40	72,5%	75,6%	73,5%	73,6%	58,2%
4101	25	75	35	45	72,9%	75,4%	74,2%	74,9%	59,8%
4102	25	75	35	50	73,2%	75,1%	74,8%	76,2%	61,4%
4103	25	75	35	55	73,5%	74,9%	75,4%	77,4%	63,1%
4104	25	75	35	60	73,9%	74,6%	76,1%	78,6%	64,6%
4105	25	75	35	65	74,2%	74,4%	76,7%	79,8%	66,2%
4106	25	75	35	70	74,6%	74,1%	77,3%	80,8%	67,7%
4107	25	75	35	75	74,9%	73,9%	77,9%	81,9%	69,2%
4108	25	75	35	80	75,2%	73,6%	78,4%	82,9%	70,6%
4109	25	75	40	20	65,8%	72,2%	68,1%	66,6%	50,3%
4110	25	75	40	25	66,1%	71,9%	68,9%	68,1%	52,1%
4111	25	75	40	30	66,5%	71,7%	69,6%	69,6%	53,8%
4112	25	75	40	35	66,9%	71,4%	70,3%	71,0%	55,5%
4113	25	75	40	40	67,3%	71,1%	71,0%	72,4%	57,1%
4114	25	75	40	45	67,7%	70,8%	71,7%	73,8%	58,8%
4115	25	75	40	50	68,1%	70,6%	72,4%	75,1%	60,5%
4116	25	75	40	55	68,5%	70,3%	73,1%	76,4%	62,1%
4117	25	75	40	60	68,9%	70,0%	73,7%	77,6%	63,7%
4118	25	75	40	65	69,2%	69,8%	74,4%	78,8%	65,3%
4119	25	75	40	70	69,6%	69,5%	75,0%	79,9%	66,8%
4120	25	75	40	75	70,0%	69,2%	75,6%	81,0%	68,3%
4121	25	75	40	80	70,3%	68,9%	76,3%	82,0%	69,8%
4122	25	75	45	20	60,0%	67,3%	65,4%	65,2%	49,3%
4123	25	75	45	25	60,4%	67,0%	66,1%	66,8%	51,0%
4124	25	75	45	30	60,8%	66,7%	66,9%	68,3%	52,7%

4125	25	75	45	35	61,3%	66,4%	67,6%	69,8%	54,4%
4126	25	75	45	40	61,7%	66,2%	68,4%	71,2%	56,1%
4127	25	75	45	45	62,1%	65,9%	69,1%	72,6%	57,8%
4128	25	75	45	50	62,5%	65,6%	69,8%	73,9%	59,5%
4129	25	75	45	55	62,9%	65,3%	70,5%	75,2%	61,1%
4130	25	75	45	60	63,3%	65,0%	71,2%	76,5%	62,7%
4131	25	75	45	65	63,7%	64,7%	71,9%	77,7%	64,3%
4132	25	75	45	70	64,1%	64,4%	72,6%	78,9%	65,9%
4133	25	75	45	75	64,6%	64,1%	73,3%	80,0%	67,4%
4134	25	75	45	80	65,0%	63,8%	73,9%	81,1%	68,9%
4135	25	75	50	20	54,0%	62,0%	62,5%	63,8%	48,3%
4136	25	75	50	25	54,4%	61,7%	63,3%	65,4%	50,0%
4137	25	75	50	30	54,8%	61,4%	64,1%	66,9%	51,7%
4138	25	75	50	35	55,3%	61,1%	64,9%	68,4%	53,4%
4139	25	75	50	40	55,7%	60,8%	65,6%	69,9%	55,1%
4140	25	75	50	45	56,1%	60,5%	66,4%	71,3%	56,8%
4141	25	75	50	50	56,6%	60,2%	67,2%	72,7%	58,5%
4142	25	75	50	55	57,0%	59,9%	67,9%	74,1%	60,1%
4143	25	75	50	60	57,4%	59,6%	68,6%	75,4%	61,8%
4144	25	75	50	65	57,9%	59,2%	69,4%	76,6%	63,4%
4145	25	75	50	70	58,3%	58,9%	70,1%	77,9%	64,9%
4146	25	75	50	75	58,7%	58,6%	70,8%	79,0%	66,5%
4147	25	75	50	80	59,1%	58,3%	71,5%	80,1%	68,0%
4148	25	75	55	20	47,8%	56,5%	59,6%	62,4%	47,3%
4149	25	75	55	25	48,2%	56,2%	60,4%	64,0%	49,0%
4150	25	75	55	30	48,7%	55,8%	61,2%	65,6%	50,7%
4151	25	75	55	35	49,1%	55,5%	62,0%	67,1%	52,4%
4152	25	75	55	40	49,6%	55,2%	62,8%	68,6%	54,1%
4153	25	75	55	45	50,0%	54,9%	63,6%	70,1%	55,8%
4154	25	75	55	50	50,4%	54,5%	64,4%	71,5%	57,5%
4155	25	75	55	55	50,9%	54,2%	65,1%	72,9%	59,1%
4156	25	75	55	60	51,3%	53,9%	65,9%	74,2%	60,8%
4157	25	75	55	65	51,8%	53,6%	66,7%	75,5%	62,4%

4158	25	75	55	70	52,2%	53,2%	67,4%	76,8%	64,0%
4159	25	75	55	75	52,6%	52,9%	68,2%	78,0%	65,6%
4160	25	75	55	80	53,1%	52,6%	68,9%	79,2%	67,1%
4161	25	75	60	20	41,7%	50,7%	56,5%	61,0%	46,2%
4162	25	75	60	25	42,1%	50,4%	57,4%	62,6%	47,9%
4163	25	75	60	30	42,6%	50,1%	58,2%	64,2%	49,6%
4164	25	75	60	35	43,0%	49,8%	59,0%	65,8%	51,4%
4165	25	75	60	40	43,4%	49,4%	59,8%	67,3%	53,1%
4166	25	75	60	45	43,9%	49,1%	60,7%	68,8%	54,8%
4167	25	75	60	50	44,3%	48,8%	61,5%	70,3%	56,5%
4168	25	75	60	55	44,7%	48,5%	62,3%	71,7%	58,1%
4169	25	75	60	60	45,2%	48,1%	63,1%	73,1%	59,8%
4170	25	75	60	65	45,6%	47,8%	63,8%	74,4%	61,4%
4171	25	75	60	70	46,0%	47,5%	64,6%	75,7%	63,0%
4172	25	75	60	75	46,5%	47,1%	65,4%	76,9%	64,6%
4173	25	75	60	80	46,9%	46,8%	66,2%	78,1%	66,2%
4174	25	75	65	20	35,8%	45,0%	53,5%	59,5%	45,2%
4175	25	75	65	25	36,2%	44,7%	54,3%	61,2%	46,9%
4176	25	75	65	30	36,7%	44,3%	55,2%	62,8%	48,6%
4177	25	75	65	35	37,1%	44,0%	56,0%	64,4%	50,3%
4178	25	75	65	40	37,5%	43,7%	56,8%	65,9%	52,0%
4179	25	75	65	45	37,9%	43,4%	57,7%	67,5%	53,7%
4180	25	75	65	50	38,3%	43,1%	58,5%	69,0%	55,4%
4181	25	75	65	55	38,7%	42,7%	59,3%	70,4%	57,1%
4182	25	75	65	60	39,1%	42,4%	60,1%	71,8%	58,8%
4183	25	75	65	65	39,6%	42,1%	60,9%	73,2%	60,4%
4184	25	75	65	70	40,0%	41,8%	61,7%	74,5%	62,1%
4185	25	75	65	75	40,4%	41,5%	62,5%	75,8%	63,7%
4186	25	75	65	80	40,8%	41,1%	63,3%	77,1%	65,2%
4187	25	75	70	20	30,4%	39,4%	50,4%	58,0%	44,2%
4188	25	75	70	25	30,8%	39,0%	51,2%	59,7%	45,9%
4189	25	75	70	30	31,1%	38,7%	52,1%	61,3%	47,6%
4190	25	75	70	35	31,5%	38,4%	52,9%	63,0%	49,3%

4191	25	75	70	40	31,9%	38,1%	53,8%	64,6%	51,0%
4192	25	75	70	45	32,3%	37,8%	54,6%	66,1%	52,7%
4193	25	75	70	50	32,7%	37,5%	55,4%	67,6%	54,4%
4194	25	75	70	55	33,1%	37,2%	56,3%	69,1%	56,1%
4195	25	75	70	60	33,4%	36,9%	57,1%	70,6%	57,8%
4196	25	75	70	65	33,8%	36,6%	57,9%	72,0%	59,5%
4197	25	75	70	70	34,2%	36,3%	58,8%	73,4%	61,1%
4198	25	75	70	75	34,6%	36,0%	59,6%	74,7%	62,7%
4199	25	75	70	80	35,0%	35,7%	60,4%	76,0%	64,3%
4200	25	75	75	20	25,4%	34,0%	47,3%	56,5%	43,2%
4201	25	75	75	25	25,8%	33,7%	48,1%	58,2%	44,9%
4202	25	75	75	30	26,1%	33,4%	49,0%	59,9%	46,6%
4203	25	75	75	35	26,4%	33,1%	49,8%	61,5%	48,3%
4204	25	75	75	40	26,8%	32,8%	50,7%	63,1%	50,0%
4205	25	75	75	45	27,1%	32,6%	51,5%	64,7%	51,7%
4206	25	75	75	50	27,5%	32,3%	52,4%	66,3%	53,4%
4207	25	75	75	55	27,8%	32,0%	53,2%	67,8%	55,1%
4208	25	75	75	60	28,2%	31,7%	54,0%	69,3%	56,8%
4209	25	75	75	65	28,6%	31,4%	54,9%	70,8%	58,5%
4210	25	75	75	70	28,9%	31,1%	55,7%	72,2%	60,1%
4211	25	75	75	75	29,3%	30,9%	56,6%	73,5%	61,7%
4212	25	75	75	80	29,6%	30,6%	57,4%	74,8%	63,3%
4213	25	75	80	20	21,0%	29,0%	44,2%	55,0%	42,2%
4214	25	75	80	25	21,3%	28,8%	45,0%	56,7%	43,8%
4215	25	75	80	30	21,6%	28,5%	45,9%	58,4%	45,5%
4216	25	75	80	35	21,9%	28,2%	46,7%	60,1%	47,2%
4217	25	75	80	40	22,2%	28,0%	47,6%	61,7%	48,9%
4218	25	75	80	45	22,5%	27,7%	48,4%	63,3%	50,7%
4219	25	75	80	50	22,8%	27,4%	49,2%	64,9%	52,4%
4220	25	75	80	55	23,2%	27,2%	50,1%	66,5%	54,1%
4221	25	75	80	60	23,5%	26,9%	50,9%	68,0%	55,8%
4222	25	75	80	65	23,8%	26,7%	51,8%	69,5%	57,5%
4223	25	75	80	70	24,1%	26,4%	52,6%	70,9%	59,1%

4224	25	75	80	75	24,4%	26,2%	53,5%	72,3%	60,8%
4225	25	75	80	80	24,8%	25,9%	54,3%	73,7%	62,4%
4226	25	80	20	20	84,9%	87,6%	77,3%	70,3%	52,1%
4227	25	80	20	25	85,1%	87,5%	77,9%	71,7%	53,8%
4228	25	80	20	30	85,4%	87,3%	78,5%	73,1%	55,5%
4229	25	80	20	35	85,6%	87,2%	79,0%	74,4%	57,2%
4230	25	80	20	40	85,8%	87,0%	79,6%	75,7%	58,8%
4231	25	80	20	45	86,0%	86,9%	80,1%	76,9%	60,5%
4232	25	80	20	50	86,2%	86,7%	80,7%	78,1%	62,1%
4233	25	80	20	55	86,4%	86,6%	81,2%	79,3%	63,7%
4234	25	80	20	60	86,6%	86,4%	81,7%	80,4%	65,3%
4235	25	80	20	65	86,8%	86,3%	82,2%	81,5%	66,8%
4236	25	80	20	70	87,0%	86,1%	82,7%	82,5%	68,3%
4237	25	80	20	75	87,2%	86,0%	83,2%	83,5%	69,8%
4238	25	80	20	80	87,4%	85,8%	83,7%	84,4%	71,2%
4239	25	80	25	20	81,5%	84,9%	75,0%	69,0%	51,1%
4240	25	80	25	25	81,7%	84,7%	75,7%	70,4%	52,8%
4241	25	80	25	30	82,0%	84,5%	76,3%	71,8%	54,5%
4242	25	80	25	35	82,3%	84,4%	76,9%	73,2%	56,2%
4243	25	80	25	40	82,5%	84,2%	77,5%	74,5%	57,8%
4244	25	80	25	45	82,8%	84,0%	78,1%	75,8%	59,5%
4245	25	80	25	50	83,0%	83,8%	78,7%	77,1%	61,1%
4246	25	80	25	55	83,3%	83,7%	79,2%	78,3%	62,8%
4247	25	80	25	60	83,5%	83,5%	79,8%	79,4%	64,3%
4248	25	80	25	65	83,8%	83,3%	80,3%	80,5%	65,9%
4249	25	80	25	70	84,0%	83,1%	80,9%	81,6%	67,4%
4250	25	80	25	75	84,2%	82,9%	81,4%	82,6%	68,9%
4251	25	80	25	80	84,5%	82,8%	81,9%	83,6%	70,4%
4252	25	80	30	20	77,5%	81,7%	72,6%	67,6%	50,0%
4253	25	80	30	25	77,8%	81,5%	73,3%	69,1%	51,7%
4254	25	80	30	30	78,1%	81,3%	74,0%	70,6%	53,4%
4255	25	80	30	35	78,4%	81,1%	74,6%	72,0%	55,1%
4256	25	80	30	40	78,7%	80,9%	75,3%	73,4%	56,8%

4257	25	80	30	45	79,0%	80,7%	75,9%	74,7%	58,5%
4258	25	80	30	50	79,3%	80,5%	76,5%	76,0%	60,2%
4259	25	80	30	55	79,5%	80,3%	77,1%	77,2%	61,8%
4260	25	80	30	60	79,8%	80,1%	77,7%	78,4%	63,4%
4261	25	80	30	65	80,1%	79,8%	78,3%	79,5%	65,0%
4262	25	80	30	70	80,4%	79,6%	78,9%	80,6%	66,5%
4263	25	80	30	75	80,7%	79,4%	79,4%	81,7%	68,0%
4264	25	80	30	80	80,9%	79,2%	80,0%	82,7%	69,5%
4265	25	80	35	20	72,9%	78,0%	70,1%	66,3%	49,0%
4266	25	80	35	25	73,2%	77,7%	70,8%	67,8%	50,7%
4267	25	80	35	30	73,6%	77,5%	71,5%	69,3%	52,4%
4268	25	80	35	35	73,9%	77,3%	72,2%	70,8%	54,1%
4269	25	80	35	40	74,2%	77,0%	72,9%	72,2%	55,8%
4270	25	80	35	45	74,6%	76,8%	73,5%	73,5%	57,5%
4271	25	80	35	50	74,9%	76,6%	74,2%	74,8%	59,2%
4272	25	80	35	55	75,2%	76,3%	74,8%	76,1%	60,8%
4273	25	80	35	60	75,6%	76,1%	75,5%	77,3%	62,4%
4274	25	80	35	65	75,9%	75,9%	76,1%	78,5%	64,0%
4275	25	80	35	70	76,2%	75,6%	76,7%	79,7%	65,6%
4276	25	80	35	75	76,5%	75,4%	77,3%	80,8%	67,1%
4277	25	80	35	80	76,8%	75,1%	77,9%	81,8%	68,6%
4278	25	80	40	20	67,7%	73,7%	67,4%	64,9%	48,0%
4279	25	80	40	25	68,1%	73,5%	68,2%	66,5%	49,7%
4280	25	80	40	30	68,5%	73,2%	68,9%	68,0%	51,4%
4281	25	80	40	35	68,9%	73,0%	69,6%	69,5%	53,1%
4282	25	80	40	40	69,2%	72,7%	70,4%	70,9%	54,8%
4283	25	80	40	45	69,6%	72,4%	71,1%	72,3%	56,5%
4284	25	80	40	50	70,0%	72,2%	71,7%	73,7%	58,2%
4285	25	80	40	55	70,4%	71,9%	72,4%	75,0%	59,8%
4286	25	80	40	60	70,7%	71,7%	73,1%	76,3%	61,5%
4287	25	80	40	65	71,1%	71,4%	73,8%	77,5%	63,1%
4288	25	80	40	70	71,4%	71,1%	74,4%	78,7%	64,6%
4289	25	80	40	75	71,8%	70,9%	75,1%	79,8%	66,2%

4290	25	80	40	80	72,2%	70,6%	75,7%	80,9%	67,7%
4291	25	80	45	20	62,1%	69,0%	64,7%	63,5%	46,9%
4292	25	80	45	25	62,5%	68,7%	65,4%	65,1%	48,6%
4293	25	80	45	30	62,9%	68,5%	66,2%	66,6%	50,4%
4294	25	80	45	35	63,3%	68,2%	66,9%	68,2%	52,1%
4295	25	80	45	40	63,8%	67,9%	67,7%	69,6%	53,8%
4296	25	80	45	45	64,2%	67,6%	68,4%	71,1%	55,5%
4297	25	80	45	50	64,6%	67,3%	69,2%	72,5%	57,2%
4298	25	80	45	55	65,0%	67,0%	69,9%	73,8%	58,8%
4299	25	80	45	60	65,4%	66,7%	70,6%	75,1%	60,5%
4300	25	80	45	65	65,8%	66,5%	71,3%	76,4%	62,1%
4301	25	80	45	70	66,2%	66,2%	72,0%	77,6%	63,7%
4302	25	80	45	75	66,6%	65,9%	72,7%	78,8%	65,3%
4303	25	80	45	80	66,9%	65,6%	73,3%	79,9%	66,8%
4304	25	80	50	20	56,2%	63,9%	61,8%	62,1%	45,9%
4305	25	80	50	25	56,6%	63,6%	62,6%	63,7%	47,6%
4306	25	80	50	30	57,0%	63,3%	63,4%	65,3%	49,3%
4307	25	80	50	35	57,4%	63,0%	64,1%	66,8%	51,0%
4308	25	80	50	40	57,9%	62,7%	64,9%	68,3%	52,7%
4309	25	80	50	45	58,3%	62,4%	65,7%	69,8%	54,4%
4310	25	80	50	50	58,7%	62,1%	66,5%	71,2%	56,1%
4311	25	80	50	55	59,2%	61,7%	67,2%	72,6%	57,8%
4312	25	80	50	60	59,6%	61,4%	67,9%	74,0%	59,5%
4313	25	80	50	65	60,0%	61,1%	68,7%	75,3%	61,1%
4314	25	80	50	70	60,4%	60,8%	69,4%	76,6%	62,7%
4315	25	80	50	75	60,9%	60,5%	70,1%	77,8%	64,3%
4316	25	80	50	80	61,3%	60,2%	70,8%	78,9%	65,9%
4317	25	80	55	20	50,0%	58,4%	58,8%	60,6%	44,9%
4318	25	80	55	25	50,4%	58,1%	59,6%	62,3%	46,6%
4319	25	80	55	30	50,9%	57,8%	60,4%	63,9%	48,3%
4320	25	80	55	35	51,3%	57,4%	61,2%	65,5%	50,0%
4321	25	80	55	40	51,8%	57,1%	62,0%	67,0%	51,7%
4322	25	80	55	45	52,2%	56,8%	62,8%	68,5%	53,4%

4323	25	80	55	50	52,7%	56,5%	63,6%	70,0%	55,1%
4324	25	80	55	55	53,1%	56,2%	64,4%	71,4%	56,8%
4325	25	80	55	60	53,5%	55,8%	65,2%	72,8%	58,5%
4326	25	80	55	65	54,0%	55,5%	66,0%	74,1%	60,1%
4327	25	80	55	70	54,4%	55,2%	66,7%	75,4%	61,8%
4328	25	80	55	75	54,8%	54,9%	67,5%	76,7%	63,4%
4329	25	80	55	80	55,3%	54,5%	68,2%	77,9%	64,9%
4330	25	80	60	20	43,9%	52,7%	55,8%	59,2%	43,9%
4331	25	80	60	25	44,3%	52,4%	56,6%	60,8%	45,6%
4332	25	80	60	30	44,7%	52,1%	57,4%	62,5%	47,3%
4333	25	80	60	35	45,2%	51,7%	58,3%	64,1%	49,0%
4334	25	80	60	40	45,6%	51,4%	59,1%	65,6%	50,7%
4335	25	80	60	45	46,0%	51,1%	59,9%	67,2%	52,4%
4336	25	80	60	50	46,5%	50,8%	60,7%	68,7%	54,1%
4337	25	80	60	55	46,9%	50,4%	61,5%	70,1%	55,8%
4338	25	80	60	60	47,4%	50,1%	62,3%	71,6%	57,5%
4339	25	80	60	65	47,8%	49,8%	63,1%	73,0%	59,1%
4340	25	80	60	70	48,2%	49,4%	63,9%	74,3%	60,8%
4341	25	80	60	75	48,7%	49,1%	64,7%	75,6%	62,4%
4342	25	80	60	80	49,1%	48,8%	65,4%	76,8%	64,0%
4343	25	80	65	20	37,9%	46,9%	52,7%	57,7%	42,9%
4344	25	80	65	25	38,3%	46,6%	53,5%	59,4%	44,5%
4345	25	80	65	30	38,7%	46,3%	54,4%	61,0%	46,2%
4346	25	80	65	35	39,2%	46,0%	55,2%	62,7%	47,9%
4347	25	80	65	40	39,6%	45,6%	56,0%	64,3%	49,7%
4348	25	80	65	45	40,0%	45,3%	56,9%	65,8%	51,4%
4349	25	80	65	50	40,4%	45,0%	57,7%	67,4%	53,1%
4350	25	80	65	55	40,9%	44,7%	58,5%	68,9%	54,8%
4351	25	80	65	60	41,3%	44,3%	59,4%	70,3%	56,5%
4352	25	80	65	65	41,7%	44,0%	60,2%	71,7%	58,1%
4353	25	80	65	70	42,1%	43,7%	61,0%	73,1%	59,8%
4354	25	80	65	75	42,6%	43,4%	61,8%	74,4%	61,4%
4355	25	80	65	80	43,0%	43,1%	62,6%	75,7%	63,0%

4356	25	80	70	20	32,3%	41,3%	49,6%	56,2%	41,8%
4357	25	80	70	25	32,7%	40,9%	50,4%	57,9%	43,5%
4358	25	80	70	30	33,1%	40,6%	51,3%	59,6%	45,2%
4359	25	80	70	35	33,5%	40,3%	52,1%	61,2%	46,9%
4360	25	80	70	40	33,9%	40,0%	53,0%	62,8%	48,6%
4361	25	80	70	45	34,2%	39,7%	53,8%	64,4%	50,3%
4362	25	80	70	50	34,6%	39,4%	54,7%	66,0%	52,0%
4363	25	80	70	55	35,0%	39,1%	55,5%	67,5%	53,8%
4364	25	80	70	60	35,4%	38,7%	56,3%	69,0%	55,4%
4365	25	80	70	65	35,9%	38,4%	57,2%	70,5%	57,1%
4366	25	80	70	70	36,3%	38,1%	58,0%	71,9%	58,8%
4367	25	80	70	75	36,7%	37,8%	58,8%	73,3%	60,5%
4368	25	80	70	80	37,1%	37,5%	59,6%	74,6%	62,1%
4369	25	80	75	20	27,1%	35,8%	46,5%	54,7%	40,8%
4370	25	80	75	25	27,5%	35,5%	47,3%	56,4%	42,5%
4371	25	80	75	30	27,8%	35,2%	48,2%	58,1%	44,2%
4372	25	80	75	35	28,2%	34,9%	49,0%	59,8%	45,9%
4373	25	80	75	40	28,6%	34,6%	49,9%	61,4%	47,6%
4374	25	80	75	45	28,9%	34,3%	50,7%	63,0%	49,3%
4375	25	80	75	50	29,3%	34,0%	51,6%	64,6%	51,0%
4376	25	80	75	55	29,7%	33,7%	52,4%	66,2%	52,7%
4377	25	80	75	60	30,0%	33,4%	53,3%	67,7%	54,4%
4378	25	80	75	65	30,4%	33,1%	54,1%	69,2%	56,1%
4379	25	80	75	70	30,8%	32,8%	54,9%	70,6%	57,8%
4380	25	80	75	75	31,1%	32,6%	55,8%	72,1%	59,5%
4381	25	80	75	80	31,5%	32,3%	56,6%	73,4%	61,1%
4382	25	80	80	20	22,5%	30,7%	43,4%	53,2%	39,9%
4383	25	80	80	25	22,9%	30,4%	44,2%	54,9%	41,5%
4384	25	80	80	30	23,2%	30,1%	45,1%	56,6%	43,2%
4385	25	80	80	35	23,5%	29,8%	45,9%	58,3%	44,9%
4386	25	80	80	40	23,8%	29,6%	46,8%	60,0%	46,6%
4387	25	80	80	45	24,1%	29,3%	47,6%	61,6%	48,3%
4388	25	80	80	50	24,4%	29,0%	48,5%	63,2%	50,0%

4389	25	80	80	55	24,8%	28,8%	49,3%	64,8%	51,7%
4390	25	80	80	60	25,1%	28,5%	50,2%	66,4%	53,4%
4391	25	80	80	65	25,4%	28,2%	51,0%	67,9%	55,1%
4392	25	80	80	70	25,8%	28,0%	51,8%	69,4%	56,8%
4393	25	80	80	75	26,1%	27,7%	52,7%	70,8%	58,5%
4394	25	80	80	80	26,5%	27,4%	53,5%	72,2%	60,1%
4395	30	20	20	20	71,3%	79,3%	88,3%	89,6%	82,2%
4396	30	20	20	25	71,6%	79,1%	88,7%	90,2%	83,2%
4397	30	20	20	30	72,0%	78,9%	89,0%	90,8%	84,1%
4398	30	20	20	35	72,4%	78,7%	89,4%	91,4%	85,0%
4399	30	20	20	40	72,7%	78,5%	89,7%	91,9%	85,9%
4400	30	20	20	45	73,1%	78,2%	90,0%	92,4%	86,7%
4401	30	20	20	50	73,4%	78,0%	90,3%	92,9%	87,4%
4402	30	20	20	55	73,7%	77,8%	90,6%	93,3%	88,2%
4403	30	20	20	60	74,1%	77,6%	90,9%	93,7%	88,9%
4404	30	20	20	65	74,4%	77,3%	91,1%	94,1%	89,5%
4405	30	20	20	70	74,8%	77,1%	91,4%	94,5%	90,2%
4406	30	20	20	75	75,1%	76,9%	91,7%	94,8%	90,7%
4407	30	20	20	80	75,4%	76,6%	91,9%	95,2%	91,3%
4408	30	20	25	20	66,0%	75,3%	87,0%	89,0%	81,6%
4409	30	20	25	25	66,4%	75,0%	87,4%	89,7%	82,6%
4410	30	20	25	30	66,8%	74,8%	87,8%	90,3%	83,6%
4411	30	20	25	35	67,2%	74,6%	88,1%	90,9%	84,5%
4412	30	20	25	40	67,5%	74,3%	88,5%	91,4%	85,4%
4413	30	20	25	45	67,9%	74,1%	88,8%	91,9%	86,2%
4414	30	20	25	50	68,3%	73,8%	89,1%	92,4%	87,0%
4415	30	20	25	55	68,7%	73,6%	89,5%	92,9%	87,7%
4416	30	20	25	60	69,1%	73,3%	89,8%	93,4%	88,5%
4417	30	20	25	65	69,4%	73,0%	90,1%	93,8%	89,1%
4418	30	20	25	70	69,8%	72,8%	90,4%	94,2%	89,8%
4419	30	20	25	75	70,2%	72,5%	90,7%	94,5%	90,4%
4420	30	20	25	80	70,6%	72,3%	91,0%	94,9%	91,0%
4421	30	20	30	20	60,2%	70,8%	85,5%	88,4%	81,0%

4422	30	20	30	25	60,7%	70,5%	85,9%	89,1%	82,0%
4423	30	20	30	30	61,1%	70,2%	86,4%	89,7%	83,0%
4424	30	20	30	35	61,5%	69,9%	86,7%	90,3%	83,9%
4425	30	20	30	40	61,9%	69,7%	87,1%	90,9%	84,8%
4426	30	20	30	45	62,3%	69,4%	87,5%	91,5%	85,7%
4427	30	20	30	50	62,7%	69,1%	87,9%	92,0%	86,5%
4428	30	20	30	55	63,2%	68,8%	88,2%	92,5%	87,3%
4429	30	20	30	60	63,6%	68,5%	88,6%	93,0%	88,0%
4430	30	20	30	65	64,0%	68,3%	88,9%	93,4%	88,7%
4431	30	20	30	70	64,4%	68,0%	89,2%	93,8%	89,4%
4432	30	20	30	75	64,8%	67,7%	89,6%	94,2%	90,0%
4433	30	20	30	80	65,2%	67,4%	89,9%	94,6%	90,6%
4434	30	20	35	20	54,2%	65,8%	83,9%	87,7%	80,3%
4435	30	20	35	25	54,6%	65,5%	84,4%	88,5%	81,4%
4436	30	20	35	30	55,1%	65,2%	84,8%	89,1%	82,4%
4437	30	20	35	35	55,5%	64,9%	85,3%	89,8%	83,4%
4438	30	20	35	40	55,9%	64,6%	85,7%	90,4%	84,3%
4439	30	20	35	45	56,4%	64,3%	86,1%	91,0%	85,2%
4440	30	20	35	50	56,8%	64,0%	86,5%	91,5%	86,0%
4441	30	20	35	55	57,2%	63,7%	86,9%	92,1%	86,8%
4442	30	20	35	60	57,7%	63,4%	87,3%	92,6%	87,6%
4443	30	20	35	65	58,1%	63,1%	87,6%	93,0%	88,3%
4444	30	20	35	70	58,5%	62,8%	88,0%	93,4%	89,0%
4445	30	20	35	75	59,0%	62,4%	88,4%	93,9%	89,7%
4446	30	20	35	80	59,4%	62,1%	88,7%	94,2%	90,3%
4447	30	20	40	20	48,0%	60,4%	82,2%	87,1%	79,7%
4448	30	20	40	25	48,5%	60,1%	82,7%	87,8%	80,7%
4449	30	20	40	30	48,9%	59,8%	83,2%	88,5%	81,8%
4450	30	20	40	35	49,4%	59,4%	83,6%	89,2%	82,8%
4451	30	20	40	40	49,8%	59,1%	84,1%	89,9%	83,7%
4452	30	20	40	45	50,2%	58,8%	84,5%	90,5%	84,6%
4453	30	20	40	50	50,7%	58,5%	85,0%	91,1%	85,5%
4454	30	20	40	55	51,1%	58,2%	85,4%	91,6%	86,3%

4455	30	20	40	60	51,6%	57,9%	85,8%	92,1%	87,1%
4456	30	20	40	65	52,0%	57,5%	86,2%	92,6%	87,9%
4457	30	20	40	70	52,4%	57,2%	86,6%	93,1%	88,6%
4458	30	20	40	75	52,9%	56,9%	87,0%	93,5%	89,3%
4459	30	20	40	80	53,3%	56,6%	87,4%	93,9%	89,9%
4460	30	20	45	20	41,9%	54,8%	80,3%	86,4%	79,0%
4461	30	20	45	25	42,4%	54,4%	80,8%	87,2%	80,1%
4462	30	20	45	30	42,8%	54,1%	81,3%	87,9%	81,2%
4463	30	20	45	35	43,2%	53,8%	81,8%	88,6%	82,2%
4464	30	20	45	40	43,7%	53,5%	82,3%	89,3%	83,2%
4465	30	20	45	45	44,1%	53,1%	82,8%	89,9%	84,1%
4466	30	20	45	50	44,5%	52,8%	83,3%	90,6%	85,0%
4467	30	20	45	55	45,0%	52,5%	83,8%	91,1%	85,9%
4468	30	20	45	60	45,4%	52,1%	84,2%	91,7%	86,7%
4469	30	20	45	65	45,8%	51,8%	84,7%	92,2%	87,4%
4470	30	20	45	70	46,3%	51,5%	85,1%	92,7%	88,2%
4471	30	20	45	75	46,7%	51,2%	85,5%	93,1%	88,9%
4472	30	20	45	80	47,2%	50,8%	86,0%	93,5%	89,5%
4473	30	20	50	20	36,1%	49,0%	78,2%	85,6%	78,3%
4474	30	20	50	25	36,5%	48,7%	78,8%	86,5%	79,4%
4475	30	20	50	30	36,9%	48,3%	79,4%	87,2%	80,5%
4476	30	20	50	35	37,3%	48,0%	79,9%	88,0%	81,6%
4477	30	20	50	40	37,7%	47,7%	80,5%	88,7%	82,6%
4478	30	20	50	45	38,1%	47,4%	81,0%	89,4%	83,5%
4479	30	20	50	50	38,5%	47,0%	81,5%	90,0%	84,5%
4480	30	20	50	55	39,0%	46,7%	82,0%	90,6%	85,3%
4481	30	20	50	60	39,4%	46,4%	82,5%	91,2%	86,2%
4482	30	20	50	65	39,8%	46,1%	83,0%	91,7%	87,0%
4483	30	20	50	70	40,2%	45,7%	83,5%	92,2%	87,7%
4484	30	20	50	75	40,7%	45,4%	83,9%	92,7%	88,4%
4485	30	20	50	80	41,1%	45,1%	84,4%	93,2%	89,1%
4486	30	20	55	20	30,6%	43,3%	76,1%	84,9%	77,6%
4487	30	20	55	25	31,0%	42,9%	76,7%	85,7%	78,7%

4488	30	20	55	30	31,4%	42,6%	77,3%	86,6%	79,9%
4489	30	20	55	35	31,7%	42,3%	77,9%	87,3%	80,9%
4490	30	20	55	40	32,1%	42,0%	78,4%	88,1%	82,0%
4491	30	20	55	45	32,5%	41,7%	79,0%	88,8%	83,0%
4492	30	20	55	50	32,9%	41,3%	79,6%	89,5%	83,9%
4493	30	20	55	55	33,3%	41,0%	80,1%	90,1%	84,8%
4494	30	20	55	60	33,7%	40,7%	80,7%	90,7%	85,7%
4495	30	20	55	65	34,1%	40,4%	81,2%	91,3%	86,5%
4496	30	20	55	70	34,5%	40,1%	81,7%	91,8%	87,3%
4497	30	20	55	75	34,9%	39,8%	82,2%	92,3%	88,0%
4498	30	20	55	80	35,3%	39,5%	82,7%	92,8%	88,7%
4499	30	20	60	20	25,6%	37,7%	73,7%	84,1%	76,8%
4500	30	20	60	25	26,0%	37,4%	74,4%	85,0%	78,0%
4501	30	20	60	30	26,3%	37,1%	75,0%	85,8%	79,2%
4502	30	20	60	35	26,6%	36,8%	75,6%	86,6%	80,3%
4503	30	20	60	40	27,0%	36,5%	76,3%	87,4%	81,4%
4504	30	20	60	45	27,3%	36,2%	76,9%	88,2%	82,4%
4505	30	20	60	50	27,7%	35,9%	77,5%	88,9%	83,4%
4506	30	20	60	55	28,0%	35,6%	78,1%	89,5%	84,3%
4507	30	20	60	60	28,4%	35,3%	78,6%	90,2%	85,2%
4508	30	20	60	65	28,8%	35,0%	79,2%	90,8%	86,0%
4509	30	20	60	70	29,1%	34,7%	79,8%	91,3%	86,8%
4510	30	20	60	75	29,5%	34,4%	80,3%	91,8%	87,6%
4511	30	20	60	80	29,9%	34,1%	80,8%	92,4%	88,3%
4512	30	20	65	20	21,2%	32,4%	71,2%	83,2%	76,1%
4513	30	20	65	25	21,5%	32,2%	71,9%	84,2%	77,3%
4514	30	20	65	30	21,8%	31,9%	72,6%	85,1%	78,5%
4515	30	20	65	35	22,1%	31,6%	73,3%	85,9%	79,6%
4516	30	20	65	40	22,4%	31,3%	73,9%	86,7%	80,7%
4517	30	20	65	45	22,7%	31,0%	74,6%	87,5%	81,8%
4518	30	20	65	50	23,0%	30,8%	75,2%	88,2%	82,8%
4519	30	20	65	55	23,3%	30,5%	75,9%	88,9%	83,7%
4520	30	20	65	60	23,7%	30,2%	76,5%	89,6%	84,6%

4521	30	20	65	65	24,0%	29,9%	77,1%	90,2%	85,5%
4522	30	20	65	70	24,3%	29,7%	77,7%	90,8%	86,3%
4523	30	20	65	75	24,6%	29,4%	78,3%	91,4%	87,1%
4524	30	20	65	80	24,9%	29,1%	78,8%	91,9%	87,9%
4525	30	20	70	20	17,4%	27,6%	68,6%	82,4%	75,3%
4526	30	20	70	25	17,6%	27,3%	69,4%	83,3%	76,6%
4527	30	20	70	30	17,9%	27,1%	70,1%	84,3%	77,8%
4528	30	20	70	35	18,1%	26,8%	70,8%	85,2%	79,0%
4529	30	20	70	40	18,4%	26,6%	71,5%	86,0%	80,1%
4530	30	20	70	45	18,7%	26,3%	72,2%	86,8%	81,1%
4531	30	20	70	50	18,9%	26,1%	72,8%	87,6%	82,2%
4532	30	20	70	55	19,2%	25,8%	73,5%	88,3%	83,2%
4533	30	20	70	60	19,5%	25,6%	74,2%	89,0%	84,1%
4534	30	20	70	65	19,8%	25,3%	74,8%	89,7%	85,0%
4535	30	20	70	70	20,0%	25,1%	75,4%	90,3%	85,8%
4536	30	20	70	75	20,3%	24,8%	76,1%	90,9%	86,7%
4537	30	20	70	80	20,6%	24,6%	76,7%	91,4%	87,4%
4538	30	20	75	20	14,1%	23,2%	65,9%	81,5%	74,6%
4539	30	20	75	25	14,3%	23,0%	66,7%	82,5%	75,9%
4540	30	20	75	30	14,5%	22,8%	67,4%	83,5%	77,1%
4541	30	20	75	35	14,8%	22,5%	68,2%	84,4%	78,3%
4542	30	20	75	40	15,0%	22,3%	68,9%	85,3%	79,4%
4543	30	20	75	45	15,2%	22,1%	69,6%	86,1%	80,5%
4544	30	20	75	50	15,4%	21,9%	70,3%	86,9%	81,6%
4545	30	20	75	55	15,7%	21,6%	71,0%	87,7%	82,6%
4546	30	20	75	60	15,9%	21,4%	71,7%	88,4%	83,5%
4547	30	20	75	65	16,1%	21,2%	72,4%	89,1%	84,5%
4548	30	20	75	70	16,4%	21,0%	73,1%	89,8%	85,3%
4549	30	20	75	75	16,6%	20,8%	73,7%	90,4%	86,2%
4550	30	20	75	80	16,9%	20,6%	74,4%	91,0%	87,0%
4551	30	20	80	20	11,4%	19,4%	63,1%	80,5%	73,8%
4552	30	20	80	25	11,5%	19,2%	63,8%	81,6%	75,1%
4553	30	20	80	30	11,7%	19,0%	64,6%	82,6%	76,3%

4554	30	20	80	35	11,9%	18,8%	65,4%	83,6%	77,6%
4555	30	20	80	40	12,1%	18,6%	66,2%	84,5%	78,7%
4556	30	20	80	45	12,3%	18,4%	66,9%	85,4%	79,9%
4557	30	20	80	50	12,5%	18,2%	67,7%	86,2%	80,9%
4558	30	20	80	55	12,7%	18,0%	68,4%	87,0%	82,0%
4559	30	20	80	60	12,9%	17,8%	69,1%	87,8%	83,0%
4560	30	20	80	65	13,1%	17,6%	69,9%	88,5%	83,9%
4561	30	20	80	70	13,3%	17,4%	70,6%	89,2%	84,8%
4562	30	20	80	75	13,5%	17,2%	71,3%	89,8%	85,7%
4563	30	20	80	80	13,7%	17,0%	72,0%	90,4%	86,5%
4564	30	25	20	20	73,1%	80,6%	88,0%	88,9%	80,8%
4565	30	25	20	25	73,4%	80,4%	88,4%	89,5%	81,8%
4566	30	25	20	30	73,8%	80,2%	88,7%	90,2%	82,8%
4567	30	25	20	35	74,1%	80,0%	89,0%	90,8%	83,7%
4568	30	25	20	40	74,4%	79,8%	89,4%	91,3%	84,7%
4569	30	25	20	45	74,8%	79,6%	89,7%	91,8%	85,5%
4570	30	25	20	50	75,1%	79,3%	90,0%	92,3%	86,4%
4571	30	25	20	55	75,4%	79,1%	90,3%	92,8%	87,1%
4572	30	25	20	60	75,7%	78,9%	90,6%	93,3%	87,9%
4573	30	25	20	65	76,1%	78,7%	90,9%	93,7%	88,6%
4574	30	25	20	70	76,4%	78,5%	91,2%	94,1%	89,3%
4575	30	25	20	75	76,7%	78,2%	91,4%	94,5%	89,9%
4576	30	25	20	80	77,0%	78,0%	91,7%	94,8%	90,5%
4577	30	25	25	20	67,9%	76,7%	86,6%	88,2%	80,1%
4578	30	25	25	25	68,3%	76,5%	87,0%	88,9%	81,2%
4579	30	25	25	30	68,7%	76,3%	87,4%	89,6%	82,2%
4580	30	25	25	35	69,1%	76,0%	87,8%	90,2%	83,2%
4581	30	25	25	40	69,5%	75,8%	88,1%	90,8%	84,1%
4582	30	25	25	45	69,8%	75,5%	88,5%	91,4%	85,0%
4583	30	25	25	50	70,2%	75,3%	88,8%	91,9%	85,9%
4584	30	25	25	55	70,6%	75,1%	89,2%	92,4%	86,7%
4585	30	25	25	60	70,9%	74,8%	89,5%	92,9%	87,4%
4586	30	25	25	65	71,3%	74,6%	89,8%	93,3%	88,2%

4587	30	25	25	70	71,7%	74,3%	90,1%	93,7%	88,9%
4588	30	25	25	75	72,0%	74,1%	90,4%	94,1%	89,5%
4589	30	25	25	80	72,4%	73,8%	90,7%	94,5%	90,2%
4590	30	25	30	20	62,3%	72,4%	85,1%	87,6%	79,4%
4591	30	25	30	25	62,8%	72,1%	85,6%	88,3%	80,5%
4592	30	25	30	30	63,2%	71,8%	86,0%	89,0%	81,6%
4593	30	25	30	35	63,6%	71,6%	86,4%	89,7%	82,6%
4594	30	25	30	40	64,0%	71,3%	86,8%	90,3%	83,6%
4595	30	25	30	45	64,4%	71,0%	87,2%	90,9%	84,5%
4596	30	25	30	50	64,8%	70,8%	87,5%	91,4%	85,4%
4597	30	25	30	55	65,2%	70,5%	87,9%	92,0%	86,2%
4598	30	25	30	60	65,6%	70,2%	88,3%	92,5%	87,0%
4599	30	25	30	65	66,0%	69,9%	88,6%	92,9%	87,7%
4600	30	25	30	70	66,4%	69,7%	88,9%	93,4%	88,5%
4601	30	25	30	75	66,8%	69,4%	89,3%	93,8%	89,1%
4602	30	25	30	80	67,2%	69,1%	89,6%	94,2%	89,8%
4603	30	25	35	20	56,4%	67,5%	83,5%	86,9%	78,8%
4604	30	25	35	25	56,8%	67,2%	84,0%	87,7%	79,9%
4605	30	25	35	30	57,3%	66,9%	84,4%	88,4%	81,0%
4606	30	25	35	35	57,7%	66,6%	84,8%	89,1%	82,0%
4607	30	25	35	40	58,1%	66,3%	85,3%	89,8%	83,0%
4608	30	25	35	45	58,6%	66,1%	85,7%	90,4%	83,9%
4609	30	25	35	50	59,0%	65,8%	86,1%	91,0%	84,8%
4610	30	25	35	55	59,4%	65,5%	86,5%	91,5%	85,7%
4611	30	25	35	60	59,8%	65,2%	86,9%	92,0%	86,5%
4612	30	25	35	65	60,3%	64,9%	87,3%	92,5%	87,3%
4613	30	25	35	70	60,7%	64,6%	87,7%	93,0%	88,0%
4614	30	25	35	75	61,1%	64,3%	88,0%	93,4%	88,7%
4615	30	25	35	80	61,5%	64,0%	88,4%	93,8%	89,4%
4616	30	25	40	20	50,3%	62,3%	81,7%	86,2%	78,1%
4617	30	25	40	25	50,7%	61,9%	82,2%	87,0%	79,2%
4618	30	25	40	30	51,1%	61,6%	82,7%	87,8%	80,3%
4619	30	25	40	35	51,6%	61,3%	83,2%	88,5%	81,4%

4620	30	25	40	40	52,0%	61,0%	83,7%	89,2%	82,4%
4621	30	25	40	45	52,5%	60,7%	84,1%	89,8%	83,4%
4622	30	25	40	50	52,9%	60,4%	84,6%	90,4%	84,3%
4623	30	25	40	55	53,3%	60,1%	85,0%	91,0%	85,2%
4624	30	25	40	60	53,8%	59,8%	85,4%	91,6%	86,0%
4625	30	25	40	65	54,2%	59,4%	85,8%	92,1%	86,8%
4626	30	25	40	70	54,7%	59,1%	86,3%	92,6%	87,6%
4627	30	25	40	75	55,1%	58,8%	86,6%	93,0%	88,3%
4628	30	25	40	80	55,5%	58,5%	87,0%	93,5%	89,0%
4629	30	25	45	20	44,1%	56,7%	79,8%	85,5%	77,3%
4630	30	25	45	25	44,5%	56,4%	80,3%	86,3%	78,5%
4631	30	25	45	30	45,0%	56,0%	80,9%	87,1%	79,7%
4632	30	25	45	35	45,4%	55,7%	81,4%	87,9%	80,7%
4633	30	25	45	40	45,9%	55,4%	81,9%	88,6%	81,8%
4634	30	25	45	45	46,3%	55,1%	82,4%	89,3%	82,8%
4635	30	25	45	50	46,7%	54,8%	82,9%	89,9%	83,7%
4636	30	25	45	55	47,2%	54,4%	83,3%	90,5%	84,7%
4637	30	25	45	60	47,6%	54,1%	83,8%	91,1%	85,5%
4638	30	25	45	65	48,1%	53,8%	84,3%	91,6%	86,3%
4639	30	25	45	70	48,5%	53,5%	84,7%	92,1%	87,1%
4640	30	25	45	75	48,9%	53,1%	85,1%	92,6%	87,9%
4641	30	25	45	80	49,4%	52,8%	85,6%	93,1%	88,6%
4642	30	25	50	20	38,1%	51,0%	77,7%	84,7%	76,6%
4643	30	25	50	25	38,6%	50,6%	78,3%	85,6%	77,8%
4644	30	25	50	30	39,0%	50,3%	78,9%	86,4%	79,0%
4645	30	25	50	35	39,4%	50,0%	79,4%	87,2%	80,1%
4646	30	25	50	40	39,8%	49,7%	80,0%	87,9%	81,2%
4647	30	25	50	45	40,2%	49,3%	80,5%	88,7%	82,2%
4648	30	25	50	50	40,7%	49,0%	81,0%	89,3%	83,2%
4649	30	25	50	55	41,1%	48,7%	81,5%	90,0%	84,1%
4650	30	25	50	60	41,5%	48,3%	82,1%	90,6%	85,0%
4651	30	25	50	65	42,0%	48,0%	82,5%	91,1%	85,9%
4652	30	25	50	70	42,4%	47,7%	83,0%	91,7%	86,7%

4653	30	25	50	75	42,8%	47,4%	83,5%	92,2%	87,4%
4654	30	25	50	80	43,2%	47,0%	84,0%	92,7%	88,2%
4655	30	25	55	20	32,5%	45,2%	75,5%	83,9%	75,9%
4656	30	25	55	25	32,9%	44,9%	76,1%	84,8%	77,1%
4657	30	25	55	30	33,3%	44,6%	76,7%	85,7%	78,3%
4658	30	25	55	35	33,7%	44,2%	77,3%	86,5%	79,4%
4659	30	25	55	40	34,1%	43,9%	77,9%	87,3%	80,5%
4660	30	25	55	45	34,5%	43,6%	78,5%	88,0%	81,6%
4661	30	25	55	50	34,9%	43,3%	79,0%	88,7%	82,6%
4662	30	25	55	55	35,3%	42,9%	79,6%	89,4%	83,5%
4663	30	25	55	60	35,7%	42,6%	80,1%	90,0%	84,5%
4664	30	25	55	65	36,1%	42,3%	80,7%	90,6%	85,3%
4665	30	25	55	70	36,5%	42,0%	81,2%	91,2%	86,2%
4666	30	25	55	75	36,9%	41,7%	81,7%	91,7%	87,0%
4667	30	25	55	80	37,3%	41,3%	82,2%	92,3%	87,7%
4668	30	25	60	20	27,3%	39,6%	73,1%	83,1%	75,1%
4669	30	25	60	25	27,7%	39,3%	73,8%	84,0%	76,4%
4670	30	25	60	30	28,1%	38,9%	74,4%	84,9%	77,6%
4671	30	25	60	35	28,4%	38,6%	75,1%	85,8%	78,7%
4672	30	25	60	40	28,8%	38,3%	75,7%	86,6%	79,9%
4673	30	25	60	45	29,1%	38,0%	76,3%	87,4%	80,9%
4674	30	25	60	50	29,5%	37,7%	76,9%	88,1%	82,0%
4675	30	25	60	55	29,9%	37,4%	77,5%	88,8%	83,0%
4676	30	25	60	60	30,2%	37,1%	78,1%	89,5%	83,9%
4677	30	25	60	65	30,6%	36,8%	78,7%	90,1%	84,8%
4678	30	25	60	70	31,0%	36,5%	79,2%	90,7%	85,7%
4679	30	25	60	75	31,4%	36,2%	79,8%	91,3%	86,5%
4680	30	25	60	80	31,7%	35,9%	80,3%	91,8%	87,3%
4681	30	25	65	20	22,7%	34,2%	70,6%	82,2%	74,3%
4682	30	25	65	25	23,0%	33,9%	71,3%	83,2%	75,6%
4683	30	25	65	30	23,3%	33,6%	72,0%	84,1%	76,9%
4684	30	25	65	35	23,7%	33,3%	72,7%	85,0%	78,0%
4685	30	25	65	40	24,0%	33,0%	73,3%	85,9%	79,2%

4686	30	25	65	45	24,3%	32,7%	74,0%	86,7%	80,3%
4687	30	25	65	50	24,6%	32,5%	74,6%	87,5%	81,4%
4688	30	25	65	55	25,0%	32,2%	75,3%	88,2%	82,4%
4689	30	25	65	60	25,3%	31,9%	75,9%	88,9%	83,4%
4690	30	25	65	65	25,6%	31,6%	76,5%	89,6%	84,3%
4691	30	25	65	70	26,0%	31,3%	77,1%	90,2%	85,2%
4692	30	25	65	75	26,3%	31,0%	77,7%	90,8%	86,0%
4693	30	25	65	80	26,6%	30,8%	78,3%	91,3%	86,8%
4694	30	25	70	20	18,7%	29,2%	67,9%	81,3%	73,5%
4695	30	25	70	25	18,9%	28,9%	68,7%	82,3%	74,8%
4696	30	25	70	30	19,2%	28,7%	69,4%	83,3%	76,1%
4697	30	25	70	35	19,5%	28,4%	70,1%	84,2%	77,3%
4698	30	25	70	40	19,8%	28,1%	70,8%	85,1%	78,5%
4699	30	25	70	45	20,1%	27,9%	71,5%	86,0%	79,6%
4700	30	25	70	50	20,3%	27,6%	72,2%	86,8%	80,7%
4701	30	25	70	55	20,6%	27,4%	72,9%	87,5%	81,8%
4702	30	25	70	60	20,9%	27,1%	73,6%	88,3%	82,8%
4703	30	25	70	65	21,2%	26,8%	74,2%	89,0%	83,7%
4704	30	25	70	70	21,5%	26,6%	74,9%	89,6%	84,6%
4705	30	25	70	75	21,8%	26,3%	75,5%	90,3%	85,5%
4706	30	25	70	80	22,1%	26,1%	76,1%	90,8%	86,3%
4707	30	25	75	20	15,2%	24,7%	65,2%	80,3%	72,7%
4708	30	25	75	25	15,4%	24,4%	65,9%	81,4%	74,1%
4709	30	25	75	30	15,7%	24,2%	66,7%	82,4%	75,4%
4710	30	25	75	35	15,9%	23,9%	67,5%	83,4%	76,6%
4711	30	25	75	40	16,1%	23,7%	68,2%	84,3%	77,8%
4712	30	25	75	45	16,4%	23,5%	68,9%	85,2%	79,0%
4713	30	25	75	50	16,6%	23,2%	69,7%	86,1%	80,1%
4714	30	25	75	55	16,9%	23,0%	70,4%	86,9%	81,2%
4715	30	25	75	60	17,1%	22,8%	71,1%	87,6%	82,2%
4716	30	25	75	65	17,4%	22,5%	71,8%	88,4%	83,2%
4717	30	25	75	70	17,6%	22,3%	72,4%	89,1%	84,1%
4718	30	25	75	75	17,9%	22,1%	73,1%	89,7%	85,0%

4719	30	25	75	80	18,1%	21,9%	73,8%	90,3%	85,8%
4720	30	25	80	20	12,3%	20,6%	62,3%	79,3%	71,9%
4721	30	25	80	25	12,5%	20,4%	63,1%	80,4%	73,3%
4722	30	25	80	30	12,7%	20,2%	63,9%	81,5%	74,6%
4723	30	25	80	35	12,9%	20,0%	64,7%	82,5%	75,9%
4724	30	25	80	40	13,1%	19,8%	65,4%	83,5%	77,1%
4725	30	25	80	45	13,3%	19,6%	66,2%	84,4%	78,3%
4726	30	25	80	50	13,5%	19,4%	67,0%	85,3%	79,4%
4727	30	25	80	55	13,7%	19,2%	67,7%	86,2%	80,5%
4728	30	25	80	60	13,9%	19,0%	68,4%	87,0%	81,6%
4729	30	25	80	65	14,1%	18,8%	69,2%	87,7%	82,6%
4730	30	25	80	70	14,3%	18,6%	69,9%	88,4%	83,5%
4731	30	25	80	75	14,5%	18,4%	70,6%	89,1%	84,5%
4732	30	25	80	80	14,8%	18,2%	71,3%	89,8%	85,3%
4733	30	30	20	20	74,8%	81,8%	87,7%	88,1%	79,2%
4734	30	30	20	25	75,1%	81,6%	88,0%	88,8%	80,3%
4735	30	30	20	30	75,4%	81,4%	88,4%	89,5%	81,4%
4736	30	30	20	35	75,8%	81,2%	88,7%	90,1%	82,4%
4737	30	30	20	40	76,1%	81,0%	89,1%	90,7%	83,4%
4738	30	30	20	45	76,4%	80,8%	89,4%	91,3%	84,3%
4739	30	30	20	50	76,7%	80,6%	89,7%	91,8%	85,2%
4740	30	30	20	55	77,0%	80,4%	90,0%	92,3%	86,0%
4741	30	30	20	60	77,3%	80,2%	90,3%	92,8%	86,8%
4742	30	30	20	65	77,6%	80,0%	90,6%	93,2%	87,6%
4743	30	30	20	70	78,0%	79,8%	90,9%	93,7%	88,3%
4744	30	30	20	75	78,3%	79,6%	91,2%	94,1%	89,0%
4745	30	30	20	80	78,6%	79,3%	91,4%	94,4%	89,7%
4746	30	30	25	20	69,8%	78,1%	86,3%	87,5%	78,5%
4747	30	30	25	25	70,2%	77,9%	86,7%	88,2%	79,7%
4748	30	30	25	30	70,6%	77,7%	87,1%	88,9%	80,8%
4749	30	30	25	35	70,9%	77,4%	87,4%	89,6%	81,8%
4750	30	30	25	40	71,3%	77,2%	87,8%	90,2%	82,8%
4751	30	30	25	45	71,7%	77,0%	88,2%	90,8%	83,8%

4752	30	30	25	50	72,0%	76,7%	88,5%	91,3%	84,7%
4753	30	30	25	55	72,4%	76,5%	88,8%	91,9%	85,5%
4754	30	30	25	60	72,7%	76,3%	89,2%	92,4%	86,4%
4755	30	30	25	65	73,1%	76,0%	89,5%	92,8%	87,1%
4756	30	30	25	70	73,4%	75,8%	89,8%	93,3%	87,9%
4757	30	30	25	75	73,8%	75,5%	90,1%	93,7%	88,6%
4758	30	30	25	80	74,1%	75,3%	90,4%	94,1%	89,3%
4759	30	30	30	20	64,4%	73,9%	84,7%	86,8%	77,8%
4760	30	30	30	25	64,8%	73,6%	85,2%	87,5%	79,0%
4761	30	30	30	30	65,2%	73,4%	85,6%	88,3%	80,1%
4762	30	30	30	35	65,6%	73,1%	86,0%	89,0%	81,2%
4763	30	30	30	40	66,0%	72,9%	86,4%	89,6%	82,2%
4764	30	30	30	45	66,4%	72,6%	86,8%	90,3%	83,2%
4765	30	30	30	50	66,8%	72,4%	87,2%	90,8%	84,1%
4766	30	30	30	55	67,2%	72,1%	87,6%	91,4%	85,0%
4767	30	30	30	60	67,6%	71,8%	87,9%	91,9%	85,9%
4768	30	30	30	65	68,0%	71,6%	88,3%	92,4%	86,7%
4769	30	30	30	70	68,3%	71,3%	88,6%	92,9%	87,4%
4770	30	30	30	75	68,7%	71,0%	89,0%	93,3%	88,2%
4771	30	30	30	80	69,1%	70,8%	89,3%	93,8%	88,9%
4772	30	30	35	20	58,6%	69,2%	83,0%	86,1%	77,1%
4773	30	30	35	25	59,0%	68,9%	83,5%	86,9%	78,3%
4774	30	30	35	30	59,4%	68,6%	84,0%	87,6%	79,4%
4775	30	30	35	35	59,8%	68,4%	84,4%	88,4%	80,5%
4776	30	30	35	40	60,3%	68,1%	84,9%	89,0%	81,6%
4777	30	30	35	45	60,7%	67,8%	85,3%	89,7%	82,6%
4778	30	30	35	50	61,1%	67,5%	85,7%	90,3%	83,6%
4779	30	30	35	55	61,5%	67,2%	86,1%	90,9%	84,5%
4780	30	30	35	60	61,9%	66,9%	86,5%	91,5%	85,4%
4781	30	30	35	65	62,4%	66,6%	86,9%	92,0%	86,2%
4782	30	30	35	70	62,8%	66,4%	87,3%	92,5%	87,0%
4783	30	30	35	75	63,2%	66,1%	87,7%	92,9%	87,7%
4784	30	30	35	80	63,6%	65,8%	88,0%	93,4%	88,5%

4785	30	30	40	20	52,5%	64,1%	81,2%	85,3%	76,4%
4786	30	30	40	25	52,9%	63,8%	81,7%	86,2%	77,6%
4787	30	30	40	30	53,4%	63,5%	82,2%	87,0%	78,8%
4788	30	30	40	35	53,8%	63,2%	82,7%	87,7%	79,9%
4789	30	30	40	40	54,2%	62,9%	83,2%	88,4%	81,0%
4790	30	30	40	45	54,7%	62,6%	83,7%	89,1%	82,0%
4791	30	30	40	50	55,1%	62,3%	84,1%	89,8%	83,0%
4792	30	30	40	55	55,5%	62,0%	84,6%	90,4%	83,9%
4793	30	30	40	60	56,0%	61,6%	85,0%	91,0%	84,8%
4794	30	30	40	65	56,4%	61,3%	85,5%	91,5%	85,7%
4795	30	30	40	70	56,8%	61,0%	85,9%	92,0%	86,5%
4796	30	30	40	75	57,3%	60,7%	86,3%	92,5%	87,3%
4797	30	30	40	80	57,7%	60,4%	86,7%	93,0%	88,0%
4798	30	30	45	20	46,3%	58,6%	79,3%	84,5%	75,6%
4799	30	30	45	25	46,8%	58,3%	79,8%	85,4%	76,9%
4800	30	30	45	30	47,2%	58,0%	80,4%	86,2%	78,1%
4801	30	30	45	35	47,6%	57,7%	80,9%	87,0%	79,2%
4802	30	30	45	40	48,1%	57,3%	81,4%	87,8%	80,3%
4803	30	30	45	45	48,5%	57,0%	81,9%	88,5%	81,4%
4804	30	30	45	50	48,9%	56,7%	82,4%	89,2%	82,4%
4805	30	30	45	55	49,4%	56,4%	82,9%	89,9%	83,4%
4806	30	30	45	60	49,8%	56,1%	83,4%	90,5%	84,3%
4807	30	30	45	65	50,3%	55,7%	83,8%	91,0%	85,2%
4808	30	30	45	70	50,7%	55,4%	84,3%	91,6%	86,0%
4809	30	30	45	75	51,2%	55,1%	84,7%	92,1%	86,8%
4810	30	30	45	80	51,6%	54,8%	85,2%	92,6%	87,6%
4811	30	30	50	20	40,3%	52,9%	77,1%	83,7%	74,9%
4812	30	30	50	25	40,7%	52,6%	77,7%	84,6%	76,1%
4813	30	30	50	30	41,1%	52,3%	78,3%	85,5%	77,4%
4814	30	30	50	35	41,5%	51,9%	78,9%	86,3%	78,5%
4815	30	30	50	40	42,0%	51,6%	79,5%	87,1%	79,7%
4816	30	30	50	45	42,4%	51,3%	80,0%	87,9%	80,7%
4817	30	30	50	50	42,8%	51,0%	80,5%	88,6%	81,8%

4818	30	30	50	55	43,3%	50,6%	81,1%	89,3%	82,8%
4819	30	30	50	60	43,7%	50,3%	81,6%	89,9%	83,7%
4820	30	30	50	65	44,1%	50,0%	82,1%	90,5%	84,7%
4821	30	30	50	70	44,6%	49,7%	82,6%	91,1%	85,5%
4822	30	30	50	75	45,0%	49,3%	83,1%	91,6%	86,4%
4823	30	30	50	80	45,4%	49,0%	83,5%	92,2%	87,1%
4824	30	30	55	20	34,5%	47,2%	74,9%	82,9%	74,1%
4825	30	30	55	25	34,9%	46,8%	75,5%	83,8%	75,4%
4826	30	30	55	30	35,3%	46,5%	76,1%	84,7%	76,6%
4827	30	30	55	35	35,7%	46,2%	76,7%	85,6%	77,8%
4828	30	30	55	40	36,1%	45,9%	77,3%	86,4%	79,0%
4829	30	30	55	45	36,5%	45,5%	77,9%	87,2%	80,1%
4830	30	30	55	50	36,9%	45,2%	78,5%	88,0%	81,2%
4831	30	30	55	55	37,3%	44,9%	79,1%	88,7%	82,2%
4832	30	30	55	60	37,7%	44,6%	79,6%	89,4%	83,2%
4833	30	30	55	65	38,2%	44,2%	80,2%	90,0%	84,1%
4834	30	30	55	70	38,6%	43,9%	80,7%	90,6%	85,0%
4835	30	30	55	75	39,0%	43,6%	81,2%	91,2%	85,9%
4836	30	30	55	80	39,4%	43,3%	81,8%	91,7%	86,7%
4837	30	30	60	20	29,1%	41,5%	72,5%	82,0%	73,3%
4838	30	30	60	25	29,5%	41,1%	73,1%	83,0%	74,6%
4839	30	30	60	30	29,9%	40,8%	73,8%	83,9%	75,9%
4840	30	30	60	35	30,2%	40,5%	74,5%	84,8%	77,1%
4841	30	30	60	40	30,6%	40,2%	75,1%	85,7%	78,3%
4842	30	30	60	45	31,0%	39,9%	75,7%	86,5%	79,4%
4843	30	30	60	50	31,4%	39,6%	76,3%	87,3%	80,5%
4844	30	30	60	55	31,8%	39,3%	77,0%	88,1%	81,6%
4845	30	30	60	60	32,1%	39,0%	77,6%	88,8%	82,6%
4846	30	30	60	65	32,5%	38,6%	78,1%	89,4%	83,6%
4847	30	30	60	70	32,9%	38,3%	78,7%	90,1%	84,5%
4848	30	30	60	75	33,3%	38,0%	79,3%	90,7%	85,3%
4849	30	30	60	80	33,7%	37,7%	79,8%	91,2%	86,2%
4850	30	30	65	20	24,3%	36,0%	69,9%	81,1%	72,5%

4851	30	30	65	25	24,6%	35,7%	70,6%	82,1%	73,8%
4852	30	30	65	30	25,0%	35,4%	71,3%	83,1%	75,1%
4853	30	30	65	35	25,3%	35,1%	72,0%	84,0%	76,4%
4854	30	30	65	40	25,6%	34,8%	72,7%	84,9%	77,6%
4855	30	30	65	45	26,0%	34,5%	73,4%	85,8%	78,8%
4856	30	30	65	50	26,3%	34,2%	74,0%	86,6%	79,9%
4857	30	30	65	55	26,7%	33,9%	74,7%	87,4%	81,0%
4858	30	30	65	60	27,0%	33,6%	75,3%	88,1%	82,0%
4859	30	30	65	65	27,4%	33,3%	75,9%	88,8%	83,0%
4860	30	30	65	70	27,7%	33,0%	76,6%	89,5%	83,9%
4861	30	30	65	75	28,1%	32,7%	77,2%	90,1%	84,8%
4862	30	30	65	80	28,4%	32,5%	77,8%	90,7%	85,7%
4863	30	30	70	20	20,1%	30,9%	67,2%	80,1%	71,6%
4864	30	30	70	25	20,3%	30,6%	68,0%	81,2%	73,0%
4865	30	30	70	30	20,6%	30,3%	68,7%	82,2%	74,3%
4866	30	30	70	35	20,9%	30,0%	69,5%	83,2%	75,6%
4867	30	30	70	40	21,2%	29,8%	70,2%	84,1%	76,9%
4868	30	30	70	45	21,5%	29,5%	70,9%	85,0%	78,1%
4869	30	30	70	50	21,8%	29,2%	71,6%	85,9%	79,2%
4870	30	30	70	55	22,1%	28,9%	72,3%	86,7%	80,3%
4871	30	30	70	60	22,4%	28,7%	72,9%	87,5%	81,4%
4872	30	30	70	65	22,7%	28,4%	73,6%	88,2%	82,4%
4873	30	30	70	70	23,0%	28,1%	74,3%	88,9%	83,4%
4874	30	30	70	75	23,4%	27,9%	74,9%	89,6%	84,3%
4875	30	30	70	80	23,7%	27,6%	75,5%	90,2%	85,2%
4876	30	30	75	20	16,4%	26,2%	64,5%	79,1%	70,8%
4877	30	30	75	25	16,6%	25,9%	65,2%	80,2%	72,2%
4878	30	30	75	30	16,9%	25,7%	66,0%	81,3%	73,5%
4879	30	30	75	35	17,1%	25,4%	66,8%	82,3%	74,8%
4880	30	30	75	40	17,4%	25,2%	67,5%	83,3%	76,1%
4881	30	30	75	45	17,6%	24,9%	68,2%	84,3%	77,3%
4882	30	30	75	50	17,9%	24,7%	69,0%	85,1%	78,5%
4883	30	30	75	55	18,2%	24,4%	69,7%	86,0%	79,6%

4884	30	30	75	60	18,4%	24,2%	70,4%	86,8%	80,7%
4885	30	30	75	65	18,7%	24,0%	71,1%	87,6%	81,8%
4886	30	30	75	70	19,0%	23,7%	71,8%	88,3%	82,8%
4887	30	30	75	75	19,2%	23,5%	72,5%	89,0%	83,7%
4888	30	30	75	80	19,5%	23,2%	73,2%	89,7%	84,6%
4889	30	30	80	20	13,3%	22,0%	61,6%	78,1%	69,9%
4890	30	30	80	25	13,5%	21,7%	62,4%	79,3%	71,3%
4891	30	30	80	30	13,7%	21,5%	63,2%	80,4%	72,7%
4892	30	30	80	35	13,9%	21,3%	63,9%	81,4%	74,1%
4893	30	30	80	40	14,1%	21,1%	64,7%	82,4%	75,4%
4894	30	30	80	45	14,3%	20,9%	65,5%	83,4%	76,6%
4895	30	30	80	50	14,5%	20,6%	66,3%	84,4%	77,8%
4896	30	30	80	55	14,8%	20,4%	67,0%	85,2%	79,0%
4897	30	30	80	60	15,0%	20,2%	67,8%	86,1%	80,1%
4898	30	30	80	65	15,2%	20,0%	68,5%	86,9%	81,2%
4899	30	30	80	70	15,4%	19,8%	69,2%	87,7%	82,2%
4900	30	30	80	75	15,7%	19,6%	69,9%	88,4%	83,2%
4901	30	30	80	80	15,9%	19,4%	70,7%	89,1%	84,1%
4902	30	35	20	20	76,4%	82,9%	87,3%	87,3%	77,6%
4903	30	35	20	25	76,7%	82,8%	87,7%	88,1%	78,8%
4904	30	35	20	30	77,0%	82,6%	88,1%	88,8%	79,9%
4905	30	35	20	35	77,4%	82,4%	88,4%	89,4%	81,0%
4906	30	35	20	40	77,7%	82,2%	88,8%	90,1%	82,0%
4907	30	35	20	45	78,0%	82,0%	89,1%	90,7%	83,0%
4908	30	35	20	50	78,3%	81,8%	89,4%	91,2%	83,9%
4909	30	35	20	55	78,6%	81,6%	89,7%	91,8%	84,8%
4910	30	35	20	60	78,9%	81,4%	90,0%	92,3%	85,7%
4911	30	35	20	65	79,2%	81,2%	90,3%	92,8%	86,5%
4912	30	35	20	70	79,4%	81,0%	90,6%	93,2%	87,3%
4913	30	35	20	75	79,7%	80,8%	90,9%	93,6%	88,0%
4914	30	35	20	80	80,0%	80,6%	91,2%	94,0%	88,7%
4915	30	35	25	20	71,7%	79,4%	85,9%	86,6%	76,9%
4916	30	35	25	25	72,0%	79,2%	86,3%	87,4%	78,1%

4917	30	35	25	30	72,4%	79,0%	86,7%	88,1%	79,2%
4918	30	35	25	35	72,7%	78,8%	87,1%	88,8%	80,3%
4919	30	35	25	40	73,1%	78,6%	87,5%	89,5%	81,4%
4920	30	35	25	45	73,4%	78,3%	87,8%	90,1%	82,4%
4921	30	35	25	50	73,8%	78,1%	88,2%	90,7%	83,4%
4922	30	35	25	55	74,1%	77,9%	88,5%	91,3%	84,3%
4923	30	35	25	60	74,5%	77,7%	88,9%	91,8%	85,2%
4924	30	35	25	65	74,8%	77,4%	89,2%	92,3%	86,0%
4925	30	35	25	70	75,1%	77,2%	89,5%	92,8%	86,8%
4926	30	35	25	75	75,4%	77,0%	89,8%	93,3%	87,6%
4927	30	35	25	80	75,8%	76,7%	90,1%	93,7%	88,3%
4928	30	35	30	20	66,4%	75,4%	84,3%	85,9%	76,1%
4929	30	35	30	25	66,8%	75,1%	84,8%	86,7%	77,4%
4930	30	35	30	30	67,2%	74,9%	85,2%	87,5%	78,5%
4931	30	35	30	35	67,6%	74,7%	85,6%	88,2%	79,7%
4932	30	35	30	40	68,0%	74,4%	86,0%	88,9%	80,8%
4933	30	35	30	45	68,3%	74,2%	86,4%	89,6%	81,8%
4934	30	35	30	50	68,7%	73,9%	86,8%	90,2%	82,8%
4935	30	35	30	55	69,1%	73,7%	87,2%	90,8%	83,8%
4936	30	35	30	60	69,5%	73,4%	87,6%	91,4%	84,7%
4937	30	35	30	65	69,9%	73,1%	87,9%	91,9%	85,5%
4938	30	35	30	70	70,2%	72,9%	88,3%	92,4%	86,4%
4939	30	35	30	75	70,6%	72,6%	88,6%	92,9%	87,1%
4940	30	35	30	80	71,0%	72,4%	89,0%	93,3%	87,9%
4941	30	35	35	20	60,7%	70,9%	82,6%	85,1%	75,4%
4942	30	35	35	25	61,1%	70,6%	83,1%	86,0%	76,6%
4943	30	35	35	30	61,5%	70,3%	83,6%	86,8%	77,8%
4944	30	35	35	35	62,0%	70,0%	84,0%	87,6%	79,0%
4945	30	35	35	40	62,4%	69,8%	84,5%	88,3%	80,1%
4946	30	35	35	45	62,8%	69,5%	84,9%	89,0%	81,2%
4947	30	35	35	50	63,2%	69,2%	85,3%	89,7%	82,2%
4948	30	35	35	55	63,6%	68,9%	85,8%	90,3%	83,2%
4949	30	35	35	60	64,0%	68,7%	86,2%	90,9%	84,1%

4950	30	35	35	65	64,4%	68,4%	86,6%	91,4%	85,0%
4951	30	35	35	70	64,8%	68,1%	87,0%	91,9%	85,9%
4952	30	35	35	75	65,2%	67,8%	87,3%	92,4%	86,7%
4953	30	35	35	80	65,6%	67,5%	87,7%	92,9%	87,5%
4954	30	35	40	20	54,7%	65,9%	80,7%	84,4%	74,6%
4955	30	35	40	25	55,1%	65,6%	81,3%	85,2%	75,9%
4956	30	35	40	30	55,6%	65,3%	81,8%	86,1%	77,1%
4957	30	35	40	35	56,0%	65,0%	82,3%	86,9%	78,3%
4958	30	35	40	40	56,4%	64,7%	82,8%	87,7%	79,4%
4959	30	35	40	45	56,9%	64,4%	83,2%	88,4%	80,5%
4960	30	35	40	50	57,3%	64,1%	83,7%	89,1%	81,6%
4961	30	35	40	55	57,7%	63,8%	84,2%	89,7%	82,6%
4962	30	35	40	60	58,1%	63,5%	84,6%	90,3%	83,6%
4963	30	35	40	65	58,6%	63,2%	85,1%	90,9%	84,5%
4964	30	35	40	70	59,0%	62,9%	85,5%	91,5%	85,4%
4965	30	35	40	75	59,4%	62,6%	85,9%	92,0%	86,2%
4966	30	35	40	80	59,9%	62,3%	86,3%	92,5%	87,0%
4967	30	35	45	20	48,5%	60,5%	78,7%	83,5%	73,8%
4968	30	35	45	25	49,0%	60,2%	79,3%	84,5%	75,1%
4969	30	35	45	30	49,4%	59,9%	79,8%	85,3%	76,4%
4970	30	35	45	35	49,8%	59,6%	80,4%	86,2%	77,6%
4971	30	35	45	40	50,3%	59,3%	80,9%	87,0%	78,8%
4972	30	35	45	45	50,7%	58,9%	81,4%	87,7%	79,9%
4973	30	35	45	50	51,2%	58,6%	81,9%	88,5%	81,0%
4974	30	35	45	55	51,6%	58,3%	82,4%	89,2%	82,0%
4975	30	35	45	60	52,0%	58,0%	82,9%	89,8%	83,0%
4976	30	35	45	65	52,5%	57,7%	83,4%	90,4%	83,9%
4977	30	35	45	70	52,9%	57,3%	83,9%	91,0%	84,8%
4978	30	35	45	75	53,4%	57,0%	84,3%	91,5%	85,7%
4979	30	35	45	80	53,8%	56,7%	84,8%	92,1%	86,5%
4980	30	35	50	20	42,4%	54,9%	76,6%	82,7%	73,0%
4981	30	35	50	25	42,8%	54,6%	77,2%	83,6%	74,3%
4982	30	35	50	30	43,3%	54,2%	77,8%	84,6%	75,6%

4983	30	35	50	35	43,7%	53,9%	78,4%	85,4%	76,9%
4984	30	35	50	40	44,1%	53,6%	78,9%	86,3%	78,1%
4985	30	35	50	45	44,6%	53,3%	79,5%	87,1%	79,2%
4986	30	35	50	50	45,0%	52,9%	80,0%	87,8%	80,3%
4987	30	35	50	55	45,4%	52,6%	80,6%	88,6%	81,4%
4988	30	35	50	60	45,9%	52,3%	81,1%	89,2%	82,4%
4989	30	35	50	65	46,3%	52,0%	81,6%	89,9%	83,4%
4990	30	35	50	70	46,8%	51,6%	82,1%	90,5%	84,3%
4991	30	35	50	75	47,2%	51,3%	82,6%	91,1%	85,2%
4992	30	35	50	80	47,6%	51,0%	83,1%	91,6%	86,0%
4993	30	35	55	20	36,5%	49,1%	74,3%	81,8%	72,2%
4994	30	35	55	25	36,9%	48,8%	74,9%	82,8%	73,6%
4995	30	35	55	30	37,3%	48,5%	75,6%	83,8%	74,9%
4996	30	35	55	35	37,8%	48,1%	76,2%	84,7%	76,1%
4997	30	35	55	40	38,2%	47,8%	76,8%	85,5%	77,4%
4998	30	35	55	45	38,6%	47,5%	77,4%	86,4%	78,5%
4999	30	35	55	50	39,0%	47,2%	78,0%	87,2%	79,7%
5000	30	35	55	55	39,4%	46,8%	78,6%	87,9%	80,8%
5001	30	35	55	60	39,8%	46,5%	79,1%	88,6%	81,8%
5002	30	35	55	65	40,3%	46,2%	79,7%	89,3%	82,8%
5003	30	35	55	70	40,7%	45,9%	80,2%	89,9%	83,7%
5004	30	35	55	75	41,1%	45,5%	80,8%	90,6%	84,7%
5005	30	35	55	80	41,5%	45,2%	81,3%	91,1%	85,5%
5006	30	35	60	20	31,0%	43,4%	71,8%	80,9%	71,4%
5007	30	35	60	25	31,4%	43,1%	72,5%	81,9%	72,7%
5008	30	35	60	30	31,8%	42,7%	73,2%	82,9%	74,1%
5009	30	35	60	35	32,1%	42,4%	73,8%	83,9%	75,4%
5010	30	35	60	40	32,5%	42,1%	74,5%	84,8%	76,6%
5011	30	35	60	45	32,9%	41,8%	75,1%	85,6%	77,8%
5012	30	35	60	50	33,3%	41,5%	75,8%	86,5%	79,0%
5013	30	35	60	55	33,7%	41,2%	76,4%	87,3%	80,1%
5014	30	35	60	60	34,1%	40,8%	77,0%	88,0%	81,2%
5015	30	35	60	65	34,5%	40,5%	77,6%	88,7%	82,2%

5016	30	35	60	70	34,9%	40,2%	78,2%	89,4%	83,2%
5017	30	35	60	75	35,3%	39,9%	78,7%	90,0%	84,1%
5018	30	35	60	80	35,7%	39,6%	79,3%	90,6%	85,0%
5019	30	35	65	20	26,0%	37,8%	69,3%	79,9%	70,5%
5020	30	35	65	25	26,3%	37,5%	70,0%	81,0%	71,9%
5021	30	35	65	30	26,7%	37,2%	70,7%	82,0%	73,3%
5022	30	35	65	35	27,0%	36,9%	71,4%	83,0%	74,6%
5023	30	35	65	40	27,4%	36,6%	72,1%	84,0%	75,9%
5024	30	35	65	45	27,7%	36,3%	72,7%	84,9%	77,1%
5025	30	35	65	50	28,1%	36,0%	73,4%	85,7%	78,3%
5026	30	35	65	55	28,4%	35,7%	74,1%	86,6%	79,4%
5027	30	35	65	60	28,8%	35,4%	74,7%	87,3%	80,5%
5028	30	35	65	65	29,2%	35,1%	75,4%	88,1%	81,6%
5029	30	35	65	70	29,5%	34,8%	76,0%	88,8%	82,6%
5030	30	35	65	75	29,9%	34,5%	76,6%	89,5%	83,6%
5031	30	35	65	80	30,3%	34,2%	77,2%	90,1%	84,5%
5032	30	35	70	20	21,5%	32,6%	66,5%	78,9%	69,7%
5033	30	35	70	25	21,8%	32,3%	67,3%	80,0%	71,1%
5034	30	35	70	30	22,1%	32,0%	68,0%	81,1%	72,5%
5035	30	35	70	35	22,4%	31,7%	68,8%	82,1%	73,8%
5036	30	35	70	40	22,7%	31,4%	69,5%	83,1%	75,1%
5037	30	35	70	45	23,1%	31,1%	70,2%	84,1%	76,4%
5038	30	35	70	50	23,4%	30,9%	70,9%	85,0%	77,6%
5039	30	35	70	55	23,7%	30,6%	71,6%	85,8%	78,8%
5040	30	35	70	60	24,0%	30,3%	72,3%	86,7%	79,9%
5041	30	35	70	65	24,3%	30,0%	73,0%	87,4%	81,0%
5042	30	35	70	70	24,7%	29,8%	73,6%	88,2%	82,0%
5043	30	35	70	75	25,0%	29,5%	74,3%	88,9%	83,0%
5044	30	35	70	80	25,3%	29,2%	74,9%	89,5%	83,9%
5045	30	35	75	20	17,6%	27,7%	63,7%	77,9%	68,8%
5046	30	35	75	25	17,9%	27,5%	64,5%	79,0%	70,2%
5047	30	35	75	30	18,2%	27,2%	65,3%	80,2%	71,6%
5048	30	35	75	35	18,4%	26,9%	66,0%	81,2%	73,0%

5049	30	35	75	40	18,7%	26,7%	66,8%	82,3%	74,3%
5050	30	35	75	45	19,0%	26,4%	67,6%	83,2%	75,6%
5051	30	35	75	50	19,2%	26,2%	68,3%	84,2%	76,9%
5052	30	35	75	55	19,5%	25,9%	69,0%	85,1%	78,1%
5053	30	35	75	60	19,8%	25,7%	69,7%	85,9%	79,2%
5054	30	35	75	65	20,1%	25,4%	70,5%	86,7%	80,3%
5055	30	35	75	70	20,4%	25,2%	71,2%	87,5%	81,4%
5056	30	35	75	75	20,6%	24,9%	71,9%	88,3%	82,4%
5057	30	35	75	80	20,9%	24,7%	72,5%	89,0%	83,4%
5058	30	35	80	20	14,3%	23,3%	60,8%	76,8%	67,9%
5059	30	35	80	25	14,6%	23,1%	61,6%	78,0%	69,4%
5060	30	35	80	30	14,8%	22,9%	62,4%	79,2%	70,8%
5061	30	35	80	35	15,0%	22,6%	63,2%	80,3%	72,2%
5062	30	35	80	40	15,2%	22,4%	64,0%	81,4%	73,5%
5063	30	35	80	45	15,5%	22,2%	64,8%	82,4%	74,9%
5064	30	35	80	50	15,7%	22,0%	65,5%	83,4%	76,1%
5065	30	35	80	55	15,9%	21,7%	66,3%	84,3%	77,3%
5066	30	35	80	60	16,2%	21,5%	67,1%	85,2%	78,5%
5067	30	35	80	65	16,4%	21,3%	67,8%	86,0%	79,7%
5068	30	35	80	70	16,6%	21,1%	68,5%	86,8%	80,7%
5069	30	35	80	75	16,9%	20,9%	69,3%	87,6%	81,8%
5070	30	35	80	80	17,1%	20,6%	70,0%	88,3%	82,8%
5071	30	40	20	20	78,0%	84,0%	87,0%	86,5%	75,9%
5072	30	40	20	25	78,3%	83,9%	87,3%	87,3%	77,1%
5073	30	40	20	30	78,6%	83,7%	87,7%	88,0%	78,3%
5074	30	40	20	35	78,9%	83,5%	88,1%	88,7%	79,5%
5075	30	40	20	40	79,2%	83,3%	88,4%	89,4%	80,6%
5076	30	40	20	45	79,5%	83,1%	88,8%	90,0%	81,6%
5077	30	40	20	50	79,7%	82,9%	89,1%	90,6%	82,6%
5078	30	40	20	55	80,0%	82,8%	89,4%	91,2%	83,6%
5079	30	40	20	60	80,3%	82,6%	89,8%	91,7%	84,5%
5080	30	40	20	65	80,6%	82,4%	90,1%	92,2%	85,4%
5081	30	40	20	70	80,9%	82,2%	90,4%	92,7%	86,2%

5082	30	40	20	75	81,1%	82,0%	90,7%	93,2%	87,0%
5083	30	40	20	80	81,4%	81,8%	90,9%	93,6%	87,8%
5084	30	40	25	20	73,4%	80,7%	85,5%	85,7%	75,1%
5085	30	40	25	25	73,8%	80,5%	85,9%	86,6%	76,4%
5086	30	40	25	30	74,1%	80,3%	86,3%	87,3%	77,6%
5087	30	40	25	35	74,5%	80,1%	86,7%	88,1%	78,8%
5088	30	40	25	40	74,8%	79,9%	87,1%	88,8%	79,9%
5089	30	40	25	45	75,1%	79,6%	87,5%	89,5%	81,0%
5090	30	40	25	50	75,5%	79,4%	87,8%	90,1%	82,0%
5091	30	40	25	55	75,8%	79,2%	88,2%	90,7%	83,0%
5092	30	40	25	60	76,1%	79,0%	88,6%	91,3%	83,9%
5093	30	40	25	65	76,4%	78,8%	88,9%	91,8%	84,9%
5094	30	40	25	70	76,7%	78,6%	89,2%	92,3%	85,7%
5095	30	40	25	75	77,1%	78,3%	89,5%	92,8%	86,5%
5096	30	40	25	80	77,4%	78,1%	89,9%	93,2%	87,3%
5097	30	40	30	20	68,4%	76,8%	83,9%	85,0%	74,4%
5098	30	40	30	25	68,7%	76,6%	84,3%	85,8%	75,7%
5099	30	40	30	30	69,1%	76,4%	84,8%	86,7%	76,9%
5100	30	40	30	35	69,5%	76,1%	85,2%	87,4%	78,1%
5101	30	40	30	40	69,9%	75,9%	85,6%	88,2%	79,2%
5102	30	40	30	45	70,2%	75,6%	86,1%	88,9%	80,3%
5103	30	40	30	50	70,6%	75,4%	86,5%	89,5%	81,4%
5104	30	40	30	55	71,0%	75,2%	86,8%	90,2%	82,4%
5105	30	40	30	60	71,3%	74,9%	87,2%	90,8%	83,4%
5106	30	40	30	65	71,7%	74,7%	87,6%	91,3%	84,3%
5107	30	40	30	70	72,0%	74,4%	88,0%	91,9%	85,2%
5108	30	40	30	75	72,4%	74,2%	88,3%	92,4%	86,0%
5109	30	40	30	80	72,7%	73,9%	88,7%	92,8%	86,8%
5110	30	40	35	20	62,8%	72,5%	82,1%	84,2%	73,6%
5111	30	40	35	25	63,2%	72,2%	82,6%	85,1%	74,9%
5112	30	40	35	30	63,6%	71,9%	83,1%	85,9%	76,2%
5113	30	40	35	35	64,0%	71,7%	83,6%	86,7%	77,4%
5114	30	40	35	40	64,4%	71,4%	84,0%	87,5%	78,5%

5115	30	40	35	45	64,8%	71,1%	84,5%	88,3%	79,7%
5116	30	40	35	50	65,2%	70,9%	84,9%	88,9%	80,8%
5117	30	40	35	55	65,6%	70,6%	85,4%	89,6%	81,8%
5118	30	40	35	60	66,0%	70,3%	85,8%	90,2%	82,8%
5119	30	40	35	65	66,4%	70,1%	86,2%	90,8%	83,8%
5120	30	40	35	70	66,8%	69,8%	86,6%	91,4%	84,7%
5121	30	40	35	75	67,2%	69,5%	87,0%	91,9%	85,5%
5122	30	40	35	80	67,6%	69,2%	87,4%	92,4%	86,4%
5123	30	40	40	20	56,9%	67,6%	80,2%	83,4%	72,8%
5124	30	40	40	25	57,3%	67,3%	80,8%	84,3%	74,1%
5125	30	40	40	30	57,7%	67,0%	81,3%	85,2%	75,4%
5126	30	40	40	35	58,2%	66,8%	81,8%	86,0%	76,6%
5127	30	40	40	40	58,6%	66,5%	82,3%	86,8%	77,8%
5128	30	40	40	45	59,0%	66,2%	82,8%	87,6%	79,0%
5129	30	40	40	50	59,4%	65,9%	83,3%	88,3%	80,1%
5130	30	40	40	55	59,9%	65,6%	83,7%	89,0%	81,2%
5131	30	40	40	60	60,3%	65,3%	84,2%	89,7%	82,2%
5132	30	40	40	65	60,7%	65,0%	84,6%	90,3%	83,2%
5133	30	40	40	70	61,1%	64,7%	85,1%	90,9%	84,1%
5134	30	40	40	75	61,5%	64,4%	85,5%	91,4%	85,0%
5135	30	40	40	80	62,0%	64,1%	85,9%	92,0%	85,9%
5136	30	40	45	20	50,7%	62,4%	78,2%	82,5%	71,9%
5137	30	40	45	25	51,2%	62,1%	78,8%	83,5%	73,3%
5138	30	40	45	30	51,6%	61,8%	79,3%	84,4%	74,6%
5139	30	40	45	35	52,1%	61,5%	79,9%	85,3%	75,9%
5140	30	40	45	40	52,5%	61,1%	80,4%	86,1%	77,1%
5141	30	40	45	45	52,9%	60,8%	81,0%	86,9%	78,3%
5142	30	40	45	50	53,4%	60,5%	81,5%	87,7%	79,5%
5143	30	40	45	55	53,8%	60,2%	82,0%	88,4%	80,5%
5144	30	40	45	60	54,3%	59,9%	82,5%	89,1%	81,6%
5145	30	40	45	65	54,7%	59,6%	83,0%	89,8%	82,6%
5146	30	40	45	70	55,1%	59,3%	83,4%	90,4%	83,6%
5147	30	40	45	75	55,6%	58,9%	83,9%	91,0%	84,5%

5148	30	40	45	80	56,0%	58,6%	84,4%	91,5%	85,4%
5149	30	40	50	20	44,6%	56,8%	76,0%	81,6%	71,1%
5150	30	40	50	25	45,0%	56,5%	76,6%	82,6%	72,5%
5151	30	40	50	30	45,5%	56,2%	77,2%	83,6%	73,8%
5152	30	40	50	35	45,9%	55,9%	77,8%	84,5%	75,1%
5153	30	40	50	40	46,3%	55,5%	78,4%	85,4%	76,4%
5154	30	40	50	45	46,8%	55,2%	79,0%	86,2%	77,6%
5155	30	40	50	50	47,2%	54,9%	79,5%	87,0%	78,8%
5156	30	40	50	55	47,7%	54,6%	80,1%	87,8%	79,9%
5157	30	40	50	60	48,1%	54,2%	80,6%	88,5%	81,0%
5158	30	40	50	65	48,5%	53,9%	81,1%	89,2%	82,0%
5159	30	40	50	70	49,0%	53,6%	81,6%	89,8%	83,0%
5160	30	40	50	75	49,4%	53,3%	82,1%	90,4%	83,9%
5161	30	40	50	80	49,9%	52,9%	82,6%	91,0%	84,8%
5162	30	40	55	20	38,6%	51,1%	73,7%	80,7%	70,2%
5163	30	40	55	25	39,0%	50,8%	74,3%	81,7%	71,7%
5164	30	40	55	30	39,4%	50,4%	75,0%	82,7%	73,0%
5165	30	40	55	35	39,9%	50,1%	75,6%	83,7%	74,4%
5166	30	40	55	40	40,3%	49,8%	76,2%	84,6%	75,6%
5167	30	40	55	45	40,7%	49,5%	76,8%	85,5%	76,9%
5168	30	40	55	50	41,1%	49,1%	77,4%	86,3%	78,1%
5169	30	40	55	55	41,6%	48,8%	78,0%	87,1%	79,2%
5170	30	40	55	60	42,0%	48,5%	78,6%	87,9%	80,3%
5171	30	40	55	65	42,4%	48,2%	79,2%	88,6%	81,4%
5172	30	40	55	70	42,9%	47,8%	79,7%	89,3%	82,4%
5173	30	40	55	75	43,3%	47,5%	80,3%	89,9%	83,4%
5174	30	40	55	80	43,7%	47,2%	80,8%	90,5%	84,3%
5175	30	40	60	20	32,9%	45,3%	71,2%	79,7%	69,4%
5176	30	40	60	25	33,3%	45,0%	71,9%	80,8%	70,8%
5177	30	40	60	30	33,7%	44,7%	72,6%	81,8%	72,2%
5178	30	40	60	35	34,1%	44,4%	73,2%	82,8%	73,6%
5179	30	40	60	40	34,5%	44,0%	73,9%	83,8%	74,9%
5180	30	40	60	45	34,9%	43,7%	74,5%	84,7%	76,1%

5181	30	40	60	50	35,3%	43,4%	75,2%	85,6%	77,4%
5182	30	40	60	55	35,7%	43,1%	75,8%	86,4%	78,5%
5183	30	40	60	60	36,1%	42,8%	76,4%	87,2%	79,7%
5184	30	40	60	65	36,5%	42,4%	77,0%	87,9%	80,8%
5185	30	40	60	70	36,9%	42,1%	77,6%	88,7%	81,8%
5186	30	40	60	75	37,4%	41,8%	78,2%	89,3%	82,8%
5187	30	40	60	80	37,8%	41,5%	78,8%	90,0%	83,7%
5188	30	40	65	20	27,7%	39,7%	68,6%	78,7%	68,5%
5189	30	40	65	25	28,1%	39,4%	69,3%	79,8%	70,0%
5190	30	40	65	30	28,4%	39,1%	70,0%	80,9%	71,4%
5191	30	40	65	35	28,8%	38,8%	70,7%	81,9%	72,8%
5192	30	40	65	40	29,2%	38,5%	71,4%	82,9%	74,1%
5193	30	40	65	45	29,5%	38,1%	72,1%	83,9%	75,4%
5194	30	40	65	50	29,9%	37,8%	72,8%	84,8%	76,6%
5195	30	40	65	55	30,3%	37,5%	73,5%	85,7%	77,8%
5196	30	40	65	60	30,6%	37,2%	74,1%	86,5%	79,0%
5197	30	40	65	65	31,0%	36,9%	74,8%	87,3%	80,1%
5198	30	40	65	70	31,4%	36,6%	75,4%	88,0%	81,2%
5199	30	40	65	75	31,8%	36,3%	76,0%	88,7%	82,2%
5200	30	40	65	80	32,2%	36,0%	76,6%	89,4%	83,2%
5201	30	40	70	20	23,1%	34,3%	65,8%	77,6%	67,6%
5202	30	40	70	25	23,4%	34,0%	66,6%	78,8%	69,1%
5203	30	40	70	30	23,7%	33,7%	67,3%	79,9%	70,5%
5204	30	40	70	35	24,0%	33,4%	68,1%	81,0%	71,9%
5205	30	40	70	40	24,3%	33,2%	68,8%	82,1%	73,3%
5206	30	40	70	45	24,7%	32,9%	69,5%	83,1%	74,6%
5207	30	40	70	50	25,0%	32,6%	70,3%	84,0%	75,9%
5208	30	40	70	55	25,3%	32,3%	71,0%	84,9%	77,1%
5209	30	40	70	60	25,7%	32,0%	71,7%	85,8%	78,3%
5210	30	40	70	65	26,0%	31,7%	72,3%	86,6%	79,4%
5211	30	40	70	70	26,3%	31,4%	73,0%	87,4%	80,5%
5212	30	40	70	75	26,7%	31,2%	73,7%	88,1%	81,6%
5213	30	40	70	80	27,0%	30,9%	74,3%	88,8%	82,6%

5214	30	40	75	20	19,0%	29,3%	63,0%	76,6%	66,7%
5215	30	40	75	25	19,2%	29,0%	63,8%	77,8%	68,2%
5216	30	40	75	30	19,5%	28,8%	64,6%	79,0%	69,7%
5217	30	40	75	35	19,8%	28,5%	65,3%	80,1%	71,1%
5218	30	40	75	40	20,1%	28,2%	66,1%	81,2%	72,5%
5219	30	40	75	45	20,4%	28,0%	66,9%	82,2%	73,8%
5220	30	40	75	50	20,7%	27,7%	67,6%	83,2%	75,1%
5221	30	40	75	55	20,9%	27,5%	68,3%	84,1%	76,4%
5222	30	40	75	60	21,2%	27,2%	69,1%	85,0%	77,6%
5223	30	40	75	65	21,5%	26,9%	69,8%	85,9%	78,8%
5224	30	40	75	70	21,8%	26,7%	70,5%	86,7%	79,9%
5225	30	40	75	75	22,1%	26,4%	71,2%	87,5%	81,0%
5226	30	40	75	80	22,4%	26,2%	71,9%	88,2%	82,0%
5227	30	40	80	20	15,5%	24,8%	60,0%	75,5%	65,8%
5228	30	40	80	25	15,7%	24,5%	60,9%	76,7%	67,3%
5229	30	40	80	30	15,9%	24,3%	61,7%	77,9%	68,8%
5230	30	40	80	35	16,2%	24,0%	62,5%	79,1%	70,2%
5231	30	40	80	40	16,4%	23,8%	63,3%	80,2%	71,6%
5232	30	40	80	45	16,7%	23,6%	64,0%	81,3%	73,0%
5233	30	40	80	50	16,9%	23,3%	64,8%	82,3%	74,3%
5234	30	40	80	55	17,1%	23,1%	65,6%	83,3%	75,6%
5235	30	40	80	60	17,4%	22,9%	66,4%	84,2%	76,9%
5236	30	40	80	65	17,7%	22,6%	67,1%	85,1%	78,1%
5237	30	40	80	70	17,9%	22,4%	67,9%	86,0%	79,2%
5238	30	40	80	75	18,2%	22,2%	68,6%	86,8%	80,3%
5239	30	40	80	80	18,4%	22,0%	69,3%	87,6%	81,4%
5240	30	45	20	20	79,5%	85,1%	86,6%	85,6%	74,1%
5241	30	45	20	25	79,7%	84,9%	87,0%	86,4%	75,4%
5242	30	45	20	30	80,0%	84,7%	87,4%	87,2%	76,7%
5243	30	45	20	35	80,3%	84,6%	87,7%	87,9%	77,9%
5244	30	45	20	40	80,6%	84,4%	88,1%	88,7%	79,0%
5245	30	45	20	45	80,9%	84,2%	88,5%	89,3%	80,1%
5246	30	45	20	50	81,1%	84,0%	88,8%	90,0%	81,2%

5247	30	45	20	55	81,4%	83,9%	89,1%	90,6%	82,2%
5248	30	45	20	60	81,7%	83,7%	89,5%	91,2%	83,2%
5249	30	45	20	65	81,9%	83,5%	89,8%	91,7%	84,1%
5250	30	45	20	70	82,2%	83,3%	90,1%	92,2%	85,0%
5251	30	45	20	75	82,4%	83,1%	90,4%	92,7%	85,9%
5252	30	45	20	80	82,7%	82,9%	90,7%	93,1%	86,7%
5253	30	45	25	20	75,1%	81,9%	85,1%	84,8%	73,3%
5254	30	45	25	25	75,5%	81,7%	85,5%	85,7%	74,6%
5255	30	45	25	30	75,8%	81,5%	85,9%	86,5%	75,9%
5256	30	45	25	35	76,1%	81,3%	86,3%	87,3%	77,1%
5257	30	45	25	40	76,4%	81,1%	86,7%	88,0%	78,3%
5258	30	45	25	45	76,7%	80,9%	87,1%	88,7%	79,5%
5259	30	45	25	50	77,1%	80,7%	87,5%	89,4%	80,6%
5260	30	45	25	55	77,4%	80,5%	87,9%	90,0%	81,6%
5261	30	45	25	60	77,7%	80,3%	88,2%	90,6%	82,6%
5262	30	45	25	65	78,0%	80,1%	88,6%	91,2%	83,6%
5263	30	45	25	70	78,3%	79,9%	88,9%	91,8%	84,5%
5264	30	45	25	75	78,6%	79,6%	89,2%	92,3%	85,4%
5265	30	45	25	80	78,9%	79,4%	89,6%	92,7%	86,2%
5266	30	45	30	20	70,2%	78,2%	83,5%	84,0%	72,5%
5267	30	45	30	25	70,6%	78,0%	83,9%	84,9%	73,9%
5268	30	45	30	30	71,0%	77,7%	84,4%	85,8%	75,2%
5269	30	45	30	35	71,3%	77,5%	84,8%	86,6%	76,4%
5270	30	45	30	40	71,7%	77,3%	85,2%	87,4%	77,6%
5271	30	45	30	45	72,1%	77,1%	85,7%	88,1%	78,8%
5272	30	45	30	50	72,4%	76,8%	86,1%	88,8%	79,9%
5273	30	45	30	55	72,8%	76,6%	86,5%	89,5%	81,0%
5274	30	45	30	60	73,1%	76,4%	86,9%	90,1%	82,0%
5275	30	45	30	65	73,5%	76,1%	87,3%	90,7%	83,0%
5276	30	45	30	70	73,8%	75,9%	87,6%	91,3%	84,0%
5277	30	45	30	75	74,1%	75,6%	88,0%	91,8%	84,9%
5278	30	45	30	80	74,5%	75,4%	88,3%	92,3%	85,7%
5279	30	45	35	20	64,8%	74,0%	81,7%	83,2%	71,7%

5280	30	45	35	25	65,2%	73,8%	82,2%	84,1%	73,0%
5281	30	45	35	30	65,6%	73,5%	82,7%	85,0%	74,4%
5282	30	45	35	35	66,0%	73,2%	83,1%	85,9%	75,7%
5283	30	45	35	40	66,4%	73,0%	83,6%	86,7%	76,9%
5284	30	45	35	45	66,8%	72,7%	84,1%	87,5%	78,1%
5285	30	45	35	50	67,2%	72,5%	84,5%	88,2%	79,2%
5286	30	45	35	55	67,6%	72,2%	85,0%	88,9%	80,3%
5287	30	45	35	60	68,0%	71,9%	85,4%	89,6%	81,4%
5288	30	45	35	65	68,4%	71,7%	85,8%	90,2%	82,4%
5289	30	45	35	70	68,7%	71,4%	86,2%	90,8%	83,4%
5290	30	45	35	75	69,1%	71,1%	86,6%	91,3%	84,3%
5291	30	45	35	80	69,5%	70,9%	87,0%	91,9%	85,2%
5292	30	45	40	20	59,0%	69,3%	79,7%	82,3%	70,8%
5293	30	45	40	25	59,5%	69,0%	80,3%	83,3%	72,2%
5294	30	45	40	30	59,9%	68,8%	80,8%	84,2%	73,6%
5295	30	45	40	35	60,3%	68,5%	81,3%	85,1%	74,9%
5296	30	45	40	40	60,7%	68,2%	81,8%	86,0%	76,2%
5297	30	45	40	45	61,1%	67,9%	82,3%	86,8%	77,4%
5298	30	45	40	50	61,6%	67,6%	82,8%	87,5%	78,6%
5299	30	45	40	55	62,0%	67,3%	83,3%	88,3%	79,7%
5300	30	45	40	60	62,4%	67,1%	83,8%	89,0%	80,8%
5301	30	45	40	65	62,8%	66,8%	84,2%	89,6%	81,8%
5302	30	45	40	70	63,2%	66,5%	84,7%	90,3%	82,8%
5303	30	45	40	75	63,6%	66,2%	85,1%	90,8%	83,8%
5304	30	45	40	80	64,0%	65,9%	85,5%	91,4%	84,7%
5305	30	45	45	20	53,0%	64,2%	77,6%	81,4%	70,0%
5306	30	45	45	25	53,4%	63,9%	78,2%	82,4%	71,4%
5307	30	45	45	30	53,8%	63,6%	78,8%	83,4%	72,8%
5308	30	45	45	35	54,3%	63,3%	79,4%	84,3%	74,1%
5309	30	45	45	40	54,7%	63,0%	79,9%	85,2%	75,4%
5310	30	45	45	45	55,1%	62,7%	80,5%	86,1%	76,6%
5311	30	45	45	50	55,6%	62,4%	81,0%	86,9%	77,9%
5312	30	45	45	55	56,0%	62,1%	81,5%	87,6%	79,0%

5313	30	45	45	60	56,4%	61,8%	82,0%	88,4%	80,1%
5314	30	45	45	65	56,9%	61,5%	82,5%	89,1%	81,2%
5315	30	45	45	70	57,3%	61,1%	83,0%	89,7%	82,2%
5316	30	45	45	75	57,7%	60,8%	83,5%	90,3%	83,2%
5317	30	45	45	80	58,2%	60,5%	83,9%	90,9%	84,1%
5318	30	45	50	20	46,8%	58,7%	75,4%	80,5%	69,1%
5319	30	45	50	25	47,2%	58,4%	76,0%	81,5%	70,5%
5320	30	45	50	30	47,7%	58,1%	76,7%	82,5%	71,9%
5321	30	45	50	35	48,1%	57,8%	77,3%	83,5%	73,3%
5322	30	45	50	40	48,5%	57,5%	77,8%	84,4%	74,6%
5323	30	45	50	45	49,0%	57,2%	78,4%	85,3%	75,9%
5324	30	45	50	50	49,4%	56,8%	79,0%	86,2%	77,1%
5325	30	45	50	55	49,9%	56,5%	79,6%	87,0%	78,3%
5326	30	45	50	60	50,3%	56,2%	80,1%	87,7%	79,5%
5327	30	45	50	65	50,8%	55,9%	80,6%	88,4%	80,6%
5328	30	45	50	70	51,2%	55,5%	81,2%	89,1%	81,6%
5329	30	45	50	75	51,6%	55,2%	81,7%	89,8%	82,6%
5330	30	45	50	80	52,1%	54,9%	82,2%	90,4%	83,6%
5331	30	45	55	20	40,7%	53,1%	73,0%	79,5%	68,2%
5332	30	45	55	25	41,1%	52,7%	73,7%	80,6%	69,7%
5333	30	45	55	30	41,6%	52,4%	74,4%	81,6%	71,1%
5334	30	45	55	35	42,0%	52,1%	75,0%	82,6%	72,5%
5335	30	45	55	40	42,4%	51,8%	75,6%	83,6%	73,8%
5336	30	45	55	45	42,9%	51,4%	76,3%	84,5%	75,1%
5337	30	45	55	50	43,3%	51,1%	76,9%	85,4%	76,4%
5338	30	45	55	55	43,7%	50,8%	77,5%	86,3%	77,6%
5339	30	45	55	60	44,2%	50,4%	78,0%	87,1%	78,8%
5340	30	45	55	65	44,6%	50,1%	78,6%	87,8%	79,9%
5341	30	45	55	70	45,0%	49,8%	79,2%	88,5%	81,0%
5342	30	45	55	75	45,5%	49,5%	79,7%	89,2%	82,0%
5343	30	45	55	80	45,9%	49,1%	80,3%	89,9%	83,0%
5344	30	45	60	20	34,9%	47,3%	70,5%	78,5%	67,3%
5345	30	45	60	25	35,3%	47,0%	71,2%	79,6%	68,8%

5346	30	45	60	30	35,7%	46,6%	71,9%	80,7%	70,3%
5347	30	45	60	35	36,1%	46,3%	72,6%	81,8%	71,7%
5348	30	45	60	40	36,5%	46,0%	73,3%	82,8%	73,0%
5349	30	45	60	45	37,0%	45,7%	73,9%	83,7%	74,4%
5350	30	45	60	50	37,4%	45,3%	74,6%	84,6%	75,6%
5351	30	45	60	55	37,8%	45,0%	75,2%	85,5%	76,9%
5352	30	45	60	60	38,2%	44,7%	75,8%	86,3%	78,1%
5353	30	45	60	65	38,6%	44,4%	76,5%	87,1%	79,2%
5354	30	45	60	70	39,0%	44,0%	77,1%	87,9%	80,3%
5355	30	45	60	75	39,4%	43,7%	77,7%	88,6%	81,4%
5356	30	45	60	80	39,9%	43,4%	78,2%	89,3%	82,4%
5357	30	45	65	20	29,5%	41,6%	67,9%	77,4%	66,4%
5358	30	45	65	25	29,9%	41,3%	68,6%	78,6%	67,9%
5359	30	45	65	30	30,3%	41,0%	69,3%	79,7%	69,4%
5360	30	45	65	35	30,7%	40,6%	70,1%	80,8%	70,8%
5361	30	45	65	40	31,0%	40,3%	70,8%	81,9%	72,2%
5362	30	45	65	45	31,4%	40,0%	71,5%	82,9%	73,6%
5363	30	45	65	50	31,8%	39,7%	72,2%	83,8%	74,9%
5364	30	45	65	55	32,2%	39,4%	72,8%	84,7%	76,1%
5365	30	45	65	60	32,6%	39,1%	73,5%	85,6%	77,4%
5366	30	45	65	65	32,9%	38,8%	74,2%	86,4%	78,5%
5367	30	45	65	70	33,3%	38,5%	74,8%	87,2%	79,7%
5368	30	45	65	75	33,7%	38,1%	75,4%	88,0%	80,8%
5369	30	45	65	80	34,1%	37,8%	76,1%	88,7%	81,8%
5370	30	45	70	20	24,7%	36,1%	65,1%	76,3%	65,5%
5371	30	45	70	25	25,0%	35,8%	65,9%	77,6%	67,0%
5372	30	45	70	30	25,3%	35,5%	66,6%	78,7%	68,5%
5373	30	45	70	35	25,7%	35,2%	67,4%	79,9%	70,0%
5374	30	45	70	40	26,0%	34,9%	68,1%	80,9%	71,4%
5375	30	45	70	45	26,3%	34,6%	68,9%	82,0%	72,8%
5376	30	45	70	50	26,7%	34,3%	69,6%	83,0%	74,1%
5377	30	45	70	55	27,0%	34,0%	70,3%	83,9%	75,4%
5378	30	45	70	60	27,4%	33,7%	71,0%	84,8%	76,6%

5379	30	45	70	65	27,7%	33,4%	71,7%	85,7%	77,8%
5380	30	45	70	70	28,1%	33,2%	72,4%	86,5%	79,0%
5381	30	45	70	75	28,4%	32,9%	73,1%	87,3%	80,1%
5382	30	45	70	80	28,8%	32,6%	73,7%	88,1%	81,2%
5383	30	45	75	20	20,4%	31,0%	62,2%	75,2%	64,5%
5384	30	45	75	25	20,7%	30,7%	63,0%	76,5%	66,1%
5385	30	45	75	30	21,0%	30,4%	63,8%	77,7%	67,6%
5386	30	45	75	35	21,2%	30,1%	64,6%	78,9%	69,1%
5387	30	45	75	40	21,5%	29,9%	65,4%	80,0%	70,5%
5388	30	45	75	45	21,8%	29,6%	66,1%	81,1%	71,9%
5389	30	45	75	50	22,1%	29,3%	66,9%	82,1%	73,3%
5390	30	45	75	55	22,4%	29,1%	67,6%	83,1%	74,6%
5391	30	45	75	60	22,8%	28,8%	68,4%	84,0%	75,9%
5392	30	45	75	65	23,1%	28,5%	69,1%	84,9%	77,1%
5393	30	45	75	70	23,4%	28,3%	69,8%	85,8%	78,3%
5394	30	45	75	75	23,7%	28,0%	70,5%	86,6%	79,4%
5395	30	45	75	80	24,0%	27,7%	71,2%	87,4%	80,5%
5396	30	45	80	20	16,7%	26,3%	59,3%	74,1%	63,6%
5397	30	45	80	25	16,9%	26,0%	60,1%	75,4%	65,2%
5398	30	45	80	30	17,2%	25,8%	60,9%	76,6%	66,7%
5399	30	45	80	35	17,4%	25,5%	61,7%	77,8%	68,2%
5400	30	45	80	40	17,7%	25,3%	62,5%	79,0%	69,7%
5401	30	45	80	45	17,9%	25,0%	63,3%	80,1%	71,1%
5402	30	45	80	50	18,2%	24,8%	64,1%	81,2%	72,5%
5403	30	45	80	55	18,4%	24,5%	64,9%	82,2%	73,8%
5404	30	45	80	60	18,7%	24,3%	65,6%	83,2%	75,1%
5405	30	45	80	65	19,0%	24,1%	66,4%	84,2%	76,4%
5406	30	45	80	70	19,3%	23,8%	67,2%	85,1%	77,6%
5407	30	45	80	75	19,5%	23,6%	67,9%	85,9%	78,8%
5408	30	45	80	80	19,8%	23,3%	68,6%	86,7%	79,9%
5409	30	50	20	20	80,9%	86,0%	86,2%	84,6%	72,2%
5410	30	50	20	25	81,1%	85,9%	86,6%	85,5%	73,6%
5411	30	50	20	30	81,4%	85,7%	87,0%	86,3%	74,9%

5412	30	50	20	35	81,7%	85,6%	87,4%	87,1%	76,2%
5413	30	50	20	40	81,9%	85,4%	87,8%	87,9%	77,4%
5414	30	50	20	45	82,2%	85,2%	88,1%	88,6%	78,6%
5415	30	50	20	50	82,5%	85,1%	88,5%	89,3%	79,7%
5416	30	50	20	55	82,7%	84,9%	88,8%	89,9%	80,8%
5417	30	50	20	60	83,0%	84,7%	89,2%	90,5%	81,8%
5418	30	50	20	65	83,2%	84,6%	89,5%	91,1%	82,8%
5419	30	50	20	70	83,5%	84,4%	89,8%	91,7%	83,8%
5420	30	50	20	75	83,7%	84,2%	90,1%	92,2%	84,7%
5421	30	50	20	80	83,9%	84,0%	90,4%	92,6%	85,6%
5422	30	50	25	20	76,8%	83,0%	84,7%	83,8%	71,4%
5423	30	50	25	25	77,1%	82,8%	85,1%	84,7%	72,8%
5424	30	50	25	30	77,4%	82,6%	85,5%	85,6%	74,1%
5425	30	50	25	35	77,7%	82,5%	86,0%	86,4%	75,4%
5426	30	50	25	40	78,0%	82,3%	86,4%	87,2%	76,7%
5427	30	50	25	45	78,3%	82,1%	86,8%	88,0%	77,9%
5428	30	50	25	50	78,6%	81,9%	87,1%	88,7%	79,0%
5429	30	50	25	55	78,9%	81,7%	87,5%	89,4%	80,1%
5430	30	50	25	60	79,2%	81,5%	87,9%	90,0%	81,2%
5431	30	50	25	65	79,5%	81,3%	88,2%	90,6%	82,2%
5432	30	50	25	70	79,8%	81,1%	88,6%	91,2%	83,2%
5433	30	50	25	75	80,0%	80,9%	88,9%	91,7%	84,1%
5434	30	50	25	80	80,3%	80,7%	89,3%	92,2%	85,0%
5435	30	50	30	20	72,1%	79,5%	83,0%	83,0%	70,6%
5436	30	50	30	25	72,4%	79,3%	83,5%	83,9%	72,0%
5437	30	50	30	30	72,8%	79,1%	83,9%	84,8%	73,3%
5438	30	50	30	35	73,1%	78,9%	84,4%	85,7%	74,6%
5439	30	50	30	40	73,5%	78,6%	84,8%	86,5%	75,9%
5440	30	50	30	45	73,8%	78,4%	85,3%	87,3%	77,1%
5441	30	50	30	50	74,1%	78,2%	85,7%	88,1%	78,3%
5442	30	50	30	55	74,5%	78,0%	86,1%	88,8%	79,5%
5443	30	50	30	60	74,8%	77,8%	86,5%	89,4%	80,6%
5444	30	50	30	65	75,1%	77,5%	86,9%	90,1%	81,6%

5445	30	50	30	70	75,5%	77,3%	87,3%	90,7%	82,6%
5446	30	50	30	75	75,8%	77,1%	87,7%	91,2%	83,6%
5447	30	50	30	80	76,1%	76,8%	88,0%	91,8%	84,5%
5448	30	50	35	20	66,8%	75,5%	81,2%	82,1%	69,7%
5449	30	50	35	25	67,2%	75,2%	81,7%	83,1%	71,1%
5450	30	50	35	30	67,6%	75,0%	82,2%	84,0%	72,5%
5451	30	50	35	35	68,0%	74,8%	82,7%	84,9%	73,9%
5452	30	50	35	40	68,4%	74,5%	83,2%	85,8%	75,2%
5453	30	50	35	45	68,8%	74,3%	83,6%	86,6%	76,4%
5454	30	50	35	50	69,1%	74,0%	84,1%	87,4%	77,6%
5455	30	50	35	55	69,5%	73,8%	84,6%	88,1%	78,8%
5456	30	50	35	60	69,9%	73,5%	85,0%	88,8%	79,9%
5457	30	50	35	65	70,3%	73,2%	85,4%	89,5%	81,0%
5458	30	50	35	70	70,6%	73,0%	85,8%	90,1%	82,0%
5459	30	50	35	75	71,0%	72,7%	86,2%	90,7%	83,0%
5460	30	50	35	80	71,3%	72,5%	86,6%	91,3%	84,0%
5461	30	50	40	20	61,2%	71,0%	79,2%	81,2%	68,8%
5462	30	50	40	25	61,6%	70,7%	79,8%	82,2%	70,3%
5463	30	50	40	30	62,0%	70,4%	80,3%	83,2%	71,7%
5464	30	50	40	35	62,4%	70,2%	80,8%	84,2%	73,1%
5465	30	50	40	40	62,8%	69,9%	81,4%	85,0%	74,4%
5466	30	50	40	45	63,2%	69,6%	81,9%	85,9%	75,7%
5467	30	50	40	50	63,6%	69,3%	82,4%	86,7%	76,9%
5468	30	50	40	55	64,0%	69,1%	82,9%	87,5%	78,1%
5469	30	50	40	60	64,5%	68,8%	83,3%	88,2%	79,2%
5470	30	50	40	65	64,9%	68,5%	83,8%	88,9%	80,3%
5471	30	50	40	70	65,3%	68,2%	84,3%	89,6%	81,4%
5472	30	50	40	75	65,7%	67,9%	84,7%	90,2%	82,4%
5473	30	50	40	80	66,0%	67,6%	85,1%	90,8%	83,4%
5474	30	50	45	20	55,2%	66,0%	77,1%	80,2%	67,9%
5475	30	50	45	25	55,6%	65,7%	77,7%	81,3%	69,4%
5476	30	50	45	30	56,0%	65,4%	78,3%	82,3%	70,8%
5477	30	50	45	35	56,5%	65,1%	78,8%	83,3%	72,2%

5478	30	50	45	40	56,9%	64,8%	79,4%	84,3%	73,6%
5479	30	50	45	45	57,3%	64,5%	80,0%	85,2%	74,9%
5480	30	50	45	50	57,8%	64,2%	80,5%	86,0%	76,2%
5481	30	50	45	55	58,2%	63,9%	81,0%	86,8%	77,4%
5482	30	50	45	60	58,6%	63,6%	81,5%	87,6%	78,6%
5483	30	50	45	65	59,0%	63,3%	82,0%	88,3%	79,7%
5484	30	50	45	70	59,5%	63,0%	82,5%	89,0%	80,8%
5485	30	50	45	75	59,9%	62,7%	83,0%	89,7%	81,8%
5486	30	50	45	80	60,3%	62,4%	83,5%	90,3%	82,8%
5487	30	50	50	20	49,0%	60,6%	74,8%	79,3%	67,0%
5488	30	50	50	25	49,4%	60,3%	75,5%	80,4%	68,5%
5489	30	50	50	30	49,9%	60,0%	76,1%	81,4%	70,0%
5490	30	50	50	35	50,3%	59,7%	76,7%	82,5%	71,4%
5491	30	50	50	40	50,8%	59,4%	77,3%	83,4%	72,8%
5492	30	50	50	45	51,2%	59,1%	77,9%	84,4%	74,1%
5493	30	50	50	50	51,6%	58,8%	78,5%	85,3%	75,4%
5494	30	50	50	55	52,1%	58,4%	79,0%	86,1%	76,7%
5495	30	50	50	60	52,5%	58,1%	79,6%	86,9%	77,9%
5496	30	50	50	65	53,0%	57,8%	80,1%	87,7%	79,0%
5497	30	50	50	70	53,4%	57,5%	80,7%	88,4%	80,1%
5498	30	50	50	75	53,8%	57,2%	81,2%	89,1%	81,2%
5499	30	50	50	80	54,3%	56,8%	81,7%	89,7%	82,2%
5500	30	50	55	20	42,9%	55,0%	72,4%	78,2%	66,1%
5501	30	50	55	25	43,3%	54,7%	73,1%	79,4%	67,6%
5502	30	50	55	30	43,7%	54,4%	73,8%	80,5%	69,1%
5503	30	50	55	35	44,2%	54,0%	74,4%	81,6%	70,5%
5504	30	50	55	40	44,6%	53,7%	75,0%	82,6%	71,9%
5505	30	50	55	45	45,1%	53,4%	75,7%	83,5%	73,3%
5506	30	50	55	50	45,5%	53,1%	76,3%	84,5%	74,6%
5507	30	50	55	55	45,9%	52,7%	76,9%	85,4%	75,9%
5508	30	50	55	60	46,4%	52,4%	77,5%	86,2%	77,1%
5509	30	50	55	65	46,8%	52,1%	78,1%	87,0%	78,3%
5510	30	50	55	70	47,2%	51,8%	78,7%	87,8%	79,5%

5511	30	50	55	75	47,7%	51,4%	79,2%	88,5%	80,6%
5512	30	50	55	80	48,1%	51,1%	79,8%	89,2%	81,6%
5513	30	50	60	20	37,0%	49,3%	69,9%	77,2%	65,2%
5514	30	50	60	25	37,4%	48,9%	70,6%	78,4%	66,7%
5515	30	50	60	30	37,8%	48,6%	71,3%	79,5%	68,2%
5516	30	50	60	35	38,2%	48,3%	72,0%	80,6%	69,7%
5517	30	50	60	40	38,6%	48,0%	72,6%	81,7%	71,1%
5518	30	50	60	45	39,0%	47,6%	73,3%	82,7%	72,5%
5519	30	50	60	50	39,5%	47,3%	74,0%	83,7%	73,8%
5520	30	50	60	55	39,9%	47,0%	74,6%	84,6%	75,1%
5521	30	50	60	60	40,3%	46,6%	75,3%	85,5%	76,4%
5522	30	50	60	65	40,7%	46,3%	75,9%	86,3%	77,6%
5523	30	50	60	70	41,2%	46,0%	76,5%	87,1%	78,8%
5524	30	50	60	75	41,6%	45,7%	77,1%	87,8%	79,9%
5525	30	50	60	80	42,0%	45,3%	77,7%	88,6%	81,0%
5526	30	50	65	20	31,4%	43,5%	67,2%	76,1%	64,2%
5527	30	50	65	25	31,8%	43,2%	67,9%	77,3%	65,8%
5528	30	50	65	30	32,2%	42,9%	68,7%	78,5%	67,3%
5529	30	50	65	35	32,6%	42,6%	69,4%	79,7%	68,8%
5530	30	50	65	40	33,0%	42,2%	70,1%	80,7%	70,3%
5531	30	50	65	45	33,4%	41,9%	70,8%	81,8%	71,7%
5532	30	50	65	50	33,7%	41,6%	71,5%	82,8%	73,0%
5533	30	50	65	55	34,1%	41,3%	72,2%	83,8%	74,4%
5534	30	50	65	60	34,5%	41,0%	72,9%	84,7%	75,6%
5535	30	50	65	65	34,9%	40,7%	73,5%	85,6%	76,9%
5536	30	50	65	70	35,3%	40,3%	74,2%	86,4%	78,1%
5537	30	50	65	75	35,7%	40,0%	74,8%	87,2%	79,2%
5538	30	50	65	80	36,1%	39,7%	75,5%	87,9%	80,3%
5539	30	50	70	20	26,4%	38,0%	64,4%	75,0%	63,3%
5540	30	50	70	25	26,7%	37,6%	65,2%	76,2%	64,9%
5541	30	50	70	30	27,0%	37,3%	65,9%	77,5%	66,4%
5542	30	50	70	35	27,4%	37,0%	66,7%	78,6%	67,9%
5543	30	50	70	40	27,7%	36,7%	67,4%	79,8%	69,4%

5544	30	50	70	45	28,1%	36,4%	68,2%	80,9%	70,8%
5545	30	50	70	50	28,5%	36,1%	68,9%	81,9%	72,2%
5546	30	50	70	55	28,8%	35,8%	69,6%	82,9%	73,6%
5547	30	50	70	60	29,2%	35,5%	70,4%	83,9%	74,9%
5548	30	50	70	65	29,6%	35,2%	71,1%	84,8%	76,1%
5549	30	50	70	70	29,9%	34,9%	71,7%	85,6%	77,4%
5550	30	50	70	75	30,3%	34,6%	72,4%	86,5%	78,5%
5551	30	50	70	80	30,7%	34,3%	73,1%	87,3%	79,7%
5552	30	50	75	20	21,9%	32,7%	61,5%	73,8%	62,3%
5553	30	50	75	25	22,2%	32,4%	62,3%	75,1%	63,9%
5554	30	50	75	30	22,5%	32,1%	63,1%	76,4%	65,5%
5555	30	50	75	35	22,8%	31,8%	63,9%	77,6%	67,0%
5556	30	50	75	40	23,1%	31,5%	64,7%	78,8%	68,5%
5557	30	50	75	45	23,4%	31,3%	65,4%	79,9%	70,0%
5558	30	50	75	50	23,7%	31,0%	66,2%	81,0%	71,4%
5559	30	50	75	55	24,0%	30,7%	66,9%	82,0%	72,8%
5560	30	50	75	60	24,4%	30,4%	67,7%	83,0%	74,1%
5561	30	50	75	65	24,7%	30,1%	68,4%	84,0%	75,4%
5562	30	50	75	70	25,0%	29,9%	69,2%	84,9%	76,6%
5563	30	50	75	75	25,3%	29,6%	69,9%	85,7%	77,8%
5564	30	50	75	80	25,7%	29,3%	70,6%	86,6%	79,0%
5565	30	50	80	20	17,9%	27,8%	58,5%	72,6%	61,3%
5566	30	50	80	25	18,2%	27,6%	59,3%	74,0%	63,0%
5567	30	50	80	30	18,5%	27,3%	60,2%	75,3%	64,5%
5568	30	50	80	35	18,7%	27,0%	61,0%	76,5%	66,1%
5569	30	50	80	40	19,0%	26,8%	61,8%	77,7%	67,6%
5570	30	50	80	45	19,3%	26,5%	62,6%	78,9%	69,1%
5571	30	50	80	50	19,5%	26,3%	63,4%	80,0%	70,5%
5572	30	50	80	55	19,8%	26,0%	64,1%	81,1%	71,9%
5573	30	50	80	60	20,1%	25,8%	64,9%	82,2%	73,3%
5574	30	50	80	65	20,4%	25,5%	65,7%	83,1%	74,6%
5575	30	50	80	70	20,7%	25,3%	66,5%	84,1%	75,9%
5576	30	50	80	75	21,0%	25,0%	67,2%	85,0%	77,1%

5577	30	50	80	80	21,3%	24,8%	67,9%	85,8%	78,3%
5578	30	55	20	20	82,2%	87,0%	85,9%	83,6%	70,3%
5579	30	55	20	25	82,5%	86,8%	86,3%	84,6%	71,7%
5580	30	55	20	30	82,7%	86,7%	86,7%	85,4%	73,1%
5581	30	55	20	35	83,0%	86,5%	87,0%	86,3%	74,4%
5582	30	55	20	40	83,2%	86,3%	87,4%	87,1%	75,7%
5583	30	55	20	45	83,5%	86,2%	87,8%	87,8%	76,9%
5584	30	55	20	50	83,7%	86,0%	88,1%	88,6%	78,1%
5585	30	55	20	55	83,9%	85,9%	88,5%	89,2%	79,3%
5586	30	55	20	60	84,2%	85,7%	88,8%	89,9%	80,4%
5587	30	55	20	65	84,4%	85,6%	89,2%	90,5%	81,4%
5588	30	55	20	70	84,6%	85,4%	89,5%	91,1%	82,4%
5589	30	55	20	75	84,9%	85,2%	89,8%	91,6%	83,4%
5590	30	55	20	80	85,1%	85,1%	90,1%	92,1%	84,3%
5591	30	55	25	20	78,3%	84,1%	84,3%	82,8%	69,4%
5592	30	55	25	25	78,6%	83,9%	84,7%	83,8%	70,9%
5593	30	55	25	30	78,9%	83,7%	85,2%	84,7%	72,3%
5594	30	55	25	35	79,2%	83,6%	85,6%	85,5%	73,6%
5595	30	55	25	40	79,5%	83,4%	86,0%	86,4%	74,9%
5596	30	55	25	45	79,8%	83,2%	86,4%	87,2%	76,2%
5597	30	55	25	50	80,0%	83,0%	86,8%	87,9%	77,4%
5598	30	55	25	55	80,3%	82,8%	87,2%	88,6%	78,6%
5599	30	55	25	60	80,6%	82,6%	87,5%	89,3%	79,7%
5600	30	55	25	65	80,9%	82,5%	87,9%	90,0%	80,8%
5601	30	55	25	70	81,1%	82,3%	88,3%	90,6%	81,8%
5602	30	55	25	75	81,4%	82,1%	88,6%	91,1%	82,8%
5603	30	55	25	80	81,7%	81,9%	89,0%	91,7%	83,8%
5604	30	55	30	20	73,8%	80,8%	82,6%	81,9%	68,5%
5605	30	55	30	25	74,2%	80,6%	83,0%	82,9%	70,0%
5606	30	55	30	30	74,5%	80,4%	83,5%	83,9%	71,4%
5607	30	55	30	35	74,8%	80,1%	84,0%	84,8%	72,8%
5608	30	55	30	40	75,2%	79,9%	84,4%	85,6%	74,1%
5609	30	55	30	45	75,5%	79,7%	84,9%	86,5%	75,4%

5610	30	55	30	50	75,8%	79,5%	85,3%	87,3%	76,7%
5611	30	55	30	55	76,1%	79,3%	85,7%	88,0%	77,9%
5612	30	55	30	60	76,5%	79,1%	86,1%	88,7%	79,0%
5613	30	55	30	65	76,8%	78,9%	86,5%	89,4%	80,1%
5614	30	55	30	70	77,1%	78,6%	86,9%	90,0%	81,2%
5615	30	55	30	75	77,4%	78,4%	87,3%	90,6%	82,2%
5616	30	55	30	80	77,7%	78,2%	87,7%	91,2%	83,2%
5617	30	55	35	20	68,8%	76,9%	80,7%	81,0%	67,6%
5618	30	55	35	25	69,2%	76,7%	81,2%	82,0%	69,1%
5619	30	55	35	30	69,5%	76,5%	81,7%	83,0%	70,6%
5620	30	55	35	35	69,9%	76,2%	82,2%	84,0%	72,0%
5621	30	55	35	40	70,3%	76,0%	82,7%	84,9%	73,3%
5622	30	55	35	45	70,6%	75,7%	83,2%	85,7%	74,6%
5623	30	55	35	50	71,0%	75,5%	83,7%	86,6%	75,9%
5624	30	55	35	55	71,4%	75,3%	84,1%	87,3%	77,2%
5625	30	55	35	60	71,7%	75,0%	84,6%	88,1%	78,3%
5626	30	55	35	65	72,1%	74,8%	85,0%	88,8%	79,5%
5627	30	55	35	70	72,4%	74,5%	85,4%	89,5%	80,6%
5628	30	55	35	75	72,8%	74,3%	85,9%	90,1%	81,6%
5629	30	55	35	80	73,1%	74,0%	86,3%	90,7%	82,6%
5630	30	55	40	20	63,2%	72,6%	78,7%	80,0%	66,7%
5631	30	55	40	25	63,7%	72,3%	79,2%	81,1%	68,2%
5632	30	55	40	30	64,1%	72,0%	79,8%	82,1%	69,7%
5633	30	55	40	35	64,5%	71,8%	80,3%	83,1%	71,1%
5634	30	55	40	40	64,9%	71,5%	80,9%	84,1%	72,5%
5635	30	55	40	45	65,3%	71,2%	81,4%	85,0%	73,9%
5636	30	55	40	50	65,7%	71,0%	81,9%	85,8%	75,2%
5637	30	55	40	55	66,1%	70,7%	82,4%	86,7%	76,4%
5638	30	55	40	60	66,5%	70,4%	82,9%	87,4%	77,6%
5639	30	55	40	65	66,8%	70,2%	83,4%	88,2%	78,8%
5640	30	55	40	70	67,2%	69,9%	83,8%	88,9%	79,9%
5641	30	55	40	75	67,6%	69,6%	84,3%	89,5%	81,0%
5642	30	55	40	80	68,0%	69,3%	84,7%	90,2%	82,0%

5643	30	55	45	20	57,3%	67,7%	76,5%	79,0%	65,8%
5644	30	55	45	25	57,8%	67,5%	77,1%	80,2%	67,3%
5645	30	55	45	30	58,2%	67,2%	77,7%	81,2%	68,8%
5646	30	55	45	35	58,6%	66,9%	78,3%	82,3%	70,3%
5647	30	55	45	40	59,1%	66,6%	78,9%	83,2%	71,7%
5648	30	55	45	45	59,5%	66,3%	79,4%	84,2%	73,1%
5649	30	55	45	50	59,9%	66,0%	80,0%	85,1%	74,4%
5650	30	55	45	55	60,3%	65,7%	80,5%	85,9%	75,7%
5651	30	55	45	60	60,8%	65,4%	81,1%	86,8%	76,9%
5652	30	55	45	65	61,2%	65,1%	81,6%	87,5%	78,1%
5653	30	55	45	70	61,6%	64,8%	82,1%	88,3%	79,2%
5654	30	55	45	75	62,0%	64,5%	82,6%	89,0%	80,4%
5655	30	55	45	80	62,4%	64,2%	83,1%	89,6%	81,4%
5656	30	55	50	20	51,2%	62,5%	74,2%	78,0%	64,9%
5657	30	55	50	25	51,7%	62,2%	74,9%	79,2%	66,4%
5658	30	55	50	30	52,1%	61,9%	75,5%	80,3%	67,9%
5659	30	55	50	35	52,5%	61,6%	76,1%	81,4%	69,4%
5660	30	55	50	40	53,0%	61,3%	76,7%	82,4%	70,8%
5661	30	55	50	45	53,4%	61,0%	77,3%	83,4%	72,2%
5662	30	55	50	50	53,9%	60,6%	77,9%	84,3%	73,6%
5663	30	55	50	55	54,3%	60,3%	78,5%	85,2%	74,9%
5664	30	55	50	60	54,7%	60,0%	79,1%	86,0%	76,2%
5665	30	55	50	65	55,2%	59,7%	79,6%	86,8%	77,4%
5666	30	55	50	70	55,6%	59,4%	80,2%	87,6%	78,6%
5667	30	55	50	75	56,0%	59,1%	80,7%	88,3%	79,7%
5668	30	55	50	80	56,5%	58,8%	81,2%	89,0%	80,8%
5669	30	55	55	20	45,1%	57,0%	71,8%	77,0%	63,9%
5670	30	55	55	25	45,5%	56,6%	72,5%	78,2%	65,5%
5671	30	55	55	30	45,9%	56,3%	73,1%	79,3%	67,0%
5672	30	55	55	35	46,4%	56,0%	73,8%	80,4%	68,5%
5673	30	55	55	40	46,8%	55,7%	74,4%	81,5%	70,0%
5674	30	55	55	45	47,3%	55,3%	75,1%	82,5%	71,4%
5675	30	55	55	50	47,7%	55,0%	75,7%	83,5%	72,8%

5676	30	55	55	55	48,1%	54,7%	76,3%	84,4%	74,1%
5677	30	55	55	60	48,6%	54,4%	76,9%	85,3%	75,4%
5678	30	55	55	65	49,0%	54,0%	77,5%	86,1%	76,7%
5679	30	55	55	70	49,5%	53,7%	78,1%	86,9%	77,9%
5680	30	55	55	75	49,9%	53,4%	78,7%	87,7%	79,0%
5681	30	55	55	80	50,3%	53,1%	79,3%	88,4%	80,1%
5682	30	55	60	20	39,1%	51,2%	69,2%	75,9%	63,0%
5683	30	55	60	25	39,5%	50,9%	69,9%	77,1%	64,6%
5684	30	55	60	30	39,9%	50,6%	70,6%	78,3%	66,1%
5685	30	55	60	35	40,3%	50,2%	71,3%	79,4%	67,6%
5686	30	55	60	40	40,7%	49,9%	72,0%	80,5%	69,1%
5687	30	55	60	45	41,2%	49,6%	72,7%	81,6%	70,6%
5688	30	55	60	50	41,6%	49,3%	73,4%	82,6%	72,0%
5689	30	55	60	55	42,0%	48,9%	74,0%	83,6%	73,3%
5690	30	55	60	60	42,5%	48,6%	74,7%	84,5%	74,6%
5691	30	55	60	65	42,9%	48,3%	75,3%	85,4%	75,9%
5692	30	55	60	70	43,3%	48,0%	75,9%	86,2%	77,1%
5693	30	55	60	75	43,8%	47,6%	76,5%	87,0%	78,3%
5694	30	55	60	80	44,2%	47,3%	77,1%	87,8%	79,5%
5695	30	55	65	20	33,4%	45,5%	66,5%	74,7%	62,0%
5696	30	55	65	25	33,8%	45,1%	67,2%	76,0%	63,6%
5697	30	55	65	30	34,2%	44,8%	68,0%	77,2%	65,2%
5698	30	55	65	35	34,5%	44,5%	68,7%	78,4%	66,7%
5699	30	55	65	40	34,9%	44,2%	69,4%	79,6%	68,2%
5700	30	55	65	45	35,4%	43,8%	70,2%	80,7%	69,7%
5701	30	55	65	50	35,8%	43,5%	70,9%	81,7%	71,1%
5702	30	55	65	55	36,2%	43,2%	71,6%	82,7%	72,5%
5703	30	55	65	60	36,6%	42,9%	72,2%	83,7%	73,8%
5704	30	55	65	65	37,0%	42,6%	72,9%	84,6%	75,1%
5705	30	55	65	70	37,4%	42,2%	73,6%	85,5%	76,4%
5706	30	55	65	75	37,8%	41,9%	74,2%	86,3%	77,6%
5707	30	55	65	80	38,2%	41,6%	74,9%	87,1%	78,8%
5708	30	55	70	20	28,1%	39,8%	63,7%	73,5%	61,0%

5709	30	55	70	25	28,5%	39,5%	64,4%	74,9%	62,7%
5710	30	55	70	30	28,8%	39,2%	65,2%	76,1%	64,2%
5711	30	55	70	35	29,2%	38,9%	66,0%	77,4%	65,8%
5712	30	55	70	40	29,6%	38,6%	66,7%	78,6%	67,3%
5713	30	55	70	45	29,9%	38,3%	67,5%	79,7%	68,8%
5714	30	55	70	50	30,3%	38,0%	68,2%	80,8%	70,3%
5715	30	55	70	55	30,7%	37,7%	69,0%	81,8%	71,7%
5716	30	55	70	60	31,1%	37,3%	69,7%	82,8%	73,0%
5717	30	55	70	65	31,4%	37,0%	70,4%	83,8%	74,4%
5718	30	55	70	70	31,8%	36,7%	71,1%	84,7%	75,7%
5719	30	55	70	75	32,2%	36,4%	71,8%	85,6%	76,9%
5720	30	55	70	80	32,6%	36,1%	72,5%	86,4%	78,1%
5721	30	55	75	20	23,4%	34,4%	60,7%	72,3%	60,0%
5722	30	55	75	25	23,7%	34,1%	61,5%	73,7%	61,7%
5723	30	55	75	30	24,0%	33,9%	62,3%	75,0%	63,3%
5724	30	55	75	35	24,4%	33,6%	63,1%	76,3%	64,9%
5725	30	55	75	40	24,7%	33,3%	63,9%	77,5%	66,4%
5726	30	55	75	45	25,0%	33,0%	64,7%	78,7%	67,9%
5727	30	55	75	50	25,4%	32,7%	65,5%	79,8%	69,4%
5728	30	55	75	55	25,7%	32,4%	66,2%	80,9%	70,8%
5729	30	55	75	60	26,0%	32,1%	67,0%	82,0%	72,2%
5730	30	55	75	65	26,4%	31,8%	67,7%	83,0%	73,6%
5731	30	55	75	70	26,7%	31,5%	68,5%	83,9%	74,9%
5732	30	55	75	75	27,1%	31,3%	69,2%	84,8%	76,2%
5733	30	55	75	80	27,4%	31,0%	69,9%	85,7%	77,4%
5734	30	55	80	20	19,3%	29,4%	57,7%	71,1%	59,1%
5735	30	55	80	25	19,5%	29,2%	58,6%	72,5%	60,7%
5736	30	55	80	30	19,8%	28,9%	59,4%	73,9%	62,3%
5737	30	55	80	35	20,1%	28,6%	60,2%	75,2%	63,9%
5738	30	55	80	40	20,4%	28,4%	61,0%	76,4%	65,5%
5739	30	55	80	45	20,7%	28,1%	61,8%	77,7%	67,0%
5740	30	55	80	50	21,0%	27,8%	62,6%	78,8%	68,5%
5741	30	55	80	55	21,3%	27,6%	63,4%	80,0%	70,0%

5742	30	55	80	60	21,6%	27,3%	64,2%	81,0%	71,4%
5743	30	55	80	65	21,9%	27,0%	65,0%	82,1%	72,8%
5744	30	55	80	70	22,2%	26,8%	65,7%	83,1%	74,1%
5745	30	55	80	75	22,5%	26,5%	66,5%	84,0%	75,4%
5746	30	55	80	80	22,8%	26,3%	67,3%	84,9%	76,6%
5747	30	60	20	20	83,5%	87,8%	85,5%	82,6%	68,3%
5748	30	60	20	25	83,7%	87,7%	85,9%	83,6%	69,7%
5749	30	60	20	30	83,9%	87,5%	86,3%	84,5%	71,2%
5750	30	60	20	35	84,2%	87,4%	86,7%	85,4%	72,5%
5751	30	60	20	40	84,4%	87,2%	87,1%	86,2%	73,9%
5752	30	60	20	45	84,6%	87,1%	87,4%	87,0%	75,2%
5753	30	60	20	50	84,9%	87,0%	87,8%	87,8%	76,4%
5754	30	60	20	55	85,1%	86,8%	88,2%	88,5%	77,6%
5755	30	60	20	60	85,3%	86,7%	88,5%	89,2%	78,8%
5756	30	60	20	65	85,5%	86,5%	88,9%	89,8%	79,9%
5757	30	60	20	70	85,8%	86,3%	89,2%	90,4%	81,0%
5758	30	60	20	75	86,0%	86,2%	89,5%	91,0%	82,0%
5759	30	60	20	80	86,2%	86,0%	89,8%	91,6%	83,0%
5760	30	60	25	20	79,8%	85,1%	83,8%	81,7%	67,4%
5761	30	60	25	25	80,1%	85,0%	84,3%	82,7%	68,8%
5762	30	60	25	30	80,3%	84,8%	84,7%	83,7%	70,3%
5763	30	60	25	35	80,6%	84,6%	85,2%	84,6%	71,7%
5764	30	60	25	40	80,9%	84,4%	85,6%	85,5%	73,1%
5765	30	60	25	45	81,2%	84,3%	86,0%	86,3%	74,4%
5766	30	60	25	50	81,4%	84,1%	86,4%	87,1%	75,7%
5767	30	60	25	55	81,7%	83,9%	86,8%	87,9%	76,9%
5768	30	60	25	60	82,0%	83,7%	87,2%	88,6%	78,1%
5769	30	60	25	65	82,2%	83,6%	87,6%	89,3%	79,3%
5770	30	60	25	70	82,5%	83,4%	87,9%	89,9%	80,4%
5771	30	60	25	75	82,7%	83,2%	88,3%	90,5%	81,4%
5772	30	60	25	80	83,0%	83,0%	88,6%	91,1%	82,4%
5773	30	60	30	20	75,5%	82,0%	82,1%	80,8%	66,4%
5774	30	60	30	25	75,8%	81,8%	82,6%	81,8%	68,0%

5775	30	60	30	30	76,1%	81,6%	83,1%	82,8%	69,4%
5776	30	60	30	35	76,5%	81,4%	83,5%	83,8%	70,9%
5777	30	60	30	40	76,8%	81,2%	84,0%	84,7%	72,3%
5778	30	60	30	45	77,1%	81,0%	84,5%	85,6%	73,6%
5779	30	60	30	50	77,4%	80,8%	84,9%	86,4%	74,9%
5780	30	60	30	55	77,7%	80,6%	85,3%	87,2%	76,2%
5781	30	60	30	60	78,0%	80,4%	85,7%	88,0%	77,4%
5782	30	60	30	65	78,3%	80,2%	86,2%	88,7%	78,6%
5783	30	60	30	70	78,6%	79,9%	86,6%	89,3%	79,7%
5784	30	60	30	75	78,9%	79,7%	86,9%	90,0%	80,8%
5785	30	60	30	80	79,2%	79,5%	87,3%	90,6%	81,8%
5786	30	60	35	20	70,6%	78,3%	80,2%	79,8%	65,5%
5787	30	60	35	25	71,0%	78,1%	80,7%	80,9%	67,1%
5788	30	60	35	30	71,4%	77,8%	81,3%	82,0%	68,5%
5789	30	60	35	35	71,7%	77,6%	81,8%	83,0%	70,0%
5790	30	60	35	40	72,1%	77,4%	82,3%	83,9%	71,4%
5791	30	60	35	45	72,4%	77,2%	82,8%	84,8%	72,8%
5792	30	60	35	50	72,8%	76,9%	83,2%	85,7%	74,1%
5793	30	60	35	55	73,1%	76,7%	83,7%	86,5%	75,4%
5794	30	60	35	60	73,5%	76,5%	84,2%	87,3%	76,7%
5795	30	60	35	65	73,8%	76,2%	84,6%	88,0%	77,9%
5796	30	60	35	70	74,2%	76,0%	85,0%	88,7%	79,0%
5797	30	60	35	75	74,5%	75,7%	85,5%	89,4%	80,1%
5798	30	60	35	80	74,8%	75,5%	85,9%	90,1%	81,2%
5799	30	60	40	20	65,3%	74,1%	78,1%	78,8%	64,6%
5800	30	60	40	25	65,7%	73,9%	78,7%	80,0%	66,1%
5801	30	60	40	30	66,1%	73,6%	79,3%	81,0%	67,6%
5802	30	60	40	35	66,5%	73,3%	79,8%	82,1%	69,1%
5803	30	60	40	40	66,9%	73,1%	80,4%	83,1%	70,6%
5804	30	60	40	45	67,3%	72,8%	80,9%	84,0%	72,0%
5805	30	60	40	50	67,6%	72,6%	81,4%	84,9%	73,3%
5806	30	60	40	55	68,0%	72,3%	81,9%	85,8%	74,7%
5807	30	60	40	60	68,4%	72,0%	82,4%	86,6%	75,9%

5808	30	60	40	65	68,8%	71,8%	82,9%	87,4%	77,2%
5809	30	60	40	70	69,2%	71,5%	83,4%	88,1%	78,3%
5810	30	60	40	75	69,5%	71,3%	83,9%	88,8%	79,5%
5811	30	60	40	80	69,9%	71,0%	84,3%	89,5%	80,6%
5812	30	60	45	20	59,5%	69,4%	76,0%	77,8%	63,6%
5813	30	60	45	25	59,9%	69,2%	76,6%	79,0%	65,2%
5814	30	60	45	30	60,3%	68,9%	77,2%	80,1%	66,7%
5815	30	60	45	35	60,8%	68,6%	77,8%	81,2%	68,2%
5816	30	60	45	40	61,2%	68,3%	78,3%	82,2%	69,7%
5817	30	60	45	45	61,6%	68,0%	78,9%	83,2%	71,1%
5818	30	60	45	50	62,0%	67,7%	79,5%	84,1%	72,5%
5819	30	60	45	55	62,4%	67,5%	80,0%	85,0%	73,9%
5820	30	60	45	60	62,8%	67,2%	80,6%	85,9%	75,2%
5821	30	60	45	65	63,3%	66,9%	81,1%	86,7%	76,4%
5822	30	60	45	70	63,7%	66,6%	81,6%	87,5%	77,6%
5823	30	60	45	75	64,1%	66,3%	82,1%	88,2%	78,8%
5824	30	60	45	80	64,5%	66,0%	82,6%	88,9%	79,9%
5825	30	60	50	20	53,4%	64,3%	73,6%	76,7%	62,7%
5826	30	60	50	25	53,9%	64,0%	74,3%	77,9%	64,3%
5827	30	60	50	30	54,3%	63,7%	74,9%	79,1%	65,8%
5828	30	60	50	35	54,7%	63,4%	75,5%	80,2%	67,3%
5829	30	60	50	40	55,2%	63,1%	76,2%	81,3%	68,8%
5830	30	60	50	45	55,6%	62,8%	76,8%	82,3%	70,3%
5831	30	60	50	50	56,1%	62,5%	77,4%	83,3%	71,7%
5832	30	60	50	55	56,5%	62,2%	78,0%	84,2%	73,1%
5833	30	60	50	60	56,9%	61,9%	78,5%	85,1%	74,4%
5834	30	60	50	65	57,4%	61,6%	79,1%	86,0%	75,7%
5835	30	60	50	70	57,8%	61,3%	79,7%	86,8%	76,9%
5836	30	60	50	75	58,2%	61,0%	80,2%	87,6%	78,1%
5837	30	60	50	80	58,6%	60,7%	80,7%	88,3%	79,3%
5838	30	60	55	20	47,3%	58,9%	71,1%	75,6%	61,7%
5839	30	60	55	25	47,7%	58,6%	71,8%	76,9%	63,3%
5840	30	60	55	30	48,2%	58,2%	72,5%	78,1%	64,9%

5841	30	60	55	35	48,6%	57,9%	73,2%	79,2%	66,4%
5842	30	60	55	40	49,0%	57,6%	73,8%	80,3%	67,9%
5843	30	60	55	45	49,5%	57,3%	74,5%	81,4%	69,4%
5844	30	60	55	50	49,9%	57,0%	75,1%	82,4%	70,8%
5845	30	60	55	55	50,4%	56,6%	75,8%	83,4%	72,2%
5846	30	60	55	60	50,8%	56,3%	76,4%	84,3%	73,6%
5847	30	60	55	65	51,2%	56,0%	77,0%	85,2%	74,9%
5848	30	60	55	70	51,7%	55,7%	77,6%	86,1%	76,2%
5849	30	60	55	75	52,1%	55,4%	78,2%	86,9%	77,4%
5850	30	60	55	80	52,6%	55,0%	78,7%	87,6%	78,6%
5851	30	60	60	20	41,2%	53,2%	68,5%	74,5%	60,7%
5852	30	60	60	25	41,6%	52,9%	69,2%	75,8%	62,3%
5853	30	60	60	30	42,0%	52,5%	70,0%	77,0%	63,9%
5854	30	60	60	35	42,5%	52,2%	70,7%	78,2%	65,5%
5855	30	60	60	40	42,9%	51,9%	71,4%	79,4%	67,0%
5856	30	60	60	45	43,3%	51,6%	72,1%	80,5%	68,5%
5857	30	60	60	50	43,8%	51,2%	72,7%	81,5%	70,0%
5858	30	60	60	55	44,2%	50,9%	73,4%	82,5%	71,4%
5859	30	60	60	60	44,6%	50,6%	74,1%	83,5%	72,8%
5860	30	60	60	65	45,1%	50,3%	74,7%	84,4%	74,1%
5861	30	60	60	70	45,5%	49,9%	75,3%	85,3%	75,4%
5862	30	60	60	75	46,0%	49,6%	76,0%	86,2%	76,7%
5863	30	60	60	80	46,4%	49,3%	76,6%	87,0%	77,9%
5864	30	60	65	20	35,4%	47,4%	65,8%	73,3%	59,7%
5865	30	60	65	25	35,8%	47,1%	66,5%	74,6%	61,4%
5866	30	60	65	30	36,2%	46,8%	67,3%	75,9%	63,0%
5867	30	60	65	35	36,6%	46,4%	68,0%	77,1%	64,6%
5868	30	60	65	40	37,0%	46,1%	68,8%	78,3%	66,1%
5869	30	60	65	45	37,4%	45,8%	69,5%	79,5%	67,6%
5870	30	60	65	50	37,8%	45,5%	70,2%	80,6%	69,1%
5871	30	60	65	55	38,2%	45,1%	70,9%	81,6%	70,6%
5872	30	60	65	60	38,6%	44,8%	71,6%	82,7%	72,0%
5873	30	60	65	65	39,1%	44,5%	72,3%	83,6%	73,3%

5874	30	60	65	70	39,5%	44,2%	73,0%	84,5%	74,6%
5875	30	60	65	75	39,9%	43,9%	73,6%	85,4%	75,9%
5876	30	60	65	80	40,3%	43,5%	74,3%	86,3%	77,1%
5877	30	60	70	20	29,9%	41,7%	62,9%	72,1%	58,7%
5878	30	60	70	25	30,3%	41,4%	63,7%	73,4%	60,4%
5879	30	60	70	30	30,7%	41,1%	64,5%	74,8%	62,0%
5880	30	60	70	35	31,1%	40,8%	65,3%	76,1%	63,6%
5881	30	60	70	40	31,4%	40,5%	66,0%	77,3%	65,2%
5882	30	60	70	45	31,8%	40,1%	66,8%	78,5%	66,7%
5883	30	60	70	50	32,2%	39,8%	67,5%	79,6%	68,2%
5884	30	60	70	55	32,6%	39,5%	68,3%	80,7%	69,7%
5885	30	60	70	60	33,0%	39,2%	69,0%	81,8%	71,1%
5886	30	60	70	65	33,4%	38,9%	69,7%	82,8%	72,5%
5887	30	60	70	70	33,8%	38,6%	70,4%	83,7%	73,9%
5888	30	60	70	75	34,2%	38,3%	71,1%	84,6%	75,2%
5889	30	60	70	80	34,6%	38,0%	71,8%	85,5%	76,4%
5890	30	60	75	20	25,0%	36,2%	60,0%	70,8%	57,7%
5891	30	60	75	25	25,4%	35,9%	60,8%	72,2%	59,4%
5892	30	60	75	30	25,7%	35,6%	61,6%	73,6%	61,0%
5893	30	60	75	35	26,0%	35,3%	62,4%	74,9%	62,7%
5894	30	60	75	40	26,4%	35,0%	63,2%	76,2%	64,2%
5895	30	60	75	45	26,7%	34,7%	64,0%	77,4%	65,8%
5896	30	60	75	50	27,1%	34,4%	64,8%	78,6%	67,3%
5897	30	60	75	55	27,4%	34,2%	65,5%	79,7%	68,8%
5898	30	60	75	60	27,8%	33,9%	66,3%	80,8%	70,3%
5899	30	60	75	65	28,1%	33,6%	67,0%	81,9%	71,7%
5900	30	60	75	70	28,5%	33,3%	67,8%	82,9%	73,0%
5901	30	60	75	75	28,8%	33,0%	68,5%	83,8%	74,4%
5902	30	60	75	80	29,2%	32,7%	69,3%	84,8%	75,7%
5903	30	60	80	20	20,7%	31,1%	57,0%	69,6%	56,7%
5904	30	60	80	25	21,0%	30,8%	57,8%	71,0%	58,4%
5905	30	60	80	30	21,3%	30,5%	58,6%	72,4%	60,1%
5906	30	60	80	35	21,6%	30,3%	59,4%	73,8%	61,7%

5907	30	60	80	40	21,9%	30,0%	60,3%	75,1%	63,3%
5908	30	60	80	45	22,2%	29,7%	61,1%	76,3%	64,9%
5909	30	60	80	50	22,5%	29,4%	61,9%	77,6%	66,4%
5910	30	60	80	55	22,8%	29,2%	62,7%	78,7%	67,9%
5911	30	60	80	60	23,1%	28,9%	63,5%	79,9%	69,4%
5912	30	60	80	65	23,4%	28,6%	64,2%	81,0%	70,8%
5913	30	60	80	70	23,7%	28,4%	65,0%	82,0%	72,2%
5914	30	60	80	75	24,1%	28,1%	65,8%	83,0%	73,6%
5915	30	60	80	80	24,4%	27,8%	66,6%	83,9%	74,9%
5916	30	65	20	20	84,7%	88,6%	85,1%	81,5%	66,2%
5917	30	65	20	25	84,9%	88,5%	85,5%	82,5%	67,7%
5918	30	65	20	30	85,1%	88,4%	85,9%	83,5%	69,2%
5919	30	65	20	35	85,3%	88,2%	86,3%	84,4%	70,6%
5920	30	65	20	40	85,5%	88,1%	86,7%	85,3%	72,0%
5921	30	65	20	45	85,8%	88,0%	87,1%	86,2%	73,4%
5922	30	65	20	50	86,0%	87,8%	87,5%	87,0%	74,7%
5923	30	65	20	55	86,2%	87,7%	87,8%	87,7%	75,9%
5924	30	65	20	60	86,4%	87,5%	88,2%	88,5%	77,2%
5925	30	65	20	65	86,6%	87,4%	88,5%	89,1%	78,4%
5926	30	65	20	70	86,8%	87,3%	88,9%	89,8%	79,5%
5927	30	65	20	75	87,0%	87,1%	89,2%	90,4%	80,6%
5928	30	65	20	80	87,2%	87,0%	89,5%	91,0%	81,6%
5929	30	65	25	20	81,2%	86,1%	83,4%	80,6%	65,2%
5930	30	65	25	25	81,4%	85,9%	83,9%	81,6%	66,8%
5931	30	65	25	30	81,7%	85,8%	84,3%	82,7%	68,3%
5932	30	65	25	35	82,0%	85,6%	84,8%	83,6%	69,7%
5933	30	65	25	40	82,2%	85,5%	85,2%	84,5%	71,2%
5934	30	65	25	45	82,5%	85,3%	85,6%	85,4%	72,5%
5935	30	65	25	50	82,7%	85,1%	86,0%	86,3%	73,9%
5936	30	65	25	55	83,0%	85,0%	86,4%	87,1%	75,2%
5937	30	65	25	60	83,2%	84,8%	86,8%	87,8%	76,4%
5938	30	65	25	65	83,5%	84,6%	87,2%	88,5%	77,7%
5939	30	65	25	70	83,7%	84,5%	87,6%	89,2%	78,8%

5940	30	65	25	75	84,0%	84,3%	88,0%	89,9%	79,9%
5941	30	65	25	80	84,2%	84,1%	88,3%	90,5%	81,0%
5942	30	65	30	20	77,1%	83,1%	81,6%	79,6%	64,3%
5943	30	65	30	25	77,4%	82,9%	82,1%	80,7%	65,8%
5944	30	65	30	30	77,7%	82,7%	82,6%	81,8%	67,4%
5945	30	65	30	35	78,0%	82,5%	83,1%	82,8%	68,9%
5946	30	65	30	40	78,3%	82,3%	83,6%	83,7%	70,3%
5947	30	65	30	45	78,6%	82,2%	84,0%	84,6%	71,7%
5948	30	65	30	50	78,9%	82,0%	84,5%	85,5%	73,1%
5949	30	65	30	55	79,2%	81,8%	84,9%	86,4%	74,4%
5950	30	65	30	60	79,5%	81,6%	85,4%	87,1%	75,7%
5951	30	65	30	65	79,8%	81,4%	85,8%	87,9%	76,9%
5952	30	65	30	70	80,1%	81,2%	86,2%	88,6%	78,1%
5953	30	65	30	75	80,3%	81,0%	86,6%	89,3%	79,3%
5954	30	65	30	80	80,6%	80,8%	87,0%	89,9%	80,4%
5955	30	65	35	20	72,5%	79,6%	79,7%	78,6%	63,3%
5956	30	65	35	25	72,8%	79,4%	80,2%	79,7%	64,9%
5957	30	65	35	30	73,2%	79,2%	80,8%	80,8%	66,5%
5958	30	65	35	35	73,5%	79,0%	81,3%	81,9%	68,0%
5959	30	65	35	40	73,8%	78,7%	81,8%	82,9%	69,4%
5960	30	65	35	45	74,2%	78,5%	82,3%	83,8%	70,9%
5961	30	65	35	50	74,5%	78,3%	82,8%	84,7%	72,3%
5962	30	65	35	55	74,8%	78,1%	83,3%	85,6%	73,6%
5963	30	65	35	60	75,2%	77,8%	83,7%	86,4%	74,9%
5964	30	65	35	65	75,5%	77,6%	84,2%	87,2%	76,2%
5965	30	65	35	70	75,8%	77,4%	84,6%	88,0%	77,4%
5966	30	65	35	75	76,2%	77,2%	85,1%	88,7%	78,6%
5967	30	65	35	80	76,5%	76,9%	85,5%	89,4%	79,7%
5968	30	65	40	20	67,3%	75,6%	77,6%	77,6%	62,4%
5969	30	65	40	25	67,7%	75,3%	78,2%	78,7%	64,0%
5970	30	65	40	30	68,0%	75,1%	78,8%	79,9%	65,5%
5971	30	65	40	35	68,4%	74,9%	79,3%	81,0%	67,1%
5972	30	65	40	40	68,8%	74,6%	79,9%	82,0%	68,6%

5973	30	65	40	45	69,2%	74,4%	80,4%	83,0%	70,0%
5974	30	65	40	50	69,5%	74,1%	80,9%	83,9%	71,4%
5975	30	65	40	55	69,9%	73,9%	81,5%	84,9%	72,8%
5976	30	65	40	60	70,3%	73,6%	82,0%	85,7%	74,1%
5977	30	65	40	65	70,7%	73,4%	82,5%	86,5%	75,4%
5978	30	65	40	70	71,0%	73,1%	82,9%	87,3%	76,7%
5979	30	65	40	75	71,4%	72,8%	83,4%	88,1%	77,9%
5980	30	65	40	80	71,7%	72,6%	83,9%	88,8%	79,0%
5981	30	65	45	20	61,6%	71,1%	75,4%	76,5%	61,4%
5982	30	65	45	25	62,0%	70,8%	76,0%	77,7%	63,0%
5983	30	65	45	30	62,4%	70,5%	76,6%	78,9%	64,6%
5984	30	65	45	35	62,9%	70,3%	77,2%	80,0%	66,1%
5985	30	65	45	40	63,3%	70,0%	77,8%	81,1%	67,7%
5986	30	65	45	45	63,7%	69,7%	78,4%	82,1%	69,1%
5987	30	65	45	50	64,1%	69,4%	79,0%	83,1%	70,6%
5988	30	65	45	55	64,5%	69,2%	79,5%	84,1%	72,0%
5989	30	65	45	60	64,9%	68,9%	80,1%	85,0%	73,3%
5990	30	65	45	65	65,3%	68,6%	80,6%	85,8%	74,7%
5991	30	65	45	70	65,7%	68,3%	81,1%	86,6%	75,9%
5992	30	65	45	75	66,1%	68,0%	81,6%	87,4%	77,2%
5993	30	65	45	80	66,5%	67,8%	82,1%	88,2%	78,3%
5994	30	65	50	20	55,6%	66,1%	73,0%	75,4%	60,4%
5995	30	65	50	25	56,1%	65,8%	73,7%	76,6%	62,0%
5996	30	65	50	30	56,5%	65,5%	74,3%	77,8%	63,6%
5997	30	65	50	35	56,9%	65,2%	74,9%	79,0%	65,2%
5998	30	65	50	40	57,4%	64,9%	75,6%	80,1%	66,7%
5999	30	65	50	45	57,8%	64,6%	76,2%	81,2%	68,2%
6000	30	65	50	50	58,2%	64,3%	76,8%	82,2%	69,7%
6001	30	65	50	55	58,7%	64,0%	77,4%	83,2%	71,1%
6002	30	65	50	60	59,1%	63,7%	78,0%	84,2%	72,5%
6003	30	65	50	65	59,5%	63,4%	78,6%	85,1%	73,9%
6004	30	65	50	70	59,9%	63,1%	79,1%	85,9%	75,2%
6005	30	65	50	75	60,4%	62,8%	79,7%	86,7%	76,4%

6006	30	65	50	80	60,8%	62,5%	80,2%	87,5%	77,6%
6007	30	65	55	20	49,5%	60,8%	70,5%	74,2%	59,4%
6008	30	65	55	25	49,9%	60,5%	71,2%	75,5%	61,1%
6009	30	65	55	30	50,4%	60,1%	71,9%	76,8%	62,7%
6010	30	65	55	35	50,8%	59,8%	72,5%	78,0%	64,3%
6011	30	65	55	40	51,2%	59,5%	73,2%	79,1%	65,8%
6012	30	65	55	45	51,7%	59,2%	73,9%	80,3%	67,3%
6013	30	65	55	50	52,1%	58,9%	74,5%	81,3%	68,8%
6014	30	65	55	55	52,6%	58,6%	75,2%	82,3%	70,3%
6015	30	65	55	60	53,0%	58,2%	75,8%	83,3%	71,7%
6016	30	65	55	65	53,4%	57,9%	76,4%	84,3%	73,1%
6017	30	65	55	70	53,9%	57,6%	77,0%	85,2%	74,4%
6018	30	65	55	75	54,3%	57,3%	77,6%	86,0%	75,7%
6019	30	65	55	80	54,8%	57,0%	78,2%	86,8%	76,9%
6020	30	65	60	20	43,4%	55,1%	67,8%	73,0%	58,4%
6021	30	65	60	25	43,8%	54,8%	68,6%	74,4%	60,1%
6022	30	65	60	30	44,2%	54,5%	69,3%	75,7%	61,7%
6023	30	65	60	35	44,7%	54,2%	70,0%	76,9%	63,3%
6024	30	65	60	40	45,1%	53,8%	70,7%	78,1%	64,9%
6025	30	65	60	45	45,5%	53,5%	71,4%	79,3%	66,4%
6026	30	65	60	50	46,0%	53,2%	72,1%	80,4%	67,9%
6027	30	65	60	55	46,4%	52,9%	72,8%	81,4%	69,4%
6028	30	65	60	60	46,8%	52,5%	73,4%	82,5%	70,9%
6029	30	65	60	65	47,3%	52,2%	74,1%	83,4%	72,2%
6030	30	65	60	70	47,7%	51,9%	74,7%	84,4%	73,6%
6031	30	65	60	75	48,2%	51,6%	75,4%	85,3%	74,9%
6032	30	65	60	80	48,6%	51,2%	76,0%	86,1%	76,2%
6033	30	65	65	20	37,4%	49,4%	65,1%	71,8%	57,4%
6034	30	65	65	25	37,8%	49,1%	65,8%	73,2%	59,1%
6035	30	65	65	30	38,2%	48,7%	66,6%	74,5%	60,7%
6036	30	65	65	35	38,7%	48,4%	67,3%	75,8%	62,4%
6037	30	65	65	40	39,1%	48,1%	68,1%	77,1%	63,9%
6038	30	65	65	45	39,5%	47,8%	68,8%	78,2%	65,5%

6039	30	65	65	50	39,9%	47,4%	69,5%	79,4%	67,0%
6040	30	65	65	55	40,3%	47,1%	70,2%	80,5%	68,5%
6041	30	65	65	60	40,8%	46,8%	71,0%	81,6%	70,0%
6042	30	65	65	65	41,2%	46,5%	71,6%	82,6%	71,4%
6043	30	65	65	70	41,6%	46,1%	72,3%	83,6%	72,8%
6044	30	65	65	75	42,1%	45,8%	73,0%	84,5%	74,1%
6045	30	65	65	80	42,5%	45,5%	73,7%	85,4%	75,4%
6046	30	65	70	20	31,8%	43,7%	62,2%	70,6%	56,4%
6047	30	65	70	25	32,2%	43,3%	63,0%	72,0%	58,1%
6048	30	65	70	30	32,6%	43,0%	63,8%	73,3%	59,7%
6049	30	65	70	35	33,0%	42,7%	64,5%	74,7%	61,4%
6050	30	65	70	40	33,4%	42,4%	65,3%	76,0%	63,0%
6051	30	65	70	45	33,8%	42,1%	66,1%	77,2%	64,6%
6052	30	65	70	50	34,2%	41,7%	66,8%	78,4%	66,1%
6053	30	65	70	55	34,6%	41,4%	67,6%	79,5%	67,6%
6054	30	65	70	60	35,0%	41,1%	68,3%	80,6%	69,1%
6055	30	65	70	65	35,4%	40,8%	69,1%	81,7%	70,6%
6056	30	65	70	70	35,8%	40,5%	69,8%	82,7%	72,0%
6057	30	65	70	75	36,2%	40,2%	70,5%	83,7%	73,3%
6058	30	65	70	80	36,6%	39,8%	71,2%	84,6%	74,6%
6059	30	65	75	20	26,7%	38,1%	59,2%	69,3%	55,4%
6060	30	65	75	25	27,1%	37,8%	60,0%	70,7%	57,1%
6061	30	65	75	30	27,4%	37,5%	60,8%	72,1%	58,7%
6062	30	65	75	35	27,8%	37,2%	61,6%	73,5%	60,4%
6063	30	65	75	40	28,1%	36,9%	62,4%	74,8%	62,0%
6064	30	65	75	45	28,5%	36,5%	63,2%	76,1%	63,6%
6065	30	65	75	50	28,9%	36,2%	64,0%	77,3%	65,2%
6066	30	65	75	55	29,2%	35,9%	64,8%	78,5%	66,7%
6067	30	65	75	60	29,6%	35,6%	65,6%	79,7%	68,2%
6068	30	65	75	65	30,0%	35,3%	66,3%	80,8%	69,7%
6069	30	65	75	70	30,3%	35,0%	67,1%	81,8%	71,1%
6070	30	65	75	75	30,7%	34,7%	67,8%	82,8%	72,5%
6071	30	65	75	80	31,1%	34,5%	68,6%	83,8%	73,9%

6072	30	65	80	20	22,2%	32,8%	56,2%	68,0%	54,4%
6073	30	65	80	25	22,5%	32,5%	57,0%	69,5%	56,1%
6074	30	65	80	30	22,8%	32,2%	57,8%	70,9%	57,7%
6075	30	65	80	35	23,1%	31,9%	58,7%	72,3%	59,4%
6076	30	65	80	40	23,4%	31,7%	59,5%	73,7%	61,0%
6077	30	65	80	45	23,7%	31,4%	60,3%	75,0%	62,7%
6078	30	65	80	50	24,1%	31,1%	61,1%	76,2%	64,3%
6079	30	65	80	55	24,4%	30,8%	61,9%	77,5%	65,8%
6080	30	65	80	60	24,7%	30,5%	62,7%	78,7%	67,3%
6081	30	65	80	65	25,0%	30,3%	63,5%	79,8%	68,8%
6082	30	65	80	70	25,4%	30,0%	64,3%	80,9%	70,3%
6083	30	65	80	75	25,7%	29,7%	65,1%	81,9%	71,7%
6084	30	65	80	80	26,0%	29,4%	65,8%	82,9%	73,1%
6085	30	70	20	20	85,8%	89,4%	84,7%	80,4%	64,0%
6086	30	70	20	25	86,0%	89,3%	85,1%	81,4%	65,5%
6087	30	70	20	30	86,2%	89,2%	85,5%	82,5%	67,1%
6088	30	70	20	35	86,4%	89,0%	85,9%	83,4%	68,6%
6089	30	70	20	40	86,6%	88,9%	86,3%	84,4%	70,0%
6090	30	70	20	45	86,8%	88,8%	86,7%	85,3%	71,4%
6091	30	70	20	50	87,0%	88,6%	87,1%	86,1%	72,8%
6092	30	70	20	55	87,2%	88,5%	87,5%	86,9%	74,2%
6093	30	70	20	60	87,4%	88,4%	87,9%	87,7%	75,4%
6094	30	70	20	65	87,6%	88,2%	88,2%	88,4%	76,7%
6095	30	70	20	70	87,8%	88,1%	88,6%	89,1%	77,9%
6096	30	70	20	75	88,0%	88,0%	88,9%	89,7%	79,1%
6097	30	70	20	80	88,2%	87,8%	89,2%	90,4%	80,2%
6098	30	70	25	20	82,5%	87,0%	83,0%	79,4%	63,0%
6099	30	70	25	25	82,7%	86,9%	83,4%	80,5%	64,6%
6100	30	70	25	30	83,0%	86,7%	83,9%	81,6%	66,2%
6101	30	70	25	35	83,2%	86,6%	84,4%	82,6%	67,7%
6102	30	70	25	40	83,5%	86,4%	84,8%	83,5%	69,2%
6103	30	70	25	45	83,7%	86,3%	85,2%	84,5%	70,6%
6104	30	70	25	50	84,0%	86,1%	85,7%	85,4%	72,0%

6105	30	70	25	55	84,2%	85,9%	86,1%	86,2%	73,4%
6106	30	70	25	60	84,4%	85,8%	86,5%	87,0%	74,7%
6107	30	70	25	65	84,7%	85,6%	86,9%	87,8%	76,0%
6108	30	70	25	70	84,9%	85,5%	87,2%	88,5%	77,2%
6109	30	70	25	75	85,1%	85,3%	87,6%	89,2%	78,4%
6110	30	70	25	80	85,3%	85,1%	88,0%	89,8%	79,5%
6111	30	70	30	20	78,6%	84,2%	81,1%	78,4%	62,1%
6112	30	70	30	25	78,9%	84,0%	81,7%	79,5%	63,7%
6113	30	70	30	30	79,2%	83,8%	82,2%	80,6%	65,2%
6114	30	70	30	35	79,5%	83,6%	82,7%	81,7%	66,8%
6115	30	70	30	40	79,8%	83,5%	83,1%	82,7%	68,3%
6116	30	70	30	45	80,1%	83,3%	83,6%	83,7%	69,7%
6117	30	70	30	50	80,4%	83,1%	84,1%	84,6%	71,2%
6118	30	70	30	55	80,6%	82,9%	84,5%	85,5%	72,5%
6119	30	70	30	60	80,9%	82,7%	85,0%	86,3%	73,9%
6120	30	70	30	65	81,2%	82,5%	85,4%	87,1%	75,2%
6121	30	70	30	70	81,4%	82,3%	85,8%	87,8%	76,4%
6122	30	70	30	75	81,7%	82,2%	86,2%	88,6%	77,7%
6123	30	70	30	80	82,0%	82,0%	86,6%	89,2%	78,8%
6124	30	70	35	20	74,2%	80,8%	79,2%	77,3%	61,1%
6125	30	70	35	25	74,5%	80,6%	79,7%	78,5%	62,7%
6126	30	70	35	30	74,9%	80,4%	80,3%	79,7%	64,3%
6127	30	70	35	35	75,2%	80,2%	80,8%	80,8%	65,8%
6128	30	70	35	40	75,5%	80,0%	81,3%	81,8%	67,4%
6129	30	70	35	45	75,8%	79,8%	81,8%	82,8%	68,9%
6130	30	70	35	50	76,2%	79,6%	82,3%	83,8%	70,3%
6131	30	70	35	55	76,5%	79,4%	82,8%	84,7%	71,7%
6132	30	70	35	60	76,8%	79,2%	83,3%	85,6%	73,1%
6133	30	70	35	65	77,1%	79,0%	83,8%	86,4%	74,4%
6134	30	70	35	70	77,4%	78,7%	84,2%	87,2%	75,7%
6135	30	70	35	75	77,7%	78,5%	84,7%	87,9%	76,9%
6136	30	70	35	80	78,0%	78,3%	85,1%	88,6%	78,1%
6137	30	70	40	20	69,2%	77,0%	77,0%	76,2%	60,1%

6138	30	70	40	25	69,6%	76,8%	77,6%	77,5%	61,7%
6139	30	70	40	30	69,9%	76,5%	78,2%	78,7%	63,3%
6140	30	70	40	35	70,3%	76,3%	78,8%	79,8%	64,9%
6141	30	70	40	40	70,7%	76,1%	79,4%	80,9%	66,5%
6142	30	70	40	45	71,0%	75,8%	79,9%	81,9%	68,0%
6143	30	70	40	50	71,4%	75,6%	80,4%	82,9%	69,4%
6144	30	70	40	55	71,8%	75,4%	81,0%	83,9%	70,9%
6145	30	70	40	60	72,1%	75,1%	81,5%	84,8%	72,3%
6146	30	70	40	65	72,5%	74,9%	82,0%	85,7%	73,6%
6147	30	70	40	70	72,8%	74,6%	82,5%	86,5%	74,9%
6148	30	70	40	75	73,2%	74,4%	83,0%	87,3%	76,2%
6149	30	70	40	80	73,5%	74,1%	83,5%	88,0%	77,4%
6150	30	70	45	20	63,7%	72,7%	74,8%	75,1%	59,1%
6151	30	70	45	25	64,1%	72,4%	75,4%	76,4%	60,8%
6152	30	70	45	30	64,5%	72,2%	76,0%	77,6%	62,4%
6153	30	70	45	35	64,9%	71,9%	76,6%	78,8%	64,0%
6154	30	70	45	40	65,3%	71,6%	77,2%	79,9%	65,5%
6155	30	70	45	45	65,7%	71,4%	77,8%	81,0%	67,1%
6156	30	70	45	50	66,1%	71,1%	78,4%	82,0%	68,6%
6157	30	70	45	55	66,5%	70,8%	79,0%	83,0%	70,0%
6158	30	70	45	60	66,9%	70,5%	79,5%	84,0%	71,4%
6159	30	70	45	65	67,3%	70,3%	80,1%	84,9%	72,8%
6160	30	70	45	70	67,7%	70,0%	80,6%	85,8%	74,1%
6161	30	70	45	75	68,0%	69,7%	81,2%	86,6%	75,4%
6162	30	70	45	80	68,4%	69,4%	81,7%	87,4%	76,7%
6163	30	70	50	20	57,8%	67,9%	72,4%	74,0%	58,1%
6164	30	70	50	25	58,2%	67,6%	73,0%	75,3%	59,8%
6165	30	70	50	30	58,7%	67,3%	73,7%	76,5%	61,4%
6166	30	70	50	35	59,1%	67,0%	74,3%	77,7%	63,0%
6167	30	70	50	40	59,5%	66,7%	75,0%	78,9%	64,6%
6168	30	70	50	45	59,9%	66,4%	75,6%	80,0%	66,1%
6169	30	70	50	50	60,4%	66,1%	76,2%	81,1%	67,7%
6170	30	70	50	55	60,8%	65,8%	76,9%	82,2%	69,1%

6171	30	70	50	60	61,2%	65,5%	77,5%	83,1%	70,6%
6172	30	70	50	65	61,6%	65,2%	78,0%	84,1%	72,0%
6173	30	70	50	70	62,0%	64,9%	78,6%	85,0%	73,3%
6174	30	70	50	75	62,5%	64,6%	79,2%	85,9%	74,7%
6175	30	70	50	80	62,9%	64,3%	79,7%	86,7%	75,9%
6176	30	70	55	20	51,7%	62,6%	69,8%	72,8%	57,1%
6177	30	70	55	25	52,1%	62,3%	70,5%	74,1%	58,8%
6178	30	70	55	30	52,6%	62,0%	71,2%	75,4%	60,4%
6179	30	70	55	35	53,0%	61,7%	71,9%	76,7%	62,0%
6180	30	70	55	40	53,5%	61,4%	72,6%	77,9%	63,6%
6181	30	70	55	45	53,9%	61,1%	73,3%	79,1%	65,2%
6182	30	70	55	50	54,3%	60,8%	73,9%	80,2%	66,8%
6183	30	70	55	55	54,8%	60,5%	74,6%	81,2%	68,3%
6184	30	70	55	60	55,2%	60,1%	75,2%	82,3%	69,7%
6185	30	70	55	65	55,6%	59,8%	75,8%	83,3%	71,1%
6186	30	70	55	70	56,1%	59,5%	76,5%	84,2%	72,5%
6187	30	70	55	75	56,5%	59,2%	77,1%	85,1%	73,9%
6188	30	70	55	80	56,9%	58,9%	77,7%	85,9%	75,2%
6189	30	70	60	20	45,5%	57,1%	67,1%	71,6%	56,1%
6190	30	70	60	25	46,0%	56,8%	67,9%	72,9%	57,8%
6191	30	70	60	30	46,4%	56,4%	68,6%	74,3%	59,4%
6192	30	70	60	35	46,9%	56,1%	69,3%	75,6%	61,1%
6193	30	70	60	40	47,3%	55,8%	70,0%	76,8%	62,7%
6194	30	70	60	45	47,7%	55,5%	70,8%	78,0%	64,3%
6195	30	70	60	50	48,2%	55,2%	71,5%	79,2%	65,8%
6196	30	70	60	55	48,6%	54,8%	72,1%	80,3%	67,4%
6197	30	70	60	60	49,1%	54,5%	72,8%	81,4%	68,8%
6198	30	70	60	65	49,5%	54,2%	73,5%	82,4%	70,3%
6199	30	70	60	70	49,9%	53,9%	74,1%	83,4%	71,7%
6200	30	70	60	75	50,4%	53,5%	74,8%	84,3%	73,1%
6201	30	70	60	80	50,8%	53,2%	75,4%	85,2%	74,4%
6202	30	70	65	20	39,5%	51,4%	64,3%	70,3%	55,1%
6203	30	70	65	25	39,9%	51,0%	65,1%	71,7%	56,8%

6204	30	70	65	30	40,4%	50,7%	65,9%	73,1%	58,4%
6205	30	70	65	35	40,8%	50,4%	66,6%	74,4%	60,1%
6206	30	70	65	40	41,2%	50,1%	67,4%	75,7%	61,7%
6207	30	70	65	45	41,6%	49,7%	68,1%	77,0%	63,3%
6208	30	70	65	50	42,1%	49,4%	68,9%	78,2%	64,9%
6209	30	70	65	55	42,5%	49,1%	69,6%	79,3%	66,4%
6210	30	70	65	60	42,9%	48,7%	70,3%	80,4%	68,0%
6211	30	70	65	65	43,4%	48,4%	71,0%	81,5%	69,4%
6212	30	70	65	70	43,8%	48,1%	71,7%	82,5%	70,9%
6213	30	70	65	75	44,2%	47,8%	72,4%	83,5%	72,3%
6214	30	70	65	80	44,7%	47,4%	73,0%	84,4%	73,6%
6215	30	70	70	20	33,8%	45,6%	61,4%	69,0%	54,0%
6216	30	70	70	25	34,2%	45,3%	62,2%	70,5%	55,7%
6217	30	70	70	30	34,6%	45,0%	63,0%	71,9%	57,4%
6218	30	70	70	35	35,0%	44,6%	63,8%	73,2%	59,1%
6219	30	70	70	40	35,4%	44,3%	64,6%	74,6%	60,7%
6220	30	70	70	45	35,8%	44,0%	65,4%	75,9%	62,4%
6221	30	70	70	50	36,2%	43,7%	66,1%	77,1%	64,0%
6222	30	70	70	55	36,6%	43,3%	66,9%	78,3%	65,5%
6223	30	70	70	60	37,0%	43,0%	67,6%	79,4%	67,0%
6224	30	70	70	65	37,4%	42,7%	68,4%	80,6%	68,5%
6225	30	70	70	70	37,8%	42,4%	69,1%	81,6%	70,0%
6226	30	70	70	75	38,3%	42,1%	69,8%	82,6%	71,4%
6227	30	70	70	80	38,7%	41,7%	70,5%	83,6%	72,8%
6228	30	70	75	20	28,5%	40,0%	58,4%	67,7%	53,0%
6229	30	70	75	25	28,9%	39,6%	59,3%	69,2%	54,7%
6230	30	70	75	30	29,2%	39,3%	60,1%	70,6%	56,4%
6231	30	70	75	35	29,6%	39,0%	60,9%	72,0%	58,1%
6232	30	70	75	40	30,0%	38,7%	61,7%	73,4%	59,7%
6233	30	70	75	45	30,3%	38,4%	62,5%	74,7%	61,4%
6234	30	70	75	50	30,7%	38,1%	63,3%	76,0%	63,0%
6235	30	70	75	55	31,1%	37,8%	64,1%	77,2%	64,6%
6236	30	70	75	60	31,5%	37,5%	64,9%	78,4%	66,1%

6237	30	70	75	65	31,8%	37,2%	65,6%	79,6%	67,6%
6238	30	70	75	70	32,2%	36,9%	66,4%	80,7%	69,1%
6239	30	70	75	75	32,6%	36,6%	67,1%	81,7%	70,6%
6240	30	70	75	80	33,0%	36,3%	67,9%	82,7%	72,0%
6241	30	70	80	20	23,8%	34,6%	55,4%	66,3%	52,0%
6242	30	70	80	25	24,1%	34,3%	56,2%	67,9%	53,7%
6243	30	70	80	30	24,4%	34,0%	57,1%	69,3%	55,4%
6244	30	70	80	35	24,7%	33,7%	57,9%	70,8%	57,1%
6245	30	70	80	40	25,1%	33,4%	58,7%	72,2%	58,8%
6246	30	70	80	45	25,4%	33,1%	59,5%	73,6%	60,4%
6247	30	70	80	50	25,7%	32,8%	60,4%	74,9%	62,0%
6248	30	70	80	55	26,1%	32,5%	61,2%	76,2%	63,6%
6249	30	70	80	60	26,4%	32,2%	62,0%	77,4%	65,2%
6250	30	70	80	65	26,7%	31,9%	62,8%	78,6%	66,7%
6251	30	70	80	70	27,1%	31,7%	63,6%	79,7%	68,2%
6252	30	70	80	75	27,4%	31,4%	64,3%	80,8%	69,7%
6253	30	70	80	80	27,8%	31,1%	65,1%	81,8%	71,1%
6254	30	75	20	20	86,8%	90,1%	84,2%	79,2%	61,8%
6255	30	75	20	25	87,0%	90,0%	84,7%	80,3%	63,4%
6256	30	75	20	30	87,2%	89,9%	85,1%	81,4%	64,9%
6257	30	75	20	35	87,4%	89,8%	85,5%	82,4%	66,5%
6258	30	75	20	40	87,6%	89,7%	86,0%	83,4%	68,0%
6259	30	75	20	45	87,8%	89,5%	86,4%	84,3%	69,5%
6260	30	75	20	50	88,0%	89,4%	86,8%	85,2%	70,9%
6261	30	75	20	55	88,2%	89,3%	87,1%	86,0%	72,3%
6262	30	75	20	60	88,4%	89,2%	87,5%	86,8%	73,6%
6263	30	75	20	65	88,5%	89,0%	87,9%	87,6%	74,9%
6264	30	75	20	70	88,7%	88,9%	88,2%	88,3%	76,2%
6265	30	75	20	75	88,9%	88,8%	88,6%	89,0%	77,4%
6266	30	75	20	80	89,1%	88,6%	88,9%	89,7%	78,6%
6267	30	75	25	20	83,7%	87,9%	82,5%	78,2%	60,8%
6268	30	75	25	25	84,0%	87,7%	83,0%	79,3%	62,4%
6269	30	75	25	30	84,2%	87,6%	83,5%	80,4%	64,0%

6270	30	75	25	35	84,4%	87,5%	83,9%	81,5%	65,6%
6271	30	75	25	40	84,7%	87,3%	84,4%	82,5%	67,1%
6272	30	75	25	45	84,9%	87,2%	84,8%	83,5%	68,6%
6273	30	75	25	50	85,1%	87,0%	85,3%	84,4%	70,0%
6274	30	75	25	55	85,3%	86,9%	85,7%	85,3%	71,5%
6275	30	75	25	60	85,6%	86,7%	86,1%	86,1%	72,8%
6276	30	75	25	65	85,8%	86,6%	86,5%	86,9%	74,2%
6277	30	75	25	70	86,0%	86,4%	86,9%	87,7%	75,5%
6278	30	75	25	75	86,2%	86,3%	87,3%	88,4%	76,7%
6279	30	75	25	80	86,4%	86,1%	87,6%	89,1%	77,9%
6280	30	75	30	20	80,1%	85,2%	80,6%	77,1%	59,8%
6281	30	75	30	25	80,4%	85,0%	81,2%	78,3%	61,4%
6282	30	75	30	30	80,6%	84,9%	81,7%	79,4%	63,0%
6283	30	75	30	35	80,9%	84,7%	82,2%	80,5%	64,6%
6284	30	75	30	40	81,2%	84,5%	82,7%	81,6%	66,2%
6285	30	75	30	45	81,5%	84,3%	83,2%	82,6%	67,7%
6286	30	75	30	50	81,7%	84,2%	83,6%	83,6%	69,2%
6287	30	75	30	55	82,0%	84,0%	84,1%	84,5%	70,6%
6288	30	75	30	60	82,2%	83,8%	84,5%	85,4%	72,0%
6289	30	75	30	65	82,5%	83,6%	85,0%	86,2%	73,4%
6290	30	75	30	70	82,7%	83,5%	85,4%	87,0%	74,7%
6291	30	75	30	75	83,0%	83,3%	85,8%	87,8%	76,0%
6292	30	75	30	80	83,2%	83,1%	86,2%	88,5%	77,2%
6293	30	75	35	20	75,9%	82,0%	78,6%	76,0%	58,8%
6294	30	75	35	25	76,2%	81,8%	79,2%	77,2%	60,4%
6295	30	75	35	30	76,5%	81,6%	79,8%	78,4%	62,1%
6296	30	75	35	35	76,8%	81,5%	80,3%	79,6%	63,7%
6297	30	75	35	40	77,1%	81,3%	80,8%	80,7%	65,2%
6298	30	75	35	45	77,4%	81,1%	81,4%	81,7%	66,8%
6299	30	75	35	50	77,7%	80,9%	81,9%	82,7%	68,3%
6300	30	75	35	55	78,0%	80,6%	82,4%	83,7%	69,7%
6301	30	75	35	60	78,3%	80,4%	82,8%	84,6%	71,2%
6302	30	75	35	65	78,6%	80,2%	83,3%	85,5%	72,6%

6303	30	75	35	70	78,9%	80,0%	83,8%	86,3%	73,9%
6304	30	75	35	75	79,2%	79,8%	84,2%	87,1%	75,2%
6305	30	75	35	80	79,5%	79,6%	84,7%	87,9%	76,4%
6306	30	75	40	20	71,0%	78,4%	76,5%	74,9%	57,8%
6307	30	75	40	25	71,4%	78,2%	77,1%	76,2%	59,5%
6308	30	75	40	30	71,8%	77,9%	77,7%	77,4%	61,1%
6309	30	75	40	35	72,1%	77,7%	78,3%	78,6%	62,7%
6310	30	75	40	40	72,5%	77,5%	78,8%	79,7%	64,3%
6311	30	75	40	45	72,8%	77,2%	79,4%	80,8%	65,9%
6312	30	75	40	50	73,2%	77,0%	79,9%	81,8%	67,4%
6313	30	75	40	55	73,5%	76,8%	80,5%	82,8%	68,9%
6314	30	75	40	60	73,9%	76,6%	81,0%	83,8%	70,3%
6315	30	75	40	65	74,2%	76,3%	81,5%	84,7%	71,7%
6316	30	75	40	70	74,5%	76,1%	82,0%	85,6%	73,1%
6317	30	75	40	75	74,9%	75,8%	82,5%	86,4%	74,4%
6318	30	75	40	80	75,2%	75,6%	83,0%	87,2%	75,7%
6319	30	75	45	20	65,7%	74,2%	74,2%	73,7%	56,8%
6320	30	75	45	25	66,1%	74,0%	74,8%	75,0%	58,5%
6321	30	75	45	30	66,5%	73,7%	75,4%	76,3%	60,1%
6322	30	75	45	35	66,9%	73,5%	76,1%	77,5%	61,7%
6323	30	75	45	40	67,3%	73,2%	76,7%	78,7%	63,3%
6324	30	75	45	45	67,7%	72,9%	77,3%	79,8%	64,9%
6325	30	75	45	50	68,1%	72,7%	77,9%	80,9%	66,5%
6326	30	75	45	55	68,4%	72,4%	78,5%	82,0%	68,0%
6327	30	75	45	60	68,8%	72,2%	79,0%	83,0%	69,4%
6328	30	75	45	65	69,2%	71,9%	79,6%	83,9%	70,9%
6329	30	75	45	70	69,6%	71,6%	80,1%	84,8%	72,3%
6330	30	75	45	75	69,9%	71,4%	80,7%	85,7%	73,6%
6331	30	75	45	80	70,3%	71,1%	81,2%	86,5%	74,9%
6332	30	75	50	20	60,0%	69,6%	71,7%	72,5%	55,8%
6333	30	75	50	25	60,4%	69,3%	72,4%	73,9%	57,5%
6334	30	75	50	30	60,8%	69,0%	73,1%	75,2%	59,1%
6335	30	75	50	35	61,2%	68,7%	73,7%	76,4%	60,8%

6336	30	75	50	40	61,6%	68,4%	74,4%	77,7%	62,4%
6337	30	75	50	45	62,1%	68,1%	75,0%	78,8%	64,0%
6338	30	75	50	50	62,5%	67,9%	75,7%	80,0%	65,5%
6339	30	75	50	55	62,9%	67,6%	76,3%	81,0%	67,1%
6340	30	75	50	60	63,3%	67,3%	76,9%	82,1%	68,6%
6341	30	75	50	65	63,7%	67,0%	77,5%	83,1%	70,0%
6342	30	75	50	70	64,1%	66,7%	78,1%	84,0%	71,4%
6343	30	75	50	75	64,5%	66,4%	78,7%	84,9%	72,8%
6344	30	75	50	80	64,9%	66,1%	79,2%	85,8%	74,1%
6345	30	75	55	20	53,9%	64,5%	69,1%	71,3%	54,7%
6346	30	75	55	25	54,4%	64,2%	69,9%	72,7%	56,4%
6347	30	75	55	30	54,8%	63,9%	70,6%	74,0%	58,1%
6348	30	75	55	35	55,2%	63,5%	71,3%	75,3%	59,8%
6349	30	75	55	40	55,7%	63,2%	72,0%	76,6%	61,4%
6350	30	75	55	45	56,1%	62,9%	72,6%	77,8%	63,0%
6351	30	75	55	50	56,5%	62,6%	73,3%	79,0%	64,6%
6352	30	75	55	55	57,0%	62,3%	74,0%	80,1%	66,2%
6353	30	75	55	60	57,4%	62,0%	74,6%	81,2%	67,7%
6354	30	75	55	65	57,8%	61,7%	75,2%	82,2%	69,1%
6355	30	75	55	70	58,3%	61,4%	75,9%	83,2%	70,6%
6356	30	75	55	75	58,7%	61,1%	76,5%	84,1%	72,0%
6357	30	75	55	80	59,1%	60,8%	77,1%	85,0%	73,4%
6358	30	75	60	20	47,8%	59,0%	66,4%	70,0%	53,7%
6359	30	75	60	25	48,2%	58,7%	67,2%	71,4%	55,4%
6360	30	75	60	30	48,6%	58,4%	67,9%	72,8%	57,1%
6361	30	75	60	35	49,1%	58,1%	68,7%	74,2%	58,8%
6362	30	75	60	40	49,5%	57,7%	69,4%	75,5%	60,4%
6363	30	75	60	45	50,0%	57,4%	70,1%	76,7%	62,1%
6364	30	75	60	50	50,4%	57,1%	70,8%	77,9%	63,7%
6365	30	75	60	55	50,8%	56,8%	71,5%	79,1%	65,2%
6366	30	75	60	60	51,3%	56,5%	72,2%	80,2%	66,8%
6367	30	75	60	65	51,7%	56,1%	72,9%	81,3%	68,3%
6368	30	75	60	70	52,2%	55,8%	73,5%	82,3%	69,7%

6369	30	75	60	75	52,6%	55,5%	74,2%	83,3%	71,2%
6370	30	75	60	80	53,0%	55,2%	74,8%	84,2%	72,5%
6371	30	75	65	20	41,7%	53,3%	63,6%	68,7%	52,7%
6372	30	75	65	25	42,1%	53,0%	64,4%	70,2%	54,4%
6373	30	75	65	30	42,5%	52,7%	65,2%	71,6%	56,1%
6374	30	75	65	35	42,9%	52,3%	65,9%	73,0%	57,8%
6375	30	75	65	40	43,4%	52,0%	66,7%	74,3%	59,4%
6376	30	75	65	45	43,8%	51,7%	67,4%	75,6%	61,1%
6377	30	75	65	50	44,2%	51,4%	68,2%	76,9%	62,7%
6378	30	75	65	55	44,7%	51,0%	68,9%	78,1%	64,3%
6379	30	75	65	60	45,1%	50,7%	69,6%	79,2%	65,8%
6380	30	75	65	65	45,6%	50,4%	70,3%	80,3%	67,4%
6381	30	75	65	70	46,0%	50,1%	71,0%	81,4%	68,8%
6382	30	75	65	75	46,4%	49,7%	71,7%	82,4%	70,3%
6383	30	75	65	80	46,9%	49,4%	72,4%	83,4%	71,7%
6384	30	75	70	20	35,8%	47,6%	60,7%	67,4%	51,7%
6385	30	75	70	25	36,2%	47,2%	61,5%	68,9%	53,4%
6386	30	75	70	30	36,6%	46,9%	62,3%	70,4%	55,1%
6387	30	75	70	35	37,0%	46,6%	63,1%	71,8%	56,8%
6388	30	75	70	40	37,4%	46,3%	63,9%	73,1%	58,4%
6389	30	75	70	45	37,9%	45,9%	64,6%	74,5%	60,1%
6390	30	75	70	50	38,3%	45,6%	65,4%	75,8%	61,7%
6391	30	75	70	55	38,7%	45,3%	66,2%	77,0%	63,3%
6392	30	75	70	60	39,1%	45,0%	66,9%	78,2%	64,9%
6393	30	75	70	65	39,5%	44,6%	67,7%	79,4%	66,4%
6394	30	75	70	70	39,9%	44,3%	68,4%	80,5%	68,0%
6395	30	75	70	75	40,4%	44,0%	69,1%	81,5%	69,4%
6396	30	75	70	80	40,8%	43,7%	69,9%	82,5%	70,9%
6397	30	75	75	20	30,3%	41,9%	57,7%	66,0%	50,6%
6398	30	75	75	25	30,7%	41,5%	58,5%	67,6%	52,3%
6399	30	75	75	30	31,1%	41,2%	59,3%	69,1%	54,1%
6400	30	75	75	35	31,5%	40,9%	60,1%	70,5%	55,8%
6401	30	75	75	40	31,9%	40,6%	60,9%	71,9%	57,4%

6402	30	75	75	45	32,2%	40,3%	61,8%	73,3%	59,1%
6403	30	75	75	50	32,6%	40,0%	62,6%	74,6%	60,7%
6404	30	75	75	55	33,0%	39,6%	63,3%	75,9%	62,4%
6405	30	75	75	60	33,4%	39,3%	64,1%	77,2%	64,0%
6406	30	75	75	65	33,8%	39,0%	64,9%	78,3%	65,5%
6407	30	75	75	70	34,2%	38,7%	65,7%	79,5%	67,1%
6408	30	75	75	75	34,6%	38,4%	66,4%	80,6%	68,5%
6409	30	75	75	80	35,0%	38,1%	67,2%	81,7%	70,0%
6410	30	75	80	20	25,4%	36,4%	54,6%	64,7%	49,6%
6411	30	75	80	25	25,7%	36,1%	55,5%	66,2%	51,3%
6412	30	75	80	30	26,1%	35,8%	56,3%	67,7%	53,0%
6413	30	75	80	35	26,4%	35,5%	57,1%	69,2%	54,7%
6414	30	75	80	40	26,8%	35,2%	58,0%	70,7%	56,4%
6415	30	75	80	45	27,1%	34,9%	58,8%	72,1%	58,1%
6416	30	75	80	50	27,5%	34,6%	59,6%	73,5%	59,8%
6417	30	75	80	55	27,8%	34,3%	60,4%	74,8%	61,4%
6418	30	75	80	60	28,2%	34,0%	61,2%	76,1%	63,0%
6419	30	75	80	65	28,5%	33,7%	62,0%	77,3%	64,6%
6420	30	75	80	70	28,9%	33,4%	62,8%	78,5%	66,1%
6421	30	75	80	75	29,2%	33,1%	63,6%	79,6%	67,7%
6422	30	75	80	80	29,6%	32,8%	64,4%	80,7%	69,1%
6423	30	80	20	20	87,8%	90,8%	83,8%	77,9%	59,5%
6424	30	80	20	25	88,0%	90,7%	84,3%	79,1%	61,1%
6425	30	80	20	30	88,2%	90,6%	84,7%	80,2%	62,7%
6426	30	80	20	35	88,4%	90,5%	85,1%	81,3%	64,3%
6427	30	80	20	40	88,5%	90,4%	85,6%	82,3%	65,9%
6428	30	80	20	45	88,7%	90,2%	86,0%	83,3%	67,4%
6429	30	80	20	50	88,9%	90,1%	86,4%	84,2%	68,9%
6430	30	80	20	55	89,1%	90,0%	86,8%	85,1%	70,3%
6431	30	80	20	60	89,2%	89,9%	87,2%	86,0%	71,7%
6432	30	80	20	65	89,4%	89,8%	87,5%	86,8%	73,1%
6433	30	80	20	70	89,6%	89,7%	87,9%	87,6%	74,4%
6434	30	80	20	75	89,7%	89,5%	88,3%	88,3%	75,7%

6435	30	80	20	80	89,9%	89,4%	88,6%	89,0%	77,0%
6436	30	80	25	20	84,9%	88,7%	82,1%	76,9%	58,5%
6437	30	80	25	25	85,1%	88,6%	82,5%	78,1%	60,1%
6438	30	80	25	30	85,3%	88,4%	83,0%	79,2%	61,8%
6439	30	80	25	35	85,6%	88,3%	83,5%	80,3%	63,4%
6440	30	80	25	40	85,8%	88,2%	84,0%	81,4%	64,9%
6441	30	80	25	45	86,0%	88,0%	84,4%	82,4%	66,5%
6442	30	80	25	50	86,2%	87,9%	84,9%	83,4%	68,0%
6443	30	80	25	55	86,4%	87,7%	85,3%	84,3%	69,5%
6444	30	80	25	60	86,6%	87,6%	85,7%	85,2%	70,9%
6445	30	80	25	65	86,8%	87,5%	86,1%	86,1%	72,3%
6446	30	80	25	70	87,0%	87,3%	86,5%	86,9%	73,6%
6447	30	80	25	75	87,2%	87,2%	86,9%	87,6%	75,0%
6448	30	80	25	80	87,4%	87,0%	87,3%	88,4%	76,2%
6449	30	80	30	20	81,5%	86,2%	80,1%	75,8%	57,5%
6450	30	80	30	25	81,7%	86,0%	80,7%	77,0%	59,1%
6451	30	80	30	30	82,0%	85,8%	81,2%	78,2%	60,8%
6452	30	80	30	35	82,2%	85,7%	81,7%	79,4%	62,4%
6453	30	80	30	40	82,5%	85,5%	82,2%	80,5%	64,0%
6454	30	80	30	45	82,8%	85,4%	82,7%	81,5%	65,6%
6455	30	80	30	50	83,0%	85,2%	83,2%	82,5%	67,1%
6456	30	80	30	55	83,3%	85,0%	83,7%	83,5%	68,6%
6457	30	80	30	60	83,5%	84,9%	84,1%	84,4%	70,0%
6458	30	80	30	65	83,7%	84,7%	84,6%	85,3%	71,5%
6459	30	80	30	70	84,0%	84,5%	85,0%	86,2%	72,8%
6460	30	80	30	75	84,2%	84,4%	85,4%	87,0%	74,2%
6461	30	80	30	80	84,4%	84,2%	85,9%	87,7%	75,5%
6462	30	80	35	20	77,4%	83,2%	78,1%	74,6%	56,5%
6463	30	80	35	25	77,7%	83,0%	78,7%	75,9%	58,1%
6464	30	80	35	30	78,0%	82,8%	79,2%	77,2%	59,8%
6465	30	80	35	35	78,3%	82,6%	79,8%	78,3%	61,4%
6466	30	80	35	40	78,6%	82,4%	80,3%	79,5%	63,0%
6467	30	80	35	45	78,9%	82,2%	80,9%	80,6%	64,6%

6468	30	80	35	50	79,2%	82,0%	81,4%	81,6%	66,2%
6469	30	80	35	55	79,5%	81,8%	81,9%	82,7%	67,7%
6470	30	80	35	60	79,8%	81,7%	82,4%	83,6%	69,2%
6471	30	80	35	65	80,1%	81,5%	82,9%	84,5%	70,6%
6472	30	80	35	70	80,4%	81,3%	83,4%	85,4%	72,0%
6473	30	80	35	75	80,6%	81,1%	83,8%	86,3%	73,4%
6474	30	80	35	80	80,9%	80,9%	84,3%	87,1%	74,7%
6475	30	80	40	20	72,8%	79,7%	75,9%	73,5%	55,4%
6476	30	80	40	25	73,2%	79,5%	76,5%	74,8%	57,1%
6477	30	80	40	30	73,5%	79,3%	77,1%	76,1%	58,8%
6478	30	80	40	35	73,9%	79,0%	77,7%	77,3%	60,4%
6479	30	80	40	40	74,2%	78,8%	78,3%	78,5%	62,1%
6480	30	80	40	45	74,5%	78,6%	78,9%	79,6%	63,7%
6481	30	80	40	50	74,9%	78,4%	79,4%	80,7%	65,2%
6482	30	80	40	55	75,2%	78,2%	80,0%	81,8%	66,8%
6483	30	80	40	60	75,5%	77,9%	80,5%	82,8%	68,3%
6484	30	80	40	65	75,9%	77,7%	81,0%	83,7%	69,7%
6485	30	80	40	70	76,2%	77,5%	81,6%	84,7%	71,2%
6486	30	80	40	75	76,5%	77,3%	82,1%	85,5%	72,6%
6487	30	80	40	80	76,8%	77,0%	82,6%	86,4%	73,9%
6488	30	80	45	20	67,7%	75,7%	73,6%	72,3%	54,4%
6489	30	80	45	25	68,1%	75,4%	74,2%	73,6%	56,1%
6490	30	80	45	30	68,5%	75,2%	74,9%	74,9%	57,8%
6491	30	80	45	35	68,8%	75,0%	75,5%	76,2%	59,5%
6492	30	80	45	40	69,2%	74,7%	76,1%	77,4%	61,1%
6493	30	80	45	45	69,6%	74,5%	76,7%	78,6%	62,7%
6494	30	80	45	50	70,0%	74,2%	77,3%	79,8%	64,3%
6495	30	80	45	55	70,3%	74,0%	77,9%	80,8%	65,9%
6496	30	80	45	60	70,7%	73,7%	78,5%	81,9%	67,4%
6497	30	80	45	65	71,1%	73,5%	79,1%	82,9%	68,9%
6498	30	80	45	70	71,4%	73,2%	79,6%	83,8%	70,3%
6499	30	80	45	75	71,8%	72,9%	80,2%	84,8%	71,7%
6500	30	80	45	80	72,1%	72,7%	80,7%	85,6%	73,1%

6501	30	80	50	20	62,1%	71,2%	71,1%	71,0%	53,4%
6502	30	80	50	25	62,5%	70,9%	71,8%	72,4%	55,1%
6503	30	80	50	30	62,9%	70,7%	72,4%	73,8%	56,8%
6504	30	80	50	35	63,3%	70,4%	73,1%	75,1%	58,5%
6505	30	80	50	40	63,7%	70,1%	73,8%	76,3%	60,1%
6506	30	80	50	45	64,1%	69,8%	74,4%	77,6%	61,7%
6507	30	80	50	50	64,5%	69,6%	75,1%	78,7%	63,3%
6508	30	80	50	55	64,9%	69,3%	75,7%	79,9%	64,9%
6509	30	80	50	60	65,3%	69,0%	76,3%	81,0%	66,5%
6510	30	80	50	65	65,7%	68,7%	76,9%	82,0%	68,0%
6511	30	80	50	70	66,1%	68,4%	77,5%	83,0%	69,5%
6512	30	80	50	75	66,5%	68,2%	78,1%	84,0%	70,9%
6513	30	80	50	80	66,9%	67,9%	78,7%	84,9%	72,3%
6514	30	80	55	20	56,1%	66,2%	68,4%	69,7%	52,4%
6515	30	80	55	25	56,5%	65,9%	69,2%	71,2%	54,1%
6516	30	80	55	30	57,0%	65,6%	69,9%	72,6%	55,8%
6517	30	80	55	35	57,4%	65,4%	70,6%	73,9%	57,5%
6518	30	80	55	40	57,8%	65,1%	71,3%	75,2%	59,1%
6519	30	80	55	45	58,3%	64,8%	72,0%	76,5%	60,8%
6520	30	80	55	50	58,7%	64,5%	72,7%	77,7%	62,4%
6521	30	80	55	55	59,1%	64,2%	73,3%	78,9%	64,0%
6522	30	80	55	60	59,5%	63,9%	74,0%	80,0%	65,5%
6523	30	80	55	65	60,0%	63,6%	74,7%	81,1%	67,1%
6524	30	80	55	70	60,4%	63,3%	75,3%	82,1%	68,6%
6525	30	80	55	75	60,8%	62,9%	75,9%	83,1%	70,0%
6526	30	80	55	80	61,2%	62,6%	76,5%	84,1%	71,4%
6527	30	80	60	20	50,0%	60,9%	65,7%	68,4%	51,3%
6528	30	80	60	25	50,4%	60,6%	66,5%	69,9%	53,1%
6529	30	80	60	30	50,8%	60,3%	67,2%	71,3%	54,8%
6530	30	80	60	35	51,3%	60,0%	68,0%	72,7%	56,4%
6531	30	80	60	40	51,7%	59,6%	68,7%	74,1%	58,1%
6532	30	80	60	45	52,2%	59,3%	69,4%	75,4%	59,8%
6533	30	80	60	50	52,6%	59,0%	70,1%	76,6%	61,4%

6534	30	80	60	55	53,0%	58,7%	70,8%	77,8%	63,0%
6535	30	80	60	60	53,5%	58,4%	71,5%	79,0%	64,6%
6536	30	80	60	65	53,9%	58,1%	72,2%	80,1%	66,2%
6537	30	80	60	70	54,4%	57,7%	72,9%	81,2%	67,7%
6538	30	80	60	75	54,8%	57,4%	73,6%	82,2%	69,2%
6539	30	80	60	80	55,2%	57,1%	74,2%	83,2%	70,6%
6540	30	80	65	20	43,8%	55,3%	62,9%	67,1%	50,3%
6541	30	80	65	25	44,3%	55,0%	63,6%	68,6%	52,0%
6542	30	80	65	30	44,7%	54,6%	64,4%	70,1%	53,7%
6543	30	80	65	35	45,1%	54,3%	65,2%	71,5%	55,4%
6544	30	80	65	40	45,6%	54,0%	66,0%	72,9%	57,1%
6545	30	80	65	45	46,0%	53,7%	66,7%	74,2%	58,8%
6546	30	80	65	50	46,4%	53,3%	67,5%	75,5%	60,4%
6547	30	80	65	55	46,9%	53,0%	68,2%	76,8%	62,1%
6548	30	80	65	60	47,3%	52,7%	68,9%	78,0%	63,7%
6549	30	80	65	65	47,8%	52,4%	69,7%	79,1%	65,2%
6550	30	80	65	70	48,2%	52,0%	70,4%	80,3%	66,8%
6551	30	80	65	75	48,6%	51,7%	71,1%	81,3%	68,3%
6552	30	80	65	80	49,1%	51,4%	71,8%	82,4%	69,7%
6553	30	80	70	20	37,9%	49,5%	59,9%	65,7%	49,3%
6554	30	80	70	25	38,3%	49,2%	60,7%	67,3%	51,0%
6555	30	80	70	30	38,7%	48,9%	61,5%	68,8%	52,7%
6556	30	80	70	35	39,1%	48,5%	62,3%	70,2%	54,4%
6557	30	80	70	40	39,5%	48,2%	63,1%	71,7%	56,1%
6558	30	80	70	45	40,0%	47,9%	63,9%	73,0%	57,8%
6559	30	80	70	50	40,4%	47,6%	64,7%	74,4%	59,4%
6560	30	80	70	55	40,8%	47,2%	65,5%	75,7%	61,1%
6561	30	80	70	60	41,2%	46,9%	66,2%	76,9%	62,7%
6562	30	80	70	65	41,7%	46,6%	67,0%	78,1%	64,3%
6563	30	80	70	70	42,1%	46,3%	67,7%	79,3%	65,8%
6564	30	80	70	75	42,5%	45,9%	68,5%	80,4%	67,4%
6565	30	80	70	80	43,0%	45,6%	69,2%	81,5%	68,9%
6566	30	80	75	20	32,3%	43,8%	56,9%	64,4%	48,3%

6567	30	80	75	25	32,6%	43,5%	57,7%	65,9%	50,0%
6568	30	80	75	30	33,0%	43,1%	58,6%	67,5%	51,7%
6569	30	80	75	35	33,4%	42,8%	59,4%	69,0%	53,4%
6570	30	80	75	40	33,8%	42,5%	60,2%	70,4%	55,1%
6571	30	80	75	45	34,2%	42,2%	61,0%	71,8%	56,8%
6572	30	80	75	50	34,6%	41,9%	61,8%	73,2%	58,4%
6573	30	80	75	55	35,0%	41,5%	62,6%	74,5%	60,1%
6574	30	80	75	60	35,4%	41,2%	63,4%	75,8%	61,7%
6575	30	80	75	65	35,8%	40,9%	64,2%	77,1%	63,3%
6576	30	80	75	70	36,2%	40,6%	65,0%	78,3%	64,9%
6577	30	80	75	75	36,6%	40,3%	65,7%	79,4%	66,5%
6578	30	80	75	80	37,0%	40,0%	66,5%	80,5%	68,0%
6579	30	80	80	20	27,1%	38,2%	53,8%	63,0%	47,2%
6580	30	80	80	25	27,5%	37,9%	54,7%	64,5%	48,9%
6581	30	80	80	30	27,8%	37,6%	55,5%	66,1%	50,6%
6582	30	80	80	35	28,2%	37,3%	56,3%	67,6%	52,4%
6583	30	80	80	40	28,5%	37,0%	57,2%	69,1%	54,1%
6584	30	80	80	45	28,9%	36,7%	58,0%	70,6%	55,8%
6585	30	80	80	50	29,3%	36,4%	58,8%	72,0%	57,4%
6586	30	80	80	55	29,6%	36,1%	59,7%	73,4%	59,1%
6587	30	80	80	60	30,0%	35,8%	60,5%	74,7%	60,7%
6588	30	80	80	65	30,4%	35,5%	61,3%	76,0%	62,4%
6589	30	80	80	70	30,7%	35,2%	62,1%	77,2%	64,0%
6590	30	80	80	75	31,1%	34,9%	62,9%	78,4%	65,5%
6591	30	80	80	80	31,5%	34,6%	63,7%	79,5%	67,1%
6592	35	20	20	20	76,0%	84,3%	92,0%	92,8%	86,2%
6593	35	20	20	25	76,4%	84,1%	92,3%	93,2%	87,0%
6594	35	20	20	30	76,7%	83,9%	92,5%	93,7%	87,7%
6595	35	20	20	35	77,0%	83,8%	92,7%	94,1%	88,4%
6596	35	20	20	40	77,3%	83,6%	93,0%	94,4%	89,1%
6597	35	20	20	45	77,6%	83,4%	93,2%	94,8%	89,8%
6598	35	20	20	50	77,9%	83,2%	93,4%	95,1%	90,4%
6599	35	20	20	55	78,2%	83,0%	93,6%	95,4%	91,0%

6600	35	20	20	60	78,5%	82,8%	93,8%	95,7%	91,5%
6601	35	20	20	65	78,8%	82,7%	94,0%	96,0%	92,0%
6602	35	20	20	70	79,1%	82,5%	94,2%	96,2%	92,5%
6603	35	20	20	75	79,4%	82,3%	94,4%	96,5%	93,0%
6604	35	20	20	80	79,7%	82,1%	94,5%	96,7%	93,4%
6605	35	20	25	20	71,3%	81,0%	91,1%	92,4%	85,7%
6606	35	20	25	25	71,6%	80,8%	91,3%	92,8%	86,5%
6607	35	20	25	30	72,0%	80,6%	91,6%	93,3%	87,3%
6608	35	20	25	35	72,3%	80,4%	91,8%	93,7%	88,0%
6609	35	20	25	40	72,7%	80,2%	92,1%	94,1%	88,7%
6610	35	20	25	45	73,0%	80,0%	92,3%	94,5%	89,4%
6611	35	20	25	50	73,4%	79,7%	92,6%	94,8%	90,0%
6612	35	20	25	55	73,7%	79,5%	92,8%	95,1%	90,6%
6613	35	20	25	60	74,1%	79,3%	93,0%	95,5%	91,2%
6614	35	20	25	65	74,4%	79,1%	93,2%	95,7%	91,7%
6615	35	20	25	70	74,7%	78,9%	93,5%	96,0%	92,2%
6616	35	20	25	75	75,1%	78,7%	93,7%	96,3%	92,7%
6617	35	20	25	80	75,4%	78,4%	93,9%	96,5%	93,2%
6618	35	20	30	20	65,9%	77,2%	90,0%	91,9%	85,2%
6619	35	20	30	25	66,3%	76,9%	90,3%	92,4%	86,0%
6620	35	20	30	30	66,7%	76,7%	90,6%	92,9%	86,8%
6621	35	20	30	35	67,1%	76,5%	90,9%	93,3%	87,6%
6622	35	20	30	40	67,5%	76,2%	91,1%	93,7%	88,3%
6623	35	20	30	45	67,9%	76,0%	91,4%	94,1%	89,0%
6624	35	20	30	50	68,3%	75,8%	91,7%	94,5%	89,6%
6625	35	20	30	55	68,7%	75,5%	91,9%	94,9%	90,3%
6626	35	20	30	60	69,0%	75,3%	92,2%	95,2%	90,9%
6627	35	20	30	65	69,4%	75,0%	92,4%	95,5%	91,4%
6628	35	20	30	70	69,8%	74,8%	92,7%	95,8%	91,9%
6629	35	20	30	75	70,2%	74,5%	92,9%	96,0%	92,4%
6630	35	20	30	80	70,5%	74,3%	93,1%	96,3%	92,9%
6631	35	20	35	20	60,2%	72,8%	88,8%	91,5%	84,6%
6632	35	20	35	25	60,6%	72,6%	89,1%	92,0%	85,5%

6633	35	20	35	30	61,0%	72,3%	89,5%	92,5%	86,3%
6634	35	20	35	35	61,5%	72,1%	89,8%	92,9%	87,1%
6635	35	20	35	40	61,9%	71,8%	90,1%	93,4%	87,9%
6636	35	20	35	45	62,3%	71,5%	90,4%	93,8%	88,6%
6637	35	20	35	50	62,7%	71,3%	90,7%	94,2%	89,3%
6638	35	20	35	55	63,1%	71,0%	91,0%	94,5%	89,9%
6639	35	20	35	60	63,5%	70,7%	91,2%	94,9%	90,5%
6640	35	20	35	65	63,9%	70,5%	91,5%	95,2%	91,1%
6641	35	20	35	70	64,3%	70,2%	91,8%	95,5%	91,6%
6642	35	20	35	75	64,7%	69,9%	92,0%	95,8%	92,1%
6643	35	20	35	80	65,1%	69,6%	92,3%	96,1%	92,6%
6644	35	20	40	20	54,2%	68,0%	87,5%	91,0%	84,1%
6645	35	20	40	25	54,6%	67,8%	87,9%	91,5%	85,0%
6646	35	20	40	30	55,0%	67,5%	88,2%	92,0%	85,8%
6647	35	20	40	35	55,5%	67,2%	88,6%	92,5%	86,7%
6648	35	20	40	40	55,9%	66,9%	88,9%	93,0%	87,4%
6649	35	20	40	45	56,3%	66,6%	89,3%	93,4%	88,2%
6650	35	20	40	50	56,8%	66,3%	89,6%	93,8%	88,9%
6651	35	20	40	55	57,2%	66,0%	89,9%	94,2%	89,5%
6652	35	20	40	60	57,6%	65,7%	90,2%	94,6%	90,1%
6653	35	20	40	65	58,1%	65,4%	90,5%	94,9%	90,7%
6654	35	20	40	70	58,5%	65,1%	90,8%	95,3%	91,3%
6655	35	20	40	75	58,9%	64,8%	91,1%	95,6%	91,8%
6656	35	20	40	80	59,3%	64,5%	91,3%	95,8%	92,3%
6657	35	20	45	20	48,0%	62,8%	86,1%	90,5%	83,5%
6658	35	20	45	25	48,4%	62,5%	86,5%	91,0%	84,5%
6659	35	20	45	30	48,9%	62,2%	86,9%	91,6%	85,3%
6660	35	20	45	35	49,3%	61,9%	87,3%	92,1%	86,2%
6661	35	20	45	40	49,8%	61,6%	87,6%	92,6%	87,0%
6662	35	20	45	45	50,2%	61,3%	88,0%	93,0%	87,7%
6663	35	20	45	50	50,6%	61,0%	88,4%	93,5%	88,4%
6664	35	20	45	55	51,1%	60,7%	88,7%	93,9%	89,1%
6665	35	20	45	60	51,5%	60,4%	89,0%	94,3%	89,8%

6666	35	20	45	65	52,0%	60,0%	89,4%	94,6%	90,4%
6667	35	20	45	70	52,4%	59,7%	89,7%	95,0%	91,0%
6668	35	20	45	75	52,8%	59,4%	90,0%	95,3%	91,5%
6669	35	20	45	80	53,3%	59,1%	90,3%	95,6%	92,0%
6670	35	20	50	20	41,9%	57,3%	84,5%	89,9%	83,0%
6671	35	20	50	25	42,3%	57,0%	85,0%	90,5%	83,9%
6672	35	20	50	30	42,8%	56,7%	85,4%	91,1%	84,8%
6673	35	20	50	35	43,2%	56,3%	85,8%	91,6%	85,7%
6674	35	20	50	40	43,6%	56,0%	86,2%	92,2%	86,5%
6675	35	20	50	45	44,1%	55,7%	86,6%	92,6%	87,3%
6676	35	20	50	50	44,5%	55,4%	87,0%	93,1%	88,0%
6677	35	20	50	55	44,9%	55,0%	87,4%	93,5%	88,7%
6678	35	20	50	60	45,4%	54,7%	87,8%	93,9%	89,4%
6679	35	20	50	65	45,8%	54,4%	88,1%	94,3%	90,0%
6680	35	20	50	70	46,2%	54,1%	88,5%	94,7%	90,6%
6681	35	20	50	75	46,7%	53,7%	88,8%	95,0%	91,2%
6682	35	20	50	80	47,1%	53,4%	89,2%	95,3%	91,7%
6683	35	20	55	20	36,0%	51,6%	82,8%	89,3%	82,4%
6684	35	20	55	25	36,4%	51,2%	83,3%	90,0%	83,3%
6685	35	20	55	30	36,9%	50,9%	83,8%	90,6%	84,3%
6686	35	20	55	35	37,3%	50,6%	84,2%	91,2%	85,2%
6687	35	20	55	40	37,7%	50,3%	84,7%	91,7%	86,0%
6688	35	20	55	45	38,1%	49,9%	85,1%	92,2%	86,8%
6689	35	20	55	50	38,5%	49,6%	85,6%	92,7%	87,6%
6690	35	20	55	55	38,9%	49,3%	86,0%	93,1%	88,3%
6691	35	20	55	60	39,3%	49,0%	86,4%	93,6%	89,0%
6692	35	20	55	65	39,8%	48,6%	86,8%	94,0%	89,6%
6693	35	20	55	70	40,2%	48,3%	87,2%	94,4%	90,3%
6694	35	20	55	75	40,6%	48,0%	87,5%	94,7%	90,8%
6695	35	20	55	80	41,0%	47,7%	87,9%	95,0%	91,4%
6696	35	20	60	20	30,6%	45,8%	81,0%	88,8%	81,8%
6697	35	20	60	25	30,9%	45,5%	81,5%	89,4%	82,8%
6698	35	20	60	30	31,3%	45,2%	82,0%	90,1%	83,7%

6699	35	20	60	35	31,7%	44,8%	82,5%	90,7%	84,6%
6700	35	20	60	40	32,1%	44,5%	83,0%	91,2%	85,5%
6701	35	20	60	45	32,5%	44,2%	83,5%	91,8%	86,3%
6702	35	20	60	50	32,9%	43,9%	83,9%	92,3%	87,1%
6703	35	20	60	55	33,2%	43,6%	84,4%	92,7%	87,9%
6704	35	20	60	60	33,6%	43,2%	84,8%	93,2%	88,6%
6705	35	20	60	65	34,0%	42,9%	85,3%	93,6%	89,3%
6706	35	20	60	70	34,4%	42,6%	85,7%	94,0%	89,9%
6707	35	20	60	75	34,8%	42,3%	86,1%	94,4%	90,5%
6708	35	20	60	80	35,2%	41,9%	86,5%	94,8%	91,1%
6709	35	20	65	20	25,6%	40,2%	79,0%	88,1%	81,1%
6710	35	20	65	25	25,9%	39,8%	79,6%	88,8%	82,2%
6711	35	20	65	30	26,3%	39,5%	80,1%	89,5%	83,1%
6712	35	20	65	35	26,6%	39,2%	80,7%	90,1%	84,1%
6713	35	20	65	40	27,0%	38,9%	81,2%	90,7%	85,0%
6714	35	20	65	45	27,3%	38,6%	81,7%	91,3%	85,8%
6715	35	20	65	50	27,7%	38,3%	82,2%	91,8%	86,6%
6716	35	20	65	55	28,0%	38,0%	82,7%	92,3%	87,4%
6717	35	20	65	60	28,4%	37,7%	83,2%	92,8%	88,2%
6718	35	20	65	65	28,7%	37,4%	83,6%	93,2%	88,8%
6719	35	20	65	70	29,1%	37,1%	84,1%	93,7%	89,5%
6720	35	20	65	75	29,4%	36,8%	84,6%	94,1%	90,1%
6721	35	20	65	80	29,8%	36,5%	85,0%	94,4%	90,7%
6722	35	20	70	20	21,2%	34,8%	76,9%	87,5%	80,5%
6723	35	20	70	25	21,5%	34,5%	77,5%	88,2%	81,6%
6724	35	20	70	30	21,8%	34,2%	78,1%	88,9%	82,6%
6725	35	20	70	35	22,1%	33,9%	78,6%	89,6%	83,5%
6726	35	20	70	40	22,4%	33,6%	79,2%	90,2%	84,4%
6727	35	20	70	45	22,7%	33,3%	79,8%	90,8%	85,3%
6728	35	20	70	50	23,0%	33,0%	80,3%	91,4%	86,2%
6729	35	20	70	55	23,3%	32,7%	80,8%	91,9%	87,0%
6730	35	20	70	60	23,6%	32,4%	81,4%	92,4%	87,7%
6731	35	20	70	65	23,9%	32,1%	81,9%	92,9%	88,4%

6732	35	20	70	70	24,3%	31,9%	82,4%	93,3%	89,1%
6733	35	20	70	75	24,6%	31,6%	82,9%	93,7%	89,8%
6734	35	20	70	80	24,9%	31,3%	83,3%	94,1%	90,4%
6735	35	20	75	20	17,3%	29,7%	74,6%	86,8%	79,8%
6736	35	20	75	25	17,6%	29,4%	75,2%	87,6%	80,9%
6737	35	20	75	30	17,9%	29,2%	75,9%	88,3%	82,0%
6738	35	20	75	35	18,1%	28,9%	76,5%	89,0%	82,9%
6739	35	20	75	40	18,4%	28,6%	77,1%	89,6%	83,9%
6740	35	20	75	45	18,6%	28,4%	77,7%	90,3%	84,8%
6741	35	20	75	50	18,9%	28,1%	78,3%	90,9%	85,7%
6742	35	20	75	55	19,2%	27,8%	78,8%	91,4%	86,5%
6743	35	20	75	60	19,5%	27,6%	79,4%	91,9%	87,3%
6744	35	20	75	65	19,7%	27,3%	80,0%	92,4%	88,0%
6745	35	20	75	70	20,0%	27,1%	80,5%	92,9%	88,7%
6746	35	20	75	75	20,3%	26,8%	81,0%	93,3%	89,4%
6747	35	20	75	80	20,6%	26,5%	81,5%	93,8%	90,0%
6748	35	20	80	20	14,1%	25,1%	72,2%	86,1%	79,2%
6749	35	20	80	25	14,3%	24,9%	72,9%	86,9%	80,3%
6750	35	20	80	30	14,5%	24,6%	73,5%	87,7%	81,3%
6751	35	20	80	35	14,7%	24,4%	74,2%	88,4%	82,4%
6752	35	20	80	40	15,0%	24,2%	74,8%	89,1%	83,3%
6753	35	20	80	45	15,2%	23,9%	75,5%	89,7%	84,3%
6754	35	20	80	50	15,4%	23,7%	76,1%	90,3%	85,1%
6755	35	20	80	55	15,6%	23,4%	76,7%	90,9%	86,0%
6756	35	20	80	60	15,9%	23,2%	77,3%	91,5%	86,8%
6757	35	20	80	65	16,1%	23,0%	77,9%	92,0%	87,6%
6758	35	20	80	70	16,4%	22,8%	78,5%	92,5%	88,3%
6759	35	20	80	75	16,6%	22,5%	79,0%	93,0%	89,0%
6760	35	20	80	80	16,8%	22,3%	79,6%	93,4%	89,6%
6761	35	25	20	20	77,6%	85,3%	91,8%	92,3%	85,0%
6762	35	25	20	25	77,9%	85,1%	92,0%	92,7%	85,9%
6763	35	25	20	30	78,2%	85,0%	92,3%	93,2%	86,7%
6764	35	25	20	35	78,5%	84,8%	92,5%	93,6%	87,4%

6765	35	25	20	40	78,8%	84,6%	92,7%	94,0%	88,2%
6766	35	25	20	45	79,1%	84,5%	93,0%	94,4%	88,9%
6767	35	25	20	50	79,4%	84,3%	93,2%	94,8%	89,5%
6768	35	25	20	55	79,7%	84,1%	93,4%	95,1%	90,2%
6769	35	25	20	60	80,0%	83,9%	93,6%	95,4%	90,7%
6770	35	25	20	65	80,2%	83,8%	93,8%	95,7%	91,3%
6771	35	25	20	70	80,5%	83,6%	94,0%	96,0%	91,8%
6772	35	25	20	75	80,8%	83,4%	94,2%	96,2%	92,3%
6773	35	25	20	80	81,1%	83,2%	94,4%	96,5%	92,8%
6774	35	25	25	20	73,0%	82,2%	90,8%	91,8%	84,5%
6775	35	25	25	25	73,4%	82,0%	91,1%	92,3%	85,3%
6776	35	25	25	30	73,7%	81,8%	91,3%	92,8%	86,2%
6777	35	25	25	35	74,1%	81,6%	91,6%	93,2%	87,0%
6778	35	25	25	40	74,4%	81,4%	91,9%	93,7%	87,7%
6779	35	25	25	45	74,7%	81,2%	92,1%	94,1%	88,5%
6780	35	25	25	50	75,1%	81,0%	92,4%	94,4%	89,1%
6781	35	25	25	55	75,4%	80,8%	92,6%	94,8%	89,8%
6782	35	25	25	60	75,7%	80,6%	92,8%	95,1%	90,4%
6783	35	25	25	65	76,0%	80,4%	93,0%	95,4%	91,0%
6784	35	25	25	70	76,4%	80,2%	93,3%	95,7%	91,5%
6785	35	25	25	75	76,7%	80,0%	93,5%	96,0%	92,0%
6786	35	25	25	80	77,0%	79,7%	93,7%	96,3%	92,5%
6787	35	25	30	20	67,9%	78,5%	89,7%	91,4%	83,9%
6788	35	25	30	25	68,3%	78,3%	90,0%	91,9%	84,8%
6789	35	25	30	30	68,7%	78,1%	90,3%	92,4%	85,7%
6790	35	25	30	35	69,0%	77,9%	90,6%	92,9%	86,5%
6791	35	25	30	40	69,4%	77,6%	90,9%	93,3%	87,3%
6792	35	25	30	45	69,8%	77,4%	91,2%	93,7%	88,0%
6793	35	25	30	50	70,2%	77,2%	91,4%	94,1%	88,7%
6794	35	25	30	55	70,5%	76,9%	91,7%	94,5%	89,4%
6795	35	25	30	60	70,9%	76,7%	92,0%	94,8%	90,0%
6796	35	25	30	65	71,3%	76,5%	92,2%	95,2%	90,6%
6797	35	25	30	70	71,6%	76,2%	92,4%	95,5%	91,2%

6798	35	25	30	75	72,0%	76,0%	92,7%	95,8%	91,7%
6799	35	25	30	80	72,3%	75,8%	92,9%	96,0%	92,2%
6800	35	25	35	20	62,3%	74,4%	88,5%	90,9%	83,4%
6801	35	25	35	25	62,7%	74,1%	88,8%	91,4%	84,3%
6802	35	25	35	30	63,1%	73,9%	89,2%	91,9%	85,2%
6803	35	25	35	35	63,5%	73,6%	89,5%	92,4%	86,0%
6804	35	25	35	40	63,9%	73,4%	89,8%	92,9%	86,8%
6805	35	25	35	45	64,4%	73,1%	90,1%	93,3%	87,6%
6806	35	25	35	50	64,8%	72,9%	90,4%	93,8%	88,3%
6807	35	25	35	55	65,2%	72,6%	90,7%	94,2%	89,0%
6808	35	25	35	60	65,6%	72,3%	91,0%	94,5%	89,6%
6809	35	25	35	65	66,0%	72,1%	91,3%	94,9%	90,3%
6810	35	25	35	70	66,3%	71,8%	91,5%	95,2%	90,9%
6811	35	25	35	75	66,7%	71,5%	91,8%	95,5%	91,4%
6812	35	25	35	80	67,1%	71,3%	92,0%	95,8%	91,9%
6813	35	25	40	20	56,4%	69,7%	87,2%	90,3%	82,8%
6814	35	25	40	25	56,8%	69,5%	87,5%	90,9%	83,7%
6815	35	25	40	30	57,2%	69,2%	87,9%	91,5%	84,6%
6816	35	25	40	35	57,7%	68,9%	88,3%	92,0%	85,5%
6817	35	25	40	40	58,1%	68,6%	88,6%	92,5%	86,3%
6818	35	25	40	45	58,5%	68,3%	88,9%	93,0%	87,1%
6819	35	25	40	50	58,9%	68,1%	89,3%	93,4%	87,9%
6820	35	25	40	55	59,4%	67,8%	89,6%	93,8%	88,6%
6821	35	25	40	60	59,8%	67,5%	89,9%	94,2%	89,3%
6822	35	25	40	65	60,2%	67,2%	90,2%	94,6%	89,9%
6823	35	25	40	70	60,6%	66,9%	90,5%	94,9%	90,5%
6824	35	25	40	75	61,1%	66,6%	90,8%	95,2%	91,1%
6825	35	25	40	80	61,5%	66,3%	91,1%	95,5%	91,6%
6826	35	25	45	20	50,2%	64,7%	85,7%	89,8%	82,2%
6827	35	25	45	25	50,7%	64,4%	86,1%	90,4%	83,2%
6828	35	25	45	30	51,1%	64,1%	86,5%	91,0%	84,1%
6829	35	25	45	35	51,5%	63,7%	86,9%	91,5%	85,0%
6830	35	25	45	40	52,0%	63,4%	87,3%	92,1%	85,8%

6831	35	25	45	45	52,4%	63,1%	87,7%	92,5%	86,7%
6832	35	25	45	50	52,9%	62,8%	88,0%	93,0%	87,4%
6833	35	25	45	55	53,3%	62,5%	88,4%	93,4%	88,2%
6834	35	25	45	60	53,7%	62,2%	88,7%	93,9%	88,9%
6835	35	25	45	65	54,2%	61,9%	89,1%	94,2%	89,5%
6836	35	25	45	70	54,6%	61,6%	89,4%	94,6%	90,1%
6837	35	25	45	75	55,0%	61,3%	89,7%	94,9%	90,7%
6838	35	25	45	80	55,5%	61,0%	90,0%	95,3%	91,3%
6839	35	25	50	20	44,1%	59,2%	84,1%	89,2%	81,6%
6840	35	25	50	25	44,5%	58,9%	84,6%	89,9%	82,6%
6841	35	25	50	30	44,9%	58,6%	85,0%	90,5%	83,5%
6842	35	25	50	35	45,4%	58,3%	85,4%	91,1%	84,5%
6843	35	25	50	40	45,8%	57,9%	85,9%	91,6%	85,3%
6844	35	25	50	45	46,3%	57,6%	86,3%	92,1%	86,2%
6845	35	25	50	50	46,7%	57,3%	86,7%	92,6%	87,0%
6846	35	25	50	55	47,1%	57,0%	87,0%	93,1%	87,7%
6847	35	25	50	60	47,6%	56,7%	87,4%	93,5%	88,4%
6848	35	25	50	65	48,0%	56,3%	87,8%	93,9%	89,1%
6849	35	25	50	70	48,5%	56,0%	88,2%	94,3%	89,8%
6850	35	25	50	75	48,9%	55,7%	88,5%	94,6%	90,4%
6851	35	25	50	80	49,3%	55,4%	88,8%	95,0%	91,0%
6852	35	25	55	20	38,1%	53,5%	82,4%	88,6%	80,9%
6853	35	25	55	25	38,5%	53,2%	82,9%	89,3%	82,0%
6854	35	25	55	30	38,9%	52,9%	83,4%	89,9%	83,0%
6855	35	25	55	35	39,4%	52,6%	83,8%	90,5%	83,9%
6856	35	25	55	40	39,8%	52,2%	84,3%	91,1%	84,8%
6857	35	25	55	45	40,2%	51,9%	84,7%	91,7%	85,7%
6858	35	25	55	50	40,6%	51,6%	85,2%	92,2%	86,5%
6859	35	25	55	55	41,1%	51,3%	85,6%	92,7%	87,3%
6860	35	25	55	60	41,5%	50,9%	86,0%	93,1%	88,0%
6861	35	25	55	65	41,9%	50,6%	86,4%	93,5%	88,7%
6862	35	25	55	70	42,3%	50,3%	86,8%	93,9%	89,4%
6863	35	25	55	75	42,8%	49,9%	87,2%	94,3%	90,0%

6864	35	25	55	80	43,2%	49,6%	87,5%	94,7%	90,6%
6865	35	25	60	20	32,5%	47,8%	80,5%	88,0%	80,3%
6866	35	25	60	25	32,9%	47,4%	81,0%	88,7%	81,4%
6867	35	25	60	30	33,3%	47,1%	81,6%	89,4%	82,4%
6868	35	25	60	35	33,6%	46,8%	82,1%	90,0%	83,3%
6869	35	25	60	40	34,0%	46,5%	82,6%	90,6%	84,3%
6870	35	25	60	45	34,4%	46,1%	83,0%	91,2%	85,2%
6871	35	25	60	50	34,8%	45,8%	83,5%	91,7%	86,0%
6872	35	25	60	55	35,2%	45,5%	84,0%	92,2%	86,8%
6873	35	25	60	60	35,6%	45,2%	84,4%	92,7%	87,6%
6874	35	25	60	65	36,0%	44,8%	84,9%	93,2%	88,3%
6875	35	25	60	70	36,5%	44,5%	85,3%	93,6%	89,0%
6876	35	25	60	75	36,9%	44,2%	85,7%	94,0%	89,6%
6877	35	25	60	80	37,3%	43,9%	86,1%	94,4%	90,3%
6878	35	25	65	20	27,3%	42,1%	78,5%	87,3%	79,6%
6879	35	25	65	25	27,7%	41,7%	79,1%	88,1%	80,7%
6880	35	25	65	30	28,0%	41,4%	79,6%	88,8%	81,8%
6881	35	25	65	35	28,4%	41,1%	80,2%	89,5%	82,8%
6882	35	25	65	40	28,7%	40,8%	80,7%	90,1%	83,7%
6883	35	25	65	45	29,1%	40,5%	81,2%	90,7%	84,6%
6884	35	25	65	50	29,5%	40,2%	81,7%	91,3%	85,5%
6885	35	25	65	55	29,8%	39,9%	82,2%	91,8%	86,3%
6886	35	25	65	60	30,2%	39,5%	82,7%	92,3%	87,1%
6887	35	25	65	65	30,6%	39,2%	83,2%	92,8%	87,9%
6888	35	25	65	70	30,9%	38,9%	83,7%	93,2%	88,6%
6889	35	25	65	75	31,3%	38,6%	84,1%	93,6%	89,3%
6890	35	25	65	80	31,7%	38,3%	84,6%	94,0%	89,9%
6891	35	25	70	20	22,7%	36,6%	76,3%	86,6%	79,0%
6892	35	25	70	25	23,0%	36,3%	76,9%	87,4%	80,1%
6893	35	25	70	30	23,3%	36,0%	77,5%	88,2%	81,1%
6894	35	25	70	35	23,6%	35,7%	78,1%	88,9%	82,2%
6895	35	25	70	40	24,0%	35,4%	78,7%	89,5%	83,1%
6896	35	25	70	45	24,3%	35,1%	79,2%	90,2%	84,1%

6897	35	25	70	50	24,6%	34,8%	79,8%	90,8%	85,0%
6898	35	25	70	55	24,9%	34,5%	80,3%	91,3%	85,8%
6899	35	25	70	60	25,3%	34,2%	80,9%	91,8%	86,6%
6900	35	25	70	65	25,6%	33,9%	81,4%	92,3%	87,4%
6901	35	25	70	70	25,9%	33,6%	81,9%	92,8%	88,2%
6902	35	25	70	75	26,3%	33,3%	82,4%	93,3%	88,9%
6903	35	25	70	80	26,6%	33,0%	82,9%	93,7%	89,5%
6904	35	25	75	20	18,7%	31,4%	74,0%	85,9%	78,3%
6905	35	25	75	25	18,9%	31,1%	74,6%	86,7%	79,4%
6906	35	25	75	30	19,2%	30,8%	75,3%	87,5%	80,5%
6907	35	25	75	35	19,5%	30,6%	75,9%	88,2%	81,6%
6908	35	25	75	40	19,7%	30,3%	76,5%	88,9%	82,6%
6909	35	25	75	45	20,0%	30,0%	77,1%	89,6%	83,5%
6910	35	25	75	50	20,3%	29,7%	77,7%	90,2%	84,4%
6911	35	25	75	55	20,6%	29,5%	78,3%	90,8%	85,3%
6912	35	25	75	60	20,9%	29,2%	78,9%	91,4%	86,2%
6913	35	25	75	65	21,2%	28,9%	79,4%	91,9%	87,0%
6914	35	25	75	70	21,5%	28,6%	80,0%	92,4%	87,7%
6915	35	25	75	75	21,8%	28,4%	80,5%	92,9%	88,4%
6916	35	25	75	80	22,1%	28,1%	81,1%	93,3%	89,1%
6917	35	25	80	20	15,2%	26,6%	71,5%	85,2%	77,6%
6918	35	25	80	25	15,4%	26,4%	72,2%	86,0%	78,7%
6919	35	25	80	30	15,7%	26,1%	72,9%	86,8%	79,8%
6920	35	25	80	35	15,9%	25,9%	73,6%	87,6%	80,9%
6921	35	25	80	40	16,1%	25,6%	74,2%	88,3%	82,0%
6922	35	25	80	45	16,4%	25,4%	74,9%	89,0%	83,0%
6923	35	25	80	50	16,6%	25,1%	75,5%	89,7%	83,9%
6924	35	25	80	55	16,9%	24,9%	76,1%	90,3%	84,8%
6925	35	25	80	60	17,1%	24,6%	76,7%	90,9%	85,7%
6926	35	25	80	65	17,4%	24,4%	77,3%	91,4%	86,5%
6927	35	25	80	70	17,6%	24,2%	77,9%	92,0%	87,3%
6928	35	25	80	75	17,9%	23,9%	78,5%	92,5%	88,0%
6929	35	25	80	80	18,1%	23,7%	79,1%	92,9%	88,7%

6930	35	30	20	20	79,1%	86,3%	91,5%	91,7%	83,7%
6931	35	30	20	25	79,4%	86,1%	91,8%	92,2%	84,7%
6932	35	30	20	30	79,7%	85,9%	92,0%	92,7%	85,5%
6933	35	30	20	35	80,0%	85,8%	92,3%	93,2%	86,3%
6934	35	30	20	40	80,3%	85,6%	92,5%	93,6%	87,1%
6935	35	30	20	45	80,5%	85,5%	92,8%	94,0%	87,9%
6936	35	30	20	50	80,8%	85,3%	93,0%	94,4%	88,6%
6937	35	30	20	55	81,1%	85,1%	93,2%	94,7%	89,3%
6938	35	30	20	60	81,4%	85,0%	93,4%	95,1%	89,9%
6939	35	30	20	65	81,6%	84,8%	93,6%	95,4%	90,5%
6940	35	30	20	70	81,9%	84,6%	93,8%	95,7%	91,1%
6941	35	30	20	75	82,1%	84,5%	94,0%	95,9%	91,6%
6942	35	30	20	80	82,4%	84,3%	94,2%	96,2%	92,1%
6943	35	30	25	20	74,7%	83,3%	90,5%	91,2%	83,2%
6944	35	30	25	25	75,1%	83,1%	90,8%	91,8%	84,1%
6945	35	30	25	30	75,4%	82,9%	91,1%	92,3%	85,0%
6946	35	30	25	35	75,7%	82,7%	91,4%	92,8%	85,9%
6947	35	30	25	40	76,1%	82,5%	91,6%	93,2%	86,7%
6948	35	30	25	45	76,4%	82,4%	91,9%	93,6%	87,4%
6949	35	30	25	50	76,7%	82,2%	92,1%	94,0%	88,2%
6950	35	30	25	55	77,0%	82,0%	92,4%	94,4%	88,9%
6951	35	30	25	60	77,3%	81,8%	92,6%	94,8%	89,5%
6952	35	30	25	65	77,6%	81,6%	92,8%	95,1%	90,2%
6953	35	30	25	70	77,9%	81,4%	93,1%	95,4%	90,7%
6954	35	30	25	75	78,2%	81,2%	93,3%	95,7%	91,3%
6955	35	30	25	80	78,5%	81,0%	93,5%	96,0%	91,8%
6956	35	30	30	20	69,8%	79,8%	89,4%	90,7%	82,6%
6957	35	30	30	25	70,2%	79,6%	89,7%	91,3%	83,5%
6958	35	30	30	30	70,5%	79,4%	90,0%	91,8%	84,5%
6959	35	30	30	35	70,9%	79,2%	90,3%	92,3%	85,3%
6960	35	30	30	40	71,3%	79,0%	90,6%	92,8%	86,2%
6961	35	30	30	45	71,6%	78,7%	90,9%	93,3%	87,0%
6962	35	30	30	50	72,0%	78,5%	91,2%	93,7%	87,7%

6963	35	30	30	55	72,3%	78,3%	91,5%	94,1%	88,5%
6964	35	30	30	60	72,7%	78,1%	91,7%	94,5%	89,1%
6965	35	30	30	65	73,0%	77,9%	92,0%	94,8%	89,8%
6966	35	30	30	70	73,4%	77,6%	92,2%	95,1%	90,4%
6967	35	30	30	75	73,7%	77,4%	92,5%	95,4%	91,0%
6968	35	30	30	80	74,1%	77,2%	92,7%	95,7%	91,5%
6969	35	30	35	20	64,4%	75,8%	88,2%	90,2%	82,0%
6970	35	30	35	25	64,8%	75,6%	88,5%	90,8%	83,0%
6971	35	30	35	30	65,2%	75,4%	88,9%	91,4%	83,9%
6972	35	30	35	35	65,6%	75,1%	89,2%	91,9%	84,8%
6973	35	30	35	40	66,0%	74,9%	89,5%	92,4%	85,7%
6974	35	30	35	45	66,4%	74,6%	89,8%	92,9%	86,5%
6975	35	30	35	50	66,8%	74,4%	90,1%	93,3%	87,3%
6976	35	30	35	55	67,1%	74,1%	90,4%	93,7%	88,0%
6977	35	30	35	60	67,5%	73,9%	90,7%	94,1%	88,7%
6978	35	30	35	65	67,9%	73,6%	91,0%	94,5%	89,4%
6979	35	30	35	70	68,3%	73,4%	91,3%	94,8%	90,0%
6980	35	30	35	75	68,7%	73,1%	91,5%	95,2%	90,6%
6981	35	30	35	80	69,1%	72,9%	91,8%	95,5%	91,2%
6982	35	30	40	20	58,5%	71,4%	86,8%	89,7%	81,4%
6983	35	30	40	25	59,0%	71,1%	87,2%	90,3%	82,4%
6984	35	30	40	30	59,4%	70,8%	87,6%	90,9%	83,4%
6985	35	30	40	35	59,8%	70,6%	87,9%	91,4%	84,3%
6986	35	30	40	40	60,2%	70,3%	88,3%	92,0%	85,2%
6987	35	30	40	45	60,6%	70,0%	88,6%	92,5%	86,0%
6988	35	30	40	50	61,1%	69,7%	89,0%	92,9%	86,8%
6989	35	30	40	55	61,5%	69,5%	89,3%	93,4%	87,6%
6990	35	30	40	60	61,9%	69,2%	89,6%	93,8%	88,3%
6991	35	30	40	65	62,3%	68,9%	89,9%	94,2%	89,0%
6992	35	30	40	70	62,7%	68,6%	90,2%	94,5%	89,7%
6993	35	30	40	75	63,1%	68,3%	90,5%	94,9%	90,3%
6994	35	30	40	80	63,5%	68,1%	90,8%	95,2%	90,9%
6995	35	30	45	20	52,4%	66,4%	85,3%	89,1%	80,7%

6996	35	30	45	25	52,9%	66,1%	85,7%	89,7%	81,8%
6997	35	30	45	30	53,3%	65,8%	86,1%	90,4%	82,8%
6998	35	30	45	35	53,8%	65,5%	86,5%	90,9%	83,7%
6999	35	30	45	40	54,2%	65,3%	86,9%	91,5%	84,6%
7000	35	30	45	45	54,6%	65,0%	87,3%	92,0%	85,5%
7001	35	30	45	50	55,1%	64,7%	87,7%	92,5%	86,3%
7002	35	30	45	55	55,5%	64,4%	88,1%	93,0%	87,1%
7003	35	30	45	60	55,9%	64,1%	88,4%	93,4%	87,9%
7004	35	30	45	65	56,4%	63,8%	88,7%	93,8%	88,6%
7005	35	30	45	70	56,8%	63,5%	89,1%	94,2%	89,3%
7006	35	30	45	75	57,2%	63,1%	89,4%	94,6%	89,9%
7007	35	30	45	80	57,7%	62,8%	89,7%	94,9%	90,5%
7008	35	30	50	20	46,3%	61,1%	83,7%	88,5%	80,1%
7009	35	30	50	25	46,7%	60,8%	84,1%	89,2%	81,2%
7010	35	30	50	30	47,1%	60,5%	84,6%	89,8%	82,2%
7011	35	30	50	35	47,6%	60,2%	85,0%	90,4%	83,2%
7012	35	30	50	40	48,0%	59,8%	85,5%	91,0%	84,1%
7013	35	30	50	45	48,5%	59,5%	85,9%	91,6%	85,0%
7014	35	30	50	50	48,9%	59,2%	86,3%	92,1%	85,8%
7015	35	30	50	55	49,3%	58,9%	86,7%	92,6%	86,7%
7016	35	30	50	60	49,8%	58,6%	87,1%	93,0%	87,4%
7017	35	30	50	65	50,2%	58,3%	87,4%	93,5%	88,2%
7018	35	30	50	70	50,7%	57,9%	87,8%	93,9%	88,9%
7019	35	30	50	75	51,1%	57,6%	88,2%	94,3%	89,5%
7020	35	30	50	80	51,6%	57,3%	88,5%	94,6%	90,1%
7021	35	30	55	20	40,2%	55,5%	81,9%	87,9%	79,4%
7022	35	30	55	25	40,6%	55,2%	82,4%	88,6%	80,5%
7023	35	30	55	30	41,1%	54,8%	82,9%	89,3%	81,6%
7024	35	30	55	35	41,5%	54,5%	83,4%	89,9%	82,6%
7025	35	30	55	40	41,9%	54,2%	83,8%	90,5%	83,5%
7026	35	30	55	45	42,4%	53,9%	84,3%	91,1%	84,5%
7027	35	30	55	50	42,8%	53,5%	84,7%	91,6%	85,3%
7028	35	30	55	55	43,2%	53,2%	85,2%	92,1%	86,2%

7029	35	30	55	60	43,7%	52,9%	85,6%	92,6%	87,0%
7030	35	30	55	65	44,1%	52,6%	86,0%	93,1%	87,7%
7031	35	30	55	70	44,5%	52,2%	86,4%	93,5%	88,4%
7032	35	30	55	75	45,0%	51,9%	86,8%	93,9%	89,1%
7033	35	30	55	80	45,4%	51,6%	87,2%	94,3%	89,8%
7034	35	30	60	20	34,5%	49,7%	80,0%	87,2%	78,7%
7035	35	30	60	25	34,9%	49,4%	80,5%	87,9%	79,9%
7036	35	30	60	30	35,3%	49,1%	81,1%	88,7%	80,9%
7037	35	30	60	35	35,7%	48,8%	81,6%	89,3%	82,0%
7038	35	30	60	40	36,1%	48,4%	82,1%	90,0%	83,0%
7039	35	30	60	45	36,5%	48,1%	82,6%	90,6%	83,9%
7040	35	30	60	50	36,9%	47,8%	83,1%	91,1%	84,8%
7041	35	30	60	55	37,3%	47,5%	83,5%	91,7%	85,7%
7042	35	30	60	60	37,7%	47,1%	84,0%	92,2%	86,5%
7043	35	30	60	65	38,1%	46,8%	84,5%	92,7%	87,3%
7044	35	30	60	70	38,5%	46,5%	84,9%	93,1%	88,0%
7045	35	30	60	75	39,0%	46,2%	85,3%	93,6%	88,7%
7046	35	30	60	80	39,4%	45,8%	85,7%	94,0%	89,4%
7047	35	30	65	20	29,1%	44,0%	77,9%	86,5%	78,0%
7048	35	30	65	25	29,5%	43,7%	78,5%	87,3%	79,2%
7049	35	30	65	30	29,8%	43,4%	79,1%	88,0%	80,3%
7050	35	30	65	35	30,2%	43,0%	79,6%	88,7%	81,4%
7051	35	30	65	40	30,6%	42,7%	80,2%	89,4%	82,4%
7052	35	30	65	45	31,0%	42,4%	80,7%	90,0%	83,3%
7053	35	30	65	50	31,3%	42,1%	81,3%	90,6%	84,3%
7054	35	30	65	55	31,7%	41,8%	81,8%	91,2%	85,2%
7055	35	30	65	60	32,1%	41,4%	82,3%	91,7%	86,0%
7056	35	30	65	65	32,5%	41,1%	82,8%	92,3%	86,8%
7057	35	30	65	70	32,9%	40,8%	83,2%	92,7%	87,6%
7058	35	30	65	75	33,3%	40,5%	83,7%	93,2%	88,3%
7059	35	30	65	80	33,7%	40,2%	84,2%	93,6%	89,0%
7060	35	30	70	20	24,3%	38,4%	75,7%	85,8%	77,3%
7061	35	30	70	25	24,6%	38,1%	76,4%	86,6%	78,5%

7062	35	30	70	30	24,9%	37,8%	77,0%	87,4%	79,6%
7063	35	30	70	35	25,3%	37,5%	77,6%	88,1%	80,7%
7064	35	30	70	40	25,6%	37,2%	78,1%	88,8%	81,8%
7065	35	30	70	45	25,9%	36,9%	78,7%	89,5%	82,8%
7066	35	30	70	50	26,3%	36,6%	79,3%	90,1%	83,7%
7067	35	30	70	55	26,6%	36,3%	79,8%	90,7%	84,6%
7068	35	30	70	60	27,0%	36,0%	80,4%	91,3%	85,5%
7069	35	30	70	65	27,3%	35,7%	80,9%	91,8%	86,3%
7070	35	30	70	70	27,7%	35,4%	81,4%	92,3%	87,1%
7071	35	30	70	75	28,0%	35,1%	81,9%	92,8%	87,9%
7072	35	30	70	80	28,4%	34,8%	82,4%	93,2%	88,6%
7073	35	30	75	20	20,0%	33,1%	73,4%	85,0%	76,6%
7074	35	30	75	25	20,3%	32,8%	74,0%	85,9%	77,8%
7075	35	30	75	30	20,6%	32,5%	74,7%	86,7%	79,0%
7076	35	30	75	35	20,9%	32,2%	75,3%	87,5%	80,1%
7077	35	30	75	40	21,2%	32,0%	76,0%	88,2%	81,1%
7078	35	30	75	45	21,5%	31,7%	76,6%	88,9%	82,2%
7079	35	30	75	50	21,8%	31,4%	77,2%	89,6%	83,2%
7080	35	30	75	55	22,1%	31,1%	77,8%	90,2%	84,1%
7081	35	30	75	60	22,4%	30,8%	78,3%	90,8%	85,0%
7082	35	30	75	65	22,7%	30,6%	78,9%	91,3%	85,8%
7083	35	30	75	70	23,0%	30,3%	79,5%	91,9%	86,7%
7084	35	30	75	75	23,3%	30,0%	80,0%	92,4%	87,4%
7085	35	30	75	80	23,6%	29,7%	80,6%	92,8%	88,2%
7086	35	30	80	20	16,4%	28,2%	70,9%	84,2%	75,8%
7087	35	30	80	25	16,6%	27,9%	71,6%	85,1%	77,1%
7088	35	30	80	30	16,9%	27,7%	72,3%	86,0%	78,3%
7089	35	30	80	35	17,1%	27,4%	72,9%	86,8%	79,4%
7090	35	30	80	40	17,4%	27,2%	73,6%	87,5%	80,5%
7091	35	30	80	45	17,6%	26,9%	74,3%	88,3%	81,6%
7092	35	30	80	50	17,9%	26,6%	74,9%	89,0%	82,6%
7093	35	30	80	55	18,1%	26,4%	75,5%	89,6%	83,5%
7094	35	30	80	60	18,4%	26,1%	76,2%	90,3%	84,5%

7095	35	30	80	65	18,7%	25,9%	76,8%	90,8%	85,3%
7096	35	30	80	70	18,9%	25,6%	77,4%	91,4%	86,2%
7097	35	30	80	75	19,2%	25,4%	78,0%	91,9%	87,0%
7098	35	30	80	80	19,5%	25,1%	78,5%	92,4%	87,7%
7099	35	35	20	20	80,5%	87,2%	91,3%	91,1%	82,4%
7100	35	35	20	25	80,8%	87,0%	91,6%	91,7%	83,4%
7101	35	35	20	30	81,1%	86,9%	91,8%	92,2%	84,3%
7102	35	35	20	35	81,4%	86,7%	92,1%	92,7%	85,2%
7103	35	35	20	40	81,6%	86,6%	92,3%	93,1%	86,0%
7104	35	35	20	45	81,9%	86,4%	92,5%	93,6%	86,8%
7105	35	35	20	50	82,1%	86,3%	92,8%	94,0%	87,6%
7106	35	35	20	55	82,4%	86,1%	93,0%	94,3%	88,3%
7107	35	35	20	60	82,7%	86,0%	93,2%	94,7%	89,0%
7108	35	35	20	65	82,9%	85,8%	93,4%	95,0%	89,7%
7109	35	35	20	70	83,2%	85,6%	93,6%	95,4%	90,3%
7110	35	35	20	75	83,4%	85,5%	93,8%	95,6%	90,9%
7111	35	35	20	80	83,6%	85,3%	94,0%	95,9%	91,4%
7112	35	35	25	20	76,4%	84,4%	90,2%	90,6%	81,8%
7113	35	35	25	25	76,7%	84,2%	90,5%	91,2%	82,8%
7114	35	35	25	30	77,0%	84,0%	90,8%	91,7%	83,7%
7115	35	35	25	35	77,3%	83,8%	91,1%	92,3%	84,7%
7116	35	35	25	40	77,6%	83,7%	91,4%	92,7%	85,5%
7117	35	35	25	45	77,9%	83,5%	91,6%	93,2%	86,4%
7118	35	35	25	50	78,2%	83,3%	91,9%	93,6%	87,1%
7119	35	35	25	55	78,5%	83,1%	92,1%	94,0%	87,9%
7120	35	35	25	60	78,8%	82,9%	92,4%	94,4%	88,6%
7121	35	35	25	65	79,1%	82,7%	92,6%	94,7%	89,3%
7122	35	35	25	70	79,4%	82,6%	92,9%	95,1%	89,9%
7123	35	35	25	75	79,7%	82,4%	93,1%	95,4%	90,5%
7124	35	35	25	80	80,0%	82,2%	93,3%	95,7%	91,1%
7125	35	35	30	20	71,6%	81,1%	89,1%	90,1%	81,2%
7126	35	35	30	25	72,0%	80,9%	89,4%	90,7%	82,2%
7127	35	35	30	30	72,4%	80,7%	89,7%	91,3%	83,2%

7128	35	35	30	35	72,7%	80,5%	90,0%	91,8%	84,1%
7129	35	35	30	40	73,1%	80,2%	90,3%	92,3%	85,0%
7130	35	35	30	45	73,4%	80,0%	90,6%	92,8%	85,9%
7131	35	35	30	50	73,7%	79,8%	90,9%	93,2%	86,7%
7132	35	35	30	55	74,1%	79,6%	91,2%	93,7%	87,4%
7133	35	35	30	60	74,4%	79,4%	91,5%	94,1%	88,2%
7134	35	35	30	65	74,8%	79,2%	91,7%	94,4%	88,9%
7135	35	35	30	70	75,1%	79,0%	92,0%	94,8%	89,5%
7136	35	35	30	75	75,4%	78,8%	92,2%	95,1%	90,2%
7137	35	35	30	80	75,7%	78,5%	92,5%	95,4%	90,7%
7138	35	35	35	20	66,4%	77,3%	87,8%	89,6%	80,5%
7139	35	35	35	25	66,8%	77,0%	88,2%	90,2%	81,6%
7140	35	35	35	30	67,2%	76,8%	88,5%	90,8%	82,6%
7141	35	35	35	35	67,5%	76,6%	88,9%	91,3%	83,6%
7142	35	35	35	40	67,9%	76,3%	89,2%	91,9%	84,5%
7143	35	35	35	45	68,3%	76,1%	89,5%	92,4%	85,4%
7144	35	35	35	50	68,7%	75,9%	89,8%	92,8%	86,2%
7145	35	35	35	55	69,1%	75,6%	90,2%	93,3%	87,0%
7146	35	35	35	60	69,4%	75,4%	90,4%	93,7%	87,7%
7147	35	35	35	65	69,8%	75,1%	90,7%	94,1%	88,5%
7148	35	35	35	70	70,2%	74,9%	91,0%	94,5%	89,1%
7149	35	35	35	75	70,6%	74,6%	91,3%	94,8%	89,8%
7150	35	35	35	80	70,9%	74,4%	91,6%	95,1%	90,4%
7151	35	35	40	20	60,7%	73,0%	86,4%	89,0%	79,9%
7152	35	35	40	25	61,1%	72,7%	86,8%	89,6%	81,0%
7153	35	35	40	30	61,5%	72,4%	87,2%	90,3%	82,0%
7154	35	35	40	35	61,9%	72,2%	87,6%	90,8%	83,0%
7155	35	35	40	40	62,3%	71,9%	88,0%	91,4%	83,9%
7156	35	35	40	45	62,7%	71,6%	88,3%	91,9%	84,8%
7157	35	35	40	50	63,2%	71,4%	88,7%	92,4%	85,7%
7158	35	35	40	55	63,6%	71,1%	89,0%	92,9%	86,5%
7159	35	35	40	60	64,0%	70,8%	89,3%	93,3%	87,3%
7160	35	35	40	65	64,4%	70,6%	89,6%	93,8%	88,0%

7161	35	35	40	70	64,8%	70,3%	89,9%	94,1%	88,7%
7162	35	35	40	75	65,2%	70,0%	90,3%	94,5%	89,4%
7163	35	35	40	80	65,6%	69,7%	90,5%	94,9%	90,0%
7164	35	35	45	20	54,6%	68,2%	84,9%	88,4%	79,2%
7165	35	35	45	25	55,1%	67,9%	85,3%	89,0%	80,3%
7166	35	35	45	30	55,5%	67,6%	85,8%	89,7%	81,4%
7167	35	35	45	35	55,9%	67,3%	86,2%	90,3%	82,4%
7168	35	35	45	40	56,4%	67,0%	86,6%	90,9%	83,4%
7169	35	35	45	45	56,8%	66,7%	87,0%	91,5%	84,3%
7170	35	35	45	50	57,2%	66,4%	87,3%	92,0%	85,2%
7171	35	35	45	55	57,7%	66,1%	87,7%	92,5%	86,0%
7172	35	35	45	60	58,1%	65,8%	88,1%	92,9%	86,8%
7173	35	35	45	65	58,5%	65,6%	88,4%	93,4%	87,6%
7174	35	35	45	70	59,0%	65,3%	88,8%	93,8%	88,3%
7175	35	35	45	75	59,4%	65,0%	89,1%	94,2%	89,0%
7176	35	35	45	80	59,8%	64,7%	89,4%	94,6%	89,7%
7177	35	35	50	20	48,5%	63,0%	83,3%	87,7%	78,5%
7178	35	35	50	25	48,9%	62,6%	83,7%	88,4%	79,7%
7179	35	35	50	30	49,4%	62,3%	84,2%	89,1%	80,7%
7180	35	35	50	35	49,8%	62,0%	84,6%	89,8%	81,8%
7181	35	35	50	40	50,2%	61,7%	85,1%	90,4%	82,8%
7182	35	35	50	45	50,7%	61,4%	85,5%	91,0%	83,7%
7183	35	35	50	50	51,1%	61,1%	85,9%	91,5%	84,6%
7184	35	35	50	55	51,6%	60,8%	86,3%	92,0%	85,5%
7185	35	35	50	60	52,0%	60,5%	86,7%	92,5%	86,3%
7186	35	35	50	65	52,4%	60,2%	87,1%	93,0%	87,1%
7187	35	35	50	70	52,9%	59,9%	87,5%	93,4%	87,9%
7188	35	35	50	75	53,3%	59,5%	87,8%	93,8%	88,6%
7189	35	35	50	80	53,8%	59,2%	88,2%	94,2%	89,3%
7190	35	35	55	20	42,4%	57,4%	81,4%	87,0%	77,8%
7191	35	35	55	25	42,8%	57,1%	82,0%	87,8%	79,0%
7192	35	35	55	30	43,2%	56,8%	82,5%	88,5%	80,1%
7193	35	35	55	35	43,7%	56,5%	82,9%	89,2%	81,2%

7194	35	35	55	40	44,1%	56,1%	83,4%	89,8%	82,2%
7195	35	35	55	45	44,5%	55,8%	83,9%	90,5%	83,2%
7196	35	35	55	50	45,0%	55,5%	84,3%	91,0%	84,1%
7197	35	35	55	55	45,4%	55,2%	84,8%	91,6%	85,0%
7198	35	35	55	60	45,8%	54,9%	85,2%	92,1%	85,9%
7199	35	35	55	65	46,3%	54,5%	85,6%	92,6%	86,7%
7200	35	35	55	70	46,7%	54,2%	86,0%	93,0%	87,4%
7201	35	35	55	75	47,2%	53,9%	86,4%	93,5%	88,2%
7202	35	35	55	80	47,6%	53,6%	86,8%	93,9%	88,9%
7203	35	35	60	20	36,5%	51,7%	79,5%	86,3%	77,1%
7204	35	35	60	25	36,9%	51,4%	80,0%	87,1%	78,3%
7205	35	35	60	30	37,3%	51,1%	80,6%	87,9%	79,4%
7206	35	35	60	35	37,7%	50,7%	81,1%	88,6%	80,5%
7207	35	35	60	40	38,1%	50,4%	81,6%	89,3%	81,6%
7208	35	35	60	45	38,5%	50,1%	82,1%	89,9%	82,6%
7209	35	35	60	50	39,0%	49,7%	82,6%	90,5%	83,5%
7210	35	35	60	55	39,4%	49,4%	83,1%	91,1%	84,5%
7211	35	35	60	60	39,8%	49,1%	83,6%	91,6%	85,3%
7212	35	35	60	65	40,2%	48,8%	84,0%	92,2%	86,2%
7213	35	35	60	70	40,7%	48,4%	84,5%	92,6%	87,0%
7214	35	35	60	75	41,1%	48,1%	84,9%	93,1%	87,7%
7215	35	35	60	80	41,5%	47,8%	85,4%	93,5%	88,4%
7216	35	35	65	20	31,0%	45,9%	77,4%	85,6%	76,4%
7217	35	35	65	25	31,3%	45,6%	78,0%	86,4%	77,6%
7218	35	35	65	30	31,7%	45,3%	78,6%	87,2%	78,7%
7219	35	35	65	35	32,1%	45,0%	79,1%	88,0%	79,9%
7220	35	35	65	40	32,5%	44,6%	79,7%	88,7%	80,9%
7221	35	35	65	45	32,9%	44,3%	80,2%	89,4%	82,0%
7222	35	35	65	50	33,3%	44,0%	80,8%	90,0%	83,0%
7223	35	35	65	55	33,7%	43,7%	81,3%	90,6%	83,9%
7224	35	35	65	60	34,1%	43,4%	81,8%	91,2%	84,8%
7225	35	35	65	65	34,5%	43,0%	82,3%	91,7%	85,7%
7226	35	35	65	70	34,9%	42,7%	82,8%	92,2%	86,5%

7227	35	35	65	75	35,3%	42,4%	83,3%	92,7%	87,3%
7228	35	35	65	80	35,7%	42,1%	83,7%	93,2%	88,0%
7229	35	35	70	20	26,0%	40,3%	75,2%	84,8%	75,6%
7230	35	35	70	25	26,3%	40,0%	75,8%	85,7%	76,8%
7231	35	35	70	30	26,6%	39,7%	76,4%	86,5%	78,0%
7232	35	35	70	35	27,0%	39,3%	77,0%	87,3%	79,2%
7233	35	35	70	40	27,3%	39,0%	77,6%	88,1%	80,3%
7234	35	35	70	45	27,7%	38,7%	78,2%	88,8%	81,4%
7235	35	35	70	50	28,0%	38,4%	78,8%	89,4%	82,4%
7236	35	35	70	55	28,4%	38,1%	79,3%	90,1%	83,4%
7237	35	35	70	60	28,8%	37,8%	79,9%	90,7%	84,3%
7238	35	35	70	65	29,1%	37,5%	80,4%	91,2%	85,2%
7239	35	35	70	70	29,5%	37,2%	80,9%	91,8%	86,0%
7240	35	35	70	75	29,9%	36,9%	81,5%	92,3%	86,8%
7241	35	35	70	80	30,2%	36,6%	82,0%	92,8%	87,6%
7242	35	35	75	20	21,5%	34,9%	72,8%	84,0%	74,8%
7243	35	35	75	25	21,8%	34,6%	73,4%	84,9%	76,1%
7244	35	35	75	30	22,1%	34,3%	74,1%	85,8%	77,3%
7245	35	35	75	35	22,4%	34,0%	74,7%	86,6%	78,5%
7246	35	35	75	40	22,7%	33,7%	75,4%	87,4%	79,6%
7247	35	35	75	45	23,0%	33,4%	76,0%	88,1%	80,7%
7248	35	35	75	50	23,3%	33,1%	76,6%	88,8%	81,8%
7249	35	35	75	55	23,7%	32,8%	77,2%	89,5%	82,8%
7250	35	35	75	60	24,0%	32,5%	77,8%	90,1%	83,7%
7251	35	35	75	65	24,3%	32,3%	78,4%	90,7%	84,6%
7252	35	35	75	70	24,6%	32,0%	79,0%	91,3%	85,5%
7253	35	35	75	75	24,9%	31,7%	79,5%	91,8%	86,3%
7254	35	35	75	80	25,3%	31,4%	80,1%	92,3%	87,1%
7255	35	35	80	20	17,6%	29,8%	70,2%	83,2%	74,1%
7256	35	35	80	25	17,9%	29,6%	70,9%	84,1%	75,3%
7257	35	35	80	30	18,1%	29,3%	71,6%	85,0%	76,6%
7258	35	35	80	35	18,4%	29,0%	72,3%	85,9%	77,8%
7259	35	35	80	40	18,7%	28,7%	73,0%	86,7%	79,0%

7260	35	35	80	45	18,9%	28,5%	73,7%	87,5%	80,1%
7261	35	35	80	50	19,2%	28,2%	74,3%	88,2%	81,1%
7262	35	35	80	55	19,5%	28,0%	74,9%	88,9%	82,2%
7263	35	35	80	60	19,8%	27,7%	75,6%	89,6%	83,2%
7264	35	35	80	65	20,0%	27,4%	76,2%	90,2%	84,1%
7265	35	35	80	70	20,3%	27,2%	76,8%	90,8%	85,0%
7266	35	35	80	75	20,6%	26,9%	77,4%	91,4%	85,8%
7267	35	35	80	80	20,9%	26,7%	78,0%	91,9%	86,7%
7268	35	40	20	20	81,9%	88,0%	91,0%	90,5%	81,0%
7269	35	40	20	25	82,2%	87,9%	91,3%	91,1%	82,0%
7270	35	40	20	30	82,4%	87,7%	91,6%	91,6%	83,0%
7271	35	40	20	35	82,7%	87,6%	91,8%	92,2%	83,9%
7272	35	40	20	40	82,9%	87,5%	92,1%	92,6%	84,8%
7273	35	40	20	45	83,2%	87,3%	92,3%	93,1%	85,7%
7274	35	40	20	50	83,4%	87,2%	92,6%	93,5%	86,5%
7275	35	40	20	55	83,7%	87,0%	92,8%	93,9%	87,3%
7276	35	40	20	60	83,9%	86,9%	93,0%	94,3%	88,0%
7277	35	40	20	65	84,1%	86,7%	93,2%	94,7%	88,7%
7278	35	40	20	70	84,4%	86,6%	93,4%	95,0%	89,4%
7279	35	40	20	75	84,6%	86,4%	93,6%	95,3%	90,0%
7280	35	40	20	80	84,8%	86,3%	93,8%	95,6%	90,6%
7281	35	40	25	20	77,9%	85,4%	90,0%	90,0%	80,3%
7282	35	40	25	25	78,2%	85,2%	90,3%	90,6%	81,4%
7283	35	40	25	30	78,5%	85,0%	90,6%	91,2%	82,4%
7284	35	40	25	35	78,8%	84,9%	90,8%	91,7%	83,4%
7285	35	40	25	40	79,1%	84,7%	91,1%	92,2%	84,3%
7286	35	40	25	45	79,4%	84,5%	91,4%	92,7%	85,2%
7287	35	40	25	50	79,7%	84,4%	91,7%	93,2%	86,0%
7288	35	40	25	55	80,0%	84,2%	91,9%	93,6%	86,8%
7289	35	40	25	60	80,3%	84,0%	92,2%	94,0%	87,6%
7290	35	40	25	65	80,6%	83,8%	92,4%	94,4%	88,3%
7291	35	40	25	70	80,8%	83,7%	92,6%	94,7%	89,0%
7292	35	40	25	75	81,1%	83,5%	92,9%	95,0%	89,7%

7293	35	40	25	80	81,4%	83,3%	93,1%	95,4%	90,3%
7294	35	40	30	20	73,4%	82,2%	88,8%	89,4%	79,7%
7295	35	40	30	25	73,8%	82,0%	89,1%	90,1%	80,8%
7296	35	40	30	30	74,1%	81,9%	89,4%	90,7%	81,8%
7297	35	40	30	35	74,4%	81,7%	89,8%	91,2%	82,8%
7298	35	40	30	40	74,8%	81,5%	90,1%	91,8%	83,7%
7299	35	40	30	45	75,1%	81,3%	90,4%	92,3%	84,7%
7300	35	40	30	50	75,4%	81,1%	90,7%	92,8%	85,5%
7301	35	40	30	55	75,7%	80,9%	90,9%	93,2%	86,4%
7302	35	40	30	60	76,1%	80,7%	91,2%	93,6%	87,1%
7303	35	40	30	65	76,4%	80,5%	91,5%	94,0%	87,9%
7304	35	40	30	70	76,7%	80,2%	91,7%	94,4%	88,6%
7305	35	40	30	75	77,0%	80,0%	92,0%	94,8%	89,3%
7306	35	40	30	80	77,3%	79,8%	92,2%	95,1%	89,9%
7307	35	40	35	20	68,3%	78,6%	87,5%	88,8%	79,0%
7308	35	40	35	25	68,7%	78,4%	87,9%	89,5%	80,1%
7309	35	40	35	30	69,1%	78,2%	88,2%	90,1%	81,2%
7310	35	40	35	35	69,5%	77,9%	88,6%	90,7%	82,2%
7311	35	40	35	40	69,8%	77,7%	88,9%	91,3%	83,2%
7312	35	40	35	45	70,2%	77,5%	89,2%	91,8%	84,1%
7313	35	40	35	50	70,6%	77,3%	89,6%	92,3%	85,0%
7314	35	40	35	55	70,9%	77,0%	89,9%	92,8%	85,9%
7315	35	40	35	60	71,3%	76,8%	90,2%	93,3%	86,7%
7316	35	40	35	65	71,7%	76,6%	90,5%	93,7%	87,4%
7317	35	40	35	70	72,0%	76,3%	90,8%	94,1%	88,2%
7318	35	40	35	75	72,4%	76,1%	91,0%	94,4%	88,9%
7319	35	40	35	80	72,7%	75,9%	91,3%	94,8%	89,5%
7320	35	40	40	20	62,8%	74,5%	86,1%	88,2%	78,3%
7321	35	40	40	25	63,2%	74,2%	86,5%	88,9%	79,4%
7322	35	40	40	30	63,6%	74,0%	86,9%	89,6%	80,5%
7323	35	40	40	35	64,0%	73,7%	87,2%	90,2%	81,6%
7324	35	40	40	40	64,4%	73,5%	87,6%	90,8%	82,6%
7325	35	40	40	45	64,8%	73,2%	88,0%	91,4%	83,6%

7326	35	40	40	50	65,2%	73,0%	88,3%	91,9%	84,5%
7327	35	40	40	55	65,6%	72,7%	88,7%	92,4%	85,4%
7328	35	40	40	60	66,0%	72,4%	89,0%	92,9%	86,2%
7329	35	40	40	65	66,4%	72,2%	89,3%	93,3%	87,0%
7330	35	40	40	70	66,8%	71,9%	89,7%	93,7%	87,7%
7331	35	40	40	75	67,2%	71,6%	90,0%	94,1%	88,5%
7332	35	40	40	80	67,6%	71,4%	90,3%	94,5%	89,1%
7333	35	40	45	20	56,8%	69,8%	84,5%	87,6%	77,6%
7334	35	40	45	25	57,3%	69,6%	84,9%	88,3%	78,8%
7335	35	40	45	30	57,7%	69,3%	85,4%	89,0%	79,9%
7336	35	40	45	35	58,1%	69,0%	85,8%	89,7%	81,0%
7337	35	40	45	40	58,6%	68,7%	86,2%	90,3%	82,0%
7338	35	40	45	45	59,0%	68,5%	86,6%	90,9%	83,0%
7339	35	40	45	50	59,4%	68,2%	87,0%	91,4%	83,9%
7340	35	40	45	55	59,8%	67,9%	87,4%	91,9%	84,8%
7341	35	40	45	60	60,3%	67,6%	87,7%	92,4%	85,7%
7342	35	40	45	65	60,7%	67,3%	88,1%	92,9%	86,5%
7343	35	40	45	70	61,1%	67,0%	88,4%	93,4%	87,3%
7344	35	40	45	75	61,5%	66,7%	88,8%	93,8%	88,0%
7345	35	40	45	80	61,9%	66,4%	89,1%	94,2%	88,7%
7346	35	40	50	20	50,7%	64,8%	82,8%	86,9%	76,9%
7347	35	40	50	25	51,1%	64,5%	83,3%	87,7%	78,1%
7348	35	40	50	30	51,6%	64,2%	83,7%	88,4%	79,2%
7349	35	40	50	35	52,0%	63,9%	84,2%	89,1%	80,3%
7350	35	40	50	40	52,5%	63,6%	84,7%	89,7%	81,4%
7351	35	40	50	45	52,9%	63,3%	85,1%	90,3%	82,4%
7352	35	40	50	50	53,3%	63,0%	85,5%	90,9%	83,4%
7353	35	40	50	55	53,8%	62,7%	85,9%	91,5%	84,3%
7354	35	40	50	60	54,2%	62,3%	86,3%	92,0%	85,2%
7355	35	40	50	65	54,7%	62,0%	86,7%	92,5%	86,0%
7356	35	40	50	70	55,1%	61,7%	87,1%	93,0%	86,8%
7357	35	40	50	75	55,5%	61,4%	87,5%	93,4%	87,6%
7358	35	40	50	80	56,0%	61,1%	87,9%	93,8%	88,3%

7359	35	40	55	20	44,5%	59,3%	81,0%	86,2%	76,1%
7360	35	40	55	25	45,0%	59,0%	81,5%	87,0%	77,3%
7361	35	40	55	30	45,4%	58,7%	82,0%	87,7%	78,5%
7362	35	40	55	35	45,9%	58,4%	82,5%	88,5%	79,7%
7363	35	40	55	40	46,3%	58,1%	83,0%	89,2%	80,7%
7364	35	40	55	45	46,7%	57,8%	83,4%	89,8%	81,8%
7365	35	40	55	50	47,2%	57,4%	83,9%	90,4%	82,8%
7366	35	40	55	55	47,6%	57,1%	84,4%	91,0%	83,7%
7367	35	40	55	60	48,1%	56,8%	84,8%	91,5%	84,6%
7368	35	40	55	65	48,5%	56,5%	85,2%	92,1%	85,5%
7369	35	40	55	70	48,9%	56,2%	85,7%	92,6%	86,3%
7370	35	40	55	75	49,4%	55,8%	86,1%	93,0%	87,1%
7371	35	40	55	80	49,8%	55,5%	86,5%	93,5%	87,9%
7372	35	40	60	20	38,6%	53,7%	79,0%	85,4%	75,4%
7373	35	40	60	25	39,0%	53,3%	79,5%	86,3%	76,6%
7374	35	40	60	30	39,4%	53,0%	80,1%	87,1%	77,8%
7375	35	40	60	35	39,8%	52,7%	80,6%	87,8%	79,0%
7376	35	40	60	40	40,2%	52,4%	81,1%	88,5%	80,1%
7377	35	40	60	45	40,7%	52,0%	81,7%	89,2%	81,2%
7378	35	40	60	50	41,1%	51,7%	82,2%	89,9%	82,2%
7379	35	40	60	55	41,5%	51,4%	82,7%	90,5%	83,2%
7380	35	40	60	60	42,0%	51,1%	83,1%	91,1%	84,1%
7381	35	40	60	65	42,4%	50,7%	83,6%	91,6%	85,0%
7382	35	40	60	70	42,8%	50,4%	84,1%	92,1%	85,9%
7383	35	40	60	75	43,2%	50,1%	84,5%	92,6%	86,7%
7384	35	40	60	80	43,7%	49,8%	85,0%	93,1%	87,4%
7385	35	40	65	20	32,9%	47,9%	76,8%	84,7%	74,6%
7386	35	40	65	25	33,3%	47,6%	77,4%	85,5%	75,9%
7387	35	40	65	30	33,7%	47,3%	78,0%	86,4%	77,1%
7388	35	40	65	35	34,1%	46,9%	78,6%	87,2%	78,3%
7389	35	40	65	40	34,5%	46,6%	79,2%	87,9%	79,4%
7390	35	40	65	45	34,9%	46,3%	79,7%	88,6%	80,5%
7391	35	40	65	50	35,3%	46,0%	80,3%	89,3%	81,6%

7392	35	40	65	55	35,7%	45,6%	80,8%	89,9%	82,6%
7393	35	40	65	60	36,1%	45,3%	81,3%	90,6%	83,5%
7394	35	40	65	65	36,5%	45,0%	81,8%	91,1%	84,5%
7395	35	40	65	70	36,9%	44,7%	82,3%	91,7%	85,3%
7396	35	40	65	75	37,3%	44,3%	82,8%	92,2%	86,2%
7397	35	40	65	80	37,7%	44,0%	83,3%	92,7%	87,0%
7398	35	40	70	20	27,7%	42,2%	74,6%	83,9%	73,8%
7399	35	40	70	25	28,0%	41,9%	75,2%	84,8%	75,1%
7400	35	40	70	30	28,4%	41,6%	75,8%	85,6%	76,4%
7401	35	40	70	35	28,8%	41,2%	76,4%	86,5%	77,6%
7402	35	40	70	40	29,1%	40,9%	77,0%	87,3%	78,7%
7403	35	40	70	45	29,5%	40,6%	77,6%	88,0%	79,9%
7404	35	40	70	50	29,9%	40,3%	78,2%	88,7%	80,9%
7405	35	40	70	55	30,2%	40,0%	78,8%	89,4%	82,0%
7406	35	40	70	60	30,6%	39,7%	79,4%	90,0%	83,0%
7407	35	40	70	65	31,0%	39,4%	79,9%	90,6%	83,9%
7408	35	40	70	70	31,4%	39,0%	80,4%	91,2%	84,8%
7409	35	40	70	75	31,7%	38,7%	81,0%	91,7%	85,7%
7410	35	40	70	80	32,1%	38,4%	81,5%	92,2%	86,5%
7411	35	40	75	20	23,0%	36,7%	72,1%	83,0%	73,0%
7412	35	40	75	25	23,3%	36,4%	72,8%	84,0%	74,3%
7413	35	40	75	30	23,7%	36,1%	73,5%	84,9%	75,6%
7414	35	40	75	35	24,0%	35,8%	74,1%	85,7%	76,9%
7415	35	40	75	40	24,3%	35,5%	74,8%	86,6%	78,0%
7416	35	40	75	45	24,6%	35,2%	75,4%	87,3%	79,2%
7417	35	40	75	50	25,0%	34,9%	76,0%	88,1%	80,3%
7418	35	40	75	55	25,3%	34,6%	76,6%	88,8%	81,4%
7419	35	40	75	60	25,6%	34,3%	77,2%	89,5%	82,4%
7420	35	40	75	65	26,0%	34,0%	77,8%	90,1%	83,4%
7421	35	40	75	70	26,3%	33,7%	78,4%	90,7%	84,3%
7422	35	40	75	75	26,6%	33,4%	79,0%	91,3%	85,2%
7423	35	40	75	80	27,0%	33,1%	79,5%	91,8%	86,0%
7424	35	40	80	20	18,9%	31,5%	69,6%	82,1%	72,2%

7425	35	40	80	25	19,2%	31,2%	70,3%	83,1%	73,5%
7426	35	40	80	30	19,5%	30,9%	71,0%	84,1%	74,8%
7427	35	40	80	35	19,8%	30,7%	71,7%	85,0%	76,1%
7428	35	40	80	40	20,1%	30,4%	72,4%	85,8%	77,3%
7429	35	40	80	45	20,3%	30,1%	73,0%	86,7%	78,5%
7430	35	40	80	50	20,6%	29,8%	73,7%	87,4%	79,6%
7431	35	40	80	55	20,9%	29,6%	74,3%	88,2%	80,7%
7432	35	40	80	60	21,2%	29,3%	75,0%	88,9%	81,8%
7433	35	40	80	65	21,5%	29,0%	75,6%	89,5%	82,8%
7434	35	40	80	70	21,8%	28,8%	76,2%	90,2%	83,7%
7435	35	40	80	75	22,1%	28,5%	76,9%	90,8%	84,6%
7436	35	40	80	80	22,4%	28,2%	77,5%	91,3%	85,5%
7437	35	45	20	20	83,2%	88,8%	90,8%	89,9%	79,5%
7438	35	45	20	25	83,4%	88,7%	91,0%	90,5%	80,6%
7439	35	45	20	30	83,7%	88,6%	91,3%	91,1%	81,6%
7440	35	45	20	35	83,9%	88,4%	91,6%	91,6%	82,6%
7441	35	45	20	40	84,1%	88,3%	91,8%	92,1%	83,6%
7442	35	45	20	45	84,4%	88,2%	92,1%	92,6%	84,5%
7443	35	45	20	50	84,6%	88,0%	92,3%	93,1%	85,4%
7444	35	45	20	55	84,8%	87,9%	92,6%	93,5%	86,2%
7445	35	45	20	60	85,1%	87,7%	92,8%	93,9%	87,0%
7446	35	45	20	65	85,3%	87,6%	93,0%	94,3%	87,8%
7447	35	45	20	70	85,5%	87,5%	93,2%	94,6%	88,5%
7448	35	45	20	75	85,7%	87,3%	93,5%	95,0%	89,1%
7449	35	45	20	80	85,9%	87,2%	93,7%	95,3%	89,8%
7450	35	45	25	20	79,4%	86,3%	89,7%	89,3%	78,8%
7451	35	45	25	25	79,7%	86,2%	90,0%	89,9%	79,9%
7452	35	45	25	30	80,0%	86,0%	90,3%	90,6%	81,0%
7453	35	45	25	35	80,3%	85,9%	90,6%	91,1%	82,0%
7454	35	45	25	40	80,6%	85,7%	90,9%	91,7%	83,0%
7455	35	45	25	45	80,8%	85,5%	91,1%	92,2%	83,9%
7456	35	45	25	50	81,1%	85,4%	91,4%	92,7%	84,8%
7457	35	45	25	55	81,4%	85,2%	91,7%	93,1%	85,7%

7458	35	45	25	60	81,6%	85,0%	91,9%	93,5%	86,5%
7459	35	45	25	65	81,9%	84,9%	92,2%	94,0%	87,3%
7460	35	45	25	70	82,2%	84,7%	92,4%	94,3%	88,0%
7461	35	45	25	75	82,4%	84,5%	92,7%	94,7%	88,7%
7462	35	45	25	80	82,7%	84,4%	92,9%	95,0%	89,4%
7463	35	45	30	20	75,1%	83,4%	88,5%	88,7%	78,1%
7464	35	45	30	25	75,4%	83,2%	88,8%	89,4%	79,2%
7465	35	45	30	30	75,8%	83,0%	89,1%	90,0%	80,3%
7466	35	45	30	35	76,1%	82,8%	89,5%	90,6%	81,4%
7467	35	45	30	40	76,4%	82,6%	89,8%	91,2%	82,4%
7468	35	45	30	45	76,7%	82,4%	90,1%	91,7%	83,4%
7469	35	45	30	50	77,0%	82,2%	90,4%	92,2%	84,3%
7470	35	45	30	55	77,3%	82,1%	90,7%	92,7%	85,2%
7471	35	45	30	60	77,6%	81,9%	91,0%	93,2%	86,0%
7472	35	45	30	65	78,0%	81,7%	91,2%	93,6%	86,8%
7473	35	45	30	70	78,3%	81,5%	91,5%	94,0%	87,6%
7474	35	45	30	75	78,6%	81,3%	91,8%	94,4%	88,3%
7475	35	45	30	80	78,8%	81,1%	92,0%	94,7%	89,0%
7476	35	45	35	20	70,2%	79,9%	87,1%	88,1%	77,4%
7477	35	45	35	25	70,6%	79,7%	87,5%	88,8%	78,5%
7478	35	45	35	30	70,9%	79,5%	87,9%	89,5%	79,7%
7479	35	45	35	35	71,3%	79,3%	88,2%	90,1%	80,8%
7480	35	45	35	40	71,7%	79,1%	88,6%	90,7%	81,8%
7481	35	45	35	45	72,0%	78,8%	88,9%	91,3%	82,8%
7482	35	45	35	50	72,4%	78,6%	89,2%	91,8%	83,8%
7483	35	45	35	55	72,7%	78,4%	89,6%	92,3%	84,7%
7484	35	45	35	60	73,1%	78,2%	89,9%	92,8%	85,5%
7485	35	45	35	65	73,4%	78,0%	90,2%	93,2%	86,4%
7486	35	45	35	70	73,8%	77,7%	90,5%	93,6%	87,1%
7487	35	45	35	75	74,1%	77,5%	90,8%	94,0%	87,9%
7488	35	45	35	80	74,4%	77,3%	91,1%	94,4%	88,6%
7489	35	45	40	20	64,8%	75,9%	85,7%	87,4%	76,6%
7490	35	45	40	25	65,2%	75,7%	86,1%	88,2%	77,8%

7491	35	45	40	30	65,6%	75,5%	86,5%	88,9%	79,0%
7492	35	45	40	35	66,0%	75,2%	86,9%	89,5%	80,1%
7493	35	45	40	40	66,4%	75,0%	87,3%	90,2%	81,2%
7494	35	45	40	45	66,8%	74,7%	87,6%	90,8%	82,2%
7495	35	45	40	50	67,2%	74,5%	88,0%	91,3%	83,2%
7496	35	45	40	55	67,6%	74,2%	88,4%	91,8%	84,1%
7497	35	45	40	60	68,0%	74,0%	88,7%	92,4%	85,0%
7498	35	45	40	65	68,3%	73,7%	89,0%	92,8%	85,9%
7499	35	45	40	70	68,7%	73,5%	89,4%	93,3%	86,7%
7500	35	45	40	75	69,1%	73,2%	89,7%	93,7%	87,4%
7501	35	45	40	80	69,5%	73,0%	90,0%	94,1%	88,2%
7502	35	45	45	20	59,0%	71,5%	84,1%	86,7%	75,9%
7503	35	45	45	25	59,4%	71,2%	84,5%	87,5%	77,1%
7504	35	45	45	30	59,8%	70,9%	85,0%	88,2%	78,3%
7505	35	45	45	35	60,3%	70,7%	85,4%	88,9%	79,4%
7506	35	45	45	40	60,7%	70,4%	85,8%	89,6%	80,5%
7507	35	45	45	45	61,1%	70,1%	86,2%	90,2%	81,6%
7508	35	45	45	50	61,5%	69,9%	86,6%	90,8%	82,6%
7509	35	45	45	55	61,9%	69,6%	87,0%	91,4%	83,6%
7510	35	45	45	60	62,4%	69,3%	87,4%	91,9%	84,5%
7511	35	45	45	65	62,8%	69,0%	87,8%	92,4%	85,4%
7512	35	45	45	70	63,2%	68,7%	88,1%	92,9%	86,2%
7513	35	45	45	75	63,6%	68,5%	88,5%	93,3%	87,0%
7514	35	45	45	80	64,0%	68,2%	88,8%	93,7%	87,7%
7515	35	45	50	20	52,9%	66,5%	82,3%	86,0%	75,1%
7516	35	45	50	25	53,4%	66,3%	82,8%	86,8%	76,4%
7517	35	45	50	30	53,8%	66,0%	83,3%	87,6%	77,6%
7518	35	45	50	35	54,2%	65,7%	83,8%	88,3%	78,8%
7519	35	45	50	40	54,7%	65,4%	84,2%	89,0%	79,9%
7520	35	45	50	45	55,1%	65,1%	84,7%	89,7%	81,0%
7521	35	45	50	50	55,5%	64,8%	85,1%	90,3%	82,0%
7522	35	45	50	55	56,0%	64,5%	85,5%	90,9%	83,0%
7523	35	45	50	60	56,4%	64,2%	86,0%	91,4%	83,9%

7524	35	45	50	65	56,8%	63,9%	86,4%	92,0%	84,8%
7525	35	45	50	70	57,3%	63,6%	86,8%	92,5%	85,7%
7526	35	45	50	75	57,7%	63,3%	87,1%	92,9%	86,5%
7527	35	45	50	80	58,1%	63,0%	87,5%	93,4%	87,3%
7528	35	45	55	20	46,7%	61,2%	80,5%	85,3%	74,3%
7529	35	45	55	25	47,2%	60,9%	81,0%	86,1%	75,6%
7530	35	45	55	30	47,6%	60,6%	81,5%	86,9%	76,9%
7531	35	45	55	35	48,1%	60,3%	82,0%	87,7%	78,1%
7532	35	45	55	40	48,5%	60,0%	82,5%	88,4%	79,2%
7533	35	45	55	45	48,9%	59,7%	83,0%	89,1%	80,3%
7534	35	45	55	50	49,4%	59,3%	83,5%	89,8%	81,4%
7535	35	45	55	55	49,8%	59,0%	83,9%	90,4%	82,4%
7536	35	45	55	60	50,3%	58,7%	84,4%	91,0%	83,4%
7537	35	45	55	65	50,7%	58,4%	84,8%	91,5%	84,3%
7538	35	45	55	70	51,2%	58,1%	85,3%	92,0%	85,2%
7539	35	45	55	75	51,6%	57,8%	85,7%	92,5%	86,0%
7540	35	45	55	80	52,0%	57,4%	86,1%	93,0%	86,8%
7541	35	45	60	20	40,7%	55,6%	78,4%	84,5%	73,5%
7542	35	45	60	25	41,1%	55,3%	79,0%	85,4%	74,9%
7543	35	45	60	30	41,5%	55,0%	79,6%	86,2%	76,1%
7544	35	45	60	35	42,0%	54,7%	80,1%	87,0%	77,3%
7545	35	45	60	40	42,4%	54,3%	80,6%	87,8%	78,5%
7546	35	45	60	45	42,8%	54,0%	81,2%	88,5%	79,7%
7547	35	45	60	50	43,3%	53,7%	81,7%	89,2%	80,7%
7548	35	45	60	55	43,7%	53,4%	82,2%	89,8%	81,8%
7549	35	45	60	60	44,1%	53,0%	82,7%	90,4%	82,8%
7550	35	45	60	65	44,6%	52,7%	83,2%	91,0%	83,7%
7551	35	45	60	70	45,0%	52,4%	83,6%	91,6%	84,7%
7552	35	45	60	75	45,4%	52,0%	84,1%	92,1%	85,5%
7553	35	45	60	80	45,9%	51,7%	84,5%	92,6%	86,3%
7554	35	45	65	20	34,9%	49,9%	76,3%	83,7%	72,7%
7555	35	45	65	25	35,3%	49,5%	76,9%	84,6%	74,1%
7556	35	45	65	30	35,7%	49,2%	77,5%	85,5%	75,4%

7557	35	45	65	35	36,1%	48,9%	78,1%	86,3%	76,6%
7558	35	45	65	40	36,5%	48,6%	78,6%	87,1%	77,8%
7559	35	45	65	45	36,9%	48,2%	79,2%	87,9%	79,0%
7560	35	45	65	50	37,3%	47,9%	79,8%	88,6%	80,1%
7561	35	45	65	55	37,7%	47,6%	80,3%	89,3%	81,2%
7562	35	45	65	60	38,2%	47,3%	80,8%	89,9%	82,2%
7563	35	45	65	65	38,6%	46,9%	81,4%	90,5%	83,2%
7564	35	45	65	70	39,0%	46,6%	81,9%	91,1%	84,1%
7565	35	45	65	75	39,4%	46,3%	82,4%	91,6%	85,0%
7566	35	45	65	80	39,8%	46,0%	82,8%	92,1%	85,9%
7567	35	45	70	20	29,5%	44,1%	73,9%	82,8%	71,9%
7568	35	45	70	25	29,9%	43,8%	74,6%	83,8%	73,3%
7569	35	45	70	30	30,2%	43,5%	75,2%	84,7%	74,6%
7570	35	45	70	35	30,6%	43,2%	75,9%	85,6%	75,9%
7571	35	45	70	40	31,0%	42,8%	76,5%	86,4%	77,1%
7572	35	45	70	45	31,4%	42,5%	77,1%	87,2%	78,3%
7573	35	45	70	50	31,8%	42,2%	77,7%	87,9%	79,4%
7574	35	45	70	55	32,1%	41,9%	78,3%	88,7%	80,5%
7575	35	45	70	60	32,5%	41,6%	78,8%	89,3%	81,6%
7576	35	45	70	65	32,9%	41,2%	79,4%	90,0%	82,6%
7577	35	45	70	70	33,3%	40,9%	79,9%	90,6%	83,6%
7578	35	45	70	75	33,7%	40,6%	80,5%	91,2%	84,5%
7579	35	45	70	80	34,1%	40,3%	81,0%	91,7%	85,3%
7580	35	45	75	20	24,6%	38,5%	71,5%	81,9%	71,1%
7581	35	45	75	25	25,0%	38,2%	72,2%	82,9%	72,5%
7582	35	45	75	30	25,3%	37,9%	72,8%	83,9%	73,8%
7583	35	45	75	35	25,6%	37,6%	73,5%	84,8%	75,1%
7584	35	45	75	40	26,0%	37,3%	74,2%	85,7%	76,4%
7585	35	45	75	45	26,3%	37,0%	74,8%	86,5%	77,6%
7586	35	45	75	50	26,7%	36,7%	75,4%	87,3%	78,8%
7587	35	45	75	55	27,0%	36,4%	76,1%	88,0%	79,9%
7588	35	45	75	60	27,4%	36,1%	76,7%	88,7%	81,0%
7589	35	45	75	65	27,7%	35,8%	77,3%	89,4%	82,0%

7590	35	45	75	70	28,1%	35,5%	77,9%	90,0%	83,0%
7591	35	45	75	75	28,4%	35,2%	78,5%	90,6%	83,9%
7592	35	45	75	80	28,8%	34,9%	79,0%	91,2%	84,8%
7593	35	45	80	20	20,3%	33,2%	68,9%	81,0%	70,2%
7594	35	45	80	25	20,6%	32,9%	69,6%	82,1%	71,6%
7595	35	45	80	30	20,9%	32,7%	70,3%	83,1%	73,0%
7596	35	45	80	35	21,2%	32,4%	71,0%	84,0%	74,3%
7597	35	45	80	40	21,5%	32,1%	71,7%	84,9%	75,6%
7598	35	45	80	45	21,8%	31,8%	72,4%	85,8%	76,9%
7599	35	45	80	50	22,1%	31,5%	73,1%	86,6%	78,1%
7600	35	45	80	55	22,4%	31,2%	73,7%	87,4%	79,2%
7601	35	45	80	60	22,7%	30,9%	74,4%	88,1%	80,3%
7602	35	45	80	65	23,0%	30,7%	75,0%	88,8%	81,4%
7603	35	45	80	70	23,4%	30,4%	75,7%	89,5%	82,4%
7604	35	45	80	75	23,7%	30,1%	76,3%	90,1%	83,4%
7605	35	45	80	80	24,0%	29,8%	76,9%	90,7%	84,3%
7606	35	50	20	20	84,4%	89,6%	90,5%	89,2%	77,9%
7607	35	50	20	25	84,6%	89,5%	90,8%	89,8%	79,0%
7608	35	50	20	30	84,8%	89,3%	91,1%	90,4%	80,1%
7609	35	50	20	35	85,1%	89,2%	91,3%	91,0%	81,2%
7610	35	50	20	40	85,3%	89,1%	91,6%	91,6%	82,2%
7611	35	50	20	45	85,5%	89,0%	91,9%	92,1%	83,2%
7612	35	50	20	50	85,7%	88,8%	92,1%	92,6%	84,1%
7613	35	50	20	55	85,9%	88,7%	92,4%	93,0%	85,0%
7614	35	50	20	60	86,2%	88,6%	92,6%	93,5%	85,9%
7615	35	50	20	65	86,4%	88,4%	92,8%	93,9%	86,7%
7616	35	50	20	70	86,6%	88,3%	93,0%	94,3%	87,5%
7617	35	50	20	75	86,8%	88,2%	93,3%	94,6%	88,2%
7618	35	50	20	80	87,0%	88,0%	93,5%	95,0%	88,9%
7619	35	50	25	20	80,8%	87,2%	89,4%	88,6%	77,1%
7620	35	50	25	25	81,1%	87,1%	89,7%	89,3%	78,3%
7621	35	50	25	30	81,4%	86,9%	90,0%	89,9%	79,5%
7622	35	50	25	35	81,7%	86,8%	90,3%	90,5%	80,6%

7623	35	50	25	40	81,9%	86,6%	90,6%	91,1%	81,6%
7624	35	50	25	45	82,2%	86,5%	90,9%	91,6%	82,6%
7625	35	50	25	50	82,4%	86,3%	91,2%	92,1%	83,6%
7626	35	50	25	55	82,7%	86,2%	91,4%	92,6%	84,5%
7627	35	50	25	60	82,9%	86,0%	91,7%	93,1%	85,4%
7628	35	50	25	65	83,2%	85,9%	91,9%	93,5%	86,2%
7629	35	50	25	70	83,4%	85,7%	92,2%	93,9%	87,0%
7630	35	50	25	75	83,7%	85,5%	92,4%	94,3%	87,8%
7631	35	50	25	80	83,9%	85,4%	92,7%	94,7%	88,5%
7632	35	50	30	20	76,7%	84,4%	88,1%	87,9%	76,4%
7633	35	50	30	25	77,0%	84,3%	88,5%	88,7%	77,6%
7634	35	50	30	30	77,4%	84,1%	88,8%	89,3%	78,8%
7635	35	50	30	35	77,7%	83,9%	89,2%	90,0%	79,9%
7636	35	50	30	40	78,0%	83,7%	89,5%	90,6%	81,0%
7637	35	50	30	45	78,3%	83,5%	89,8%	91,1%	82,0%
7638	35	50	30	50	78,6%	83,4%	90,1%	91,7%	83,0%
7639	35	50	30	55	78,9%	83,2%	90,4%	92,2%	83,9%
7640	35	50	30	60	79,2%	83,0%	90,7%	92,7%	84,8%
7641	35	50	30	65	79,4%	82,8%	91,0%	93,1%	85,7%
7642	35	50	30	70	79,7%	82,6%	91,3%	93,6%	86,5%
7643	35	50	30	75	80,0%	82,4%	91,5%	94,0%	87,3%
7644	35	50	30	80	80,3%	82,2%	91,8%	94,3%	88,0%
7645	35	50	35	20	72,0%	81,1%	86,8%	87,3%	75,6%
7646	35	50	35	25	72,4%	80,9%	87,2%	88,0%	76,9%
7647	35	50	35	30	72,7%	80,7%	87,5%	88,7%	78,1%
7648	35	50	35	35	73,1%	80,5%	87,9%	89,4%	79,2%
7649	35	50	35	40	73,4%	80,3%	88,3%	90,0%	80,3%
7650	35	50	35	45	73,8%	80,1%	88,6%	90,6%	81,4%
7651	35	50	35	50	74,1%	79,9%	88,9%	91,2%	82,4%
7652	35	50	35	55	74,5%	79,7%	89,3%	91,8%	83,4%
7653	35	50	35	60	74,8%	79,5%	89,6%	92,3%	84,3%
7654	35	50	35	65	75,1%	79,3%	89,9%	92,7%	85,2%
7655	35	50	35	70	75,4%	79,1%	90,2%	93,2%	86,0%

7656	35	50	35	75	75,8%	78,8%	90,5%	93,6%	86,8%
7657	35	50	35	80	76,1%	78,6%	90,8%	94,0%	87,6%
7658	35	50	40	20	66,8%	77,4%	85,3%	86,6%	74,9%
7659	35	50	40	25	67,2%	77,1%	85,7%	87,4%	76,1%
7660	35	50	40	30	67,6%	76,9%	86,1%	88,1%	77,4%
7661	35	50	40	35	68,0%	76,7%	86,5%	88,8%	78,5%
7662	35	50	40	40	68,3%	76,4%	86,9%	89,5%	79,7%
7663	35	50	40	45	68,7%	76,2%	87,3%	90,1%	80,8%
7664	35	50	40	50	69,1%	76,0%	87,7%	90,7%	81,8%
7665	35	50	40	55	69,5%	75,7%	88,0%	91,3%	82,8%
7666	35	50	40	60	69,9%	75,5%	88,4%	91,8%	83,8%
7667	35	50	40	65	70,2%	75,2%	88,7%	92,3%	84,7%
7668	35	50	40	70	70,6%	75,0%	89,1%	92,8%	85,5%
7669	35	50	40	75	71,0%	74,7%	89,4%	93,2%	86,4%
7670	35	50	40	80	71,3%	74,5%	89,7%	93,7%	87,1%
7671	35	50	45	20	61,1%	73,1%	83,7%	85,9%	74,1%
7672	35	50	45	25	61,5%	72,8%	84,1%	86,7%	75,4%
7673	35	50	45	30	62,0%	72,5%	84,6%	87,5%	76,6%
7674	35	50	45	35	62,4%	72,3%	85,0%	88,2%	77,8%
7675	35	50	45	40	62,8%	72,0%	85,4%	88,9%	79,0%
7676	35	50	45	45	63,2%	71,8%	85,8%	89,6%	80,1%
7677	35	50	45	50	63,6%	71,5%	86,3%	90,2%	81,2%
7678	35	50	45	55	64,0%	71,2%	86,6%	90,8%	82,2%
7679	35	50	45	60	64,4%	70,9%	87,0%	91,3%	83,2%
7680	35	50	45	65	64,8%	70,7%	87,4%	91,9%	84,1%
7681	35	50	45	70	65,2%	70,4%	87,8%	92,4%	85,0%
7682	35	50	45	75	65,6%	70,1%	88,1%	92,8%	85,9%
7683	35	50	45	80	66,0%	69,9%	88,5%	93,3%	86,7%
7684	35	50	50	20	55,1%	68,3%	81,9%	85,1%	73,3%
7685	35	50	50	25	55,6%	68,0%	82,4%	86,0%	74,6%
7686	35	50	50	30	56,0%	67,7%	82,9%	86,8%	75,9%
7687	35	50	50	35	56,4%	67,4%	83,3%	87,5%	77,1%
7688	35	50	50	40	56,9%	67,1%	83,8%	88,3%	78,3%

7689	35	50	50	45	57,3%	66,8%	84,3%	89,0%	79,4%
7690	35	50	50	50	57,7%	66,6%	84,7%	89,6%	80,5%
7691	35	50	50	55	58,1%	66,3%	85,1%	90,3%	81,6%
7692	35	50	50	60	58,6%	66,0%	85,6%	90,8%	82,6%
7693	35	50	50	65	59,0%	65,7%	86,0%	91,4%	83,6%
7694	35	50	50	70	59,4%	65,4%	86,4%	91,9%	84,5%
7695	35	50	50	75	59,9%	65,1%	86,8%	92,4%	85,4%
7696	35	50	50	80	60,3%	64,8%	87,2%	92,9%	86,2%
7697	35	50	55	20	49,0%	63,1%	80,0%	84,3%	72,5%
7698	35	50	55	25	49,4%	62,8%	80,5%	85,2%	73,8%
7699	35	50	55	30	49,8%	62,5%	81,0%	86,1%	75,1%
7700	35	50	55	35	50,3%	62,2%	81,5%	86,9%	76,4%
7701	35	50	55	40	50,7%	61,9%	82,1%	87,6%	77,6%
7702	35	50	55	45	51,2%	61,5%	82,5%	88,4%	78,8%
7703	35	50	55	50	51,6%	61,2%	83,0%	89,1%	79,9%
7704	35	50	55	55	52,0%	60,9%	83,5%	89,7%	81,0%
7705	35	50	55	60	52,5%	60,6%	84,0%	90,3%	82,0%
7706	35	50	55	65	52,9%	60,3%	84,4%	90,9%	83,0%
7707	35	50	55	70	53,4%	60,0%	84,9%	91,5%	83,9%
7708	35	50	55	75	53,8%	59,7%	85,3%	92,0%	84,8%
7709	35	50	55	80	54,2%	59,4%	85,7%	92,5%	85,7%
7710	35	50	60	20	42,8%	57,6%	77,9%	83,5%	71,7%
7711	35	50	60	25	43,3%	57,2%	78,5%	84,4%	73,0%
7712	35	50	60	30	43,7%	56,9%	79,0%	85,3%	74,3%
7713	35	50	60	35	44,1%	56,6%	79,6%	86,2%	75,6%
7714	35	50	60	40	44,6%	56,3%	80,1%	87,0%	76,9%
7715	35	50	60	45	45,0%	56,0%	80,7%	87,7%	78,1%
7716	35	50	60	50	45,4%	55,6%	81,2%	88,4%	79,2%
7717	35	50	60	55	45,9%	55,3%	81,7%	89,1%	80,3%
7718	35	50	60	60	46,3%	55,0%	82,2%	89,8%	81,4%
7719	35	50	60	65	46,8%	54,7%	82,7%	90,4%	82,4%
7720	35	50	60	70	47,2%	54,3%	83,2%	91,0%	83,4%
7721	35	50	60	75	47,6%	54,0%	83,7%	91,5%	84,3%

7722	35	50	60	80	48,1%	53,7%	84,1%	92,0%	85,2%
7723	35	50	65	20	36,9%	51,8%	75,7%	82,6%	70,8%
7724	35	50	65	25	37,3%	51,5%	76,3%	83,6%	72,2%
7725	35	50	65	30	37,8%	51,2%	76,9%	84,5%	73,6%
7726	35	50	65	35	38,2%	50,9%	77,5%	85,4%	74,9%
7727	35	50	65	40	38,6%	50,5%	78,1%	86,2%	76,1%
7728	35	50	65	45	39,0%	50,2%	78,7%	87,0%	77,4%
7729	35	50	65	50	39,4%	49,9%	79,2%	87,8%	78,5%
7730	35	50	65	55	39,8%	49,6%	79,8%	88,5%	79,7%
7731	35	50	65	60	40,3%	49,2%	80,3%	89,2%	80,8%
7732	35	50	65	65	40,7%	48,9%	80,9%	89,9%	81,8%
7733	35	50	65	70	41,1%	48,6%	81,4%	90,5%	82,8%
7734	35	50	65	75	41,5%	48,2%	81,9%	91,0%	83,7%
7735	35	50	65	80	42,0%	47,9%	82,4%	91,6%	84,7%
7736	35	50	70	20	31,4%	46,1%	73,3%	81,7%	69,9%
7737	35	50	70	25	31,8%	45,8%	74,0%	82,8%	71,4%
7738	35	50	70	30	32,1%	45,4%	74,6%	83,7%	72,7%
7739	35	50	70	35	32,5%	45,1%	75,3%	84,6%	74,1%
7740	35	50	70	40	32,9%	44,8%	75,9%	85,5%	75,4%
7741	35	50	70	45	33,3%	44,5%	76,5%	86,3%	76,6%
7742	35	50	70	50	33,7%	44,1%	77,1%	87,1%	77,8%
7743	35	50	70	55	34,1%	43,8%	77,7%	87,9%	79,0%
7744	35	50	70	60	34,5%	43,5%	78,3%	88,6%	80,1%
7745	35	50	70	65	34,9%	43,2%	78,9%	89,3%	81,2%
7746	35	50	70	70	35,3%	42,8%	79,4%	89,9%	82,2%
7747	35	50	70	75	35,7%	42,5%	80,0%	90,5%	83,2%
7748	35	50	70	80	36,1%	42,2%	80,5%	91,1%	84,1%
7749	35	50	75	20	26,3%	40,4%	70,8%	80,8%	69,1%
7750	35	50	75	25	26,7%	40,1%	71,5%	81,9%	70,5%
7751	35	50	75	30	27,0%	39,8%	72,2%	82,9%	71,9%
7752	35	50	75	35	27,4%	39,5%	72,9%	83,8%	73,3%
7753	35	50	75	40	27,7%	39,2%	73,6%	84,7%	74,6%
7754	35	50	75	45	28,1%	38,9%	74,2%	85,6%	75,9%

7755	35	50	75	50	28,4%	38,5%	74,9%	86,4%	77,1%
7756	35	50	75	55	28,8%	38,2%	75,5%	87,2%	78,3%
7757	35	50	75	60	29,2%	37,9%	76,1%	88,0%	79,4%
7758	35	50	75	65	29,5%	37,6%	76,7%	88,7%	80,5%
7759	35	50	75	70	29,9%	37,3%	77,3%	89,4%	81,6%
7760	35	50	75	75	30,3%	37,0%	77,9%	90,0%	82,6%
7761	35	50	75	80	30,6%	36,7%	78,5%	90,6%	83,6%
7762	35	50	80	20	21,8%	35,0%	68,2%	79,9%	68,2%
7763	35	50	80	25	22,1%	34,7%	68,9%	80,9%	69,7%
7764	35	50	80	30	22,4%	34,4%	69,7%	82,0%	71,1%
7765	35	50	80	35	22,7%	34,1%	70,4%	83,0%	72,5%
7766	35	50	80	40	23,1%	33,8%	71,1%	83,9%	73,8%
7767	35	50	80	45	23,4%	33,5%	71,8%	84,8%	75,1%
7768	35	50	80	50	23,7%	33,2%	72,4%	85,7%	76,4%
7769	35	50	80	55	24,0%	32,9%	73,1%	86,5%	77,6%
7770	35	50	80	60	24,3%	32,7%	73,8%	87,3%	78,8%
7771	35	50	80	65	24,7%	32,4%	74,4%	88,1%	79,9%
7772	35	50	80	70	25,0%	32,1%	75,1%	88,8%	81,0%
7773	35	50	80	75	25,3%	31,8%	75,7%	89,4%	82,0%
7774	35	50	80	80	25,6%	31,5%	76,3%	90,1%	83,0%
7775	35	55	20	20	85,5%	90,3%	90,2%	88,4%	76,2%
7776	35	55	20	25	85,7%	90,2%	90,5%	89,1%	77,4%
7777	35	55	20	30	85,9%	90,1%	90,8%	89,8%	78,6%
7778	35	55	20	35	86,2%	90,0%	91,1%	90,4%	79,7%
7779	35	55	20	40	86,4%	89,8%	91,4%	91,0%	80,8%
7780	35	55	20	45	86,6%	89,7%	91,6%	91,5%	81,8%
7781	35	55	20	50	86,8%	89,6%	91,9%	92,0%	82,8%
7782	35	55	20	55	87,0%	89,5%	92,1%	92,5%	83,8%
7783	35	55	20	60	87,2%	89,3%	92,4%	93,0%	84,7%
7784	35	55	20	65	87,4%	89,2%	92,6%	93,4%	85,5%
7785	35	55	20	70	87,6%	89,1%	92,8%	93,8%	86,4%
7786	35	55	20	75	87,8%	89,0%	93,1%	94,2%	87,2%
7787	35	55	20	80	87,9%	88,8%	93,3%	94,6%	87,9%

7788	35	55	25	20	82,2%	88,1%	89,1%	87,8%	75,4%
7789	35	55	25	25	82,4%	87,9%	89,4%	88,5%	76,7%
7790	35	55	25	30	82,7%	87,8%	89,7%	89,2%	77,9%
7791	35	55	25	35	82,9%	87,7%	90,0%	89,9%	79,0%
7792	35	55	25	40	83,2%	87,5%	90,3%	90,5%	80,1%
7793	35	55	25	45	83,4%	87,4%	90,6%	91,0%	81,2%
7794	35	55	25	50	83,7%	87,2%	90,9%	91,6%	82,2%
7795	35	55	25	55	83,9%	87,1%	91,2%	92,1%	83,2%
7796	35	55	25	60	84,2%	86,9%	91,4%	92,6%	84,1%
7797	35	55	25	65	84,4%	86,8%	91,7%	93,1%	85,0%
7798	35	55	25	70	84,6%	86,6%	92,0%	93,5%	85,9%
7799	35	55	25	75	84,8%	86,5%	92,2%	93,9%	86,7%
7800	35	55	25	80	85,1%	86,3%	92,5%	94,3%	87,5%
7801	35	55	30	20	78,3%	85,4%	87,8%	87,1%	74,6%
7802	35	55	30	25	78,6%	85,3%	88,2%	87,9%	75,9%
7803	35	55	30	30	78,9%	85,1%	88,5%	88,6%	77,1%
7804	35	55	30	35	79,2%	84,9%	88,8%	89,3%	78,3%
7805	35	55	30	40	79,4%	84,8%	89,2%	89,9%	79,5%
7806	35	55	30	45	79,7%	84,6%	89,5%	90,5%	80,6%
7807	35	55	30	50	80,0%	84,4%	89,8%	91,1%	81,6%
7808	35	55	30	55	80,3%	84,3%	90,1%	91,7%	82,6%
7809	35	55	30	60	80,6%	84,1%	90,4%	92,2%	83,6%
7810	35	55	30	65	80,9%	83,9%	90,7%	92,6%	84,5%
7811	35	55	30	70	81,1%	83,7%	91,0%	93,1%	85,4%
7812	35	55	30	75	81,4%	83,6%	91,3%	93,5%	86,2%
7813	35	55	30	80	81,7%	83,4%	91,5%	93,9%	87,0%
7814	35	55	35	20	73,8%	82,3%	86,4%	86,4%	73,8%
7815	35	55	35	25	74,1%	82,1%	86,8%	87,2%	75,1%
7816	35	55	35	30	74,5%	81,9%	87,2%	88,0%	76,4%
7817	35	55	35	35	74,8%	81,7%	87,6%	88,7%	77,6%
7818	35	55	35	40	75,1%	81,5%	87,9%	89,4%	78,8%
7819	35	55	35	45	75,5%	81,3%	88,3%	90,0%	79,9%
7820	35	55	35	50	75,8%	81,1%	88,6%	90,6%	81,0%

7821	35	55	35	55	76,1%	80,9%	89,0%	91,2%	82,0%
7822	35	55	35	60	76,4%	80,7%	89,3%	91,7%	83,0%
7823	35	55	35	65	76,7%	80,5%	89,6%	92,2%	83,9%
7824	35	55	35	70	77,0%	80,3%	89,9%	92,7%	84,9%
7825	35	55	35	75	77,4%	80,1%	90,2%	93,2%	85,7%
7826	35	55	35	80	77,7%	79,9%	90,5%	93,6%	86,5%
7827	35	55	40	20	68,7%	78,7%	84,9%	85,7%	73,0%
7828	35	55	40	25	69,1%	78,5%	85,3%	86,5%	74,4%
7829	35	55	40	30	69,5%	78,3%	85,7%	87,3%	75,7%
7830	35	55	40	35	69,9%	78,0%	86,1%	88,1%	76,9%
7831	35	55	40	40	70,2%	77,8%	86,5%	88,8%	78,1%
7832	35	55	40	45	70,6%	77,6%	86,9%	89,4%	79,2%
7833	35	55	40	50	71,0%	77,4%	87,3%	90,1%	80,3%
7834	35	55	40	55	71,3%	77,1%	87,7%	90,7%	81,4%
7835	35	55	40	60	71,7%	76,9%	88,0%	91,2%	82,4%
7836	35	55	40	65	72,0%	76,7%	88,4%	91,8%	83,4%
7837	35	55	40	70	72,4%	76,4%	88,7%	92,3%	84,3%
7838	35	55	40	75	72,7%	76,2%	89,1%	92,8%	85,2%
7839	35	55	40	80	73,1%	76,0%	89,4%	93,2%	86,0%
7840	35	55	45	20	63,2%	74,6%	83,2%	84,9%	72,2%
7841	35	55	45	25	63,6%	74,3%	83,7%	85,8%	73,6%
7842	35	55	45	30	64,0%	74,1%	84,1%	86,6%	74,9%
7843	35	55	45	35	64,4%	73,8%	84,6%	87,4%	76,1%
7844	35	55	45	40	64,8%	73,6%	85,0%	88,1%	77,4%
7845	35	55	45	45	65,2%	73,3%	85,5%	88,8%	78,5%
7846	35	55	45	50	65,6%	73,1%	85,9%	89,5%	79,7%
7847	35	55	45	55	66,0%	72,8%	86,3%	90,1%	80,8%
7848	35	55	45	60	66,4%	72,5%	86,7%	90,7%	81,8%
7849	35	55	45	65	66,8%	72,3%	87,1%	91,3%	82,8%
7850	35	55	45	70	67,2%	72,0%	87,4%	91,8%	83,8%
7851	35	55	45	75	67,6%	71,8%	87,8%	92,3%	84,7%
7852	35	55	45	80	68,0%	71,5%	88,2%	92,8%	85,5%
7853	35	55	50	20	57,3%	70,0%	81,4%	84,1%	71,4%

7854	35	55	50	25	57,7%	69,7%	81,9%	85,0%	72,8%
7855	35	55	50	30	58,2%	69,4%	82,4%	85,9%	74,1%
7856	35	55	50	35	58,6%	69,1%	82,9%	86,7%	75,4%
7857	35	55	50	40	59,0%	68,8%	83,4%	87,5%	76,6%
7858	35	55	50	45	59,4%	68,6%	83,8%	88,2%	77,8%
7859	35	55	50	50	59,9%	68,3%	84,3%	88,9%	79,0%
7860	35	55	50	55	60,3%	68,0%	84,7%	89,6%	80,1%
7861	35	55	50	60	60,7%	67,7%	85,2%	90,2%	81,2%
7862	35	55	50	65	61,1%	67,4%	85,6%	90,8%	82,2%
7863	35	55	50	70	61,5%	67,1%	86,0%	91,4%	83,2%
7864	35	55	50	75	62,0%	66,9%	86,4%	91,9%	84,1%
7865	35	55	50	80	62,4%	66,6%	86,8%	92,4%	85,0%
7866	35	55	55	20	51,2%	64,9%	79,4%	83,3%	70,5%
7867	35	55	55	25	51,6%	64,6%	80,0%	84,3%	71,9%
7868	35	55	55	30	52,1%	64,3%	80,5%	85,1%	73,3%
7869	35	55	55	35	52,5%	64,0%	81,1%	86,0%	74,6%
7870	35	55	55	40	52,9%	63,7%	81,6%	86,8%	75,9%
7871	35	55	55	45	53,4%	63,4%	82,1%	87,6%	77,1%
7872	35	55	55	50	53,8%	63,1%	82,6%	88,3%	78,3%
7873	35	55	55	55	54,3%	62,8%	83,1%	89,0%	79,5%
7874	35	55	55	60	54,7%	62,5%	83,5%	89,7%	80,5%
7875	35	55	55	65	55,1%	62,2%	84,0%	90,3%	81,6%
7876	35	55	55	70	55,6%	61,9%	84,4%	90,9%	82,6%
7877	35	55	55	75	56,0%	61,6%	84,9%	91,4%	83,6%
7878	35	55	55	80	56,4%	61,2%	85,3%	92,0%	84,5%
7879	35	55	60	20	45,0%	59,5%	77,3%	82,5%	69,7%
7880	35	55	60	25	45,5%	59,2%	77,9%	83,4%	71,1%
7881	35	55	60	30	45,9%	58,8%	78,5%	84,4%	72,5%
7882	35	55	60	35	46,3%	58,5%	79,1%	85,2%	73,8%
7883	35	55	60	40	46,8%	58,2%	79,6%	86,1%	75,1%
7884	35	55	60	45	47,2%	57,9%	80,2%	86,9%	76,4%
7885	35	55	60	50	47,7%	57,6%	80,7%	87,7%	77,6%
7886	35	55	60	55	48,1%	57,2%	81,2%	88,4%	78,8%

7887	35	55	60	60	48,5%	56,9%	81,8%	89,1%	79,9%
7888	35	55	60	65	49,0%	56,6%	82,3%	89,7%	81,0%
7889	35	55	60	70	49,4%	56,3%	82,7%	90,4%	82,0%
7890	35	55	60	75	49,9%	56,0%	83,2%	90,9%	83,0%
7891	35	55	60	80	50,3%	55,6%	83,7%	91,5%	83,9%
7892	35	55	65	20	39,0%	53,8%	75,1%	81,6%	68,8%
7893	35	55	65	25	39,4%	53,5%	75,7%	82,6%	70,2%
7894	35	55	65	30	39,9%	53,2%	76,3%	83,5%	71,7%
7895	35	55	65	35	40,3%	52,8%	77,0%	84,5%	73,0%
7896	35	55	65	40	40,7%	52,5%	77,5%	85,3%	74,4%
7897	35	55	65	45	41,1%	52,2%	78,1%	86,2%	75,6%
7898	35	55	65	50	41,6%	51,9%	78,7%	87,0%	76,9%
7899	35	55	65	55	42,0%	51,5%	79,3%	87,8%	78,1%
7900	35	55	65	60	42,4%	51,2%	79,8%	88,5%	79,2%
7901	35	55	65	65	42,9%	50,9%	80,4%	89,2%	80,3%
7902	35	55	65	70	43,3%	50,5%	80,9%	89,8%	81,4%
7903	35	55	65	75	43,7%	50,2%	81,4%	90,4%	82,4%
7904	35	55	65	80	44,2%	49,9%	81,9%	91,0%	83,4%
7905	35	55	70	20	33,3%	48,0%	72,7%	80,6%	67,9%
7906	35	55	70	25	33,7%	47,7%	73,4%	81,7%	69,4%
7907	35	55	70	30	34,1%	47,4%	74,0%	82,7%	70,8%
7908	35	55	70	35	34,5%	47,1%	74,7%	83,6%	72,2%
7909	35	55	70	40	34,9%	46,7%	75,3%	84,6%	73,6%
7910	35	55	70	45	35,3%	46,4%	75,9%	85,4%	74,9%
7911	35	55	70	50	35,7%	46,1%	76,6%	86,3%	76,1%
7912	35	55	70	55	36,1%	45,8%	77,2%	87,1%	77,4%
7913	35	55	70	60	36,5%	45,4%	77,8%	87,8%	78,5%
7914	35	55	70	65	36,9%	45,1%	78,3%	88,6%	79,7%
7915	35	55	70	70	37,4%	44,8%	78,9%	89,2%	80,8%
7916	35	55	70	75	37,8%	44,5%	79,5%	89,9%	81,8%
7917	35	55	70	80	38,2%	44,1%	80,0%	90,5%	82,8%
7918	35	55	75	20	28,1%	42,3%	70,2%	79,6%	67,0%
7919	35	55	75	25	28,4%	42,0%	70,9%	80,7%	68,5%

7920	35	55	75	30	28,8%	41,7%	71,6%	81,8%	70,0%
7921	35	55	75	35	29,2%	41,4%	72,3%	82,8%	71,4%
7922	35	55	75	40	29,5%	41,1%	72,9%	83,8%	72,7%
7923	35	55	75	45	29,9%	40,7%	73,6%	84,7%	74,1%
7924	35	55	75	50	30,3%	40,4%	74,3%	85,5%	75,4%
7925	35	55	75	55	30,6%	40,1%	74,9%	86,4%	76,6%
7926	35	55	75	60	31,0%	39,8%	75,5%	87,2%	77,8%
7927	35	55	75	65	31,4%	39,5%	76,2%	87,9%	79,0%
7928	35	55	75	70	31,8%	39,2%	76,8%	88,6%	80,1%
7929	35	55	75	75	32,2%	38,9%	77,4%	89,3%	81,2%
7930	35	55	75	80	32,5%	38,5%	78,0%	90,0%	82,2%
7931	35	55	80	20	23,4%	36,8%	67,5%	78,6%	66,1%
7932	35	55	80	25	23,7%	36,5%	68,2%	79,8%	67,6%
7933	35	55	80	30	24,0%	36,2%	69,0%	80,9%	69,1%
7934	35	55	80	35	24,3%	35,9%	69,7%	81,9%	70,5%
7935	35	55	80	40	24,7%	35,6%	70,4%	82,9%	71,9%
7936	35	55	80	45	25,0%	35,3%	71,1%	83,9%	73,3%
7937	35	55	80	50	25,3%	35,0%	71,8%	84,8%	74,6%
7938	35	55	80	55	25,7%	34,7%	72,5%	85,6%	75,9%
7939	35	55	80	60	26,0%	34,4%	73,2%	86,5%	77,1%
7940	35	55	80	65	26,3%	34,1%	73,8%	87,3%	78,3%
7941	35	55	80	70	26,7%	33,8%	74,5%	88,0%	79,4%
7942	35	55	80	75	27,0%	33,5%	75,1%	88,7%	80,5%
7943	35	55	80	80	27,4%	33,2%	75,7%	89,4%	81,6%
7944	35	60	20	20	86,6%	91,0%	89,9%	87,7%	74,4%
7945	35	60	20	25	86,8%	90,9%	90,2%	88,4%	75,7%
7946	35	60	20	30	87,0%	90,8%	90,5%	89,1%	76,9%
7947	35	60	20	35	87,2%	90,6%	90,8%	89,7%	78,1%
7948	35	60	20	40	87,4%	90,5%	91,1%	90,3%	79,3%
7949	35	60	20	45	87,6%	90,4%	91,4%	90,9%	80,4%
7950	35	60	20	50	87,8%	90,3%	91,6%	91,5%	81,4%
7951	35	60	20	55	87,9%	90,2%	91,9%	92,0%	82,4%
7952	35	60	20	60	88,1%	90,1%	92,1%	92,5%	83,4%

7953	35	60	20	65	88,3%	90,0%	92,4%	93,0%	84,3%
7954	35	60	20	70	88,5%	89,8%	92,6%	93,4%	85,2%
7955	35	60	20	75	88,7%	89,7%	92,8%	93,8%	86,1%
7956	35	60	20	80	88,9%	89,6%	93,1%	94,2%	86,9%
7957	35	60	25	20	83,4%	88,9%	88,8%	87,0%	73,6%
7958	35	60	25	25	83,7%	88,8%	89,1%	87,7%	74,9%
7959	35	60	25	30	83,9%	88,6%	89,4%	88,5%	76,2%
7960	35	60	25	35	84,2%	88,5%	89,7%	89,2%	77,4%
7961	35	60	25	40	84,4%	88,4%	90,0%	89,8%	78,6%
7962	35	60	25	45	84,6%	88,2%	90,3%	90,4%	79,7%
7963	35	60	25	50	84,9%	88,1%	90,6%	91,0%	80,8%
7964	35	60	25	55	85,1%	87,9%	90,9%	91,5%	81,8%
7965	35	60	25	60	85,3%	87,8%	91,2%	92,1%	82,8%
7966	35	60	25	65	85,5%	87,7%	91,5%	92,6%	83,8%
7967	35	60	25	70	85,7%	87,5%	91,7%	93,0%	84,7%
7968	35	60	25	75	86,0%	87,4%	92,0%	93,5%	85,5%
7969	35	60	25	80	86,2%	87,2%	92,2%	93,9%	86,4%
7970	35	60	30	20	79,7%	86,4%	87,5%	86,3%	72,8%
7971	35	60	30	25	80,0%	86,2%	87,8%	87,1%	74,1%
7972	35	60	30	30	80,3%	86,1%	88,2%	87,8%	75,4%
7973	35	60	30	35	80,6%	85,9%	88,5%	88,6%	76,7%
7974	35	60	30	40	80,9%	85,8%	88,9%	89,2%	77,9%
7975	35	60	30	45	81,1%	85,6%	89,2%	89,9%	79,0%
7976	35	60	30	50	81,4%	85,4%	89,5%	90,5%	80,1%
7977	35	60	30	55	81,7%	85,3%	89,8%	91,1%	81,2%
7978	35	60	30	60	81,9%	85,1%	90,1%	91,6%	82,2%
7979	35	60	30	65	82,2%	84,9%	90,4%	92,1%	83,2%
7980	35	60	30	70	82,4%	84,8%	90,7%	92,6%	84,1%
7981	35	60	30	75	82,7%	84,6%	91,0%	93,1%	85,0%
7982	35	60	30	80	82,9%	84,4%	91,3%	93,5%	85,9%
7983	35	60	35	20	75,5%	83,4%	86,0%	85,5%	72,0%
7984	35	60	35	25	75,8%	83,3%	86,4%	86,4%	73,3%
7985	35	60	35	30	76,1%	83,1%	86,8%	87,2%	74,6%

7986	35	60	35	35	76,4%	82,9%	87,2%	87,9%	75,9%
7987	35	60	35	40	76,7%	82,7%	87,6%	88,6%	77,1%
7988	35	60	35	45	77,1%	82,5%	87,9%	89,3%	78,3%
7989	35	60	35	50	77,4%	82,3%	88,3%	90,0%	79,5%
7990	35	60	35	55	77,7%	82,1%	88,6%	90,6%	80,6%
7991	35	60	35	60	78,0%	81,9%	89,0%	91,1%	81,6%
7992	35	60	35	65	78,3%	81,7%	89,3%	91,7%	82,6%
7993	35	60	35	70	78,6%	81,5%	89,6%	92,2%	83,6%
7994	35	60	35	75	78,9%	81,4%	89,9%	92,7%	84,5%
7995	35	60	35	80	79,2%	81,2%	90,2%	93,1%	85,4%
7996	35	60	40	20	70,6%	80,0%	84,5%	84,8%	71,1%
7997	35	60	40	25	71,0%	79,8%	84,9%	85,6%	72,5%
7998	35	60	40	30	71,3%	79,6%	85,3%	86,5%	73,8%
7999	35	60	40	35	71,7%	79,4%	85,8%	87,3%	75,2%
8000	35	60	40	40	72,1%	79,1%	86,2%	88,0%	76,4%
8001	35	60	40	45	72,4%	78,9%	86,6%	88,7%	77,6%
8002	35	60	40	50	72,8%	78,7%	87,0%	89,4%	78,8%
8003	35	60	40	55	73,1%	78,5%	87,3%	90,0%	79,9%
8004	35	60	40	60	73,5%	78,3%	87,7%	90,6%	81,0%
8005	35	60	40	65	73,8%	78,0%	88,1%	91,2%	82,0%
8006	35	60	40	70	74,1%	77,8%	88,4%	91,7%	83,0%
8007	35	60	40	75	74,5%	77,6%	88,8%	92,2%	84,0%
8008	35	60	40	80	74,8%	77,4%	89,1%	92,7%	84,9%
8009	35	60	45	20	65,2%	76,0%	82,8%	84,0%	70,3%
8010	35	60	45	25	65,6%	75,8%	83,2%	84,9%	71,7%
8011	35	60	45	30	66,0%	75,6%	83,7%	85,7%	73,0%
8012	35	60	45	35	66,4%	75,3%	84,2%	86,6%	74,4%
8013	35	60	45	40	66,8%	75,1%	84,6%	87,3%	75,7%
8014	35	60	45	45	67,2%	74,8%	85,1%	88,1%	76,9%
8015	35	60	45	50	67,6%	74,6%	85,5%	88,8%	78,1%
8016	35	60	45	55	68,0%	74,3%	85,9%	89,5%	79,2%
8017	35	60	45	60	68,4%	74,1%	86,3%	90,1%	80,3%
8018	35	60	45	65	68,7%	73,8%	86,7%	90,7%	81,4%

8019	35	60	45	70	69,1%	73,6%	87,1%	91,3%	82,4%
8020	35	60	45	75	69,5%	73,3%	87,5%	91,8%	83,4%
8021	35	60	45	80	69,9%	73,1%	87,8%	92,3%	84,3%
8022	35	60	50	20	59,5%	71,6%	80,9%	83,1%	69,4%
8023	35	60	50	25	59,9%	71,3%	81,4%	84,1%	70,8%
8024	35	60	50	30	60,3%	71,1%	81,9%	85,0%	72,2%
8025	35	60	50	35	60,7%	70,8%	82,4%	85,8%	73,6%
8026	35	60	50	40	61,1%	70,5%	82,9%	86,7%	74,9%
8027	35	60	50	45	61,6%	70,2%	83,4%	87,4%	76,2%
8028	35	60	50	50	62,0%	70,0%	83,9%	88,2%	77,4%
8029	35	60	50	55	62,4%	69,7%	84,3%	88,9%	78,6%
8030	35	60	50	60	62,8%	69,4%	84,8%	89,5%	79,7%
8031	35	60	50	65	63,2%	69,1%	85,2%	90,2%	80,8%
8032	35	60	50	70	63,6%	68,9%	85,6%	90,8%	81,8%
8033	35	60	50	75	64,0%	68,6%	86,0%	91,3%	82,8%
8034	35	60	50	80	64,4%	68,3%	86,4%	91,9%	83,8%
8035	35	60	55	20	53,4%	66,7%	78,9%	82,3%	68,5%
8036	35	60	55	25	53,8%	66,4%	79,5%	83,2%	70,0%
8037	35	60	55	30	54,3%	66,1%	80,0%	84,2%	71,4%
8038	35	60	55	35	54,7%	65,8%	80,6%	85,1%	72,8%
8039	35	60	55	40	55,1%	65,5%	81,1%	85,9%	74,1%
8040	35	60	55	45	55,6%	65,2%	81,6%	86,7%	75,4%
8041	35	60	55	50	56,0%	64,9%	82,1%	87,5%	76,6%
8042	35	60	55	55	56,4%	64,6%	82,6%	88,3%	77,8%
8043	35	60	55	60	56,9%	64,3%	83,1%	89,0%	79,0%
8044	35	60	55	65	57,3%	64,0%	83,6%	89,6%	80,1%
8045	35	60	55	70	57,7%	63,7%	84,0%	90,2%	81,2%
8046	35	60	55	75	58,2%	63,4%	84,5%	90,8%	82,2%
8047	35	60	55	80	58,6%	63,1%	84,9%	91,4%	83,2%
8048	35	60	60	20	47,2%	61,4%	76,8%	81,4%	67,6%
8049	35	60	60	25	47,7%	61,0%	77,4%	82,4%	69,1%
8050	35	60	60	30	48,1%	60,7%	78,0%	83,4%	70,5%
8051	35	60	60	35	48,5%	60,4%	78,6%	84,3%	71,9%

8052	35	60	60	40	49,0%	60,1%	79,1%	85,2%	73,3%
8053	35	60	60	45	49,4%	59,8%	79,7%	86,0%	74,6%
8054	35	60	60	50	49,9%	59,5%	80,2%	86,8%	75,9%
8055	35	60	60	55	50,3%	59,2%	80,8%	87,6%	77,1%
8056	35	60	60	60	50,8%	58,8%	81,3%	88,3%	78,3%
8057	35	60	60	65	51,2%	58,5%	81,8%	89,0%	79,5%
8058	35	60	60	70	51,6%	58,2%	82,3%	89,7%	80,6%
8059	35	60	60	75	52,1%	57,9%	82,8%	90,3%	81,6%
8060	35	60	60	80	52,5%	57,6%	83,3%	90,9%	82,6%
8061	35	60	65	20	41,1%	55,8%	74,5%	80,4%	66,7%
8062	35	60	65	25	41,6%	55,4%	75,1%	81,5%	68,2%
8063	35	60	65	30	42,0%	55,1%	75,8%	82,5%	69,7%
8064	35	60	65	35	42,4%	54,8%	76,4%	83,5%	71,1%
8065	35	60	65	40	42,9%	54,5%	77,0%	84,4%	72,5%
8066	35	60	65	45	43,3%	54,1%	77,6%	85,3%	73,8%
8067	35	60	65	50	43,7%	53,8%	78,2%	86,1%	75,1%
8068	35	60	65	55	44,2%	53,5%	78,7%	86,9%	76,4%
8069	35	60	65	60	44,6%	53,2%	79,3%	87,7%	77,6%
8070	35	60	65	65	45,0%	52,8%	79,9%	88,4%	78,8%
8071	35	60	65	70	45,5%	52,5%	80,4%	89,1%	79,9%
8072	35	60	65	75	45,9%	52,2%	80,9%	89,8%	81,0%
8073	35	60	65	80	46,3%	51,9%	81,5%	90,4%	82,0%
8074	35	60	70	20	35,3%	50,0%	72,1%	79,4%	65,8%
8075	35	60	70	25	35,7%	49,7%	72,7%	80,5%	67,3%
8076	35	60	70	30	36,1%	49,4%	73,4%	81,6%	68,8%
8077	35	60	70	35	36,5%	49,0%	74,1%	82,6%	70,2%
8078	35	60	70	40	37,0%	48,7%	74,7%	83,6%	71,7%
8079	35	60	70	45	37,4%	48,4%	75,4%	84,5%	73,0%
8080	35	60	70	50	37,8%	48,0%	76,0%	85,4%	74,4%
8081	35	60	70	55	38,2%	47,7%	76,6%	86,2%	75,6%
8082	35	60	70	60	38,6%	47,4%	77,2%	87,0%	76,9%
8083	35	60	70	65	39,0%	47,1%	77,8%	87,8%	78,1%
8084	35	60	70	70	39,4%	46,7%	78,4%	88,5%	79,2%

8085	35	60	70	75	39,9%	46,4%	78,9%	89,2%	80,3%
8086	35	60	70	80	40,3%	46,1%	79,5%	89,8%	81,4%
8087	35	60	75	20	29,9%	44,3%	69,5%	78,4%	64,9%
8088	35	60	75	25	30,3%	43,9%	70,2%	79,6%	66,4%
8089	35	60	75	30	30,7%	43,6%	70,9%	80,7%	67,9%
8090	35	60	75	35	31,0%	43,3%	71,6%	81,7%	69,4%
8091	35	60	75	40	31,4%	43,0%	72,3%	82,7%	70,8%
8092	35	60	75	45	31,8%	42,7%	73,0%	83,7%	72,2%
8093	35	60	75	50	32,2%	42,3%	73,6%	84,6%	73,6%
8094	35	60	75	55	32,6%	42,0%	74,3%	85,5%	74,9%
8095	35	60	75	60	32,9%	41,7%	74,9%	86,3%	76,1%
8096	35	60	75	65	33,3%	41,4%	75,6%	87,1%	77,4%
8097	35	60	75	70	33,7%	41,1%	76,2%	87,9%	78,5%
8098	35	60	75	75	34,1%	40,7%	76,8%	88,6%	79,7%
8099	35	60	75	80	34,5%	40,4%	77,4%	89,3%	80,8%
8100	35	60	80	20	25,0%	38,7%	66,8%	77,4%	63,9%
8101	35	60	80	25	25,3%	38,4%	67,6%	78,6%	65,5%
8102	35	60	80	30	25,7%	38,0%	68,3%	79,7%	67,0%
8103	35	60	80	35	26,0%	37,7%	69,0%	80,8%	68,5%
8104	35	60	80	40	26,3%	37,4%	69,7%	81,8%	70,0%
8105	35	60	80	45	26,7%	37,1%	70,5%	82,8%	71,4%
8106	35	60	80	50	27,0%	36,8%	71,2%	83,8%	72,8%
8107	35	60	80	55	27,4%	36,5%	71,9%	84,7%	74,1%
8108	35	60	80	60	27,7%	36,2%	72,5%	85,6%	75,4%
8109	35	60	80	65	28,1%	35,9%	73,2%	86,4%	76,6%
8110	35	60	80	70	28,4%	35,6%	73,9%	87,2%	77,8%
8111	35	60	80	75	28,8%	35,3%	74,5%	88,0%	79,0%
8112	35	60	80	80	29,2%	35,0%	75,2%	88,7%	80,1%
8113	35	65	20	20	87,6%	91,6%	89,6%	86,8%	72,5%
8114	35	65	20	25	87,8%	91,5%	90,0%	87,6%	73,9%
8115	35	65	20	30	88,0%	91,4%	90,3%	88,3%	75,2%
8116	35	65	20	35	88,1%	91,3%	90,6%	89,0%	76,4%
8117	35	65	20	40	88,3%	91,2%	90,8%	89,7%	77,6%

8118	35	65	20	45	88,5%	91,1%	91,1%	90,3%	78,8%
8119	35	65	20	50	88,7%	91,0%	91,4%	90,9%	79,9%
8120	35	65	20	55	88,9%	90,9%	91,7%	91,4%	81,0%
8121	35	65	20	60	89,0%	90,8%	91,9%	92,0%	82,0%
8122	35	65	20	65	89,2%	90,6%	92,2%	92,5%	83,0%
8123	35	65	20	70	89,4%	90,5%	92,4%	92,9%	84,0%
8124	35	65	20	75	89,5%	90,4%	92,6%	93,4%	84,9%
8125	35	65	20	80	89,7%	90,3%	92,9%	93,8%	85,7%
8126	35	65	25	20	84,6%	89,6%	88,4%	86,1%	71,7%
8127	35	65	25	25	84,9%	89,5%	88,8%	86,9%	73,1%
8128	35	65	25	30	85,1%	89,4%	89,1%	87,7%	74,4%
8129	35	65	25	35	85,3%	89,3%	89,4%	88,4%	75,7%
8130	35	65	25	40	85,5%	89,1%	89,8%	89,1%	76,9%
8131	35	65	25	45	85,7%	89,0%	90,1%	89,8%	78,1%
8132	35	65	25	50	86,0%	88,9%	90,4%	90,4%	79,3%
8133	35	65	25	55	86,2%	88,8%	90,7%	91,0%	80,4%
8134	35	65	25	60	86,4%	88,6%	90,9%	91,5%	81,4%
8135	35	65	25	65	86,6%	88,5%	91,2%	92,0%	82,4%
8136	35	65	25	70	86,8%	88,4%	91,5%	92,5%	83,4%
8137	35	65	25	75	87,0%	88,2%	91,7%	93,0%	84,3%
8138	35	65	25	80	87,2%	88,1%	92,0%	93,4%	85,2%
8139	35	65	30	20	81,1%	87,3%	87,1%	85,4%	70,9%
8140	35	65	30	25	81,4%	87,1%	87,5%	86,2%	72,2%
8141	35	65	30	30	81,7%	87,0%	87,8%	87,0%	73,6%
8142	35	65	30	35	81,9%	86,8%	88,2%	87,8%	74,9%
8143	35	65	30	40	82,2%	86,7%	88,6%	88,5%	76,2%
8144	35	65	30	45	82,5%	86,5%	88,9%	89,2%	77,4%
8145	35	65	30	50	82,7%	86,4%	89,2%	89,8%	78,6%
8146	35	65	30	55	83,0%	86,2%	89,5%	90,4%	79,7%
8147	35	65	30	60	83,2%	86,1%	89,9%	91,0%	80,8%
8148	35	65	30	65	83,4%	85,9%	90,2%	91,6%	81,8%
8149	35	65	30	70	83,7%	85,8%	90,5%	92,1%	82,8%
8150	35	65	30	75	83,9%	85,6%	90,7%	92,6%	83,8%

8151	35	65	30	80	84,2%	85,4%	91,0%	93,0%	84,7%
8152	35	65	35	20	77,1%	84,5%	85,6%	84,6%	70,0%
8153	35	65	35	25	77,4%	84,3%	86,1%	85,5%	71,4%
8154	35	65	35	30	77,7%	84,2%	86,5%	86,3%	72,8%
8155	35	65	35	35	78,0%	84,0%	86,8%	87,1%	74,1%
8156	35	65	35	40	78,3%	83,8%	87,2%	87,9%	75,4%
8157	35	65	35	45	78,6%	83,6%	87,6%	88,6%	76,7%
8158	35	65	35	50	78,9%	83,4%	88,0%	89,3%	77,9%
8159	35	65	35	55	79,2%	83,3%	88,3%	89,9%	79,0%
8160	35	65	35	60	79,5%	83,1%	88,7%	90,5%	80,1%
8161	35	65	35	65	79,8%	82,9%	89,0%	91,1%	81,2%
8162	35	65	35	70	80,0%	82,7%	89,3%	91,6%	82,2%
8163	35	65	35	75	80,3%	82,5%	89,7%	92,1%	83,2%
8164	35	65	35	80	80,6%	82,3%	90,0%	92,6%	84,1%
8165	35	65	40	20	72,4%	81,2%	84,0%	83,8%	69,1%
8166	35	65	40	25	72,8%	81,0%	84,5%	84,7%	70,6%
8167	35	65	40	30	73,1%	80,8%	84,9%	85,6%	72,0%
8168	35	65	40	35	73,5%	80,6%	85,4%	86,4%	73,3%
8169	35	65	40	40	73,8%	80,4%	85,8%	87,2%	74,6%
8170	35	65	40	45	74,1%	80,2%	86,2%	87,9%	75,9%
8171	35	65	40	50	74,5%	80,0%	86,6%	88,7%	77,1%
8172	35	65	40	55	74,8%	79,8%	87,0%	89,3%	78,3%
8173	35	65	40	60	75,1%	79,6%	87,4%	90,0%	79,5%
8174	35	65	40	65	75,5%	79,4%	87,7%	90,6%	80,6%
8175	35	65	40	70	75,8%	79,1%	88,1%	91,2%	81,6%
8176	35	65	40	75	76,1%	78,9%	88,4%	91,7%	82,6%
8177	35	65	40	80	76,4%	78,7%	88,8%	92,2%	83,6%
8178	35	65	45	20	67,2%	77,4%	82,3%	82,9%	68,2%
8179	35	65	45	25	67,6%	77,2%	82,8%	83,9%	69,7%
8180	35	65	45	30	68,0%	77,0%	83,3%	84,8%	71,1%
8181	35	65	45	35	68,4%	76,8%	83,7%	85,7%	72,5%
8182	35	65	45	40	68,8%	76,5%	84,2%	86,5%	73,9%
8183	35	65	45	45	69,1%	76,3%	84,6%	87,3%	75,2%

8184	35	65	45	50	69,5%	76,0%	85,1%	88,0%	76,4%
8185	35	65	45	55	69,9%	75,8%	85,5%	88,7%	77,6%
8186	35	65	45	60	70,3%	75,6%	85,9%	89,4%	78,8%
8187	35	65	45	65	70,6%	75,3%	86,3%	90,0%	79,9%
8188	35	65	45	70	71,0%	75,1%	86,7%	90,6%	81,0%
8189	35	65	45	75	71,3%	74,8%	87,1%	91,2%	82,0%
8190	35	65	45	80	71,7%	74,6%	87,5%	91,8%	83,0%
8191	35	65	50	20	61,6%	73,2%	80,4%	82,1%	67,3%
8192	35	65	50	25	62,0%	72,9%	81,0%	83,1%	68,8%
8193	35	65	50	30	62,4%	72,6%	81,5%	84,0%	70,3%
8194	35	65	50	35	62,8%	72,4%	82,0%	84,9%	71,7%
8195	35	65	50	40	63,2%	72,1%	82,5%	85,8%	73,1%
8196	35	65	50	45	63,6%	71,9%	83,0%	86,6%	74,4%
8197	35	65	50	50	64,0%	71,6%	83,4%	87,4%	75,7%
8198	35	65	50	55	64,5%	71,3%	83,9%	88,1%	76,9%
8199	35	65	50	60	64,9%	71,1%	84,4%	88,8%	78,1%
8200	35	65	50	65	65,3%	70,8%	84,8%	89,5%	79,2%
8201	35	65	50	70	65,7%	70,5%	85,2%	90,1%	80,3%
8202	35	65	50	75	66,0%	70,2%	85,6%	90,7%	81,4%
8203	35	65	50	80	66,4%	70,0%	86,1%	91,3%	82,4%
8204	35	65	55	20	55,6%	68,4%	78,4%	81,2%	66,4%
8205	35	65	55	25	56,0%	68,1%	79,0%	82,2%	67,9%
8206	35	65	55	30	56,5%	67,8%	79,5%	83,2%	69,4%
8207	35	65	55	35	56,9%	67,5%	80,1%	84,1%	70,8%
8208	35	65	55	40	57,3%	67,3%	80,6%	85,0%	72,2%
8209	35	65	55	45	57,8%	67,0%	81,1%	85,9%	73,6%
8210	35	65	55	50	58,2%	66,7%	81,6%	86,7%	74,9%
8211	35	65	55	55	58,6%	66,4%	82,1%	87,5%	76,2%
8212	35	65	55	60	59,0%	66,1%	82,6%	88,2%	77,4%
8213	35	65	55	65	59,5%	65,8%	83,1%	88,9%	78,6%
8214	35	65	55	70	59,9%	65,5%	83,6%	89,6%	79,7%
8215	35	65	55	75	60,3%	65,2%	84,1%	90,2%	80,8%
8216	35	65	55	80	60,7%	64,9%	84,5%	90,8%	81,8%

8217	35	65	60	20	49,4%	63,2%	76,2%	80,2%	65,5%
8218	35	65	60	25	49,9%	62,9%	76,8%	81,3%	67,0%
8219	35	65	60	30	50,3%	62,6%	77,4%	82,3%	68,5%
8220	35	65	60	35	50,8%	62,3%	78,0%	83,3%	70,0%
8221	35	65	60	40	51,2%	62,0%	78,6%	84,2%	71,4%
8222	35	65	60	45	51,6%	61,7%	79,2%	85,1%	72,8%
8223	35	65	60	50	52,1%	61,4%	79,7%	86,0%	74,1%
8224	35	65	60	55	52,5%	61,0%	80,3%	86,8%	75,4%
8225	35	65	60	60	53,0%	60,7%	80,8%	87,6%	76,7%
8226	35	65	60	65	53,4%	60,4%	81,3%	88,3%	77,9%
8227	35	65	60	70	53,8%	60,1%	81,8%	89,0%	79,0%
8228	35	65	60	75	54,3%	59,8%	82,3%	89,6%	80,1%
8229	35	65	60	80	54,7%	59,5%	82,8%	90,3%	81,2%
8230	35	65	65	20	43,3%	57,7%	73,9%	79,2%	64,6%
8231	35	65	65	25	43,7%	57,4%	74,5%	80,3%	66,1%
8232	35	65	65	30	44,2%	57,0%	75,2%	81,4%	67,6%
8233	35	65	65	35	44,6%	56,7%	75,8%	82,4%	69,1%
8234	35	65	65	40	45,1%	56,4%	76,4%	83,4%	70,5%
8235	35	65	65	45	45,5%	56,1%	77,0%	84,3%	71,9%
8236	35	65	65	50	45,9%	55,8%	77,6%	85,2%	73,3%
8237	35	65	65	55	46,4%	55,4%	78,2%	86,1%	74,6%
8238	35	65	65	60	46,8%	55,1%	78,8%	86,9%	75,9%
8239	35	65	65	65	47,2%	54,8%	79,3%	87,6%	77,1%
8240	35	65	65	70	47,7%	54,5%	79,9%	88,4%	78,3%
8241	35	65	65	75	48,1%	54,1%	80,4%	89,1%	79,5%
8242	35	65	65	80	48,6%	53,8%	81,0%	89,7%	80,6%
8243	35	65	70	20	37,4%	52,0%	71,4%	78,2%	63,6%
8244	35	65	70	25	37,8%	51,7%	72,1%	79,3%	65,2%
8245	35	65	70	30	38,2%	51,3%	72,8%	80,5%	66,7%
8246	35	65	70	35	38,6%	51,0%	73,5%	81,5%	68,2%
8247	35	65	70	40	39,0%	50,7%	74,1%	82,5%	69,7%
8248	35	65	70	45	39,5%	50,3%	74,8%	83,5%	71,1%
8249	35	65	70	50	39,9%	50,0%	75,4%	84,4%	72,5%

8250	35	65	70	55	40,3%	49,7%	76,0%	85,3%	73,8%
8251	35	65	70	60	40,7%	49,4%	76,6%	86,2%	75,1%
8252	35	65	70	65	41,2%	49,0%	77,2%	87,0%	76,4%
8253	35	65	70	70	41,6%	48,7%	77,8%	87,7%	77,6%
8254	35	65	70	75	42,0%	48,4%	78,4%	88,4%	78,8%
8255	35	65	70	80	42,4%	48,1%	79,0%	89,1%	79,9%
8256	35	65	75	20	31,8%	46,2%	68,8%	77,1%	62,6%
8257	35	65	75	25	32,2%	45,9%	69,5%	78,3%	64,2%
8258	35	65	75	30	32,6%	45,6%	70,3%	79,5%	65,8%
8259	35	65	75	35	33,0%	45,2%	71,0%	80,6%	67,3%
8260	35	65	75	40	33,3%	44,9%	71,7%	81,6%	68,8%
8261	35	65	75	45	33,7%	44,6%	72,3%	82,6%	70,3%
8262	35	65	75	50	34,1%	44,3%	73,0%	83,6%	71,7%
8263	35	65	75	55	34,5%	43,9%	73,7%	84,5%	73,0%
8264	35	65	75	60	34,9%	43,6%	74,3%	85,4%	74,4%
8265	35	65	75	65	35,3%	43,3%	75,0%	86,3%	75,6%
8266	35	65	75	70	35,7%	43,0%	75,6%	87,1%	76,9%
8267	35	65	75	75	36,1%	42,7%	76,2%	87,8%	78,1%
8268	35	65	75	80	36,6%	42,3%	76,8%	88,5%	79,2%
8269	35	65	80	20	26,7%	40,5%	66,1%	76,0%	61,7%
8270	35	65	80	25	27,0%	40,2%	66,9%	77,3%	63,3%
8271	35	65	80	30	27,4%	39,9%	67,6%	78,5%	64,9%
8272	35	65	80	35	27,7%	39,6%	68,3%	79,6%	66,4%
8273	35	65	80	40	28,1%	39,3%	69,1%	80,7%	67,9%
8274	35	65	80	45	28,5%	39,0%	69,8%	81,8%	69,4%
8275	35	65	80	50	28,8%	38,7%	70,5%	82,8%	70,8%
8276	35	65	80	55	29,2%	38,4%	71,2%	83,7%	72,2%
8277	35	65	80	60	29,6%	38,1%	71,9%	84,6%	73,6%
8278	35	65	80	65	29,9%	37,7%	72,6%	85,5%	74,9%
8279	35	65	80	70	30,3%	37,4%	73,2%	86,4%	76,1%
8280	35	65	80	75	30,7%	37,1%	73,9%	87,1%	77,4%
8281	35	65	80	80	31,0%	36,8%	74,6%	87,9%	78,5%
8282	35	70	20	20	88,5%	92,2%	89,3%	86,0%	70,6%

8283	35	70	20	25	88,7%	92,1%	89,7%	86,8%	72,0%
8284	35	70	20	30	88,9%	92,0%	90,0%	87,6%	73,3%
8285	35	70	20	35	89,0%	91,9%	90,3%	88,3%	74,7%
8286	35	70	20	40	89,2%	91,8%	90,6%	89,0%	75,9%
8287	35	70	20	45	89,4%	91,7%	90,9%	89,6%	77,2%
8288	35	70	20	50	89,5%	91,6%	91,1%	90,3%	78,3%
8289	35	70	20	55	89,7%	91,5%	91,4%	90,8%	79,5%
8290	35	70	20	60	89,9%	91,4%	91,7%	91,4%	80,6%
8291	35	70	20	65	90,0%	91,3%	91,9%	91,9%	81,6%
8292	35	70	20	70	90,2%	91,2%	92,2%	92,4%	82,6%
8293	35	70	20	75	90,3%	91,1%	92,4%	92,9%	83,6%
8294	35	70	20	80	90,5%	91,0%	92,6%	93,3%	84,5%
8295	35	70	25	20	85,8%	90,3%	88,1%	85,2%	69,7%
8296	35	70	25	25	86,0%	90,2%	88,5%	86,1%	71,1%
8297	35	70	25	30	86,2%	90,1%	88,8%	86,9%	72,5%
8298	35	70	25	35	86,4%	90,0%	89,1%	87,6%	73,9%
8299	35	70	25	40	86,6%	89,9%	89,5%	88,4%	75,2%
8300	35	70	25	45	86,8%	89,8%	89,8%	89,1%	76,4%
8301	35	70	25	50	87,0%	89,6%	90,1%	89,7%	77,6%
8302	35	70	25	55	87,2%	89,5%	90,4%	90,3%	78,8%
8303	35	70	25	60	87,4%	89,4%	90,7%	90,9%	79,9%
8304	35	70	25	65	87,6%	89,3%	91,0%	91,5%	81,0%
8305	35	70	25	70	87,8%	89,1%	91,2%	92,0%	82,0%
8306	35	70	25	75	88,0%	89,0%	91,5%	92,5%	83,0%
8307	35	70	25	80	88,1%	88,9%	91,8%	93,0%	84,0%
8308	35	70	30	20	82,5%	88,1%	86,7%	84,4%	68,8%
8309	35	70	30	25	82,7%	88,0%	87,1%	85,3%	70,3%
8310	35	70	30	30	83,0%	87,9%	87,5%	86,2%	71,7%
8311	35	70	30	35	83,2%	87,7%	87,9%	87,0%	73,1%
8312	35	70	30	40	83,5%	87,6%	88,2%	87,7%	74,4%
8313	35	70	30	45	83,7%	87,4%	88,6%	88,4%	75,7%
8314	35	70	30	50	83,9%	87,3%	88,9%	89,1%	76,9%
8315	35	70	30	55	84,2%	87,1%	89,2%	89,8%	78,1%

8316	35	70	30	60	84,4%	87,0%	89,6%	90,4%	79,3%
8317	35	70	30	65	84,6%	86,8%	89,9%	91,0%	80,4%
8318	35	70	30	70	84,9%	86,7%	90,2%	91,5%	81,4%
8319	35	70	30	75	85,1%	86,5%	90,5%	92,1%	82,4%
8320	35	70	30	80	85,3%	86,4%	90,8%	92,5%	83,4%
8321	35	70	35	20	78,6%	85,5%	85,2%	83,6%	67,9%
8322	35	70	35	25	78,9%	85,3%	85,7%	84,5%	69,4%
8323	35	70	35	30	79,2%	85,2%	86,1%	85,4%	70,9%
8324	35	70	35	35	79,5%	85,0%	86,5%	86,3%	72,3%
8325	35	70	35	40	79,8%	84,8%	86,9%	87,1%	73,6%
8326	35	70	35	45	80,0%	84,7%	87,3%	87,8%	74,9%
8327	35	70	35	50	80,3%	84,5%	87,6%	88,5%	76,2%
8328	35	70	35	55	80,6%	84,3%	88,0%	89,2%	77,4%
8329	35	70	35	60	80,9%	84,2%	88,3%	89,9%	78,6%
8330	35	70	35	65	81,1%	84,0%	88,7%	90,5%	79,7%
8331	35	70	35	70	81,4%	83,8%	89,0%	91,0%	80,8%
8332	35	70	35	75	81,7%	83,6%	89,4%	91,6%	81,8%
8333	35	70	35	80	81,9%	83,4%	89,7%	92,1%	82,8%
8334	35	70	40	20	74,2%	82,4%	83,6%	82,8%	67,0%
8335	35	70	40	25	74,5%	82,2%	84,1%	83,7%	68,5%
8336	35	70	40	30	74,8%	82,0%	84,5%	84,6%	70,0%
8337	35	70	40	35	75,2%	81,8%	85,0%	85,5%	71,4%
8338	35	70	40	40	75,5%	81,6%	85,4%	86,3%	72,8%
8339	35	70	40	45	75,8%	81,4%	85,8%	87,1%	74,1%
8340	35	70	40	50	76,1%	81,2%	86,2%	87,9%	75,4%
8341	35	70	40	55	76,4%	81,0%	86,6%	88,6%	76,7%
8342	35	70	40	60	76,8%	80,8%	87,0%	89,3%	77,9%
8343	35	70	40	65	77,1%	80,6%	87,4%	89,9%	79,0%
8344	35	70	40	70	77,4%	80,4%	87,8%	90,5%	80,1%
8345	35	70	40	75	77,7%	80,2%	88,1%	91,1%	81,2%
8346	35	70	40	80	78,0%	80,0%	88,5%	91,7%	82,2%
8347	35	70	45	20	69,2%	78,8%	81,8%	81,9%	66,1%
8348	35	70	45	25	69,5%	78,6%	82,3%	82,9%	67,6%

8349	35	70	45	30	69,9%	78,4%	82,8%	83,8%	69,1%
8350	35	70	45	35	70,3%	78,1%	83,3%	84,7%	70,6%
8351	35	70	45	40	70,6%	77,9%	83,8%	85,6%	72,0%
8352	35	70	45	45	71,0%	77,7%	84,2%	86,4%	73,3%
8353	35	70	45	50	71,4%	77,5%	84,7%	87,2%	74,6%
8354	35	70	45	55	71,7%	77,2%	85,1%	88,0%	75,9%
8355	35	70	45	60	72,1%	77,0%	85,5%	88,7%	77,2%
8356	35	70	45	65	72,4%	76,8%	85,9%	89,4%	78,3%
8357	35	70	45	70	72,8%	76,5%	86,4%	90,0%	79,5%
8358	35	70	45	75	73,1%	76,3%	86,7%	90,6%	80,6%
8359	35	70	45	80	73,5%	76,1%	87,1%	91,2%	81,6%
8360	35	70	50	20	63,7%	74,7%	79,9%	81,0%	65,2%
8361	35	70	50	25	64,1%	74,4%	80,5%	82,0%	66,7%
8362	35	70	50	30	64,5%	74,2%	81,0%	83,0%	68,2%
8363	35	70	50	35	64,9%	73,9%	81,5%	83,9%	69,7%
8364	35	70	50	40	65,3%	73,7%	82,0%	84,8%	71,1%
8365	35	70	50	45	65,7%	73,4%	82,5%	85,7%	72,5%
8366	35	70	50	50	66,1%	73,2%	83,0%	86,5%	73,9%
8367	35	70	50	55	66,5%	72,9%	83,5%	87,3%	75,2%
8368	35	70	50	60	66,8%	72,7%	83,9%	88,1%	76,4%
8369	35	70	50	65	67,2%	72,4%	84,4%	88,8%	77,6%
8370	35	70	50	70	67,6%	72,1%	84,8%	89,4%	78,8%
8371	35	70	50	75	68,0%	71,9%	85,3%	90,1%	79,9%
8372	35	70	50	80	68,4%	71,6%	85,7%	90,7%	81,0%
8373	35	70	55	20	57,8%	70,1%	77,8%	80,0%	64,3%
8374	35	70	55	25	58,2%	69,8%	78,4%	81,1%	65,8%
8375	35	70	55	30	58,6%	69,5%	79,0%	82,1%	67,3%
8376	35	70	55	35	59,1%	69,2%	79,6%	83,1%	68,8%
8377	35	70	55	40	59,5%	69,0%	80,1%	84,0%	70,3%
8378	35	70	55	45	59,9%	68,7%	80,6%	84,9%	71,7%
8379	35	70	55	50	60,3%	68,4%	81,2%	85,8%	73,1%
8380	35	70	55	55	60,7%	68,1%	81,7%	86,6%	74,4%
8381	35	70	55	60	61,2%	67,8%	82,2%	87,4%	75,7%

8382	35	70	55	65	61,6%	67,5%	82,7%	88,1%	76,9%
8383	35	70	55	70	62,0%	67,3%	83,2%	88,9%	78,1%
8384	35	70	55	75	62,4%	67,0%	83,6%	89,5%	79,2%
8385	35	70	55	80	62,8%	66,7%	84,1%	90,1%	80,4%
8386	35	70	60	20	51,7%	65,0%	75,6%	79,0%	63,3%
8387	35	70	60	25	52,1%	64,7%	76,3%	80,1%	64,9%
8388	35	70	60	30	52,5%	64,4%	76,9%	81,2%	66,4%
8389	35	70	60	35	53,0%	64,1%	77,5%	82,2%	67,9%
8390	35	70	60	40	53,4%	63,8%	78,0%	83,2%	69,4%
8391	35	70	60	45	53,9%	63,5%	78,6%	84,2%	70,8%
8392	35	70	60	50	54,3%	63,2%	79,2%	85,1%	72,2%
8393	35	70	60	55	54,7%	62,9%	79,7%	85,9%	73,6%
8394	35	70	60	60	55,2%	62,6%	80,3%	86,7%	74,9%
8395	35	70	60	65	55,6%	62,3%	80,8%	87,5%	76,2%
8396	35	70	60	70	56,0%	62,0%	81,3%	88,2%	77,4%
8397	35	70	60	75	56,5%	61,7%	81,9%	88,9%	78,6%
8398	35	70	60	80	56,9%	61,4%	82,3%	89,6%	79,7%
8399	35	70	65	20	45,5%	59,6%	73,3%	78,0%	62,3%
8400	35	70	65	25	45,9%	59,3%	73,9%	79,1%	63,9%
8401	35	70	65	30	46,4%	59,0%	74,6%	80,2%	65,5%
8402	35	70	65	35	46,8%	58,7%	75,2%	81,3%	67,0%
8403	35	70	65	40	47,3%	58,3%	75,8%	82,3%	68,5%
8404	35	70	65	45	47,7%	58,0%	76,5%	83,3%	70,0%
8405	35	70	65	50	48,1%	57,7%	77,1%	84,3%	71,4%
8406	35	70	65	55	48,6%	57,4%	77,7%	85,2%	72,8%
8407	35	70	65	60	49,0%	57,1%	78,2%	86,0%	74,1%
8408	35	70	65	65	49,5%	56,7%	78,8%	86,8%	75,4%
8409	35	70	65	70	49,9%	56,4%	79,4%	87,6%	76,7%
8410	35	70	65	75	50,3%	56,1%	79,9%	88,3%	77,9%
8411	35	70	65	80	50,8%	55,8%	80,5%	89,0%	79,0%
8412	35	70	70	20	39,5%	53,9%	70,8%	76,9%	61,4%
8413	35	70	70	25	39,9%	53,6%	71,5%	78,1%	63,0%
8414	35	70	70	30	40,3%	53,3%	72,2%	79,3%	64,6%

8415	35	70	70	35	40,7%	53,0%	72,8%	80,4%	66,1%
8416	35	70	70	40	41,2%	52,6%	73,5%	81,4%	67,6%
8417	35	70	70	45	41,6%	52,3%	74,2%	82,5%	69,1%
8418	35	70	70	50	42,0%	52,0%	74,8%	83,4%	70,6%
8419	35	70	70	55	42,5%	51,7%	75,4%	84,4%	72,0%
8420	35	70	70	60	42,9%	51,3%	76,1%	85,3%	73,3%
8421	35	70	70	65	43,3%	51,0%	76,7%	86,1%	74,6%
8422	35	70	70	70	43,8%	50,7%	77,3%	86,9%	75,9%
8423	35	70	70	75	44,2%	50,3%	77,9%	87,7%	77,1%
8424	35	70	70	80	44,6%	50,0%	78,4%	88,4%	78,3%
8425	35	70	75	20	33,8%	48,2%	68,1%	75,8%	60,4%
8426	35	70	75	25	34,2%	47,8%	68,9%	77,0%	62,0%
8427	35	70	75	30	34,5%	47,5%	69,6%	78,2%	63,6%
8428	35	70	75	35	34,9%	47,2%	70,3%	79,4%	65,2%
8429	35	70	75	40	35,3%	46,9%	71,0%	80,5%	66,7%
8430	35	70	75	45	35,8%	46,5%	71,7%	81,6%	68,2%
8431	35	70	75	50	36,2%	46,2%	72,4%	82,6%	69,7%
8432	35	70	75	55	36,6%	45,9%	73,1%	83,5%	71,1%
8433	35	70	75	60	37,0%	45,6%	73,7%	84,5%	72,5%
8434	35	70	75	65	37,4%	45,2%	74,4%	85,4%	73,8%
8435	35	70	75	70	37,8%	44,9%	75,0%	86,2%	75,1%
8436	35	70	75	75	38,2%	44,6%	75,6%	87,0%	76,4%
8437	35	70	75	80	38,6%	44,3%	76,3%	87,8%	77,6%
8438	35	70	80	20	28,5%	42,5%	65,4%	74,7%	59,4%
8439	35	70	80	25	28,8%	42,1%	66,1%	76,0%	61,0%
8440	35	70	80	30	29,2%	41,8%	66,9%	77,2%	62,6%
8441	35	70	80	35	29,6%	41,5%	67,6%	78,4%	64,2%
8442	35	70	80	40	29,9%	41,2%	68,4%	79,5%	65,8%
8443	35	70	80	45	30,3%	40,9%	69,1%	80,6%	67,3%
8444	35	70	80	50	30,7%	40,6%	69,8%	81,7%	68,8%
8445	35	70	80	55	31,1%	40,2%	70,5%	82,7%	70,3%
8446	35	70	80	60	31,4%	39,9%	71,2%	83,7%	71,7%
8447	35	70	80	65	31,8%	39,6%	71,9%	84,6%	73,0%

8448	35	70	80	70	32,2%	39,3%	72,6%	85,5%	74,4%
8449	35	70	80	75	32,6%	39,0%	73,3%	86,3%	75,7%
8450	35	70	80	80	33,0%	38,7%	73,9%	87,1%	76,9%
8451	35	75	20	20	89,4%	92,7%	89,0%	85,0%	68,6%
8452	35	75	20	25	89,5%	92,6%	89,4%	85,9%	70,0%
8453	35	75	20	30	89,7%	92,6%	89,7%	86,7%	71,4%
8454	35	75	20	35	89,9%	92,5%	90,0%	87,5%	72,8%
8455	35	75	20	40	90,0%	92,4%	90,3%	88,2%	74,1%
8456	35	75	20	45	90,2%	92,3%	90,6%	88,9%	75,4%
8457	35	75	20	50	90,3%	92,2%	90,9%	89,6%	76,7%
8458	35	75	20	55	90,5%	92,1%	91,2%	90,2%	77,9%
8459	35	75	20	60	90,6%	92,0%	91,4%	90,8%	79,0%
8460	35	75	20	65	90,8%	91,9%	91,7%	91,4%	80,2%
8461	35	75	20	70	90,9%	91,8%	91,9%	91,9%	81,2%
8462	35	75	20	75	91,1%	91,7%	92,2%	92,4%	82,2%
8463	35	75	20	80	91,2%	91,6%	92,4%	92,9%	83,2%
8464	35	75	25	20	86,8%	91,0%	87,8%	84,3%	67,7%
8465	35	75	25	25	87,0%	90,9%	88,1%	85,2%	69,1%
8466	35	75	25	30	87,2%	90,8%	88,5%	86,0%	70,6%
8467	35	75	25	35	87,4%	90,7%	88,8%	86,8%	72,0%
8468	35	75	25	40	87,6%	90,6%	89,2%	87,6%	73,3%
8469	35	75	25	45	87,8%	90,5%	89,5%	88,3%	74,7%
8470	35	75	25	50	88,0%	90,3%	89,8%	89,0%	75,9%
8471	35	75	25	55	88,2%	90,2%	90,1%	89,7%	77,2%
8472	35	75	25	60	88,3%	90,1%	90,4%	90,3%	78,4%
8473	35	75	25	65	88,5%	90,0%	90,7%	90,9%	79,5%
8474	35	75	25	70	88,7%	89,9%	91,0%	91,4%	80,6%
8475	35	75	25	75	88,9%	89,8%	91,2%	92,0%	81,6%
8476	35	75	25	80	89,0%	89,6%	91,5%	92,5%	82,6%
8477	35	75	30	20	83,7%	88,9%	86,4%	83,4%	66,8%
8478	35	75	30	25	83,9%	88,8%	86,8%	84,4%	68,3%
8479	35	75	30	30	84,2%	88,7%	87,1%	85,3%	69,7%
8480	35	75	30	35	84,4%	88,5%	87,5%	86,1%	71,1%

8481	35	75	30	40	84,6%	88,4%	87,9%	86,9%	72,5%
8482	35	75	30	45	84,9%	88,3%	88,2%	87,7%	73,9%
8483	35	75	30	50	85,1%	88,1%	88,6%	88,4%	75,2%
8484	35	75	30	55	85,3%	88,0%	88,9%	89,1%	76,4%
8485	35	75	30	60	85,5%	87,9%	89,3%	89,7%	77,6%
8486	35	75	30	65	85,8%	87,7%	89,6%	90,4%	78,8%
8487	35	75	30	70	86,0%	87,6%	89,9%	90,9%	79,9%
8488	35	75	30	75	86,2%	87,4%	90,2%	91,5%	81,0%
8489	35	75	30	80	86,4%	87,3%	90,5%	92,0%	82,0%
8490	35	75	35	20	80,1%	86,5%	84,8%	82,6%	65,8%
8491	35	75	35	25	80,3%	86,3%	85,3%	83,5%	67,4%
8492	35	75	35	30	80,6%	86,1%	85,7%	84,5%	68,8%
8493	35	75	35	35	80,9%	86,0%	86,1%	85,4%	70,3%
8494	35	75	35	40	81,2%	85,8%	86,5%	86,2%	71,7%
8495	35	75	35	45	81,4%	85,7%	86,9%	87,0%	73,1%
8496	35	75	35	50	81,7%	85,5%	87,3%	87,8%	74,4%
8497	35	75	35	55	82,0%	85,3%	87,7%	88,5%	75,7%
8498	35	75	35	60	82,2%	85,2%	88,0%	89,2%	76,9%
8499	35	75	35	65	82,5%	85,0%	88,4%	89,8%	78,1%
8500	35	75	35	70	82,7%	84,8%	88,7%	90,4%	79,3%
8501	35	75	35	75	83,0%	84,7%	89,0%	91,0%	80,4%
8502	35	75	35	80	83,2%	84,5%	89,4%	91,6%	81,4%
8503	35	75	40	20	75,8%	83,5%	83,2%	81,7%	64,9%
8504	35	75	40	25	76,1%	83,3%	83,6%	82,7%	66,4%
8505	35	75	40	30	76,5%	83,1%	84,1%	83,7%	68,0%
8506	35	75	40	35	76,8%	83,0%	84,5%	84,6%	69,4%
8507	35	75	40	40	77,1%	82,8%	85,0%	85,5%	70,9%
8508	35	75	40	45	77,4%	82,6%	85,4%	86,3%	72,3%
8509	35	75	40	50	77,7%	82,4%	85,8%	87,1%	73,6%
8510	35	75	40	55	78,0%	82,2%	86,2%	87,8%	74,9%
8511	35	75	40	60	78,3%	82,0%	86,6%	88,6%	76,2%
8512	35	75	40	65	78,6%	81,8%	87,0%	89,2%	77,4%
8513	35	75	40	70	78,9%	81,6%	87,4%	89,9%	78,6%

8514	35	75	40	75	79,2%	81,4%	87,8%	90,5%	79,7%
8515	35	75	40	80	79,5%	81,2%	88,1%	91,1%	80,8%
8516	35	75	45	20	71,0%	80,1%	81,4%	80,7%	64,0%
8517	35	75	45	25	71,4%	79,9%	81,9%	81,8%	65,5%
8518	35	75	45	30	71,7%	79,7%	82,4%	82,8%	67,0%
8519	35	75	45	35	72,1%	79,4%	82,9%	83,8%	68,5%
8520	35	75	45	40	72,4%	79,2%	83,3%	84,7%	70,0%
8521	35	75	45	45	72,8%	79,0%	83,8%	85,6%	71,4%
8522	35	75	45	50	73,1%	78,8%	84,3%	86,4%	72,8%
8523	35	75	45	55	73,5%	78,6%	84,7%	87,2%	74,1%
8524	35	75	45	60	73,8%	78,4%	85,1%	87,9%	75,4%
8525	35	75	45	65	74,2%	78,1%	85,6%	88,6%	76,7%
8526	35	75	45	70	74,5%	77,9%	86,0%	89,3%	77,9%
8527	35	75	45	75	74,8%	77,7%	86,4%	90,0%	79,0%
8528	35	75	45	80	75,2%	77,5%	86,8%	90,6%	80,1%
8529	35	75	50	20	65,7%	76,1%	79,4%	79,8%	63,0%
8530	35	75	50	25	66,1%	75,9%	80,0%	80,9%	64,6%
8531	35	75	50	30	66,5%	75,7%	80,5%	81,9%	66,1%
8532	35	75	50	35	66,9%	75,4%	81,0%	82,9%	67,6%
8533	35	75	50	40	67,3%	75,2%	81,5%	83,9%	69,1%
8534	35	75	50	45	67,6%	74,9%	82,0%	84,8%	70,6%
8535	35	75	50	50	68,0%	74,7%	82,5%	85,7%	72,0%
8536	35	75	50	55	68,4%	74,4%	83,0%	86,5%	73,3%
8537	35	75	50	60	68,8%	74,2%	83,5%	87,3%	74,7%
8538	35	75	50	65	69,2%	73,9%	84,0%	88,0%	75,9%
8539	35	75	50	70	69,5%	73,7%	84,4%	88,7%	77,2%
8540	35	75	50	75	69,9%	73,4%	84,9%	89,4%	78,3%
8541	35	75	50	80	70,3%	73,2%	85,3%	90,0%	79,5%
8542	35	75	55	20	59,9%	71,7%	77,3%	78,8%	62,0%
8543	35	75	55	25	60,3%	71,4%	77,9%	79,9%	63,6%
8544	35	75	55	30	60,8%	71,2%	78,5%	81,0%	65,2%
8545	35	75	55	35	61,2%	70,9%	79,0%	82,0%	66,7%
8546	35	75	55	40	61,6%	70,6%	79,6%	83,0%	68,2%

8547	35	75	55	45	62,0%	70,4%	80,1%	84,0%	69,7%
8548	35	75	55	50	62,4%	70,1%	80,7%	84,9%	71,1%
8549	35	75	55	55	62,8%	69,8%	81,2%	85,7%	72,5%
8550	35	75	55	60	63,3%	69,5%	81,7%	86,6%	73,9%
8551	35	75	55	65	63,7%	69,2%	82,2%	87,4%	75,2%
8552	35	75	55	70	64,1%	69,0%	82,7%	88,1%	76,4%
8553	35	75	55	75	64,5%	68,7%	83,2%	88,8%	77,6%
8554	35	75	55	80	64,9%	68,4%	83,7%	89,5%	78,8%
8555	35	75	60	20	53,9%	66,8%	75,0%	77,7%	61,1%
8556	35	75	60	25	54,3%	66,5%	75,7%	78,9%	62,7%
8557	35	75	60	30	54,7%	66,2%	76,3%	80,0%	64,3%
8558	35	75	60	35	55,2%	65,9%	76,9%	81,1%	65,8%
8559	35	75	60	40	55,6%	65,6%	77,5%	82,2%	67,3%
8560	35	75	60	45	56,1%	65,3%	78,1%	83,1%	68,8%
8561	35	75	60	50	56,5%	65,0%	78,7%	84,1%	70,3%
8562	35	75	60	55	56,9%	64,7%	79,2%	85,0%	71,7%
8563	35	75	60	60	57,4%	64,4%	79,8%	85,8%	73,1%
8564	35	75	60	65	57,8%	64,1%	80,3%	86,7%	74,4%
8565	35	75	60	70	58,2%	63,8%	80,9%	87,4%	75,7%
8566	35	75	60	75	58,6%	63,5%	81,4%	88,2%	76,9%
8567	35	75	60	80	59,1%	63,2%	81,9%	88,9%	78,1%
8568	35	75	65	20	47,7%	61,5%	72,6%	76,7%	60,1%
8569	35	75	65	25	48,1%	61,2%	73,3%	77,9%	61,7%
8570	35	75	65	30	48,6%	60,9%	74,0%	79,0%	63,3%
8571	35	75	65	35	49,0%	60,5%	74,6%	80,2%	64,9%
8572	35	75	65	40	49,5%	60,2%	75,3%	81,2%	66,4%
8573	35	75	65	45	49,9%	59,9%	75,9%	82,3%	67,9%
8574	35	75	65	50	50,4%	59,6%	76,5%	83,3%	69,4%
8575	35	75	65	55	50,8%	59,3%	77,1%	84,2%	70,8%
8576	35	75	65	60	51,2%	59,0%	77,7%	85,1%	72,2%
8577	35	75	65	65	51,7%	58,7%	78,3%	85,9%	73,6%
8578	35	75	65	70	52,1%	58,3%	78,9%	86,8%	74,9%
8579	35	75	65	75	52,6%	58,0%	79,4%	87,5%	76,2%

8580	35	75	65	80	53,0%	57,7%	80,0%	88,3%	77,4%
8581	35	75	70	20	41,6%	55,9%	70,1%	75,6%	59,1%
8582	35	75	70	25	42,0%	55,6%	70,8%	76,8%	60,7%
8583	35	75	70	30	42,5%	55,2%	71,5%	78,0%	62,3%
8584	35	75	70	35	42,9%	54,9%	72,2%	79,2%	63,9%
8585	35	75	70	40	43,3%	54,6%	72,9%	80,3%	65,5%
8586	35	75	70	45	43,8%	54,3%	73,5%	81,4%	67,0%
8587	35	75	70	50	44,2%	53,9%	74,2%	82,4%	68,5%
8588	35	75	70	55	44,6%	53,6%	74,8%	83,4%	70,0%
8589	35	75	70	60	45,1%	53,3%	75,5%	84,3%	71,4%
8590	35	75	70	65	45,5%	53,0%	76,1%	85,2%	72,8%
8591	35	75	70	70	46,0%	52,6%	76,7%	86,0%	74,1%
8592	35	75	70	75	46,4%	52,3%	77,3%	86,8%	75,4%
8593	35	75	70	80	46,8%	52,0%	77,9%	87,6%	76,7%
8594	35	75	75	20	35,8%	50,1%	67,4%	74,4%	58,1%
8595	35	75	75	25	36,2%	49,8%	68,2%	75,7%	59,7%
8596	35	75	75	30	36,6%	49,5%	68,9%	77,0%	61,4%
8597	35	75	75	35	37,0%	49,2%	69,6%	78,2%	63,0%
8598	35	75	75	40	37,4%	48,8%	70,4%	79,3%	64,6%
8599	35	75	75	45	37,8%	48,5%	71,1%	80,4%	66,1%
8600	35	75	75	50	38,2%	48,2%	71,7%	81,5%	67,6%
8601	35	75	75	55	38,6%	47,9%	72,4%	82,5%	69,1%
8602	35	75	75	60	39,1%	47,5%	73,1%	83,5%	70,6%
8603	35	75	75	65	39,5%	47,2%	73,8%	84,4%	72,0%
8604	35	75	75	70	39,9%	46,9%	74,4%	85,3%	73,3%
8605	35	75	75	75	40,3%	46,6%	75,1%	86,1%	74,6%
8606	35	75	75	80	40,8%	46,2%	75,7%	86,9%	75,9%
8607	35	75	80	20	30,3%	44,4%	64,7%	73,2%	57,1%
8608	35	75	80	25	30,7%	44,1%	65,4%	74,6%	58,7%
8609	35	75	80	30	31,1%	43,7%	66,2%	75,9%	60,4%
8610	35	75	80	35	31,4%	43,4%	66,9%	77,1%	62,0%
8611	35	75	80	40	31,8%	43,1%	67,7%	78,3%	63,6%
8612	35	75	80	45	32,2%	42,8%	68,4%	79,4%	65,2%

8613	35	75	80	50	32,6%	42,5%	69,2%	80,5%	66,7%
8614	35	75	80	55	33,0%	42,1%	69,9%	81,6%	68,2%
8615	35	75	80	60	33,4%	41,8%	70,6%	82,6%	69,7%
8616	35	75	80	65	33,8%	41,5%	71,3%	83,6%	71,1%
8617	35	75	80	70	34,2%	41,2%	72,0%	84,5%	72,5%
8618	35	75	80	75	34,6%	40,9%	72,7%	85,4%	73,9%
8619	35	75	80	80	35,0%	40,6%	73,3%	86,2%	75,2%
8620	35	80	20	20	90,2%	93,2%	88,7%	84,1%	66,5%
8621	35	80	20	25	90,4%	93,2%	89,1%	85,0%	68,0%
8622	35	80	20	30	90,5%	93,1%	89,4%	85,8%	69,4%
8623	35	80	20	35	90,7%	93,0%	89,7%	86,7%	70,9%
8624	35	80	20	40	90,8%	92,9%	90,0%	87,4%	72,3%
8625	35	80	20	45	90,9%	92,8%	90,3%	88,2%	73,6%
8626	35	80	20	50	91,1%	92,7%	90,6%	88,9%	74,9%
8627	35	80	20	55	91,2%	92,6%	90,9%	89,5%	76,2%
8628	35	80	20	60	91,4%	92,6%	91,2%	90,2%	77,4%
8629	35	80	20	65	91,5%	92,5%	91,4%	90,8%	78,6%
8630	35	80	20	70	91,6%	92,4%	91,7%	91,3%	79,7%
8631	35	80	20	75	91,8%	92,3%	92,0%	91,9%	80,8%
8632	35	80	20	80	91,9%	92,2%	92,2%	92,4%	81,8%
8633	35	80	25	20	87,8%	91,6%	87,4%	83,2%	65,5%
8634	35	80	25	25	88,0%	91,5%	87,8%	84,2%	67,1%
8635	35	80	25	30	88,2%	91,4%	88,1%	85,1%	68,6%
8636	35	80	25	35	88,3%	91,3%	88,5%	85,9%	70,0%
8637	35	80	25	40	88,5%	91,2%	88,8%	86,8%	71,4%
8638	35	80	25	45	88,7%	91,1%	89,2%	87,5%	72,8%
8639	35	80	25	50	88,9%	91,0%	89,5%	88,3%	74,2%
8640	35	80	25	55	89,0%	90,9%	89,8%	89,0%	75,4%
8641	35	80	25	60	89,2%	90,8%	90,1%	89,6%	76,7%
8642	35	80	25	65	89,4%	90,7%	90,4%	90,2%	77,9%
8643	35	80	25	70	89,6%	90,6%	90,7%	90,8%	79,0%
8644	35	80	25	75	89,7%	90,5%	91,0%	91,4%	80,2%
8645	35	80	25	80	89,9%	90,4%	91,3%	91,9%	81,2%

8646	35	80	30	20	84,9%	89,7%	86,0%	82,4%	64,6%
8647	35	80	30	25	85,1%	89,6%	86,4%	83,4%	66,2%
8648	35	80	30	30	85,3%	89,4%	86,8%	84,3%	67,7%
8649	35	80	30	35	85,5%	89,3%	87,2%	85,2%	69,2%
8650	35	80	30	40	85,8%	89,2%	87,5%	86,0%	70,6%
8651	35	80	30	45	86,0%	89,1%	87,9%	86,8%	72,0%
8652	35	80	30	50	86,2%	88,9%	88,3%	87,6%	73,4%
8653	35	80	30	55	86,4%	88,8%	88,6%	88,3%	74,7%
8654	35	80	30	60	86,6%	88,7%	89,0%	89,0%	75,9%
8655	35	80	30	65	86,8%	88,5%	89,3%	89,7%	77,2%
8656	35	80	30	70	87,0%	88,4%	89,6%	90,3%	78,4%
8657	35	80	30	75	87,2%	88,3%	89,9%	90,9%	79,5%
8658	35	80	30	80	87,4%	88,1%	90,2%	91,5%	80,6%
8659	35	80	35	20	81,4%	87,3%	84,4%	81,5%	63,7%
8660	35	80	35	25	81,7%	87,2%	84,9%	82,5%	65,2%
8661	35	80	35	30	82,0%	87,1%	85,3%	83,5%	66,8%
8662	35	80	35	35	82,2%	86,9%	85,7%	84,4%	68,3%
8663	35	80	35	40	82,5%	86,8%	86,1%	85,3%	69,7%
8664	35	80	35	45	82,7%	86,6%	86,5%	86,1%	71,2%
8665	35	80	35	50	83,0%	86,5%	86,9%	86,9%	72,5%
8666	35	80	35	55	83,2%	86,3%	87,3%	87,7%	73,9%
8667	35	80	35	60	83,5%	86,1%	87,7%	88,4%	75,2%
8668	35	80	35	65	83,7%	86,0%	88,0%	89,1%	76,4%
8669	35	80	35	70	84,0%	85,8%	88,4%	89,8%	77,7%
8670	35	80	35	75	84,2%	85,7%	88,7%	90,4%	78,8%
8671	35	80	35	80	84,4%	85,5%	89,1%	91,0%	79,9%
8672	35	80	40	20	77,4%	84,6%	82,7%	80,5%	62,7%
8673	35	80	40	25	77,7%	84,4%	83,2%	81,6%	64,3%
8674	35	80	40	30	78,0%	84,2%	83,7%	82,6%	65,8%
8675	35	80	40	35	78,3%	84,0%	84,1%	83,6%	67,4%
8676	35	80	40	40	78,6%	83,9%	84,6%	84,5%	68,9%
8677	35	80	40	45	78,9%	83,7%	85,0%	85,4%	70,3%
8678	35	80	40	50	79,2%	83,5%	85,4%	86,2%	71,7%

8679	35	80	40	55	79,5%	83,3%	85,9%	87,0%	73,1%
8680	35	80	40	60	79,8%	83,2%	86,3%	87,8%	74,4%
8681	35	80	40	65	80,1%	83,0%	86,7%	88,5%	75,7%
8682	35	80	40	70	80,3%	82,8%	87,1%	89,2%	76,9%
8683	35	80	40	75	80,6%	82,6%	87,4%	89,8%	78,1%
8684	35	80	40	80	80,9%	82,4%	87,8%	90,4%	79,3%
8685	35	80	45	20	72,8%	81,3%	80,9%	79,6%	61,7%
8686	35	80	45	25	73,2%	81,1%	81,4%	80,7%	63,3%
8687	35	80	45	30	73,5%	80,9%	81,9%	81,7%	64,9%
8688	35	80	45	35	73,8%	80,7%	82,4%	82,7%	66,5%
8689	35	80	45	40	74,2%	80,5%	82,9%	83,7%	68,0%
8690	35	80	45	45	74,5%	80,3%	83,4%	84,6%	69,4%
8691	35	80	45	50	74,8%	80,1%	83,8%	85,5%	70,9%
8692	35	80	45	55	75,2%	79,9%	84,3%	86,3%	72,3%
8693	35	80	45	60	75,5%	79,7%	84,7%	87,1%	73,6%
8694	35	80	45	65	75,8%	79,5%	85,2%	87,9%	74,9%
8695	35	80	45	70	76,2%	79,2%	85,6%	88,6%	76,2%
8696	35	80	45	75	76,5%	79,0%	86,0%	89,3%	77,4%
8697	35	80	45	80	76,8%	78,8%	86,4%	89,9%	78,6%
8698	35	80	50	20	67,7%	77,5%	78,9%	78,6%	60,7%
8699	35	80	50	25	68,0%	77,3%	79,4%	79,7%	62,4%
8700	35	80	50	30	68,4%	77,1%	80,0%	80,8%	64,0%
8701	35	80	50	35	68,8%	76,9%	80,5%	81,8%	65,5%
8702	35	80	50	40	69,2%	76,6%	81,1%	82,8%	67,1%
8703	35	80	50	45	69,5%	76,4%	81,6%	83,8%	68,6%
8704	35	80	50	50	69,9%	76,1%	82,1%	84,7%	70,0%
8705	35	80	50	55	70,3%	75,9%	82,6%	85,6%	71,4%
8706	35	80	50	60	70,7%	75,7%	83,1%	86,4%	72,8%
8707	35	80	50	65	71,0%	75,4%	83,5%	87,2%	74,1%
8708	35	80	50	70	71,4%	75,2%	84,0%	88,0%	75,4%
8709	35	80	50	75	71,7%	74,9%	84,4%	88,7%	76,7%
8710	35	80	50	80	72,1%	74,7%	84,9%	89,3%	77,9%
8711	35	80	55	20	62,0%	73,3%	76,7%	77,5%	59,8%

8712	35	80	55	25	62,4%	73,0%	77,3%	78,7%	61,4%
8713	35	80	55	30	62,9%	72,8%	77,9%	79,8%	63,0%
8714	35	80	55	35	63,3%	72,5%	78,5%	80,9%	64,6%
8715	35	80	55	40	63,7%	72,2%	79,1%	82,0%	66,1%
8716	35	80	55	45	64,1%	72,0%	79,6%	83,0%	67,7%
8717	35	80	55	50	64,5%	71,7%	80,2%	83,9%	69,1%
8718	35	80	55	55	64,9%	71,4%	80,7%	84,8%	70,6%
8719	35	80	55	60	65,3%	71,2%	81,2%	85,7%	72,0%
8720	35	80	55	65	65,7%	70,9%	81,7%	86,5%	73,3%
8721	35	80	55	70	66,1%	70,6%	82,2%	87,3%	74,7%
8722	35	80	55	75	66,5%	70,4%	82,7%	88,0%	75,9%
8723	35	80	55	80	66,9%	70,1%	83,2%	88,7%	77,2%
8724	35	80	60	20	56,1%	68,5%	74,4%	76,4%	58,8%
8725	35	80	60	25	56,5%	68,2%	75,1%	77,7%	60,4%
8726	35	80	60	30	56,9%	67,9%	75,7%	78,8%	62,0%
8727	35	80	60	35	57,4%	67,7%	76,3%	80,0%	63,6%
8728	35	80	60	40	57,8%	67,4%	76,9%	81,0%	65,2%
8729	35	80	60	45	58,2%	67,1%	77,5%	82,1%	66,7%
8730	35	80	60	50	58,7%	66,8%	78,1%	83,1%	68,2%
8731	35	80	60	55	59,1%	66,5%	78,7%	84,0%	69,7%
8732	35	80	60	60	59,5%	66,2%	79,3%	84,9%	71,1%
8733	35	80	60	65	59,9%	65,9%	79,8%	85,8%	72,5%
8734	35	80	60	70	60,4%	65,6%	80,4%	86,6%	73,9%
8735	35	80	60	75	60,8%	65,3%	80,9%	87,4%	75,2%
8736	35	80	60	80	61,2%	65,0%	81,4%	88,1%	76,4%
8737	35	80	65	20	49,9%	63,3%	72,0%	75,3%	57,8%
8738	35	80	65	25	50,4%	63,0%	72,7%	76,6%	59,4%
8739	35	80	65	30	50,8%	62,7%	73,4%	77,8%	61,1%
8740	35	80	65	35	51,2%	62,4%	74,0%	79,0%	62,7%
8741	35	80	65	40	51,7%	62,1%	74,7%	80,1%	64,3%
8742	35	80	65	45	52,1%	61,8%	75,3%	81,2%	65,8%
8743	35	80	65	50	52,6%	61,5%	75,9%	82,2%	67,3%
8744	35	80	65	55	53,0%	61,2%	76,5%	83,2%	68,8%

8745	35	80	65	60	53,4%	60,9%	77,1%	84,1%	70,3%
8746	35	80	65	65	53,9%	60,6%	77,7%	85,0%	71,7%
8747	35	80	65	70	54,3%	60,2%	78,3%	85,9%	73,1%
8748	35	80	65	75	54,8%	59,9%	78,9%	86,7%	74,4%
8749	35	80	65	80	55,2%	59,6%	79,5%	87,5%	75,7%
8750	35	80	70	20	43,8%	57,8%	69,4%	74,2%	56,8%
8751	35	80	70	25	44,2%	57,5%	70,2%	75,5%	58,4%
8752	35	80	70	30	44,7%	57,2%	70,9%	76,7%	60,1%
8753	35	80	70	35	45,1%	56,9%	71,6%	77,9%	61,7%
8754	35	80	70	40	45,5%	56,5%	72,2%	79,1%	63,3%
8755	35	80	70	45	46,0%	56,2%	72,9%	80,2%	64,9%
8756	35	80	70	50	46,4%	55,9%	73,6%	81,3%	66,4%
8757	35	80	70	55	46,8%	55,6%	74,2%	82,3%	67,9%
8758	35	80	70	60	47,3%	55,2%	74,9%	83,3%	69,4%
8759	35	80	70	65	47,7%	54,9%	75,5%	84,2%	70,9%
8760	35	80	70	70	48,2%	54,6%	76,1%	85,1%	72,2%
8761	35	80	70	75	48,6%	54,3%	76,7%	86,0%	73,6%
8762	35	80	70	80	49,0%	54,0%	77,4%	86,8%	74,9%
8763	35	80	75	20	37,8%	52,1%	66,7%	73,0%	55,7%
8764	35	80	75	25	38,2%	51,8%	67,5%	74,3%	57,4%
8765	35	80	75	30	38,7%	51,5%	68,2%	75,6%	59,1%
8766	35	80	75	35	39,1%	51,1%	69,0%	76,9%	60,7%
8767	35	80	75	40	39,5%	50,8%	69,7%	78,1%	62,4%
8768	35	80	75	45	39,9%	50,5%	70,4%	79,2%	63,9%
8769	35	80	75	50	40,3%	50,1%	71,1%	80,3%	65,5%
8770	35	80	75	55	40,8%	49,8%	71,8%	81,4%	67,0%
8771	35	80	75	60	41,2%	49,5%	72,5%	82,4%	68,5%
8772	35	80	75	65	41,6%	49,2%	73,1%	83,4%	70,0%
8773	35	80	75	70	42,1%	48,8%	73,8%	84,3%	71,4%
8774	35	80	75	75	42,5%	48,5%	74,5%	85,2%	72,8%
8775	35	80	75	80	42,9%	48,2%	75,1%	86,1%	74,1%
8776	35	80	80	20	32,2%	46,3%	63,9%	71,8%	54,7%
8777	35	80	80	25	32,6%	46,0%	64,7%	73,1%	56,4%

8778	35	80	80	30	33,0%	45,7%	65,5%	74,5%	58,1%
8779	35	80	80	35	33,4%	45,4%	66,2%	75,8%	59,7%
8780	35	80	80	40	33,8%	45,0%	67,0%	77,0%	61,4%
8781	35	80	80	45	34,2%	44,7%	67,7%	78,2%	63,0%
8782	35	80	80	50	34,6%	44,4%	68,5%	79,4%	64,6%
8783	35	80	80	55	35,0%	44,1%	69,2%	80,5%	66,1%
8784	35	80	80	60	35,4%	43,8%	69,9%	81,5%	67,6%
8785	35	80	80	65	35,8%	43,4%	70,6%	82,5%	69,1%
8786	35	80	80	70	36,2%	43,1%	71,3%	83,5%	70,6%
8787	35	80	80	75	36,6%	42,8%	72,0%	84,4%	72,0%
8788	35	80	80	80	37,0%	42,5%	72,7%	85,3%	73,3%
8789	40	20	20	20	80,2%	88,2%	94,6%	95,1%	89,4%
8790	40	20	20	25	80,5%	88,1%	94,8%	95,4%	90,0%
8791	40	20	20	30	80,8%	88,0%	94,9%	95,7%	90,6%
8792	40	20	20	35	81,0%	87,8%	95,1%	95,9%	91,2%
8793	40	20	20	40	81,3%	87,7%	95,3%	96,2%	91,7%
8794	40	20	20	45	81,6%	87,5%	95,4%	96,4%	92,2%
8795	40	20	20	50	81,8%	87,4%	95,6%	96,7%	92,7%
8796	40	20	20	55	82,1%	87,2%	95,7%	96,9%	93,2%
8797	40	20	20	60	82,4%	87,1%	95,8%	97,1%	93,6%
8798	40	20	20	65	82,6%	86,9%	96,0%	97,3%	94,0%
8799	40	20	20	70	82,9%	86,8%	96,1%	97,5%	94,4%
8800	40	20	20	75	83,1%	86,6%	96,2%	97,6%	94,7%
8801	40	20	20	80	83,4%	86,5%	96,3%	97,8%	95,0%
8802	40	20	25	20	76,0%	85,6%	93,9%	94,8%	89,0%
8803	40	20	25	25	76,3%	85,4%	94,1%	95,1%	89,6%
8804	40	20	25	30	76,6%	85,3%	94,3%	95,4%	90,3%
8805	40	20	25	35	77,0%	85,1%	94,5%	95,7%	90,8%
8806	40	20	25	40	77,3%	85,0%	94,7%	96,0%	91,4%
8807	40	20	25	45	77,6%	84,8%	94,8%	96,2%	91,9%
8808	40	20	25	50	77,9%	84,6%	95,0%	96,5%	92,4%
8809	40	20	25	55	78,2%	84,4%	95,2%	96,7%	92,9%
8810	40	20	25	60	78,5%	84,3%	95,3%	96,9%	93,3%

8811	40	20	25	65	78,8%	84,1%	95,5%	97,1%	93,7%
8812	40	20	25	70	79,1%	83,9%	95,6%	97,3%	94,1%
8813	40	20	25	75	79,4%	83,7%	95,7%	97,5%	94,5%
8814	40	20	25	80	79,6%	83,6%	95,9%	97,6%	94,8%
8815	40	20	30	20	71,2%	82,5%	93,2%	94,4%	88,6%
8816	40	20	30	25	71,6%	82,3%	93,4%	94,8%	89,3%
8817	40	20	30	30	71,9%	82,1%	93,6%	95,1%	89,9%
8818	40	20	30	35	72,3%	81,9%	93,8%	95,4%	90,5%
8819	40	20	30	40	72,6%	81,8%	94,0%	95,7%	91,1%
8820	40	20	30	45	73,0%	81,6%	94,2%	96,0%	91,6%
8821	40	20	30	50	73,3%	81,4%	94,4%	96,3%	92,1%
8822	40	20	30	55	73,7%	81,2%	94,5%	96,5%	92,6%
8823	40	20	30	60	74,0%	81,0%	94,7%	96,7%	93,1%
8824	40	20	30	65	74,4%	80,8%	94,9%	96,9%	93,5%
8825	40	20	30	70	74,7%	80,6%	95,0%	97,1%	93,9%
8826	40	20	30	75	75,0%	80,3%	95,2%	97,3%	94,3%
8827	40	20	30	80	75,4%	80,1%	95,4%	97,5%	94,6%
8828	40	20	35	20	65,9%	78,9%	92,3%	94,1%	88,2%
8829	40	20	35	25	66,3%	78,7%	92,6%	94,5%	88,8%
8830	40	20	35	30	66,7%	78,5%	92,8%	94,8%	89,5%
8831	40	20	35	35	67,1%	78,3%	93,0%	95,2%	90,1%
8832	40	20	35	40	67,5%	78,1%	93,3%	95,5%	90,7%
8833	40	20	35	45	67,9%	77,8%	93,5%	95,8%	91,3%
8834	40	20	35	50	68,2%	77,6%	93,7%	96,0%	91,8%
8835	40	20	35	55	68,6%	77,4%	93,9%	96,3%	92,3%
8836	40	20	35	60	69,0%	77,1%	94,1%	96,5%	92,8%
8837	40	20	35	65	69,4%	76,9%	94,2%	96,8%	93,2%
8838	40	20	35	70	69,7%	76,7%	94,4%	97,0%	93,7%
8839	40	20	35	75	70,1%	76,4%	94,6%	97,2%	94,0%
8840	40	20	35	80	70,5%	76,2%	94,8%	97,3%	94,4%
8841	40	20	40	20	60,2%	74,8%	91,4%	93,8%	87,7%
8842	40	20	40	25	60,6%	74,6%	91,7%	94,2%	88,4%
8843	40	20	40	30	61,0%	74,3%	91,9%	94,5%	89,1%

8844	40	20	40	35	61,4%	74,1%	92,2%	94,9%	89,8%
8845	40	20	40	40	61,8%	73,8%	92,4%	95,2%	90,4%
8846	40	20	40	45	62,3%	73,6%	92,7%	95,5%	91,0%
8847	40	20	40	50	62,7%	73,3%	92,9%	95,8%	91,5%
8848	40	20	40	55	63,1%	73,1%	93,1%	96,1%	92,0%
8849	40	20	40	60	63,5%	72,8%	93,3%	96,3%	92,5%
8850	40	20	40	65	63,9%	72,6%	93,5%	96,6%	93,0%
8851	40	20	40	70	64,3%	72,3%	93,7%	96,8%	93,4%
8852	40	20	40	75	64,7%	72,0%	93,9%	97,0%	93,8%
8853	40	20	40	80	65,1%	71,8%	94,1%	97,2%	94,2%
8854	40	20	45	20	54,1%	70,3%	90,4%	93,4%	87,3%
8855	40	20	45	25	54,6%	70,0%	90,7%	93,8%	88,0%
8856	40	20	45	30	55,0%	69,7%	91,0%	94,2%	88,7%
8857	40	20	45	35	55,4%	69,4%	91,2%	94,6%	89,4%
8858	40	20	45	40	55,9%	69,1%	91,5%	94,9%	90,0%
8859	40	20	45	45	56,3%	68,9%	91,8%	95,2%	90,6%
8860	40	20	45	50	56,7%	68,6%	92,0%	95,5%	91,2%
8861	40	20	45	55	57,2%	68,3%	92,3%	95,8%	91,7%
8862	40	20	45	60	57,6%	68,0%	92,5%	96,1%	92,2%
8863	40	20	45	65	58,0%	67,7%	92,7%	96,3%	92,7%
8864	40	20	45	70	58,5%	67,4%	93,0%	96,6%	93,1%
8865	40	20	45	75	58,9%	67,2%	93,2%	96,8%	93,6%
8866	40	20	45	80	59,3%	66,9%	93,4%	97,0%	94,0%
8867	40	20	50	20	48,0%	65,2%	89,3%	93,0%	86,8%
8868	40	20	50	25	48,4%	64,9%	89,6%	93,5%	87,6%
8869	40	20	50	30	48,8%	64,6%	89,9%	93,9%	88,3%
8870	40	20	50	35	49,3%	64,3%	90,2%	94,3%	89,0%
8871	40	20	50	40	49,7%	64,0%	90,5%	94,6%	89,6%
8872	40	20	50	45	50,2%	63,7%	90,8%	95,0%	90,3%
8873	40	20	50	50	50,6%	63,4%	91,1%	95,3%	90,8%
8874	40	20	50	55	51,0%	63,1%	91,3%	95,6%	91,4%
8875	40	20	50	60	51,5%	62,8%	91,6%	95,9%	91,9%
8876	40	20	50	65	51,9%	62,5%	91,9%	96,1%	92,4%

8877	40	20	50	70	52,4%	62,2%	92,1%	96,4%	92,9%
8878	40	20	50	75	52,8%	61,9%	92,4%	96,6%	93,3%
8879	40	20	50	80	53,2%	61,6%	92,6%	96,8%	93,7%
8880	40	20	55	20	41,9%	59,8%	88,0%	92,6%	86,3%
8881	40	20	55	25	42,3%	59,5%	88,4%	93,1%	87,1%
8882	40	20	55	30	42,7%	59,2%	88,7%	93,5%	87,9%
8883	40	20	55	35	43,2%	58,9%	89,0%	93,9%	88,6%
8884	40	20	55	40	43,6%	58,5%	89,4%	94,3%	89,2%
8885	40	20	55	45	44,0%	58,2%	89,7%	94,7%	89,9%
8886	40	20	55	50	44,5%	57,9%	90,0%	95,0%	90,5%
8887	40	20	55	55	44,9%	57,6%	90,3%	95,3%	91,1%
8888	40	20	55	60	45,3%	57,3%	90,6%	95,6%	91,6%
8889	40	20	55	65	45,8%	56,9%	90,9%	95,9%	92,1%
8890	40	20	55	70	46,2%	56,6%	91,2%	96,2%	92,6%
8891	40	20	55	75	46,6%	56,3%	91,4%	96,4%	93,1%
8892	40	20	55	80	47,1%	56,0%	91,7%	96,6%	93,5%
8893	40	20	60	20	36,0%	54,2%	86,6%	92,2%	85,8%
8894	40	20	60	25	36,4%	53,8%	87,0%	92,7%	86,6%
8895	40	20	60	30	36,8%	53,5%	87,4%	93,1%	87,4%
8896	40	20	60	35	37,2%	53,2%	87,8%	93,6%	88,1%
8897	40	20	60	40	37,6%	52,9%	88,1%	94,0%	88,8%
8898	40	20	60	45	38,1%	52,5%	88,5%	94,3%	89,5%
8899	40	20	60	50	38,5%	52,2%	88,8%	94,7%	90,1%
8900	40	20	60	55	38,9%	51,9%	89,2%	95,0%	90,7%
8901	40	20	60	60	39,3%	51,5%	89,5%	95,3%	91,3%
8902	40	20	60	65	39,7%	51,2%	89,8%	95,6%	91,8%
8903	40	20	60	70	40,2%	50,9%	90,1%	95,9%	92,3%
8904	40	20	60	75	40,6%	50,6%	90,4%	96,2%	92,8%
8905	40	20	60	80	41,0%	50,2%	90,7%	96,4%	93,2%
8906	40	20	65	20	30,5%	48,4%	85,1%	91,7%	85,3%
8907	40	20	65	25	30,9%	48,1%	85,6%	92,2%	86,2%
8908	40	20	65	30	31,3%	47,7%	86,0%	92,7%	86,9%
8909	40	20	65	35	31,7%	47,4%	86,4%	93,2%	87,7%

8910	40	20	65	40	32,0%	47,1%	86,8%	93,6%	88,4%
8911	40	20	65	45	32,4%	46,8%	87,2%	94,0%	89,1%
8912	40	20	65	50	32,8%	46,4%	87,5%	94,4%	89,8%
8913	40	20	65	55	33,2%	46,1%	87,9%	94,7%	90,4%
8914	40	20	65	60	33,6%	45,8%	88,3%	95,1%	90,9%
8915	40	20	65	65	34,0%	45,5%	88,6%	95,4%	91,5%
8916	40	20	65	70	34,4%	45,1%	88,9%	95,7%	92,0%
8917	40	20	65	75	34,8%	44,8%	89,3%	96,0%	92,5%
8918	40	20	65	80	35,2%	44,5%	89,6%	96,2%	93,0%
8919	40	20	70	20	25,6%	42,7%	83,5%	91,3%	84,8%
8920	40	20	70	25	25,9%	42,3%	84,0%	91,8%	85,7%
8921	40	20	70	30	26,2%	42,0%	84,4%	92,3%	86,5%
8922	40	20	70	35	26,6%	41,7%	84,8%	92,8%	87,3%
8923	40	20	70	40	26,9%	41,4%	85,3%	93,2%	88,0%
8924	40	20	70	45	27,3%	41,1%	85,7%	93,7%	88,7%
8925	40	20	70	50	27,6%	40,8%	86,1%	94,0%	89,4%
8926	40	20	70	55	28,0%	40,4%	86,5%	94,4%	90,0%
8927	40	20	70	60	28,3%	40,1%	86,9%	94,8%	90,6%
8928	40	20	70	65	28,7%	39,8%	87,3%	95,1%	91,2%
8929	40	20	70	70	29,0%	39,5%	87,7%	95,4%	91,7%
8930	40	20	70	75	29,4%	39,2%	88,0%	95,7%	92,2%
8931	40	20	70	80	29,8%	38,9%	88,4%	96,0%	92,7%
8932	40	20	75	20	21,1%	37,1%	81,7%	90,8%	84,2%
8933	40	20	75	25	21,4%	36,8%	82,2%	91,3%	85,1%
8934	40	20	75	30	21,7%	36,5%	82,7%	91,9%	86,0%
8935	40	20	75	35	22,0%	36,2%	83,2%	92,4%	86,8%
8936	40	20	75	40	22,3%	35,9%	83,7%	92,8%	87,6%
8937	40	20	75	45	22,7%	35,6%	84,1%	93,3%	88,3%
8938	40	20	75	50	23,0%	35,3%	84,6%	93,7%	89,0%
8939	40	20	75	55	23,3%	35,0%	85,0%	94,1%	89,6%
8940	40	20	75	60	23,6%	34,7%	85,4%	94,5%	90,2%
8941	40	20	75	65	23,9%	34,4%	85,8%	94,8%	90,8%
8942	40	20	75	70	24,2%	34,1%	86,3%	95,1%	91,4%

8943	40	20	75	75	24,6%	33,8%	86,6%	95,5%	91,9%
8944	40	20	75	80	24,9%	33,6%	87,0%	95,7%	92,4%
8945	40	20	80	20	17,3%	31,9%	79,8%	90,2%	83,7%
8946	40	20	80	25	17,6%	31,6%	80,3%	90,8%	84,6%
8947	40	20	80	30	17,8%	31,4%	80,9%	91,4%	85,5%
8948	40	20	80	35	18,1%	31,1%	81,4%	91,9%	86,3%
8949	40	20	80	40	18,4%	30,8%	81,9%	92,4%	87,1%
8950	40	20	80	45	18,6%	30,5%	82,4%	92,9%	87,8%
8951	40	20	80	50	18,9%	30,2%	82,9%	93,3%	88,6%
8952	40	20	80	55	19,2%	30,0%	83,3%	93,7%	89,2%
8953	40	20	80	60	19,4%	29,7%	83,8%	94,1%	89,9%
8954	40	20	80	65	19,7%	29,4%	84,3%	94,5%	90,5%
8955	40	20	80	70	20,0%	29,2%	84,7%	94,9%	91,1%
8956	40	20	80	75	20,3%	28,9%	85,1%	95,2%	91,6%
8957	40	20	80	80	20,6%	28,6%	85,6%	95,5%	92,1%
8958	40	25	20	20	81,6%	89,0%	94,4%	94,7%	88,4%
8959	40	25	20	25	81,8%	88,9%	94,6%	95,0%	89,1%
8960	40	25	20	30	82,1%	88,8%	94,8%	95,3%	89,8%
8961	40	25	20	35	82,4%	88,6%	94,9%	95,6%	90,4%
8962	40	25	20	40	82,6%	88,5%	95,1%	95,9%	91,0%
8963	40	25	20	45	82,9%	88,4%	95,3%	96,2%	91,5%
8964	40	25	20	50	83,1%	88,2%	95,4%	96,4%	92,0%
8965	40	25	20	55	83,4%	88,1%	95,6%	96,7%	92,5%
8966	40	25	20	60	83,6%	88,0%	95,7%	96,9%	93,0%
8967	40	25	20	65	83,9%	87,8%	95,8%	97,1%	93,4%
8968	40	25	20	70	84,1%	87,7%	96,0%	97,3%	93,8%
8969	40	25	20	75	84,3%	87,5%	96,1%	97,4%	94,2%
8970	40	25	20	80	84,6%	87,4%	96,2%	97,6%	94,6%
8971	40	25	25	20	77,6%	86,6%	93,7%	94,4%	88,0%
8972	40	25	25	25	77,9%	86,4%	93,9%	94,7%	88,7%
8973	40	25	25	30	78,2%	86,2%	94,1%	95,1%	89,4%
8974	40	25	25	35	78,5%	86,1%	94,3%	95,4%	90,0%
8975	40	25	25	40	78,8%	85,9%	94,5%	95,7%	90,6%

8976	40	25	25	45	79,1%	85,8%	94,7%	96,0%	91,2%
8977	40	25	25	50	79,4%	85,6%	94,8%	96,2%	91,7%
8978	40	25	25	55	79,7%	85,4%	95,0%	96,5%	92,2%
8979	40	25	25	60	79,9%	85,3%	95,2%	96,7%	92,7%
8980	40	25	25	65	80,2%	85,1%	95,3%	96,9%	93,2%
8981	40	25	25	70	80,5%	85,0%	95,5%	97,1%	93,6%
8982	40	25	25	75	80,8%	84,8%	95,6%	97,3%	94,0%
8983	40	25	25	80	81,0%	84,6%	95,7%	97,5%	94,4%
8984	40	25	30	20	73,0%	83,6%	93,0%	94,0%	87,6%
8985	40	25	30	25	73,3%	83,4%	93,2%	94,4%	88,3%
8986	40	25	30	30	73,7%	83,3%	93,4%	94,8%	89,0%
8987	40	25	30	35	74,0%	83,1%	93,6%	95,1%	89,6%
8988	40	25	30	40	74,4%	82,9%	93,8%	95,4%	90,3%
8989	40	25	30	45	74,7%	82,7%	94,0%	95,7%	90,8%
8990	40	25	30	50	75,0%	82,5%	94,2%	96,0%	91,4%
8991	40	25	30	55	75,4%	82,3%	94,4%	96,2%	91,9%
8992	40	25	30	60	75,7%	82,1%	94,6%	96,5%	92,4%
8993	40	25	30	65	76,0%	82,0%	94,7%	96,7%	92,9%
8994	40	25	30	70	76,3%	81,8%	94,9%	96,9%	93,3%
8995	40	25	30	75	76,6%	81,6%	95,1%	97,1%	93,7%
8996	40	25	30	80	77,0%	81,4%	95,2%	97,3%	94,1%
8997	40	25	35	20	67,9%	80,2%	92,1%	93,7%	87,1%
8998	40	25	35	25	68,3%	80,0%	92,4%	94,1%	87,9%
8999	40	25	35	30	68,6%	79,8%	92,6%	94,5%	88,6%
9000	40	25	35	35	69,0%	79,6%	92,8%	94,8%	89,3%
9001	40	25	35	40	69,4%	79,4%	93,1%	95,1%	89,9%
9002	40	25	35	45	69,8%	79,2%	93,3%	95,5%	90,5%
9003	40	25	35	50	70,1%	78,9%	93,5%	95,7%	91,1%
9004	40	25	35	55	70,5%	78,7%	93,7%	96,0%	91,6%
9005	40	25	35	60	70,9%	78,5%	93,9%	96,3%	92,1%
9006	40	25	35	65	71,2%	78,3%	94,1%	96,5%	92,6%
9007	40	25	35	70	71,6%	78,1%	94,3%	96,7%	93,1%
9008	40	25	35	75	71,9%	77,8%	94,4%	96,9%	93,5%

9009	40	25	35	80	72,3%	77,6%	94,6%	97,1%	93,9%
9010	40	25	40	20	62,3%	76,3%	91,2%	93,3%	86,6%
9011	40	25	40	25	62,7%	76,1%	91,4%	93,7%	87,4%
9012	40	25	40	30	63,1%	75,8%	91,7%	94,1%	88,2%
9013	40	25	40	35	63,5%	75,6%	92,0%	94,5%	88,9%
9014	40	25	40	40	63,9%	75,3%	92,2%	94,9%	89,5%
9015	40	25	40	45	64,3%	75,1%	92,4%	95,2%	90,1%
9016	40	25	40	50	64,7%	74,8%	92,7%	95,5%	90,7%
9017	40	25	40	55	65,1%	74,6%	92,9%	95,8%	91,3%
9018	40	25	40	60	65,5%	74,4%	93,1%	96,0%	91,8%
9019	40	25	40	65	65,9%	74,1%	93,3%	96,3%	92,3%
9020	40	25	40	70	66,3%	73,9%	93,5%	96,5%	92,8%
9021	40	25	40	75	66,7%	73,6%	93,8%	96,8%	93,2%
9022	40	25	40	80	67,1%	73,3%	93,9%	97,0%	93,7%
9023	40	25	45	20	56,3%	71,9%	90,1%	92,9%	86,2%
9024	40	25	45	25	56,7%	71,6%	90,4%	93,4%	87,0%
9025	40	25	45	30	57,2%	71,3%	90,7%	93,8%	87,7%
9026	40	25	45	35	57,6%	71,1%	91,0%	94,2%	88,4%
9027	40	25	45	40	58,0%	70,8%	91,3%	94,5%	89,1%
9028	40	25	45	45	58,5%	70,5%	91,5%	94,9%	89,8%
9029	40	25	45	50	58,9%	70,3%	91,8%	95,2%	90,4%
9030	40	25	45	55	59,3%	70,0%	92,0%	95,5%	91,0%
9031	40	25	45	60	59,7%	69,7%	92,3%	95,8%	91,5%
9032	40	25	45	65	60,2%	69,4%	92,5%	96,1%	92,0%
9033	40	25	45	70	60,6%	69,2%	92,8%	96,3%	92,5%
9034	40	25	45	75	61,0%	68,9%	93,0%	96,6%	93,0%
9035	40	25	45	80	61,4%	68,6%	93,2%	96,8%	93,4%
9036	40	25	50	20	50,2%	67,0%	89,0%	92,5%	85,7%
9037	40	25	50	25	50,6%	66,7%	89,3%	93,0%	86,5%
9038	40	25	50	30	51,1%	66,4%	89,6%	93,4%	87,3%
9039	40	25	50	35	51,5%	66,1%	89,9%	93,8%	88,0%
9040	40	25	50	40	51,9%	65,8%	90,2%	94,2%	88,7%
9041	40	25	50	45	52,4%	65,5%	90,5%	94,6%	89,4%

9042	40	25	50	50	52,8%	65,2%	90,8%	94,9%	90,0%
9043	40	25	50	55	53,3%	64,9%	91,1%	95,3%	90,6%
9044	40	25	50	60	53,7%	64,6%	91,4%	95,6%	91,2%
9045	40	25	50	65	54,1%	64,3%	91,6%	95,8%	91,7%
9046	40	25	50	70	54,6%	64,0%	91,9%	96,1%	92,2%
9047	40	25	50	75	55,0%	63,7%	92,1%	96,4%	92,7%
9048	40	25	50	80	55,4%	63,4%	92,4%	96,6%	93,1%
9049	40	25	55	20	44,0%	61,7%	87,7%	92,1%	85,1%
9050	40	25	55	25	44,5%	61,4%	88,0%	92,6%	86,0%
9051	40	25	55	30	44,9%	61,1%	88,4%	93,0%	86,8%
9052	40	25	55	35	45,3%	60,8%	88,7%	93,5%	87,6%
9053	40	25	55	40	45,8%	60,4%	89,1%	93,9%	88,3%
9054	40	25	55	45	46,2%	60,1%	89,4%	94,3%	89,0%
9055	40	25	55	50	46,7%	59,8%	89,7%	94,6%	89,6%
9056	40	25	55	55	47,1%	59,5%	90,0%	95,0%	90,3%
9057	40	25	55	60	47,5%	59,2%	90,3%	95,3%	90,8%
9058	40	25	55	65	48,0%	58,9%	90,6%	95,6%	91,4%
9059	40	25	55	70	48,4%	58,6%	90,9%	95,9%	91,9%
9060	40	25	55	75	48,9%	58,2%	91,2%	96,1%	92,4%
9061	40	25	55	80	49,3%	57,9%	91,4%	96,4%	92,9%
9062	40	25	60	20	38,1%	56,1%	86,3%	91,6%	84,6%
9063	40	25	60	25	38,5%	55,8%	86,7%	92,2%	85,5%
9064	40	25	60	30	38,9%	55,5%	87,1%	92,6%	86,3%
9065	40	25	60	35	39,3%	55,1%	87,4%	93,1%	87,1%
9066	40	25	60	40	39,7%	54,8%	87,8%	93,5%	87,9%
9067	40	25	60	45	40,2%	54,5%	88,2%	93,9%	88,6%
9068	40	25	60	50	40,6%	54,2%	88,5%	94,3%	89,2%
9069	40	25	60	55	41,0%	53,8%	88,8%	94,7%	89,9%
9070	40	25	60	60	41,4%	53,5%	89,2%	95,0%	90,5%
9071	40	25	60	65	41,9%	53,2%	89,5%	95,3%	91,1%
9072	40	25	60	70	42,3%	52,9%	89,8%	95,6%	91,6%
9073	40	25	60	75	42,7%	52,5%	90,1%	95,9%	92,1%
9074	40	25	60	80	43,2%	52,2%	90,4%	96,2%	92,6%

9075	40	25	65	20	32,4%	50,4%	84,7%	91,2%	84,1%
9076	40	25	65	25	32,8%	50,0%	85,2%	91,7%	85,0%
9077	40	25	65	30	33,2%	49,7%	85,6%	92,2%	85,8%
9078	40	25	65	35	33,6%	49,4%	86,0%	92,7%	86,6%
9079	40	25	65	40	34,0%	49,1%	86,4%	93,1%	87,4%
9080	40	25	65	45	34,4%	48,7%	86,8%	93,6%	88,1%
9081	40	25	65	50	34,8%	48,4%	87,2%	94,0%	88,8%
9082	40	25	65	55	35,2%	48,1%	87,6%	94,4%	89,5%
9083	40	25	65	60	35,6%	47,7%	87,9%	94,7%	90,1%
9084	40	25	65	65	36,0%	47,4%	88,3%	95,0%	90,7%
9085	40	25	65	70	36,4%	47,1%	88,6%	95,4%	91,3%
9086	40	25	65	75	36,8%	46,8%	89,0%	95,7%	91,8%
9087	40	25	65	80	37,2%	46,4%	89,3%	95,9%	92,3%
9088	40	25	70	20	27,3%	44,6%	83,0%	90,7%	83,5%
9089	40	25	70	25	27,6%	44,3%	83,5%	91,2%	84,4%
9090	40	25	70	30	28,0%	44,0%	84,0%	91,8%	85,3%
9091	40	25	70	35	28,3%	43,6%	84,4%	92,3%	86,2%
9092	40	25	70	40	28,7%	43,3%	84,9%	92,7%	87,0%
9093	40	25	70	45	29,1%	43,0%	85,3%	93,2%	87,7%
9094	40	25	70	50	29,4%	42,7%	85,7%	93,6%	88,4%
9095	40	25	70	55	29,8%	42,4%	86,1%	94,0%	89,1%
9096	40	25	70	60	30,2%	42,0%	86,5%	94,4%	89,8%
9097	40	25	70	65	30,5%	41,7%	86,9%	94,8%	90,4%
9098	40	25	70	70	30,9%	41,4%	87,3%	95,1%	91,0%
9099	40	25	70	75	31,3%	41,1%	87,7%	95,4%	91,5%
9100	40	25	70	80	31,7%	40,8%	88,0%	95,7%	92,0%
9101	40	25	75	20	22,7%	39,0%	81,2%	90,1%	82,9%
9102	40	25	75	25	23,0%	38,7%	81,7%	90,7%	83,9%
9103	40	25	75	30	23,3%	38,4%	82,2%	91,3%	84,8%
9104	40	25	75	35	23,6%	38,1%	82,7%	91,8%	85,7%
9105	40	25	75	40	23,9%	37,8%	83,2%	92,3%	86,5%
9106	40	25	75	45	24,2%	37,5%	83,7%	92,8%	87,3%
9107	40	25	75	50	24,6%	37,1%	84,1%	93,2%	88,0%

9108	40	25	75	55	24,9%	36,8%	84,6%	93,7%	88,7%
9109	40	25	75	60	25,2%	36,5%	85,0%	94,1%	89,4%
9110	40	25	75	65	25,6%	36,2%	85,5%	94,4%	90,0%
9111	40	25	75	70	25,9%	35,9%	85,9%	94,8%	90,6%
9112	40	25	75	75	26,2%	35,6%	86,3%	95,1%	91,2%
9113	40	25	75	80	26,6%	35,3%	86,7%	95,4%	91,7%
9114	40	25	80	20	18,6%	33,7%	79,3%	89,6%	82,3%
9115	40	25	80	25	18,9%	33,4%	79,8%	90,2%	83,3%
9116	40	25	80	30	19,2%	33,1%	80,4%	90,8%	84,3%
9117	40	25	80	35	19,4%	32,8%	80,9%	91,4%	85,1%
9118	40	25	80	40	19,7%	32,5%	81,4%	91,9%	86,0%
9119	40	25	80	45	20,0%	32,2%	81,9%	92,4%	86,8%
9120	40	25	80	50	20,3%	31,9%	82,4%	92,9%	87,6%
9121	40	25	80	55	20,6%	31,6%	82,9%	93,3%	88,3%
9122	40	25	80	60	20,9%	31,4%	83,4%	93,7%	89,0%
9123	40	25	80	65	21,2%	31,1%	83,8%	94,1%	89,6%
9124	40	25	80	70	21,4%	30,8%	84,3%	94,5%	90,2%
9125	40	25	80	75	21,7%	30,5%	84,7%	94,8%	90,8%
9126	40	25	80	80	22,0%	30,2%	85,2%	95,2%	91,4%
9127	40	30	20	20	82,9%	89,8%	94,3%	94,3%	87,4%
9128	40	30	20	25	83,1%	89,6%	94,4%	94,7%	88,2%
9129	40	30	20	30	83,4%	89,5%	94,6%	95,0%	88,9%
9130	40	30	20	35	83,6%	89,4%	94,8%	95,3%	89,5%
9131	40	30	20	40	83,9%	89,3%	95,0%	95,6%	90,1%
9132	40	30	20	45	84,1%	89,1%	95,1%	95,9%	90,7%
9133	40	30	20	50	84,3%	89,0%	95,3%	96,2%	91,3%
9134	40	30	20	55	84,6%	88,9%	95,4%	96,4%	91,8%
9135	40	30	20	60	84,8%	88,8%	95,6%	96,6%	92,3%
9136	40	30	20	65	85,0%	88,6%	95,7%	96,9%	92,8%
9137	40	30	20	70	85,2%	88,5%	95,8%	97,1%	93,2%
9138	40	30	20	75	85,5%	88,4%	96,0%	97,3%	93,7%
9139	40	30	20	80	85,7%	88,2%	96,1%	97,4%	94,1%
9140	40	30	25	20	79,1%	87,4%	93,6%	94,0%	87,0%

9141	40	30	25	25	79,4%	87,3%	93,8%	94,4%	87,7%
9142	40	30	25	30	79,7%	87,2%	94,0%	94,7%	88,4%
9143	40	30	25	35	80,0%	87,0%	94,1%	95,0%	89,1%
9144	40	30	25	40	80,2%	86,9%	94,3%	95,4%	89,8%
9145	40	30	25	45	80,5%	86,7%	94,5%	95,7%	90,4%
9146	40	30	25	50	80,8%	86,6%	94,7%	95,9%	91,0%
9147	40	30	25	55	81,1%	86,4%	94,8%	96,2%	91,5%
9148	40	30	25	60	81,3%	86,2%	95,0%	96,4%	92,0%
9149	40	30	25	65	81,6%	86,1%	95,2%	96,7%	92,5%
9150	40	30	25	70	81,9%	85,9%	95,3%	96,9%	93,0%
9151	40	30	25	75	82,1%	85,8%	95,5%	97,1%	93,4%
9152	40	30	25	80	82,4%	85,6%	95,6%	97,3%	93,8%
9153	40	30	30	20	74,7%	84,7%	92,8%	93,6%	86,5%
9154	40	30	30	25	75,0%	84,5%	93,0%	94,0%	87,3%
9155	40	30	30	30	75,4%	84,3%	93,2%	94,4%	88,0%
9156	40	30	30	35	75,7%	84,2%	93,4%	94,7%	88,7%
9157	40	30	30	40	76,0%	84,0%	93,6%	95,1%	89,4%
9158	40	30	30	45	76,3%	83,8%	93,8%	95,4%	90,0%
9159	40	30	30	50	76,7%	83,6%	94,0%	95,7%	90,6%
9160	40	30	30	55	77,0%	83,5%	94,2%	96,0%	91,2%
9161	40	30	30	60	77,3%	83,3%	94,4%	96,2%	91,7%
9162	40	30	30	65	77,6%	83,1%	94,6%	96,5%	92,2%
9163	40	30	30	70	77,9%	82,9%	94,7%	96,7%	92,7%
9164	40	30	30	75	78,2%	82,7%	94,9%	96,9%	93,2%
9165	40	30	30	80	78,5%	82,5%	95,1%	97,1%	93,6%
9166	40	30	35	20	69,8%	81,4%	91,9%	93,2%	86,0%
9167	40	30	35	25	70,1%	81,2%	92,1%	93,7%	86,8%
9168	40	30	35	30	70,5%	81,0%	92,4%	94,1%	87,6%
9169	40	30	35	35	70,9%	80,8%	92,6%	94,4%	88,3%
9170	40	30	35	40	71,2%	80,6%	92,8%	94,8%	89,0%
9171	40	30	35	45	71,6%	80,4%	93,1%	95,1%	89,6%
9172	40	30	35	50	72,0%	80,2%	93,3%	95,4%	90,3%
9173	40	30	35	55	72,3%	80,0%	93,5%	95,7%	90,8%

9174	40	30	35	60	72,7%	79,8%	93,7%	96,0%	91,4%
9175	40	30	35	65	73,0%	79,6%	93,9%	96,3%	91,9%
9176	40	30	35	70	73,4%	79,4%	94,1%	96,5%	92,4%
9177	40	30	35	75	73,7%	79,2%	94,3%	96,7%	92,9%
9178	40	30	35	80	74,0%	78,9%	94,5%	96,9%	93,3%
9179	40	30	40	20	64,3%	77,7%	90,9%	92,9%	85,5%
9180	40	30	40	25	64,7%	77,5%	91,2%	93,3%	86,3%
9181	40	30	40	30	65,1%	77,2%	91,5%	93,7%	87,1%
9182	40	30	40	35	65,5%	77,0%	91,7%	94,1%	87,9%
9183	40	30	40	40	65,9%	76,8%	92,0%	94,5%	88,6%
9184	40	30	40	45	66,3%	76,5%	92,2%	94,8%	89,3%
9185	40	30	40	50	66,7%	76,3%	92,5%	95,2%	89,9%
9186	40	30	40	55	67,1%	76,1%	92,7%	95,5%	90,5%
9187	40	30	40	60	67,5%	75,8%	92,9%	95,8%	91,1%
9188	40	30	40	65	67,9%	75,6%	93,1%	96,0%	91,6%
9189	40	30	40	70	68,3%	75,3%	93,4%	96,3%	92,1%
9190	40	30	40	75	68,6%	75,1%	93,6%	96,5%	92,6%
9191	40	30	40	80	69,0%	74,9%	93,8%	96,7%	93,1%
9192	40	30	45	20	58,5%	73,4%	89,8%	92,4%	85,0%
9193	40	30	45	25	58,9%	73,2%	90,1%	92,9%	85,8%
9194	40	30	45	30	59,3%	72,9%	90,4%	93,3%	86,6%
9195	40	30	45	35	59,8%	72,7%	90,7%	93,8%	87,4%
9196	40	30	45	40	60,2%	72,4%	91,0%	94,2%	88,2%
9197	40	30	45	45	60,6%	72,1%	91,3%	94,5%	88,9%
9198	40	30	45	50	61,0%	71,9%	91,5%	94,9%	89,5%
9199	40	30	45	55	61,4%	71,6%	91,8%	95,2%	90,1%
9200	40	30	45	60	61,9%	71,3%	92,1%	95,5%	90,7%
9201	40	30	45	65	62,3%	71,1%	92,3%	95,8%	91,3%
9202	40	30	45	70	62,7%	70,8%	92,5%	96,1%	91,8%
9203	40	30	45	75	63,1%	70,5%	92,8%	96,3%	92,3%
9204	40	30	45	80	63,5%	70,3%	93,0%	96,5%	92,8%
9205	40	30	50	20	52,4%	68,7%	88,6%	92,0%	84,4%
9206	40	30	50	25	52,8%	68,4%	89,0%	92,5%	85,3%

9207	40	30	50	30	53,3%	68,1%	89,3%	93,0%	86,2%
9208	40	30	50	35	53,7%	67,8%	89,6%	93,4%	87,0%
9209	40	30	50	40	54,1%	67,6%	89,9%	93,8%	87,7%
9210	40	30	50	45	54,6%	67,3%	90,2%	94,2%	88,4%
9211	40	30	50	50	55,0%	67,0%	90,5%	94,6%	89,1%
9212	40	30	50	55	55,5%	66,7%	90,8%	94,9%	89,8%
9213	40	30	50	60	55,9%	66,4%	91,1%	95,2%	90,4%
9214	40	30	50	65	56,3%	66,1%	91,4%	95,5%	91,0%
9215	40	30	50	70	56,8%	65,8%	91,6%	95,8%	91,5%
9216	40	30	50	75	57,2%	65,5%	91,9%	96,1%	92,0%
9217	40	30	50	80	57,6%	65,2%	92,1%	96,3%	92,5%
9218	40	30	55	20	46,2%	63,5%	87,3%	91,5%	83,9%
9219	40	30	55	25	46,7%	63,2%	87,7%	92,1%	84,8%
9220	40	30	55	30	47,1%	62,9%	88,1%	92,5%	85,7%
9221	40	30	55	35	47,5%	62,6%	88,4%	93,0%	86,5%
9222	40	30	55	40	48,0%	62,3%	88,8%	93,4%	87,3%
9223	40	30	55	45	48,4%	62,0%	89,1%	93,9%	88,0%
9224	40	30	55	50	48,9%	61,7%	89,4%	94,2%	88,7%
9225	40	30	55	55	49,3%	61,4%	89,7%	94,6%	89,4%
9226	40	30	55	60	49,7%	61,1%	90,0%	94,9%	90,0%
9227	40	30	55	65	50,2%	60,8%	90,3%	95,3%	90,6%
9228	40	30	55	70	50,6%	60,4%	90,6%	95,6%	91,2%
9229	40	30	55	75	51,1%	60,1%	90,9%	95,9%	91,7%
9230	40	30	55	80	51,5%	59,8%	91,2%	96,1%	92,2%
9231	40	30	60	20	40,2%	58,0%	85,9%	91,1%	83,3%
9232	40	30	60	25	40,6%	57,7%	86,3%	91,6%	84,3%
9233	40	30	60	30	41,0%	57,4%	86,7%	92,1%	85,2%
9234	40	30	60	35	41,5%	57,1%	87,1%	92,6%	86,0%
9235	40	30	60	40	41,9%	56,8%	87,5%	93,1%	86,8%
9236	40	30	60	45	42,3%	56,4%	87,8%	93,5%	87,6%
9237	40	30	60	50	42,7%	56,1%	88,2%	93,9%	88,3%
9238	40	30	60	55	43,2%	55,8%	88,5%	94,3%	89,0%
9239	40	30	60	60	43,6%	55,5%	88,9%	94,6%	89,6%

9240	40	30	60	65	44,0%	55,1%	89,2%	95,0%	90,3%
9241	40	30	60	70	44,5%	54,8%	89,5%	95,3%	90,8%
9242	40	30	60	75	44,9%	54,5%	89,8%	95,6%	91,4%
9243	40	30	60	80	45,4%	54,2%	90,1%	95,9%	91,9%
9244	40	30	65	20	34,4%	52,3%	84,3%	90,5%	82,8%
9245	40	30	65	25	34,8%	52,0%	84,8%	91,1%	83,7%
9246	40	30	65	30	35,2%	51,7%	85,2%	91,7%	84,6%
9247	40	30	65	35	35,6%	51,3%	85,6%	92,2%	85,5%
9248	40	30	65	40	36,0%	51,0%	86,0%	92,7%	86,3%
9249	40	30	65	45	36,4%	50,7%	86,4%	93,1%	87,1%
9250	40	30	65	50	36,8%	50,4%	86,8%	93,5%	87,9%
9251	40	30	65	55	37,2%	50,0%	87,2%	93,9%	88,6%
9252	40	30	65	60	37,7%	49,7%	87,6%	94,3%	89,2%
9253	40	30	65	65	38,1%	49,4%	87,9%	94,7%	89,9%
9254	40	30	65	70	38,5%	49,1%	88,3%	95,0%	90,5%
9255	40	30	65	75	38,9%	48,7%	88,6%	95,3%	91,1%
9256	40	30	65	80	39,3%	48,4%	89,0%	95,6%	91,6%
9257	40	30	70	20	29,1%	46,6%	82,6%	90,0%	82,2%
9258	40	30	70	25	29,4%	46,2%	83,1%	90,6%	83,1%
9259	40	30	70	30	29,8%	45,9%	83,6%	91,2%	84,1%
9260	40	30	70	35	30,2%	45,6%	84,0%	91,7%	85,0%
9261	40	30	70	40	30,5%	45,3%	84,5%	92,2%	85,8%
9262	40	30	70	45	30,9%	44,9%	84,9%	92,7%	86,6%
9263	40	30	70	50	31,3%	44,6%	85,3%	93,2%	87,4%
9264	40	30	70	55	31,7%	44,3%	85,8%	93,6%	88,1%
9265	40	30	70	60	32,1%	44,0%	86,2%	94,0%	88,8%
9266	40	30	70	65	32,4%	43,6%	86,6%	94,4%	89,5%
9267	40	30	70	70	32,8%	43,3%	87,0%	94,7%	90,1%
9268	40	30	70	75	33,2%	43,0%	87,3%	95,1%	90,7%
9269	40	30	70	80	33,6%	42,7%	87,7%	95,4%	91,3%
9270	40	30	75	20	24,3%	40,9%	80,7%	89,4%	81,5%
9271	40	30	75	25	24,6%	40,6%	81,3%	90,1%	82,6%
9272	40	30	75	30	24,9%	40,3%	81,8%	90,7%	83,5%

9273	40	30	75	35	25,2%	39,9%	82,3%	91,2%	84,4%
9274	40	30	75	40	25,6%	39,6%	82,8%	91,8%	85,3%
9275	40	30	75	45	25,9%	39,3%	83,2%	92,3%	86,2%
9276	40	30	75	50	26,3%	39,0%	83,7%	92,8%	87,0%
9277	40	30	75	55	26,6%	38,7%	84,2%	93,2%	87,7%
9278	40	30	75	60	26,9%	38,4%	84,6%	93,6%	88,4%
9279	40	30	75	65	27,3%	38,1%	85,1%	94,0%	89,1%
9280	40	30	75	70	27,6%	37,8%	85,5%	94,4%	89,8%
9281	40	30	75	75	28,0%	37,5%	85,9%	94,8%	90,4%
9282	40	30	75	80	28,3%	37,2%	86,3%	95,1%	91,0%
9283	40	30	80	20	20,0%	35,4%	78,7%	88,9%	80,9%
9284	40	30	80	25	20,3%	35,1%	79,3%	89,5%	82,0%
9285	40	30	80	30	20,6%	34,8%	79,8%	90,2%	82,9%
9286	40	30	80	35	20,9%	34,5%	80,4%	90,7%	83,9%
9287	40	30	80	40	21,2%	34,3%	80,9%	91,3%	84,8%
9288	40	30	80	45	21,5%	34,0%	81,4%	91,8%	85,7%
9289	40	30	80	50	21,8%	33,7%	81,9%	92,3%	86,5%
9290	40	30	80	55	22,1%	33,4%	82,4%	92,8%	87,3%
9291	40	30	80	60	22,4%	33,1%	82,9%	93,3%	88,0%
9292	40	30	80	65	22,7%	32,8%	83,4%	93,7%	88,7%
9293	40	30	80	70	23,0%	32,5%	83,9%	94,1%	89,4%
9294	40	30	80	75	23,3%	32,2%	84,3%	94,5%	90,0%
9295	40	30	80	80	23,6%	31,9%	84,8%	94,8%	90,6%
9296	40	35	20	20	84,1%	90,5%	94,1%	93,9%	86,3%
9297	40	35	20	25	84,3%	90,4%	94,3%	94,3%	87,1%
9298	40	35	20	30	84,6%	90,2%	94,5%	94,6%	87,9%
9299	40	35	20	35	84,8%	90,1%	94,6%	95,0%	88,6%
9300	40	35	20	40	85,0%	90,0%	94,8%	95,3%	89,3%
9301	40	35	20	45	85,2%	89,9%	95,0%	95,6%	89,9%
9302	40	35	20	50	85,5%	89,8%	95,1%	95,9%	90,5%
9303	40	35	20	55	85,7%	89,6%	95,3%	96,1%	91,1%
9304	40	35	20	60	85,9%	89,5%	95,4%	96,4%	91,6%
9305	40	35	20	65	86,1%	89,4%	95,6%	96,6%	92,1%

9306	40	35	20	70	86,3%	89,3%	95,7%	96,8%	92,6%
9307	40	35	20	75	86,5%	89,2%	95,9%	97,0%	93,1%
9308	40	35	20	80	86,7%	89,0%	96,0%	97,2%	93,5%
9309	40	35	25	20	80,5%	88,3%	93,4%	93,5%	85,8%
9310	40	35	25	25	80,8%	88,1%	93,6%	93,9%	86,7%
9311	40	35	25	30	81,1%	88,0%	93,8%	94,3%	87,4%
9312	40	35	25	35	81,3%	87,9%	94,0%	94,7%	88,2%
9313	40	35	25	40	81,6%	87,7%	94,2%	95,0%	88,9%
9314	40	35	25	45	81,9%	87,6%	94,3%	95,3%	89,5%
9315	40	35	25	50	82,1%	87,4%	94,5%	95,6%	90,1%
9316	40	35	25	55	82,4%	87,3%	94,7%	95,9%	90,7%
9317	40	35	25	60	82,6%	87,2%	94,9%	96,2%	91,3%
9318	40	35	25	65	82,9%	87,0%	95,0%	96,4%	91,8%
9319	40	35	25	70	83,1%	86,9%	95,2%	96,7%	92,3%
9320	40	35	25	75	83,4%	86,7%	95,3%	96,9%	92,8%
9321	40	35	25	80	83,6%	86,6%	95,5%	97,1%	93,2%
9322	40	35	30	20	76,4%	85,7%	92,5%	93,2%	85,3%
9323	40	35	30	25	76,7%	85,5%	92,8%	93,6%	86,2%
9324	40	35	30	30	77,0%	85,3%	93,0%	94,0%	87,0%
9325	40	35	30	35	77,3%	85,2%	93,2%	94,4%	87,7%
9326	40	35	30	40	77,6%	85,0%	93,4%	94,7%	88,4%
9327	40	35	30	45	77,9%	84,9%	93,6%	95,1%	89,1%
9328	40	35	30	50	78,2%	84,7%	93,8%	95,4%	89,8%
9329	40	35	30	55	78,5%	84,5%	94,0%	95,7%	90,4%
9330	40	35	30	60	78,8%	84,3%	94,2%	95,9%	91,0%
9331	40	35	30	65	79,1%	84,2%	94,4%	96,2%	91,5%
9332	40	35	30	70	79,4%	84,0%	94,6%	96,4%	92,0%
9333	40	35	30	75	79,7%	83,8%	94,7%	96,7%	92,5%
9334	40	35	30	80	80,0%	83,6%	94,9%	96,9%	93,0%
9335	40	35	35	20	71,6%	82,6%	91,6%	92,8%	84,8%
9336	40	35	35	25	72,0%	82,4%	91,9%	93,2%	85,7%
9337	40	35	35	30	72,3%	82,2%	92,2%	93,6%	86,5%
9338	40	35	35	35	72,7%	82,0%	92,4%	94,0%	87,3%

9339	40	35	35	40	73,0%	81,8%	92,6%	94,4%	88,0%
9340	40	35	35	45	73,4%	81,6%	92,9%	94,8%	88,7%
9341	40	35	35	50	73,7%	81,4%	93,1%	95,1%	89,4%
9342	40	35	35	55	74,1%	81,2%	93,3%	95,4%	90,0%
9343	40	35	35	60	74,4%	81,0%	93,5%	95,7%	90,6%
9344	40	35	35	65	74,7%	80,8%	93,7%	96,0%	91,2%
9345	40	35	35	70	75,1%	80,6%	93,9%	96,2%	91,7%
9346	40	35	35	75	75,4%	80,4%	94,1%	96,5%	92,2%
9347	40	35	35	80	75,7%	80,2%	94,3%	96,7%	92,7%
9348	40	35	40	20	66,3%	79,0%	90,6%	92,3%	84,3%
9349	40	35	40	25	66,7%	78,8%	90,9%	92,8%	85,2%
9350	40	35	40	30	67,1%	78,6%	91,2%	93,3%	86,0%
9351	40	35	40	35	67,5%	78,4%	91,5%	93,7%	86,8%
9352	40	35	40	40	67,9%	78,1%	91,7%	94,1%	87,6%
9353	40	35	40	45	68,3%	77,9%	92,0%	94,5%	88,3%
9354	40	35	40	50	68,7%	77,7%	92,2%	94,8%	89,0%
9355	40	35	40	55	69,0%	77,5%	92,5%	95,1%	89,6%
9356	40	35	40	60	69,4%	77,2%	92,7%	95,4%	90,3%
9357	40	35	40	65	69,8%	77,0%	92,9%	95,7%	90,9%
9358	40	35	40	70	70,2%	76,8%	93,2%	96,0%	91,4%
9359	40	35	40	75	70,5%	76,5%	93,4%	96,3%	91,9%
9360	40	35	40	80	70,9%	76,3%	93,6%	96,5%	92,4%
9361	40	35	45	20	60,6%	74,9%	89,5%	91,9%	83,7%
9362	40	35	45	25	61,0%	74,7%	89,9%	92,4%	84,6%
9363	40	35	45	30	61,5%	74,4%	90,2%	92,9%	85,5%
9364	40	35	45	35	61,9%	74,2%	90,5%	93,3%	86,3%
9365	40	35	45	40	62,3%	73,9%	90,7%	93,7%	87,1%
9366	40	35	45	45	62,7%	73,7%	91,0%	94,1%	87,9%
9367	40	35	45	50	63,1%	73,4%	91,3%	94,5%	88,6%
9368	40	35	45	55	63,5%	73,2%	91,6%	94,8%	89,3%
9369	40	35	45	60	63,9%	72,9%	91,8%	95,2%	89,9%
9370	40	35	45	65	64,3%	72,7%	92,1%	95,5%	90,5%
9371	40	35	45	70	64,7%	72,4%	92,3%	95,8%	91,1%

9372	40	35	45	75	65,1%	72,1%	92,6%	96,0%	91,6%
9373	40	35	45	80	65,5%	71,9%	92,8%	96,3%	92,1%
9374	40	35	50	20	54,6%	70,4%	88,3%	91,4%	83,2%
9375	40	35	50	25	55,0%	70,1%	88,7%	92,0%	84,1%
9376	40	35	50	30	55,5%	69,8%	89,0%	92,5%	85,0%
9377	40	35	50	35	55,9%	69,5%	89,3%	92,9%	85,8%
9378	40	35	50	40	56,3%	69,3%	89,6%	93,4%	86,7%
9379	40	35	50	45	56,8%	69,0%	90,0%	93,8%	87,4%
9380	40	35	50	50	57,2%	68,7%	90,3%	94,2%	88,2%
9381	40	35	50	55	57,6%	68,4%	90,6%	94,5%	88,9%
9382	40	35	50	60	58,1%	68,1%	90,8%	94,9%	89,5%
9383	40	35	50	65	58,5%	67,9%	91,1%	95,2%	90,1%
9384	40	35	50	70	58,9%	67,6%	91,4%	95,5%	90,7%
9385	40	35	50	75	59,3%	67,3%	91,7%	95,8%	91,3%
9386	40	35	50	80	59,8%	67,0%	91,9%	96,1%	91,8%
9387	40	35	55	20	48,4%	65,3%	87,0%	90,9%	82,6%
9388	40	35	55	25	48,9%	65,0%	87,3%	91,5%	83,5%
9389	40	35	55	30	49,3%	64,7%	87,7%	92,0%	84,5%
9390	40	35	55	35	49,8%	64,4%	88,1%	92,5%	85,3%
9391	40	35	55	40	50,2%	64,1%	88,4%	93,0%	86,2%
9392	40	35	55	45	50,6%	63,8%	88,8%	93,4%	87,0%
9393	40	35	55	50	51,1%	63,5%	89,1%	93,8%	87,7%
9394	40	35	55	55	51,5%	63,2%	89,4%	94,2%	88,4%
9395	40	35	55	60	52,0%	62,9%	89,8%	94,6%	89,1%
9396	40	35	55	65	52,4%	62,6%	90,1%	94,9%	89,8%
9397	40	35	55	70	52,8%	62,3%	90,4%	95,2%	90,4%
9398	40	35	55	75	53,3%	62,0%	90,7%	95,5%	91,0%
9399	40	35	55	80	53,7%	61,7%	90,9%	95,8%	91,5%
9400	40	35	60	20	42,3%	59,9%	85,5%	90,4%	82,0%
9401	40	35	60	25	42,8%	59,6%	85,9%	91,0%	83,0%
9402	40	35	60	30	43,2%	59,3%	86,3%	91,6%	83,9%
9403	40	35	60	35	43,6%	59,0%	86,7%	92,1%	84,8%
9404	40	35	60	40	44,1%	58,7%	87,1%	92,6%	85,7%

9405	40	35	60	45	44,5%	58,4%	87,5%	93,0%	86,5%
9406	40	35	60	50	44,9%	58,0%	87,8%	93,5%	87,3%
9407	40	35	60	55	45,4%	57,7%	88,2%	93,9%	88,0%
9408	40	35	60	60	45,8%	57,4%	88,6%	94,3%	88,7%
9409	40	35	60	65	46,2%	57,1%	88,9%	94,6%	89,4%
9410	40	35	60	70	46,7%	56,8%	89,2%	95,0%	90,0%
9411	40	35	60	75	47,1%	56,4%	89,5%	95,3%	90,6%
9412	40	35	60	80	47,6%	56,1%	89,9%	95,6%	91,2%
9413	40	35	65	20	36,4%	54,3%	83,9%	89,9%	81,3%
9414	40	35	65	25	36,9%	54,0%	84,3%	90,5%	82,4%
9415	40	35	65	30	37,3%	53,6%	84,8%	91,1%	83,3%
9416	40	35	65	35	37,7%	53,3%	85,2%	91,6%	84,3%
9417	40	35	65	40	38,1%	53,0%	85,6%	92,1%	85,2%
9418	40	35	65	45	38,5%	52,7%	86,1%	92,6%	86,0%
9419	40	35	65	50	38,9%	52,3%	86,5%	93,1%	86,8%
9420	40	35	65	55	39,3%	52,0%	86,8%	93,5%	87,6%
9421	40	35	65	60	39,8%	51,7%	87,2%	93,9%	88,3%
9422	40	35	65	65	40,2%	51,4%	87,6%	94,3%	89,0%
9423	40	35	65	70	40,6%	51,0%	88,0%	94,7%	89,6%
9424	40	35	65	75	41,0%	50,7%	88,3%	95,0%	90,3%
9425	40	35	65	80	41,5%	50,4%	88,7%	95,3%	90,8%
9426	40	35	70	20	30,9%	48,5%	82,1%	89,3%	80,7%
9427	40	35	70	25	31,3%	48,2%	82,6%	90,0%	81,8%
9428	40	35	70	30	31,7%	47,9%	83,1%	90,6%	82,8%
9429	40	35	70	35	32,1%	47,5%	83,6%	91,1%	83,7%
9430	40	35	70	40	32,5%	47,2%	84,0%	91,7%	84,6%
9431	40	35	70	45	32,9%	46,9%	84,5%	92,2%	85,5%
9432	40	35	70	50	33,2%	46,6%	84,9%	92,7%	86,3%
9433	40	35	70	55	33,6%	46,2%	85,4%	93,1%	87,1%
9434	40	35	70	60	34,0%	45,9%	85,8%	93,6%	87,9%
9435	40	35	70	65	34,4%	45,6%	86,2%	94,0%	88,6%
9436	40	35	70	70	34,8%	45,3%	86,6%	94,3%	89,3%
9437	40	35	70	75	35,2%	44,9%	87,0%	94,7%	89,9%

9438	40	35	70	80	35,6%	44,6%	87,4%	95,0%	90,5%
9439	40	35	75	20	25,9%	42,8%	80,2%	88,7%	80,1%
9440	40	35	75	25	26,3%	42,5%	80,8%	89,4%	81,1%
9441	40	35	75	30	26,6%	42,2%	81,3%	90,0%	82,2%
9442	40	35	75	35	27,0%	41,8%	81,8%	90,6%	83,1%
9443	40	35	75	40	27,3%	41,5%	82,3%	91,2%	84,1%
9444	40	35	75	45	27,7%	41,2%	82,8%	91,7%	85,0%
9445	40	35	75	50	28,0%	40,9%	83,3%	92,3%	85,8%
9446	40	35	75	55	28,4%	40,6%	83,7%	92,7%	86,6%
9447	40	35	75	60	28,7%	40,3%	84,2%	93,2%	87,4%
9448	40	35	75	65	29,1%	39,9%	84,6%	93,6%	88,2%
9449	40	35	75	70	29,4%	39,6%	85,1%	94,0%	88,8%
9450	40	35	75	75	29,8%	39,3%	85,5%	94,4%	89,5%
9451	40	35	75	80	30,2%	39,0%	85,9%	94,7%	90,1%
9452	40	35	80	20	21,5%	37,3%	78,2%	88,1%	79,4%
9453	40	35	80	25	21,8%	37,0%	78,8%	88,8%	80,5%
9454	40	35	80	30	22,1%	36,7%	79,3%	89,5%	81,5%
9455	40	35	80	35	22,4%	36,4%	79,9%	90,1%	82,6%
9456	40	35	80	40	22,7%	36,0%	80,4%	90,7%	83,5%
9457	40	35	80	45	23,0%	35,7%	81,0%	91,3%	84,4%
9458	40	35	80	50	23,3%	35,4%	81,5%	91,8%	85,3%
9459	40	35	80	55	23,6%	35,1%	82,0%	92,3%	86,2%
9460	40	35	80	60	23,9%	34,9%	82,5%	92,8%	87,0%
9461	40	35	80	65	24,3%	34,6%	83,0%	93,2%	87,7%
9462	40	35	80	70	24,6%	34,3%	83,4%	93,7%	88,4%
9463	40	35	80	75	24,9%	34,0%	83,9%	94,1%	89,1%
9464	40	35	80	80	25,2%	33,7%	84,4%	94,4%	89,8%
9465	40	40	20	20	85,3%	91,1%	93,9%	93,5%	85,2%
9466	40	40	20	25	85,5%	91,0%	94,1%	93,9%	86,0%
9467	40	40	20	30	85,7%	90,9%	94,3%	94,3%	86,8%
9468	40	40	20	35	85,9%	90,8%	94,5%	94,6%	87,6%
9469	40	40	20	40	86,1%	90,7%	94,6%	95,0%	88,3%
9470	40	40	20	45	86,3%	90,6%	94,8%	95,3%	89,0%

9471	40	40	20	50	86,5%	90,5%	95,0%	95,6%	89,7%
9472	40	40	20	55	86,7%	90,4%	95,1%	95,9%	90,3%
9473	40	40	20	60	86,9%	90,2%	95,3%	96,1%	90,9%
9474	40	40	20	65	87,1%	90,1%	95,4%	96,4%	91,4%
9475	40	40	20	70	87,3%	90,0%	95,6%	96,6%	91,9%
9476	40	40	20	75	87,5%	89,9%	95,7%	96,8%	92,4%
9477	40	40	20	80	87,7%	89,8%	95,9%	97,0%	92,9%
9478	40	40	25	20	81,9%	89,1%	93,2%	93,1%	84,6%
9479	40	40	25	25	82,1%	88,9%	93,4%	93,5%	85,5%
9480	40	40	25	30	82,4%	88,8%	93,6%	93,9%	86,3%
9481	40	40	25	35	82,6%	88,7%	93,8%	94,3%	87,1%
9482	40	40	25	40	82,9%	88,6%	94,0%	94,7%	87,9%
9483	40	40	25	45	83,1%	88,4%	94,2%	95,0%	88,6%
9484	40	40	25	50	83,4%	88,3%	94,4%	95,3%	89,3%
9485	40	40	25	55	83,6%	88,1%	94,5%	95,6%	89,9%
9486	40	40	25	60	83,9%	88,0%	94,7%	95,9%	90,5%
9487	40	40	25	65	84,1%	87,9%	94,9%	96,2%	91,1%
9488	40	40	25	70	84,3%	87,7%	95,0%	96,4%	91,6%
9489	40	40	25	75	84,6%	87,6%	95,2%	96,6%	92,1%
9490	40	40	25	80	84,8%	87,4%	95,3%	96,9%	92,6%
9491	40	40	30	20	77,9%	86,6%	92,3%	92,7%	84,1%
9492	40	40	30	25	78,2%	86,5%	92,6%	93,1%	85,0%
9493	40	40	30	30	78,5%	86,3%	92,8%	93,6%	85,9%
9494	40	40	30	35	78,8%	86,2%	93,0%	94,0%	86,7%
9495	40	40	30	40	79,1%	86,0%	93,2%	94,3%	87,4%
9496	40	40	30	45	79,4%	85,8%	93,4%	94,7%	88,2%
9497	40	40	30	50	79,7%	85,7%	93,7%	95,0%	88,9%
9498	40	40	30	55	80,0%	85,5%	93,9%	95,3%	89,5%
9499	40	40	30	60	80,2%	85,4%	94,0%	95,6%	90,2%
9500	40	40	30	65	80,5%	85,2%	94,2%	95,9%	90,7%
9501	40	40	30	70	80,8%	85,0%	94,4%	96,2%	91,3%
9502	40	40	30	75	81,1%	84,9%	94,6%	96,4%	91,8%
9503	40	40	30	80	81,3%	84,7%	94,8%	96,7%	92,3%

9504	40	40	35	20	73,4%	83,7%	91,4%	92,3%	83,5%
9505	40	40	35	25	73,7%	83,5%	91,7%	92,7%	84,5%
9506	40	40	35	30	74,1%	83,3%	91,9%	93,2%	85,3%
9507	40	40	35	35	74,4%	83,2%	92,2%	93,6%	86,2%
9508	40	40	35	40	74,7%	83,0%	92,4%	94,0%	87,0%
9509	40	40	35	45	75,1%	82,8%	92,6%	94,4%	87,7%
9510	40	40	35	50	75,4%	82,6%	92,9%	94,7%	88,5%
9511	40	40	35	55	75,7%	82,4%	93,1%	95,1%	89,1%
9512	40	40	35	60	76,0%	82,2%	93,3%	95,4%	89,8%
9513	40	40	35	65	76,4%	82,0%	93,5%	95,7%	90,4%
9514	40	40	35	70	76,7%	81,8%	93,7%	96,0%	91,0%
9515	40	40	35	75	77,0%	81,6%	93,9%	96,2%	91,5%
9516	40	40	35	80	77,3%	81,4%	94,1%	96,5%	92,0%
9517	40	40	40	20	68,3%	80,3%	90,4%	91,8%	83,0%
9518	40	40	40	25	68,7%	80,1%	90,7%	92,3%	83,9%
9519	40	40	40	30	69,0%	79,9%	90,9%	92,8%	84,8%
9520	40	40	40	35	69,4%	79,7%	91,2%	93,2%	85,7%
9521	40	40	40	40	69,8%	79,5%	91,5%	93,7%	86,5%
9522	40	40	40	45	70,2%	79,2%	91,8%	94,1%	87,3%
9523	40	40	40	50	70,5%	79,0%	92,0%	94,4%	88,0%
9524	40	40	40	55	70,9%	78,8%	92,3%	94,8%	88,7%
9525	40	40	40	60	71,3%	78,6%	92,5%	95,1%	89,4%
9526	40	40	40	65	71,6%	78,4%	92,7%	95,4%	90,0%
9527	40	40	40	70	72,0%	78,2%	92,9%	95,7%	90,6%
9528	40	40	40	75	72,3%	77,9%	93,2%	96,0%	91,2%
9529	40	40	40	80	72,7%	77,7%	93,4%	96,2%	91,7%
9530	40	40	45	20	62,7%	76,4%	89,2%	91,3%	82,4%
9531	40	40	45	25	63,1%	76,2%	89,6%	91,9%	83,4%
9532	40	40	45	30	63,5%	75,9%	89,9%	92,4%	84,3%
9533	40	40	45	35	63,9%	75,7%	90,2%	92,8%	85,2%
9534	40	40	45	40	64,4%	75,4%	90,5%	93,3%	86,0%
9535	40	40	45	45	64,8%	75,2%	90,8%	93,7%	86,8%
9536	40	40	45	50	65,2%	74,9%	91,0%	94,1%	87,6%

9537	40	40	45	55	65,6%	74,7%	91,3%	94,5%	88,3%
9538	40	40	45	60	66,0%	74,5%	91,6%	94,8%	89,0%
9539	40	40	45	65	66,3%	74,2%	91,8%	95,1%	89,6%
9540	40	40	45	70	66,7%	74,0%	92,1%	95,5%	90,3%
9541	40	40	45	75	67,1%	73,7%	92,3%	95,7%	90,9%
9542	40	40	45	80	67,5%	73,4%	92,6%	96,0%	91,4%
9543	40	40	50	20	56,8%	72,0%	88,0%	90,8%	81,8%
9544	40	40	50	25	57,2%	71,7%	88,3%	91,4%	82,8%
9545	40	40	50	30	57,7%	71,4%	88,7%	91,9%	83,7%
9546	40	40	50	35	58,1%	71,2%	89,0%	92,4%	84,6%
9547	40	40	50	40	58,5%	70,9%	89,3%	92,9%	85,5%
9548	40	40	50	45	58,9%	70,6%	89,7%	93,3%	86,3%
9549	40	40	50	50	59,4%	70,4%	90,0%	93,7%	87,1%
9550	40	40	50	55	59,8%	70,1%	90,3%	94,1%	87,9%
9551	40	40	50	60	60,2%	69,8%	90,6%	94,5%	88,6%
9552	40	40	50	65	60,6%	69,5%	90,9%	94,9%	89,3%
9553	40	40	50	70	61,1%	69,3%	91,1%	95,2%	89,9%
9554	40	40	50	75	61,5%	69,0%	91,4%	95,5%	90,5%
9555	40	40	50	80	61,9%	68,7%	91,7%	95,8%	91,1%
9556	40	40	55	20	50,7%	67,1%	86,6%	90,3%	81,2%
9557	40	40	55	25	51,1%	66,8%	87,0%	90,9%	82,2%
9558	40	40	55	30	51,5%	66,5%	87,4%	91,5%	83,2%
9559	40	40	55	35	52,0%	66,2%	87,7%	92,0%	84,1%
9560	40	40	55	40	52,4%	65,9%	88,1%	92,5%	85,0%
9561	40	40	55	45	52,9%	65,6%	88,5%	92,9%	85,8%
9562	40	40	55	50	53,3%	65,3%	88,8%	93,4%	86,7%
9563	40	40	55	55	53,7%	65,0%	89,1%	93,8%	87,4%
9564	40	40	55	60	54,2%	64,7%	89,5%	94,2%	88,2%
9565	40	40	55	65	54,6%	64,4%	89,8%	94,6%	88,9%
9566	40	40	55	70	55,0%	64,1%	90,1%	94,9%	89,5%
9567	40	40	55	75	55,5%	63,8%	90,4%	95,2%	90,1%
9568	40	40	55	80	55,9%	63,5%	90,7%	95,5%	90,7%
9569	40	40	60	20	44,5%	61,8%	85,1%	89,8%	80,5%

9570	40	40	60	25	44,9%	61,5%	85,5%	90,4%	81,6%
9571	40	40	60	30	45,4%	61,2%	85,9%	91,0%	82,6%
9572	40	40	60	35	45,8%	60,9%	86,3%	91,5%	83,5%
9573	40	40	60	40	46,3%	60,6%	86,7%	92,0%	84,5%
9574	40	40	60	45	46,7%	60,3%	87,1%	92,5%	85,3%
9575	40	40	60	50	47,1%	59,9%	87,5%	93,0%	86,2%
9576	40	40	60	55	47,6%	59,6%	87,9%	93,4%	87,0%
9577	40	40	60	60	48,0%	59,3%	88,2%	93,8%	87,7%
9578	40	40	60	65	48,5%	59,0%	88,6%	94,2%	88,4%
9579	40	40	60	70	48,9%	58,7%	88,9%	94,6%	89,1%
9580	40	40	60	75	49,3%	58,4%	89,2%	94,9%	89,8%
9581	40	40	60	80	49,8%	58,0%	89,6%	95,3%	90,4%
9582	40	40	65	20	38,5%	56,2%	83,5%	89,2%	79,9%
9583	40	40	65	25	38,9%	55,9%	83,9%	89,8%	80,9%
9584	40	40	65	30	39,4%	55,6%	84,4%	90,5%	82,0%
9585	40	40	65	35	39,8%	55,3%	84,8%	91,0%	83,0%
9586	40	40	65	40	40,2%	54,9%	85,2%	91,6%	83,9%
9587	40	40	65	45	40,6%	54,6%	85,7%	92,1%	84,8%
9588	40	40	65	50	41,1%	54,3%	86,1%	92,6%	85,7%
9589	40	40	65	55	41,5%	54,0%	86,5%	93,0%	86,5%
9590	40	40	65	60	41,9%	53,6%	86,9%	93,5%	87,3%
9591	40	40	65	65	42,3%	53,3%	87,3%	93,9%	88,0%
9592	40	40	65	70	42,8%	53,0%	87,6%	94,3%	88,7%
9593	40	40	65	75	43,2%	52,7%	88,0%	94,6%	89,4%
9594	40	40	65	80	43,6%	52,3%	88,3%	95,0%	90,0%
9595	40	40	70	20	32,9%	50,5%	81,7%	88,6%	79,2%
9596	40	40	70	25	33,3%	50,2%	82,2%	89,3%	80,3%
9597	40	40	70	30	33,6%	49,8%	82,7%	89,9%	81,4%
9598	40	40	70	35	34,0%	49,5%	83,1%	90,5%	82,4%
9599	40	40	70	40	34,4%	49,2%	83,6%	91,1%	83,3%
9600	40	40	70	45	34,8%	48,9%	84,1%	91,6%	84,3%
9601	40	40	70	50	35,2%	48,5%	84,5%	92,2%	85,2%
9602	40	40	70	55	35,6%	48,2%	85,0%	92,6%	86,0%

9603	40	40	70	60	36,0%	47,9%	85,4%	93,1%	86,8%
9604	40	40	70	65	36,5%	47,6%	85,8%	93,5%	87,6%
9605	40	40	70	70	36,9%	47,2%	86,2%	93,9%	88,3%
9606	40	40	70	75	37,3%	46,9%	86,6%	94,3%	89,0%
9607	40	40	70	80	37,7%	46,6%	87,0%	94,7%	89,6%
9608	40	40	75	20	27,7%	44,7%	79,7%	88,0%	78,5%
9609	40	40	75	25	28,0%	44,4%	80,3%	88,7%	79,6%
9610	40	40	75	30	28,4%	44,1%	80,8%	89,4%	80,7%
9611	40	40	75	35	28,7%	43,8%	81,3%	90,0%	81,8%
9612	40	40	75	40	29,1%	43,4%	81,8%	90,6%	82,8%
9613	40	40	75	45	29,5%	43,1%	82,3%	91,2%	83,7%
9614	40	40	75	50	29,8%	42,8%	82,8%	91,7%	84,6%
9615	40	40	75	55	30,2%	42,5%	83,3%	92,2%	85,5%
9616	40	40	75	60	30,6%	42,2%	83,8%	92,7%	86,3%
9617	40	40	75	65	30,9%	41,8%	84,2%	93,1%	87,1%
9618	40	40	75	70	31,3%	41,5%	84,7%	93,6%	87,9%
9619	40	40	75	75	31,7%	41,2%	85,1%	94,0%	88,6%
9620	40	40	75	80	32,1%	40,9%	85,5%	94,4%	89,3%
9621	40	40	80	20	23,0%	39,1%	77,6%	87,3%	77,8%
9622	40	40	80	25	23,3%	38,8%	78,2%	88,0%	79,0%
9623	40	40	80	30	23,6%	38,5%	78,8%	88,8%	80,1%
9624	40	40	80	35	24,0%	38,2%	79,4%	89,4%	81,1%
9625	40	40	80	40	24,3%	37,9%	79,9%	90,1%	82,2%
9626	40	40	80	45	24,6%	37,6%	80,5%	90,7%	83,1%
9627	40	40	80	50	24,9%	37,3%	81,0%	91,2%	84,1%
9628	40	40	80	55	25,3%	37,0%	81,5%	91,8%	85,0%
9629	40	40	80	60	25,6%	36,7%	82,0%	92,3%	85,8%
9630	40	40	80	65	25,9%	36,4%	82,5%	92,7%	86,6%
9631	40	40	80	70	26,3%	36,1%	83,0%	93,2%	87,4%
9632	40	40	80	75	26,6%	35,8%	83,5%	93,6%	88,2%
9633	40	40	80	80	27,0%	35,5%	83,9%	94,0%	88,9%
9634	40	45	20	20	86,3%	91,7%	93,7%	93,0%	83,9%
9635	40	45	20	25	86,5%	91,6%	93,9%	93,4%	84,8%

9636	40	45	20	30	86,7%	91,5%	94,1%	93,8%	85,7%
9637	40	45	20	35	86,9%	91,4%	94,3%	94,2%	86,5%
9638	40	45	20	40	87,1%	91,3%	94,5%	94,6%	87,3%
9639	40	45	20	45	87,3%	91,2%	94,7%	94,9%	88,0%
9640	40	45	20	50	87,5%	91,1%	94,8%	95,3%	88,7%
9641	40	45	20	55	87,7%	91,0%	95,0%	95,6%	89,4%
9642	40	45	20	60	87,9%	90,9%	95,1%	95,8%	90,0%
9643	40	45	20	65	88,1%	90,8%	95,3%	96,1%	90,6%
9644	40	45	20	70	88,3%	90,7%	95,5%	96,4%	91,2%
9645	40	45	20	75	88,5%	90,6%	95,6%	96,6%	91,7%
9646	40	45	20	80	88,6%	90,5%	95,7%	96,8%	92,2%
9647	40	45	25	20	83,2%	89,8%	93,0%	92,6%	83,4%
9648	40	45	25	25	83,4%	89,7%	93,2%	93,0%	84,3%
9649	40	45	25	30	83,6%	89,6%	93,4%	93,5%	85,2%
9650	40	45	25	35	83,9%	89,5%	93,6%	93,9%	86,0%
9651	40	45	25	40	84,1%	89,3%	93,8%	94,3%	86,8%
9652	40	45	25	45	84,4%	89,2%	94,0%	94,6%	87,6%
9653	40	45	25	50	84,6%	89,1%	94,2%	95,0%	88,3%
9654	40	45	25	55	84,8%	88,9%	94,4%	95,3%	89,0%
9655	40	45	25	60	85,0%	88,8%	94,5%	95,6%	89,7%
9656	40	45	25	65	85,3%	88,7%	94,7%	95,9%	90,3%
9657	40	45	25	70	85,5%	88,6%	94,9%	96,1%	90,9%
9658	40	45	25	75	85,7%	88,4%	95,0%	96,4%	91,4%
9659	40	45	25	80	85,9%	88,3%	95,2%	96,6%	91,9%
9660	40	45	30	20	79,4%	87,5%	92,1%	92,2%	82,8%
9661	40	45	30	25	79,7%	87,4%	92,3%	92,6%	83,7%
9662	40	45	30	30	80,0%	87,2%	92,6%	93,1%	84,7%
9663	40	45	30	35	80,3%	87,1%	92,8%	93,5%	85,5%
9664	40	45	30	40	80,5%	86,9%	93,0%	93,9%	86,3%
9665	40	45	30	45	80,8%	86,8%	93,2%	94,3%	87,1%
9666	40	45	30	50	81,1%	86,6%	93,5%	94,7%	87,9%
9667	40	45	30	55	81,4%	86,5%	93,7%	95,0%	88,6%
9668	40	45	30	60	81,6%	86,3%	93,9%	95,3%	89,3%

9669	40	45	30	65	81,9%	86,2%	94,1%	95,6%	89,9%
9670	40	45	30	70	82,1%	86,0%	94,2%	95,9%	90,5%
9671	40	45	30	75	82,4%	85,8%	94,4%	96,2%	91,1%
9672	40	45	30	80	82,7%	85,7%	94,6%	96,4%	91,6%
9673	40	45	35	20	75,1%	84,7%	91,1%	91,7%	82,2%
9674	40	45	35	25	75,4%	84,6%	91,4%	92,2%	83,2%
9675	40	45	35	30	75,7%	84,4%	91,7%	92,7%	84,1%
9676	40	45	35	35	76,1%	84,2%	91,9%	93,1%	85,0%
9677	40	45	35	40	76,4%	84,1%	92,2%	93,6%	85,9%
9678	40	45	35	45	76,7%	83,9%	92,4%	94,0%	86,7%
9679	40	45	35	50	77,0%	83,7%	92,7%	94,4%	87,4%
9680	40	45	35	55	77,3%	83,5%	92,9%	94,7%	88,2%
9681	40	45	35	60	77,6%	83,3%	93,1%	95,0%	88,9%
9682	40	45	35	65	77,9%	83,2%	93,3%	95,4%	89,5%
9683	40	45	35	70	78,2%	83,0%	93,5%	95,7%	90,2%
9684	40	45	35	75	78,5%	82,8%	93,7%	95,9%	90,7%
9685	40	45	35	80	78,8%	82,6%	93,9%	96,2%	91,3%
9686	40	45	40	20	70,2%	81,5%	90,1%	91,2%	81,6%
9687	40	45	40	25	70,5%	81,3%	90,4%	91,8%	82,6%
9688	40	45	40	30	70,9%	81,1%	90,7%	92,3%	83,5%
9689	40	45	40	35	71,3%	80,9%	91,0%	92,7%	84,5%
9690	40	45	40	40	71,6%	80,7%	91,2%	93,2%	85,3%
9691	40	45	40	45	72,0%	80,5%	91,5%	93,6%	86,2%
9692	40	45	40	50	72,3%	80,3%	91,8%	94,0%	87,0%
9693	40	45	40	55	72,7%	80,1%	92,0%	94,4%	87,7%
9694	40	45	40	60	73,0%	79,9%	92,3%	94,8%	88,5%
9695	40	45	40	65	73,4%	79,7%	92,5%	95,1%	89,1%
9696	40	45	40	70	73,7%	79,5%	92,7%	95,4%	89,8%
9697	40	45	40	75	74,1%	79,3%	93,0%	95,7%	90,4%
9698	40	45	40	80	74,4%	79,0%	93,2%	96,0%	91,0%
9699	40	45	45	20	64,8%	77,8%	88,9%	90,7%	81,0%
9700	40	45	45	25	65,2%	77,6%	89,3%	91,3%	82,0%
9701	40	45	45	30	65,6%	77,3%	89,6%	91,8%	83,0%

9702	40	45	45	35	66,0%	77,1%	89,9%	92,3%	83,9%
9703	40	45	45	40	66,4%	76,9%	90,2%	92,8%	84,8%
9704	40	45	45	45	66,8%	76,6%	90,5%	93,2%	85,7%
9705	40	45	45	50	67,1%	76,4%	90,8%	93,7%	86,5%
9706	40	45	45	55	67,5%	76,2%	91,1%	94,1%	87,3%
9707	40	45	45	60	67,9%	75,9%	91,3%	94,4%	88,0%
9708	40	45	45	65	68,3%	75,7%	91,6%	94,8%	88,7%
9709	40	45	45	70	68,7%	75,4%	91,9%	95,1%	89,4%
9710	40	45	45	75	69,1%	75,2%	92,1%	95,4%	90,0%
9711	40	45	45	80	69,4%	75,0%	92,3%	95,7%	90,6%
9712	40	45	50	20	59,0%	73,5%	87,6%	90,2%	80,3%
9713	40	45	50	25	59,4%	73,3%	88,0%	90,8%	81,4%
9714	40	45	50	30	59,8%	73,0%	88,4%	91,4%	82,4%
9715	40	45	50	35	60,2%	72,8%	88,7%	91,9%	83,4%
9716	40	45	50	40	60,6%	72,5%	89,0%	92,4%	84,3%
9717	40	45	50	45	61,1%	72,2%	89,4%	92,9%	85,2%
9718	40	45	50	50	61,5%	72,0%	89,7%	93,3%	86,0%
9719	40	45	50	55	61,9%	71,7%	90,0%	93,7%	86,8%
9720	40	45	50	60	62,3%	71,5%	90,3%	94,1%	87,6%
9721	40	45	50	65	62,7%	71,2%	90,6%	94,5%	88,3%
9722	40	45	50	70	63,1%	70,9%	90,9%	94,8%	89,0%
9723	40	45	50	75	63,5%	70,6%	91,2%	95,2%	89,7%
9724	40	45	50	80	64,0%	70,4%	91,4%	95,5%	90,3%
9725	40	45	55	20	52,9%	68,8%	86,2%	89,6%	79,6%
9726	40	45	55	25	53,3%	68,5%	86,6%	90,3%	80,7%
9727	40	45	55	30	53,8%	68,2%	87,0%	90,9%	81,8%
9728	40	45	55	35	54,2%	68,0%	87,4%	91,4%	82,8%
9729	40	45	55	40	54,6%	67,7%	87,8%	91,9%	83,7%
9730	40	45	55	45	55,1%	67,4%	88,1%	92,4%	84,6%
9731	40	45	55	50	55,5%	67,1%	88,5%	92,9%	85,5%
9732	40	45	55	55	55,9%	66,8%	88,8%	93,3%	86,3%
9733	40	45	55	60	56,4%	66,5%	89,2%	93,8%	87,1%
9734	40	45	55	65	56,8%	66,2%	89,5%	94,2%	87,9%

9735	40	45	55	70	57,2%	65,9%	89,8%	94,5%	88,6%
9736	40	45	55	75	57,7%	65,6%	90,1%	94,9%	89,3%
9737	40	45	55	80	58,1%	65,3%	90,4%	95,2%	89,9%
9738	40	45	60	20	46,7%	63,7%	84,7%	89,1%	79,0%
9739	40	45	60	25	47,1%	63,3%	85,1%	89,7%	80,1%
9740	40	45	60	30	47,6%	63,0%	85,6%	90,3%	81,2%
9741	40	45	60	35	48,0%	62,7%	86,0%	90,9%	82,2%
9742	40	45	60	40	48,5%	62,4%	86,4%	91,5%	83,2%
9743	40	45	60	45	48,9%	62,1%	86,8%	92,0%	84,1%
9744	40	45	60	50	49,3%	61,8%	87,1%	92,5%	85,0%
9745	40	45	60	55	49,8%	61,5%	87,5%	93,0%	85,8%
9746	40	45	60	60	50,2%	61,2%	87,9%	93,4%	86,7%
9747	40	45	60	65	50,7%	60,9%	88,2%	93,8%	87,4%
9748	40	45	60	70	51,1%	60,6%	88,6%	94,2%	88,2%
9749	40	45	60	75	51,6%	60,3%	88,9%	94,6%	88,9%
9750	40	45	60	80	52,0%	59,9%	89,3%	94,9%	89,5%
9751	40	45	65	20	40,6%	58,2%	83,0%	88,5%	78,3%
9752	40	45	65	25	41,1%	57,8%	83,5%	89,1%	79,4%
9753	40	45	65	30	41,5%	57,5%	83,9%	89,8%	80,5%
9754	40	45	65	35	41,9%	57,2%	84,4%	90,4%	81,6%
9755	40	45	65	40	42,4%	56,9%	84,8%	91,0%	82,6%
9756	40	45	65	45	42,8%	56,6%	85,3%	91,5%	83,5%
9757	40	45	65	50	43,2%	56,2%	85,7%	92,1%	84,5%
9758	40	45	65	55	43,7%	55,9%	86,1%	92,6%	85,3%
9759	40	45	65	60	44,1%	55,6%	86,5%	93,0%	86,2%
9760	40	45	65	65	44,5%	55,3%	86,9%	93,4%	87,0%
9761	40	45	65	70	45,0%	54,9%	87,3%	93,9%	87,7%
9762	40	45	65	75	45,4%	54,6%	87,7%	94,2%	88,4%
9763	40	45	65	80	45,8%	54,3%	88,0%	94,6%	89,1%
9764	40	45	70	20	34,8%	52,5%	81,2%	87,8%	77,6%
9765	40	45	70	25	35,3%	52,1%	81,7%	88,5%	78,7%
9766	40	45	70	30	35,7%	51,8%	82,2%	89,2%	79,9%
9767	40	45	70	35	36,1%	51,5%	82,7%	89,9%	80,9%

9768	40	45	70	40	36,5%	51,2%	83,2%	90,5%	82,0%
9769	40	45	70	45	36,9%	50,8%	83,6%	91,1%	83,0%
9770	40	45	70	50	37,3%	50,5%	84,1%	91,6%	83,9%
9771	40	45	70	55	37,7%	50,2%	84,6%	92,1%	84,8%
9772	40	45	70	60	38,1%	49,8%	85,0%	92,6%	85,7%
9773	40	45	70	65	38,5%	49,5%	85,4%	93,1%	86,5%
9774	40	45	70	70	39,0%	49,2%	85,8%	93,5%	87,3%
9775	40	45	70	75	39,4%	48,9%	86,2%	93,9%	88,0%
9776	40	45	70	80	39,8%	48,5%	86,6%	94,3%	88,7%
9777	40	45	75	20	29,5%	46,7%	79,2%	87,2%	76,8%
9778	40	45	75	25	29,8%	46,4%	79,8%	87,9%	78,0%
9779	40	45	75	30	30,2%	46,0%	80,3%	88,6%	79,2%
9780	40	45	75	35	30,6%	45,7%	80,8%	89,3%	80,3%
9781	40	45	75	40	31,0%	45,4%	81,4%	89,9%	81,4%
9782	40	45	75	45	31,3%	45,1%	81,9%	90,5%	82,4%
9783	40	45	75	50	31,7%	44,7%	82,4%	91,1%	83,3%
9784	40	45	75	55	32,1%	44,4%	82,9%	91,7%	84,3%
9785	40	45	75	60	32,5%	44,1%	83,3%	92,2%	85,2%
9786	40	45	75	65	32,9%	43,8%	83,8%	92,7%	86,0%
9787	40	45	75	70	33,3%	43,5%	84,3%	93,1%	86,8%
9788	40	45	75	75	33,7%	43,1%	84,7%	93,5%	87,6%
9789	40	45	75	80	34,1%	42,8%	85,1%	93,9%	88,3%
9790	40	45	80	20	24,6%	41,0%	77,1%	86,5%	76,1%
9791	40	45	80	25	24,9%	40,7%	77,7%	87,2%	77,3%
9792	40	45	80	30	25,3%	40,4%	78,3%	88,0%	78,5%
9793	40	45	80	35	25,6%	40,1%	78,8%	88,7%	79,6%
9794	40	45	80	40	25,9%	39,8%	79,4%	89,4%	80,7%
9795	40	45	80	45	26,3%	39,4%	80,0%	90,0%	81,8%
9796	40	45	80	50	26,6%	39,1%	80,5%	90,6%	82,8%
9797	40	45	80	55	27,0%	38,8%	81,0%	91,2%	83,7%
9798	40	45	80	60	27,3%	38,5%	81,5%	91,7%	84,6%
9799	40	45	80	65	27,7%	38,2%	82,0%	92,2%	85,5%
9800	40	45	80	70	28,0%	37,9%	82,5%	92,7%	86,3%

9801	40	45	80	75	28,4%	37,6%	83,0%	93,2%	87,1%
9802	40	45	80	80	28,7%	37,3%	83,5%	93,6%	87,9%
9803	40	50	20	20	87,3%	92,3%	93,5%	92,5%	82,6%
9804	40	50	20	25	87,5%	92,2%	93,7%	93,0%	83,6%
9805	40	50	20	30	87,7%	92,1%	93,9%	93,4%	84,5%
9806	40	50	20	35	87,9%	92,0%	94,1%	93,8%	85,4%
9807	40	50	20	40	88,1%	91,9%	94,3%	94,2%	86,2%
9808	40	50	20	45	88,3%	91,8%	94,5%	94,6%	87,0%
9809	40	50	20	50	88,5%	91,7%	94,7%	94,9%	87,7%
9810	40	50	20	55	88,6%	91,6%	94,8%	95,2%	88,5%
9811	40	50	20	60	88,8%	91,5%	95,0%	95,5%	89,1%
9812	40	50	20	65	89,0%	91,4%	95,2%	95,8%	89,8%
9813	40	50	20	70	89,2%	91,3%	95,3%	96,1%	90,4%
9814	40	50	20	75	89,3%	91,2%	95,5%	96,3%	91,0%
9815	40	50	20	80	89,5%	91,1%	95,6%	96,6%	91,5%
9816	40	50	25	20	84,4%	90,5%	92,7%	92,1%	82,0%
9817	40	50	25	25	84,6%	90,4%	93,0%	92,6%	83,0%
9818	40	50	25	30	84,8%	90,3%	93,2%	93,0%	83,9%
9819	40	50	25	35	85,0%	90,2%	93,4%	93,4%	84,8%
9820	40	50	25	40	85,3%	90,1%	93,6%	93,9%	85,7%
9821	40	50	25	45	85,5%	89,9%	93,8%	94,2%	86,5%
9822	40	50	25	50	85,7%	89,8%	94,0%	94,6%	87,3%
9823	40	50	25	55	85,9%	89,7%	94,2%	94,9%	88,0%
9824	40	50	25	60	86,1%	89,6%	94,4%	95,3%	88,7%
9825	40	50	25	65	86,3%	89,5%	94,6%	95,6%	89,4%
9826	40	50	25	70	86,5%	89,3%	94,7%	95,9%	90,0%
9827	40	50	25	75	86,8%	89,2%	94,9%	96,1%	90,6%
9828	40	50	25	80	87,0%	89,1%	95,1%	96,4%	91,2%
9829	40	50	30	20	80,8%	88,3%	91,9%	91,6%	81,4%
9830	40	50	30	25	81,1%	88,2%	92,1%	92,1%	82,4%
9831	40	50	30	30	81,4%	88,1%	92,4%	92,6%	83,4%
9832	40	50	30	35	81,6%	87,9%	92,6%	93,1%	84,3%
9833	40	50	30	40	81,9%	87,8%	92,8%	93,5%	85,2%

9834	40	50	30	45	82,1%	87,6%	93,0%	93,9%	86,0%
9835	40	50	30	50	82,4%	87,5%	93,3%	94,3%	86,8%
9836	40	50	30	55	82,7%	87,4%	93,5%	94,6%	87,6%
9837	40	50	30	60	82,9%	87,2%	93,7%	95,0%	88,3%
9838	40	50	30	65	83,2%	87,1%	93,9%	95,3%	89,0%
9839	40	50	30	70	83,4%	86,9%	94,1%	95,6%	89,7%
9840	40	50	30	75	83,6%	86,8%	94,3%	95,9%	90,3%
9841	40	50	30	80	83,9%	86,6%	94,4%	96,1%	90,9%
9842	40	50	35	20	76,7%	85,7%	90,9%	91,1%	80,7%
9843	40	50	35	25	77,0%	85,6%	91,2%	91,7%	81,8%
9844	40	50	35	30	77,3%	85,4%	91,4%	92,2%	82,8%
9845	40	50	35	35	77,6%	85,3%	91,7%	92,7%	83,7%
9846	40	50	35	40	77,9%	85,1%	92,0%	93,1%	84,7%
9847	40	50	35	45	78,2%	84,9%	92,2%	93,5%	85,5%
9848	40	50	35	50	78,5%	84,8%	92,4%	93,9%	86,4%
9849	40	50	35	55	78,8%	84,6%	92,7%	94,3%	87,1%
9850	40	50	35	60	79,1%	84,4%	92,9%	94,7%	87,9%
9851	40	50	35	65	79,4%	84,2%	93,1%	95,0%	88,6%
9852	40	50	35	70	79,7%	84,1%	93,3%	95,3%	89,3%
9853	40	50	35	75	80,0%	83,9%	93,5%	95,6%	89,9%
9854	40	50	35	80	80,3%	83,7%	93,7%	95,9%	90,5%
9855	40	50	40	20	72,0%	82,7%	89,8%	90,6%	80,1%
9856	40	50	40	25	72,4%	82,5%	90,1%	91,2%	81,2%
9857	40	50	40	30	72,7%	82,3%	90,4%	91,7%	82,2%
9858	40	50	40	35	73,1%	82,1%	90,7%	92,2%	83,2%
9859	40	50	40	40	73,4%	81,9%	91,0%	92,7%	84,1%
9860	40	50	40	45	73,7%	81,7%	91,3%	93,2%	85,0%
9861	40	50	40	50	74,1%	81,5%	91,5%	93,6%	85,9%
9862	40	50	40	55	74,4%	81,3%	91,8%	94,0%	86,7%
9863	40	50	40	60	74,8%	81,1%	92,0%	94,4%	87,4%
9864	40	50	40	65	75,1%	80,9%	92,3%	94,7%	88,2%
9865	40	50	40	70	75,4%	80,7%	92,5%	95,1%	88,9%
9866	40	50	40	75	75,7%	80,5%	92,8%	95,4%	89,5%

9867	40	50	40	80	76,1%	80,3%	93,0%	95,7%	90,2%
9868	40	50	45	20	66,8%	79,1%	88,6%	90,1%	79,4%
9869	40	50	45	25	67,2%	78,9%	88,9%	90,7%	80,5%
9870	40	50	45	30	67,5%	78,7%	89,3%	91,3%	81,6%
9871	40	50	45	35	67,9%	78,5%	89,6%	91,8%	82,6%
9872	40	50	45	40	68,3%	78,2%	89,9%	92,3%	83,6%
9873	40	50	45	45	68,7%	78,0%	90,2%	92,8%	84,5%
9874	40	50	45	50	69,1%	77,8%	90,5%	93,2%	85,4%
9875	40	50	45	55	69,4%	77,6%	90,8%	93,6%	86,2%
9876	40	50	45	60	69,8%	77,3%	91,1%	94,0%	87,0%
9877	40	50	45	65	70,2%	77,1%	91,3%	94,4%	87,7%
9878	40	50	45	70	70,6%	76,9%	91,6%	94,8%	88,5%
9879	40	50	45	75	70,9%	76,6%	91,9%	95,1%	89,1%
9880	40	50	45	80	71,3%	76,4%	92,1%	95,4%	89,8%
9881	40	50	50	20	61,1%	75,0%	87,3%	89,5%	78,8%
9882	40	50	50	25	61,5%	74,8%	87,7%	90,2%	79,9%
9883	40	50	50	30	61,9%	74,6%	88,0%	90,8%	81,0%
9884	40	50	50	35	62,3%	74,3%	88,4%	91,3%	82,0%
9885	40	50	50	40	62,7%	74,1%	88,7%	91,8%	83,0%
9886	40	50	50	45	63,2%	73,8%	89,1%	92,3%	83,9%
9887	40	50	50	50	63,6%	73,5%	89,4%	92,8%	84,8%
9888	40	50	50	55	64,0%	73,3%	89,7%	93,3%	85,7%
9889	40	50	50	60	64,4%	73,0%	90,0%	93,7%	86,5%
9890	40	50	50	65	64,8%	72,8%	90,3%	94,1%	87,3%
9891	40	50	50	70	65,2%	72,5%	90,6%	94,5%	88,0%
9892	40	50	50	75	65,6%	72,3%	90,9%	94,8%	88,7%
9893	40	50	50	80	66,0%	72,0%	91,2%	95,1%	89,4%
9894	40	50	55	20	55,1%	70,5%	85,9%	88,9%	78,1%
9895	40	50	55	25	55,5%	70,2%	86,3%	89,6%	79,2%
9896	40	50	55	30	55,9%	69,9%	86,7%	90,2%	80,3%
9897	40	50	55	35	56,4%	69,7%	87,0%	90,8%	81,4%
9898	40	50	55	40	56,8%	69,4%	87,4%	91,4%	82,4%
9899	40	50	55	45	57,2%	69,1%	87,8%	91,9%	83,4%

9900	40	50	55	50	57,7%	68,8%	88,2%	92,4%	84,3%
9901	40	50	55	55	58,1%	68,5%	88,5%	92,9%	85,2%
9902	40	50	55	60	58,5%	68,3%	88,8%	93,3%	86,0%
9903	40	50	55	65	59,0%	68,0%	89,2%	93,7%	86,8%
9904	40	50	55	70	59,4%	67,7%	89,5%	94,1%	87,6%
9905	40	50	55	75	59,8%	67,4%	89,8%	94,5%	88,3%
9906	40	50	55	80	60,2%	67,1%	90,1%	94,8%	89,0%
9907	40	50	60	20	48,9%	65,5%	84,3%	88,3%	77,3%
9908	40	50	60	25	49,4%	65,2%	84,7%	89,0%	78,5%
9909	40	50	60	30	49,8%	64,9%	85,2%	89,7%	79,7%
9910	40	50	60	35	50,2%	64,6%	85,6%	90,3%	80,7%
9911	40	50	60	40	50,7%	64,3%	86,0%	90,9%	81,8%
9912	40	50	60	45	51,1%	64,0%	86,4%	91,4%	82,8%
9913	40	50	60	50	51,6%	63,7%	86,8%	92,0%	83,7%
9914	40	50	60	55	52,0%	63,4%	87,2%	92,5%	84,6%
9915	40	50	60	60	52,4%	63,1%	87,5%	92,9%	85,5%
9916	40	50	60	65	52,9%	62,7%	87,9%	93,4%	86,3%
9917	40	50	60	70	53,3%	62,4%	88,3%	93,8%	87,1%
9918	40	50	60	75	53,8%	62,1%	88,6%	94,2%	87,9%
9919	40	50	60	80	54,2%	61,8%	89,0%	94,5%	88,6%
9920	40	50	65	20	42,8%	60,1%	82,6%	87,7%	76,6%
9921	40	50	65	25	43,2%	59,8%	83,0%	88,4%	77,8%
9922	40	50	65	30	43,7%	59,4%	83,5%	89,1%	79,0%
9923	40	50	65	35	44,1%	59,1%	84,0%	89,8%	80,1%
9924	40	50	65	40	44,5%	58,8%	84,4%	90,4%	81,2%
9925	40	50	65	45	45,0%	58,5%	84,9%	91,0%	82,2%
9926	40	50	65	50	45,4%	58,2%	85,3%	91,5%	83,2%
9927	40	50	65	55	45,8%	57,8%	85,7%	92,0%	84,1%
9928	40	50	65	60	46,3%	57,5%	86,1%	92,5%	85,0%
9929	40	50	65	65	46,7%	57,2%	86,5%	93,0%	85,8%
9930	40	50	65	70	47,2%	56,9%	86,9%	93,4%	86,7%
9931	40	50	65	75	47,6%	56,6%	87,3%	93,8%	87,4%
9932	40	50	65	80	48,0%	56,2%	87,7%	94,2%	88,2%

9933	40	50	70	20	36,9%	54,4%	80,7%	87,0%	75,9%
9934	40	50	70	25	37,3%	54,1%	81,2%	87,8%	77,1%
9935	40	50	70	30	37,7%	53,8%	81,7%	88,5%	78,3%
9936	40	50	70	35	38,1%	53,4%	82,2%	89,2%	79,4%
9937	40	50	70	40	38,5%	53,1%	82,7%	89,8%	80,5%
9938	40	50	70	45	39,0%	52,8%	83,2%	90,4%	81,6%
9939	40	50	70	50	39,4%	52,5%	83,7%	91,0%	82,6%
9940	40	50	70	55	39,8%	52,1%	84,1%	91,6%	83,5%
9941	40	50	70	60	40,2%	51,8%	84,6%	92,1%	84,5%
9942	40	50	70	65	40,7%	51,5%	85,0%	92,6%	85,3%
9943	40	50	70	70	41,1%	51,2%	85,4%	93,0%	86,2%
9944	40	50	70	75	41,5%	50,8%	85,9%	93,5%	87,0%
9945	40	50	70	80	41,9%	50,5%	86,3%	93,9%	87,7%
9946	40	50	75	20	31,3%	48,7%	78,7%	86,3%	75,1%
9947	40	50	75	25	31,7%	48,3%	79,2%	87,1%	76,4%
9948	40	50	75	30	32,1%	48,0%	79,8%	87,9%	77,6%
9949	40	50	75	35	32,5%	47,7%	80,3%	88,6%	78,7%
9950	40	50	75	40	32,9%	47,4%	80,9%	89,3%	79,9%
9951	40	50	75	45	33,3%	47,0%	81,4%	89,9%	80,9%
9952	40	50	75	50	33,7%	46,7%	81,9%	90,5%	82,0%
9953	40	50	75	55	34,1%	46,4%	82,4%	91,1%	83,0%
9954	40	50	75	60	34,5%	46,0%	82,9%	91,6%	83,9%
9955	40	50	75	65	34,9%	45,7%	83,4%	92,1%	84,8%
9956	40	50	75	70	35,3%	45,4%	83,8%	92,6%	85,7%
9957	40	50	75	75	35,7%	45,1%	84,3%	93,1%	86,5%
9958	40	50	75	80	36,1%	44,8%	84,7%	93,5%	87,3%
9959	40	50	80	20	26,3%	42,9%	76,5%	85,6%	74,3%
9960	40	50	80	25	26,6%	42,6%	77,1%	86,4%	75,6%
9961	40	50	80	30	27,0%	42,3%	77,7%	87,2%	76,8%
9962	40	50	80	35	27,3%	42,0%	78,3%	87,9%	78,0%
9963	40	50	80	40	27,7%	41,7%	78,9%	88,7%	79,2%
9964	40	50	80	45	28,0%	41,3%	79,4%	89,3%	80,3%
9965	40	50	80	50	28,4%	41,0%	80,0%	90,0%	81,4%

9966	40	50	80	55	28,8%	40,7%	80,5%	90,6%	82,4%
9967	40	50	80	60	29,1%	40,4%	81,1%	91,1%	83,3%
9968	40	50	80	65	29,5%	40,1%	81,6%	91,7%	84,3%
9969	40	50	80	70	29,8%	39,8%	82,1%	92,2%	85,2%
9970	40	50	80	75	30,2%	39,4%	82,6%	92,7%	86,0%
9971	40	50	80	80	30,6%	39,1%	83,1%	93,1%	86,8%
9972	40	55	20	20	88,3%	92,9%	93,3%	92,0%	81,2%
9973	40	55	20	25	88,5%	92,8%	93,6%	92,5%	82,2%
9974	40	55	20	30	88,7%	92,7%	93,8%	92,9%	83,2%
9975	40	55	20	35	88,8%	92,6%	93,9%	93,4%	84,1%
9976	40	55	20	40	89,0%	92,5%	94,1%	93,8%	85,0%
9977	40	55	20	45	89,2%	92,4%	94,3%	94,2%	85,9%
9978	40	55	20	50	89,3%	92,3%	94,5%	94,5%	86,7%
9979	40	55	20	55	89,5%	92,2%	94,7%	94,9%	87,5%
9980	40	55	20	60	89,7%	92,1%	94,8%	95,2%	88,2%
9981	40	55	20	65	89,8%	92,0%	95,0%	95,5%	88,9%
9982	40	55	20	70	90,0%	91,9%	95,2%	95,8%	89,5%
9983	40	55	20	75	90,2%	91,8%	95,3%	96,1%	90,2%
9984	40	55	20	80	90,3%	91,7%	95,5%	96,3%	90,8%
9985	40	55	25	20	85,5%	91,2%	92,5%	91,5%	80,5%
9986	40	55	25	25	85,7%	91,1%	92,8%	92,0%	81,6%
9987	40	55	25	30	85,9%	91,0%	93,0%	92,5%	82,6%
9988	40	55	25	35	86,1%	90,8%	93,2%	93,0%	83,6%
9989	40	55	25	40	86,3%	90,7%	93,4%	93,4%	84,5%
9990	40	55	25	45	86,6%	90,6%	93,6%	93,8%	85,4%
9991	40	55	25	50	86,8%	90,5%	93,8%	94,2%	86,2%
9992	40	55	25	55	87,0%	90,4%	94,0%	94,6%	87,0%
9993	40	55	25	60	87,2%	90,3%	94,2%	94,9%	87,7%
9994	40	55	25	65	87,4%	90,2%	94,4%	95,2%	88,5%
9995	40	55	25	70	87,5%	90,1%	94,6%	95,5%	89,1%
9996	40	55	25	75	87,7%	89,9%	94,7%	95,8%	89,8%
9997	40	55	25	80	87,9%	89,8%	94,9%	96,1%	90,4%
9998	40	55	30	20	82,2%	89,1%	91,6%	91,0%	79,9%

9999	40	55	30	25	82,4%	89,0%	91,9%	91,6%	81,0%
10000	40	55	30	30	82,7%	88,9%	92,1%	92,1%	82,0%
10001	40	55	30	35	82,9%	88,7%	92,4%	92,6%	83,0%
10002	40	55	30	40	83,2%	88,6%	92,6%	93,0%	83,9%
10003	40	55	30	45	83,4%	88,5%	92,8%	93,5%	84,8%
10004	40	55	30	50	83,7%	88,3%	93,1%	93,9%	85,7%
10005	40	55	30	55	83,9%	88,2%	93,3%	94,3%	86,5%
10006	40	55	30	60	84,1%	88,1%	93,5%	94,6%	87,3%
10007	40	55	30	65	84,4%	87,9%	93,7%	95,0%	88,0%
10008	40	55	30	70	84,6%	87,8%	93,9%	95,3%	88,7%
10009	40	55	30	75	84,8%	87,6%	94,1%	95,6%	89,4%
10010	40	55	30	80	85,0%	87,5%	94,3%	95,9%	90,0%
10011	40	55	35	20	78,2%	86,7%	90,6%	90,5%	79,2%
10012	40	55	35	25	78,5%	86,5%	90,9%	91,1%	80,3%
10013	40	55	35	30	78,8%	86,4%	91,2%	91,6%	81,4%
10014	40	55	35	35	79,1%	86,2%	91,5%	92,1%	82,4%
10015	40	55	35	40	79,4%	86,1%	91,7%	92,6%	83,4%
10016	40	55	35	45	79,7%	85,9%	92,0%	93,1%	84,3%
10017	40	55	35	50	80,0%	85,7%	92,2%	93,5%	85,2%
10018	40	55	35	55	80,3%	85,6%	92,5%	93,9%	86,0%
10019	40	55	35	60	80,6%	85,4%	92,7%	94,3%	86,8%
10020	40	55	35	65	80,8%	85,3%	92,9%	94,7%	87,6%
10021	40	55	35	70	81,1%	85,1%	93,1%	95,0%	88,3%
10022	40	55	35	75	81,4%	84,9%	93,3%	95,3%	89,0%
10023	40	55	35	80	81,6%	84,8%	93,6%	95,6%	89,7%
10024	40	55	40	20	73,8%	83,8%	89,5%	90,0%	78,5%
10025	40	55	40	25	74,1%	83,6%	89,8%	90,6%	79,7%
10026	40	55	40	30	74,4%	83,4%	90,1%	91,1%	80,8%
10027	40	55	40	35	74,8%	83,2%	90,4%	91,7%	81,8%
10028	40	55	40	40	75,1%	83,1%	90,7%	92,2%	82,8%
10029	40	55	40	45	75,4%	82,9%	91,0%	92,7%	83,7%
10030	40	55	40	50	75,7%	82,7%	91,3%	93,1%	84,7%
10031	40	55	40	55	76,1%	82,5%	91,5%	93,6%	85,5%

10032	40	55	40	60	76,4%	82,3%	91,8%	94,0%	86,4%
10033	40	55	40	65	76,7%	82,1%	92,1%	94,3%	87,1%
10034	40	55	40	70	77,0%	81,9%	92,3%	94,7%	87,9%
10035	40	55	40	75	77,3%	81,7%	92,5%	95,0%	88,6%
10036	40	55	40	80	77,6%	81,5%	92,8%	95,4%	89,3%
10037	40	55	45	20	68,7%	80,4%	88,3%	89,4%	77,8%
10038	40	55	45	25	69,1%	80,2%	88,6%	90,0%	79,0%
10039	40	55	45	30	69,5%	80,0%	89,0%	90,6%	80,1%
10040	40	55	45	35	69,8%	79,8%	89,3%	91,2%	81,2%
10041	40	55	45	40	70,2%	79,5%	89,6%	91,7%	82,2%
10042	40	55	45	45	70,6%	79,3%	89,9%	92,3%	83,2%
10043	40	55	45	50	70,9%	79,1%	90,2%	92,7%	84,1%
10044	40	55	45	55	71,3%	78,9%	90,5%	93,2%	85,0%
10045	40	55	45	60	71,7%	78,7%	90,8%	93,6%	85,9%
10046	40	55	45	65	72,0%	78,5%	91,1%	94,0%	86,7%
10047	40	55	45	70	72,4%	78,2%	91,4%	94,4%	87,4%
10048	40	55	45	75	72,7%	78,0%	91,6%	94,7%	88,2%
10049	40	55	45	80	73,1%	77,8%	91,9%	95,1%	88,9%
10050	40	55	50	20	63,2%	76,5%	86,9%	88,8%	77,1%
10051	40	55	50	25	63,6%	76,3%	87,3%	89,5%	78,3%
10052	40	55	50	30	64,0%	76,0%	87,7%	90,1%	79,4%
10053	40	55	50	35	64,4%	75,8%	88,1%	90,7%	80,5%
10054	40	55	50	40	64,8%	75,5%	88,4%	91,3%	81,6%
10055	40	55	50	45	65,2%	75,3%	88,7%	91,8%	82,6%
10056	40	55	50	50	65,6%	75,0%	89,1%	92,3%	83,6%
10057	40	55	50	55	66,0%	74,8%	89,4%	92,8%	84,5%
10058	40	55	50	60	66,4%	74,6%	89,7%	93,2%	85,4%
10059	40	55	50	65	66,8%	74,3%	90,0%	93,7%	86,2%
10060	40	55	50	70	67,2%	74,1%	90,3%	94,1%	87,0%
10061	40	55	50	75	67,6%	73,8%	90,6%	94,4%	87,7%
10062	40	55	50	80	67,9%	73,6%	90,9%	94,8%	88,5%
10063	40	55	55	20	57,3%	72,1%	85,5%	88,2%	76,4%
10064	40	55	55	25	57,7%	71,8%	85,9%	88,9%	77,6%

10065	40	55	55	30	58,1%	71,6%	86,3%	89,6%	78,8%
10066	40	55	55	35	58,5%	71,3%	86,7%	90,2%	79,9%
10067	40	55	55	40	59,0%	71,0%	87,1%	90,8%	81,0%
10068	40	55	55	45	59,4%	70,8%	87,4%	91,3%	82,0%
10069	40	55	55	50	59,8%	70,5%	87,8%	91,9%	83,0%
10070	40	55	55	55	60,2%	70,2%	88,2%	92,4%	83,9%
10071	40	55	55	60	60,7%	69,9%	88,5%	92,8%	84,8%
10072	40	55	55	65	61,1%	69,7%	88,9%	93,3%	85,7%
10073	40	55	55	70	61,5%	69,4%	89,2%	93,7%	86,5%
10074	40	55	55	75	61,9%	69,1%	89,5%	94,1%	87,3%
10075	40	55	55	80	62,3%	68,8%	89,8%	94,5%	88,0%
10076	40	55	60	20	51,1%	67,2%	83,8%	87,5%	75,6%
10077	40	55	60	25	51,6%	66,9%	84,3%	88,3%	76,9%
10078	40	55	60	30	52,0%	66,6%	84,7%	89,0%	78,1%
10079	40	55	60	35	52,5%	66,3%	85,2%	89,6%	79,2%
10080	40	55	60	40	52,9%	66,0%	85,6%	90,3%	80,3%
10081	40	55	60	45	53,3%	65,8%	86,0%	90,8%	81,4%
10082	40	55	60	50	53,8%	65,5%	86,4%	91,4%	82,4%
10083	40	55	60	55	54,2%	65,2%	86,8%	91,9%	83,4%
10084	40	55	60	60	54,7%	64,9%	87,2%	92,4%	84,3%
10085	40	55	60	65	55,1%	64,6%	87,6%	92,9%	85,2%
10086	40	55	60	70	55,5%	64,3%	87,9%	93,3%	86,0%
10087	40	55	60	75	56,0%	64,0%	88,3%	93,8%	86,8%
10088	40	55	60	80	56,4%	63,7%	88,6%	94,1%	87,6%
10089	40	55	65	20	45,0%	61,9%	82,1%	86,9%	74,9%
10090	40	55	65	25	45,4%	61,6%	82,6%	87,6%	76,1%
10091	40	55	65	30	45,9%	61,3%	83,1%	88,4%	77,3%
10092	40	55	65	35	46,3%	61,0%	83,5%	89,0%	78,5%
10093	40	55	65	40	46,7%	60,7%	84,0%	89,7%	79,7%
10094	40	55	65	45	47,2%	60,4%	84,5%	90,3%	80,7%
10095	40	55	65	50	47,6%	60,1%	84,9%	90,9%	81,8%
10096	40	55	65	55	48,1%	59,8%	85,3%	91,5%	82,8%
10097	40	55	65	60	48,5%	59,4%	85,7%	92,0%	83,7%

10098	40	55	65	65	48,9%	59,1%	86,2%	92,5%	84,6%
10099	40	55	65	70	49,4%	58,8%	86,6%	92,9%	85,5%
10100	40	55	65	75	49,8%	58,5%	86,9%	93,4%	86,3%
10101	40	55	65	80	50,3%	58,2%	87,3%	93,8%	87,1%
10102	40	55	70	20	39,0%	56,4%	80,2%	86,1%	74,1%
10103	40	55	70	25	39,4%	56,0%	80,7%	87,0%	75,4%
10104	40	55	70	30	39,8%	55,7%	81,3%	87,7%	76,6%
10105	40	55	70	35	40,2%	55,4%	81,8%	88,4%	77,8%
10106	40	55	70	40	40,7%	55,1%	82,3%	89,1%	79,0%
10107	40	55	70	45	41,1%	54,7%	82,8%	89,8%	80,1%
10108	40	55	70	50	41,5%	54,4%	83,2%	90,4%	81,2%
10109	40	55	70	55	42,0%	54,1%	83,7%	91,0%	82,2%
10110	40	55	70	60	42,4%	53,8%	84,2%	91,5%	83,2%
10111	40	55	70	65	42,8%	53,5%	84,6%	92,0%	84,1%
10112	40	55	70	70	43,2%	53,1%	85,0%	92,5%	85,0%
10113	40	55	70	75	43,7%	52,8%	85,5%	93,0%	85,9%
10114	40	55	70	80	44,1%	52,5%	85,9%	93,4%	86,7%
10115	40	55	75	20	33,3%	50,6%	78,1%	85,4%	73,3%
10116	40	55	75	25	33,7%	50,3%	78,7%	86,2%	74,6%
10117	40	55	75	30	34,1%	50,0%	79,3%	87,0%	75,9%
10118	40	55	75	35	34,5%	49,6%	79,8%	87,8%	77,1%
10119	40	55	75	40	34,9%	49,3%	80,4%	88,5%	78,3%
10120	40	55	75	45	35,3%	49,0%	80,9%	89,2%	79,4%
10121	40	55	75	50	35,7%	48,7%	81,4%	89,8%	80,5%
10122	40	55	75	55	36,1%	48,3%	81,9%	90,5%	81,6%
10123	40	55	75	60	36,5%	48,0%	82,4%	91,0%	82,6%
10124	40	55	75	65	36,9%	47,7%	82,9%	91,6%	83,5%
10125	40	55	75	70	37,3%	47,4%	83,4%	92,1%	84,5%
10126	40	55	75	75	37,7%	47,0%	83,9%	92,6%	85,3%
10127	40	55	75	80	38,1%	46,7%	84,3%	93,1%	86,2%
10128	40	55	80	20	28,0%	44,9%	76,0%	84,6%	72,5%
10129	40	55	80	25	28,4%	44,5%	76,6%	85,5%	73,8%
10130	40	55	80	30	28,8%	44,2%	77,2%	86,3%	75,1%

10131	40	55	80	35	29,1%	43,9%	77,8%	87,1%	76,4%
10132	40	55	80	40	29,5%	43,6%	78,3%	87,9%	77,6%
10133	40	55	80	45	29,9%	43,3%	78,9%	88,6%	78,7%
10134	40	55	80	50	30,2%	42,9%	79,5%	89,3%	79,9%
10135	40	55	80	55	30,6%	42,6%	80,0%	89,9%	80,9%
10136	40	55	80	60	31,0%	42,3%	80,6%	90,5%	82,0%
10137	40	55	80	65	31,4%	42,0%	81,1%	91,1%	83,0%
10138	40	55	80	70	31,7%	41,7%	81,6%	91,6%	83,9%
10139	40	55	80	75	32,1%	41,3%	82,1%	92,2%	84,8%
10140	40	55	80	80	32,5%	41,0%	82,6%	92,6%	85,7%
10141	40	60	20	20	89,2%	93,4%	93,1%	91,4%	79,7%
10142	40	60	20	25	89,4%	93,3%	93,4%	91,9%	80,8%
10143	40	60	20	30	89,5%	93,2%	93,6%	92,4%	81,8%
10144	40	60	20	35	89,7%	93,1%	93,8%	92,9%	82,8%
10145	40	60	20	40	89,8%	93,0%	94,0%	93,3%	83,8%
10146	40	60	20	45	90,0%	92,9%	94,2%	93,8%	84,7%
10147	40	60	20	50	90,2%	92,9%	94,3%	94,1%	85,5%
10148	40	60	20	55	90,3%	92,8%	94,5%	94,5%	86,4%
10149	40	60	20	60	90,5%	92,7%	94,7%	94,9%	87,2%
10150	40	60	20	65	90,6%	92,6%	94,9%	95,2%	87,9%
10151	40	60	20	70	90,8%	92,5%	95,0%	95,5%	88,6%
10152	40	60	20	75	90,9%	92,4%	95,2%	95,8%	89,3%
10153	40	60	20	80	91,1%	92,3%	95,3%	96,0%	89,9%
10154	40	60	25	20	86,6%	91,8%	92,3%	90,9%	79,0%
10155	40	60	25	25	86,8%	91,7%	92,5%	91,5%	80,1%
10156	40	60	25	30	87,0%	91,6%	92,8%	92,0%	81,2%
10157	40	60	25	35	87,2%	91,5%	93,0%	92,5%	82,2%
10158	40	60	25	40	87,4%	91,4%	93,2%	92,9%	83,2%
10159	40	60	25	45	87,6%	91,3%	93,4%	93,4%	84,1%
10160	40	60	25	50	87,7%	91,2%	93,6%	93,8%	85,0%
10161	40	60	25	55	87,9%	91,1%	93,8%	94,2%	85,9%
10162	40	60	25	60	88,1%	91,0%	94,0%	94,6%	86,7%
10163	40	60	25	65	88,3%	90,8%	94,2%	94,9%	87,5%

10164	40	60	25	70	88,5%	90,7%	94,4%	95,2%	88,2%
10165	40	60	25	75	88,7%	90,6%	94,6%	95,5%	88,9%
10166	40	60	25	80	88,8%	90,5%	94,7%	95,8%	89,5%
10167	40	60	30	20	83,4%	89,9%	91,4%	90,4%	78,3%
10168	40	60	30	25	83,7%	89,7%	91,6%	91,0%	79,5%
10169	40	60	30	30	83,9%	89,6%	91,9%	91,5%	80,6%
10170	40	60	30	35	84,1%	89,5%	92,1%	92,0%	81,6%
10171	40	60	30	40	84,4%	89,4%	92,4%	92,5%	82,6%
10172	40	60	30	45	84,6%	89,3%	92,6%	93,0%	83,6%
10173	40	60	30	50	84,8%	89,1%	92,9%	93,4%	84,5%
10174	40	60	30	55	85,1%	89,0%	93,1%	93,8%	85,4%
10175	40	60	30	60	85,3%	88,9%	93,3%	94,2%	86,2%
10176	40	60	30	65	85,5%	88,7%	93,5%	94,6%	87,0%
10177	40	60	30	70	85,7%	88,6%	93,7%	94,9%	87,8%
10178	40	60	30	75	85,9%	88,5%	93,9%	95,3%	88,5%
10179	40	60	30	80	86,1%	88,3%	94,1%	95,6%	89,1%
10180	40	60	35	20	79,7%	87,6%	90,3%	89,8%	77,6%
10181	40	60	35	25	80,0%	87,4%	90,6%	90,5%	78,8%
10182	40	60	35	30	80,3%	87,3%	90,9%	91,0%	79,9%
10183	40	60	35	35	80,6%	87,1%	91,2%	91,6%	81,0%
10184	40	60	35	40	80,8%	87,0%	91,5%	92,1%	82,0%
10185	40	60	35	45	81,1%	86,8%	91,7%	92,6%	83,0%
10186	40	60	35	50	81,4%	86,7%	92,0%	93,0%	83,9%
10187	40	60	35	55	81,6%	86,5%	92,2%	93,5%	84,8%
10188	40	60	35	60	81,9%	86,4%	92,5%	93,9%	85,7%
10189	40	60	35	65	82,2%	86,2%	92,7%	94,3%	86,5%
10190	40	60	35	70	82,4%	86,1%	92,9%	94,6%	87,3%
10191	40	60	35	75	82,7%	85,9%	93,1%	95,0%	88,0%
10192	40	60	35	80	82,9%	85,7%	93,4%	95,3%	88,7%
10193	40	60	40	20	75,4%	84,8%	89,2%	89,3%	76,9%
10194	40	60	40	25	75,8%	84,6%	89,5%	89,9%	78,1%
10195	40	60	40	30	76,1%	84,5%	89,8%	90,5%	79,2%
10196	40	60	40	35	76,4%	84,3%	90,1%	91,1%	80,3%

10197	40	60	40	40	76,7%	84,1%	90,4%	91,6%	81,4%
10198	40	60	40	45	77,0%	84,0%	90,7%	92,2%	82,4%
10199	40	60	40	50	77,3%	83,8%	91,0%	92,6%	83,4%
10200	40	60	40	55	77,6%	83,6%	91,3%	93,1%	84,3%
10201	40	60	40	60	78,0%	83,4%	91,6%	93,5%	85,2%
10202	40	60	40	65	78,3%	83,2%	91,8%	93,9%	86,0%
10203	40	60	40	70	78,6%	83,1%	92,1%	94,3%	86,8%
10204	40	60	40	75	78,8%	82,9%	92,3%	94,7%	87,6%
10205	40	60	40	80	79,1%	82,7%	92,6%	95,0%	88,3%
10206	40	60	45	20	70,6%	81,6%	88,0%	88,7%	76,1%
10207	40	60	45	25	70,9%	81,4%	88,3%	89,4%	77,4%
10208	40	60	45	30	71,3%	81,2%	88,7%	90,0%	78,5%
10209	40	60	45	35	71,7%	81,0%	89,0%	90,6%	79,7%
10210	40	60	45	40	72,0%	80,8%	89,3%	91,2%	80,8%
10211	40	60	45	45	72,4%	80,6%	89,6%	91,7%	81,8%
10212	40	60	45	50	72,7%	80,4%	89,9%	92,2%	82,8%
10213	40	60	45	55	73,1%	80,2%	90,3%	92,7%	83,8%
10214	40	60	45	60	73,4%	80,0%	90,5%	93,2%	84,7%
10215	40	60	45	65	73,8%	79,8%	90,8%	93,6%	85,5%
10216	40	60	45	70	74,1%	79,6%	91,1%	94,0%	86,4%
10217	40	60	45	75	74,4%	79,3%	91,4%	94,4%	87,1%
10218	40	60	45	80	74,8%	79,1%	91,6%	94,7%	87,9%
10219	40	60	50	20	65,2%	77,9%	86,6%	88,1%	75,4%
10220	40	60	50	25	65,6%	77,7%	87,0%	88,8%	76,6%
10221	40	60	50	30	66,0%	77,4%	87,3%	89,4%	77,8%
10222	40	60	50	35	66,4%	77,2%	87,7%	90,1%	79,0%
10223	40	60	50	40	66,8%	77,0%	88,1%	90,7%	80,1%
10224	40	60	50	45	67,2%	76,7%	88,4%	91,2%	81,2%
10225	40	60	50	50	67,6%	76,5%	88,8%	91,8%	82,2%
10226	40	60	50	55	68,0%	76,3%	89,1%	92,3%	83,2%
10227	40	60	50	60	68,3%	76,0%	89,4%	92,8%	84,1%
10228	40	60	50	65	68,7%	75,8%	89,7%	93,2%	85,0%
10229	40	60	50	70	69,1%	75,5%	90,1%	93,6%	85,9%

10230	40	60	50	75	69,5%	75,3%	90,4%	94,0%	86,7%
10231	40	60	50	80	69,8%	75,1%	90,6%	94,4%	87,4%
10232	40	60	55	20	59,4%	73,6%	85,1%	87,4%	74,6%
10233	40	60	55	25	59,8%	73,4%	85,5%	88,1%	75,9%
10234	40	60	55	30	60,3%	73,1%	85,9%	88,8%	77,1%
10235	40	60	55	35	60,7%	72,9%	86,3%	89,5%	78,3%
10236	40	60	55	40	61,1%	72,6%	86,7%	90,1%	79,4%
10237	40	60	55	45	61,5%	72,4%	87,1%	90,7%	80,5%
10238	40	60	55	50	61,9%	72,1%	87,5%	91,3%	81,6%
10239	40	60	55	55	62,4%	71,8%	87,8%	91,8%	82,6%
10240	40	60	55	60	62,8%	71,6%	88,2%	92,3%	83,6%
10241	40	60	55	65	63,2%	71,3%	88,5%	92,8%	84,5%
10242	40	60	55	70	63,6%	71,0%	88,9%	93,3%	85,4%
10243	40	60	55	75	64,0%	70,8%	89,2%	93,7%	86,2%
10244	40	60	55	80	64,4%	70,5%	89,5%	94,1%	87,0%
10245	40	60	60	20	53,4%	68,9%	83,4%	86,7%	73,8%
10246	40	60	60	25	53,8%	68,6%	83,9%	87,5%	75,1%
10247	40	60	60	30	54,2%	68,4%	84,3%	88,2%	76,4%
10248	40	60	60	35	54,7%	68,1%	84,8%	88,9%	77,6%
10249	40	60	60	40	55,1%	67,8%	85,2%	89,6%	78,8%
10250	40	60	60	45	55,5%	67,5%	85,6%	90,2%	79,9%
10251	40	60	60	50	56,0%	67,2%	86,0%	90,8%	81,0%
10252	40	60	60	55	56,4%	66,9%	86,4%	91,4%	82,0%
10253	40	60	60	60	56,8%	66,6%	86,8%	91,9%	83,0%
10254	40	60	60	65	57,3%	66,3%	87,2%	92,4%	83,9%
10255	40	60	60	70	57,7%	66,1%	87,6%	92,9%	84,8%
10256	40	60	60	75	58,1%	65,8%	88,0%	93,3%	85,7%
10257	40	60	60	80	58,6%	65,5%	88,3%	93,7%	86,5%
10258	40	60	65	20	47,2%	63,8%	81,6%	86,0%	73,0%
10259	40	60	65	25	47,6%	63,5%	82,1%	86,8%	74,3%
10260	40	60	65	30	48,1%	63,2%	82,6%	87,6%	75,6%
10261	40	60	65	35	48,5%	62,9%	83,1%	88,3%	76,9%
10262	40	60	65	40	48,9%	62,6%	83,6%	89,0%	78,1%

10263	40	60	65	45	49,4%	62,3%	84,0%	89,7%	79,2%
10264	40	60	65	50	49,8%	61,9%	84,5%	90,3%	80,3%
10265	40	60	65	55	50,3%	61,6%	84,9%	90,9%	81,4%
10266	40	60	65	60	50,7%	61,3%	85,4%	91,4%	82,4%
10267	40	60	65	65	51,2%	61,0%	85,8%	91,9%	83,4%
10268	40	60	65	70	51,6%	60,7%	86,2%	92,4%	84,3%
10269	40	60	65	75	52,0%	60,4%	86,6%	92,9%	85,2%
10270	40	60	65	80	52,5%	60,1%	87,0%	93,4%	86,0%
10271	40	60	70	20	41,1%	58,3%	79,7%	85,2%	72,2%
10272	40	60	70	25	41,5%	58,0%	80,2%	86,1%	73,5%
10273	40	60	70	30	42,0%	57,7%	80,8%	86,9%	74,9%
10274	40	60	70	35	42,4%	57,3%	81,3%	87,7%	76,1%
10275	40	60	70	40	42,8%	57,0%	81,8%	88,4%	77,3%
10276	40	60	70	45	43,3%	56,7%	82,3%	89,1%	78,5%
10277	40	60	70	50	43,7%	56,4%	82,8%	89,7%	79,7%
10278	40	60	70	55	44,1%	56,0%	83,3%	90,3%	80,7%
10279	40	60	70	60	44,6%	55,7%	83,7%	90,9%	81,8%
10280	40	60	70	65	45,0%	55,4%	84,2%	91,5%	82,8%
10281	40	60	70	70	45,4%	55,1%	84,6%	92,0%	83,7%
10282	40	60	70	75	45,9%	54,8%	85,1%	92,5%	84,7%
10283	40	60	70	80	46,3%	54,4%	85,5%	93,0%	85,5%
10284	40	60	75	20	35,3%	52,6%	77,6%	84,5%	71,4%
10285	40	60	75	25	35,7%	52,3%	78,2%	85,3%	72,7%
10286	40	60	75	30	36,1%	51,9%	78,8%	86,2%	74,1%
10287	40	60	75	35	36,5%	51,6%	79,3%	87,0%	75,4%
10288	40	60	75	40	36,9%	51,3%	79,9%	87,7%	76,6%
10289	40	60	75	45	37,3%	51,0%	80,4%	88,5%	77,8%
10290	40	60	75	50	37,7%	50,6%	80,9%	89,2%	79,0%
10291	40	60	75	55	38,2%	50,3%	81,5%	89,8%	80,1%
10292	40	60	75	60	38,6%	50,0%	82,0%	90,4%	81,2%
10293	40	60	75	65	39,0%	49,7%	82,5%	91,0%	82,2%
10294	40	60	75	70	39,4%	49,3%	82,9%	91,5%	83,2%
10295	40	60	75	75	39,8%	49,0%	83,4%	92,1%	84,1%

10296	40	60	75	80	40,3%	48,7%	83,9%	92,6%	85,0%
10297	40	60	80	20	29,9%	46,8%	75,4%	83,6%	70,5%
10298	40	60	80	25	30,2%	46,5%	76,0%	84,6%	71,9%
10299	40	60	80	30	30,6%	46,2%	76,6%	85,4%	73,3%
10300	40	60	80	35	31,0%	45,8%	77,2%	86,3%	74,6%
10301	40	60	80	40	31,4%	45,5%	77,8%	87,1%	75,9%
10302	40	60	80	45	31,8%	45,2%	78,4%	87,8%	77,1%
10303	40	60	80	50	32,1%	44,9%	79,0%	88,6%	78,3%
10304	40	60	80	55	32,5%	44,6%	79,5%	89,2%	79,4%
10305	40	60	80	60	32,9%	44,2%	80,1%	89,9%	80,5%
10306	40	60	80	65	33,3%	43,9%	80,6%	90,5%	81,6%
10307	40	60	80	70	33,7%	43,6%	81,1%	91,1%	82,6%
10308	40	60	80	75	34,1%	43,3%	81,6%	91,6%	83,6%
10309	40	60	80	80	34,5%	42,9%	82,1%	92,1%	84,5%
10310	40	65	20	20	90,0%	93,8%	92,9%	90,8%	78,1%
10311	40	65	20	25	90,2%	93,8%	93,2%	91,4%	79,2%
10312	40	65	20	30	90,3%	93,7%	93,4%	91,9%	80,3%
10313	40	65	20	35	90,5%	93,6%	93,6%	92,4%	81,4%
10314	40	65	20	40	90,6%	93,5%	93,8%	92,9%	82,4%
10315	40	65	20	45	90,8%	93,4%	94,0%	93,3%	83,4%
10316	40	65	20	50	90,9%	93,4%	94,2%	93,7%	84,3%
10317	40	65	20	55	91,1%	93,3%	94,3%	94,1%	85,2%
10318	40	65	20	60	91,2%	93,2%	94,5%	94,5%	86,0%
10319	40	65	20	65	91,3%	93,1%	94,7%	94,8%	86,8%
10320	40	65	20	70	91,5%	93,0%	94,9%	95,2%	87,6%
10321	40	65	20	75	91,6%	92,9%	95,0%	95,5%	88,3%
10322	40	65	20	80	91,8%	92,9%	95,2%	95,8%	89,0%
10323	40	65	25	20	87,6%	92,4%	92,1%	90,3%	77,4%
10324	40	65	25	25	87,8%	92,3%	92,3%	90,9%	78,6%
10325	40	65	25	30	87,9%	92,2%	92,6%	91,4%	79,7%
10326	40	65	25	35	88,1%	92,1%	92,8%	91,9%	80,8%
10327	40	65	25	40	88,3%	92,0%	93,0%	92,4%	81,8%
10328	40	65	25	45	88,5%	91,9%	93,2%	92,9%	82,8%

10329	40	65	25	50	88,7%	91,8%	93,4%	93,4%	83,8%
10330	40	65	25	55	88,8%	91,7%	93,6%	93,8%	84,7%
10331	40	65	25	60	89,0%	91,6%	93,8%	94,2%	85,5%
10332	40	65	25	65	89,2%	91,5%	94,0%	94,5%	86,4%
10333	40	65	25	70	89,4%	91,4%	94,2%	94,9%	87,2%
10334	40	65	25	75	89,5%	91,3%	94,4%	95,2%	87,9%
10335	40	65	25	80	89,7%	91,2%	94,6%	95,5%	88,6%
10336	40	65	30	20	84,6%	90,6%	91,1%	89,7%	76,6%
10337	40	65	30	25	84,8%	90,4%	91,4%	90,3%	77,9%
10338	40	65	30	30	85,1%	90,3%	91,7%	90,9%	79,0%
10339	40	65	30	35	85,3%	90,2%	91,9%	91,5%	80,1%
10340	40	65	30	40	85,5%	90,1%	92,2%	92,0%	81,2%
10341	40	65	30	45	85,7%	90,0%	92,4%	92,5%	82,2%
10342	40	65	30	50	85,9%	89,9%	92,6%	93,0%	83,2%
10343	40	65	30	55	86,2%	89,7%	92,9%	93,4%	84,1%
10344	40	65	30	60	86,4%	89,6%	93,1%	93,8%	85,0%
10345	40	65	30	65	86,6%	89,5%	93,3%	94,2%	85,9%
10346	40	65	30	70	86,8%	89,4%	93,5%	94,6%	86,7%
10347	40	65	30	75	87,0%	89,3%	93,7%	94,9%	87,5%
10348	40	65	30	80	87,2%	89,1%	93,9%	95,2%	88,2%
10349	40	65	35	20	81,1%	88,4%	90,1%	89,2%	75,9%
10350	40	65	35	25	81,4%	88,3%	90,4%	89,8%	77,1%
10351	40	65	35	30	81,6%	88,1%	90,7%	90,4%	78,3%
10352	40	65	35	35	81,9%	88,0%	90,9%	91,0%	79,5%
10353	40	65	35	40	82,2%	87,8%	91,2%	91,5%	80,6%
10354	40	65	35	45	82,4%	87,7%	91,5%	92,1%	81,6%
10355	40	65	35	50	82,7%	87,6%	91,7%	92,6%	82,6%
10356	40	65	35	55	82,9%	87,4%	92,0%	93,0%	83,6%
10357	40	65	35	60	83,2%	87,3%	92,2%	93,5%	84,5%
10358	40	65	35	65	83,4%	87,1%	92,5%	93,9%	85,4%
10359	40	65	35	70	83,7%	87,0%	92,7%	94,2%	86,2%
10360	40	65	35	75	83,9%	86,8%	92,9%	94,6%	87,0%
10361	40	65	35	80	84,1%	86,7%	93,2%	95,0%	87,8%

10362	40	65	40	20	77,0%	85,8%	88,9%	88,5%	75,1%
10363	40	65	40	25	77,4%	85,6%	89,2%	89,2%	76,4%
10364	40	65	40	30	77,7%	85,5%	89,6%	89,9%	77,6%
10365	40	65	40	35	78,0%	85,3%	89,9%	90,5%	78,8%
10366	40	65	40	40	78,3%	85,2%	90,2%	91,1%	79,9%
10367	40	65	40	45	78,6%	85,0%	90,5%	91,6%	81,0%
10368	40	65	40	50	78,9%	84,8%	90,8%	92,1%	82,0%
10369	40	65	40	55	79,2%	84,7%	91,0%	92,6%	83,0%
10370	40	65	40	60	79,4%	84,5%	91,3%	93,1%	83,9%
10371	40	65	40	65	79,7%	84,3%	91,6%	93,5%	84,8%
10372	40	65	40	70	80,0%	84,1%	91,8%	93,9%	85,7%
10373	40	65	40	75	80,3%	84,0%	92,1%	94,3%	86,5%
10374	40	65	40	80	80,6%	83,8%	92,3%	94,6%	87,3%
10375	40	65	45	20	72,4%	82,8%	87,6%	87,9%	74,4%
10376	40	65	45	25	72,7%	82,6%	88,0%	88,6%	75,6%
10377	40	65	45	30	73,1%	82,4%	88,3%	89,3%	76,9%
10378	40	65	45	35	73,4%	82,2%	88,7%	89,9%	78,1%
10379	40	65	45	40	73,8%	82,0%	89,0%	90,6%	79,2%
10380	40	65	45	45	74,1%	81,8%	89,3%	91,1%	80,3%
10381	40	65	45	50	74,5%	81,6%	89,7%	91,7%	81,4%
10382	40	65	45	55	74,8%	81,4%	90,0%	92,2%	82,4%
10383	40	65	45	60	75,1%	81,2%	90,3%	92,7%	83,4%
10384	40	65	45	65	75,4%	81,0%	90,6%	93,1%	84,3%
10385	40	65	45	70	75,8%	80,8%	90,9%	93,5%	85,2%
10386	40	65	45	75	76,1%	80,6%	91,1%	94,0%	86,0%
10387	40	65	45	80	76,4%	80,4%	91,4%	94,3%	86,8%
10388	40	65	50	20	67,2%	79,2%	86,2%	87,3%	73,6%
10389	40	65	50	25	67,6%	79,0%	86,6%	88,0%	74,9%
10390	40	65	50	30	68,0%	78,8%	87,0%	88,7%	76,1%
10391	40	65	50	35	68,3%	78,5%	87,4%	89,4%	77,4%
10392	40	65	50	40	68,7%	78,3%	87,7%	90,0%	78,5%
10393	40	65	50	45	69,1%	78,1%	88,1%	90,6%	79,7%
10394	40	65	50	50	69,5%	77,9%	88,4%	91,2%	80,8%

10395	40	65	50	55	69,9%	77,7%	88,8%	91,7%	81,8%
10396	40	65	50	60	70,2%	77,4%	89,1%	92,2%	82,8%
10397	40	65	50	65	70,6%	77,2%	89,4%	92,7%	83,8%
10398	40	65	50	70	71,0%	77,0%	89,8%	93,2%	84,7%
10399	40	65	50	75	71,3%	76,7%	90,1%	93,6%	85,5%
10400	40	65	50	80	71,7%	76,5%	90,4%	94,0%	86,4%
10401	40	65	55	20	61,5%	75,1%	84,7%	86,6%	72,8%
10402	40	65	55	25	62,0%	74,9%	85,1%	87,3%	74,1%
10403	40	65	55	30	62,4%	74,7%	85,5%	88,1%	75,4%
10404	40	65	55	35	62,8%	74,4%	85,9%	88,8%	76,6%
10405	40	65	55	40	63,2%	74,2%	86,3%	89,5%	77,8%
10406	40	65	55	45	63,6%	73,9%	86,7%	90,1%	79,0%
10407	40	65	55	50	64,0%	73,6%	87,1%	90,7%	80,1%
10408	40	65	55	55	64,4%	73,4%	87,5%	91,3%	81,2%
10409	40	65	55	60	64,8%	73,1%	87,9%	91,8%	82,2%
10410	40	65	55	65	65,2%	72,9%	88,2%	92,3%	83,2%
10411	40	65	55	70	65,6%	72,6%	88,6%	92,8%	84,1%
10412	40	65	55	75	66,0%	72,4%	88,9%	93,2%	85,0%
10413	40	65	55	80	66,4%	72,1%	89,2%	93,6%	85,9%
10414	40	65	60	20	55,6%	70,6%	83,0%	85,8%	71,9%
10415	40	65	60	25	56,0%	70,3%	83,4%	86,7%	73,3%
10416	40	65	60	30	56,4%	70,0%	83,9%	87,4%	74,6%
10417	40	65	60	35	56,9%	69,8%	84,4%	88,2%	75,9%
10418	40	65	60	40	57,3%	69,5%	84,8%	88,9%	77,1%
10419	40	65	60	45	57,7%	69,2%	85,2%	89,5%	78,3%
10420	40	65	60	50	58,1%	68,9%	85,7%	90,2%	79,4%
10421	40	65	60	55	58,6%	68,6%	86,1%	90,8%	80,5%
10422	40	65	60	60	59,0%	68,4%	86,5%	91,3%	81,6%
10423	40	65	60	65	59,4%	68,1%	86,9%	91,9%	82,6%
10424	40	65	60	70	59,9%	67,8%	87,2%	92,4%	83,6%
10425	40	65	60	75	60,3%	67,5%	87,6%	92,8%	84,5%
10426	40	65	60	80	60,7%	67,2%	88,0%	93,3%	85,4%
10427	40	65	65	20	49,4%	65,6%	81,1%	85,1%	71,1%

10428	40	65	65	25	49,8%	65,3%	81,7%	85,9%	72,5%
10429	40	65	65	30	50,3%	65,0%	82,2%	86,7%	73,8%
10430	40	65	65	35	50,7%	64,7%	82,7%	87,5%	75,1%
10431	40	65	65	40	51,2%	64,4%	83,1%	88,3%	76,4%
10432	40	65	65	45	51,6%	64,1%	83,6%	88,9%	77,6%
10433	40	65	65	50	52,0%	63,8%	84,1%	89,6%	78,8%
10434	40	65	65	55	52,5%	63,5%	84,5%	90,2%	79,9%
10435	40	65	65	60	52,9%	63,2%	85,0%	90,8%	81,0%
10436	40	65	65	65	53,4%	62,9%	85,4%	91,4%	82,0%
10437	40	65	65	70	53,8%	62,6%	85,8%	91,9%	83,0%
10438	40	65	65	75	54,2%	62,3%	86,2%	92,4%	83,9%
10439	40	65	65	80	54,7%	62,0%	86,6%	92,9%	84,8%
10440	40	65	70	20	43,3%	60,2%	79,2%	84,3%	70,2%
10441	40	65	70	25	43,7%	59,9%	79,7%	85,2%	71,6%
10442	40	65	70	30	44,1%	59,6%	80,3%	86,0%	73,0%
10443	40	65	70	35	44,6%	59,2%	80,8%	86,8%	74,3%
10444	40	65	70	40	45,0%	58,9%	81,3%	87,6%	75,6%
10445	40	65	70	45	45,4%	58,6%	81,8%	88,3%	76,9%
10446	40	65	70	50	45,9%	58,3%	82,3%	89,0%	78,1%
10447	40	65	70	55	46,3%	58,0%	82,8%	89,7%	79,2%
10448	40	65	70	60	46,8%	57,7%	83,3%	90,3%	80,3%
10449	40	65	70	65	47,2%	57,3%	83,8%	90,9%	81,4%
10450	40	65	70	70	47,6%	57,0%	84,2%	91,4%	82,4%
10451	40	65	70	75	48,1%	56,7%	84,7%	92,0%	83,4%
10452	40	65	70	80	48,5%	56,4%	85,1%	92,5%	84,3%
10453	40	65	75	20	37,3%	54,6%	77,0%	83,5%	69,4%
10454	40	65	75	25	37,8%	54,2%	77,6%	84,4%	70,8%
10455	40	65	75	30	38,2%	53,9%	78,2%	85,3%	72,2%
10456	40	65	75	35	38,6%	53,6%	78,8%	86,1%	73,6%
10457	40	65	75	40	39,0%	53,3%	79,4%	86,9%	74,9%
10458	40	65	75	45	39,4%	52,9%	79,9%	87,7%	76,1%
10459	40	65	75	50	39,8%	52,6%	80,4%	88,4%	77,4%
10460	40	65	75	55	40,3%	52,3%	81,0%	89,1%	78,5%

10461	40	65	75	60	40,7%	51,9%	81,5%	89,8%	79,7%
10462	40	65	75	65	41,1%	51,6%	82,0%	90,4%	80,8%
10463	40	65	75	70	41,5%	51,3%	82,5%	91,0%	81,8%
10464	40	65	75	75	42,0%	51,0%	83,0%	91,5%	82,8%
10465	40	65	75	80	42,4%	50,6%	83,5%	92,0%	83,7%
10466	40	65	80	20	31,8%	48,8%	74,8%	82,6%	68,5%
10467	40	65	80	25	32,1%	48,5%	75,4%	83,6%	69,9%
10468	40	65	80	30	32,5%	48,1%	76,0%	84,5%	71,4%
10469	40	65	80	35	32,9%	47,8%	76,6%	85,4%	72,7%
10470	40	65	80	40	33,3%	47,5%	77,2%	86,2%	74,1%
10471	40	65	80	45	33,7%	47,2%	77,8%	87,0%	75,4%
10472	40	65	80	50	34,1%	46,8%	78,4%	87,8%	76,6%
10473	40	65	80	55	34,5%	46,5%	79,0%	88,5%	77,8%
10474	40	65	80	60	34,9%	46,2%	79,5%	89,2%	79,0%
10475	40	65	80	65	35,3%	45,9%	80,1%	89,8%	80,1%
10476	40	65	80	70	35,7%	45,5%	80,6%	90,4%	81,2%
10477	40	65	80	75	36,1%	45,2%	81,2%	91,0%	82,2%
10478	40	65	80	80	36,5%	44,9%	81,7%	91,6%	83,2%
10479	40	70	20	20	90,8%	94,3%	92,7%	90,2%	76,4%
10480	40	70	20	25	90,9%	94,2%	93,0%	90,8%	77,6%
10481	40	70	20	30	91,1%	94,1%	93,2%	91,3%	78,8%
10482	40	70	20	35	91,2%	94,1%	93,4%	91,8%	79,9%
10483	40	70	20	40	91,4%	94,0%	93,6%	92,4%	81,0%
10484	40	70	20	45	91,5%	93,9%	93,8%	92,8%	82,0%
10485	40	70	20	50	91,6%	93,8%	94,0%	93,3%	83,0%
10486	40	70	20	55	91,8%	93,8%	94,2%	93,7%	84,0%
10487	40	70	20	60	91,9%	93,7%	94,4%	94,1%	84,9%
10488	40	70	20	65	92,0%	93,6%	94,5%	94,5%	85,7%
10489	40	70	20	70	92,2%	93,5%	94,7%	94,8%	86,5%
10490	40	70	20	75	92,3%	93,4%	94,9%	95,1%	87,3%
10491	40	70	20	80	92,4%	93,4%	95,0%	95,4%	88,1%
10492	40	70	25	20	88,5%	92,9%	91,8%	89,6%	75,7%
10493	40	70	25	25	88,7%	92,8%	92,1%	90,2%	76,9%

10494	40	70	25	30	88,8%	92,7%	92,3%	90,8%	78,1%
10495	40	70	25	35	89,0%	92,6%	92,6%	91,4%	79,2%
10496	40	70	25	40	89,2%	92,5%	92,8%	91,9%	80,4%
10497	40	70	25	45	89,4%	92,5%	93,0%	92,4%	81,4%
10498	40	70	25	50	89,5%	92,4%	93,2%	92,9%	82,4%
10499	40	70	25	55	89,7%	92,3%	93,5%	93,3%	83,4%
10500	40	70	25	60	89,9%	92,2%	93,7%	93,7%	84,3%
10501	40	70	25	65	90,0%	92,1%	93,9%	94,1%	85,2%
10502	40	70	25	70	90,2%	92,0%	94,1%	94,5%	86,0%
10503	40	70	25	75	90,3%	91,9%	94,2%	94,8%	86,9%
10504	40	70	25	80	90,5%	91,8%	94,4%	95,2%	87,6%
10505	40	70	30	20	85,7%	91,2%	90,9%	89,0%	74,9%
10506	40	70	30	25	85,9%	91,1%	91,1%	89,7%	76,2%
10507	40	70	30	30	86,2%	91,0%	91,4%	90,3%	77,4%
10508	40	70	30	35	86,4%	90,9%	91,7%	90,9%	78,6%
10509	40	70	30	40	86,6%	90,8%	91,9%	91,4%	79,7%
10510	40	70	30	45	86,8%	90,7%	92,2%	92,0%	80,8%
10511	40	70	30	50	87,0%	90,6%	92,4%	92,5%	81,8%
10512	40	70	30	55	87,2%	90,4%	92,7%	92,9%	82,8%
10513	40	70	30	60	87,4%	90,3%	92,9%	93,4%	83,8%
10514	40	70	30	65	87,6%	90,2%	93,1%	93,8%	84,7%
10515	40	70	30	70	87,8%	90,1%	93,3%	94,2%	85,5%
10516	40	70	30	75	87,9%	90,0%	93,5%	94,5%	86,4%
10517	40	70	30	80	88,1%	89,9%	93,7%	94,9%	87,2%
10518	40	70	35	20	82,4%	89,2%	89,8%	88,4%	74,1%
10519	40	70	35	25	82,7%	89,0%	90,1%	89,1%	75,4%
10520	40	70	35	30	82,9%	88,9%	90,4%	89,8%	76,7%
10521	40	70	35	35	83,2%	88,8%	90,7%	90,4%	77,9%
10522	40	70	35	40	83,4%	88,7%	91,0%	91,0%	79,0%
10523	40	70	35	45	83,7%	88,5%	91,2%	91,5%	80,1%
10524	40	70	35	50	83,9%	88,4%	91,5%	92,0%	81,2%
10525	40	70	35	55	84,2%	88,3%	91,8%	92,5%	82,2%
10526	40	70	35	60	84,4%	88,1%	92,0%	93,0%	83,2%

10527	40	70	35	65	84,6%	88,0%	92,3%	93,4%	84,1%
10528	40	70	35	70	84,8%	87,8%	92,5%	93,8%	85,0%
10529	40	70	35	75	85,1%	87,7%	92,7%	94,2%	85,9%
10530	40	70	35	80	85,3%	87,6%	93,0%	94,6%	86,7%
10531	40	70	40	20	78,6%	86,7%	88,6%	87,8%	73,3%
10532	40	70	40	25	78,9%	86,6%	88,9%	88,5%	74,6%
10533	40	70	40	30	79,2%	86,4%	89,2%	89,2%	75,9%
10534	40	70	40	35	79,4%	86,3%	89,6%	89,8%	77,1%
10535	40	70	40	40	79,7%	86,1%	89,9%	90,4%	78,3%
10536	40	70	40	45	80,0%	86,0%	90,2%	91,0%	79,5%
10537	40	70	40	50	80,3%	85,8%	90,5%	91,6%	80,6%
10538	40	70	40	55	80,6%	85,6%	90,8%	92,1%	81,6%
10539	40	70	40	60	80,9%	85,5%	91,1%	92,6%	82,6%
10540	40	70	40	65	81,1%	85,3%	91,3%	93,0%	83,6%
10541	40	70	40	70	81,4%	85,2%	91,6%	93,5%	84,5%
10542	40	70	40	75	81,7%	85,0%	91,8%	93,9%	85,4%
10543	40	70	40	80	81,9%	84,8%	92,1%	94,3%	86,2%
10544	40	70	45	20	74,1%	83,8%	87,3%	87,1%	72,5%
10545	40	70	45	25	74,5%	83,7%	87,6%	87,9%	73,8%
10546	40	70	45	30	74,8%	83,5%	88,0%	88,6%	75,1%
10547	40	70	45	35	75,1%	83,3%	88,4%	89,3%	76,4%
10548	40	70	45	40	75,5%	83,1%	88,7%	89,9%	77,6%
10549	40	70	45	45	75,8%	82,9%	89,0%	90,5%	78,8%
10550	40	70	45	50	76,1%	82,8%	89,4%	91,1%	79,9%
10551	40	70	45	55	76,4%	82,6%	89,7%	91,6%	81,0%
10552	40	70	45	60	76,7%	82,4%	90,0%	92,1%	82,0%
10553	40	70	45	65	77,0%	82,2%	90,3%	92,6%	83,0%
10554	40	70	45	70	77,4%	82,0%	90,6%	93,1%	83,9%
10555	40	70	45	75	77,7%	81,8%	90,9%	93,5%	84,8%
10556	40	70	45	80	78,0%	81,6%	91,1%	93,9%	85,7%
10557	40	70	50	20	69,1%	80,5%	85,8%	86,4%	71,7%
10558	40	70	50	25	69,5%	80,3%	86,2%	87,2%	73,0%
10559	40	70	50	30	69,9%	80,1%	86,6%	87,9%	74,4%

10560	40	70	50	35	70,2%	79,8%	87,0%	88,7%	75,7%
10561	40	70	50	40	70,6%	79,6%	87,4%	89,3%	76,9%
10562	40	70	50	45	71,0%	79,4%	87,8%	90,0%	78,1%
10563	40	70	50	50	71,3%	79,2%	88,1%	90,6%	79,2%
10564	40	70	50	55	71,7%	79,0%	88,5%	91,2%	80,3%
10565	40	70	50	60	72,0%	78,8%	88,8%	91,7%	81,4%
10566	40	70	50	65	72,4%	78,6%	89,1%	92,2%	82,4%
10567	40	70	50	70	72,7%	78,3%	89,5%	92,7%	83,4%
10568	40	70	50	75	73,1%	78,1%	89,8%	93,1%	84,3%
10569	40	70	50	80	73,4%	77,9%	90,1%	93,6%	85,2%
10570	40	70	55	20	63,6%	76,6%	84,2%	85,7%	70,8%
10571	40	70	55	25	64,0%	76,4%	84,7%	86,5%	72,2%
10572	40	70	55	30	64,4%	76,1%	85,1%	87,3%	73,6%
10573	40	70	55	35	64,8%	75,9%	85,5%	88,0%	74,9%
10574	40	70	55	40	65,2%	75,6%	86,0%	88,7%	76,1%
10575	40	70	55	45	65,6%	75,4%	86,4%	89,4%	77,4%
10576	40	70	55	50	66,0%	75,1%	86,8%	90,0%	78,5%
10577	40	70	55	55	66,4%	74,9%	87,1%	90,6%	79,7%
10578	40	70	55	60	66,8%	74,7%	87,5%	91,2%	80,8%
10579	40	70	55	65	67,2%	74,4%	87,9%	91,8%	81,8%
10580	40	70	55	70	67,6%	74,2%	88,2%	92,3%	82,8%
10581	40	70	55	75	68,0%	73,9%	88,6%	92,7%	83,8%
10582	40	70	55	80	68,4%	73,7%	88,9%	93,2%	84,7%
10583	40	70	60	20	57,7%	72,2%	82,5%	84,9%	70,0%
10584	40	70	60	25	58,2%	71,9%	83,0%	85,8%	71,4%
10585	40	70	60	30	58,6%	71,7%	83,5%	86,6%	72,8%
10586	40	70	60	35	59,0%	71,4%	83,9%	87,4%	74,1%
10587	40	70	60	40	59,4%	71,1%	84,4%	88,1%	75,4%
10588	40	70	60	45	59,9%	70,9%	84,8%	88,8%	76,6%
10589	40	70	60	50	60,3%	70,6%	85,3%	89,5%	77,8%
10590	40	70	60	55	60,7%	70,3%	85,7%	90,1%	79,0%
10591	40	70	60	60	61,1%	70,0%	86,1%	90,7%	80,1%
10592	40	70	60	65	61,5%	69,8%	86,5%	91,3%	81,2%

10593	40	70	60	70	62,0%	69,5%	86,9%	91,8%	82,2%
10594	40	70	60	75	62,4%	69,2%	87,3%	92,3%	83,2%
10595	40	70	60	80	62,8%	68,9%	87,6%	92,8%	84,1%
10596	40	70	65	20	51,6%	67,3%	80,6%	84,1%	69,1%
10597	40	70	65	25	52,1%	67,0%	81,2%	85,0%	70,5%
10598	40	70	65	30	52,5%	66,8%	81,7%	85,9%	71,9%
10599	40	70	65	35	52,9%	66,5%	82,2%	86,7%	73,3%
10600	40	70	65	40	53,4%	66,2%	82,7%	87,5%	74,6%
10601	40	70	65	45	53,8%	65,9%	83,2%	88,2%	75,9%
10602	40	70	65	50	54,3%	65,6%	83,6%	88,9%	77,1%
10603	40	70	65	55	54,7%	65,3%	84,1%	89,6%	78,3%
10604	40	70	65	60	55,1%	65,0%	84,5%	90,2%	79,5%
10605	40	70	65	65	55,6%	64,7%	85,0%	90,8%	80,5%
10606	40	70	65	70	56,0%	64,4%	85,4%	91,3%	81,6%
10607	40	70	65	75	56,4%	64,1%	85,8%	91,9%	82,6%
10608	40	70	65	80	56,9%	63,8%	86,2%	92,4%	83,6%
10609	40	70	70	20	45,5%	62,1%	78,6%	83,3%	68,2%
10610	40	70	70	25	45,9%	61,8%	79,2%	84,2%	69,7%
10611	40	70	70	30	46,3%	61,4%	79,8%	85,1%	71,1%
10612	40	70	70	35	46,8%	61,1%	80,3%	86,0%	72,5%
10613	40	70	70	40	47,2%	60,8%	80,8%	86,8%	73,8%
10614	40	70	70	45	47,7%	60,5%	81,4%	87,5%	75,1%
10615	40	70	70	50	48,1%	60,2%	81,9%	88,3%	76,4%
10616	40	70	70	55	48,5%	59,9%	82,4%	89,0%	77,6%
10617	40	70	70	60	49,0%	59,6%	82,8%	89,6%	78,8%
10618	40	70	70	65	49,4%	59,3%	83,3%	90,3%	79,9%
10619	40	70	70	70	49,9%	58,9%	83,8%	90,8%	81,0%
10620	40	70	70	75	50,3%	58,6%	84,2%	91,4%	82,0%
10621	40	70	70	80	50,7%	58,3%	84,7%	91,9%	83,0%
10622	40	70	75	20	39,4%	56,5%	76,5%	82,4%	67,3%
10623	40	70	75	25	39,9%	56,2%	77,1%	83,4%	68,8%
10624	40	70	75	30	40,3%	55,9%	77,7%	84,3%	70,2%
10625	40	70	75	35	40,7%	55,5%	78,3%	85,2%	71,7%

10626	40	70	75	40	41,1%	55,2%	78,8%	86,1%	73,0%
10627	40	70	75	45	41,6%	54,9%	79,4%	86,9%	74,4%
10628	40	70	75	50	42,0%	54,6%	79,9%	87,6%	75,6%
10629	40	70	75	55	42,4%	54,2%	80,5%	88,4%	76,9%
10630	40	70	75	60	42,9%	53,9%	81,0%	89,1%	78,1%
10631	40	70	75	65	43,3%	53,6%	81,5%	89,7%	79,2%
10632	40	70	75	70	43,7%	53,3%	82,0%	90,3%	80,3%
10633	40	70	75	75	44,2%	52,9%	82,5%	90,9%	81,4%
10634	40	70	75	80	44,6%	52,6%	83,0%	91,5%	82,4%
10635	40	70	80	20	33,7%	50,8%	74,2%	81,5%	66,4%
10636	40	70	80	25	34,1%	50,4%	74,8%	82,5%	67,9%
10637	40	70	80	30	34,5%	50,1%	75,4%	83,5%	69,4%
10638	40	70	80	35	34,9%	49,8%	76,1%	84,4%	70,8%
10639	40	70	80	40	35,3%	49,5%	76,7%	85,3%	72,2%
10640	40	70	80	45	35,7%	49,1%	77,3%	86,2%	73,6%
10641	40	70	80	50	36,1%	48,8%	77,9%	87,0%	74,9%
10642	40	70	80	55	36,5%	48,5%	78,5%	87,7%	76,1%
10643	40	70	80	60	36,9%	48,1%	79,0%	88,4%	77,4%
10644	40	70	80	65	37,4%	47,8%	79,6%	89,1%	78,5%
10645	40	70	80	70	37,8%	47,5%	80,1%	89,8%	79,7%
10646	40	70	80	75	38,2%	47,2%	80,7%	90,4%	80,8%
10647	40	70	80	80	38,6%	46,8%	81,2%	91,0%	81,8%
10648	40	75	20	20	91,5%	94,7%	92,5%	89,5%	74,7%
10649	40	75	20	25	91,6%	94,6%	92,7%	90,1%	75,9%
10650	40	75	20	30	91,8%	94,6%	93,0%	90,7%	77,2%
10651	40	75	20	35	91,9%	94,5%	93,2%	91,3%	78,3%
10652	40	75	20	40	92,0%	94,4%	93,4%	91,8%	79,5%
10653	40	75	20	45	92,2%	94,3%	93,6%	92,3%	80,6%
10654	40	75	20	50	92,3%	94,3%	93,8%	92,8%	81,6%
10655	40	75	20	55	92,4%	94,2%	94,0%	93,2%	82,6%
10656	40	75	20	60	92,5%	94,1%	94,2%	93,7%	83,6%
10657	40	75	20	65	92,7%	94,1%	94,4%	94,1%	84,5%
10658	40	75	20	70	92,8%	94,0%	94,5%	94,4%	85,4%

10659	40	75	20	75	92,9%	93,9%	94,7%	94,8%	86,2%
10660	40	75	20	80	93,0%	93,8%	94,9%	95,1%	87,0%
10661	40	75	25	20	89,4%	93,4%	91,6%	88,9%	73,9%
10662	40	75	25	25	89,5%	93,3%	91,9%	89,6%	75,2%
10663	40	75	25	30	89,7%	93,2%	92,1%	90,2%	76,4%
10664	40	75	25	35	89,9%	93,2%	92,4%	90,8%	77,6%
10665	40	75	25	40	90,0%	93,1%	92,6%	91,3%	78,8%
10666	40	75	25	45	90,2%	93,0%	92,8%	91,9%	79,9%
10667	40	75	25	50	90,3%	92,9%	93,0%	92,4%	81,0%
10668	40	75	25	55	90,5%	92,8%	93,3%	92,8%	82,0%
10669	40	75	25	60	90,6%	92,7%	93,5%	93,3%	83,0%
10670	40	75	25	65	90,8%	92,6%	93,7%	93,7%	84,0%
10671	40	75	25	70	90,9%	92,5%	93,9%	94,1%	84,9%
10672	40	75	25	75	91,1%	92,5%	94,1%	94,5%	85,7%
10673	40	75	25	80	91,2%	92,4%	94,3%	94,8%	86,5%
10674	40	75	30	20	86,8%	91,8%	90,6%	88,3%	73,1%
10675	40	75	30	25	87,0%	91,7%	90,9%	89,0%	74,4%
10676	40	75	30	30	87,2%	91,6%	91,2%	89,6%	75,7%
10677	40	75	30	35	87,4%	91,5%	91,4%	90,3%	76,9%
10678	40	75	30	40	87,6%	91,4%	91,7%	90,8%	78,1%
10679	40	75	30	45	87,8%	91,3%	91,9%	91,4%	79,3%
10680	40	75	30	50	87,9%	91,2%	92,2%	91,9%	80,4%
10681	40	75	30	55	88,1%	91,1%	92,4%	92,4%	81,4%
10682	40	75	30	60	88,3%	91,0%	92,7%	92,9%	82,4%
10683	40	75	30	65	88,5%	90,9%	92,9%	93,3%	83,4%
10684	40	75	30	70	88,7%	90,8%	93,1%	93,8%	84,3%
10685	40	75	30	75	88,9%	90,7%	93,3%	94,1%	85,2%
10686	40	75	30	80	89,0%	90,6%	93,5%	94,5%	86,1%
10687	40	75	35	20	83,7%	89,9%	89,5%	87,6%	72,2%
10688	40	75	35	25	83,9%	89,8%	89,8%	88,4%	73,6%
10689	40	75	35	30	84,2%	89,7%	90,1%	89,1%	74,9%
10690	40	75	35	35	84,4%	89,6%	90,4%	89,7%	76,2%
10691	40	75	35	40	84,6%	89,4%	90,7%	90,3%	77,4%

10692	40	75	35	45	84,9%	89,3%	91,0%	90,9%	78,6%
10693	40	75	35	50	85,1%	89,2%	91,3%	91,5%	79,7%
10694	40	75	35	55	85,3%	89,1%	91,5%	92,0%	80,8%
10695	40	75	35	60	85,5%	88,9%	91,8%	92,5%	81,8%
10696	40	75	35	65	85,7%	88,8%	92,0%	92,9%	82,8%
10697	40	75	35	70	86,0%	88,7%	92,3%	93,4%	83,8%
10698	40	75	35	75	86,2%	88,5%	92,5%	93,8%	84,7%
10699	40	75	35	80	86,4%	88,4%	92,7%	94,2%	85,5%
10700	40	75	40	20	80,0%	87,6%	88,3%	87,0%	71,4%
10701	40	75	40	25	80,3%	87,5%	88,6%	87,7%	72,8%
10702	40	75	40	30	80,6%	87,3%	88,9%	88,4%	74,1%
10703	40	75	40	35	80,9%	87,2%	89,3%	89,1%	75,4%
10704	40	75	40	40	81,1%	87,0%	89,6%	89,8%	76,7%
10705	40	75	40	45	81,4%	86,9%	89,9%	90,4%	77,9%
10706	40	75	40	50	81,7%	86,7%	90,2%	91,0%	79,0%
10707	40	75	40	55	81,9%	86,6%	90,5%	91,5%	80,1%
10708	40	75	40	60	82,2%	86,4%	90,8%	92,0%	81,2%
10709	40	75	40	65	82,4%	86,3%	91,1%	92,5%	82,2%
10710	40	75	40	70	82,7%	86,1%	91,3%	93,0%	83,2%
10711	40	75	40	75	82,9%	86,0%	91,6%	93,4%	84,1%
10712	40	75	40	80	83,2%	85,8%	91,9%	93,8%	85,0%
10713	40	75	45	20	75,8%	84,9%	86,9%	86,3%	70,6%
10714	40	75	45	25	76,1%	84,7%	87,3%	87,0%	72,0%
10715	40	75	45	30	76,4%	84,5%	87,7%	87,8%	73,3%
10716	40	75	45	35	76,7%	84,4%	88,0%	88,5%	74,6%
10717	40	75	45	40	77,1%	84,2%	88,4%	89,2%	75,9%
10718	40	75	45	45	77,4%	84,0%	88,7%	89,9%	77,1%
10719	40	75	45	50	77,7%	83,9%	89,1%	90,5%	78,3%
10720	40	75	45	55	78,0%	83,7%	89,4%	91,0%	79,5%
10721	40	75	45	60	78,3%	83,5%	89,7%	91,6%	80,6%
10722	40	75	45	65	78,6%	83,3%	90,0%	92,1%	81,6%
10723	40	75	45	70	78,9%	83,1%	90,3%	92,6%	82,6%
10724	40	75	45	75	79,2%	82,9%	90,6%	93,1%	83,6%

10725	40	75	45	80	79,5%	82,8%	90,9%	93,5%	84,5%
10726	40	75	50	20	71,0%	81,7%	85,4%	85,5%	69,7%
10727	40	75	50	25	71,3%	81,5%	85,8%	86,3%	71,1%
10728	40	75	50	30	71,7%	81,3%	86,3%	87,1%	72,5%
10729	40	75	50	35	72,1%	81,1%	86,6%	87,9%	73,8%
10730	40	75	50	40	72,4%	80,9%	87,0%	88,6%	75,1%
10731	40	75	50	45	72,8%	80,7%	87,4%	89,3%	76,4%
10732	40	75	50	50	73,1%	80,5%	87,8%	89,9%	77,6%
10733	40	75	50	55	73,5%	80,3%	88,1%	90,5%	78,8%
10734	40	75	50	60	73,8%	80,1%	88,5%	91,1%	79,9%
10735	40	75	50	65	74,1%	79,9%	88,8%	91,7%	81,0%
10736	40	75	50	70	74,5%	79,6%	89,2%	92,2%	82,0%
10737	40	75	50	75	74,8%	79,4%	89,5%	92,6%	83,0%
10738	40	75	50	80	75,1%	79,2%	89,8%	93,1%	84,0%
10739	40	75	55	20	65,6%	78,0%	83,8%	84,7%	68,8%
10740	40	75	55	25	66,0%	77,7%	84,3%	85,6%	70,3%
10741	40	75	55	30	66,4%	77,5%	84,7%	86,4%	71,7%
10742	40	75	55	35	66,8%	77,3%	85,1%	87,2%	73,0%
10743	40	75	55	40	67,2%	77,1%	85,6%	88,0%	74,4%
10744	40	75	55	45	67,6%	76,8%	86,0%	88,7%	75,7%
10745	40	75	55	50	68,0%	76,6%	86,4%	89,4%	76,9%
10746	40	75	55	55	68,4%	76,4%	86,8%	90,0%	78,1%
10747	40	75	55	60	68,7%	76,1%	87,2%	90,6%	79,2%
10748	40	75	55	65	69,1%	75,9%	87,5%	91,2%	80,3%
10749	40	75	55	70	69,5%	75,6%	87,9%	91,7%	81,4%
10750	40	75	55	75	69,9%	75,4%	88,3%	92,2%	82,4%
10751	40	75	55	80	70,2%	75,2%	88,6%	92,7%	83,4%
10752	40	75	60	20	59,9%	73,7%	82,1%	83,9%	67,9%
10753	40	75	60	25	60,3%	73,5%	82,5%	84,8%	69,4%
10754	40	75	60	30	60,7%	73,2%	83,0%	85,7%	70,8%
10755	40	75	60	35	61,1%	73,0%	83,5%	86,5%	72,2%
10756	40	75	60	40	61,6%	72,7%	84,0%	87,3%	73,6%
10757	40	75	60	45	62,0%	72,5%	84,4%	88,1%	74,9%

10758	40	75	60	50	62,4%	72,2%	84,9%	88,8%	76,2%
10759	40	75	60	55	62,8%	71,9%	85,3%	89,4%	77,4%
10760	40	75	60	60	63,2%	71,7%	85,7%	90,1%	78,6%
10761	40	75	60	65	63,6%	71,4%	86,1%	90,7%	79,7%
10762	40	75	60	70	64,0%	71,1%	86,5%	91,2%	80,8%
10763	40	75	60	75	64,4%	70,9%	86,9%	91,8%	81,8%
10764	40	75	60	80	64,8%	70,6%	87,3%	92,3%	82,8%
10765	40	75	65	20	53,8%	69,0%	80,1%	83,1%	67,0%
10766	40	75	65	25	54,3%	68,8%	80,7%	84,0%	68,5%
10767	40	75	65	30	54,7%	68,5%	81,2%	84,9%	70,0%
10768	40	75	65	35	55,1%	68,2%	81,7%	85,8%	71,4%
10769	40	75	65	40	55,6%	67,9%	82,2%	86,6%	72,8%
10770	40	75	65	45	56,0%	67,6%	82,7%	87,4%	74,1%
10771	40	75	65	50	56,4%	67,3%	83,2%	88,1%	75,4%
10772	40	75	65	55	56,9%	67,0%	83,7%	88,8%	76,6%
10773	40	75	65	60	57,3%	66,8%	84,1%	89,5%	77,8%
10774	40	75	65	65	57,7%	66,5%	84,6%	90,1%	79,0%
10775	40	75	65	70	58,2%	66,2%	85,0%	90,7%	80,1%
10776	40	75	65	75	58,6%	65,9%	85,4%	91,3%	81,2%
10777	40	75	65	80	59,0%	65,6%	85,9%	91,8%	82,2%
10778	40	75	70	20	47,7%	63,9%	78,1%	82,2%	66,1%
10779	40	75	70	25	48,1%	63,6%	78,7%	83,2%	67,6%
10780	40	75	70	30	48,5%	63,3%	79,2%	84,1%	69,1%
10781	40	75	70	35	49,0%	63,0%	79,8%	85,0%	70,5%
10782	40	75	70	40	49,4%	62,7%	80,3%	85,9%	71,9%
10783	40	75	70	45	49,9%	62,4%	80,9%	86,7%	73,3%
10784	40	75	70	50	50,3%	62,1%	81,4%	87,5%	74,6%
10785	40	75	70	55	50,8%	61,8%	81,9%	88,2%	75,9%
10786	40	75	70	60	51,2%	61,5%	82,4%	88,9%	77,1%
10787	40	75	70	65	51,6%	61,1%	82,9%	89,6%	78,3%
10788	40	75	70	70	52,1%	60,8%	83,4%	90,2%	79,5%
10789	40	75	70	75	52,5%	60,5%	83,8%	90,8%	80,6%
10790	40	75	70	80	53,0%	60,2%	84,3%	91,4%	81,6%

10791	40	75	75	20	41,6%	58,4%	75,9%	81,3%	65,2%
10792	40	75	75	25	42,0%	58,1%	76,5%	82,3%	66,7%
10793	40	75	75	30	42,4%	57,8%	77,1%	83,3%	68,2%
10794	40	75	75	35	42,9%	57,5%	77,7%	84,3%	69,7%
10795	40	75	75	40	43,3%	57,1%	78,3%	85,1%	71,1%
10796	40	75	75	45	43,7%	56,8%	78,9%	86,0%	72,5%
10797	40	75	75	50	44,2%	56,5%	79,4%	86,8%	73,8%
10798	40	75	75	55	44,6%	56,2%	80,0%	87,6%	75,1%
10799	40	75	75	60	45,0%	55,9%	80,5%	88,3%	76,4%
10800	40	75	75	65	45,5%	55,5%	81,0%	89,0%	77,6%
10801	40	75	75	70	45,9%	55,2%	81,6%	89,7%	78,8%
10802	40	75	75	75	46,3%	54,9%	82,1%	90,3%	79,9%
10803	40	75	75	80	46,8%	54,6%	82,6%	90,9%	81,0%
10804	40	75	80	20	35,7%	52,7%	73,6%	80,4%	64,2%
10805	40	75	80	25	36,1%	52,4%	74,2%	81,4%	65,8%
10806	40	75	80	30	36,5%	52,1%	74,9%	82,5%	67,3%
10807	40	75	80	35	37,0%	51,7%	75,5%	83,4%	68,8%
10808	40	75	80	40	37,4%	51,4%	76,1%	84,4%	70,2%
10809	40	75	80	45	37,8%	51,1%	76,7%	85,2%	71,7%
10810	40	75	80	50	38,2%	50,8%	77,3%	86,1%	73,0%
10811	40	75	80	55	38,6%	50,4%	77,9%	86,9%	74,4%
10812	40	75	80	60	39,0%	50,1%	78,5%	87,7%	75,6%
10813	40	75	80	65	39,4%	49,8%	79,1%	88,4%	76,9%
10814	40	75	80	70	39,9%	49,5%	79,6%	89,1%	78,1%
10815	40	75	80	75	40,3%	49,1%	80,2%	89,7%	79,2%
10816	40	75	80	80	40,7%	48,8%	80,7%	90,4%	80,3%
10817	40	80	20	20	92,2%	95,1%	92,3%	88,8%	72,8%
10818	40	80	20	25	92,3%	95,0%	92,5%	89,4%	74,1%
10819	40	80	20	30	92,4%	94,9%	92,8%	90,1%	75,4%
10820	40	80	20	35	92,5%	94,9%	93,0%	90,7%	76,7%
10821	40	80	20	40	92,7%	94,8%	93,2%	91,2%	77,9%
10822	40	80	20	45	92,8%	94,8%	93,4%	91,8%	79,0%
10823	40	80	20	50	92,9%	94,7%	93,6%	92,3%	80,1%

10824	40	80	20	55	93,0%	94,6%	93,8%	92,8%	81,2%
10825	40	80	20	60	93,1%	94,6%	94,0%	93,2%	82,2%
10826	40	80	20	65	93,2%	94,5%	94,2%	93,6%	83,2%
10827	40	80	20	70	93,3%	94,4%	94,4%	94,0%	84,2%
10828	40	80	20	75	93,5%	94,3%	94,6%	94,4%	85,0%
10829	40	80	20	80	93,6%	94,3%	94,7%	94,8%	85,9%
10830	40	80	25	20	90,2%	93,9%	91,4%	88,1%	72,0%
10831	40	80	25	25	90,3%	93,8%	91,6%	88,8%	73,3%
10832	40	80	25	30	90,5%	93,7%	91,9%	89,5%	74,7%
10833	40	80	25	35	90,6%	93,6%	92,1%	90,1%	75,9%
10834	40	80	25	40	90,8%	93,6%	92,4%	90,7%	77,2%
10835	40	80	25	45	90,9%	93,5%	92,6%	91,3%	78,3%
10836	40	80	25	50	91,1%	93,4%	92,8%	91,8%	79,5%
10837	40	80	25	55	91,2%	93,3%	93,1%	92,3%	80,6%
10838	40	80	25	60	91,4%	93,2%	93,3%	92,8%	81,6%
10839	40	80	25	65	91,5%	93,2%	93,5%	93,3%	82,6%
10840	40	80	25	70	91,6%	93,1%	93,7%	93,7%	83,6%
10841	40	80	25	75	91,8%	93,0%	93,9%	94,1%	84,5%
10842	40	80	25	80	91,9%	92,9%	94,1%	94,4%	85,4%
10843	40	80	30	20	87,8%	92,4%	90,3%	87,5%	71,1%
10844	40	80	30	25	88,0%	92,3%	90,6%	88,2%	72,5%
10845	40	80	30	30	88,1%	92,2%	90,9%	88,9%	73,9%
10846	40	80	30	35	88,3%	92,1%	91,2%	89,6%	75,2%
10847	40	80	30	40	88,5%	92,0%	91,4%	90,2%	76,4%
10848	40	80	30	45	88,7%	91,9%	91,7%	90,8%	77,6%
10849	40	80	30	50	88,9%	91,8%	92,0%	91,4%	78,8%
10850	40	80	30	55	89,0%	91,7%	92,2%	91,9%	79,9%
10851	40	80	30	60	89,2%	91,6%	92,5%	92,4%	81,0%
10852	40	80	30	65	89,4%	91,5%	92,7%	92,9%	82,0%
10853	40	80	30	70	89,5%	91,4%	92,9%	93,3%	83,0%
10854	40	80	30	75	89,7%	91,3%	93,1%	93,7%	84,0%
10855	40	80	30	80	89,9%	91,2%	93,3%	94,1%	84,9%
10856	40	80	35	20	84,9%	90,6%	89,2%	86,8%	70,3%

10857	40	80	35	25	85,1%	90,5%	89,5%	87,6%	71,7%
10858	40	80	35	30	85,3%	90,4%	89,8%	88,3%	73,1%
10859	40	80	35	35	85,5%	90,3%	90,1%	89,0%	74,4%
10860	40	80	35	40	85,7%	90,1%	90,4%	89,7%	75,7%
10861	40	80	35	45	86,0%	90,0%	90,7%	90,3%	76,9%
10862	40	80	35	50	86,2%	89,9%	91,0%	90,9%	78,1%
10863	40	80	35	55	86,4%	89,8%	91,3%	91,4%	79,3%
10864	40	80	35	60	86,6%	89,7%	91,5%	92,0%	80,4%
10865	40	80	35	65	86,8%	89,6%	91,8%	92,4%	81,4%
10866	40	80	35	70	87,0%	89,4%	92,0%	92,9%	82,4%
10867	40	80	35	75	87,2%	89,3%	92,3%	93,4%	83,4%
10868	40	80	35	80	87,4%	89,2%	92,5%	93,8%	84,3%
10869	40	80	40	20	81,4%	88,4%	87,9%	86,1%	69,4%
10870	40	80	40	25	81,7%	88,3%	88,3%	86,9%	70,9%
10871	40	80	40	30	81,9%	88,2%	88,6%	87,7%	72,2%
10872	40	80	40	35	82,2%	88,0%	89,0%	88,4%	73,6%
10873	40	80	40	40	82,5%	87,9%	89,3%	89,1%	74,9%
10874	40	80	40	45	82,7%	87,8%	89,6%	89,7%	76,2%
10875	40	80	40	50	83,0%	87,6%	89,9%	90,4%	77,4%
10876	40	80	40	55	83,2%	87,5%	90,2%	90,9%	78,6%
10877	40	80	40	60	83,4%	87,3%	90,5%	91,5%	79,7%
10878	40	80	40	65	83,7%	87,2%	90,8%	92,0%	80,8%
10879	40	80	40	70	83,9%	87,0%	91,1%	92,5%	81,8%
10880	40	80	40	75	84,2%	86,9%	91,4%	93,0%	82,8%
10881	40	80	40	80	84,4%	86,7%	91,6%	93,4%	83,8%
10882	40	80	45	20	77,4%	85,9%	86,5%	85,3%	68,5%
10883	40	80	45	25	77,7%	85,7%	86,9%	86,2%	70,0%
10884	40	80	45	30	78,0%	85,5%	87,3%	87,0%	71,4%
10885	40	80	45	35	78,3%	85,4%	87,7%	87,8%	72,8%
10886	40	80	45	40	78,6%	85,2%	88,0%	88,5%	74,1%
10887	40	80	45	45	78,9%	85,1%	88,4%	89,2%	75,4%
10888	40	80	45	50	79,2%	84,9%	88,7%	89,8%	76,7%
10889	40	80	45	55	79,5%	84,7%	89,1%	90,4%	77,9%

10890	40	80	45	60	79,8%	84,6%	89,4%	91,0%	79,0%
10891	40	80	45	65	80,0%	84,4%	89,7%	91,6%	80,1%
10892	40	80	45	70	80,3%	84,2%	90,0%	92,1%	81,2%
10893	40	80	45	75	80,6%	84,0%	90,3%	92,6%	82,2%
10894	40	80	45	80	80,9%	83,9%	90,6%	93,0%	83,2%
10895	40	80	50	20	72,8%	82,8%	85,0%	84,6%	67,6%
10896	40	80	50	25	73,1%	82,6%	85,5%	85,4%	69,1%
10897	40	80	50	30	73,5%	82,5%	85,9%	86,3%	70,6%
10898	40	80	50	35	73,8%	82,3%	86,3%	87,1%	72,0%
10899	40	80	50	40	74,1%	82,1%	86,7%	87,8%	73,3%
10900	40	80	50	45	74,5%	81,9%	87,1%	88,6%	74,6%
10901	40	80	50	50	74,8%	81,7%	87,4%	89,2%	75,9%
10902	40	80	50	55	75,1%	81,5%	87,8%	89,9%	77,1%
10903	40	80	50	60	75,5%	81,3%	88,2%	90,5%	78,3%
10904	40	80	50	65	75,8%	81,1%	88,5%	91,1%	79,5%
10905	40	80	50	70	76,1%	80,9%	88,9%	91,6%	80,6%
10906	40	80	50	75	76,4%	80,7%	89,2%	92,1%	81,6%
10907	40	80	50	80	76,8%	80,5%	89,5%	92,6%	82,6%
10908	40	80	55	20	67,6%	79,3%	83,4%	83,8%	66,7%
10909	40	80	55	25	68,0%	79,1%	83,8%	84,7%	68,2%
10910	40	80	55	30	68,4%	78,9%	84,3%	85,5%	69,7%
10911	40	80	55	35	68,8%	78,6%	84,7%	86,4%	71,1%
10912	40	80	55	40	69,1%	78,4%	85,2%	87,2%	72,5%
10913	40	80	55	45	69,5%	78,2%	85,6%	87,9%	73,9%
10914	40	80	55	50	69,9%	78,0%	86,0%	88,6%	75,2%
10915	40	80	55	55	70,3%	77,7%	86,4%	89,3%	76,4%
10916	40	80	55	60	70,6%	77,5%	86,8%	90,0%	77,6%
10917	40	80	55	65	71,0%	77,3%	87,2%	90,6%	78,8%
10918	40	80	55	70	71,3%	77,1%	87,6%	91,1%	79,9%
10919	40	80	55	75	71,7%	76,8%	87,9%	91,7%	81,0%
10920	40	80	55	80	72,1%	76,6%	88,3%	92,2%	82,0%
10921	40	80	60	20	62,0%	75,2%	81,6%	82,9%	65,8%
10922	40	80	60	25	62,4%	75,0%	82,1%	83,9%	67,3%

10923	40	80	60	30	62,8%	74,8%	82,6%	84,8%	68,8%
10924	40	80	60	35	63,2%	74,5%	83,1%	85,6%	70,3%
10925	40	80	60	40	63,6%	74,3%	83,5%	86,5%	71,7%
10926	40	80	60	45	64,0%	74,0%	84,0%	87,3%	73,0%
10927	40	80	60	50	64,5%	73,8%	84,4%	88,0%	74,4%
10928	40	80	60	55	64,9%	73,5%	84,9%	88,7%	75,7%
10929	40	80	60	60	65,3%	73,2%	85,3%	89,4%	76,9%
10930	40	80	60	65	65,7%	73,0%	85,7%	90,0%	78,1%
10931	40	80	60	70	66,0%	72,7%	86,1%	90,6%	79,2%
10932	40	80	60	75	66,4%	72,5%	86,5%	91,2%	80,3%
10933	40	80	60	80	66,8%	72,2%	86,9%	91,7%	81,4%
10934	40	80	65	20	56,0%	70,7%	79,6%	82,0%	64,9%
10935	40	80	65	25	56,5%	70,4%	80,2%	83,0%	66,4%
10936	40	80	65	30	56,9%	70,2%	80,7%	84,0%	67,9%
10937	40	80	65	35	57,3%	69,9%	81,2%	84,9%	69,4%
10938	40	80	65	40	57,8%	69,6%	81,8%	85,7%	70,8%
10939	40	80	65	45	58,2%	69,3%	82,3%	86,6%	72,2%
10940	40	80	65	50	58,6%	69,0%	82,7%	87,3%	73,6%
10941	40	80	65	55	59,0%	68,8%	83,2%	88,1%	74,9%
10942	40	80	65	60	59,5%	68,5%	83,7%	88,8%	76,2%
10943	40	80	65	65	59,9%	68,2%	84,2%	89,5%	77,4%
10944	40	80	65	70	60,3%	67,9%	84,6%	90,1%	78,6%
10945	40	80	65	75	60,7%	67,6%	85,0%	90,7%	79,7%
10946	40	80	65	80	61,2%	67,3%	85,5%	91,3%	80,8%
10947	40	80	70	20	49,9%	65,7%	77,5%	81,1%	63,9%
10948	40	80	70	25	50,3%	65,4%	78,1%	82,1%	65,5%
10949	40	80	70	30	50,8%	65,1%	78,7%	83,1%	67,0%
10950	40	80	70	35	51,2%	64,8%	79,3%	84,1%	68,5%
10951	40	80	70	40	51,6%	64,5%	79,8%	85,0%	70,0%
10952	40	80	70	45	52,1%	64,2%	80,4%	85,8%	71,4%
10953	40	80	70	50	52,5%	63,9%	80,9%	86,7%	72,8%
10954	40	80	70	55	53,0%	63,6%	81,4%	87,4%	74,1%
10955	40	80	70	60	53,4%	63,3%	81,9%	88,2%	75,4%

10956	40	80	70	65	53,8%	63,0%	82,4%	88,9%	76,6%
10957	40	80	70	70	54,3%	62,7%	82,9%	89,5%	77,9%
10958	40	80	70	75	54,7%	62,4%	83,4%	90,2%	79,0%
10959	40	80	70	80	55,2%	62,1%	83,9%	90,8%	80,1%
10960	40	80	75	20	43,7%	60,3%	75,3%	80,2%	63,0%
10961	40	80	75	25	44,2%	60,0%	75,9%	81,2%	64,6%
10962	40	80	75	30	44,6%	59,7%	76,6%	82,3%	66,1%
10963	40	80	75	35	45,1%	59,4%	77,2%	83,2%	67,6%
10964	40	80	75	40	45,5%	59,1%	77,8%	84,2%	69,1%
10965	40	80	75	45	45,9%	58,7%	78,3%	85,1%	70,5%
10966	40	80	75	50	46,4%	58,4%	78,9%	85,9%	71,9%
10967	40	80	75	55	46,8%	58,1%	79,5%	86,8%	73,3%
10968	40	80	75	60	47,2%	57,8%	80,0%	87,5%	74,6%
10969	40	80	75	65	47,7%	57,5%	80,5%	88,3%	75,9%
10970	40	80	75	70	48,1%	57,2%	81,1%	89,0%	77,1%
10971	40	80	75	75	48,6%	56,8%	81,6%	89,6%	78,3%
10972	40	80	75	80	49,0%	56,5%	82,1%	90,2%	79,5%
10973	40	80	80	20	37,8%	54,7%	72,9%	79,2%	62,0%
10974	40	80	80	25	38,2%	54,4%	73,6%	80,3%	63,6%
10975	40	80	80	30	38,6%	54,0%	74,3%	81,4%	65,2%
10976	40	80	80	35	39,0%	53,7%	74,9%	82,4%	66,7%
10977	40	80	80	40	39,5%	53,4%	75,5%	83,4%	68,2%
10978	40	80	80	45	39,9%	53,1%	76,2%	84,3%	69,7%
10979	40	80	80	50	40,3%	52,7%	76,8%	85,2%	71,1%
10980	40	80	80	55	40,7%	52,4%	77,4%	86,0%	72,5%
10981	40	80	80	60	41,2%	52,1%	78,0%	86,8%	73,8%
10982	40	80	80	65	41,6%	51,8%	78,5%	87,6%	75,1%
10983	40	80	80	70	42,0%	51,4%	79,1%	88,3%	76,4%
10984	40	80	80	75	42,4%	51,1%	79,7%	89,0%	77,6%
10985	40	80	80	80	42,9%	50,8%	80,2%	89,7%	78,8%
10986	45	20	20	20	83,8%	91,3%	96,4%	96,6%	91,9%
10987	45	20	20	25	84,1%	91,2%	96,5%	96,9%	92,4%
10988	45	20	20	30	84,3%	91,1%	96,6%	97,1%	92,9%

10989	45	20	20	35	84,5%	91,0%	96,7%	97,2%	93,3%
10990	45	20	20	40	84,8%	90,9%	96,8%	97,4%	93,7%
10991	45	20	20	45	85,0%	90,7%	96,9%	97,6%	94,1%
10992	45	20	20	50	85,2%	90,6%	97,0%	97,8%	94,5%
10993	45	20	20	55	85,4%	90,5%	97,1%	97,9%	94,8%
10994	45	20	20	60	85,6%	90,4%	97,2%	98,0%	95,2%
10995	45	20	20	65	85,9%	90,3%	97,3%	98,2%	95,5%
10996	45	20	20	70	86,1%	90,2%	97,4%	98,3%	95,8%
10997	45	20	20	75	86,3%	90,1%	97,5%	98,4%	96,0%
10998	45	20	20	80	86,5%	89,9%	97,6%	98,5%	96,3%
10999	45	20	25	20	80,2%	89,3%	95,9%	96,4%	91,6%
11000	45	20	25	25	80,5%	89,1%	96,1%	96,7%	92,1%
11001	45	20	25	30	80,7%	89,0%	96,2%	96,9%	92,6%
11002	45	20	25	35	81,0%	88,9%	96,3%	97,1%	93,1%
11003	45	20	25	40	81,3%	88,7%	96,4%	97,3%	93,5%
11004	45	20	25	45	81,6%	88,6%	96,5%	97,4%	93,9%
11005	45	20	25	50	81,8%	88,5%	96,6%	97,6%	94,3%
11006	45	20	25	55	82,1%	88,3%	96,8%	97,8%	94,6%
11007	45	20	25	60	82,3%	88,2%	96,9%	97,9%	95,0%
11008	45	20	25	65	82,6%	88,1%	97,0%	98,1%	95,3%
11009	45	20	25	70	82,8%	87,9%	97,1%	98,2%	95,6%
11010	45	20	25	75	83,1%	87,8%	97,2%	98,3%	95,9%
11011	45	20	25	80	83,3%	87,7%	97,2%	98,4%	96,1%
11012	45	20	30	20	76,0%	86,8%	95,4%	96,2%	91,3%
11013	45	20	30	25	76,3%	86,7%	95,6%	96,5%	91,8%
11014	45	20	30	30	76,6%	86,5%	95,7%	96,7%	92,3%
11015	45	20	30	35	76,9%	86,4%	95,8%	96,9%	92,8%
11016	45	20	30	40	77,2%	86,2%	96,0%	97,1%	93,2%
11017	45	20	30	45	77,5%	86,1%	96,1%	97,3%	93,6%
11018	45	20	30	50	77,9%	85,9%	96,2%	97,5%	94,0%
11019	45	20	30	55	78,2%	85,8%	96,3%	97,6%	94,4%
11020	45	20	30	60	78,5%	85,6%	96,5%	97,8%	94,8%
11021	45	20	30	65	78,7%	85,4%	96,6%	97,9%	95,1%

11022	45	20	30	70	79,0%	85,3%	96,7%	98,1%	95,4%
11023	45	20	30	75	79,3%	85,1%	96,8%	98,2%	95,7%
11024	45	20	30	80	79,6%	84,9%	96,9%	98,3%	96,0%
11025	45	20	35	20	71,2%	84,0%	94,8%	96,0%	90,9%
11026	45	20	35	25	71,5%	83,8%	95,0%	96,2%	91,5%
11027	45	20	35	30	71,9%	83,6%	95,2%	96,5%	92,0%
11028	45	20	35	35	72,3%	83,4%	95,3%	96,7%	92,5%
11029	45	20	35	40	72,6%	83,2%	95,5%	96,9%	93,0%
11030	45	20	35	45	73,0%	83,1%	95,6%	97,1%	93,4%
11031	45	20	35	50	73,3%	82,9%	95,7%	97,3%	93,8%
11032	45	20	35	55	73,6%	82,7%	95,9%	97,5%	94,2%
11033	45	20	35	60	74,0%	82,5%	96,0%	97,7%	94,6%
11034	45	20	35	65	74,3%	82,3%	96,1%	97,8%	94,9%
11035	45	20	35	70	74,7%	82,1%	96,3%	97,9%	95,2%
11036	45	20	35	75	75,0%	81,9%	96,4%	98,1%	95,5%
11037	45	20	35	80	75,3%	81,7%	96,5%	98,2%	95,8%
11038	45	20	40	20	65,9%	80,6%	94,2%	95,8%	90,6%
11039	45	20	40	25	66,3%	80,4%	94,4%	96,0%	91,2%
11040	45	20	40	30	66,7%	80,2%	94,5%	96,3%	91,7%
11041	45	20	40	35	67,0%	80,0%	94,7%	96,5%	92,2%
11042	45	20	40	40	67,4%	79,8%	94,9%	96,7%	92,7%
11043	45	20	40	45	67,8%	79,6%	95,1%	97,0%	93,1%
11044	45	20	40	50	68,2%	79,4%	95,2%	97,1%	93,6%
11045	45	20	40	55	68,6%	79,1%	95,4%	97,3%	94,0%
11046	45	20	40	60	69,0%	78,9%	95,5%	97,5%	94,3%
11047	45	20	40	65	69,3%	78,7%	95,7%	97,7%	94,7%
11048	45	20	40	70	69,7%	78,5%	95,8%	97,8%	95,0%
11049	45	20	40	75	70,1%	78,3%	95,9%	98,0%	95,3%
11050	45	20	40	80	70,5%	78,0%	96,1%	98,1%	95,6%
11051	45	20	45	20	60,1%	76,7%	93,5%	95,5%	90,2%
11052	45	20	45	25	60,5%	76,5%	93,7%	95,8%	90,8%
11053	45	20	45	30	61,0%	76,3%	93,9%	96,1%	91,4%
11054	45	20	45	35	61,4%	76,0%	94,1%	96,3%	91,9%

11055	45	20	45	40	61,8%	75,8%	94,3%	96,5%	92,4%
11056	45	20	45	45	62,2%	75,6%	94,4%	96,8%	92,9%
11057	45	20	45	50	62,6%	75,3%	94,6%	97,0%	93,3%
11058	45	20	45	55	63,0%	75,1%	94,8%	97,2%	93,7%
11059	45	20	45	60	63,4%	74,8%	94,9%	97,4%	94,1%
11060	45	20	45	65	63,9%	74,6%	95,1%	97,5%	94,5%
11061	45	20	45	70	64,3%	74,3%	95,3%	97,7%	94,8%
11062	45	20	45	75	64,7%	74,1%	95,4%	97,8%	95,2%
11063	45	20	45	80	65,1%	73,8%	95,6%	98,0%	95,5%
11064	45	20	50	20	54,1%	72,4%	92,7%	95,2%	89,9%
11065	45	20	50	25	54,5%	72,1%	92,9%	95,5%	90,5%
11066	45	20	50	30	55,0%	71,8%	93,1%	95,8%	91,1%
11067	45	20	50	35	55,4%	71,6%	93,3%	96,1%	91,6%
11068	45	20	50	40	55,8%	71,3%	93,5%	96,3%	92,1%
11069	45	20	50	45	56,3%	71,0%	93,7%	96,6%	92,6%
11070	45	20	50	50	56,7%	70,8%	93,9%	96,8%	93,1%
11071	45	20	50	55	57,1%	70,5%	94,1%	97,0%	93,5%
11072	45	20	50	60	57,6%	70,2%	94,3%	97,2%	93,9%
11073	45	20	50	65	58,0%	70,0%	94,5%	97,4%	94,3%
11074	45	20	50	70	58,4%	69,7%	94,7%	97,5%	94,6%
11075	45	20	50	75	58,8%	69,4%	94,8%	97,7%	95,0%
11076	45	20	50	80	59,3%	69,1%	95,0%	97,9%	95,3%
11077	45	20	55	20	47,9%	67,5%	91,8%	94,9%	89,5%
11078	45	20	55	25	48,4%	67,2%	92,0%	95,3%	90,1%
11079	45	20	55	30	48,8%	66,9%	92,3%	95,6%	90,7%
11080	45	20	55	35	49,2%	66,7%	92,5%	95,8%	91,3%
11081	45	20	55	40	49,7%	66,4%	92,7%	96,1%	91,8%
11082	45	20	55	45	50,1%	66,1%	93,0%	96,4%	92,3%
11083	45	20	55	50	50,6%	65,8%	93,2%	96,6%	92,8%
11084	45	20	55	55	51,0%	65,5%	93,4%	96,8%	93,2%
11085	45	20	55	60	51,4%	65,2%	93,6%	97,0%	93,6%
11086	45	20	55	65	51,9%	64,9%	93,8%	97,2%	94,0%
11087	45	20	55	70	52,3%	64,6%	94,0%	97,4%	94,4%

11088	45	20	55	75	52,8%	64,3%	94,2%	97,6%	94,8%
11089	45	20	55	80	53,2%	64,0%	94,4%	97,7%	95,1%
11090	45	20	60	20	41,8%	62,3%	90,8%	94,6%	89,1%
11091	45	20	60	25	42,2%	62,0%	91,1%	95,0%	89,7%
11092	45	20	60	30	42,7%	61,7%	91,3%	95,3%	90,4%
11093	45	20	60	35	43,1%	61,3%	91,6%	95,6%	90,9%
11094	45	20	60	40	43,5%	61,0%	91,9%	95,9%	91,5%
11095	45	20	60	45	44,0%	60,7%	92,1%	96,1%	92,0%
11096	45	20	60	50	44,4%	60,4%	92,4%	96,4%	92,5%
11097	45	20	60	55	44,8%	60,1%	92,6%	96,6%	93,0%
11098	45	20	60	60	45,3%	59,8%	92,8%	96,8%	93,4%
11099	45	20	60	65	45,7%	59,5%	93,0%	97,0%	93,8%
11100	45	20	60	70	46,2%	59,1%	93,3%	97,2%	94,2%
11101	45	20	60	75	46,6%	58,8%	93,5%	97,4%	94,6%
11102	45	20	60	80	47,0%	58,5%	93,7%	97,6%	94,9%
11103	45	20	65	20	36,0%	56,7%	89,7%	94,3%	88,7%
11104	45	20	65	25	36,4%	56,4%	90,0%	94,7%	89,4%
11105	45	20	65	30	36,8%	56,1%	90,3%	95,0%	90,0%
11106	45	20	65	35	37,2%	55,7%	90,6%	95,3%	90,6%
11107	45	20	65	40	37,6%	55,4%	90,9%	95,6%	91,2%
11108	45	20	65	45	38,0%	55,1%	91,2%	95,9%	91,7%
11109	45	20	65	50	38,4%	54,8%	91,4%	96,2%	92,2%
11110	45	20	65	55	38,8%	54,4%	91,7%	96,4%	92,7%
11111	45	20	65	60	39,3%	54,1%	92,0%	96,6%	93,1%
11112	45	20	65	65	39,7%	53,8%	92,2%	96,9%	93,6%
11113	45	20	65	70	40,1%	53,5%	92,4%	97,1%	94,0%
11114	45	20	65	75	40,5%	53,1%	92,7%	97,3%	94,3%
11115	45	20	65	80	41,0%	52,8%	92,9%	97,4%	94,7%
11116	45	20	70	20	30,5%	51,0%	88,5%	94,0%	88,3%
11117	45	20	70	25	30,9%	50,6%	88,8%	94,4%	89,0%
11118	45	20	70	30	31,2%	50,3%	89,2%	94,7%	89,6%
11119	45	20	70	35	31,6%	50,0%	89,5%	95,1%	90,2%
11120	45	20	70	40	32,0%	49,7%	89,8%	95,4%	90,8%

11121	45	20	70	45	32,4%	49,3%	90,1%	95,7%	91,4%
11122	45	20	70	50	32,8%	49,0%	90,4%	95,9%	91,9%
11123	45	20	70	55	33,2%	48,7%	90,7%	96,2%	92,4%
11124	45	20	70	60	33,6%	48,4%	91,0%	96,4%	92,9%
11125	45	20	70	65	34,0%	48,0%	91,3%	96,7%	93,3%
11126	45	20	70	70	34,3%	47,7%	91,5%	96,9%	93,7%
11127	45	20	70	75	34,7%	47,4%	91,8%	97,1%	94,1%
11128	45	20	70	80	35,1%	47,1%	92,0%	97,3%	94,5%
11129	45	20	75	20	25,5%	45,2%	87,2%	93,6%	87,8%
11130	45	20	75	25	25,9%	44,9%	87,5%	94,0%	88,6%
11131	45	20	75	30	26,2%	44,6%	87,9%	94,4%	89,2%
11132	45	20	75	35	26,5%	44,2%	88,3%	94,8%	89,9%
11133	45	20	75	40	26,9%	43,9%	88,6%	95,1%	90,5%
11134	45	20	75	45	27,2%	43,6%	88,9%	95,4%	91,1%
11135	45	20	75	50	27,6%	43,3%	89,3%	95,7%	91,6%
11136	45	20	75	55	27,9%	43,0%	89,6%	96,0%	92,1%
11137	45	20	75	60	28,3%	42,6%	89,9%	96,2%	92,6%
11138	45	20	75	65	28,7%	42,3%	90,2%	96,5%	93,0%
11139	45	20	75	70	29,0%	42,0%	90,5%	96,7%	93,5%
11140	45	20	75	75	29,4%	41,7%	90,8%	96,9%	93,9%
11141	45	20	75	80	29,7%	41,4%	91,1%	97,1%	94,3%
11142	45	20	80	20	21,1%	39,6%	85,7%	93,3%	87,4%
11143	45	20	80	25	21,4%	39,3%	86,1%	93,7%	88,1%
11144	45	20	80	30	21,7%	39,0%	86,5%	94,1%	88,8%
11145	45	20	80	35	22,0%	38,6%	86,9%	94,4%	89,5%
11146	45	20	80	40	22,3%	38,3%	87,3%	94,8%	90,1%
11147	45	20	80	45	22,6%	38,0%	87,7%	95,1%	90,7%
11148	45	20	80	50	22,9%	37,7%	88,0%	95,4%	91,3%
11149	45	20	80	55	23,2%	37,4%	88,4%	95,7%	91,8%
11150	45	20	80	60	23,6%	37,1%	88,7%	96,0%	92,3%
11151	45	20	80	65	23,9%	36,8%	89,1%	96,3%	92,8%
11152	45	20	80	70	24,2%	36,5%	89,4%	96,5%	93,2%
11153	45	20	80	75	24,5%	36,2%	89,7%	96,7%	93,6%

11154	45	20	80	80	24,9%	35,9%	90,0%	96,9%	94,0%
11155	45	25	20	20	85,0%	91,9%	96,3%	96,4%	91,2%
11156	45	25	20	25	85,2%	91,8%	96,4%	96,6%	91,7%
11157	45	25	20	30	85,4%	91,7%	96,5%	96,8%	92,2%
11158	45	25	20	35	85,7%	91,6%	96,6%	97,0%	92,7%
11159	45	25	20	40	85,9%	91,5%	96,7%	97,2%	93,1%
11160	45	25	20	45	86,1%	91,4%	96,8%	97,4%	93,6%
11161	45	25	20	50	86,3%	91,3%	96,9%	97,6%	94,0%
11162	45	25	20	55	86,5%	91,2%	97,0%	97,7%	94,3%
11163	45	25	20	60	86,7%	91,1%	97,1%	97,9%	94,7%
11164	45	25	20	65	86,9%	91,0%	97,2%	98,0%	95,0%
11165	45	25	20	70	87,1%	90,9%	97,3%	98,2%	95,3%
11166	45	25	20	75	87,3%	90,7%	97,4%	98,3%	95,6%
11167	45	25	20	80	87,5%	90,6%	97,5%	98,4%	95,9%
11168	45	25	25	20	81,6%	90,0%	95,8%	96,2%	90,8%
11169	45	25	25	25	81,8%	89,9%	95,9%	96,4%	91,4%
11170	45	25	25	30	82,1%	89,8%	96,1%	96,6%	91,9%
11171	45	25	25	35	82,3%	89,6%	96,2%	96,9%	92,4%
11172	45	25	25	40	82,6%	89,5%	96,3%	97,1%	92,9%
11173	45	25	25	45	82,8%	89,4%	96,4%	97,3%	93,3%
11174	45	25	25	50	83,1%	89,3%	96,5%	97,4%	93,7%
11175	45	25	25	55	83,3%	89,1%	96,7%	97,6%	94,1%
11176	45	25	25	60	83,6%	89,0%	96,8%	97,8%	94,5%
11177	45	25	25	65	83,8%	88,9%	96,9%	97,9%	94,8%
11178	45	25	25	70	84,1%	88,7%	97,0%	98,0%	95,2%
11179	45	25	25	75	84,3%	88,6%	97,1%	98,2%	95,5%
11180	45	25	25	80	84,5%	88,5%	97,2%	98,3%	95,8%
11181	45	25	30	20	77,6%	87,7%	95,3%	95,9%	90,5%
11182	45	25	30	25	77,9%	87,6%	95,4%	96,2%	91,1%
11183	45	25	30	30	78,2%	87,4%	95,6%	96,4%	91,6%
11184	45	25	30	35	78,5%	87,3%	95,7%	96,7%	92,1%
11185	45	25	30	40	78,8%	87,1%	95,8%	96,9%	92,6%
11186	45	25	30	45	79,1%	87,0%	96,0%	97,1%	93,1%

11187	45	25	30	50	79,3%	86,8%	96,1%	97,3%	93,5%
11188	45	25	30	55	79,6%	86,7%	96,2%	97,5%	93,9%
11189	45	25	30	60	79,9%	86,5%	96,4%	97,6%	94,3%
11190	45	25	30	65	80,2%	86,4%	96,5%	97,8%	94,6%
11191	45	25	30	70	80,5%	86,2%	96,6%	97,9%	95,0%
11192	45	25	30	75	80,7%	86,1%	96,7%	98,1%	95,3%
11193	45	25	30	80	81,0%	85,9%	96,8%	98,2%	95,6%
11194	45	25	35	20	73,0%	85,0%	94,7%	95,7%	90,1%
11195	45	25	35	25	73,3%	84,8%	94,8%	96,0%	90,7%
11196	45	25	35	30	73,7%	84,7%	95,0%	96,2%	91,3%
11197	45	25	35	35	74,0%	84,5%	95,2%	96,5%	91,8%
11198	45	25	35	40	74,3%	84,3%	95,3%	96,7%	92,3%
11199	45	25	35	45	74,7%	84,1%	95,5%	96,9%	92,8%
11200	45	25	35	50	75,0%	84,0%	95,6%	97,1%	93,2%
11201	45	25	35	55	75,3%	83,8%	95,8%	97,3%	93,7%
11202	45	25	35	60	75,7%	83,6%	95,9%	97,5%	94,0%
11203	45	25	35	65	76,0%	83,4%	96,0%	97,6%	94,4%
11204	45	25	35	70	76,3%	83,3%	96,1%	97,8%	94,8%
11205	45	25	35	75	76,6%	83,1%	96,3%	97,9%	95,1%
11206	45	25	35	80	76,9%	82,9%	96,4%	98,1%	95,4%
11207	45	25	40	20	67,8%	81,8%	94,0%	95,4%	89,8%
11208	45	25	40	25	68,2%	81,6%	94,2%	95,7%	90,4%
11209	45	25	40	30	68,6%	81,4%	94,4%	96,0%	90,9%
11210	45	25	40	35	69,0%	81,2%	94,6%	96,3%	91,5%
11211	45	25	40	40	69,4%	81,0%	94,7%	96,5%	92,0%
11212	45	25	40	45	69,7%	80,8%	94,9%	96,7%	92,5%
11213	45	25	40	50	70,1%	80,6%	95,1%	96,9%	93,0%
11214	45	25	40	55	70,5%	80,4%	95,2%	97,1%	93,4%
11215	45	25	40	60	70,8%	80,2%	95,4%	97,3%	93,8%
11216	45	25	40	65	71,2%	80,0%	95,5%	97,5%	94,2%
11217	45	25	40	70	71,6%	79,8%	95,7%	97,7%	94,6%
11218	45	25	40	75	71,9%	79,6%	95,8%	97,8%	94,9%
11219	45	25	40	80	72,3%	79,4%	95,9%	98,0%	95,2%

11220	45	25	45	20	62,2%	78,1%	93,3%	95,2%	89,4%
11221	45	25	45	25	62,6%	77,9%	93,5%	95,5%	90,0%
11222	45	25	45	30	63,1%	77,7%	93,7%	95,8%	90,6%
11223	45	25	45	35	63,5%	77,4%	93,9%	96,0%	91,2%
11224	45	25	45	40	63,9%	77,2%	94,1%	96,3%	91,7%
11225	45	25	45	45	64,3%	77,0%	94,3%	96,5%	92,2%
11226	45	25	45	50	64,7%	76,7%	94,4%	96,8%	92,7%
11227	45	25	45	55	65,1%	76,5%	94,6%	97,0%	93,1%
11228	45	25	45	60	65,5%	76,3%	94,8%	97,2%	93,6%
11229	45	25	45	65	65,9%	76,0%	95,0%	97,3%	94,0%
11230	45	25	45	70	66,3%	75,8%	95,1%	97,5%	94,3%
11231	45	25	45	75	66,7%	75,6%	95,3%	97,7%	94,7%
11232	45	25	45	80	67,1%	75,3%	95,4%	97,8%	95,0%
11233	45	25	50	20	56,3%	73,9%	92,4%	94,9%	89,0%
11234	45	25	50	25	56,7%	73,7%	92,7%	95,2%	89,6%
11235	45	25	50	30	57,1%	73,4%	92,9%	95,5%	90,2%
11236	45	25	50	35	57,6%	73,2%	93,1%	95,8%	90,8%
11237	45	25	50	40	58,0%	72,9%	93,3%	96,1%	91,4%
11238	45	25	50	45	58,4%	72,6%	93,6%	96,3%	91,9%
11239	45	25	50	50	58,9%	72,4%	93,8%	96,6%	92,4%
11240	45	25	50	55	59,3%	72,1%	94,0%	96,8%	92,9%
11241	45	25	50	60	59,7%	71,8%	94,1%	97,0%	93,3%
11242	45	25	50	65	60,1%	71,6%	94,3%	97,2%	93,7%
11243	45	25	50	70	60,6%	71,3%	94,5%	97,4%	94,1%
11244	45	25	50	75	61,0%	71,0%	94,7%	97,5%	94,5%
11245	45	25	50	80	61,4%	70,8%	94,8%	97,7%	94,8%
11246	45	25	55	20	50,1%	69,2%	91,5%	94,6%	88,6%
11247	45	25	55	25	50,6%	68,9%	91,8%	94,9%	89,2%
11248	45	25	55	30	51,0%	68,7%	92,0%	95,2%	89,9%
11249	45	25	55	35	51,5%	68,4%	92,3%	95,5%	90,5%
11250	45	25	55	40	51,9%	68,1%	92,5%	95,8%	91,1%
11251	45	25	55	45	52,3%	67,8%	92,8%	96,1%	91,6%
11252	45	25	55	50	52,8%	67,5%	93,0%	96,3%	92,1%

11253	45	25	55	55	53,2%	67,2%	93,2%	96,6%	92,6%
11254	45	25	55	60	53,7%	67,0%	93,4%	96,8%	93,1%
11255	45	25	55	65	54,1%	66,7%	93,6%	97,0%	93,5%
11256	45	25	55	70	54,5%	66,4%	93,8%	97,2%	93,9%
11257	45	25	55	75	55,0%	66,1%	94,0%	97,4%	94,3%
11258	45	25	55	80	55,4%	65,8%	94,2%	97,6%	94,6%
11259	45	25	60	20	44,0%	64,1%	90,5%	94,3%	88,1%
11260	45	25	60	25	44,4%	63,8%	90,8%	94,6%	88,8%
11261	45	25	60	30	44,9%	63,5%	91,1%	95,0%	89,5%
11262	45	25	60	35	45,3%	63,2%	91,4%	95,3%	90,1%
11263	45	25	60	40	45,7%	62,9%	91,6%	95,6%	90,7%
11264	45	25	60	45	46,2%	62,6%	91,9%	95,9%	91,3%
11265	45	25	60	50	46,6%	62,3%	92,1%	96,1%	91,8%
11266	45	25	60	55	47,1%	62,0%	92,4%	96,4%	92,3%
11267	45	25	60	60	47,5%	61,7%	92,6%	96,6%	92,8%
11268	45	25	60	65	47,9%	61,3%	92,8%	96,8%	93,2%
11269	45	25	60	70	48,4%	61,0%	93,1%	97,0%	93,6%
11270	45	25	60	75	48,8%	60,7%	93,3%	97,2%	94,0%
11271	45	25	60	80	49,3%	60,4%	93,5%	97,4%	94,4%
11272	45	25	65	20	38,0%	58,6%	89,4%	93,9%	87,7%
11273	45	25	65	25	38,4%	58,3%	89,7%	94,3%	88,4%
11274	45	25	65	30	38,9%	58,0%	90,0%	94,7%	89,1%
11275	45	25	65	35	39,3%	57,7%	90,3%	95,0%	89,8%
11276	45	25	65	40	39,7%	57,4%	90,6%	95,3%	90,4%
11277	45	25	65	45	40,1%	57,0%	90,9%	95,6%	90,9%
11278	45	25	65	50	40,5%	56,7%	91,2%	95,9%	91,5%
11279	45	25	65	55	41,0%	56,4%	91,5%	96,2%	92,0%
11280	45	25	65	60	41,4%	56,1%	91,7%	96,4%	92,5%
11281	45	25	65	65	41,8%	55,7%	92,0%	96,6%	93,0%
11282	45	25	65	70	42,3%	55,4%	92,2%	96,8%	93,4%
11283	45	25	65	75	42,7%	55,1%	92,5%	97,1%	93,8%
11284	45	25	65	80	43,1%	54,8%	92,7%	97,2%	94,2%
11285	45	25	70	20	32,4%	52,9%	88,2%	93,6%	87,2%

11286	45	25	70	25	32,8%	52,6%	88,5%	94,0%	88,0%
11287	45	25	70	30	33,2%	52,3%	88,9%	94,3%	88,7%
11288	45	25	70	35	33,6%	52,0%	89,2%	94,7%	89,4%
11289	45	25	70	40	34,0%	51,6%	89,5%	95,0%	90,0%
11290	45	25	70	45	34,4%	51,3%	89,8%	95,3%	90,6%
11291	45	25	70	50	34,8%	51,0%	90,1%	95,6%	91,2%
11292	45	25	70	55	35,2%	50,7%	90,4%	95,9%	91,7%
11293	45	25	70	60	35,6%	50,3%	90,7%	96,2%	92,2%
11294	45	25	70	65	36,0%	50,0%	91,0%	96,4%	92,7%
11295	45	25	70	70	36,4%	49,7%	91,3%	96,7%	93,1%
11296	45	25	70	75	36,8%	49,3%	91,5%	96,9%	93,6%
11297	45	25	70	80	37,2%	49,0%	91,8%	97,1%	94,0%
11298	45	25	75	20	27,2%	47,2%	86,8%	93,2%	86,8%
11299	45	25	75	25	27,6%	46,8%	87,2%	93,6%	87,5%
11300	45	25	75	30	28,0%	46,5%	87,6%	94,0%	88,3%
11301	45	25	75	35	28,3%	46,2%	87,9%	94,4%	89,0%
11302	45	25	75	40	28,7%	45,9%	88,3%	94,7%	89,6%
11303	45	25	75	45	29,0%	45,5%	88,6%	95,1%	90,2%
11304	45	25	75	50	29,4%	45,2%	89,0%	95,4%	90,8%
11305	45	25	75	55	29,8%	44,9%	89,3%	95,7%	91,4%
11306	45	25	75	60	30,1%	44,6%	89,6%	96,0%	91,9%
11307	45	25	75	65	30,5%	44,3%	89,9%	96,2%	92,4%
11308	45	25	75	70	30,9%	43,9%	90,2%	96,5%	92,9%
11309	45	25	75	75	31,3%	43,6%	90,5%	96,7%	93,3%
11310	45	25	75	80	31,6%	43,3%	90,8%	96,9%	93,7%
11311	45	25	80	20	22,6%	41,5%	85,3%	92,8%	86,3%
11312	45	25	80	25	22,9%	41,2%	85,7%	93,2%	87,1%
11313	45	25	80	30	23,3%	40,8%	86,1%	93,6%	87,8%
11314	45	25	80	35	23,6%	40,5%	86,5%	94,0%	88,6%
11315	45	25	80	40	23,9%	40,2%	86,9%	94,4%	89,2%
11316	45	25	80	45	24,2%	39,9%	87,3%	94,8%	89,9%
11317	45	25	80	50	24,5%	39,6%	87,7%	95,1%	90,5%
11318	45	25	80	55	24,9%	39,3%	88,1%	95,4%	91,1%

11319	45	25	80	60	25,2%	39,0%	88,4%	95,7%	91,6%
11320	45	25	80	65	25,5%	38,7%	88,7%	96,0%	92,1%
11321	45	25	80	70	25,9%	38,3%	89,1%	96,2%	92,6%
11322	45	25	80	75	26,2%	38,0%	89,4%	96,5%	93,0%
11323	45	25	80	80	26,6%	37,7%	89,7%	96,7%	93,5%
11324	45	30	20	20	86,1%	92,5%	96,2%	96,1%	90,4%
11325	45	30	20	25	86,3%	92,4%	96,3%	96,4%	91,0%
11326	45	30	20	30	86,5%	92,3%	96,4%	96,6%	91,5%
11327	45	30	20	35	86,7%	92,2%	96,5%	96,8%	92,0%
11328	45	30	20	40	86,9%	92,1%	96,6%	97,0%	92,5%
11329	45	30	20	45	87,1%	92,0%	96,7%	97,2%	93,0%
11330	45	30	20	50	87,3%	91,9%	96,8%	97,4%	93,4%
11331	45	30	20	55	87,5%	91,8%	96,9%	97,6%	93,8%
11332	45	30	20	60	87,7%	91,7%	97,0%	97,7%	94,2%
11333	45	30	20	65	87,9%	91,6%	97,1%	97,9%	94,6%
11334	45	30	20	70	88,1%	91,5%	97,2%	98,0%	94,9%
11335	45	30	20	75	88,3%	91,4%	97,3%	98,1%	95,2%
11336	45	30	20	80	88,4%	91,3%	97,4%	98,3%	95,5%
11337	45	30	25	20	82,9%	90,7%	95,7%	95,9%	90,0%
11338	45	30	25	25	83,1%	90,6%	95,8%	96,2%	90,6%
11339	45	30	25	30	83,4%	90,5%	95,9%	96,4%	91,2%
11340	45	30	25	35	83,6%	90,3%	96,1%	96,6%	91,7%
11341	45	30	25	40	83,8%	90,2%	96,2%	96,8%	92,2%
11342	45	30	25	45	84,1%	90,1%	96,3%	97,1%	92,7%
11343	45	30	25	50	84,3%	90,0%	96,4%	97,2%	93,1%
11344	45	30	25	55	84,5%	89,9%	96,6%	97,4%	93,6%
11345	45	30	25	60	84,8%	89,8%	96,7%	97,6%	94,0%
11346	45	30	25	65	85,0%	89,6%	96,8%	97,7%	94,3%
11347	45	30	25	70	85,2%	89,5%	96,9%	97,9%	94,7%
11348	45	30	25	75	85,4%	89,4%	97,0%	98,0%	95,0%
11349	45	30	25	80	85,7%	89,3%	97,1%	98,2%	95,4%
11350	45	30	30	20	79,1%	88,5%	95,1%	95,6%	89,6%
11351	45	30	30	25	79,4%	88,4%	95,3%	95,9%	90,3%

11352	45	30	30	30	79,6%	88,3%	95,4%	96,2%	90,8%
11353	45	30	30	35	79,9%	88,1%	95,6%	96,4%	91,4%
11354	45	30	30	40	80,2%	88,0%	95,7%	96,7%	91,9%
11355	45	30	30	45	80,5%	87,9%	95,9%	96,9%	92,4%
11356	45	30	30	50	80,8%	87,7%	96,0%	97,1%	92,9%
11357	45	30	30	55	81,0%	87,6%	96,1%	97,3%	93,3%
11358	45	30	30	60	81,3%	87,4%	96,2%	97,4%	93,7%
11359	45	30	30	65	81,6%	87,3%	96,4%	97,6%	94,1%
11360	45	30	30	70	81,8%	87,1%	96,5%	97,8%	94,5%
11361	45	30	30	75	82,1%	87,0%	96,6%	97,9%	94,8%
11362	45	30	30	80	82,3%	86,8%	96,7%	98,0%	95,2%
11363	45	30	35	20	74,7%	86,0%	94,5%	95,4%	89,2%
11364	45	30	35	25	75,0%	85,8%	94,7%	95,7%	89,9%
11365	45	30	35	30	75,3%	85,7%	94,9%	96,0%	90,5%
11366	45	30	35	35	75,7%	85,5%	95,0%	96,2%	91,1%
11367	45	30	35	40	76,0%	85,3%	95,2%	96,5%	91,6%
11368	45	30	35	45	76,3%	85,2%	95,3%	96,7%	92,1%
11369	45	30	35	50	76,6%	85,0%	95,5%	96,9%	92,6%
11370	45	30	35	55	76,9%	84,8%	95,6%	97,1%	93,1%
11371	45	30	35	60	77,3%	84,7%	95,8%	97,3%	93,5%
11372	45	30	35	65	77,6%	84,5%	95,9%	97,5%	93,9%
11373	45	30	35	70	77,9%	84,3%	96,0%	97,6%	94,3%
11374	45	30	35	75	78,2%	84,1%	96,2%	97,8%	94,6%
11375	45	30	35	80	78,5%	84,0%	96,3%	97,9%	95,0%
11376	45	30	40	20	69,7%	83,0%	93,8%	95,1%	88,8%
11377	45	30	40	25	70,1%	82,8%	94,0%	95,4%	89,5%
11378	45	30	40	30	70,5%	82,6%	94,2%	95,7%	90,1%
11379	45	30	40	35	70,8%	82,4%	94,4%	96,0%	90,7%
11380	45	30	40	40	71,2%	82,2%	94,6%	96,2%	91,3%
11381	45	30	40	45	71,6%	82,0%	94,7%	96,5%	91,8%
11382	45	30	40	50	71,9%	81,8%	94,9%	96,7%	92,3%
11383	45	30	40	55	72,3%	81,6%	95,1%	96,9%	92,8%
11384	45	30	40	60	72,6%	81,4%	95,2%	97,1%	93,2%

11385	45	30	40	65	73,0%	81,2%	95,4%	97,3%	93,7%
11386	45	30	40	70	73,3%	81,0%	95,5%	97,5%	94,0%
11387	45	30	40	75	73,7%	80,8%	95,7%	97,6%	94,4%
11388	45	30	40	80	74,0%	80,6%	95,8%	97,8%	94,8%
11389	45	30	45	20	64,3%	79,4%	93,1%	94,8%	88,4%
11390	45	30	45	25	64,7%	79,2%	93,3%	95,1%	89,1%
11391	45	30	45	30	65,1%	79,0%	93,5%	95,5%	89,8%
11392	45	30	45	35	65,5%	78,8%	93,7%	95,7%	90,4%
11393	45	30	45	40	65,9%	78,6%	93,9%	96,0%	91,0%
11394	45	30	45	45	66,3%	78,3%	94,1%	96,3%	91,5%
11395	45	30	45	50	66,7%	78,1%	94,3%	96,5%	92,0%
11396	45	30	45	55	67,1%	77,9%	94,5%	96,7%	92,5%
11397	45	30	45	60	67,5%	77,7%	94,6%	96,9%	93,0%
11398	45	30	45	65	67,8%	77,4%	94,8%	97,1%	93,4%
11399	45	30	45	70	68,2%	77,2%	95,0%	97,3%	93,8%
11400	45	30	45	75	68,6%	77,0%	95,1%	97,5%	94,2%
11401	45	30	45	80	69,0%	76,8%	95,3%	97,7%	94,6%
11402	45	30	50	20	58,4%	75,4%	92,2%	94,5%	88,0%
11403	45	30	50	25	58,9%	75,2%	92,5%	94,9%	88,7%
11404	45	30	50	30	59,3%	74,9%	92,7%	95,2%	89,4%
11405	45	30	50	35	59,7%	74,7%	92,9%	95,5%	90,0%
11406	45	30	50	40	60,1%	74,4%	93,1%	95,8%	90,6%
11407	45	30	50	45	60,6%	74,2%	93,4%	96,0%	91,2%
11408	45	30	50	50	61,0%	73,9%	93,6%	96,3%	91,7%
11409	45	30	50	55	61,4%	73,7%	93,8%	96,5%	92,2%
11410	45	30	50	60	61,8%	73,4%	94,0%	96,8%	92,7%
11411	45	30	50	65	62,2%	73,2%	94,2%	97,0%	93,1%
11412	45	30	50	70	62,7%	72,9%	94,3%	97,2%	93,6%
11413	45	30	50	75	63,1%	72,6%	94,5%	97,3%	94,0%
11414	45	30	50	80	63,5%	72,4%	94,7%	97,5%	94,3%
11415	45	30	55	20	52,4%	70,9%	91,3%	94,2%	87,6%
11416	45	30	55	25	52,8%	70,6%	91,6%	94,5%	88,3%
11417	45	30	55	30	53,2%	70,3%	91,8%	94,9%	89,0%

11418	45	30	55	35	53,7%	70,1%	92,1%	95,2%	89,6%
11419	45	30	55	40	54,1%	69,8%	92,3%	95,5%	90,2%
11420	45	30	55	45	54,5%	69,5%	92,5%	95,8%	90,8%
11421	45	30	55	50	55,0%	69,2%	92,8%	96,1%	91,4%
11422	45	30	55	55	55,4%	69,0%	93,0%	96,3%	91,9%
11423	45	30	55	60	55,9%	68,7%	93,2%	96,6%	92,4%
11424	45	30	55	65	56,3%	68,4%	93,4%	96,8%	92,9%
11425	45	30	55	70	56,7%	68,1%	93,6%	97,0%	93,3%
11426	45	30	55	75	57,2%	67,8%	93,8%	97,2%	93,7%
11427	45	30	55	80	57,6%	67,5%	94,0%	97,4%	94,1%
11428	45	30	60	20	46,2%	65,9%	90,2%	93,8%	87,1%
11429	45	30	60	25	46,6%	65,6%	90,5%	94,2%	87,9%
11430	45	30	60	30	47,1%	65,3%	90,8%	94,6%	88,6%
11431	45	30	60	35	47,5%	65,0%	91,1%	94,9%	89,2%
11432	45	30	60	40	47,9%	64,7%	91,4%	95,3%	89,9%
11433	45	30	60	45	48,4%	64,4%	91,6%	95,6%	90,5%
11434	45	30	60	50	48,8%	64,1%	91,9%	95,8%	91,1%
11435	45	30	60	55	49,3%	63,8%	92,1%	96,1%	91,6%
11436	45	30	60	60	49,7%	63,5%	92,4%	96,4%	92,1%
11437	45	30	60	65	50,1%	63,2%	92,6%	96,6%	92,6%
11438	45	30	60	70	50,6%	62,9%	92,9%	96,8%	93,1%
11439	45	30	60	75	51,0%	62,6%	93,1%	97,0%	93,5%
11440	45	30	60	80	51,5%	62,3%	93,3%	97,2%	93,9%
11441	45	30	65	20	40,1%	60,5%	89,1%	93,5%	86,6%
11442	45	30	65	25	40,6%	60,2%	89,4%	93,9%	87,4%
11443	45	30	65	30	41,0%	59,9%	89,7%	94,3%	88,1%
11444	45	30	65	35	41,4%	59,6%	90,0%	94,6%	88,8%
11445	45	30	65	40	41,8%	59,3%	90,3%	95,0%	89,5%
11446	45	30	65	45	42,3%	59,0%	90,6%	95,3%	90,1%
11447	45	30	65	50	42,7%	58,6%	90,9%	95,6%	90,7%
11448	45	30	65	55	43,1%	58,3%	91,2%	95,9%	91,3%
11449	45	30	65	60	43,6%	58,0%	91,5%	96,1%	91,8%
11450	45	30	65	65	44,0%	57,7%	91,7%	96,4%	92,3%

11451	45	30	65	70	44,4%	57,4%	92,0%	96,6%	92,8%
11452	45	30	65	75	44,9%	57,0%	92,2%	96,8%	93,2%
11453	45	30	65	80	45,3%	56,7%	92,5%	97,0%	93,6%
11454	45	30	70	20	34,4%	54,9%	87,8%	93,1%	86,1%
11455	45	30	70	25	34,8%	54,6%	88,2%	93,5%	86,9%
11456	45	30	70	30	35,2%	54,2%	88,5%	93,9%	87,7%
11457	45	30	70	35	35,6%	53,9%	88,9%	94,3%	88,4%
11458	45	30	70	40	36,0%	53,6%	89,2%	94,7%	89,1%
11459	45	30	70	45	36,4%	53,3%	89,5%	95,0%	89,8%
11460	45	30	70	50	36,8%	52,9%	89,8%	95,3%	90,4%
11461	45	30	70	55	37,2%	52,6%	90,2%	95,6%	90,9%
11462	45	30	70	60	37,6%	52,3%	90,4%	95,9%	91,5%
11463	45	30	70	65	38,0%	52,0%	90,7%	96,2%	92,0%
11464	45	30	70	70	38,5%	51,6%	91,0%	96,4%	92,5%
11465	45	30	70	75	38,9%	51,3%	91,3%	96,6%	93,0%
11466	45	30	70	80	39,3%	51,0%	91,6%	96,9%	93,4%
11467	45	30	75	20	29,0%	49,1%	86,4%	92,7%	85,6%
11468	45	30	75	25	29,4%	48,8%	86,8%	93,1%	86,5%
11469	45	30	75	30	29,8%	48,5%	87,2%	93,6%	87,3%
11470	45	30	75	35	30,1%	48,2%	87,6%	94,0%	88,0%
11471	45	30	75	40	30,5%	47,8%	88,0%	94,4%	88,7%
11472	45	30	75	45	30,9%	47,5%	88,3%	94,7%	89,4%
11473	45	30	75	50	31,3%	47,2%	88,7%	95,0%	90,0%
11474	45	30	75	55	31,6%	46,9%	89,0%	95,4%	90,6%
11475	45	30	75	60	32,0%	46,5%	89,3%	95,7%	91,2%
11476	45	30	75	65	32,4%	46,2%	89,6%	95,9%	91,7%
11477	45	30	75	70	32,8%	45,9%	89,9%	96,2%	92,2%
11478	45	30	75	75	33,2%	45,6%	90,3%	96,4%	92,7%
11479	45	30	75	80	33,6%	45,2%	90,5%	96,7%	93,1%
11480	45	30	80	20	24,2%	43,4%	84,9%	92,3%	85,1%
11481	45	30	80	25	24,6%	43,1%	85,3%	92,7%	86,0%
11482	45	30	80	30	24,9%	42,8%	85,8%	93,2%	86,8%
11483	45	30	80	35	25,2%	42,4%	86,2%	93,6%	87,6%

11484	45	30	80	40	25,5%	42,1%	86,6%	94,0%	88,3%
11485	45	30	80	45	25,9%	41,8%	87,0%	94,4%	89,0%
11486	45	30	80	50	26,2%	41,5%	87,3%	94,7%	89,6%
11487	45	30	80	55	26,6%	41,2%	87,7%	95,1%	90,2%
11488	45	30	80	60	26,9%	40,9%	88,1%	95,4%	90,8%
11489	45	30	80	65	27,3%	40,5%	88,4%	95,7%	91,4%
11490	45	30	80	70	27,6%	40,2%	88,8%	96,0%	91,9%
11491	45	30	80	75	28,0%	39,9%	89,1%	96,2%	92,4%
11492	45	30	80	80	28,3%	39,6%	89,4%	96,5%	92,9%
11493	45	35	20	20	87,1%	93,0%	96,0%	95,8%	89,5%
11494	45	35	20	25	87,3%	92,9%	96,2%	96,1%	90,1%
11495	45	35	20	30	87,5%	92,8%	96,3%	96,4%	90,7%
11496	45	35	20	35	87,7%	92,7%	96,4%	96,6%	91,3%
11497	45	35	20	40	87,9%	92,6%	96,5%	96,8%	91,8%
11498	45	35	20	45	88,1%	92,5%	96,6%	97,0%	92,3%
11499	45	35	20	50	88,3%	92,5%	96,7%	97,2%	92,8%
11500	45	35	20	55	88,4%	92,4%	96,8%	97,4%	93,2%
11501	45	35	20	60	88,6%	92,3%	96,9%	97,6%	93,7%
11502	45	35	20	65	88,8%	92,2%	97,0%	97,7%	94,1%
11503	45	35	20	70	89,0%	92,1%	97,1%	97,9%	94,4%
11504	45	35	20	75	89,1%	92,0%	97,2%	98,0%	94,8%
11505	45	35	20	80	89,3%	91,9%	97,3%	98,1%	95,1%
11506	45	35	25	20	84,1%	91,3%	95,5%	95,6%	89,1%
11507	45	35	25	25	84,3%	91,2%	95,7%	95,9%	89,8%
11508	45	35	25	30	84,5%	91,1%	95,8%	96,1%	90,4%
11509	45	35	25	35	84,8%	91,0%	95,9%	96,4%	91,0%
11510	45	35	25	40	85,0%	90,9%	96,1%	96,6%	91,5%
11511	45	35	25	45	85,2%	90,8%	96,2%	96,8%	92,0%
11512	45	35	25	50	85,4%	90,7%	96,3%	97,0%	92,5%
11513	45	35	25	55	85,7%	90,6%	96,4%	97,2%	93,0%
11514	45	35	25	60	85,9%	90,5%	96,6%	97,4%	93,4%
11515	45	35	25	65	86,1%	90,3%	96,7%	97,6%	93,8%
11516	45	35	25	70	86,3%	90,2%	96,8%	97,7%	94,2%

11517	45	35	25	75	86,5%	90,1%	96,9%	97,9%	94,6%
11518	45	35	25	80	86,7%	90,0%	97,0%	98,0%	94,9%
11519	45	35	30	20	80,5%	89,3%	95,0%	95,3%	88,7%
11520	45	35	30	25	80,8%	89,2%	95,1%	95,6%	89,4%
11521	45	35	30	30	81,0%	89,1%	95,3%	95,9%	90,0%
11522	45	35	30	35	81,3%	88,9%	95,4%	96,2%	90,6%
11523	45	35	30	40	81,6%	88,8%	95,6%	96,4%	91,2%
11524	45	35	30	45	81,8%	88,7%	95,7%	96,6%	91,7%
11525	45	35	30	50	82,1%	88,5%	95,9%	96,9%	92,2%
11526	45	35	30	55	82,4%	88,4%	96,0%	97,1%	92,7%
11527	45	35	30	60	82,6%	88,3%	96,1%	97,3%	93,2%
11528	45	35	30	65	82,9%	88,1%	96,2%	97,4%	93,6%
11529	45	35	30	70	83,1%	88,0%	96,4%	97,6%	94,0%
11530	45	35	30	75	83,4%	87,9%	96,5%	97,8%	94,4%
11531	45	35	30	80	83,6%	87,7%	96,6%	97,9%	94,7%
11532	45	35	35	20	76,3%	86,9%	94,3%	95,0%	88,3%
11533	45	35	35	25	76,6%	86,7%	94,5%	95,4%	89,0%
11534	45	35	35	30	77,0%	86,6%	94,7%	95,7%	89,6%
11535	45	35	35	35	77,3%	86,4%	94,9%	95,9%	90,3%
11536	45	35	35	40	77,6%	86,3%	95,0%	96,2%	90,8%
11537	45	35	35	45	77,9%	86,1%	95,2%	96,4%	91,4%
11538	45	35	35	50	78,2%	86,0%	95,3%	96,7%	91,9%
11539	45	35	35	55	78,5%	85,8%	95,5%	96,9%	92,4%
11540	45	35	35	60	78,8%	85,7%	95,6%	97,1%	92,9%
11541	45	35	35	65	79,1%	85,5%	95,8%	97,3%	93,3%
11542	45	35	35	70	79,4%	85,3%	95,9%	97,4%	93,7%
11543	45	35	35	75	79,6%	85,2%	96,0%	97,6%	94,1%
11544	45	35	35	80	79,9%	85,0%	96,2%	97,8%	94,5%
11545	45	35	40	20	71,6%	84,0%	93,6%	94,7%	87,9%
11546	45	35	40	25	71,9%	83,9%	93,8%	95,1%	88,6%
11547	45	35	40	30	72,3%	83,7%	94,0%	95,4%	89,2%
11548	45	35	40	35	72,6%	83,5%	94,2%	95,7%	89,9%
11549	45	35	40	40	73,0%	83,3%	94,4%	96,0%	90,5%

11550	45	35	40	45	73,3%	83,1%	94,6%	96,2%	91,1%
11551	45	35	40	50	73,7%	83,0%	94,8%	96,5%	91,6%
11552	45	35	40	55	74,0%	82,8%	94,9%	96,7%	92,1%
11553	45	35	40	60	74,4%	82,6%	95,1%	96,9%	92,6%
11554	45	35	40	65	74,7%	82,4%	95,2%	97,1%	93,1%
11555	45	35	40	70	75,0%	82,2%	95,4%	97,3%	93,5%
11556	45	35	40	75	75,4%	82,0%	95,5%	97,5%	93,9%
11557	45	35	40	80	75,7%	81,8%	95,7%	97,6%	94,3%
11558	45	35	45	20	66,3%	80,7%	92,9%	94,4%	87,4%
11559	45	35	45	25	66,7%	80,5%	93,1%	94,8%	88,2%
11560	45	35	45	30	67,1%	80,3%	93,3%	95,1%	88,8%
11561	45	35	45	35	67,5%	80,1%	93,5%	95,4%	89,5%
11562	45	35	45	40	67,9%	79,9%	93,7%	95,7%	90,1%
11563	45	35	45	45	68,2%	79,7%	93,9%	96,0%	90,7%
11564	45	35	45	50	68,6%	79,4%	94,1%	96,3%	91,3%
11565	45	35	45	55	69,0%	79,2%	94,3%	96,5%	91,8%
11566	45	35	45	60	69,4%	79,0%	94,5%	96,7%	92,3%
11567	45	35	45	65	69,7%	78,8%	94,6%	96,9%	92,8%
11568	45	35	45	70	70,1%	78,6%	94,8%	97,1%	93,2%
11569	45	35	45	75	70,5%	78,3%	95,0%	97,3%	93,7%
11570	45	35	45	80	70,9%	78,1%	95,1%	97,5%	94,0%
11571	45	35	50	20	60,6%	76,8%	92,0%	94,1%	87,0%
11572	45	35	50	25	61,0%	76,6%	92,2%	94,5%	87,7%
11573	45	35	50	30	61,4%	76,4%	92,5%	94,8%	88,4%
11574	45	35	50	35	61,8%	76,1%	92,7%	95,2%	89,1%
11575	45	35	50	40	62,3%	75,9%	92,9%	95,5%	89,8%
11576	45	35	50	45	62,7%	75,7%	93,2%	95,8%	90,4%
11577	45	35	50	50	63,1%	75,4%	93,4%	96,0%	91,0%
11578	45	35	50	55	63,5%	75,2%	93,6%	96,3%	91,5%
11579	45	35	50	60	63,9%	74,9%	93,8%	96,5%	92,0%
11580	45	35	50	65	64,3%	74,7%	94,0%	96,7%	92,5%
11581	45	35	50	70	64,7%	74,4%	94,2%	97,0%	93,0%
11582	45	35	50	75	65,1%	74,2%	94,3%	97,2%	93,4%

11583	45	35	50	80	65,5%	73,9%	94,5%	97,3%	93,8%
11584	45	35	55	20	54,6%	72,5%	91,0%	93,8%	86,5%
11585	45	35	55	25	55,0%	72,2%	91,3%	94,2%	87,3%
11586	45	35	55	30	55,4%	71,9%	91,6%	94,5%	88,0%
11587	45	35	55	35	55,9%	71,7%	91,8%	94,9%	88,7%
11588	45	35	55	40	56,3%	71,4%	92,1%	95,2%	89,4%
11589	45	35	55	45	56,7%	71,2%	92,3%	95,5%	90,0%
11590	45	35	55	50	57,2%	70,9%	92,6%	95,8%	90,6%
11591	45	35	55	55	57,6%	70,6%	92,8%	96,1%	91,2%
11592	45	35	55	60	58,0%	70,3%	93,0%	96,3%	91,7%
11593	45	35	55	65	58,5%	70,1%	93,2%	96,5%	92,2%
11594	45	35	55	70	58,9%	69,8%	93,4%	96,8%	92,7%
11595	45	35	55	75	59,3%	69,5%	93,6%	97,0%	93,1%
11596	45	35	55	80	59,7%	69,2%	93,8%	97,2%	93,6%
11597	45	35	60	20	48,4%	67,6%	90,0%	93,4%	86,0%
11598	45	35	60	25	48,8%	67,4%	90,3%	93,8%	86,8%
11599	45	35	60	30	49,3%	67,1%	90,6%	94,2%	87,6%
11600	45	35	60	35	49,7%	66,8%	90,8%	94,6%	88,3%
11601	45	35	60	40	50,2%	66,5%	91,1%	94,9%	89,0%
11602	45	35	60	45	50,6%	66,2%	91,4%	95,2%	89,6%
11603	45	35	60	50	51,0%	65,9%	91,7%	95,5%	90,3%
11604	45	35	60	55	51,5%	65,6%	91,9%	95,8%	90,8%
11605	45	35	60	60	51,9%	65,3%	92,2%	96,1%	91,4%
11606	45	35	60	65	52,4%	65,0%	92,4%	96,3%	91,9%
11607	45	35	60	70	52,8%	64,7%	92,6%	96,6%	92,4%
11608	45	35	60	75	53,2%	64,4%	92,9%	96,8%	92,9%
11609	45	35	60	80	53,7%	64,1%	93,1%	97,0%	93,3%
11610	45	35	65	20	42,3%	62,4%	88,8%	93,0%	85,5%
11611	45	35	65	25	42,7%	62,1%	89,1%	93,4%	86,3%
11612	45	35	65	30	43,2%	61,8%	89,4%	93,9%	87,1%
11613	45	35	65	35	43,6%	61,5%	89,8%	94,2%	87,9%
11614	45	35	65	40	44,0%	61,2%	90,1%	94,6%	88,6%
11615	45	35	65	45	44,5%	60,8%	90,4%	94,9%	89,2%

11616	45	35	65	50	44,9%	60,5%	90,7%	95,3%	89,9%
11617	45	35	65	55	45,3%	60,2%	90,9%	95,6%	90,5%
11618	45	35	65	60	45,8%	59,9%	91,2%	95,8%	91,1%
11619	45	35	65	65	46,2%	59,6%	91,5%	96,1%	91,6%
11620	45	35	65	70	46,6%	59,3%	91,7%	96,4%	92,1%
11621	45	35	65	75	47,1%	59,0%	92,0%	96,6%	92,6%
11622	45	35	65	80	47,5%	58,6%	92,2%	96,8%	93,1%
11623	45	35	70	20	36,4%	56,8%	87,5%	92,6%	85,0%
11624	45	35	70	25	36,8%	56,5%	87,9%	93,1%	85,8%
11625	45	35	70	30	37,2%	56,2%	88,2%	93,5%	86,6%
11626	45	35	70	35	37,6%	55,9%	88,6%	93,9%	87,4%
11627	45	35	70	40	38,1%	55,6%	88,9%	94,3%	88,1%
11628	45	35	70	45	38,5%	55,2%	89,2%	94,6%	88,8%
11629	45	35	70	50	38,9%	54,9%	89,6%	95,0%	89,5%
11630	45	35	70	55	39,3%	54,6%	89,9%	95,3%	90,1%
11631	45	35	70	60	39,7%	54,3%	90,2%	95,6%	90,7%
11632	45	35	70	65	40,2%	53,9%	90,5%	95,9%	91,3%
11633	45	35	70	70	40,6%	53,6%	90,8%	96,1%	91,8%
11634	45	35	70	75	41,0%	53,3%	91,0%	96,4%	92,3%
11635	45	35	70	80	41,4%	53,0%	91,3%	96,6%	92,8%
11636	45	35	75	20	30,9%	51,1%	86,1%	92,2%	84,4%
11637	45	35	75	25	31,3%	50,8%	86,5%	92,7%	85,3%
11638	45	35	75	30	31,7%	50,5%	86,9%	93,1%	86,1%
11639	45	35	75	35	32,0%	50,1%	87,2%	93,5%	86,9%
11640	45	35	75	40	32,4%	49,8%	87,6%	93,9%	87,7%
11641	45	35	75	45	32,8%	49,5%	88,0%	94,3%	88,4%
11642	45	35	75	50	33,2%	49,1%	88,3%	94,7%	89,1%
11643	45	35	75	55	33,6%	48,8%	88,7%	95,0%	89,8%
11644	45	35	75	60	34,0%	48,5%	89,0%	95,3%	90,4%
11645	45	35	75	65	34,4%	48,2%	89,3%	95,6%	90,9%
11646	45	35	75	70	34,8%	47,8%	89,7%	95,9%	91,5%
11647	45	35	75	75	35,2%	47,5%	90,0%	96,2%	92,0%
11648	45	35	75	80	35,6%	47,2%	90,3%	96,4%	92,5%

11649	45	35	80	20	25,9%	45,3%	84,5%	91,7%	83,9%
11650	45	35	80	25	26,2%	45,0%	84,9%	92,2%	84,8%
11651	45	35	80	30	26,6%	44,7%	85,4%	92,7%	85,7%
11652	45	35	80	35	26,9%	44,4%	85,8%	93,2%	86,5%
11653	45	35	80	40	27,3%	44,1%	86,2%	93,6%	87,3%
11654	45	35	80	45	27,6%	43,7%	86,6%	94,0%	88,0%
11655	45	35	80	50	28,0%	43,4%	87,0%	94,4%	88,7%
11656	45	35	80	55	28,3%	43,1%	87,4%	94,7%	89,4%
11657	45	35	80	60	28,7%	42,8%	87,7%	95,1%	90,0%
11658	45	35	80	65	29,0%	42,5%	88,1%	95,4%	90,6%
11659	45	35	80	70	29,4%	42,1%	88,4%	95,7%	91,2%
11660	45	35	80	75	29,8%	41,8%	88,8%	95,9%	91,7%
11661	45	35	80	80	30,2%	41,5%	89,1%	96,2%	92,2%
11662	45	40	20	20	88,1%	93,5%	95,9%	95,5%	88,6%
11663	45	40	20	25	88,3%	93,4%	96,0%	95,8%	89,3%
11664	45	40	20	30	88,4%	93,3%	96,2%	96,1%	89,9%
11665	45	40	20	35	88,6%	93,2%	96,3%	96,3%	90,5%
11666	45	40	20	40	88,8%	93,2%	96,4%	96,6%	91,1%
11667	45	40	20	45	89,0%	93,1%	96,5%	96,8%	91,6%
11668	45	40	20	50	89,1%	93,0%	96,6%	97,0%	92,1%
11669	45	40	20	55	89,3%	92,9%	96,7%	97,2%	92,6%
11670	45	40	20	60	89,5%	92,8%	96,9%	97,4%	93,1%
11671	45	40	20	65	89,6%	92,7%	97,0%	97,5%	93,5%
11672	45	40	20	70	89,8%	92,6%	97,1%	97,7%	93,9%
11673	45	40	20	75	90,0%	92,6%	97,1%	97,9%	94,3%
11674	45	40	20	80	90,1%	92,5%	97,2%	98,0%	94,6%
11675	45	40	25	20	85,2%	91,9%	95,4%	95,3%	88,2%
11676	45	40	25	25	85,5%	91,8%	95,5%	95,6%	88,9%
11677	45	40	25	30	85,7%	91,7%	95,7%	95,8%	89,5%
11678	45	40	25	35	85,9%	91,6%	95,8%	96,1%	90,1%
11679	45	40	25	40	86,1%	91,5%	96,0%	96,4%	90,7%
11680	45	40	25	45	86,3%	91,4%	96,1%	96,6%	91,3%
11681	45	40	25	50	86,5%	91,3%	96,2%	96,8%	91,8%

11682	45	40	25	55	86,7%	91,2%	96,3%	97,0%	92,3%
11683	45	40	25	60	86,9%	91,1%	96,5%	97,2%	92,8%
11684	45	40	25	65	87,1%	91,0%	96,6%	97,4%	93,2%
11685	45	40	25	70	87,3%	90,9%	96,7%	97,6%	93,7%
11686	45	40	25	75	87,5%	90,8%	96,8%	97,7%	94,1%
11687	45	40	25	80	87,7%	90,7%	96,9%	97,9%	94,4%
11688	45	40	30	20	81,8%	90,0%	94,8%	95,0%	87,7%
11689	45	40	30	25	82,1%	89,9%	95,0%	95,3%	88,4%
11690	45	40	30	30	82,4%	89,8%	95,1%	95,6%	89,1%
11691	45	40	30	35	82,6%	89,7%	95,3%	95,9%	89,8%
11692	45	40	30	40	82,9%	89,6%	95,4%	96,1%	90,4%
11693	45	40	30	45	83,1%	89,4%	95,6%	96,4%	91,0%
11694	45	40	30	50	83,4%	89,3%	95,7%	96,6%	91,5%
11695	45	40	30	55	83,6%	89,2%	95,9%	96,8%	92,0%
11696	45	40	30	60	83,8%	89,1%	96,0%	97,0%	92,5%
11697	45	40	30	65	84,1%	88,9%	96,1%	97,2%	93,0%
11698	45	40	30	70	84,3%	88,8%	96,3%	97,4%	93,4%
11699	45	40	30	75	84,6%	88,7%	96,4%	97,6%	93,8%
11700	45	40	30	80	84,8%	88,5%	96,5%	97,7%	94,2%
11701	45	40	35	20	77,9%	87,8%	94,2%	94,7%	87,3%
11702	45	40	35	25	78,2%	87,6%	94,4%	95,0%	88,0%
11703	45	40	35	30	78,5%	87,5%	94,5%	95,3%	88,7%
11704	45	40	35	35	78,8%	87,3%	94,7%	95,6%	89,4%
11705	45	40	35	40	79,1%	87,2%	94,9%	95,9%	90,0%
11706	45	40	35	45	79,4%	87,0%	95,0%	96,2%	90,6%
11707	45	40	35	50	79,7%	86,9%	95,2%	96,4%	91,2%
11708	45	40	35	55	79,9%	86,8%	95,3%	96,7%	91,7%
11709	45	40	35	60	80,2%	86,6%	95,5%	96,9%	92,2%
11710	45	40	35	65	80,5%	86,4%	95,6%	97,1%	92,7%
11711	45	40	35	70	80,8%	86,3%	95,8%	97,3%	93,2%
11712	45	40	35	75	81,0%	86,1%	95,9%	97,4%	93,6%
11713	45	40	35	80	81,3%	86,0%	96,0%	97,6%	94,0%
11714	45	40	40	20	73,3%	85,1%	93,5%	94,4%	86,8%

11715	45	40	40	25	73,7%	84,9%	93,7%	94,7%	87,6%
11716	45	40	40	30	74,0%	84,7%	93,9%	95,1%	88,3%
11717	45	40	40	35	74,4%	84,6%	94,0%	95,4%	89,0%
11718	45	40	40	40	74,7%	84,4%	94,2%	95,7%	89,6%
11719	45	40	40	45	75,0%	84,2%	94,4%	95,9%	90,3%
11720	45	40	40	50	75,4%	84,0%	94,6%	96,2%	90,8%
11721	45	40	40	55	75,7%	83,9%	94,8%	96,4%	91,4%
11722	45	40	40	60	76,0%	83,7%	94,9%	96,7%	91,9%
11723	45	40	40	65	76,3%	83,5%	95,1%	96,9%	92,4%
11724	45	40	40	70	76,6%	83,3%	95,2%	97,1%	92,9%
11725	45	40	40	75	77,0%	83,1%	95,4%	97,3%	93,3%
11726	45	40	40	80	77,3%	83,0%	95,5%	97,5%	93,7%
11727	45	40	45	20	68,3%	81,9%	92,6%	94,0%	86,3%
11728	45	40	45	25	68,6%	81,7%	92,9%	94,4%	87,1%
11729	45	40	45	30	69,0%	81,5%	93,1%	94,8%	87,9%
11730	45	40	45	35	69,4%	81,3%	93,3%	95,1%	88,6%
11731	45	40	45	40	69,8%	81,1%	93,5%	95,4%	89,3%
11732	45	40	45	45	70,1%	80,9%	93,7%	95,7%	89,9%
11733	45	40	45	50	70,5%	80,7%	93,9%	96,0%	90,5%
11734	45	40	45	55	70,9%	80,5%	94,1%	96,2%	91,1%
11735	45	40	45	60	71,2%	80,3%	94,3%	96,5%	91,6%
11736	45	40	45	65	71,6%	80,1%	94,5%	96,7%	92,1%
11737	45	40	45	70	71,9%	79,9%	94,7%	96,9%	92,6%
11738	45	40	45	75	72,3%	79,7%	94,8%	97,1%	93,1%
11739	45	40	45	80	72,6%	79,4%	95,0%	97,3%	93,5%
11740	45	40	50	20	62,7%	78,2%	91,8%	93,7%	85,8%
11741	45	40	50	25	63,1%	78,0%	92,0%	94,1%	86,6%
11742	45	40	50	30	63,5%	77,8%	92,3%	94,5%	87,4%
11743	45	40	50	35	63,9%	77,5%	92,5%	94,8%	88,2%
11744	45	40	50	40	64,3%	77,3%	92,7%	95,1%	88,9%
11745	45	40	50	45	64,7%	77,1%	93,0%	95,4%	89,5%
11746	45	40	50	50	65,1%	76,8%	93,2%	95,7%	90,1%
11747	45	40	50	55	65,5%	76,6%	93,4%	96,0%	90,7%

11748	45	40	50	60	65,9%	76,4%	93,6%	96,3%	91,3%
11749	45	40	50	65	66,3%	76,1%	93,8%	96,5%	91,8%
11750	45	40	50	70	66,7%	75,9%	94,0%	96,7%	92,3%
11751	45	40	50	75	67,1%	75,7%	94,2%	96,9%	92,8%
11752	45	40	50	80	67,5%	75,4%	94,4%	97,1%	93,2%
11753	45	40	55	20	56,7%	74,0%	90,8%	93,3%	85,3%
11754	45	40	55	25	57,2%	73,8%	91,0%	93,7%	86,2%
11755	45	40	55	30	57,6%	73,5%	91,3%	94,1%	87,0%
11756	45	40	55	35	58,0%	73,3%	91,6%	94,5%	87,7%
11757	45	40	55	40	58,5%	73,0%	91,8%	94,8%	88,4%
11758	45	40	55	45	58,9%	72,7%	92,1%	95,2%	89,1%
11759	45	40	55	50	59,3%	72,5%	92,3%	95,5%	89,8%
11760	45	40	55	55	59,7%	72,2%	92,6%	95,8%	90,4%
11761	45	40	55	60	60,2%	72,0%	92,8%	96,0%	91,0%
11762	45	40	55	65	60,6%	71,7%	93,0%	96,3%	91,5%
11763	45	40	55	70	61,0%	71,4%	93,2%	96,5%	92,0%
11764	45	40	55	75	61,4%	71,2%	93,5%	96,8%	92,5%
11765	45	40	55	80	61,8%	70,9%	93,7%	97,0%	93,0%
11766	45	40	60	20	50,6%	69,3%	89,7%	92,9%	84,8%
11767	45	40	60	25	51,1%	69,1%	90,0%	93,4%	85,7%
11768	45	40	60	30	51,5%	68,8%	90,3%	93,8%	86,5%
11769	45	40	60	35	51,9%	68,5%	90,6%	94,2%	87,3%
11770	45	40	60	40	52,4%	68,2%	90,9%	94,5%	88,0%
11771	45	40	60	45	52,8%	67,9%	91,1%	94,9%	88,7%
11772	45	40	60	50	53,3%	67,6%	91,4%	95,2%	89,4%
11773	45	40	60	55	53,7%	67,4%	91,7%	95,5%	90,0%
11774	45	40	60	60	54,1%	67,1%	91,9%	95,8%	90,6%
11775	45	40	60	65	54,6%	66,8%	92,2%	96,1%	91,2%
11776	45	40	60	70	55,0%	66,5%	92,4%	96,3%	91,7%
11777	45	40	60	75	55,4%	66,2%	92,7%	96,6%	92,2%
11778	45	40	60	80	55,9%	65,9%	92,9%	96,8%	92,7%
11779	45	40	65	20	44,5%	64,2%	88,5%	92,5%	84,3%
11780	45	40	65	25	44,9%	63,9%	88,8%	93,0%	85,1%

11781	45	40	65	30	45,3%	63,6%	89,1%	93,4%	86,0%
11782	45	40	65	35	45,8%	63,3%	89,5%	93,8%	86,8%
11783	45	40	65	40	46,2%	63,0%	89,8%	94,2%	87,6%
11784	45	40	65	45	46,7%	62,7%	90,1%	94,6%	88,3%
11785	45	40	65	50	47,1%	62,4%	90,4%	94,9%	89,0%
11786	45	40	65	55	47,5%	62,1%	90,7%	95,2%	89,6%
11787	45	40	65	60	48,0%	61,8%	91,0%	95,5%	90,3%
11788	45	40	65	65	48,4%	61,5%	91,2%	95,8%	90,8%
11789	45	40	65	70	48,9%	61,2%	91,5%	96,1%	91,4%
11790	45	40	65	75	49,3%	60,9%	91,8%	96,3%	91,9%
11791	45	40	65	80	49,7%	60,5%	92,0%	96,6%	92,4%
11792	45	40	70	20	38,5%	58,8%	87,1%	92,1%	83,7%
11793	45	40	70	25	38,9%	58,4%	87,5%	92,6%	84,6%
11794	45	40	70	30	39,3%	58,1%	87,9%	93,0%	85,5%
11795	45	40	70	35	39,7%	57,8%	88,2%	93,5%	86,3%
11796	45	40	70	40	40,2%	57,5%	88,6%	93,9%	87,1%
11797	45	40	70	45	40,6%	57,2%	88,9%	94,3%	87,9%
11798	45	40	70	50	41,0%	56,8%	89,2%	94,6%	88,6%
11799	45	40	70	55	41,4%	56,5%	89,6%	95,0%	89,2%
11800	45	40	70	60	41,9%	56,2%	89,9%	95,3%	89,9%
11801	45	40	70	65	42,3%	55,9%	90,2%	95,6%	90,5%
11802	45	40	70	70	42,7%	55,6%	90,5%	95,9%	91,1%
11803	45	40	70	75	43,2%	55,2%	90,8%	96,1%	91,6%
11804	45	40	70	80	43,6%	54,9%	91,1%	96,4%	92,1%
11805	45	40	75	20	32,8%	53,1%	85,7%	91,6%	83,1%
11806	45	40	75	25	33,2%	52,7%	86,1%	92,1%	84,1%
11807	45	40	75	30	33,6%	52,4%	86,5%	92,6%	85,0%
11808	45	40	75	35	34,0%	52,1%	86,9%	93,1%	85,8%
11809	45	40	75	40	34,4%	51,8%	87,3%	93,5%	86,6%
11810	45	40	75	45	34,8%	51,4%	87,6%	93,9%	87,4%
11811	45	40	75	50	35,2%	51,1%	88,0%	94,3%	88,1%
11812	45	40	75	55	35,6%	50,8%	88,4%	94,7%	88,8%
11813	45	40	75	60	36,0%	50,5%	88,7%	95,0%	89,5%

11814	45	40	75	65	36,4%	50,1%	89,0%	95,3%	90,1%
11815	45	40	75	70	36,8%	49,8%	89,4%	95,6%	90,7%
11816	45	40	75	75	37,2%	49,5%	89,7%	95,9%	91,3%
11817	45	40	75	80	37,6%	49,2%	90,0%	96,2%	91,8%
11818	45	40	80	20	27,6%	47,3%	84,1%	91,1%	82,6%
11819	45	40	80	25	28,0%	47,0%	84,5%	91,7%	83,5%
11820	45	40	80	30	28,3%	46,7%	85,0%	92,2%	84,4%
11821	45	40	80	35	28,7%	46,3%	85,4%	92,7%	85,3%
11822	45	40	80	40	29,1%	46,0%	85,8%	93,1%	86,2%
11823	45	40	80	45	29,4%	45,7%	86,2%	93,6%	87,0%
11824	45	40	80	50	29,8%	45,4%	86,6%	94,0%	87,7%
11825	45	40	80	55	30,2%	45,0%	87,0%	94,3%	88,4%
11826	45	40	80	60	30,5%	44,7%	87,4%	94,7%	89,1%
11827	45	40	80	65	30,9%	44,4%	87,8%	95,0%	89,8%
11828	45	40	80	70	31,3%	44,1%	88,1%	95,3%	90,4%
11829	45	40	80	75	31,7%	43,7%	88,5%	95,6%	90,9%
11830	45	40	80	80	32,1%	43,4%	88,8%	95,9%	91,5%
11831	45	45	20	20	89,0%	93,9%	95,8%	95,2%	87,6%
11832	45	45	20	25	89,2%	93,9%	95,9%	95,5%	88,3%
11833	45	45	20	30	89,3%	93,8%	96,1%	95,8%	89,0%
11834	45	45	20	35	89,5%	93,7%	96,2%	96,1%	89,7%
11835	45	45	20	40	89,7%	93,6%	96,3%	96,3%	90,3%
11836	45	45	20	45	89,8%	93,6%	96,4%	96,6%	90,9%
11837	45	45	20	50	90,0%	93,5%	96,5%	96,8%	91,4%
11838	45	45	20	55	90,1%	93,4%	96,6%	97,0%	91,9%
11839	45	45	20	60	90,3%	93,3%	96,8%	97,2%	92,4%
11840	45	45	20	65	90,4%	93,2%	96,9%	97,4%	92,9%
11841	45	45	20	70	90,6%	93,2%	97,0%	97,5%	93,3%
11842	45	45	20	75	90,7%	93,1%	97,1%	97,7%	93,7%
11843	45	45	20	80	90,9%	93,0%	97,2%	97,8%	94,1%
11844	45	45	25	20	86,3%	92,5%	95,3%	94,9%	87,1%
11845	45	45	25	25	86,5%	92,4%	95,4%	95,2%	87,9%
11846	45	45	25	30	86,7%	92,3%	95,6%	95,5%	88,6%

11847	45	45	25	35	86,9%	92,2%	95,7%	95,8%	89,3%
11848	45	45	25	40	87,1%	92,1%	95,8%	96,1%	89,9%
11849	45	45	25	45	87,3%	92,0%	96,0%	96,3%	90,5%
11850	45	45	25	50	87,5%	91,9%	96,1%	96,6%	91,1%
11851	45	45	25	55	87,7%	91,8%	96,2%	96,8%	91,6%
11852	45	45	25	60	87,9%	91,7%	96,3%	97,0%	92,1%
11853	45	45	25	65	88,1%	91,6%	96,5%	97,2%	92,6%
11854	45	45	25	70	88,3%	91,5%	96,6%	97,4%	93,1%
11855	45	45	25	75	88,4%	91,4%	96,7%	97,6%	93,5%
11856	45	45	25	80	88,6%	91,3%	96,8%	97,7%	93,9%
11857	45	45	30	20	83,1%	90,7%	94,7%	94,6%	86,7%
11858	45	45	30	25	83,4%	90,6%	94,8%	95,0%	87,4%
11859	45	45	30	30	83,6%	90,5%	95,0%	95,3%	88,2%
11860	45	45	30	35	83,9%	90,4%	95,2%	95,6%	88,9%
11861	45	45	30	40	84,1%	90,3%	95,3%	95,9%	89,5%
11862	45	45	30	45	84,3%	90,2%	95,5%	96,1%	90,1%
11863	45	45	30	50	84,6%	90,0%	95,6%	96,4%	90,7%
11864	45	45	30	55	84,8%	89,9%	95,7%	96,6%	91,3%
11865	45	45	30	60	85,0%	89,8%	95,9%	96,8%	91,8%
11866	45	45	30	65	85,2%	89,7%	96,0%	97,0%	92,3%
11867	45	45	30	70	85,5%	89,6%	96,1%	97,2%	92,8%
11868	45	45	30	75	85,7%	89,4%	96,3%	97,4%	93,2%
11869	45	45	30	80	85,9%	89,3%	96,4%	97,6%	93,7%
11870	45	45	35	20	79,4%	88,6%	94,0%	94,3%	86,2%
11871	45	45	35	25	79,7%	88,5%	94,2%	94,7%	87,0%
11872	45	45	35	30	79,9%	88,3%	94,4%	95,0%	87,7%
11873	45	45	35	35	80,2%	88,2%	94,5%	95,3%	88,4%
11874	45	45	35	40	80,5%	88,0%	94,7%	95,6%	89,1%
11875	45	45	35	45	80,8%	87,9%	94,9%	95,9%	89,8%
11876	45	45	35	50	81,1%	87,8%	95,0%	96,2%	90,4%
11877	45	45	35	55	81,3%	87,6%	95,2%	96,4%	91,0%
11878	45	45	35	60	81,6%	87,5%	95,4%	96,6%	91,5%
11879	45	45	35	65	81,9%	87,3%	95,5%	96,9%	92,0%

11880	45	45	35	70	82,1%	87,2%	95,6%	97,1%	92,5%
11881	45	45	35	75	82,4%	87,1%	95,8%	97,2%	93,0%
11882	45	45	35	80	82,6%	86,9%	95,9%	97,4%	93,4%
11883	45	45	40	20	75,0%	86,0%	93,3%	94,0%	85,7%
11884	45	45	40	25	75,4%	85,9%	93,5%	94,3%	86,5%
11885	45	45	40	30	75,7%	85,7%	93,7%	94,7%	87,3%
11886	45	45	40	35	76,0%	85,6%	93,9%	95,0%	88,0%
11887	45	45	40	40	76,3%	85,4%	94,1%	95,3%	88,7%
11888	45	45	40	45	76,7%	85,2%	94,2%	95,6%	89,4%
11889	45	45	40	50	77,0%	85,1%	94,4%	95,9%	90,0%
11890	45	45	40	55	77,3%	84,9%	94,6%	96,2%	90,6%
11891	45	45	40	60	77,6%	84,7%	94,8%	96,4%	91,2%
11892	45	45	40	65	77,9%	84,6%	94,9%	96,7%	91,7%
11893	45	45	40	70	78,2%	84,4%	95,1%	96,9%	92,2%
11894	45	45	40	75	78,5%	84,2%	95,3%	97,1%	92,7%
11895	45	45	40	80	78,8%	84,0%	95,4%	97,3%	93,2%
11896	45	45	45	20	70,1%	83,0%	92,4%	93,6%	85,2%
11897	45	45	45	25	70,5%	82,8%	92,7%	94,0%	86,0%
11898	45	45	45	30	70,9%	82,7%	92,9%	94,4%	86,8%
11899	45	45	45	35	71,2%	82,5%	93,1%	94,7%	87,6%
11900	45	45	45	40	71,6%	82,3%	93,3%	95,1%	88,3%
11901	45	45	45	45	72,0%	82,1%	93,5%	95,4%	89,0%
11902	45	45	45	50	72,3%	81,9%	93,7%	95,7%	89,6%
11903	45	45	45	55	72,7%	81,7%	93,9%	96,0%	90,3%
11904	45	45	45	60	73,0%	81,5%	94,1%	96,2%	90,8%
11905	45	45	45	65	73,4%	81,3%	94,3%	96,5%	91,4%
11906	45	45	45	70	73,7%	81,1%	94,5%	96,7%	91,9%
11907	45	45	45	75	74,0%	80,9%	94,7%	96,9%	92,4%
11908	45	45	45	80	74,4%	80,7%	94,8%	97,1%	92,9%
11909	45	45	50	20	64,7%	79,5%	91,5%	93,2%	84,6%
11910	45	45	50	25	65,1%	79,3%	91,8%	93,7%	85,5%
11911	45	45	50	30	65,5%	79,1%	92,0%	94,0%	86,3%
11912	45	45	50	35	65,9%	78,9%	92,3%	94,4%	87,1%

11913	45	45	50	40	66,3%	78,7%	92,5%	94,8%	87,9%
11914	45	45	50	45	66,7%	78,4%	92,7%	95,1%	88,6%
11915	45	45	50	50	67,1%	78,2%	93,0%	95,4%	89,3%
11916	45	45	50	55	67,5%	78,0%	93,2%	95,7%	89,9%
11917	45	45	50	60	67,9%	77,8%	93,4%	96,0%	90,5%
11918	45	45	50	65	68,3%	77,5%	93,6%	96,2%	91,1%
11919	45	45	50	70	68,6%	77,3%	93,8%	96,5%	91,6%
11920	45	45	50	75	69,0%	77,1%	94,0%	96,7%	92,1%
11921	45	45	50	80	69,4%	76,8%	94,2%	96,9%	92,6%
11922	45	45	55	20	58,9%	75,5%	90,5%	92,8%	84,1%
11923	45	45	55	25	59,3%	75,3%	90,8%	93,3%	85,0%
11924	45	45	55	30	59,8%	75,0%	91,1%	93,7%	85,8%
11925	45	45	55	35	60,2%	74,8%	91,3%	94,1%	86,6%
11926	45	45	55	40	60,6%	74,5%	91,6%	94,5%	87,4%
11927	45	45	55	45	61,0%	74,3%	91,9%	94,8%	88,2%
11928	45	45	55	50	61,4%	74,0%	92,1%	95,1%	88,9%
11929	45	45	55	55	61,9%	73,8%	92,4%	95,5%	89,5%
11930	45	45	55	60	62,3%	73,5%	92,6%	95,7%	90,1%
11931	45	45	55	65	62,7%	73,3%	92,8%	96,0%	90,7%
11932	45	45	55	70	63,1%	73,0%	93,0%	96,3%	91,3%
11933	45	45	55	75	63,5%	72,7%	93,3%	96,5%	91,8%
11934	45	45	55	80	63,9%	72,5%	93,5%	96,7%	92,3%
11935	45	45	60	20	52,8%	71,0%	89,4%	92,4%	83,5%
11936	45	45	60	25	53,3%	70,7%	89,7%	92,9%	84,4%
11937	45	45	60	30	53,7%	70,4%	90,0%	93,3%	85,3%
11938	45	45	60	35	54,1%	70,2%	90,3%	93,7%	86,2%
11939	45	45	60	40	54,6%	69,9%	90,6%	94,1%	87,0%
11940	45	45	60	45	55,0%	69,6%	90,9%	94,5%	87,7%
11941	45	45	60	50	55,5%	69,3%	91,2%	94,9%	88,4%
11942	45	45	60	55	55,9%	69,1%	91,4%	95,2%	89,1%
11943	45	45	60	60	56,3%	68,8%	91,7%	95,5%	89,8%
11944	45	45	60	65	56,8%	68,5%	91,9%	95,8%	90,4%
11945	45	45	60	70	57,2%	68,2%	92,2%	96,0%	91,0%

11946	45	45	60	75	57,6%	67,9%	92,4%	96,3%	91,5%
11947	45	45	60	80	58,1%	67,6%	92,7%	96,5%	92,0%
11948	45	45	65	20	46,7%	66,0%	88,1%	92,0%	83,0%
11949	45	45	65	25	47,1%	65,7%	88,5%	92,5%	83,9%
11950	45	45	65	30	47,5%	65,4%	88,8%	92,9%	84,8%
11951	45	45	65	35	48,0%	65,1%	89,2%	93,4%	85,7%
11952	45	45	65	40	48,4%	64,8%	89,5%	93,8%	86,5%
11953	45	45	65	45	48,9%	64,5%	89,8%	94,2%	87,3%
11954	45	45	65	50	49,3%	64,2%	90,1%	94,5%	88,0%
11955	45	45	65	55	49,7%	63,9%	90,4%	94,9%	88,7%
11956	45	45	65	60	50,2%	63,6%	90,7%	95,2%	89,4%
11957	45	45	65	65	50,6%	63,3%	91,0%	95,5%	90,0%
11958	45	45	65	70	51,1%	63,0%	91,3%	95,8%	90,6%
11959	45	45	65	75	51,5%	62,7%	91,5%	96,1%	91,2%
11960	45	45	65	80	51,9%	62,4%	91,8%	96,3%	91,7%
11961	45	45	70	20	40,6%	60,7%	86,8%	91,5%	82,4%
11962	45	45	70	25	41,0%	60,3%	87,2%	92,0%	83,3%
11963	45	45	70	30	41,5%	60,0%	87,5%	92,5%	84,3%
11964	45	45	70	35	41,9%	59,7%	87,9%	93,0%	85,2%
11965	45	45	70	40	42,3%	59,4%	88,3%	93,4%	86,0%
11966	45	45	70	45	42,7%	59,1%	88,6%	93,8%	86,8%
11967	45	45	70	50	43,2%	58,8%	88,9%	94,2%	87,6%
11968	45	45	70	55	43,6%	58,4%	89,3%	94,6%	88,3%
11969	45	45	70	60	44,0%	58,1%	89,6%	94,9%	89,0%
11970	45	45	70	65	44,5%	57,8%	89,9%	95,3%	89,6%
11971	45	45	70	70	44,9%	57,5%	90,2%	95,6%	90,3%
11972	45	45	70	75	45,4%	57,2%	90,5%	95,8%	90,8%
11973	45	45	70	80	45,8%	56,9%	90,8%	96,1%	91,4%
11974	45	45	75	20	34,8%	55,0%	85,3%	91,0%	81,8%
11975	45	45	75	25	35,2%	54,7%	85,7%	91,6%	82,8%
11976	45	45	75	30	35,6%	54,4%	86,1%	92,1%	83,7%
11977	45	45	75	35	36,0%	54,1%	86,5%	92,6%	84,6%
11978	45	45	75	40	36,4%	53,7%	86,9%	93,0%	85,5%

11979	45	45	75	45	36,8%	53,4%	87,3%	93,5%	86,3%
11980	45	45	75	50	37,2%	53,1%	87,7%	93,9%	87,1%
11981	45	45	75	55	37,7%	52,8%	88,0%	94,3%	87,9%
11982	45	45	75	60	38,1%	52,4%	88,4%	94,6%	88,6%
11983	45	45	75	65	38,5%	52,1%	88,7%	95,0%	89,2%
11984	45	45	75	70	38,9%	51,8%	89,1%	95,3%	89,9%
11985	45	45	75	75	39,3%	51,4%	89,4%	95,6%	90,5%
11986	45	45	75	80	39,8%	51,1%	89,7%	95,9%	91,1%
11987	45	45	80	20	29,4%	49,3%	83,7%	90,5%	81,1%
11988	45	45	80	25	29,8%	48,9%	84,1%	91,1%	82,2%
11989	45	45	80	30	30,2%	48,6%	84,6%	91,6%	83,1%
11990	45	45	80	35	30,5%	48,3%	85,0%	92,2%	84,1%
11991	45	45	80	40	30,9%	48,0%	85,4%	92,6%	85,0%
11992	45	45	80	45	31,3%	47,6%	85,8%	93,1%	85,8%
11993	45	45	80	50	31,7%	47,3%	86,3%	93,5%	86,6%
11994	45	45	80	55	32,1%	47,0%	86,6%	93,9%	87,4%
11995	45	45	80	60	32,4%	46,7%	87,0%	94,3%	88,1%
11996	45	45	80	65	32,8%	46,3%	87,4%	94,7%	88,8%
11997	45	45	80	70	33,2%	46,0%	87,8%	95,0%	89,5%
11998	45	45	80	75	33,6%	45,7%	88,1%	95,3%	90,1%
11999	45	45	80	80	34,0%	45,4%	88,5%	95,6%	90,7%
12000	45	50	20	20	89,8%	94,4%	95,7%	94,9%	86,5%
12001	45	50	20	25	90,0%	94,3%	95,8%	95,2%	87,3%
12002	45	50	20	30	90,1%	94,2%	95,9%	95,5%	88,0%
12003	45	50	20	35	90,3%	94,2%	96,1%	95,8%	88,7%
12004	45	50	20	40	90,4%	94,1%	96,2%	96,0%	89,4%
12005	45	50	20	45	90,6%	94,0%	96,3%	96,3%	90,0%
12006	45	50	20	50	90,7%	93,9%	96,4%	96,5%	90,6%
12007	45	50	20	55	90,9%	93,9%	96,5%	96,8%	91,2%
12008	45	50	20	60	91,0%	93,8%	96,7%	97,0%	91,7%
12009	45	50	20	65	91,2%	93,7%	96,8%	97,2%	92,2%
12010	45	50	20	70	91,3%	93,6%	96,9%	97,4%	92,7%
12011	45	50	20	75	91,5%	93,6%	97,0%	97,5%	93,2%

12012	45	50	20	80	91,6%	93,5%	97,1%	97,7%	93,6%
12013	45	50	25	20	87,3%	93,0%	95,1%	94,5%	86,0%
12014	45	50	25	25	87,5%	92,9%	95,3%	94,9%	86,8%
12015	45	50	25	30	87,7%	92,8%	95,4%	95,2%	87,6%
12016	45	50	25	35	87,9%	92,8%	95,6%	95,5%	88,3%
12017	45	50	25	40	88,1%	92,7%	95,7%	95,8%	89,0%
12018	45	50	25	45	88,3%	92,6%	95,8%	96,1%	89,7%
12019	45	50	25	50	88,5%	92,5%	96,0%	96,3%	90,3%
12020	45	50	25	55	88,6%	92,4%	96,1%	96,6%	90,9%
12021	45	50	25	60	88,8%	92,3%	96,2%	96,8%	91,4%
12022	45	50	25	65	89,0%	92,2%	96,3%	97,0%	91,9%
12023	45	50	25	70	89,2%	92,1%	96,5%	97,2%	92,4%
12024	45	50	25	75	89,3%	92,0%	96,6%	97,4%	92,9%
12025	45	50	25	80	89,5%	91,9%	96,7%	97,5%	93,3%
12026	45	50	30	20	84,3%	91,4%	94,5%	94,2%	85,5%
12027	45	50	30	25	84,6%	91,3%	94,7%	94,6%	86,3%
12028	45	50	30	30	84,8%	91,2%	94,8%	94,9%	87,1%
12029	45	50	30	35	85,0%	91,1%	95,0%	95,3%	87,9%
12030	45	50	30	40	85,2%	90,9%	95,2%	95,6%	88,6%
12031	45	50	30	45	85,5%	90,8%	95,3%	95,8%	89,3%
12032	45	50	30	50	85,7%	90,7%	95,5%	96,1%	89,9%
12033	45	50	30	55	85,9%	90,6%	95,6%	96,4%	90,5%
12034	45	50	30	60	86,1%	90,5%	95,7%	96,6%	91,1%
12035	45	50	30	65	86,3%	90,4%	95,9%	96,8%	91,6%
12036	45	50	30	70	86,5%	90,3%	96,0%	97,0%	92,1%
12037	45	50	30	75	86,7%	90,2%	96,1%	97,2%	92,6%
12038	45	50	30	80	86,9%	90,0%	96,3%	97,4%	93,1%
12039	45	50	35	20	80,8%	89,4%	93,8%	93,9%	85,0%
12040	45	50	35	25	81,1%	89,2%	94,0%	94,3%	85,8%
12041	45	50	35	30	81,3%	89,1%	94,2%	94,6%	86,7%
12042	45	50	35	35	81,6%	89,0%	94,4%	95,0%	87,4%
12043	45	50	35	40	81,9%	88,9%	94,6%	95,3%	88,2%
12044	45	50	35	45	82,1%	88,7%	94,7%	95,6%	88,9%

12045	45	50	35	50	82,4%	88,6%	94,9%	95,9%	89,5%
12046	45	50	35	55	82,6%	88,5%	95,1%	96,1%	90,1%
12047	45	50	35	60	82,9%	88,3%	95,2%	96,4%	90,7%
12048	45	50	35	65	83,1%	88,2%	95,4%	96,6%	91,3%
12049	45	50	35	70	83,4%	88,1%	95,5%	96,8%	91,8%
12050	45	50	35	75	83,6%	87,9%	95,7%	97,0%	92,3%
12051	45	50	35	80	83,9%	87,8%	95,8%	97,2%	92,8%
12052	45	50	40	20	76,7%	87,0%	93,1%	93,5%	84,5%
12053	45	50	40	25	77,0%	86,8%	93,3%	93,9%	85,3%
12054	45	50	40	30	77,3%	86,7%	93,5%	94,3%	86,2%
12055	45	50	40	35	77,6%	86,5%	93,7%	94,7%	87,0%
12056	45	50	40	40	77,9%	86,4%	93,9%	95,0%	87,7%
12057	45	50	40	45	78,2%	86,2%	94,1%	95,3%	88,4%
12058	45	50	40	50	78,5%	86,0%	94,3%	95,6%	89,1%
12059	45	50	40	55	78,8%	85,9%	94,4%	95,9%	89,8%
12060	45	50	40	60	79,1%	85,7%	94,6%	96,2%	90,4%
12061	45	50	40	65	79,4%	85,6%	94,8%	96,4%	91,0%
12062	45	50	40	70	79,7%	85,4%	95,0%	96,6%	91,5%
12063	45	50	40	75	80,0%	85,2%	95,1%	96,9%	92,0%
12064	45	50	40	80	80,2%	85,1%	95,3%	97,1%	92,5%
12065	45	50	45	20	72,0%	84,1%	92,2%	93,1%	83,9%
12066	45	50	45	25	72,3%	83,9%	92,4%	93,6%	84,8%
12067	45	50	45	30	72,7%	83,8%	92,7%	94,0%	85,7%
12068	45	50	45	35	73,0%	83,6%	92,9%	94,4%	86,5%
12069	45	50	45	40	73,4%	83,4%	93,1%	94,7%	87,3%
12070	45	50	45	45	73,7%	83,2%	93,3%	95,0%	88,0%
12071	45	50	45	50	74,1%	83,0%	93,5%	95,4%	88,7%
12072	45	50	45	55	74,4%	82,8%	93,8%	95,7%	89,4%
12073	45	50	45	60	74,7%	82,7%	93,9%	95,9%	90,0%
12074	45	50	45	65	75,1%	82,5%	94,1%	96,2%	90,6%
12075	45	50	45	70	75,4%	82,3%	94,3%	96,4%	91,2%
12076	45	50	45	75	75,7%	82,1%	94,5%	96,7%	91,7%
12077	45	50	45	80	76,0%	81,9%	94,7%	96,9%	92,2%

12078	45	50	50	20	66,7%	80,8%	91,3%	92,7%	83,3%
12079	45	50	50	25	67,1%	80,6%	91,5%	93,2%	84,3%
12080	45	50	50	30	67,5%	80,4%	91,8%	93,6%	85,2%
12081	45	50	50	35	67,9%	80,2%	92,0%	94,0%	86,0%
12082	45	50	50	40	68,3%	79,9%	92,3%	94,4%	86,8%
12083	45	50	50	45	68,7%	79,7%	92,5%	94,8%	87,6%
12084	45	50	50	50	69,0%	79,5%	92,8%	95,1%	88,3%
12085	45	50	50	55	69,4%	79,3%	93,0%	95,4%	89,0%
12086	45	50	50	60	69,8%	79,1%	93,2%	95,7%	89,6%
12087	45	50	50	65	70,2%	78,9%	93,4%	96,0%	90,3%
12088	45	50	50	70	70,5%	78,7%	93,6%	96,2%	90,9%
12089	45	50	50	75	70,9%	78,4%	93,8%	96,5%	91,4%
12090	45	50	50	80	71,2%	78,2%	94,0%	96,7%	91,9%
12091	45	50	55	20	61,0%	76,9%	90,2%	92,3%	82,8%
12092	45	50	55	25	61,5%	76,7%	90,5%	92,8%	83,7%
12093	45	50	55	30	61,9%	76,5%	90,8%	93,2%	84,6%
12094	45	50	55	35	62,3%	76,2%	91,1%	93,7%	85,5%
12095	45	50	55	40	62,7%	76,0%	91,4%	94,1%	86,3%
12096	45	50	55	45	63,1%	75,7%	91,6%	94,4%	87,1%
12097	45	50	55	50	63,5%	75,5%	91,9%	94,8%	87,9%
12098	45	50	55	55	63,9%	75,3%	92,1%	95,1%	88,6%
12099	45	50	55	60	64,3%	75,0%	92,4%	95,4%	89,3%
12100	45	50	55	65	64,7%	74,8%	92,6%	95,7%	89,9%
12101	45	50	55	70	65,1%	74,5%	92,8%	96,0%	90,5%
12102	45	50	55	75	65,5%	74,3%	93,1%	96,3%	91,1%
12103	45	50	55	80	65,9%	74,0%	93,3%	96,5%	91,6%
12104	45	50	60	20	55,0%	72,6%	89,1%	91,9%	82,2%
12105	45	50	60	25	55,5%	72,3%	89,4%	92,4%	83,2%
12106	45	50	60	30	55,9%	72,1%	89,7%	92,9%	84,1%
12107	45	50	60	35	56,3%	71,8%	90,0%	93,3%	85,0%
12108	45	50	60	40	56,8%	71,5%	90,3%	93,7%	85,8%
12109	45	50	60	45	57,2%	71,3%	90,6%	94,1%	86,7%
12110	45	50	60	50	57,6%	71,0%	90,9%	94,5%	87,4%

12111	45	50	60	55	58,1%	70,7%	91,2%	94,8%	88,2%
12112	45	50	60	60	58,5%	70,4%	91,4%	95,2%	88,9%
12113	45	50	60	65	58,9%	70,2%	91,7%	95,5%	89,5%
12114	45	50	60	70	59,3%	69,9%	92,0%	95,8%	90,1%
12115	45	50	60	75	59,8%	69,6%	92,2%	96,0%	90,7%
12116	45	50	60	80	60,2%	69,3%	92,5%	96,3%	91,3%
12117	45	50	65	20	48,9%	67,8%	87,8%	91,4%	81,6%
12118	45	50	65	25	49,3%	67,5%	88,2%	91,9%	82,6%
12119	45	50	65	30	49,8%	67,2%	88,5%	92,4%	83,5%
12120	45	50	65	35	50,2%	66,9%	88,8%	92,9%	84,5%
12121	45	50	65	40	50,6%	66,6%	89,2%	93,3%	85,3%
12122	45	50	65	45	51,1%	66,3%	89,5%	93,8%	86,2%
12123	45	50	65	50	51,5%	66,0%	89,8%	94,2%	87,0%
12124	45	50	65	55	52,0%	65,7%	90,1%	94,5%	87,7%
12125	45	50	65	60	52,4%	65,4%	90,4%	94,9%	88,4%
12126	45	50	65	65	52,8%	65,1%	90,7%	95,2%	89,1%
12127	45	50	65	70	53,3%	64,8%	91,0%	95,5%	89,8%
12128	45	50	65	75	53,7%	64,5%	91,3%	95,8%	90,4%
12129	45	50	65	80	54,2%	64,2%	91,5%	96,1%	91,0%
12130	45	50	70	20	42,8%	62,5%	86,4%	90,9%	80,9%
12131	45	50	70	25	43,2%	62,2%	86,8%	91,5%	82,0%
12132	45	50	70	30	43,6%	61,9%	87,2%	92,0%	83,0%
12133	45	50	70	35	44,1%	61,6%	87,6%	92,5%	83,9%
12134	45	50	70	40	44,5%	61,3%	87,9%	93,0%	84,8%
12135	45	50	70	45	44,9%	61,0%	88,3%	93,4%	85,7%
12136	45	50	70	50	45,4%	60,7%	88,6%	93,8%	86,5%
12137	45	50	70	55	45,8%	60,3%	89,0%	94,2%	87,3%
12138	45	50	70	60	46,2%	60,0%	89,3%	94,6%	88,0%
12139	45	50	70	65	46,7%	59,7%	89,6%	94,9%	88,7%
12140	45	50	70	70	47,1%	59,4%	89,9%	95,2%	89,4%
12141	45	50	70	75	47,6%	59,1%	90,2%	95,5%	90,0%
12142	45	50	70	80	48,0%	58,8%	90,5%	95,8%	90,6%
12143	45	50	75	20	36,9%	57,0%	84,9%	90,4%	80,3%

12144	45	50	75	25	37,3%	56,6%	85,3%	91,0%	81,3%
12145	45	50	75	30	37,7%	56,3%	85,7%	91,5%	82,4%
12146	45	50	75	35	38,1%	56,0%	86,1%	92,1%	83,3%
12147	45	50	75	40	38,5%	55,7%	86,5%	92,5%	84,3%
12148	45	50	75	45	38,9%	55,4%	86,9%	93,0%	85,2%
12149	45	50	75	50	39,3%	55,0%	87,3%	93,4%	86,0%
12150	45	50	75	55	39,8%	54,7%	87,7%	93,9%	86,8%
12151	45	50	75	60	40,2%	54,4%	88,0%	94,2%	87,6%
12152	45	50	75	65	40,6%	54,1%	88,4%	94,6%	88,3%
12153	45	50	75	70	41,0%	53,7%	88,7%	94,9%	89,0%
12154	45	50	75	75	41,5%	53,4%	89,1%	95,3%	89,6%
12155	45	50	75	80	41,9%	53,1%	89,4%	95,6%	90,3%
12156	45	50	80	20	31,3%	51,2%	83,2%	89,9%	79,6%
12157	45	50	80	25	31,7%	50,9%	83,7%	90,5%	80,7%
12158	45	50	80	30	32,1%	50,6%	84,1%	91,1%	81,8%
12159	45	50	80	35	32,5%	50,3%	84,6%	91,6%	82,8%
12160	45	50	80	40	32,8%	49,9%	85,0%	92,1%	83,7%
12161	45	50	80	45	33,2%	49,6%	85,5%	92,6%	84,6%
12162	45	50	80	50	33,6%	49,3%	85,9%	93,1%	85,5%
12163	45	50	80	55	34,0%	49,0%	86,3%	93,5%	86,3%
12164	45	50	80	60	34,4%	48,6%	86,7%	93,9%	87,1%
12165	45	50	80	65	34,8%	48,3%	87,1%	94,3%	87,9%
12166	45	50	80	70	35,2%	48,0%	87,4%	94,6%	88,6%
12167	45	50	80	75	35,6%	47,6%	87,8%	95,0%	89,3%
12168	45	50	80	80	36,0%	47,3%	88,2%	95,3%	89,9%
12169	45	55	20	20	90,6%	94,8%	95,5%	94,5%	85,4%
12170	45	55	20	25	90,8%	94,7%	95,7%	94,8%	86,2%
12171	45	55	20	30	90,9%	94,7%	95,8%	95,2%	87,0%
12172	45	55	20	35	91,0%	94,6%	95,9%	95,5%	87,7%
12173	45	55	20	40	91,2%	94,5%	96,1%	95,8%	88,5%
12174	45	55	20	45	91,3%	94,5%	96,2%	96,0%	89,1%
12175	45	55	20	50	91,5%	94,4%	96,3%	96,3%	89,8%
12176	45	55	20	55	91,6%	94,3%	96,4%	96,5%	90,4%

12177	45	55	20	60	91,7%	94,2%	96,5%	96,7%	91,0%
12178	45	55	20	65	91,9%	94,2%	96,7%	97,0%	91,5%
12179	45	55	20	70	92,0%	94,1%	96,8%	97,2%	92,0%
12180	45	55	20	75	92,1%	94,0%	96,9%	97,3%	92,5%
12181	45	55	20	80	92,3%	94,0%	97,0%	97,5%	93,0%
12182	45	55	25	20	88,3%	93,5%	95,0%	94,2%	84,8%
12183	45	55	25	25	88,5%	93,4%	95,1%	94,5%	85,7%
12184	45	55	25	30	88,6%	93,4%	95,3%	94,9%	86,5%
12185	45	55	25	35	88,8%	93,3%	95,4%	95,2%	87,3%
12186	45	55	25	40	89,0%	93,2%	95,6%	95,5%	88,0%
12187	45	55	25	45	89,2%	93,1%	95,7%	95,8%	88,7%
12188	45	55	25	50	89,3%	93,0%	95,8%	96,1%	89,4%
12189	45	55	25	55	89,5%	92,9%	96,0%	96,3%	90,0%
12190	45	55	25	60	89,7%	92,9%	96,1%	96,5%	90,6%
12191	45	55	25	65	89,8%	92,8%	96,2%	96,8%	91,2%
12192	45	55	25	70	90,0%	92,7%	96,4%	97,0%	91,7%
12193	45	55	25	75	90,1%	92,6%	96,5%	97,2%	92,2%
12194	45	55	25	80	90,3%	92,5%	96,6%	97,4%	92,7%
12195	45	55	30	20	85,5%	92,0%	94,3%	93,8%	84,3%
12196	45	55	30	25	85,7%	91,9%	94,5%	94,2%	85,2%
12197	45	55	30	30	85,9%	91,8%	94,7%	94,6%	86,0%
12198	45	55	30	35	86,1%	91,7%	94,8%	94,9%	86,8%
12199	45	55	30	40	86,3%	91,6%	95,0%	95,2%	87,6%
12200	45	55	30	45	86,5%	91,5%	95,2%	95,5%	88,3%
12201	45	55	30	50	86,7%	91,4%	95,3%	95,8%	89,0%
12202	45	55	30	55	86,9%	91,3%	95,5%	96,1%	89,7%
12203	45	55	30	60	87,1%	91,2%	95,6%	96,3%	90,3%
12204	45	55	30	65	87,3%	91,1%	95,8%	96,6%	90,9%
12205	45	55	30	70	87,5%	90,9%	95,9%	96,8%	91,4%
12206	45	55	30	75	87,7%	90,8%	96,0%	97,0%	91,9%
12207	45	55	30	80	87,9%	90,7%	96,2%	97,2%	92,4%
12208	45	55	35	20	82,1%	90,1%	93,6%	93,4%	83,7%
12209	45	55	35	25	82,4%	90,0%	93,8%	93,9%	84,6%

12210	45	55	35	30	82,6%	89,9%	94,0%	94,2%	85,5%
12211	45	55	35	35	82,9%	89,7%	94,2%	94,6%	86,3%
12212	45	55	35	40	83,1%	89,6%	94,4%	94,9%	87,1%
12213	45	55	35	45	83,4%	89,5%	94,6%	95,3%	87,9%
12214	45	55	35	50	83,6%	89,4%	94,7%	95,6%	88,6%
12215	45	55	35	55	83,9%	89,2%	94,9%	95,9%	89,3%
12216	45	55	35	60	84,1%	89,1%	95,1%	96,1%	89,9%
12217	45	55	35	65	84,3%	89,0%	95,2%	96,4%	90,5%
12218	45	55	35	70	84,6%	88,9%	95,4%	96,6%	91,1%
12219	45	55	35	75	84,8%	88,7%	95,5%	96,8%	91,6%
12220	45	55	35	80	85,0%	88,6%	95,7%	97,0%	92,1%
12221	45	55	40	20	78,2%	87,8%	92,8%	93,1%	83,2%
12222	45	55	40	25	78,5%	87,7%	93,1%	93,5%	84,1%
12223	45	55	40	30	78,8%	87,5%	93,3%	93,9%	85,0%
12224	45	55	40	35	79,1%	87,4%	93,5%	94,3%	85,9%
12225	45	55	40	40	79,4%	87,3%	93,7%	94,6%	86,7%
12226	45	55	40	45	79,7%	87,1%	93,9%	95,0%	87,4%
12227	45	55	40	50	80,0%	87,0%	94,1%	95,3%	88,2%
12228	45	55	40	55	80,2%	86,8%	94,3%	95,6%	88,9%
12229	45	55	40	60	80,5%	86,7%	94,5%	95,9%	89,5%
12230	45	55	40	65	80,8%	86,5%	94,6%	96,1%	90,2%
12231	45	55	40	70	81,1%	86,4%	94,8%	96,4%	90,7%
12232	45	55	40	75	81,3%	86,2%	95,0%	96,6%	91,3%
12233	45	55	40	80	81,6%	86,0%	95,1%	96,8%	91,8%
12234	45	55	45	20	73,7%	85,1%	92,0%	92,7%	82,6%
12235	45	55	45	25	74,1%	85,0%	92,2%	93,1%	83,5%
12236	45	55	45	30	74,4%	84,8%	92,5%	93,5%	84,5%
12237	45	55	45	35	74,7%	84,6%	92,7%	93,9%	85,3%
12238	45	55	45	40	75,1%	84,5%	92,9%	94,3%	86,2%
12239	45	55	45	45	75,4%	84,3%	93,1%	94,7%	87,0%
12240	45	55	45	50	75,7%	84,1%	93,4%	95,0%	87,7%
12241	45	55	45	55	76,0%	83,9%	93,6%	95,3%	88,5%
12242	45	55	45	60	76,4%	83,8%	93,8%	95,6%	89,1%

12243	45	55	45	65	76,7%	83,6%	94,0%	95,9%	89,8%
12244	45	55	45	70	77,0%	83,4%	94,1%	96,2%	90,4%
12245	45	55	45	75	77,3%	83,2%	94,3%	96,4%	91,0%
12246	45	55	45	80	77,6%	83,0%	94,5%	96,7%	91,5%
12247	45	55	50	20	68,7%	82,0%	91,0%	92,2%	82,0%
12248	45	55	50	25	69,0%	81,8%	91,3%	92,7%	83,0%
12249	45	55	50	30	69,4%	81,6%	91,5%	93,2%	83,9%
12250	45	55	50	35	69,8%	81,4%	91,8%	93,6%	84,8%
12251	45	55	50	40	70,2%	81,2%	92,1%	94,0%	85,7%
12252	45	55	50	45	70,5%	81,0%	92,3%	94,4%	86,5%
12253	45	55	50	50	70,9%	80,8%	92,5%	94,7%	87,3%
12254	45	55	50	55	71,3%	80,6%	92,8%	95,1%	88,0%
12255	45	55	50	60	71,6%	80,4%	93,0%	95,4%	88,7%
12256	45	55	50	65	72,0%	80,2%	93,2%	95,7%	89,4%
12257	45	55	50	70	72,3%	80,0%	93,4%	95,9%	90,0%
12258	45	55	50	75	72,7%	79,7%	93,6%	96,2%	90,6%
12259	45	55	50	80	73,0%	79,5%	93,8%	96,4%	91,2%
12260	45	55	55	20	63,1%	78,3%	89,9%	91,8%	81,4%
12261	45	55	55	25	63,5%	78,1%	90,2%	92,3%	82,4%
12262	45	55	55	30	63,9%	77,9%	90,5%	92,8%	83,4%
12263	45	55	55	35	64,4%	77,6%	90,8%	93,2%	84,3%
12264	45	55	55	40	64,8%	77,4%	91,1%	93,6%	85,2%
12265	45	55	55	45	65,2%	77,2%	91,4%	94,0%	86,0%
12266	45	55	55	50	65,6%	76,9%	91,6%	94,4%	86,8%
12267	45	55	55	55	66,0%	76,7%	91,9%	94,8%	87,6%
12268	45	55	55	60	66,3%	76,5%	92,1%	95,1%	88,3%
12269	45	55	55	65	66,7%	76,2%	92,4%	95,4%	89,0%
12270	45	55	55	70	67,1%	76,0%	92,6%	95,7%	89,6%
12271	45	55	55	75	67,5%	75,8%	92,8%	96,0%	90,3%
12272	45	55	55	80	67,9%	75,5%	93,1%	96,2%	90,9%
12273	45	55	60	20	57,2%	74,1%	88,8%	91,3%	80,7%
12274	45	55	60	25	57,7%	73,9%	89,1%	91,8%	81,8%
12275	45	55	60	30	58,1%	73,6%	89,4%	92,3%	82,8%

12276	45	55	60	35	58,5%	73,4%	89,7%	92,8%	83,7%
12277	45	55	60	40	58,9%	73,1%	90,0%	93,3%	84,6%
12278	45	55	60	45	59,4%	72,8%	90,3%	93,7%	85,5%
12279	45	55	60	50	59,8%	72,6%	90,6%	94,1%	86,3%
12280	45	55	60	55	60,2%	72,3%	90,9%	94,5%	87,1%
12281	45	55	60	60	60,6%	72,1%	91,2%	94,8%	87,9%
12282	45	55	60	65	61,1%	71,8%	91,5%	95,1%	88,6%
12283	45	55	60	70	61,5%	71,5%	91,7%	95,4%	89,3%
12284	45	55	60	75	61,9%	71,3%	92,0%	95,7%	89,9%
12285	45	55	60	80	62,3%	71,0%	92,2%	96,0%	90,5%
12286	45	55	65	20	51,1%	69,5%	87,5%	90,8%	80,1%
12287	45	55	65	25	51,5%	69,2%	87,8%	91,4%	81,2%
12288	45	55	65	30	52,0%	68,9%	88,2%	91,9%	82,2%
12289	45	55	65	35	52,4%	68,6%	88,5%	92,4%	83,2%
12290	45	55	65	40	52,9%	68,3%	88,9%	92,9%	84,1%
12291	45	55	65	45	53,3%	68,0%	89,2%	93,3%	85,0%
12292	45	55	65	50	53,7%	67,8%	89,5%	93,7%	85,8%
12293	45	55	65	55	54,2%	67,5%	89,8%	94,1%	86,7%
12294	45	55	65	60	54,6%	67,2%	90,1%	94,5%	87,4%
12295	45	55	65	65	55,0%	66,9%	90,4%	94,8%	88,2%
12296	45	55	65	70	55,5%	66,6%	90,7%	95,2%	88,9%
12297	45	55	65	75	55,9%	66,3%	91,0%	95,5%	89,5%
12298	45	55	65	80	56,4%	66,0%	91,3%	95,8%	90,1%
12299	45	55	70	20	44,9%	64,3%	86,0%	90,3%	79,4%
12300	45	55	70	25	45,4%	64,0%	86,4%	90,9%	80,5%
12301	45	55	70	30	45,8%	63,7%	86,8%	91,4%	81,6%
12302	45	55	70	35	46,3%	63,4%	87,2%	92,0%	82,6%
12303	45	55	70	40	46,7%	63,1%	87,6%	92,5%	83,5%
12304	45	55	70	45	47,1%	62,8%	87,9%	92,9%	84,5%
12305	45	55	70	50	47,6%	62,5%	88,3%	93,4%	85,3%
12306	45	55	70	55	48,0%	62,2%	88,6%	93,8%	86,2%
12307	45	55	70	60	48,5%	61,9%	89,0%	94,2%	87,0%
12308	45	55	70	65	48,9%	61,6%	89,3%	94,5%	87,7%

12309	45	55	70	70	49,3%	61,3%	89,6%	94,9%	88,4%
12310	45	55	70	75	49,8%	61,0%	89,9%	95,2%	89,1%
12311	45	55	70	80	50,2%	60,7%	90,2%	95,5%	89,8%
12312	45	55	75	20	38,9%	58,9%	84,5%	89,7%	78,7%
12313	45	55	75	25	39,4%	58,6%	84,9%	90,4%	79,9%
12314	45	55	75	30	39,8%	58,3%	85,3%	90,9%	80,9%
12315	45	55	75	35	40,2%	57,9%	85,8%	91,5%	82,0%
12316	45	55	75	40	40,6%	57,6%	86,2%	92,0%	83,0%
12317	45	55	75	45	41,1%	57,3%	86,6%	92,5%	83,9%
12318	45	55	75	50	41,5%	57,0%	87,0%	93,0%	84,8%
12319	45	55	75	55	41,9%	56,7%	87,3%	93,4%	85,7%
12320	45	55	75	60	42,3%	56,3%	87,7%	93,8%	86,5%
12321	45	55	75	65	42,8%	56,0%	88,1%	94,2%	87,3%
12322	45	55	75	70	43,2%	55,7%	88,4%	94,6%	88,0%
12323	45	55	75	75	43,6%	55,4%	88,8%	94,9%	88,7%
12324	45	55	75	80	44,1%	55,0%	89,1%	95,2%	89,4%
12325	45	55	80	20	33,3%	53,2%	82,8%	89,2%	78,0%
12326	45	55	80	25	33,6%	52,9%	83,2%	89,8%	79,2%
12327	45	55	80	30	34,0%	52,6%	83,7%	90,4%	80,3%
12328	45	55	80	35	34,4%	52,2%	84,2%	91,0%	81,4%
12329	45	55	80	40	34,8%	51,9%	84,6%	91,6%	82,4%
12330	45	55	80	45	35,2%	51,6%	85,1%	92,1%	83,3%
12331	45	55	80	50	35,6%	51,2%	85,5%	92,6%	84,3%
12332	45	55	80	55	36,0%	50,9%	85,9%	93,0%	85,2%
12333	45	55	80	60	36,5%	50,6%	86,3%	93,5%	86,0%
12334	45	55	80	65	36,9%	50,3%	86,7%	93,9%	86,8%
12335	45	55	80	70	37,3%	49,9%	87,1%	94,3%	87,6%
12336	45	55	80	75	37,7%	49,6%	87,5%	94,6%	88,3%
12337	45	55	80	80	38,1%	49,3%	87,8%	95,0%	89,0%
12338	45	60	20	20	91,3%	95,2%	95,4%	94,1%	84,1%
12339	45	60	20	25	91,5%	95,1%	95,5%	94,5%	85,0%
12340	45	60	20	30	91,6%	95,0%	95,7%	94,8%	85,9%
12341	45	60	20	35	91,7%	95,0%	95,8%	95,1%	86,7%

12342	45	60	20	40	91,9%	94,9%	95,9%	95,4%	87,4%
12343	45	60	20	45	92,0%	94,8%	96,1%	95,7%	88,2%
12344	45	60	20	50	92,1%	94,8%	96,2%	96,0%	88,9%
12345	45	60	20	55	92,3%	94,7%	96,3%	96,3%	89,5%
12346	45	60	20	60	92,4%	94,7%	96,4%	96,5%	90,2%
12347	45	60	20	65	92,5%	94,6%	96,6%	96,7%	90,8%
12348	45	60	20	70	92,6%	94,5%	96,7%	96,9%	91,3%
12349	45	60	20	75	92,7%	94,5%	96,8%	97,1%	91,8%
12350	45	60	20	80	92,9%	94,4%	96,9%	97,3%	92,3%
12351	45	60	25	20	89,2%	94,0%	94,8%	93,7%	83,6%
12352	45	60	25	25	89,3%	93,9%	95,0%	94,1%	84,5%
12353	45	60	25	30	89,5%	93,8%	95,1%	94,5%	85,4%
12354	45	60	25	35	89,7%	93,8%	95,3%	94,8%	86,2%
12355	45	60	25	40	89,8%	93,7%	95,4%	95,2%	87,0%
12356	45	60	25	45	90,0%	93,6%	95,6%	95,5%	87,7%
12357	45	60	25	50	90,1%	93,5%	95,7%	95,8%	88,5%
12358	45	60	25	55	90,3%	93,4%	95,9%	96,0%	89,1%
12359	45	60	25	60	90,5%	93,4%	96,0%	96,3%	89,8%
12360	45	60	25	65	90,6%	93,3%	96,1%	96,5%	90,4%
12361	45	60	25	70	90,8%	93,2%	96,2%	96,8%	91,0%
12362	45	60	25	75	90,9%	93,1%	96,4%	97,0%	91,5%
12363	45	60	25	80	91,0%	93,0%	96,5%	97,2%	92,0%
12364	45	60	30	20	86,5%	92,5%	94,2%	93,4%	83,0%
12365	45	60	30	25	86,7%	92,4%	94,3%	93,8%	83,9%
12366	45	60	30	30	86,9%	92,3%	94,5%	94,2%	84,8%
12367	45	60	30	35	87,1%	92,3%	94,7%	94,5%	85,7%
12368	45	60	30	40	87,3%	92,2%	94,9%	94,9%	86,5%
12369	45	60	30	45	87,5%	92,1%	95,0%	95,2%	87,3%
12370	45	60	30	50	87,7%	92,0%	95,2%	95,5%	88,0%
12371	45	60	30	55	87,9%	91,9%	95,3%	95,8%	88,7%
12372	45	60	30	60	88,1%	91,8%	95,5%	96,1%	89,4%
12373	45	60	30	65	88,3%	91,7%	95,6%	96,3%	90,0%
12374	45	60	30	70	88,5%	91,6%	95,8%	96,6%	90,6%

12375	45	60	30	75	88,6%	91,5%	95,9%	96,8%	91,2%
12376	45	60	30	80	88,8%	91,4%	96,0%	97,0%	91,7%
12377	45	60	35	20	83,4%	90,8%	93,4%	93,0%	82,4%
12378	45	60	35	25	83,6%	90,7%	93,6%	93,4%	83,4%
12379	45	60	35	30	83,9%	90,5%	93,8%	93,8%	84,3%
12380	45	60	35	35	84,1%	90,4%	94,0%	94,2%	85,2%
12381	45	60	35	40	84,4%	90,3%	94,2%	94,6%	86,0%
12382	45	60	35	45	84,6%	90,2%	94,4%	94,9%	86,8%
12383	45	60	35	50	84,8%	90,1%	94,6%	95,2%	87,6%
12384	45	60	35	55	85,0%	90,0%	94,7%	95,5%	88,3%
12385	45	60	35	60	85,3%	89,9%	94,9%	95,8%	89,0%
12386	45	60	35	65	85,5%	89,7%	95,1%	96,1%	89,7%
12387	45	60	35	70	85,7%	89,6%	95,2%	96,3%	90,3%
12388	45	60	35	75	85,9%	89,5%	95,4%	96,6%	90,9%
12389	45	60	35	80	86,1%	89,4%	95,5%	96,8%	91,4%
12390	45	60	40	20	79,7%	88,6%	92,6%	92,6%	81,8%
12391	45	60	40	25	80,0%	88,5%	92,9%	93,0%	82,8%
12392	45	60	40	30	80,3%	88,4%	93,1%	93,5%	83,7%
12393	45	60	40	35	80,5%	88,2%	93,3%	93,9%	84,7%
12394	45	60	40	40	80,8%	88,1%	93,5%	94,3%	85,5%
12395	45	60	40	45	81,1%	88,0%	93,7%	94,6%	86,3%
12396	45	60	40	50	81,4%	87,8%	93,9%	95,0%	87,1%
12397	45	60	40	55	81,6%	87,7%	94,1%	95,3%	87,9%
12398	45	60	40	60	81,9%	87,5%	94,3%	95,6%	88,6%
12399	45	60	40	65	82,1%	87,4%	94,5%	95,9%	89,3%
12400	45	60	40	70	82,4%	87,3%	94,6%	96,1%	89,9%
12401	45	60	40	75	82,7%	87,1%	94,8%	96,4%	90,5%
12402	45	60	40	80	82,9%	87,0%	95,0%	96,6%	91,1%
12403	45	60	45	20	75,4%	86,1%	91,7%	92,1%	81,2%
12404	45	60	45	25	75,7%	85,9%	92,0%	92,6%	82,2%
12405	45	60	45	30	76,1%	85,8%	92,2%	93,1%	83,2%
12406	45	60	45	35	76,4%	85,6%	92,5%	93,5%	84,1%
12407	45	60	45	40	76,7%	85,5%	92,7%	93,9%	85,0%

12408	45	60	45	45	77,0%	85,3%	92,9%	94,3%	85,9%
12409	45	60	45	50	77,3%	85,1%	93,2%	94,7%	86,7%
12410	45	60	45	55	77,6%	85,0%	93,4%	95,0%	87,4%
12411	45	60	45	60	77,9%	84,8%	93,6%	95,3%	88,2%
12412	45	60	45	65	78,2%	84,6%	93,8%	95,6%	88,9%
12413	45	60	45	70	78,5%	84,5%	94,0%	95,9%	89,5%
12414	45	60	45	75	78,8%	84,3%	94,2%	96,2%	90,2%
12415	45	60	45	80	79,1%	84,1%	94,3%	96,4%	90,7%
12416	45	60	50	20	70,5%	83,1%	90,7%	91,7%	80,5%
12417	45	60	50	25	70,9%	82,9%	91,0%	92,2%	81,6%
12418	45	60	50	30	71,3%	82,7%	91,3%	92,7%	82,6%
12419	45	60	50	35	71,6%	82,5%	91,6%	93,1%	83,5%
12420	45	60	50	40	72,0%	82,4%	91,8%	93,6%	84,5%
12421	45	60	50	45	72,3%	82,2%	92,1%	94,0%	85,3%
12422	45	60	50	50	72,7%	82,0%	92,3%	94,3%	86,2%
12423	45	60	50	55	73,0%	81,8%	92,6%	94,7%	87,0%
12424	45	60	50	60	73,4%	81,6%	92,8%	95,0%	87,7%
12425	45	60	50	65	73,7%	81,4%	93,0%	95,4%	88,5%
12426	45	60	50	70	74,1%	81,2%	93,2%	95,6%	89,1%
12427	45	60	50	75	74,4%	81,0%	93,4%	95,9%	89,8%
12428	45	60	50	80	74,7%	80,8%	93,6%	96,2%	90,4%
12429	45	60	55	20	65,2%	79,6%	89,6%	91,2%	79,9%
12430	45	60	55	25	65,6%	79,4%	90,0%	91,7%	81,0%
12431	45	60	55	30	66,0%	79,2%	90,3%	92,3%	82,0%
12432	45	60	55	35	66,4%	79,0%	90,6%	92,7%	83,0%
12433	45	60	55	40	66,8%	78,7%	90,8%	93,2%	83,9%
12434	45	60	55	45	67,1%	78,5%	91,1%	93,6%	84,8%
12435	45	60	55	50	67,5%	78,3%	91,4%	94,0%	85,7%
12436	45	60	55	55	67,9%	78,1%	91,7%	94,4%	86,5%
12437	45	60	55	60	68,3%	77,9%	91,9%	94,7%	87,3%
12438	45	60	55	65	68,7%	77,6%	92,2%	95,1%	88,0%
12439	45	60	55	70	69,1%	77,4%	92,4%	95,4%	88,7%
12440	45	60	55	75	69,4%	77,2%	92,6%	95,7%	89,4%

12441	45	60	55	80	69,8%	76,9%	92,9%	96,0%	90,0%
12442	45	60	60	20	59,4%	75,6%	88,4%	90,7%	79,2%
12443	45	60	60	25	59,8%	75,4%	88,8%	91,3%	80,3%
12444	45	60	60	30	60,2%	75,1%	89,1%	91,8%	81,4%
12445	45	60	60	35	60,6%	74,9%	89,4%	92,3%	82,4%
12446	45	60	60	40	61,1%	74,6%	89,8%	92,8%	83,4%
12447	45	60	60	45	61,5%	74,4%	90,1%	93,2%	84,3%
12448	45	60	60	50	61,9%	74,1%	90,4%	93,7%	85,2%
12449	45	60	60	55	62,3%	73,9%	90,7%	94,1%	86,0%
12450	45	60	60	60	62,7%	73,6%	90,9%	94,4%	86,8%
12451	45	60	60	65	63,1%	73,4%	91,2%	94,8%	87,6%
12452	45	60	60	70	63,5%	73,1%	91,5%	95,1%	88,3%
12453	45	60	60	75	64,0%	72,9%	91,7%	95,4%	89,0%
12454	45	60	60	80	64,4%	72,6%	92,0%	95,7%	89,7%
12455	45	60	65	20	53,3%	71,1%	87,1%	90,2%	78,5%
12456	45	60	65	25	53,7%	70,8%	87,5%	90,8%	79,6%
12457	45	60	65	30	54,2%	70,6%	87,8%	91,3%	80,7%
12458	45	60	65	35	54,6%	70,3%	88,2%	91,9%	81,8%
12459	45	60	65	40	55,1%	70,0%	88,6%	92,4%	82,8%
12460	45	60	65	45	55,5%	69,7%	88,9%	92,8%	83,7%
12461	45	60	65	50	55,9%	69,5%	89,2%	93,3%	84,6%
12462	45	60	65	55	56,4%	69,2%	89,5%	93,7%	85,5%
12463	45	60	65	60	56,8%	68,9%	89,9%	94,1%	86,3%
12464	45	60	65	65	57,2%	68,6%	90,2%	94,5%	87,1%
12465	45	60	65	70	57,7%	68,3%	90,5%	94,8%	87,9%
12466	45	60	65	75	58,1%	68,1%	90,7%	95,1%	88,6%
12467	45	60	65	80	58,5%	67,8%	91,0%	95,5%	89,3%
12468	45	60	70	20	47,1%	66,1%	85,6%	89,6%	77,8%
12469	45	60	70	25	47,6%	65,8%	86,1%	90,2%	79,0%
12470	45	60	70	30	48,0%	65,5%	86,5%	90,8%	80,1%
12471	45	60	70	35	48,5%	65,2%	86,8%	91,4%	81,2%
12472	45	60	70	40	48,9%	64,9%	87,2%	91,9%	82,2%
12473	45	60	70	45	49,3%	64,7%	87,6%	92,4%	83,2%

12474	45	60	70	50	49,8%	64,4%	88,0%	92,9%	84,1%
12475	45	60	70	55	50,2%	64,1%	88,3%	93,3%	85,0%
12476	45	60	70	60	50,7%	63,7%	88,7%	93,7%	85,8%
12477	45	60	70	65	51,1%	63,4%	89,0%	94,1%	86,7%
12478	45	60	70	70	51,6%	63,1%	89,3%	94,5%	87,4%
12479	45	60	70	75	52,0%	62,8%	89,7%	94,9%	88,2%
12480	45	60	70	80	52,4%	62,5%	90,0%	95,2%	88,9%
12481	45	60	75	20	41,1%	60,8%	84,0%	89,0%	77,1%
12482	45	60	75	25	41,5%	60,5%	84,5%	89,7%	78,3%
12483	45	60	75	30	41,9%	60,2%	84,9%	90,3%	79,4%
12484	45	60	75	35	42,4%	59,8%	85,4%	90,9%	80,5%
12485	45	60	75	40	42,8%	59,5%	85,8%	91,5%	81,6%
12486	45	60	75	45	43,2%	59,2%	86,2%	92,0%	82,6%
12487	45	60	75	50	43,6%	58,9%	86,6%	92,5%	83,5%
12488	45	60	75	55	44,1%	58,6%	87,0%	92,9%	84,5%
12489	45	60	75	60	44,5%	58,3%	87,4%	93,4%	85,3%
12490	45	60	75	65	45,0%	57,9%	87,7%	93,8%	86,2%
12491	45	60	75	70	45,4%	57,6%	88,1%	94,2%	87,0%
12492	45	60	75	75	45,8%	57,3%	88,4%	94,6%	87,7%
12493	45	60	75	80	46,3%	57,0%	88,8%	94,9%	88,4%
12494	45	60	80	20	35,3%	55,2%	82,3%	88,4%	76,4%
12495	45	60	80	25	35,7%	54,8%	82,8%	89,1%	77,6%
12496	45	60	80	30	36,1%	54,5%	83,3%	89,8%	78,7%
12497	45	60	80	35	36,5%	54,2%	83,7%	90,4%	79,9%
12498	45	60	80	40	36,9%	53,9%	84,2%	91,0%	80,9%
12499	45	60	80	45	37,3%	53,5%	84,6%	91,5%	82,0%
12500	45	60	80	50	37,7%	53,2%	85,1%	92,0%	83,0%
12501	45	60	80	55	38,1%	52,9%	85,5%	92,5%	83,9%
12502	45	60	80	60	38,5%	52,6%	85,9%	93,0%	84,8%
12503	45	60	80	65	39,0%	52,2%	86,3%	93,4%	85,7%
12504	45	60	80	70	39,4%	51,9%	86,7%	93,8%	86,5%
12505	45	60	80	75	39,8%	51,6%	87,1%	94,2%	87,3%
12506	45	60	80	80	40,2%	51,3%	87,5%	94,6%	88,0%

12507	45	65	20	20	92,0%	95,5%	95,2%	93,7%	82,8%
12508	45	65	20	25	92,1%	95,5%	95,4%	94,1%	83,8%
12509	45	65	20	30	92,3%	95,4%	95,5%	94,4%	84,7%
12510	45	65	20	35	92,4%	95,3%	95,7%	94,8%	85,5%
12511	45	65	20	40	92,5%	95,3%	95,8%	95,1%	86,4%
12512	45	65	20	45	92,6%	95,2%	96,0%	95,4%	87,1%
12513	45	65	20	50	92,8%	95,2%	96,1%	95,7%	87,9%
12514	45	65	20	55	92,9%	95,1%	96,2%	96,0%	88,6%
12515	45	65	20	60	93,0%	95,0%	96,3%	96,2%	89,3%
12516	45	65	20	65	93,1%	95,0%	96,4%	96,5%	89,9%
12517	45	65	20	70	93,2%	94,9%	96,6%	96,7%	90,5%
12518	45	65	20	75	93,3%	94,9%	96,7%	96,9%	91,1%
12519	45	65	20	80	93,4%	94,8%	96,8%	97,1%	91,6%
12520	45	65	25	20	90,0%	94,4%	94,6%	93,3%	82,2%
12521	45	65	25	25	90,2%	94,3%	94,8%	93,7%	83,2%
12522	45	65	25	30	90,3%	94,3%	95,0%	94,1%	84,1%
12523	45	65	25	35	90,5%	94,2%	95,1%	94,5%	85,0%
12524	45	65	25	40	90,6%	94,1%	95,3%	94,8%	85,9%
12525	45	65	25	45	90,8%	94,1%	95,4%	95,1%	86,7%
12526	45	65	25	50	90,9%	94,0%	95,6%	95,5%	87,4%
12527	45	65	25	55	91,1%	93,9%	95,7%	95,7%	88,2%
12528	45	65	25	60	91,2%	93,8%	95,9%	96,0%	88,9%
12529	45	65	25	65	91,3%	93,8%	96,0%	96,3%	89,5%
12530	45	65	25	70	91,5%	93,7%	96,1%	96,5%	90,2%
12531	45	65	25	75	91,6%	93,6%	96,3%	96,7%	90,8%
12532	45	65	25	80	91,7%	93,5%	96,4%	96,9%	91,3%
12533	45	65	30	20	87,5%	93,1%	94,0%	92,9%	81,6%
12534	45	65	30	25	87,7%	93,0%	94,2%	93,3%	82,6%
12535	45	65	30	30	87,9%	92,9%	94,4%	93,7%	83,6%
12536	45	65	30	35	88,1%	92,8%	94,5%	94,1%	84,5%
12537	45	65	30	40	88,3%	92,7%	94,7%	94,5%	85,4%
12538	45	65	30	45	88,5%	92,6%	94,9%	94,9%	86,2%
12539	45	65	30	50	88,6%	92,5%	95,0%	95,2%	87,0%

12540	45	65	30	55	88,8%	92,4%	95,2%	95,5%	87,7%
12541	45	65	30	60	89,0%	92,3%	95,3%	95,8%	88,5%
12542	45	65	30	65	89,2%	92,3%	95,5%	96,0%	89,1%
12543	45	65	30	70	89,3%	92,2%	95,6%	96,3%	89,8%
12544	45	65	30	75	89,5%	92,1%	95,8%	96,5%	90,4%
12545	45	65	30	80	89,7%	92,0%	95,9%	96,8%	91,0%
12546	45	65	35	20	84,6%	91,4%	93,2%	92,5%	81,0%
12547	45	65	35	25	84,8%	91,3%	93,4%	92,9%	82,0%
12548	45	65	35	30	85,0%	91,2%	93,7%	93,4%	83,0%
12549	45	65	35	35	85,3%	91,1%	93,9%	93,8%	83,9%
12550	45	65	35	40	85,5%	91,0%	94,0%	94,2%	84,8%
12551	45	65	35	45	85,7%	90,9%	94,2%	94,6%	85,7%
12552	45	65	35	50	85,9%	90,8%	94,4%	94,9%	86,5%
12553	45	65	35	55	86,1%	90,7%	94,6%	95,2%	87,3%
12554	45	65	35	60	86,3%	90,5%	94,8%	95,5%	88,0%
12555	45	65	35	65	86,5%	90,4%	94,9%	95,8%	88,7%
12556	45	65	35	70	86,8%	90,3%	95,1%	96,1%	89,4%
12557	45	65	35	75	87,0%	90,2%	95,2%	96,3%	90,0%
12558	45	65	35	80	87,2%	90,1%	95,4%	96,6%	90,6%
12559	45	65	40	20	81,1%	89,4%	92,4%	92,0%	80,3%
12560	45	65	40	25	81,4%	89,3%	92,6%	92,5%	81,4%
12561	45	65	40	30	81,6%	89,2%	92,9%	93,0%	82,4%
12562	45	65	40	35	81,9%	89,0%	93,1%	93,4%	83,4%
12563	45	65	40	40	82,1%	88,9%	93,3%	93,8%	84,3%
12564	45	65	40	45	82,4%	88,8%	93,5%	94,2%	85,2%
12565	45	65	40	50	82,7%	88,6%	93,7%	94,6%	86,0%
12566	45	65	40	55	82,9%	88,5%	93,9%	94,9%	86,8%
12567	45	65	40	60	83,2%	88,4%	94,1%	95,3%	87,6%
12568	45	65	40	65	83,4%	88,2%	94,3%	95,6%	88,3%
12569	45	65	40	70	83,6%	88,1%	94,5%	95,8%	89,0%
12570	45	65	40	75	83,9%	88,0%	94,6%	96,1%	89,7%
12571	45	65	40	80	84,1%	87,8%	94,8%	96,4%	90,3%
12572	45	65	45	20	77,0%	87,0%	91,5%	91,6%	79,7%

12573	45	65	45	25	77,3%	86,9%	91,8%	92,1%	80,7%
12574	45	65	45	30	77,6%	86,7%	92,0%	92,6%	81,8%
12575	45	65	45	35	77,9%	86,6%	92,3%	93,0%	82,8%
12576	45	65	45	40	78,2%	86,4%	92,5%	93,5%	83,7%
12577	45	65	45	45	78,5%	86,3%	92,7%	93,9%	84,7%
12578	45	65	45	50	78,8%	86,1%	92,9%	94,3%	85,5%
12579	45	65	45	55	79,1%	85,9%	93,2%	94,6%	86,4%
12580	45	65	45	60	79,4%	85,8%	93,4%	95,0%	87,1%
12581	45	65	45	65	79,7%	85,6%	93,6%	95,3%	87,9%
12582	45	65	45	70	80,0%	85,5%	93,8%	95,6%	88,6%
12583	45	65	45	75	80,3%	85,3%	94,0%	95,9%	89,3%
12584	45	65	45	80	80,5%	85,1%	94,2%	96,1%	89,9%
12585	45	65	50	20	72,4%	84,2%	90,5%	91,1%	79,0%
12586	45	65	50	25	72,7%	84,0%	90,8%	91,6%	80,1%
12587	45	65	50	30	73,1%	83,8%	91,0%	92,2%	81,2%
12588	45	65	50	35	73,4%	83,6%	91,3%	92,6%	82,2%
12589	45	65	50	40	73,7%	83,5%	91,6%	93,1%	83,2%
12590	45	65	50	45	74,1%	83,3%	91,8%	93,5%	84,1%
12591	45	65	50	50	74,4%	83,1%	92,1%	93,9%	85,0%
12592	45	65	50	55	74,8%	82,9%	92,3%	94,3%	85,9%
12593	45	65	50	60	75,1%	82,7%	92,6%	94,7%	86,7%
12594	45	65	50	65	75,4%	82,5%	92,8%	95,0%	87,4%
12595	45	65	50	70	75,7%	82,4%	93,0%	95,3%	88,2%
12596	45	65	50	75	76,1%	82,2%	93,2%	95,6%	88,9%
12597	45	65	50	80	76,4%	82,0%	93,5%	95,9%	89,5%
12598	45	65	55	20	67,2%	80,9%	89,3%	90,6%	78,3%
12599	45	65	55	25	67,5%	80,7%	89,7%	91,2%	79,4%
12600	45	65	55	30	67,9%	80,4%	90,0%	91,7%	80,5%
12601	45	65	55	35	68,3%	80,2%	90,3%	92,2%	81,6%
12602	45	65	55	40	68,7%	80,0%	90,6%	92,7%	82,6%
12603	45	65	55	45	69,1%	79,8%	90,9%	93,1%	83,6%
12604	45	65	55	50	69,4%	79,6%	91,1%	93,6%	84,5%
12605	45	65	55	55	69,8%	79,4%	91,4%	94,0%	85,3%

12606	45	65	55	60	70,2%	79,2%	91,7%	94,4%	86,2%
12607	45	65	55	65	70,6%	79,0%	91,9%	94,7%	87,0%
12608	45	65	55	70	70,9%	78,7%	92,2%	95,0%	87,7%
12609	45	65	55	75	71,3%	78,5%	92,4%	95,4%	88,5%
12610	45	65	55	80	71,6%	78,3%	92,6%	95,7%	89,1%
12611	45	65	60	20	61,5%	77,0%	88,1%	90,1%	77,6%
12612	45	65	60	25	61,9%	76,8%	88,5%	90,7%	78,8%
12613	45	65	60	30	62,3%	76,6%	88,8%	91,2%	79,9%
12614	45	65	60	35	62,7%	76,3%	89,1%	91,8%	81,0%
12615	45	65	60	40	63,2%	76,1%	89,5%	92,3%	82,0%
12616	45	65	60	45	63,6%	75,8%	89,8%	92,8%	83,0%
12617	45	65	60	50	64,0%	75,6%	90,1%	93,2%	83,9%
12618	45	65	60	55	64,4%	75,4%	90,4%	93,6%	84,8%
12619	45	65	60	60	64,8%	75,1%	90,7%	94,0%	85,7%
12620	45	65	60	65	65,2%	74,9%	91,0%	94,4%	86,5%
12621	45	65	60	70	65,6%	74,6%	91,2%	94,8%	87,3%
12622	45	65	60	75	66,0%	74,4%	91,5%	95,1%	88,0%
12623	45	65	60	80	66,4%	74,1%	91,8%	95,4%	88,7%
12624	45	65	65	20	55,5%	72,7%	86,7%	89,5%	76,9%
12625	45	65	65	25	55,9%	72,4%	87,1%	90,1%	78,1%
12626	45	65	65	30	56,4%	72,2%	87,5%	90,7%	79,2%
12627	45	65	65	35	56,8%	71,9%	87,9%	91,3%	80,3%
12628	45	65	65	40	57,2%	71,6%	88,2%	91,8%	81,4%
12629	45	65	65	45	57,7%	71,4%	88,6%	92,3%	82,4%
12630	45	65	65	50	58,1%	71,1%	88,9%	92,8%	83,4%
12631	45	65	65	55	58,5%	70,8%	89,2%	93,3%	84,3%
12632	45	65	65	60	59,0%	70,6%	89,6%	93,7%	85,2%
12633	45	65	65	65	59,4%	70,3%	89,9%	94,1%	86,0%
12634	45	65	65	70	59,8%	70,0%	90,2%	94,4%	86,8%
12635	45	65	65	75	60,2%	69,7%	90,5%	94,8%	87,6%
12636	45	65	65	80	60,7%	69,5%	90,8%	95,1%	88,3%
12637	45	65	70	20	49,4%	67,9%	85,2%	88,9%	76,1%
12638	45	65	70	25	49,8%	67,6%	85,7%	89,6%	77,3%

12639	45	65	70	30	50,2%	67,3%	86,1%	90,2%	78,5%
12640	45	65	70	35	50,7%	67,0%	86,5%	90,8%	79,7%
12641	45	65	70	40	51,1%	66,7%	86,9%	91,4%	80,7%
12642	45	65	70	45	51,6%	66,4%	87,3%	91,9%	81,8%
12643	45	65	70	50	52,0%	66,1%	87,6%	92,4%	82,8%
12644	45	65	70	55	52,4%	65,8%	88,0%	92,9%	83,7%
12645	45	65	70	60	52,9%	65,5%	88,3%	93,3%	84,6%
12646	45	65	70	65	53,3%	65,3%	88,7%	93,7%	85,5%
12647	45	65	70	70	53,8%	65,0%	89,0%	94,1%	86,3%
12648	45	65	70	75	54,2%	64,7%	89,4%	94,5%	87,1%
12649	45	65	70	80	54,6%	64,4%	89,7%	94,8%	87,9%
12650	45	65	75	20	43,2%	62,6%	83,6%	88,3%	75,4%
12651	45	65	75	25	43,7%	62,3%	84,1%	89,0%	76,6%
12652	45	65	75	30	44,1%	62,0%	84,5%	89,7%	77,8%
12653	45	65	75	35	44,5%	61,7%	85,0%	90,3%	79,0%
12654	45	65	75	40	45,0%	61,4%	85,4%	90,9%	80,1%
12655	45	65	75	45	45,4%	61,1%	85,8%	91,4%	81,2%
12656	45	65	75	50	45,8%	60,8%	86,2%	91,9%	82,2%
12657	45	65	75	55	46,3%	60,5%	86,6%	92,4%	83,2%
12658	45	65	75	60	46,7%	60,2%	87,0%	92,9%	84,1%
12659	45	65	75	65	47,2%	59,8%	87,4%	93,4%	85,0%
12660	45	65	75	70	47,6%	59,5%	87,8%	93,8%	85,8%
12661	45	65	75	75	48,0%	59,2%	88,1%	94,2%	86,7%
12662	45	65	75	80	48,5%	58,9%	88,5%	94,5%	87,4%
12663	45	65	80	20	37,3%	57,1%	81,8%	87,7%	74,6%
12664	45	65	80	25	37,7%	56,8%	82,3%	88,4%	75,9%
12665	45	65	80	30	38,1%	56,5%	82,8%	89,1%	77,1%
12666	45	65	80	35	38,5%	56,1%	83,3%	89,7%	78,3%
12667	45	65	80	40	39,0%	55,8%	83,8%	90,3%	79,4%
12668	45	65	80	45	39,4%	55,5%	84,2%	90,9%	80,5%
12669	45	65	80	50	39,8%	55,2%	84,7%	91,5%	81,6%
12670	45	65	80	55	40,2%	54,8%	85,1%	92,0%	82,6%
12671	45	65	80	60	40,7%	54,5%	85,5%	92,5%	83,5%

12672	45	65	80	65	41,1%	54,2%	85,9%	93,0%	84,5%
12673	45	65	80	70	41,5%	53,9%	86,4%	93,4%	85,3%
12674	45	65	80	75	41,9%	53,5%	86,7%	93,8%	86,2%
12675	45	65	80	80	42,4%	53,2%	87,1%	94,2%	87,0%
12676	45	70	20	20	92,6%	95,8%	95,1%	93,2%	81,4%
12677	45	70	20	25	92,8%	95,8%	95,2%	93,6%	82,4%
12678	45	70	20	30	92,9%	95,7%	95,4%	94,0%	83,4%
12679	45	70	20	35	93,0%	95,7%	95,5%	94,4%	84,3%
12680	45	70	20	40	93,1%	95,6%	95,7%	94,8%	85,2%
12681	45	70	20	45	93,2%	95,6%	95,8%	95,1%	86,0%
12682	45	70	20	50	93,3%	95,5%	96,0%	95,4%	86,8%
12683	45	70	20	55	93,4%	95,5%	96,1%	95,7%	87,6%
12684	45	70	20	60	93,5%	95,4%	96,2%	96,0%	88,3%
12685	45	70	20	65	93,6%	95,3%	96,3%	96,2%	89,0%
12686	45	70	20	70	93,8%	95,3%	96,5%	96,5%	89,7%
12687	45	70	20	75	93,9%	95,2%	96,6%	96,7%	90,3%
12688	45	70	20	80	94,0%	95,2%	96,7%	96,9%	90,9%
12689	45	70	25	20	90,8%	94,8%	94,5%	92,8%	80,8%
12690	45	70	25	25	90,9%	94,7%	94,7%	93,2%	81,8%
12691	45	70	25	30	91,1%	94,7%	94,8%	93,7%	82,8%
12692	45	70	25	35	91,2%	94,6%	95,0%	94,1%	83,8%
12693	45	70	25	40	91,3%	94,5%	95,1%	94,4%	84,7%
12694	45	70	25	45	91,5%	94,5%	95,3%	94,8%	85,5%
12695	45	70	25	50	91,6%	94,4%	95,5%	95,1%	86,4%
12696	45	70	25	55	91,7%	94,3%	95,6%	95,4%	87,1%
12697	45	70	25	60	91,9%	94,3%	95,7%	95,7%	87,9%
12698	45	70	25	65	92,0%	94,2%	95,9%	96,0%	88,6%
12699	45	70	25	70	92,1%	94,1%	96,0%	96,3%	89,3%
12700	45	70	25	75	92,3%	94,1%	96,1%	96,5%	89,9%
12701	45	70	25	80	92,4%	94,0%	96,3%	96,7%	90,5%
12702	45	70	30	20	88,5%	93,5%	93,8%	92,4%	80,1%
12703	45	70	30	25	88,7%	93,5%	94,0%	92,9%	81,2%
12704	45	70	30	30	88,8%	93,4%	94,2%	93,3%	82,2%

12705	45	70	30	35	89,0%	93,3%	94,4%	93,7%	83,2%
12706	45	70	30	40	89,2%	93,2%	94,5%	94,1%	84,1%
12707	45	70	30	45	89,3%	93,1%	94,7%	94,5%	85,0%
12708	45	70	30	50	89,5%	93,1%	94,9%	94,8%	85,9%
12709	45	70	30	55	89,7%	93,0%	95,0%	95,2%	86,7%
12710	45	70	30	60	89,8%	92,9%	95,2%	95,5%	87,5%
12711	45	70	30	65	90,0%	92,8%	95,4%	95,8%	88,2%
12712	45	70	30	70	90,2%	92,7%	95,5%	96,0%	88,9%
12713	45	70	30	75	90,3%	92,6%	95,6%	96,3%	89,5%
12714	45	70	30	80	90,5%	92,5%	95,8%	96,5%	90,2%
12715	45	70	35	20	85,7%	92,0%	93,0%	91,9%	79,5%
12716	45	70	35	25	85,9%	91,9%	93,2%	92,4%	80,5%
12717	45	70	35	30	86,1%	91,8%	93,5%	92,9%	81,6%
12718	45	70	35	35	86,3%	91,7%	93,7%	93,3%	82,6%
12719	45	70	35	40	86,6%	91,6%	93,9%	93,8%	83,6%
12720	45	70	35	45	86,8%	91,5%	94,1%	94,2%	84,5%
12721	45	70	35	50	87,0%	91,4%	94,2%	94,5%	85,4%
12722	45	70	35	55	87,2%	91,3%	94,4%	94,9%	86,2%
12723	45	70	35	60	87,4%	91,2%	94,6%	95,2%	87,0%
12724	45	70	35	65	87,5%	91,1%	94,8%	95,5%	87,7%
12725	45	70	35	70	87,7%	91,0%	94,9%	95,8%	88,5%
12726	45	70	35	75	87,9%	90,9%	95,1%	96,1%	89,1%
12727	45	70	35	80	88,1%	90,8%	95,3%	96,3%	89,8%
12728	45	70	40	20	82,4%	90,1%	92,2%	91,5%	78,8%
12729	45	70	40	25	82,7%	90,0%	92,4%	92,0%	79,9%
12730	45	70	40	30	82,9%	89,9%	92,7%	92,5%	81,0%
12731	45	70	40	35	83,2%	89,8%	92,9%	93,0%	82,0%
12732	45	70	40	40	83,4%	89,7%	93,1%	93,4%	83,0%
12733	45	70	40	45	83,7%	89,5%	93,3%	93,8%	83,9%
12734	45	70	40	50	83,9%	89,4%	93,5%	94,2%	84,8%
12735	45	70	40	55	84,1%	89,3%	93,7%	94,6%	85,7%
12736	45	70	40	60	84,4%	89,2%	93,9%	94,9%	86,5%
12737	45	70	40	65	84,6%	89,0%	94,1%	95,2%	87,3%

12738	45	70	40	70	84,8%	88,9%	94,3%	95,5%	88,0%
12739	45	70	40	75	85,0%	88,8%	94,5%	95,8%	88,7%
12740	45	70	40	80	85,3%	88,6%	94,7%	96,1%	89,4%
12741	45	70	45	20	78,5%	87,9%	91,2%	91,0%	78,1%
12742	45	70	45	25	78,8%	87,7%	91,5%	91,5%	79,2%
12743	45	70	45	30	79,1%	87,6%	91,8%	92,1%	80,3%
12744	45	70	45	35	79,4%	87,5%	92,0%	92,6%	81,4%
12745	45	70	45	40	79,7%	87,3%	92,3%	93,0%	82,4%
12746	45	70	45	45	80,0%	87,2%	92,5%	93,4%	83,4%
12747	45	70	45	50	80,3%	87,0%	92,7%	93,9%	84,3%
12748	45	70	45	55	80,6%	86,9%	93,0%	94,2%	85,2%
12749	45	70	45	60	80,8%	86,7%	93,2%	94,6%	86,0%
12750	45	70	45	65	81,1%	86,6%	93,4%	94,9%	86,8%
12751	45	70	45	70	81,4%	86,4%	93,6%	95,3%	87,6%
12752	45	70	45	75	81,6%	86,3%	93,8%	95,6%	88,3%
12753	45	70	45	80	81,9%	86,1%	94,0%	95,9%	89,0%
12754	45	70	50	20	74,1%	85,2%	90,2%	90,5%	77,4%
12755	45	70	50	25	74,4%	85,0%	90,5%	91,1%	78,5%
12756	45	70	50	30	74,8%	84,9%	90,8%	91,6%	79,7%
12757	45	70	50	35	75,1%	84,7%	91,1%	92,1%	80,8%
12758	45	70	50	40	75,4%	84,5%	91,3%	92,6%	81,8%
12759	45	70	50	45	75,7%	84,4%	91,6%	93,1%	82,8%
12760	45	70	50	50	76,1%	84,2%	91,9%	93,5%	83,7%
12761	45	70	50	55	76,4%	84,0%	92,1%	93,9%	84,7%
12762	45	70	50	60	76,7%	83,8%	92,3%	94,3%	85,5%
12763	45	70	50	65	77,0%	83,7%	92,6%	94,6%	86,4%
12764	45	70	50	70	77,3%	83,5%	92,8%	95,0%	87,1%
12765	45	70	50	75	77,6%	83,3%	93,0%	95,3%	87,9%
12766	45	70	50	80	77,9%	83,1%	93,3%	95,6%	88,6%
12767	45	70	55	20	69,1%	82,0%	89,0%	89,9%	76,6%
12768	45	70	55	25	69,5%	81,9%	89,4%	90,6%	77,8%
12769	45	70	55	30	69,8%	81,7%	89,7%	91,1%	79,0%
12770	45	70	55	35	70,2%	81,5%	90,0%	91,7%	80,1%

12771	45	70	55	40	70,6%	81,3%	90,3%	92,2%	81,2%
12772	45	70	55	45	70,9%	81,1%	90,6%	92,7%	82,2%
12773	45	70	55	50	71,3%	80,9%	90,9%	93,1%	83,2%
12774	45	70	55	55	71,7%	80,7%	91,2%	93,5%	84,1%
12775	45	70	55	60	72,0%	80,5%	91,4%	94,0%	85,0%
12776	45	70	55	65	72,4%	80,2%	91,7%	94,3%	85,9%
12777	45	70	55	70	72,7%	80,0%	91,9%	94,7%	86,7%
12778	45	70	55	75	73,1%	79,8%	92,2%	95,0%	87,4%
12779	45	70	55	80	73,4%	79,6%	92,4%	95,3%	88,2%
12780	45	70	60	20	63,6%	78,4%	87,8%	89,4%	75,9%
12781	45	70	60	25	64,0%	78,2%	88,1%	90,0%	77,1%
12782	45	70	60	30	64,4%	77,9%	88,5%	90,6%	78,3%
12783	45	70	60	35	64,8%	77,7%	88,8%	91,2%	79,4%
12784	45	70	60	40	65,2%	77,5%	89,2%	91,7%	80,5%
12785	45	70	60	45	65,6%	77,3%	89,5%	92,2%	81,6%
12786	45	70	60	50	66,0%	77,0%	89,8%	92,7%	82,6%
12787	45	70	60	55	66,4%	76,8%	90,1%	93,2%	83,6%
12788	45	70	60	60	66,8%	76,6%	90,4%	93,6%	84,5%
12789	45	70	60	65	67,2%	76,3%	90,7%	94,0%	85,4%
12790	45	70	60	70	67,6%	76,1%	91,0%	94,4%	86,2%
12791	45	70	60	75	67,9%	75,9%	91,2%	94,7%	87,0%
12792	45	70	60	80	68,3%	75,6%	91,5%	95,1%	87,7%
12793	45	70	65	20	57,7%	74,2%	86,4%	88,8%	75,1%
12794	45	70	65	25	58,1%	74,0%	86,8%	89,5%	76,4%
12795	45	70	65	30	58,5%	73,7%	87,1%	90,1%	77,6%
12796	45	70	65	35	59,0%	73,5%	87,5%	90,7%	78,8%
12797	45	70	65	40	59,4%	73,2%	87,9%	91,3%	79,9%
12798	45	70	65	45	59,8%	73,0%	88,2%	91,8%	81,0%
12799	45	70	65	50	60,2%	72,7%	88,6%	92,3%	82,0%
12800	45	70	65	55	60,7%	72,4%	88,9%	92,8%	83,0%
12801	45	70	65	60	61,1%	72,2%	89,3%	93,2%	83,9%
12802	45	70	65	65	61,5%	71,9%	89,6%	93,6%	84,8%
12803	45	70	65	70	61,9%	71,6%	89,9%	94,0%	85,7%

12804	45	70	65	75	62,3%	71,4%	90,2%	94,4%	86,5%
12805	45	70	65	80	62,8%	71,1%	90,5%	94,8%	87,3%
12806	45	70	70	20	51,6%	69,6%	84,8%	88,2%	74,3%
12807	45	70	70	25	52,0%	69,3%	85,3%	88,9%	75,6%
12808	45	70	70	30	52,5%	69,0%	85,7%	89,5%	76,9%
12809	45	70	70	35	52,9%	68,7%	86,1%	90,2%	78,1%
12810	45	70	70	40	53,3%	68,4%	86,5%	90,8%	79,2%
12811	45	70	70	45	53,8%	68,2%	86,9%	91,3%	80,3%
12812	45	70	70	50	54,2%	67,9%	87,3%	91,8%	81,4%
12813	45	70	70	55	54,7%	67,6%	87,7%	92,3%	82,4%
12814	45	70	70	60	55,1%	67,3%	88,0%	92,8%	83,4%
12815	45	70	70	65	55,5%	67,0%	88,4%	93,3%	84,3%
12816	45	70	70	70	56,0%	66,7%	88,7%	93,7%	85,2%
12817	45	70	70	75	56,4%	66,4%	89,0%	94,1%	86,0%
12818	45	70	70	80	56,8%	66,1%	89,4%	94,5%	86,8%
12819	45	70	75	20	45,4%	64,5%	83,2%	87,5%	73,5%
12820	45	70	75	25	45,9%	64,2%	83,6%	88,2%	74,9%
12821	45	70	75	30	46,3%	63,9%	84,1%	88,9%	76,1%
12822	45	70	75	35	46,7%	63,6%	84,5%	89,6%	77,3%
12823	45	70	75	40	47,2%	63,3%	85,0%	90,2%	78,5%
12824	45	70	75	45	47,6%	63,0%	85,4%	90,8%	79,7%
12825	45	70	75	50	48,1%	62,6%	85,8%	91,4%	80,7%
12826	45	70	75	55	48,5%	62,3%	86,2%	91,9%	81,8%
12827	45	70	75	60	48,9%	62,0%	86,6%	92,4%	82,8%
12828	45	70	75	65	49,4%	61,7%	87,0%	92,9%	83,7%
12829	45	70	75	70	49,8%	61,4%	87,4%	93,3%	84,6%
12830	45	70	75	75	50,3%	61,1%	87,8%	93,7%	85,5%
12831	45	70	75	80	50,7%	60,8%	88,1%	94,1%	86,3%
12832	45	70	80	20	39,4%	59,0%	81,4%	86,8%	72,7%
12833	45	70	80	25	39,8%	58,7%	81,9%	87,6%	74,1%
12834	45	70	80	30	40,2%	58,4%	82,4%	88,3%	75,4%
12835	45	70	80	35	40,7%	58,1%	82,9%	89,0%	76,6%
12836	45	70	80	40	41,1%	57,7%	83,3%	89,7%	77,8%

12837	45	70	80	45	41,5%	57,4%	83,8%	90,3%	79,0%
12838	45	70	80	50	41,9%	57,1%	84,3%	90,9%	80,1%
12839	45	70	80	55	42,4%	56,8%	84,7%	91,4%	81,2%
12840	45	70	80	60	42,8%	56,5%	85,1%	92,0%	82,2%
12841	45	70	80	65	43,2%	56,1%	85,6%	92,5%	83,2%
12842	45	70	80	70	43,7%	55,8%	86,0%	92,9%	84,1%
12843	45	70	80	75	44,1%	55,5%	86,4%	93,4%	85,0%
12844	45	70	80	80	44,5%	55,2%	86,8%	93,8%	85,9%
12845	45	75	20	20	93,2%	96,1%	94,9%	92,7%	79,9%
12846	45	75	20	25	93,3%	96,1%	95,1%	93,2%	81,0%
12847	45	75	20	30	93,4%	96,0%	95,3%	93,6%	82,0%
12848	45	75	20	35	93,5%	96,0%	95,4%	94,0%	83,0%
12849	45	75	20	40	93,7%	95,9%	95,6%	94,4%	84,0%
12850	45	75	20	45	93,8%	95,9%	95,7%	94,7%	84,9%
12851	45	75	20	50	93,9%	95,8%	95,8%	95,1%	85,7%
12852	45	75	20	55	94,0%	95,8%	96,0%	95,4%	86,5%
12853	45	75	20	60	94,1%	95,7%	96,1%	95,7%	87,3%
12854	45	75	20	65	94,2%	95,7%	96,2%	95,9%	88,0%
12855	45	75	20	70	94,3%	95,6%	96,3%	96,2%	88,8%
12856	45	75	20	75	94,3%	95,6%	96,5%	96,5%	89,4%
12857	45	75	20	80	94,4%	95,5%	96,6%	96,7%	90,0%
12858	45	75	25	20	91,5%	95,2%	94,3%	92,3%	79,2%
12859	45	75	25	25	91,6%	95,1%	94,5%	92,8%	80,3%
12860	45	75	25	30	91,8%	95,1%	94,7%	93,2%	81,4%
12861	45	75	25	35	91,9%	95,0%	94,8%	93,6%	82,4%
12862	45	75	25	40	92,0%	94,9%	95,0%	94,0%	83,4%
12863	45	75	25	45	92,1%	94,9%	95,2%	94,4%	84,3%
12864	45	75	25	50	92,3%	94,8%	95,3%	94,8%	85,2%
12865	45	75	25	55	92,4%	94,7%	95,5%	95,1%	86,0%
12866	45	75	25	60	92,5%	94,7%	95,6%	95,4%	86,8%
12867	45	75	25	65	92,6%	94,6%	95,7%	95,7%	87,6%
12868	45	75	25	70	92,8%	94,5%	95,9%	96,0%	88,3%
12869	45	75	25	75	92,9%	94,5%	96,0%	96,2%	89,0%

12870	45	75	25	80	93,0%	94,4%	96,1%	96,5%	89,7%
12871	45	75	30	20	89,4%	94,0%	93,6%	91,8%	78,6%
12872	45	75	30	25	89,5%	93,9%	93,8%	92,3%	79,7%
12873	45	75	30	30	89,7%	93,9%	94,0%	92,8%	80,8%
12874	45	75	30	35	89,8%	93,8%	94,2%	93,3%	81,8%
12875	45	75	30	40	90,0%	93,7%	94,4%	93,7%	82,8%
12876	45	75	30	45	90,2%	93,6%	94,6%	94,1%	83,8%
12877	45	75	30	50	90,3%	93,5%	94,7%	94,5%	84,7%
12878	45	75	30	55	90,5%	93,5%	94,9%	94,8%	85,5%
12879	45	75	30	60	90,6%	93,4%	95,1%	95,1%	86,4%
12880	45	75	30	65	90,8%	93,3%	95,2%	95,4%	87,2%
12881	45	75	30	70	90,9%	93,2%	95,4%	95,7%	87,9%
12882	45	75	30	75	91,1%	93,1%	95,5%	96,0%	88,6%
12883	45	75	30	80	91,2%	93,1%	95,7%	96,3%	89,3%
12884	45	75	35	20	86,8%	92,6%	92,8%	91,4%	77,8%
12885	45	75	35	25	87,0%	92,5%	93,0%	91,9%	79,0%
12886	45	75	35	30	87,2%	92,4%	93,3%	92,4%	80,1%
12887	45	75	35	35	87,4%	92,3%	93,5%	92,9%	81,2%
12888	45	75	35	40	87,6%	92,2%	93,7%	93,3%	82,2%
12889	45	75	35	45	87,7%	92,1%	93,9%	93,7%	83,2%
12890	45	75	35	50	87,9%	92,0%	94,1%	94,1%	84,1%
12891	45	75	35	55	88,1%	91,9%	94,3%	94,5%	85,0%
12892	45	75	35	60	88,3%	91,8%	94,4%	94,8%	85,9%
12893	45	75	35	65	88,5%	91,7%	94,6%	95,2%	86,7%
12894	45	75	35	70	88,7%	91,6%	94,8%	95,5%	87,5%
12895	45	75	35	75	88,8%	91,5%	94,9%	95,8%	88,2%
12896	45	75	35	80	89,0%	91,4%	95,1%	96,0%	88,9%
12897	45	75	40	20	83,7%	90,8%	92,0%	90,9%	77,1%
12898	45	75	40	25	83,9%	90,7%	92,2%	91,4%	78,3%
12899	45	75	40	30	84,1%	90,6%	92,4%	92,0%	79,5%
12900	45	75	40	35	84,4%	90,5%	92,7%	92,5%	80,6%
12901	45	75	40	40	84,6%	90,4%	92,9%	92,9%	81,6%
12902	45	75	40	45	84,8%	90,3%	93,1%	93,4%	82,6%

12903	45	75	40	50	85,1%	90,1%	93,3%	93,8%	83,6%
12904	45	75	40	55	85,3%	90,0%	93,5%	94,2%	84,5%
12905	45	75	40	60	85,5%	89,9%	93,7%	94,5%	85,4%
12906	45	75	40	65	85,7%	89,8%	93,9%	94,9%	86,2%
12907	45	75	40	70	85,9%	89,7%	94,1%	95,2%	87,0%
12908	45	75	40	75	86,1%	89,5%	94,3%	95,5%	87,8%
12909	45	75	40	80	86,4%	89,4%	94,5%	95,8%	88,5%
12910	45	75	45	20	80,0%	88,7%	91,0%	90,4%	76,4%
12911	45	75	45	25	80,3%	88,6%	91,3%	91,0%	77,6%
12912	45	75	45	30	80,6%	88,4%	91,5%	91,5%	78,8%
12913	45	75	45	35	80,8%	88,3%	91,8%	92,0%	79,9%
12914	45	75	45	40	81,1%	88,2%	92,0%	92,5%	81,0%
12915	45	75	45	45	81,4%	88,0%	92,3%	93,0%	82,0%
12916	45	75	45	50	81,6%	87,9%	92,5%	93,4%	83,0%
12917	45	75	45	55	81,9%	87,7%	92,8%	93,8%	83,9%
12918	45	75	45	60	82,2%	87,6%	93,0%	94,2%	84,8%
12919	45	75	45	65	82,4%	87,5%	93,2%	94,6%	85,7%
12920	45	75	45	70	82,7%	87,3%	93,4%	94,9%	86,5%
12921	45	75	45	75	82,9%	87,2%	93,6%	95,2%	87,3%
12922	45	75	45	80	83,2%	87,0%	93,8%	95,5%	88,0%
12923	45	75	50	20	75,8%	86,2%	89,9%	89,8%	75,6%
12924	45	75	50	25	76,1%	86,0%	90,2%	90,4%	76,9%
12925	45	75	50	30	76,4%	85,9%	90,5%	91,0%	78,1%
12926	45	75	50	35	76,7%	85,7%	90,8%	91,6%	79,2%
12927	45	75	50	40	77,0%	85,5%	91,1%	92,1%	80,3%
12928	45	75	50	45	77,3%	85,4%	91,3%	92,6%	81,4%
12929	45	75	50	50	77,6%	85,2%	91,6%	93,0%	82,4%
12930	45	75	50	55	78,0%	85,0%	91,9%	93,5%	83,4%
12931	45	75	50	60	78,3%	84,9%	92,1%	93,9%	84,3%
12932	45	75	50	65	78,6%	84,7%	92,4%	94,3%	85,2%
12933	45	75	50	70	78,8%	84,5%	92,6%	94,6%	86,0%
12934	45	75	50	75	79,1%	84,4%	92,8%	95,0%	86,8%
12935	45	75	50	80	79,4%	84,2%	93,1%	95,3%	87,6%

12936	45	75	55	20	70,9%	83,2%	88,7%	89,3%	74,9%
12937	45	75	55	25	71,3%	83,0%	89,1%	89,9%	76,1%
12938	45	75	55	30	71,7%	82,8%	89,4%	90,5%	77,4%
12939	45	75	55	35	72,0%	82,6%	89,7%	91,1%	78,5%
12940	45	75	55	40	72,4%	82,4%	90,0%	91,6%	79,7%
12941	45	75	55	45	72,7%	82,2%	90,3%	92,1%	80,8%
12942	45	75	55	50	73,1%	82,0%	90,6%	92,6%	81,8%
12943	45	75	55	55	73,4%	81,9%	90,9%	93,1%	82,8%
12944	45	75	55	60	73,8%	81,7%	91,2%	93,5%	83,8%
12945	45	75	55	65	74,1%	81,5%	91,4%	93,9%	84,7%
12946	45	75	55	70	74,4%	81,3%	91,7%	94,3%	85,5%
12947	45	75	55	75	74,8%	81,1%	92,0%	94,7%	86,4%
12948	45	75	55	80	75,1%	80,9%	92,2%	95,0%	87,1%
12949	45	75	60	20	65,6%	79,7%	87,4%	88,7%	74,1%
12950	45	75	60	25	66,0%	79,5%	87,8%	89,3%	75,4%
12951	45	75	60	30	66,4%	79,3%	88,1%	90,0%	76,6%
12952	45	75	60	35	66,8%	79,1%	88,5%	90,6%	77,8%
12953	45	75	60	40	67,2%	78,8%	88,8%	91,1%	79,0%
12954	45	75	60	45	67,6%	78,6%	89,2%	91,7%	80,1%
12955	45	75	60	50	68,0%	78,4%	89,5%	92,2%	81,2%
12956	45	75	60	55	68,3%	78,2%	89,8%	92,7%	82,2%
12957	45	75	60	60	68,7%	77,9%	90,1%	93,1%	83,2%
12958	45	75	60	65	69,1%	77,7%	90,4%	93,6%	84,1%
12959	45	75	60	70	69,5%	77,5%	90,7%	94,0%	85,0%
12960	45	75	60	75	69,8%	77,3%	91,0%	94,3%	85,9%
12961	45	75	60	80	70,2%	77,0%	91,3%	94,7%	86,7%
12962	45	75	65	20	59,8%	75,7%	86,0%	88,0%	73,3%
12963	45	75	65	25	60,3%	75,5%	86,4%	88,7%	74,6%
12964	45	75	65	30	60,7%	75,2%	86,8%	89,4%	75,9%
12965	45	75	65	35	61,1%	75,0%	87,2%	90,0%	77,1%
12966	45	75	65	40	61,5%	74,7%	87,5%	90,6%	78,3%
12967	45	75	65	45	61,9%	74,5%	87,9%	91,2%	79,4%
12968	45	75	65	50	62,4%	74,2%	88,3%	91,7%	80,5%

12969	45	75	65	55	62,8%	74,0%	88,6%	92,3%	81,6%
12970	45	75	65	60	63,2%	73,7%	89,0%	92,7%	82,6%
12971	45	75	65	65	63,6%	73,5%	89,3%	93,2%	83,6%
12972	45	75	65	70	64,0%	73,2%	89,6%	93,6%	84,5%
12973	45	75	65	75	64,4%	73,0%	89,9%	94,0%	85,4%
12974	45	75	65	80	64,8%	72,7%	90,2%	94,4%	86,2%
12975	45	75	70	20	53,8%	71,2%	84,4%	87,4%	72,5%
12976	45	75	70	25	54,2%	70,9%	84,9%	88,1%	73,8%
12977	45	75	70	30	54,7%	70,7%	85,3%	88,8%	75,1%
12978	45	75	70	35	55,1%	70,4%	85,7%	89,5%	76,4%
12979	45	75	70	40	55,5%	70,1%	86,1%	90,1%	77,6%
12980	45	75	70	45	56,0%	69,8%	86,5%	90,7%	78,8%
12981	45	75	70	50	56,4%	69,6%	86,9%	91,3%	79,9%
12982	45	75	70	55	56,8%	69,3%	87,3%	91,8%	81,0%
12983	45	75	70	60	57,3%	69,0%	87,7%	92,3%	82,0%
12984	45	75	70	65	57,7%	68,7%	88,0%	92,8%	83,0%
12985	45	75	70	70	58,1%	68,5%	88,4%	93,2%	83,9%
12986	45	75	70	75	58,6%	68,2%	88,7%	93,7%	84,8%
12987	45	75	70	80	59,0%	67,9%	89,1%	94,1%	85,7%
12988	45	75	75	20	47,6%	66,3%	82,7%	86,7%	71,6%
12989	45	75	75	25	48,1%	66,0%	83,2%	87,5%	73,0%
12990	45	75	75	30	48,5%	65,7%	83,7%	88,2%	74,3%
12991	45	75	75	35	48,9%	65,4%	84,1%	88,9%	75,6%
12992	45	75	75	40	49,4%	65,1%	84,6%	89,6%	76,9%
12993	45	75	75	45	49,8%	64,8%	85,0%	90,2%	78,1%
12994	45	75	75	50	50,3%	64,5%	85,4%	90,8%	79,2%
12995	45	75	75	55	50,7%	64,2%	85,9%	91,3%	80,3%
12996	45	75	75	60	51,2%	63,9%	86,3%	91,9%	81,4%
12997	45	75	75	65	51,6%	63,6%	86,7%	92,4%	82,4%
12998	45	75	75	70	52,0%	63,3%	87,1%	92,8%	83,4%
12999	45	75	75	75	52,5%	63,0%	87,4%	93,3%	84,3%
13000	45	75	75	80	52,9%	62,7%	87,8%	93,7%	85,2%
13001	45	75	80	20	41,5%	60,9%	80,9%	86,0%	70,8%

13002	45	75	80	25	42,0%	60,6%	81,4%	86,8%	72,2%
13003	45	75	80	30	42,4%	60,3%	81,9%	87,5%	73,5%
13004	45	75	80	35	42,8%	60,0%	82,4%	88,3%	74,9%
13005	45	75	80	40	43,3%	59,7%	82,9%	89,0%	76,1%
13006	45	75	80	45	43,7%	59,3%	83,4%	89,6%	77,3%
13007	45	75	80	50	44,1%	59,0%	83,8%	90,3%	78,5%
13008	45	75	80	55	44,6%	58,7%	84,3%	90,8%	79,7%
13009	45	75	80	60	45,0%	58,4%	84,7%	91,4%	80,7%
13010	45	75	80	65	45,4%	58,1%	85,2%	91,9%	81,8%
13011	45	75	80	70	45,9%	57,8%	85,6%	92,4%	82,8%
13012	45	75	80	75	46,3%	57,4%	86,0%	92,9%	83,7%
13013	45	75	80	80	46,7%	57,1%	86,4%	93,3%	84,7%
13014	45	80	20	20	93,8%	96,4%	94,8%	92,2%	78,3%
13015	45	80	20	25	93,9%	96,4%	95,0%	92,7%	79,5%
13016	45	80	20	30	94,0%	96,3%	95,1%	93,1%	80,6%
13017	45	80	20	35	94,1%	96,3%	95,3%	93,6%	81,6%
13018	45	80	20	40	94,2%	96,2%	95,4%	94,0%	82,6%
13019	45	80	20	45	94,3%	96,2%	95,6%	94,3%	83,6%
13020	45	80	20	50	94,4%	96,1%	95,7%	94,7%	84,5%
13021	45	80	20	55	94,4%	96,1%	95,8%	95,0%	85,4%
13022	45	80	20	60	94,5%	96,0%	96,0%	95,4%	86,2%
13023	45	80	20	65	94,6%	96,0%	96,1%	95,6%	87,0%
13024	45	80	20	70	94,7%	95,9%	96,2%	95,9%	87,8%
13025	45	80	20	75	94,8%	95,9%	96,4%	96,2%	88,5%
13026	45	80	20	80	94,9%	95,8%	96,5%	96,4%	89,2%
13027	45	80	25	20	92,1%	95,5%	94,1%	91,7%	77,6%
13028	45	80	25	25	92,3%	95,5%	94,3%	92,3%	78,8%
13029	45	80	25	30	92,4%	95,4%	94,5%	92,7%	79,9%
13030	45	80	25	35	92,5%	95,4%	94,7%	93,2%	81,0%
13031	45	80	25	40	92,6%	95,3%	94,8%	93,6%	82,0%
13032	45	80	25	45	92,8%	95,2%	95,0%	94,0%	83,0%
13033	45	80	25	50	92,9%	95,2%	95,2%	94,4%	84,0%
13034	45	80	25	55	93,0%	95,1%	95,3%	94,7%	84,9%

13035	45	80	25	60	93,1%	95,1%	95,5%	95,1%	85,7%
13036	45	80	25	65	93,2%	95,0%	95,6%	95,4%	86,5%
13037	45	80	25	70	93,3%	94,9%	95,8%	95,7%	87,3%
13038	45	80	25	75	93,4%	94,9%	95,9%	96,0%	88,1%
13039	45	80	25	80	93,5%	94,8%	96,0%	96,2%	88,8%
13040	45	80	30	20	90,2%	94,4%	93,4%	91,3%	76,9%
13041	45	80	30	25	90,3%	94,4%	93,6%	91,8%	78,1%
13042	45	80	30	30	90,5%	94,3%	93,8%	92,3%	79,2%
13043	45	80	30	35	90,6%	94,2%	94,0%	92,8%	80,3%
13044	45	80	30	40	90,8%	94,2%	94,2%	93,2%	81,4%
13045	45	80	30	45	90,9%	94,1%	94,4%	93,7%	82,4%
13046	45	80	30	50	91,1%	94,0%	94,6%	94,1%	83,4%
13047	45	80	30	55	91,2%	93,9%	94,7%	94,4%	84,3%
13048	45	80	30	60	91,3%	93,9%	94,9%	94,8%	85,2%
13049	45	80	30	65	91,5%	93,8%	95,1%	95,1%	86,0%
13050	45	80	30	70	91,6%	93,7%	95,2%	95,4%	86,8%
13051	45	80	30	75	91,8%	93,6%	95,4%	95,7%	87,6%
13052	45	80	30	80	91,9%	93,6%	95,5%	96,0%	88,3%
13053	45	80	35	20	87,7%	93,1%	92,6%	90,8%	76,2%
13054	45	80	35	25	87,9%	93,0%	92,8%	91,3%	77,4%
13055	45	80	35	30	88,1%	92,9%	93,1%	91,9%	78,6%
13056	45	80	35	35	88,3%	92,8%	93,3%	92,4%	79,7%
13057	45	80	35	40	88,5%	92,7%	93,5%	92,8%	80,8%
13058	45	80	35	45	88,7%	92,7%	93,7%	93,3%	81,8%
13059	45	80	35	50	88,8%	92,6%	93,9%	93,7%	82,8%
13060	45	80	35	55	89,0%	92,5%	94,1%	94,1%	83,8%
13061	45	80	35	60	89,2%	92,4%	94,3%	94,5%	84,7%
13062	45	80	35	65	89,4%	92,3%	94,4%	94,8%	85,5%
13063	45	80	35	70	89,5%	92,2%	94,6%	95,1%	86,4%
13064	45	80	35	75	89,7%	92,1%	94,8%	95,5%	87,2%
13065	45	80	35	80	89,8%	92,0%	95,0%	95,7%	87,9%
13066	45	80	40	20	84,8%	91,4%	91,7%	90,3%	75,4%
13067	45	80	40	25	85,1%	91,3%	92,0%	90,8%	76,6%

13068	45	80	40	30	85,3%	91,2%	92,2%	91,4%	77,9%
13069	45	80	40	35	85,5%	91,1%	92,5%	91,9%	79,0%
13070	45	80	40	40	85,7%	91,0%	92,7%	92,4%	80,1%
13071	45	80	40	45	85,9%	90,9%	92,9%	92,9%	81,2%
13072	45	80	40	50	86,2%	90,8%	93,1%	93,3%	82,2%
13073	45	80	40	55	86,4%	90,7%	93,3%	93,8%	83,2%
13074	45	80	40	60	86,6%	90,6%	93,6%	94,1%	84,1%
13075	45	80	40	65	86,8%	90,5%	93,8%	94,5%	85,0%
13076	45	80	40	70	87,0%	90,4%	94,0%	94,9%	85,9%
13077	45	80	40	75	87,2%	90,3%	94,1%	95,2%	86,7%
13078	45	80	40	80	87,4%	90,1%	94,3%	95,5%	87,5%
13079	45	80	45	20	81,4%	89,5%	90,7%	89,7%	74,6%
13080	45	80	45	25	81,6%	89,3%	91,0%	90,3%	75,9%
13081	45	80	45	30	81,9%	89,2%	91,3%	90,9%	77,1%
13082	45	80	45	35	82,2%	89,1%	91,5%	91,5%	78,3%
13083	45	80	45	40	82,4%	89,0%	91,8%	92,0%	79,5%
13084	45	80	45	45	82,7%	88,8%	92,1%	92,5%	80,6%
13085	45	80	45	50	82,9%	88,7%	92,3%	92,9%	81,6%
13086	45	80	45	55	83,2%	88,6%	92,5%	93,4%	82,6%
13087	45	80	45	60	83,4%	88,4%	92,8%	93,8%	83,6%
13088	45	80	45	65	83,7%	88,3%	93,0%	94,2%	84,5%
13089	45	80	45	70	83,9%	88,2%	93,2%	94,6%	85,4%
13090	45	80	45	75	84,1%	88,0%	93,4%	94,9%	86,2%
13091	45	80	45	80	84,4%	87,9%	93,6%	95,2%	87,0%
13092	45	80	50	20	77,4%	87,1%	89,6%	89,1%	73,8%
13093	45	80	50	25	77,7%	86,9%	89,9%	89,8%	75,1%
13094	45	80	50	30	78,0%	86,8%	90,2%	90,4%	76,4%
13095	45	80	50	35	78,3%	86,6%	90,5%	91,0%	77,6%
13096	45	80	50	40	78,6%	86,5%	90,8%	91,5%	78,8%
13097	45	80	50	45	78,9%	86,3%	91,1%	92,0%	79,9%
13098	45	80	50	50	79,2%	86,2%	91,4%	92,5%	81,0%
13099	45	80	50	55	79,4%	86,0%	91,6%	93,0%	82,0%
13100	45	80	50	60	79,7%	85,9%	91,9%	93,4%	83,0%

13101	45	80	50	65	80,0%	85,7%	92,1%	93,8%	83,9%
13102	45	80	50	70	80,3%	85,5%	92,4%	94,2%	84,8%
13103	45	80	50	75	80,6%	85,4%	92,6%	94,6%	85,7%
13104	45	80	50	80	80,8%	85,2%	92,8%	94,9%	86,5%
13105	45	80	55	20	72,7%	84,3%	88,4%	88,5%	73,0%
13106	45	80	55	25	73,1%	84,1%	88,7%	89,2%	74,4%
13107	45	80	55	30	73,4%	83,9%	89,1%	89,9%	75,6%
13108	45	80	55	35	73,8%	83,7%	89,4%	90,5%	76,9%
13109	45	80	55	40	74,1%	83,5%	89,7%	91,0%	78,1%
13110	45	80	55	45	74,4%	83,4%	90,0%	91,6%	79,2%
13111	45	80	55	50	74,8%	83,2%	90,3%	92,1%	80,3%
13112	45	80	55	55	75,1%	83,0%	90,6%	92,6%	81,4%
13113	45	80	55	60	75,4%	82,8%	90,9%	93,1%	82,4%
13114	45	80	55	65	75,8%	82,6%	91,2%	93,5%	83,4%
13115	45	80	55	70	76,1%	82,4%	91,5%	93,9%	84,3%
13116	45	80	55	75	76,4%	82,2%	91,7%	94,3%	85,2%
13117	45	80	55	80	76,7%	82,1%	92,0%	94,6%	86,0%
13118	45	80	60	20	67,6%	80,9%	87,1%	87,9%	72,2%
13119	45	80	60	25	68,0%	80,7%	87,4%	88,6%	73,6%
13120	45	80	60	30	68,3%	80,5%	87,8%	89,3%	74,9%
13121	45	80	60	35	68,7%	80,3%	88,2%	89,9%	76,1%
13122	45	80	60	40	69,1%	80,1%	88,5%	90,5%	77,4%
13123	45	80	60	45	69,5%	79,9%	88,9%	91,1%	78,5%
13124	45	80	60	50	69,9%	79,7%	89,2%	91,6%	79,7%
13125	45	80	60	55	70,2%	79,5%	89,5%	92,2%	80,8%
13126	45	80	60	60	70,6%	79,3%	89,8%	92,6%	81,8%
13127	45	80	60	65	71,0%	79,1%	90,1%	93,1%	82,8%
13128	45	80	60	70	71,3%	78,8%	90,4%	93,5%	83,8%
13129	45	80	60	75	71,7%	78,6%	90,7%	93,9%	84,7%
13130	45	80	60	80	72,0%	78,4%	91,0%	94,3%	85,5%
13131	45	80	65	20	62,0%	77,1%	85,6%	87,2%	71,4%
13132	45	80	65	25	62,4%	76,9%	86,0%	88,0%	72,8%
13133	45	80	65	30	62,8%	76,7%	86,4%	88,7%	74,1%

13134	45	80	65	35	63,2%	76,4%	86,8%	89,4%	75,4%
13135	45	80	65	40	63,6%	76,2%	87,2%	90,0%	76,6%
13136	45	80	65	45	64,0%	75,9%	87,6%	90,6%	77,8%
13137	45	80	65	50	64,4%	75,7%	87,9%	91,2%	79,0%
13138	45	80	65	55	64,8%	75,5%	88,3%	91,7%	80,1%
13139	45	80	65	60	65,2%	75,2%	88,6%	92,2%	81,2%
13140	45	80	65	65	65,6%	75,0%	89,0%	92,7%	82,2%
13141	45	80	65	70	66,0%	74,7%	89,3%	93,2%	83,2%
13142	45	80	65	75	66,4%	74,5%	89,6%	93,6%	84,1%
13143	45	80	65	80	66,8%	74,2%	89,9%	94,0%	85,0%
13144	45	80	70	20	56,0%	72,8%	84,0%	86,5%	70,5%
13145	45	80	70	25	56,4%	72,5%	84,5%	87,3%	71,9%
13146	45	80	70	30	56,9%	72,3%	84,9%	88,1%	73,3%
13147	45	80	70	35	57,3%	72,0%	85,3%	88,8%	74,6%
13148	45	80	70	40	57,7%	71,7%	85,7%	89,4%	75,9%
13149	45	80	70	45	58,1%	71,5%	86,2%	90,1%	77,1%
13150	45	80	70	50	58,6%	71,2%	86,6%	90,7%	78,3%
13151	45	80	70	55	59,0%	70,9%	86,9%	91,2%	79,4%
13152	45	80	70	60	59,4%	70,7%	87,3%	91,8%	80,5%
13153	45	80	70	65	59,9%	70,4%	87,7%	92,3%	81,6%
13154	45	80	70	70	60,3%	70,1%	88,1%	92,8%	82,6%
13155	45	80	70	75	60,7%	69,9%	88,4%	93,2%	83,6%
13156	45	80	70	80	61,1%	69,6%	88,8%	93,6%	84,5%
13157	45	80	75	20	49,8%	68,0%	82,3%	85,8%	69,7%
13158	45	80	75	25	50,3%	67,7%	82,8%	86,6%	71,1%
13159	45	80	75	30	50,7%	67,4%	83,2%	87,4%	72,5%
13160	45	80	75	35	51,2%	67,1%	83,7%	88,1%	73,8%
13161	45	80	75	40	51,6%	66,8%	84,2%	88,8%	75,1%
13162	45	80	75	45	52,0%	66,5%	84,6%	89,5%	76,4%
13163	45	80	75	50	52,5%	66,3%	85,0%	90,1%	77,6%
13164	45	80	75	55	52,9%	66,0%	85,5%	90,7%	78,8%
13165	45	80	75	60	53,4%	65,7%	85,9%	91,3%	79,9%
13166	45	80	75	65	53,8%	65,4%	86,3%	91,8%	81,0%

13167	45	80	75	70	54,2%	65,1%	86,7%	92,3%	82,0%
13168	45	80	75	75	54,7%	64,8%	87,1%	92,8%	83,0%
13169	45	80	75	80	55,1%	64,5%	87,5%	93,3%	83,9%
13170	45	80	80	20	43,7%	62,8%	80,4%	85,0%	68,8%
13171	45	80	80	25	44,1%	62,5%	80,9%	85,9%	70,2%
13172	45	80	80	30	44,6%	62,2%	81,4%	86,7%	71,6%
13173	45	80	80	35	45,0%	61,8%	81,9%	87,5%	73,0%
13174	45	80	80	40	45,4%	61,5%	82,4%	88,2%	74,3%
13175	45	80	80	45	45,9%	61,2%	82,9%	88,9%	75,6%
13176	45	80	80	50	46,3%	60,9%	83,4%	89,6%	76,9%
13177	45	80	80	55	46,8%	60,6%	83,9%	90,2%	78,1%
13178	45	80	80	60	47,2%	60,3%	84,3%	90,8%	79,2%
13179	45	80	80	65	47,6%	60,0%	84,8%	91,4%	80,3%
13180	45	80	80	70	48,1%	59,7%	85,2%	91,9%	81,4%
13181	45	80	80	75	48,5%	59,3%	85,6%	92,4%	82,4%
13182	45	80	80	80	49,0%	59,0%	86,0%	92,9%	83,4%
13183	50	20	20	20	86,9%	93,6%	97,6%	97,7%	93,9%
13184	50	20	20	25	87,1%	93,5%	97,7%	97,9%	94,3%
13185	50	20	20	30	87,3%	93,4%	97,7%	98,0%	94,6%
13186	50	20	20	35	87,5%	93,4%	97,8%	98,1%	95,0%
13187	50	20	20	40	87,7%	93,3%	97,9%	98,3%	95,3%
13188	50	20	20	45	87,9%	93,2%	98,0%	98,4%	95,6%
13189	50	20	20	50	88,0%	93,1%	98,0%	98,5%	95,9%
13190	50	20	20	55	88,2%	93,0%	98,1%	98,6%	96,1%
13191	50	20	20	60	88,4%	92,9%	98,2%	98,7%	96,4%
13192	50	20	20	65	88,6%	92,9%	98,2%	98,8%	96,6%
13193	50	20	20	70	88,8%	92,8%	98,3%	98,8%	96,8%
13194	50	20	20	75	88,9%	92,7%	98,3%	98,9%	97,0%
13195	50	20	20	80	89,1%	92,6%	98,4%	99,0%	97,2%
13196	50	20	25	20	83,8%	92,1%	97,3%	97,6%	93,6%
13197	50	20	25	25	84,0%	92,0%	97,4%	97,7%	94,0%
13198	50	20	25	30	84,3%	91,9%	97,5%	97,9%	94,4%
13199	50	20	25	35	84,5%	91,8%	97,5%	98,0%	94,8%

13200	50	20	25	40	84,7%	91,7%	97,6%	98,2%	95,1%
13201	50	20	25	45	85,0%	91,6%	97,7%	98,3%	95,4%
13202	50	20	25	50	85,2%	91,5%	97,8%	98,4%	95,7%
13203	50	20	25	55	85,4%	91,4%	97,8%	98,5%	96,0%
13204	50	20	25	60	85,6%	91,3%	97,9%	98,6%	96,2%
13205	50	20	25	65	85,8%	91,2%	98,0%	98,7%	96,5%
13206	50	20	25	70	86,1%	91,1%	98,0%	98,8%	96,7%
13207	50	20	25	75	86,3%	91,0%	98,1%	98,9%	96,9%
13208	50	20	25	80	86,5%	90,8%	98,2%	98,9%	97,1%
13209	50	20	30	20	80,2%	90,2%	96,9%	97,4%	93,4%
13210	50	20	30	25	80,4%	90,1%	97,0%	97,6%	93,8%
13211	50	20	30	30	80,7%	90,0%	97,1%	97,8%	94,2%
13212	50	20	30	35	81,0%	89,9%	97,2%	97,9%	94,6%
13213	50	20	30	40	81,3%	89,7%	97,3%	98,0%	94,9%
13214	50	20	30	45	81,5%	89,6%	97,4%	98,2%	95,2%
13215	50	20	30	50	81,8%	89,5%	97,5%	98,3%	95,5%
13216	50	20	30	55	82,1%	89,4%	97,6%	98,4%	95,8%
13217	50	20	30	60	82,3%	89,2%	97,6%	98,5%	96,1%
13218	50	20	30	65	82,6%	89,1%	97,7%	98,6%	96,3%
13219	50	20	30	70	82,8%	89,0%	97,8%	98,7%	96,6%
13220	50	20	30	75	83,1%	88,9%	97,9%	98,8%	96,8%
13221	50	20	30	80	83,3%	88,7%	97,9%	98,9%	97,0%
13222	50	20	35	20	75,9%	88,0%	96,5%	97,3%	93,1%
13223	50	20	35	25	76,3%	87,8%	96,7%	97,5%	93,6%
13224	50	20	35	30	76,6%	87,7%	96,8%	97,6%	94,0%
13225	50	20	35	35	76,9%	87,6%	96,9%	97,8%	94,3%
13226	50	20	35	40	77,2%	87,4%	97,0%	97,9%	94,7%
13227	50	20	35	45	77,5%	87,3%	97,1%	98,1%	95,0%
13228	50	20	35	50	77,8%	87,1%	97,2%	98,2%	95,3%
13229	50	20	35	55	78,1%	87,0%	97,3%	98,3%	95,6%
13230	50	20	35	60	78,4%	86,8%	97,3%	98,4%	95,9%
13231	50	20	35	65	78,7%	86,7%	97,4%	98,5%	96,2%
13232	50	20	35	70	79,0%	86,5%	97,5%	98,6%	96,4%

13233	50	20	35	75	79,3%	86,4%	97,6%	98,7%	96,6%
13234	50	20	35	80	79,6%	86,2%	97,7%	98,8%	96,9%
13235	50	20	40	20	71,1%	85,3%	96,1%	97,1%	92,9%
13236	50	20	40	25	71,5%	85,1%	96,2%	97,3%	93,3%
13237	50	20	40	30	71,9%	85,0%	96,3%	97,5%	93,7%
13238	50	20	40	35	72,2%	84,8%	96,5%	97,6%	94,1%
13239	50	20	40	40	72,6%	84,6%	96,6%	97,8%	94,5%
13240	50	20	40	45	72,9%	84,5%	96,7%	97,9%	94,8%
13241	50	20	40	50	73,3%	84,3%	96,8%	98,1%	95,2%
13242	50	20	40	55	73,6%	84,1%	96,9%	98,2%	95,5%
13243	50	20	40	60	74,0%	83,9%	97,0%	98,3%	95,7%
13244	50	20	40	65	74,3%	83,8%	97,1%	98,4%	96,0%
13245	50	20	40	70	74,6%	83,6%	97,2%	98,5%	96,3%
13246	50	20	40	75	75,0%	83,4%	97,3%	98,6%	96,5%
13247	50	20	40	80	75,3%	83,2%	97,4%	98,7%	96,7%
13248	50	20	45	20	65,8%	82,2%	95,6%	96,9%	92,6%
13249	50	20	45	25	66,2%	82,0%	95,7%	97,1%	93,0%
13250	50	20	45	30	66,6%	81,8%	95,9%	97,3%	93,5%
13251	50	20	45	35	67,0%	81,6%	96,0%	97,5%	93,9%
13252	50	20	45	40	67,4%	81,4%	96,1%	97,7%	94,3%
13253	50	20	45	45	67,8%	81,2%	96,3%	97,8%	94,6%
13254	50	20	45	50	68,2%	81,0%	96,4%	98,0%	95,0%
13255	50	20	45	55	68,5%	80,8%	96,5%	98,1%	95,3%
13256	50	20	45	60	68,9%	80,6%	96,6%	98,2%	95,6%
13257	50	20	45	65	69,3%	80,4%	96,7%	98,3%	95,9%
13258	50	20	45	70	69,7%	80,2%	96,8%	98,4%	96,1%
13259	50	20	45	75	70,0%	80,0%	96,9%	98,5%	96,4%
13260	50	20	45	80	70,4%	79,8%	97,0%	98,6%	96,6%
13261	50	20	50	20	60,1%	78,5%	95,1%	96,8%	92,3%
13262	50	20	50	25	60,5%	78,3%	95,2%	97,0%	92,8%
13263	50	20	50	30	60,9%	78,1%	95,4%	97,2%	93,2%
13264	50	20	50	35	61,3%	77,9%	95,5%	97,3%	93,6%
13265	50	20	50	40	61,8%	77,6%	95,7%	97,5%	94,0%

13266	50	20	50	45	62,2%	77,4%	95,8%	97,7%	94,4%
13267	50	20	50	50	62,6%	77,2%	95,9%	97,8%	94,8%
13268	50	20	50	55	63,0%	77,0%	96,1%	98,0%	95,1%
13269	50	20	50	60	63,4%	76,7%	96,2%	98,1%	95,4%
13270	50	20	50	65	63,8%	76,5%	96,3%	98,2%	95,7%
13271	50	20	50	70	64,2%	76,2%	96,4%	98,3%	96,0%
13272	50	20	50	75	64,6%	76,0%	96,5%	98,5%	96,2%
13273	50	20	50	80	65,0%	75,8%	96,7%	98,6%	96,5%
13274	50	20	55	20	54,0%	74,4%	94,4%	96,6%	92,0%
13275	50	20	55	25	54,5%	74,1%	94,6%	96,8%	92,5%
13276	50	20	55	30	54,9%	73,9%	94,8%	97,0%	93,0%
13277	50	20	55	35	55,3%	73,6%	94,9%	97,2%	93,4%
13278	50	20	55	40	55,8%	73,4%	95,1%	97,4%	93,8%
13279	50	20	55	45	56,2%	73,1%	95,3%	97,5%	94,2%
13280	50	20	55	50	56,7%	72,9%	95,4%	97,7%	94,6%
13281	50	20	55	55	57,1%	72,6%	95,6%	97,8%	94,9%
13282	50	20	55	60	57,5%	72,3%	95,7%	98,0%	95,2%
13283	50	20	55	65	57,9%	72,1%	95,8%	98,1%	95,5%
13284	50	20	55	70	58,4%	71,8%	96,0%	98,2%	95,8%
13285	50	20	55	75	58,8%	71,6%	96,1%	98,4%	96,1%
13286	50	20	55	80	59,2%	71,3%	96,2%	98,5%	96,3%
13287	50	20	60	20	47,9%	69,7%	93,7%	96,4%	91,7%
13288	50	20	60	25	48,3%	69,5%	93,9%	96,6%	92,2%
13289	50	20	60	30	48,8%	69,2%	94,1%	96,8%	92,7%
13290	50	20	60	35	49,2%	68,9%	94,3%	97,0%	93,1%
13291	50	20	60	40	49,6%	68,6%	94,5%	97,2%	93,6%
13292	50	20	60	45	50,1%	68,3%	94,7%	97,4%	94,0%
13293	50	20	60	50	50,5%	68,1%	94,8%	97,6%	94,3%
13294	50	20	60	55	51,0%	67,8%	95,0%	97,7%	94,7%
13295	50	20	60	60	51,4%	67,5%	95,2%	97,9%	95,0%
13296	50	20	60	65	51,8%	67,2%	95,3%	98,0%	95,3%
13297	50	20	60	70	52,3%	66,9%	95,5%	98,1%	95,6%
13298	50	20	60	75	52,7%	66,6%	95,6%	98,3%	95,9%

13299	50	20	60	80	53,2%	66,3%	95,7%	98,4%	96,2%
13300	50	20	65	20	41,8%	64,7%	93,0%	96,1%	91,4%
13301	50	20	65	25	42,2%	64,4%	93,2%	96,4%	91,9%
13302	50	20	65	30	42,6%	64,1%	93,4%	96,6%	92,4%
13303	50	20	65	35	43,1%	63,8%	93,6%	96,8%	92,9%
13304	50	20	65	40	43,5%	63,5%	93,8%	97,0%	93,3%
13305	50	20	65	45	43,9%	63,2%	94,0%	97,2%	93,7%
13306	50	20	65	50	44,4%	62,9%	94,2%	97,4%	94,1%
13307	50	20	65	55	44,8%	62,5%	94,4%	97,6%	94,5%
13308	50	20	65	60	45,2%	62,2%	94,6%	97,7%	94,8%
13309	50	20	65	65	45,7%	61,9%	94,7%	97,9%	95,2%
13310	50	20	65	70	46,1%	61,6%	94,9%	98,0%	95,5%
13311	50	20	65	75	46,6%	61,3%	95,1%	98,1%	95,7%
13312	50	20	65	80	47,0%	61,0%	95,2%	98,3%	96,0%
13313	50	20	70	20	35,9%	59,2%	92,1%	95,9%	91,0%
13314	50	20	70	25	36,3%	58,9%	92,4%	96,2%	91,6%
13315	50	20	70	30	36,7%	58,6%	92,6%	96,4%	92,1%
13316	50	20	70	35	37,1%	58,3%	92,8%	96,6%	92,6%
13317	50	20	70	40	37,6%	58,0%	93,1%	96,9%	93,0%
13318	50	20	70	45	38,0%	57,6%	93,3%	97,1%	93,5%
13319	50	20	70	50	38,4%	57,3%	93,5%	97,2%	93,9%
13320	50	20	70	55	38,8%	57,0%	93,7%	97,4%	94,3%
13321	50	20	70	60	39,2%	56,7%	93,9%	97,6%	94,6%
13322	50	20	70	65	39,6%	56,4%	94,1%	97,8%	95,0%
13323	50	20	70	70	40,1%	56,0%	94,3%	97,9%	95,3%
13324	50	20	70	75	40,5%	55,7%	94,4%	98,0%	95,6%
13325	50	20	70	80	40,9%	55,4%	94,6%	98,2%	95,9%
13326	50	20	75	20	30,5%	53,6%	91,2%	95,7%	90,7%
13327	50	20	75	25	30,8%	53,2%	91,4%	95,9%	91,3%
13328	50	20	75	30	31,2%	52,9%	91,7%	96,2%	91,8%
13329	50	20	75	35	31,6%	52,6%	92,0%	96,4%	92,3%
13330	50	20	75	40	32,0%	52,3%	92,2%	96,7%	92,8%
13331	50	20	75	45	32,4%	51,9%	92,4%	96,9%	93,2%

13332	50	20	75	50	32,7%	51,6%	92,7%	97,1%	93,6%
13333	50	20	75	55	33,1%	51,3%	92,9%	97,3%	94,0%
13334	50	20	75	60	33,5%	50,9%	93,1%	97,4%	94,4%
13335	50	20	75	65	33,9%	50,6%	93,3%	97,6%	94,8%
13336	50	20	75	70	34,3%	50,3%	93,5%	97,8%	95,1%
13337	50	20	75	75	34,7%	50,0%	93,8%	97,9%	95,4%
13338	50	20	75	80	35,1%	49,6%	93,9%	98,1%	95,7%
13339	50	20	80	20	25,5%	47,8%	90,1%	95,4%	90,3%
13340	50	20	80	25	25,8%	47,5%	90,4%	95,7%	90,9%
13341	50	20	80	30	26,2%	47,1%	90,7%	96,0%	91,5%
13342	50	20	80	35	26,5%	46,8%	91,0%	96,2%	92,0%
13343	50	20	80	40	26,9%	46,5%	91,3%	96,5%	92,5%
13344	50	20	80	45	27,2%	46,2%	91,5%	96,7%	92,9%
13345	50	20	80	50	27,6%	45,8%	91,8%	96,9%	93,4%
13346	50	20	80	55	27,9%	45,5%	92,0%	97,1%	93,8%
13347	50	20	80	60	28,3%	45,2%	92,3%	97,3%	94,2%
13348	50	20	80	65	28,6%	44,9%	92,5%	97,5%	94,5%
13349	50	20	80	70	29,0%	44,5%	92,8%	97,6%	94,9%
13350	50	20	80	75	29,3%	44,2%	93,0%	97,8%	95,2%
13351	50	20	80	80	29,7%	43,9%	93,2%	97,9%	95,5%
13352	50	25	20	20	87,9%	94,1%	97,5%	97,6%	93,3%
13353	50	25	20	25	88,0%	94,0%	97,6%	97,7%	93,7%
13354	50	25	20	30	88,2%	93,9%	97,7%	97,9%	94,1%
13355	50	25	20	35	88,4%	93,8%	97,7%	98,0%	94,5%
13356	50	25	20	40	88,6%	93,8%	97,8%	98,1%	94,8%
13357	50	25	20	45	88,8%	93,7%	97,9%	98,3%	95,2%
13358	50	25	20	50	88,9%	93,6%	98,0%	98,4%	95,5%
13359	50	25	20	55	89,1%	93,5%	98,0%	98,5%	95,8%
13360	50	25	20	60	89,3%	93,4%	98,1%	98,6%	96,0%
13361	50	25	20	65	89,5%	93,4%	98,2%	98,7%	96,3%
13362	50	25	20	70	89,6%	93,3%	98,2%	98,8%	96,5%
13363	50	25	20	75	89,8%	93,2%	98,3%	98,8%	96,7%
13364	50	25	20	80	89,9%	93,1%	98,3%	98,9%	96,9%

13365	50	25	25	20	85,0%	92,6%	97,2%	97,4%	93,1%
13366	50	25	25	25	85,2%	92,5%	97,3%	97,6%	93,5%
13367	50	25	25	30	85,4%	92,4%	97,4%	97,7%	93,9%
13368	50	25	25	35	85,6%	92,4%	97,5%	97,9%	94,3%
13369	50	25	25	40	85,8%	92,3%	97,5%	98,0%	94,6%
13370	50	25	25	45	86,1%	92,2%	97,6%	98,1%	95,0%
13371	50	25	25	50	86,3%	92,1%	97,7%	98,3%	95,3%
13372	50	25	25	55	86,5%	92,0%	97,8%	98,4%	95,6%
13373	50	25	25	60	86,7%	91,9%	97,8%	98,5%	95,9%
13374	50	25	25	65	86,9%	91,8%	97,9%	98,6%	96,1%
13375	50	25	25	70	87,1%	91,7%	98,0%	98,7%	96,4%
13376	50	25	25	75	87,3%	91,6%	98,1%	98,8%	96,6%
13377	50	25	25	80	87,5%	91,5%	98,1%	98,9%	96,8%
13378	50	25	30	20	81,5%	90,9%	96,8%	97,2%	92,8%
13379	50	25	30	25	81,8%	90,8%	96,9%	97,4%	93,2%
13380	50	25	30	30	82,1%	90,7%	97,0%	97,6%	93,6%
13381	50	25	30	35	82,3%	90,6%	97,1%	97,8%	94,0%
13382	50	25	30	40	82,6%	90,4%	97,2%	97,9%	94,4%
13383	50	25	30	45	82,8%	90,3%	97,3%	98,0%	94,8%
13384	50	25	30	50	83,1%	90,2%	97,4%	98,2%	95,1%
13385	50	25	30	55	83,3%	90,1%	97,5%	98,3%	95,4%
13386	50	25	30	60	83,6%	90,0%	97,6%	98,4%	95,7%
13387	50	25	30	65	83,8%	89,9%	97,6%	98,5%	96,0%
13388	50	25	30	70	84,0%	89,7%	97,7%	98,6%	96,2%
13389	50	25	30	75	84,3%	89,6%	97,8%	98,7%	96,5%
13390	50	25	30	80	84,5%	89,5%	97,9%	98,8%	96,7%
13391	50	25	35	20	77,5%	88,8%	96,4%	97,1%	92,5%
13392	50	25	35	25	77,8%	88,7%	96,5%	97,3%	93,0%
13393	50	25	35	30	78,1%	88,5%	96,7%	97,4%	93,4%
13394	50	25	35	35	78,4%	88,4%	96,8%	97,6%	93,8%
13395	50	25	35	40	78,7%	88,3%	96,9%	97,8%	94,2%
13396	50	25	35	45	79,0%	88,1%	97,0%	97,9%	94,6%
13397	50	25	35	50	79,3%	88,0%	97,1%	98,1%	94,9%

13398	50	25	35	55	79,6%	87,8%	97,2%	98,2%	95,2%
13399	50	25	35	60	79,9%	87,7%	97,3%	98,3%	95,5%
13400	50	25	35	65	80,2%	87,6%	97,3%	98,4%	95,8%
13401	50	25	35	70	80,4%	87,4%	97,4%	98,5%	96,1%
13402	50	25	35	75	80,7%	87,3%	97,5%	98,6%	96,3%
13403	50	25	35	80	81,0%	87,1%	97,6%	98,7%	96,6%
13404	50	25	40	20	72,9%	86,3%	96,0%	96,9%	92,2%
13405	50	25	40	25	73,3%	86,1%	96,1%	97,1%	92,7%
13406	50	25	40	30	73,6%	86,0%	96,2%	97,3%	93,1%
13407	50	25	40	35	74,0%	85,8%	96,4%	97,5%	93,6%
13408	50	25	40	40	74,3%	85,6%	96,5%	97,6%	94,0%
13409	50	25	40	45	74,6%	85,5%	96,6%	97,8%	94,3%
13410	50	25	40	50	75,0%	85,3%	96,7%	97,9%	94,7%
13411	50	25	40	55	75,3%	85,1%	96,8%	98,1%	95,0%
13412	50	25	40	60	75,6%	85,0%	96,9%	98,2%	95,3%
13413	50	25	40	65	76,0%	84,8%	97,0%	98,3%	95,6%
13414	50	25	40	70	76,3%	84,6%	97,1%	98,4%	95,9%
13415	50	25	40	75	76,6%	84,5%	97,2%	98,5%	96,2%
13416	50	25	40	80	76,9%	84,3%	97,3%	98,6%	96,4%
13417	50	25	45	20	67,8%	83,3%	95,5%	96,7%	91,9%
13418	50	25	45	25	68,2%	83,1%	95,6%	96,9%	92,4%
13419	50	25	45	30	68,6%	82,9%	95,8%	97,1%	92,9%
13420	50	25	45	35	68,9%	82,7%	95,9%	97,3%	93,3%
13421	50	25	45	40	69,3%	82,6%	96,0%	97,5%	93,7%
13422	50	25	45	45	69,7%	82,4%	96,2%	97,7%	94,1%
13423	50	25	45	50	70,1%	82,2%	96,3%	97,8%	94,5%
13424	50	25	45	55	70,4%	82,0%	96,4%	97,9%	94,8%
13425	50	25	45	60	70,8%	81,8%	96,5%	98,1%	95,2%
13426	50	25	45	65	71,2%	81,6%	96,6%	98,2%	95,5%
13427	50	25	45	70	71,5%	81,4%	96,7%	98,3%	95,7%
13428	50	25	45	75	71,9%	81,2%	96,8%	98,4%	96,0%
13429	50	25	45	80	72,2%	81,0%	96,9%	98,5%	96,3%
13430	50	25	50	20	62,2%	79,8%	94,9%	96,5%	91,6%

13431	50	25	50	25	62,6%	79,6%	95,1%	96,7%	92,1%
13432	50	25	50	30	63,0%	79,4%	95,2%	97,0%	92,6%
13433	50	25	50	35	63,4%	79,2%	95,4%	97,1%	93,0%
13434	50	25	50	40	63,8%	79,0%	95,5%	97,3%	93,5%
13435	50	25	50	45	64,2%	78,8%	95,7%	97,5%	93,9%
13436	50	25	50	50	64,6%	78,5%	95,8%	97,7%	94,3%
13437	50	25	50	55	65,0%	78,3%	95,9%	97,8%	94,6%
13438	50	25	50	60	65,4%	78,1%	96,1%	98,0%	95,0%
13439	50	25	50	65	65,8%	77,9%	96,2%	98,1%	95,3%
13440	50	25	50	70	66,2%	77,6%	96,3%	98,2%	95,6%
13441	50	25	50	75	66,6%	77,4%	96,4%	98,3%	95,9%
13442	50	25	50	80	67,0%	77,2%	96,5%	98,4%	96,1%
13443	50	25	55	20	56,2%	75,9%	94,3%	96,3%	91,3%
13444	50	25	55	25	56,7%	75,6%	94,4%	96,5%	91,8%
13445	50	25	55	30	57,1%	75,4%	94,6%	96,8%	92,3%
13446	50	25	55	35	57,5%	75,1%	94,8%	97,0%	92,8%
13447	50	25	55	40	58,0%	74,9%	95,0%	97,2%	93,2%
13448	50	25	55	45	58,4%	74,6%	95,1%	97,4%	93,6%
13449	50	25	55	50	58,8%	74,4%	95,3%	97,5%	94,0%
13450	50	25	55	55	59,2%	74,1%	95,4%	97,7%	94,4%
13451	50	25	55	60	59,7%	73,9%	95,6%	97,8%	94,8%
13452	50	25	55	65	60,1%	73,6%	95,7%	98,0%	95,1%
13453	50	25	55	70	60,5%	73,4%	95,8%	98,1%	95,4%
13454	50	25	55	75	60,9%	73,1%	96,0%	98,2%	95,7%
13455	50	25	55	80	61,4%	72,9%	96,1%	98,4%	96,0%
13456	50	25	60	20	50,1%	71,4%	93,6%	96,1%	90,9%
13457	50	25	60	25	50,5%	71,1%	93,8%	96,3%	91,5%
13458	50	25	60	30	51,0%	70,8%	94,0%	96,6%	92,0%
13459	50	25	60	35	51,4%	70,6%	94,1%	96,8%	92,5%
13460	50	25	60	40	51,9%	70,3%	94,3%	97,0%	93,0%
13461	50	25	60	45	52,3%	70,0%	94,5%	97,2%	93,4%
13462	50	25	60	50	52,7%	69,8%	94,7%	97,4%	93,8%
13463	50	25	60	55	53,2%	69,5%	94,8%	97,5%	94,2%

13464	50	25	60	60	53,6%	69,2%	95,0%	97,7%	94,6%
13465	50	25	60	65	54,1%	68,9%	95,2%	97,9%	94,9%
13466	50	25	60	70	54,5%	68,6%	95,3%	98,0%	95,2%
13467	50	25	60	75	54,9%	68,4%	95,5%	98,1%	95,5%
13468	50	25	60	80	55,4%	68,1%	95,6%	98,2%	95,8%
13469	50	25	65	20	44,0%	66,4%	92,8%	95,8%	90,6%
13470	50	25	65	25	44,4%	66,2%	93,0%	96,1%	91,2%
13471	50	25	65	30	44,8%	65,9%	93,2%	96,4%	91,7%
13472	50	25	65	35	45,3%	65,6%	93,4%	96,6%	92,2%
13473	50	25	65	40	45,7%	65,3%	93,6%	96,8%	92,7%
13474	50	25	65	45	46,1%	65,0%	93,8%	97,0%	93,1%
13475	50	25	65	50	46,6%	64,7%	94,0%	97,2%	93,6%
13476	50	25	65	55	47,0%	64,4%	94,2%	97,4%	94,0%
13477	50	25	65	60	47,4%	64,1%	94,4%	97,6%	94,3%
13478	50	25	65	65	47,9%	63,8%	94,6%	97,7%	94,7%
13479	50	25	65	70	48,3%	63,5%	94,7%	97,9%	95,0%
13480	50	25	65	75	48,8%	63,2%	94,9%	98,0%	95,3%
13481	50	25	65	80	49,2%	62,9%	95,1%	98,1%	95,6%
13482	50	25	70	20	38,0%	61,1%	91,9%	95,6%	90,2%
13483	50	25	70	25	38,4%	60,8%	92,1%	95,9%	90,8%
13484	50	25	70	30	38,8%	60,5%	92,4%	96,1%	91,4%
13485	50	25	70	35	39,2%	60,2%	92,6%	96,4%	91,9%
13486	50	25	70	40	39,7%	59,9%	92,8%	96,6%	92,4%
13487	50	25	70	45	40,1%	59,6%	93,1%	96,8%	92,9%
13488	50	25	70	50	40,5%	59,2%	93,3%	97,0%	93,3%
13489	50	25	70	55	40,9%	58,9%	93,5%	97,2%	93,7%
13490	50	25	70	60	41,4%	58,6%	93,7%	97,4%	94,1%
13491	50	25	70	65	41,8%	58,3%	93,9%	97,6%	94,5%
13492	50	25	70	70	42,2%	58,0%	94,1%	97,7%	94,8%
13493	50	25	70	75	42,6%	57,6%	94,3%	97,9%	95,2%
13494	50	25	70	80	43,1%	57,3%	94,5%	98,0%	95,5%
13495	50	25	75	20	32,4%	55,5%	90,9%	95,3%	89,9%
13496	50	25	75	25	32,8%	55,2%	91,2%	95,6%	90,5%

13497	50	25	75	30	33,1%	54,9%	91,5%	95,9%	91,0%
13498	50	25	75	35	33,5%	54,5%	91,7%	96,2%	91,6%
13499	50	25	75	40	33,9%	54,2%	92,0%	96,4%	92,1%
13500	50	25	75	45	34,3%	53,9%	92,2%	96,6%	92,6%
13501	50	25	75	50	34,7%	53,6%	92,5%	96,9%	93,0%
13502	50	25	75	55	35,1%	53,2%	92,7%	97,1%	93,5%
13503	50	25	75	60	35,5%	52,9%	92,9%	97,3%	93,9%
13504	50	25	75	65	35,9%	52,6%	93,1%	97,4%	94,3%
13505	50	25	75	70	36,3%	52,3%	93,4%	97,6%	94,6%
13506	50	25	75	75	36,7%	51,9%	93,6%	97,8%	95,0%
13507	50	25	75	80	37,2%	51,6%	93,8%	97,9%	95,3%
13508	50	25	80	20	27,2%	49,8%	89,8%	95,1%	89,5%
13509	50	25	80	25	27,6%	49,4%	90,1%	95,4%	90,1%
13510	50	25	80	30	27,9%	49,1%	90,4%	95,7%	90,7%
13511	50	25	80	35	28,3%	48,8%	90,7%	95,9%	91,3%
13512	50	25	80	40	28,6%	48,5%	91,0%	96,2%	91,8%
13513	50	25	80	45	29,0%	48,1%	91,3%	96,4%	92,3%
13514	50	25	80	50	29,4%	47,8%	91,5%	96,7%	92,8%
13515	50	25	80	55	29,7%	47,5%	91,8%	96,9%	93,2%
13516	50	25	80	60	30,1%	47,1%	92,1%	97,1%	93,6%
13517	50	25	80	65	30,5%	46,8%	92,3%	97,3%	94,0%
13518	50	25	80	70	30,8%	46,5%	92,5%	97,5%	94,4%
13519	50	25	80	75	31,2%	46,2%	92,8%	97,6%	94,8%
13520	50	25	80	80	31,6%	45,8%	93,0%	97,8%	95,1%
13521	50	30	20	20	88,8%	94,5%	97,4%	97,4%	92,7%
13522	50	30	20	25	88,9%	94,4%	97,5%	97,5%	93,1%
13523	50	30	20	30	89,1%	94,3%	97,6%	97,7%	93,6%
13524	50	30	20	35	89,3%	94,3%	97,7%	97,9%	94,0%
13525	50	30	20	40	89,5%	94,2%	97,8%	98,0%	94,3%
13526	50	30	20	45	89,6%	94,1%	97,8%	98,1%	94,7%
13527	50	30	20	50	89,8%	94,1%	97,9%	98,2%	95,0%
13528	50	30	20	55	89,9%	94,0%	98,0%	98,4%	95,3%
13529	50	30	20	60	90,1%	93,9%	98,0%	98,5%	95,6%

13530	50	30	20	65	90,3%	93,8%	98,1%	98,6%	95,9%
13531	50	30	20	70	90,4%	93,8%	98,2%	98,7%	96,2%
13532	50	30	20	75	90,6%	93,7%	98,2%	98,8%	96,4%
13533	50	30	20	80	90,7%	93,6%	98,3%	98,8%	96,7%
13534	50	30	25	20	86,1%	93,1%	97,1%	97,2%	92,4%
13535	50	30	25	25	86,3%	93,1%	97,2%	97,4%	92,9%
13536	50	30	25	30	86,5%	93,0%	97,3%	97,6%	93,3%
13537	50	30	25	35	86,7%	92,9%	97,4%	97,7%	93,7%
13538	50	30	25	40	86,9%	92,8%	97,5%	97,9%	94,1%
13539	50	30	25	45	87,1%	92,7%	97,5%	98,0%	94,5%
13540	50	30	25	50	87,3%	92,6%	97,6%	98,1%	94,8%
13541	50	30	25	55	87,5%	92,5%	97,7%	98,3%	95,2%
13542	50	30	25	60	87,7%	92,4%	97,8%	98,4%	95,5%
13543	50	30	25	65	87,9%	92,4%	97,9%	98,5%	95,8%
13544	50	30	25	70	88,0%	92,3%	97,9%	98,6%	96,0%
13545	50	30	25	75	88,2%	92,2%	98,0%	98,7%	96,3%
13546	50	30	25	80	88,4%	92,1%	98,1%	98,8%	96,5%
13547	50	30	30	20	82,8%	91,5%	96,7%	97,0%	92,1%
13548	50	30	30	25	83,1%	91,4%	96,8%	97,2%	92,6%
13549	50	30	30	30	83,3%	91,3%	96,9%	97,4%	93,1%
13550	50	30	30	35	83,6%	91,2%	97,0%	97,6%	93,5%
13551	50	30	30	40	83,8%	91,1%	97,1%	97,7%	93,9%
13552	50	30	30	45	84,1%	91,0%	97,2%	97,9%	94,3%
13553	50	30	30	50	84,3%	90,9%	97,3%	98,0%	94,6%
13554	50	30	30	55	84,5%	90,8%	97,4%	98,2%	95,0%
13555	50	30	30	60	84,7%	90,7%	97,5%	98,3%	95,3%
13556	50	30	30	65	85,0%	90,6%	97,6%	98,4%	95,6%
13557	50	30	30	70	85,2%	90,4%	97,7%	98,5%	95,9%
13558	50	30	30	75	85,4%	90,3%	97,7%	98,6%	96,1%
13559	50	30	30	80	85,6%	90,2%	97,8%	98,7%	96,4%
13560	50	30	35	20	79,0%	89,5%	96,3%	96,9%	91,8%
13561	50	30	35	25	79,3%	89,4%	96,4%	97,1%	92,3%
13562	50	30	35	30	79,6%	89,3%	96,6%	97,3%	92,8%

13563	50	30	35	35	79,9%	89,2%	96,7%	97,4%	93,2%
13564	50	30	35	40	80,2%	89,0%	96,8%	97,6%	93,6%
13565	50	30	35	45	80,5%	88,9%	96,9%	97,8%	94,0%
13566	50	30	35	50	80,7%	88,8%	97,0%	97,9%	94,4%
13567	50	30	35	55	81,0%	88,7%	97,1%	98,0%	94,8%
13568	50	30	35	60	81,3%	88,5%	97,2%	98,2%	95,1%
13569	50	30	35	65	81,5%	88,4%	97,3%	98,3%	95,4%
13570	50	30	35	70	81,8%	88,3%	97,4%	98,4%	95,7%
13571	50	30	35	75	82,1%	88,1%	97,4%	98,5%	96,0%
13572	50	30	35	80	82,3%	88,0%	97,5%	98,6%	96,2%
13573	50	30	40	20	74,7%	87,2%	95,9%	96,7%	91,5%
13574	50	30	40	25	75,0%	87,0%	96,0%	96,9%	92,0%
13575	50	30	40	30	75,3%	86,9%	96,1%	97,1%	92,5%
13576	50	30	40	35	75,6%	86,7%	96,2%	97,3%	93,0%
13577	50	30	40	40	76,0%	86,6%	96,4%	97,5%	93,4%
13578	50	30	40	45	76,3%	86,4%	96,5%	97,6%	93,8%
13579	50	30	40	50	76,6%	86,3%	96,6%	97,8%	94,2%
13580	50	30	40	55	76,9%	86,1%	96,7%	97,9%	94,6%
13581	50	30	40	60	77,2%	86,0%	96,8%	98,1%	94,9%
13582	50	30	40	65	77,5%	85,8%	96,9%	98,2%	95,2%
13583	50	30	40	70	77,8%	85,6%	97,0%	98,3%	95,5%
13584	50	30	40	75	78,1%	85,5%	97,1%	98,4%	95,8%
13585	50	30	40	80	78,4%	85,3%	97,2%	98,5%	96,1%
13586	50	30	45	20	69,7%	84,4%	95,3%	96,5%	91,2%
13587	50	30	45	25	70,1%	84,2%	95,5%	96,7%	91,7%
13588	50	30	45	30	70,4%	84,0%	95,6%	96,9%	92,2%
13589	50	30	45	35	70,8%	83,8%	95,8%	97,1%	92,7%
13590	50	30	45	40	71,2%	83,7%	95,9%	97,3%	93,1%
13591	50	30	45	45	71,5%	83,5%	96,0%	97,5%	93,6%
13592	50	30	45	50	71,9%	83,3%	96,2%	97,6%	94,0%
13593	50	30	45	55	72,2%	83,1%	96,3%	97,8%	94,3%
13594	50	30	45	60	72,6%	82,9%	96,4%	97,9%	94,7%
13595	50	30	45	65	72,9%	82,7%	96,5%	98,1%	95,0%

13596	50	30	45	70	73,3%	82,6%	96,6%	98,2%	95,3%
13597	50	30	45	75	73,6%	82,4%	96,7%	98,3%	95,6%
13598	50	30	45	80	74,0%	82,2%	96,8%	98,4%	95,9%
13599	50	30	50	20	64,3%	81,1%	94,7%	96,3%	90,8%
13600	50	30	50	25	64,7%	80,9%	94,9%	96,5%	91,4%
13601	50	30	50	30	65,1%	80,7%	95,1%	96,7%	91,9%
13602	50	30	50	35	65,5%	80,5%	95,2%	96,9%	92,4%
13603	50	30	50	40	65,9%	80,3%	95,4%	97,1%	92,9%
13604	50	30	50	45	66,2%	80,0%	95,5%	97,3%	93,3%
13605	50	30	50	50	66,6%	79,8%	95,7%	97,5%	93,7%
13606	50	30	50	55	67,0%	79,6%	95,8%	97,7%	94,1%
13607	50	30	50	60	67,4%	79,4%	95,9%	97,8%	94,5%
13608	50	30	50	65	67,8%	79,2%	96,1%	98,0%	94,8%
13609	50	30	50	70	68,2%	79,0%	96,2%	98,1%	95,2%
13610	50	30	50	75	68,6%	78,8%	96,3%	98,2%	95,5%
13611	50	30	50	80	69,0%	78,5%	96,4%	98,3%	95,8%
13612	50	30	55	20	58,4%	77,3%	94,1%	96,0%	90,5%
13613	50	30	55	25	58,8%	77,0%	94,3%	96,3%	91,1%
13614	50	30	55	30	59,3%	76,8%	94,5%	96,5%	91,6%
13615	50	30	55	35	59,7%	76,6%	94,6%	96,7%	92,1%
13616	50	30	55	40	60,1%	76,3%	94,8%	97,0%	92,6%
13617	50	30	55	45	60,5%	76,1%	95,0%	97,2%	93,0%
13618	50	30	55	50	60,9%	75,9%	95,1%	97,3%	93,5%
13619	50	30	55	55	61,4%	75,6%	95,3%	97,5%	93,9%
13620	50	30	55	60	61,8%	75,4%	95,4%	97,7%	94,3%
13621	50	30	55	65	62,2%	75,1%	95,6%	97,8%	94,6%
13622	50	30	55	70	62,6%	74,9%	95,7%	98,0%	95,0%
13623	50	30	55	75	63,0%	74,6%	95,9%	98,1%	95,3%
13624	50	30	55	80	63,4%	74,4%	96,0%	98,2%	95,6%
13625	50	30	60	20	52,3%	73,0%	93,4%	95,8%	90,1%
13626	50	30	60	25	52,7%	72,7%	93,6%	96,1%	90,7%
13627	50	30	60	30	53,2%	72,4%	93,8%	96,3%	91,3%
13628	50	30	60	35	53,6%	72,2%	94,0%	96,6%	91,8%

13629	50	30	60	40	54,1%	71,9%	94,2%	96,8%	92,3%
13630	50	30	60	45	54,5%	71,7%	94,3%	97,0%	92,8%
13631	50	30	60	50	54,9%	71,4%	94,5%	97,2%	93,2%
13632	50	30	60	55	55,4%	71,1%	94,7%	97,4%	93,6%
13633	50	30	60	60	55,8%	70,9%	94,9%	97,5%	94,0%
13634	50	30	60	65	56,2%	70,6%	95,0%	97,7%	94,4%
13635	50	30	60	70	56,7%	70,3%	95,2%	97,8%	94,8%
13636	50	30	60	75	57,1%	70,0%	95,3%	98,0%	95,1%
13637	50	30	60	80	57,5%	69,8%	95,5%	98,1%	95,4%
13638	50	30	65	20	46,1%	68,2%	92,5%	95,5%	89,7%
13639	50	30	65	25	46,6%	67,9%	92,8%	95,8%	90,4%
13640	50	30	65	30	47,0%	67,6%	93,0%	96,1%	90,9%
13641	50	30	65	35	47,5%	67,3%	93,2%	96,3%	91,5%
13642	50	30	65	40	47,9%	67,0%	93,4%	96,6%	92,0%
13643	50	30	65	45	48,3%	66,7%	93,6%	96,8%	92,5%
13644	50	30	65	50	48,8%	66,4%	93,8%	97,0%	93,0%
13645	50	30	65	55	49,2%	66,2%	94,0%	97,2%	93,4%
13646	50	30	65	60	49,7%	65,9%	94,2%	97,4%	93,8%
13647	50	30	65	65	50,1%	65,6%	94,4%	97,6%	94,2%
13648	50	30	65	70	50,5%	65,3%	94,6%	97,7%	94,6%
13649	50	30	65	75	51,0%	65,0%	94,8%	97,9%	94,9%
13650	50	30	65	80	51,4%	64,7%	94,9%	98,0%	95,2%
13651	50	30	70	20	40,1%	63,0%	91,6%	95,3%	89,4%
13652	50	30	70	25	40,5%	62,7%	91,9%	95,6%	90,0%
13653	50	30	70	30	40,9%	62,4%	92,2%	95,9%	90,6%
13654	50	30	70	35	41,4%	62,1%	92,4%	96,1%	91,2%
13655	50	30	70	40	41,8%	61,7%	92,6%	96,4%	91,7%
13656	50	30	70	45	42,2%	61,4%	92,9%	96,6%	92,2%
13657	50	30	70	50	42,7%	61,1%	93,1%	96,8%	92,7%
13658	50	30	70	55	43,1%	60,8%	93,3%	97,0%	93,1%
13659	50	30	70	60	43,5%	60,5%	93,5%	97,2%	93,6%
13660	50	30	70	65	44,0%	60,2%	93,7%	97,4%	94,0%
13661	50	30	70	70	44,4%	59,9%	93,9%	97,6%	94,3%

13662	50	30	70	75	44,8%	59,6%	94,1%	97,7%	94,7%
13663	50	30	70	80	45,3%	59,2%	94,3%	97,9%	95,0%
13664	50	30	75	20	34,3%	57,4%	90,6%	95,0%	89,0%
13665	50	30	75	25	34,7%	57,1%	90,9%	95,3%	89,6%
13666	50	30	75	30	35,1%	56,8%	91,2%	95,6%	90,2%
13667	50	30	75	35	35,5%	56,5%	91,5%	95,9%	90,8%
13668	50	30	75	40	35,9%	56,2%	91,7%	96,2%	91,4%
13669	50	30	75	45	36,4%	55,8%	92,0%	96,4%	91,9%
13670	50	30	75	50	36,8%	55,5%	92,2%	96,6%	92,4%
13671	50	30	75	55	37,2%	55,2%	92,5%	96,8%	92,9%
13672	50	30	75	60	37,6%	54,9%	92,7%	97,1%	93,3%
13673	50	30	75	65	38,0%	54,5%	92,9%	97,2%	93,7%
13674	50	30	75	70	38,4%	54,2%	93,2%	97,4%	94,1%
13675	50	30	75	75	38,8%	53,9%	93,4%	97,6%	94,5%
13676	50	30	75	80	39,3%	53,6%	93,6%	97,7%	94,8%
13677	50	30	80	20	29,0%	51,7%	89,5%	94,7%	88,5%
13678	50	30	80	25	29,4%	51,4%	89,9%	95,0%	89,2%
13679	50	30	80	30	29,7%	51,1%	90,2%	95,3%	89,9%
13680	50	30	80	35	30,1%	50,7%	90,5%	95,6%	90,5%
13681	50	30	80	40	30,5%	50,4%	90,7%	95,9%	91,0%
13682	50	30	80	45	30,9%	50,1%	91,0%	96,2%	91,6%
13683	50	30	80	50	31,2%	49,8%	91,3%	96,4%	92,1%
13684	50	30	80	55	31,6%	49,4%	91,6%	96,7%	92,6%
13685	50	30	80	60	32,0%	49,1%	91,8%	96,9%	93,0%
13686	50	30	80	65	32,4%	48,8%	92,1%	97,1%	93,5%
13687	50	30	80	70	32,8%	48,5%	92,3%	97,3%	93,9%
13688	50	30	80	75	33,2%	48,1%	92,6%	97,4%	94,3%
13689	50	30	80	80	33,5%	47,8%	92,8%	97,6%	94,6%
13690	50	35	20	20	89,6%	94,9%	97,4%	97,2%	92,0%
13691	50	35	20	25	89,8%	94,8%	97,4%	97,4%	92,5%
13692	50	35	20	30	89,9%	94,7%	97,5%	97,5%	93,0%
13693	50	35	20	35	90,1%	94,7%	97,6%	97,7%	93,4%
13694	50	35	20	40	90,3%	94,6%	97,7%	97,8%	93,8%

13695	50	35	20	45	90,4%	94,6%	97,8%	98,0%	94,2%
13696	50	35	20	50	90,6%	94,5%	97,8%	98,1%	94,6%
13697	50	35	20	55	90,7%	94,4%	97,9%	98,2%	94,9%
13698	50	35	20	60	90,9%	94,3%	98,0%	98,4%	95,2%
13699	50	35	20	65	91,0%	94,3%	98,0%	98,5%	95,5%
13700	50	35	20	70	91,2%	94,2%	98,1%	98,6%	95,8%
13701	50	35	20	75	91,3%	94,1%	98,2%	98,7%	96,1%
13702	50	35	20	80	91,4%	94,1%	98,2%	98,7%	96,3%
13703	50	35	25	20	87,1%	93,6%	97,0%	97,0%	91,7%
13704	50	35	25	25	87,3%	93,6%	97,1%	97,2%	92,2%
13705	50	35	25	30	87,5%	93,5%	97,2%	97,4%	92,7%
13706	50	35	25	35	87,7%	93,4%	97,3%	97,6%	93,1%
13707	50	35	25	40	87,9%	93,3%	97,4%	97,7%	93,6%
13708	50	35	25	45	88,1%	93,2%	97,5%	97,9%	94,0%
13709	50	35	25	50	88,2%	93,1%	97,6%	98,0%	94,3%
13710	50	35	25	55	88,4%	93,1%	97,6%	98,1%	94,7%
13711	50	35	25	60	88,6%	93,0%	97,7%	98,3%	95,0%
13712	50	35	25	65	88,8%	92,9%	97,8%	98,4%	95,3%
13713	50	35	25	70	89,0%	92,8%	97,9%	98,5%	95,6%
13714	50	35	25	75	89,1%	92,7%	97,9%	98,6%	95,9%
13715	50	35	25	80	89,3%	92,6%	98,0%	98,7%	96,2%
13716	50	35	30	20	84,1%	92,1%	96,6%	96,8%	91,4%
13717	50	35	30	25	84,3%	92,0%	96,7%	97,0%	91,9%
13718	50	35	30	30	84,5%	91,9%	96,8%	97,2%	92,4%
13719	50	35	30	35	84,8%	91,8%	97,0%	97,4%	92,9%
13720	50	35	30	40	85,0%	91,7%	97,0%	97,6%	93,3%
13721	50	35	30	45	85,2%	91,6%	97,1%	97,7%	93,7%
13722	50	35	30	50	85,4%	91,5%	97,2%	97,9%	94,1%
13723	50	35	30	55	85,6%	91,4%	97,3%	98,0%	94,5%
13724	50	35	30	60	85,9%	91,3%	97,4%	98,1%	94,8%
13725	50	35	30	65	86,1%	91,2%	97,5%	98,3%	95,2%
13726	50	35	30	70	86,3%	91,1%	97,6%	98,4%	95,5%
13727	50	35	30	75	86,5%	91,0%	97,7%	98,5%	95,8%

13728	50	35	30	80	86,7%	90,9%	97,7%	98,6%	96,0%
13729	50	35	35	20	80,5%	90,3%	96,2%	96,6%	91,1%
13730	50	35	35	25	80,7%	90,1%	96,3%	96,8%	91,6%
13731	50	35	35	30	81,0%	90,0%	96,4%	97,1%	92,1%
13732	50	35	35	35	81,3%	89,9%	96,6%	97,2%	92,6%
13733	50	35	35	40	81,6%	89,8%	96,7%	97,4%	93,1%
13734	50	35	35	45	81,8%	89,7%	96,8%	97,6%	93,5%
13735	50	35	35	50	82,1%	89,5%	96,9%	97,7%	93,9%
13736	50	35	35	55	82,3%	89,4%	97,0%	97,9%	94,3%
13737	50	35	35	60	82,6%	89,3%	97,1%	98,0%	94,6%
13738	50	35	35	65	82,8%	89,2%	97,2%	98,2%	95,0%
13739	50	35	35	70	83,1%	89,0%	97,3%	98,3%	95,3%
13740	50	35	35	75	83,3%	88,9%	97,4%	98,4%	95,6%
13741	50	35	35	80	83,6%	88,8%	97,4%	98,5%	95,9%
13742	50	35	40	20	76,3%	88,0%	95,7%	96,4%	90,7%
13743	50	35	40	25	76,6%	87,9%	95,9%	96,7%	91,3%
13744	50	35	40	30	76,9%	87,8%	96,0%	96,9%	91,8%
13745	50	35	40	35	77,2%	87,6%	96,1%	97,1%	92,3%
13746	50	35	40	40	77,5%	87,5%	96,2%	97,3%	92,8%
13747	50	35	40	45	77,8%	87,3%	96,4%	97,4%	93,2%
13748	50	35	40	50	78,2%	87,2%	96,5%	97,6%	93,6%
13749	50	35	40	55	78,5%	87,0%	96,6%	97,8%	94,0%
13750	50	35	40	60	78,7%	86,9%	96,7%	97,9%	94,4%
13751	50	35	40	65	79,0%	86,7%	96,8%	98,0%	94,8%
13752	50	35	40	70	79,3%	86,6%	96,9%	98,2%	95,1%
13753	50	35	40	75	79,6%	86,4%	97,0%	98,3%	95,4%
13754	50	35	40	80	79,9%	86,3%	97,1%	98,4%	95,7%
13755	50	35	45	20	71,5%	85,4%	95,2%	96,2%	90,4%
13756	50	35	45	25	71,9%	85,2%	95,3%	96,5%	90,9%
13757	50	35	45	30	72,3%	85,0%	95,5%	96,7%	91,5%
13758	50	35	45	35	72,6%	84,9%	95,6%	96,9%	92,0%
13759	50	35	45	40	73,0%	84,7%	95,8%	97,1%	92,5%
13760	50	35	45	45	73,3%	84,5%	95,9%	97,3%	93,0%

13761	50	35	45	50	73,6%	84,4%	96,0%	97,5%	93,4%
13762	50	35	45	55	74,0%	84,2%	96,2%	97,6%	93,8%
13763	50	35	45	60	74,3%	84,0%	96,3%	97,8%	94,2%
13764	50	35	45	65	74,7%	83,8%	96,4%	97,9%	94,6%
13765	50	35	45	70	75,0%	83,7%	96,5%	98,1%	94,9%
13766	50	35	45	75	75,3%	83,5%	96,6%	98,2%	95,2%
13767	50	35	45	80	75,6%	83,3%	96,7%	98,3%	95,5%
13768	50	35	50	20	66,3%	82,3%	94,6%	96,0%	90,0%
13769	50	35	50	25	66,7%	82,1%	94,8%	96,2%	90,6%
13770	50	35	50	30	67,0%	81,9%	94,9%	96,5%	91,2%
13771	50	35	50	35	67,4%	81,7%	95,1%	96,7%	91,7%
13772	50	35	50	40	67,8%	81,5%	95,2%	96,9%	92,2%
13773	50	35	50	45	68,2%	81,3%	95,4%	97,1%	92,7%
13774	50	35	50	50	68,6%	81,1%	95,5%	97,3%	93,1%
13775	50	35	50	55	69,0%	80,9%	95,7%	97,5%	93,6%
13776	50	35	50	60	69,3%	80,7%	95,8%	97,6%	94,0%
13777	50	35	50	65	69,7%	80,5%	96,0%	97,8%	94,3%
13778	50	35	50	70	70,1%	80,3%	96,1%	97,9%	94,7%
13779	50	35	50	75	70,5%	80,1%	96,2%	98,1%	95,0%
13780	50	35	50	80	70,8%	79,8%	96,3%	98,2%	95,3%
13781	50	35	55	20	60,5%	78,6%	93,9%	95,7%	89,6%
13782	50	35	55	25	61,0%	78,4%	94,1%	96,0%	90,2%
13783	50	35	55	30	61,4%	78,2%	94,3%	96,3%	90,8%
13784	50	35	55	35	61,8%	78,0%	94,5%	96,5%	91,4%
13785	50	35	55	40	62,2%	77,7%	94,6%	96,7%	91,9%
13786	50	35	55	45	62,6%	77,5%	94,8%	96,9%	92,4%
13787	50	35	55	50	63,0%	77,3%	95,0%	97,1%	92,9%
13788	50	35	55	55	63,4%	77,0%	95,1%	97,3%	93,3%
13789	50	35	55	60	63,9%	76,8%	95,3%	97,5%	93,7%
13790	50	35	55	65	64,3%	76,6%	95,4%	97,7%	94,1%
13791	50	35	55	70	64,7%	76,3%	95,6%	97,8%	94,5%
13792	50	35	55	75	65,1%	76,1%	95,7%	98,0%	94,8%
13793	50	35	55	80	65,5%	75,9%	95,9%	98,1%	95,2%

13794	50	35	60	20	54,5%	74,5%	93,2%	95,5%	89,2%
13795	50	35	60	25	55,0%	74,2%	93,4%	95,8%	89,9%
13796	50	35	60	30	55,4%	74,0%	93,6%	96,0%	90,5%
13797	50	35	60	35	55,8%	73,7%	93,8%	96,3%	91,1%
13798	50	35	60	40	56,3%	73,5%	94,0%	96,5%	91,6%
13799	50	35	60	45	56,7%	73,2%	94,2%	96,8%	92,1%
13800	50	35	60	50	57,1%	73,0%	94,4%	97,0%	92,6%
13801	50	35	60	55	57,6%	72,7%	94,5%	97,2%	93,1%
13802	50	35	60	60	58,0%	72,5%	94,7%	97,3%	93,5%
13803	50	35	60	65	58,4%	72,2%	94,9%	97,5%	93,9%
13804	50	35	60	70	58,8%	71,9%	95,0%	97,7%	94,3%
13805	50	35	60	75	59,3%	71,7%	95,2%	97,8%	94,6%
13806	50	35	60	80	59,7%	71,4%	95,3%	98,0%	95,0%
13807	50	35	65	20	48,4%	69,9%	92,3%	95,2%	88,8%
13808	50	35	65	25	48,8%	69,6%	92,6%	95,5%	89,5%
13809	50	35	65	30	49,2%	69,3%	92,8%	95,8%	90,1%
13810	50	35	65	35	49,7%	69,0%	93,0%	96,1%	90,7%
13811	50	35	65	40	50,1%	68,7%	93,2%	96,3%	91,3%
13812	50	35	65	45	50,6%	68,5%	93,4%	96,6%	91,8%
13813	50	35	65	50	51,0%	68,2%	93,7%	96,8%	92,3%
13814	50	35	65	55	51,4%	67,9%	93,9%	97,0%	92,8%
13815	50	35	65	60	51,9%	67,6%	94,0%	97,2%	93,2%
13816	50	35	65	65	52,3%	67,3%	94,2%	97,4%	93,6%
13817	50	35	65	70	52,8%	67,0%	94,4%	97,5%	94,0%
13818	50	35	65	75	53,2%	66,7%	94,6%	97,7%	94,4%
13819	50	35	65	80	53,6%	66,5%	94,8%	97,8%	94,8%
13820	50	35	70	20	42,2%	64,8%	91,4%	94,9%	88,4%
13821	50	35	70	25	42,7%	64,5%	91,7%	95,2%	89,1%
13822	50	35	70	30	43,1%	64,2%	91,9%	95,6%	89,7%
13823	50	35	70	35	43,5%	63,9%	92,2%	95,8%	90,4%
13824	50	35	70	40	44,0%	63,6%	92,4%	96,1%	90,9%
13825	50	35	70	45	44,4%	63,3%	92,6%	96,4%	91,5%
13826	50	35	70	50	44,8%	63,0%	92,9%	96,6%	92,0%

13827	50	35	70	55	45,3%	62,7%	93,1%	96,8%	92,5%
13828	50	35	70	60	45,7%	62,4%	93,3%	97,0%	93,0%
13829	50	35	70	65	46,2%	62,1%	93,5%	97,2%	93,4%
13830	50	35	70	70	46,6%	61,7%	93,7%	97,4%	93,8%
13831	50	35	70	75	47,0%	61,4%	93,9%	97,6%	94,2%
13832	50	35	70	80	47,5%	61,1%	94,1%	97,7%	94,6%
13833	50	35	75	20	36,4%	59,4%	90,4%	94,6%	88,0%
13834	50	35	75	25	36,8%	59,0%	90,7%	95,0%	88,7%
13835	50	35	75	30	37,2%	58,7%	90,9%	95,3%	89,4%
13836	50	35	75	35	37,6%	58,4%	91,2%	95,6%	90,0%
13837	50	35	75	40	38,0%	58,1%	91,5%	95,9%	90,6%
13838	50	35	75	45	38,4%	57,8%	91,8%	96,1%	91,2%
13839	50	35	75	50	38,8%	57,5%	92,0%	96,4%	91,7%
13840	50	35	75	55	39,3%	57,1%	92,3%	96,6%	92,2%
13841	50	35	75	60	39,7%	56,8%	92,5%	96,8%	92,7%
13842	50	35	75	65	40,1%	56,5%	92,7%	97,0%	93,1%
13843	50	35	75	70	40,5%	56,2%	92,9%	97,2%	93,6%
13844	50	35	75	75	41,0%	55,8%	93,2%	97,4%	94,0%
13845	50	35	75	80	41,4%	55,5%	93,4%	97,6%	94,3%
13846	50	35	80	20	30,9%	53,7%	89,2%	94,3%	87,5%
13847	50	35	80	25	31,2%	53,4%	89,6%	94,7%	88,3%
13848	50	35	80	30	31,6%	53,0%	89,9%	95,0%	89,0%
13849	50	35	80	35	32,0%	52,7%	90,2%	95,3%	89,6%
13850	50	35	80	40	32,4%	52,4%	90,5%	95,6%	90,2%
13851	50	35	80	45	32,8%	52,1%	90,8%	95,9%	90,8%
13852	50	35	80	50	33,2%	51,7%	91,0%	96,2%	91,4%
13853	50	35	80	55	33,6%	51,4%	91,3%	96,4%	91,9%
13854	50	35	80	60	34,0%	51,1%	91,6%	96,6%	92,4%
13855	50	35	80	65	34,3%	50,8%	91,8%	96,9%	92,9%
13856	50	35	80	70	34,7%	50,4%	92,1%	97,1%	93,3%
13857	50	35	80	75	35,1%	50,1%	92,3%	97,3%	93,7%
13858	50	35	80	80	35,6%	49,8%	92,6%	97,4%	94,1%
13859	50	40	20	20	90,4%	95,2%	97,3%	97,0%	91,3%

13860	50	40	20	25	90,6%	95,2%	97,4%	97,2%	91,8%
13861	50	40	20	30	90,7%	95,1%	97,4%	97,3%	92,3%
13862	50	40	20	35	90,9%	95,1%	97,5%	97,5%	92,8%
13863	50	40	20	40	91,0%	95,0%	97,6%	97,7%	93,2%
13864	50	40	20	45	91,2%	94,9%	97,7%	97,8%	93,7%
13865	50	40	20	50	91,3%	94,9%	97,8%	98,0%	94,0%
13866	50	40	20	55	91,4%	94,8%	97,8%	98,1%	94,4%
13867	50	40	20	60	91,6%	94,8%	97,9%	98,2%	94,8%
13868	50	40	20	65	91,7%	94,7%	98,0%	98,3%	95,1%
13869	50	40	20	70	91,8%	94,6%	98,0%	98,5%	95,4%
13870	50	40	20	75	92,0%	94,6%	98,1%	98,6%	95,7%
13871	50	40	20	80	92,1%	94,5%	98,2%	98,7%	96,0%
13872	50	40	25	20	88,1%	94,1%	96,9%	96,8%	91,0%
13873	50	40	25	25	88,2%	94,0%	97,0%	97,0%	91,5%
13874	50	40	25	30	88,4%	93,9%	97,1%	97,2%	92,0%
13875	50	40	25	35	88,6%	93,9%	97,2%	97,4%	92,5%
13876	50	40	25	40	88,8%	93,8%	97,3%	97,5%	93,0%
13877	50	40	25	45	89,0%	93,7%	97,4%	97,7%	93,4%
13878	50	40	25	50	89,1%	93,6%	97,5%	97,8%	93,8%
13879	50	40	25	55	89,3%	93,6%	97,6%	98,0%	94,2%
13880	50	40	25	60	89,5%	93,5%	97,6%	98,1%	94,6%
13881	50	40	25	65	89,6%	93,4%	97,7%	98,2%	94,9%
13882	50	40	25	70	89,8%	93,3%	97,8%	98,4%	95,2%
13883	50	40	25	75	90,0%	93,2%	97,9%	98,5%	95,5%
13884	50	40	25	80	90,1%	93,1%	97,9%	98,6%	95,8%
13885	50	40	30	20	85,2%	92,7%	96,5%	96,6%	90,6%
13886	50	40	30	25	85,4%	92,6%	96,6%	96,8%	91,2%
13887	50	40	30	30	85,7%	92,5%	96,7%	97,0%	91,7%
13888	50	40	30	35	85,9%	92,4%	96,9%	97,2%	92,2%
13889	50	40	30	40	86,1%	92,3%	97,0%	97,4%	92,7%
13890	50	40	30	45	86,3%	92,2%	97,1%	97,6%	93,1%
13891	50	40	30	50	86,5%	92,1%	97,2%	97,7%	93,6%
13892	50	40	30	55	86,7%	92,0%	97,2%	97,9%	94,0%

13893	50	40	30	60	86,9%	91,9%	97,3%	98,0%	94,3%
13894	50	40	30	65	87,1%	91,8%	97,4%	98,1%	94,7%
13895	50	40	30	70	87,3%	91,7%	97,5%	98,3%	95,0%
13896	50	40	30	75	87,5%	91,6%	97,6%	98,4%	95,3%
13897	50	40	30	80	87,7%	91,5%	97,7%	98,5%	95,6%
13898	50	40	35	20	81,8%	90,9%	96,1%	96,4%	90,3%
13899	50	40	35	25	82,1%	90,8%	96,2%	96,6%	90,8%
13900	50	40	35	30	82,3%	90,7%	96,3%	96,8%	91,4%
13901	50	40	35	35	82,6%	90,6%	96,5%	97,0%	91,9%
13902	50	40	35	40	82,8%	90,5%	96,6%	97,2%	92,4%
13903	50	40	35	45	83,1%	90,4%	96,7%	97,4%	92,9%
13904	50	40	35	50	83,3%	90,3%	96,8%	97,6%	93,3%
13905	50	40	35	55	83,6%	90,1%	96,9%	97,7%	93,7%
13906	50	40	35	60	83,8%	90,0%	97,0%	97,9%	94,1%
13907	50	40	35	65	84,1%	89,9%	97,1%	98,0%	94,5%
13908	50	40	35	70	84,3%	89,8%	97,2%	98,2%	94,8%
13909	50	40	35	75	84,5%	89,7%	97,3%	98,3%	95,2%
13910	50	40	35	80	84,8%	89,5%	97,4%	98,4%	95,5%
13911	50	40	40	20	77,9%	88,8%	95,6%	96,2%	89,9%
13912	50	40	40	25	78,2%	88,7%	95,7%	96,4%	90,5%
13913	50	40	40	30	78,5%	88,6%	95,9%	96,6%	91,1%
13914	50	40	40	35	78,8%	88,4%	96,0%	96,9%	91,6%
13915	50	40	40	40	79,1%	88,3%	96,1%	97,1%	92,1%
13916	50	40	40	45	79,3%	88,2%	96,3%	97,3%	92,6%
13917	50	40	40	50	79,6%	88,0%	96,4%	97,4%	93,1%
13918	50	40	40	55	79,9%	87,9%	96,5%	97,6%	93,5%
13919	50	40	40	60	80,2%	87,8%	96,6%	97,8%	93,9%
13920	50	40	40	65	80,5%	87,6%	96,7%	97,9%	94,3%
13921	50	40	40	70	80,7%	87,5%	96,8%	98,0%	94,6%
13922	50	40	40	75	81,0%	87,3%	96,9%	98,2%	95,0%
13923	50	40	40	80	81,3%	87,2%	97,0%	98,3%	95,3%
13924	50	40	45	20	73,3%	86,3%	95,0%	95,9%	89,5%
13925	50	40	45	25	73,7%	86,2%	95,2%	96,2%	90,1%

13926	50	40	45	30	74,0%	86,0%	95,4%	96,4%	90,7%
13927	50	40	45	35	74,3%	85,9%	95,5%	96,7%	91,3%
13928	50	40	45	40	74,7%	85,7%	95,6%	96,9%	91,8%
13929	50	40	45	45	75,0%	85,5%	95,8%	97,1%	92,3%
13930	50	40	45	50	75,3%	85,4%	95,9%	97,3%	92,8%
13931	50	40	45	55	75,7%	85,2%	96,0%	97,4%	93,2%
13932	50	40	45	60	76,0%	85,0%	96,2%	97,6%	93,7%
13933	50	40	45	65	76,3%	84,9%	96,3%	97,8%	94,0%
13934	50	40	45	70	76,6%	84,7%	96,4%	97,9%	94,4%
13935	50	40	45	75	76,9%	84,5%	96,5%	98,1%	94,8%
13936	50	40	45	80	77,2%	84,4%	96,6%	98,2%	95,1%
13937	50	40	50	20	68,2%	83,4%	94,4%	95,7%	89,1%
13938	50	40	50	25	68,6%	83,2%	94,6%	96,0%	89,8%
13939	50	40	50	30	69,0%	83,0%	94,8%	96,2%	90,4%
13940	50	40	50	35	69,4%	82,8%	94,9%	96,5%	90,9%
13941	50	40	50	40	69,7%	82,6%	95,1%	96,7%	91,5%
13942	50	40	50	45	70,1%	82,4%	95,3%	96,9%	92,0%
13943	50	40	50	50	70,5%	82,3%	95,4%	97,1%	92,5%
13944	50	40	50	55	70,8%	82,1%	95,5%	97,3%	93,0%
13945	50	40	50	60	71,2%	81,9%	95,7%	97,5%	93,4%
13946	50	40	50	65	71,6%	81,7%	95,8%	97,6%	93,8%
13947	50	40	50	70	71,9%	81,5%	96,0%	97,8%	94,2%
13948	50	40	50	75	72,3%	81,3%	96,1%	97,9%	94,6%
13949	50	40	50	80	72,6%	81,1%	96,2%	98,1%	94,9%
13950	50	40	55	20	62,6%	79,9%	93,7%	95,4%	88,7%
13951	50	40	55	25	63,1%	79,7%	93,9%	95,7%	89,4%
13952	50	40	55	30	63,5%	79,5%	94,1%	96,0%	90,0%
13953	50	40	55	35	63,9%	79,3%	94,3%	96,2%	90,6%
13954	50	40	55	40	64,3%	79,1%	94,5%	96,5%	91,2%
13955	50	40	55	45	64,7%	78,8%	94,7%	96,7%	91,7%
13956	50	40	55	50	65,1%	78,6%	94,8%	96,9%	92,2%
13957	50	40	55	55	65,5%	78,4%	95,0%	97,1%	92,7%
13958	50	40	55	60	65,9%	78,2%	95,1%	97,3%	93,1%

13959	50	40	55	65	66,3%	78,0%	95,3%	97,5%	93,6%
13960	50	40	55	70	66,7%	77,7%	95,5%	97,7%	94,0%
13961	50	40	55	75	67,1%	77,5%	95,6%	97,8%	94,3%
13962	50	40	55	80	67,4%	77,3%	95,7%	97,9%	94,7%
13963	50	40	60	20	56,7%	76,0%	93,0%	95,2%	88,3%
13964	50	40	60	25	57,1%	75,7%	93,2%	95,5%	89,0%
13965	50	40	60	30	57,6%	75,5%	93,4%	95,8%	89,6%
13966	50	40	60	35	58,0%	75,2%	93,6%	96,0%	90,2%
13967	50	40	60	40	58,4%	75,0%	93,8%	96,3%	90,8%
13968	50	40	60	45	58,9%	74,7%	94,0%	96,5%	91,4%
13969	50	40	60	50	59,3%	74,5%	94,2%	96,7%	91,9%
13970	50	40	60	55	59,7%	74,2%	94,4%	97,0%	92,4%
13971	50	40	60	60	60,1%	74,0%	94,5%	97,1%	92,9%
13972	50	40	60	65	60,6%	73,7%	94,7%	97,3%	93,3%
13973	50	40	60	70	61,0%	73,5%	94,9%	97,5%	93,7%
13974	50	40	60	75	61,4%	73,2%	95,0%	97,7%	94,1%
13975	50	40	60	80	61,8%	73,0%	95,2%	97,8%	94,5%
13976	50	40	65	20	50,6%	71,5%	92,1%	94,9%	87,8%
13977	50	40	65	25	51,0%	71,2%	92,3%	95,2%	88,6%
13978	50	40	65	30	51,5%	71,0%	92,6%	95,5%	89,2%
13979	50	40	65	35	51,9%	70,7%	92,8%	95,8%	89,9%
13980	50	40	65	40	52,3%	70,4%	93,0%	96,1%	90,5%
13981	50	40	65	45	52,8%	70,1%	93,2%	96,3%	91,1%
13982	50	40	65	50	53,2%	69,9%	93,5%	96,5%	91,6%
13983	50	40	65	55	53,7%	69,6%	93,7%	96,8%	92,1%
13984	50	40	65	60	54,1%	69,3%	93,9%	97,0%	92,6%
13985	50	40	65	65	54,5%	69,0%	94,1%	97,2%	93,1%
13986	50	40	65	70	55,0%	68,8%	94,2%	97,4%	93,5%
13987	50	40	65	75	55,4%	68,5%	94,4%	97,5%	93,9%
13988	50	40	65	80	55,8%	68,2%	94,6%	97,7%	94,3%
13989	50	40	70	20	44,4%	66,6%	91,1%	94,6%	87,4%
13990	50	40	70	25	44,9%	66,3%	91,4%	94,9%	88,1%
13991	50	40	70	30	45,3%	66,0%	91,7%	95,2%	88,8%

13992	50	40	70	35	45,7%	65,7%	91,9%	95,5%	89,5%
13993	50	40	70	40	46,2%	65,4%	92,2%	95,8%	90,1%
13994	50	40	70	45	46,6%	65,1%	92,4%	96,1%	90,7%
13995	50	40	70	50	47,1%	64,8%	92,7%	96,3%	91,3%
13996	50	40	70	55	47,5%	64,5%	92,9%	96,6%	91,8%
13997	50	40	70	60	47,9%	64,2%	93,1%	96,8%	92,3%
13998	50	40	70	65	48,4%	63,9%	93,3%	97,0%	92,8%
13999	50	40	70	70	48,8%	63,6%	93,5%	97,2%	93,2%
14000	50	40	70	75	49,3%	63,3%	93,7%	97,4%	93,6%
14001	50	40	70	80	49,7%	63,0%	93,9%	97,5%	94,0%
14002	50	40	75	20	38,4%	61,2%	90,1%	94,2%	86,9%
14003	50	40	75	25	38,9%	60,9%	90,4%	94,6%	87,7%
14004	50	40	75	30	39,3%	60,6%	90,7%	94,9%	88,4%
14005	50	40	75	35	39,7%	60,3%	91,0%	95,3%	89,1%
14006	50	40	75	40	40,1%	60,0%	91,2%	95,6%	89,7%
14007	50	40	75	45	40,5%	59,7%	91,5%	95,8%	90,4%
14008	50	40	75	50	41,0%	59,4%	91,8%	96,1%	90,9%
14009	50	40	75	55	41,4%	59,0%	92,0%	96,4%	91,5%
14010	50	40	75	60	41,8%	58,7%	92,3%	96,6%	92,0%
14011	50	40	75	65	42,3%	58,4%	92,5%	96,8%	92,5%
14012	50	40	75	70	42,7%	58,1%	92,7%	97,0%	93,0%
14013	50	40	75	75	43,1%	57,8%	93,0%	97,2%	93,4%
14014	50	40	75	80	43,6%	57,5%	93,2%	97,4%	93,8%
14015	50	40	80	20	32,8%	55,6%	88,9%	93,9%	86,5%
14016	50	40	80	25	33,2%	55,3%	89,3%	94,3%	87,2%
14017	50	40	80	30	33,6%	55,0%	89,6%	94,6%	88,0%
14018	50	40	80	35	34,0%	54,7%	89,9%	95,0%	88,7%
14019	50	40	80	40	34,4%	54,3%	90,2%	95,3%	89,4%
14020	50	40	80	45	34,8%	54,0%	90,5%	95,6%	90,0%
14021	50	40	80	50	35,2%	53,7%	90,8%	95,9%	90,6%
14022	50	40	80	55	35,6%	53,4%	91,1%	96,1%	91,2%
14023	50	40	80	60	36,0%	53,0%	91,3%	96,4%	91,7%
14024	50	40	80	65	36,4%	52,7%	91,6%	96,6%	92,2%

14025	50	40	80	70	36,8%	52,4%	91,9%	96,8%	92,7%
14026	50	40	80	75	37,2%	52,1%	92,1%	97,0%	93,1%
14027	50	40	80	80	37,6%	51,7%	92,3%	97,2%	93,6%
14028	50	45	20	20	91,2%	95,6%	97,2%	96,7%	90,5%
14029	50	45	20	25	91,3%	95,5%	97,3%	97,0%	91,1%
14030	50	45	20	30	91,4%	95,5%	97,4%	97,1%	91,6%
14031	50	45	20	35	91,6%	95,4%	97,5%	97,3%	92,1%
14032	50	45	20	40	91,7%	95,4%	97,5%	97,5%	92,6%
14033	50	45	20	45	91,8%	95,3%	97,6%	97,7%	93,1%
14034	50	45	20	50	92,0%	95,2%	97,7%	97,8%	93,5%
14035	50	45	20	55	92,1%	95,2%	97,8%	98,0%	93,9%
14036	50	45	20	60	92,2%	95,1%	97,8%	98,1%	94,3%
14037	50	45	20	65	92,4%	95,1%	97,9%	98,2%	94,6%
14038	50	45	20	70	92,5%	95,0%	98,0%	98,3%	95,0%
14039	50	45	20	75	92,6%	94,9%	98,0%	98,4%	95,3%
14040	50	45	20	80	92,7%	94,9%	98,1%	98,5%	95,6%
14041	50	45	25	20	89,0%	94,5%	96,8%	96,5%	90,1%
14042	50	45	25	25	89,1%	94,4%	96,9%	96,8%	90,7%
14043	50	45	25	30	89,3%	94,4%	97,0%	97,0%	91,3%
14044	50	45	25	35	89,5%	94,3%	97,1%	97,2%	91,8%
14045	50	45	25	40	89,6%	94,2%	97,2%	97,4%	92,3%
14046	50	45	25	45	89,8%	94,2%	97,3%	97,5%	92,8%
14047	50	45	25	50	90,0%	94,1%	97,4%	97,7%	93,2%
14048	50	45	25	55	90,1%	94,0%	97,5%	97,8%	93,7%
14049	50	45	25	60	90,3%	93,9%	97,6%	98,0%	94,1%
14050	50	45	25	65	90,4%	93,9%	97,6%	98,1%	94,4%
14051	50	45	25	70	90,6%	93,8%	97,7%	98,2%	94,8%
14052	50	45	25	75	90,7%	93,7%	97,8%	98,4%	95,1%
14053	50	45	25	80	90,9%	93,6%	97,9%	98,5%	95,4%
14054	50	45	30	20	86,3%	93,2%	96,4%	96,3%	89,8%
14055	50	45	30	25	86,5%	93,1%	96,5%	96,6%	90,4%
14056	50	45	30	30	86,7%	93,0%	96,6%	96,8%	91,0%
14057	50	45	30	35	86,9%	92,9%	96,8%	97,0%	91,5%

14058	50	45	30	40	87,1%	92,8%	96,9%	97,2%	92,0%
14059	50	45	30	45	87,3%	92,8%	97,0%	97,4%	92,5%
14060	50	45	30	50	87,5%	92,7%	97,1%	97,5%	93,0%
14061	50	45	30	55	87,7%	92,6%	97,2%	97,7%	93,4%
14062	50	45	30	60	87,9%	92,5%	97,2%	97,9%	93,8%
14063	50	45	30	65	88,1%	92,4%	97,3%	98,0%	94,2%
14064	50	45	30	70	88,3%	92,3%	97,4%	98,1%	94,6%
14065	50	45	30	75	88,4%	92,2%	97,5%	98,2%	94,9%
14066	50	45	30	80	88,6%	92,1%	97,6%	98,4%	95,2%
14067	50	45	35	20	83,1%	91,6%	96,0%	96,1%	89,4%
14068	50	45	35	25	83,4%	91,5%	96,1%	96,4%	90,0%
14069	50	45	35	30	83,6%	91,4%	96,2%	96,6%	90,6%
14070	50	45	35	35	83,8%	91,2%	96,3%	96,8%	91,2%
14071	50	45	35	40	84,1%	91,1%	96,5%	97,0%	91,7%
14072	50	45	35	45	84,3%	91,0%	96,6%	97,2%	92,2%
14073	50	45	35	50	84,5%	90,9%	96,7%	97,4%	92,7%
14074	50	45	35	55	84,8%	90,8%	96,8%	97,6%	93,1%
14075	50	45	35	60	85,0%	90,7%	96,9%	97,7%	93,6%
14076	50	45	35	65	85,2%	90,6%	97,0%	97,9%	94,0%
14077	50	45	35	70	85,4%	90,5%	97,1%	98,0%	94,3%
14078	50	45	35	75	85,7%	90,4%	97,2%	98,1%	94,7%
14079	50	45	35	80	85,9%	90,3%	97,3%	98,3%	95,0%
14080	50	45	40	20	79,4%	89,6%	95,5%	95,9%	89,0%
14081	50	45	40	25	79,6%	89,5%	95,6%	96,1%	89,6%
14082	50	45	40	30	79,9%	89,3%	95,7%	96,4%	90,3%
14083	50	45	40	35	80,2%	89,2%	95,9%	96,6%	90,8%
14084	50	45	40	40	80,5%	89,1%	96,0%	96,8%	91,4%
14085	50	45	40	45	80,8%	89,0%	96,1%	97,0%	91,9%
14086	50	45	40	50	81,0%	88,8%	96,3%	97,2%	92,4%
14087	50	45	40	55	81,3%	88,7%	96,4%	97,4%	92,9%
14088	50	45	40	60	81,6%	88,6%	96,5%	97,6%	93,3%
14089	50	45	40	65	81,8%	88,4%	96,6%	97,7%	93,7%
14090	50	45	40	70	82,1%	88,3%	96,7%	97,9%	94,1%

14091	50	45	40	75	82,3%	88,2%	96,8%	98,0%	94,5%
14092	50	45	40	80	82,6%	88,0%	96,9%	98,2%	94,8%
14093	50	45	45	20	75,0%	87,2%	94,9%	95,6%	88,6%
14094	50	45	45	25	75,3%	87,1%	95,0%	95,9%	89,2%
14095	50	45	45	30	75,7%	86,9%	95,2%	96,2%	89,9%
14096	50	45	45	35	76,0%	86,8%	95,4%	96,4%	90,5%
14097	50	45	45	40	76,3%	86,6%	95,5%	96,6%	91,1%
14098	50	45	45	45	76,6%	86,5%	95,7%	96,9%	91,6%
14099	50	45	45	50	76,9%	86,3%	95,8%	97,1%	92,1%
14100	50	45	45	55	77,3%	86,2%	95,9%	97,3%	92,6%
14101	50	45	45	60	77,6%	86,0%	96,1%	97,4%	93,1%
14102	50	45	45	65	77,9%	85,9%	96,2%	97,6%	93,5%
14103	50	45	45	70	78,2%	85,7%	96,3%	97,8%	93,9%
14104	50	45	45	75	78,5%	85,5%	96,4%	97,9%	94,3%
14105	50	45	45	80	78,8%	85,4%	96,5%	98,0%	94,6%
14106	50	45	50	20	70,1%	84,4%	94,3%	95,4%	88,1%
14107	50	45	50	25	70,5%	84,3%	94,4%	95,7%	88,8%
14108	50	45	50	30	70,8%	84,1%	94,6%	95,9%	89,5%
14109	50	45	50	35	71,2%	83,9%	94,8%	96,2%	90,1%
14110	50	45	50	40	71,6%	83,7%	94,9%	96,4%	90,7%
14111	50	45	50	45	71,9%	83,6%	95,1%	96,7%	91,3%
14112	50	45	50	50	72,3%	83,4%	95,3%	96,9%	91,8%
14113	50	45	50	55	72,6%	83,2%	95,4%	97,1%	92,3%
14114	50	45	50	60	73,0%	83,0%	95,6%	97,3%	92,8%
14115	50	45	50	65	73,3%	82,8%	95,7%	97,5%	93,2%
14116	50	45	50	70	73,7%	82,6%	95,8%	97,6%	93,7%
14117	50	45	50	75	74,0%	82,4%	96,0%	97,8%	94,0%
14118	50	45	50	80	74,3%	82,3%	96,1%	97,9%	94,4%
14119	50	45	55	20	64,7%	81,2%	93,5%	95,1%	87,7%
14120	50	45	55	25	65,1%	81,0%	93,7%	95,4%	88,4%
14121	50	45	55	30	65,5%	80,8%	93,9%	95,7%	89,1%
14122	50	45	55	35	65,9%	80,5%	94,1%	96,0%	89,8%
14123	50	45	55	40	66,3%	80,3%	94,3%	96,2%	90,4%

14124	50	45	55	45	66,7%	80,1%	94,5%	96,5%	91,0%
14125	50	45	55	50	67,1%	79,9%	94,7%	96,7%	91,5%
14126	50	45	55	55	67,5%	79,7%	94,8%	96,9%	92,0%
14127	50	45	55	60	67,8%	79,5%	95,0%	97,1%	92,5%
14128	50	45	55	65	68,2%	79,3%	95,2%	97,3%	93,0%
14129	50	45	55	70	68,6%	79,1%	95,3%	97,5%	93,4%
14130	50	45	55	75	69,0%	78,9%	95,5%	97,6%	93,8%
14131	50	45	55	80	69,4%	78,6%	95,6%	97,8%	94,2%
14132	50	45	60	20	58,9%	77,4%	92,7%	94,8%	87,3%
14133	50	45	60	25	59,3%	77,1%	93,0%	95,1%	88,0%
14134	50	45	60	30	59,7%	76,9%	93,2%	95,4%	88,7%
14135	50	45	60	35	60,1%	76,7%	93,4%	95,7%	89,4%
14136	50	45	60	40	60,6%	76,4%	93,6%	96,0%	90,0%
14137	50	45	60	45	61,0%	76,2%	93,8%	96,3%	90,6%
14138	50	45	60	50	61,4%	76,0%	94,0%	96,5%	91,2%
14139	50	45	60	55	61,8%	75,7%	94,2%	96,7%	91,7%
14140	50	45	60	60	62,2%	75,5%	94,4%	96,9%	92,2%
14141	50	45	60	65	62,7%	75,2%	94,6%	97,1%	92,7%
14142	50	45	60	70	63,1%	75,0%	94,7%	97,3%	93,1%
14143	50	45	60	75	63,5%	74,7%	94,9%	97,5%	93,6%
14144	50	45	60	80	63,9%	74,5%	95,1%	97,7%	94,0%
14145	50	45	65	20	52,8%	73,1%	91,9%	94,5%	86,8%
14146	50	45	65	25	53,2%	72,8%	92,1%	94,8%	87,6%
14147	50	45	65	30	53,7%	72,6%	92,4%	95,2%	88,3%
14148	50	45	65	35	54,1%	72,3%	92,6%	95,5%	89,0%
14149	50	45	65	40	54,5%	72,0%	92,8%	95,8%	89,6%
14150	50	45	65	45	55,0%	71,8%	93,0%	96,0%	90,2%
14151	50	45	65	50	55,4%	71,5%	93,3%	96,3%	90,8%
14152	50	45	65	55	55,9%	71,2%	93,5%	96,5%	91,4%
14153	50	45	65	60	56,3%	71,0%	93,7%	96,8%	91,9%
14154	50	45	65	65	56,7%	70,7%	93,9%	97,0%	92,4%
14155	50	45	65	70	57,2%	70,4%	94,1%	97,2%	92,9%
14156	50	45	65	75	57,6%	70,1%	94,3%	97,3%	93,3%

14157	50	45	65	80	58,0%	69,9%	94,4%	97,5%	93,7%
14158	50	45	70	20	46,6%	68,3%	90,9%	94,2%	86,3%
14159	50	45	70	25	47,1%	68,0%	91,2%	94,5%	87,1%
14160	50	45	70	30	47,5%	67,7%	91,4%	94,9%	87,9%
14161	50	45	70	35	47,9%	67,4%	91,7%	95,2%	88,6%
14162	50	45	70	40	48,4%	67,1%	92,0%	95,5%	89,2%
14163	50	45	70	45	48,8%	66,9%	92,2%	95,8%	89,9%
14164	50	45	70	50	49,3%	66,6%	92,4%	96,1%	90,5%
14165	50	45	70	55	49,7%	66,3%	92,7%	96,3%	91,1%
14166	50	45	70	60	50,1%	66,0%	92,9%	96,6%	91,6%
14167	50	45	70	65	50,6%	65,7%	93,1%	96,8%	92,1%
14168	50	45	70	70	51,0%	65,4%	93,3%	97,0%	92,6%
14169	50	45	70	75	51,5%	65,1%	93,5%	97,2%	93,1%
14170	50	45	70	80	51,9%	64,8%	93,7%	97,4%	93,5%
14171	50	45	75	20	40,6%	63,1%	89,8%	93,8%	85,8%
14172	50	45	75	25	41,0%	62,8%	90,1%	94,2%	86,6%
14173	50	45	75	30	41,4%	62,5%	90,4%	94,6%	87,4%
14174	50	45	75	35	41,8%	62,2%	90,7%	94,9%	88,1%
14175	50	45	75	40	42,3%	61,9%	91,0%	95,2%	88,8%
14176	50	45	75	45	42,7%	61,6%	91,3%	95,5%	89,5%
14177	50	45	75	50	43,1%	61,2%	91,5%	95,8%	90,1%
14178	50	45	75	55	43,6%	60,9%	91,8%	96,1%	90,7%
14179	50	45	75	60	44,0%	60,6%	92,0%	96,3%	91,3%
14180	50	45	75	65	44,4%	60,3%	92,3%	96,6%	91,8%
14181	50	45	75	70	44,9%	60,0%	92,5%	96,8%	92,3%
14182	50	45	75	75	45,3%	59,7%	92,8%	97,0%	92,8%
14183	50	45	75	80	45,7%	59,4%	93,0%	97,2%	93,2%
14184	50	45	80	20	34,8%	57,6%	88,6%	93,5%	85,3%
14185	50	45	80	25	35,2%	57,3%	88,9%	93,9%	86,1%
14186	50	45	80	30	35,6%	56,9%	89,3%	94,3%	86,9%
14187	50	45	80	35	36,0%	56,6%	89,6%	94,6%	87,7%
14188	50	45	80	40	36,4%	56,3%	89,9%	95,0%	88,4%
14189	50	45	80	45	36,8%	56,0%	90,2%	95,3%	89,1%

14190	50	45	80	50	37,2%	55,6%	90,5%	95,6%	89,8%
14191	50	45	80	55	37,6%	55,3%	90,8%	95,9%	90,4%
14192	50	45	80	60	38,0%	55,0%	91,1%	96,1%	90,9%
14193	50	45	80	65	38,5%	54,7%	91,3%	96,4%	91,5%
14194	50	45	80	70	38,9%	54,4%	91,6%	96,6%	92,0%
14195	50	45	80	75	39,3%	54,0%	91,9%	96,8%	92,5%
14196	50	45	80	80	39,7%	53,7%	92,1%	97,0%	93,0%
14197	50	50	20	20	91,9%	95,9%	97,1%	96,5%	89,6%
14198	50	50	20	25	92,0%	95,9%	97,2%	96,7%	90,3%
14199	50	50	20	30	92,1%	95,8%	97,3%	96,9%	90,8%
14200	50	50	20	35	92,2%	95,8%	97,4%	97,1%	91,4%
14201	50	50	20	40	92,4%	95,7%	97,5%	97,3%	91,9%
14202	50	50	20	45	92,5%	95,6%	97,5%	97,5%	92,4%
14203	50	50	20	50	92,6%	95,6%	97,6%	97,7%	92,9%
14204	50	50	20	55	92,7%	95,5%	97,7%	97,8%	93,3%
14205	50	50	20	60	92,8%	95,5%	97,8%	98,0%	93,7%
14206	50	50	20	65	93,0%	95,4%	97,8%	98,1%	94,1%
14207	50	50	20	70	93,1%	95,4%	97,9%	98,2%	94,5%
14208	50	50	20	75	93,2%	95,3%	98,0%	98,3%	94,8%
14209	50	50	20	80	93,3%	95,2%	98,1%	98,4%	95,2%
14210	50	50	25	20	89,8%	94,9%	96,7%	96,3%	89,3%
14211	50	50	25	25	90,0%	94,8%	96,8%	96,5%	89,9%
14212	50	50	25	30	90,1%	94,8%	96,9%	96,8%	90,5%
14213	50	50	25	35	90,3%	94,7%	97,0%	97,0%	91,1%
14214	50	50	25	40	90,4%	94,6%	97,1%	97,2%	91,6%
14215	50	50	25	45	90,6%	94,6%	97,2%	97,3%	92,1%
14216	50	50	25	50	90,7%	94,5%	97,3%	97,5%	92,6%
14217	50	50	25	55	90,9%	94,4%	97,4%	97,7%	93,1%
14218	50	50	25	60	91,0%	94,4%	97,5%	97,8%	93,5%
14219	50	50	25	65	91,2%	94,3%	97,6%	98,0%	93,9%
14220	50	50	25	70	91,3%	94,2%	97,6%	98,1%	94,3%
14221	50	50	25	75	91,4%	94,2%	97,7%	98,2%	94,6%
14222	50	50	25	80	91,6%	94,1%	97,8%	98,3%	95,0%

14223	50	50	30	20	87,3%	93,7%	96,3%	96,1%	88,9%
14224	50	50	30	25	87,5%	93,6%	96,4%	96,3%	89,5%
14225	50	50	30	30	87,7%	93,5%	96,5%	96,6%	90,1%
14226	50	50	30	35	87,9%	93,4%	96,7%	96,8%	90,7%
14227	50	50	30	40	88,1%	93,3%	96,8%	97,0%	91,3%
14228	50	50	30	45	88,3%	93,3%	96,9%	97,2%	91,8%
14229	50	50	30	50	88,4%	93,2%	97,0%	97,4%	92,3%
14230	50	50	30	55	88,6%	93,1%	97,1%	97,5%	92,8%
14231	50	50	30	60	88,8%	93,0%	97,2%	97,7%	93,2%
14232	50	50	30	65	89,0%	92,9%	97,3%	97,8%	93,7%
14233	50	50	30	70	89,1%	92,8%	97,3%	98,0%	94,1%
14234	50	50	30	75	89,3%	92,8%	97,4%	98,1%	94,4%
14235	50	50	30	80	89,5%	92,7%	97,5%	98,2%	94,8%
14236	50	50	35	20	84,3%	92,1%	95,8%	95,8%	88,4%
14237	50	50	35	25	84,5%	92,1%	96,0%	96,1%	89,1%
14238	50	50	35	30	84,8%	92,0%	96,1%	96,3%	89,8%
14239	50	50	35	35	85,0%	91,9%	96,2%	96,6%	90,4%
14240	50	50	35	40	85,2%	91,8%	96,4%	96,8%	91,0%
14241	50	50	35	45	85,4%	91,7%	96,5%	97,0%	91,5%
14242	50	50	35	50	85,7%	91,6%	96,6%	97,2%	92,0%
14243	50	50	35	55	85,9%	91,5%	96,7%	97,4%	92,5%
14244	50	50	35	60	86,1%	91,4%	96,8%	97,6%	93,0%
14245	50	50	35	65	86,3%	91,3%	96,9%	97,7%	93,4%
14246	50	50	35	70	86,5%	91,1%	97,0%	97,9%	93,8%
14247	50	50	35	75	86,7%	91,0%	97,1%	98,0%	94,2%
14248	50	50	35	80	86,9%	90,9%	97,2%	98,1%	94,6%
14249	50	50	40	20	80,8%	90,3%	95,3%	95,6%	88,0%
14250	50	50	40	25	81,0%	90,2%	95,5%	95,9%	88,7%
14251	50	50	40	30	81,3%	90,1%	95,6%	96,1%	89,4%
14252	50	50	40	35	81,6%	90,0%	95,8%	96,4%	90,0%
14253	50	50	40	40	81,8%	89,8%	95,9%	96,6%	90,6%
14254	50	50	40	45	82,1%	89,7%	96,0%	96,8%	91,2%
14255	50	50	40	50	82,4%	89,6%	96,1%	97,0%	91,7%

14256	50	50	40	55	82,6%	89,5%	96,3%	97,2%	92,2%
14257	50	50	40	60	82,9%	89,3%	96,4%	97,4%	92,7%
14258	50	50	40	65	83,1%	89,2%	96,5%	97,6%	93,1%
14259	50	50	40	70	83,4%	89,1%	96,6%	97,7%	93,6%
14260	50	50	40	75	83,6%	89,0%	96,7%	97,9%	94,0%
14261	50	50	40	80	83,8%	88,8%	96,8%	98,0%	94,4%
14262	50	50	45	20	76,6%	88,1%	94,7%	95,3%	87,6%
14263	50	50	45	25	77,0%	87,9%	94,9%	95,6%	88,3%
14264	50	50	45	30	77,3%	87,8%	95,1%	95,9%	89,0%
14265	50	50	45	35	77,6%	87,7%	95,2%	96,2%	89,6%
14266	50	50	45	40	77,9%	87,5%	95,4%	96,4%	90,3%
14267	50	50	45	45	78,2%	87,4%	95,5%	96,6%	90,8%
14268	50	50	45	50	78,5%	87,2%	95,7%	96,8%	91,4%
14269	50	50	45	55	78,8%	87,1%	95,8%	97,1%	91,9%
14270	50	50	45	60	79,1%	86,9%	95,9%	97,2%	92,4%
14271	50	50	45	65	79,4%	86,8%	96,1%	97,4%	92,9%
14272	50	50	45	70	79,6%	86,6%	96,2%	97,6%	93,3%
14273	50	50	45	75	79,9%	86,5%	96,3%	97,7%	93,7%
14274	50	50	45	80	80,2%	86,3%	96,4%	97,9%	94,1%
14275	50	50	50	20	71,9%	85,4%	94,1%	95,0%	87,1%
14276	50	50	50	25	72,3%	85,3%	94,3%	95,3%	87,9%
14277	50	50	50	30	72,6%	85,1%	94,4%	95,6%	88,6%
14278	50	50	50	35	73,0%	84,9%	94,6%	95,9%	89,2%
14279	50	50	50	40	73,3%	84,8%	94,8%	96,2%	89,9%
14280	50	50	50	45	73,7%	84,6%	95,0%	96,4%	90,5%
14281	50	50	50	50	74,0%	84,4%	95,1%	96,7%	91,1%
14282	50	50	50	55	74,4%	84,3%	95,3%	96,9%	91,6%
14283	50	50	50	60	74,7%	84,1%	95,4%	97,1%	92,1%
14284	50	50	50	65	75,0%	83,9%	95,6%	97,3%	92,6%
14285	50	50	50	70	75,4%	83,7%	95,7%	97,4%	93,1%
14286	50	50	50	75	75,7%	83,6%	95,8%	97,6%	93,5%
14287	50	50	50	80	76,0%	83,4%	96,0%	97,8%	93,9%
14288	50	50	55	20	66,7%	82,3%	93,3%	94,7%	86,6%

14289	50	50	55	25	67,1%	82,1%	93,6%	95,1%	87,4%
14290	50	50	55	30	67,5%	81,9%	93,8%	95,4%	88,2%
14291	50	50	55	35	67,9%	81,8%	93,9%	95,7%	88,8%
14292	50	50	55	40	68,2%	81,6%	94,1%	96,0%	89,5%
14293	50	50	55	45	68,6%	81,4%	94,3%	96,2%	90,1%
14294	50	50	55	50	69,0%	81,2%	94,5%	96,5%	90,7%
14295	50	50	55	55	69,4%	81,0%	94,7%	96,7%	91,3%
14296	50	50	55	60	69,7%	80,8%	94,8%	96,9%	91,8%
14297	50	50	55	65	70,1%	80,6%	95,0%	97,1%	92,3%
14298	50	50	55	70	70,5%	80,3%	95,2%	97,3%	92,8%
14299	50	50	55	75	70,9%	80,1%	95,3%	97,5%	93,2%
14300	50	50	55	80	71,2%	79,9%	95,5%	97,6%	93,7%
14301	50	50	60	20	61,0%	78,7%	92,5%	94,4%	86,2%
14302	50	50	60	25	61,4%	78,5%	92,8%	94,8%	87,0%
14303	50	50	60	30	61,8%	78,3%	93,0%	95,1%	87,7%
14304	50	50	60	35	62,3%	78,1%	93,2%	95,4%	88,4%
14305	50	50	60	40	62,7%	77,8%	93,4%	95,7%	89,1%
14306	50	50	60	45	63,1%	77,6%	93,6%	96,0%	89,8%
14307	50	50	60	50	63,5%	77,4%	93,8%	96,2%	90,4%
14308	50	50	60	55	63,9%	77,1%	94,0%	96,5%	91,0%
14309	50	50	60	60	64,3%	76,9%	94,2%	96,7%	91,5%
14310	50	50	60	65	64,7%	76,7%	94,4%	96,9%	92,0%
14311	50	50	60	70	65,1%	76,4%	94,6%	97,1%	92,5%
14312	50	50	60	75	65,5%	76,2%	94,7%	97,3%	93,0%
14313	50	50	60	80	65,9%	76,0%	94,9%	97,5%	93,4%
14314	50	50	65	20	55,0%	74,6%	91,6%	94,1%	85,7%
14315	50	50	65	25	55,4%	74,3%	91,9%	94,5%	86,5%
14316	50	50	65	30	55,9%	74,1%	92,1%	94,8%	87,3%
14317	50	50	65	35	56,3%	73,8%	92,4%	95,1%	88,0%
14318	50	50	65	40	56,7%	73,6%	92,6%	95,5%	88,7%
14319	50	50	65	45	57,2%	73,3%	92,8%	95,7%	89,4%
14320	50	50	65	50	57,6%	73,1%	93,1%	96,0%	90,0%
14321	50	50	65	55	58,0%	72,8%	93,3%	96,3%	90,6%

14322	50	50	65	60	58,5%	72,6%	93,5%	96,5%	91,2%
14323	50	50	65	65	58,9%	72,3%	93,7%	96,7%	91,7%
14324	50	50	65	70	59,3%	72,0%	93,9%	96,9%	92,2%
14325	50	50	65	75	59,7%	71,8%	94,1%	97,1%	92,7%
14326	50	50	65	80	60,2%	71,5%	94,3%	97,3%	93,1%
14327	50	50	70	20	48,8%	70,0%	90,6%	93,7%	85,1%
14328	50	50	70	25	49,3%	69,7%	90,9%	94,1%	86,0%
14329	50	50	70	30	49,7%	69,4%	91,2%	94,5%	86,8%
14330	50	50	70	35	50,2%	69,1%	91,5%	94,9%	87,6%
14331	50	50	70	40	50,6%	68,9%	91,7%	95,2%	88,3%
14332	50	50	70	45	51,0%	68,6%	92,0%	95,5%	89,0%
14333	50	50	70	50	51,5%	68,3%	92,2%	95,8%	89,6%
14334	50	50	70	55	51,9%	68,0%	92,5%	96,0%	90,3%
14335	50	50	70	60	52,4%	67,7%	92,7%	96,3%	90,8%
14336	50	50	70	65	52,8%	67,4%	92,9%	96,5%	91,4%
14337	50	50	70	70	53,2%	67,2%	93,1%	96,8%	91,9%
14338	50	50	70	75	53,7%	66,9%	93,3%	97,0%	92,4%
14339	50	50	70	80	54,1%	66,6%	93,6%	97,2%	92,9%
14340	50	50	75	20	42,7%	64,9%	89,5%	93,4%	84,6%
14341	50	50	75	25	43,2%	64,6%	89,8%	93,8%	85,5%
14342	50	50	75	30	43,6%	64,3%	90,1%	94,2%	86,3%
14343	50	50	75	35	44,0%	64,0%	90,4%	94,5%	87,1%
14344	50	50	75	40	44,5%	63,7%	90,7%	94,9%	87,9%
14345	50	50	75	45	44,9%	63,4%	91,0%	95,2%	88,6%
14346	50	50	75	50	45,3%	63,1%	91,3%	95,5%	89,2%
14347	50	50	75	55	45,8%	62,8%	91,5%	95,8%	89,9%
14348	50	50	75	60	46,2%	62,5%	91,8%	96,1%	90,5%
14349	50	50	75	65	46,6%	62,2%	92,1%	96,3%	91,1%
14350	50	50	75	70	47,1%	61,9%	92,3%	96,6%	91,6%
14351	50	50	75	75	47,5%	61,6%	92,5%	96,8%	92,1%
14352	50	50	75	80	48,0%	61,3%	92,8%	97,0%	92,6%
14353	50	50	80	20	36,8%	59,5%	88,3%	93,0%	84,1%
14354	50	50	80	25	37,2%	59,2%	88,6%	93,4%	85,0%

14355	50	50	80	30	37,6%	58,9%	89,0%	93,8%	85,8%
14356	50	50	80	35	38,1%	58,5%	89,3%	94,2%	86,6%
14357	50	50	80	40	38,5%	58,2%	89,6%	94,6%	87,4%
14358	50	50	80	45	38,9%	57,9%	89,9%	94,9%	88,1%
14359	50	50	80	50	39,3%	57,6%	90,2%	95,3%	88,8%
14360	50	50	80	55	39,7%	57,3%	90,5%	95,6%	89,5%
14361	50	50	80	60	40,1%	56,9%	90,8%	95,8%	90,1%
14362	50	50	80	65	40,6%	56,6%	91,1%	96,1%	90,7%
14363	50	50	80	70	41,0%	56,3%	91,4%	96,4%	91,3%
14364	50	50	80	75	41,4%	56,0%	91,6%	96,6%	91,8%
14365	50	50	80	80	41,9%	55,7%	91,9%	96,8%	92,3%
14366	50	55	20	20	92,5%	96,2%	97,0%	96,2%	88,7%
14367	50	55	20	25	92,6%	96,2%	97,1%	96,5%	89,4%
14368	50	55	20	30	92,7%	96,1%	97,2%	96,7%	90,0%
14369	50	55	20	35	92,9%	96,1%	97,3%	96,9%	90,6%
14370	50	55	20	40	93,0%	96,0%	97,4%	97,1%	91,2%
14371	50	55	20	45	93,1%	96,0%	97,5%	97,3%	91,7%
14372	50	55	20	50	93,2%	95,9%	97,5%	97,5%	92,2%
14373	50	55	20	55	93,3%	95,9%	97,6%	97,6%	92,7%
14374	50	55	20	60	93,4%	95,8%	97,7%	97,8%	93,2%
14375	50	55	20	65	93,5%	95,8%	97,8%	97,9%	93,6%
14376	50	55	20	70	93,6%	95,7%	97,9%	98,1%	94,0%
14377	50	55	20	75	93,7%	95,6%	97,9%	98,2%	94,4%
14378	50	55	20	80	93,8%	95,6%	98,0%	98,3%	94,7%
14379	50	55	25	20	90,6%	95,3%	96,6%	96,0%	88,3%
14380	50	55	25	25	90,7%	95,2%	96,7%	96,3%	89,0%
14381	50	55	25	30	90,9%	95,2%	96,8%	96,5%	89,6%
14382	50	55	25	35	91,0%	95,1%	96,9%	96,7%	90,3%
14383	50	55	25	40	91,2%	95,0%	97,0%	96,9%	90,9%
14384	50	55	25	45	91,3%	95,0%	97,1%	97,1%	91,4%
14385	50	55	25	50	91,5%	94,9%	97,2%	97,3%	91,9%
14386	50	55	25	55	91,6%	94,8%	97,3%	97,5%	92,4%
14387	50	55	25	60	91,7%	94,8%	97,4%	97,7%	92,9%

14388	50	55	25	65	91,9%	94,7%	97,5%	97,8%	93,3%
14389	50	55	25	70	92,0%	94,6%	97,6%	98,0%	93,7%
14390	50	55	25	75	92,1%	94,6%	97,7%	98,1%	94,1%
14391	50	55	25	80	92,2%	94,5%	97,7%	98,2%	94,5%
14392	50	55	30	20	88,3%	94,1%	96,2%	95,8%	87,9%
14393	50	55	30	25	88,4%	94,0%	96,3%	96,0%	88,6%
14394	50	55	30	30	88,6%	94,0%	96,4%	96,3%	89,3%
14395	50	55	30	35	88,8%	93,9%	96,6%	96,5%	89,9%
14396	50	55	30	40	89,0%	93,8%	96,7%	96,8%	90,5%
14397	50	55	30	45	89,1%	93,7%	96,8%	97,0%	91,1%
14398	50	55	30	50	89,3%	93,7%	96,9%	97,2%	91,6%
14399	50	55	30	55	89,5%	93,6%	97,0%	97,3%	92,1%
14400	50	55	30	60	89,6%	93,5%	97,1%	97,5%	92,6%
14401	50	55	30	65	89,8%	93,4%	97,2%	97,7%	93,1%
14402	50	55	30	70	90,0%	93,3%	97,3%	97,8%	93,5%
14403	50	55	30	75	90,1%	93,3%	97,4%	98,0%	93,9%
14404	50	55	30	80	90,3%	93,2%	97,4%	98,1%	94,3%
14405	50	55	35	20	85,5%	92,7%	95,7%	95,5%	87,4%
14406	50	55	35	25	85,7%	92,6%	95,9%	95,8%	88,2%
14407	50	55	35	30	85,9%	92,5%	96,0%	96,1%	88,9%
14408	50	55	35	35	86,1%	92,4%	96,1%	96,3%	89,5%
14409	50	55	35	40	86,3%	92,3%	96,2%	96,6%	90,1%
14410	50	55	35	45	86,5%	92,2%	96,4%	96,8%	90,7%
14411	50	55	35	50	86,7%	92,1%	96,5%	97,0%	91,3%
14412	50	55	35	55	86,9%	92,1%	96,6%	97,2%	91,8%
14413	50	55	35	60	87,1%	92,0%	96,7%	97,4%	92,3%
14414	50	55	35	65	87,3%	91,9%	96,8%	97,5%	92,8%
14415	50	55	35	70	87,5%	91,8%	96,9%	97,7%	93,2%
14416	50	55	35	75	87,7%	91,7%	97,0%	97,9%	93,7%
14417	50	55	35	80	87,9%	91,6%	97,1%	98,0%	94,1%
14418	50	55	40	20	82,1%	91,0%	95,2%	95,3%	87,0%
14419	50	55	40	25	82,4%	90,9%	95,3%	95,6%	87,7%
14420	50	55	40	30	82,6%	90,8%	95,5%	95,8%	88,4%

14421	50	55	40	35	82,9%	90,6%	95,6%	96,1%	89,1%
14422	50	55	40	40	83,1%	90,5%	95,8%	96,4%	89,8%
14423	50	55	40	45	83,4%	90,4%	95,9%	96,6%	90,4%
14424	50	55	40	50	83,6%	90,3%	96,0%	96,8%	91,0%
14425	50	55	40	55	83,8%	90,2%	96,2%	97,0%	91,5%
14426	50	55	40	60	84,1%	90,1%	96,3%	97,2%	92,0%
14427	50	55	40	65	84,3%	90,0%	96,4%	97,4%	92,5%
14428	50	55	40	70	84,6%	89,8%	96,5%	97,6%	93,0%
14429	50	55	40	75	84,8%	89,7%	96,6%	97,7%	93,4%
14430	50	55	40	80	85,0%	89,6%	96,7%	97,9%	93,8%
14431	50	55	45	20	78,2%	88,9%	94,6%	95,0%	86,5%
14432	50	55	45	25	78,5%	88,8%	94,7%	95,3%	87,3%
14433	50	55	45	30	78,8%	88,6%	94,9%	95,6%	88,0%
14434	50	55	45	35	79,1%	88,5%	95,1%	95,9%	88,7%
14435	50	55	45	40	79,4%	88,4%	95,2%	96,1%	89,4%
14436	50	55	45	45	79,7%	88,2%	95,4%	96,4%	90,0%
14437	50	55	45	50	79,9%	88,1%	95,5%	96,6%	90,6%
14438	50	55	45	55	80,2%	88,0%	95,7%	96,8%	91,2%
14439	50	55	45	60	80,5%	87,8%	95,8%	97,0%	91,7%
14440	50	55	45	65	80,8%	87,7%	95,9%	97,2%	92,2%
14441	50	55	45	70	81,0%	87,5%	96,1%	97,4%	92,7%
14442	50	55	45	75	81,3%	87,4%	96,2%	97,6%	93,2%
14443	50	55	45	80	81,6%	87,2%	96,3%	97,7%	93,6%
14444	50	55	50	20	73,7%	86,4%	93,9%	94,7%	86,0%
14445	50	55	50	25	74,0%	86,2%	94,1%	95,0%	86,8%
14446	50	55	50	30	74,4%	86,1%	94,3%	95,3%	87,6%
14447	50	55	50	35	74,7%	85,9%	94,5%	95,6%	88,3%
14448	50	55	50	40	75,0%	85,8%	94,6%	95,9%	89,0%
14449	50	55	50	45	75,4%	85,6%	94,8%	96,2%	89,6%
14450	50	55	50	50	75,7%	85,4%	95,0%	96,4%	90,3%
14451	50	55	50	55	76,0%	85,3%	95,1%	96,6%	90,8%
14452	50	55	50	60	76,3%	85,1%	95,3%	96,9%	91,4%
14453	50	55	50	65	76,6%	85,0%	95,4%	97,1%	91,9%

14454	50	55	50	70	77,0%	84,8%	95,6%	97,3%	92,4%
14455	50	55	50	75	77,3%	84,6%	95,7%	97,4%	92,9%
14456	50	55	50	80	77,6%	84,4%	95,9%	97,6%	93,3%
14457	50	55	55	20	68,6%	83,4%	93,1%	94,4%	85,5%
14458	50	55	55	25	69,0%	83,3%	93,4%	94,7%	86,3%
14459	50	55	55	30	69,4%	83,1%	93,6%	95,0%	87,1%
14460	50	55	55	35	69,8%	82,9%	93,8%	95,4%	87,9%
14461	50	55	55	40	70,1%	82,7%	94,0%	95,7%	88,6%
14462	50	55	55	45	70,5%	82,5%	94,2%	95,9%	89,3%
14463	50	55	55	50	70,9%	82,3%	94,3%	96,2%	89,9%
14464	50	55	55	55	71,2%	82,1%	94,5%	96,4%	90,5%
14465	50	55	55	60	71,6%	81,9%	94,7%	96,7%	91,1%
14466	50	55	55	65	71,9%	81,8%	94,9%	96,9%	91,6%
14467	50	55	55	70	72,3%	81,6%	95,0%	97,1%	92,1%
14468	50	55	55	75	72,6%	81,4%	95,2%	97,3%	92,6%
14469	50	55	55	80	73,0%	81,2%	95,3%	97,5%	93,1%
14470	50	55	60	20	63,1%	80,0%	92,3%	94,0%	85,0%
14471	50	55	60	25	63,5%	79,8%	92,5%	94,4%	85,8%
14472	50	55	60	30	63,9%	79,6%	92,8%	94,7%	86,6%
14473	50	55	60	35	64,3%	79,4%	93,0%	95,1%	87,4%
14474	50	55	60	40	64,7%	79,2%	93,2%	95,4%	88,2%
14475	50	55	60	45	65,1%	78,9%	93,4%	95,7%	88,8%
14476	50	55	60	50	65,5%	78,7%	93,6%	96,0%	89,5%
14477	50	55	60	55	65,9%	78,5%	93,8%	96,2%	90,1%
14478	50	55	60	60	66,3%	78,3%	94,0%	96,5%	90,7%
14479	50	55	60	65	66,7%	78,1%	94,2%	96,7%	91,3%
14480	50	55	60	70	67,1%	77,8%	94,4%	96,9%	91,8%
14481	50	55	60	75	67,5%	77,6%	94,6%	97,1%	92,3%
14482	50	55	60	80	67,9%	77,4%	94,7%	97,3%	92,8%
14483	50	55	65	20	57,2%	76,1%	91,4%	93,7%	84,4%
14484	50	55	65	25	57,6%	75,8%	91,6%	94,1%	85,3%
14485	50	55	65	30	58,0%	75,6%	91,9%	94,4%	86,2%
14486	50	55	65	35	58,5%	75,3%	92,1%	94,8%	87,0%

14487	50	55	65	40	58,9%	75,1%	92,4%	95,1%	87,7%
14488	50	55	65	45	59,3%	74,8%	92,6%	95,4%	88,4%
14489	50	55	65	50	59,7%	74,6%	92,9%	95,7%	89,1%
14490	50	55	65	55	60,2%	74,3%	93,1%	96,0%	89,8%
14491	50	55	65	60	60,6%	74,1%	93,3%	96,3%	90,4%
14492	50	55	65	65	61,0%	73,8%	93,5%	96,5%	91,0%
14493	50	55	65	70	61,4%	73,6%	93,7%	96,7%	91,5%
14494	50	55	65	75	61,8%	73,3%	93,9%	96,9%	92,0%
14495	50	55	65	80	62,3%	73,1%	94,1%	97,1%	92,5%
14496	50	55	70	20	51,1%	71,6%	90,3%	93,3%	83,9%
14497	50	55	70	25	51,5%	71,3%	90,6%	93,7%	84,8%
14498	50	55	70	30	51,9%	71,1%	90,9%	94,1%	85,7%
14499	50	55	70	35	52,4%	70,8%	91,2%	94,5%	86,5%
14500	50	55	70	40	52,8%	70,5%	91,5%	94,8%	87,3%
14501	50	55	70	45	53,3%	70,3%	91,7%	95,2%	88,0%
14502	50	55	70	50	53,7%	70,0%	92,0%	95,5%	88,7%
14503	50	55	70	55	54,1%	69,7%	92,2%	95,8%	89,4%
14504	50	55	70	60	54,6%	69,4%	92,5%	96,0%	90,0%
14505	50	55	70	65	55,0%	69,1%	92,7%	96,3%	90,6%
14506	50	55	70	70	55,4%	68,9%	92,9%	96,5%	91,2%
14507	50	55	70	75	55,9%	68,6%	93,1%	96,7%	91,7%
14508	50	55	70	80	56,3%	68,3%	93,4%	97,0%	92,2%
14509	50	55	75	20	44,9%	66,7%	89,2%	92,9%	83,3%
14510	50	55	75	25	45,3%	66,4%	89,5%	93,3%	84,3%
14511	50	55	75	30	45,8%	66,1%	89,8%	93,8%	85,1%
14512	50	55	75	35	46,2%	65,8%	90,1%	94,2%	86,0%
14513	50	55	75	40	46,7%	65,5%	90,4%	94,5%	86,8%
14514	50	55	75	45	47,1%	65,2%	90,7%	94,9%	87,6%
14515	50	55	75	50	47,5%	64,9%	91,0%	95,2%	88,3%
14516	50	55	75	55	48,0%	64,6%	91,3%	95,5%	89,0%
14517	50	55	75	60	48,4%	64,3%	91,6%	95,8%	89,6%
14518	50	55	75	65	48,9%	64,0%	91,8%	96,1%	90,3%
14519	50	55	75	70	49,3%	63,7%	92,1%	96,3%	90,8%

14520	50	55	75	75	49,7%	63,4%	92,3%	96,5%	91,4%
14521	50	55	75	80	50,2%	63,1%	92,6%	96,8%	91,9%
14522	50	55	80	20	38,9%	61,4%	88,0%	92,5%	82,8%
14523	50	55	80	25	39,3%	61,1%	88,3%	93,0%	83,7%
14524	50	55	80	30	39,7%	60,7%	88,7%	93,4%	84,6%
14525	50	55	80	35	40,2%	60,4%	89,0%	93,8%	85,5%
14526	50	55	80	40	40,6%	60,1%	89,3%	94,2%	86,3%
14527	50	55	80	45	41,0%	59,8%	89,6%	94,6%	87,1%
14528	50	55	80	50	41,4%	59,5%	89,9%	94,9%	87,9%
14529	50	55	80	55	41,9%	59,2%	90,3%	95,2%	88,6%
14530	50	55	80	60	42,3%	58,9%	90,5%	95,5%	89,2%
14531	50	55	80	65	42,7%	58,5%	90,8%	95,8%	89,9%
14532	50	55	80	70	43,2%	58,2%	91,1%	96,1%	90,5%
14533	50	55	80	75	43,6%	57,9%	91,4%	96,3%	91,1%
14534	50	55	80	80	44,0%	57,6%	91,6%	96,6%	91,6%
14535	50	60	20	20	93,1%	96,5%	96,9%	96,0%	87,7%
14536	50	60	20	25	93,2%	96,4%	97,0%	96,2%	88,5%
14537	50	60	20	30	93,3%	96,4%	97,1%	96,5%	89,1%
14538	50	60	20	35	93,4%	96,4%	97,2%	96,7%	89,8%
14539	50	60	20	40	93,5%	96,3%	97,3%	96,9%	90,4%
14540	50	60	20	45	93,6%	96,3%	97,4%	97,1%	91,0%
14541	50	60	20	50	93,7%	96,2%	97,5%	97,3%	91,5%
14542	50	60	20	55	93,8%	96,2%	97,6%	97,5%	92,0%
14543	50	60	20	60	93,9%	96,1%	97,6%	97,6%	92,5%
14544	50	60	20	65	94,0%	96,1%	97,7%	97,8%	93,0%
14545	50	60	20	70	94,1%	96,0%	97,8%	97,9%	93,4%
14546	50	60	20	75	94,2%	96,0%	97,9%	98,1%	93,8%
14547	50	60	20	80	94,3%	95,9%	97,9%	98,2%	94,2%
14548	50	60	25	20	91,3%	95,6%	96,5%	95,7%	87,3%
14549	50	60	25	25	91,5%	95,6%	96,6%	96,0%	88,0%
14550	50	60	25	30	91,6%	95,5%	96,7%	96,3%	88,7%
14551	50	60	25	35	91,7%	95,4%	96,8%	96,5%	89,4%
14552	50	60	25	40	91,9%	95,4%	96,9%	96,7%	90,0%

14553	50	60	25	45	92,0%	95,3%	97,0%	96,9%	90,6%
14554	50	60	25	50	92,1%	95,3%	97,1%	97,1%	91,2%
14555	50	60	25	55	92,2%	95,2%	97,2%	97,3%	91,7%
14556	50	60	25	60	92,4%	95,2%	97,3%	97,5%	92,2%
14557	50	60	25	65	92,5%	95,1%	97,4%	97,7%	92,7%
14558	50	60	25	70	92,6%	95,0%	97,5%	97,8%	93,2%
14559	50	60	25	75	92,7%	95,0%	97,6%	97,9%	93,6%
14560	50	60	25	80	92,9%	94,9%	97,7%	98,1%	94,0%
14561	50	60	30	20	89,2%	94,5%	96,1%	95,5%	86,8%
14562	50	60	30	25	89,3%	94,5%	96,2%	95,8%	87,6%
14563	50	60	30	30	89,5%	94,4%	96,3%	96,0%	88,3%
14564	50	60	30	35	89,7%	94,3%	96,4%	96,3%	89,0%
14565	50	60	30	40	89,8%	94,3%	96,6%	96,5%	89,7%
14566	50	60	30	45	90,0%	94,2%	96,7%	96,7%	90,3%
14567	50	60	30	50	90,1%	94,1%	96,8%	97,0%	90,9%
14568	50	60	30	55	90,3%	94,0%	96,9%	97,2%	91,4%
14569	50	60	30	60	90,4%	94,0%	97,0%	97,3%	91,9%
14570	50	60	30	65	90,6%	93,9%	97,1%	97,5%	92,4%
14571	50	60	30	70	90,7%	93,8%	97,2%	97,7%	92,9%
14572	50	60	30	75	90,9%	93,7%	97,3%	97,8%	93,3%
14573	50	60	30	80	91,0%	93,7%	97,4%	98,0%	93,7%
14574	50	60	35	20	86,5%	93,2%	95,6%	95,2%	86,3%
14575	50	60	35	25	86,7%	93,1%	95,7%	95,5%	87,1%
14576	50	60	35	30	86,9%	93,0%	95,9%	95,8%	87,9%
14577	50	60	35	35	87,1%	93,0%	96,0%	96,1%	88,6%
14578	50	60	35	40	87,3%	92,9%	96,1%	96,3%	89,3%
14579	50	60	35	45	87,5%	92,8%	96,2%	96,5%	89,9%
14580	50	60	35	50	87,7%	92,7%	96,4%	96,8%	90,5%
14581	50	60	35	55	87,9%	92,6%	96,5%	97,0%	91,1%
14582	50	60	35	60	88,1%	92,5%	96,6%	97,2%	91,6%
14583	50	60	35	65	88,3%	92,4%	96,7%	97,4%	92,1%
14584	50	60	35	70	88,4%	92,3%	96,8%	97,5%	92,6%
14585	50	60	35	75	88,6%	92,2%	96,9%	97,7%	93,1%

14586	50	60	35	80	88,8%	92,2%	97,0%	97,8%	93,5%
14587	50	60	40	20	83,4%	91,6%	95,0%	94,9%	85,8%
14588	50	60	40	25	83,6%	91,5%	95,2%	95,2%	86,7%
14589	50	60	40	30	83,9%	91,4%	95,3%	95,5%	87,4%
14590	50	60	40	35	84,1%	91,3%	95,5%	95,8%	88,2%
14591	50	60	40	40	84,3%	91,2%	95,6%	96,1%	88,9%
14592	50	60	40	45	84,6%	91,1%	95,8%	96,3%	89,5%
14593	50	60	40	50	84,8%	91,0%	95,9%	96,6%	90,1%
14594	50	60	40	55	85,0%	90,9%	96,0%	96,8%	90,7%
14595	50	60	40	60	85,2%	90,8%	96,2%	97,0%	91,3%
14596	50	60	40	65	85,5%	90,6%	96,3%	97,2%	91,8%
14597	50	60	40	70	85,7%	90,5%	96,4%	97,4%	92,3%
14598	50	60	40	75	85,9%	90,4%	96,5%	97,5%	92,8%
14599	50	60	40	80	86,1%	90,3%	96,6%	97,7%	93,2%
14600	50	60	45	20	79,7%	89,6%	94,4%	94,6%	85,3%
14601	50	60	45	25	79,9%	89,5%	94,6%	94,9%	86,2%
14602	50	60	45	30	80,2%	89,4%	94,8%	95,3%	87,0%
14603	50	60	45	35	80,5%	89,3%	94,9%	95,6%	87,7%
14604	50	60	45	40	80,8%	89,1%	95,1%	95,8%	88,4%
14605	50	60	45	45	81,1%	89,0%	95,2%	96,1%	89,1%
14606	50	60	45	50	81,3%	88,9%	95,4%	96,4%	89,8%
14607	50	60	45	55	81,6%	88,8%	95,5%	96,6%	90,4%
14608	50	60	45	60	81,9%	88,6%	95,7%	96,8%	91,0%
14609	50	60	45	65	82,1%	88,5%	95,8%	97,0%	91,5%
14610	50	60	45	70	82,4%	88,4%	96,0%	97,2%	92,0%
14611	50	60	45	75	82,6%	88,2%	96,1%	97,4%	92,5%
14612	50	60	45	80	82,9%	88,1%	96,2%	97,6%	93,0%
14613	50	60	50	20	75,4%	87,3%	93,7%	94,3%	84,8%
14614	50	60	50	25	75,7%	87,1%	93,9%	94,6%	85,7%
14615	50	60	50	30	76,0%	87,0%	94,1%	95,0%	86,5%
14616	50	60	50	35	76,3%	86,9%	94,3%	95,3%	87,3%
14617	50	60	50	40	76,7%	86,7%	94,5%	95,6%	88,0%
14618	50	60	50	45	77,0%	86,6%	94,6%	95,9%	88,7%

14619	50	60	50	50	77,3%	86,4%	94,8%	96,1%	89,4%
14620	50	60	50	55	77,6%	86,2%	95,0%	96,4%	90,0%
14621	50	60	50	60	77,9%	86,1%	95,1%	96,6%	90,6%
14622	50	60	50	65	78,2%	85,9%	95,3%	96,8%	91,2%
14623	50	60	50	70	78,5%	85,8%	95,4%	97,0%	91,7%
14624	50	60	50	75	78,8%	85,6%	95,6%	97,2%	92,2%
14625	50	60	50	80	79,1%	85,4%	95,7%	97,4%	92,7%
14626	50	60	55	20	70,5%	84,5%	92,9%	93,9%	84,3%
14627	50	60	55	25	70,9%	84,3%	93,2%	94,3%	85,2%
14628	50	60	55	30	71,2%	84,2%	93,4%	94,7%	86,0%
14629	50	60	55	35	71,6%	84,0%	93,6%	95,0%	86,8%
14630	50	60	55	40	72,0%	83,8%	93,8%	95,3%	87,6%
14631	50	60	55	45	72,3%	83,6%	94,0%	95,6%	88,3%
14632	50	60	55	50	72,7%	83,4%	94,2%	95,9%	89,0%
14633	50	60	55	55	73,0%	83,3%	94,3%	96,2%	89,6%
14634	50	60	55	60	73,4%	83,1%	94,5%	96,4%	90,3%
14635	50	60	55	65	73,7%	82,9%	94,7%	96,7%	90,8%
14636	50	60	55	70	74,0%	82,7%	94,9%	96,9%	91,4%
14637	50	60	55	75	74,4%	82,5%	95,0%	97,1%	91,9%
14638	50	60	55	80	74,7%	82,3%	95,2%	97,3%	92,4%
14639	50	60	60	20	65,1%	81,2%	92,1%	93,6%	83,7%
14640	50	60	60	25	65,5%	81,0%	92,3%	94,0%	84,6%
14641	50	60	60	30	65,9%	80,8%	92,6%	94,4%	85,5%
14642	50	60	60	35	66,3%	80,6%	92,8%	94,7%	86,3%
14643	50	60	60	40	66,7%	80,4%	93,0%	95,1%	87,1%
14644	50	60	60	45	67,1%	80,2%	93,2%	95,4%	87,9%
14645	50	60	60	50	67,5%	80,0%	93,4%	95,7%	88,6%
14646	50	60	60	55	67,9%	79,8%	93,6%	95,9%	89,3%
14647	50	60	60	60	68,3%	79,6%	93,8%	96,2%	89,9%
14648	50	60	60	65	68,6%	79,4%	94,0%	96,4%	90,5%
14649	50	60	60	70	69,0%	79,2%	94,2%	96,7%	91,1%
14650	50	60	60	75	69,4%	78,9%	94,4%	96,9%	91,6%
14651	50	60	60	80	69,8%	78,7%	94,6%	97,1%	92,1%

14652	50	60	65	20	59,3%	77,5%	91,1%	93,2%	83,1%
14653	50	60	65	25	59,8%	77,2%	91,4%	93,6%	84,1%
14654	50	60	65	30	60,2%	77,0%	91,7%	94,0%	85,0%
14655	50	60	65	35	60,6%	76,8%	91,9%	94,4%	85,8%
14656	50	60	65	40	61,0%	76,5%	92,2%	94,8%	86,6%
14657	50	60	65	45	61,4%	76,3%	92,4%	95,1%	87,4%
14658	50	60	65	50	61,9%	76,1%	92,6%	95,4%	88,2%
14659	50	60	65	55	62,3%	75,8%	92,9%	95,7%	88,9%
14660	50	60	65	60	62,7%	75,6%	93,1%	96,0%	89,5%
14661	50	60	65	65	63,1%	75,3%	93,3%	96,2%	90,1%
14662	50	60	65	70	63,5%	75,1%	93,5%	96,5%	90,7%
14663	50	60	65	75	63,9%	74,8%	93,7%	96,7%	91,3%
14664	50	60	65	80	64,3%	74,6%	93,9%	96,9%	91,8%
14665	50	60	70	20	53,3%	73,2%	90,1%	92,8%	82,6%
14666	50	60	70	25	53,7%	72,9%	90,4%	93,3%	83,5%
14667	50	60	70	30	54,1%	72,7%	90,7%	93,7%	84,4%
14668	50	60	70	35	54,6%	72,4%	90,9%	94,1%	85,3%
14669	50	60	70	40	55,0%	72,1%	91,2%	94,5%	86,2%
14670	50	60	70	45	55,5%	71,9%	91,5%	94,8%	87,0%
14671	50	60	70	50	55,9%	71,6%	91,7%	95,1%	87,7%
14672	50	60	70	55	56,3%	71,3%	92,0%	95,4%	88,4%
14673	50	60	70	60	56,8%	71,1%	92,2%	95,7%	89,1%
14674	50	60	70	65	57,2%	70,8%	92,5%	96,0%	89,8%
14675	50	60	70	70	57,6%	70,5%	92,7%	96,3%	90,4%
14676	50	60	70	75	58,1%	70,3%	92,9%	96,5%	91,0%
14677	50	60	70	80	58,5%	70,0%	93,2%	96,7%	91,5%
14678	50	60	75	20	47,1%	68,4%	88,9%	92,4%	82,0%
14679	50	60	75	25	47,5%	68,1%	89,2%	92,9%	83,0%
14680	50	60	75	30	48,0%	67,8%	89,6%	93,3%	83,9%
14681	50	60	75	35	48,4%	67,6%	89,9%	93,7%	84,8%
14682	50	60	75	40	48,9%	67,3%	90,2%	94,1%	85,7%
14683	50	60	75	45	49,3%	67,0%	90,5%	94,5%	86,5%
14684	50	60	75	50	49,7%	66,7%	90,8%	94,8%	87,3%

14685	50	60	75	55	50,2%	66,4%	91,0%	95,2%	88,0%
14686	50	60	75	60	50,6%	66,1%	91,3%	95,5%	88,7%
14687	50	60	75	65	51,1%	65,8%	91,6%	95,8%	89,4%
14688	50	60	75	70	51,5%	65,5%	91,8%	96,0%	90,0%
14689	50	60	75	75	51,9%	65,2%	92,1%	96,3%	90,6%
14690	50	60	75	80	52,4%	64,9%	92,3%	96,5%	91,2%
14691	50	60	80	20	41,0%	63,2%	87,6%	92,0%	81,3%
14692	50	60	80	25	41,5%	62,9%	88,0%	92,5%	82,4%
14693	50	60	80	30	41,9%	62,6%	88,3%	92,9%	83,3%
14694	50	60	80	35	42,3%	62,3%	88,7%	93,4%	84,3%
14695	50	60	80	40	42,7%	62,0%	89,0%	93,8%	85,2%
14696	50	60	80	45	43,2%	61,7%	89,3%	94,2%	86,0%
14697	50	60	80	50	43,6%	61,4%	89,7%	94,5%	86,8%
14698	50	60	80	55	44,0%	61,1%	90,0%	94,9%	87,6%
14699	50	60	80	60	44,5%	60,8%	90,3%	95,2%	88,3%
14700	50	60	80	65	44,9%	60,4%	90,6%	95,5%	89,0%
14701	50	60	80	70	45,4%	60,1%	90,9%	95,8%	89,6%
14702	50	60	80	75	45,8%	59,8%	91,1%	96,1%	90,3%
14703	50	60	80	80	46,2%	59,5%	91,4%	96,3%	90,8%
14704	50	65	20	20	93,6%	96,7%	96,8%	95,7%	86,7%
14705	50	65	20	25	93,7%	96,7%	96,9%	95,9%	87,4%
14706	50	65	20	30	93,8%	96,7%	97,0%	96,2%	88,2%
14707	50	65	20	35	93,9%	96,6%	97,1%	96,4%	88,9%
14708	50	65	20	40	94,0%	96,6%	97,2%	96,7%	89,5%
14709	50	65	20	45	94,1%	96,5%	97,3%	96,9%	90,2%
14710	50	65	20	50	94,2%	96,5%	97,4%	97,1%	90,7%
14711	50	65	20	55	94,3%	96,4%	97,5%	97,3%	91,3%
14712	50	65	20	60	94,4%	96,4%	97,6%	97,5%	91,8%
14713	50	65	20	65	94,5%	96,4%	97,6%	97,6%	92,3%
14714	50	65	20	70	94,6%	96,3%	97,7%	97,8%	92,8%
14715	50	65	20	75	94,7%	96,3%	97,8%	97,9%	93,2%
14716	50	65	20	80	94,8%	96,2%	97,9%	98,1%	93,7%
14717	50	65	25	20	92,0%	95,9%	96,4%	95,4%	86,2%

14718	50	65	25	25	92,1%	95,9%	96,5%	95,7%	87,0%
14719	50	65	25	30	92,3%	95,8%	96,6%	96,0%	87,7%
14720	50	65	25	35	92,4%	95,8%	96,7%	96,2%	88,5%
14721	50	65	25	40	92,5%	95,7%	96,9%	96,5%	89,1%
14722	50	65	25	45	92,6%	95,7%	97,0%	96,7%	89,8%
14723	50	65	25	50	92,7%	95,6%	97,1%	96,9%	90,4%
14724	50	65	25	55	92,9%	95,6%	97,1%	97,1%	91,0%
14725	50	65	25	60	93,0%	95,5%	97,2%	97,3%	91,5%
14726	50	65	25	65	93,1%	95,4%	97,3%	97,5%	92,0%
14727	50	65	25	70	93,2%	95,4%	97,4%	97,6%	92,5%
14728	50	65	25	75	93,3%	95,3%	97,5%	97,8%	93,0%
14729	50	65	25	80	93,4%	95,3%	97,6%	97,9%	93,4%
14730	50	65	30	20	90,0%	94,9%	96,0%	95,1%	85,7%
14731	50	65	30	25	90,1%	94,9%	96,1%	95,4%	86,5%
14732	50	65	30	30	90,3%	94,8%	96,2%	95,7%	87,3%
14733	50	65	30	35	90,4%	94,7%	96,3%	96,0%	88,0%
14734	50	65	30	40	90,6%	94,7%	96,5%	96,3%	88,7%
14735	50	65	30	45	90,7%	94,6%	96,6%	96,5%	89,4%
14736	50	65	30	50	90,9%	94,5%	96,7%	96,7%	90,0%
14737	50	65	30	55	91,0%	94,5%	96,8%	96,9%	90,6%
14738	50	65	30	60	91,2%	94,4%	96,9%	97,1%	91,2%
14739	50	65	30	65	91,3%	94,3%	97,0%	97,3%	91,7%
14740	50	65	30	70	91,5%	94,3%	97,1%	97,5%	92,2%
14741	50	65	30	75	91,6%	94,2%	97,2%	97,7%	92,7%
14742	50	65	30	80	91,7%	94,1%	97,3%	97,8%	93,2%
14743	50	65	35	20	87,5%	93,7%	95,4%	94,8%	85,2%
14744	50	65	35	25	87,7%	93,6%	95,6%	95,2%	86,0%
14745	50	65	35	30	87,9%	93,5%	95,7%	95,5%	86,8%
14746	50	65	35	35	88,1%	93,5%	95,9%	95,8%	87,6%
14747	50	65	35	40	88,3%	93,4%	96,0%	96,0%	88,3%
14748	50	65	35	45	88,5%	93,3%	96,1%	96,3%	89,0%
14749	50	65	35	50	88,6%	93,2%	96,3%	96,5%	89,7%
14750	50	65	35	55	88,8%	93,1%	96,4%	96,8%	90,3%

14751	50	65	35	60	89,0%	93,0%	96,5%	97,0%	90,9%
14752	50	65	35	65	89,2%	93,0%	96,6%	97,2%	91,4%
14753	50	65	35	70	89,3%	92,9%	96,7%	97,3%	91,9%
14754	50	65	35	75	89,5%	92,8%	96,8%	97,5%	92,4%
14755	50	65	35	80	89,7%	92,7%	96,9%	97,7%	92,9%
14756	50	65	40	20	84,6%	92,2%	94,9%	94,5%	84,6%
14757	50	65	40	25	84,8%	92,1%	95,0%	94,9%	85,5%
14758	50	65	40	30	85,0%	92,0%	95,2%	95,2%	86,3%
14759	50	65	40	35	85,2%	91,9%	95,3%	95,5%	87,1%
14760	50	65	40	40	85,5%	91,8%	95,5%	95,8%	87,9%
14761	50	65	40	45	85,7%	91,7%	95,6%	96,1%	88,6%
14762	50	65	40	50	85,9%	91,6%	95,8%	96,3%	89,3%
14763	50	65	40	55	86,1%	91,5%	95,9%	96,6%	89,9%
14764	50	65	40	60	86,3%	91,4%	96,0%	96,8%	90,5%
14765	50	65	40	65	86,5%	91,3%	96,2%	97,0%	91,1%
14766	50	65	40	70	86,7%	91,2%	96,3%	97,2%	91,6%
14767	50	65	40	75	86,9%	91,1%	96,4%	97,4%	92,1%
14768	50	65	40	80	87,1%	91,0%	96,5%	97,5%	92,6%
14769	50	65	45	20	81,1%	90,4%	94,2%	94,2%	84,1%
14770	50	65	45	25	81,3%	90,2%	94,4%	94,6%	85,0%
14771	50	65	45	30	81,6%	90,1%	94,6%	94,9%	85,8%
14772	50	65	45	35	81,9%	90,0%	94,8%	95,2%	86,7%
14773	50	65	45	40	82,1%	89,9%	94,9%	95,5%	87,4%
14774	50	65	45	45	82,4%	89,8%	95,1%	95,8%	88,2%
14775	50	65	45	50	82,6%	89,6%	95,2%	96,1%	88,9%
14776	50	65	45	55	82,9%	89,5%	95,4%	96,3%	89,5%
14777	50	65	45	60	83,1%	89,4%	95,5%	96,6%	90,1%
14778	50	65	45	65	83,4%	89,3%	95,7%	96,8%	90,7%
14779	50	65	45	70	83,6%	89,1%	95,8%	97,0%	91,3%
14780	50	65	45	75	83,9%	89,0%	96,0%	97,2%	91,8%
14781	50	65	45	80	84,1%	88,9%	96,1%	97,4%	92,3%
14782	50	65	50	20	77,0%	88,1%	93,5%	93,9%	83,5%
14783	50	65	50	25	77,3%	88,0%	93,7%	94,3%	84,5%

14784	50	65	50	30	77,6%	87,9%	93,9%	94,6%	85,3%
14785	50	65	50	35	77,9%	87,7%	94,1%	95,0%	86,2%
14786	50	65	50	40	78,2%	87,6%	94,3%	95,3%	87,0%
14787	50	65	50	45	78,5%	87,4%	94,5%	95,6%	87,7%
14788	50	65	50	50	78,8%	87,3%	94,7%	95,9%	88,4%
14789	50	65	50	55	79,1%	87,2%	94,8%	96,1%	89,1%
14790	50	65	50	60	79,4%	87,0%	95,0%	96,4%	89,8%
14791	50	65	50	65	79,7%	86,9%	95,1%	96,6%	90,4%
14792	50	65	50	70	80,0%	86,7%	95,3%	96,8%	91,0%
14793	50	65	50	75	80,2%	86,6%	95,4%	97,0%	91,5%
14794	50	65	50	80	80,5%	86,4%	95,6%	97,2%	92,0%
14795	50	65	55	20	72,3%	85,5%	92,7%	93,5%	83,0%
14796	50	65	55	25	72,7%	85,3%	93,0%	93,9%	83,9%
14797	50	65	55	30	73,0%	85,2%	93,2%	94,3%	84,8%
14798	50	65	55	35	73,4%	85,0%	93,4%	94,7%	85,7%
14799	50	65	55	40	73,7%	84,8%	93,6%	95,0%	86,5%
14800	50	65	55	45	74,0%	84,7%	93,8%	95,3%	87,3%
14801	50	65	55	50	74,4%	84,5%	94,0%	95,6%	88,0%
14802	50	65	55	55	74,7%	84,3%	94,2%	95,9%	88,7%
14803	50	65	55	60	75,1%	84,2%	94,4%	96,2%	89,4%
14804	50	65	55	65	75,4%	84,0%	94,5%	96,4%	90,0%
14805	50	65	55	70	75,7%	83,8%	94,7%	96,6%	90,6%
14806	50	65	55	75	76,0%	83,6%	94,9%	96,9%	91,2%
14807	50	65	55	80	76,3%	83,5%	95,0%	97,1%	91,7%
14808	50	65	60	20	67,1%	82,4%	91,8%	93,1%	82,4%
14809	50	65	60	25	67,5%	82,2%	92,1%	93,6%	83,3%
14810	50	65	60	30	67,9%	82,0%	92,3%	94,0%	84,3%
14811	50	65	60	35	68,3%	81,8%	92,6%	94,3%	85,2%
14812	50	65	60	40	68,7%	81,6%	92,8%	94,7%	86,0%
14813	50	65	60	45	69,0%	81,4%	93,0%	95,0%	86,8%
14814	50	65	60	50	69,4%	81,2%	93,2%	95,3%	87,6%
14815	50	65	60	55	69,8%	81,0%	93,5%	95,6%	88,3%
14816	50	65	60	60	70,2%	80,8%	93,7%	95,9%	89,0%

14817	50	65	60	65	70,5%	80,6%	93,9%	96,2%	89,6%
14818	50	65	60	70	70,9%	80,4%	94,1%	96,4%	90,3%
14819	50	65	60	75	71,2%	80,2%	94,2%	96,7%	90,9%
14820	50	65	60	80	71,6%	80,0%	94,4%	96,9%	91,4%
14821	50	65	65	20	61,5%	78,8%	90,9%	92,7%	81,8%
14822	50	65	65	25	61,9%	78,6%	91,1%	93,2%	82,8%
14823	50	65	65	30	62,3%	78,4%	91,4%	93,6%	83,7%
14824	50	65	65	35	62,7%	78,1%	91,7%	94,0%	84,6%
14825	50	65	65	40	63,1%	77,9%	91,9%	94,4%	85,5%
14826	50	65	65	45	63,5%	77,7%	92,2%	94,7%	86,3%
14827	50	65	65	50	63,9%	77,5%	92,4%	95,1%	87,1%
14828	50	65	65	55	64,3%	77,2%	92,7%	95,4%	87,9%
14829	50	65	65	60	64,7%	77,0%	92,9%	95,7%	88,6%
14830	50	65	65	65	65,1%	76,8%	93,1%	96,0%	89,3%
14831	50	65	65	70	65,5%	76,5%	93,3%	96,2%	89,9%
14832	50	65	65	75	65,9%	76,3%	93,5%	96,5%	90,5%
14833	50	65	65	80	66,3%	76,1%	93,7%	96,7%	91,1%
14834	50	65	70	20	55,5%	74,7%	89,8%	92,3%	81,1%
14835	50	65	70	25	55,9%	74,4%	90,1%	92,8%	82,2%
14836	50	65	70	30	56,3%	74,2%	90,4%	93,2%	83,2%
14837	50	65	70	35	56,8%	73,9%	90,7%	93,7%	84,1%
14838	50	65	70	40	57,2%	73,7%	91,0%	94,1%	85,0%
14839	50	65	70	45	57,6%	73,4%	91,2%	94,4%	85,8%
14840	50	65	70	50	58,1%	73,2%	91,5%	94,8%	86,7%
14841	50	65	70	55	58,5%	72,9%	91,8%	95,1%	87,4%
14842	50	65	70	60	58,9%	72,7%	92,0%	95,4%	88,2%
14843	50	65	70	65	59,3%	72,4%	92,3%	95,7%	88,9%
14844	50	65	70	70	59,8%	72,1%	92,5%	96,0%	89,5%
14845	50	65	70	75	60,2%	71,9%	92,7%	96,2%	90,1%
14846	50	65	70	80	60,6%	71,6%	93,0%	96,5%	90,7%
14847	50	65	75	20	49,3%	70,1%	88,6%	91,9%	80,5%
14848	50	65	75	25	49,8%	69,8%	88,9%	92,4%	81,6%
14849	50	65	75	30	50,2%	69,5%	89,2%	92,8%	82,6%

14850	50	65	75	35	50,6%	69,3%	89,6%	93,3%	83,5%
14851	50	65	75	40	51,1%	69,0%	89,9%	93,7%	84,5%
14852	50	65	75	45	51,5%	68,7%	90,2%	94,1%	85,3%
14853	50	65	75	50	52,0%	68,4%	90,5%	94,5%	86,2%
14854	50	65	75	55	52,4%	68,1%	90,8%	94,8%	87,0%
14855	50	65	75	60	52,8%	67,8%	91,1%	95,1%	87,7%
14856	50	65	75	65	53,3%	67,6%	91,3%	95,5%	88,4%
14857	50	65	75	70	53,7%	67,3%	91,6%	95,7%	89,1%
14858	50	65	75	75	54,2%	67,0%	91,8%	96,0%	89,8%
14859	50	65	75	80	54,6%	66,7%	92,1%	96,3%	90,4%
14860	50	65	80	20	43,2%	65,0%	87,3%	91,4%	79,9%
14861	50	65	80	25	43,6%	64,7%	87,6%	91,9%	80,9%
14862	50	65	80	30	44,1%	64,4%	88,0%	92,4%	82,0%
14863	50	65	80	35	44,5%	64,1%	88,4%	92,9%	83,0%
14864	50	65	80	40	44,9%	63,8%	88,7%	93,3%	83,9%
14865	50	65	80	45	45,4%	63,5%	89,0%	93,7%	84,8%
14866	50	65	80	50	45,8%	63,2%	89,4%	94,1%	85,7%
14867	50	65	80	55	46,2%	62,9%	89,7%	94,5%	86,5%
14868	50	65	80	60	46,7%	62,6%	90,0%	94,9%	87,3%
14869	50	65	80	65	47,1%	62,3%	90,3%	95,2%	88,0%
14870	50	65	80	70	47,6%	62,0%	90,6%	95,5%	88,7%
14871	50	65	80	75	48,0%	61,7%	90,9%	95,8%	89,4%
14872	50	65	80	80	48,4%	61,4%	91,1%	96,0%	90,0%
14873	50	70	20	20	94,1%	97,0%	96,7%	95,3%	85,5%
14874	50	70	20	25	94,2%	96,9%	96,8%	95,6%	86,4%
14875	50	70	20	30	94,3%	96,9%	96,9%	95,9%	87,1%
14876	50	70	20	35	94,4%	96,9%	97,0%	96,2%	87,9%
14877	50	70	20	40	94,5%	96,8%	97,1%	96,4%	88,6%
14878	50	70	20	45	94,6%	96,8%	97,2%	96,7%	89,3%
14879	50	70	20	50	94,7%	96,7%	97,3%	96,9%	89,9%
14880	50	70	20	55	94,8%	96,7%	97,4%	97,1%	90,5%
14881	50	70	20	60	94,9%	96,7%	97,5%	97,3%	91,1%
14882	50	70	20	65	95,0%	96,6%	97,6%	97,4%	91,6%

14883	50	70	20	70	95,0%	96,6%	97,6%	97,6%	92,1%
14884	50	70	20	75	95,1%	96,5%	97,7%	97,8%	92,6%
14885	50	70	20	80	95,2%	96,5%	97,8%	97,9%	93,1%
14886	50	70	25	20	92,6%	96,2%	96,3%	95,1%	85,0%
14887	50	70	25	25	92,7%	96,2%	96,4%	95,4%	85,9%
14888	50	70	25	30	92,9%	96,1%	96,5%	95,7%	86,7%
14889	50	70	25	35	93,0%	96,1%	96,6%	96,0%	87,4%
14890	50	70	25	40	93,1%	96,0%	96,8%	96,2%	88,2%
14891	50	70	25	45	93,2%	96,0%	96,9%	96,5%	88,9%
14892	50	70	25	50	93,3%	95,9%	97,0%	96,7%	89,5%
14893	50	70	25	55	93,4%	95,9%	97,1%	96,9%	90,2%
14894	50	70	25	60	93,5%	95,8%	97,2%	97,1%	90,7%
14895	50	70	25	65	93,6%	95,8%	97,2%	97,3%	91,3%
14896	50	70	25	70	93,7%	95,7%	97,3%	97,5%	91,8%
14897	50	70	25	75	93,8%	95,7%	97,4%	97,6%	92,3%
14898	50	70	25	80	93,9%	95,6%	97,5%	97,8%	92,8%
14899	50	70	30	20	90,8%	95,3%	95,8%	94,8%	84,5%
14900	50	70	30	25	90,9%	95,2%	96,0%	95,1%	85,4%
14901	50	70	30	30	91,0%	95,2%	96,1%	95,4%	86,2%
14902	50	70	30	35	91,2%	95,1%	96,2%	95,7%	87,0%
14903	50	70	30	40	91,3%	95,1%	96,3%	96,0%	87,7%
14904	50	70	30	45	91,5%	95,0%	96,5%	96,2%	88,5%
14905	50	70	30	50	91,6%	94,9%	96,6%	96,5%	89,1%
14906	50	70	30	55	91,7%	94,9%	96,7%	96,7%	89,8%
14907	50	70	30	60	91,9%	94,8%	96,8%	96,9%	90,4%
14908	50	70	30	65	92,0%	94,7%	96,9%	97,1%	91,0%
14909	50	70	30	70	92,1%	94,7%	97,0%	97,3%	91,5%
14910	50	70	30	75	92,3%	94,6%	97,1%	97,5%	92,0%
14911	50	70	30	80	92,4%	94,5%	97,2%	97,6%	92,5%
14912	50	70	35	20	88,5%	94,1%	95,3%	94,5%	83,9%
14913	50	70	35	25	88,6%	94,1%	95,5%	94,8%	84,8%
14914	50	70	35	30	88,8%	94,0%	95,6%	95,1%	85,7%
14915	50	70	35	35	89,0%	93,9%	95,7%	95,5%	86,5%

14916	50	70	35	40	89,2%	93,9%	95,9%	95,7%	87,3%
14917	50	70	35	45	89,3%	93,8%	96,0%	96,0%	88,0%
14918	50	70	35	50	89,5%	93,7%	96,1%	96,3%	88,7%
14919	50	70	35	55	89,7%	93,6%	96,3%	96,5%	89,4%
14920	50	70	35	60	89,8%	93,5%	96,4%	96,7%	90,0%
14921	50	70	35	65	90,0%	93,5%	96,5%	96,9%	90,6%
14922	50	70	35	70	90,1%	93,4%	96,6%	97,1%	91,2%
14923	50	70	35	75	90,3%	93,3%	96,7%	97,3%	91,7%
14924	50	70	35	80	90,5%	93,2%	96,8%	97,5%	92,2%
14925	50	70	40	20	85,7%	92,7%	94,7%	94,1%	83,4%
14926	50	70	40	25	85,9%	92,6%	94,9%	94,5%	84,3%
14927	50	70	40	30	86,1%	92,6%	95,0%	94,9%	85,2%
14928	50	70	40	35	86,3%	92,5%	95,2%	95,2%	86,0%
14929	50	70	40	40	86,5%	92,4%	95,4%	95,5%	86,8%
14930	50	70	40	45	86,7%	92,3%	95,5%	95,8%	87,6%
14931	50	70	40	50	86,9%	92,2%	95,6%	96,0%	88,3%
14932	50	70	40	55	87,1%	92,1%	95,8%	96,3%	89,0%
14933	50	70	40	60	87,3%	92,0%	95,9%	96,5%	89,7%
14934	50	70	40	65	87,5%	91,9%	96,1%	96,8%	90,3%
14935	50	70	40	70	87,7%	91,8%	96,2%	97,0%	90,9%
14936	50	70	40	75	87,9%	91,7%	96,3%	97,2%	91,4%
14937	50	70	40	80	88,1%	91,6%	96,4%	97,4%	91,9%
14938	50	70	45	20	82,4%	91,0%	94,1%	93,8%	82,8%
14939	50	70	45	25	82,6%	90,9%	94,2%	94,2%	83,7%
14940	50	70	45	30	82,9%	90,8%	94,4%	94,5%	84,6%
14941	50	70	45	35	83,1%	90,7%	94,6%	94,9%	85,5%
14942	50	70	45	40	83,4%	90,6%	94,8%	95,2%	86,3%
14943	50	70	45	45	83,6%	90,5%	94,9%	95,5%	87,1%
14944	50	70	45	50	83,9%	90,4%	95,1%	95,8%	87,9%
14945	50	70	45	55	84,1%	90,2%	95,3%	96,1%	88,6%
14946	50	70	45	60	84,3%	90,1%	95,4%	96,3%	89,3%
14947	50	70	45	65	84,6%	90,0%	95,6%	96,6%	89,9%
14948	50	70	45	70	84,8%	89,9%	95,7%	96,8%	90,5%

14949	50	70	45	75	85,0%	89,8%	95,8%	97,0%	91,1%
14950	50	70	45	80	85,3%	89,6%	96,0%	97,2%	91,6%
14951	50	70	50	20	78,5%	88,9%	93,3%	93,4%	82,2%
14952	50	70	50	25	78,8%	88,8%	93,5%	93,8%	83,2%
14953	50	70	50	30	79,1%	88,7%	93,7%	94,2%	84,1%
14954	50	70	50	35	79,4%	88,5%	93,9%	94,6%	85,0%
14955	50	70	50	40	79,7%	88,4%	94,1%	94,9%	85,9%
14956	50	70	50	45	80,0%	88,3%	94,3%	95,3%	86,7%
14957	50	70	50	50	80,2%	88,1%	94,5%	95,6%	87,4%
14958	50	70	50	55	80,5%	88,0%	94,7%	95,8%	88,2%
14959	50	70	50	60	80,8%	87,9%	94,8%	96,1%	88,9%
14960	50	70	50	65	81,1%	87,7%	95,0%	96,4%	89,5%
14961	50	70	50	70	81,3%	87,6%	95,2%	96,6%	90,1%
14962	50	70	50	75	81,6%	87,4%	95,3%	96,8%	90,7%
14963	50	70	50	80	81,9%	87,3%	95,5%	97,0%	91,3%
14964	50	70	55	20	74,1%	86,5%	92,5%	93,0%	81,6%
14965	50	70	55	25	74,4%	86,3%	92,7%	93,5%	82,6%
14966	50	70	55	30	74,7%	86,1%	93,0%	93,9%	83,5%
14967	50	70	55	35	75,1%	86,0%	93,2%	94,3%	84,5%
14968	50	70	55	40	75,4%	85,8%	93,4%	94,6%	85,3%
14969	50	70	55	45	75,7%	85,7%	93,6%	95,0%	86,2%
14970	50	70	55	50	76,0%	85,5%	93,8%	95,3%	87,0%
14971	50	70	55	55	76,4%	85,3%	94,0%	95,6%	87,7%
14972	50	70	55	60	76,7%	85,2%	94,2%	95,9%	88,4%
14973	50	70	55	65	77,0%	85,0%	94,4%	96,1%	89,1%
14974	50	70	55	70	77,3%	84,9%	94,5%	96,4%	89,8%
14975	50	70	55	75	77,6%	84,7%	94,7%	96,6%	90,4%
14976	50	70	55	80	77,9%	84,5%	94,9%	96,8%	91,0%
14977	50	70	60	20	69,0%	83,5%	91,6%	92,6%	80,9%
14978	50	70	60	25	69,4%	83,3%	91,9%	93,1%	82,0%
14979	50	70	60	30	69,8%	83,2%	92,1%	93,5%	83,0%
14980	50	70	60	35	70,2%	83,0%	92,4%	93,9%	83,9%
14981	50	70	60	40	70,5%	82,8%	92,6%	94,3%	84,8%

14982	50	70	60	45	70,9%	82,6%	92,8%	94,7%	85,7%
14983	50	70	60	50	71,3%	82,4%	93,0%	95,0%	86,5%
14984	50	70	60	55	71,6%	82,2%	93,3%	95,3%	87,3%
14985	50	70	60	60	72,0%	82,0%	93,5%	95,6%	88,0%
14986	50	70	60	65	72,3%	81,8%	93,7%	95,9%	88,7%
14987	50	70	60	70	72,7%	81,6%	93,9%	96,2%	89,4%
14988	50	70	60	75	73,0%	81,4%	94,1%	96,4%	90,0%
14989	50	70	60	80	73,4%	81,2%	94,3%	96,6%	90,6%
14990	50	70	65	20	63,5%	80,1%	90,6%	92,2%	80,3%
14991	50	70	65	25	63,9%	79,9%	90,9%	92,7%	81,4%
14992	50	70	65	30	64,4%	79,7%	91,2%	93,1%	82,4%
14993	50	70	65	35	64,8%	79,5%	91,4%	93,6%	83,4%
14994	50	70	65	40	65,2%	79,2%	91,7%	94,0%	84,3%
14995	50	70	65	45	65,6%	79,0%	91,9%	94,4%	85,2%
14996	50	70	65	50	66,0%	78,8%	92,2%	94,7%	86,0%
14997	50	70	65	55	66,3%	78,6%	92,4%	95,0%	86,8%
14998	50	70	65	60	66,7%	78,4%	92,7%	95,4%	87,6%
14999	50	70	65	65	67,1%	78,1%	92,9%	95,7%	88,3%
15000	50	70	65	70	67,5%	77,9%	93,1%	95,9%	89,0%
15001	50	70	65	75	67,9%	77,7%	93,3%	96,2%	89,6%
15002	50	70	65	80	68,3%	77,5%	93,5%	96,4%	90,3%
15003	50	70	70	20	57,7%	76,2%	89,5%	91,8%	79,6%
15004	50	70	70	25	58,1%	75,9%	89,8%	92,3%	80,7%
15005	50	70	70	30	58,5%	75,7%	90,1%	92,7%	81,8%
15006	50	70	70	35	58,9%	75,4%	90,4%	93,2%	82,8%
15007	50	70	70	40	59,4%	75,2%	90,7%	93,6%	83,7%
15008	50	70	70	45	59,8%	74,9%	91,0%	94,0%	84,6%
15009	50	70	70	50	60,2%	74,7%	91,3%	94,4%	85,5%
15010	50	70	70	55	60,6%	74,4%	91,5%	94,8%	86,3%
15011	50	70	70	60	61,1%	74,2%	91,8%	95,1%	87,1%
15012	50	70	70	65	61,5%	73,9%	92,0%	95,4%	87,9%
15013	50	70	70	70	61,9%	73,7%	92,3%	95,7%	88,6%
15014	50	70	70	75	62,3%	73,4%	92,5%	96,0%	89,3%

15015	50	70	70	80	62,7%	73,2%	92,7%	96,2%	89,9%
15016	50	70	75	20	51,5%	71,7%	88,3%	91,3%	79,0%
15017	50	70	75	25	52,0%	71,4%	88,6%	91,8%	80,1%
15018	50	70	75	30	52,4%	71,2%	88,9%	92,3%	81,1%
15019	50	70	75	35	52,9%	70,9%	89,3%	92,8%	82,2%
15020	50	70	75	40	53,3%	70,6%	89,6%	93,2%	83,2%
15021	50	70	75	45	53,7%	70,4%	89,9%	93,7%	84,1%
15022	50	70	75	50	54,2%	70,1%	90,2%	94,1%	85,0%
15023	50	70	75	55	54,6%	69,8%	90,5%	94,4%	85,8%
15024	50	70	75	60	55,0%	69,5%	90,8%	94,8%	86,7%
15025	50	70	75	65	55,5%	69,3%	91,1%	95,1%	87,4%
15026	50	70	75	70	55,9%	69,0%	91,3%	95,4%	88,2%
15027	50	70	75	75	56,4%	68,7%	91,6%	95,7%	88,9%
15028	50	70	75	80	56,8%	68,4%	91,9%	96,0%	89,5%
15029	50	70	80	20	45,4%	66,8%	86,9%	90,8%	78,3%
15030	50	70	80	25	45,8%	66,5%	87,3%	91,4%	79,4%
15031	50	70	80	30	46,3%	66,2%	87,7%	91,9%	80,5%
15032	50	70	80	35	46,7%	65,9%	88,0%	92,4%	81,6%
15033	50	70	80	40	47,1%	65,6%	88,4%	92,9%	82,6%
15034	50	70	80	45	47,6%	65,3%	88,7%	93,3%	83,5%
15035	50	70	80	50	48,0%	65,0%	89,1%	93,7%	84,5%
15036	50	70	80	55	48,5%	64,7%	89,4%	94,1%	85,3%
15037	50	70	80	60	48,9%	64,4%	89,7%	94,5%	86,2%
15038	50	70	80	65	49,3%	64,1%	90,0%	94,8%	87,0%
15039	50	70	80	70	49,8%	63,8%	90,3%	95,2%	87,7%
15040	50	70	80	75	50,2%	63,5%	90,6%	95,5%	88,4%
15041	50	70	80	80	50,7%	63,2%	90,9%	95,8%	89,1%
15042	50	75	20	20	94,6%	97,2%	96,6%	95,0%	84,3%
15043	50	75	20	25	94,7%	97,2%	96,7%	95,3%	85,2%
15044	50	75	20	30	94,8%	97,1%	96,8%	95,6%	86,0%
15045	50	75	20	35	94,9%	97,1%	96,9%	95,9%	86,8%
15046	50	75	20	40	95,0%	97,1%	97,0%	96,2%	87,6%
15047	50	75	20	45	95,0%	97,0%	97,1%	96,4%	88,3%

15048	50	75	20	50	95,1%	97,0%	97,2%	96,6%	89,0%
15049	50	75	20	55	95,2%	96,9%	97,3%	96,9%	89,7%
15050	50	75	20	60	95,3%	96,9%	97,4%	97,1%	90,3%
15051	50	75	20	65	95,4%	96,9%	97,5%	97,3%	90,9%
15052	50	75	20	70	95,4%	96,8%	97,6%	97,4%	91,4%
15053	50	75	20	75	95,5%	96,8%	97,6%	97,6%	91,9%
15054	50	75	20	80	95,6%	96,7%	97,7%	97,8%	92,4%
15055	50	75	25	20	93,2%	96,5%	96,2%	94,7%	83,7%
15056	50	75	25	25	93,3%	96,5%	96,3%	95,0%	84,7%
15057	50	75	25	30	93,4%	96,4%	96,4%	95,4%	85,5%
15058	50	75	25	35	93,5%	96,4%	96,5%	95,7%	86,4%
15059	50	75	25	40	93,6%	96,3%	96,7%	95,9%	87,1%
15060	50	75	25	45	93,7%	96,3%	96,8%	96,2%	87,9%
15061	50	75	25	50	93,8%	96,2%	96,9%	96,4%	88,6%
15062	50	75	25	55	93,9%	96,2%	97,0%	96,7%	89,3%
15063	50	75	25	60	94,0%	96,1%	97,1%	96,9%	89,9%
15064	50	75	25	65	94,1%	96,1%	97,2%	97,1%	90,5%
15065	50	75	25	70	94,2%	96,0%	97,3%	97,3%	91,1%
15066	50	75	25	75	94,3%	96,0%	97,3%	97,5%	91,6%
15067	50	75	25	80	94,4%	95,9%	97,4%	97,6%	92,1%
15068	50	75	30	20	91,5%	95,6%	95,7%	94,4%	83,2%
15069	50	75	30	25	91,6%	95,6%	95,8%	94,8%	84,1%
15070	50	75	30	30	91,7%	95,5%	96,0%	95,1%	85,0%
15071	50	75	30	35	91,9%	95,5%	96,1%	95,4%	85,9%
15072	50	75	30	40	92,0%	95,4%	96,2%	95,7%	86,7%
15073	50	75	30	45	92,1%	95,4%	96,3%	96,0%	87,4%
15074	50	75	30	50	92,3%	95,3%	96,5%	96,2%	88,2%
15075	50	75	30	55	92,4%	95,2%	96,6%	96,5%	88,9%
15076	50	75	30	60	92,5%	95,2%	96,7%	96,7%	89,5%
15077	50	75	30	65	92,6%	95,1%	96,8%	96,9%	90,2%
15078	50	75	30	70	92,7%	95,1%	96,9%	97,1%	90,7%
15079	50	75	30	75	92,9%	95,0%	97,0%	97,3%	91,3%
15080	50	75	30	80	93,0%	94,9%	97,1%	97,5%	91,8%

15081	50	75	35	20	89,3%	94,6%	95,2%	94,1%	82,6%
15082	50	75	35	25	89,5%	94,5%	95,3%	94,4%	83,6%
15083	50	75	35	30	89,7%	94,4%	95,5%	94,8%	84,5%
15084	50	75	35	35	89,8%	94,4%	95,6%	95,1%	85,4%
15085	50	75	35	40	90,0%	94,3%	95,7%	95,4%	86,2%
15086	50	75	35	45	90,1%	94,2%	95,9%	95,7%	87,0%
15087	50	75	35	50	90,3%	94,1%	96,0%	96,0%	87,7%
15088	50	75	35	55	90,5%	94,1%	96,1%	96,3%	88,5%
15089	50	75	35	60	90,6%	94,0%	96,3%	96,5%	89,1%
15090	50	75	35	65	90,8%	93,9%	96,4%	96,7%	89,8%
15091	50	75	35	70	90,9%	93,9%	96,5%	96,9%	90,4%
15092	50	75	35	75	91,0%	93,8%	96,6%	97,1%	91,0%
15093	50	75	35	80	91,2%	93,7%	96,7%	97,3%	91,5%
15094	50	75	40	20	86,7%	93,2%	94,6%	93,7%	82,0%
15095	50	75	40	25	86,9%	93,2%	94,7%	94,1%	83,0%
15096	50	75	40	30	87,1%	93,1%	94,9%	94,5%	83,9%
15097	50	75	40	35	87,3%	93,0%	95,1%	94,8%	84,8%
15098	50	75	40	40	87,5%	92,9%	95,2%	95,2%	85,7%
15099	50	75	40	45	87,7%	92,8%	95,4%	95,5%	86,5%
15100	50	75	40	50	87,9%	92,7%	95,5%	95,8%	87,3%
15101	50	75	40	55	88,1%	92,6%	95,7%	96,0%	88,0%
15102	50	75	40	60	88,3%	92,6%	95,8%	96,3%	88,7%
15103	50	75	40	65	88,5%	92,5%	95,9%	96,5%	89,4%
15104	50	75	40	70	88,6%	92,4%	96,1%	96,7%	90,0%
15105	50	75	40	75	88,8%	92,3%	96,2%	97,0%	90,6%
15106	50	75	40	80	89,0%	92,2%	96,3%	97,2%	91,2%
15107	50	75	45	20	83,6%	91,6%	93,9%	93,3%	81,4%
15108	50	75	45	25	83,9%	91,5%	94,1%	93,8%	82,4%
15109	50	75	45	30	84,1%	91,4%	94,3%	94,2%	83,4%
15110	50	75	45	35	84,4%	91,3%	94,4%	94,5%	84,3%
15111	50	75	45	40	84,6%	91,2%	94,6%	94,9%	85,2%
15112	50	75	45	45	84,8%	91,1%	94,8%	95,2%	86,0%
15113	50	75	45	50	85,0%	91,0%	95,0%	95,5%	86,8%

15114	50	75	45	55	85,3%	90,9%	95,1%	95,8%	87,6%
15115	50	75	45	60	85,5%	90,8%	95,3%	96,1%	88,3%
15116	50	75	45	65	85,7%	90,7%	95,4%	96,3%	89,0%
15117	50	75	45	70	85,9%	90,6%	95,6%	96,5%	89,7%
15118	50	75	45	75	86,1%	90,5%	95,7%	96,8%	90,3%
15119	50	75	45	80	86,3%	90,4%	95,8%	97,0%	90,9%
15120	50	75	50	20	80,0%	89,7%	93,1%	93,0%	80,7%
15121	50	75	50	25	80,3%	89,6%	93,3%	93,4%	81,8%
15122	50	75	50	30	80,5%	89,4%	93,5%	93,8%	82,8%
15123	50	75	50	35	80,8%	89,3%	93,7%	94,2%	83,7%
15124	50	75	50	40	81,1%	89,2%	93,9%	94,6%	84,7%
15125	50	75	50	45	81,3%	89,1%	94,1%	94,9%	85,5%
15126	50	75	50	50	81,6%	88,9%	94,3%	95,2%	86,3%
15127	50	75	50	55	81,9%	88,8%	94,5%	95,5%	87,1%
15128	50	75	50	60	82,1%	88,7%	94,7%	95,8%	87,9%
15129	50	75	50	65	82,4%	88,6%	94,8%	96,1%	88,6%
15130	50	75	50	70	82,7%	88,4%	95,0%	96,3%	89,3%
15131	50	75	50	75	82,9%	88,3%	95,2%	96,6%	89,9%
15132	50	75	50	80	83,2%	88,1%	95,3%	96,8%	90,5%
15133	50	75	55	20	75,7%	87,4%	92,3%	92,5%	80,1%
15134	50	75	55	25	76,1%	87,2%	92,5%	93,0%	81,2%
15135	50	75	55	30	76,4%	87,1%	92,8%	93,4%	82,2%
15136	50	75	55	35	76,7%	86,9%	93,0%	93,9%	83,2%
15137	50	75	55	40	77,0%	86,8%	93,2%	94,2%	84,1%
15138	50	75	55	45	77,3%	86,6%	93,4%	94,6%	85,0%
15139	50	75	55	50	77,6%	86,5%	93,6%	94,9%	85,9%
15140	50	75	55	55	77,9%	86,3%	93,8%	95,3%	86,7%
15141	50	75	55	60	78,2%	86,2%	94,0%	95,6%	87,4%
15142	50	75	55	65	78,5%	86,0%	94,2%	95,9%	88,2%
15143	50	75	55	70	78,8%	85,8%	94,4%	96,1%	88,9%
15144	50	75	55	75	79,1%	85,7%	94,6%	96,4%	89,5%
15145	50	75	55	80	79,4%	85,5%	94,7%	96,6%	90,2%
15146	50	75	60	20	70,9%	84,6%	91,4%	92,1%	79,4%

15147	50	75	60	25	71,3%	84,4%	91,6%	92,6%	80,5%
15148	50	75	60	30	71,6%	84,2%	91,9%	93,1%	81,6%
15149	50	75	60	35	72,0%	84,1%	92,1%	93,5%	82,6%
15150	50	75	60	40	72,3%	83,9%	92,4%	93,9%	83,5%
15151	50	75	60	45	72,7%	83,7%	92,6%	94,3%	84,5%
15152	50	75	60	50	73,0%	83,5%	92,8%	94,6%	85,3%
15153	50	75	60	55	73,4%	83,3%	93,1%	95,0%	86,2%
15154	50	75	60	60	73,7%	83,2%	93,3%	95,3%	87,0%
15155	50	75	60	65	74,1%	83,0%	93,5%	95,6%	87,7%
15156	50	75	60	70	74,4%	82,8%	93,7%	95,9%	88,5%
15157	50	75	60	75	74,7%	82,6%	93,9%	96,1%	89,1%
15158	50	75	60	80	75,1%	82,4%	94,1%	96,4%	89,8%
15159	50	75	65	20	65,6%	81,3%	90,3%	91,7%	78,7%
15160	50	75	65	25	66,0%	81,1%	90,6%	92,2%	79,9%
15161	50	75	65	30	66,4%	80,9%	90,9%	92,7%	81,0%
15162	50	75	65	35	66,8%	80,7%	91,2%	93,1%	82,0%
15163	50	75	65	40	67,1%	80,5%	91,4%	93,5%	83,0%
15164	50	75	65	45	67,5%	80,3%	91,7%	93,9%	83,9%
15165	50	75	65	50	67,9%	80,1%	92,0%	94,3%	84,8%
15166	50	75	65	55	68,3%	79,9%	92,2%	94,7%	85,7%
15167	50	75	65	60	68,7%	79,7%	92,5%	95,0%	86,5%
15168	50	75	65	65	69,1%	79,5%	92,7%	95,3%	87,3%
15169	50	75	65	70	69,4%	79,2%	92,9%	95,6%	88,0%
15170	50	75	65	75	69,8%	79,0%	93,1%	95,9%	88,7%
15171	50	75	65	80	70,2%	78,8%	93,3%	96,2%	89,4%
15172	50	75	70	20	59,8%	77,6%	89,2%	91,2%	78,0%
15173	50	75	70	25	60,2%	77,3%	89,5%	91,7%	79,2%
15174	50	75	70	30	60,6%	77,1%	89,8%	92,2%	80,3%
15175	50	75	70	35	61,1%	76,9%	90,1%	92,7%	81,4%
15176	50	75	70	40	61,5%	76,6%	90,4%	93,2%	82,4%
15177	50	75	70	45	61,9%	76,4%	90,7%	93,6%	83,4%
15178	50	75	70	50	62,3%	76,2%	91,0%	94,0%	84,3%
15179	50	75	70	55	62,7%	75,9%	91,3%	94,4%	85,2%

15180	50	75	70	60	63,1%	75,7%	91,5%	94,7%	86,0%
15181	50	75	70	65	63,5%	75,4%	91,8%	95,1%	86,8%
15182	50	75	70	70	64,0%	75,2%	92,0%	95,4%	87,6%
15183	50	75	70	75	64,4%	74,9%	92,3%	95,7%	88,3%
15184	50	75	70	80	64,8%	74,7%	92,5%	95,9%	89,0%
15185	50	75	75	20	53,7%	73,3%	87,9%	90,7%	77,3%
15186	50	75	75	25	54,2%	73,0%	88,3%	91,2%	78,5%
15187	50	75	75	30	54,6%	72,8%	88,6%	91,8%	79,6%
15188	50	75	75	35	55,1%	72,5%	89,0%	92,3%	80,7%
15189	50	75	75	40	55,5%	72,2%	89,3%	92,8%	81,8%
15190	50	75	75	45	55,9%	72,0%	89,6%	93,2%	82,8%
15191	50	75	75	50	56,4%	71,7%	89,9%	93,6%	83,7%
15192	50	75	75	55	56,8%	71,4%	90,2%	94,0%	84,6%
15193	50	75	75	60	57,2%	71,2%	90,5%	94,4%	85,5%
15194	50	75	75	65	57,7%	70,9%	90,8%	94,8%	86,3%
15195	50	75	75	70	58,1%	70,6%	91,1%	95,1%	87,1%
15196	50	75	75	75	58,5%	70,4%	91,4%	95,4%	87,9%
15197	50	75	75	80	58,9%	70,1%	91,6%	95,7%	88,6%
15198	50	75	80	20	47,6%	68,5%	86,5%	90,2%	76,6%
15199	50	75	80	25	48,0%	68,2%	86,9%	90,8%	77,8%
15200	50	75	80	30	48,5%	68,0%	87,3%	91,3%	79,0%
15201	50	75	80	35	48,9%	67,7%	87,7%	91,8%	80,1%
15202	50	75	80	40	49,3%	67,4%	88,0%	92,3%	81,2%
15203	50	75	80	45	49,8%	67,1%	88,4%	92,8%	82,2%
15204	50	75	80	50	50,2%	66,8%	88,7%	93,3%	83,2%
15205	50	75	80	55	50,7%	66,5%	89,1%	93,7%	84,1%
15206	50	75	80	60	51,1%	66,2%	89,4%	94,1%	85,0%
15207	50	75	80	65	51,5%	65,9%	89,7%	94,5%	85,8%
15208	50	75	80	70	52,0%	65,6%	90,0%	94,8%	86,7%
15209	50	75	80	75	52,4%	65,3%	90,3%	95,1%	87,4%
15210	50	75	80	80	52,9%	65,0%	90,6%	95,4%	88,2%
15211	50	80	20	20	95,0%	97,4%	96,5%	94,6%	83,0%
15212	50	80	20	25	95,1%	97,4%	96,6%	95,0%	83,9%

15213	50	80	20	30	95,2%	97,3%	96,7%	95,3%	84,8%
15214	50	80	20	35	95,3%	97,3%	96,8%	95,6%	85,7%
15215	50	80	20	40	95,4%	97,3%	96,9%	95,9%	86,5%
15216	50	80	20	45	95,4%	97,2%	97,0%	96,1%	87,3%
15217	50	80	20	50	95,5%	97,2%	97,1%	96,4%	88,0%
15218	50	80	20	55	95,6%	97,2%	97,2%	96,6%	88,7%
15219	50	80	20	60	95,7%	97,1%	97,3%	96,8%	89,4%
15220	50	80	20	65	95,7%	97,1%	97,4%	97,0%	90,0%
15221	50	80	20	70	95,8%	97,1%	97,5%	97,2%	90,6%
15222	50	80	20	75	95,9%	97,0%	97,6%	97,4%	91,2%
15223	50	80	20	80	96,0%	97,0%	97,7%	97,6%	91,7%
15224	50	80	25	20	93,7%	96,8%	96,1%	94,3%	82,4%
15225	50	80	25	25	93,9%	96,7%	96,2%	94,7%	83,4%
15226	50	80	25	30	94,0%	96,7%	96,3%	95,0%	84,3%
15227	50	80	25	35	94,1%	96,6%	96,4%	95,3%	85,2%
15228	50	80	25	40	94,2%	96,6%	96,5%	95,6%	86,0%
15229	50	80	25	45	94,2%	96,5%	96,7%	95,9%	86,8%
15230	50	80	25	50	94,3%	96,5%	96,8%	96,2%	87,6%
15231	50	80	25	55	94,4%	96,5%	96,9%	96,4%	88,3%
15232	50	80	25	60	94,5%	96,4%	97,0%	96,7%	89,0%
15233	50	80	25	65	94,6%	96,4%	97,1%	96,9%	89,7%
15234	50	80	25	70	94,7%	96,3%	97,2%	97,1%	90,3%
15235	50	80	25	75	94,8%	96,3%	97,3%	97,3%	90,9%
15236	50	80	25	80	94,9%	96,2%	97,3%	97,4%	91,4%
15237	50	80	30	20	92,1%	96,0%	95,6%	94,0%	81,8%
15238	50	80	30	25	92,3%	95,9%	95,7%	94,4%	82,8%
15239	50	80	30	30	92,4%	95,9%	95,8%	94,7%	83,8%
15240	50	80	30	35	92,5%	95,8%	96,0%	95,1%	84,7%
15241	50	80	30	40	92,6%	95,7%	96,1%	95,4%	85,5%
15242	50	80	30	45	92,8%	95,7%	96,2%	95,7%	86,4%
15243	50	80	30	50	92,9%	95,6%	96,4%	95,9%	87,1%
15244	50	80	30	55	93,0%	95,6%	96,5%	96,2%	87,9%
15245	50	80	30	60	93,1%	95,5%	96,6%	96,4%	88,6%

15246	50	80	30	65	93,2%	95,5%	96,7%	96,7%	89,3%
15247	50	80	30	70	93,3%	95,4%	96,8%	96,9%	89,9%
15248	50	80	30	75	93,4%	95,4%	96,9%	97,1%	90,5%
15249	50	80	30	80	93,5%	95,3%	97,0%	97,3%	91,1%
15250	50	80	35	20	90,2%	95,0%	95,0%	93,6%	81,2%
15251	50	80	35	25	90,3%	94,9%	95,2%	94,0%	82,2%
15252	50	80	35	30	90,5%	94,8%	95,3%	94,4%	83,2%
15253	50	80	35	35	90,6%	94,8%	95,5%	94,8%	84,1%
15254	50	80	35	40	90,8%	94,7%	95,6%	95,1%	85,0%
15255	50	80	35	45	90,9%	94,6%	95,8%	95,4%	85,9%
15256	50	80	35	50	91,1%	94,6%	95,9%	95,7%	86,7%
15257	50	80	35	55	91,2%	94,5%	96,0%	96,0%	87,4%
15258	50	80	35	60	91,3%	94,4%	96,2%	96,2%	88,2%
15259	50	80	35	65	91,5%	94,4%	96,3%	96,5%	88,9%
15260	50	80	35	70	91,6%	94,3%	96,4%	96,7%	89,5%
15261	50	80	35	75	91,7%	94,2%	96,5%	96,9%	90,2%
15262	50	80	35	80	91,9%	94,1%	96,6%	97,1%	90,8%
15263	50	80	40	20	87,7%	93,7%	94,4%	93,3%	80,5%
15264	50	80	40	25	87,9%	93,6%	94,6%	93,7%	81,6%
15265	50	80	40	30	88,1%	93,6%	94,7%	94,1%	82,6%
15266	50	80	40	35	88,3%	93,5%	94,9%	94,5%	83,6%
15267	50	80	40	40	88,5%	93,4%	95,1%	94,8%	84,5%
15268	50	80	40	45	88,6%	93,3%	95,2%	95,1%	85,4%
15269	50	80	40	50	88,8%	93,2%	95,4%	95,4%	86,2%
15270	50	80	40	55	89,0%	93,2%	95,5%	95,7%	87,0%
15271	50	80	40	60	89,2%	93,1%	95,7%	96,0%	87,7%
15272	50	80	40	65	89,3%	93,0%	95,8%	96,3%	88,5%
15273	50	80	40	70	89,5%	92,9%	95,9%	96,5%	89,1%
15274	50	80	40	75	89,7%	92,8%	96,1%	96,7%	89,8%
15275	50	80	40	80	89,8%	92,7%	96,2%	96,9%	90,4%
15276	50	80	45	20	84,8%	92,2%	93,7%	92,9%	79,9%
15277	50	80	45	25	85,0%	92,1%	93,9%	93,3%	81,0%
15278	50	80	45	30	85,3%	92,0%	94,1%	93,7%	82,0%

15279	50	80	45	35	85,5%	91,9%	94,3%	94,1%	83,0%
15280	50	80	45	40	85,7%	91,8%	94,5%	94,5%	83,9%
15281	50	80	45	45	85,9%	91,7%	94,6%	94,8%	84,8%
15282	50	80	45	50	86,1%	91,6%	94,8%	95,2%	85,7%
15283	50	80	45	55	86,3%	91,5%	95,0%	95,5%	86,5%
15284	50	80	45	60	86,5%	91,4%	95,1%	95,8%	87,3%
15285	50	80	45	65	86,8%	91,3%	95,3%	96,0%	88,0%
15286	50	80	45	70	87,0%	91,2%	95,4%	96,3%	88,7%
15287	50	80	45	75	87,2%	91,1%	95,6%	96,5%	89,4%
15288	50	80	45	80	87,3%	91,0%	95,7%	96,8%	90,0%
15289	50	80	50	20	81,4%	90,4%	92,9%	92,5%	79,2%
15290	50	80	50	25	81,6%	90,3%	93,1%	92,9%	80,3%
15291	50	80	50	30	81,9%	90,2%	93,4%	93,4%	81,4%
15292	50	80	50	35	82,1%	90,1%	93,6%	93,8%	82,4%
15293	50	80	50	40	82,4%	89,9%	93,8%	94,2%	83,4%
15294	50	80	50	45	82,7%	89,8%	94,0%	94,5%	84,3%
15295	50	80	50	50	82,9%	89,7%	94,1%	94,9%	85,2%
15296	50	80	50	55	83,2%	89,6%	94,3%	95,2%	86,0%
15297	50	80	50	60	83,4%	89,5%	94,5%	95,5%	86,8%
15298	50	80	50	65	83,6%	89,3%	94,7%	95,8%	87,6%
15299	50	80	50	70	83,9%	89,2%	94,9%	96,1%	88,3%
15300	50	80	50	75	84,1%	89,1%	95,0%	96,3%	89,0%
15301	50	80	50	80	84,4%	88,9%	95,2%	96,6%	89,7%
15302	50	80	55	20	77,3%	88,2%	92,1%	92,0%	78,5%
15303	50	80	55	25	77,6%	88,1%	92,3%	92,5%	79,7%
15304	50	80	55	30	77,9%	87,9%	92,5%	93,0%	80,7%
15305	50	80	55	35	78,2%	87,8%	92,8%	93,4%	81,8%
15306	50	80	55	40	78,5%	87,6%	93,0%	93,8%	82,8%
15307	50	80	55	45	78,8%	87,5%	93,2%	94,2%	83,7%
15308	50	80	55	50	79,1%	87,4%	93,4%	94,6%	84,7%
15309	50	80	55	55	79,4%	87,2%	93,6%	94,9%	85,5%
15310	50	80	55	60	79,7%	87,1%	93,8%	95,2%	86,4%
15311	50	80	55	65	80,0%	86,9%	94,0%	95,5%	87,1%

15312	50	80	55	70	80,3%	86,8%	94,2%	95,8%	87,9%
15313	50	80	55	75	80,5%	86,6%	94,4%	96,1%	88,6%
15314	50	80	55	80	80,8%	86,5%	94,6%	96,3%	89,3%
15315	50	80	60	20	72,7%	85,6%	91,1%	91,6%	77,8%
15316	50	80	60	25	73,1%	85,4%	91,4%	92,1%	79,0%
15317	50	80	60	30	73,4%	85,2%	91,6%	92,6%	80,1%
15318	50	80	60	35	73,7%	85,1%	91,9%	93,0%	81,2%
15319	50	80	60	40	74,1%	84,9%	92,1%	93,5%	82,2%
15320	50	80	60	45	74,4%	84,7%	92,4%	93,9%	83,2%
15321	50	80	60	50	74,8%	84,6%	92,6%	94,3%	84,1%
15322	50	80	60	55	75,1%	84,4%	92,8%	94,6%	85,0%
15323	50	80	60	60	75,4%	84,2%	93,1%	95,0%	85,9%
15324	50	80	60	65	75,7%	84,1%	93,3%	95,3%	86,7%
15325	50	80	60	70	76,1%	83,9%	93,5%	95,6%	87,4%
15326	50	80	60	75	76,4%	83,7%	93,7%	95,9%	88,2%
15327	50	80	60	80	76,7%	83,5%	93,9%	96,1%	88,9%
15328	50	80	65	20	67,5%	82,5%	90,0%	91,1%	77,1%
15329	50	80	65	25	67,9%	82,3%	90,3%	91,6%	78,3%
15330	50	80	65	30	68,3%	82,1%	90,6%	92,1%	79,4%
15331	50	80	65	35	68,7%	81,9%	90,9%	92,6%	80,5%
15332	50	80	65	40	69,1%	81,7%	91,2%	93,1%	81,6%
15333	50	80	65	45	69,4%	81,5%	91,5%	93,5%	82,6%
15334	50	80	65	50	69,8%	81,3%	91,7%	93,9%	83,6%
15335	50	80	65	55	70,2%	81,1%	92,0%	94,3%	84,5%
15336	50	80	65	60	70,6%	80,9%	92,2%	94,7%	85,3%
15337	50	80	65	65	70,9%	80,7%	92,5%	95,0%	86,2%
15338	50	80	65	70	71,3%	80,5%	92,7%	95,3%	87,0%
15339	50	80	65	75	71,6%	80,3%	92,9%	95,6%	87,7%
15340	50	80	65	80	72,0%	80,1%	93,1%	95,9%	88,5%
15341	50	80	70	20	61,9%	78,9%	88,9%	90,6%	76,4%
15342	50	80	70	25	62,3%	78,7%	89,2%	91,1%	77,6%
15343	50	80	70	30	62,7%	78,5%	89,5%	91,7%	78,8%
15344	50	80	70	35	63,2%	78,2%	89,8%	92,2%	79,9%

15345	50	80	70	40	63,6%	78,0%	90,1%	92,7%	81,0%
15346	50	80	70	45	64,0%	77,8%	90,4%	93,1%	82,0%
15347	50	80	70	50	64,4%	77,6%	90,7%	93,6%	83,0%
15348	50	80	70	55	64,8%	77,3%	91,0%	94,0%	83,9%
15349	50	80	70	60	65,2%	77,1%	91,3%	94,3%	84,8%
15350	50	80	70	65	65,6%	76,9%	91,6%	94,7%	85,7%
15351	50	80	70	70	66,0%	76,6%	91,8%	95,0%	86,5%
15352	50	80	70	75	66,4%	76,4%	92,1%	95,4%	87,3%
15353	50	80	70	80	66,8%	76,2%	92,3%	95,6%	88,0%
15354	50	80	75	20	55,9%	74,8%	87,6%	90,0%	75,6%
15355	50	80	75	25	56,4%	74,5%	87,9%	90,6%	76,9%
15356	50	80	75	30	56,8%	74,3%	88,3%	91,2%	78,1%
15357	50	80	75	35	57,2%	74,0%	88,6%	91,7%	79,2%
15358	50	80	75	40	57,7%	73,8%	89,0%	92,3%	80,3%
15359	50	80	75	45	58,1%	73,5%	89,3%	92,7%	81,4%
15360	50	80	75	50	58,5%	73,3%	89,6%	93,2%	82,4%
15361	50	80	75	55	59,0%	73,0%	89,9%	93,6%	83,4%
15362	50	80	75	60	59,4%	72,8%	90,2%	94,0%	84,3%
15363	50	80	75	65	59,8%	72,5%	90,5%	94,4%	85,2%
15364	50	80	75	70	60,2%	72,2%	90,8%	94,7%	86,0%
15365	50	80	75	75	60,7%	72,0%	91,1%	95,1%	86,8%
15366	50	80	75	80	61,1%	71,7%	91,4%	95,4%	87,6%
15367	50	80	80	20	49,8%	70,2%	86,2%	89,5%	74,8%
15368	50	80	80	25	50,2%	69,9%	86,6%	90,1%	76,1%
15369	50	80	80	30	50,7%	69,6%	87,0%	90,7%	77,3%
15370	50	80	80	35	51,1%	69,4%	87,3%	91,3%	78,5%
15371	50	80	80	40	51,6%	69,1%	87,7%	91,8%	79,6%
15372	50	80	80	45	52,0%	68,8%	88,1%	92,3%	80,7%
15373	50	80	80	50	52,4%	68,5%	88,4%	92,8%	81,8%
15374	50	80	80	55	52,9%	68,2%	88,8%	93,2%	82,8%
15375	50	80	80	60	53,3%	68,0%	89,1%	93,7%	83,7%
15376	50	80	80	65	53,8%	67,7%	89,4%	94,1%	84,6%
15377	50	80	80	70	54,2%	67,4%	89,7%	94,4%	85,5%

15378	50	80	80	75	54,6%	67,1%	90,0%	94,8%	86,3%
15379	50	80	80	80	55,1%	66,8%	90,3%	95,1%	87,1%
15380	55	20	20	20	89,4%	95,3%	98,4%	98,5%	95,4%
15381	55	20	20	25	89,6%	95,3%	98,5%	98,6%	95,7%
15382	55	20	20	30	89,8%	95,2%	98,5%	98,7%	96,0%
15383	55	20	20	35	89,9%	95,2%	98,6%	98,8%	96,2%
15384	55	20	20	40	90,1%	95,1%	98,6%	98,8%	96,5%
15385	55	20	20	45	90,2%	95,0%	98,6%	98,9%	96,7%
15386	55	20	20	50	90,4%	95,0%	98,7%	99,0%	96,9%
15387	55	20	20	55	90,5%	94,9%	98,7%	99,0%	97,1%
15388	55	20	20	60	90,7%	94,8%	98,8%	99,1%	97,3%
15389	55	20	20	65	90,8%	94,8%	98,8%	99,2%	97,5%
15390	55	20	20	70	91,0%	94,7%	98,9%	99,2%	97,6%
15391	55	20	20	75	91,1%	94,7%	98,9%	99,3%	97,8%
15392	55	20	20	80	91,3%	94,6%	98,9%	99,3%	97,9%
15393	55	20	25	20	86,9%	94,2%	98,2%	98,4%	95,2%
15394	55	20	25	25	87,1%	94,1%	98,3%	98,5%	95,5%
15395	55	20	25	30	87,3%	94,0%	98,3%	98,6%	95,8%
15396	55	20	25	35	87,5%	94,0%	98,4%	98,7%	96,1%
15397	55	20	25	40	87,6%	93,9%	98,4%	98,8%	96,3%
15398	55	20	25	45	87,8%	93,8%	98,5%	98,8%	96,6%
15399	55	20	25	50	88,0%	93,7%	98,5%	98,9%	96,8%
15400	55	20	25	55	88,2%	93,7%	98,6%	99,0%	97,0%
15401	55	20	25	60	88,4%	93,6%	98,6%	99,1%	97,2%
15402	55	20	25	65	88,6%	93,5%	98,7%	99,1%	97,4%
15403	55	20	25	70	88,7%	93,4%	98,7%	99,2%	97,5%
15404	55	20	25	75	88,9%	93,4%	98,7%	99,2%	97,7%
15405	55	20	25	80	89,1%	93,3%	98,8%	99,3%	97,8%
15406	55	20	30	20	83,8%	92,8%	98,0%	98,3%	95,0%
15407	55	20	30	25	84,0%	92,7%	98,0%	98,4%	95,3%
15408	55	20	30	30	84,3%	92,6%	98,1%	98,5%	95,6%
15409	55	20	30	35	84,5%	92,5%	98,2%	98,6%	95,9%
15410	55	20	30	40	84,7%	92,4%	98,2%	98,7%	96,2%

15411	55	20	30	45	84,9%	92,3%	98,3%	98,8%	96,4%
15412	55	20	30	50	85,2%	92,2%	98,3%	98,9%	96,6%
15413	55	20	30	55	85,4%	92,2%	98,4%	98,9%	96,9%
15414	55	20	30	60	85,6%	92,1%	98,4%	99,0%	97,1%
15415	55	20	30	65	85,8%	92,0%	98,5%	99,1%	97,3%
15416	55	20	30	70	86,0%	91,9%	98,5%	99,1%	97,4%
15417	55	20	30	75	86,2%	91,8%	98,6%	99,2%	97,6%
15418	55	20	30	80	86,5%	91,7%	98,6%	99,2%	97,7%
15419	55	20	35	20	80,1%	91,1%	97,7%	98,2%	94,8%
15420	55	20	35	25	80,4%	91,0%	97,8%	98,3%	95,2%
15421	55	20	35	30	80,7%	90,9%	97,8%	98,4%	95,5%
15422	55	20	35	35	81,0%	90,8%	97,9%	98,5%	95,7%
15423	55	20	35	40	81,2%	90,7%	98,0%	98,6%	96,0%
15424	55	20	35	45	81,5%	90,5%	98,0%	98,7%	96,3%
15425	55	20	35	50	81,8%	90,4%	98,1%	98,8%	96,5%
15426	55	20	35	55	82,0%	90,3%	98,2%	98,9%	96,7%
15427	55	20	35	60	82,3%	90,2%	98,2%	98,9%	96,9%
15428	55	20	35	65	82,5%	90,1%	98,3%	99,0%	97,1%
15429	55	20	35	70	82,8%	90,0%	98,3%	99,1%	97,3%
15430	55	20	35	75	83,0%	89,8%	98,4%	99,1%	97,5%
15431	55	20	35	80	83,3%	89,7%	98,5%	99,2%	97,7%
15432	55	20	40	20	75,9%	89,0%	97,4%	98,1%	94,6%
15433	55	20	40	25	76,2%	88,9%	97,5%	98,2%	95,0%
15434	55	20	40	30	76,6%	88,8%	97,6%	98,3%	95,3%
15435	55	20	40	35	76,9%	88,6%	97,6%	98,4%	95,6%
15436	55	20	40	40	77,2%	88,5%	97,7%	98,5%	95,9%
15437	55	20	40	45	77,5%	88,4%	97,8%	98,6%	96,1%
15438	55	20	40	50	77,8%	88,2%	97,9%	98,7%	96,4%
15439	55	20	40	55	78,1%	88,1%	97,9%	98,8%	96,6%
15440	55	20	40	60	78,4%	88,0%	98,0%	98,9%	96,8%
15441	55	20	40	65	78,7%	87,8%	98,1%	98,9%	97,0%
15442	55	20	40	70	79,0%	87,7%	98,1%	99,0%	97,2%
15443	55	20	40	75	79,3%	87,5%	98,2%	99,1%	97,4%

15444	55	20	40	80	79,6%	87,4%	98,3%	99,1%	97,6%
15445	55	20	45	20	71,1%	86,6%	97,1%	97,9%	94,4%
15446	55	20	45	25	71,5%	86,4%	97,2%	98,1%	94,8%
15447	55	20	45	30	71,8%	86,3%	97,3%	98,2%	95,1%
15448	55	20	45	35	72,2%	86,1%	97,3%	98,3%	95,4%
15449	55	20	45	40	72,5%	85,9%	97,4%	98,4%	95,7%
15450	55	20	45	45	72,9%	85,8%	97,5%	98,5%	96,0%
15451	55	20	45	50	73,2%	85,6%	97,6%	98,6%	96,2%
15452	55	20	45	55	73,6%	85,5%	97,7%	98,7%	96,5%
15453	55	20	45	60	73,9%	85,3%	97,7%	98,8%	96,7%
15454	55	20	45	65	74,3%	85,1%	97,8%	98,9%	96,9%
15455	55	20	45	70	74,6%	85,0%	97,9%	99,0%	97,1%
15456	55	20	45	75	74,9%	84,8%	98,0%	99,0%	97,3%
15457	55	20	45	80	75,3%	84,6%	98,0%	99,1%	97,5%
15458	55	20	50	20	65,8%	83,6%	96,7%	97,8%	94,2%
15459	55	20	50	25	66,2%	83,5%	96,8%	98,0%	94,5%
15460	55	20	50	30	66,6%	83,3%	96,9%	98,1%	94,9%
15461	55	20	50	35	67,0%	83,1%	97,0%	98,2%	95,2%
15462	55	20	50	40	67,4%	82,9%	97,1%	98,3%	95,5%
15463	55	20	50	45	67,7%	82,7%	97,2%	98,4%	95,8%
15464	55	20	50	50	68,1%	82,5%	97,3%	98,5%	96,1%
15465	55	20	50	55	68,5%	82,3%	97,4%	98,6%	96,3%
15466	55	20	50	60	68,9%	82,2%	97,5%	98,7%	96,6%
15467	55	20	50	65	69,3%	82,0%	97,5%	98,8%	96,8%
15468	55	20	50	70	69,6%	81,8%	97,6%	98,9%	97,0%
15469	55	20	50	75	70,0%	81,6%	97,7%	99,0%	97,2%
15470	55	20	50	80	70,4%	81,4%	97,8%	99,0%	97,4%
15471	55	20	55	20	60,0%	80,2%	96,3%	97,7%	94,0%
15472	55	20	55	25	60,5%	80,0%	96,4%	97,8%	94,3%
15473	55	20	55	30	60,9%	79,8%	96,5%	98,0%	94,7%
15474	55	20	55	35	61,3%	79,6%	96,6%	98,1%	95,0%
15475	55	20	55	40	61,7%	79,4%	96,7%	98,2%	95,3%
15476	55	20	55	45	62,1%	79,2%	96,8%	98,3%	95,6%

15477	55	20	55	50	62,5%	79,0%	96,9%	98,4%	95,9%
15478	55	20	55	55	63,0%	78,7%	97,0%	98,6%	96,2%
15479	55	20	55	60	63,4%	78,5%	97,1%	98,6%	96,4%
15480	55	20	55	65	63,8%	78,3%	97,2%	98,7%	96,6%
15481	55	20	55	70	64,2%	78,1%	97,3%	98,8%	96,9%
15482	55	20	55	75	64,6%	77,8%	97,4%	98,9%	97,1%
15483	55	20	55	80	65,0%	77,6%	97,5%	99,0%	97,2%
15484	55	20	60	20	54,0%	76,3%	95,8%	97,5%	93,7%
15485	55	20	60	25	54,4%	76,1%	95,9%	97,7%	94,1%
15486	55	20	60	30	54,9%	75,8%	96,1%	97,8%	94,5%
15487	55	20	60	35	55,3%	75,6%	96,2%	98,0%	94,8%
15488	55	20	60	40	55,7%	75,3%	96,3%	98,1%	95,1%
15489	55	20	60	45	56,2%	75,1%	96,4%	98,2%	95,5%
15490	55	20	60	50	56,6%	74,9%	96,5%	98,4%	95,7%
15491	55	20	60	55	57,0%	74,6%	96,7%	98,5%	96,0%
15492	55	20	60	60	57,5%	74,4%	96,8%	98,6%	96,3%
15493	55	20	60	65	57,9%	74,1%	96,9%	98,7%	96,5%
15494	55	20	60	70	58,3%	73,9%	97,0%	98,7%	96,7%
15495	55	20	60	75	58,8%	73,6%	97,1%	98,8%	96,9%
15496	55	20	60	80	59,2%	73,4%	97,2%	98,9%	97,1%
15497	55	20	65	20	47,8%	71,9%	95,3%	97,4%	93,5%
15498	55	20	65	25	48,3%	71,6%	95,4%	97,5%	93,9%
15499	55	20	65	30	48,7%	71,4%	95,6%	97,7%	94,3%
15500	55	20	65	35	49,2%	71,1%	95,7%	97,9%	94,6%
15501	55	20	65	40	49,6%	70,8%	95,8%	98,0%	95,0%
15502	55	20	65	45	50,0%	70,5%	96,0%	98,1%	95,3%
15503	55	20	65	50	50,5%	70,3%	96,1%	98,3%	95,6%
15504	55	20	65	55	50,9%	70,0%	96,2%	98,4%	95,9%
15505	55	20	65	60	51,4%	69,7%	96,4%	98,5%	96,1%
15506	55	20	65	65	51,8%	69,4%	96,5%	98,6%	96,4%
15507	55	20	65	70	52,2%	69,2%	96,6%	98,7%	96,6%
15508	55	20	65	75	52,7%	68,9%	96,7%	98,8%	96,8%
15509	55	20	65	80	53,1%	68,6%	96,8%	98,8%	97,0%

15510	55	20	70	20	41,7%	67,0%	94,7%	97,2%	93,2%
15511	55	20	70	25	42,2%	66,7%	94,8%	97,4%	93,6%
15512	55	20	70	30	42,6%	66,4%	95,0%	97,6%	94,0%
15513	55	20	70	35	43,0%	66,1%	95,2%	97,7%	94,4%
15514	55	20	70	40	43,5%	65,8%	95,3%	97,9%	94,8%
15515	55	20	70	45	43,9%	65,5%	95,5%	98,0%	95,1%
15516	55	20	70	50	44,3%	65,2%	95,6%	98,1%	95,4%
15517	55	20	70	55	44,8%	64,9%	95,8%	98,3%	95,7%
15518	55	20	70	60	45,2%	64,6%	95,9%	98,4%	96,0%
15519	55	20	70	65	45,6%	64,3%	96,0%	98,5%	96,2%
15520	55	20	70	70	46,1%	64,0%	96,1%	98,6%	96,5%
15521	55	20	70	75	46,5%	63,7%	96,3%	98,7%	96,7%
15522	55	20	70	80	47,0%	63,4%	96,4%	98,8%	96,9%
15523	55	20	75	20	35,9%	61,7%	94,0%	97,0%	92,9%
15524	55	20	75	25	36,3%	61,4%	94,2%	97,2%	93,4%
15525	55	20	75	30	36,7%	61,1%	94,4%	97,4%	93,8%
15526	55	20	75	35	37,1%	60,8%	94,6%	97,6%	94,2%
15527	55	20	75	40	37,5%	60,5%	94,7%	97,7%	94,5%
15528	55	20	75	45	37,9%	60,1%	94,9%	97,9%	94,9%
15529	55	20	75	50	38,4%	59,8%	95,1%	98,0%	95,2%
15530	55	20	75	55	38,8%	59,5%	95,2%	98,2%	95,5%
15531	55	20	75	60	39,2%	59,2%	95,4%	98,3%	95,8%
15532	55	20	75	65	39,6%	58,9%	95,5%	98,4%	96,1%
15533	55	20	75	70	40,0%	58,6%	95,7%	98,5%	96,3%
15534	55	20	75	75	40,5%	58,2%	95,8%	98,6%	96,5%
15535	55	20	75	80	40,9%	57,9%	95,9%	98,7%	96,8%
15536	55	20	80	20	30,4%	56,1%	93,3%	96,9%	92,7%
15537	55	20	80	25	30,8%	55,8%	93,5%	97,1%	93,1%
15538	55	20	80	30	31,2%	55,5%	93,7%	97,3%	93,5%
15539	55	20	80	35	31,6%	55,1%	93,9%	97,4%	93,9%
15540	55	20	80	40	31,9%	54,8%	94,1%	97,6%	94,3%
15541	55	20	80	45	32,3%	54,5%	94,3%	97,8%	94,7%
15542	55	20	80	50	32,7%	54,2%	94,4%	97,9%	95,0%

15543	55	20	80	55	33,1%	53,9%	94,6%	98,0%	95,3%
15544	55	20	80	60	33,5%	53,5%	94,8%	98,2%	95,6%
15545	55	20	80	65	33,9%	53,2%	95,0%	98,3%	95,9%
15546	55	20	80	70	34,3%	52,9%	95,1%	98,4%	96,2%
15547	55	20	80	75	34,7%	52,5%	95,3%	98,5%	96,4%
15548	55	20	80	80	35,1%	52,2%	95,4%	98,6%	96,6%
15549	55	25	20	20	90,2%	95,7%	98,4%	98,4%	95,0%
15550	55	25	20	25	90,4%	95,6%	98,4%	98,5%	95,3%
15551	55	25	20	30	90,5%	95,6%	98,5%	98,6%	95,6%
15552	55	25	20	35	90,7%	95,5%	98,5%	98,7%	95,9%
15553	55	25	20	40	90,8%	95,5%	98,6%	98,7%	96,1%
15554	55	25	20	45	91,0%	95,4%	98,6%	98,8%	96,4%
15555	55	25	20	50	91,1%	95,3%	98,7%	98,9%	96,6%
15556	55	25	20	55	91,3%	95,3%	98,7%	99,0%	96,8%
15557	55	25	20	60	91,4%	95,2%	98,7%	99,0%	97,0%
15558	55	25	20	65	91,6%	95,2%	98,8%	99,1%	97,2%
15559	55	25	20	70	91,7%	95,1%	98,8%	99,2%	97,4%
15560	55	25	20	75	91,8%	95,0%	98,9%	99,2%	97,6%
15561	55	25	20	80	92,0%	95,0%	98,9%	99,3%	97,7%
15562	55	25	25	20	87,8%	94,6%	98,1%	98,3%	94,8%
15563	55	25	25	25	88,0%	94,5%	98,2%	98,4%	95,1%
15564	55	25	25	30	88,2%	94,5%	98,3%	98,5%	95,4%
15565	55	25	25	35	88,4%	94,4%	98,3%	98,6%	95,7%
15566	55	25	25	40	88,6%	94,3%	98,4%	98,7%	96,0%
15567	55	25	25	45	88,8%	94,3%	98,4%	98,8%	96,2%
15568	55	25	25	50	88,9%	94,2%	98,5%	98,8%	96,5%
15569	55	25	25	55	89,1%	94,1%	98,5%	98,9%	96,7%
15570	55	25	25	60	89,3%	94,0%	98,6%	99,0%	96,9%
15571	55	25	25	65	89,4%	94,0%	98,6%	99,1%	97,1%
15572	55	25	25	70	89,6%	93,9%	98,7%	99,1%	97,3%
15573	55	25	25	75	89,8%	93,8%	98,7%	99,2%	97,5%
15574	55	25	25	80	89,9%	93,7%	98,8%	99,2%	97,6%
15575	55	25	30	20	84,9%	93,3%	97,9%	98,1%	94,6%

15576	55	25	30	25	85,2%	93,2%	98,0%	98,3%	94,9%
15577	55	25	30	30	85,4%	93,1%	98,0%	98,4%	95,2%
15578	55	25	30	35	85,6%	93,1%	98,1%	98,5%	95,5%
15579	55	25	30	40	85,8%	93,0%	98,2%	98,6%	95,8%
15580	55	25	30	45	86,0%	92,9%	98,2%	98,7%	96,1%
15581	55	25	30	50	86,2%	92,8%	98,3%	98,8%	96,3%
15582	55	25	30	55	86,5%	92,7%	98,3%	98,8%	96,6%
15583	55	25	30	60	86,7%	92,6%	98,4%	98,9%	96,8%
15584	55	25	30	65	86,9%	92,5%	98,4%	99,0%	97,0%
15585	55	25	30	70	87,1%	92,4%	98,5%	99,1%	97,2%
15586	55	25	30	75	87,3%	92,3%	98,5%	99,1%	97,4%
15587	55	25	30	80	87,5%	92,3%	98,6%	99,2%	97,5%
15588	55	25	35	20	81,5%	91,7%	97,6%	98,0%	94,3%
15589	55	25	35	25	81,8%	91,6%	97,7%	98,2%	94,7%
15590	55	25	35	30	82,0%	91,5%	97,8%	98,3%	95,0%
15591	55	25	35	35	82,3%	91,4%	97,8%	98,4%	95,3%
15592	55	25	35	40	82,5%	91,3%	97,9%	98,5%	95,6%
15593	55	25	35	45	82,8%	91,2%	98,0%	98,6%	95,9%
15594	55	25	35	50	83,1%	91,1%	98,1%	98,7%	96,2%
15595	55	25	35	55	83,3%	91,0%	98,1%	98,8%	96,4%
15596	55	25	35	60	83,5%	90,9%	98,2%	98,9%	96,6%
15597	55	25	35	65	83,8%	90,8%	98,2%	98,9%	96,9%
15598	55	25	35	70	84,0%	90,7%	98,3%	99,0%	97,1%
15599	55	25	35	75	84,3%	90,5%	98,4%	99,1%	97,3%
15600	55	25	35	80	84,5%	90,4%	98,4%	99,1%	97,4%
15601	55	25	40	20	77,5%	89,8%	97,3%	97,9%	94,1%
15602	55	25	40	25	77,8%	89,7%	97,4%	98,0%	94,5%
15603	55	25	40	30	78,1%	89,5%	97,5%	98,2%	94,8%
15604	55	25	40	35	78,4%	89,4%	97,6%	98,3%	95,2%
15605	55	25	40	40	78,7%	89,3%	97,7%	98,4%	95,5%
15606	55	25	40	45	79,0%	89,2%	97,7%	98,5%	95,7%
15607	55	25	40	50	79,3%	89,0%	97,8%	98,6%	96,0%
15608	55	25	40	55	79,6%	88,9%	97,9%	98,7%	96,3%

15609	55	25	40	60	79,9%	88,8%	97,9%	98,8%	96,5%
15610	55	25	40	65	80,1%	88,6%	98,0%	98,9%	96,7%
15611	55	25	40	70	80,4%	88,5%	98,1%	98,9%	96,9%
15612	55	25	40	75	80,7%	88,4%	98,1%	99,0%	97,1%
15613	55	25	40	80	81,0%	88,2%	98,2%	99,1%	97,3%
15614	55	25	45	20	72,9%	87,4%	97,0%	97,8%	93,9%
15615	55	25	45	25	73,2%	87,3%	97,1%	97,9%	94,3%
15616	55	25	45	30	73,6%	87,2%	97,2%	98,1%	94,6%
15617	55	25	45	35	73,9%	87,0%	97,3%	98,2%	95,0%
15618	55	25	45	40	74,3%	86,9%	97,3%	98,3%	95,3%
15619	55	25	45	45	74,6%	86,7%	97,4%	98,4%	95,6%
15620	55	25	45	50	74,9%	86,6%	97,5%	98,5%	95,9%
15621	55	25	45	55	75,3%	86,4%	97,6%	98,6%	96,1%
15622	55	25	45	60	75,6%	86,3%	97,7%	98,7%	96,4%
15623	55	25	45	65	75,9%	86,1%	97,8%	98,8%	96,6%
15624	55	25	45	70	76,2%	85,9%	97,8%	98,9%	96,8%
15625	55	25	45	75	76,6%	85,8%	97,9%	98,9%	97,0%
15626	55	25	45	80	76,9%	85,6%	98,0%	99,0%	97,2%
15627	55	25	50	20	67,8%	84,7%	96,6%	97,6%	93,6%
15628	55	25	50	25	68,1%	84,5%	96,7%	97,8%	94,0%
15629	55	25	50	30	68,5%	84,3%	96,8%	97,9%	94,4%
15630	55	25	50	35	68,9%	84,2%	96,9%	98,1%	94,8%
15631	55	25	50	40	69,3%	84,0%	97,0%	98,2%	95,1%
15632	55	25	50	45	69,7%	83,8%	97,1%	98,3%	95,4%
15633	55	25	50	50	70,0%	83,6%	97,2%	98,4%	95,7%
15634	55	25	50	55	70,4%	83,5%	97,3%	98,5%	96,0%
15635	55	25	50	60	70,8%	83,3%	97,4%	98,6%	96,2%
15636	55	25	50	65	71,1%	83,1%	97,5%	98,7%	96,5%
15637	55	25	50	70	71,5%	82,9%	97,5%	98,8%	96,7%
15638	55	25	50	75	71,8%	82,7%	97,6%	98,9%	96,9%
15639	55	25	50	80	72,2%	82,5%	97,7%	99,0%	97,1%
15640	55	25	55	20	62,1%	81,4%	96,2%	97,5%	93,4%
15641	55	25	55	25	62,6%	81,2%	96,3%	97,7%	93,8%

15642	55	25	55	30	63,0%	81,0%	96,4%	97,8%	94,2%
15643	55	25	55	35	63,4%	80,8%	96,5%	98,0%	94,5%
15644	55	25	55	40	63,8%	80,6%	96,6%	98,1%	94,9%
15645	55	25	55	45	64,2%	80,4%	96,7%	98,2%	95,2%
15646	55	25	55	50	64,6%	80,2%	96,8%	98,3%	95,5%
15647	55	25	55	55	65,0%	80,0%	96,9%	98,4%	95,8%
15648	55	25	55	60	65,4%	79,8%	97,0%	98,5%	96,1%
15649	55	25	55	65	65,8%	79,6%	97,1%	98,6%	96,3%
15650	55	25	55	70	66,2%	79,4%	97,2%	98,7%	96,6%
15651	55	25	55	75	66,6%	79,2%	97,3%	98,8%	96,8%
15652	55	25	55	80	67,0%	79,0%	97,4%	98,9%	97,0%
15653	55	25	60	20	56,2%	77,7%	95,7%	97,3%	93,1%
15654	55	25	60	25	56,6%	77,5%	95,8%	97,5%	93,6%
15655	55	25	60	30	57,1%	77,2%	95,9%	97,7%	94,0%
15656	55	25	60	35	57,5%	77,0%	96,1%	97,8%	94,3%
15657	55	25	60	40	57,9%	76,8%	96,2%	98,0%	94,7%
15658	55	25	60	45	58,3%	76,5%	96,3%	98,1%	95,0%
15659	55	25	60	50	58,8%	76,3%	96,4%	98,2%	95,3%
15660	55	25	60	55	59,2%	76,1%	96,6%	98,3%	95,6%
15661	55	25	60	60	59,6%	75,8%	96,7%	98,5%	95,9%
15662	55	25	60	65	60,1%	75,6%	96,8%	98,6%	96,2%
15663	55	25	60	70	60,5%	75,4%	96,9%	98,7%	96,4%
15664	55	25	60	75	60,9%	75,1%	97,0%	98,7%	96,6%
15665	55	25	60	80	61,3%	74,9%	97,1%	98,8%	96,9%
15666	55	25	65	20	50,1%	73,4%	95,1%	97,2%	92,9%
15667	55	25	65	25	50,5%	73,2%	95,3%	97,4%	93,3%
15668	55	25	65	30	50,9%	72,9%	95,4%	97,5%	93,7%
15669	55	25	65	35	51,4%	72,7%	95,6%	97,7%	94,1%
15670	55	25	65	40	51,8%	72,4%	95,7%	97,8%	94,5%
15671	55	25	65	45	52,3%	72,2%	95,9%	98,0%	94,8%
15672	55	25	65	50	52,7%	71,9%	96,0%	98,1%	95,1%
15673	55	25	65	55	53,1%	71,6%	96,1%	98,2%	95,5%
15674	55	25	65	60	53,6%	71,4%	96,2%	98,4%	95,7%

15675	55	25	65	65	54,0%	71,1%	96,4%	98,5%	96,0%
15676	55	25	65	70	54,4%	70,8%	96,5%	98,6%	96,3%
15677	55	25	65	75	54,9%	70,5%	96,6%	98,7%	96,5%
15678	55	25	65	80	55,3%	70,3%	96,7%	98,7%	96,7%
15679	55	25	70	20	43,9%	68,7%	94,5%	97,0%	92,6%
15680	55	25	70	25	44,3%	68,4%	94,7%	97,2%	93,0%
15681	55	25	70	30	44,8%	68,1%	94,9%	97,4%	93,5%
15682	55	25	70	35	45,2%	67,9%	95,0%	97,6%	93,9%
15683	55	25	70	40	45,7%	67,6%	95,2%	97,7%	94,3%
15684	55	25	70	45	46,1%	67,3%	95,3%	97,9%	94,6%
15685	55	25	70	50	46,5%	67,0%	95,5%	98,0%	95,0%
15686	55	25	70	55	47,0%	66,7%	95,6%	98,1%	95,3%
15687	55	25	70	60	47,4%	66,4%	95,8%	98,3%	95,6%
15688	55	25	70	65	47,8%	66,1%	95,9%	98,4%	95,9%
15689	55	25	70	70	48,3%	65,8%	96,0%	98,5%	96,1%
15690	55	25	70	75	48,7%	65,5%	96,2%	98,6%	96,4%
15691	55	25	70	80	49,2%	65,2%	96,3%	98,7%	96,6%
15692	55	25	75	20	37,9%	63,5%	93,8%	96,8%	92,3%
15693	55	25	75	25	38,4%	63,2%	94,0%	97,0%	92,8%
15694	55	25	75	30	38,8%	62,9%	94,2%	97,2%	93,2%
15695	55	25	75	35	39,2%	62,6%	94,4%	97,4%	93,6%
15696	55	25	75	40	39,6%	62,3%	94,6%	97,6%	94,0%
15697	55	25	75	45	40,0%	62,0%	94,7%	97,7%	94,4%
15698	55	25	75	50	40,5%	61,7%	94,9%	97,9%	94,8%
15699	55	25	75	55	40,9%	61,4%	95,1%	98,0%	95,1%
15700	55	25	75	60	41,3%	61,1%	95,2%	98,1%	95,4%
15701	55	25	75	65	41,7%	60,8%	95,4%	98,3%	95,7%
15702	55	25	75	70	42,2%	60,5%	95,5%	98,4%	96,0%
15703	55	25	75	75	42,6%	60,2%	95,7%	98,5%	96,2%
15704	55	25	75	80	43,0%	59,8%	95,8%	98,6%	96,5%
15705	55	25	80	20	32,3%	58,0%	93,1%	96,6%	92,0%
15706	55	25	80	25	32,7%	57,7%	93,3%	96,9%	92,5%
15707	55	25	80	30	33,1%	57,4%	93,5%	97,1%	92,9%

15708	55	25	80	35	33,5%	57,1%	93,7%	97,2%	93,4%
15709	55	25	80	40	33,9%	56,8%	93,9%	97,4%	93,8%
15710	55	25	80	45	34,3%	56,4%	94,1%	97,6%	94,2%
15711	55	25	80	50	34,7%	56,1%	94,3%	97,8%	94,5%
15712	55	25	80	55	35,1%	55,8%	94,5%	97,9%	94,9%
15713	55	25	80	60	35,5%	55,5%	94,6%	98,0%	95,2%
15714	55	25	80	65	35,9%	55,2%	94,8%	98,2%	95,5%
15715	55	25	80	70	36,3%	54,8%	95,0%	98,3%	95,8%
15716	55	25	80	75	36,7%	54,5%	95,1%	98,4%	96,1%
15717	55	25	80	80	37,1%	54,2%	95,3%	98,5%	96,3%
15718	55	30	20	20	91,0%	96,0%	98,3%	98,2%	94,5%
15719	55	30	20	25	91,1%	95,9%	98,4%	98,3%	94,8%
15720	55	30	20	30	91,3%	95,9%	98,4%	98,5%	95,2%
15721	55	30	20	35	91,4%	95,8%	98,5%	98,6%	95,5%
15722	55	30	20	40	91,6%	95,8%	98,5%	98,7%	95,8%
15723	55	30	20	45	91,7%	95,7%	98,6%	98,7%	96,0%
15724	55	30	20	50	91,8%	95,7%	98,6%	98,8%	96,3%
15725	55	30	20	55	92,0%	95,6%	98,7%	98,9%	96,5%
15726	55	30	20	60	92,1%	95,6%	98,7%	99,0%	96,7%
15727	55	30	20	65	92,2%	95,5%	98,7%	99,0%	96,9%
15728	55	30	20	70	92,3%	95,5%	98,8%	99,1%	97,1%
15729	55	30	20	75	92,5%	95,4%	98,8%	99,2%	97,3%
15730	55	30	20	80	92,6%	95,3%	98,9%	99,2%	97,5%
15731	55	30	25	20	88,8%	95,0%	98,1%	98,1%	94,3%
15732	55	30	25	25	88,9%	94,9%	98,1%	98,2%	94,6%
15733	55	30	25	30	89,1%	94,9%	98,2%	98,4%	95,0%
15734	55	30	25	35	89,3%	94,8%	98,3%	98,5%	95,3%
15735	55	30	25	40	89,4%	94,7%	98,3%	98,6%	95,6%
15736	55	30	25	45	89,6%	94,7%	98,4%	98,7%	95,9%
15737	55	30	25	50	89,8%	94,6%	98,4%	98,7%	96,1%
15738	55	30	25	55	89,9%	94,5%	98,5%	98,8%	96,4%
15739	55	30	25	60	90,1%	94,5%	98,5%	98,9%	96,6%
15740	55	30	25	65	90,2%	94,4%	98,6%	99,0%	96,8%

15741	55	30	25	70	90,4%	94,3%	98,6%	99,0%	97,0%
15742	55	30	25	75	90,6%	94,3%	98,7%	99,1%	97,2%
15743	55	30	25	80	90,7%	94,2%	98,7%	99,2%	97,4%
15744	55	30	30	20	86,0%	93,8%	97,8%	98,0%	94,0%
15745	55	30	30	25	86,3%	93,7%	97,9%	98,1%	94,4%
15746	55	30	30	30	86,5%	93,6%	98,0%	98,3%	94,8%
15747	55	30	30	35	86,7%	93,5%	98,0%	98,4%	95,1%
15748	55	30	30	40	86,9%	93,5%	98,1%	98,5%	95,4%
15749	55	30	30	45	87,1%	93,4%	98,2%	98,6%	95,7%
15750	55	30	30	50	87,3%	93,3%	98,2%	98,7%	96,0%
15751	55	30	30	55	87,5%	93,2%	98,3%	98,8%	96,2%
15752	55	30	30	60	87,7%	93,1%	98,3%	98,8%	96,5%
15753	55	30	30	65	87,8%	93,1%	98,4%	98,9%	96,7%
15754	55	30	30	70	88,0%	93,0%	98,4%	99,0%	96,9%
15755	55	30	30	75	88,2%	92,9%	98,5%	99,1%	97,1%
15756	55	30	30	80	88,4%	92,8%	98,5%	99,1%	97,3%
15757	55	30	35	20	82,8%	92,3%	97,5%	97,9%	93,8%
15758	55	30	35	25	83,1%	92,2%	97,6%	98,0%	94,2%
15759	55	30	35	30	83,3%	92,1%	97,7%	98,1%	94,6%
15760	55	30	35	35	83,5%	92,0%	97,8%	98,3%	94,9%
15761	55	30	35	40	83,8%	91,9%	97,9%	98,4%	95,2%
15762	55	30	35	45	84,0%	91,8%	97,9%	98,5%	95,5%
15763	55	30	35	50	84,3%	91,7%	98,0%	98,6%	95,8%
15764	55	30	35	55	84,5%	91,6%	98,1%	98,7%	96,1%
15765	55	30	35	60	84,7%	91,5%	98,1%	98,8%	96,3%
15766	55	30	35	65	85,0%	91,4%	98,2%	98,8%	96,6%
15767	55	30	35	70	85,2%	91,3%	98,2%	98,9%	96,8%
15768	55	30	35	75	85,4%	91,2%	98,3%	99,0%	97,0%
15769	55	30	35	80	85,6%	91,1%	98,4%	99,1%	97,2%
15770	55	30	40	20	79,0%	90,5%	97,2%	97,8%	93,6%
15771	55	30	40	25	79,3%	90,4%	97,3%	97,9%	94,0%
15772	55	30	40	30	79,6%	90,2%	97,4%	98,0%	94,3%
15773	55	30	40	35	79,9%	90,1%	97,5%	98,2%	94,7%

15774	55	30	40	40	80,2%	90,0%	97,6%	98,3%	95,0%
15775	55	30	40	45	80,4%	89,9%	97,7%	98,4%	95,3%
15776	55	30	40	50	80,7%	89,8%	97,7%	98,5%	95,6%
15777	55	30	40	55	81,0%	89,7%	97,8%	98,6%	95,9%
15778	55	30	40	60	81,3%	89,5%	97,9%	98,7%	96,2%
15779	55	30	40	65	81,5%	89,4%	97,9%	98,8%	96,4%
15780	55	30	40	70	81,8%	89,3%	98,0%	98,9%	96,6%
15781	55	30	40	75	82,0%	89,2%	98,1%	98,9%	96,9%
15782	55	30	40	80	82,3%	89,0%	98,1%	99,0%	97,1%
15783	55	30	45	20	74,6%	88,3%	96,9%	97,6%	93,3%
15784	55	30	45	25	75,0%	88,2%	97,0%	97,8%	93,7%
15785	55	30	45	30	75,3%	88,0%	97,1%	97,9%	94,1%
15786	55	30	45	35	75,6%	87,9%	97,2%	98,1%	94,5%
15787	55	30	45	40	75,9%	87,7%	97,3%	98,2%	94,8%
15788	55	30	45	45	76,3%	87,6%	97,4%	98,3%	95,2%
15789	55	30	45	50	76,6%	87,5%	97,4%	98,4%	95,5%
15790	55	30	45	55	76,9%	87,3%	97,5%	98,5%	95,7%
15791	55	30	45	60	77,2%	87,2%	97,6%	98,6%	96,0%
15792	55	30	45	65	77,5%	87,0%	97,7%	98,7%	96,3%
15793	55	30	45	70	77,8%	86,9%	97,8%	98,8%	96,5%
15794	55	30	45	75	78,1%	86,7%	97,8%	98,9%	96,7%
15795	55	30	45	80	78,4%	86,6%	97,9%	98,9%	96,9%
15796	55	30	50	20	69,7%	85,7%	96,5%	97,5%	93,0%
15797	55	30	50	25	70,0%	85,5%	96,6%	97,6%	93,5%
15798	55	30	50	30	70,4%	85,4%	96,7%	97,8%	93,9%
15799	55	30	50	35	70,8%	85,2%	96,8%	97,9%	94,3%
15800	55	30	50	40	71,1%	85,0%	96,9%	98,1%	94,6%
15801	55	30	50	45	71,5%	84,9%	97,0%	98,2%	95,0%
15802	55	30	50	50	71,9%	84,7%	97,1%	98,3%	95,3%
15803	55	30	50	55	72,2%	84,5%	97,2%	98,4%	95,6%
15804	55	30	50	60	72,6%	84,3%	97,3%	98,5%	95,9%
15805	55	30	50	65	72,9%	84,2%	97,4%	98,6%	96,1%
15806	55	30	50	70	73,3%	84,0%	97,5%	98,7%	96,4%

15807	55	30	50	75	73,6%	83,8%	97,6%	98,8%	96,6%
15808	55	30	50	80	73,9%	83,6%	97,6%	98,9%	96,8%
15809	55	30	55	20	64,2%	82,6%	96,0%	97,3%	92,8%
15810	55	30	55	25	64,6%	82,4%	96,2%	97,5%	93,2%
15811	55	30	55	30	65,0%	82,2%	96,3%	97,6%	93,6%
15812	55	30	55	35	65,4%	82,0%	96,4%	97,8%	94,0%
15813	55	30	55	40	65,8%	81,8%	96,5%	97,9%	94,4%
15814	55	30	55	45	66,2%	81,6%	96,6%	98,1%	94,8%
15815	55	30	55	50	66,6%	81,5%	96,7%	98,2%	95,1%
15816	55	30	55	55	67,0%	81,3%	96,8%	98,3%	95,4%
15817	55	30	55	60	67,4%	81,1%	96,9%	98,4%	95,7%
15818	55	30	55	65	67,8%	80,9%	97,0%	98,5%	96,0%
15819	55	30	55	70	68,2%	80,6%	97,1%	98,6%	96,2%
15820	55	30	55	75	68,5%	80,4%	97,2%	98,7%	96,5%
15821	55	30	55	80	68,9%	80,2%	97,3%	98,8%	96,7%
15822	55	30	60	20	58,4%	79,0%	95,5%	97,1%	92,5%
15823	55	30	60	25	58,8%	78,8%	95,7%	97,3%	93,0%
15824	55	30	60	30	59,2%	78,6%	95,8%	97,5%	93,4%
15825	55	30	60	35	59,6%	78,4%	95,9%	97,7%	93,8%
15826	55	30	60	40	60,1%	78,2%	96,1%	97,8%	94,2%
15827	55	30	60	45	60,5%	77,9%	96,2%	98,0%	94,5%
15828	55	30	60	50	60,9%	77,7%	96,3%	98,1%	94,9%
15829	55	30	60	55	61,3%	77,5%	96,4%	98,2%	95,2%
15830	55	30	60	60	61,7%	77,3%	96,6%	98,3%	95,5%
15831	55	30	60	65	62,2%	77,0%	96,7%	98,4%	95,8%
15832	55	30	60	70	62,6%	76,8%	96,8%	98,5%	96,1%
15833	55	30	60	75	63,0%	76,6%	96,9%	98,6%	96,3%
15834	55	30	60	80	63,4%	76,3%	97,0%	98,7%	96,6%
15835	55	30	65	20	52,3%	75,0%	95,0%	97,0%	92,2%
15836	55	30	65	25	52,7%	74,7%	95,1%	97,2%	92,7%
15837	55	30	65	30	53,1%	74,5%	95,3%	97,4%	93,1%
15838	55	30	65	35	53,6%	74,2%	95,4%	97,5%	93,6%
15839	55	30	65	40	54,0%	74,0%	95,6%	97,7%	94,0%

15840	55	30	65	45	54,5%	73,7%	95,7%	97,8%	94,3%
15841	55	30	65	50	54,9%	73,5%	95,9%	98,0%	94,7%
15842	55	30	65	55	55,3%	73,2%	96,0%	98,1%	95,0%
15843	55	30	65	60	55,8%	72,9%	96,1%	98,2%	95,3%
15844	55	30	65	65	56,2%	72,7%	96,2%	98,3%	95,6%
15845	55	30	65	70	56,6%	72,4%	96,4%	98,5%	95,9%
15846	55	30	65	75	57,1%	72,2%	96,5%	98,6%	96,2%
15847	55	30	65	80	57,5%	71,9%	96,6%	98,7%	96,4%
15848	55	30	70	20	46,1%	70,4%	94,3%	96,8%	91,9%
15849	55	30	70	25	46,5%	70,1%	94,5%	97,0%	92,4%
15850	55	30	70	30	47,0%	69,8%	94,7%	97,2%	92,9%
15851	55	30	70	35	47,4%	69,6%	94,9%	97,4%	93,3%
15852	55	30	70	40	47,9%	69,3%	95,0%	97,5%	93,7%
15853	55	30	70	45	48,3%	69,0%	95,2%	97,7%	94,1%
15854	55	30	70	50	48,7%	68,7%	95,3%	97,9%	94,5%
15855	55	30	70	55	49,2%	68,4%	95,5%	98,0%	94,8%
15856	55	30	70	60	49,6%	68,1%	95,6%	98,1%	95,1%
15857	55	30	70	65	50,1%	67,9%	95,8%	98,2%	95,5%
15858	55	30	70	70	50,5%	67,6%	95,9%	98,4%	95,7%
15859	55	30	70	75	50,9%	67,3%	96,0%	98,5%	96,0%
15860	55	30	70	80	51,4%	67,0%	96,2%	98,6%	96,3%
15861	55	30	75	20	40,1%	65,3%	93,6%	96,6%	91,6%
15862	55	30	75	25	40,5%	65,1%	93,8%	96,8%	92,1%
15863	55	30	75	30	40,9%	64,8%	94,0%	97,0%	92,6%
15864	55	30	75	35	41,3%	64,5%	94,2%	97,2%	93,0%
15865	55	30	75	40	41,8%	64,2%	94,4%	97,4%	93,5%
15866	55	30	75	45	42,2%	63,9%	94,6%	97,6%	93,9%
15867	55	30	75	50	42,6%	63,6%	94,8%	97,7%	94,3%
15868	55	30	75	55	43,1%	63,2%	94,9%	97,9%	94,6%
15869	55	30	75	60	43,5%	62,9%	95,1%	98,0%	95,0%
15870	55	30	75	65	43,9%	62,6%	95,2%	98,1%	95,3%
15871	55	30	75	70	44,4%	62,3%	95,4%	98,3%	95,6%
15872	55	30	75	75	44,8%	62,0%	95,5%	98,4%	95,9%

15873	55	30	75	80	45,2%	61,7%	95,7%	98,5%	96,1%
15874	55	30	80	20	34,3%	60,0%	92,9%	96,4%	91,3%
15875	55	30	80	25	34,7%	59,6%	93,1%	96,6%	91,8%
15876	55	30	80	30	35,1%	59,3%	93,3%	96,8%	92,3%
15877	55	30	80	35	35,5%	59,0%	93,5%	97,0%	92,8%
15878	55	30	80	40	35,9%	58,7%	93,7%	97,2%	93,2%
15879	55	30	80	45	36,3%	58,4%	93,9%	97,4%	93,6%
15880	55	30	80	50	36,7%	58,1%	94,1%	97,6%	94,0%
15881	55	30	80	55	37,1%	57,7%	94,3%	97,7%	94,4%
15882	55	30	80	60	37,5%	57,4%	94,5%	97,9%	94,8%
15883	55	30	80	65	38,0%	57,1%	94,6%	98,0%	95,1%
15884	55	30	80	70	38,4%	56,8%	94,8%	98,2%	95,4%
15885	55	30	80	75	38,8%	56,5%	95,0%	98,3%	95,7%
15886	55	30	80	80	39,2%	56,1%	95,1%	98,4%	96,0%
15887	55	35	20	20	91,7%	96,3%	98,2%	98,1%	94,0%
15888	55	35	20	25	91,8%	96,2%	98,3%	98,2%	94,3%
15889	55	35	20	30	92,0%	96,2%	98,4%	98,3%	94,7%
15890	55	35	20	35	92,1%	96,1%	98,4%	98,4%	95,0%
15891	55	35	20	40	92,2%	96,1%	98,5%	98,5%	95,3%
15892	55	35	20	45	92,3%	96,0%	98,5%	98,6%	95,6%
15893	55	35	20	50	92,5%	96,0%	98,6%	98,7%	95,9%
15894	55	35	20	55	92,6%	95,9%	98,6%	98,8%	96,2%
15895	55	35	20	60	92,7%	95,9%	98,7%	98,9%	96,4%
15896	55	35	20	65	92,8%	95,8%	98,7%	99,0%	96,6%
15897	55	35	20	70	92,9%	95,8%	98,7%	99,0%	96,9%
15898	55	35	20	75	93,1%	95,7%	98,8%	99,1%	97,1%
15899	55	35	20	80	93,2%	95,7%	98,8%	99,2%	97,3%
15900	55	35	25	20	89,6%	95,4%	98,0%	98,0%	93,7%
15901	55	35	25	25	89,8%	95,3%	98,1%	98,1%	94,1%
15902	55	35	25	30	89,9%	95,2%	98,1%	98,2%	94,5%
15903	55	35	25	35	90,1%	95,2%	98,2%	98,3%	94,8%
15904	55	35	25	40	90,2%	95,1%	98,3%	98,5%	95,2%
15905	55	35	25	45	90,4%	95,1%	98,3%	98,6%	95,5%

15906	55	35	25	50	90,6%	95,0%	98,4%	98,7%	95,8%
15907	55	35	25	55	90,7%	94,9%	98,4%	98,7%	96,0%
15908	55	35	25	60	90,9%	94,9%	98,5%	98,8%	96,3%
15909	55	35	25	65	91,0%	94,8%	98,5%	98,9%	96,5%
15910	55	35	25	70	91,1%	94,7%	98,6%	99,0%	96,7%
15911	55	35	25	75	91,3%	94,7%	98,6%	99,0%	96,9%
15912	55	35	25	80	91,4%	94,6%	98,7%	99,1%	97,1%
15913	55	35	30	20	87,1%	94,2%	97,8%	97,9%	93,5%
15914	55	35	30	25	87,3%	94,2%	97,8%	98,0%	93,9%
15915	55	35	30	30	87,5%	94,1%	97,9%	98,1%	94,3%
15916	55	35	30	35	87,7%	94,0%	98,0%	98,2%	94,6%
15917	55	35	30	40	87,9%	93,9%	98,0%	98,4%	95,0%
15918	55	35	30	45	88,0%	93,9%	98,1%	98,5%	95,3%
15919	55	35	30	50	88,2%	93,8%	98,2%	98,6%	95,6%
15920	55	35	30	55	88,4%	93,7%	98,2%	98,7%	95,9%
15921	55	35	30	60	88,6%	93,6%	98,3%	98,8%	96,1%
15922	55	35	30	65	88,8%	93,5%	98,3%	98,8%	96,4%
15923	55	35	30	70	88,9%	93,5%	98,4%	98,9%	96,6%
15924	55	35	30	75	89,1%	93,4%	98,4%	99,0%	96,8%
15925	55	35	30	80	89,3%	93,3%	98,5%	99,1%	97,0%
15926	55	35	35	20	84,0%	92,8%	97,5%	97,7%	93,2%
15927	55	35	35	25	84,3%	92,7%	97,6%	97,9%	93,6%
15928	55	35	35	30	84,5%	92,7%	97,6%	98,0%	94,0%
15929	55	35	35	35	84,7%	92,6%	97,7%	98,1%	94,4%
15930	55	35	35	40	85,0%	92,5%	97,8%	98,3%	94,8%
15931	55	35	35	45	85,2%	92,4%	97,9%	98,4%	95,1%
15932	55	35	35	50	85,4%	92,3%	97,9%	98,5%	95,4%
15933	55	35	35	55	85,6%	92,2%	98,0%	98,6%	95,7%
15934	55	35	35	60	85,8%	92,1%	98,1%	98,7%	96,0%
15935	55	35	35	65	86,1%	92,0%	98,1%	98,8%	96,2%
15936	55	35	35	70	86,3%	91,9%	98,2%	98,8%	96,5%
15937	55	35	35	75	86,5%	91,8%	98,2%	98,9%	96,7%
15938	55	35	35	80	86,7%	91,7%	98,3%	99,0%	96,9%

15939	55	35	40	20	80,4%	91,1%	97,1%	97,6%	93,0%
15940	55	35	40	25	80,7%	91,0%	97,2%	97,7%	93,4%
15941	55	35	40	30	81,0%	90,9%	97,3%	97,9%	93,8%
15942	55	35	40	35	81,3%	90,8%	97,4%	98,0%	94,2%
15943	55	35	40	40	81,5%	90,7%	97,5%	98,2%	94,6%
15944	55	35	40	45	81,8%	90,6%	97,6%	98,3%	94,9%
15945	55	35	40	50	82,1%	90,5%	97,7%	98,4%	95,2%
15946	55	35	40	55	82,3%	90,4%	97,7%	98,5%	95,5%
15947	55	35	40	60	82,6%	90,2%	97,8%	98,6%	95,8%
15948	55	35	40	65	82,8%	90,1%	97,9%	98,7%	96,1%
15949	55	35	40	70	83,1%	90,0%	98,0%	98,8%	96,3%
15950	55	35	40	75	83,3%	89,9%	98,0%	98,9%	96,6%
15951	55	35	40	80	83,6%	89,8%	98,1%	98,9%	96,8%
15952	55	35	45	20	76,3%	89,1%	96,8%	97,4%	92,7%
15953	55	35	45	25	76,6%	89,0%	96,9%	97,6%	93,1%
15954	55	35	45	30	76,9%	88,8%	97,0%	97,8%	93,6%
15955	55	35	45	35	77,2%	88,7%	97,1%	97,9%	94,0%
15956	55	35	45	40	77,5%	88,6%	97,2%	98,0%	94,3%
15957	55	35	45	45	77,8%	88,4%	97,3%	98,2%	94,7%
15958	55	35	45	50	78,1%	88,3%	97,4%	98,3%	95,0%
15959	55	35	45	55	78,4%	88,2%	97,4%	98,4%	95,3%
15960	55	35	45	60	78,7%	88,0%	97,5%	98,5%	95,6%
15961	55	35	45	65	79,0%	87,9%	97,6%	98,6%	95,9%
15962	55	35	45	70	79,3%	87,7%	97,7%	98,7%	96,2%
15963	55	35	45	75	79,6%	87,6%	97,8%	98,8%	96,4%
15964	55	35	45	80	79,9%	87,5%	97,8%	98,9%	96,6%
15965	55	35	50	20	71,5%	86,6%	96,4%	97,3%	92,4%
15966	55	35	50	25	71,9%	86,5%	96,5%	97,5%	92,9%
15967	55	35	50	30	72,2%	86,3%	96,6%	97,6%	93,3%
15968	55	35	50	35	72,6%	86,2%	96,7%	97,8%	93,7%
15969	55	35	50	40	72,9%	86,0%	96,8%	97,9%	94,1%
15970	55	35	50	45	73,3%	85,8%	96,9%	98,1%	94,5%
15971	55	35	50	50	73,6%	85,7%	97,0%	98,2%	94,8%

15972	55	35	50	55	74,0%	85,5%	97,1%	98,3%	95,2%
15973	55	35	50	60	74,3%	85,4%	97,2%	98,4%	95,5%
15974	55	35	50	65	74,6%	85,2%	97,3%	98,5%	95,7%
15975	55	35	50	70	75,0%	85,0%	97,4%	98,6%	96,0%
15976	55	35	50	75	75,3%	84,9%	97,5%	98,7%	96,3%
15977	55	35	50	80	75,6%	84,7%	97,6%	98,8%	96,5%
15978	55	35	55	20	66,2%	83,7%	95,9%	97,1%	92,1%
15979	55	35	55	25	66,6%	83,5%	96,0%	97,3%	92,6%
15980	55	35	55	30	67,0%	83,4%	96,2%	97,5%	93,0%
15981	55	35	55	35	67,4%	83,2%	96,3%	97,6%	93,5%
15982	55	35	55	40	67,8%	83,0%	96,4%	97,8%	93,9%
15983	55	35	55	45	68,2%	82,8%	96,5%	97,9%	94,3%
15984	55	35	55	50	68,5%	82,6%	96,6%	98,1%	94,6%
15985	55	35	55	55	68,9%	82,4%	96,7%	98,2%	95,0%
15986	55	35	55	60	69,3%	82,2%	96,9%	98,3%	95,3%
15987	55	35	55	65	69,7%	82,0%	97,0%	98,4%	95,6%
15988	55	35	55	70	70,0%	81,8%	97,1%	98,5%	95,9%
15989	55	35	55	75	70,4%	81,7%	97,1%	98,6%	96,1%
15990	55	35	55	80	70,8%	81,5%	97,2%	98,7%	96,4%
15991	55	35	60	20	60,5%	80,3%	95,4%	96,9%	91,8%
15992	55	35	60	25	60,9%	80,1%	95,5%	97,1%	92,3%
15993	55	35	60	30	61,3%	79,9%	95,7%	97,3%	92,8%
15994	55	35	60	35	61,8%	79,7%	95,8%	97,5%	93,2%
15995	55	35	60	40	62,2%	79,5%	96,0%	97,7%	93,6%
15996	55	35	60	45	62,6%	79,3%	96,1%	97,8%	94,0%
15997	55	35	60	50	63,0%	79,0%	96,2%	98,0%	94,4%
15998	55	35	60	55	63,4%	78,8%	96,3%	98,1%	94,8%
15999	55	35	60	60	63,8%	78,6%	96,5%	98,2%	95,1%
16000	55	35	60	65	64,2%	78,4%	96,6%	98,3%	95,4%
16001	55	35	60	70	64,6%	78,2%	96,7%	98,4%	95,7%
16002	55	35	60	75	65,0%	77,9%	96,8%	98,5%	96,0%
16003	55	35	60	80	65,4%	77,7%	96,9%	98,6%	96,2%
16004	55	35	65	20	54,5%	76,4%	94,8%	96,7%	91,5%

16005	55	35	65	25	54,9%	76,2%	95,0%	97,0%	92,0%
16006	55	35	65	30	55,3%	75,9%	95,1%	97,2%	92,5%
16007	55	35	65	35	55,8%	75,7%	95,3%	97,3%	93,0%
16008	55	35	65	40	56,2%	75,4%	95,4%	97,5%	93,4%
16009	55	35	65	45	56,7%	75,2%	95,6%	97,7%	93,8%
16010	55	35	65	50	57,1%	75,0%	95,7%	97,8%	94,2%
16011	55	35	65	55	57,5%	74,7%	95,9%	98,0%	94,6%
16012	55	35	65	60	57,9%	74,5%	96,0%	98,1%	94,9%
16013	55	35	65	65	58,4%	74,2%	96,1%	98,2%	95,2%
16014	55	35	65	70	58,8%	74,0%	96,3%	98,3%	95,5%
16015	55	35	65	75	59,2%	73,7%	96,4%	98,5%	95,8%
16016	55	35	65	80	59,7%	73,5%	96,5%	98,6%	96,1%
16017	55	35	70	20	48,3%	72,0%	94,2%	96,6%	91,2%
16018	55	35	70	25	48,8%	71,7%	94,4%	96,8%	91,7%
16019	55	35	70	30	49,2%	71,5%	94,5%	97,0%	92,2%
16020	55	35	70	35	49,6%	71,2%	94,7%	97,2%	92,7%
16021	55	35	70	40	50,1%	70,9%	94,9%	97,4%	93,1%
16022	55	35	70	45	50,5%	70,7%	95,0%	97,5%	93,6%
16023	55	35	70	50	51,0%	70,4%	95,2%	97,7%	94,0%
16024	55	35	70	55	51,4%	70,1%	95,3%	97,8%	94,3%
16025	55	35	70	60	51,8%	69,8%	95,5%	98,0%	94,7%
16026	55	35	70	65	52,3%	69,6%	95,6%	98,1%	95,0%
16027	55	35	70	70	52,7%	69,3%	95,8%	98,2%	95,3%
16028	55	35	70	75	53,2%	69,0%	95,9%	98,4%	95,6%
16029	55	35	70	80	53,6%	68,7%	96,0%	98,5%	95,9%
16030	55	35	75	20	42,2%	67,1%	93,5%	96,3%	90,8%
16031	55	35	75	25	42,6%	66,8%	93,7%	96,6%	91,4%
16032	55	35	75	30	43,1%	66,5%	93,9%	96,8%	91,9%
16033	55	35	75	35	43,5%	66,2%	94,0%	97,0%	92,4%
16034	55	35	75	40	43,9%	65,9%	94,2%	97,2%	92,9%
16035	55	35	75	45	44,4%	65,7%	94,4%	97,4%	93,3%
16036	55	35	75	50	44,8%	65,4%	94,6%	97,6%	93,7%
16037	55	35	75	55	45,2%	65,1%	94,8%	97,7%	94,1%

16038	55	35	75	60	45,7%	64,8%	94,9%	97,9%	94,5%
16039	55	35	75	65	46,1%	64,5%	95,1%	98,0%	94,8%
16040	55	35	75	70	46,6%	64,2%	95,2%	98,1%	95,2%
16041	55	35	75	75	47,0%	63,9%	95,4%	98,3%	95,5%
16042	55	35	75	80	47,4%	63,6%	95,5%	98,4%	95,7%
16043	55	35	80	20	36,3%	61,8%	92,6%	96,1%	90,5%
16044	55	35	80	25	36,7%	61,5%	92,9%	96,4%	91,0%
16045	55	35	80	30	37,1%	61,2%	93,1%	96,6%	91,6%
16046	55	35	80	35	37,6%	60,9%	93,3%	96,8%	92,1%
16047	55	35	80	40	38,0%	60,6%	93,5%	97,0%	92,6%
16048	55	35	80	45	38,4%	60,3%	93,7%	97,2%	93,0%
16049	55	35	80	50	38,8%	60,0%	93,9%	97,4%	93,5%
16050	55	35	80	55	39,2%	59,6%	94,1%	97,6%	93,9%
16051	55	35	80	60	39,6%	59,3%	94,3%	97,7%	94,3%
16052	55	35	80	65	40,1%	59,0%	94,5%	97,9%	94,6%
16053	55	35	80	70	40,5%	58,7%	94,7%	98,0%	95,0%
16054	55	35	80	75	40,9%	58,4%	94,8%	98,1%	95,3%
16055	55	35	80	80	41,3%	58,1%	95,0%	98,3%	95,6%
16056	55	40	20	20	92,3%	96,6%	98,2%	98,0%	93,4%
16057	55	40	20	25	92,5%	96,5%	98,2%	98,1%	93,8%
16058	55	40	20	30	92,6%	96,5%	98,3%	98,2%	94,2%
16059	55	40	20	35	92,7%	96,4%	98,4%	98,3%	94,6%
16060	55	40	20	40	92,8%	96,4%	98,4%	98,4%	94,9%
16061	55	40	20	45	92,9%	96,3%	98,5%	98,5%	95,2%
16062	55	40	20	50	93,1%	96,3%	98,5%	98,6%	95,5%
16063	55	40	20	55	93,2%	96,2%	98,6%	98,7%	95,8%
16064	55	40	20	60	93,3%	96,2%	98,6%	98,8%	96,1%
16065	55	40	20	65	93,4%	96,1%	98,7%	98,9%	96,3%
16066	55	40	20	70	93,5%	96,1%	98,7%	99,0%	96,6%
16067	55	40	20	75	93,6%	96,0%	98,7%	99,0%	96,8%
16068	55	40	20	80	93,7%	96,0%	98,8%	99,1%	97,0%
16069	55	40	25	20	90,4%	95,7%	98,0%	97,8%	93,1%
16070	55	40	25	25	90,6%	95,6%	98,0%	98,0%	93,6%

16071	55	40	25	30	90,7%	95,6%	98,1%	98,1%	94,0%
16072	55	40	25	35	90,9%	95,5%	98,1%	98,2%	94,3%
16073	55	40	25	40	91,0%	95,5%	98,2%	98,3%	94,7%
16074	55	40	25	45	91,1%	95,4%	98,3%	98,5%	95,0%
16075	55	40	25	50	91,3%	95,4%	98,3%	98,6%	95,3%
16076	55	40	25	55	91,4%	95,3%	98,4%	98,6%	95,6%
16077	55	40	25	60	91,6%	95,2%	98,4%	98,7%	95,9%
16078	55	40	25	65	91,7%	95,2%	98,5%	98,8%	96,2%
16079	55	40	25	70	91,8%	95,1%	98,5%	98,9%	96,4%
16080	55	40	25	75	92,0%	95,1%	98,6%	99,0%	96,6%
16081	55	40	25	80	92,1%	95,0%	98,6%	99,0%	96,9%
16082	55	40	30	20	88,0%	94,6%	97,7%	97,7%	92,9%
16083	55	40	30	25	88,2%	94,6%	97,8%	97,8%	93,3%
16084	55	40	30	30	88,4%	94,5%	97,8%	98,0%	93,7%
16085	55	40	30	35	88,6%	94,4%	97,9%	98,1%	94,1%
16086	55	40	30	40	88,8%	94,4%	98,0%	98,2%	94,5%
16087	55	40	30	45	88,9%	94,3%	98,0%	98,4%	94,8%
16088	55	40	30	50	89,1%	94,2%	98,1%	98,5%	95,2%
16089	55	40	30	55	89,3%	94,2%	98,2%	98,6%	95,5%
16090	55	40	30	60	89,5%	94,1%	98,2%	98,7%	95,8%
16091	55	40	30	65	89,6%	94,0%	98,3%	98,7%	96,0%
16092	55	40	30	70	89,8%	93,9%	98,3%	98,8%	96,3%
16093	55	40	30	75	89,9%	93,9%	98,4%	98,9%	96,5%
16094	55	40	30	80	90,1%	93,8%	98,5%	99,0%	96,7%
16095	55	40	35	20	85,2%	93,3%	97,4%	97,6%	92,6%
16096	55	40	35	25	85,4%	93,3%	97,5%	97,7%	93,1%
16097	55	40	35	30	85,6%	93,2%	97,6%	97,9%	93,5%
16098	55	40	35	35	85,8%	93,1%	97,6%	98,0%	93,9%
16099	55	40	35	40	86,1%	93,0%	97,7%	98,1%	94,3%
16100	55	40	35	45	86,3%	92,9%	97,8%	98,3%	94,6%
16101	55	40	35	50	86,5%	92,8%	97,9%	98,4%	95,0%
16102	55	40	35	55	86,7%	92,7%	97,9%	98,5%	95,3%
16103	55	40	35	60	86,9%	92,7%	98,0%	98,6%	95,6%

16104	55	40	35	65	87,1%	92,6%	98,1%	98,7%	95,9%
16105	55	40	35	70	87,3%	92,5%	98,1%	98,8%	96,1%
16106	55	40	35	75	87,5%	92,4%	98,2%	98,8%	96,4%
16107	55	40	35	80	87,7%	92,3%	98,2%	98,9%	96,6%
16108	55	40	40	20	81,8%	91,7%	97,1%	97,4%	92,3%
16109	55	40	40	25	82,1%	91,6%	97,2%	97,6%	92,8%
16110	55	40	40	30	82,3%	91,5%	97,2%	97,7%	93,2%
16111	55	40	40	35	82,6%	91,4%	97,3%	97,9%	93,6%
16112	55	40	40	40	82,8%	91,3%	97,4%	98,0%	94,0%
16113	55	40	40	45	83,1%	91,2%	97,5%	98,1%	94,4%
16114	55	40	40	50	83,3%	91,1%	97,6%	98,3%	94,8%
16115	55	40	40	55	83,6%	91,0%	97,7%	98,4%	95,1%
16116	55	40	40	60	83,8%	90,9%	97,7%	98,5%	95,4%
16117	55	40	40	65	84,0%	90,8%	97,8%	98,6%	95,7%
16118	55	40	40	70	84,3%	90,7%	97,9%	98,7%	96,0%
16119	55	40	40	75	84,5%	90,6%	98,0%	98,8%	96,2%
16120	55	40	40	80	84,7%	90,5%	98,0%	98,8%	96,5%
16121	55	40	45	20	77,8%	89,8%	96,7%	97,2%	92,0%
16122	55	40	45	25	78,1%	89,7%	96,8%	97,4%	92,5%
16123	55	40	45	30	78,4%	89,6%	96,9%	97,6%	93,0%
16124	55	40	45	35	78,7%	89,5%	97,0%	97,7%	93,4%
16125	55	40	45	40	79,0%	89,3%	97,1%	97,9%	93,8%
16126	55	40	45	45	79,3%	89,2%	97,2%	98,0%	94,2%
16127	55	40	45	50	79,6%	89,1%	97,3%	98,2%	94,6%
16128	55	40	45	55	79,9%	89,0%	97,4%	98,3%	94,9%
16129	55	40	45	60	80,2%	88,8%	97,5%	98,4%	95,2%
16130	55	40	45	65	80,4%	88,7%	97,5%	98,5%	95,5%
16131	55	40	45	70	80,7%	88,6%	97,6%	98,6%	95,8%
16132	55	40	45	75	81,0%	88,4%	97,7%	98,7%	96,1%
16133	55	40	45	80	81,3%	88,3%	97,8%	98,8%	96,3%
16134	55	40	50	20	73,3%	87,5%	96,3%	97,1%	91,7%
16135	55	40	50	25	73,6%	87,4%	96,4%	97,3%	92,2%
16136	55	40	50	30	74,0%	87,2%	96,5%	97,4%	92,7%

16137	55	40	50	35	74,3%	87,1%	96,6%	97,6%	93,1%
16138	55	40	50	40	74,6%	86,9%	96,7%	97,8%	93,6%
16139	55	40	50	45	75,0%	86,8%	96,8%	97,9%	94,0%
16140	55	40	50	50	75,3%	86,6%	96,9%	98,0%	94,3%
16141	55	40	50	55	75,6%	86,5%	97,0%	98,2%	94,7%
16142	55	40	50	60	76,0%	86,3%	97,1%	98,3%	95,0%
16143	55	40	50	65	76,3%	86,2%	97,2%	98,4%	95,3%
16144	55	40	50	70	76,6%	86,0%	97,3%	98,5%	95,6%
16145	55	40	50	75	76,9%	85,8%	97,4%	98,6%	95,9%
16146	55	40	50	80	77,2%	85,7%	97,5%	98,7%	96,2%
16147	55	40	55	20	68,2%	84,8%	95,8%	96,9%	91,4%
16148	55	40	55	25	68,6%	84,6%	95,9%	97,1%	91,9%
16149	55	40	55	30	68,9%	84,4%	96,1%	97,3%	92,4%
16150	55	40	55	35	69,3%	84,2%	96,2%	97,5%	92,9%
16151	55	40	55	40	69,7%	84,1%	96,3%	97,6%	93,3%
16152	55	40	55	45	70,1%	83,9%	96,4%	97,8%	93,7%
16153	55	40	55	50	70,4%	83,7%	96,5%	97,9%	94,1%
16154	55	40	55	55	70,8%	83,5%	96,6%	98,1%	94,5%
16155	55	40	55	60	71,2%	83,4%	96,8%	98,2%	94,8%
16156	55	40	55	65	71,5%	83,2%	96,9%	98,3%	95,2%
16157	55	40	55	70	71,9%	83,0%	97,0%	98,4%	95,5%
16158	55	40	55	75	72,2%	82,8%	97,1%	98,5%	95,7%
16159	55	40	55	80	72,6%	82,6%	97,2%	98,6%	96,0%
16160	55	40	60	20	62,6%	81,5%	95,3%	96,7%	91,1%
16161	55	40	60	25	63,0%	81,3%	95,4%	96,9%	91,6%
16162	55	40	60	30	63,4%	81,1%	95,6%	97,1%	92,1%
16163	55	40	60	35	63,8%	80,9%	95,7%	97,3%	92,6%
16164	55	40	60	40	64,2%	80,7%	95,8%	97,5%	93,0%
16165	55	40	60	45	64,6%	80,5%	96,0%	97,6%	93,5%
16166	55	40	60	50	65,0%	80,3%	96,1%	97,8%	93,9%
16167	55	40	60	55	65,4%	80,1%	96,2%	97,9%	94,3%
16168	55	40	60	60	65,8%	79,9%	96,3%	98,1%	94,6%
16169	55	40	60	65	66,2%	79,7%	96,5%	98,2%	95,0%

16170	55	40	60	70	66,6%	79,5%	96,6%	98,3%	95,3%
16171	55	40	60	75	67,0%	79,3%	96,7%	98,4%	95,6%
16172	55	40	60	80	67,4%	79,0%	96,8%	98,5%	95,9%
16173	55	40	65	20	56,7%	77,8%	94,7%	96,5%	90,7%
16174	55	40	65	25	57,1%	77,6%	94,8%	96,7%	91,3%
16175	55	40	65	30	57,5%	77,3%	95,0%	96,9%	91,8%
16176	55	40	65	35	58,0%	77,1%	95,2%	97,1%	92,3%
16177	55	40	65	40	58,4%	76,9%	95,3%	97,3%	92,8%
16178	55	40	65	45	58,8%	76,6%	95,5%	97,5%	93,2%
16179	55	40	65	50	59,2%	76,4%	95,6%	97,7%	93,6%
16180	55	40	65	55	59,7%	76,2%	95,7%	97,8%	94,0%
16181	55	40	65	60	60,1%	75,9%	95,9%	98,0%	94,4%
16182	55	40	65	65	60,5%	75,7%	96,0%	98,1%	94,8%
16183	55	40	65	70	60,9%	75,5%	96,1%	98,2%	95,1%
16184	55	40	65	75	61,4%	75,2%	96,3%	98,3%	95,4%
16185	55	40	65	80	61,8%	75,0%	96,4%	98,4%	95,7%
16186	55	40	70	20	50,5%	73,6%	94,0%	96,3%	90,4%
16187	55	40	70	25	51,0%	73,3%	94,2%	96,5%	90,9%
16188	55	40	70	30	51,4%	73,0%	94,4%	96,8%	91,5%
16189	55	40	70	35	51,9%	72,8%	94,5%	97,0%	92,0%
16190	55	40	70	40	52,3%	72,5%	94,7%	97,2%	92,5%
16191	55	40	70	45	52,7%	72,3%	94,9%	97,3%	93,0%
16192	55	40	70	50	53,2%	72,0%	95,0%	97,5%	93,4%
16193	55	40	70	55	53,6%	71,7%	95,2%	97,7%	93,8%
16194	55	40	70	60	54,1%	71,5%	95,4%	97,8%	94,2%
16195	55	40	70	65	54,5%	71,2%	95,5%	98,0%	94,6%
16196	55	40	70	70	54,9%	70,9%	95,6%	98,1%	94,9%
16197	55	40	70	75	55,4%	70,7%	95,8%	98,2%	95,2%
16198	55	40	70	80	55,8%	70,4%	95,9%	98,3%	95,5%
16199	55	40	75	20	44,4%	68,8%	93,3%	96,1%	90,0%
16200	55	40	75	25	44,8%	68,5%	93,5%	96,3%	90,6%
16201	55	40	75	30	45,3%	68,3%	93,7%	96,6%	91,2%
16202	55	40	75	35	45,7%	68,0%	93,9%	96,8%	91,7%

16203	55	40	75	40	46,1%	67,7%	94,1%	97,0%	92,2%
16204	55	40	75	45	46,6%	67,4%	94,2%	97,2%	92,7%
16205	55	40	75	50	47,0%	67,1%	94,4%	97,4%	93,1%
16206	55	40	75	55	47,4%	66,8%	94,6%	97,5%	93,6%
16207	55	40	75	60	47,9%	66,5%	94,8%	97,7%	94,0%
16208	55	40	75	65	48,3%	66,2%	94,9%	97,8%	94,3%
16209	55	40	75	70	48,8%	66,0%	95,1%	98,0%	94,7%
16210	55	40	75	75	49,2%	65,7%	95,3%	98,1%	95,0%
16211	55	40	75	80	49,6%	65,4%	95,4%	98,2%	95,3%
16212	55	40	80	20	38,4%	63,7%	92,4%	95,8%	89,6%
16213	55	40	80	25	38,8%	63,4%	92,7%	96,1%	90,2%
16214	55	40	80	30	39,2%	63,1%	92,9%	96,4%	90,8%
16215	55	40	80	35	39,7%	62,8%	93,1%	96,6%	91,4%
16216	55	40	80	40	40,1%	62,4%	93,3%	96,8%	91,9%
16217	55	40	80	45	40,5%	62,1%	93,5%	97,0%	92,4%
16218	55	40	80	50	40,9%	61,8%	93,7%	97,2%	92,9%
16219	55	40	80	55	41,4%	61,5%	93,9%	97,4%	93,3%
16220	55	40	80	60	41,8%	61,2%	94,1%	97,6%	93,7%
16221	55	40	80	65	42,2%	60,9%	94,3%	97,7%	94,1%
16222	55	40	80	70	42,6%	60,6%	94,5%	97,9%	94,5%
16223	55	40	80	75	43,1%	60,3%	94,7%	98,0%	94,8%
16224	55	40	80	80	43,5%	60,0%	94,8%	98,1%	95,2%
16225	55	45	20	20	93,0%	96,8%	98,1%	97,8%	92,8%
16226	55	45	20	25	93,1%	96,8%	98,2%	97,9%	93,2%
16227	55	45	20	30	93,2%	96,7%	98,3%	98,1%	93,7%
16228	55	45	20	35	93,3%	96,7%	98,3%	98,2%	94,0%
16229	55	45	20	40	93,4%	96,6%	98,4%	98,3%	94,4%
16230	55	45	20	45	93,5%	96,6%	98,4%	98,4%	94,8%
16231	55	45	20	50	93,6%	96,6%	98,5%	98,5%	95,1%
16232	55	45	20	55	93,7%	96,5%	98,5%	98,6%	95,4%
16233	55	45	20	60	93,8%	96,5%	98,6%	98,7%	95,7%
16234	55	45	20	65	93,9%	96,4%	98,6%	98,8%	96,0%
16235	55	45	20	70	94,0%	96,4%	98,7%	98,9%	96,2%

16236	55	45	20	75	94,1%	96,3%	98,7%	99,0%	96,5%
16237	55	45	20	80	94,2%	96,3%	98,7%	99,0%	96,7%
16238	55	45	25	20	91,2%	96,0%	97,9%	97,7%	92,5%
16239	55	45	25	25	91,3%	96,0%	98,0%	97,8%	93,0%
16240	55	45	25	30	91,4%	95,9%	98,0%	98,0%	93,4%
16241	55	45	25	35	91,6%	95,9%	98,1%	98,1%	93,8%
16242	55	45	25	40	91,7%	95,8%	98,2%	98,2%	94,2%
16243	55	45	25	45	91,8%	95,7%	98,2%	98,3%	94,6%
16244	55	45	25	50	92,0%	95,7%	98,3%	98,4%	94,9%
16245	55	45	25	55	92,1%	95,6%	98,3%	98,5%	95,2%
16246	55	45	25	60	92,2%	95,6%	98,4%	98,6%	95,5%
16247	55	45	25	65	92,3%	95,5%	98,4%	98,7%	95,8%
16248	55	45	25	70	92,5%	95,5%	98,5%	98,8%	96,1%
16249	55	45	25	75	92,6%	95,4%	98,5%	98,9%	96,3%
16250	55	45	25	80	92,7%	95,4%	98,6%	99,0%	96,6%
16251	55	45	30	20	88,9%	95,0%	97,6%	97,5%	92,2%
16252	55	45	30	25	89,1%	95,0%	97,7%	97,7%	92,7%
16253	55	45	30	30	89,3%	94,9%	97,8%	97,8%	93,1%
16254	55	45	30	35	89,5%	94,8%	97,8%	98,0%	93,6%
16255	55	45	30	40	89,6%	94,8%	97,9%	98,1%	94,0%
16256	55	45	30	45	89,8%	94,7%	98,0%	98,2%	94,3%
16257	55	45	30	50	89,9%	94,6%	98,0%	98,3%	94,7%
16258	55	45	30	55	90,1%	94,6%	98,1%	98,5%	95,0%
16259	55	45	30	60	90,3%	94,5%	98,2%	98,6%	95,3%
16260	55	45	30	65	90,4%	94,4%	98,2%	98,7%	95,6%
16261	55	45	30	70	90,6%	94,4%	98,3%	98,7%	95,9%
16262	55	45	30	75	90,7%	94,3%	98,3%	98,8%	96,2%
16263	55	45	30	80	90,9%	94,2%	98,4%	98,9%	96,4%
16264	55	45	35	20	86,3%	93,8%	97,3%	97,4%	91,9%
16265	55	45	35	25	86,5%	93,7%	97,4%	97,5%	92,4%
16266	55	45	35	30	86,7%	93,7%	97,5%	97,7%	92,9%
16267	55	45	35	35	86,9%	93,6%	97,6%	97,8%	93,3%
16268	55	45	35	40	87,1%	93,5%	97,6%	98,0%	93,7%

16269	55	45	35	45	87,3%	93,4%	97,7%	98,1%	94,1%
16270	55	45	35	50	87,5%	93,3%	97,8%	98,2%	94,5%
16271	55	45	35	55	87,7%	93,3%	97,9%	98,4%	94,8%
16272	55	45	35	60	87,9%	93,2%	97,9%	98,5%	95,2%
16273	55	45	35	65	88,0%	93,1%	98,0%	98,6%	95,5%
16274	55	45	35	70	88,2%	93,0%	98,1%	98,7%	95,8%
16275	55	45	35	75	88,4%	92,9%	98,1%	98,7%	96,0%
16276	55	45	35	80	88,6%	92,8%	98,2%	98,8%	96,3%
16277	55	45	40	20	83,1%	92,3%	97,0%	97,2%	91,6%
16278	55	45	40	25	83,3%	92,2%	97,1%	97,4%	92,1%
16279	55	45	40	30	83,6%	92,1%	97,2%	97,6%	92,6%
16280	55	45	40	35	83,8%	92,0%	97,3%	97,7%	93,1%
16281	55	45	40	40	84,1%	91,9%	97,3%	97,9%	93,5%
16282	55	45	40	45	84,3%	91,8%	97,4%	98,0%	93,9%
16283	55	45	40	50	84,5%	91,7%	97,5%	98,1%	94,3%
16284	55	45	40	55	84,7%	91,6%	97,6%	98,3%	94,6%
16285	55	45	40	60	85,0%	91,5%	97,7%	98,4%	95,0%
16286	55	45	40	65	85,2%	91,4%	97,7%	98,5%	95,3%
16287	55	45	40	70	85,4%	91,3%	97,8%	98,6%	95,6%
16288	55	45	40	75	85,6%	91,2%	97,9%	98,7%	95,9%
16289	55	45	40	80	85,9%	91,1%	98,0%	98,8%	96,1%
16290	55	45	45	20	79,3%	90,5%	96,6%	97,0%	91,3%
16291	55	45	45	25	79,6%	90,4%	96,7%	97,2%	91,8%
16292	55	45	45	30	79,9%	90,3%	96,8%	97,4%	92,3%
16293	55	45	45	35	80,2%	90,2%	96,9%	97,6%	92,8%
16294	55	45	45	40	80,5%	90,1%	97,0%	97,7%	93,2%
16295	55	45	45	45	80,7%	89,9%	97,1%	97,9%	93,6%
16296	55	45	45	50	81,0%	89,8%	97,2%	98,0%	94,0%
16297	55	45	45	55	81,3%	89,7%	97,3%	98,1%	94,4%
16298	55	45	45	60	81,5%	89,6%	97,4%	98,3%	94,8%
16299	55	45	45	65	81,8%	89,5%	97,5%	98,4%	95,1%
16300	55	45	45	70	82,1%	89,3%	97,5%	98,5%	95,4%
16301	55	45	45	75	82,3%	89,2%	97,6%	98,6%	95,7%

16302	55	45	45	80	82,6%	89,1%	97,7%	98,7%	96,0%
16303	55	45	50	20	75,0%	88,3%	96,1%	96,9%	90,9%
16304	55	45	50	25	75,3%	88,2%	96,3%	97,1%	91,5%
16305	55	45	50	30	75,6%	88,1%	96,4%	97,2%	92,0%
16306	55	45	50	35	76,0%	87,9%	96,5%	97,4%	92,5%
16307	55	45	50	40	76,3%	87,8%	96,6%	97,6%	93,0%
16308	55	45	50	45	76,6%	87,7%	96,7%	97,8%	93,4%
16309	55	45	50	50	76,9%	87,5%	96,8%	97,9%	93,8%
16310	55	45	50	55	77,2%	87,4%	96,9%	98,0%	94,2%
16311	55	45	50	60	77,5%	87,2%	97,0%	98,2%	94,6%
16312	55	45	50	65	77,8%	87,1%	97,1%	98,3%	94,9%
16313	55	45	50	70	78,1%	86,9%	97,2%	98,4%	95,2%
16314	55	45	50	75	78,4%	86,8%	97,3%	98,5%	95,5%
16315	55	45	50	80	78,7%	86,6%	97,4%	98,6%	95,8%
16316	55	45	55	20	70,1%	85,7%	95,7%	96,7%	90,6%
16317	55	45	55	25	70,4%	85,6%	95,8%	96,9%	91,2%
16318	55	45	55	30	70,8%	85,4%	95,9%	97,1%	91,7%
16319	55	45	55	35	71,2%	85,3%	96,1%	97,3%	92,2%
16320	55	45	55	40	71,5%	85,1%	96,2%	97,4%	92,7%
16321	55	45	55	45	71,9%	84,9%	96,3%	97,6%	93,1%
16322	55	45	55	50	72,2%	84,8%	96,4%	97,8%	93,6%
16323	55	45	55	55	72,6%	84,6%	96,5%	97,9%	94,0%
16324	55	45	55	60	72,9%	84,4%	96,7%	98,1%	94,3%
16325	55	45	55	65	73,3%	84,2%	96,8%	98,2%	94,7%
16326	55	45	55	70	73,6%	84,1%	96,9%	98,3%	95,0%
16327	55	45	55	75	74,0%	83,9%	97,0%	98,4%	95,3%
16328	55	45	55	80	74,3%	83,7%	97,1%	98,5%	95,6%
16329	55	45	60	20	64,7%	82,7%	95,1%	96,5%	90,2%
16330	55	45	60	25	65,1%	82,5%	95,3%	96,7%	90,8%
16331	55	45	60	30	65,5%	82,3%	95,4%	96,9%	91,4%
16332	55	45	60	35	65,9%	82,1%	95,6%	97,1%	91,9%
16333	55	45	60	40	66,2%	81,9%	95,7%	97,3%	92,4%
16334	55	45	60	45	66,6%	81,7%	95,8%	97,5%	92,9%

16335	55	45	60	50	67,0%	81,5%	96,0%	97,6%	93,3%
16336	55	45	60	55	67,4%	81,3%	96,1%	97,8%	93,7%
16337	55	45	60	60	67,8%	81,1%	96,2%	97,9%	94,1%
16338	55	45	60	65	68,2%	80,9%	96,3%	98,1%	94,5%
16339	55	45	60	70	68,6%	80,7%	96,5%	98,2%	94,8%
16340	55	45	60	75	69,0%	80,5%	96,6%	98,3%	95,2%
16341	55	45	60	80	69,3%	80,3%	96,7%	98,4%	95,5%
16342	55	45	65	20	58,8%	79,1%	94,5%	96,2%	89,9%
16343	55	45	65	25	59,3%	78,9%	94,7%	96,5%	90,5%
16344	55	45	65	30	59,7%	78,7%	94,8%	96,7%	91,1%
16345	55	45	65	35	60,1%	78,5%	95,0%	96,9%	91,6%
16346	55	45	65	40	60,5%	78,2%	95,2%	97,1%	92,1%
16347	55	45	65	45	60,9%	78,0%	95,3%	97,3%	92,6%
16348	55	45	65	50	61,4%	77,8%	95,5%	97,5%	93,0%
16349	55	45	65	55	61,8%	77,6%	95,6%	97,7%	93,5%
16350	55	45	65	60	62,2%	77,3%	95,7%	97,8%	93,9%
16351	55	45	65	65	62,6%	77,1%	95,9%	97,9%	94,3%
16352	55	45	65	70	63,0%	76,9%	96,0%	98,1%	94,6%
16353	55	45	65	75	63,4%	76,6%	96,1%	98,2%	95,0%
16354	55	45	65	80	63,8%	76,4%	96,3%	98,3%	95,3%
16355	55	45	70	20	52,7%	75,1%	93,8%	96,0%	89,5%
16356	55	45	70	25	53,2%	74,8%	94,0%	96,3%	90,1%
16357	55	45	70	30	53,6%	74,6%	94,2%	96,5%	90,7%
16358	55	45	70	35	54,1%	74,3%	94,4%	96,7%	91,3%
16359	55	45	70	40	54,5%	74,1%	94,6%	97,0%	91,8%
16360	55	45	70	45	54,9%	73,8%	94,7%	97,1%	92,3%
16361	55	45	70	50	55,4%	73,6%	94,9%	97,3%	92,8%
16362	55	45	70	55	55,8%	73,3%	95,1%	97,5%	93,2%
16363	55	45	70	60	56,2%	73,0%	95,2%	97,7%	93,6%
16364	55	45	70	65	56,7%	72,8%	95,4%	97,8%	94,0%
16365	55	45	70	70	57,1%	72,5%	95,5%	98,0%	94,4%
16366	55	45	70	75	57,5%	72,3%	95,7%	98,1%	94,8%
16367	55	45	70	80	58,0%	72,0%	95,8%	98,2%	95,1%

16368	55	45	75	20	46,6%	70,5%	93,1%	95,8%	89,1%
16369	55	45	75	25	47,0%	70,2%	93,3%	96,1%	89,7%
16370	55	45	75	30	47,5%	69,9%	93,5%	96,3%	90,4%
16371	55	45	75	35	47,9%	69,7%	93,7%	96,5%	90,9%
16372	55	45	75	40	48,3%	69,4%	93,9%	96,8%	91,5%
16373	55	45	75	45	48,8%	69,1%	94,1%	97,0%	92,0%
16374	55	45	75	50	49,2%	68,8%	94,3%	97,2%	92,5%
16375	55	45	75	55	49,7%	68,5%	94,4%	97,4%	93,0%
16376	55	45	75	60	50,1%	68,3%	94,6%	97,5%	93,4%
16377	55	45	75	65	50,5%	68,0%	94,8%	97,7%	93,8%
16378	55	45	75	70	51,0%	67,7%	95,0%	97,8%	94,2%
16379	55	45	75	75	51,4%	67,4%	95,1%	98,0%	94,6%
16380	55	45	75	80	51,9%	67,1%	95,3%	98,1%	94,9%
16381	55	45	80	20	40,5%	65,5%	92,2%	95,5%	88,7%
16382	55	45	80	25	40,9%	65,2%	92,4%	95,8%	89,4%
16383	55	45	80	30	41,4%	64,9%	92,7%	96,1%	90,0%
16384	55	45	80	35	41,8%	64,6%	92,9%	96,3%	90,6%
16385	55	45	80	40	42,2%	64,3%	93,1%	96,6%	91,2%
16386	55	45	80	45	42,7%	64,0%	93,3%	96,8%	91,7%
16387	55	45	80	50	43,1%	63,7%	93,5%	97,0%	92,2%
16388	55	45	80	55	43,5%	63,4%	93,8%	97,2%	92,7%
16389	55	45	80	60	44,0%	63,1%	93,9%	97,4%	93,1%
16390	55	45	80	65	44,4%	62,8%	94,1%	97,5%	93,6%
16391	55	45	80	70	44,8%	62,5%	94,3%	97,7%	94,0%
16392	55	45	80	75	45,3%	62,1%	94,5%	97,9%	94,3%
16393	55	45	80	80	45,7%	61,8%	94,7%	98,0%	94,7%
16394	55	50	20	20	93,5%	97,0%	98,1%	97,6%	92,1%
16395	55	50	20	25	93,6%	97,0%	98,1%	97,8%	92,6%
16396	55	50	20	30	93,7%	97,0%	98,2%	97,9%	93,1%
16397	55	50	20	35	93,8%	96,9%	98,3%	98,1%	93,5%
16398	55	50	20	40	93,9%	96,9%	98,3%	98,2%	93,9%
16399	55	50	20	45	94,0%	96,8%	98,4%	98,3%	94,3%
16400	55	50	20	50	94,1%	96,8%	98,4%	98,4%	94,6%

16401	55	50	20	55	94,2%	96,8%	98,5%	98,5%	95,0%
16402	55	50	20	60	94,3%	96,7%	98,5%	98,6%	95,3%
16403	55	50	20	65	94,4%	96,7%	98,6%	98,7%	95,6%
16404	55	50	20	70	94,5%	96,6%	98,6%	98,8%	95,9%
16405	55	50	20	75	94,6%	96,6%	98,7%	98,9%	96,1%
16406	55	50	20	80	94,7%	96,6%	98,7%	99,0%	96,4%
16407	55	50	25	20	91,8%	96,3%	97,8%	97,5%	91,8%
16408	55	50	25	25	92,0%	96,3%	97,9%	97,7%	92,3%
16409	55	50	25	30	92,1%	96,2%	98,0%	97,8%	92,8%
16410	55	50	25	35	92,2%	96,2%	98,0%	97,9%	93,2%
16411	55	50	25	40	92,4%	96,1%	98,1%	98,1%	93,7%
16412	55	50	25	45	92,5%	96,1%	98,2%	98,2%	94,0%
16413	55	50	25	50	92,6%	96,0%	98,2%	98,3%	94,4%
16414	55	50	25	55	92,7%	96,0%	98,3%	98,4%	94,8%
16415	55	50	25	60	92,8%	95,9%	98,3%	98,5%	95,1%
16416	55	50	25	65	93,0%	95,9%	98,4%	98,6%	95,4%
16417	55	50	25	70	93,1%	95,8%	98,4%	98,7%	95,7%
16418	55	50	25	75	93,2%	95,8%	98,5%	98,8%	96,0%
16419	55	50	25	80	93,3%	95,7%	98,5%	98,9%	96,2%
16420	55	50	30	20	89,8%	95,4%	97,5%	97,3%	91,5%
16421	55	50	30	25	89,9%	95,3%	97,6%	97,5%	92,0%
16422	55	50	30	30	90,1%	95,3%	97,7%	97,7%	92,5%
16423	55	50	30	35	90,3%	95,2%	97,8%	97,8%	93,0%
16424	55	50	30	40	90,4%	95,1%	97,8%	98,0%	93,4%
16425	55	50	30	45	90,6%	95,1%	97,9%	98,1%	93,8%
16426	55	50	30	50	90,7%	95,0%	98,0%	98,2%	94,2%
16427	55	50	30	55	90,9%	95,0%	98,1%	98,3%	94,6%
16428	55	50	30	60	91,0%	94,9%	98,1%	98,4%	94,9%
16429	55	50	30	65	91,2%	94,8%	98,2%	98,5%	95,2%
16430	55	50	30	70	91,3%	94,8%	98,2%	98,6%	95,5%
16431	55	50	30	75	91,4%	94,7%	98,3%	98,7%	95,8%
16432	55	50	30	80	91,6%	94,6%	98,4%	98,8%	96,1%
16433	55	50	35	20	87,3%	94,3%	97,2%	97,2%	91,2%

16434	55	50	35	25	87,5%	94,2%	97,3%	97,4%	91,7%
16435	55	50	35	30	87,7%	94,1%	97,4%	97,5%	92,2%
16436	55	50	35	35	87,9%	94,0%	97,5%	97,7%	92,7%
16437	55	50	35	40	88,1%	94,0%	97,6%	97,8%	93,1%
16438	55	50	35	45	88,2%	93,9%	97,6%	98,0%	93,6%
16439	55	50	35	50	88,4%	93,8%	97,7%	98,1%	94,0%
16440	55	50	35	55	88,6%	93,7%	97,8%	98,2%	94,3%
16441	55	50	35	60	88,8%	93,7%	97,9%	98,4%	94,7%
16442	55	50	35	65	89,0%	93,6%	97,9%	98,5%	95,0%
16443	55	50	35	70	89,1%	93,5%	98,0%	98,6%	95,3%
16444	55	50	35	75	89,3%	93,4%	98,1%	98,7%	95,6%
16445	55	50	35	80	89,5%	93,3%	98,1%	98,7%	95,9%
16446	55	50	40	20	84,3%	92,9%	96,9%	97,0%	90,8%
16447	55	50	40	25	84,5%	92,8%	97,0%	97,2%	91,4%
16448	55	50	40	30	84,8%	92,7%	97,1%	97,4%	91,9%
16449	55	50	40	35	85,0%	92,6%	97,2%	97,5%	92,4%
16450	55	50	40	40	85,2%	92,5%	97,3%	97,7%	92,9%
16451	55	50	40	45	85,4%	92,4%	97,3%	97,9%	93,3%
16452	55	50	40	50	85,6%	92,3%	97,4%	98,0%	93,7%
16453	55	50	40	55	85,9%	92,2%	97,5%	98,1%	94,1%
16454	55	50	40	60	86,1%	92,1%	97,6%	98,2%	94,5%
16455	55	50	40	65	86,3%	92,0%	97,7%	98,4%	94,8%
16456	55	50	40	70	86,5%	91,9%	97,8%	98,5%	95,2%
16457	55	50	40	75	86,7%	91,8%	97,8%	98,6%	95,5%
16458	55	50	40	80	86,9%	91,8%	97,9%	98,7%	95,8%
16459	55	50	45	20	80,7%	91,2%	96,5%	96,8%	90,5%
16460	55	50	45	25	81,0%	91,1%	96,6%	97,0%	91,1%
16461	55	50	45	30	81,3%	91,0%	96,7%	97,2%	91,6%
16462	55	50	45	35	81,6%	90,9%	96,8%	97,4%	92,1%
16463	55	50	45	40	81,8%	90,7%	96,9%	97,6%	92,6%
16464	55	50	45	45	82,1%	90,6%	97,0%	97,7%	93,1%
16465	55	50	45	50	82,3%	90,5%	97,1%	97,9%	93,5%
16466	55	50	45	55	82,6%	90,4%	97,2%	98,0%	93,9%

16467	55	50	45	60	82,8%	90,3%	97,3%	98,1%	94,3%
16468	55	50	45	65	83,1%	90,2%	97,4%	98,3%	94,6%
16469	55	50	45	70	83,3%	90,1%	97,5%	98,4%	95,0%
16470	55	50	45	75	83,6%	89,9%	97,5%	98,5%	95,3%
16471	55	50	45	80	83,8%	89,8%	97,6%	98,6%	95,6%
16472	55	50	50	20	76,6%	89,1%	96,0%	96,6%	90,1%
16473	55	50	50	25	76,9%	89,0%	96,2%	96,8%	90,7%
16474	55	50	50	30	77,2%	88,9%	96,3%	97,0%	91,3%
16475	55	50	50	35	77,5%	88,7%	96,4%	97,2%	91,8%
16476	55	50	50	40	77,8%	88,6%	96,5%	97,4%	92,3%
16477	55	50	50	45	78,2%	88,5%	96,6%	97,6%	92,8%
16478	55	50	50	50	78,5%	88,3%	96,7%	97,7%	93,2%
16479	55	50	50	55	78,7%	88,2%	96,8%	97,9%	93,6%
16480	55	50	50	60	79,0%	88,1%	96,9%	98,0%	94,0%
16481	55	50	50	65	79,3%	87,9%	97,0%	98,2%	94,4%
16482	55	50	50	70	79,6%	87,8%	97,1%	98,3%	94,8%
16483	55	50	50	75	79,9%	87,7%	97,2%	98,4%	95,1%
16484	55	50	50	80	80,2%	87,5%	97,3%	98,5%	95,4%
16485	55	50	55	20	71,9%	86,7%	95,5%	96,4%	89,8%
16486	55	50	55	25	72,3%	86,5%	95,7%	96,6%	90,4%
16487	55	50	55	30	72,6%	86,4%	95,8%	96,9%	90,9%
16488	55	50	55	35	73,0%	86,2%	95,9%	97,1%	91,5%
16489	55	50	55	40	73,3%	86,1%	96,1%	97,3%	92,0%
16490	55	50	55	45	73,6%	85,9%	96,2%	97,4%	92,5%
16491	55	50	55	50	74,0%	85,8%	96,3%	97,6%	93,0%
16492	55	50	55	55	74,3%	85,6%	96,4%	97,8%	93,4%
16493	55	50	55	60	74,7%	85,4%	96,5%	97,9%	93,8%
16494	55	50	55	65	75,0%	85,3%	96,7%	98,0%	94,2%
16495	55	50	55	70	75,3%	85,1%	96,8%	98,2%	94,6%
16496	55	50	55	75	75,6%	84,9%	96,9%	98,3%	94,9%
16497	55	50	55	80	76,0%	84,8%	97,0%	98,4%	95,2%
16498	55	50	60	20	66,7%	83,8%	95,0%	96,2%	89,4%
16499	55	50	60	25	67,0%	83,6%	95,1%	96,4%	90,0%

16500	55	50	60	30	67,4%	83,4%	95,3%	96,7%	90,6%
16501	55	50	60	35	67,8%	83,2%	95,4%	96,9%	91,2%
16502	55	50	60	40	68,2%	83,1%	95,6%	97,1%	91,7%
16503	55	50	60	45	68,6%	82,9%	95,7%	97,3%	92,2%
16504	55	50	60	50	69,0%	82,7%	95,8%	97,5%	92,7%
16505	55	50	60	55	69,3%	82,5%	96,0%	97,6%	93,1%
16506	55	50	60	60	69,7%	82,3%	96,1%	97,8%	93,6%
16507	55	50	60	65	70,1%	82,1%	96,2%	97,9%	94,0%
16508	55	50	60	70	70,5%	81,9%	96,4%	98,1%	94,3%
16509	55	50	60	75	70,8%	81,7%	96,5%	98,2%	94,7%
16510	55	50	60	80	71,2%	81,5%	96,6%	98,3%	95,0%
16511	55	50	65	20	61,0%	80,4%	94,3%	96,0%	89,0%
16512	55	50	65	25	61,4%	80,2%	94,5%	96,2%	89,6%
16513	55	50	65	30	61,8%	80,0%	94,7%	96,5%	90,2%
16514	55	50	65	35	62,2%	79,8%	94,8%	96,7%	90,8%
16515	55	50	65	40	62,6%	79,6%	95,0%	96,9%	91,4%
16516	55	50	65	45	63,0%	79,3%	95,2%	97,1%	91,9%
16517	55	50	65	50	63,4%	79,1%	95,3%	97,3%	92,4%
16518	55	50	65	55	63,9%	78,9%	95,5%	97,5%	92,9%
16519	55	50	65	60	64,3%	78,7%	95,6%	97,6%	93,3%
16520	55	50	65	65	64,7%	78,5%	95,8%	97,8%	93,7%
16521	55	50	65	70	65,1%	78,3%	95,9%	97,9%	94,1%
16522	55	50	65	75	65,5%	78,0%	96,0%	98,1%	94,5%
16523	55	50	65	80	65,9%	77,8%	96,2%	98,2%	94,8%
16524	55	50	70	20	55,0%	76,5%	93,6%	95,7%	88,6%
16525	55	50	70	25	55,4%	76,3%	93,8%	96,0%	89,2%
16526	55	50	70	30	55,8%	76,0%	94,0%	96,3%	89,9%
16527	55	50	70	35	56,3%	75,8%	94,2%	96,5%	90,5%
16528	55	50	70	40	56,7%	75,5%	94,4%	96,7%	91,1%
16529	55	50	70	45	57,1%	75,3%	94,6%	96,9%	91,6%
16530	55	50	70	50	57,6%	75,1%	94,7%	97,1%	92,1%
16531	55	50	70	55	58,0%	74,8%	94,9%	97,3%	92,6%
16532	55	50	70	60	58,4%	74,6%	95,1%	97,5%	93,1%

16533	55	50	70	65	58,8%	74,3%	95,2%	97,7%	93,5%
16534	55	50	70	70	59,3%	74,1%	95,4%	97,8%	93,9%
16535	55	50	70	75	59,7%	73,8%	95,5%	98,0%	94,3%
16536	55	50	70	80	60,1%	73,6%	95,7%	98,1%	94,6%
16537	55	50	75	20	48,8%	72,1%	92,8%	95,5%	88,1%
16538	55	50	75	25	49,2%	71,8%	93,1%	95,8%	88,8%
16539	55	50	75	30	49,7%	71,6%	93,3%	96,0%	89,5%
16540	55	50	75	35	50,1%	71,3%	93,5%	96,3%	90,1%
16541	55	50	75	40	50,6%	71,0%	93,7%	96,5%	90,7%
16542	55	50	75	45	51,0%	70,8%	93,9%	96,7%	91,3%
16543	55	50	75	50	51,4%	70,5%	94,1%	97,0%	91,8%
16544	55	50	75	55	51,9%	70,2%	94,3%	97,2%	92,3%
16545	55	50	75	60	52,3%	69,9%	94,5%	97,3%	92,8%
16546	55	50	75	65	52,8%	69,7%	94,6%	97,5%	93,2%
16547	55	50	75	70	53,2%	69,4%	94,8%	97,7%	93,6%
16548	55	50	75	75	53,6%	69,1%	95,0%	97,8%	94,0%
16549	55	50	75	80	54,1%	68,8%	95,1%	98,0%	94,4%
16550	55	50	80	20	42,7%	67,2%	92,0%	95,2%	87,7%
16551	55	50	80	25	43,1%	66,9%	92,2%	95,5%	88,4%
16552	55	50	80	30	43,5%	66,6%	92,5%	95,8%	89,1%
16553	55	50	80	35	44,0%	66,4%	92,7%	96,1%	89,7%
16554	55	50	80	40	44,4%	66,1%	92,9%	96,3%	90,4%
16555	55	50	80	45	44,8%	65,8%	93,1%	96,6%	90,9%
16556	55	50	80	50	45,3%	65,5%	93,4%	96,8%	91,5%
16557	55	50	80	55	45,7%	65,2%	93,6%	97,0%	92,0%
16558	55	50	80	60	46,2%	64,9%	93,8%	97,2%	92,5%
16559	55	50	80	65	46,6%	64,6%	94,0%	97,4%	93,0%
16560	55	50	80	70	47,0%	64,3%	94,1%	97,5%	93,4%
16561	55	50	80	75	47,5%	64,0%	94,3%	97,7%	93,8%
16562	55	50	80	80	47,9%	63,7%	94,5%	97,8%	94,2%
16563	55	55	20	20	94,0%	97,3%	98,0%	97,5%	91,4%
16564	55	55	20	25	94,1%	97,2%	98,1%	97,6%	91,9%
16565	55	55	20	30	94,2%	97,2%	98,1%	97,8%	92,4%

16566	55	55	20	35	94,3%	97,2%	98,2%	97,9%	92,9%
16567	55	55	20	40	94,4%	97,1%	98,3%	98,1%	93,3%
16568	55	55	20	45	94,5%	97,1%	98,3%	98,2%	93,7%
16569	55	55	20	50	94,6%	97,0%	98,4%	98,3%	94,1%
16570	55	55	20	55	94,7%	97,0%	98,4%	98,4%	94,5%
16571	55	55	20	60	94,8%	97,0%	98,5%	98,5%	94,8%
16572	55	55	20	65	94,9%	96,9%	98,5%	98,6%	95,2%
16573	55	55	20	70	94,9%	96,9%	98,6%	98,7%	95,5%
16574	55	55	20	75	95,0%	96,8%	98,6%	98,8%	95,8%
16575	55	55	20	80	95,1%	96,8%	98,7%	98,9%	96,0%
16576	55	55	25	20	92,5%	96,6%	97,8%	97,3%	91,1%
16577	55	55	25	25	92,6%	96,5%	97,8%	97,5%	91,6%
16578	55	55	25	30	92,7%	96,5%	97,9%	97,6%	92,1%
16579	55	55	25	35	92,8%	96,4%	98,0%	97,8%	92,6%
16580	55	55	25	40	93,0%	96,4%	98,0%	97,9%	93,1%
16581	55	55	25	45	93,1%	96,3%	98,1%	98,1%	93,5%
16582	55	55	25	50	93,2%	96,3%	98,2%	98,2%	93,9%
16583	55	55	25	55	93,3%	96,3%	98,2%	98,3%	94,3%
16584	55	55	25	60	93,4%	96,2%	98,3%	98,4%	94,6%
16585	55	55	25	65	93,5%	96,2%	98,3%	98,5%	95,0%
16586	55	55	25	70	93,6%	96,1%	98,4%	98,6%	95,3%
16587	55	55	25	75	93,7%	96,1%	98,4%	98,7%	95,6%
16588	55	55	25	80	93,8%	96,0%	98,5%	98,8%	95,9%
16589	55	55	30	20	90,6%	95,7%	97,5%	97,1%	90,7%
16590	55	55	30	25	90,7%	95,7%	97,5%	97,3%	91,3%
16591	55	55	30	30	90,9%	95,6%	97,6%	97,5%	91,8%
16592	55	55	30	35	91,0%	95,6%	97,7%	97,7%	92,3%
16593	55	55	30	40	91,2%	95,5%	97,8%	97,8%	92,8%
16594	55	55	30	45	91,3%	95,4%	97,9%	98,0%	93,2%
16595	55	55	30	50	91,4%	95,4%	97,9%	98,1%	93,7%
16596	55	55	30	55	91,6%	95,3%	98,0%	98,2%	94,0%
16597	55	55	30	60	91,7%	95,3%	98,1%	98,3%	94,4%
16598	55	55	30	65	91,8%	95,2%	98,1%	98,4%	94,8%

16599	55	55	30	70	92,0%	95,1%	98,2%	98,5%	95,1%
16600	55	55	30	75	92,1%	95,1%	98,2%	98,6%	95,4%
16601	55	55	30	80	92,2%	95,0%	98,3%	98,7%	95,7%
16602	55	55	35	20	88,2%	94,7%	97,1%	97,0%	90,4%
16603	55	55	35	25	88,4%	94,6%	97,2%	97,2%	91,0%
16604	55	55	35	30	88,6%	94,5%	97,3%	97,3%	91,5%
16605	55	55	35	35	88,8%	94,5%	97,4%	97,5%	92,0%
16606	55	55	35	40	89,0%	94,4%	97,5%	97,7%	92,5%
16607	55	55	35	45	89,1%	94,3%	97,6%	97,8%	93,0%
16608	55	55	35	50	89,3%	94,3%	97,7%	98,0%	93,4%
16609	55	55	35	55	89,5%	94,2%	97,7%	98,1%	93,8%
16610	55	55	35	60	89,6%	94,1%	97,8%	98,2%	94,2%
16611	55	55	35	65	89,8%	94,0%	97,9%	98,3%	94,6%
16612	55	55	35	70	90,0%	94,0%	97,9%	98,5%	94,9%
16613	55	55	35	75	90,1%	93,9%	98,0%	98,6%	95,2%
16614	55	55	35	80	90,3%	93,8%	98,1%	98,6%	95,5%
16615	55	55	40	20	85,4%	93,4%	96,8%	96,8%	90,0%
16616	55	55	40	25	85,7%	93,3%	96,9%	97,0%	90,6%
16617	55	55	40	30	85,9%	93,2%	97,0%	97,2%	91,2%
16618	55	55	40	35	86,1%	93,1%	97,1%	97,4%	91,7%
16619	55	55	40	40	86,3%	93,0%	97,2%	97,5%	92,2%
16620	55	55	40	45	86,5%	93,0%	97,3%	97,7%	92,7%
16621	55	55	40	50	86,7%	92,9%	97,4%	97,8%	93,1%
16622	55	55	40	55	86,9%	92,8%	97,4%	98,0%	93,6%
16623	55	55	40	60	87,1%	92,7%	97,5%	98,1%	94,0%
16624	55	55	40	65	87,3%	92,6%	97,6%	98,2%	94,3%
16625	55	55	40	70	87,5%	92,5%	97,7%	98,4%	94,7%
16626	55	55	40	75	87,7%	92,4%	97,8%	98,5%	95,0%
16627	55	55	40	80	87,9%	92,3%	97,8%	98,6%	95,3%
16628	55	55	45	20	82,1%	91,8%	96,4%	96,6%	89,6%
16629	55	55	45	25	82,3%	91,7%	96,5%	96,8%	90,3%
16630	55	55	45	30	82,6%	91,6%	96,6%	97,0%	90,8%
16631	55	55	45	35	82,8%	91,5%	96,7%	97,2%	91,4%

16632	55	55	45	40	83,1%	91,4%	96,8%	97,4%	91,9%
16633	55	55	45	45	83,3%	91,3%	96,9%	97,6%	92,4%
16634	55	55	45	50	83,6%	91,2%	97,0%	97,7%	92,9%
16635	55	55	45	55	83,8%	91,1%	97,1%	97,9%	93,3%
16636	55	55	45	60	84,1%	91,0%	97,2%	98,0%	93,7%
16637	55	55	45	65	84,3%	90,9%	97,3%	98,1%	94,1%
16638	55	55	45	70	84,5%	90,7%	97,4%	98,3%	94,5%
16639	55	55	45	75	84,8%	90,6%	97,5%	98,4%	94,8%
16640	55	55	45	80	85,0%	90,5%	97,5%	98,5%	95,2%
16641	55	55	50	20	78,2%	89,9%	95,9%	96,4%	89,2%
16642	55	55	50	25	78,5%	89,8%	96,0%	96,6%	89,9%
16643	55	55	50	30	78,8%	89,6%	96,2%	96,8%	90,5%
16644	55	55	50	35	79,1%	89,5%	96,3%	97,0%	91,1%
16645	55	55	50	40	79,3%	89,4%	96,4%	97,2%	91,6%
16646	55	55	50	45	79,6%	89,3%	96,5%	97,4%	92,1%
16647	55	55	50	50	79,9%	89,1%	96,6%	97,6%	92,6%
16648	55	55	50	55	80,2%	89,0%	96,7%	97,7%	93,1%
16649	55	55	50	60	80,5%	88,9%	96,8%	97,9%	93,5%
16650	55	55	50	65	80,7%	88,7%	96,9%	98,0%	93,9%
16651	55	55	50	70	81,0%	88,6%	97,0%	98,1%	94,3%
16652	55	55	50	75	81,3%	88,5%	97,1%	98,3%	94,6%
16653	55	55	50	80	81,6%	88,3%	97,2%	98,4%	95,0%
16654	55	55	55	20	73,7%	87,6%	95,4%	96,2%	88,8%
16655	55	55	55	25	74,0%	87,4%	95,5%	96,4%	89,5%
16656	55	55	55	30	74,3%	87,3%	95,7%	96,6%	90,1%
16657	55	55	55	35	74,7%	87,1%	95,8%	96,8%	90,7%
16658	55	55	55	40	75,0%	87,0%	95,9%	97,1%	91,3%
16659	55	55	55	45	75,3%	86,8%	96,1%	97,2%	91,8%
16660	55	55	55	50	75,7%	86,7%	96,2%	97,4%	92,3%
16661	55	55	55	55	76,0%	86,5%	96,3%	97,6%	92,8%
16662	55	55	55	60	76,3%	86,4%	96,4%	97,7%	93,2%
16663	55	55	55	65	76,6%	86,2%	96,6%	97,9%	93,7%
16664	55	55	55	70	76,9%	86,1%	96,7%	98,0%	94,0%

16665	55	55	55	75	77,2%	85,9%	96,8%	98,2%	94,4%
16666	55	55	55	80	77,6%	85,8%	96,9%	98,3%	94,8%
16667	55	55	60	20	68,6%	84,8%	94,8%	95,9%	88,4%
16668	55	55	60	25	69,0%	84,7%	95,0%	96,2%	89,1%
16669	55	55	60	30	69,4%	84,5%	95,1%	96,4%	89,8%
16670	55	55	60	35	69,7%	84,3%	95,3%	96,7%	90,4%
16671	55	55	60	40	70,1%	84,1%	95,4%	96,9%	90,9%
16672	55	55	60	45	70,5%	84,0%	95,6%	97,1%	91,5%
16673	55	55	60	50	70,8%	83,8%	95,7%	97,3%	92,0%
16674	55	55	60	55	71,2%	83,6%	95,9%	97,4%	92,5%
16675	55	55	60	60	71,6%	83,4%	96,0%	97,6%	93,0%
16676	55	55	60	65	71,9%	83,2%	96,1%	97,8%	93,4%
16677	55	55	60	70	72,3%	83,1%	96,2%	97,9%	93,8%
16678	55	55	60	75	72,6%	82,9%	96,4%	98,0%	94,2%
16679	55	55	60	80	73,0%	82,7%	96,5%	98,2%	94,6%
16680	55	55	65	20	63,1%	81,6%	94,2%	95,7%	88,0%
16681	55	55	65	25	63,5%	81,4%	94,3%	96,0%	88,7%
16682	55	55	65	30	63,9%	81,2%	94,5%	96,2%	89,4%
16683	55	55	65	35	64,3%	81,0%	94,7%	96,5%	90,0%
16684	55	55	65	40	64,7%	80,8%	94,9%	96,7%	90,6%
16685	55	55	65	45	65,1%	80,6%	95,0%	96,9%	91,2%
16686	55	55	65	50	65,5%	80,4%	95,2%	97,1%	91,7%
16687	55	55	65	55	65,9%	80,2%	95,3%	97,3%	92,2%
16688	55	55	65	60	66,3%	80,0%	95,5%	97,5%	92,7%
16689	55	55	65	65	66,7%	79,8%	95,6%	97,6%	93,1%
16690	55	55	65	70	67,1%	79,6%	95,8%	97,8%	93,6%
16691	55	55	65	75	67,4%	79,4%	95,9%	97,9%	94,0%
16692	55	55	65	80	67,8%	79,1%	96,0%	98,1%	94,3%
16693	55	55	70	20	57,1%	77,9%	93,4%	95,4%	87,6%
16694	55	55	70	25	57,6%	77,7%	93,6%	95,7%	88,3%
16695	55	55	70	30	58,0%	77,4%	93,8%	96,0%	89,0%
16696	55	55	70	35	58,4%	77,2%	94,0%	96,2%	89,6%
16697	55	55	70	40	58,9%	77,0%	94,2%	96,5%	90,2%

16698	55	55	70	45	59,3%	76,7%	94,4%	96,7%	90,8%
16699	55	55	70	50	59,7%	76,5%	94,6%	96,9%	91,4%
16700	55	55	70	55	60,1%	76,3%	94,7%	97,1%	91,9%
16701	55	55	70	60	60,6%	76,0%	94,9%	97,3%	92,4%
16702	55	55	70	65	61,0%	75,8%	95,1%	97,5%	92,9%
16703	55	55	70	70	61,4%	75,6%	95,2%	97,6%	93,3%
16704	55	55	70	75	61,8%	75,3%	95,4%	97,8%	93,7%
16705	55	55	70	80	62,2%	75,1%	95,5%	97,9%	94,1%
16706	55	55	75	20	51,0%	73,7%	92,6%	95,1%	87,1%
16707	55	55	75	25	51,5%	73,4%	92,9%	95,5%	87,8%
16708	55	55	75	30	51,9%	73,1%	93,1%	95,7%	88,6%
16709	55	55	75	35	52,3%	72,9%	93,3%	96,0%	89,2%
16710	55	55	75	40	52,8%	72,6%	93,5%	96,3%	89,9%
16711	55	55	75	45	53,2%	72,4%	93,7%	96,5%	90,5%
16712	55	55	75	50	53,7%	72,1%	93,9%	96,7%	91,1%
16713	55	55	75	55	54,1%	71,8%	94,1%	96,9%	91,6%
16714	55	55	75	60	54,5%	71,6%	94,3%	97,1%	92,1%
16715	55	55	75	65	55,0%	71,3%	94,5%	97,3%	92,6%
16716	55	55	75	70	55,4%	71,0%	94,6%	97,5%	93,1%
16717	55	55	75	75	55,8%	70,8%	94,8%	97,7%	93,5%
16718	55	55	75	80	56,3%	70,5%	95,0%	97,8%	93,9%
16719	55	55	80	20	44,9%	68,9%	91,7%	94,9%	86,6%
16720	55	55	80	25	45,3%	68,7%	92,0%	95,2%	87,4%
16721	55	55	80	30	45,7%	68,4%	92,2%	95,5%	88,1%
16722	55	55	80	35	46,2%	68,1%	92,5%	95,8%	88,8%
16723	55	55	80	40	46,6%	67,8%	92,7%	96,0%	89,5%
16724	55	55	80	45	47,0%	67,5%	92,9%	96,3%	90,1%
16725	55	55	80	50	47,5%	67,2%	93,2%	96,5%	90,7%
16726	55	55	80	55	47,9%	66,9%	93,4%	96,8%	91,3%
16727	55	55	80	60	48,4%	66,7%	93,6%	97,0%	91,8%
16728	55	55	80	65	48,8%	66,4%	93,8%	97,2%	92,3%
16729	55	55	80	70	49,2%	66,1%	94,0%	97,3%	92,8%
16730	55	55	80	75	49,7%	65,8%	94,2%	97,5%	93,2%

16731	55	55	80	80	50,1%	65,5%	94,3%	97,7%	93,6%
16732	55	60	20	20	94,5%	97,5%	97,9%	97,3%	90,6%
16733	55	60	20	25	94,6%	97,4%	98,0%	97,4%	91,2%
16734	55	60	20	30	94,7%	97,4%	98,1%	97,6%	91,7%
16735	55	60	20	35	94,8%	97,4%	98,1%	97,8%	92,2%
16736	55	60	20	40	94,9%	97,3%	98,2%	97,9%	92,7%
16737	55	60	20	45	94,9%	97,3%	98,3%	98,0%	93,2%
16738	55	60	20	50	95,0%	97,3%	98,3%	98,2%	93,6%
16739	55	60	20	55	95,1%	97,2%	98,4%	98,3%	94,0%
16740	55	60	20	60	95,2%	97,2%	98,4%	98,4%	94,4%
16741	55	60	20	65	95,3%	97,2%	98,5%	98,5%	94,7%
16742	55	60	20	70	95,4%	97,1%	98,5%	98,6%	95,0%
16743	55	60	20	75	95,4%	97,1%	98,6%	98,7%	95,4%
16744	55	60	20	80	95,5%	97,0%	98,6%	98,8%	95,6%
16745	55	60	25	20	93,1%	96,8%	97,7%	97,1%	90,3%
16746	55	60	25	25	93,2%	96,8%	97,8%	97,3%	90,8%
16747	55	60	25	30	93,3%	96,7%	97,8%	97,5%	91,4%
16748	55	60	25	35	93,4%	96,7%	97,9%	97,6%	91,9%
16749	55	60	25	40	93,5%	96,7%	98,0%	97,8%	92,4%
16750	55	60	25	45	93,6%	96,6%	98,0%	97,9%	92,9%
16751	55	60	25	50	93,7%	96,6%	98,1%	98,1%	93,3%
16752	55	60	25	55	93,8%	96,5%	98,2%	98,2%	93,7%
16753	55	60	25	60	93,9%	96,5%	98,2%	98,3%	94,1%
16754	55	60	25	65	94,0%	96,4%	98,3%	98,4%	94,5%
16755	55	60	25	70	94,1%	96,4%	98,3%	98,5%	94,8%
16756	55	60	25	75	94,2%	96,3%	98,4%	98,6%	95,2%
16757	55	60	25	80	94,3%	96,3%	98,4%	98,7%	95,5%
16758	55	60	30	20	91,3%	96,0%	97,4%	96,9%	89,9%
16759	55	60	30	25	91,4%	96,0%	97,5%	97,1%	90,5%
16760	55	60	30	30	91,6%	95,9%	97,6%	97,3%	91,1%
16761	55	60	30	35	91,7%	95,9%	97,6%	97,5%	91,6%
16762	55	60	30	40	91,8%	95,8%	97,7%	97,6%	92,1%
16763	55	60	30	45	92,0%	95,8%	97,8%	97,8%	92,6%

16764	55	60	30	50	92,1%	95,7%	97,9%	97,9%	93,1%
16765	55	60	30	55	92,2%	95,7%	97,9%	98,1%	93,5%
16766	55	60	30	60	92,4%	95,6%	98,0%	98,2%	93,9%
16767	55	60	30	65	92,5%	95,6%	98,1%	98,3%	94,3%
16768	55	60	30	70	92,6%	95,5%	98,1%	98,4%	94,6%
16769	55	60	30	75	92,7%	95,4%	98,2%	98,5%	95,0%
16770	55	60	30	80	92,8%	95,4%	98,2%	98,6%	95,3%
16771	55	60	35	20	89,1%	95,0%	97,0%	96,7%	89,5%
16772	55	60	35	25	89,3%	95,0%	97,1%	96,9%	90,1%
16773	55	60	35	30	89,5%	94,9%	97,2%	97,1%	90,7%
16774	55	60	35	35	89,6%	94,9%	97,3%	97,3%	91,3%
16775	55	60	35	40	89,8%	94,8%	97,4%	97,5%	91,8%
16776	55	60	35	45	90,0%	94,7%	97,5%	97,7%	92,3%
16777	55	60	35	50	90,1%	94,7%	97,6%	97,8%	92,8%
16778	55	60	35	55	90,3%	94,6%	97,7%	98,0%	93,2%
16779	55	60	35	60	90,4%	94,5%	97,7%	98,1%	93,7%
16780	55	60	35	65	90,6%	94,5%	97,8%	98,2%	94,1%
16781	55	60	35	70	90,7%	94,4%	97,9%	98,3%	94,4%
16782	55	60	35	75	90,9%	94,3%	98,0%	98,4%	94,8%
16783	55	60	35	80	91,0%	94,3%	98,0%	98,5%	95,1%
16784	55	60	40	20	86,5%	93,8%	96,7%	96,5%	89,1%
16785	55	60	40	25	86,7%	93,8%	96,8%	96,8%	89,8%
16786	55	60	40	30	86,9%	93,7%	96,9%	97,0%	90,4%
16787	55	60	40	35	87,1%	93,6%	97,0%	97,2%	91,0%
16788	55	60	40	40	87,3%	93,5%	97,1%	97,3%	91,5%
16789	55	60	40	45	87,5%	93,5%	97,2%	97,5%	92,0%
16790	55	60	40	50	87,7%	93,4%	97,3%	97,7%	92,5%
16791	55	60	40	55	87,9%	93,3%	97,4%	97,8%	93,0%
16792	55	60	40	60	88,1%	93,2%	97,4%	98,0%	93,4%
16793	55	60	40	65	88,2%	93,1%	97,5%	98,1%	93,8%
16794	55	60	40	70	88,4%	93,0%	97,6%	98,2%	94,2%
16795	55	60	40	75	88,6%	93,0%	97,7%	98,3%	94,6%
16796	55	60	40	80	88,8%	92,9%	97,8%	98,5%	94,9%

16797	55	60	45	20	83,4%	92,4%	96,2%	96,3%	88,7%
16798	55	60	45	25	83,6%	92,3%	96,4%	96,6%	89,4%
16799	55	60	45	30	83,8%	92,2%	96,5%	96,8%	90,0%
16800	55	60	45	35	84,1%	92,1%	96,6%	97,0%	90,6%
16801	55	60	45	40	84,3%	92,0%	96,7%	97,2%	91,2%
16802	55	60	45	45	84,5%	91,9%	96,8%	97,4%	91,7%
16803	55	60	45	50	84,8%	91,8%	96,9%	97,5%	92,2%
16804	55	60	45	55	85,0%	91,7%	97,0%	97,7%	92,7%
16805	55	60	45	60	85,2%	91,6%	97,1%	97,9%	93,1%
16806	55	60	45	65	85,4%	91,5%	97,2%	98,0%	93,6%
16807	55	60	45	70	85,7%	91,4%	97,3%	98,1%	94,0%
16808	55	60	45	75	85,9%	91,3%	97,4%	98,2%	94,3%
16809	55	60	45	80	86,1%	91,2%	97,5%	98,4%	94,7%
16810	55	60	50	20	79,6%	90,6%	95,8%	96,1%	88,3%
16811	55	60	50	25	79,9%	90,5%	95,9%	96,4%	89,0%
16812	55	60	50	30	80,2%	90,3%	96,0%	96,6%	89,6%
16813	55	60	50	35	80,5%	90,2%	96,2%	96,8%	90,3%
16814	55	60	50	40	80,8%	90,1%	96,3%	97,0%	90,8%
16815	55	60	50	45	81,0%	90,0%	96,4%	97,2%	91,4%
16816	55	60	50	50	81,3%	89,9%	96,5%	97,4%	91,9%
16817	55	60	50	55	81,6%	89,8%	96,6%	97,6%	92,4%
16818	55	60	50	60	81,8%	89,6%	96,7%	97,7%	92,9%
16819	55	60	50	65	82,1%	89,5%	96,9%	97,9%	93,3%
16820	55	60	50	70	82,3%	89,4%	97,0%	98,0%	93,7%
16821	55	60	50	75	82,6%	89,3%	97,1%	98,1%	94,1%
16822	55	60	50	80	82,9%	89,1%	97,1%	98,3%	94,5%
16823	55	60	55	20	75,3%	88,4%	95,2%	95,9%	87,9%
16824	55	60	55	25	75,7%	88,3%	95,4%	96,1%	88,6%
16825	55	60	55	30	76,0%	88,1%	95,5%	96,4%	89,2%
16826	55	60	55	35	76,3%	88,0%	95,7%	96,6%	89,9%
16827	55	60	55	40	76,6%	87,9%	95,8%	96,8%	90,5%
16828	55	60	55	45	76,9%	87,7%	96,0%	97,0%	91,1%
16829	55	60	55	50	77,3%	87,6%	96,1%	97,2%	91,6%

16830	55	60	55	55	77,6%	87,4%	96,2%	97,4%	92,1%
16831	55	60	55	60	77,9%	87,3%	96,3%	97,6%	92,6%
16832	55	60	55	65	78,2%	87,1%	96,4%	97,7%	93,1%
16833	55	60	55	70	78,5%	87,0%	96,6%	97,9%	93,5%
16834	55	60	55	75	78,8%	86,8%	96,7%	98,0%	93,9%
16835	55	60	55	80	79,1%	86,7%	96,8%	98,2%	94,3%
16836	55	60	60	20	70,5%	85,8%	94,6%	95,6%	87,4%
16837	55	60	60	25	70,8%	85,7%	94,8%	95,9%	88,1%
16838	55	60	60	30	71,2%	85,5%	95,0%	96,2%	88,8%
16839	55	60	60	35	71,6%	85,3%	95,1%	96,4%	89,5%
16840	55	60	60	40	71,9%	85,2%	95,3%	96,6%	90,1%
16841	55	60	60	45	72,3%	85,0%	95,4%	96,9%	90,7%
16842	55	60	60	50	72,6%	84,8%	95,6%	97,1%	91,3%
16843	55	60	60	55	73,0%	84,7%	95,7%	97,3%	91,8%
16844	55	60	60	60	73,3%	84,5%	95,9%	97,4%	92,3%
16845	55	60	60	65	73,7%	84,3%	96,0%	97,6%	92,8%
16846	55	60	60	70	74,0%	84,1%	96,1%	97,8%	93,2%
16847	55	60	60	75	74,3%	84,0%	96,3%	97,9%	93,7%
16848	55	60	60	80	74,7%	83,8%	96,4%	98,0%	94,0%
16849	55	60	65	20	65,1%	82,8%	94,0%	95,4%	87,0%
16850	55	60	65	25	65,5%	82,6%	94,2%	95,7%	87,7%
16851	55	60	65	30	65,9%	82,4%	94,4%	95,9%	88,4%
16852	55	60	65	35	66,3%	82,2%	94,5%	96,2%	89,1%
16853	55	60	65	40	66,7%	82,0%	94,7%	96,4%	89,8%
16854	55	60	65	45	67,1%	81,8%	94,9%	96,7%	90,4%
16855	55	60	65	50	67,5%	81,6%	95,0%	96,9%	91,0%
16856	55	60	65	55	67,8%	81,4%	95,2%	97,1%	91,5%
16857	55	60	65	60	68,2%	81,2%	95,3%	97,3%	92,0%
16858	55	60	65	65	68,6%	81,0%	95,5%	97,4%	92,5%
16859	55	60	65	70	69,0%	80,8%	95,6%	97,6%	93,0%
16860	55	60	65	75	69,4%	80,6%	95,8%	97,8%	93,4%
16861	55	60	65	80	69,7%	80,4%	95,9%	97,9%	93,8%
16862	55	60	70	20	59,3%	79,2%	93,2%	95,1%	86,5%

16863	55	60	70	25	59,7%	79,0%	93,4%	95,4%	87,3%
16864	55	60	70	30	60,1%	78,8%	93,7%	95,7%	88,0%
16865	55	60	70	35	60,6%	78,6%	93,9%	96,0%	88,7%
16866	55	60	70	40	61,0%	78,3%	94,0%	96,2%	89,4%
16867	55	60	70	45	61,4%	78,1%	94,2%	96,5%	90,0%
16868	55	60	70	50	61,8%	77,9%	94,4%	96,7%	90,6%
16869	55	60	70	55	62,2%	77,7%	94,6%	96,9%	91,2%
16870	55	60	70	60	62,7%	77,4%	94,8%	97,1%	91,7%
16871	55	60	70	65	63,1%	77,2%	94,9%	97,3%	92,2%
16872	55	60	70	70	63,5%	77,0%	95,1%	97,5%	92,7%
16873	55	60	70	75	63,9%	76,7%	95,2%	97,6%	93,1%
16874	55	60	70	80	64,3%	76,5%	95,4%	97,8%	93,6%
16875	55	60	75	20	53,2%	75,2%	92,4%	94,8%	86,0%
16876	55	60	75	25	53,7%	74,9%	92,6%	95,1%	86,8%
16877	55	60	75	30	54,1%	74,7%	92,9%	95,4%	87,6%
16878	55	60	75	35	54,5%	74,4%	93,1%	95,7%	88,3%
16879	55	60	75	40	55,0%	74,2%	93,3%	96,0%	89,0%
16880	55	60	75	45	55,4%	73,9%	93,5%	96,3%	89,6%
16881	55	60	75	50	55,9%	73,7%	93,7%	96,5%	90,2%
16882	55	60	75	55	56,3%	73,4%	93,9%	96,7%	90,8%
16883	55	60	75	60	56,7%	73,2%	94,1%	96,9%	91,4%
16884	55	60	75	65	57,2%	72,9%	94,3%	97,1%	91,9%
16885	55	60	75	70	57,6%	72,6%	94,5%	97,3%	92,4%
16886	55	60	75	75	58,0%	72,4%	94,6%	97,5%	92,9%
16887	55	60	75	80	58,4%	72,1%	94,8%	97,7%	93,3%
16888	55	60	80	20	47,1%	70,6%	91,5%	94,5%	85,5%
16889	55	60	80	25	47,5%	70,3%	91,8%	94,8%	86,3%
16890	55	60	80	30	47,9%	70,1%	92,0%	95,2%	87,1%
16891	55	60	80	35	48,4%	69,8%	92,3%	95,5%	87,9%
16892	55	60	80	40	48,8%	69,5%	92,5%	95,8%	88,6%
16893	55	60	80	45	49,3%	69,2%	92,7%	96,0%	89,2%
16894	55	60	80	50	49,7%	68,9%	92,9%	96,3%	89,9%
16895	55	60	80	55	50,1%	68,7%	93,2%	96,5%	90,5%

16896	55	60	80	60	50,6%	68,4%	93,4%	96,7%	91,1%
16897	55	60	80	65	51,0%	68,1%	93,6%	97,0%	91,6%
16898	55	60	80	70	51,5%	67,8%	93,8%	97,2%	92,1%
16899	55	60	80	75	51,9%	67,5%	94,0%	97,3%	92,6%
16900	55	60	80	80	52,3%	67,2%	94,2%	97,5%	93,1%
16901	55	65	20	20	95,0%	97,6%	97,9%	97,1%	89,8%
16902	55	65	20	25	95,0%	97,6%	98,0%	97,3%	90,4%
16903	55	65	20	30	95,1%	97,6%	98,0%	97,4%	91,0%
16904	55	65	20	35	95,2%	97,6%	98,1%	97,6%	91,5%
16905	55	65	20	40	95,3%	97,5%	98,1%	97,8%	92,0%
16906	55	65	20	45	95,4%	97,5%	98,2%	97,9%	92,5%
16907	55	65	20	50	95,4%	97,5%	98,3%	98,0%	93,0%
16908	55	65	20	55	95,5%	97,4%	98,3%	98,2%	93,4%
16909	55	65	20	60	95,6%	97,4%	98,4%	98,3%	93,8%
16910	55	65	20	65	95,7%	97,4%	98,4%	98,4%	94,2%
16911	55	65	20	70	95,7%	97,3%	98,5%	98,5%	94,6%
16912	55	65	20	75	95,8%	97,3%	98,5%	98,6%	94,9%
16913	55	65	20	80	95,9%	97,3%	98,6%	98,7%	95,2%
16914	55	65	25	20	93,6%	97,1%	97,6%	96,9%	89,4%
16915	55	65	25	25	93,7%	97,0%	97,7%	97,1%	90,0%
16916	55	65	25	30	93,8%	97,0%	97,8%	97,3%	90,6%
16917	55	65	25	35	93,9%	96,9%	97,8%	97,4%	91,2%
16918	55	65	25	40	94,0%	96,9%	97,9%	97,6%	91,7%
16919	55	65	25	45	94,1%	96,9%	98,0%	97,8%	92,2%
16920	55	65	25	50	94,2%	96,8%	98,0%	97,9%	92,7%
16921	55	65	25	55	94,3%	96,8%	98,1%	98,1%	93,2%
16922	55	65	25	60	94,4%	96,7%	98,2%	98,2%	93,6%
16923	55	65	25	65	94,5%	96,7%	98,2%	98,3%	94,0%
16924	55	65	25	70	94,6%	96,7%	98,3%	98,4%	94,4%
16925	55	65	25	75	94,7%	96,6%	98,3%	98,5%	94,7%
16926	55	65	25	80	94,8%	96,6%	98,4%	98,6%	95,0%
16927	55	65	30	20	92,0%	96,3%	97,3%	96,7%	89,0%
16928	55	65	30	25	92,1%	96,3%	97,4%	96,9%	89,6%

16929	55	65	30	30	92,2%	96,2%	97,5%	97,1%	90,3%
16930	55	65	30	35	92,4%	96,2%	97,6%	97,3%	90,8%
16931	55	65	30	40	92,5%	96,1%	97,6%	97,5%	91,4%
16932	55	65	30	45	92,6%	96,1%	97,7%	97,6%	91,9%
16933	55	65	30	50	92,7%	96,0%	97,8%	97,8%	92,4%
16934	55	65	30	55	92,8%	96,0%	97,9%	97,9%	92,9%
16935	55	65	30	60	93,0%	95,9%	97,9%	98,1%	93,3%
16936	55	65	30	65	93,1%	95,9%	98,0%	98,2%	93,7%
16937	55	65	30	70	93,2%	95,8%	98,1%	98,3%	94,1%
16938	55	65	30	75	93,3%	95,8%	98,1%	98,4%	94,5%
16939	55	65	30	80	93,4%	95,7%	98,2%	98,5%	94,8%
16940	55	65	35	20	90,0%	95,4%	97,0%	96,5%	88,6%
16941	55	65	35	25	90,1%	95,3%	97,1%	96,7%	89,3%
16942	55	65	35	30	90,3%	95,3%	97,2%	96,9%	89,9%
16943	55	65	35	35	90,4%	95,2%	97,2%	97,1%	90,5%
16944	55	65	35	40	90,6%	95,2%	97,3%	97,3%	91,1%
16945	55	65	35	45	90,7%	95,1%	97,4%	97,5%	91,6%
16946	55	65	35	50	90,9%	95,0%	97,5%	97,7%	92,1%
16947	55	65	35	55	91,0%	95,0%	97,6%	97,8%	92,6%
16948	55	65	35	60	91,2%	94,9%	97,7%	97,9%	93,1%
16949	55	65	35	65	91,3%	94,9%	97,7%	98,1%	93,5%
16950	55	65	35	70	91,4%	94,8%	97,8%	98,2%	93,9%
16951	55	65	35	75	91,6%	94,7%	97,9%	98,3%	94,3%
16952	55	65	35	80	91,7%	94,7%	98,0%	98,4%	94,6%
16953	55	65	40	20	87,5%	94,3%	96,6%	96,3%	88,2%
16954	55	65	40	25	87,7%	94,2%	96,7%	96,5%	88,9%
16955	55	65	40	30	87,9%	94,1%	96,8%	96,7%	89,5%
16956	55	65	40	35	88,1%	94,1%	96,9%	97,0%	90,1%
16957	55	65	40	40	88,3%	94,0%	97,0%	97,2%	90,7%
16958	55	65	40	45	88,4%	93,9%	97,1%	97,3%	91,3%
16959	55	65	40	50	88,6%	93,8%	97,2%	97,5%	91,8%
16960	55	65	40	55	88,8%	93,8%	97,3%	97,7%	92,3%
16961	55	65	40	60	89,0%	93,7%	97,4%	97,8%	92,8%

16962	55	65	40	65	89,1%	93,6%	97,4%	98,0%	93,2%
16963	55	65	40	70	89,3%	93,5%	97,5%	98,1%	93,7%
16964	55	65	40	75	89,5%	93,5%	97,6%	98,2%	94,1%
16965	55	65	40	80	89,6%	93,4%	97,7%	98,3%	94,4%
16966	55	65	45	20	84,5%	92,9%	96,1%	96,1%	87,7%
16967	55	65	45	25	84,8%	92,8%	96,3%	96,3%	88,4%
16968	55	65	45	30	85,0%	92,7%	96,4%	96,5%	89,1%
16969	55	65	45	35	85,2%	92,6%	96,5%	96,8%	89,8%
16970	55	65	45	40	85,4%	92,5%	96,6%	97,0%	90,4%
16971	55	65	45	45	85,7%	92,5%	96,7%	97,2%	91,0%
16972	55	65	45	50	85,9%	92,4%	96,8%	97,4%	91,5%
16973	55	65	45	55	86,1%	92,3%	96,9%	97,5%	92,0%
16974	55	65	45	60	86,3%	92,2%	97,0%	97,7%	92,5%
16975	55	65	45	65	86,5%	92,1%	97,1%	97,8%	93,0%
16976	55	65	45	70	86,7%	92,0%	97,2%	98,0%	93,4%
16977	55	65	45	75	86,9%	91,9%	97,3%	98,1%	93,8%
16978	55	65	45	80	87,1%	91,8%	97,4%	98,2%	94,2%
16979	55	65	50	20	81,0%	91,2%	95,6%	95,8%	87,3%
16980	55	65	50	25	81,3%	91,1%	95,8%	96,1%	88,0%
16981	55	65	50	30	81,6%	91,0%	95,9%	96,3%	88,7%
16982	55	65	50	35	81,8%	90,9%	96,0%	96,6%	89,4%
16983	55	65	50	40	82,1%	90,8%	96,2%	96,8%	90,0%
16984	55	65	50	45	82,4%	90,7%	96,3%	97,0%	90,6%
16985	55	65	50	50	82,6%	90,6%	96,4%	97,2%	91,2%
16986	55	65	50	55	82,9%	90,5%	96,5%	97,4%	91,7%
16987	55	65	50	60	83,1%	90,3%	96,6%	97,5%	92,2%
16988	55	65	50	65	83,4%	90,2%	96,8%	97,7%	92,7%
16989	55	65	50	70	83,6%	90,1%	96,9%	97,9%	93,1%
16990	55	65	50	75	83,8%	90,0%	97,0%	98,0%	93,6%
16991	55	65	50	80	84,1%	89,9%	97,1%	98,1%	94,0%
16992	55	65	55	20	77,0%	89,2%	95,1%	95,6%	86,8%
16993	55	65	55	25	77,3%	89,1%	95,3%	95,8%	87,6%
16994	55	65	55	30	77,6%	88,9%	95,4%	96,1%	88,3%

16995	55	65	55	35	77,9%	88,8%	95,5%	96,4%	89,0%
16996	55	65	55	40	78,2%	88,7%	95,7%	96,6%	89,6%
16997	55	65	55	45	78,5%	88,5%	95,8%	96,8%	90,3%
16998	55	65	55	50	78,8%	88,4%	96,0%	97,0%	90,8%
16999	55	65	55	55	79,1%	88,3%	96,1%	97,2%	91,4%
17000	55	65	55	60	79,4%	88,1%	96,2%	97,4%	91,9%
17001	55	65	55	65	79,6%	88,0%	96,3%	97,6%	92,4%
17002	55	65	55	70	79,9%	87,9%	96,5%	97,7%	92,9%
17003	55	65	55	75	80,2%	87,7%	96,6%	97,9%	93,3%
17004	55	65	55	80	80,5%	87,6%	96,7%	98,0%	93,7%
17005	55	65	60	20	72,3%	86,7%	94,5%	95,3%	86,3%
17006	55	65	60	25	72,6%	86,6%	94,7%	95,6%	87,1%
17007	55	65	60	30	73,0%	86,4%	94,8%	95,9%	87,9%
17008	55	65	60	35	73,3%	86,3%	95,0%	96,1%	88,6%
17009	55	65	60	40	73,7%	86,1%	95,1%	96,4%	89,2%
17010	55	65	60	45	74,0%	86,0%	95,3%	96,6%	89,9%
17011	55	65	60	50	74,4%	85,8%	95,5%	96,8%	90,5%
17012	55	65	60	55	74,7%	85,7%	95,6%	97,0%	91,1%
17013	55	65	60	60	75,0%	85,5%	95,7%	97,2%	91,6%
17014	55	65	60	65	75,4%	85,3%	95,9%	97,4%	92,1%
17015	55	65	60	70	75,7%	85,2%	96,0%	97,6%	92,6%
17016	55	65	60	75	76,0%	85,0%	96,1%	97,7%	93,1%
17017	55	65	60	80	76,3%	84,8%	96,3%	97,9%	93,5%
17018	55	65	65	20	67,1%	83,9%	93,8%	95,0%	85,8%
17019	55	65	65	25	67,5%	83,7%	94,0%	95,3%	86,6%
17020	55	65	65	30	67,9%	83,5%	94,2%	95,6%	87,4%
17021	55	65	65	35	68,2%	83,3%	94,4%	95,9%	88,1%
17022	55	65	65	40	68,6%	83,1%	94,5%	96,2%	88,8%
17023	55	65	65	45	69,0%	83,0%	94,7%	96,4%	89,5%
17024	55	65	65	50	69,4%	82,8%	94,9%	96,6%	90,1%
17025	55	65	65	55	69,7%	82,6%	95,0%	96,9%	90,7%
17026	55	65	65	60	70,1%	82,4%	95,2%	97,1%	91,3%
17027	55	65	65	65	70,5%	82,2%	95,4%	97,3%	91,8%

17028	55	65	65	70	70,9%	82,0%	95,5%	97,4%	92,3%
17029	55	65	65	75	71,2%	81,8%	95,6%	97,6%	92,8%
17030	55	65	65	80	71,6%	81,6%	95,8%	97,8%	93,2%
17031	55	65	70	20	61,4%	80,5%	93,0%	94,7%	85,3%
17032	55	65	70	25	61,8%	80,3%	93,2%	95,1%	86,2%
17033	55	65	70	30	62,3%	80,1%	93,5%	95,4%	87,0%
17034	55	65	70	35	62,7%	79,9%	93,7%	95,7%	87,7%
17035	55	65	70	40	63,1%	79,6%	93,9%	95,9%	88,4%
17036	55	65	70	45	63,5%	79,4%	94,1%	96,2%	89,1%
17037	55	65	70	50	63,9%	79,2%	94,2%	96,4%	89,8%
17038	55	65	70	55	64,3%	79,0%	94,4%	96,7%	90,4%
17039	55	65	70	60	64,7%	78,8%	94,6%	96,9%	91,0%
17040	55	65	70	65	65,1%	78,6%	94,8%	97,1%	91,5%
17041	55	65	70	70	65,5%	78,3%	94,9%	97,3%	92,0%
17042	55	65	70	75	65,9%	78,1%	95,1%	97,5%	92,5%
17043	55	65	70	80	66,3%	77,9%	95,3%	97,6%	93,0%
17044	55	65	75	20	55,4%	76,6%	92,2%	94,4%	84,8%
17045	55	65	75	25	55,9%	76,4%	92,4%	94,8%	85,7%
17046	55	65	75	30	56,3%	76,1%	92,7%	95,1%	86,5%
17047	55	65	75	35	56,7%	75,9%	92,9%	95,4%	87,3%
17048	55	65	75	40	57,2%	75,6%	93,1%	95,7%	88,0%
17049	55	65	75	45	57,6%	75,4%	93,3%	96,0%	88,7%
17050	55	65	75	50	58,0%	75,2%	93,5%	96,2%	89,4%
17051	55	65	75	55	58,5%	74,9%	93,7%	96,5%	90,0%
17052	55	65	75	60	58,9%	74,7%	93,9%	96,7%	90,6%
17053	55	65	75	65	59,3%	74,4%	94,1%	96,9%	91,2%
17054	55	65	75	70	59,7%	74,2%	94,3%	97,1%	91,7%
17055	55	65	75	75	60,2%	73,9%	94,5%	97,3%	92,2%
17056	55	65	75	80	60,6%	73,7%	94,7%	97,5%	92,7%
17057	55	65	80	20	49,3%	72,2%	91,2%	94,1%	84,3%
17058	55	65	80	25	49,7%	71,9%	91,5%	94,5%	85,1%
17059	55	65	80	30	50,2%	71,7%	91,8%	94,8%	86,0%
17060	55	65	80	35	50,6%	71,4%	92,0%	95,1%	86,8%

17061	55	65	80	40	51,0%	71,1%	92,3%	95,4%	87,6%
17062	55	65	80	45	51,5%	70,9%	92,5%	95,7%	88,3%
17063	55	65	80	50	51,9%	70,6%	92,7%	96,0%	89,0%
17064	55	65	80	55	52,4%	70,3%	93,0%	96,3%	89,6%
17065	55	65	80	60	52,8%	70,1%	93,2%	96,5%	90,3%
17066	55	65	80	65	53,2%	69,8%	93,4%	96,7%	90,8%
17067	55	65	80	70	53,7%	69,5%	93,6%	96,9%	91,4%
17068	55	65	80	75	54,1%	69,2%	93,8%	97,1%	91,9%
17069	55	65	80	80	54,6%	68,9%	94,0%	97,3%	92,4%
17070	55	70	20	20	95,4%	97,8%	97,8%	96,8%	88,9%
17071	55	70	20	25	95,4%	97,8%	97,9%	97,0%	89,5%
17072	55	70	20	30	95,5%	97,8%	98,0%	97,2%	90,1%
17073	55	70	20	35	95,6%	97,7%	98,0%	97,4%	90,7%
17074	55	70	20	40	95,7%	97,7%	98,1%	97,6%	91,3%
17075	55	70	20	45	95,7%	97,7%	98,2%	97,7%	91,8%
17076	55	70	20	50	95,8%	97,6%	98,2%	97,9%	92,3%
17077	55	70	20	55	95,9%	97,6%	98,3%	98,0%	92,8%
17078	55	70	20	60	95,9%	97,6%	98,3%	98,2%	93,2%
17079	55	70	20	65	96,0%	97,6%	98,4%	98,3%	93,7%
17080	55	70	20	70	96,1%	97,5%	98,4%	98,4%	94,1%
17081	55	70	20	75	96,1%	97,5%	98,5%	98,5%	94,4%
17082	55	70	20	80	96,2%	97,5%	98,5%	98,6%	94,8%
17083	55	70	25	20	94,1%	97,3%	97,5%	96,6%	88,4%
17084	55	70	25	25	94,2%	97,2%	97,6%	96,9%	89,1%
17085	55	70	25	30	94,3%	97,2%	97,7%	97,1%	89,8%
17086	55	70	25	35	94,4%	97,2%	97,8%	97,3%	90,4%
17087	55	70	25	40	94,5%	97,1%	97,8%	97,4%	91,0%
17088	55	70	25	45	94,6%	97,1%	97,9%	97,6%	91,5%
17089	55	70	25	50	94,7%	97,1%	98,0%	97,8%	92,0%
17090	55	70	25	55	94,8%	97,0%	98,0%	97,9%	92,5%
17091	55	70	25	60	94,9%	97,0%	98,1%	98,0%	93,0%
17092	55	70	25	65	95,0%	96,9%	98,2%	98,2%	93,4%
17093	55	70	25	70	95,0%	96,9%	98,2%	98,3%	93,8%

17094	55	70	25	75	95,1%	96,9%	98,3%	98,4%	94,2%
17095	55	70	25	80	95,2%	96,8%	98,3%	98,5%	94,6%
17096	55	70	30	20	92,6%	96,6%	97,2%	96,4%	88,0%
17097	55	70	30	25	92,7%	96,5%	97,3%	96,7%	88,7%
17098	55	70	30	30	92,9%	96,5%	97,4%	96,9%	89,4%
17099	55	70	30	35	93,0%	96,5%	97,5%	97,1%	90,0%
17100	55	70	30	40	93,1%	96,4%	97,6%	97,3%	90,6%
17101	55	70	30	45	93,2%	96,4%	97,6%	97,5%	91,2%
17102	55	70	30	50	93,3%	96,3%	97,7%	97,6%	91,7%
17103	55	70	30	55	93,4%	96,3%	97,8%	97,8%	92,2%
17104	55	70	30	60	93,5%	96,2%	97,9%	97,9%	92,7%
17105	55	70	30	65	93,6%	96,2%	97,9%	98,1%	93,2%
17106	55	70	30	70	93,7%	96,1%	98,0%	98,2%	93,6%
17107	55	70	30	75	93,8%	96,1%	98,1%	98,3%	94,0%
17108	55	70	30	80	93,9%	96,0%	98,1%	98,4%	94,4%
17109	55	70	35	20	90,7%	95,7%	96,9%	96,2%	87,6%
17110	55	70	35	25	90,9%	95,7%	97,0%	96,5%	88,3%
17111	55	70	35	30	91,0%	95,6%	97,1%	96,7%	89,0%
17112	55	70	35	35	91,2%	95,6%	97,2%	96,9%	89,6%
17113	55	70	35	40	91,3%	95,5%	97,2%	97,1%	90,3%
17114	55	70	35	45	91,5%	95,5%	97,3%	97,3%	90,9%
17115	55	70	35	50	91,6%	95,4%	97,4%	97,5%	91,4%
17116	55	70	35	55	91,7%	95,3%	97,5%	97,6%	91,9%
17117	55	70	35	60	91,9%	95,3%	97,6%	97,8%	92,4%
17118	55	70	35	65	92,0%	95,2%	97,7%	97,9%	92,9%
17119	55	70	35	70	92,1%	95,2%	97,7%	98,1%	93,3%
17120	55	70	35	75	92,2%	95,1%	97,8%	98,2%	93,7%
17121	55	70	35	80	92,4%	95,0%	97,9%	98,3%	94,1%
17122	55	70	40	20	88,4%	94,7%	96,5%	96,0%	87,1%
17123	55	70	40	25	88,6%	94,6%	96,6%	96,3%	87,9%
17124	55	70	40	30	88,8%	94,6%	96,7%	96,5%	88,6%
17125	55	70	40	35	89,0%	94,5%	96,8%	96,7%	89,3%
17126	55	70	40	40	89,1%	94,4%	96,9%	96,9%	89,9%

17127	55	70	40	45	89,3%	94,4%	97,0%	97,1%	90,5%
17128	55	70	40	50	89,5%	94,3%	97,1%	97,3%	91,1%
17129	55	70	40	55	89,6%	94,2%	97,2%	97,5%	91,6%
17130	55	70	40	60	89,8%	94,1%	97,3%	97,7%	92,1%
17131	55	70	40	65	90,0%	94,1%	97,4%	97,8%	92,6%
17132	55	70	40	70	90,1%	94,0%	97,5%	98,0%	93,1%
17133	55	70	40	75	90,3%	93,9%	97,5%	98,1%	93,5%
17134	55	70	40	80	90,4%	93,8%	97,6%	98,2%	93,9%
17135	55	70	45	20	85,7%	93,4%	96,0%	95,8%	86,7%
17136	55	70	45	25	85,9%	93,3%	96,1%	96,0%	87,4%
17137	55	70	45	30	86,1%	93,2%	96,3%	96,3%	88,2%
17138	55	70	45	35	86,3%	93,2%	96,4%	96,5%	88,9%
17139	55	70	45	40	86,5%	93,1%	96,5%	96,8%	89,5%
17140	55	70	45	45	86,7%	93,0%	96,6%	97,0%	90,1%
17141	55	70	45	50	86,9%	92,9%	96,7%	97,2%	90,7%
17142	55	70	45	55	87,1%	92,8%	96,8%	97,3%	91,3%
17143	55	70	45	60	87,3%	92,7%	96,9%	97,5%	91,8%
17144	55	70	45	65	87,5%	92,6%	97,0%	97,7%	92,3%
17145	55	70	45	70	87,7%	92,5%	97,1%	97,8%	92,8%
17146	55	70	45	75	87,9%	92,5%	97,2%	98,0%	93,2%
17147	55	70	45	80	88,1%	92,4%	97,3%	98,1%	93,7%
17148	55	70	50	20	82,4%	91,8%	95,5%	95,5%	86,2%
17149	55	70	50	25	82,6%	91,7%	95,7%	95,8%	87,0%
17150	55	70	50	30	82,9%	91,6%	95,8%	96,1%	87,7%
17151	55	70	50	35	83,1%	91,5%	95,9%	96,3%	88,4%
17152	55	70	50	40	83,4%	91,4%	96,1%	96,6%	89,1%
17153	55	70	50	45	83,6%	91,3%	96,2%	96,8%	89,8%
17154	55	70	50	50	83,8%	91,2%	96,3%	97,0%	90,4%
17155	55	70	50	55	84,1%	91,1%	96,4%	97,2%	91,0%
17156	55	70	50	60	84,3%	91,0%	96,5%	97,4%	91,5%
17157	55	70	50	65	84,6%	90,9%	96,7%	97,5%	92,0%
17158	55	70	50	70	84,8%	90,8%	96,8%	97,7%	92,5%
17159	55	70	50	75	85,0%	90,7%	96,9%	97,8%	93,0%

17160	55	70	50	80	85,2%	90,6%	97,0%	98,0%	93,4%
17161	55	70	55	20	78,5%	89,9%	94,9%	95,2%	85,7%
17162	55	70	55	25	78,8%	89,8%	95,1%	95,5%	86,5%
17163	55	70	55	30	79,1%	89,7%	95,3%	95,8%	87,3%
17164	55	70	55	35	79,4%	89,6%	95,4%	96,1%	88,0%
17165	55	70	55	40	79,7%	89,4%	95,6%	96,3%	88,7%
17166	55	70	55	45	79,9%	89,3%	95,7%	96,6%	89,4%
17167	55	70	55	50	80,2%	89,2%	95,8%	96,8%	90,0%
17168	55	70	55	55	80,5%	89,1%	96,0%	97,0%	90,6%
17169	55	70	55	60	80,8%	88,9%	96,1%	97,2%	91,2%
17170	55	70	55	65	81,0%	88,8%	96,2%	97,4%	91,7%
17171	55	70	55	70	81,3%	88,7%	96,3%	97,6%	92,2%
17172	55	70	55	75	81,6%	88,5%	96,5%	97,7%	92,7%
17173	55	70	55	80	81,8%	88,4%	96,6%	97,9%	93,2%
17174	55	70	60	20	74,0%	87,6%	94,3%	95,0%	85,2%
17175	55	70	60	25	74,4%	87,5%	94,5%	95,3%	86,0%
17176	55	70	60	30	74,7%	87,3%	94,7%	95,6%	86,8%
17177	55	70	60	35	75,0%	87,2%	94,8%	95,9%	87,6%
17178	55	70	60	40	75,4%	87,0%	95,0%	96,1%	88,3%
17179	55	70	60	45	75,7%	86,9%	95,2%	96,4%	89,0%
17180	55	70	60	50	76,0%	86,7%	95,3%	96,6%	89,6%
17181	55	70	60	55	76,3%	86,6%	95,5%	96,8%	90,3%
17182	55	70	60	60	76,6%	86,4%	95,6%	97,0%	90,8%
17183	55	70	60	65	77,0%	86,3%	95,7%	97,2%	91,4%
17184	55	70	60	70	77,3%	86,1%	95,9%	97,4%	91,9%
17185	55	70	60	75	77,6%	86,0%	96,0%	97,6%	92,4%
17186	55	70	60	80	77,9%	85,8%	96,1%	97,7%	92,9%
17187	55	70	65	20	69,0%	84,9%	93,6%	94,7%	84,6%
17188	55	70	65	25	69,4%	84,7%	93,8%	95,0%	85,5%
17189	55	70	65	30	69,8%	84,6%	94,0%	95,3%	86,3%
17190	55	70	65	35	70,1%	84,4%	94,2%	95,6%	87,1%
17191	55	70	65	40	70,5%	84,2%	94,4%	95,9%	87,9%
17192	55	70	65	45	70,9%	84,0%	94,6%	96,2%	88,6%

17193	55	70	65	50	71,2%	83,9%	94,7%	96,4%	89,3%
17194	55	70	65	55	71,6%	83,7%	94,9%	96,6%	89,9%
17195	55	70	65	60	71,9%	83,5%	95,1%	96,9%	90,5%
17196	55	70	65	65	72,3%	83,3%	95,2%	97,1%	91,1%
17197	55	70	65	70	72,6%	83,1%	95,4%	97,2%	91,6%
17198	55	70	65	75	73,0%	83,0%	95,5%	97,4%	92,1%
17199	55	70	65	80	73,3%	82,8%	95,7%	97,6%	92,6%
17200	55	70	70	20	63,5%	81,7%	92,8%	94,3%	84,1%
17201	55	70	70	25	63,9%	81,5%	93,0%	94,7%	85,0%
17202	55	70	70	30	64,3%	81,3%	93,3%	95,0%	85,8%
17203	55	70	70	35	64,7%	81,1%	93,5%	95,3%	86,6%
17204	55	70	70	40	65,1%	80,9%	93,7%	95,6%	87,4%
17205	55	70	70	45	65,5%	80,7%	93,9%	95,9%	88,2%
17206	55	70	70	50	65,9%	80,5%	94,1%	96,2%	88,8%
17207	55	70	70	55	66,3%	80,3%	94,3%	96,4%	89,5%
17208	55	70	70	60	66,7%	80,1%	94,4%	96,7%	90,1%
17209	55	70	70	65	67,1%	79,9%	94,6%	96,9%	90,7%
17210	55	70	70	70	67,5%	79,7%	94,8%	97,1%	91,3%
17211	55	70	70	75	67,9%	79,4%	94,9%	97,3%	91,8%
17212	55	70	70	80	68,2%	79,2%	95,1%	97,4%	92,3%
17213	55	70	75	20	57,6%	78,0%	92,0%	94,0%	83,5%
17214	55	70	75	25	58,0%	77,8%	92,2%	94,4%	84,4%
17215	55	70	75	30	58,5%	77,5%	92,4%	94,7%	85,3%
17216	55	70	75	35	58,9%	77,3%	92,7%	95,1%	86,2%
17217	55	70	75	40	59,3%	77,1%	92,9%	95,4%	87,0%
17218	55	70	75	45	59,7%	76,8%	93,1%	95,7%	87,7%
17219	55	70	75	50	60,2%	76,6%	93,3%	96,0%	88,4%
17220	55	70	75	55	60,6%	76,4%	93,5%	96,2%	89,1%
17221	55	70	75	60	61,0%	76,1%	93,7%	96,5%	89,8%
17222	55	70	75	65	61,4%	75,9%	93,9%	96,7%	90,4%
17223	55	70	75	70	61,8%	75,7%	94,1%	96,9%	91,0%
17224	55	70	75	75	62,3%	75,4%	94,3%	97,1%	91,5%
17225	55	70	75	80	62,7%	75,2%	94,5%	97,3%	92,0%

17226	55	70	80	20	51,5%	73,8%	91,0%	93,7%	82,9%
17227	55	70	80	25	51,9%	73,5%	91,3%	94,0%	83,9%
17228	55	70	80	30	52,4%	73,3%	91,5%	94,4%	84,8%
17229	55	70	80	35	52,8%	73,0%	91,8%	94,8%	85,7%
17230	55	70	80	40	53,3%	72,7%	92,0%	95,1%	86,5%
17231	55	70	80	45	53,7%	72,5%	92,3%	95,4%	87,3%
17232	55	70	80	50	54,1%	72,2%	92,5%	95,7%	88,0%
17233	55	70	80	55	54,6%	71,9%	92,8%	96,0%	88,7%
17234	55	70	80	60	55,0%	71,7%	93,0%	96,2%	89,4%
17235	55	70	80	65	55,4%	71,4%	93,2%	96,5%	90,0%
17236	55	70	80	70	55,9%	71,2%	93,4%	96,7%	90,6%
17237	55	70	80	75	56,3%	70,9%	93,6%	96,9%	91,2%
17238	55	70	80	80	56,7%	70,6%	93,8%	97,1%	91,7%
17239	55	75	20	20	95,7%	98,0%	97,7%	96,6%	87,9%
17240	55	75	20	25	95,8%	98,0%	97,8%	96,8%	88,6%
17241	55	75	20	30	95,9%	97,9%	97,9%	97,0%	89,3%
17242	55	75	20	35	95,9%	97,9%	98,0%	97,2%	89,9%
17243	55	75	20	40	96,0%	97,9%	98,0%	97,4%	90,5%
17244	55	75	20	45	96,1%	97,9%	98,1%	97,6%	91,1%
17245	55	75	20	50	96,1%	97,8%	98,2%	97,7%	91,6%
17246	55	75	20	55	96,2%	97,8%	98,2%	97,9%	92,1%
17247	55	75	20	60	96,3%	97,8%	98,3%	98,0%	92,6%
17248	55	75	20	65	96,3%	97,7%	98,3%	98,1%	93,1%
17249	55	75	20	70	96,4%	97,7%	98,4%	98,3%	93,5%
17250	55	75	20	75	96,5%	97,7%	98,4%	98,4%	93,9%
17251	55	75	20	80	96,5%	97,6%	98,5%	98,5%	94,3%
17252	55	75	25	20	94,6%	97,5%	97,5%	96,4%	87,4%
17253	55	75	25	25	94,7%	97,4%	97,5%	96,6%	88,2%
17254	55	75	25	30	94,8%	97,4%	97,6%	96,8%	88,9%
17255	55	75	25	35	94,9%	97,4%	97,7%	97,1%	89,5%
17256	55	75	25	40	95,0%	97,3%	97,8%	97,2%	90,2%
17257	55	75	25	45	95,0%	97,3%	97,8%	97,4%	90,7%
17258	55	75	25	50	95,1%	97,3%	97,9%	97,6%	91,3%

17259	55	75	25	55	95,2%	97,2%	98,0%	97,7%	91,8%
17260	55	75	25	60	95,3%	97,2%	98,1%	97,9%	92,3%
17261	55	75	25	65	95,4%	97,2%	98,1%	98,0%	92,8%
17262	55	75	25	70	95,4%	97,1%	98,2%	98,2%	93,2%
17263	55	75	25	75	95,5%	97,1%	98,2%	98,3%	93,7%
17264	55	75	25	80	95,6%	97,1%	98,3%	98,4%	94,1%
17265	55	75	30	20	93,2%	96,8%	97,1%	96,2%	87,0%
17266	55	75	30	25	93,3%	96,8%	97,2%	96,4%	87,7%
17267	55	75	30	30	93,4%	96,8%	97,3%	96,7%	88,5%
17268	55	75	30	35	93,5%	96,7%	97,4%	96,9%	89,1%
17269	55	75	30	40	93,6%	96,7%	97,5%	97,1%	89,8%
17270	55	75	30	45	93,7%	96,6%	97,6%	97,3%	90,4%
17271	55	75	30	50	93,8%	96,6%	97,6%	97,4%	91,0%
17272	55	75	30	55	93,9%	96,5%	97,7%	97,6%	91,5%
17273	55	75	30	60	94,0%	96,5%	97,8%	97,8%	92,0%
17274	55	75	30	65	94,1%	96,5%	97,9%	97,9%	92,5%
17275	55	75	30	70	94,2%	96,4%	97,9%	98,0%	93,0%
17276	55	75	30	75	94,3%	96,4%	98,0%	98,2%	93,4%
17277	55	75	30	80	94,4%	96,3%	98,1%	98,3%	93,8%
17278	55	75	35	20	91,5%	96,0%	96,8%	96,0%	86,5%
17279	55	75	35	25	91,6%	96,0%	96,9%	96,2%	87,3%
17280	55	75	35	30	91,7%	95,9%	97,0%	96,5%	88,0%
17281	55	75	35	35	91,9%	95,9%	97,1%	96,7%	88,7%
17282	55	75	35	40	92,0%	95,8%	97,2%	96,9%	89,4%
17283	55	75	35	45	92,1%	95,8%	97,3%	97,1%	90,0%
17284	55	75	35	50	92,2%	95,7%	97,3%	97,3%	90,6%
17285	55	75	35	55	92,4%	95,7%	97,4%	97,5%	91,2%
17286	55	75	35	60	92,5%	95,6%	97,5%	97,6%	91,7%
17287	55	75	35	65	92,6%	95,6%	97,6%	97,8%	92,2%
17288	55	75	35	70	92,7%	95,5%	97,7%	97,9%	92,7%
17289	55	75	35	75	92,9%	95,5%	97,7%	98,1%	93,2%
17290	55	75	35	80	93,0%	95,4%	97,8%	98,2%	93,6%
17291	55	75	40	20	89,3%	95,1%	96,4%	95,7%	86,0%

17292	55	75	40	25	89,5%	95,0%	96,5%	96,0%	86,8%
17293	55	75	40	30	89,7%	94,9%	96,6%	96,2%	87,6%
17294	55	75	40	35	89,8%	94,9%	96,7%	96,5%	88,3%
17295	55	75	40	40	90,0%	94,8%	96,8%	96,7%	89,0%
17296	55	75	40	45	90,1%	94,8%	96,9%	96,9%	89,6%
17297	55	75	40	50	90,3%	94,7%	97,0%	97,1%	90,3%
17298	55	75	40	55	90,4%	94,6%	97,1%	97,3%	90,9%
17299	55	75	40	60	90,6%	94,6%	97,2%	97,5%	91,4%
17300	55	75	40	65	90,7%	94,5%	97,3%	97,6%	91,9%
17301	55	75	40	70	90,9%	94,4%	97,4%	97,8%	92,4%
17302	55	75	40	75	91,0%	94,4%	97,5%	97,9%	92,9%
17303	55	75	40	80	91,2%	94,3%	97,5%	98,1%	93,3%
17304	55	75	45	20	86,7%	93,9%	95,9%	95,5%	85,5%
17305	55	75	45	25	86,9%	93,8%	96,0%	95,7%	86,3%
17306	55	75	45	30	87,1%	93,7%	96,1%	96,0%	87,1%
17307	55	75	45	35	87,3%	93,6%	96,3%	96,3%	87,9%
17308	55	75	45	40	87,5%	93,6%	96,4%	96,5%	88,6%
17309	55	75	45	45	87,7%	93,5%	96,5%	96,7%	89,3%
17310	55	75	45	50	87,9%	93,4%	96,6%	96,9%	89,9%
17311	55	75	45	55	88,1%	93,3%	96,7%	97,1%	90,5%
17312	55	75	45	60	88,3%	93,2%	96,8%	97,3%	91,1%
17313	55	75	45	65	88,4%	93,2%	96,9%	97,5%	91,6%
17314	55	75	45	70	88,6%	93,1%	97,0%	97,7%	92,1%
17315	55	75	45	75	88,8%	93,0%	97,1%	97,8%	92,6%
17316	55	75	45	80	89,0%	92,9%	97,2%	98,0%	93,1%
17317	55	75	50	20	83,6%	92,4%	95,4%	95,2%	85,0%
17318	55	75	50	25	83,9%	92,3%	95,5%	95,5%	85,8%
17319	55	75	50	30	84,1%	92,2%	95,7%	95,8%	86,7%
17320	55	75	50	35	84,3%	92,1%	95,8%	96,0%	87,4%
17321	55	75	50	40	84,6%	92,0%	95,9%	96,3%	88,2%
17322	55	75	50	45	84,8%	91,9%	96,1%	96,5%	88,9%
17323	55	75	50	50	85,0%	91,8%	96,2%	96,8%	89,5%
17324	55	75	50	55	85,2%	91,7%	96,3%	97,0%	90,1%

17325	55	75	50	60	85,5%	91,6%	96,4%	97,2%	90,7%
17326	55	75	50	65	85,7%	91,5%	96,5%	97,3%	91,3%
17327	55	75	50	70	85,9%	91,4%	96,7%	97,5%	91,8%
17328	55	75	50	75	86,1%	91,3%	96,8%	97,7%	92,3%
17329	55	75	50	80	86,3%	91,2%	96,9%	97,8%	92,8%
17330	55	75	55	20	79,9%	90,6%	94,8%	94,9%	84,5%
17331	55	75	55	25	80,2%	90,5%	95,0%	95,2%	85,3%
17332	55	75	55	30	80,5%	90,4%	95,1%	95,5%	86,2%
17333	55	75	55	35	80,8%	90,3%	95,3%	95,8%	87,0%
17334	55	75	55	40	81,1%	90,2%	95,4%	96,1%	87,7%
17335	55	75	55	45	81,3%	90,0%	95,6%	96,3%	88,4%
17336	55	75	55	50	81,6%	89,9%	95,7%	96,6%	89,1%
17337	55	75	55	55	81,9%	89,8%	95,8%	96,8%	89,8%
17338	55	75	55	60	82,1%	89,7%	96,0%	97,0%	90,4%
17339	55	75	55	65	82,4%	89,6%	96,1%	97,2%	91,0%
17340	55	75	55	70	82,6%	89,4%	96,2%	97,4%	91,5%
17341	55	75	55	75	82,9%	89,3%	96,4%	97,5%	92,0%
17342	55	75	55	80	83,1%	89,2%	96,5%	97,7%	92,5%
17343	55	75	60	20	75,7%	88,5%	94,1%	94,6%	83,9%
17344	55	75	60	25	76,0%	88,3%	94,3%	94,9%	84,8%
17345	55	75	60	30	76,3%	88,2%	94,5%	95,3%	85,7%
17346	55	75	60	35	76,7%	88,0%	94,7%	95,6%	86,5%
17347	55	75	60	40	77,0%	87,9%	94,8%	95,8%	87,3%
17348	55	75	60	45	77,3%	87,8%	95,0%	96,1%	88,0%
17349	55	75	60	50	77,6%	87,6%	95,2%	96,4%	88,7%
17350	55	75	60	55	77,9%	87,5%	95,3%	96,6%	89,4%
17351	55	75	60	60	78,2%	87,3%	95,5%	96,8%	90,0%
17352	55	75	60	65	78,5%	87,2%	95,6%	97,0%	90,6%
17353	55	75	60	70	78,8%	87,0%	95,8%	97,2%	91,2%
17354	55	75	60	75	79,1%	86,9%	95,9%	97,4%	91,7%
17355	55	75	60	80	79,4%	86,8%	96,0%	97,6%	92,2%
17356	55	75	65	20	70,9%	85,9%	93,4%	94,3%	83,3%
17357	55	75	65	25	71,2%	85,7%	93,6%	94,6%	84,3%

17358	55	75	65	30	71,6%	85,6%	93,8%	95,0%	85,2%
17359	55	75	65	35	72,0%	85,4%	94,0%	95,3%	86,0%
17360	55	75	65	40	72,3%	85,2%	94,2%	95,6%	86,8%
17361	55	75	65	45	72,7%	85,1%	94,4%	95,9%	87,6%
17362	55	75	65	50	73,0%	84,9%	94,6%	96,1%	88,3%
17363	55	75	65	55	73,4%	84,7%	94,7%	96,4%	89,0%
17364	55	75	65	60	73,7%	84,6%	94,9%	96,6%	89,6%
17365	55	75	65	65	74,0%	84,4%	95,1%	96,8%	90,3%
17366	55	75	65	70	74,4%	84,2%	95,2%	97,0%	90,8%
17367	55	75	65	75	74,7%	84,0%	95,4%	97,2%	91,4%
17368	55	75	65	80	75,0%	83,9%	95,5%	97,4%	91,9%
17369	55	75	70	20	65,5%	82,8%	92,6%	93,9%	82,8%
17370	55	75	70	25	65,9%	82,7%	92,8%	94,3%	83,7%
17371	55	75	70	30	66,3%	82,5%	93,1%	94,7%	84,6%
17372	55	75	70	35	66,7%	82,3%	93,3%	95,0%	85,5%
17373	55	75	70	40	67,1%	82,1%	93,5%	95,3%	86,3%
17374	55	75	70	45	67,5%	81,9%	93,7%	95,6%	87,1%
17375	55	75	70	50	67,9%	81,7%	93,9%	95,9%	87,9%
17376	55	75	70	55	68,3%	81,5%	94,1%	96,2%	88,6%
17377	55	75	70	60	68,6%	81,3%	94,3%	96,4%	89,3%
17378	55	75	70	65	69,0%	81,1%	94,4%	96,6%	89,9%
17379	55	75	70	70	69,4%	80,9%	94,6%	96,9%	90,5%
17380	55	75	70	75	69,8%	80,7%	94,8%	97,1%	91,1%
17381	55	75	70	80	70,1%	80,5%	95,0%	97,3%	91,6%
17382	55	75	75	20	59,8%	79,3%	91,7%	93,6%	82,2%
17383	55	75	75	25	60,2%	79,1%	92,0%	94,0%	83,1%
17384	55	75	75	30	60,6%	78,9%	92,2%	94,4%	84,1%
17385	55	75	75	35	61,0%	78,7%	92,5%	94,7%	85,0%
17386	55	75	75	40	61,4%	78,4%	92,7%	95,0%	85,8%
17387	55	75	75	45	61,9%	78,2%	92,9%	95,4%	86,6%
17388	55	75	75	50	62,3%	78,0%	93,1%	95,7%	87,4%
17389	55	75	75	55	62,7%	77,8%	93,3%	95,9%	88,2%
17390	55	75	75	60	63,1%	77,5%	93,6%	96,2%	88,9%

17391	55	75	75	65	63,5%	77,3%	93,8%	96,4%	89,5%
17392	55	75	75	70	63,9%	77,1%	94,0%	96,7%	90,1%
17393	55	75	75	75	64,3%	76,8%	94,1%	96,9%	90,7%
17394	55	75	75	80	64,7%	76,6%	94,3%	97,1%	91,3%
17395	55	75	80	20	53,7%	75,3%	90,7%	93,2%	81,6%
17396	55	75	80	25	54,1%	75,0%	91,0%	93,6%	82,6%
17397	55	75	80	30	54,6%	74,8%	91,3%	94,0%	83,5%
17398	55	75	80	35	55,0%	74,5%	91,5%	94,4%	84,4%
17399	55	75	80	40	55,5%	74,3%	91,8%	94,8%	85,3%
17400	55	75	80	45	55,9%	74,0%	92,1%	95,1%	86,2%
17401	55	75	80	50	56,3%	73,8%	92,3%	95,4%	87,0%
17402	55	75	80	55	56,8%	73,5%	92,5%	95,7%	87,7%
17403	55	75	80	60	57,2%	73,3%	92,8%	96,0%	88,4%
17404	55	75	80	65	57,6%	73,0%	93,0%	96,2%	89,1%
17405	55	75	80	70	58,1%	72,7%	93,2%	96,5%	89,8%
17406	55	75	80	75	58,5%	72,5%	93,4%	96,7%	90,4%
17407	55	75	80	80	58,9%	72,2%	93,6%	96,9%	91,0%
17408	55	80	20	20	96,1%	98,1%	97,7%	96,4%	86,8%
17409	55	80	20	25	96,1%	98,1%	97,8%	96,6%	87,6%
17410	55	80	20	30	96,2%	98,1%	97,8%	96,8%	88,3%
17411	55	80	20	35	96,3%	98,1%	97,9%	97,0%	89,0%
17412	55	80	20	40	96,3%	98,0%	98,0%	97,2%	89,7%
17413	55	80	20	45	96,4%	98,0%	98,0%	97,4%	90,3%
17414	55	80	20	50	96,5%	98,0%	98,1%	97,6%	90,9%
17415	55	80	20	55	96,5%	98,0%	98,2%	97,7%	91,4%
17416	55	80	20	60	96,6%	97,9%	98,2%	97,9%	91,9%
17417	55	80	20	65	96,6%	97,9%	98,3%	98,0%	92,4%
17418	55	80	20	70	96,7%	97,9%	98,3%	98,1%	92,9%
17419	55	80	20	75	96,8%	97,9%	98,4%	98,3%	93,3%
17420	55	80	20	80	96,8%	97,8%	98,4%	98,4%	93,7%
17421	55	80	25	20	95,0%	97,7%	97,4%	96,1%	86,3%
17422	55	80	25	25	95,1%	97,6%	97,5%	96,4%	87,1%
17423	55	80	25	30	95,2%	97,6%	97,5%	96,6%	87,9%

17424	55	80	25	35	95,3%	97,6%	97,6%	96,8%	88,6%
17425	55	80	25	40	95,4%	97,5%	97,7%	97,0%	89,3%
17426	55	80	25	45	95,4%	97,5%	97,8%	97,2%	89,9%
17427	55	80	25	50	95,5%	97,5%	97,9%	97,4%	90,5%
17428	55	80	25	55	95,6%	97,4%	97,9%	97,6%	91,1%
17429	55	80	25	60	95,7%	97,4%	98,0%	97,7%	91,6%
17430	55	80	25	65	95,7%	97,4%	98,1%	97,9%	92,1%
17431	55	80	25	70	95,8%	97,3%	98,1%	98,0%	92,6%
17432	55	80	25	75	95,9%	97,3%	98,2%	98,2%	93,1%
17433	55	80	25	80	95,9%	97,3%	98,2%	98,3%	93,5%
17434	55	80	30	20	93,7%	97,1%	97,0%	95,9%	85,9%
17435	55	80	30	25	93,8%	97,0%	97,1%	96,2%	86,7%
17436	55	80	30	30	93,9%	97,0%	97,2%	96,4%	87,4%
17437	55	80	30	35	94,0%	97,0%	97,3%	96,6%	88,2%
17438	55	80	30	40	94,1%	96,9%	97,4%	96,9%	88,9%
17439	55	80	30	45	94,2%	96,9%	97,5%	97,1%	89,5%
17440	55	80	30	50	94,3%	96,8%	97,6%	97,3%	90,2%
17441	55	80	30	55	94,4%	96,8%	97,7%	97,4%	90,7%
17442	55	80	30	60	94,5%	96,8%	97,7%	97,6%	91,3%
17443	55	80	30	65	94,6%	96,7%	97,8%	97,8%	91,8%
17444	55	80	30	70	94,7%	96,7%	97,9%	97,9%	92,3%
17445	55	80	30	75	94,8%	96,6%	97,9%	98,0%	92,8%
17446	55	80	30	80	94,9%	96,6%	98,0%	98,2%	93,2%
17447	55	80	35	20	92,1%	96,3%	96,7%	95,7%	85,3%
17448	55	80	35	25	92,3%	96,3%	96,8%	95,9%	86,2%
17449	55	80	35	30	92,4%	96,2%	96,9%	96,2%	87,0%
17450	55	80	35	35	92,5%	96,2%	97,0%	96,4%	87,7%
17451	55	80	35	40	92,6%	96,1%	97,1%	96,7%	88,5%
17452	55	80	35	45	92,7%	96,1%	97,2%	96,9%	89,1%
17453	55	80	35	50	92,9%	96,0%	97,3%	97,1%	89,8%
17454	55	80	35	55	93,0%	96,0%	97,3%	97,3%	90,4%
17455	55	80	35	60	93,1%	95,9%	97,4%	97,5%	91,0%
17456	55	80	35	65	93,2%	95,9%	97,5%	97,6%	91,5%

17457	55	80	35	70	93,3%	95,8%	97,6%	97,8%	92,0%
17458	55	80	35	75	93,4%	95,8%	97,7%	97,9%	92,5%
17459	55	80	35	80	93,5%	95,7%	97,8%	98,1%	93,0%
17460	55	80	40	20	90,1%	95,4%	96,2%	95,4%	84,8%
17461	55	80	40	25	90,3%	95,4%	96,4%	95,7%	85,7%
17462	55	80	40	30	90,4%	95,3%	96,5%	96,0%	86,5%
17463	55	80	40	35	90,6%	95,3%	96,6%	96,2%	87,3%
17464	55	80	40	40	90,7%	95,2%	96,7%	96,5%	88,0%
17465	55	80	40	45	90,9%	95,1%	96,8%	96,7%	88,7%
17466	55	80	40	50	91,0%	95,1%	96,9%	96,9%	89,4%
17467	55	80	40	55	91,2%	95,0%	97,0%	97,1%	90,0%
17468	55	80	40	60	91,3%	94,9%	97,1%	97,3%	90,6%
17469	55	80	40	65	91,5%	94,9%	97,2%	97,5%	91,2%
17470	55	80	40	70	91,6%	94,8%	97,3%	97,6%	91,7%
17471	55	80	40	75	91,7%	94,8%	97,4%	97,8%	92,2%
17472	55	80	40	80	91,9%	94,7%	97,5%	97,9%	92,7%
17473	55	80	45	20	87,7%	94,3%	95,8%	95,1%	84,3%
17474	55	80	45	25	87,9%	94,2%	95,9%	95,4%	85,2%
17475	55	80	45	30	88,1%	94,2%	96,0%	95,7%	86,0%
17476	55	80	45	35	88,3%	94,1%	96,2%	96,0%	86,8%
17477	55	80	45	40	88,5%	94,0%	96,3%	96,3%	87,6%
17478	55	80	45	45	88,6%	93,9%	96,4%	96,5%	88,3%
17479	55	80	45	50	88,8%	93,9%	96,5%	96,7%	89,0%
17480	55	80	45	55	89,0%	93,8%	96,6%	96,9%	89,7%
17481	55	80	45	60	89,2%	93,7%	96,7%	97,1%	90,3%
17482	55	80	45	65	89,3%	93,6%	96,8%	97,3%	90,9%
17483	55	80	45	70	89,5%	93,6%	96,9%	97,5%	91,4%
17484	55	80	45	75	89,7%	93,5%	97,0%	97,7%	91,9%
17485	55	80	45	80	89,8%	93,4%	97,1%	97,8%	92,4%
17486	55	80	50	20	84,8%	92,9%	95,2%	94,8%	83,7%
17487	55	80	50	25	85,0%	92,8%	95,4%	95,2%	84,6%
17488	55	80	50	30	85,2%	92,8%	95,5%	95,5%	85,5%
17489	55	80	50	35	85,5%	92,7%	95,7%	95,8%	86,3%

17490	55	80	50	40	85,7%	92,6%	95,8%	96,0%	87,1%
17491	55	80	50	45	85,9%	92,5%	95,9%	96,3%	87,9%
17492	55	80	50	50	86,1%	92,4%	96,1%	96,5%	88,6%
17493	55	80	50	55	86,3%	92,3%	96,2%	96,7%	89,3%
17494	55	80	50	60	86,5%	92,2%	96,3%	97,0%	89,9%
17495	55	80	50	65	86,7%	92,1%	96,4%	97,2%	90,5%
17496	55	80	50	70	86,9%	92,0%	96,6%	97,3%	91,1%
17497	55	80	50	75	87,1%	91,9%	96,7%	97,5%	91,6%
17498	55	80	50	80	87,3%	91,8%	96,8%	97,7%	92,1%
17499	55	80	55	20	81,3%	91,3%	94,6%	94,5%	83,2%
17500	55	80	55	25	81,6%	91,2%	94,8%	94,9%	84,1%
17501	55	80	55	30	81,9%	91,0%	95,0%	95,2%	85,0%
17502	55	80	55	35	82,1%	90,9%	95,1%	95,5%	85,8%
17503	55	80	55	40	82,4%	90,8%	95,3%	95,8%	86,7%
17504	55	80	55	45	82,6%	90,7%	95,4%	96,1%	87,4%
17505	55	80	55	50	82,9%	90,6%	95,6%	96,3%	88,2%
17506	55	80	55	55	83,1%	90,5%	95,7%	96,5%	88,9%
17507	55	80	55	60	83,4%	90,4%	95,9%	96,8%	89,5%
17508	55	80	55	65	83,6%	90,3%	96,0%	97,0%	90,1%
17509	55	80	55	70	83,9%	90,2%	96,1%	97,2%	90,7%
17510	55	80	55	75	84,1%	90,0%	96,2%	97,4%	91,3%
17511	55	80	55	80	84,3%	89,9%	96,4%	97,5%	91,8%
17512	55	80	60	20	77,3%	89,2%	94,0%	94,2%	82,6%
17513	55	80	60	25	77,6%	89,1%	94,2%	94,6%	83,5%
17514	55	80	60	30	77,9%	89,0%	94,3%	94,9%	84,5%
17515	55	80	60	35	78,2%	88,9%	94,5%	95,2%	85,3%
17516	55	80	60	40	78,5%	88,7%	94,7%	95,5%	86,2%
17517	55	80	60	45	78,8%	88,6%	94,9%	95,8%	87,0%
17518	55	80	60	50	79,1%	88,5%	95,0%	96,1%	87,7%
17519	55	80	60	55	79,4%	88,3%	95,2%	96,3%	88,4%
17520	55	80	60	60	79,7%	88,2%	95,3%	96,6%	89,1%
17521	55	80	60	65	80,0%	88,0%	95,5%	96,8%	89,8%
17522	55	80	60	70	80,2%	87,9%	95,6%	97,0%	90,4%

17523	55	80	60	75	80,5%	87,8%	95,8%	97,2%	91,0%
17524	55	80	60	80	80,8%	87,6%	95,9%	97,4%	91,5%
17525	55	80	65	20	72,7%	86,8%	93,2%	93,9%	82,0%
17526	55	80	65	25	73,0%	86,7%	93,4%	94,2%	83,0%
17527	55	80	65	30	73,4%	86,5%	93,6%	94,6%	83,9%
17528	55	80	65	35	73,7%	86,3%	93,8%	94,9%	84,8%
17529	55	80	65	40	74,0%	86,2%	94,0%	95,3%	85,7%
17530	55	80	65	45	74,4%	86,0%	94,2%	95,6%	86,5%
17531	55	80	65	50	74,7%	85,9%	94,4%	95,9%	87,3%
17532	55	80	65	55	75,1%	85,7%	94,6%	96,1%	88,0%
17533	55	80	65	60	75,4%	85,6%	94,7%	96,4%	88,7%
17534	55	80	65	65	75,7%	85,4%	94,9%	96,6%	89,4%
17535	55	80	65	70	76,0%	85,2%	95,1%	96,8%	90,0%
17536	55	80	65	75	76,3%	85,1%	95,2%	97,0%	90,6%
17537	55	80	65	80	76,7%	84,9%	95,4%	97,2%	91,2%
17538	55	80	70	20	67,5%	83,9%	92,4%	93,5%	81,4%
17539	55	80	70	25	67,9%	83,8%	92,6%	93,9%	82,4%
17540	55	80	70	30	68,3%	83,6%	92,9%	94,3%	83,3%
17541	55	80	70	35	68,7%	83,4%	93,1%	94,6%	84,3%
17542	55	80	70	40	69,0%	83,2%	93,3%	95,0%	85,2%
17543	55	80	70	45	69,4%	83,0%	93,5%	95,3%	86,0%
17544	55	80	70	50	69,8%	82,8%	93,7%	95,6%	86,8%
17545	55	80	70	55	70,2%	82,7%	93,9%	95,9%	87,6%
17546	55	80	70	60	70,5%	82,5%	94,1%	96,1%	88,3%
17547	55	80	70	65	70,9%	82,3%	94,3%	96,4%	89,0%
17548	55	80	70	70	71,2%	82,1%	94,5%	96,6%	89,6%
17549	55	80	70	75	71,6%	81,9%	94,6%	96,8%	90,3%
17550	55	80	70	80	72,0%	81,7%	94,8%	97,0%	90,8%
17551	55	80	75	20	61,9%	80,6%	91,5%	93,1%	80,7%
17552	55	80	75	25	62,3%	80,4%	91,7%	93,5%	81,8%
17553	55	80	75	30	62,7%	80,2%	92,0%	93,9%	82,8%
17554	55	80	75	35	63,1%	79,9%	92,2%	94,3%	83,7%
17555	55	80	75	40	63,5%	79,7%	92,5%	94,7%	84,6%

17556	55	80	75	45	63,9%	79,5%	92,7%	95,0%	85,5%
17557	55	80	75	50	64,3%	79,3%	92,9%	95,3%	86,3%
17558	55	80	75	55	64,7%	79,1%	93,1%	95,6%	87,1%
17559	55	80	75	60	65,1%	78,9%	93,4%	95,9%	87,9%
17560	55	80	75	65	65,5%	78,7%	93,6%	96,2%	88,6%
17561	55	80	75	70	65,9%	78,4%	93,8%	96,4%	89,3%
17562	55	80	75	75	66,3%	78,2%	94,0%	96,7%	89,9%
17563	55	80	75	80	66,7%	78,0%	94,2%	96,9%	90,5%
17564	55	80	80	20	55,9%	76,7%	90,4%	92,7%	80,1%
17565	55	80	80	25	56,3%	76,5%	90,7%	93,2%	81,1%
17566	55	80	80	30	56,8%	76,2%	91,0%	93,6%	82,2%
17567	55	80	80	35	57,2%	76,0%	91,3%	94,0%	83,2%
17568	55	80	80	40	57,6%	75,7%	91,6%	94,4%	84,1%
17569	55	80	80	45	58,1%	75,5%	91,8%	94,7%	85,0%
17570	55	80	80	50	58,5%	75,3%	92,1%	95,1%	85,8%
17571	55	80	80	55	58,9%	75,0%	92,3%	95,4%	86,7%
17572	55	80	80	60	59,3%	74,8%	92,6%	95,7%	87,4%
17573	55	80	80	65	59,8%	74,5%	92,8%	95,9%	88,2%
17574	55	80	80	70	60,2%	74,3%	93,0%	96,2%	88,9%
17575	55	80	80	75	60,6%	74,0%	93,2%	96,4%	89,5%
17576	55	80	80	80	61,0%	73,8%	93,4%	96,7%	90,1%
17577	60	20	20	20	91,5%	96,6%	98,9%	99,0%	96,6%
17578	60	20	20	25	91,7%	96,6%	99,0%	99,0%	96,8%
17579	60	20	20	30	91,8%	96,5%	99,0%	99,1%	97,0%
17580	60	20	20	35	91,9%	96,5%	99,0%	99,2%	97,2%
17581	60	20	20	40	92,1%	96,4%	99,1%	99,2%	97,4%
17582	60	20	20	45	92,2%	96,4%	99,1%	99,3%	97,5%
17583	60	20	20	50	92,3%	96,3%	99,1%	99,3%	97,7%
17584	60	20	20	55	92,4%	96,3%	99,2%	99,4%	97,8%
17585	60	20	20	60	92,6%	96,3%	99,2%	99,4%	98,0%
17586	60	20	20	65	92,7%	96,2%	99,2%	99,4%	98,1%
17587	60	20	20	70	92,8%	96,2%	99,2%	99,5%	98,2%
17588	60	20	20	75	92,9%	96,1%	99,3%	99,5%	98,3%

17589	60	20	20	80	93,0%	96,1%	99,3%	99,5%	98,5%
17590	60	20	25	20	89,4%	95,8%	98,8%	98,9%	96,4%
17591	60	20	25	25	89,6%	95,7%	98,8%	99,0%	96,6%
17592	60	20	25	30	89,7%	95,7%	98,9%	99,0%	96,9%
17593	60	20	25	35	89,9%	95,6%	98,9%	99,1%	97,1%
17594	60	20	25	40	90,1%	95,6%	99,0%	99,2%	97,2%
17595	60	20	25	45	90,2%	95,5%	99,0%	99,2%	97,4%
17596	60	20	25	50	90,4%	95,4%	99,0%	99,3%	97,6%
17597	60	20	25	55	90,5%	95,4%	99,1%	99,3%	97,7%
17598	60	20	25	60	90,7%	95,3%	99,1%	99,4%	97,9%
17599	60	20	25	65	90,8%	95,3%	99,1%	99,4%	98,0%
17600	60	20	25	70	91,0%	95,2%	99,1%	99,4%	98,2%
17601	60	20	25	75	91,1%	95,2%	99,2%	99,5%	98,3%
17602	60	20	25	80	91,3%	95,1%	99,2%	99,5%	98,4%
17603	60	20	30	20	86,8%	94,7%	98,6%	98,8%	96,3%
17604	60	20	30	25	87,0%	94,7%	98,7%	98,9%	96,5%
17605	60	20	30	30	87,2%	94,6%	98,7%	99,0%	96,7%
17606	60	20	30	35	87,4%	94,5%	98,8%	99,1%	96,9%
17607	60	20	30	40	87,6%	94,5%	98,8%	99,1%	97,1%
17608	60	20	30	45	87,8%	94,4%	98,9%	99,2%	97,3%
17609	60	20	30	50	88,0%	94,3%	98,9%	99,2%	97,5%
17610	60	20	30	55	88,2%	94,3%	98,9%	99,3%	97,7%
17611	60	20	30	60	88,4%	94,2%	99,0%	99,3%	97,8%
17612	60	20	30	65	88,6%	94,1%	99,0%	99,4%	97,9%
17613	60	20	30	70	88,7%	94,0%	99,0%	99,4%	98,1%
17614	60	20	30	75	88,9%	94,0%	99,1%	99,5%	98,2%
17615	60	20	30	80	89,1%	93,9%	99,1%	99,5%	98,3%
17616	60	20	35	20	83,8%	93,5%	98,5%	98,8%	96,1%
17617	60	20	35	25	84,0%	93,4%	98,5%	98,8%	96,4%
17618	60	20	35	30	84,2%	93,3%	98,6%	98,9%	96,6%
17619	60	20	35	35	84,5%	93,2%	98,6%	99,0%	96,8%
17620	60	20	35	40	84,7%	93,1%	98,7%	99,1%	97,0%
17621	60	20	35	45	84,9%	93,0%	98,7%	99,1%	97,2%

17622	60	20	35	50	85,1%	93,0%	98,8%	99,2%	97,4%
17623	60	20	35	55	85,4%	92,9%	98,8%	99,2%	97,6%
17624	60	20	35	60	85,6%	92,8%	98,8%	99,3%	97,7%
17625	60	20	35	65	85,8%	92,7%	98,9%	99,3%	97,9%
17626	60	20	35	70	86,0%	92,6%	98,9%	99,4%	98,0%
17627	60	20	35	75	86,2%	92,5%	98,9%	99,4%	98,1%
17628	60	20	35	80	86,4%	92,4%	99,0%	99,5%	98,3%
17629	60	20	40	20	80,1%	91,9%	98,3%	98,7%	96,0%
17630	60	20	40	25	80,4%	91,8%	98,3%	98,8%	96,2%
17631	60	20	40	30	80,7%	91,7%	98,4%	98,9%	96,5%
17632	60	20	40	35	80,9%	91,6%	98,4%	98,9%	96,7%
17633	60	20	40	40	81,2%	91,5%	98,5%	99,0%	96,9%
17634	60	20	40	45	81,5%	91,4%	98,5%	99,1%	97,1%
17635	60	20	40	50	81,7%	91,3%	98,6%	99,1%	97,3%
17636	60	20	40	55	82,0%	91,2%	98,6%	99,2%	97,5%
17637	60	20	40	60	82,3%	91,1%	98,7%	99,2%	97,6%
17638	60	20	40	65	82,5%	91,0%	98,7%	99,3%	97,8%
17639	60	20	40	70	82,8%	90,9%	98,8%	99,3%	97,9%
17640	60	20	40	75	83,0%	90,8%	98,8%	99,4%	98,1%
17641	60	20	40	80	83,3%	90,6%	98,8%	99,4%	98,2%
17642	60	20	45	20	75,9%	90,0%	98,1%	98,6%	95,8%
17643	60	20	45	25	76,2%	89,9%	98,1%	98,7%	96,1%
17644	60	20	45	30	76,5%	89,8%	98,2%	98,8%	96,3%
17645	60	20	45	35	76,8%	89,6%	98,2%	98,9%	96,5%
17646	60	20	45	40	77,1%	89,5%	98,3%	98,9%	96,8%
17647	60	20	45	45	77,5%	89,4%	98,3%	99,0%	97,0%
17648	60	20	45	50	77,8%	89,3%	98,4%	99,1%	97,2%
17649	60	20	45	55	78,1%	89,1%	98,5%	99,1%	97,4%
17650	60	20	45	60	78,4%	89,0%	98,5%	99,2%	97,5%
17651	60	20	45	65	78,7%	88,9%	98,6%	99,2%	97,7%
17652	60	20	45	70	79,0%	88,8%	98,6%	99,3%	97,8%
17653	60	20	45	75	79,3%	88,6%	98,6%	99,3%	98,0%
17654	60	20	45	80	79,5%	88,5%	98,7%	99,4%	98,1%

17655	60	20	50	20	71,1%	87,7%	97,8%	98,5%	95,6%
17656	60	20	50	25	71,4%	87,6%	97,9%	98,6%	95,9%
17657	60	20	50	30	71,8%	87,4%	97,9%	98,7%	96,2%
17658	60	20	50	35	72,2%	87,3%	98,0%	98,8%	96,4%
17659	60	20	50	40	72,5%	87,1%	98,1%	98,9%	96,6%
17660	60	20	50	45	72,9%	87,0%	98,1%	98,9%	96,9%
17661	60	20	50	50	73,2%	86,8%	98,2%	99,0%	97,1%
17662	60	20	50	55	73,6%	86,7%	98,3%	99,1%	97,2%
17663	60	20	50	60	73,9%	86,5%	98,3%	99,1%	97,4%
17664	60	20	50	65	74,2%	86,4%	98,4%	99,2%	97,6%
17665	60	20	50	70	74,6%	86,2%	98,4%	99,3%	97,7%
17666	60	20	50	75	74,9%	86,1%	98,5%	99,3%	97,9%
17667	60	20	50	80	75,2%	85,9%	98,5%	99,3%	98,0%
17668	60	20	55	20	65,8%	85,0%	97,5%	98,4%	95,5%
17669	60	20	55	25	66,2%	84,8%	97,6%	98,5%	95,7%
17670	60	20	55	30	66,5%	84,7%	97,7%	98,6%	96,0%
17671	60	20	55	35	66,9%	84,5%	97,7%	98,7%	96,3%
17672	60	20	55	40	67,3%	84,3%	97,8%	98,8%	96,5%
17673	60	20	55	45	67,7%	84,2%	97,9%	98,9%	96,7%
17674	60	20	55	50	68,1%	84,0%	98,0%	99,0%	96,9%
17675	60	20	55	55	68,5%	83,8%	98,0%	99,0%	97,1%
17676	60	20	55	60	68,9%	83,6%	98,1%	99,1%	97,3%
17677	60	20	55	65	69,2%	83,4%	98,2%	99,2%	97,5%
17678	60	20	55	70	69,6%	83,3%	98,2%	99,2%	97,7%
17679	60	20	55	75	70,0%	83,1%	98,3%	99,3%	97,8%
17680	60	20	55	80	70,3%	82,9%	98,3%	99,3%	97,9%
17681	60	20	60	20	60,0%	81,8%	97,2%	98,3%	95,3%
17682	60	20	60	25	60,4%	81,6%	97,3%	98,4%	95,6%
17683	60	20	60	30	60,8%	81,4%	97,4%	98,5%	95,8%
17684	60	20	60	35	61,3%	81,2%	97,5%	98,6%	96,1%
17685	60	20	60	40	61,7%	81,0%	97,5%	98,7%	96,4%
17686	60	20	60	45	62,1%	80,8%	97,6%	98,8%	96,6%
17687	60	20	60	50	62,5%	80,6%	97,7%	98,9%	96,8%

17688	60	20	60	55	62,9%	80,4%	97,8%	99,0%	97,0%
17689	60	20	60	60	63,3%	80,2%	97,8%	99,0%	97,2%
17690	60	20	60	65	63,7%	80,0%	97,9%	99,1%	97,4%
17691	60	20	60	70	64,1%	79,8%	98,0%	99,2%	97,6%
17692	60	20	60	75	64,6%	79,6%	98,1%	99,2%	97,7%
17693	60	20	60	80	65,0%	79,4%	98,1%	99,3%	97,9%
17694	60	20	65	20	54,0%	78,1%	96,8%	98,2%	95,1%
17695	60	20	65	25	54,4%	77,9%	96,9%	98,3%	95,4%
17696	60	20	65	30	54,8%	77,7%	97,0%	98,5%	95,7%
17697	60	20	65	35	55,3%	77,4%	97,1%	98,6%	96,0%
17698	60	20	65	40	55,7%	77,2%	97,2%	98,7%	96,2%
17699	60	20	65	45	56,1%	77,0%	97,3%	98,7%	96,5%
17700	60	20	65	50	56,6%	76,8%	97,4%	98,8%	96,7%
17701	60	20	65	55	57,0%	76,5%	97,5%	98,9%	96,9%
17702	60	20	65	60	57,4%	76,3%	97,6%	99,0%	97,1%
17703	60	20	65	65	57,9%	76,0%	97,6%	99,0%	97,3%
17704	60	20	65	70	58,3%	75,8%	97,7%	99,1%	97,5%
17705	60	20	65	75	58,7%	75,6%	97,8%	99,2%	97,6%
17706	60	20	65	80	59,1%	75,3%	97,9%	99,2%	97,8%
17707	60	20	70	20	47,8%	73,9%	96,4%	98,1%	94,9%
17708	60	20	70	25	48,2%	73,7%	96,5%	98,2%	95,2%
17709	60	20	70	30	48,7%	73,4%	96,7%	98,4%	95,5%
17710	60	20	70	35	49,1%	73,2%	96,8%	98,5%	95,8%
17711	60	20	70	40	49,6%	72,9%	96,9%	98,6%	96,1%
17712	60	20	70	45	50,0%	72,6%	97,0%	98,7%	96,3%
17713	60	20	70	50	50,4%	72,4%	97,1%	98,8%	96,5%
17714	60	20	70	55	50,9%	72,1%	97,2%	98,8%	96,8%
17715	60	20	70	60	51,3%	71,9%	97,3%	98,9%	97,0%
17716	60	20	70	65	51,8%	71,6%	97,3%	99,0%	97,2%
17717	60	20	70	70	52,2%	71,3%	97,4%	99,0%	97,4%
17718	60	20	70	75	52,6%	71,1%	97,5%	99,1%	97,5%
17719	60	20	70	80	53,1%	70,8%	97,6%	99,2%	97,7%
17720	60	20	75	20	41,7%	69,2%	96,0%	98,0%	94,7%

17721	60	20	75	25	42,1%	69,0%	96,1%	98,1%	95,0%
17722	60	20	75	30	42,6%	68,7%	96,2%	98,3%	95,3%
17723	60	20	75	35	43,0%	68,4%	96,4%	98,4%	95,6%
17724	60	20	75	40	43,4%	68,1%	96,5%	98,5%	95,9%
17725	60	20	75	45	43,9%	67,8%	96,6%	98,6%	96,2%
17726	60	20	75	50	44,3%	67,5%	96,7%	98,7%	96,4%
17727	60	20	75	55	44,7%	67,3%	96,8%	98,8%	96,6%
17728	60	20	75	60	45,2%	67,0%	96,9%	98,8%	96,9%
17729	60	20	75	65	45,6%	66,7%	97,0%	98,9%	97,1%
17730	60	20	75	70	46,0%	66,4%	97,1%	99,0%	97,2%
17731	60	20	75	75	46,5%	66,1%	97,2%	99,1%	97,4%
17732	60	20	75	80	46,9%	65,8%	97,3%	99,1%	97,6%
17733	60	20	80	20	35,8%	64,1%	95,5%	97,9%	94,5%
17734	60	20	80	25	36,3%	63,8%	95,6%	98,0%	94,8%
17735	60	20	80	30	36,7%	63,5%	95,8%	98,2%	95,1%
17736	60	20	80	35	37,1%	63,2%	95,9%	98,3%	95,4%
17737	60	20	80	40	37,5%	62,9%	96,0%	98,4%	95,7%
17738	60	20	80	45	37,9%	62,6%	96,2%	98,5%	96,0%
17739	60	20	80	50	38,3%	62,3%	96,3%	98,6%	96,3%
17740	60	20	80	55	38,7%	62,0%	96,4%	98,7%	96,5%
17741	60	20	80	60	39,1%	61,7%	96,5%	98,8%	96,7%
17742	60	20	80	65	39,6%	61,4%	96,6%	98,9%	96,9%
17743	60	20	80	70	40,0%	61,1%	96,7%	98,9%	97,1%
17744	60	20	80	75	40,4%	60,7%	96,8%	99,0%	97,3%
17745	60	20	80	80	40,8%	60,4%	96,9%	99,1%	97,5%
17746	60	25	20	20	92,2%	96,9%	98,9%	98,9%	96,2%
17747	60	25	20	25	92,3%	96,8%	98,9%	99,0%	96,5%
17748	60	25	20	30	92,4%	96,8%	99,0%	99,0%	96,7%
17749	60	25	20	35	92,6%	96,7%	99,0%	99,1%	96,9%
17750	60	25	20	40	92,7%	96,7%	99,0%	99,2%	97,1%
17751	60	25	20	45	92,8%	96,7%	99,1%	99,2%	97,3%
17752	60	25	20	50	92,9%	96,6%	99,1%	99,3%	97,5%
17753	60	25	20	55	93,0%	96,6%	99,1%	99,3%	97,6%

17754	60	25	20	60	93,2%	96,5%	99,2%	99,4%	97,8%
17755	60	25	20	65	93,3%	96,5%	99,2%	99,4%	97,9%
17756	60	25	20	70	93,4%	96,4%	99,2%	99,4%	98,1%
17757	60	25	20	75	93,5%	96,4%	99,2%	99,5%	98,2%
17758	60	25	20	80	93,6%	96,4%	99,3%	99,5%	98,3%
17759	60	25	25	20	90,2%	96,1%	98,8%	98,8%	96,1%
17760	60	25	25	25	90,4%	96,0%	98,8%	98,9%	96,3%
17761	60	25	25	30	90,5%	96,0%	98,8%	99,0%	96,6%
17762	60	25	25	35	90,7%	95,9%	98,9%	99,0%	96,8%
17763	60	25	25	40	90,8%	95,9%	98,9%	99,1%	97,0%
17764	60	25	25	45	91,0%	95,8%	99,0%	99,2%	97,2%
17765	60	25	25	50	91,1%	95,8%	99,0%	99,2%	97,4%
17766	60	25	25	55	91,3%	95,7%	99,0%	99,3%	97,5%
17767	60	25	25	60	91,4%	95,7%	99,1%	99,3%	97,7%
17768	60	25	25	65	91,5%	95,6%	99,1%	99,4%	97,8%
17769	60	25	25	70	91,7%	95,6%	99,1%	99,4%	98,0%
17770	60	25	25	75	91,8%	95,5%	99,1%	99,4%	98,1%
17771	60	25	25	80	91,9%	95,4%	99,2%	99,5%	98,2%
17772	60	25	30	20	87,8%	95,1%	98,6%	98,8%	95,9%
17773	60	25	30	25	88,0%	95,1%	98,7%	98,8%	96,2%
17774	60	25	30	30	88,2%	95,0%	98,7%	98,9%	96,4%
17775	60	25	30	35	88,4%	94,9%	98,7%	99,0%	96,6%
17776	60	25	30	40	88,6%	94,9%	98,8%	99,0%	96,9%
17777	60	25	30	45	88,7%	94,8%	98,8%	99,1%	97,1%
17778	60	25	30	50	88,9%	94,7%	98,9%	99,2%	97,2%
17779	60	25	30	55	89,1%	94,7%	98,9%	99,2%	97,4%
17780	60	25	30	60	89,3%	94,6%	98,9%	99,3%	97,6%
17781	60	25	30	65	89,4%	94,5%	99,0%	99,3%	97,7%
17782	60	25	30	70	89,6%	94,5%	99,0%	99,4%	97,9%
17783	60	25	30	75	89,7%	94,4%	99,0%	99,4%	98,0%
17784	60	25	30	80	89,9%	94,3%	99,1%	99,5%	98,2%
17785	60	25	35	20	84,9%	93,9%	98,4%	98,7%	95,7%
17786	60	25	35	25	85,1%	93,8%	98,5%	98,8%	96,0%

17787	60	25	35	30	85,4%	93,8%	98,5%	98,8%	96,3%
17788	60	25	35	35	85,6%	93,7%	98,6%	98,9%	96,5%
17789	60	25	35	40	85,8%	93,6%	98,6%	99,0%	96,7%
17790	60	25	35	45	86,0%	93,5%	98,7%	99,1%	96,9%
17791	60	25	35	50	86,2%	93,5%	98,7%	99,1%	97,1%
17792	60	25	35	55	86,4%	93,4%	98,8%	99,2%	97,3%
17793	60	25	35	60	86,6%	93,3%	98,8%	99,2%	97,5%
17794	60	25	35	65	86,8%	93,2%	98,8%	99,3%	97,7%
17795	60	25	35	70	87,0%	93,1%	98,9%	99,3%	97,8%
17796	60	25	35	75	87,2%	93,0%	98,9%	99,4%	97,9%
17797	60	25	35	80	87,4%	93,0%	98,9%	99,4%	98,1%
17798	60	25	40	20	81,5%	92,5%	98,2%	98,6%	95,6%
17799	60	25	40	25	81,7%	92,4%	98,3%	98,7%	95,9%
17800	60	25	40	30	82,0%	92,3%	98,3%	98,8%	96,1%
17801	60	25	40	35	82,3%	92,2%	98,4%	98,9%	96,4%
17802	60	25	40	40	82,5%	92,1%	98,4%	98,9%	96,6%
17803	60	25	40	45	82,8%	92,0%	98,5%	99,0%	96,8%
17804	60	25	40	50	83,0%	91,9%	98,5%	99,1%	97,0%
17805	60	25	40	55	83,3%	91,8%	98,6%	99,1%	97,2%
17806	60	25	40	60	83,5%	91,7%	98,6%	99,2%	97,4%
17807	60	25	40	65	83,8%	91,6%	98,7%	99,2%	97,6%
17808	60	25	40	70	84,0%	91,5%	98,7%	99,3%	97,7%
17809	60	25	40	75	84,2%	91,4%	98,8%	99,3%	97,9%
17810	60	25	40	80	84,5%	91,3%	98,8%	99,4%	98,0%
17811	60	25	45	20	77,5%	90,7%	98,0%	98,5%	95,4%
17812	60	25	45	25	77,8%	90,6%	98,1%	98,6%	95,7%
17813	60	25	45	30	78,1%	90,5%	98,1%	98,7%	96,0%
17814	60	25	45	35	78,4%	90,3%	98,2%	98,8%	96,2%
17815	60	25	45	40	78,7%	90,2%	98,2%	98,9%	96,5%
17816	60	25	45	45	79,0%	90,1%	98,3%	98,9%	96,7%
17817	60	25	45	50	79,3%	90,0%	98,4%	99,0%	96,9%
17818	60	25	45	55	79,5%	89,9%	98,4%	99,1%	97,1%
17819	60	25	45	60	79,8%	89,8%	98,5%	99,1%	97,3%

17820	60	25	45	65	80,1%	89,6%	98,5%	99,2%	97,5%
17821	60	25	45	70	80,4%	89,5%	98,6%	99,2%	97,6%
17822	60	25	45	75	80,7%	89,4%	98,6%	99,3%	97,8%
17823	60	25	45	80	80,9%	89,3%	98,7%	99,3%	97,9%
17824	60	25	50	20	72,9%	88,5%	97,7%	98,4%	95,2%
17825	60	25	50	25	73,2%	88,4%	97,8%	98,5%	95,5%
17826	60	25	50	30	73,6%	88,3%	97,9%	98,6%	95,8%
17827	60	25	50	35	73,9%	88,1%	97,9%	98,7%	96,1%
17828	60	25	50	40	74,2%	88,0%	98,0%	98,8%	96,3%
17829	60	25	50	45	74,6%	87,9%	98,1%	98,9%	96,6%
17830	60	25	50	50	74,9%	87,7%	98,1%	98,9%	96,8%
17831	60	25	50	55	75,2%	87,6%	98,2%	99,0%	97,0%
17832	60	25	50	60	75,6%	87,4%	98,3%	99,1%	97,2%
17833	60	25	50	65	75,9%	87,3%	98,3%	99,1%	97,4%
17834	60	25	50	70	76,2%	87,1%	98,4%	99,2%	97,5%
17835	60	25	50	75	76,5%	87,0%	98,4%	99,2%	97,7%
17836	60	25	50	80	76,8%	86,8%	98,5%	99,3%	97,8%
17837	60	25	55	20	67,7%	86,0%	97,4%	98,3%	95,0%
17838	60	25	55	25	68,1%	85,8%	97,5%	98,4%	95,3%
17839	60	25	55	30	68,5%	85,7%	97,6%	98,5%	95,6%
17840	60	25	55	35	68,9%	85,5%	97,7%	98,6%	95,9%
17841	60	25	55	40	69,2%	85,3%	97,8%	98,7%	96,2%
17842	60	25	55	45	69,6%	85,2%	97,8%	98,8%	96,4%
17843	60	25	55	50	70,0%	85,0%	97,9%	98,9%	96,6%
17844	60	25	55	55	70,4%	84,8%	98,0%	99,0%	96,9%
17845	60	25	55	60	70,7%	84,7%	98,0%	99,0%	97,1%
17846	60	25	55	65	71,1%	84,5%	98,1%	99,1%	97,2%
17847	60	25	55	70	71,5%	84,3%	98,2%	99,1%	97,4%
17848	60	25	55	75	71,8%	84,2%	98,2%	99,2%	97,6%
17849	60	25	55	80	72,2%	84,0%	98,3%	99,3%	97,7%
17850	60	25	60	20	62,1%	83,0%	97,1%	98,2%	94,8%
17851	60	25	60	25	62,5%	82,8%	97,2%	98,3%	95,1%
17852	60	25	60	30	62,9%	82,6%	97,3%	98,4%	95,5%

17853	60	25	60	35	63,3%	82,4%	97,4%	98,5%	95,7%
17854	60	25	60	40	63,8%	82,2%	97,5%	98,6%	96,0%
17855	60	25	60	45	64,2%	82,0%	97,5%	98,7%	96,3%
17856	60	25	60	50	64,6%	81,8%	97,6%	98,8%	96,5%
17857	60	25	60	55	65,0%	81,6%	97,7%	98,9%	96,7%
17858	60	25	60	60	65,4%	81,4%	97,8%	99,0%	96,9%
17859	60	25	60	65	65,8%	81,2%	97,9%	99,0%	97,1%
17860	60	25	60	70	66,2%	81,0%	97,9%	99,1%	97,3%
17861	60	25	60	75	66,6%	80,8%	98,0%	99,2%	97,5%
17862	60	25	60	80	66,9%	80,6%	98,1%	99,2%	97,7%
17863	60	25	65	20	56,2%	79,4%	96,7%	98,1%	94,6%
17864	60	25	65	25	56,6%	79,2%	96,8%	98,2%	95,0%
17865	60	25	65	30	57,0%	79,0%	96,9%	98,3%	95,3%
17866	60	25	65	35	57,4%	78,8%	97,0%	98,4%	95,6%
17867	60	25	65	40	57,9%	78,6%	97,1%	98,6%	95,8%
17868	60	25	65	45	58,3%	78,4%	97,2%	98,6%	96,1%
17869	60	25	65	50	58,7%	78,1%	97,3%	98,7%	96,4%
17870	60	25	65	55	59,2%	77,9%	97,4%	98,8%	96,6%
17871	60	25	65	60	59,6%	77,7%	97,5%	98,9%	96,8%
17872	60	25	65	65	60,0%	77,5%	97,6%	99,0%	97,0%
17873	60	25	65	70	60,4%	77,2%	97,7%	99,0%	97,2%
17874	60	25	65	75	60,9%	77,0%	97,7%	99,1%	97,4%
17875	60	25	65	80	61,3%	76,8%	97,8%	99,2%	97,6%
17876	60	25	70	20	50,0%	75,4%	96,3%	98,0%	94,4%
17877	60	25	70	25	50,5%	75,2%	96,4%	98,1%	94,7%
17878	60	25	70	30	50,9%	74,9%	96,6%	98,2%	95,1%
17879	60	25	70	35	51,3%	74,7%	96,7%	98,4%	95,4%
17880	60	25	70	40	51,8%	74,4%	96,8%	98,5%	95,7%
17881	60	25	70	45	52,2%	74,2%	96,9%	98,6%	96,0%
17882	60	25	70	50	52,7%	73,9%	97,0%	98,7%	96,2%
17883	60	25	70	55	53,1%	73,7%	97,1%	98,7%	96,5%
17884	60	25	70	60	53,5%	73,4%	97,2%	98,8%	96,7%
17885	60	25	70	65	54,0%	73,2%	97,3%	98,9%	96,9%

17886	60	25	70	70	54,4%	72,9%	97,4%	99,0%	97,1%
17887	60	25	70	75	54,8%	72,7%	97,4%	99,0%	97,3%
17888	60	25	70	80	55,3%	72,4%	97,5%	99,1%	97,5%
17889	60	25	75	20	43,9%	70,9%	95,9%	97,9%	94,2%
17890	60	25	75	25	44,3%	70,6%	96,0%	98,0%	94,5%
17891	60	25	75	30	44,7%	70,3%	96,1%	98,1%	94,9%
17892	60	25	75	35	45,2%	70,1%	96,2%	98,3%	95,2%
17893	60	25	75	40	45,6%	69,8%	96,4%	98,4%	95,5%
17894	60	25	75	45	46,1%	69,5%	96,5%	98,5%	95,8%
17895	60	25	75	50	46,5%	69,2%	96,6%	98,6%	96,1%
17896	60	25	75	55	46,9%	69,0%	96,7%	98,7%	96,3%
17897	60	25	75	60	47,4%	68,7%	96,8%	98,8%	96,5%
17898	60	25	75	65	47,8%	68,4%	96,9%	98,8%	96,8%
17899	60	25	75	70	48,2%	68,1%	97,0%	98,9%	97,0%
17900	60	25	75	75	48,7%	67,8%	97,1%	99,0%	97,2%
17901	60	25	75	80	49,1%	67,5%	97,2%	99,1%	97,4%
17902	60	25	80	20	37,9%	65,9%	95,3%	97,7%	93,9%
17903	60	25	80	25	38,3%	65,6%	95,5%	97,9%	94,3%
17904	60	25	80	30	38,7%	65,3%	95,6%	98,0%	94,7%
17905	60	25	80	35	39,2%	65,0%	95,8%	98,1%	95,0%
17906	60	25	80	40	39,6%	64,7%	95,9%	98,3%	95,3%
17907	60	25	80	45	40,0%	64,4%	96,0%	98,4%	95,6%
17908	60	25	80	50	40,4%	64,1%	96,2%	98,5%	95,9%
17909	60	25	80	55	40,9%	63,8%	96,3%	98,6%	96,2%
17910	60	25	80	60	41,3%	63,5%	96,4%	98,7%	96,4%
17911	60	25	80	65	41,7%	63,2%	96,5%	98,8%	96,6%
17912	60	25	80	70	42,1%	62,9%	96,6%	98,8%	96,9%
17913	60	25	80	75	42,6%	62,6%	96,7%	98,9%	97,1%
17914	60	25	80	80	43,0%	62,3%	96,8%	99,0%	97,2%
17915	60	30	20	20	92,8%	97,1%	98,9%	98,8%	95,9%
17916	60	30	20	25	92,9%	97,1%	98,9%	98,9%	96,1%
17917	60	30	20	30	93,0%	97,0%	98,9%	99,0%	96,4%
17918	60	30	20	35	93,2%	97,0%	99,0%	99,0%	96,6%

17919	60	30	20	40	93,3%	96,9%	99,0%	99,1%	96,8%
17920	60	30	20	45	93,4%	96,9%	99,0%	99,2%	97,0%
17921	60	30	20	50	93,5%	96,9%	99,1%	99,2%	97,2%
17922	60	30	20	55	93,6%	96,8%	99,1%	99,3%	97,4%
17923	60	30	20	60	93,7%	96,8%	99,1%	99,3%	97,6%
17924	60	30	20	65	93,8%	96,7%	99,2%	99,4%	97,7%
17925	60	30	20	70	93,9%	96,7%	99,2%	99,4%	97,9%
17926	60	30	20	75	94,0%	96,7%	99,2%	99,4%	98,0%
17927	60	30	20	80	94,1%	96,6%	99,2%	99,5%	98,1%
17928	60	30	25	20	91,0%	96,4%	98,7%	98,7%	95,7%
17929	60	30	25	25	91,1%	96,3%	98,8%	98,8%	96,0%
17930	60	30	25	30	91,3%	96,3%	98,8%	98,9%	96,2%
17931	60	30	25	35	91,4%	96,2%	98,8%	99,0%	96,5%
17932	60	30	25	40	91,5%	96,2%	98,9%	99,0%	96,7%
17933	60	30	25	45	91,7%	96,1%	98,9%	99,1%	96,9%
17934	60	30	25	50	91,8%	96,1%	99,0%	99,2%	97,1%
17935	60	30	25	55	91,9%	96,0%	99,0%	99,2%	97,3%
17936	60	30	25	60	92,1%	96,0%	99,0%	99,3%	97,5%
17937	60	30	25	65	92,2%	95,9%	99,1%	99,3%	97,6%
17938	60	30	25	70	92,3%	95,9%	99,1%	99,4%	97,8%
17939	60	30	25	75	92,5%	95,8%	99,1%	99,4%	97,9%
17940	60	30	25	80	92,6%	95,8%	99,1%	99,4%	98,1%
17941	60	30	30	20	88,7%	95,5%	98,6%	98,7%	95,5%
17942	60	30	30	25	88,9%	95,4%	98,6%	98,7%	95,8%
17943	60	30	30	30	89,1%	95,4%	98,7%	98,8%	96,1%
17944	60	30	30	35	89,3%	95,3%	98,7%	98,9%	96,3%
17945	60	30	30	40	89,4%	95,2%	98,7%	99,0%	96,6%
17946	60	30	30	45	89,6%	95,2%	98,8%	99,0%	96,8%
17947	60	30	30	50	89,8%	95,1%	98,8%	99,1%	97,0%
17948	60	30	30	55	89,9%	95,1%	98,9%	99,2%	97,2%
17949	60	30	30	60	90,1%	95,0%	98,9%	99,2%	97,4%
17950	60	30	30	65	90,2%	94,9%	98,9%	99,3%	97,5%
17951	60	30	30	70	90,4%	94,9%	99,0%	99,3%	97,7%

17952	60	30	30	75	90,5%	94,8%	99,0%	99,4%	97,8%
17953	60	30	30	80	90,7%	94,7%	99,0%	99,4%	98,0%
17954	60	30	35	20	86,0%	94,4%	98,4%	98,6%	95,3%
17955	60	30	35	25	86,2%	94,3%	98,4%	98,7%	95,6%
17956	60	30	35	30	86,4%	94,2%	98,5%	98,8%	95,9%
17957	60	30	35	35	86,7%	94,1%	98,5%	98,8%	96,2%
17958	60	30	35	40	86,9%	94,1%	98,6%	98,9%	96,4%
17959	60	30	35	45	87,1%	94,0%	98,6%	99,0%	96,6%
17960	60	30	35	50	87,3%	93,9%	98,7%	99,1%	96,9%
17961	60	30	35	55	87,4%	93,8%	98,7%	99,1%	97,1%
17962	60	30	35	60	87,6%	93,8%	98,8%	99,2%	97,2%
17963	60	30	35	65	87,8%	93,7%	98,8%	99,2%	97,4%
17964	60	30	35	70	88,0%	93,6%	98,8%	99,3%	97,6%
17965	60	30	35	75	88,2%	93,5%	98,9%	99,3%	97,7%
17966	60	30	35	80	88,4%	93,5%	98,9%	99,4%	97,9%
17967	60	30	40	20	82,8%	93,0%	98,2%	98,5%	95,1%
17968	60	30	40	25	83,0%	92,9%	98,2%	98,6%	95,5%
17969	60	30	40	30	83,3%	92,8%	98,3%	98,7%	95,7%
17970	60	30	40	35	83,5%	92,7%	98,3%	98,8%	96,0%
17971	60	30	40	40	83,8%	92,6%	98,4%	98,8%	96,3%
17972	60	30	40	45	84,0%	92,6%	98,4%	98,9%	96,5%
17973	60	30	40	50	84,2%	92,5%	98,5%	99,0%	96,7%
17974	60	30	40	55	84,5%	92,4%	98,5%	99,1%	96,9%
17975	60	30	40	60	84,7%	92,3%	98,6%	99,1%	97,1%
17976	60	30	40	65	84,9%	92,2%	98,6%	99,2%	97,3%
17977	60	30	40	70	85,2%	92,1%	98,7%	99,2%	97,5%
17978	60	30	40	75	85,4%	92,0%	98,7%	99,3%	97,7%
17979	60	30	40	80	85,6%	91,9%	98,8%	99,3%	97,8%
17980	60	30	45	20	79,0%	91,3%	97,9%	98,4%	95,0%
17981	60	30	45	25	79,3%	91,2%	98,0%	98,5%	95,3%
17982	60	30	45	30	79,6%	91,1%	98,1%	98,6%	95,6%
17983	60	30	45	35	79,8%	91,0%	98,1%	98,7%	95,9%
17984	60	30	45	40	80,1%	90,9%	98,2%	98,8%	96,1%

17985	60	30	45	45	80,4%	90,8%	98,2%	98,9%	96,4%
17986	60	30	45	50	80,7%	90,7%	98,3%	98,9%	96,6%
17987	60	30	45	55	81,0%	90,6%	98,4%	99,0%	96,8%
17988	60	30	45	60	81,2%	90,5%	98,4%	99,1%	97,0%
17989	60	30	45	65	81,5%	90,3%	98,5%	99,1%	97,2%
17990	60	30	45	70	81,8%	90,2%	98,5%	99,2%	97,4%
17991	60	30	45	75	82,0%	90,1%	98,6%	99,2%	97,6%
17992	60	30	45	80	82,3%	90,0%	98,6%	99,3%	97,7%
17993	60	30	50	20	74,6%	89,3%	97,7%	98,3%	94,8%
17994	60	30	50	25	74,9%	89,2%	97,7%	98,4%	95,1%
17995	60	30	50	30	75,3%	89,1%	97,8%	98,5%	95,4%
17996	60	30	50	35	75,6%	88,9%	97,9%	98,6%	95,7%
17997	60	30	50	40	75,9%	88,8%	97,9%	98,7%	96,0%
17998	60	30	50	45	76,2%	88,7%	98,0%	98,8%	96,2%
17999	60	30	50	50	76,5%	88,5%	98,1%	98,9%	96,5%
18000	60	30	50	55	76,9%	88,4%	98,1%	98,9%	96,7%
18001	60	30	50	60	77,2%	88,3%	98,2%	99,0%	96,9%
18002	60	30	50	65	77,5%	88,1%	98,3%	99,1%	97,1%
18003	60	30	50	70	77,8%	88,0%	98,3%	99,1%	97,3%
18004	60	30	50	75	78,1%	87,9%	98,4%	99,2%	97,5%
18005	60	30	50	80	78,4%	87,7%	98,4%	99,2%	97,6%
18006	60	30	55	20	69,6%	86,9%	97,4%	98,2%	94,5%
18007	60	30	55	25	70,0%	86,8%	97,4%	98,3%	94,9%
18008	60	30	55	30	70,4%	86,6%	97,5%	98,4%	95,2%
18009	60	30	55	35	70,7%	86,5%	97,6%	98,5%	95,5%
18010	60	30	55	40	71,1%	86,3%	97,7%	98,6%	95,8%
18011	60	30	55	45	71,5%	86,1%	97,8%	98,7%	96,1%
18012	60	30	55	50	71,8%	86,0%	97,8%	98,8%	96,3%
18013	60	30	55	55	72,2%	85,8%	97,9%	98,9%	96,6%
18014	60	30	55	60	72,5%	85,7%	98,0%	98,9%	96,8%
18015	60	30	55	65	72,9%	85,5%	98,0%	99,0%	97,0%
18016	60	30	55	70	73,2%	85,3%	98,1%	99,1%	97,2%
18017	60	30	55	75	73,6%	85,2%	98,2%	99,1%	97,4%

18018	60	30	55	80	73,9%	85,0%	98,2%	99,2%	97,5%
18019	60	30	60	20	64,2%	84,0%	97,0%	98,1%	94,3%
18020	60	30	60	25	64,6%	83,9%	97,1%	98,2%	94,7%
18021	60	30	60	30	65,0%	83,7%	97,2%	98,3%	95,0%
18022	60	30	60	35	65,4%	83,5%	97,3%	98,4%	95,3%
18023	60	30	60	40	65,8%	83,3%	97,4%	98,5%	95,6%
18024	60	30	60	45	66,2%	83,1%	97,5%	98,6%	95,9%
18025	60	30	60	50	66,6%	83,0%	97,6%	98,7%	96,2%
18026	60	30	60	55	67,0%	82,8%	97,6%	98,8%	96,4%
18027	60	30	60	60	67,3%	82,6%	97,7%	98,9%	96,6%
18028	60	30	60	65	67,7%	82,4%	97,8%	99,0%	96,9%
18029	60	30	60	70	68,1%	82,2%	97,9%	99,0%	97,1%
18030	60	30	60	75	68,5%	82,0%	97,9%	99,1%	97,2%
18031	60	30	60	80	68,9%	81,8%	98,0%	99,1%	97,4%
18032	60	30	65	20	58,3%	80,7%	96,6%	98,0%	94,1%
18033	60	30	65	25	58,8%	80,5%	96,7%	98,1%	94,5%
18034	60	30	65	30	59,2%	80,3%	96,8%	98,2%	94,8%
18035	60	30	65	35	59,6%	80,1%	97,0%	98,3%	95,1%
18036	60	30	65	40	60,0%	79,9%	97,0%	98,4%	95,5%
18037	60	30	65	45	60,4%	79,7%	97,1%	98,5%	95,7%
18038	60	30	65	50	60,9%	79,4%	97,2%	98,6%	96,0%
18039	60	30	65	55	61,3%	79,2%	97,3%	98,7%	96,3%
18040	60	30	65	60	61,7%	79,0%	97,4%	98,8%	96,5%
18041	60	30	65	65	62,1%	78,8%	97,5%	98,9%	96,7%
18042	60	30	65	70	62,5%	78,6%	97,6%	99,0%	96,9%
18043	60	30	65	75	62,9%	78,4%	97,7%	99,0%	97,1%
18044	60	30	65	80	63,4%	78,1%	97,7%	99,1%	97,3%
18045	60	30	70	20	52,2%	76,8%	96,2%	97,8%	93,9%
18046	60	30	70	25	52,7%	76,6%	96,3%	98,0%	94,3%
18047	60	30	70	30	53,1%	76,4%	96,4%	98,1%	94,6%
18048	60	30	70	35	53,5%	76,1%	96,6%	98,2%	95,0%
18049	60	30	70	40	54,0%	75,9%	96,7%	98,3%	95,3%
18050	60	30	70	45	54,4%	75,7%	96,8%	98,5%	95,6%

18051	60	30	70	50	54,9%	75,4%	96,9%	98,6%	95,9%
18052	60	30	70	55	55,3%	75,2%	97,0%	98,6%	96,1%
18053	60	30	70	60	55,7%	74,9%	97,1%	98,7%	96,4%
18054	60	30	70	65	56,2%	74,7%	97,2%	98,8%	96,6%
18055	60	30	70	70	56,6%	74,4%	97,3%	98,9%	96,8%
18056	60	30	70	75	57,0%	74,2%	97,4%	99,0%	97,0%
18057	60	30	70	80	57,5%	73,9%	97,4%	99,0%	97,2%
18058	60	30	75	20	46,1%	72,5%	95,7%	97,7%	93,6%
18059	60	30	75	25	46,5%	72,2%	95,9%	97,8%	94,0%
18060	60	30	75	30	46,9%	72,0%	96,0%	98,0%	94,4%
18061	60	30	75	35	47,4%	71,7%	96,1%	98,1%	94,8%
18062	60	30	75	40	47,8%	71,4%	96,2%	98,2%	95,1%
18063	60	30	75	45	48,3%	71,2%	96,4%	98,4%	95,4%
18064	60	30	75	50	48,7%	70,9%	96,5%	98,5%	95,7%
18065	60	30	75	55	49,1%	70,6%	96,6%	98,6%	96,0%
18066	60	30	75	60	49,6%	70,4%	96,7%	98,7%	96,2%
18067	60	30	75	65	50,0%	70,1%	96,8%	98,7%	96,5%
18068	60	30	75	70	50,5%	69,8%	96,9%	98,8%	96,7%
18069	60	30	75	75	50,9%	69,5%	97,0%	98,9%	96,9%
18070	60	30	75	80	51,3%	69,2%	97,1%	99,0%	97,1%
18071	60	30	80	20	40,0%	67,7%	95,2%	97,6%	93,4%
18072	60	30	80	25	40,4%	67,4%	95,3%	97,7%	93,8%
18073	60	30	80	30	40,9%	67,1%	95,5%	97,9%	94,2%
18074	60	30	80	35	41,3%	66,8%	95,6%	98,0%	94,5%
18075	60	30	80	40	41,7%	66,5%	95,8%	98,1%	94,9%
18076	60	30	80	45	42,2%	66,2%	95,9%	98,3%	95,2%
18077	60	30	80	50	42,6%	65,9%	96,0%	98,4%	95,5%
18078	60	30	80	55	43,0%	65,6%	96,2%	98,5%	95,8%
18079	60	30	80	60	43,4%	65,3%	96,3%	98,6%	96,1%
18080	60	30	80	65	43,9%	65,0%	96,4%	98,7%	96,3%
18081	60	30	80	70	44,3%	64,7%	96,5%	98,8%	96,5%
18082	60	30	80	75	44,8%	64,4%	96,6%	98,8%	96,8%
18083	60	30	80	80	45,2%	64,1%	96,7%	98,9%	97,0%

18084	60	35	20	20	93,4%	97,3%	98,8%	98,7%	95,5%
18085	60	35	20	25	93,5%	97,3%	98,9%	98,8%	95,7%
18086	60	35	20	30	93,6%	97,2%	98,9%	98,9%	96,0%
18087	60	35	20	35	93,7%	97,2%	98,9%	99,0%	96,3%
18088	60	35	20	40	93,8%	97,2%	99,0%	99,0%	96,5%
18089	60	35	20	45	93,9%	97,1%	99,0%	99,1%	96,7%
18090	60	35	20	50	94,0%	97,1%	99,1%	99,1%	96,9%
18091	60	35	20	55	94,1%	97,1%	99,1%	99,2%	97,1%
18092	60	35	20	60	94,2%	97,0%	99,1%	99,3%	97,3%
18093	60	35	20	65	94,3%	97,0%	99,1%	99,3%	97,5%
18094	60	35	20	70	94,4%	96,9%	99,2%	99,4%	97,7%
18095	60	35	20	75	94,5%	96,9%	99,2%	99,4%	97,8%
18096	60	35	20	80	94,6%	96,9%	99,2%	99,4%	98,0%
18097	60	35	25	20	91,7%	96,6%	98,7%	98,6%	95,3%
18098	60	35	25	25	91,8%	96,6%	98,7%	98,7%	95,6%
18099	60	35	25	30	91,9%	96,5%	98,8%	98,8%	95,9%
18100	60	35	25	35	92,1%	96,5%	98,8%	98,9%	96,1%
18101	60	35	25	40	92,2%	96,5%	98,9%	99,0%	96,4%
18102	60	35	25	45	92,3%	96,4%	98,9%	99,0%	96,6%
18103	60	35	25	50	92,5%	96,4%	98,9%	99,1%	96,8%
18104	60	35	25	55	92,6%	96,3%	99,0%	99,2%	97,0%
18105	60	35	25	60	92,7%	96,3%	99,0%	99,2%	97,2%
18106	60	35	25	65	92,8%	96,2%	99,0%	99,3%	97,4%
18107	60	35	25	70	92,9%	96,2%	99,1%	99,3%	97,6%
18108	60	35	25	75	93,0%	96,1%	99,1%	99,4%	97,7%
18109	60	35	25	80	93,2%	96,1%	99,1%	99,4%	97,9%
18110	60	35	30	20	89,6%	95,8%	98,5%	98,6%	95,1%
18111	60	35	30	25	89,8%	95,7%	98,6%	98,6%	95,4%
18112	60	35	30	30	89,9%	95,7%	98,6%	98,7%	95,7%
18113	60	35	30	35	90,1%	95,6%	98,7%	98,8%	96,0%
18114	60	35	30	40	90,2%	95,6%	98,7%	98,9%	96,2%
18115	60	35	30	45	90,4%	95,5%	98,7%	99,0%	96,5%
18116	60	35	30	50	90,5%	95,5%	98,8%	99,0%	96,7%

18117	60	35	30	55	90,7%	95,4%	98,8%	99,1%	96,9%
18118	60	35	30	60	90,8%	95,4%	98,9%	99,2%	97,1%
18119	60	35	30	65	91,0%	95,3%	98,9%	99,2%	97,3%
18120	60	35	30	70	91,1%	95,2%	98,9%	99,3%	97,5%
18121	60	35	30	75	91,3%	95,2%	99,0%	99,3%	97,6%
18122	60	35	30	80	91,4%	95,1%	99,0%	99,4%	97,8%
18123	60	35	35	20	87,1%	94,8%	98,3%	98,5%	94,9%
18124	60	35	35	25	87,3%	94,7%	98,4%	98,6%	95,2%
18125	60	35	35	30	87,5%	94,6%	98,4%	98,7%	95,5%
18126	60	35	35	35	87,6%	94,6%	98,5%	98,7%	95,8%
18127	60	35	35	40	87,8%	94,5%	98,5%	98,8%	96,1%
18128	60	35	35	45	88,0%	94,4%	98,6%	98,9%	96,3%
18129	60	35	35	50	88,2%	94,4%	98,6%	99,0%	96,6%
18130	60	35	35	55	88,4%	94,3%	98,7%	99,0%	96,8%
18131	60	35	35	60	88,6%	94,2%	98,7%	99,1%	97,0%
18132	60	35	35	65	88,7%	94,1%	98,8%	99,2%	97,2%
18133	60	35	35	70	88,9%	94,1%	98,8%	99,2%	97,4%
18134	60	35	35	75	89,1%	94,0%	98,8%	99,3%	97,5%
18135	60	35	35	80	89,3%	93,9%	98,9%	99,3%	97,7%
18136	60	35	40	20	84,0%	93,5%	98,1%	98,4%	94,7%
18137	60	35	40	25	84,2%	93,4%	98,2%	98,5%	95,0%
18138	60	35	40	30	84,5%	93,3%	98,2%	98,6%	95,3%
18139	60	35	40	35	84,7%	93,2%	98,3%	98,7%	95,6%
18140	60	35	40	40	84,9%	93,2%	98,3%	98,8%	95,9%
18141	60	35	40	45	85,2%	93,1%	98,4%	98,8%	96,2%
18142	60	35	40	50	85,4%	93,0%	98,4%	98,9%	96,4%
18143	60	35	40	55	85,6%	92,9%	98,5%	99,0%	96,6%
18144	60	35	40	60	85,8%	92,8%	98,5%	99,1%	96,9%
18145	60	35	40	65	86,0%	92,7%	98,6%	99,1%	97,1%
18146	60	35	40	70	86,2%	92,6%	98,6%	99,2%	97,3%
18147	60	35	40	75	86,5%	92,6%	98,7%	99,2%	97,4%
18148	60	35	40	80	86,7%	92,5%	98,7%	99,3%	97,6%
18149	60	35	45	20	80,4%	91,9%	97,9%	98,3%	94,5%

18150	60	35	45	25	80,7%	91,8%	97,9%	98,4%	94,8%
18151	60	35	45	30	81,0%	91,7%	98,0%	98,5%	95,2%
18152	60	35	45	35	81,2%	91,6%	98,1%	98,6%	95,5%
18153	60	35	45	40	81,5%	91,5%	98,1%	98,7%	95,7%
18154	60	35	45	45	81,8%	91,4%	98,2%	98,8%	96,0%
18155	60	35	45	50	82,0%	91,3%	98,2%	98,8%	96,3%
18156	60	35	45	55	82,3%	91,2%	98,3%	98,9%	96,5%
18157	60	35	45	60	82,5%	91,1%	98,4%	99,0%	96,7%
18158	60	35	45	65	82,8%	91,0%	98,4%	99,1%	96,9%
18159	60	35	45	70	83,0%	90,9%	98,5%	99,1%	97,1%
18160	60	35	45	75	83,3%	90,8%	98,5%	99,2%	97,3%
18161	60	35	45	80	83,5%	90,7%	98,6%	99,2%	97,5%
18162	60	35	50	20	76,2%	90,0%	97,6%	98,2%	94,3%
18163	60	35	50	25	76,6%	89,9%	97,7%	98,3%	94,6%
18164	60	35	50	30	76,9%	89,8%	97,7%	98,4%	95,0%
18165	60	35	50	35	77,2%	89,7%	97,8%	98,5%	95,3%
18166	60	35	50	40	77,5%	89,6%	97,9%	98,6%	95,6%
18167	60	35	50	45	77,8%	89,4%	98,0%	98,7%	95,9%
18168	60	35	50	50	78,1%	89,3%	98,0%	98,8%	96,1%
18169	60	35	50	55	78,4%	89,2%	98,1%	98,9%	96,4%
18170	60	35	50	60	78,7%	89,1%	98,1%	98,9%	96,6%
18171	60	35	50	65	79,0%	88,9%	98,2%	99,0%	96,8%
18172	60	35	50	70	79,3%	88,8%	98,3%	99,1%	97,0%
18173	60	35	50	75	79,6%	88,7%	98,3%	99,1%	97,2%
18174	60	35	50	80	79,9%	88,5%	98,4%	99,2%	97,4%
18175	60	35	55	20	71,5%	87,8%	97,3%	98,0%	94,0%
18176	60	35	55	25	71,8%	87,6%	97,4%	98,2%	94,4%
18177	60	35	55	30	72,2%	87,5%	97,4%	98,3%	94,8%
18178	60	35	55	35	72,5%	87,3%	97,5%	98,4%	95,1%
18179	60	35	55	40	72,9%	87,2%	97,6%	98,5%	95,4%
18180	60	35	55	45	73,2%	87,1%	97,7%	98,6%	95,7%
18181	60	35	55	50	73,6%	86,9%	97,8%	98,7%	96,0%
18182	60	35	55	55	73,9%	86,8%	97,8%	98,8%	96,2%

18183	60	35	55	60	74,3%	86,6%	97,9%	98,9%	96,5%
18184	60	35	55	65	74,6%	86,5%	98,0%	98,9%	96,7%
18185	60	35	55	70	74,9%	86,3%	98,0%	99,0%	96,9%
18186	60	35	55	75	75,3%	86,1%	98,1%	99,1%	97,1%
18187	60	35	55	80	75,6%	86,0%	98,2%	99,1%	97,3%
18188	60	35	60	20	66,2%	85,1%	96,9%	97,9%	93,8%
18189	60	35	60	25	66,6%	84,9%	97,0%	98,1%	94,2%
18190	60	35	60	30	67,0%	84,7%	97,1%	98,2%	94,5%
18191	60	35	60	35	67,4%	84,6%	97,2%	98,3%	94,9%
18192	60	35	60	40	67,7%	84,4%	97,3%	98,4%	95,2%
18193	60	35	60	45	68,1%	84,2%	97,4%	98,5%	95,5%
18194	60	35	60	50	68,5%	84,0%	97,5%	98,6%	95,8%
18195	60	35	60	55	68,9%	83,9%	97,6%	98,7%	96,1%
18196	60	35	60	60	69,3%	83,7%	97,6%	98,8%	96,3%
18197	60	35	60	65	69,6%	83,5%	97,7%	98,9%	96,6%
18198	60	35	60	70	70,0%	83,3%	97,8%	99,0%	96,8%
18199	60	35	60	75	70,4%	83,2%	97,9%	99,0%	97,0%
18200	60	35	60	80	70,7%	83,0%	97,9%	99,1%	97,2%
18201	60	35	65	20	60,5%	81,9%	96,5%	97,8%	93,6%
18202	60	35	65	25	60,9%	81,7%	96,6%	97,9%	94,0%
18203	60	35	65	30	61,3%	81,5%	96,7%	98,1%	94,3%
18204	60	35	65	35	61,7%	81,3%	96,9%	98,2%	94,7%
18205	60	35	65	40	62,1%	81,1%	97,0%	98,3%	95,0%
18206	60	35	65	45	62,5%	80,9%	97,1%	98,4%	95,3%
18207	60	35	65	50	63,0%	80,7%	97,2%	98,5%	95,6%
18208	60	35	65	55	63,4%	80,5%	97,2%	98,6%	95,9%
18209	60	35	65	60	63,8%	80,3%	97,3%	98,7%	96,2%
18210	60	35	65	65	64,2%	80,1%	97,4%	98,8%	96,4%
18211	60	35	65	70	64,6%	79,9%	97,5%	98,9%	96,6%
18212	60	35	65	75	65,0%	79,7%	97,6%	99,0%	96,9%
18213	60	35	65	80	65,4%	79,5%	97,7%	99,0%	97,1%
18214	60	35	70	20	54,4%	78,2%	96,1%	97,7%	93,3%
18215	60	35	70	25	54,9%	78,0%	96,2%	97,8%	93,7%

18216	60	35	70	30	55,3%	77,8%	96,3%	98,0%	94,1%
18217	60	35	70	35	55,7%	77,5%	96,5%	98,1%	94,5%
18218	60	35	70	40	56,2%	77,3%	96,6%	98,2%	94,8%
18219	60	35	70	45	56,6%	77,1%	96,7%	98,3%	95,1%
18220	60	35	70	50	57,0%	76,9%	96,8%	98,4%	95,5%
18221	60	35	70	55	57,5%	76,6%	96,9%	98,5%	95,7%
18222	60	35	70	60	57,9%	76,4%	97,0%	98,6%	96,0%
18223	60	35	70	65	58,3%	76,1%	97,1%	98,7%	96,3%
18224	60	35	70	70	58,8%	75,9%	97,2%	98,8%	96,5%
18225	60	35	70	75	59,2%	75,7%	97,3%	98,9%	96,7%
18226	60	35	70	80	59,6%	75,4%	97,4%	99,0%	96,9%
18227	60	35	75	20	48,3%	74,0%	95,6%	97,5%	93,0%
18228	60	35	75	25	48,7%	73,8%	95,7%	97,7%	93,5%
18229	60	35	75	30	49,2%	73,5%	95,9%	97,8%	93,9%
18230	60	35	75	35	49,6%	73,3%	96,0%	98,0%	94,3%
18231	60	35	75	40	50,0%	73,0%	96,1%	98,1%	94,6%
18232	60	35	75	45	50,5%	72,8%	96,3%	98,2%	95,0%
18233	60	35	75	50	50,9%	72,5%	96,4%	98,3%	95,3%
18234	60	35	75	55	51,4%	72,2%	96,5%	98,5%	95,6%
18235	60	35	75	60	51,8%	72,0%	96,6%	98,6%	95,9%
18236	60	35	75	65	52,2%	71,7%	96,7%	98,7%	96,1%
18237	60	35	75	70	52,7%	71,4%	96,8%	98,7%	96,4%
18238	60	35	75	75	53,1%	71,2%	96,9%	98,8%	96,6%
18239	60	35	75	80	53,6%	70,9%	97,0%	98,9%	96,8%
18240	60	35	80	20	42,2%	69,4%	95,0%	97,4%	92,8%
18241	60	35	80	25	42,6%	69,1%	95,2%	97,5%	93,2%
18242	60	35	80	30	43,0%	68,8%	95,4%	97,7%	93,6%
18243	60	35	80	35	43,5%	68,5%	95,5%	97,9%	94,0%
18244	60	35	80	40	43,9%	68,2%	95,6%	98,0%	94,4%
18245	60	35	80	45	44,3%	67,9%	95,8%	98,1%	94,8%
18246	60	35	80	50	44,8%	67,7%	95,9%	98,2%	95,1%
18247	60	35	80	55	45,2%	67,4%	96,0%	98,4%	95,4%
18248	60	35	80	60	45,6%	67,1%	96,2%	98,5%	95,7%

18249	60	35	80	65	46,1%	66,8%	96,3%	98,6%	96,0%
18250	60	35	80	70	46,5%	66,5%	96,4%	98,7%	96,2%
18251	60	35	80	75	47,0%	66,2%	96,5%	98,8%	96,5%
18252	60	35	80	80	47,4%	65,9%	96,6%	98,8%	96,7%
18253	60	40	20	20	93,9%	97,5%	98,8%	98,6%	95,0%
18254	60	40	20	25	94,0%	97,5%	98,8%	98,7%	95,3%
18255	60	40	20	30	94,1%	97,4%	98,9%	98,8%	95,6%
18256	60	40	20	35	94,2%	97,4%	98,9%	98,9%	95,9%
18257	60	40	20	40	94,3%	97,4%	99,0%	99,0%	96,2%
18258	60	40	20	45	94,4%	97,3%	99,0%	99,0%	96,4%
18259	60	40	20	50	94,5%	97,3%	99,0%	99,1%	96,6%
18260	60	40	20	55	94,6%	97,3%	99,1%	99,1%	96,9%
18261	60	40	20	60	94,7%	97,2%	99,1%	99,2%	97,1%
18262	60	40	20	65	94,8%	97,2%	99,1%	99,3%	97,3%
18263	60	40	20	70	94,8%	97,2%	99,1%	99,3%	97,4%
18264	60	40	20	75	94,9%	97,1%	99,2%	99,3%	97,6%
18265	60	40	20	80	95,0%	97,1%	99,2%	99,4%	97,8%
18266	60	40	25	20	92,3%	96,9%	98,6%	98,5%	94,8%
18267	60	40	25	25	92,5%	96,8%	98,7%	98,6%	95,2%
18268	60	40	25	30	92,6%	96,8%	98,7%	98,7%	95,5%
18269	60	40	25	35	92,7%	96,8%	98,8%	98,8%	95,7%
18270	60	40	25	40	92,8%	96,7%	98,8%	98,9%	96,0%
18271	60	40	25	45	92,9%	96,7%	98,9%	99,0%	96,3%
18272	60	40	25	50	93,1%	96,6%	98,9%	99,0%	96,5%
18273	60	40	25	55	93,2%	96,6%	98,9%	99,1%	96,7%
18274	60	40	25	60	93,3%	96,5%	99,0%	99,2%	96,9%
18275	60	40	25	65	93,4%	96,5%	99,0%	99,2%	97,1%
18276	60	40	25	70	93,5%	96,5%	99,0%	99,3%	97,3%
18277	60	40	25	75	93,6%	96,4%	99,1%	99,3%	97,5%
18278	60	40	25	80	93,7%	96,4%	99,1%	99,4%	97,7%
18279	60	40	30	20	90,4%	96,1%	98,5%	98,4%	94,6%
18280	60	40	30	25	90,5%	96,1%	98,5%	98,5%	95,0%
18281	60	40	30	30	90,7%	96,0%	98,6%	98,6%	95,3%

18282	60	40	30	35	90,8%	96,0%	98,6%	98,7%	95,6%
18283	60	40	30	40	91,0%	95,9%	98,7%	98,8%	95,9%
18284	60	40	30	45	91,1%	95,8%	98,7%	98,9%	96,1%
18285	60	40	30	50	91,3%	95,8%	98,7%	99,0%	96,4%
18286	60	40	30	55	91,4%	95,7%	98,8%	99,0%	96,6%
18287	60	40	30	60	91,6%	95,7%	98,8%	99,1%	96,8%
18288	60	40	30	65	91,7%	95,6%	98,9%	99,2%	97,0%
18289	60	40	30	70	91,8%	95,6%	98,9%	99,2%	97,2%
18290	60	40	30	75	92,0%	95,5%	98,9%	99,3%	97,4%
18291	60	40	30	80	92,1%	95,5%	99,0%	99,3%	97,6%
18292	60	40	35	20	88,0%	95,1%	98,3%	98,3%	94,4%
18293	60	40	35	25	88,2%	95,1%	98,3%	98,5%	94,8%
18294	60	40	35	30	88,4%	95,0%	98,4%	98,6%	95,1%
18295	60	40	35	35	88,6%	95,0%	98,4%	98,7%	95,4%
18296	60	40	35	40	88,7%	94,9%	98,5%	98,7%	95,7%
18297	60	40	35	45	88,9%	94,8%	98,5%	98,8%	96,0%
18298	60	40	35	50	89,1%	94,8%	98,6%	98,9%	96,2%
18299	60	40	35	55	89,3%	94,7%	98,6%	99,0%	96,5%
18300	60	40	35	60	89,4%	94,6%	98,7%	99,0%	96,7%
18301	60	40	35	65	89,6%	94,6%	98,7%	99,1%	96,9%
18302	60	40	35	70	89,8%	94,5%	98,8%	99,2%	97,1%
18303	60	40	35	75	89,9%	94,4%	98,8%	99,2%	97,3%
18304	60	40	35	80	90,1%	94,4%	98,8%	99,3%	97,5%
18305	60	40	40	20	85,2%	94,0%	98,0%	98,2%	94,2%
18306	60	40	40	25	85,4%	93,9%	98,1%	98,4%	94,6%
18307	60	40	40	30	85,6%	93,8%	98,2%	98,5%	94,9%
18308	60	40	40	35	85,8%	93,7%	98,2%	98,6%	95,2%
18309	60	40	40	40	86,0%	93,6%	98,3%	98,7%	95,5%
18310	60	40	40	45	86,2%	93,6%	98,3%	98,8%	95,8%
18311	60	40	40	50	86,5%	93,5%	98,4%	98,8%	96,1%
18312	60	40	40	55	86,7%	93,4%	98,5%	98,9%	96,3%
18313	60	40	40	60	86,9%	93,3%	98,5%	99,0%	96,6%
18314	60	40	40	65	87,1%	93,2%	98,6%	99,0%	96,8%

18315	60	40	40	70	87,3%	93,2%	98,6%	99,1%	97,0%
18316	60	40	40	75	87,5%	93,1%	98,6%	99,2%	97,2%
18317	60	40	40	80	87,6%	93,0%	98,7%	99,2%	97,4%
18318	60	40	45	20	81,8%	92,5%	97,8%	98,1%	94,0%
18319	60	40	45	25	82,0%	92,4%	97,9%	98,3%	94,3%
18320	60	40	45	30	82,3%	92,3%	97,9%	98,4%	94,7%
18321	60	40	45	35	82,5%	92,2%	98,0%	98,5%	95,0%
18322	60	40	45	40	82,8%	92,1%	98,1%	98,6%	95,3%
18323	60	40	45	45	83,1%	92,0%	98,1%	98,7%	95,6%
18324	60	40	45	50	83,3%	91,9%	98,2%	98,8%	95,9%
18325	60	40	45	55	83,5%	91,8%	98,3%	98,8%	96,2%
18326	60	40	45	60	83,8%	91,7%	98,3%	98,9%	96,4%
18327	60	40	45	65	84,0%	91,6%	98,4%	99,0%	96,6%
18328	60	40	45	70	84,3%	91,5%	98,4%	99,1%	96,9%
18329	60	40	45	75	84,5%	91,4%	98,5%	99,1%	97,1%
18330	60	40	45	80	84,7%	91,3%	98,5%	99,2%	97,3%
18331	60	40	50	20	77,8%	90,7%	97,5%	98,0%	93,7%
18332	60	40	50	25	78,1%	90,6%	97,6%	98,2%	94,1%
18333	60	40	50	30	78,4%	90,5%	97,7%	98,3%	94,5%
18334	60	40	50	35	78,7%	90,4%	97,7%	98,4%	94,8%
18335	60	40	50	40	79,0%	90,3%	97,8%	98,5%	95,2%
18336	60	40	50	45	79,3%	90,2%	97,9%	98,6%	95,5%
18337	60	40	50	50	79,6%	90,0%	98,0%	98,7%	95,7%
18338	60	40	50	55	79,9%	89,9%	98,0%	98,8%	96,0%
18339	60	40	50	60	80,1%	89,8%	98,1%	98,9%	96,3%
18340	60	40	50	65	80,4%	89,7%	98,2%	98,9%	96,5%
18341	60	40	50	70	80,7%	89,6%	98,2%	99,0%	96,7%
18342	60	40	50	75	81,0%	89,4%	98,3%	99,1%	96,9%
18343	60	40	50	80	81,2%	89,3%	98,3%	99,1%	97,1%
18344	60	40	55	20	73,2%	88,6%	97,2%	97,9%	93,5%
18345	60	40	55	25	73,6%	88,5%	97,3%	98,0%	93,9%
18346	60	40	55	30	73,9%	88,3%	97,4%	98,2%	94,3%
18347	60	40	55	35	74,3%	88,2%	97,5%	98,3%	94,6%

18348	60	40	55	40	74,6%	88,1%	97,5%	98,4%	95,0%
18349	60	40	55	45	74,9%	87,9%	97,6%	98,5%	95,3%
18350	60	40	55	50	75,3%	87,8%	97,7%	98,6%	95,6%
18351	60	40	55	55	75,6%	87,6%	97,8%	98,7%	95,9%
18352	60	40	55	60	75,9%	87,5%	97,8%	98,8%	96,1%
18353	60	40	55	65	76,2%	87,4%	97,9%	98,9%	96,4%
18354	60	40	55	70	76,6%	87,2%	98,0%	98,9%	96,6%
18355	60	40	55	75	76,9%	87,1%	98,0%	99,0%	96,8%
18356	60	40	55	80	77,2%	86,9%	98,1%	99,1%	97,0%
18357	60	40	60	20	68,1%	86,0%	96,8%	97,8%	93,2%
18358	60	40	60	25	68,5%	85,9%	96,9%	97,9%	93,6%
18359	60	40	60	30	68,9%	85,7%	97,0%	98,1%	94,0%
18360	60	40	60	35	69,3%	85,6%	97,1%	98,2%	94,4%
18361	60	40	60	40	69,7%	85,4%	97,2%	98,3%	94,8%
18362	60	40	60	45	70,0%	85,2%	97,3%	98,4%	95,1%
18363	60	40	60	50	70,4%	85,1%	97,4%	98,5%	95,4%
18364	60	40	60	55	70,8%	84,9%	97,5%	98,6%	95,7%
18365	60	40	60	60	71,1%	84,7%	97,6%	98,7%	96,0%
18366	60	40	60	65	71,5%	84,6%	97,6%	98,8%	96,2%
18367	60	40	60	70	71,8%	84,4%	97,7%	98,9%	96,5%
18368	60	40	60	75	72,2%	84,2%	97,8%	98,9%	96,7%
18369	60	40	60	80	72,6%	84,1%	97,9%	99,0%	96,9%
18370	60	40	65	20	62,6%	83,0%	96,4%	97,6%	93,0%
18371	60	40	65	25	63,0%	82,9%	96,5%	97,8%	93,4%
18372	60	40	65	30	63,4%	82,7%	96,6%	97,9%	93,8%
18373	60	40	65	35	63,8%	82,5%	96,8%	98,1%	94,2%
18374	60	40	65	40	64,2%	82,3%	96,9%	98,2%	94,5%
18375	60	40	65	45	64,6%	82,1%	97,0%	98,3%	94,9%
18376	60	40	65	50	65,0%	81,9%	97,1%	98,4%	95,2%
18377	60	40	65	55	65,4%	81,7%	97,2%	98,5%	95,5%
18378	60	40	65	60	65,8%	81,5%	97,2%	98,6%	95,8%
18379	60	40	65	65	66,2%	81,3%	97,3%	98,7%	96,1%
18380	60	40	65	70	66,6%	81,1%	97,4%	98,8%	96,3%

18381	60	40	65	75	67,0%	80,9%	97,5%	98,9%	96,6%
18382	60	40	65	80	67,4%	80,7%	97,6%	99,0%	96,8%
18383	60	40	70	20	56,6%	79,5%	96,0%	97,5%	92,7%
18384	60	40	70	25	57,1%	79,3%	96,1%	97,7%	93,1%
18385	60	40	70	30	57,5%	79,1%	96,2%	97,8%	93,6%
18386	60	40	70	35	57,9%	78,9%	96,3%	98,0%	94,0%
18387	60	40	70	40	58,3%	78,7%	96,5%	98,1%	94,3%
18388	60	40	70	45	58,8%	78,4%	96,6%	98,2%	94,7%
18389	60	40	70	50	59,2%	78,2%	96,7%	98,3%	95,0%
18390	60	40	70	55	59,6%	78,0%	96,8%	98,4%	95,3%
18391	60	40	70	60	60,1%	77,8%	96,9%	98,5%	95,6%
18392	60	40	70	65	60,5%	77,5%	97,0%	98,6%	95,9%
18393	60	40	70	70	60,9%	77,3%	97,1%	98,7%	96,2%
18394	60	40	70	75	61,3%	77,1%	97,2%	98,8%	96,4%
18395	60	40	70	80	61,7%	76,9%	97,3%	98,9%	96,6%
18396	60	40	75	20	50,5%	75,5%	95,5%	97,3%	92,4%
18397	60	40	75	25	50,9%	75,3%	95,6%	97,5%	92,9%
18398	60	40	75	30	51,4%	75,0%	95,7%	97,7%	93,3%
18399	60	40	75	35	51,8%	74,8%	95,9%	97,8%	93,7%
18400	60	40	75	40	52,3%	74,5%	96,0%	98,0%	94,1%
18401	60	40	75	45	52,7%	74,3%	96,1%	98,1%	94,5%
18402	60	40	75	50	53,1%	74,0%	96,3%	98,2%	94,8%
18403	60	40	75	55	53,6%	73,8%	96,4%	98,3%	95,1%
18404	60	40	75	60	54,0%	73,5%	96,5%	98,4%	95,5%
18405	60	40	75	65	54,4%	73,3%	96,6%	98,6%	95,7%
18406	60	40	75	70	54,9%	73,0%	96,7%	98,6%	96,0%
18407	60	40	75	75	55,3%	72,8%	96,8%	98,7%	96,3%
18408	60	40	75	80	55,8%	72,5%	96,9%	98,8%	96,5%
18409	60	40	80	20	44,3%	71,0%	94,9%	97,2%	92,1%
18410	60	40	80	25	44,8%	70,7%	95,1%	97,4%	92,6%
18411	60	40	80	30	45,2%	70,5%	95,2%	97,5%	93,0%
18412	60	40	80	35	45,7%	70,2%	95,4%	97,7%	93,5%
18413	60	40	80	40	46,1%	69,9%	95,5%	97,8%	93,9%

18414	60	40	80	45	46,5%	69,6%	95,7%	98,0%	94,3%
18415	60	40	80	50	47,0%	69,4%	95,8%	98,1%	94,6%
18416	60	40	80	55	47,4%	69,1%	95,9%	98,2%	95,0%
18417	60	40	80	60	47,8%	68,8%	96,1%	98,4%	95,3%
18418	60	40	80	65	48,3%	68,5%	96,2%	98,5%	95,6%
18419	60	40	80	70	48,7%	68,2%	96,3%	98,6%	95,9%
18420	60	40	80	75	49,2%	67,9%	96,4%	98,7%	96,1%
18421	60	40	80	80	49,6%	67,7%	96,5%	98,7%	96,4%
18422	60	45	20	20	94,4%	97,7%	98,8%	98,5%	94,6%
18423	60	45	20	25	94,5%	97,7%	98,8%	98,6%	94,9%
18424	60	45	20	30	94,6%	97,6%	98,8%	98,7%	95,2%
18425	60	45	20	35	94,7%	97,6%	98,9%	98,8%	95,5%
18426	60	45	20	40	94,8%	97,6%	98,9%	98,9%	95,8%
18427	60	45	20	45	94,8%	97,5%	99,0%	98,9%	96,1%
18428	60	45	20	50	94,9%	97,5%	99,0%	99,0%	96,3%
18429	60	45	20	55	95,0%	97,5%	99,0%	99,1%	96,6%
18430	60	45	20	60	95,1%	97,4%	99,1%	99,1%	96,8%
18431	60	45	20	65	95,2%	97,4%	99,1%	99,2%	97,0%
18432	60	45	20	70	95,3%	97,4%	99,1%	99,3%	97,2%
18433	60	45	20	75	95,3%	97,3%	99,1%	99,3%	97,4%
18434	60	45	20	80	95,4%	97,3%	99,2%	99,3%	97,5%
18435	60	45	25	20	92,9%	97,1%	98,6%	98,4%	94,3%
18436	60	45	25	25	93,1%	97,1%	98,6%	98,5%	94,7%
18437	60	45	25	30	93,2%	97,0%	98,7%	98,6%	95,0%
18438	60	45	25	35	93,3%	97,0%	98,7%	98,7%	95,3%
18439	60	45	25	40	93,4%	97,0%	98,8%	98,8%	95,6%
18440	60	45	25	45	93,5%	96,9%	98,8%	98,9%	95,9%
18441	60	45	25	50	93,6%	96,9%	98,9%	99,0%	96,2%
18442	60	45	25	55	93,7%	96,8%	98,9%	99,0%	96,4%
18443	60	45	25	60	93,8%	96,8%	98,9%	99,1%	96,6%
18444	60	45	25	65	93,9%	96,8%	99,0%	99,1%	96,9%
18445	60	45	25	70	94,0%	96,7%	99,0%	99,2%	97,1%
18446	60	45	25	75	94,1%	96,7%	99,0%	99,3%	97,3%

18447	60	45	25	80	94,2%	96,6%	99,1%	99,3%	97,4%
18448	60	45	30	20	91,1%	96,4%	98,4%	98,3%	94,1%
18449	60	45	30	25	91,3%	96,3%	98,5%	98,4%	94,5%
18450	60	45	30	30	91,4%	96,3%	98,5%	98,5%	94,8%
18451	60	45	30	35	91,6%	96,2%	98,6%	98,6%	95,2%
18452	60	45	30	40	91,7%	96,2%	98,6%	98,7%	95,5%
18453	60	45	30	45	91,8%	96,2%	98,7%	98,8%	95,7%
18454	60	45	30	50	92,0%	96,1%	98,7%	98,9%	96,0%
18455	60	45	30	55	92,1%	96,1%	98,7%	99,0%	96,3%
18456	60	45	30	60	92,2%	96,0%	98,8%	99,0%	96,5%
18457	60	45	30	65	92,3%	96,0%	98,8%	99,1%	96,7%
18458	60	45	30	70	92,5%	95,9%	98,9%	99,2%	96,9%
18459	60	45	30	75	92,6%	95,8%	98,9%	99,2%	97,1%
18460	60	45	30	80	92,7%	95,8%	98,9%	99,3%	97,3%
18461	60	45	35	20	88,9%	95,5%	98,2%	98,2%	93,9%
18462	60	45	35	25	89,1%	95,4%	98,3%	98,3%	94,3%
18463	60	45	35	30	89,3%	95,4%	98,3%	98,4%	94,6%
18464	60	45	35	35	89,4%	95,3%	98,4%	98,6%	95,0%
18465	60	45	35	40	89,6%	95,3%	98,4%	98,6%	95,3%
18466	60	45	35	45	89,8%	95,2%	98,5%	98,7%	95,6%
18467	60	45	35	50	89,9%	95,1%	98,5%	98,8%	95,9%
18468	60	45	35	55	90,1%	95,1%	98,6%	98,9%	96,1%
18469	60	45	35	60	90,2%	95,0%	98,6%	99,0%	96,4%
18470	60	45	35	65	90,4%	95,0%	98,7%	99,0%	96,6%
18471	60	45	35	70	90,6%	94,9%	98,7%	99,1%	96,8%
18472	60	45	35	75	90,7%	94,8%	98,8%	99,2%	97,0%
18473	60	45	35	80	90,8%	94,8%	98,8%	99,2%	97,2%
18474	60	45	40	20	86,3%	94,4%	98,0%	98,1%	93,6%
18475	60	45	40	25	86,5%	94,3%	98,0%	98,2%	94,0%
18476	60	45	40	30	86,7%	94,2%	98,1%	98,4%	94,4%
18477	60	45	40	35	86,9%	94,2%	98,2%	98,5%	94,8%
18478	60	45	40	40	87,1%	94,1%	98,2%	98,6%	95,1%
18479	60	45	40	45	87,3%	94,0%	98,3%	98,7%	95,4%

18480	60	45	40	50	87,5%	94,0%	98,3%	98,7%	95,7%
18481	60	45	40	55	87,7%	93,9%	98,4%	98,8%	96,0%
18482	60	45	40	60	87,8%	93,8%	98,5%	98,9%	96,2%
18483	60	45	40	65	88,0%	93,7%	98,5%	99,0%	96,5%
18484	60	45	40	70	88,2%	93,6%	98,6%	99,0%	96,7%
18485	60	45	40	75	88,4%	93,6%	98,6%	99,1%	96,9%
18486	60	45	40	80	88,6%	93,5%	98,6%	99,2%	97,1%
18487	60	45	45	20	83,1%	93,0%	97,7%	98,0%	93,4%
18488	60	45	45	25	83,3%	92,9%	97,8%	98,1%	93,8%
18489	60	45	45	30	83,5%	92,9%	97,9%	98,3%	94,2%
18490	60	45	45	35	83,8%	92,8%	97,9%	98,4%	94,6%
18491	60	45	45	40	84,0%	92,7%	98,0%	98,5%	94,9%
18492	60	45	45	45	84,3%	92,6%	98,1%	98,6%	95,2%
18493	60	45	45	50	84,5%	92,5%	98,1%	98,7%	95,5%
18494	60	45	45	55	84,7%	92,4%	98,2%	98,8%	95,8%
18495	60	45	45	60	85,0%	92,3%	98,3%	98,8%	96,1%
18496	60	45	45	65	85,2%	92,2%	98,3%	98,9%	96,3%
18497	60	45	45	70	85,4%	92,1%	98,4%	99,0%	96,6%
18498	60	45	45	75	85,6%	92,0%	98,4%	99,1%	96,8%
18499	60	45	45	80	85,8%	91,9%	98,5%	99,1%	97,0%
18500	60	45	50	20	79,3%	91,4%	97,4%	97,9%	93,1%
18501	60	45	50	25	79,6%	91,3%	97,5%	98,0%	93,6%
18502	60	45	50	30	79,9%	91,2%	97,6%	98,1%	94,0%
18503	60	45	50	35	80,2%	91,1%	97,7%	98,3%	94,3%
18504	60	45	50	40	80,4%	90,9%	97,7%	98,4%	94,7%
18505	60	45	50	45	80,7%	90,8%	97,8%	98,5%	95,0%
18506	60	45	50	50	81,0%	90,7%	97,9%	98,6%	95,3%
18507	60	45	50	55	81,2%	90,6%	98,0%	98,7%	95,6%
18508	60	45	50	60	81,5%	90,5%	98,0%	98,8%	95,9%
18509	60	45	50	65	81,8%	90,4%	98,1%	98,8%	96,2%
18510	60	45	50	70	82,0%	90,3%	98,2%	98,9%	96,4%
18511	60	45	50	75	82,3%	90,2%	98,2%	99,0%	96,6%
18512	60	45	50	80	82,6%	90,0%	98,3%	99,1%	96,9%

18513	60	45	55	20	75,0%	89,4%	97,1%	97,7%	92,9%
18514	60	45	55	25	75,3%	89,2%	97,2%	97,9%	93,3%
18515	60	45	55	30	75,6%	89,1%	97,3%	98,0%	93,7%
18516	60	45	55	35	75,9%	89,0%	97,4%	98,2%	94,1%
18517	60	45	55	40	76,3%	88,9%	97,5%	98,3%	94,5%
18518	60	45	55	45	76,6%	88,7%	97,5%	98,4%	94,8%
18519	60	45	55	50	76,9%	88,6%	97,6%	98,5%	95,2%
18520	60	45	55	55	77,2%	88,5%	97,7%	98,6%	95,5%
18521	60	45	55	60	77,5%	88,3%	97,8%	98,7%	95,7%
18522	60	45	55	65	77,8%	88,2%	97,8%	98,8%	96,0%
18523	60	45	55	70	78,1%	88,1%	97,9%	98,9%	96,3%
18524	60	45	55	75	78,4%	87,9%	98,0%	98,9%	96,5%
18525	60	45	55	80	78,7%	87,8%	98,1%	99,0%	96,7%
18526	60	45	60	20	70,0%	87,0%	96,7%	97,6%	92,6%
18527	60	45	60	25	70,4%	86,8%	96,8%	97,8%	93,0%
18528	60	45	60	30	70,8%	86,7%	96,9%	97,9%	93,5%
18529	60	45	60	35	71,1%	86,5%	97,0%	98,0%	93,9%
18530	60	45	60	40	71,5%	86,4%	97,1%	98,2%	94,3%
18531	60	45	60	45	71,9%	86,2%	97,2%	98,3%	94,6%
18532	60	45	60	50	72,2%	86,0%	97,3%	98,4%	95,0%
18533	60	45	60	55	72,6%	85,9%	97,4%	98,5%	95,3%
18534	60	45	60	60	72,9%	85,7%	97,5%	98,6%	95,6%
18535	60	45	60	65	73,3%	85,6%	97,6%	98,7%	95,9%
18536	60	45	60	70	73,6%	85,4%	97,6%	98,8%	96,1%
18537	60	45	60	75	73,9%	85,2%	97,7%	98,9%	96,4%
18538	60	45	60	80	74,3%	85,1%	97,8%	98,9%	96,6%
18539	60	45	65	20	64,6%	84,1%	96,3%	97,5%	92,3%
18540	60	45	65	25	65,0%	83,9%	96,4%	97,6%	92,8%
18541	60	45	65	30	65,4%	83,8%	96,5%	97,8%	93,2%
18542	60	45	65	35	65,8%	83,6%	96,7%	97,9%	93,6%
18543	60	45	65	40	66,2%	83,4%	96,8%	98,1%	94,0%
18544	60	45	65	45	66,6%	83,2%	96,9%	98,2%	94,4%
18545	60	45	65	50	67,0%	83,0%	97,0%	98,3%	94,8%

18546	60	45	65	55	67,4%	82,9%	97,1%	98,4%	95,1%
18547	60	45	65	60	67,8%	82,7%	97,2%	98,5%	95,4%
18548	60	45	65	65	68,2%	82,5%	97,3%	98,6%	95,7%
18549	60	45	65	70	68,5%	82,3%	97,3%	98,7%	96,0%
18550	60	45	65	75	68,9%	82,1%	97,4%	98,8%	96,2%
18551	60	45	65	80	69,3%	81,9%	97,5%	98,9%	96,5%
18552	60	45	70	20	58,8%	80,8%	95,8%	97,3%	92,0%
18553	60	45	70	25	59,2%	80,6%	96,0%	97,5%	92,5%
18554	60	45	70	30	59,6%	80,4%	96,1%	97,6%	93,0%
18555	60	45	70	35	60,1%	80,2%	96,2%	97,8%	93,4%
18556	60	45	70	40	60,5%	80,0%	96,4%	97,9%	93,8%
18557	60	45	70	45	60,9%	79,7%	96,5%	98,1%	94,2%
18558	60	45	70	50	61,3%	79,5%	96,6%	98,2%	94,5%
18559	60	45	70	55	61,7%	79,3%	96,7%	98,3%	94,9%
18560	60	45	70	60	62,2%	79,1%	96,8%	98,4%	95,2%
18561	60	45	70	65	62,6%	78,9%	96,9%	98,5%	95,5%
18562	60	45	70	70	63,0%	78,7%	97,0%	98,6%	95,8%
18563	60	45	70	75	63,4%	78,4%	97,1%	98,7%	96,1%
18564	60	45	70	80	63,8%	78,2%	97,2%	98,8%	96,3%
18565	60	45	75	20	52,7%	76,9%	95,3%	97,1%	91,7%
18566	60	45	75	25	53,1%	76,7%	95,5%	97,3%	92,2%
18567	60	45	75	30	53,6%	76,5%	95,6%	97,5%	92,7%
18568	60	45	75	35	54,0%	76,2%	95,8%	97,7%	93,1%
18569	60	45	75	40	54,5%	76,0%	95,9%	97,8%	93,6%
18570	60	45	75	45	54,9%	75,8%	96,0%	98,0%	94,0%
18571	60	45	75	50	55,3%	75,5%	96,1%	98,1%	94,3%
18572	60	45	75	55	55,8%	75,3%	96,3%	98,2%	94,7%
18573	60	45	75	60	56,2%	75,0%	96,4%	98,3%	95,0%
18574	60	45	75	65	56,6%	74,8%	96,5%	98,4%	95,3%
18575	60	45	75	70	57,1%	74,5%	96,6%	98,5%	95,6%
18576	60	45	75	75	57,5%	74,3%	96,7%	98,6%	95,9%
18577	60	45	75	80	57,9%	74,0%	96,8%	98,7%	96,2%
18578	60	45	80	20	46,5%	72,6%	94,7%	97,0%	91,4%

18579	60	45	80	25	47,0%	72,3%	94,9%	97,2%	91,9%
18580	60	45	80	30	47,4%	72,1%	95,1%	97,3%	92,4%
18581	60	45	80	35	47,9%	71,8%	95,2%	97,5%	92,9%
18582	60	45	80	40	48,3%	71,5%	95,4%	97,7%	93,3%
18583	60	45	80	45	48,7%	71,3%	95,5%	97,8%	93,7%
18584	60	45	80	50	49,2%	71,0%	95,7%	98,0%	94,1%
18585	60	45	80	55	49,6%	70,7%	95,8%	98,1%	94,5%
18586	60	45	80	60	50,1%	70,5%	95,9%	98,2%	94,8%
18587	60	45	80	65	50,5%	70,2%	96,1%	98,3%	95,1%
18588	60	45	80	70	50,9%	69,9%	96,2%	98,5%	95,5%
18589	60	45	80	75	51,4%	69,6%	96,3%	98,6%	95,7%
18590	60	45	80	80	51,8%	69,4%	96,4%	98,7%	96,0%
18591	60	50	20	20	94,8%	97,9%	98,7%	98,4%	94,0%
18592	60	50	20	25	94,9%	97,8%	98,8%	98,5%	94,4%
18593	60	50	20	30	95,0%	97,8%	98,8%	98,6%	94,8%
18594	60	50	20	35	95,1%	97,8%	98,8%	98,7%	95,1%
18595	60	50	20	40	95,2%	97,8%	98,9%	98,8%	95,4%
18596	60	50	20	45	95,3%	97,7%	98,9%	98,9%	95,7%
18597	60	50	20	50	95,3%	97,7%	99,0%	98,9%	96,0%
18598	60	50	20	55	95,4%	97,7%	99,0%	99,0%	96,2%
18599	60	50	20	60	95,5%	97,6%	99,0%	99,1%	96,5%
18600	60	50	20	65	95,6%	97,6%	99,1%	99,1%	96,7%
18601	60	50	20	70	95,6%	97,6%	99,1%	99,2%	96,9%
18602	60	50	20	75	95,7%	97,5%	99,1%	99,2%	97,1%
18603	60	50	20	80	95,8%	97,5%	99,1%	99,3%	97,3%
18604	60	50	25	20	93,5%	97,3%	98,6%	98,3%	93,8%
18605	60	50	25	25	93,6%	97,3%	98,6%	98,4%	94,2%
18606	60	50	25	30	93,7%	97,3%	98,7%	98,5%	94,6%
18607	60	50	25	35	93,8%	97,2%	98,7%	98,6%	94,9%
18608	60	50	25	40	93,9%	97,2%	98,7%	98,7%	95,2%
18609	60	50	25	45	94,0%	97,1%	98,8%	98,8%	95,5%
18610	60	50	25	50	94,1%	97,1%	98,8%	98,9%	95,8%
18611	60	50	25	55	94,2%	97,1%	98,9%	98,9%	96,1%

18612	60	50	25	60	94,3%	97,0%	98,9%	99,0%	96,3%
18613	60	50	25	65	94,4%	97,0%	98,9%	99,1%	96,6%
18614	60	50	25	70	94,5%	97,0%	99,0%	99,1%	96,8%
18615	60	50	25	75	94,6%	96,9%	99,0%	99,2%	97,0%
18616	60	50	25	80	94,7%	96,9%	99,0%	99,3%	97,2%
18617	60	50	30	20	91,8%	96,7%	98,4%	98,2%	93,6%
18618	60	50	30	25	92,0%	96,6%	98,4%	98,3%	94,0%
18619	60	50	30	30	92,1%	96,6%	98,5%	98,4%	94,3%
18620	60	50	30	35	92,2%	96,5%	98,5%	98,5%	94,7%
18621	60	50	30	40	92,3%	96,5%	98,6%	98,6%	95,0%
18622	60	50	30	45	92,5%	96,4%	98,6%	98,7%	95,3%
18623	60	50	30	50	92,6%	96,4%	98,7%	98,8%	95,6%
18624	60	50	30	55	92,7%	96,3%	98,7%	98,9%	95,9%
18625	60	50	30	60	92,8%	96,3%	98,8%	99,0%	96,2%
18626	60	50	30	65	92,9%	96,2%	98,8%	99,0%	96,4%
18627	60	50	30	70	93,1%	96,2%	98,8%	99,1%	96,6%
18628	60	50	30	75	93,2%	96,2%	98,9%	99,1%	96,9%
18629	60	50	30	80	93,3%	96,1%	98,9%	99,2%	97,1%
18630	60	50	35	20	89,8%	95,8%	98,2%	98,1%	93,3%
18631	60	50	35	25	89,9%	95,8%	98,2%	98,2%	93,7%
18632	60	50	35	30	90,1%	95,7%	98,3%	98,3%	94,1%
18633	60	50	35	35	90,2%	95,7%	98,3%	98,4%	94,5%
18634	60	50	35	40	90,4%	95,6%	98,4%	98,5%	94,8%
18635	60	50	35	45	90,6%	95,5%	98,4%	98,6%	95,2%
18636	60	50	35	50	90,7%	95,5%	98,5%	98,7%	95,5%
18637	60	50	35	55	90,9%	95,4%	98,5%	98,8%	95,8%
18638	60	50	35	60	91,0%	95,4%	98,6%	98,9%	96,0%
18639	60	50	35	65	91,1%	95,3%	98,6%	99,0%	96,3%
18640	60	50	35	70	91,3%	95,3%	98,7%	99,0%	96,5%
18641	60	50	35	75	91,4%	95,2%	98,7%	99,1%	96,7%
18642	60	50	35	80	91,6%	95,1%	98,8%	99,2%	96,9%
18643	60	50	40	20	87,3%	94,8%	97,9%	98,0%	93,0%
18644	60	50	40	25	87,5%	94,7%	98,0%	98,1%	93,5%

18645	60	50	40	30	87,7%	94,7%	98,1%	98,2%	93,9%
18646	60	50	40	35	87,9%	94,6%	98,1%	98,3%	94,3%
18647	60	50	40	40	88,0%	94,5%	98,2%	98,5%	94,6%
18648	60	50	40	45	88,2%	94,5%	98,2%	98,6%	95,0%
18649	60	50	40	50	88,4%	94,4%	98,3%	98,7%	95,3%
18650	60	50	40	55	88,6%	94,3%	98,4%	98,7%	95,6%
18651	60	50	40	60	88,8%	94,2%	98,4%	98,8%	95,9%
18652	60	50	40	65	88,9%	94,2%	98,5%	98,9%	96,1%
18653	60	50	40	70	89,1%	94,1%	98,5%	99,0%	96,4%
18654	60	50	40	75	89,3%	94,0%	98,6%	99,0%	96,6%
18655	60	50	40	80	89,4%	94,0%	98,6%	99,1%	96,8%
18656	60	50	45	20	84,3%	93,5%	97,7%	97,8%	92,8%
18657	60	50	45	25	84,5%	93,4%	97,7%	98,0%	93,2%
18658	60	50	45	30	84,7%	93,4%	97,8%	98,1%	93,6%
18659	60	50	45	35	85,0%	93,3%	97,9%	98,2%	94,0%
18660	60	50	45	40	85,2%	93,2%	97,9%	98,4%	94,4%
18661	60	50	45	45	85,4%	93,1%	98,0%	98,5%	94,8%
18662	60	50	45	50	85,6%	93,0%	98,1%	98,6%	95,1%
18663	60	50	45	55	85,8%	92,9%	98,1%	98,7%	95,4%
18664	60	50	45	60	86,1%	92,9%	98,2%	98,7%	95,7%
18665	60	50	45	65	86,3%	92,8%	98,3%	98,8%	96,0%
18666	60	50	45	70	86,5%	92,7%	98,3%	98,9%	96,2%
18667	60	50	45	75	86,7%	92,6%	98,4%	99,0%	96,5%
18668	60	50	45	80	86,9%	92,5%	98,4%	99,0%	96,7%
18669	60	50	50	20	80,7%	92,0%	97,3%	97,7%	92,5%
18670	60	50	50	25	81,0%	91,9%	97,4%	97,9%	93,0%
18671	60	50	50	30	81,3%	91,8%	97,5%	98,0%	93,4%
18672	60	50	50	35	81,5%	91,7%	97,6%	98,1%	93,8%
18673	60	50	50	40	81,8%	91,6%	97,7%	98,3%	94,2%
18674	60	50	50	45	82,1%	91,5%	97,8%	98,4%	94,6%
18675	60	50	50	50	82,3%	91,4%	97,8%	98,5%	94,9%
18676	60	50	50	55	82,6%	91,3%	97,9%	98,6%	95,2%
18677	60	50	50	60	82,8%	91,2%	98,0%	98,7%	95,5%

18678	60	50	50	65	83,1%	91,1%	98,0%	98,8%	95,8%
18679	60	50	50	70	83,3%	91,0%	98,1%	98,8%	96,1%
18680	60	50	50	75	83,6%	90,8%	98,2%	98,9%	96,3%
18681	60	50	50	80	83,8%	90,7%	98,2%	99,0%	96,6%
18682	60	50	55	20	76,6%	90,1%	97,0%	97,6%	92,2%
18683	60	50	55	25	76,9%	90,0%	97,1%	97,7%	92,7%
18684	60	50	55	30	77,2%	89,9%	97,2%	97,9%	93,1%
18685	60	50	55	35	77,5%	89,7%	97,3%	98,0%	93,6%
18686	60	50	55	40	77,8%	89,6%	97,4%	98,1%	94,0%
18687	60	50	55	45	78,1%	89,5%	97,5%	98,3%	94,3%
18688	60	50	55	50	78,4%	89,4%	97,5%	98,4%	94,7%
18689	60	50	55	55	78,7%	89,2%	97,6%	98,5%	95,0%
18690	60	50	55	60	79,0%	89,1%	97,7%	98,6%	95,3%
18691	60	50	55	65	79,3%	89,0%	97,8%	98,7%	95,6%
18692	60	50	55	70	79,6%	88,9%	97,9%	98,8%	95,9%
18693	60	50	55	75	79,9%	88,7%	97,9%	98,8%	96,2%
18694	60	50	55	80	80,2%	88,6%	98,0%	98,9%	96,4%
18695	60	50	60	20	71,9%	87,8%	96,6%	97,4%	91,9%
18696	60	50	60	25	72,2%	87,7%	96,7%	97,6%	92,4%
18697	60	50	60	30	72,6%	87,5%	96,8%	97,8%	92,9%
18698	60	50	60	35	72,9%	87,4%	96,9%	97,9%	93,3%
18699	60	50	60	40	73,3%	87,3%	97,0%	98,0%	93,7%
18700	60	50	60	45	73,6%	87,1%	97,1%	98,2%	94,1%
18701	60	50	60	50	74,0%	87,0%	97,2%	98,3%	94,5%
18702	60	50	60	55	74,3%	86,8%	97,3%	98,4%	94,8%
18703	60	50	60	60	74,6%	86,7%	97,4%	98,5%	95,2%
18704	60	50	60	65	75,0%	86,5%	97,5%	98,6%	95,5%
18705	60	50	60	70	75,3%	86,4%	97,6%	98,7%	95,7%
18706	60	50	60	75	75,6%	86,2%	97,7%	98,8%	96,0%
18707	60	50	60	80	75,9%	86,1%	97,7%	98,9%	96,3%
18708	60	50	65	20	66,6%	85,1%	96,2%	97,3%	91,6%
18709	60	50	65	25	67,0%	85,0%	96,3%	97,4%	92,1%
18710	60	50	65	30	67,4%	84,8%	96,4%	97,6%	92,6%

18711	60	50	65	35	67,8%	84,6%	96,6%	97,8%	93,0%
18712	60	50	65	40	68,2%	84,5%	96,7%	97,9%	93,5%
18713	60	50	65	45	68,5%	84,3%	96,8%	98,1%	93,9%
18714	60	50	65	50	68,9%	84,1%	96,9%	98,2%	94,3%
18715	60	50	65	55	69,3%	83,9%	97,0%	98,3%	94,6%
18716	60	50	65	60	69,7%	83,8%	97,1%	98,4%	95,0%
18717	60	50	65	65	70,0%	83,6%	97,2%	98,5%	95,3%
18718	60	50	65	70	70,4%	83,4%	97,3%	98,6%	95,6%
18719	60	50	65	75	70,8%	83,2%	97,4%	98,7%	95,9%
18720	60	50	65	80	71,1%	83,0%	97,4%	98,8%	96,1%
18721	60	50	70	20	60,9%	82,0%	95,7%	97,1%	91,3%
18722	60	50	70	25	61,3%	81,8%	95,9%	97,3%	91,8%
18723	60	50	70	30	61,8%	81,6%	96,0%	97,5%	92,3%
18724	60	50	70	35	62,2%	81,4%	96,1%	97,6%	92,8%
18725	60	50	70	40	62,6%	81,2%	96,2%	97,8%	93,2%
18726	60	50	70	45	63,0%	81,0%	96,4%	97,9%	93,6%
18727	60	50	70	50	63,4%	80,8%	96,5%	98,1%	94,0%
18728	60	50	70	55	63,8%	80,6%	96,6%	98,2%	94,4%
18729	60	50	70	60	64,2%	80,4%	96,7%	98,3%	94,8%
18730	60	50	70	65	64,6%	80,2%	96,8%	98,4%	95,1%
18731	60	50	70	70	65,0%	80,0%	96,9%	98,5%	95,4%
18732	60	50	70	75	65,4%	79,8%	97,0%	98,6%	95,7%
18733	60	50	70	80	65,8%	79,5%	97,1%	98,7%	96,0%
18734	60	50	75	20	54,9%	78,3%	95,2%	96,9%	90,9%
18735	60	50	75	25	55,3%	78,1%	95,3%	97,1%	91,5%
18736	60	50	75	30	55,8%	77,9%	95,5%	97,3%	92,0%
18737	60	50	75	35	56,2%	77,6%	95,6%	97,5%	92,5%
18738	60	50	75	40	56,7%	77,4%	95,8%	97,7%	93,0%
18739	60	50	75	45	57,1%	77,2%	95,9%	97,8%	93,4%
18740	60	50	75	50	57,5%	76,9%	96,0%	97,9%	93,8%
18741	60	50	75	55	57,9%	76,7%	96,2%	98,1%	94,2%
18742	60	50	75	60	58,4%	76,5%	96,3%	98,2%	94,6%
18743	60	50	75	65	58,8%	76,2%	96,4%	98,3%	94,9%

18744	60	50	75	70	59,2%	76,0%	96,5%	98,4%	95,2%
18745	60	50	75	75	59,7%	75,8%	96,6%	98,5%	95,5%
18746	60	50	75	80	60,1%	75,5%	96,7%	98,6%	95,8%
18747	60	50	80	20	48,8%	74,1%	94,6%	96,7%	90,6%
18748	60	50	80	25	49,2%	73,9%	94,7%	97,0%	91,2%
18749	60	50	80	30	49,6%	73,6%	94,9%	97,1%	91,7%
18750	60	50	80	35	50,1%	73,4%	95,1%	97,3%	92,2%
18751	60	50	80	40	50,5%	73,1%	95,2%	97,5%	92,7%
18752	60	50	80	45	51,0%	72,9%	95,4%	97,7%	93,1%
18753	60	50	80	50	51,4%	72,6%	95,5%	97,8%	93,6%
18754	60	50	80	55	51,8%	72,3%	95,7%	98,0%	94,0%
18755	60	50	80	60	52,3%	72,1%	95,8%	98,1%	94,3%
18756	60	50	80	65	52,7%	71,8%	95,9%	98,2%	94,7%
18757	60	50	80	70	53,2%	71,5%	96,1%	98,3%	95,0%
18758	60	50	80	75	53,6%	71,3%	96,2%	98,4%	95,3%
18759	60	50	80	80	54,0%	71,0%	96,3%	98,5%	95,6%
18760	60	55	20	20	95,3%	98,0%	98,7%	98,3%	93,5%
18761	60	55	20	25	95,3%	98,0%	98,7%	98,4%	93,9%
18762	60	55	20	30	95,4%	98,0%	98,8%	98,5%	94,3%
18763	60	55	20	35	95,5%	97,9%	98,8%	98,6%	94,6%
18764	60	55	20	40	95,6%	97,9%	98,8%	98,7%	95,0%
18765	60	55	20	45	95,6%	97,9%	98,9%	98,8%	95,3%
18766	60	55	20	50	95,7%	97,9%	98,9%	98,9%	95,6%
18767	60	55	20	55	95,8%	97,8%	99,0%	98,9%	95,9%
18768	60	55	20	60	95,9%	97,8%	99,0%	99,0%	96,1%
18769	60	55	20	65	95,9%	97,8%	99,0%	99,1%	96,4%
18770	60	55	20	70	96,0%	97,8%	99,1%	99,1%	96,6%
18771	60	55	20	75	96,1%	97,7%	99,1%	99,2%	96,8%
18772	60	55	20	80	96,1%	97,7%	99,1%	99,2%	97,0%
18773	60	55	25	20	94,0%	97,5%	98,5%	98,2%	93,2%
18774	60	55	25	25	94,1%	97,5%	98,6%	98,3%	93,6%
18775	60	55	25	30	94,2%	97,5%	98,6%	98,4%	94,0%
18776	60	55	25	35	94,3%	97,4%	98,7%	98,5%	94,4%

18777	60	55	25	40	94,4%	97,4%	98,7%	98,6%	94,8%
18778	60	55	25	45	94,5%	97,4%	98,7%	98,7%	95,1%
18779	60	55	25	50	94,6%	97,3%	98,8%	98,8%	95,4%
18780	60	55	25	55	94,7%	97,3%	98,8%	98,9%	95,7%
18781	60	55	25	60	94,8%	97,3%	98,9%	98,9%	96,0%
18782	60	55	25	65	94,9%	97,2%	98,9%	99,0%	96,2%
18783	60	55	25	70	94,9%	97,2%	98,9%	99,1%	96,5%
18784	60	55	25	75	95,0%	97,1%	99,0%	99,1%	96,7%
18785	60	55	25	80	95,1%	97,1%	99,0%	99,2%	96,9%
18786	60	55	30	20	92,5%	96,9%	98,3%	98,1%	93,0%
18787	60	55	30	25	92,6%	96,9%	98,4%	98,2%	93,4%
18788	60	55	30	30	92,7%	96,8%	98,4%	98,3%	93,8%
18789	60	55	30	35	92,8%	96,8%	98,5%	98,4%	94,2%
18790	60	55	30	40	92,9%	96,7%	98,5%	98,5%	94,6%
18791	60	55	30	45	93,1%	96,7%	98,6%	98,6%	94,9%
18792	60	55	30	50	93,2%	96,7%	98,6%	98,7%	95,2%
18793	60	55	30	55	93,3%	96,6%	98,7%	98,8%	95,5%
18794	60	55	30	60	93,4%	96,6%	98,7%	98,9%	95,8%
18795	60	55	30	65	93,5%	96,5%	98,8%	99,0%	96,1%
18796	60	55	30	70	93,6%	96,5%	98,8%	99,0%	96,3%
18797	60	55	30	75	93,7%	96,4%	98,8%	99,1%	96,6%
18798	60	55	30	80	93,8%	96,4%	98,9%	99,1%	96,8%
18799	60	55	35	20	90,6%	96,1%	98,1%	97,9%	92,7%
18800	60	55	35	25	90,7%	96,1%	98,2%	98,1%	93,1%
18801	60	55	35	30	90,9%	96,0%	98,2%	98,2%	93,6%
18802	60	55	35	35	91,0%	96,0%	98,3%	98,3%	94,0%
18803	60	55	35	40	91,1%	95,9%	98,3%	98,4%	94,3%
18804	60	55	35	45	91,3%	95,9%	98,4%	98,5%	94,7%
18805	60	55	35	50	91,4%	95,8%	98,4%	98,6%	95,0%
18806	60	55	35	55	91,6%	95,8%	98,5%	98,7%	95,3%
18807	60	55	35	60	91,7%	95,7%	98,5%	98,8%	95,6%
18808	60	55	35	65	91,8%	95,7%	98,6%	98,9%	95,9%
18809	60	55	35	70	92,0%	95,6%	98,6%	99,0%	96,2%

18810	60	55	35	75	92,1%	95,5%	98,7%	99,0%	96,4%
18811	60	55	35	80	92,2%	95,5%	98,7%	99,1%	96,6%
18812	60	55	40	20	88,2%	95,2%	97,9%	97,8%	92,4%
18813	60	55	40	25	88,4%	95,1%	97,9%	98,0%	92,9%
18814	60	55	40	30	88,6%	95,0%	98,0%	98,1%	93,3%
18815	60	55	40	35	88,8%	95,0%	98,1%	98,2%	93,7%
18816	60	55	40	40	88,9%	94,9%	98,1%	98,3%	94,1%
18817	60	55	40	45	89,1%	94,9%	98,2%	98,4%	94,5%
18818	60	55	40	50	89,3%	94,8%	98,2%	98,5%	94,8%
18819	60	55	40	55	89,5%	94,7%	98,3%	98,6%	95,2%
18820	60	55	40	60	89,6%	94,7%	98,4%	98,7%	95,5%
18821	60	55	40	65	89,8%	94,6%	98,4%	98,8%	95,8%
18822	60	55	40	70	89,9%	94,5%	98,5%	98,9%	96,0%
18823	60	55	40	75	90,1%	94,5%	98,5%	99,0%	96,3%
18824	60	55	40	80	90,3%	94,4%	98,6%	99,0%	96,5%
18825	60	55	45	20	85,4%	94,0%	97,6%	97,7%	92,1%
18826	60	55	45	25	85,6%	93,9%	97,7%	97,8%	92,6%
18827	60	55	45	30	85,8%	93,8%	97,7%	98,0%	93,1%
18828	60	55	45	35	86,1%	93,8%	97,8%	98,1%	93,5%
18829	60	55	45	40	86,3%	93,7%	97,9%	98,2%	93,9%
18830	60	55	45	45	86,5%	93,6%	97,9%	98,3%	94,3%
18831	60	55	45	50	86,7%	93,5%	98,0%	98,5%	94,6%
18832	60	55	45	55	86,9%	93,4%	98,1%	98,6%	95,0%
18833	60	55	45	60	87,1%	93,4%	98,1%	98,7%	95,3%
18834	60	55	45	65	87,3%	93,3%	98,2%	98,7%	95,6%
18835	60	55	45	70	87,5%	93,2%	98,3%	98,8%	95,9%
18836	60	55	45	75	87,7%	93,1%	98,3%	98,9%	96,1%
18837	60	55	45	80	87,9%	93,0%	98,4%	99,0%	96,4%
18838	60	55	50	20	82,1%	92,5%	97,3%	97,5%	91,8%
18839	60	55	50	25	82,3%	92,4%	97,4%	97,7%	92,3%
18840	60	55	50	30	82,6%	92,4%	97,4%	97,9%	92,8%
18841	60	55	50	35	82,8%	92,3%	97,5%	98,0%	93,2%
18842	60	55	50	40	83,1%	92,2%	97,6%	98,1%	93,6%

18843	60	55	50	45	83,3%	92,1%	97,7%	98,2%	94,0%
18844	60	55	50	50	83,6%	92,0%	97,8%	98,4%	94,4%
18845	60	55	50	55	83,8%	91,9%	97,8%	98,5%	94,8%
18846	60	55	50	60	84,0%	91,8%	97,9%	98,6%	95,1%
18847	60	55	50	65	84,3%	91,7%	98,0%	98,7%	95,4%
18848	60	55	50	70	84,5%	91,6%	98,0%	98,8%	95,7%
18849	60	55	50	75	84,7%	91,5%	98,1%	98,8%	96,0%
18850	60	55	50	80	85,0%	91,4%	98,2%	98,9%	96,2%
18851	60	55	55	20	78,1%	90,8%	96,9%	97,4%	91,5%
18852	60	55	55	25	78,4%	90,7%	97,0%	97,6%	92,0%
18853	60	55	55	30	78,7%	90,6%	97,1%	97,7%	92,5%
18854	60	55	55	35	79,0%	90,4%	97,2%	97,9%	93,0%
18855	60	55	55	40	79,3%	90,3%	97,3%	98,0%	93,4%
18856	60	55	55	45	79,6%	90,2%	97,4%	98,1%	93,8%
18857	60	55	55	50	79,9%	90,1%	97,5%	98,3%	94,2%
18858	60	55	55	55	80,2%	90,0%	97,6%	98,4%	94,6%
18859	60	55	55	60	80,4%	89,9%	97,6%	98,5%	94,9%
18860	60	55	55	65	80,7%	89,7%	97,7%	98,6%	95,2%
18861	60	55	55	70	81,0%	89,6%	97,8%	98,7%	95,5%
18862	60	55	55	75	81,3%	89,5%	97,9%	98,8%	95,8%
18863	60	55	55	80	81,5%	89,4%	97,9%	98,8%	96,1%
18864	60	55	60	20	73,6%	88,6%	96,5%	97,2%	91,2%
18865	60	55	60	25	74,0%	88,5%	96,6%	97,4%	91,7%
18866	60	55	60	30	74,3%	88,4%	96,7%	97,6%	92,2%
18867	60	55	60	35	74,6%	88,2%	96,8%	97,7%	92,7%
18868	60	55	60	40	75,0%	88,1%	96,9%	97,9%	93,1%
18869	60	55	60	45	75,3%	88,0%	97,0%	98,0%	93,6%
18870	60	55	60	50	75,6%	87,8%	97,1%	98,2%	94,0%
18871	60	55	60	55	76,0%	87,7%	97,2%	98,3%	94,3%
18872	60	55	60	60	76,3%	87,6%	97,3%	98,4%	94,7%
18873	60	55	60	65	76,6%	87,4%	97,4%	98,5%	95,0%
18874	60	55	60	70	76,9%	87,3%	97,5%	98,6%	95,3%
18875	60	55	60	75	77,2%	87,1%	97,6%	98,7%	95,6%

18876	60	55	60	80	77,5%	87,0%	97,7%	98,8%	95,9%
18877	60	55	65	20	68,6%	86,1%	96,1%	97,1%	90,8%
18878	60	55	65	25	68,9%	86,0%	96,2%	97,3%	91,4%
18879	60	55	65	30	69,3%	85,8%	96,3%	97,4%	91,9%
18880	60	55	65	35	69,7%	85,6%	96,4%	97,6%	92,4%
18881	60	55	65	40	70,1%	85,5%	96,6%	97,8%	92,9%
18882	60	55	65	45	70,4%	85,3%	96,7%	97,9%	93,3%
18883	60	55	65	50	70,8%	85,1%	96,8%	98,0%	93,7%
18884	60	55	65	55	71,2%	85,0%	96,9%	98,2%	94,1%
18885	60	55	65	60	71,5%	84,8%	97,0%	98,3%	94,5%
18886	60	55	65	65	71,9%	84,6%	97,1%	98,4%	94,8%
18887	60	55	65	70	72,2%	84,5%	97,2%	98,5%	95,2%
18888	60	55	65	75	72,6%	84,3%	97,3%	98,6%	95,5%
18889	60	55	65	80	72,9%	84,1%	97,4%	98,7%	95,7%
18890	60	55	70	20	63,0%	83,1%	95,6%	96,9%	90,5%
18891	60	55	70	25	63,4%	82,9%	95,7%	97,1%	91,1%
18892	60	55	70	30	63,8%	82,7%	95,9%	97,3%	91,6%
18893	60	55	70	35	64,2%	82,6%	96,0%	97,5%	92,1%
18894	60	55	70	40	64,6%	82,4%	96,1%	97,6%	92,6%
18895	60	55	70	45	65,0%	82,2%	96,2%	97,8%	93,0%
18896	60	55	70	50	65,4%	82,0%	96,4%	97,9%	93,5%
18897	60	55	70	55	65,8%	81,8%	96,5%	98,1%	93,9%
18898	60	55	70	60	66,2%	81,6%	96,6%	98,2%	94,3%
18899	60	55	70	65	66,6%	81,4%	96,7%	98,3%	94,6%
18900	60	55	70	70	67,0%	81,2%	96,8%	98,4%	95,0%
18901	60	55	70	75	67,4%	81,0%	96,9%	98,5%	95,3%
18902	60	55	70	80	67,8%	80,8%	97,0%	98,6%	95,6%
18903	60	55	75	20	57,1%	79,6%	95,0%	96,7%	90,1%
18904	60	55	75	25	57,5%	79,4%	95,2%	96,9%	90,7%
18905	60	55	75	30	58,0%	79,2%	95,3%	97,1%	91,3%
18906	60	55	75	35	58,4%	79,0%	95,5%	97,3%	91,8%
18907	60	55	75	40	58,8%	78,8%	95,6%	97,5%	92,3%
18908	60	55	75	45	59,2%	78,5%	95,8%	97,6%	92,8%

18909	60	55	75	50	59,7%	78,3%	95,9%	97,8%	93,2%
18910	60	55	75	55	60,1%	78,1%	96,0%	97,9%	93,6%
18911	60	55	75	60	60,5%	77,9%	96,2%	98,1%	94,0%
18912	60	55	75	65	60,9%	77,6%	96,3%	98,2%	94,4%
18913	60	55	75	70	61,4%	77,4%	96,4%	98,3%	94,8%
18914	60	55	75	75	61,8%	77,2%	96,5%	98,4%	95,1%
18915	60	55	75	80	62,2%	77,0%	96,6%	98,5%	95,4%
18916	60	55	80	20	51,0%	75,6%	94,4%	96,5%	89,7%
18917	60	55	80	25	51,4%	75,4%	94,6%	96,7%	90,4%
18918	60	55	80	30	51,9%	75,1%	94,8%	96,9%	90,9%
18919	60	55	80	35	52,3%	74,9%	94,9%	97,1%	91,5%
18920	60	55	80	40	52,7%	74,6%	95,1%	97,3%	92,0%
18921	60	55	80	45	53,2%	74,4%	95,2%	97,5%	92,5%
18922	60	55	80	50	53,6%	74,1%	95,4%	97,7%	93,0%
18923	60	55	80	55	54,1%	73,9%	95,5%	97,8%	93,4%
18924	60	55	80	60	54,5%	73,6%	95,7%	98,0%	93,8%
18925	60	55	80	65	54,9%	73,4%	95,8%	98,1%	94,2%
18926	60	55	80	70	55,4%	73,1%	96,0%	98,2%	94,6%
18927	60	55	80	75	55,8%	72,9%	96,1%	98,3%	94,9%
18928	60	55	80	80	56,2%	72,6%	96,2%	98,4%	95,2%
18929	60	60	20	20	95,6%	98,2%	98,6%	98,2%	92,9%
18930	60	60	20	25	95,7%	98,1%	98,7%	98,3%	93,3%
18931	60	60	20	30	95,8%	98,1%	98,7%	98,4%	93,7%
18932	60	60	20	35	95,9%	98,1%	98,8%	98,5%	94,1%
18933	60	60	20	40	95,9%	98,1%	98,8%	98,6%	94,5%
18934	60	60	20	45	96,0%	98,0%	98,9%	98,7%	94,8%
18935	60	60	20	50	96,1%	98,0%	98,9%	98,8%	95,2%
18936	60	60	20	55	96,1%	98,0%	98,9%	98,9%	95,5%
18937	60	60	20	60	96,2%	98,0%	99,0%	98,9%	95,8%
18938	60	60	20	65	96,3%	97,9%	99,0%	99,0%	96,0%
18939	60	60	20	70	96,3%	97,9%	99,0%	99,1%	96,3%
18940	60	60	20	75	96,4%	97,9%	99,1%	99,1%	96,5%
18941	60	60	20	80	96,4%	97,9%	99,1%	99,2%	96,7%

18942	60	60	25	20	94,5%	97,7%	98,5%	98,0%	92,6%
18943	60	60	25	25	94,6%	97,7%	98,5%	98,2%	93,1%
18944	60	60	25	30	94,7%	97,6%	98,6%	98,3%	93,5%
18945	60	60	25	35	94,8%	97,6%	98,6%	98,4%	93,9%
18946	60	60	25	40	94,9%	97,6%	98,7%	98,5%	94,3%
18947	60	60	25	45	94,9%	97,6%	98,7%	98,6%	94,6%
18948	60	60	25	50	95,0%	97,5%	98,7%	98,7%	95,0%
18949	60	60	25	55	95,1%	97,5%	98,8%	98,8%	95,3%
18950	60	60	25	60	95,2%	97,5%	98,8%	98,9%	95,6%
18951	60	60	25	65	95,3%	97,4%	98,9%	98,9%	95,9%
18952	60	60	25	70	95,3%	97,4%	98,9%	99,0%	96,1%
18953	60	60	25	75	95,4%	97,4%	98,9%	99,1%	96,4%
18954	60	60	25	80	95,5%	97,3%	99,0%	99,1%	96,6%
18955	60	60	30	20	93,1%	97,1%	98,3%	97,9%	92,3%
18956	60	60	30	25	93,2%	97,1%	98,3%	98,1%	92,8%
18957	60	60	30	30	93,3%	97,1%	98,4%	98,2%	93,2%
18958	60	60	30	35	93,4%	97,0%	98,4%	98,3%	93,7%
18959	60	60	30	40	93,5%	97,0%	98,5%	98,4%	94,0%
18960	60	60	30	45	93,6%	96,9%	98,5%	98,5%	94,4%
18961	60	60	30	50	93,7%	96,9%	98,6%	98,6%	94,8%
18962	60	60	30	55	93,8%	96,9%	98,6%	98,7%	95,1%
18963	60	60	30	60	93,9%	96,8%	98,7%	98,8%	95,4%
18964	60	60	30	65	94,0%	96,8%	98,7%	98,9%	95,7%
18965	60	60	30	70	94,1%	96,7%	98,8%	98,9%	96,0%
18966	60	60	30	75	94,2%	96,7%	98,8%	99,0%	96,2%
18967	60	60	30	80	94,3%	96,7%	98,8%	99,1%	96,5%
18968	60	60	35	20	91,3%	96,4%	98,0%	97,8%	92,0%
18969	60	60	35	25	91,4%	96,4%	98,1%	97,9%	92,5%
18970	60	60	35	30	91,6%	96,3%	98,2%	98,1%	93,0%
18971	60	60	35	35	91,7%	96,3%	98,2%	98,2%	93,4%
18972	60	60	35	40	91,8%	96,2%	98,3%	98,3%	93,8%
18973	60	60	35	45	92,0%	96,2%	98,3%	98,4%	94,2%
18974	60	60	35	50	92,1%	96,1%	98,4%	98,5%	94,6%

18975	60	60	35	55	92,2%	96,1%	98,4%	98,6%	94,9%
18976	60	60	35	60	92,3%	96,0%	98,5%	98,7%	95,2%
18977	60	60	35	65	92,5%	96,0%	98,5%	98,8%	95,5%
18978	60	60	35	70	92,6%	95,9%	98,6%	98,9%	95,8%
18979	60	60	35	75	92,7%	95,9%	98,6%	99,0%	96,1%
18980	60	60	35	80	92,8%	95,8%	98,7%	99,0%	96,3%
18981	60	60	40	20	89,1%	95,5%	97,8%	97,7%	91,7%
18982	60	60	40	25	89,3%	95,5%	97,9%	97,8%	92,2%
18983	60	60	40	30	89,5%	95,4%	97,9%	98,0%	92,7%
18984	60	60	40	35	89,6%	95,3%	98,0%	98,1%	93,1%
18985	60	60	40	40	89,8%	95,3%	98,1%	98,2%	93,6%
18986	60	60	40	45	89,9%	95,2%	98,1%	98,3%	94,0%
18987	60	60	40	50	90,1%	95,2%	98,2%	98,4%	94,3%
18988	60	60	40	55	90,3%	95,1%	98,2%	98,5%	94,7%
18989	60	60	40	60	90,4%	95,0%	98,3%	98,6%	95,0%
18990	60	60	40	65	90,6%	95,0%	98,4%	98,7%	95,3%
18991	60	60	40	70	90,7%	94,9%	98,4%	98,8%	95,6%
18992	60	60	40	75	90,9%	94,9%	98,5%	98,9%	95,9%
18993	60	60	40	80	91,0%	94,8%	98,5%	99,0%	96,2%
18994	60	60	45	20	86,5%	94,4%	97,5%	97,5%	91,4%
18995	60	60	45	25	86,7%	94,3%	97,6%	97,7%	91,9%
18996	60	60	45	30	86,9%	94,3%	97,7%	97,8%	92,4%
18997	60	60	45	35	87,1%	94,2%	97,7%	98,0%	92,9%
18998	60	60	45	40	87,3%	94,1%	97,8%	98,1%	93,3%
18999	60	60	45	45	87,5%	94,1%	97,9%	98,2%	93,7%
19000	60	60	45	50	87,7%	94,0%	98,0%	98,3%	94,1%
19001	60	60	45	55	87,9%	93,9%	98,0%	98,5%	94,5%
19002	60	60	45	60	88,0%	93,8%	98,1%	98,6%	94,8%
19003	60	60	45	65	88,2%	93,8%	98,1%	98,6%	95,2%
19004	60	60	45	70	88,4%	93,7%	98,2%	98,7%	95,5%
19005	60	60	45	75	88,6%	93,6%	98,3%	98,8%	95,8%
19006	60	60	45	80	88,8%	93,5%	98,3%	98,9%	96,0%
19007	60	60	50	20	83,3%	93,1%	97,2%	97,4%	91,1%

19008	60	60	50	25	83,6%	93,0%	97,3%	97,5%	91,6%
19009	60	60	50	30	83,8%	92,9%	97,4%	97,7%	92,1%
19010	60	60	50	35	84,1%	92,8%	97,4%	97,8%	92,6%
19011	60	60	50	40	84,3%	92,7%	97,5%	98,0%	93,1%
19012	60	60	50	45	84,5%	92,6%	97,6%	98,1%	93,5%
19013	60	60	50	50	84,7%	92,5%	97,7%	98,2%	93,9%
19014	60	60	50	55	85,0%	92,4%	97,8%	98,4%	94,3%
19015	60	60	50	60	85,2%	92,4%	97,8%	98,5%	94,6%
19016	60	60	50	65	85,4%	92,3%	97,9%	98,6%	95,0%
19017	60	60	50	70	85,6%	92,2%	98,0%	98,7%	95,3%
19018	60	60	50	75	85,9%	92,1%	98,0%	98,7%	95,6%
19019	60	60	50	80	86,1%	92,0%	98,1%	98,8%	95,9%
19020	60	60	55	20	79,6%	91,4%	96,8%	97,2%	90,7%
19021	60	60	55	25	79,9%	91,3%	96,9%	97,4%	91,3%
19022	60	60	55	30	80,2%	91,2%	97,0%	97,6%	91,8%
19023	60	60	55	35	80,5%	91,1%	97,1%	97,7%	92,3%
19024	60	60	55	40	80,7%	91,0%	97,2%	97,9%	92,8%
19025	60	60	55	45	81,0%	90,9%	97,3%	98,0%	93,2%
19026	60	60	55	50	81,3%	90,8%	97,4%	98,1%	93,6%
19027	60	60	55	55	81,5%	90,7%	97,5%	98,3%	94,0%
19028	60	60	55	60	81,8%	90,6%	97,6%	98,4%	94,4%
19029	60	60	55	65	82,1%	90,4%	97,6%	98,5%	94,8%
19030	60	60	55	70	82,3%	90,3%	97,7%	98,6%	95,1%
19031	60	60	55	75	82,6%	90,2%	97,8%	98,7%	95,4%
19032	60	60	55	80	82,8%	90,1%	97,9%	98,8%	95,7%
19033	60	60	60	20	75,3%	89,4%	96,4%	97,0%	90,4%
19034	60	60	60	25	75,6%	89,3%	96,5%	97,2%	90,9%
19035	60	60	60	30	76,0%	89,2%	96,6%	97,4%	91,5%
19036	60	60	60	35	76,3%	89,0%	96,7%	97,6%	92,0%
19037	60	60	60	40	76,6%	88,9%	96,9%	97,7%	92,5%
19038	60	60	60	45	76,9%	88,8%	97,0%	97,9%	93,0%
19039	60	60	60	50	77,2%	88,7%	97,1%	98,0%	93,4%
19040	60	60	60	55	77,5%	88,5%	97,1%	98,1%	93,8%

19041	60	60	60	60	77,8%	88,4%	97,2%	98,3%	94,2%
19042	60	60	60	65	78,1%	88,3%	97,3%	98,4%	94,6%
19043	60	60	60	70	78,4%	88,1%	97,4%	98,5%	94,9%
19044	60	60	60	75	78,7%	88,0%	97,5%	98,6%	95,2%
19045	60	60	60	80	79,0%	87,8%	97,6%	98,7%	95,5%
19046	60	60	65	20	70,4%	87,0%	96,0%	96,8%	90,0%
19047	60	60	65	25	70,8%	86,9%	96,1%	97,1%	90,6%
19048	60	60	65	30	71,2%	86,7%	96,2%	97,2%	91,2%
19049	60	60	65	35	71,5%	86,6%	96,3%	97,4%	91,7%
19050	60	60	65	40	71,9%	86,4%	96,5%	97,6%	92,2%
19051	60	60	65	45	72,2%	86,3%	96,6%	97,7%	92,7%
19052	60	60	65	50	72,6%	86,1%	96,7%	97,9%	93,1%
19053	60	60	65	55	72,9%	86,0%	96,8%	98,0%	93,6%
19054	60	60	65	60	73,3%	85,8%	96,9%	98,2%	94,0%
19055	60	60	65	65	73,6%	85,6%	97,0%	98,3%	94,3%
19056	60	60	65	70	74,0%	85,5%	97,1%	98,4%	94,7%
19057	60	60	65	75	74,3%	85,3%	97,2%	98,5%	95,0%
19058	60	60	65	80	74,6%	85,1%	97,3%	98,6%	95,3%
19059	60	60	70	20	65,1%	84,2%	95,4%	96,7%	89,6%
19060	60	60	70	25	65,5%	84,0%	95,6%	96,9%	90,2%
19061	60	60	70	30	65,9%	83,8%	95,7%	97,1%	90,8%
19062	60	60	70	35	66,2%	83,7%	95,9%	97,3%	91,4%
19063	60	60	70	40	66,6%	83,5%	96,0%	97,4%	91,9%
19064	60	60	70	45	67,0%	83,3%	96,1%	97,6%	92,4%
19065	60	60	70	50	67,4%	83,1%	96,3%	97,8%	92,9%
19066	60	60	70	55	67,8%	82,9%	96,4%	97,9%	93,3%
19067	60	60	70	60	68,2%	82,7%	96,5%	98,0%	93,7%
19068	60	60	70	65	68,6%	82,6%	96,6%	98,2%	94,1%
19069	60	60	70	70	68,9%	82,4%	96,7%	98,3%	94,5%
19070	60	60	70	75	69,3%	82,2%	96,8%	98,4%	94,8%
19071	60	60	70	80	69,7%	82,0%	96,9%	98,5%	95,2%
19072	60	60	75	20	59,3%	80,9%	94,9%	96,5%	89,2%
19073	60	60	75	25	59,7%	80,7%	95,0%	96,7%	89,9%

19074	60	60	75	30	60,1%	80,5%	95,2%	96,9%	90,5%
19075	60	60	75	35	60,5%	80,3%	95,3%	97,1%	91,1%
19076	60	60	75	40	60,9%	80,0%	95,5%	97,3%	91,6%
19077	60	60	75	45	61,4%	79,8%	95,6%	97,5%	92,1%
19078	60	60	75	50	61,8%	79,6%	95,8%	97,6%	92,6%
19079	60	60	75	55	62,2%	79,4%	95,9%	97,8%	93,0%
19080	60	60	75	60	62,6%	79,2%	96,0%	97,9%	93,5%
19081	60	60	75	65	63,0%	79,0%	96,2%	98,1%	93,9%
19082	60	60	75	70	63,4%	78,8%	96,3%	98,2%	94,3%
19083	60	60	75	75	63,8%	78,5%	96,4%	98,3%	94,6%
19084	60	60	75	80	64,2%	78,3%	96,5%	98,4%	95,0%
19085	60	60	80	20	53,2%	77,0%	94,2%	96,2%	88,8%
19086	60	60	80	25	53,6%	76,8%	94,4%	96,5%	89,5%
19087	60	60	80	30	54,1%	76,6%	94,6%	96,7%	90,1%
19088	60	60	80	35	54,5%	76,3%	94,8%	96,9%	90,7%
19089	60	60	80	40	54,9%	76,1%	94,9%	97,1%	91,3%
19090	60	60	80	45	55,4%	75,9%	95,1%	97,3%	91,8%
19091	60	60	80	50	55,8%	75,6%	95,2%	97,5%	92,3%
19092	60	60	80	55	56,2%	75,4%	95,4%	97,6%	92,8%
19093	60	60	80	60	56,7%	75,1%	95,5%	97,8%	93,2%
19094	60	60	80	65	57,1%	74,9%	95,7%	97,9%	93,6%
19095	60	60	80	70	57,5%	74,6%	95,8%	98,1%	94,0%
19096	60	60	80	75	58,0%	74,4%	96,0%	98,2%	94,4%
19097	60	60	80	80	58,4%	74,1%	96,1%	98,3%	94,8%
19098	60	65	20	20	96,0%	98,3%	98,6%	98,0%	92,2%
19099	60	65	20	25	96,1%	98,3%	98,6%	98,1%	92,7%
19100	60	65	20	30	96,1%	98,3%	98,7%	98,3%	93,1%
19101	60	65	20	35	96,2%	98,2%	98,7%	98,4%	93,6%
19102	60	65	20	40	96,3%	98,2%	98,8%	98,5%	94,0%
19103	60	65	20	45	96,3%	98,2%	98,8%	98,6%	94,3%
19104	60	65	20	50	96,4%	98,2%	98,9%	98,7%	94,7%
19105	60	65	20	55	96,5%	98,1%	98,9%	98,8%	95,0%
19106	60	65	20	60	96,5%	98,1%	98,9%	98,8%	95,4%

19107	60	65	20	65	96,6%	98,1%	99,0%	98,9%	95,6%
19108	60	65	20	70	96,6%	98,1%	99,0%	99,0%	95,9%
19109	60	65	20	75	96,7%	98,0%	99,0%	99,1%	96,2%
19110	60	65	20	80	96,7%	98,0%	99,1%	99,1%	96,4%
19111	60	65	25	20	94,9%	97,9%	98,4%	97,9%	91,9%
19112	60	65	25	25	95,0%	97,8%	98,5%	98,0%	92,4%
19113	60	65	25	30	95,1%	97,8%	98,5%	98,2%	92,9%
19114	60	65	25	35	95,2%	97,8%	98,6%	98,3%	93,3%
19115	60	65	25	40	95,3%	97,8%	98,6%	98,4%	93,7%
19116	60	65	25	45	95,3%	97,7%	98,7%	98,5%	94,1%
19117	60	65	25	50	95,4%	97,7%	98,7%	98,6%	94,5%
19118	60	65	25	55	95,5%	97,7%	98,7%	98,7%	94,8%
19119	60	65	25	60	95,6%	97,6%	98,8%	98,8%	95,2%
19120	60	65	25	65	95,7%	97,6%	98,8%	98,9%	95,5%
19121	60	65	25	70	95,7%	97,6%	98,9%	98,9%	95,8%
19122	60	65	25	75	95,8%	97,6%	98,9%	99,0%	96,0%
19123	60	65	25	80	95,9%	97,5%	98,9%	99,1%	96,3%
19124	60	65	30	20	93,6%	97,3%	98,2%	97,8%	91,6%
19125	60	65	30	25	93,7%	97,3%	98,3%	97,9%	92,1%
19126	60	65	30	30	93,8%	97,3%	98,3%	98,0%	92,6%
19127	60	65	30	35	93,9%	97,2%	98,4%	98,2%	93,1%
19128	60	65	30	40	94,0%	97,2%	98,4%	98,3%	93,5%
19129	60	65	30	45	94,1%	97,2%	98,5%	98,4%	93,9%
19130	60	65	30	50	94,2%	97,1%	98,5%	98,5%	94,3%
19131	60	65	30	55	94,3%	97,1%	98,6%	98,6%	94,6%
19132	60	65	30	60	94,4%	97,1%	98,6%	98,7%	95,0%
19133	60	65	30	65	94,5%	97,0%	98,7%	98,8%	95,3%
19134	60	65	30	70	94,6%	97,0%	98,7%	98,9%	95,6%
19135	60	65	30	75	94,7%	96,9%	98,8%	98,9%	95,9%
19136	60	65	30	80	94,8%	96,9%	98,8%	99,0%	96,1%
19137	60	65	35	20	92,0%	96,7%	98,0%	97,6%	91,3%
19138	60	65	35	25	92,1%	96,6%	98,0%	97,8%	91,8%
19139	60	65	35	30	92,2%	96,6%	98,1%	97,9%	92,3%

19140	60	65	35	35	92,4%	96,5%	98,2%	98,1%	92,8%
19141	60	65	35	40	92,5%	96,5%	98,2%	98,2%	93,2%
19142	60	65	35	45	92,6%	96,5%	98,3%	98,3%	93,7%
19143	60	65	35	50	92,7%	96,4%	98,3%	98,4%	94,0%
19144	60	65	35	55	92,8%	96,4%	98,4%	98,5%	94,4%
19145	60	65	35	60	93,0%	96,3%	98,5%	98,6%	94,8%
19146	60	65	35	65	93,1%	96,3%	98,5%	98,7%	95,1%
19147	60	65	35	70	93,2%	96,2%	98,6%	98,8%	95,4%
19148	60	65	35	75	93,3%	96,2%	98,6%	98,9%	95,7%
19149	60	65	35	80	93,4%	96,1%	98,6%	98,9%	96,0%
19150	60	65	40	20	89,9%	95,8%	97,7%	97,5%	91,0%
19151	60	65	40	25	90,1%	95,8%	97,8%	97,6%	91,5%
19152	60	65	40	30	90,3%	95,7%	97,9%	97,8%	92,0%
19153	60	65	40	35	90,4%	95,7%	97,9%	97,9%	92,5%
19154	60	65	40	40	90,6%	95,6%	98,0%	98,1%	93,0%
19155	60	65	40	45	90,7%	95,6%	98,1%	98,2%	93,4%
19156	60	65	40	50	90,9%	95,5%	98,1%	98,3%	93,8%
19157	60	65	40	55	91,0%	95,5%	98,2%	98,4%	94,2%
19158	60	65	40	60	91,2%	95,4%	98,2%	98,5%	94,6%
19159	60	65	40	65	91,3%	95,3%	98,3%	98,6%	94,9%
19160	60	65	40	70	91,4%	95,3%	98,4%	98,7%	95,2%
19161	60	65	40	75	91,6%	95,2%	98,4%	98,8%	95,5%
19162	60	65	40	80	91,7%	95,2%	98,5%	98,9%	95,8%
19163	60	65	45	20	87,5%	94,8%	97,4%	97,3%	90,6%
19164	60	65	45	25	87,7%	94,7%	97,5%	97,5%	91,2%
19165	60	65	45	30	87,9%	94,7%	97,6%	97,7%	91,7%
19166	60	65	45	35	88,1%	94,6%	97,7%	97,8%	92,2%
19167	60	65	45	40	88,2%	94,5%	97,7%	98,0%	92,7%
19168	60	65	45	45	88,4%	94,5%	97,8%	98,1%	93,1%
19169	60	65	45	50	88,6%	94,4%	97,9%	98,2%	93,6%
19170	60	65	45	55	88,8%	94,3%	98,0%	98,3%	94,0%
19171	60	65	45	60	89,0%	94,3%	98,0%	98,4%	94,3%
19172	60	65	45	65	89,1%	94,2%	98,1%	98,5%	94,7%

19173	60	65	45	70	89,3%	94,1%	98,2%	98,6%	95,0%
19174	60	65	45	75	89,5%	94,1%	98,2%	98,7%	95,3%
19175	60	65	45	80	89,6%	94,0%	98,3%	98,8%	95,6%
19176	60	65	50	20	84,5%	93,6%	97,1%	97,2%	90,3%
19177	60	65	50	25	84,8%	93,5%	97,2%	97,3%	90,8%
19178	60	65	50	30	85,0%	93,4%	97,3%	97,5%	91,4%
19179	60	65	50	35	85,2%	93,3%	97,4%	97,7%	91,9%
19180	60	65	50	40	85,4%	93,2%	97,5%	97,8%	92,4%
19181	60	65	50	45	85,6%	93,1%	97,5%	98,0%	92,9%
19182	60	65	50	50	85,9%	93,1%	97,6%	98,1%	93,3%
19183	60	65	50	55	86,1%	93,0%	97,7%	98,2%	93,7%
19184	60	65	50	60	86,3%	92,9%	97,8%	98,3%	94,1%
19185	60	65	50	65	86,5%	92,8%	97,8%	98,5%	94,5%
19186	60	65	50	70	86,7%	92,7%	97,9%	98,6%	94,8%
19187	60	65	50	75	86,9%	92,6%	98,0%	98,7%	95,2%
19188	60	65	50	80	87,1%	92,5%	98,0%	98,7%	95,5%
19189	60	65	55	20	81,0%	92,0%	96,7%	97,0%	89,9%
19190	60	65	55	25	81,3%	91,9%	96,8%	97,2%	90,5%
19191	60	65	55	30	81,6%	91,8%	96,9%	97,4%	91,1%
19192	60	65	55	35	81,8%	91,7%	97,0%	97,5%	91,6%
19193	60	65	55	40	82,1%	91,6%	97,1%	97,7%	92,1%
19194	60	65	55	45	82,3%	91,5%	97,2%	97,8%	92,6%
19195	60	65	55	50	82,6%	91,4%	97,3%	98,0%	93,1%
19196	60	65	55	55	82,8%	91,3%	97,4%	98,1%	93,5%
19197	60	65	55	60	83,1%	91,2%	97,5%	98,2%	93,9%
19198	60	65	55	65	83,3%	91,1%	97,6%	98,4%	94,3%
19199	60	65	55	70	83,6%	91,0%	97,6%	98,5%	94,6%
19200	60	65	55	75	83,8%	90,9%	97,7%	98,6%	95,0%
19201	60	65	55	80	84,1%	90,8%	97,8%	98,7%	95,3%
19202	60	65	60	20	76,9%	90,1%	96,3%	96,8%	89,5%
19203	60	65	60	25	77,2%	90,0%	96,4%	97,0%	90,1%
19204	60	65	60	30	77,5%	89,9%	96,5%	97,2%	90,7%
19205	60	65	60	35	77,8%	89,8%	96,6%	97,4%	91,3%

19206	60	65	60	40	78,2%	89,7%	96,8%	97,6%	91,8%
19207	60	65	60	45	78,5%	89,5%	96,9%	97,7%	92,3%
19208	60	65	60	50	78,7%	89,4%	97,0%	97,9%	92,8%
19209	60	65	60	55	79,0%	89,3%	97,1%	98,0%	93,2%
19210	60	65	60	60	79,3%	89,2%	97,2%	98,1%	93,6%
19211	60	65	60	65	79,6%	89,0%	97,2%	98,3%	94,0%
19212	60	65	60	70	79,9%	88,9%	97,3%	98,4%	94,4%
19213	60	65	60	75	80,2%	88,8%	97,4%	98,5%	94,8%
19214	60	65	60	80	80,5%	88,7%	97,5%	98,6%	95,1%
19215	60	65	65	20	72,3%	87,9%	95,8%	96,6%	89,1%
19216	60	65	65	25	72,6%	87,7%	96,0%	96,8%	89,8%
19217	60	65	65	30	73,0%	87,6%	96,1%	97,0%	90,4%
19218	60	65	65	35	73,3%	87,5%	96,2%	97,2%	90,9%
19219	60	65	65	40	73,6%	87,3%	96,3%	97,4%	91,5%
19220	60	65	65	45	74,0%	87,2%	96,5%	97,6%	92,0%
19221	60	65	65	50	74,3%	87,0%	96,6%	97,7%	92,5%
19222	60	65	65	55	74,7%	86,9%	96,7%	97,9%	93,0%
19223	60	65	65	60	75,0%	86,7%	96,8%	98,0%	93,4%
19224	60	65	65	65	75,3%	86,6%	96,9%	98,2%	93,8%
19225	60	65	65	70	75,6%	86,4%	97,0%	98,3%	94,2%
19226	60	65	65	75	76,0%	86,3%	97,1%	98,4%	94,6%
19227	60	65	65	80	76,3%	86,1%	97,2%	98,5%	94,9%
19228	60	65	70	20	67,0%	85,2%	95,3%	96,4%	88,7%
19229	60	65	70	25	67,4%	85,0%	95,5%	96,6%	89,4%
19230	60	65	70	30	67,8%	84,9%	95,6%	96,9%	90,0%
19231	60	65	70	35	68,2%	84,7%	95,7%	97,1%	90,6%
19232	60	65	70	40	68,6%	84,5%	95,9%	97,3%	91,2%
19233	60	65	70	45	69,0%	84,4%	96,0%	97,4%	91,7%
19234	60	65	70	50	69,3%	84,2%	96,1%	97,6%	92,2%
19235	60	65	70	55	69,7%	84,0%	96,3%	97,8%	92,7%
19236	60	65	70	60	70,1%	83,8%	96,4%	97,9%	93,1%
19237	60	65	70	65	70,5%	83,7%	96,5%	98,0%	93,6%
19238	60	65	70	70	70,8%	83,5%	96,6%	98,2%	94,0%

19239	60	65	70	75	71,2%	83,3%	96,7%	98,3%	94,3%
19240	60	65	70	80	71,5%	83,1%	96,8%	98,4%	94,7%
19241	60	65	75	20	61,4%	82,1%	94,7%	96,2%	88,3%
19242	60	65	75	25	61,8%	81,9%	94,9%	96,4%	89,0%
19243	60	65	75	30	62,2%	81,7%	95,0%	96,7%	89,6%
19244	60	65	75	35	62,6%	81,5%	95,2%	96,9%	90,2%
19245	60	65	75	40	63,0%	81,3%	95,4%	97,1%	90,8%
19246	60	65	75	45	63,4%	81,1%	95,5%	97,3%	91,4%
19247	60	65	75	50	63,9%	80,9%	95,6%	97,4%	91,9%
19248	60	65	75	55	64,3%	80,7%	95,8%	97,6%	92,4%
19249	60	65	75	60	64,7%	80,5%	95,9%	97,8%	92,9%
19250	60	65	75	65	65,1%	80,3%	96,1%	97,9%	93,3%
19251	60	65	75	70	65,5%	80,0%	96,2%	98,1%	93,7%
19252	60	65	75	75	65,9%	79,8%	96,3%	98,2%	94,1%
19253	60	65	75	80	66,3%	79,6%	96,4%	98,3%	94,5%
19254	60	65	80	20	55,4%	78,4%	94,1%	96,0%	87,8%
19255	60	65	80	25	55,8%	78,2%	94,2%	96,2%	88,6%
19256	60	65	80	30	56,3%	78,0%	94,4%	96,5%	89,2%
19257	60	65	80	35	56,7%	77,7%	94,6%	96,7%	89,9%
19258	60	65	80	40	57,1%	77,5%	94,8%	96,9%	90,5%
19259	60	65	80	45	57,6%	77,3%	94,9%	97,1%	91,1%
19260	60	65	80	50	58,0%	77,0%	95,1%	97,3%	91,6%
19261	60	65	80	55	58,4%	76,8%	95,3%	97,5%	92,1%
19262	60	65	80	60	58,8%	76,6%	95,4%	97,6%	92,6%
19263	60	65	80	65	59,3%	76,3%	95,6%	97,8%	93,0%
19264	60	65	80	70	59,7%	76,1%	95,7%	97,9%	93,5%
19265	60	65	80	75	60,1%	75,9%	95,8%	98,1%	93,9%
19266	60	65	80	80	60,5%	75,6%	96,0%	98,2%	94,3%
19267	60	70	20	20	96,3%	98,4%	98,6%	97,9%	91,5%
19268	60	70	20	25	96,4%	98,4%	98,6%	98,0%	92,0%
19269	60	70	20	30	96,5%	98,4%	98,6%	98,1%	92,5%
19270	60	70	20	35	96,5%	98,4%	98,7%	98,3%	93,0%
19271	60	70	20	40	96,6%	98,3%	98,7%	98,4%	93,4%

19272	60	70	20	45	96,6%	98,3%	98,8%	98,5%	93,8%
19273	60	70	20	50	96,7%	98,3%	98,8%	98,6%	94,2%
19274	60	70	20	55	96,7%	98,3%	98,9%	98,7%	94,6%
19275	60	70	20	60	96,8%	98,3%	98,9%	98,8%	94,9%
19276	60	70	20	65	96,9%	98,2%	98,9%	98,8%	95,2%
19277	60	70	20	70	96,9%	98,2%	99,0%	98,9%	95,5%
19278	60	70	20	75	97,0%	98,2%	99,0%	99,0%	95,8%
19279	60	70	20	80	97,0%	98,2%	99,0%	99,1%	96,1%
19280	60	70	25	20	95,4%	98,0%	98,4%	97,7%	91,2%
19281	60	70	25	25	95,4%	98,0%	98,4%	97,9%	91,7%
19282	60	70	25	30	95,5%	98,0%	98,5%	98,0%	92,2%
19283	60	70	25	35	95,6%	98,0%	98,5%	98,2%	92,7%
19284	60	70	25	40	95,7%	97,9%	98,6%	98,3%	93,2%
19285	60	70	25	45	95,7%	97,9%	98,6%	98,4%	93,6%
19286	60	70	25	50	95,8%	97,9%	98,7%	98,5%	94,0%
19287	60	70	25	55	95,9%	97,8%	98,7%	98,6%	94,4%
19288	60	70	25	60	95,9%	97,8%	98,7%	98,7%	94,7%
19289	60	70	25	65	96,0%	97,8%	98,8%	98,8%	95,0%
19290	60	70	25	70	96,1%	97,8%	98,8%	98,9%	95,4%
19291	60	70	25	75	96,1%	97,7%	98,9%	98,9%	95,6%
19292	60	70	25	80	96,2%	97,7%	98,9%	99,0%	95,9%
19293	60	70	30	20	94,1%	97,5%	98,2%	97,6%	90,8%
19294	60	70	30	25	94,2%	97,5%	98,2%	97,8%	91,4%
19295	60	70	30	30	94,3%	97,5%	98,3%	97,9%	91,9%
19296	60	70	30	35	94,4%	97,4%	98,3%	98,0%	92,4%
19297	60	70	30	40	94,5%	97,4%	98,4%	98,2%	92,9%
19298	60	70	30	45	94,6%	97,4%	98,4%	98,3%	93,3%
19299	60	70	30	50	94,7%	97,3%	98,5%	98,4%	93,7%
19300	60	70	30	55	94,8%	97,3%	98,5%	98,5%	94,1%
19301	60	70	30	60	94,9%	97,3%	98,6%	98,6%	94,5%
19302	60	70	30	65	94,9%	97,2%	98,6%	98,7%	94,8%
19303	60	70	30	70	95,0%	97,2%	98,7%	98,8%	95,2%
19304	60	70	30	75	95,1%	97,2%	98,7%	98,9%	95,5%

19305	60	70	30	80	95,2%	97,1%	98,8%	98,9%	95,8%
19306	60	70	35	20	92,6%	96,9%	97,9%	97,4%	90,5%
19307	60	70	35	25	92,7%	96,9%	98,0%	97,6%	91,1%
19308	60	70	35	30	92,8%	96,8%	98,0%	97,8%	91,6%
19309	60	70	35	35	93,0%	96,8%	98,1%	97,9%	92,1%
19310	60	70	35	40	93,1%	96,8%	98,2%	98,1%	92,6%
19311	60	70	35	45	93,2%	96,7%	98,2%	98,2%	93,1%
19312	60	70	35	50	93,3%	96,7%	98,3%	98,3%	93,5%
19313	60	70	35	55	93,4%	96,6%	98,3%	98,4%	93,9%
19314	60	70	35	60	93,5%	96,6%	98,4%	98,5%	94,3%
19315	60	70	35	65	93,6%	96,5%	98,5%	98,6%	94,6%
19316	60	70	35	70	93,7%	96,5%	98,5%	98,7%	95,0%
19317	60	70	35	75	93,8%	96,5%	98,6%	98,8%	95,3%
19318	60	70	35	80	93,9%	96,4%	98,6%	98,9%	95,6%
19319	60	70	40	20	90,7%	96,1%	97,6%	97,3%	90,1%
19320	60	70	40	25	90,9%	96,1%	97,7%	97,5%	90,7%
19321	60	70	40	30	91,0%	96,0%	97,8%	97,6%	91,3%
19322	60	70	40	35	91,2%	96,0%	97,9%	97,8%	91,8%
19323	60	70	40	40	91,3%	95,9%	97,9%	97,9%	92,3%
19324	60	70	40	45	91,4%	95,9%	98,0%	98,1%	92,8%
19325	60	70	40	50	91,6%	95,8%	98,1%	98,2%	93,2%
19326	60	70	40	55	91,7%	95,8%	98,1%	98,3%	93,7%
19327	60	70	40	60	91,8%	95,7%	98,2%	98,4%	94,0%
19328	60	70	40	65	92,0%	95,7%	98,3%	98,5%	94,4%
19329	60	70	40	70	92,1%	95,6%	98,3%	98,6%	94,8%
19330	60	70	40	75	92,2%	95,6%	98,4%	98,7%	95,1%
19331	60	70	40	80	92,4%	95,5%	98,4%	98,8%	95,4%
19332	60	70	45	20	88,4%	95,2%	97,3%	97,1%	89,8%
19333	60	70	45	25	88,6%	95,1%	97,4%	97,3%	90,4%
19334	60	70	45	30	88,8%	95,1%	97,5%	97,5%	91,0%
19335	60	70	45	35	89,0%	95,0%	97,6%	97,7%	91,5%
19336	60	70	45	40	89,1%	94,9%	97,7%	97,8%	92,0%
19337	60	70	45	45	89,3%	94,9%	97,7%	97,9%	92,5%

19338	60	70	45	50	89,5%	94,8%	97,8%	98,1%	93,0%
19339	60	70	45	55	89,6%	94,7%	97,9%	98,2%	93,4%
19340	60	70	45	60	89,8%	94,7%	98,0%	98,3%	93,8%
19341	60	70	45	65	90,0%	94,6%	98,0%	98,4%	94,2%
19342	60	70	45	70	90,1%	94,6%	98,1%	98,5%	94,6%
19343	60	70	45	75	90,3%	94,5%	98,2%	98,6%	94,9%
19344	60	70	45	80	90,4%	94,4%	98,2%	98,7%	95,2%
19345	60	70	50	20	85,7%	94,0%	97,0%	97,0%	89,4%
19346	60	70	50	25	85,9%	93,9%	97,1%	97,1%	90,0%
19347	60	70	50	30	86,1%	93,9%	97,2%	97,3%	90,6%
19348	60	70	50	35	86,3%	93,8%	97,3%	97,5%	91,2%
19349	60	70	50	40	86,5%	93,7%	97,4%	97,7%	91,7%
19350	60	70	50	45	86,7%	93,6%	97,5%	97,8%	92,2%
19351	60	70	50	50	86,9%	93,6%	97,5%	98,0%	92,7%
19352	60	70	50	55	87,1%	93,5%	97,6%	98,1%	93,1%
19353	60	70	50	60	87,3%	93,4%	97,7%	98,2%	93,6%
19354	60	70	50	65	87,5%	93,3%	97,8%	98,3%	94,0%
19355	60	70	50	70	87,7%	93,2%	97,8%	98,4%	94,3%
19356	60	70	50	75	87,9%	93,1%	97,9%	98,5%	94,7%
19357	60	70	50	80	88,1%	93,1%	98,0%	98,6%	95,0%
19358	60	70	55	20	82,3%	92,6%	96,6%	96,8%	89,0%
19359	60	70	55	25	82,6%	92,5%	96,7%	97,0%	89,6%
19360	60	70	55	30	82,8%	92,4%	96,8%	97,2%	90,3%
19361	60	70	55	35	83,1%	92,3%	96,9%	97,4%	90,8%
19362	60	70	55	40	83,3%	92,2%	97,0%	97,5%	91,4%
19363	60	70	55	45	83,6%	92,1%	97,1%	97,7%	91,9%
19364	60	70	55	50	83,8%	92,0%	97,2%	97,8%	92,4%
19365	60	70	55	55	84,1%	91,9%	97,3%	98,0%	92,9%
19366	60	70	55	60	84,3%	91,8%	97,4%	98,1%	93,3%
19367	60	70	55	65	84,5%	91,7%	97,5%	98,2%	93,7%
19368	60	70	55	70	84,8%	91,6%	97,6%	98,4%	94,1%
19369	60	70	55	75	85,0%	91,5%	97,6%	98,5%	94,5%
19370	60	70	55	80	85,2%	91,4%	97,7%	98,6%	94,8%

19371	60	70	60	20	78,5%	90,8%	96,2%	96,6%	88,6%
19372	60	70	60	25	78,8%	90,7%	96,3%	96,8%	89,2%
19373	60	70	60	30	79,1%	90,6%	96,4%	97,0%	89,9%
19374	60	70	60	35	79,3%	90,5%	96,5%	97,2%	90,5%
19375	60	70	60	40	79,6%	90,4%	96,7%	97,4%	91,1%
19376	60	70	60	45	79,9%	90,3%	96,8%	97,5%	91,6%
19377	60	70	60	50	80,2%	90,1%	96,9%	97,7%	92,1%
19378	60	70	60	55	80,5%	90,0%	97,0%	97,9%	92,6%
19379	60	70	60	60	80,7%	89,9%	97,1%	98,0%	93,1%
19380	60	70	60	65	81,0%	89,8%	97,2%	98,1%	93,5%
19381	60	70	60	70	81,3%	89,7%	97,3%	98,2%	93,9%
19382	60	70	60	75	81,6%	89,5%	97,3%	98,4%	94,3%
19383	60	70	60	80	81,8%	89,4%	97,4%	98,5%	94,6%
19384	60	70	65	20	74,0%	88,7%	95,7%	96,4%	88,1%
19385	60	70	65	25	74,3%	88,6%	95,8%	96,6%	88,8%
19386	60	70	65	30	74,7%	88,4%	96,0%	96,8%	89,5%
19387	60	70	65	35	75,0%	88,3%	96,1%	97,0%	90,1%
19388	60	70	65	40	75,3%	88,2%	96,2%	97,2%	90,7%
19389	60	70	65	45	75,7%	88,0%	96,3%	97,4%	91,3%
19390	60	70	65	50	76,0%	87,9%	96,5%	97,6%	91,8%
19391	60	70	65	55	76,3%	87,8%	96,6%	97,7%	92,3%
19392	60	70	65	60	76,6%	87,6%	96,7%	97,9%	92,8%
19393	60	70	65	65	76,9%	87,5%	96,8%	98,0%	93,2%
19394	60	70	65	70	77,2%	87,3%	96,9%	98,1%	93,7%
19395	60	70	65	75	77,6%	87,2%	97,0%	98,3%	94,0%
19396	60	70	65	80	77,9%	87,0%	97,1%	98,4%	94,4%
19397	60	70	70	20	69,0%	86,2%	95,2%	96,1%	87,7%
19398	60	70	70	25	69,4%	86,0%	95,3%	96,4%	88,4%
19399	60	70	70	30	69,7%	85,9%	95,5%	96,6%	89,1%
19400	60	70	70	35	70,1%	85,7%	95,6%	96,8%	89,8%
19401	60	70	70	40	70,5%	85,5%	95,7%	97,0%	90,4%
19402	60	70	70	45	70,8%	85,4%	95,9%	97,2%	90,9%
19403	60	70	70	50	71,2%	85,2%	96,0%	97,4%	91,5%

19404	60	70	70	55	71,6%	85,0%	96,1%	97,6%	92,0%
19405	60	70	70	60	71,9%	84,9%	96,3%	97,7%	92,5%
19406	60	70	70	65	72,3%	84,7%	96,4%	97,9%	93,0%
19407	60	70	70	70	72,6%	84,5%	96,5%	98,0%	93,4%
19408	60	70	70	75	73,0%	84,4%	96,6%	98,2%	93,8%
19409	60	70	70	80	73,3%	84,2%	96,7%	98,3%	94,2%
19410	60	70	75	20	63,5%	83,2%	94,6%	95,9%	87,3%
19411	60	70	75	25	63,9%	83,0%	94,7%	96,2%	88,0%
19412	60	70	75	30	64,3%	82,8%	94,9%	96,4%	88,7%
19413	60	70	75	35	64,7%	82,6%	95,1%	96,6%	89,4%
19414	60	70	75	40	65,1%	82,4%	95,2%	96,9%	90,0%
19415	60	70	75	45	65,5%	82,3%	95,4%	97,1%	90,6%
19416	60	70	75	50	65,9%	82,1%	95,5%	97,3%	91,2%
19417	60	70	75	55	66,3%	81,9%	95,7%	97,4%	91,7%
19418	60	70	75	60	66,7%	81,7%	95,8%	97,6%	92,2%
19419	60	70	75	65	67,1%	81,5%	95,9%	97,8%	92,7%
19420	60	70	75	70	67,4%	81,3%	96,1%	97,9%	93,1%
19421	60	70	75	75	67,8%	81,1%	96,2%	98,0%	93,6%
19422	60	70	75	80	68,2%	80,9%	96,3%	98,2%	94,0%
19423	60	70	80	20	57,6%	79,7%	93,9%	95,7%	86,8%
19424	60	70	80	25	58,0%	79,5%	94,1%	95,9%	87,6%
19425	60	70	80	30	58,4%	79,3%	94,3%	96,2%	88,3%
19426	60	70	80	35	58,9%	79,1%	94,4%	96,4%	89,0%
19427	60	70	80	40	59,3%	78,8%	94,6%	96,7%	89,6%
19428	60	70	80	45	59,7%	78,6%	94,8%	96,9%	90,2%
19429	60	70	80	50	60,1%	78,4%	95,0%	97,1%	90,8%
19430	60	70	80	55	60,6%	78,2%	95,1%	97,3%	91,4%
19431	60	70	80	60	61,0%	78,0%	95,3%	97,5%	91,9%
19432	60	70	80	65	61,4%	77,7%	95,4%	97,6%	92,4%
19433	60	70	80	70	61,8%	77,5%	95,6%	97,8%	92,9%
19434	60	70	80	75	62,2%	77,3%	95,7%	97,9%	93,3%
19435	60	70	80	80	62,6%	77,0%	95,8%	98,1%	93,7%
19436	60	75	20	20	96,6%	98,5%	98,5%	97,7%	90,7%

19437	60	75	20	25	96,7%	98,5%	98,6%	97,9%	91,3%
19438	60	75	20	30	96,7%	98,5%	98,6%	98,0%	91,8%
19439	60	75	20	35	96,8%	98,5%	98,6%	98,1%	92,3%
19440	60	75	20	40	96,9%	98,5%	98,7%	98,2%	92,8%
19441	60	75	20	45	96,9%	98,5%	98,7%	98,4%	93,2%
19442	60	75	20	50	97,0%	98,4%	98,8%	98,5%	93,7%
19443	60	75	20	55	97,0%	98,4%	98,8%	98,6%	94,1%
19444	60	75	20	60	97,1%	98,4%	98,9%	98,7%	94,4%
19445	60	75	20	65	97,1%	98,4%	98,9%	98,8%	94,8%
19446	60	75	20	70	97,2%	98,3%	98,9%	98,8%	95,1%
19447	60	75	20	75	97,2%	98,3%	99,0%	98,9%	95,4%
19448	60	75	20	80	97,3%	98,3%	99,0%	99,0%	95,7%
19449	60	75	25	20	95,7%	98,2%	98,3%	97,6%	90,4%
19450	60	75	25	25	95,8%	98,2%	98,4%	97,7%	91,0%
19451	60	75	25	30	95,9%	98,1%	98,4%	97,9%	91,5%
19452	60	75	25	35	95,9%	98,1%	98,5%	98,0%	92,0%
19453	60	75	25	40	96,0%	98,1%	98,5%	98,1%	92,5%
19454	60	75	25	45	96,1%	98,1%	98,6%	98,3%	93,0%
19455	60	75	25	50	96,1%	98,0%	98,6%	98,4%	93,4%
19456	60	75	25	55	96,2%	98,0%	98,7%	98,5%	93,8%
19457	60	75	25	60	96,3%	98,0%	98,7%	98,6%	94,2%
19458	60	75	25	65	96,3%	98,0%	98,8%	98,7%	94,6%
19459	60	75	25	70	96,4%	97,9%	98,8%	98,8%	94,9%
19460	60	75	25	75	96,5%	97,9%	98,8%	98,8%	95,2%
19461	60	75	25	80	96,5%	97,9%	98,9%	98,9%	95,5%
19462	60	75	30	20	94,6%	97,7%	98,1%	97,4%	90,0%
19463	60	75	30	25	94,7%	97,7%	98,2%	97,6%	90,6%
19464	60	75	30	30	94,8%	97,7%	98,2%	97,7%	91,2%
19465	60	75	30	35	94,9%	97,6%	98,3%	97,9%	91,7%
19466	60	75	30	40	94,9%	97,6%	98,3%	98,0%	92,2%
19467	60	75	30	45	95,0%	97,6%	98,4%	98,2%	92,7%
19468	60	75	30	50	95,1%	97,5%	98,4%	98,3%	93,2%
19469	60	75	30	55	95,2%	97,5%	98,5%	98,4%	93,6%

19470	60	75	30	60	95,3%	97,5%	98,5%	98,5%	94,0%
19471	60	75	30	65	95,4%	97,4%	98,6%	98,6%	94,4%
19472	60	75	30	70	95,4%	97,4%	98,6%	98,7%	94,7%
19473	60	75	30	75	95,5%	97,4%	98,7%	98,8%	95,0%
19474	60	75	30	80	95,6%	97,3%	98,7%	98,9%	95,4%
19475	60	75	35	20	93,2%	97,1%	97,8%	97,3%	89,6%
19476	60	75	35	25	93,3%	97,1%	97,9%	97,4%	90,3%
19477	60	75	35	30	93,4%	97,1%	98,0%	97,6%	90,8%
19478	60	75	35	35	93,5%	97,0%	98,1%	97,8%	91,4%
19479	60	75	35	40	93,6%	97,0%	98,1%	97,9%	91,9%
19480	60	75	35	45	93,7%	97,0%	98,2%	98,0%	92,4%
19481	60	75	35	50	93,8%	96,9%	98,2%	98,2%	92,9%
19482	60	75	35	55	93,9%	96,9%	98,3%	98,3%	93,3%
19483	60	75	35	60	94,0%	96,8%	98,4%	98,4%	93,7%
19484	60	75	35	65	94,1%	96,8%	98,4%	98,5%	94,1%
19485	60	75	35	70	94,2%	96,8%	98,5%	98,6%	94,5%
19486	60	75	35	75	94,3%	96,7%	98,5%	98,7%	94,8%
19487	60	75	35	80	94,4%	96,7%	98,6%	98,8%	95,2%
19488	60	75	40	20	91,4%	96,4%	97,6%	97,1%	89,3%
19489	60	75	40	25	91,6%	96,4%	97,6%	97,3%	89,9%
19490	60	75	40	30	91,7%	96,3%	97,7%	97,5%	90,5%
19491	60	75	40	35	91,8%	96,3%	97,8%	97,6%	91,1%
19492	60	75	40	40	92,0%	96,2%	97,9%	97,8%	91,6%
19493	60	75	40	45	92,1%	96,2%	97,9%	97,9%	92,1%
19494	60	75	40	50	92,2%	96,1%	98,0%	98,1%	92,6%
19495	60	75	40	55	92,4%	96,1%	98,1%	98,2%	93,1%
19496	60	75	40	60	92,5%	96,0%	98,1%	98,3%	93,5%
19497	60	75	40	65	92,6%	96,0%	98,2%	98,4%	93,9%
19498	60	75	40	70	92,7%	95,9%	98,3%	98,5%	94,3%
19499	60	75	40	75	92,8%	95,9%	98,3%	98,6%	94,6%
19500	60	75	40	80	93,0%	95,8%	98,4%	98,7%	95,0%
19501	60	75	45	20	89,3%	95,5%	97,3%	96,9%	88,9%
19502	60	75	45	25	89,5%	95,5%	97,3%	97,1%	89,5%

19503	60	75	45	30	89,6%	95,4%	97,4%	97,3%	90,1%
19504	60	75	45	35	89,8%	95,4%	97,5%	97,5%	90,7%
19505	60	75	45	40	90,0%	95,3%	97,6%	97,6%	91,3%
19506	60	75	45	45	90,1%	95,2%	97,7%	97,8%	91,8%
19507	60	75	45	50	90,3%	95,2%	97,8%	97,9%	92,3%
19508	60	75	45	55	90,4%	95,1%	97,8%	98,1%	92,8%
19509	60	75	45	60	90,6%	95,1%	97,9%	98,2%	93,2%
19510	60	75	45	65	90,7%	95,0%	98,0%	98,3%	93,7%
19511	60	75	45	70	90,9%	94,9%	98,0%	98,4%	94,1%
19512	60	75	45	75	91,0%	94,9%	98,1%	98,5%	94,4%
19513	60	75	45	80	91,2%	94,8%	98,2%	98,6%	94,8%
19514	60	75	50	20	86,7%	94,4%	96,9%	96,7%	88,4%
19515	60	75	50	25	86,9%	94,4%	97,0%	96,9%	89,1%
19516	60	75	50	30	87,1%	94,3%	97,1%	97,1%	89,8%
19517	60	75	50	35	87,3%	94,2%	97,2%	97,3%	90,4%
19518	60	75	50	40	87,5%	94,2%	97,3%	97,5%	91,0%
19519	60	75	50	45	87,7%	94,1%	97,4%	97,7%	91,5%
19520	60	75	50	50	87,9%	94,0%	97,5%	97,8%	92,0%
19521	60	75	50	55	88,1%	93,9%	97,5%	98,0%	92,5%
19522	60	75	50	60	88,2%	93,9%	97,6%	98,1%	93,0%
19523	60	75	50	65	88,4%	93,8%	97,7%	98,2%	93,4%
19524	60	75	50	70	88,6%	93,7%	97,8%	98,3%	93,8%
19525	60	75	50	75	88,8%	93,6%	97,8%	98,4%	94,2%
19526	60	75	50	80	89,0%	93,6%	97,9%	98,5%	94,6%
19527	60	75	55	20	83,6%	93,1%	96,5%	96,5%	88,0%
19528	60	75	55	25	83,8%	93,0%	96,6%	96,8%	88,7%
19529	60	75	55	30	84,1%	92,9%	96,7%	97,0%	89,4%
19530	60	75	55	35	84,3%	92,8%	96,8%	97,2%	90,0%
19531	60	75	55	40	84,5%	92,8%	96,9%	97,3%	90,6%
19532	60	75	55	45	84,8%	92,7%	97,0%	97,5%	91,2%
19533	60	75	55	50	85,0%	92,6%	97,1%	97,7%	91,7%
19534	60	75	55	55	85,2%	92,5%	97,2%	97,8%	92,2%
19535	60	75	55	60	85,4%	92,4%	97,3%	98,0%	92,7%

19536	60	75	55	65	85,7%	92,3%	97,4%	98,1%	93,1%
19537	60	75	55	70	85,9%	92,2%	97,5%	98,2%	93,6%
19538	60	75	55	75	86,1%	92,1%	97,6%	98,3%	94,0%
19539	60	75	55	80	86,3%	92,0%	97,7%	98,5%	94,3%
19540	60	75	60	20	79,9%	91,5%	96,1%	96,3%	87,6%
19541	60	75	60	25	80,2%	91,4%	96,2%	96,6%	88,3%
19542	60	75	60	30	80,5%	91,2%	96,3%	96,8%	89,0%
19543	60	75	60	35	80,8%	91,1%	96,4%	97,0%	89,6%
19544	60	75	60	40	81,0%	91,0%	96,5%	97,2%	90,3%
19545	60	75	60	45	81,3%	90,9%	96,7%	97,4%	90,8%
19546	60	75	60	50	81,6%	90,8%	96,8%	97,5%	91,4%
19547	60	75	60	55	81,8%	90,7%	96,9%	97,7%	91,9%
19548	60	75	60	60	82,1%	90,6%	97,0%	97,8%	92,4%
19549	60	75	60	65	82,3%	90,5%	97,1%	98,0%	92,9%
19550	60	75	60	70	82,6%	90,4%	97,2%	98,1%	93,3%
19551	60	75	60	75	82,9%	90,3%	97,3%	98,2%	93,7%
19552	60	75	60	80	83,1%	90,1%	97,3%	98,4%	94,1%
19553	60	75	65	20	75,7%	89,5%	95,6%	96,1%	87,1%
19554	60	75	65	25	76,0%	89,3%	95,7%	96,3%	87,9%
19555	60	75	65	30	76,3%	89,2%	95,8%	96,6%	88,6%
19556	60	75	65	35	76,6%	89,1%	96,0%	96,8%	89,2%
19557	60	75	65	40	76,9%	89,0%	96,1%	97,0%	89,9%
19558	60	75	65	45	77,3%	88,8%	96,2%	97,2%	90,5%
19559	60	75	65	50	77,6%	88,7%	96,4%	97,4%	91,1%
19560	60	75	65	55	77,9%	88,6%	96,5%	97,6%	91,6%
19561	60	75	65	60	78,2%	88,4%	96,6%	97,7%	92,1%
19562	60	75	65	65	78,5%	88,3%	96,7%	97,9%	92,6%
19563	60	75	65	70	78,8%	88,2%	96,8%	98,0%	93,1%
19564	60	75	65	75	79,1%	88,0%	96,9%	98,1%	93,5%
19565	60	75	65	80	79,3%	87,9%	97,0%	98,3%	93,9%
19566	60	75	70	20	70,8%	87,1%	95,0%	95,9%	86,6%
19567	60	75	70	25	71,2%	86,9%	95,2%	96,1%	87,4%
19568	60	75	70	30	71,6%	86,8%	95,3%	96,4%	88,1%

19569	60	75	70	35	71,9%	86,6%	95,5%	96,6%	88,8%
19570	60	75	70	40	72,3%	86,5%	95,6%	96,8%	89,5%
19571	60	75	70	45	72,6%	86,3%	95,8%	97,0%	90,1%
19572	60	75	70	50	73,0%	86,2%	95,9%	97,2%	90,7%
19573	60	75	70	55	73,3%	86,0%	96,0%	97,4%	91,3%
19574	60	75	70	60	73,7%	85,9%	96,2%	97,6%	91,8%
19575	60	75	70	65	74,0%	85,7%	96,3%	97,7%	92,3%
19576	60	75	70	70	74,3%	85,5%	96,4%	97,9%	92,8%
19577	60	75	70	75	74,7%	85,4%	96,5%	98,0%	93,2%
19578	60	75	70	80	75,0%	85,2%	96,6%	98,1%	93,7%
19579	60	75	75	20	65,5%	84,3%	94,4%	95,6%	86,2%
19580	60	75	75	25	65,9%	84,1%	94,6%	95,9%	87,0%
19581	60	75	75	30	66,3%	83,9%	94,7%	96,2%	87,7%
19582	60	75	75	35	66,7%	83,7%	94,9%	96,4%	88,4%
19583	60	75	75	40	67,1%	83,6%	95,1%	96,6%	89,1%
19584	60	75	75	45	67,5%	83,4%	95,2%	96,8%	89,8%
19585	60	75	75	50	67,8%	83,2%	95,4%	97,1%	90,4%
19586	60	75	75	55	68,2%	83,0%	95,5%	97,2%	91,0%
19587	60	75	75	60	68,6%	82,8%	95,7%	97,4%	91,5%
19588	60	75	75	65	69,0%	82,6%	95,8%	97,6%	92,0%
19589	60	75	75	70	69,4%	82,4%	95,9%	97,7%	92,5%
19590	60	75	75	75	69,7%	82,3%	96,1%	97,9%	93,0%
19591	60	75	75	80	70,1%	82,1%	96,2%	98,0%	93,4%
19592	60	75	80	20	59,7%	80,9%	93,7%	95,3%	85,7%
19593	60	75	80	25	60,1%	80,7%	93,9%	95,6%	86,5%
19594	60	75	80	30	60,6%	80,5%	94,1%	95,9%	87,3%
19595	60	75	80	35	61,0%	80,3%	94,3%	96,2%	88,0%
19596	60	75	80	40	61,4%	80,1%	94,5%	96,4%	88,7%
19597	60	75	80	45	61,8%	79,9%	94,6%	96,7%	89,4%
19598	60	75	80	50	62,2%	79,7%	94,8%	96,9%	90,0%
19599	60	75	80	55	62,7%	79,5%	95,0%	97,1%	90,6%
19600	60	75	80	60	63,1%	79,3%	95,1%	97,3%	91,2%
19601	60	75	80	65	63,5%	79,1%	95,3%	97,4%	91,7%

19602	60	75	80	70	63,9%	78,8%	95,4%	97,6%	92,2%
19603	60	75	80	75	64,3%	78,6%	95,6%	97,8%	92,7%
19604	60	75	80	80	64,7%	78,4%	95,7%	97,9%	93,1%
19605	60	80	20	20	96,9%	98,7%	98,5%	97,5%	89,9%
19606	60	80	20	25	97,0%	98,6%	98,5%	97,7%	90,5%
19607	60	80	20	30	97,0%	98,6%	98,6%	97,8%	91,1%
19608	60	80	20	35	97,1%	98,6%	98,6%	98,0%	91,6%
19609	60	80	20	40	97,1%	98,6%	98,7%	98,1%	92,1%
19610	60	80	20	45	97,2%	98,6%	98,7%	98,2%	92,6%
19611	60	80	20	50	97,2%	98,5%	98,7%	98,4%	93,1%
19612	60	80	20	55	97,3%	98,5%	98,8%	98,5%	93,5%
19613	60	80	20	60	97,3%	98,5%	98,8%	98,6%	93,9%
19614	60	80	20	65	97,4%	98,5%	98,9%	98,7%	94,3%
19615	60	80	20	70	97,4%	98,5%	98,9%	98,7%	94,6%
19616	60	80	20	75	97,4%	98,5%	98,9%	98,8%	95,0%
19617	60	80	20	80	97,5%	98,4%	99,0%	98,9%	95,3%
19618	60	80	25	20	96,1%	98,3%	98,3%	97,4%	89,5%
19619	60	80	25	25	96,1%	98,3%	98,3%	97,6%	90,1%
19620	60	80	25	30	96,2%	98,3%	98,4%	97,7%	90,7%
19621	60	80	25	35	96,3%	98,2%	98,4%	97,9%	91,3%
19622	60	80	25	40	96,3%	98,2%	98,5%	98,0%	91,8%
19623	60	80	25	45	96,4%	98,2%	98,5%	98,1%	92,3%
19624	60	80	25	50	96,5%	98,2%	98,6%	98,3%	92,8%
19625	60	80	25	55	96,5%	98,2%	98,6%	98,4%	93,2%
19626	60	80	25	60	96,6%	98,1%	98,7%	98,5%	93,7%
19627	60	80	25	65	96,6%	98,1%	98,7%	98,6%	94,1%
19628	60	80	25	70	96,7%	98,1%	98,8%	98,7%	94,4%
19629	60	80	25	75	96,7%	98,1%	98,8%	98,8%	94,8%
19630	60	80	25	80	96,8%	98,0%	98,8%	98,8%	95,1%
19631	60	80	30	20	95,0%	97,9%	98,0%	97,2%	89,1%
19632	60	80	30	25	95,1%	97,9%	98,1%	97,4%	89,8%
19633	60	80	30	30	95,2%	97,8%	98,2%	97,6%	90,4%
19634	60	80	30	35	95,3%	97,8%	98,2%	97,7%	91,0%

19635	60	80	30	40	95,4%	97,8%	98,3%	97,9%	91,5%
19636	60	80	30	45	95,4%	97,7%	98,3%	98,0%	92,0%
19637	60	80	30	50	95,5%	97,7%	98,4%	98,1%	92,5%
19638	60	80	30	55	95,6%	97,7%	98,4%	98,3%	93,0%
19639	60	80	30	60	95,7%	97,7%	98,5%	98,4%	93,4%
19640	60	80	30	65	95,7%	97,6%	98,5%	98,5%	93,8%
19641	60	80	30	70	95,8%	97,6%	98,6%	98,6%	94,2%
19642	60	80	30	75	95,9%	97,6%	98,6%	98,7%	94,6%
19643	60	80	30	80	95,9%	97,5%	98,7%	98,8%	94,9%
19644	60	80	35	20	93,7%	97,4%	97,8%	97,1%	88,7%
19645	60	80	35	25	93,8%	97,3%	97,9%	97,2%	89,4%
19646	60	80	35	30	93,9%	97,3%	97,9%	97,4%	90,0%
19647	60	80	35	35	94,0%	97,2%	98,0%	97,6%	90,6%
19648	60	80	35	40	94,1%	97,2%	98,1%	97,7%	91,2%
19649	60	80	35	45	94,2%	97,2%	98,1%	97,9%	91,7%
19650	60	80	35	50	94,3%	97,1%	98,2%	98,0%	92,2%
19651	60	80	35	55	94,4%	97,1%	98,2%	98,2%	92,7%
19652	60	80	35	60	94,5%	97,1%	98,3%	98,3%	93,2%
19653	60	80	35	65	94,6%	97,0%	98,4%	98,4%	93,6%
19654	60	80	35	70	94,7%	97,0%	98,4%	98,5%	94,0%
19655	60	80	35	75	94,8%	97,0%	98,5%	98,6%	94,4%
19656	60	80	35	80	94,9%	96,9%	98,5%	98,7%	94,7%
19657	60	80	40	20	92,1%	96,7%	97,5%	96,9%	88,3%
19658	60	80	40	25	92,2%	96,6%	97,6%	97,1%	89,0%
19659	60	80	40	30	92,4%	96,6%	97,7%	97,3%	89,6%
19660	60	80	40	35	92,5%	96,6%	97,7%	97,4%	90,3%
19661	60	80	40	40	92,6%	96,5%	97,8%	97,6%	90,8%
19662	60	80	40	45	92,7%	96,5%	97,9%	97,8%	91,4%
19663	60	80	40	50	92,8%	96,4%	97,9%	97,9%	91,9%
19664	60	80	40	55	93,0%	96,4%	98,0%	98,0%	92,4%
19665	60	80	40	60	93,1%	96,3%	98,1%	98,2%	92,9%
19666	60	80	40	65	93,2%	96,3%	98,1%	98,3%	93,3%
19667	60	80	40	70	93,3%	96,2%	98,2%	98,4%	93,7%

19668	60	80	40	75	93,4%	96,2%	98,3%	98,5%	94,1%
19669	60	80	40	80	93,5%	96,1%	98,3%	98,6%	94,5%
19670	60	80	45	20	90,1%	95,9%	97,2%	96,7%	87,9%
19671	60	80	45	25	90,3%	95,8%	97,3%	96,9%	88,6%
19672	60	80	45	30	90,4%	95,8%	97,4%	97,1%	89,3%
19673	60	80	45	35	90,6%	95,7%	97,4%	97,3%	89,9%
19674	60	80	45	40	90,7%	95,6%	97,5%	97,5%	90,5%
19675	60	80	45	45	90,9%	95,6%	97,6%	97,6%	91,1%
19676	60	80	45	50	91,0%	95,5%	97,7%	97,8%	91,6%
19677	60	80	45	55	91,2%	95,5%	97,8%	97,9%	92,1%
19678	60	80	45	60	91,3%	95,4%	97,8%	98,1%	92,6%
19679	60	80	45	65	91,4%	95,4%	97,9%	98,2%	93,1%
19680	60	80	45	70	91,6%	95,3%	98,0%	98,3%	93,5%
19681	60	80	45	75	91,7%	95,2%	98,0%	98,4%	93,9%
19682	60	80	45	80	91,9%	95,2%	98,1%	98,5%	94,3%
19683	60	80	50	20	87,7%	94,8%	96,8%	96,5%	87,4%
19684	60	80	50	25	87,9%	94,8%	96,9%	96,7%	88,2%
19685	60	80	50	30	88,1%	94,7%	97,0%	96,9%	88,9%
19686	60	80	50	35	88,3%	94,6%	97,1%	97,1%	89,5%
19687	60	80	50	40	88,4%	94,6%	97,2%	97,3%	90,1%
19688	60	80	50	45	88,6%	94,5%	97,3%	97,5%	90,7%
19689	60	80	50	50	88,8%	94,4%	97,4%	97,6%	91,3%
19690	60	80	50	55	89,0%	94,4%	97,5%	97,8%	91,8%
19691	60	80	50	60	89,1%	94,3%	97,5%	97,9%	92,3%
19692	60	80	50	65	89,3%	94,2%	97,6%	98,1%	92,8%
19693	60	80	50	70	89,5%	94,2%	97,7%	98,2%	93,2%
19694	60	80	50	75	89,6%	94,1%	97,8%	98,3%	93,7%
19695	60	80	50	80	89,8%	94,0%	97,9%	98,4%	94,1%
19696	60	80	55	20	84,8%	93,6%	96,4%	96,3%	87,0%
19697	60	80	55	25	85,0%	93,5%	96,5%	96,5%	87,7%
19698	60	80	55	30	85,2%	93,4%	96,6%	96,7%	88,4%
19699	60	80	55	35	85,4%	93,3%	96,7%	96,9%	89,1%
19700	60	80	55	40	85,7%	93,3%	96,8%	97,1%	89,8%

19701	60	80	55	45	85,9%	93,2%	96,9%	97,3%	90,4%
19702	60	80	55	50	86,1%	93,1%	97,0%	97,5%	91,0%
19703	60	80	55	55	86,3%	93,0%	97,1%	97,7%	91,5%
19704	60	80	55	60	86,5%	92,9%	97,2%	97,8%	92,0%
19705	60	80	55	65	86,7%	92,8%	97,3%	98,0%	92,5%
19706	60	80	55	70	86,9%	92,8%	97,4%	98,1%	93,0%
19707	60	80	55	75	87,1%	92,7%	97,5%	98,2%	93,4%
19708	60	80	55	80	87,3%	92,6%	97,6%	98,3%	93,8%
19709	60	80	60	20	81,3%	92,1%	95,9%	96,0%	86,5%
19710	60	80	60	25	81,6%	92,0%	96,1%	96,3%	87,3%
19711	60	80	60	30	81,8%	91,9%	96,2%	96,5%	88,0%
19712	60	80	60	35	82,1%	91,8%	96,3%	96,8%	88,7%
19713	60	80	60	40	82,4%	91,7%	96,4%	97,0%	89,4%
19714	60	80	60	45	82,6%	91,6%	96,6%	97,2%	90,0%
19715	60	80	60	50	82,9%	91,5%	96,7%	97,3%	90,6%
19716	60	80	60	55	83,1%	91,4%	96,8%	97,5%	91,2%
19717	60	80	60	60	83,4%	91,2%	96,9%	97,7%	91,7%
19718	60	80	60	65	83,6%	91,1%	97,0%	97,8%	92,2%
19719	60	80	60	70	83,8%	91,0%	97,1%	98,0%	92,7%
19720	60	80	60	75	84,1%	90,9%	97,2%	98,1%	93,1%
19721	60	80	60	80	84,3%	90,8%	97,3%	98,2%	93,6%
19722	60	80	65	20	77,3%	90,2%	95,4%	95,8%	86,0%
19723	60	80	65	25	77,6%	90,1%	95,6%	96,1%	86,8%
19724	60	80	65	30	77,9%	90,0%	95,7%	96,3%	87,6%
19725	60	80	65	35	78,2%	89,8%	95,9%	96,6%	88,3%
19726	60	80	65	40	78,5%	89,7%	96,0%	96,8%	89,0%
19727	60	80	65	45	78,8%	89,6%	96,1%	97,0%	89,6%
19728	60	80	65	50	79,1%	89,5%	96,2%	97,2%	90,3%
19729	60	80	65	55	79,4%	89,3%	96,4%	97,4%	90,8%
19730	60	80	65	60	79,6%	89,2%	96,5%	97,5%	91,4%
19731	60	80	65	65	79,9%	89,1%	96,6%	97,7%	91,9%
19732	60	80	65	70	80,2%	89,0%	96,7%	97,9%	92,4%
19733	60	80	65	75	80,5%	88,8%	96,8%	98,0%	92,9%

19734	60	80	65	80	80,8%	88,7%	96,9%	98,1%	93,3%
19735	60	80	70	20	72,6%	87,9%	94,9%	95,6%	85,5%
19736	60	80	70	25	73,0%	87,8%	95,0%	95,8%	86,3%
19737	60	80	70	30	73,3%	87,7%	95,2%	96,1%	87,1%
19738	60	80	70	35	73,7%	87,5%	95,3%	96,4%	87,9%
19739	60	80	70	40	74,0%	87,4%	95,5%	96,6%	88,6%
19740	60	80	70	45	74,4%	87,2%	95,6%	96,8%	89,2%
19741	60	80	70	50	74,7%	87,1%	95,8%	97,0%	89,9%
19742	60	80	70	55	75,0%	86,9%	95,9%	97,2%	90,5%
19743	60	80	70	60	75,4%	86,8%	96,0%	97,4%	91,1%
19744	60	80	70	65	75,7%	86,6%	96,2%	97,6%	91,6%
19745	60	80	70	70	76,0%	86,5%	96,3%	97,7%	92,1%
19746	60	80	70	75	76,3%	86,3%	96,4%	97,9%	92,6%
19747	60	80	70	80	76,6%	86,2%	96,5%	98,0%	93,1%
19748	60	80	75	20	67,5%	85,3%	94,2%	95,3%	85,0%
19749	60	80	75	25	67,9%	85,1%	94,4%	95,6%	85,8%
19750	60	80	75	30	68,2%	84,9%	94,6%	95,9%	86,6%
19751	60	80	75	35	68,6%	84,8%	94,7%	96,1%	87,4%
19752	60	80	75	40	69,0%	84,6%	94,9%	96,4%	88,1%
19753	60	80	75	45	69,4%	84,4%	95,1%	96,6%	88,8%
19754	60	80	75	50	69,7%	84,3%	95,2%	96,8%	89,5%
19755	60	80	75	55	70,1%	84,1%	95,4%	97,0%	90,1%
19756	60	80	75	60	70,5%	83,9%	95,5%	97,2%	90,7%
19757	60	80	75	65	70,9%	83,7%	95,7%	97,4%	91,3%
19758	60	80	75	70	71,2%	83,6%	95,8%	97,6%	91,8%
19759	60	80	75	75	71,6%	83,4%	95,9%	97,7%	92,3%
19760	60	80	75	80	71,9%	83,2%	96,1%	97,9%	92,8%
19761	60	80	80	20	61,8%	82,1%	93,5%	95,0%	84,4%
19762	60	80	80	25	62,3%	81,9%	93,7%	95,3%	85,3%
19763	60	80	80	30	62,7%	81,7%	93,9%	95,6%	86,2%
19764	60	80	80	35	63,1%	81,6%	94,1%	95,9%	87,0%
19765	60	80	80	40	63,5%	81,4%	94,3%	96,2%	87,7%
19766	60	80	80	45	63,9%	81,2%	94,5%	96,4%	88,4%

19767	60	80	80	50	64,3%	81,0%	94,6%	96,6%	89,1%
19768	60	80	80	55	64,7%	80,8%	94,8%	96,9%	89,8%
19769	60	80	80	60	65,1%	80,5%	95,0%	97,1%	90,4%
19770	60	80	80	65	65,5%	80,3%	95,1%	97,3%	91,0%
19771	60	80	80	70	65,9%	80,1%	95,3%	97,4%	91,5%
19772	60	80	80	75	66,3%	79,9%	95,4%	97,6%	92,0%
19773	60	80	80	80	66,7%	79,7%	95,6%	97,8%	92,5%
19774	65	20	20	20	93,3%	97,6%	99,3%	99,3%	97,4%
19775	65	20	20	25	93,4%	97,5%	99,3%	99,4%	97,6%
19776	65	20	20	30	93,5%	97,5%	99,3%	99,4%	97,7%
19777	65	20	20	35	93,6%	97,5%	99,4%	99,4%	97,9%
19778	65	20	20	40	93,7%	97,4%	99,4%	99,5%	98,0%
19779	65	20	20	45	93,8%	97,4%	99,4%	99,5%	98,2%
19780	65	20	20	50	93,9%	97,4%	99,4%	99,5%	98,3%
19781	65	20	20	55	94,0%	97,3%	99,4%	99,6%	98,4%
19782	65	20	20	60	94,1%	97,3%	99,5%	99,6%	98,5%
19783	65	20	20	65	94,2%	97,3%	99,5%	99,6%	98,6%
19784	65	20	20	70	94,3%	97,2%	99,5%	99,7%	98,7%
19785	65	20	20	75	94,4%	97,2%	99,5%	99,7%	98,8%
19786	65	20	20	80	94,5%	97,2%	99,5%	99,7%	98,9%
19787	65	20	25	20	91,5%	96,9%	99,2%	99,3%	97,3%
19788	65	20	25	25	91,7%	96,9%	99,2%	99,3%	97,5%
19789	65	20	25	30	91,8%	96,9%	99,3%	99,4%	97,7%
19790	65	20	25	35	91,9%	96,8%	99,3%	99,4%	97,8%
19791	65	20	25	40	92,1%	96,8%	99,3%	99,4%	97,9%
19792	65	20	25	45	92,2%	96,7%	99,3%	99,5%	98,1%
19793	65	20	25	50	92,3%	96,7%	99,4%	99,5%	98,2%
19794	65	20	25	55	92,4%	96,7%	99,4%	99,5%	98,3%
19795	65	20	25	60	92,6%	96,6%	99,4%	99,6%	98,4%
19796	65	20	25	65	92,7%	96,6%	99,4%	99,6%	98,5%
19797	65	20	25	70	92,8%	96,5%	99,4%	99,6%	98,6%
19798	65	20	25	75	92,9%	96,5%	99,5%	99,7%	98,7%
19799	65	20	25	80	93,0%	96,4%	99,5%	99,7%	98,8%

19800	65	20	30	20	89,4%	96,2%	99,1%	99,2%	97,2%
19801	65	20	30	25	89,6%	96,1%	99,1%	99,3%	97,4%
19802	65	20	30	30	89,7%	96,1%	99,2%	99,3%	97,6%
19803	65	20	30	35	89,9%	96,0%	99,2%	99,4%	97,7%
19804	65	20	30	40	90,0%	96,0%	99,2%	99,4%	97,9%
19805	65	20	30	45	90,2%	95,9%	99,2%	99,4%	98,0%
19806	65	20	30	50	90,4%	95,9%	99,3%	99,5%	98,1%
19807	65	20	30	55	90,5%	95,8%	99,3%	99,5%	98,3%
19808	65	20	30	60	90,7%	95,8%	99,3%	99,5%	98,4%
19809	65	20	30	65	90,8%	95,7%	99,3%	99,6%	98,5%
19810	65	20	30	70	91,0%	95,7%	99,4%	99,6%	98,6%
19811	65	20	30	75	91,1%	95,6%	99,4%	99,6%	98,7%
19812	65	20	30	80	91,2%	95,6%	99,4%	99,7%	98,8%
19813	65	20	35	20	86,8%	95,2%	99,0%	99,2%	97,1%
19814	65	20	35	25	87,0%	95,2%	99,0%	99,2%	97,3%
19815	65	20	35	30	87,2%	95,1%	99,1%	99,3%	97,5%
19816	65	20	35	35	87,4%	95,0%	99,1%	99,3%	97,6%
19817	65	20	35	40	87,6%	95,0%	99,1%	99,4%	97,8%
19818	65	20	35	45	87,8%	94,9%	99,1%	99,4%	97,9%
19819	65	20	35	50	88,0%	94,9%	99,2%	99,5%	98,1%
19820	65	20	35	55	88,2%	94,8%	99,2%	99,5%	98,2%
19821	65	20	35	60	88,4%	94,7%	99,2%	99,5%	98,3%
19822	65	20	35	65	88,5%	94,7%	99,3%	99,6%	98,4%
19823	65	20	35	70	88,7%	94,6%	99,3%	99,6%	98,5%
19824	65	20	35	75	88,9%	94,5%	99,3%	99,6%	98,6%
19825	65	20	35	80	89,1%	94,5%	99,3%	99,6%	98,7%
19826	65	20	40	20	83,7%	94,1%	98,9%	99,1%	97,0%
19827	65	20	40	25	84,0%	94,0%	98,9%	99,2%	97,2%
19828	65	20	40	30	84,2%	93,9%	98,9%	99,2%	97,4%
19829	65	20	40	35	84,4%	93,8%	99,0%	99,3%	97,5%
19830	65	20	40	40	84,7%	93,8%	99,0%	99,3%	97,7%
19831	65	20	40	45	84,9%	93,7%	99,0%	99,4%	97,8%
19832	65	20	40	50	85,1%	93,6%	99,1%	99,4%	98,0%

19833	65	20	40	55	85,3%	93,5%	99,1%	99,5%	98,1%
19834	65	20	40	60	85,6%	93,4%	99,1%	99,5%	98,2%
19835	65	20	40	65	85,8%	93,4%	99,2%	99,5%	98,3%
19836	65	20	40	70	86,0%	93,3%	99,2%	99,6%	98,5%
19837	65	20	40	75	86,2%	93,2%	99,2%	99,6%	98,6%
19838	65	20	40	80	86,4%	93,1%	99,2%	99,6%	98,6%
19839	65	20	45	20	80,1%	92,6%	98,7%	99,1%	96,9%
19840	65	20	45	25	80,4%	92,5%	98,8%	99,1%	97,1%
19841	65	20	45	30	80,6%	92,4%	98,8%	99,2%	97,2%
19842	65	20	45	35	80,9%	92,4%	98,8%	99,2%	97,4%
19843	65	20	45	40	81,2%	92,3%	98,9%	99,3%	97,6%
19844	65	20	45	45	81,5%	92,2%	98,9%	99,3%	97,7%
19845	65	20	45	50	81,7%	92,1%	98,9%	99,4%	97,9%
19846	65	20	45	55	82,0%	92,0%	99,0%	99,4%	98,0%
19847	65	20	45	60	82,2%	91,9%	99,0%	99,5%	98,2%
19848	65	20	45	65	82,5%	91,8%	99,0%	99,5%	98,3%
19849	65	20	45	70	82,7%	91,7%	99,1%	99,5%	98,4%
19850	65	20	45	75	83,0%	91,6%	99,1%	99,6%	98,5%
19851	65	20	45	80	83,2%	91,5%	99,1%	99,6%	98,6%
19852	65	20	50	20	75,9%	90,9%	98,5%	99,0%	96,7%
19853	65	20	50	25	76,2%	90,8%	98,6%	99,1%	96,9%
19854	65	20	50	30	76,5%	90,7%	98,6%	99,1%	97,1%
19855	65	20	50	35	76,8%	90,6%	98,7%	99,2%	97,3%
19856	65	20	50	40	77,1%	90,4%	98,7%	99,2%	97,5%
19857	65	20	50	45	77,4%	90,3%	98,8%	99,3%	97,7%
19858	65	20	50	50	77,7%	90,2%	98,8%	99,3%	97,8%
19859	65	20	50	55	78,0%	90,1%	98,8%	99,4%	97,9%
19860	65	20	50	60	78,3%	90,0%	98,9%	99,4%	98,1%
19861	65	20	50	65	78,6%	89,9%	98,9%	99,5%	98,2%
19862	65	20	50	70	78,9%	89,7%	99,0%	99,5%	98,3%
19863	65	20	50	75	79,2%	89,6%	99,0%	99,5%	98,4%
19864	65	20	50	80	79,5%	89,5%	99,0%	99,6%	98,5%
19865	65	20	55	20	71,0%	88,8%	98,4%	98,9%	96,6%

19866	65	20	55	25	71,4%	88,7%	98,4%	99,0%	96,8%
19867	65	20	55	30	71,8%	88,5%	98,5%	99,1%	97,0%
19868	65	20	55	35	72,1%	88,4%	98,5%	99,1%	97,2%
19869	65	20	55	40	72,5%	88,3%	98,6%	99,2%	97,4%
19870	65	20	55	45	72,8%	88,1%	98,6%	99,3%	97,6%
19871	65	20	55	50	73,2%	88,0%	98,7%	99,3%	97,7%
19872	65	20	55	55	73,5%	87,8%	98,7%	99,3%	97,9%
19873	65	20	55	60	73,9%	87,7%	98,7%	99,4%	98,0%
19874	65	20	55	65	74,2%	87,6%	98,8%	99,4%	98,1%
19875	65	20	55	70	74,5%	87,4%	98,8%	99,5%	98,3%
19876	65	20	55	75	74,9%	87,3%	98,9%	99,5%	98,4%
19877	65	20	55	80	75,2%	87,1%	98,9%	99,5%	98,5%
19878	65	20	60	20	65,7%	86,3%	98,1%	98,9%	96,5%
19879	65	20	60	25	66,1%	86,1%	98,2%	99,0%	96,7%
19880	65	20	60	30	66,5%	86,0%	98,3%	99,0%	96,9%
19881	65	20	60	35	66,9%	85,8%	98,3%	99,1%	97,1%
19882	65	20	60	40	67,3%	85,6%	98,4%	99,1%	97,3%
19883	65	20	60	45	67,7%	85,5%	98,4%	99,2%	97,5%
19884	65	20	60	50	68,1%	85,3%	98,5%	99,3%	97,6%
19885	65	20	60	55	68,4%	85,2%	98,5%	99,3%	97,8%
19886	65	20	60	60	68,8%	85,0%	98,6%	99,4%	97,9%
19887	65	20	60	65	69,2%	84,8%	98,6%	99,4%	98,1%
19888	65	20	60	70	69,6%	84,7%	98,7%	99,4%	98,2%
19889	65	20	60	75	69,9%	84,5%	98,7%	99,5%	98,3%
19890	65	20	60	80	70,3%	84,3%	98,8%	99,5%	98,4%
19891	65	20	65	20	60,0%	83,3%	97,9%	98,8%	96,3%
19892	65	20	65	25	60,4%	83,1%	98,0%	98,9%	96,5%
19893	65	20	65	30	60,8%	82,9%	98,0%	99,0%	96,8%
19894	65	20	65	35	61,2%	82,8%	98,1%	99,0%	97,0%
19895	65	20	65	40	61,6%	82,6%	98,2%	99,1%	97,2%
19896	65	20	65	45	62,1%	82,4%	98,2%	99,2%	97,4%
19897	65	20	65	50	62,5%	82,2%	98,3%	99,2%	97,5%
19898	65	20	65	55	62,9%	82,0%	98,3%	99,3%	97,7%

19899	65	20	65	60	63,3%	81,8%	98,4%	99,3%	97,8%
19900	65	20	65	65	63,7%	81,6%	98,4%	99,4%	98,0%
19901	65	20	65	70	64,1%	81,4%	98,5%	99,4%	98,1%
19902	65	20	65	75	64,5%	81,2%	98,5%	99,4%	98,2%
19903	65	20	65	80	64,9%	81,0%	98,6%	99,5%	98,3%
19904	65	20	70	20	53,9%	79,8%	97,6%	98,7%	96,2%
19905	65	20	70	25	54,4%	79,6%	97,7%	98,8%	96,4%
19906	65	20	70	30	54,8%	79,4%	97,8%	98,9%	96,6%
19907	65	20	70	35	55,2%	79,2%	97,8%	99,0%	96,9%
19908	65	20	70	40	55,7%	79,0%	97,9%	99,0%	97,1%
19909	65	20	70	45	56,1%	78,8%	98,0%	99,1%	97,2%
19910	65	20	70	50	56,5%	78,6%	98,1%	99,2%	97,4%
19911	65	20	70	55	57,0%	78,3%	98,1%	99,2%	97,6%
19912	65	20	70	60	57,4%	78,1%	98,2%	99,3%	97,7%
19913	65	20	70	65	57,8%	77,9%	98,2%	99,3%	97,9%
19914	65	20	70	70	58,3%	77,7%	98,3%	99,4%	98,0%
19915	65	20	70	75	58,7%	77,4%	98,4%	99,4%	98,2%
19916	65	20	70	80	59,1%	77,2%	98,4%	99,4%	98,3%
19917	65	20	75	20	47,8%	75,9%	97,3%	98,7%	96,0%
19918	65	20	75	25	48,2%	75,6%	97,4%	98,7%	96,3%
19919	65	20	75	30	48,6%	75,4%	97,5%	98,8%	96,5%
19920	65	20	75	35	49,1%	75,1%	97,6%	98,9%	96,7%
19921	65	20	75	40	49,5%	74,9%	97,7%	99,0%	96,9%
19922	65	20	75	45	50,0%	74,7%	97,7%	99,0%	97,1%
19923	65	20	75	50	50,4%	74,4%	97,8%	99,1%	97,3%
19924	65	20	75	55	50,8%	74,2%	97,9%	99,2%	97,5%
19925	65	20	75	60	51,3%	73,9%	97,9%	99,2%	97,7%
19926	65	20	75	65	51,7%	73,7%	98,0%	99,3%	97,8%
19927	65	20	75	70	52,2%	73,4%	98,1%	99,3%	97,9%
19928	65	20	75	75	52,6%	73,1%	98,1%	99,4%	98,1%
19929	65	20	75	80	53,0%	72,9%	98,2%	99,4%	98,2%
19930	65	20	80	20	41,7%	71,4%	97,0%	98,6%	95,8%
19931	65	20	80	25	42,1%	71,1%	97,1%	98,7%	96,1%

19932	65	20	80	30	42,5%	70,9%	97,2%	98,8%	96,4%
19933	65	20	80	35	42,9%	70,6%	97,3%	98,8%	96,6%
19934	65	20	80	40	43,4%	70,3%	97,3%	98,9%	96,8%
19935	65	20	80	45	43,8%	70,0%	97,4%	99,0%	97,0%
19936	65	20	80	50	44,2%	69,8%	97,5%	99,1%	97,2%
19937	65	20	80	55	44,7%	69,5%	97,6%	99,1%	97,4%
19938	65	20	80	60	45,1%	69,2%	97,7%	99,2%	97,6%
19939	65	20	80	65	45,6%	68,9%	97,8%	99,2%	97,7%
19940	65	20	80	70	46,0%	68,7%	97,8%	99,3%	97,9%
19941	65	20	80	75	46,4%	68,4%	97,9%	99,3%	98,0%
19942	65	20	80	80	46,9%	68,1%	98,0%	99,4%	98,1%
19943	65	25	20	20	93,8%	97,7%	99,3%	99,3%	97,2%
19944	65	25	20	25	93,9%	97,7%	99,3%	99,3%	97,4%
19945	65	25	20	30	94,0%	97,7%	99,3%	99,4%	97,5%
19946	65	25	20	35	94,1%	97,6%	99,3%	99,4%	97,7%
19947	65	25	20	40	94,2%	97,6%	99,4%	99,4%	97,8%
19948	65	25	20	45	94,3%	97,6%	99,4%	99,5%	98,0%
19949	65	25	20	50	94,4%	97,6%	99,4%	99,5%	98,1%
19950	65	25	20	55	94,5%	97,5%	99,4%	99,5%	98,2%
19951	65	25	20	60	94,6%	97,5%	99,5%	99,6%	98,3%
19952	65	25	20	65	94,7%	97,5%	99,5%	99,6%	98,5%
19953	65	25	20	70	94,7%	97,4%	99,5%	99,6%	98,6%
19954	65	25	20	75	94,8%	97,4%	99,5%	99,7%	98,6%
19955	65	25	20	80	94,9%	97,4%	99,5%	99,7%	98,7%
19956	65	25	25	20	92,2%	97,2%	99,2%	99,2%	97,1%
19957	65	25	25	25	92,3%	97,1%	99,2%	99,3%	97,2%
19958	65	25	25	30	92,4%	97,1%	99,2%	99,3%	97,4%
19959	65	25	25	35	92,6%	97,1%	99,3%	99,4%	97,6%
19960	65	25	25	40	92,7%	97,0%	99,3%	99,4%	97,7%
19961	65	25	25	45	92,8%	97,0%	99,3%	99,4%	97,9%
19962	65	25	25	50	92,9%	96,9%	99,3%	99,5%	98,0%
19963	65	25	25	55	93,0%	96,9%	99,4%	99,5%	98,2%
19964	65	25	25	60	93,1%	96,9%	99,4%	99,5%	98,3%

19965	65	25	25	65	93,3%	96,8%	99,4%	99,6%	98,4%
19966	65	25	25	70	93,4%	96,8%	99,4%	99,6%	98,5%
19967	65	25	25	75	93,5%	96,7%	99,4%	99,6%	98,6%
19968	65	25	25	80	93,6%	96,7%	99,5%	99,7%	98,7%
19969	65	25	30	20	90,2%	96,5%	99,1%	99,2%	96,9%
19970	65	25	30	25	90,4%	96,4%	99,1%	99,2%	97,1%
19971	65	25	30	30	90,5%	96,4%	99,1%	99,3%	97,3%
19972	65	25	30	35	90,7%	96,3%	99,2%	99,3%	97,5%
19973	65	25	30	40	90,8%	96,3%	99,2%	99,4%	97,7%
19974	65	25	30	45	91,0%	96,2%	99,2%	99,4%	97,8%
19975	65	25	30	50	91,1%	96,2%	99,2%	99,4%	97,9%
19976	65	25	30	55	91,2%	96,1%	99,3%	99,5%	98,1%
19977	65	25	30	60	91,4%	96,1%	99,3%	99,5%	98,2%
19978	65	25	30	65	91,5%	96,0%	99,3%	99,5%	98,3%
19979	65	25	30	70	91,7%	96,0%	99,3%	99,6%	98,4%
19980	65	25	30	75	91,8%	95,9%	99,4%	99,6%	98,5%
19981	65	25	30	80	91,9%	95,9%	99,4%	99,6%	98,6%
19982	65	25	35	20	87,8%	95,6%	99,0%	99,1%	96,8%
19983	65	25	35	25	88,0%	95,5%	99,0%	99,2%	97,0%
19984	65	25	35	30	88,2%	95,5%	99,0%	99,2%	97,2%
19985	65	25	35	35	88,4%	95,4%	99,1%	99,3%	97,4%
19986	65	25	35	40	88,5%	95,3%	99,1%	99,3%	97,6%
19987	65	25	35	45	88,7%	95,3%	99,1%	99,4%	97,7%
19988	65	25	35	50	88,9%	95,2%	99,1%	99,4%	97,9%
19989	65	25	35	55	89,1%	95,2%	99,2%	99,4%	98,0%
19990	65	25	35	60	89,2%	95,1%	99,2%	99,5%	98,1%
19991	65	25	35	65	89,4%	95,0%	99,2%	99,5%	98,3%
19992	65	25	35	70	89,6%	95,0%	99,3%	99,6%	98,4%
19993	65	25	35	75	89,7%	94,9%	99,3%	99,6%	98,5%
19994	65	25	35	80	89,9%	94,9%	99,3%	99,6%	98,6%
19995	65	25	40	20	84,9%	94,5%	98,8%	99,1%	96,7%
19996	65	25	40	25	85,1%	94,4%	98,9%	99,1%	96,9%
19997	65	25	40	30	85,4%	94,3%	98,9%	99,2%	97,1%

19998	65	25	40	35	85,6%	94,3%	98,9%	99,2%	97,3%
19999	65	25	40	40	85,8%	94,2%	99,0%	99,3%	97,5%
20000	65	25	40	45	86,0%	94,1%	99,0%	99,3%	97,6%
20001	65	25	40	50	86,2%	94,1%	99,0%	99,4%	97,8%
20002	65	25	40	55	86,4%	94,0%	99,1%	99,4%	97,9%
20003	65	25	40	60	86,6%	93,9%	99,1%	99,5%	98,1%
20004	65	25	40	65	86,8%	93,8%	99,1%	99,5%	98,2%
20005	65	25	40	70	87,0%	93,8%	99,2%	99,5%	98,3%
20006	65	25	40	75	87,2%	93,7%	99,2%	99,6%	98,4%
20007	65	25	40	80	87,4%	93,6%	99,2%	99,6%	98,5%
20008	65	25	45	20	81,5%	93,2%	98,7%	99,0%	96,5%
20009	65	25	45	25	81,7%	93,1%	98,7%	99,1%	96,8%
20010	65	25	45	30	82,0%	93,0%	98,8%	99,1%	97,0%
20011	65	25	45	35	82,2%	92,9%	98,8%	99,2%	97,2%
20012	65	25	45	40	82,5%	92,8%	98,8%	99,2%	97,4%
20013	65	25	45	45	82,8%	92,7%	98,9%	99,3%	97,5%
20014	65	25	45	50	83,0%	92,6%	98,9%	99,3%	97,7%
20015	65	25	45	55	83,3%	92,5%	98,9%	99,4%	97,8%
20016	65	25	45	60	83,5%	92,5%	99,0%	99,4%	98,0%
20017	65	25	45	65	83,7%	92,4%	99,0%	99,5%	98,1%
20018	65	25	45	70	84,0%	92,3%	99,0%	99,5%	98,2%
20019	65	25	45	75	84,2%	92,2%	99,1%	99,5%	98,3%
20020	65	25	45	80	84,4%	92,1%	99,1%	99,6%	98,5%
20021	65	25	50	20	77,4%	91,5%	98,5%	98,9%	96,4%
20022	65	25	50	25	77,7%	91,4%	98,5%	99,0%	96,6%
20023	65	25	50	30	78,0%	91,3%	98,6%	99,1%	96,9%
20024	65	25	50	35	78,4%	91,2%	98,6%	99,1%	97,1%
20025	65	25	50	40	78,6%	91,1%	98,7%	99,2%	97,2%
20026	65	25	50	45	78,9%	91,0%	98,7%	99,2%	97,4%
20027	65	25	50	50	79,2%	90,9%	98,8%	99,3%	97,6%
20028	65	25	50	55	79,5%	90,8%	98,8%	99,3%	97,7%
20029	65	25	50	60	79,8%	90,7%	98,8%	99,4%	97,9%
20030	65	25	50	65	80,1%	90,6%	98,9%	99,4%	98,0%

20031	65	25	50	70	80,4%	90,4%	98,9%	99,5%	98,2%
20032	65	25	50	75	80,6%	90,3%	99,0%	99,5%	98,3%
20033	65	25	50	80	80,9%	90,2%	99,0%	99,5%	98,4%
20034	65	25	55	20	72,8%	89,5%	98,3%	98,9%	96,3%
20035	65	25	55	25	73,2%	89,4%	98,4%	98,9%	96,5%
20036	65	25	55	30	73,5%	89,3%	98,4%	99,0%	96,7%
20037	65	25	55	35	73,9%	89,2%	98,5%	99,1%	96,9%
20038	65	25	55	40	74,2%	89,0%	98,5%	99,1%	97,1%
20039	65	25	55	45	74,5%	88,9%	98,6%	99,2%	97,3%
20040	65	25	55	50	74,9%	88,8%	98,6%	99,2%	97,5%
20041	65	25	55	55	75,2%	88,7%	98,7%	99,3%	97,7%
20042	65	25	55	60	75,5%	88,5%	98,7%	99,3%	97,8%
20043	65	25	55	65	75,9%	88,4%	98,7%	99,4%	97,9%
20044	65	25	55	70	76,2%	88,3%	98,8%	99,4%	98,1%
20045	65	25	55	75	76,5%	88,1%	98,8%	99,5%	98,2%
20046	65	25	55	80	76,8%	88,0%	98,9%	99,5%	98,3%
20047	65	25	60	20	67,7%	87,2%	98,1%	98,8%	96,1%
20048	65	25	60	25	68,1%	87,0%	98,1%	98,9%	96,4%
20049	65	25	60	30	68,5%	86,9%	98,2%	98,9%	96,6%
20050	65	25	60	35	68,8%	86,7%	98,3%	99,0%	96,8%
20051	65	25	60	40	69,2%	86,6%	98,3%	99,1%	97,0%
20052	65	25	60	45	69,6%	86,4%	98,4%	99,1%	97,2%
20053	65	25	60	50	70,0%	86,3%	98,4%	99,2%	97,4%
20054	65	25	60	55	70,3%	86,1%	98,5%	99,3%	97,6%
20055	65	25	60	60	70,7%	86,0%	98,5%	99,3%	97,7%
20056	65	25	60	65	71,1%	85,8%	98,6%	99,3%	97,9%
20057	65	25	60	70	71,4%	85,6%	98,6%	99,4%	98,0%
20058	65	25	60	75	71,8%	85,5%	98,7%	99,4%	98,1%
20059	65	25	60	80	72,1%	85,3%	98,7%	99,5%	98,3%
20060	65	25	65	20	62,1%	84,4%	97,8%	98,7%	96,0%
20061	65	25	65	25	62,5%	84,2%	97,9%	98,8%	96,2%
20062	65	25	65	30	62,9%	84,0%	98,0%	98,9%	96,5%
20063	65	25	65	35	63,3%	83,8%	98,0%	99,0%	96,7%

20064	65	25	65	40	63,7%	83,7%	98,1%	99,0%	96,9%
20065	65	25	65	45	64,1%	83,5%	98,2%	99,1%	97,1%
20066	65	25	65	50	64,5%	83,3%	98,2%	99,2%	97,3%
20067	65	25	65	55	64,9%	83,1%	98,3%	99,2%	97,5%
20068	65	25	65	60	65,3%	82,9%	98,3%	99,3%	97,6%
20069	65	25	65	65	65,7%	82,8%	98,4%	99,3%	97,8%
20070	65	25	65	70	66,1%	82,6%	98,4%	99,4%	97,9%
20071	65	25	65	75	66,5%	82,4%	98,5%	99,4%	98,1%
20072	65	25	65	80	66,9%	82,2%	98,5%	99,4%	98,2%
20073	65	25	70	20	56,1%	81,1%	97,5%	98,6%	95,8%
20074	65	25	70	25	56,5%	80,9%	97,6%	98,7%	96,1%
20075	65	25	70	30	57,0%	80,7%	97,7%	98,8%	96,3%
20076	65	25	70	35	57,4%	80,5%	97,8%	98,9%	96,5%
20077	65	25	70	40	57,8%	80,3%	97,9%	99,0%	96,8%
20078	65	25	70	45	58,3%	80,1%	97,9%	99,0%	97,0%
20079	65	25	70	50	58,7%	79,8%	98,0%	99,1%	97,2%
20080	65	25	70	55	59,1%	79,6%	98,1%	99,2%	97,4%
20081	65	25	70	60	59,5%	79,4%	98,1%	99,2%	97,5%
20082	65	25	70	65	60,0%	79,2%	98,2%	99,3%	97,7%
20083	65	25	70	70	60,4%	79,0%	98,2%	99,3%	97,8%
20084	65	25	70	75	60,8%	78,8%	98,3%	99,4%	98,0%
20085	65	25	70	80	61,2%	78,6%	98,4%	99,4%	98,1%
20086	65	25	75	20	50,0%	77,3%	97,2%	98,6%	95,6%
20087	65	25	75	25	50,4%	77,1%	97,3%	98,7%	95,9%
20088	65	25	75	30	50,9%	76,8%	97,4%	98,7%	96,2%
20089	65	25	75	35	51,3%	76,6%	97,5%	98,8%	96,4%
20090	65	25	75	40	51,7%	76,4%	97,6%	98,9%	96,6%
20091	65	25	75	45	52,2%	76,1%	97,7%	99,0%	96,9%
20092	65	25	75	50	52,6%	75,9%	97,7%	99,0%	97,1%
20093	65	25	75	55	53,1%	75,6%	97,8%	99,1%	97,2%
20094	65	25	75	60	53,5%	75,4%	97,9%	99,2%	97,4%
20095	65	25	75	65	53,9%	75,2%	97,9%	99,2%	97,6%
20096	65	25	75	70	54,4%	74,9%	98,0%	99,3%	97,7%

20097	65	25	75	75	54,8%	74,7%	98,1%	99,3%	97,9%
20098	65	25	75	80	55,2%	74,4%	98,1%	99,4%	98,0%
20099	65	25	80	20	43,8%	73,0%	96,9%	98,5%	95,4%
20100	65	25	80	25	44,3%	72,7%	97,0%	98,6%	95,7%
20101	65	25	80	30	44,7%	72,5%	97,1%	98,7%	96,0%
20102	65	25	80	35	45,1%	72,2%	97,2%	98,8%	96,3%
20103	65	25	80	40	45,6%	71,9%	97,3%	98,8%	96,5%
20104	65	25	80	45	46,0%	71,7%	97,4%	98,9%	96,7%
20105	65	25	80	50	46,4%	71,4%	97,4%	99,0%	96,9%
20106	65	25	80	55	46,9%	71,1%	97,5%	99,0%	97,1%
20107	65	25	80	60	47,3%	70,9%	97,6%	99,1%	97,3%
20108	65	25	80	65	47,8%	70,6%	97,7%	99,2%	97,5%
20109	65	25	80	70	48,2%	70,3%	97,8%	99,2%	97,7%
20110	65	25	80	75	48,6%	70,0%	97,8%	99,3%	97,8%
20111	65	25	80	80	49,1%	69,8%	97,9%	99,3%	97,9%
20112	65	30	20	20	94,3%	97,9%	99,3%	99,2%	96,9%
20113	65	30	20	25	94,4%	97,9%	99,3%	99,3%	97,1%
20114	65	30	20	30	94,5%	97,8%	99,3%	99,3%	97,3%
20115	65	30	20	35	94,6%	97,8%	99,3%	99,3%	97,5%
20116	65	30	20	40	94,7%	97,8%	99,4%	99,4%	97,6%
20117	65	30	20	45	94,7%	97,8%	99,4%	99,4%	97,8%
20118	65	30	20	50	94,8%	97,7%	99,4%	99,5%	97,9%
20119	65	30	20	55	94,9%	97,7%	99,4%	99,5%	98,1%
20120	65	30	20	60	95,0%	97,7%	99,4%	99,5%	98,2%
20121	65	30	20	65	95,1%	97,6%	99,5%	99,6%	98,3%
20122	65	30	20	70	95,2%	97,6%	99,5%	99,6%	98,4%
20123	65	30	20	75	95,2%	97,6%	99,5%	99,6%	98,5%
20124	65	30	20	80	95,3%	97,6%	99,5%	99,6%	98,6%
20125	65	30	25	20	92,8%	97,4%	99,2%	99,2%	96,8%
20126	65	30	25	25	92,9%	97,3%	99,2%	99,2%	97,0%
20127	65	30	25	30	93,0%	97,3%	99,2%	99,3%	97,2%
20128	65	30	25	35	93,1%	97,3%	99,2%	99,3%	97,4%
20129	65	30	25	40	93,3%	97,2%	99,3%	99,4%	97,5%

20130	65	30	25	45	93,4%	97,2%	99,3%	99,4%	97,7%
20131	65	30	25	50	93,5%	97,2%	99,3%	99,4%	97,8%
20132	65	30	25	55	93,6%	97,1%	99,3%	99,5%	98,0%
20133	65	30	25	60	93,7%	97,1%	99,4%	99,5%	98,1%
20134	65	30	25	65	93,8%	97,1%	99,4%	99,5%	98,2%
20135	65	30	25	70	93,9%	97,0%	99,4%	99,6%	98,3%
20136	65	30	25	75	94,0%	97,0%	99,4%	99,6%	98,5%
20137	65	30	25	80	94,1%	96,9%	99,4%	99,6%	98,6%
20138	65	30	30	20	91,0%	96,7%	99,0%	99,1%	96,6%
20139	65	30	30	25	91,1%	96,7%	99,1%	99,2%	96,9%
20140	65	30	30	30	91,3%	96,6%	99,1%	99,2%	97,1%
20141	65	30	30	35	91,4%	96,6%	99,1%	99,3%	97,2%
20142	65	30	30	40	91,5%	96,5%	99,2%	99,3%	97,4%
20143	65	30	30	45	91,7%	96,5%	99,2%	99,4%	97,6%
20144	65	30	30	50	91,8%	96,5%	99,2%	99,4%	97,7%
20145	65	30	30	55	91,9%	96,4%	99,2%	99,4%	97,9%
20146	65	30	30	60	92,1%	96,4%	99,3%	99,5%	98,0%
20147	65	30	30	65	92,2%	96,3%	99,3%	99,5%	98,2%
20148	65	30	30	70	92,3%	96,3%	99,3%	99,5%	98,3%
20149	65	30	30	75	92,4%	96,2%	99,3%	99,6%	98,4%
20150	65	30	30	80	92,6%	96,2%	99,4%	99,6%	98,5%
20151	65	30	35	20	88,7%	95,9%	98,9%	99,0%	96,5%
20152	65	30	35	25	88,9%	95,8%	99,0%	99,1%	96,7%
20153	65	30	35	30	89,1%	95,8%	99,0%	99,2%	96,9%
20154	65	30	35	35	89,2%	95,7%	99,0%	99,2%	97,1%
20155	65	30	35	40	89,4%	95,7%	99,1%	99,3%	97,3%
20156	65	30	35	45	89,6%	95,6%	99,1%	99,3%	97,5%
20157	65	30	35	50	89,7%	95,6%	99,1%	99,4%	97,7%
20158	65	30	35	55	89,9%	95,5%	99,2%	99,4%	97,8%
20159	65	30	35	60	90,1%	95,5%	99,2%	99,4%	97,9%
20160	65	30	35	65	90,2%	95,4%	99,2%	99,5%	98,1%
20161	65	30	35	70	90,4%	95,3%	99,2%	99,5%	98,2%
20162	65	30	35	75	90,5%	95,3%	99,3%	99,5%	98,3%

20163	65	30	35	80	90,7%	95,2%	99,3%	99,6%	98,4%
20164	65	30	40	20	86,0%	94,9%	98,8%	99,0%	96,4%
20165	65	30	40	25	86,2%	94,8%	98,8%	99,0%	96,6%
20166	65	30	40	30	86,4%	94,8%	98,9%	99,1%	96,8%
20167	65	30	40	35	86,6%	94,7%	98,9%	99,2%	97,0%
20168	65	30	40	40	86,8%	94,6%	98,9%	99,2%	97,2%
20169	65	30	40	45	87,0%	94,6%	99,0%	99,3%	97,4%
20170	65	30	40	50	87,2%	94,5%	99,0%	99,3%	97,6%
20171	65	30	40	55	87,4%	94,4%	99,0%	99,4%	97,7%
20172	65	30	40	60	87,6%	94,3%	99,1%	99,4%	97,9%
20173	65	30	40	65	87,8%	94,3%	99,1%	99,5%	98,0%
20174	65	30	40	70	88,0%	94,2%	99,1%	99,5%	98,1%
20175	65	30	40	75	88,2%	94,1%	99,2%	99,5%	98,3%
20176	65	30	40	80	88,4%	94,1%	99,2%	99,6%	98,4%
20177	65	30	45	20	82,8%	93,6%	98,6%	98,9%	96,2%
20178	65	30	45	25	83,0%	93,6%	98,7%	99,0%	96,5%
20179	65	30	45	30	83,3%	93,5%	98,7%	99,1%	96,7%
20180	65	30	45	35	83,5%	93,4%	98,8%	99,1%	96,9%
20181	65	30	45	40	83,7%	93,3%	98,8%	99,2%	97,1%
20182	65	30	45	45	84,0%	93,2%	98,8%	99,2%	97,3%
20183	65	30	45	50	84,2%	93,2%	98,9%	99,3%	97,5%
20184	65	30	45	55	84,5%	93,1%	98,9%	99,3%	97,6%
20185	65	30	45	60	84,7%	93,0%	98,9%	99,4%	97,8%
20186	65	30	45	65	84,9%	92,9%	99,0%	99,4%	97,9%
20187	65	30	45	70	85,1%	92,8%	99,0%	99,5%	98,1%
20188	65	30	45	75	85,4%	92,7%	99,0%	99,5%	98,2%
20189	65	30	45	80	85,6%	92,6%	99,1%	99,5%	98,3%
20190	65	30	50	20	79,0%	92,1%	98,4%	98,9%	96,1%
20191	65	30	50	25	79,2%	92,0%	98,5%	98,9%	96,3%
20192	65	30	50	30	79,5%	91,9%	98,5%	99,0%	96,5%
20193	65	30	50	35	79,8%	91,8%	98,6%	99,1%	96,8%
20194	65	30	50	40	80,1%	91,7%	98,6%	99,1%	97,0%
20195	65	30	50	45	80,4%	91,6%	98,7%	99,2%	97,2%

20196	65	30	50	50	80,7%	91,5%	98,7%	99,2%	97,4%
20197	65	30	50	55	80,9%	91,4%	98,8%	99,3%	97,5%
20198	65	30	50	60	81,2%	91,3%	98,8%	99,3%	97,7%
20199	65	30	50	65	81,5%	91,2%	98,9%	99,4%	97,8%
20200	65	30	50	70	81,7%	91,1%	98,9%	99,4%	98,0%
20201	65	30	50	75	82,0%	91,0%	98,9%	99,5%	98,1%
20202	65	30	50	80	82,3%	90,9%	99,0%	99,5%	98,2%
20203	65	30	55	20	74,6%	90,3%	98,2%	98,8%	95,9%
20204	65	30	55	25	74,9%	90,1%	98,3%	98,9%	96,2%
20205	65	30	55	30	75,2%	90,0%	98,4%	98,9%	96,4%
20206	65	30	55	35	75,5%	89,9%	98,4%	99,0%	96,6%
20207	65	30	55	40	75,9%	89,8%	98,5%	99,1%	96,9%
20208	65	30	55	45	76,2%	89,7%	98,5%	99,1%	97,1%
20209	65	30	55	50	76,5%	89,6%	98,6%	99,2%	97,2%
20210	65	30	55	55	76,8%	89,4%	98,6%	99,2%	97,4%
20211	65	30	55	60	77,1%	89,3%	98,7%	99,3%	97,6%
20212	65	30	55	65	77,4%	89,2%	98,7%	99,3%	97,7%
20213	65	30	55	70	77,8%	89,1%	98,7%	99,4%	97,9%
20214	65	30	55	75	78,1%	88,9%	98,8%	99,4%	98,0%
20215	65	30	55	80	78,4%	88,8%	98,8%	99,5%	98,2%
20216	65	30	60	20	69,6%	88,0%	98,0%	98,7%	95,7%
20217	65	30	60	25	70,0%	87,9%	98,1%	98,8%	96,0%
20218	65	30	60	30	70,3%	87,8%	98,1%	98,9%	96,3%
20219	65	30	60	35	70,7%	87,6%	98,2%	98,9%	96,5%
20220	65	30	60	40	71,1%	87,5%	98,3%	99,0%	96,7%
20221	65	30	60	45	71,4%	87,3%	98,3%	99,1%	96,9%
20222	65	30	60	50	71,8%	87,2%	98,4%	99,1%	97,1%
20223	65	30	60	55	72,1%	87,0%	98,4%	99,2%	97,3%
20224	65	30	60	60	72,5%	86,9%	98,5%	99,2%	97,5%
20225	65	30	60	65	72,8%	86,7%	98,5%	99,3%	97,7%
20226	65	30	60	70	73,2%	86,6%	98,6%	99,3%	97,8%
20227	65	30	60	75	73,5%	86,4%	98,6%	99,4%	97,9%
20228	65	30	60	80	73,9%	86,3%	98,7%	99,4%	98,1%

20229	65	30	65	20	64,1%	85,4%	97,8%	98,6%	95,6%
20230	65	30	65	25	64,5%	85,2%	97,8%	98,7%	95,8%
20231	65	30	65	30	64,9%	85,1%	97,9%	98,8%	96,1%
20232	65	30	65	35	65,3%	84,9%	98,0%	98,9%	96,4%
20233	65	30	65	40	65,7%	84,7%	98,0%	99,0%	96,6%
20234	65	30	65	45	66,1%	84,5%	98,1%	99,0%	96,8%
20235	65	30	65	50	66,5%	84,4%	98,2%	99,1%	97,0%
20236	65	30	65	55	66,9%	84,2%	98,2%	99,1%	97,2%
20237	65	30	65	60	67,3%	84,0%	98,3%	99,2%	97,4%
20238	65	30	65	65	67,7%	83,9%	98,3%	99,3%	97,6%
20239	65	30	65	70	68,1%	83,7%	98,4%	99,3%	97,7%
20240	65	30	65	75	68,5%	83,5%	98,4%	99,4%	97,9%
20241	65	30	65	80	68,8%	83,3%	98,5%	99,4%	98,0%
20242	65	30	70	20	58,3%	82,3%	97,5%	98,5%	95,4%
20243	65	30	70	25	58,7%	82,1%	97,6%	98,6%	95,7%
20244	65	30	70	30	59,1%	81,9%	97,6%	98,7%	96,0%
20245	65	30	70	35	59,6%	81,7%	97,7%	98,8%	96,2%
20246	65	30	70	40	60,0%	81,5%	97,8%	98,9%	96,5%
20247	65	30	70	45	60,4%	81,3%	97,9%	99,0%	96,7%
20248	65	30	70	50	60,8%	81,1%	97,9%	99,0%	96,9%
20249	65	30	70	55	61,2%	80,9%	98,0%	99,1%	97,1%
20250	65	30	70	60	61,7%	80,7%	98,1%	99,2%	97,3%
20251	65	30	70	65	62,1%	80,5%	98,1%	99,2%	97,5%
20252	65	30	70	70	62,5%	80,3%	98,2%	99,3%	97,6%
20253	65	30	70	75	62,9%	80,1%	98,2%	99,3%	97,8%
20254	65	30	70	80	63,3%	79,9%	98,3%	99,4%	97,9%
20255	65	30	75	20	52,2%	78,6%	97,1%	98,4%	95,2%
20256	65	30	75	25	52,6%	78,4%	97,2%	98,6%	95,5%
20257	65	30	75	30	53,1%	78,2%	97,3%	98,6%	95,8%
20258	65	30	75	35	53,5%	78,0%	97,4%	98,7%	96,1%
20259	65	30	75	40	53,9%	77,7%	97,5%	98,8%	96,3%
20260	65	30	75	45	54,4%	77,5%	97,6%	98,9%	96,5%
20261	65	30	75	50	54,8%	77,3%	97,7%	99,0%	96,8%

20262	65	30	75	55	55,3%	77,1%	97,7%	99,0%	97,0%
20263	65	30	75	60	55,7%	76,8%	97,8%	99,1%	97,2%
20264	65	30	75	65	56,1%	76,6%	97,9%	99,2%	97,4%
20265	65	30	75	70	56,6%	76,4%	98,0%	99,2%	97,5%
20266	65	30	75	75	57,0%	76,1%	98,0%	99,3%	97,7%
20267	65	30	75	80	57,4%	75,9%	98,1%	99,3%	97,8%
20268	65	30	80	20	46,0%	74,5%	96,8%	98,4%	95,0%
20269	65	30	80	25	46,5%	74,3%	96,9%	98,5%	95,3%
20270	65	30	80	30	46,9%	74,0%	97,0%	98,6%	95,6%
20271	65	30	80	35	47,3%	73,7%	97,1%	98,7%	95,9%
20272	65	30	80	40	47,8%	73,5%	97,2%	98,7%	96,2%
20273	65	30	80	45	48,2%	73,2%	97,3%	98,8%	96,4%
20274	65	30	80	50	48,7%	73,0%	97,4%	98,9%	96,6%
20275	65	30	80	55	49,1%	72,7%	97,4%	99,0%	96,9%
20276	65	30	80	60	49,5%	72,5%	97,5%	99,0%	97,1%
20277	65	30	80	65	50,0%	72,2%	97,6%	99,1%	97,2%
20278	65	30	80	70	50,4%	71,9%	97,7%	99,2%	97,4%
20279	65	30	80	75	50,9%	71,7%	97,8%	99,2%	97,6%
20280	65	30	80	80	51,3%	71,4%	97,8%	99,3%	97,7%
20281	65	35	20	20	94,7%	98,1%	99,2%	99,1%	96,6%
20282	65	35	20	25	94,8%	98,0%	99,3%	99,2%	96,8%
20283	65	35	20	30	94,9%	98,0%	99,3%	99,2%	97,0%
20284	65	35	20	35	95,0%	98,0%	99,3%	99,3%	97,2%
20285	65	35	20	40	95,1%	98,0%	99,3%	99,3%	97,4%
20286	65	35	20	45	95,2%	97,9%	99,4%	99,4%	97,6%
20287	65	35	20	50	95,2%	97,9%	99,4%	99,4%	97,7%
20288	65	35	20	55	95,3%	97,9%	99,4%	99,5%	97,9%
20289	65	35	20	60	95,4%	97,8%	99,4%	99,5%	98,0%
20290	65	35	20	65	95,5%	97,8%	99,4%	99,5%	98,1%
20291	65	35	20	70	95,6%	97,8%	99,5%	99,6%	98,3%
20292	65	35	20	75	95,6%	97,8%	99,5%	99,6%	98,4%
20293	65	35	20	80	95,7%	97,7%	99,5%	99,6%	98,5%
20294	65	35	25	20	93,4%	97,6%	99,1%	99,1%	96,5%

20295	65	35	25	25	93,5%	97,5%	99,2%	99,1%	96,7%
20296	65	35	25	30	93,6%	97,5%	99,2%	99,2%	96,9%
20297	65	35	25	35	93,7%	97,5%	99,2%	99,3%	97,1%
20298	65	35	25	40	93,8%	97,4%	99,2%	99,3%	97,3%
20299	65	35	25	45	93,9%	97,4%	99,3%	99,4%	97,5%
20300	65	35	25	50	94,0%	97,4%	99,3%	99,4%	97,6%
20301	65	35	25	55	94,1%	97,3%	99,3%	99,4%	97,8%
20302	65	35	25	60	94,2%	97,3%	99,3%	99,5%	97,9%
20303	65	35	25	65	94,3%	97,3%	99,4%	99,5%	98,1%
20304	65	35	25	70	94,4%	97,2%	99,4%	99,5%	98,2%
20305	65	35	25	75	94,5%	97,2%	99,4%	99,6%	98,3%
20306	65	35	25	80	94,6%	97,2%	99,4%	99,6%	98,4%
20307	65	35	30	20	91,7%	97,0%	99,0%	99,0%	96,3%
20308	65	35	30	25	91,8%	96,9%	99,1%	99,1%	96,6%
20309	65	35	30	30	91,9%	96,9%	99,1%	99,2%	96,8%
20310	65	35	30	35	92,1%	96,8%	99,1%	99,2%	97,0%
20311	65	35	30	40	92,2%	96,8%	99,1%	99,3%	97,2%
20312	65	35	30	45	92,3%	96,8%	99,2%	99,3%	97,4%
20313	65	35	30	50	92,4%	96,7%	99,2%	99,4%	97,5%
20314	65	35	30	55	92,6%	96,7%	99,2%	99,4%	97,7%
20315	65	35	30	60	92,7%	96,6%	99,3%	99,4%	97,8%
20316	65	35	30	65	92,8%	96,6%	99,3%	99,5%	98,0%
20317	65	35	30	70	92,9%	96,5%	99,3%	99,5%	98,1%
20318	65	35	30	75	93,0%	96,5%	99,3%	99,5%	98,2%
20319	65	35	30	80	93,1%	96,5%	99,3%	99,6%	98,3%
20320	65	35	35	20	89,6%	96,2%	98,9%	99,0%	96,2%
20321	65	35	35	25	89,7%	96,1%	98,9%	99,0%	96,4%
20322	65	35	35	30	89,9%	96,1%	99,0%	99,1%	96,6%
20323	65	35	35	35	90,1%	96,0%	99,0%	99,2%	96,9%
20324	65	35	35	40	90,2%	96,0%	99,0%	99,2%	97,1%
20325	65	35	35	45	90,4%	95,9%	99,1%	99,3%	97,2%
20326	65	35	35	50	90,5%	95,9%	99,1%	99,3%	97,4%
20327	65	35	35	55	90,7%	95,8%	99,1%	99,4%	97,6%

20328	65	35	35	60	90,8%	95,8%	99,2%	99,4%	97,7%
20329	65	35	35	65	91,0%	95,7%	99,2%	99,4%	97,9%
20330	65	35	35	70	91,1%	95,7%	99,2%	99,5%	98,0%
20331	65	35	35	75	91,3%	95,6%	99,2%	99,5%	98,2%
20332	65	35	35	80	91,4%	95,6%	99,3%	99,5%	98,3%
20333	65	35	40	20	87,0%	95,3%	98,7%	98,9%	96,0%
20334	65	35	40	25	87,2%	95,2%	98,8%	99,0%	96,3%
20335	65	35	40	30	87,4%	95,1%	98,8%	99,0%	96,5%
20336	65	35	40	35	87,6%	95,1%	98,9%	99,1%	96,7%
20337	65	35	40	40	87,8%	95,0%	98,9%	99,2%	96,9%
20338	65	35	40	45	88,0%	94,9%	98,9%	99,2%	97,1%
20339	65	35	40	50	88,2%	94,9%	99,0%	99,3%	97,3%
20340	65	35	40	55	88,4%	94,8%	99,0%	99,3%	97,5%
20341	65	35	40	60	88,5%	94,8%	99,0%	99,4%	97,7%
20342	65	35	40	65	88,7%	94,7%	99,1%	99,4%	97,8%
20343	65	35	40	70	88,9%	94,6%	99,1%	99,4%	97,9%
20344	65	35	40	75	89,1%	94,6%	99,1%	99,5%	98,1%
20345	65	35	40	80	89,2%	94,5%	99,2%	99,5%	98,2%
20346	65	35	45	20	84,0%	94,1%	98,6%	98,8%	95,9%
20347	65	35	45	25	84,2%	94,0%	98,6%	98,9%	96,1%
20348	65	35	45	30	84,5%	93,9%	98,7%	99,0%	96,4%
20349	65	35	45	35	84,7%	93,9%	98,7%	99,1%	96,6%
20350	65	35	45	40	84,9%	93,8%	98,8%	99,1%	96,8%
20351	65	35	45	45	85,1%	93,7%	98,8%	99,2%	97,0%
20352	65	35	45	50	85,4%	93,6%	98,8%	99,2%	97,2%
20353	65	35	45	55	85,6%	93,6%	98,9%	99,3%	97,4%
20354	65	35	45	60	85,8%	93,5%	98,9%	99,3%	97,6%
20355	65	35	45	65	86,0%	93,4%	99,0%	99,4%	97,7%
20356	65	35	45	70	86,2%	93,3%	99,0%	99,4%	97,9%
20357	65	35	45	75	86,4%	93,2%	99,0%	99,5%	98,0%
20358	65	35	45	80	86,6%	93,2%	99,1%	99,5%	98,1%
20359	65	35	50	20	80,4%	92,7%	98,4%	98,8%	95,7%
20360	65	35	50	25	80,7%	92,6%	98,4%	98,8%	96,0%

20361	65	35	50	30	80,9%	92,5%	98,5%	98,9%	96,2%
20362	65	35	50	35	81,2%	92,4%	98,6%	99,0%	96,5%
20363	65	35	50	40	81,5%	92,3%	98,6%	99,1%	96,7%
20364	65	35	50	45	81,7%	92,2%	98,6%	99,1%	96,9%
20365	65	35	50	50	82,0%	92,1%	98,7%	99,2%	97,1%
20366	65	35	50	55	82,3%	92,0%	98,7%	99,2%	97,3%
20367	65	35	50	60	82,5%	91,9%	98,8%	99,3%	97,5%
20368	65	35	50	65	82,8%	91,8%	98,8%	99,3%	97,6%
20369	65	35	50	70	83,0%	91,7%	98,9%	99,4%	97,8%
20370	65	35	50	75	83,3%	91,6%	98,9%	99,4%	97,9%
20371	65	35	50	80	83,5%	91,5%	98,9%	99,5%	98,1%
20372	65	35	55	20	76,2%	90,9%	98,2%	98,7%	95,5%
20373	65	35	55	25	76,5%	90,8%	98,2%	98,8%	95,8%
20374	65	35	55	30	76,8%	90,7%	98,3%	98,9%	96,1%
20375	65	35	55	35	77,1%	90,6%	98,4%	98,9%	96,3%
20376	65	35	55	40	77,5%	90,5%	98,4%	99,0%	96,5%
20377	65	35	55	45	77,8%	90,4%	98,5%	99,1%	96,8%
20378	65	35	55	50	78,1%	90,3%	98,5%	99,1%	97,0%
20379	65	35	55	55	78,4%	90,2%	98,6%	99,2%	97,2%
20380	65	35	55	60	78,7%	90,0%	98,6%	99,2%	97,4%
20381	65	35	55	65	79,0%	89,9%	98,7%	99,3%	97,5%
20382	65	35	55	70	79,3%	89,8%	98,7%	99,3%	97,7%
20383	65	35	55	75	79,5%	89,7%	98,7%	99,4%	97,8%
20384	65	35	55	80	79,8%	89,6%	98,8%	99,4%	98,0%
20385	65	35	60	20	71,4%	88,8%	98,0%	98,6%	95,3%
20386	65	35	60	25	71,8%	88,7%	98,0%	98,7%	95,6%
20387	65	35	60	30	72,2%	88,6%	98,1%	98,8%	95,9%
20388	65	35	60	35	72,5%	88,4%	98,1%	98,9%	96,2%
20389	65	35	60	40	72,9%	88,3%	98,2%	98,9%	96,4%
20390	65	35	60	45	73,2%	88,2%	98,3%	99,0%	96,6%
20391	65	35	60	50	73,5%	88,0%	98,3%	99,1%	96,9%
20392	65	35	60	55	73,9%	87,9%	98,4%	99,1%	97,1%
20393	65	35	60	60	74,2%	87,8%	98,4%	99,2%	97,2%

20394	65	35	60	65	74,6%	87,6%	98,5%	99,2%	97,4%
20395	65	35	60	70	74,9%	87,5%	98,5%	99,3%	97,6%
20396	65	35	60	75	75,2%	87,3%	98,6%	99,3%	97,7%
20397	65	35	60	80	75,6%	87,2%	98,6%	99,4%	97,9%
20398	65	35	65	20	66,2%	86,3%	97,7%	98,5%	95,1%
20399	65	35	65	25	66,5%	86,2%	97,8%	98,6%	95,5%
20400	65	35	65	30	66,9%	86,0%	97,8%	98,7%	95,7%
20401	65	35	65	35	67,3%	85,9%	97,9%	98,8%	96,0%
20402	65	35	65	40	67,7%	85,7%	98,0%	98,9%	96,3%
20403	65	35	65	45	68,1%	85,5%	98,0%	98,9%	96,5%
20404	65	35	65	50	68,5%	85,4%	98,1%	99,0%	96,7%
20405	65	35	65	55	68,9%	85,2%	98,2%	99,1%	96,9%
20406	65	35	65	60	69,2%	85,1%	98,2%	99,1%	97,1%
20407	65	35	65	65	69,6%	84,9%	98,3%	99,2%	97,3%
20408	65	35	65	70	70,0%	84,7%	98,3%	99,3%	97,5%
20409	65	35	65	75	70,3%	84,6%	98,4%	99,3%	97,7%
20410	65	35	65	80	70,7%	84,4%	98,5%	99,3%	97,8%
20411	65	35	70	20	60,4%	83,4%	97,4%	98,4%	94,9%
20412	65	35	70	25	60,8%	83,2%	97,5%	98,5%	95,3%
20413	65	35	70	30	61,3%	83,0%	97,6%	98,6%	95,6%
20414	65	35	70	35	61,7%	82,8%	97,6%	98,7%	95,8%
20415	65	35	70	40	62,1%	82,6%	97,7%	98,8%	96,1%
20416	65	35	70	45	62,5%	82,5%	97,8%	98,9%	96,4%
20417	65	35	70	50	62,9%	82,3%	97,9%	99,0%	96,6%
20418	65	35	70	55	63,3%	82,1%	97,9%	99,0%	96,8%
20419	65	35	70	60	63,7%	81,9%	98,0%	99,1%	97,0%
20420	65	35	70	65	64,1%	81,7%	98,1%	99,1%	97,2%
20421	65	35	70	70	64,6%	81,5%	98,1%	99,2%	97,4%
20422	65	35	70	75	65,0%	81,3%	98,2%	99,3%	97,6%
20423	65	35	70	80	65,4%	81,1%	98,2%	99,3%	97,7%
20424	65	35	75	20	54,4%	79,9%	97,1%	98,3%	94,7%
20425	65	35	75	25	54,8%	79,7%	97,2%	98,4%	95,1%
20426	65	35	75	30	55,3%	79,5%	97,2%	98,5%	95,4%

20427	65	35	75	35	55,7%	79,3%	97,3%	98,6%	95,7%
20428	65	35	75	40	56,1%	79,1%	97,4%	98,7%	96,0%
20429	65	35	75	45	56,6%	78,9%	97,5%	98,8%	96,2%
20430	65	35	75	50	57,0%	78,6%	97,6%	98,9%	96,5%
20431	65	35	75	55	57,4%	78,4%	97,7%	99,0%	96,7%
20432	65	35	75	60	57,9%	78,2%	97,7%	99,0%	96,9%
20433	65	35	75	65	58,3%	78,0%	97,8%	99,1%	97,1%
20434	65	35	75	70	58,7%	77,7%	97,9%	99,2%	97,3%
20435	65	35	75	75	59,1%	77,5%	98,0%	99,2%	97,5%
20436	65	35	75	80	59,6%	77,3%	98,0%	99,3%	97,6%
20437	65	35	80	20	48,2%	76,0%	96,7%	98,2%	94,5%
20438	65	35	80	25	48,7%	75,7%	96,8%	98,3%	94,9%
20439	65	35	80	30	49,1%	75,5%	96,9%	98,5%	95,2%
20440	65	35	80	35	49,6%	75,2%	97,0%	98,6%	95,5%
20441	65	35	80	40	50,0%	75,0%	97,1%	98,6%	95,8%
20442	65	35	80	45	50,4%	74,8%	97,2%	98,7%	96,1%
20443	65	35	80	50	50,9%	74,5%	97,3%	98,8%	96,3%
20444	65	35	80	55	51,3%	74,3%	97,4%	98,9%	96,5%
20445	65	35	80	60	51,8%	74,0%	97,5%	99,0%	96,8%
20446	65	35	80	65	52,2%	73,8%	97,5%	99,0%	97,0%
20447	65	35	80	70	52,6%	73,5%	97,6%	99,1%	97,2%
20448	65	35	80	75	53,1%	73,2%	97,7%	99,2%	97,4%
20449	65	35	80	80	53,5%	73,0%	97,8%	99,2%	97,5%
20450	65	40	20	20	95,2%	98,2%	99,2%	99,1%	96,3%
20451	65	40	20	25	95,3%	98,2%	99,2%	99,1%	96,5%
20452	65	40	20	30	95,3%	98,2%	99,3%	99,2%	96,7%
20453	65	40	20	35	95,4%	98,1%	99,3%	99,2%	96,9%
20454	65	40	20	40	95,5%	98,1%	99,3%	99,3%	97,1%
20455	65	40	20	45	95,6%	98,1%	99,3%	99,3%	97,3%
20456	65	40	20	50	95,6%	98,1%	99,4%	99,4%	97,5%
20457	65	40	20	55	95,7%	98,0%	99,4%	99,4%	97,7%
20458	65	40	20	60	95,8%	98,0%	99,4%	99,5%	97,8%
20459	65	40	20	65	95,8%	98,0%	99,4%	99,5%	98,0%

20460	65	40	20	70	95,9%	98,0%	99,4%	99,5%	98,1%
20461	65	40	20	75	96,0%	97,9%	99,5%	99,6%	98,2%
20462	65	40	20	80	96,1%	97,9%	99,5%	99,6%	98,3%
20463	65	40	25	20	93,9%	97,7%	99,1%	99,0%	96,1%
20464	65	40	25	25	94,0%	97,7%	99,1%	99,1%	96,4%
20465	65	40	25	30	94,1%	97,7%	99,2%	99,1%	96,6%
20466	65	40	25	35	94,2%	97,7%	99,2%	99,2%	96,8%
20467	65	40	25	40	94,3%	97,6%	99,2%	99,3%	97,0%
20468	65	40	25	45	94,4%	97,6%	99,2%	99,3%	97,2%
20469	65	40	25	50	94,5%	97,6%	99,3%	99,3%	97,4%
20470	65	40	25	55	94,6%	97,5%	99,3%	99,4%	97,6%
20471	65	40	25	60	94,7%	97,5%	99,3%	99,4%	97,7%
20472	65	40	25	65	94,7%	97,5%	99,3%	99,5%	97,9%
20473	65	40	25	70	94,8%	97,4%	99,4%	99,5%	98,0%
20474	65	40	25	75	94,9%	97,4%	99,4%	99,5%	98,1%
20475	65	40	25	80	95,0%	97,4%	99,4%	99,6%	98,3%
20476	65	40	30	20	92,3%	97,2%	99,0%	99,0%	96,0%
20477	65	40	30	25	92,4%	97,1%	99,0%	99,0%	96,2%
20478	65	40	30	30	92,6%	97,1%	99,1%	99,1%	96,5%
20479	65	40	30	35	92,7%	97,1%	99,1%	99,1%	96,7%
20480	65	40	30	40	92,8%	97,0%	99,1%	99,2%	96,9%
20481	65	40	30	45	92,9%	97,0%	99,1%	99,3%	97,1%
20482	65	40	30	50	93,0%	97,0%	99,2%	99,3%	97,3%
20483	65	40	30	55	93,2%	96,9%	99,2%	99,4%	97,5%
20484	65	40	30	60	93,3%	96,9%	99,2%	99,4%	97,6%
20485	65	40	30	65	93,4%	96,8%	99,3%	99,4%	97,8%
20486	65	40	30	70	93,5%	96,8%	99,3%	99,5%	97,9%
20487	65	40	30	75	93,6%	96,8%	99,3%	99,5%	98,1%
20488	65	40	30	80	93,7%	96,7%	99,3%	99,5%	98,2%
20489	65	40	35	20	90,4%	96,5%	98,9%	98,9%	95,8%
20490	65	40	35	25	90,5%	96,4%	98,9%	99,0%	96,1%
20491	65	40	35	30	90,7%	96,4%	98,9%	99,0%	96,3%
20492	65	40	35	35	90,8%	96,3%	99,0%	99,1%	96,6%

20493	65	40	35	40	91,0%	96,3%	99,0%	99,2%	96,8%
20494	65	40	35	45	91,1%	96,2%	99,0%	99,2%	97,0%
20495	65	40	35	50	91,3%	96,2%	99,1%	99,3%	97,2%
20496	65	40	35	55	91,4%	96,1%	99,1%	99,3%	97,4%
20497	65	40	35	60	91,5%	96,1%	99,1%	99,4%	97,5%
20498	65	40	35	65	91,7%	96,0%	99,2%	99,4%	97,7%
20499	65	40	35	70	91,8%	96,0%	99,2%	99,4%	97,8%
20500	65	40	35	75	91,9%	95,9%	99,2%	99,5%	98,0%
20501	65	40	35	80	92,1%	95,9%	99,2%	99,5%	98,1%
20502	65	40	40	20	88,0%	95,6%	98,7%	98,8%	95,6%
20503	65	40	40	25	88,2%	95,5%	98,7%	98,9%	95,9%
20504	65	40	40	30	88,4%	95,5%	98,8%	99,0%	96,2%
20505	65	40	40	35	88,6%	95,4%	98,8%	99,0%	96,4%
20506	65	40	40	40	88,7%	95,4%	98,9%	99,1%	96,6%
20507	65	40	40	45	88,9%	95,3%	98,9%	99,2%	96,9%
20508	65	40	40	50	89,1%	95,3%	98,9%	99,2%	97,1%
20509	65	40	40	55	89,3%	95,2%	99,0%	99,3%	97,2%
20510	65	40	40	60	89,4%	95,1%	99,0%	99,3%	97,4%
20511	65	40	40	65	89,6%	95,1%	99,0%	99,4%	97,6%
20512	65	40	40	70	89,7%	95,0%	99,1%	99,4%	97,7%
20513	65	40	40	75	89,9%	94,9%	99,1%	99,4%	97,9%
20514	65	40	40	80	90,1%	94,9%	99,1%	99,5%	98,0%
20515	65	40	45	20	85,1%	94,5%	98,5%	98,7%	95,5%
20516	65	40	45	25	85,4%	94,4%	98,6%	98,8%	95,7%
20517	65	40	45	30	85,6%	94,4%	98,6%	98,9%	96,0%
20518	65	40	45	35	85,8%	94,3%	98,7%	99,0%	96,3%
20519	65	40	45	40	86,0%	94,2%	98,7%	99,0%	96,5%
20520	65	40	45	45	86,2%	94,2%	98,8%	99,1%	96,7%
20521	65	40	45	50	86,4%	94,1%	98,8%	99,2%	96,9%
20522	65	40	45	55	86,6%	94,0%	98,8%	99,2%	97,1%
20523	65	40	45	60	86,8%	93,9%	98,9%	99,3%	97,3%
20524	65	40	45	65	87,0%	93,9%	98,9%	99,3%	97,5%
20525	65	40	45	70	87,2%	93,8%	99,0%	99,4%	97,7%

20526	65	40	45	75	87,4%	93,7%	99,0%	99,4%	97,8%
20527	65	40	45	80	87,6%	93,6%	99,0%	99,4%	97,9%
20528	65	40	50	20	81,7%	93,2%	98,3%	98,7%	95,3%
20529	65	40	50	25	82,0%	93,1%	98,4%	98,8%	95,6%
20530	65	40	50	30	82,3%	93,0%	98,5%	98,8%	95,9%
20531	65	40	50	35	82,5%	92,9%	98,5%	98,9%	96,1%
20532	65	40	50	40	82,8%	92,8%	98,6%	99,0%	96,4%
20533	65	40	50	45	83,0%	92,8%	98,6%	99,1%	96,6%
20534	65	40	50	50	83,3%	92,7%	98,6%	99,1%	96,8%
20535	65	40	50	55	83,5%	92,6%	98,7%	99,2%	97,0%
20536	65	40	50	60	83,8%	92,5%	98,7%	99,2%	97,2%
20537	65	40	50	65	84,0%	92,4%	98,8%	99,3%	97,4%
20538	65	40	50	70	84,2%	92,3%	98,8%	99,3%	97,6%
20539	65	40	50	75	84,5%	92,2%	98,9%	99,4%	97,7%
20540	65	40	50	80	84,7%	92,1%	98,9%	99,4%	97,9%
20541	65	40	55	20	77,8%	91,6%	98,1%	98,6%	95,1%
20542	65	40	55	25	78,1%	91,5%	98,2%	98,7%	95,4%
20543	65	40	55	30	78,4%	91,4%	98,3%	98,8%	95,7%
20544	65	40	55	35	78,7%	91,3%	98,3%	98,8%	96,0%
20545	65	40	55	40	79,0%	91,2%	98,4%	98,9%	96,2%
20546	65	40	55	45	79,3%	91,0%	98,4%	99,0%	96,5%
20547	65	40	55	50	79,5%	90,9%	98,5%	99,1%	96,7%
20548	65	40	55	55	79,8%	90,8%	98,5%	99,1%	96,9%
20549	65	40	55	60	80,1%	90,7%	98,6%	99,2%	97,1%
20550	65	40	55	65	80,4%	90,6%	98,6%	99,2%	97,3%
20551	65	40	55	70	80,7%	90,5%	98,7%	99,3%	97,5%
20552	65	40	55	75	80,9%	90,4%	98,7%	99,3%	97,6%
20553	65	40	55	80	81,2%	90,3%	98,7%	99,4%	97,8%
20554	65	40	60	20	73,2%	89,6%	97,9%	98,5%	94,9%
20555	65	40	60	25	73,6%	89,5%	98,0%	98,6%	95,2%
20556	65	40	60	30	73,9%	89,4%	98,0%	98,7%	95,5%
20557	65	40	60	35	74,2%	89,2%	98,1%	98,8%	95,8%
20558	65	40	60	40	74,6%	89,1%	98,2%	98,9%	96,1%

20559	65	40	60	45	74,9%	89,0%	98,2%	98,9%	96,3%
20560	65	40	60	50	75,2%	88,8%	98,3%	99,0%	96,6%
20561	65	40	60	55	75,6%	88,7%	98,3%	99,1%	96,8%
20562	65	40	60	60	75,9%	88,6%	98,4%	99,1%	97,0%
20563	65	40	60	65	76,2%	88,4%	98,4%	99,2%	97,2%
20564	65	40	60	70	76,5%	88,3%	98,5%	99,2%	97,4%
20565	65	40	60	75	76,8%	88,2%	98,5%	99,3%	97,5%
20566	65	40	60	80	77,2%	88,0%	98,6%	99,3%	97,7%
20567	65	40	65	20	68,1%	87,2%	97,6%	98,4%	94,7%
20568	65	40	65	25	68,5%	87,1%	97,7%	98,5%	95,0%
20569	65	40	65	30	68,9%	86,9%	97,8%	98,6%	95,3%
20570	65	40	65	35	69,2%	86,8%	97,8%	98,7%	95,6%
20571	65	40	65	40	69,6%	86,6%	97,9%	98,8%	95,9%
20572	65	40	65	45	70,0%	86,5%	98,0%	98,9%	96,2%
20573	65	40	65	50	70,4%	86,3%	98,0%	98,9%	96,4%
20574	65	40	65	55	70,7%	86,2%	98,1%	99,0%	96,6%
20575	65	40	65	60	71,1%	86,0%	98,2%	99,1%	96,9%
20576	65	40	65	65	71,5%	85,9%	98,2%	99,1%	97,1%
20577	65	40	65	70	71,8%	85,7%	98,3%	99,2%	97,2%
20578	65	40	65	75	72,2%	85,6%	98,3%	99,2%	97,4%
20579	65	40	65	80	72,5%	85,4%	98,4%	99,3%	97,6%
20580	65	40	70	20	62,5%	84,4%	97,3%	98,3%	94,5%
20581	65	40	70	25	62,9%	84,3%	97,4%	98,4%	94,8%
20582	65	40	70	30	63,3%	84,1%	97,5%	98,5%	95,1%
20583	65	40	70	35	63,8%	83,9%	97,6%	98,6%	95,5%
20584	65	40	70	40	64,2%	83,7%	97,6%	98,7%	95,7%
20585	65	40	70	45	64,6%	83,6%	97,7%	98,8%	96,0%
20586	65	40	70	50	65,0%	83,4%	97,8%	98,9%	96,3%
20587	65	40	70	55	65,4%	83,2%	97,9%	98,9%	96,5%
20588	65	40	70	60	65,8%	83,0%	97,9%	99,0%	96,7%
20589	65	40	70	65	66,2%	82,8%	98,0%	99,1%	96,9%
20590	65	40	70	70	66,6%	82,6%	98,1%	99,1%	97,1%
20591	65	40	70	75	66,9%	82,5%	98,1%	99,2%	97,3%

20592	65	40	70	80	67,3%	82,3%	98,2%	99,3%	97,5%
20593	65	40	75	20	56,6%	81,2%	97,0%	98,2%	94,3%
20594	65	40	75	25	57,0%	81,0%	97,1%	98,3%	94,6%
20595	65	40	75	30	57,4%	80,8%	97,2%	98,4%	95,0%
20596	65	40	75	35	57,9%	80,6%	97,3%	98,5%	95,3%
20597	65	40	75	40	58,3%	80,4%	97,3%	98,6%	95,6%
20598	65	40	75	45	58,7%	80,1%	97,4%	98,7%	95,8%
20599	65	40	75	50	59,2%	79,9%	97,5%	98,8%	96,1%
20600	65	40	75	55	59,6%	79,7%	97,6%	98,9%	96,4%
20601	65	40	75	60	60,0%	79,5%	97,7%	99,0%	96,6%
20602	65	40	75	65	60,4%	79,3%	97,7%	99,0%	96,8%
20603	65	40	75	70	60,9%	79,1%	97,8%	99,1%	97,0%
20604	65	40	75	75	61,3%	78,9%	97,9%	99,2%	97,2%
20605	65	40	75	80	61,7%	78,6%	98,0%	99,2%	97,4%
20606	65	40	80	20	50,5%	77,4%	96,6%	98,1%	94,0%
20607	65	40	80	25	50,9%	77,1%	96,7%	98,2%	94,4%
20608	65	40	80	30	51,3%	76,9%	96,8%	98,3%	94,7%
20609	65	40	80	35	51,8%	76,7%	96,9%	98,4%	95,1%
20610	65	40	80	40	52,2%	76,4%	97,0%	98,5%	95,4%
20611	65	40	80	45	52,7%	76,2%	97,1%	98,6%	95,7%
20612	65	40	80	50	53,1%	76,0%	97,2%	98,7%	96,0%
20613	65	40	80	55	53,5%	75,7%	97,3%	98,8%	96,2%
20614	65	40	80	60	54,0%	75,5%	97,4%	98,9%	96,5%
20615	65	40	80	65	54,4%	75,3%	97,5%	99,0%	96,7%
20616	65	40	80	70	54,8%	75,0%	97,5%	99,0%	96,9%
20617	65	40	80	75	55,3%	74,8%	97,6%	99,1%	97,1%
20618	65	40	80	80	55,7%	74,5%	97,7%	99,2%	97,3%
20619	65	45	20	20	95,6%	98,3%	99,2%	99,0%	95,9%
20620	65	45	20	25	95,6%	98,3%	99,2%	99,1%	96,2%
20621	65	45	20	30	95,7%	98,3%	99,2%	99,1%	96,4%
20622	65	45	20	35	95,8%	98,3%	99,3%	99,2%	96,6%
20623	65	45	20	40	95,9%	98,2%	99,3%	99,2%	96,9%
20624	65	45	20	45	95,9%	98,2%	99,3%	99,3%	97,1%

20625	65	45	20	50	96,0%	98,2%	99,3%	99,3%	97,3%
20626	65	45	20	55	96,1%	98,2%	99,4%	99,4%	97,4%
20627	65	45	20	60	96,1%	98,2%	99,4%	99,4%	97,6%
20628	65	45	20	65	96,2%	98,1%	99,4%	99,5%	97,8%
20629	65	45	20	70	96,3%	98,1%	99,4%	99,5%	97,9%
20630	65	45	20	75	96,3%	98,1%	99,4%	99,5%	98,0%
20631	65	45	20	80	96,4%	98,1%	99,5%	99,6%	98,2%
20632	65	45	25	20	94,4%	97,9%	99,1%	98,9%	95,7%
20633	65	45	25	25	94,5%	97,9%	99,1%	99,0%	96,0%
20634	65	45	25	30	94,6%	97,9%	99,1%	99,1%	96,3%
20635	65	45	25	35	94,7%	97,8%	99,2%	99,1%	96,5%
20636	65	45	25	40	94,8%	97,8%	99,2%	99,2%	96,7%
20637	65	45	25	45	94,8%	97,8%	99,2%	99,2%	96,9%
20638	65	45	25	50	94,9%	97,7%	99,2%	99,3%	97,1%
20639	65	45	25	55	95,0%	97,7%	99,3%	99,3%	97,3%
20640	65	45	25	60	95,1%	97,7%	99,3%	99,4%	97,5%
20641	65	45	25	65	95,2%	97,7%	99,3%	99,4%	97,7%
20642	65	45	25	70	95,3%	97,6%	99,3%	99,5%	97,8%
20643	65	45	25	75	95,3%	97,6%	99,4%	99,5%	98,0%
20644	65	45	25	80	95,4%	97,6%	99,4%	99,5%	98,1%
20645	65	45	30	20	92,9%	97,4%	99,0%	98,9%	95,6%
20646	65	45	30	25	93,0%	97,4%	99,0%	98,9%	95,9%
20647	65	45	30	30	93,2%	97,3%	99,0%	99,0%	96,1%
20648	65	45	30	35	93,3%	97,3%	99,1%	99,1%	96,4%
20649	65	45	30	40	93,4%	97,2%	99,1%	99,1%	96,6%
20650	65	45	30	45	93,5%	97,2%	99,1%	99,2%	96,8%
20651	65	45	30	50	93,6%	97,2%	99,1%	99,3%	97,0%
20652	65	45	30	55	93,7%	97,1%	99,2%	99,3%	97,2%
20653	65	45	30	60	93,8%	97,1%	99,2%	99,3%	97,4%
20654	65	45	30	65	93,9%	97,1%	99,2%	99,4%	97,6%
20655	65	45	30	70	94,0%	97,0%	99,3%	99,4%	97,7%
20656	65	45	30	75	94,1%	97,0%	99,3%	99,5%	97,9%
20657	65	45	30	80	94,2%	97,0%	99,3%	99,5%	98,0%

20658	65	45	35	20	91,1%	96,7%	98,8%	98,8%	95,4%
20659	65	45	35	25	91,3%	96,7%	98,9%	98,9%	95,7%
20660	65	45	35	30	91,4%	96,6%	98,9%	99,0%	96,0%
20661	65	45	35	35	91,5%	96,6%	98,9%	99,0%	96,2%
20662	65	45	35	40	91,7%	96,6%	99,0%	99,1%	96,5%
20663	65	45	35	45	91,8%	96,5%	99,0%	99,2%	96,7%
20664	65	45	35	50	91,9%	96,5%	99,0%	99,2%	96,9%
20665	65	45	35	55	92,1%	96,4%	99,1%	99,3%	97,1%
20666	65	45	35	60	92,2%	96,4%	99,1%	99,3%	97,3%
20667	65	45	35	65	92,3%	96,3%	99,1%	99,4%	97,5%
20668	65	45	35	70	92,5%	96,3%	99,2%	99,4%	97,6%
20669	65	45	35	75	92,6%	96,2%	99,2%	99,4%	97,8%
20670	65	45	35	80	92,7%	96,2%	99,2%	99,5%	97,9%
20671	65	45	40	20	88,9%	95,9%	98,7%	98,7%	95,2%
20672	65	45	40	25	89,1%	95,9%	98,7%	98,8%	95,5%
20673	65	45	40	30	89,3%	95,8%	98,8%	98,9%	95,8%
20674	65	45	40	35	89,4%	95,8%	98,8%	99,0%	96,1%
20675	65	45	40	40	89,6%	95,7%	98,8%	99,0%	96,3%
20676	65	45	40	45	89,8%	95,7%	98,9%	99,1%	96,6%
20677	65	45	40	50	89,9%	95,6%	98,9%	99,2%	96,8%
20678	65	45	40	55	90,1%	95,5%	98,9%	99,2%	97,0%
20679	65	45	40	60	90,2%	95,5%	99,0%	99,3%	97,2%
20680	65	45	40	65	90,4%	95,4%	99,0%	99,3%	97,4%
20681	65	45	40	70	90,5%	95,4%	99,0%	99,4%	97,5%
20682	65	45	40	75	90,7%	95,3%	99,1%	99,4%	97,7%
20683	65	45	40	80	90,8%	95,3%	99,1%	99,4%	97,8%
20684	65	45	45	20	86,2%	94,9%	98,5%	98,7%	95,0%
20685	65	45	45	25	86,4%	94,8%	98,5%	98,7%	95,3%
20686	65	45	45	30	86,6%	94,8%	98,6%	98,8%	95,6%
20687	65	45	45	35	86,9%	94,7%	98,6%	98,9%	95,9%
20688	65	45	45	40	87,1%	94,6%	98,7%	99,0%	96,2%
20689	65	45	45	45	87,2%	94,6%	98,7%	99,0%	96,4%
20690	65	45	45	50	87,4%	94,5%	98,8%	99,1%	96,6%

20691	65	45	45	55	87,6%	94,4%	98,8%	99,2%	96,9%
20692	65	45	45	60	87,8%	94,4%	98,8%	99,2%	97,1%
20693	65	45	45	65	88,0%	94,3%	98,9%	99,3%	97,2%
20694	65	45	45	70	88,2%	94,2%	98,9%	99,3%	97,4%
20695	65	45	45	75	88,4%	94,2%	99,0%	99,4%	97,6%
20696	65	45	45	80	88,6%	94,1%	99,0%	99,4%	97,7%
20697	65	45	50	20	83,0%	93,7%	98,3%	98,6%	94,8%
20698	65	45	50	25	83,3%	93,6%	98,3%	98,7%	95,1%
20699	65	45	50	30	83,5%	93,5%	98,4%	98,8%	95,5%
20700	65	45	50	35	83,8%	93,4%	98,5%	98,8%	95,7%
20701	65	45	50	40	84,0%	93,4%	98,5%	98,9%	96,0%
20702	65	45	50	45	84,2%	93,3%	98,6%	99,0%	96,3%
20703	65	45	50	50	84,5%	93,2%	98,6%	99,0%	96,5%
20704	65	45	50	55	84,7%	93,1%	98,6%	99,1%	96,7%
20705	65	45	50	60	84,9%	93,0%	98,7%	99,2%	96,9%
20706	65	45	50	65	85,2%	92,9%	98,7%	99,2%	97,1%
20707	65	45	50	70	85,4%	92,8%	98,8%	99,3%	97,3%
20708	65	45	50	75	85,6%	92,8%	98,8%	99,3%	97,5%
20709	65	45	50	80	85,8%	92,7%	98,9%	99,4%	97,7%
20710	65	45	55	20	79,3%	92,2%	98,1%	98,5%	94,6%
20711	65	45	55	25	79,6%	92,1%	98,1%	98,6%	95,0%
20712	65	45	55	30	79,8%	92,0%	98,2%	98,7%	95,3%
20713	65	45	55	35	80,1%	91,9%	98,3%	98,8%	95,6%
20714	65	45	55	40	80,4%	91,8%	98,3%	98,8%	95,9%
20715	65	45	55	45	80,7%	91,7%	98,4%	98,9%	96,1%
20716	65	45	55	50	81,0%	91,6%	98,4%	99,0%	96,4%
20717	65	45	55	55	81,2%	91,5%	98,5%	99,1%	96,6%
20718	65	45	55	60	81,5%	91,4%	98,5%	99,1%	96,8%
20719	65	45	55	65	81,8%	91,3%	98,6%	99,2%	97,0%
20720	65	45	55	70	82,0%	91,2%	98,6%	99,2%	97,2%
20721	65	45	55	75	82,3%	91,0%	98,7%	99,3%	97,4%
20722	65	45	55	80	82,5%	90,9%	98,7%	99,3%	97,6%
20723	65	45	60	20	74,9%	90,3%	97,8%	98,4%	94,4%

20724	65	45	60	25	75,3%	90,2%	97,9%	98,5%	94,8%
20725	65	45	60	30	75,6%	90,1%	98,0%	98,6%	95,1%
20726	65	45	60	35	75,9%	90,0%	98,0%	98,7%	95,4%
20727	65	45	60	40	76,2%	89,8%	98,1%	98,8%	95,7%
20728	65	45	60	45	76,5%	89,7%	98,2%	98,9%	96,0%
20729	65	45	60	50	76,9%	89,6%	98,2%	98,9%	96,2%
20730	65	45	60	55	77,2%	89,5%	98,3%	99,0%	96,5%
20731	65	45	60	60	77,5%	89,4%	98,3%	99,1%	96,7%
20732	65	45	60	65	77,8%	89,2%	98,4%	99,1%	96,9%
20733	65	45	60	70	78,1%	89,1%	98,4%	99,2%	97,1%
20734	65	45	60	75	78,4%	89,0%	98,5%	99,2%	97,3%
20735	65	45	60	80	78,7%	88,8%	98,5%	99,3%	97,5%
20736	65	45	65	20	70,0%	88,1%	97,5%	98,3%	94,2%
20737	65	45	65	25	70,4%	88,0%	97,6%	98,4%	94,5%
20738	65	45	65	30	70,7%	87,8%	97,7%	98,5%	94,9%
20739	65	45	65	35	71,1%	87,7%	97,8%	98,6%	95,2%
20740	65	45	65	40	71,5%	87,5%	97,8%	98,7%	95,5%
20741	65	45	65	45	71,8%	87,4%	97,9%	98,8%	95,8%
20742	65	45	65	50	72,2%	87,2%	98,0%	98,9%	96,1%
20743	65	45	65	55	72,5%	87,1%	98,1%	98,9%	96,3%
20744	65	45	65	60	72,9%	87,0%	98,1%	99,0%	96,6%
20745	65	45	65	65	73,2%	86,8%	98,2%	99,1%	96,8%
20746	65	45	65	70	73,6%	86,7%	98,2%	99,1%	97,0%
20747	65	45	65	75	73,9%	86,5%	98,3%	99,2%	97,2%
20748	65	45	65	80	74,3%	86,3%	98,4%	99,2%	97,4%
20749	65	45	70	20	64,6%	85,5%	97,2%	98,2%	94,0%
20750	65	45	70	25	65,0%	85,3%	97,3%	98,3%	94,3%
20751	65	45	70	30	65,4%	85,1%	97,4%	98,4%	94,7%
20752	65	45	70	35	65,8%	85,0%	97,5%	98,5%	95,0%
20753	65	45	70	40	66,2%	84,8%	97,6%	98,6%	95,3%
20754	65	45	70	45	66,6%	84,6%	97,6%	98,7%	95,6%
20755	65	45	70	50	67,0%	84,4%	97,7%	98,8%	95,9%
20756	65	45	70	55	67,3%	84,3%	97,8%	98,9%	96,2%

20757	65	45	70	60	67,7%	84,1%	97,9%	98,9%	96,4%
20758	65	45	70	65	68,1%	83,9%	97,9%	99,0%	96,6%
20759	65	45	70	70	68,5%	83,7%	98,0%	99,1%	96,9%
20760	65	45	70	75	68,9%	83,6%	98,1%	99,1%	97,1%
20761	65	45	70	80	69,3%	83,4%	98,1%	99,2%	97,2%
20762	65	45	75	20	58,8%	82,3%	96,9%	98,1%	93,7%
20763	65	45	75	25	59,2%	82,1%	97,0%	98,2%	94,1%
20764	65	45	75	30	59,6%	82,0%	97,1%	98,3%	94,5%
20765	65	45	75	35	60,0%	81,8%	97,2%	98,4%	94,8%
20766	65	45	75	40	60,4%	81,6%	97,3%	98,5%	95,1%
20767	65	45	75	45	60,9%	81,4%	97,3%	98,6%	95,5%
20768	65	45	75	50	61,3%	81,2%	97,4%	98,7%	95,7%
20769	65	45	75	55	61,7%	81,0%	97,5%	98,8%	96,0%
20770	65	45	75	60	62,1%	80,8%	97,6%	98,9%	96,3%
20771	65	45	75	65	62,5%	80,6%	97,7%	99,0%	96,5%
20772	65	45	75	70	62,9%	80,4%	97,8%	99,0%	96,7%
20773	65	45	75	75	63,4%	80,1%	97,8%	99,1%	96,9%
20774	65	45	75	80	63,8%	79,9%	97,9%	99,1%	97,1%
20775	65	45	80	20	52,7%	78,7%	96,5%	98,0%	93,5%
20776	65	45	80	25	53,1%	78,5%	96,6%	98,1%	93,9%
20777	65	45	80	30	53,5%	78,3%	96,7%	98,2%	94,3%
20778	65	45	80	35	54,0%	78,1%	96,8%	98,3%	94,6%
20779	65	45	80	40	54,4%	77,8%	96,9%	98,4%	95,0%
20780	65	45	80	45	54,9%	77,6%	97,0%	98,5%	95,3%
20781	65	45	80	50	55,3%	77,4%	97,1%	98,6%	95,6%
20782	65	45	80	55	55,7%	77,2%	97,2%	98,7%	95,9%
20783	65	45	80	60	56,2%	76,9%	97,3%	98,8%	96,1%
20784	65	45	80	65	56,6%	76,7%	97,4%	98,9%	96,4%
20785	65	45	80	70	57,0%	76,5%	97,5%	99,0%	96,6%
20786	65	45	80	75	57,5%	76,2%	97,5%	99,0%	96,8%
20787	65	45	80	80	57,9%	76,0%	97,6%	99,1%	97,0%
20788	65	50	20	20	95,9%	98,5%	99,2%	98,9%	95,5%
20789	65	50	20	25	96,0%	98,4%	99,2%	99,0%	95,8%

20790	65	50	20	30	96,1%	98,4%	99,2%	99,1%	96,1%
20791	65	50	20	35	96,1%	98,4%	99,2%	99,1%	96,3%
20792	65	50	20	40	96,2%	98,4%	99,3%	99,2%	96,6%
20793	65	50	20	45	96,3%	98,4%	99,3%	99,2%	96,8%
20794	65	50	20	50	96,3%	98,3%	99,3%	99,3%	97,0%
20795	65	50	20	55	96,4%	98,3%	99,3%	99,3%	97,2%
20796	65	50	20	60	96,4%	98,3%	99,4%	99,4%	97,4%
20797	65	50	20	65	96,5%	98,3%	99,4%	99,4%	97,5%
20798	65	50	20	70	96,6%	98,2%	99,4%	99,5%	97,7%
20799	65	50	20	75	96,6%	98,2%	99,4%	99,5%	97,8%
20800	65	50	20	80	96,7%	98,2%	99,4%	99,5%	98,0%
20801	65	50	25	20	94,8%	98,1%	99,0%	98,9%	95,3%
20802	65	50	25	25	94,9%	98,0%	99,1%	98,9%	95,6%
20803	65	50	25	30	95,0%	98,0%	99,1%	99,0%	95,9%
20804	65	50	25	35	95,1%	98,0%	99,1%	99,1%	96,2%
20805	65	50	25	40	95,2%	98,0%	99,2%	99,1%	96,4%
20806	65	50	25	45	95,3%	97,9%	99,2%	99,2%	96,6%
20807	65	50	25	50	95,3%	97,9%	99,2%	99,2%	96,9%
20808	65	50	25	55	95,4%	97,9%	99,2%	99,3%	97,1%
20809	65	50	25	60	95,5%	97,9%	99,3%	99,3%	97,3%
20810	65	50	25	65	95,6%	97,8%	99,3%	99,4%	97,4%
20811	65	50	25	70	95,6%	97,8%	99,3%	99,4%	97,6%
20812	65	50	25	75	95,7%	97,8%	99,3%	99,5%	97,8%
20813	65	50	25	80	95,8%	97,7%	99,4%	99,5%	97,9%
20814	65	50	30	20	93,5%	97,6%	98,9%	98,8%	95,2%
20815	65	50	30	25	93,6%	97,5%	99,0%	98,9%	95,5%
20816	65	50	30	30	93,7%	97,5%	99,0%	98,9%	95,7%
20817	65	50	30	35	93,8%	97,5%	99,0%	99,0%	96,0%
20818	65	50	30	40	93,9%	97,5%	99,1%	99,1%	96,3%
20819	65	50	30	45	94,0%	97,4%	99,1%	99,1%	96,5%
20820	65	50	30	50	94,1%	97,4%	99,1%	99,2%	96,7%
20821	65	50	30	55	94,2%	97,4%	99,1%	99,2%	96,9%
20822	65	50	30	60	94,3%	97,3%	99,2%	99,3%	97,1%

20823	65	50	30	65	94,4%	97,3%	99,2%	99,3%	97,3%
20824	65	50	30	70	94,5%	97,3%	99,2%	99,4%	97,5%
20825	65	50	30	75	94,6%	97,2%	99,3%	99,4%	97,7%
20826	65	50	30	80	94,7%	97,2%	99,3%	99,5%	97,8%
20827	65	50	35	20	91,8%	97,0%	98,8%	98,7%	95,0%
20828	65	50	35	25	91,9%	96,9%	98,8%	98,8%	95,3%
20829	65	50	35	30	92,1%	96,9%	98,9%	98,9%	95,6%
20830	65	50	35	35	92,2%	96,9%	98,9%	99,0%	95,9%
20831	65	50	35	40	92,3%	96,8%	98,9%	99,0%	96,1%
20832	65	50	35	45	92,5%	96,8%	99,0%	99,1%	96,4%
20833	65	50	35	50	92,6%	96,7%	99,0%	99,1%	96,6%
20834	65	50	35	55	92,7%	96,7%	99,0%	99,2%	96,8%
20835	65	50	35	60	92,8%	96,6%	99,1%	99,3%	97,0%
20836	65	50	35	65	92,9%	96,6%	99,1%	99,3%	97,2%
20837	65	50	35	70	93,0%	96,6%	99,1%	99,4%	97,4%
20838	65	50	35	75	93,2%	96,5%	99,2%	99,4%	97,6%
20839	65	50	35	80	93,3%	96,5%	99,2%	99,4%	97,7%
20840	65	50	40	20	89,8%	96,2%	98,6%	98,6%	94,8%
20841	65	50	40	25	89,9%	96,2%	98,7%	98,7%	95,1%
20842	65	50	40	30	90,1%	96,1%	98,7%	98,8%	95,4%
20843	65	50	40	35	90,2%	96,1%	98,8%	98,9%	95,7%
20844	65	50	40	40	90,4%	96,0%	98,8%	99,0%	96,0%
20845	65	50	40	45	90,5%	96,0%	98,8%	99,0%	96,2%
20846	65	50	40	50	90,7%	95,9%	98,9%	99,1%	96,5%
20847	65	50	40	55	90,8%	95,9%	98,9%	99,2%	96,7%
20848	65	50	40	60	91,0%	95,8%	98,9%	99,2%	96,9%
20849	65	50	40	65	91,1%	95,8%	99,0%	99,3%	97,1%
20850	65	50	40	70	91,3%	95,7%	99,0%	99,3%	97,3%
20851	65	50	40	75	91,4%	95,7%	99,0%	99,4%	97,5%
20852	65	50	40	80	91,5%	95,6%	99,1%	99,4%	97,6%
20853	65	50	45	20	87,3%	95,3%	98,4%	98,6%	94,6%
20854	65	50	45	25	87,5%	95,2%	98,5%	98,6%	94,9%
20855	65	50	45	30	87,6%	95,2%	98,5%	98,7%	95,2%

20856	65	50	45	35	87,8%	95,1%	98,6%	98,8%	95,5%
20857	65	50	45	40	88,0%	95,0%	98,6%	98,9%	95,8%
20858	65	50	45	45	88,2%	95,0%	98,7%	99,0%	96,1%
20859	65	50	45	50	88,4%	94,9%	98,7%	99,0%	96,3%
20860	65	50	45	55	88,6%	94,8%	98,8%	99,1%	96,6%
20861	65	50	45	60	88,7%	94,8%	98,8%	99,2%	96,8%
20862	65	50	45	65	88,9%	94,7%	98,8%	99,2%	97,0%
20863	65	50	45	70	89,1%	94,6%	98,9%	99,3%	97,2%
20864	65	50	45	75	89,3%	94,6%	98,9%	99,3%	97,4%
20865	65	50	45	80	89,4%	94,5%	99,0%	99,4%	97,5%
20866	65	50	50	20	84,2%	94,1%	98,2%	98,5%	94,3%
20867	65	50	50	25	84,5%	94,0%	98,3%	98,6%	94,7%
20868	65	50	50	30	84,7%	94,0%	98,4%	98,7%	95,0%
20869	65	50	50	35	84,9%	93,9%	98,4%	98,7%	95,3%
20870	65	50	50	40	85,2%	93,8%	98,5%	98,8%	95,6%
20871	65	50	50	45	85,4%	93,7%	98,5%	98,9%	95,9%
20872	65	50	50	50	85,6%	93,7%	98,6%	99,0%	96,2%
20873	65	50	50	55	85,8%	93,6%	98,6%	99,0%	96,4%
20874	65	50	50	60	86,0%	93,5%	98,7%	99,1%	96,6%
20875	65	50	50	65	86,2%	93,4%	98,7%	99,2%	96,9%
20876	65	50	50	70	86,5%	93,4%	98,7%	99,2%	97,1%
20877	65	50	50	75	86,7%	93,3%	98,8%	99,3%	97,3%
20878	65	50	50	80	86,9%	93,2%	98,8%	99,3%	97,4%
20879	65	50	55	20	80,7%	92,7%	98,0%	98,4%	94,1%
20880	65	50	55	25	81,0%	92,6%	98,1%	98,5%	94,5%
20881	65	50	55	30	81,2%	92,5%	98,1%	98,6%	94,8%
20882	65	50	55	35	81,5%	92,4%	98,2%	98,7%	95,2%
20883	65	50	55	40	81,8%	92,3%	98,3%	98,8%	95,5%
20884	65	50	55	45	82,0%	92,2%	98,3%	98,8%	95,7%
20885	65	50	55	50	82,3%	92,2%	98,4%	98,9%	96,0%
20886	65	50	55	55	82,5%	92,1%	98,4%	99,0%	96,3%
20887	65	50	55	60	82,8%	92,0%	98,5%	99,1%	96,5%
20888	65	50	55	65	83,0%	91,9%	98,5%	99,1%	96,7%

20889	65	50	55	70	83,3%	91,8%	98,6%	99,2%	96,9%
20890	65	50	55	75	83,5%	91,7%	98,6%	99,2%	97,1%
20891	65	50	55	80	83,8%	91,6%	98,7%	99,3%	97,3%
20892	65	50	60	20	76,6%	91,0%	97,8%	98,3%	93,9%
20893	65	50	60	25	76,9%	90,9%	97,8%	98,4%	94,3%
20894	65	50	60	30	77,2%	90,8%	97,9%	98,5%	94,6%
20895	65	50	60	35	77,5%	90,7%	98,0%	98,6%	95,0%
20896	65	50	60	40	77,8%	90,5%	98,0%	98,7%	95,3%
20897	65	50	60	45	78,1%	90,4%	98,1%	98,8%	95,6%
20898	65	50	60	50	78,4%	90,3%	98,2%	98,8%	95,9%
20899	65	50	60	55	78,7%	90,2%	98,2%	98,9%	96,1%
20900	65	50	60	60	79,0%	90,1%	98,3%	99,0%	96,4%
20901	65	50	60	65	79,3%	90,0%	98,3%	99,1%	96,6%
20902	65	50	60	70	79,6%	89,8%	98,4%	99,1%	96,8%
20903	65	50	60	75	79,9%	89,7%	98,4%	99,2%	97,0%
20904	65	50	60	80	80,1%	89,6%	98,5%	99,2%	97,2%
20905	65	50	65	20	71,8%	88,9%	97,5%	98,2%	93,6%
20906	65	50	65	25	72,2%	88,8%	97,5%	98,3%	94,0%
20907	65	50	65	30	72,5%	88,6%	97,6%	98,4%	94,4%
20908	65	50	65	35	72,9%	88,5%	97,7%	98,5%	94,8%
20909	65	50	65	40	73,2%	88,4%	97,8%	98,6%	95,1%
20910	65	50	65	45	73,6%	88,2%	97,9%	98,7%	95,4%
20911	65	50	65	50	73,9%	88,1%	97,9%	98,8%	95,7%
20912	65	50	65	55	74,3%	88,0%	98,0%	98,9%	96,0%
20913	65	50	65	60	74,6%	87,8%	98,1%	98,9%	96,2%
20914	65	50	65	65	74,9%	87,7%	98,1%	99,0%	96,5%
20915	65	50	65	70	75,3%	87,5%	98,2%	99,1%	96,7%
20916	65	50	65	75	75,6%	87,4%	98,2%	99,1%	96,9%
20917	65	50	65	80	75,9%	87,2%	98,3%	99,2%	97,1%
20918	65	50	70	20	66,6%	86,4%	97,1%	98,0%	93,4%
20919	65	50	70	25	67,0%	86,2%	97,2%	98,2%	93,8%
20920	65	50	70	30	67,4%	86,1%	97,3%	98,3%	94,2%
20921	65	50	70	35	67,7%	85,9%	97,4%	98,4%	94,5%

20922	65	50	70	40	68,1%	85,8%	97,5%	98,5%	94,9%
20923	65	50	70	45	68,5%	85,6%	97,6%	98,6%	95,2%
20924	65	50	70	50	68,9%	85,5%	97,7%	98,7%	95,5%
20925	65	50	70	55	69,3%	85,3%	97,7%	98,8%	95,8%
20926	65	50	70	60	69,6%	85,1%	97,8%	98,9%	96,1%
20927	65	50	70	65	70,0%	85,0%	97,9%	98,9%	96,3%
20928	65	50	70	70	70,4%	84,8%	97,9%	99,0%	96,6%
20929	65	50	70	75	70,7%	84,6%	98,0%	99,1%	96,8%
20930	65	50	70	80	71,1%	84,5%	98,1%	99,1%	97,0%
20931	65	50	75	20	60,9%	83,5%	96,8%	97,9%	93,1%
20932	65	50	75	25	61,3%	83,3%	96,9%	98,1%	93,6%
20933	65	50	75	30	61,7%	83,1%	97,0%	98,2%	94,0%
20934	65	50	75	35	62,1%	82,9%	97,1%	98,3%	94,3%
20935	65	50	75	40	62,5%	82,7%	97,2%	98,4%	94,7%
20936	65	50	75	45	63,0%	82,5%	97,3%	98,5%	95,0%
20937	65	50	75	50	63,4%	82,3%	97,4%	98,6%	95,3%
20938	65	50	75	55	63,8%	82,2%	97,4%	98,7%	95,6%
20939	65	50	75	60	64,2%	82,0%	97,5%	98,8%	95,9%
20940	65	50	75	65	64,6%	81,8%	97,6%	98,9%	96,2%
20941	65	50	75	70	65,0%	81,6%	97,7%	98,9%	96,4%
20942	65	50	75	75	65,4%	81,4%	97,8%	99,0%	96,6%
20943	65	50	75	80	65,8%	81,2%	97,8%	99,1%	96,9%
20944	65	50	80	20	54,9%	80,0%	96,4%	97,8%	92,9%
20945	65	50	80	25	55,3%	79,8%	96,5%	97,9%	93,3%
20946	65	50	80	30	55,7%	79,6%	96,6%	98,1%	93,7%
20947	65	50	80	35	56,2%	79,4%	96,7%	98,2%	94,1%
20948	65	50	80	40	56,6%	79,2%	96,8%	98,3%	94,5%
20949	65	50	80	45	57,0%	78,9%	96,9%	98,4%	94,8%
20950	65	50	80	50	57,5%	78,7%	97,0%	98,5%	95,1%
20951	65	50	80	55	57,9%	78,5%	97,1%	98,6%	95,5%
20952	65	50	80	60	58,3%	78,3%	97,2%	98,7%	95,7%
20953	65	50	80	65	58,8%	78,1%	97,3%	98,8%	96,0%
20954	65	50	80	70	59,2%	77,8%	97,4%	98,9%	96,3%

20955	65	50	80	75	59,6%	77,6%	97,5%	99,0%	96,5%
20956	65	50	80	80	60,0%	77,4%	97,5%	99,0%	96,7%
20957	65	55	20	20	96,3%	98,6%	99,1%	98,8%	95,1%
20958	65	55	20	25	96,3%	98,6%	99,2%	98,9%	95,4%
20959	65	55	20	30	96,4%	98,5%	99,2%	99,0%	95,7%
20960	65	55	20	35	96,4%	98,5%	99,2%	99,1%	96,0%
20961	65	55	20	40	96,5%	98,5%	99,2%	99,1%	96,2%
20962	65	55	20	45	96,6%	98,5%	99,3%	99,2%	96,5%
20963	65	55	20	50	96,6%	98,5%	99,3%	99,2%	96,7%
20964	65	55	20	55	96,7%	98,4%	99,3%	99,3%	96,9%
20965	65	55	20	60	96,7%	98,4%	99,3%	99,3%	97,1%
20966	65	55	20	65	96,8%	98,4%	99,4%	99,4%	97,3%
20967	65	55	20	70	96,8%	98,4%	99,4%	99,4%	97,5%
20968	65	55	20	75	96,9%	98,4%	99,4%	99,5%	97,6%
20969	65	55	20	80	96,9%	98,3%	99,4%	99,5%	97,8%
20970	65	55	25	20	95,3%	98,2%	99,0%	98,8%	94,9%
20971	65	55	25	25	95,3%	98,2%	99,0%	98,9%	95,2%
20972	65	55	25	30	95,4%	98,2%	99,1%	98,9%	95,5%
20973	65	55	25	35	95,5%	98,1%	99,1%	99,0%	95,8%
20974	65	55	25	40	95,6%	98,1%	99,1%	99,1%	96,1%
20975	65	55	25	45	95,6%	98,1%	99,2%	99,1%	96,3%
20976	65	55	25	50	95,7%	98,1%	99,2%	99,2%	96,6%
20977	65	55	25	55	95,8%	98,0%	99,2%	99,2%	96,8%
20978	65	55	25	60	95,9%	98,0%	99,2%	99,3%	97,0%
20979	65	55	25	65	95,9%	98,0%	99,3%	99,3%	97,2%
20980	65	55	25	70	96,0%	98,0%	99,3%	99,4%	97,4%
20981	65	55	25	75	96,1%	97,9%	99,3%	99,4%	97,5%
20982	65	55	25	80	96,1%	97,9%	99,3%	99,5%	97,7%
20983	65	55	30	20	94,0%	97,8%	98,9%	98,7%	94,7%
20984	65	55	30	25	94,1%	97,7%	98,9%	98,8%	95,0%
20985	65	55	30	30	94,2%	97,7%	99,0%	98,9%	95,3%
20986	65	55	30	35	94,3%	97,7%	99,0%	98,9%	95,6%
20987	65	55	30	40	94,4%	97,6%	99,0%	99,0%	95,9%

20988	65	55	30	45	94,5%	97,6%	99,1%	99,1%	96,2%
20989	65	55	30	50	94,6%	97,6%	99,1%	99,1%	96,4%
20990	65	55	30	55	94,7%	97,5%	99,1%	99,2%	96,6%
20991	65	55	30	60	94,8%	97,5%	99,1%	99,2%	96,9%
20992	65	55	30	65	94,8%	97,5%	99,2%	99,3%	97,1%
20993	65	55	30	70	94,9%	97,5%	99,2%	99,3%	97,3%
20994	65	55	30	75	95,0%	97,4%	99,2%	99,4%	97,4%
20995	65	55	30	80	95,1%	97,4%	99,3%	99,4%	97,6%
20996	65	55	35	20	92,5%	97,2%	98,7%	98,6%	94,5%
20997	65	55	35	25	92,6%	97,2%	98,8%	98,7%	94,8%
20998	65	55	35	30	92,7%	97,1%	98,8%	98,8%	95,2%
20999	65	55	35	35	92,8%	97,1%	98,9%	98,9%	95,5%
21000	65	55	35	40	92,9%	97,0%	98,9%	98,9%	95,7%
21001	65	55	35	45	93,1%	97,0%	98,9%	99,0%	96,0%
21002	65	55	35	50	93,2%	97,0%	99,0%	99,1%	96,3%
21003	65	55	35	55	93,3%	96,9%	99,0%	99,1%	96,5%
21004	65	55	35	60	93,4%	96,9%	99,0%	99,2%	96,7%
21005	65	55	35	65	93,5%	96,9%	99,1%	99,3%	96,9%
21006	65	55	35	70	93,6%	96,8%	99,1%	99,3%	97,1%
21007	65	55	35	75	93,7%	96,8%	99,1%	99,3%	97,3%
21008	65	55	35	80	93,8%	96,7%	99,2%	99,4%	97,5%
21009	65	55	40	20	90,5%	96,5%	98,6%	98,5%	94,3%
21010	65	55	40	25	90,7%	96,4%	98,6%	98,6%	94,6%
21011	65	55	40	30	90,8%	96,4%	98,7%	98,7%	95,0%
21012	65	55	40	35	91,0%	96,4%	98,7%	98,8%	95,3%
21013	65	55	40	40	91,1%	96,3%	98,8%	98,9%	95,6%
21014	65	55	40	45	91,3%	96,3%	98,8%	99,0%	95,9%
21015	65	55	40	50	91,4%	96,2%	98,8%	99,0%	96,1%
21016	65	55	40	55	91,6%	96,2%	98,9%	99,1%	96,4%
21017	65	55	40	60	91,7%	96,1%	98,9%	99,1%	96,6%
21018	65	55	40	65	91,8%	96,1%	98,9%	99,2%	96,8%
21019	65	55	40	70	92,0%	96,0%	99,0%	99,3%	97,0%
21020	65	55	40	75	92,1%	96,0%	99,0%	99,3%	97,2%

21021	65	55	40	80	92,2%	95,9%	99,0%	99,4%	97,4%
21022	65	55	45	20	88,2%	95,6%	98,4%	98,4%	94,0%
21023	65	55	45	25	88,4%	95,6%	98,4%	98,5%	94,4%
21024	65	55	45	30	88,6%	95,5%	98,5%	98,6%	94,8%
21025	65	55	45	35	88,7%	95,4%	98,5%	98,7%	95,1%
21026	65	55	45	40	88,9%	95,4%	98,6%	98,8%	95,4%
21027	65	55	45	45	89,1%	95,3%	98,6%	98,9%	95,7%
21028	65	55	45	50	89,3%	95,3%	98,7%	99,0%	96,0%
21029	65	55	45	55	89,4%	95,2%	98,7%	99,0%	96,2%
21030	65	55	45	60	89,6%	95,2%	98,8%	99,1%	96,5%
21031	65	55	45	65	89,8%	95,1%	98,8%	99,2%	96,7%
21032	65	55	45	70	89,9%	95,0%	98,9%	99,2%	96,9%
21033	65	55	45	75	90,1%	95,0%	98,9%	99,3%	97,1%
21034	65	55	45	80	90,2%	94,9%	98,9%	99,3%	97,3%
21035	65	55	50	20	85,4%	94,5%	98,2%	98,3%	93,8%
21036	65	55	50	25	85,6%	94,5%	98,2%	98,5%	94,2%
21037	65	55	50	30	85,8%	94,4%	98,3%	98,6%	94,6%
21038	65	55	50	35	86,0%	94,3%	98,4%	98,6%	94,9%
21039	65	55	50	40	86,2%	94,3%	98,4%	98,7%	95,2%
21040	65	55	50	45	86,5%	94,2%	98,5%	98,8%	95,5%
21041	65	55	50	50	86,7%	94,1%	98,5%	98,9%	95,8%
21042	65	55	50	55	86,9%	94,0%	98,6%	99,0%	96,1%
21043	65	55	50	60	87,1%	94,0%	98,6%	99,0%	96,3%
21044	65	55	50	65	87,3%	93,9%	98,7%	99,1%	96,6%
21045	65	55	50	70	87,5%	93,8%	98,7%	99,2%	96,8%
21046	65	55	50	75	87,6%	93,7%	98,7%	99,2%	97,0%
21047	65	55	50	80	87,8%	93,7%	98,8%	99,3%	97,2%
21048	65	55	55	20	82,0%	93,2%	97,9%	98,2%	93,6%
21049	65	55	55	25	82,3%	93,1%	98,0%	98,4%	94,0%
21050	65	55	55	30	82,5%	93,1%	98,1%	98,5%	94,3%
21051	65	55	55	35	82,8%	93,0%	98,1%	98,6%	94,7%
21052	65	55	55	40	83,1%	92,9%	98,2%	98,7%	95,0%
21053	65	55	55	45	83,3%	92,8%	98,3%	98,7%	95,3%

21054	65	55	55	50	83,5%	92,7%	98,3%	98,8%	95,6%
21055	65	55	55	55	83,8%	92,6%	98,4%	98,9%	95,9%
21056	65	55	55	60	84,0%	92,5%	98,4%	99,0%	96,2%
21057	65	55	55	65	84,3%	92,4%	98,5%	99,0%	96,4%
21058	65	55	55	70	84,5%	92,3%	98,5%	99,1%	96,6%
21059	65	55	55	75	84,7%	92,2%	98,6%	99,2%	96,9%
21060	65	55	55	80	84,9%	92,2%	98,6%	99,2%	97,1%
21061	65	55	60	20	78,1%	91,6%	97,7%	98,1%	93,3%
21062	65	55	60	25	78,4%	91,5%	97,8%	98,3%	93,7%
21063	65	55	60	30	78,7%	91,4%	97,8%	98,4%	94,1%
21064	65	55	60	35	79,0%	91,3%	97,9%	98,5%	94,5%
21065	65	55	60	40	79,3%	91,2%	98,0%	98,6%	94,8%
21066	65	55	60	45	79,6%	91,1%	98,0%	98,7%	95,2%
21067	65	55	60	50	79,9%	91,0%	98,1%	98,8%	95,5%
21068	65	55	60	55	80,1%	90,9%	98,2%	98,8%	95,7%
21069	65	55	60	60	80,4%	90,8%	98,2%	98,9%	96,0%
21070	65	55	60	65	80,7%	90,7%	98,3%	99,0%	96,3%
21071	65	55	60	70	81,0%	90,5%	98,3%	99,1%	96,5%
21072	65	55	60	75	81,2%	90,4%	98,4%	99,1%	96,7%
21073	65	55	60	80	81,5%	90,3%	98,4%	99,2%	96,9%
21074	65	55	65	20	73,6%	89,6%	97,4%	98,0%	93,0%
21075	65	55	65	25	73,9%	89,5%	97,5%	98,1%	93,5%
21076	65	55	65	30	74,3%	89,4%	97,6%	98,3%	93,9%
21077	65	55	65	35	74,6%	89,3%	97,6%	98,4%	94,3%
21078	65	55	65	40	74,9%	89,2%	97,7%	98,5%	94,6%
21079	65	55	65	45	75,3%	89,0%	97,8%	98,6%	95,0%
21080	65	55	65	50	75,6%	88,9%	97,9%	98,7%	95,3%
21081	65	55	65	55	75,9%	88,8%	97,9%	98,8%	95,6%
21082	65	55	65	60	76,2%	88,6%	98,0%	98,8%	95,9%
21083	65	55	65	65	76,6%	88,5%	98,1%	98,9%	96,1%
21084	65	55	65	70	76,9%	88,4%	98,1%	99,0%	96,4%
21085	65	55	65	75	77,2%	88,2%	98,2%	99,1%	96,6%
21086	65	55	65	80	77,5%	88,1%	98,2%	99,1%	96,8%

21087	65	55	70	20	68,5%	87,3%	97,0%	97,9%	92,8%
21088	65	55	70	25	68,9%	87,2%	97,1%	98,0%	93,2%
21089	65	55	70	30	69,3%	87,0%	97,2%	98,2%	93,6%
21090	65	55	70	35	69,7%	86,9%	97,3%	98,3%	94,0%
21091	65	55	70	40	70,0%	86,7%	97,4%	98,4%	94,4%
21092	65	55	70	45	70,4%	86,6%	97,5%	98,5%	94,8%
21093	65	55	70	50	70,8%	86,4%	97,6%	98,6%	95,1%
21094	65	55	70	55	71,1%	86,3%	97,7%	98,7%	95,4%
21095	65	55	70	60	71,5%	86,1%	97,7%	98,8%	95,7%
21096	65	55	70	65	71,8%	85,9%	97,8%	98,9%	96,0%
21097	65	55	70	70	72,2%	85,8%	97,9%	98,9%	96,2%
21098	65	55	70	75	72,6%	85,6%	98,0%	99,0%	96,5%
21099	65	55	70	80	72,9%	85,5%	98,0%	99,1%	96,7%
21100	65	55	75	20	63,0%	84,5%	96,7%	97,8%	92,5%
21101	65	55	75	25	63,4%	84,3%	96,8%	97,9%	93,0%
21102	65	55	75	30	63,8%	84,2%	96,9%	98,0%	93,4%
21103	65	55	75	35	64,2%	84,0%	97,0%	98,2%	93,8%
21104	65	55	75	40	64,6%	83,8%	97,1%	98,3%	94,2%
21105	65	55	75	45	65,0%	83,6%	97,2%	98,4%	94,5%
21106	65	55	75	50	65,4%	83,5%	97,3%	98,5%	94,9%
21107	65	55	75	55	65,8%	83,3%	97,4%	98,6%	95,2%
21108	65	55	75	60	66,2%	83,1%	97,4%	98,7%	95,5%
21109	65	55	75	65	66,6%	82,9%	97,5%	98,8%	95,8%
21110	65	55	75	70	67,0%	82,7%	97,6%	98,9%	96,1%
21111	65	55	75	75	67,4%	82,5%	97,7%	98,9%	96,3%
21112	65	55	75	80	67,8%	82,3%	97,8%	99,0%	96,6%
21113	65	55	80	20	57,1%	81,2%	96,2%	97,6%	92,2%
21114	65	55	80	25	57,5%	81,0%	96,4%	97,8%	92,7%
21115	65	55	80	30	57,9%	80,8%	96,5%	97,9%	93,1%
21116	65	55	80	35	58,3%	80,6%	96,6%	98,1%	93,6%
21117	65	55	80	40	58,8%	80,4%	96,7%	98,2%	94,0%
21118	65	55	80	45	59,2%	80,2%	96,8%	98,3%	94,3%
21119	65	55	80	50	59,6%	80,0%	96,9%	98,4%	94,7%

21120	65	55	80	55	60,1%	79,8%	97,0%	98,5%	95,0%
21121	65	55	80	60	60,5%	79,6%	97,1%	98,6%	95,3%
21122	65	55	80	65	60,9%	79,4%	97,2%	98,7%	95,6%
21123	65	55	80	70	61,3%	79,2%	97,3%	98,8%	95,9%
21124	65	55	80	75	61,7%	79,0%	97,4%	98,9%	96,2%
21125	65	55	80	80	62,1%	78,7%	97,5%	99,0%	96,4%
21126	65	60	20	20	96,6%	98,7%	99,1%	98,8%	94,6%
21127	65	60	20	25	96,6%	98,7%	99,1%	98,8%	95,0%
21128	65	60	20	30	96,7%	98,6%	99,2%	98,9%	95,3%
21129	65	60	20	35	96,7%	98,6%	99,2%	99,0%	95,6%
21130	65	60	20	40	96,8%	98,6%	99,2%	99,1%	95,9%
21131	65	60	20	45	96,8%	98,6%	99,2%	99,1%	96,1%
21132	65	60	20	50	96,9%	98,6%	99,3%	99,2%	96,4%
21133	65	60	20	55	97,0%	98,6%	99,3%	99,2%	96,6%
21134	65	60	20	60	97,0%	98,5%	99,3%	99,3%	96,8%
21135	65	60	20	65	97,1%	98,5%	99,3%	99,3%	97,0%
21136	65	60	20	70	97,1%	98,5%	99,4%	99,4%	97,2%
21137	65	60	20	75	97,2%	98,5%	99,4%	99,4%	97,4%
21138	65	60	20	80	97,2%	98,5%	99,4%	99,5%	97,6%
21139	65	60	25	20	95,6%	98,3%	99,0%	98,7%	94,4%
21140	65	60	25	25	95,7%	98,3%	99,0%	98,8%	94,8%
21141	65	60	25	30	95,8%	98,3%	99,1%	98,8%	95,1%
21142	65	60	25	35	95,9%	98,3%	99,1%	98,9%	95,4%
21143	65	60	25	40	95,9%	98,3%	99,1%	99,0%	95,7%
21144	65	60	25	45	96,0%	98,2%	99,1%	99,1%	96,0%
21145	65	60	25	50	96,1%	98,2%	99,2%	99,1%	96,2%
21146	65	60	25	55	96,1%	98,2%	99,2%	99,2%	96,5%
21147	65	60	25	60	96,2%	98,2%	99,2%	99,2%	96,7%
21148	65	60	25	65	96,3%	98,1%	99,2%	99,3%	96,9%
21149	65	60	25	70	96,3%	98,1%	99,3%	99,3%	97,1%
21150	65	60	25	75	96,4%	98,1%	99,3%	99,4%	97,3%
21151	65	60	25	80	96,4%	98,1%	99,3%	99,4%	97,5%
21152	65	60	30	20	94,5%	97,9%	98,9%	98,6%	94,2%

21153	65	60	30	25	94,6%	97,9%	98,9%	98,7%	94,6%
21154	65	60	30	30	94,7%	97,9%	98,9%	98,8%	94,9%
21155	65	60	30	35	94,8%	97,8%	99,0%	98,9%	95,2%
21156	65	60	30	40	94,8%	97,8%	99,0%	98,9%	95,5%
21157	65	60	30	45	94,9%	97,8%	99,0%	99,0%	95,8%
21158	65	60	30	50	95,0%	97,8%	99,1%	99,1%	96,1%
21159	65	60	30	55	95,1%	97,7%	99,1%	99,1%	96,3%
21160	65	60	30	60	95,2%	97,7%	99,1%	99,2%	96,6%
21161	65	60	30	65	95,3%	97,7%	99,2%	99,2%	96,8%
21162	65	60	30	70	95,3%	97,6%	99,2%	99,3%	97,0%
21163	65	60	30	75	95,4%	97,6%	99,2%	99,3%	97,2%
21164	65	60	30	80	95,5%	97,6%	99,2%	99,4%	97,4%
21165	65	60	35	20	93,1%	97,4%	98,7%	98,5%	94,0%
21166	65	60	35	25	93,2%	97,4%	98,7%	98,6%	94,3%
21167	65	60	35	30	93,3%	97,3%	98,8%	98,7%	94,7%
21168	65	60	35	35	93,4%	97,3%	98,8%	98,8%	95,0%
21169	65	60	35	40	93,5%	97,3%	98,9%	98,9%	95,3%
21170	65	60	35	45	93,6%	97,2%	98,9%	98,9%	95,6%
21171	65	60	35	50	93,7%	97,2%	98,9%	99,0%	95,9%
21172	65	60	35	55	93,8%	97,2%	99,0%	99,1%	96,2%
21173	65	60	35	60	93,9%	97,1%	99,0%	99,1%	96,4%
21174	65	60	35	65	94,0%	97,1%	99,0%	99,2%	96,6%
21175	65	60	35	70	94,1%	97,0%	99,1%	99,2%	96,9%
21176	65	60	35	75	94,2%	97,0%	99,1%	99,3%	97,1%
21177	65	60	35	80	94,3%	97,0%	99,1%	99,3%	97,3%
21178	65	60	40	20	91,3%	96,7%	98,5%	98,4%	93,7%
21179	65	60	40	25	91,4%	96,7%	98,6%	98,5%	94,1%
21180	65	60	40	30	91,6%	96,7%	98,6%	98,6%	94,5%
21181	65	60	40	35	91,7%	96,6%	98,7%	98,7%	94,8%
21182	65	60	40	40	91,8%	96,6%	98,7%	98,8%	95,2%
21183	65	60	40	45	92,0%	96,5%	98,8%	98,9%	95,5%
21184	65	60	40	50	92,1%	96,5%	98,8%	99,0%	95,7%
21185	65	60	40	55	92,2%	96,4%	98,8%	99,0%	96,0%

21186	65	60	40	60	92,3%	96,4%	98,9%	99,1%	96,3%
21187	65	60	40	65	92,5%	96,4%	98,9%	99,1%	96,5%
21188	65	60	40	70	92,6%	96,3%	99,0%	99,2%	96,7%
21189	65	60	40	75	92,7%	96,3%	99,0%	99,3%	96,9%
21190	65	60	40	80	92,8%	96,2%	99,0%	99,3%	97,1%
21191	65	60	45	20	89,1%	95,9%	98,3%	98,3%	93,5%
21192	65	60	45	25	89,3%	95,9%	98,4%	98,4%	93,9%
21193	65	60	45	30	89,4%	95,8%	98,4%	98,5%	94,3%
21194	65	60	45	35	89,6%	95,8%	98,5%	98,6%	94,6%
21195	65	60	45	40	89,8%	95,7%	98,5%	98,7%	95,0%
21196	65	60	45	45	89,9%	95,7%	98,6%	98,8%	95,3%
21197	65	60	45	50	90,1%	95,6%	98,6%	98,9%	95,6%
21198	65	60	45	55	90,2%	95,6%	98,7%	99,0%	95,9%
21199	65	60	45	60	90,4%	95,5%	98,7%	99,0%	96,1%
21200	65	60	45	65	90,6%	95,5%	98,8%	99,1%	96,4%
21201	65	60	45	70	90,7%	95,4%	98,8%	99,2%	96,6%
21202	65	60	45	75	90,8%	95,3%	98,9%	99,2%	96,8%
21203	65	60	45	80	91,0%	95,3%	98,9%	99,3%	97,0%
21204	65	60	50	20	86,5%	94,9%	98,1%	98,2%	93,2%
21205	65	60	50	25	86,7%	94,9%	98,2%	98,3%	93,6%
21206	65	60	50	30	86,9%	94,8%	98,2%	98,4%	94,0%
21207	65	60	50	35	87,1%	94,7%	98,3%	98,5%	94,4%
21208	65	60	50	40	87,3%	94,7%	98,4%	98,6%	94,8%
21209	65	60	50	45	87,5%	94,6%	98,4%	98,7%	95,1%
21210	65	60	50	50	87,7%	94,5%	98,5%	98,8%	95,4%
21211	65	60	50	55	87,8%	94,5%	98,5%	98,9%	95,7%
21212	65	60	50	60	88,0%	94,4%	98,6%	99,0%	96,0%
21213	65	60	50	65	88,2%	94,3%	98,6%	99,0%	96,2%
21214	65	60	50	70	88,4%	94,3%	98,7%	99,1%	96,5%
21215	65	60	50	75	88,6%	94,2%	98,7%	99,2%	96,7%
21216	65	60	50	80	88,8%	94,1%	98,7%	99,2%	96,9%
21217	65	60	55	20	83,3%	93,7%	97,9%	98,1%	93,0%
21218	65	60	55	25	83,5%	93,6%	98,0%	98,2%	93,4%

21219	65	60	55	30	83,8%	93,5%	98,0%	98,3%	93,8%
21220	65	60	55	35	84,0%	93,5%	98,1%	98,5%	94,2%
21221	65	60	55	40	84,3%	93,4%	98,1%	98,6%	94,6%
21222	65	60	55	45	84,5%	93,3%	98,2%	98,7%	94,9%
21223	65	60	55	50	84,7%	93,2%	98,3%	98,7%	95,2%
21224	65	60	55	55	85,0%	93,1%	98,3%	98,8%	95,5%
21225	65	60	55	60	85,2%	93,1%	98,4%	98,9%	95,8%
21226	65	60	55	65	85,4%	93,0%	98,4%	99,0%	96,1%
21227	65	60	55	70	85,6%	92,9%	98,5%	99,0%	96,3%
21228	65	60	55	75	85,8%	92,8%	98,5%	99,1%	96,6%
21229	65	60	55	80	86,0%	92,7%	98,6%	99,2%	96,8%
21230	65	60	60	20	79,6%	92,2%	97,6%	98,0%	92,7%
21231	65	60	60	25	79,9%	92,1%	97,7%	98,1%	93,1%
21232	65	60	60	30	80,2%	92,0%	97,8%	98,2%	93,6%
21233	65	60	60	35	80,4%	91,9%	97,8%	98,4%	94,0%
21234	65	60	60	40	80,7%	91,8%	97,9%	98,5%	94,3%
21235	65	60	60	45	81,0%	91,7%	98,0%	98,6%	94,7%
21236	65	60	60	50	81,2%	91,6%	98,0%	98,7%	95,0%
21237	65	60	60	55	81,5%	91,5%	98,1%	98,8%	95,3%
21238	65	60	60	60	81,8%	91,4%	98,2%	98,8%	95,6%
21239	65	60	60	65	82,0%	91,3%	98,2%	98,9%	95,9%
21240	65	60	60	70	82,3%	91,2%	98,3%	99,0%	96,2%
21241	65	60	60	75	82,6%	91,1%	98,3%	99,1%	96,4%
21242	65	60	60	80	82,8%	91,0%	98,4%	99,1%	96,6%
21243	65	60	65	20	75,3%	90,4%	97,3%	97,9%	92,4%
21244	65	60	65	25	75,6%	90,2%	97,4%	98,0%	92,9%
21245	65	60	65	30	75,9%	90,1%	97,5%	98,1%	93,3%
21246	65	60	65	35	76,3%	90,0%	97,6%	98,3%	93,7%
21247	65	60	65	40	76,6%	89,9%	97,6%	98,4%	94,1%
21248	65	60	65	45	76,9%	89,8%	97,7%	98,5%	94,5%
21249	65	60	65	50	77,2%	89,7%	97,8%	98,6%	94,8%
21250	65	60	65	55	77,5%	89,5%	97,9%	98,7%	95,2%
21251	65	60	65	60	77,8%	89,4%	97,9%	98,8%	95,5%

21252	65	60	65	65	78,1%	89,3%	98,0%	98,8%	95,7%
21253	65	60	65	70	78,4%	89,2%	98,1%	98,9%	96,0%
21254	65	60	65	75	78,7%	89,0%	98,1%	99,0%	96,3%
21255	65	60	65	80	79,0%	88,9%	98,2%	99,1%	96,5%
21256	65	60	70	20	70,4%	88,1%	97,0%	97,7%	92,1%
21257	65	60	70	25	70,8%	88,0%	97,1%	97,9%	92,6%
21258	65	60	70	30	71,1%	87,9%	97,2%	98,0%	93,0%
21259	65	60	70	35	71,5%	87,7%	97,2%	98,2%	93,5%
21260	65	60	70	40	71,9%	87,6%	97,3%	98,3%	93,9%
21261	65	60	70	45	72,2%	87,4%	97,4%	98,4%	94,3%
21262	65	60	70	50	72,6%	87,3%	97,5%	98,5%	94,6%
21263	65	60	70	55	72,9%	87,2%	97,6%	98,6%	95,0%
21264	65	60	70	60	73,3%	87,0%	97,7%	98,7%	95,3%
21265	65	60	70	65	73,6%	86,9%	97,7%	98,8%	95,6%
21266	65	60	70	70	73,9%	86,7%	97,8%	98,9%	95,9%
21267	65	60	70	75	74,3%	86,6%	97,9%	98,9%	96,1%
21268	65	60	70	80	74,6%	86,4%	98,0%	99,0%	96,4%
21269	65	60	75	20	65,0%	85,5%	96,6%	97,6%	91,8%
21270	65	60	75	25	65,4%	85,4%	96,7%	97,8%	92,3%
21271	65	60	75	30	65,8%	85,2%	96,8%	97,9%	92,8%
21272	65	60	75	35	66,2%	85,0%	96,9%	98,0%	93,2%
21273	65	60	75	40	66,6%	84,9%	97,0%	98,2%	93,6%
21274	65	60	75	45	67,0%	84,7%	97,1%	98,3%	94,0%
21275	65	60	75	50	67,4%	84,5%	97,2%	98,4%	94,4%
21276	65	60	75	55	67,8%	84,3%	97,3%	98,5%	94,8%
21277	65	60	75	60	68,2%	84,2%	97,4%	98,6%	95,1%
21278	65	60	75	65	68,5%	84,0%	97,4%	98,7%	95,4%
21279	65	60	75	70	68,9%	83,8%	97,5%	98,8%	95,7%
21280	65	60	75	75	69,3%	83,6%	97,6%	98,9%	96,0%
21281	65	60	75	80	69,7%	83,5%	97,7%	98,9%	96,2%
21282	65	60	80	20	59,2%	82,4%	96,1%	97,5%	91,5%
21283	65	60	80	25	59,6%	82,2%	96,3%	97,6%	92,0%
21284	65	60	80	30	60,1%	82,0%	96,4%	97,8%	92,5%

21285	65	60	80	35	60,5%	81,8%	96,5%	97,9%	93,0%
21286	65	60	80	40	60,9%	81,6%	96,6%	98,1%	93,4%
21287	65	60	80	45	61,3%	81,4%	96,7%	98,2%	93,8%
21288	65	60	80	50	61,7%	81,2%	96,8%	98,3%	94,2%
21289	65	60	80	55	62,2%	81,0%	96,9%	98,4%	94,5%
21290	65	60	80	60	62,6%	80,8%	97,0%	98,5%	94,9%
21291	65	60	80	65	63,0%	80,6%	97,1%	98,6%	95,2%
21292	65	60	80	70	63,4%	80,4%	97,2%	98,7%	95,5%
21293	65	60	80	75	63,8%	80,2%	97,3%	98,8%	95,8%
21294	65	60	80	80	64,2%	80,0%	97,4%	98,9%	96,1%
21295	65	65	20	20	96,8%	98,8%	99,1%	98,7%	94,1%
21296	65	65	20	25	96,9%	98,8%	99,1%	98,8%	94,5%
21297	65	65	20	30	97,0%	98,7%	99,1%	98,8%	94,8%
21298	65	65	20	35	97,0%	98,7%	99,2%	98,9%	95,2%
21299	65	65	20	40	97,1%	98,7%	99,2%	99,0%	95,5%
21300	65	65	20	45	97,1%	98,7%	99,2%	99,0%	95,8%
21301	65	65	20	50	97,2%	98,7%	99,2%	99,1%	96,0%
21302	65	65	20	55	97,2%	98,7%	99,3%	99,2%	96,3%
21303	65	65	20	60	97,2%	98,6%	99,3%	99,2%	96,5%
21304	65	65	20	65	97,3%	98,6%	99,3%	99,3%	96,7%
21305	65	65	20	70	97,3%	98,6%	99,3%	99,3%	96,9%
21306	65	65	20	75	97,4%	98,6%	99,4%	99,4%	97,1%
21307	65	65	20	80	97,4%	98,6%	99,4%	99,4%	97,3%
21308	65	65	25	20	96,0%	98,5%	99,0%	98,6%	93,9%
21309	65	65	25	25	96,1%	98,4%	99,0%	98,7%	94,3%
21310	65	65	25	30	96,1%	98,4%	99,0%	98,8%	94,6%
21311	65	65	25	35	96,2%	98,4%	99,1%	98,8%	95,0%
21312	65	65	25	40	96,3%	98,4%	99,1%	98,9%	95,3%
21313	65	65	25	45	96,3%	98,4%	99,1%	99,0%	95,6%
21314	65	65	25	50	96,4%	98,3%	99,1%	99,1%	95,9%
21315	65	65	25	55	96,4%	98,3%	99,2%	99,1%	96,1%
21316	65	65	25	60	96,5%	98,3%	99,2%	99,2%	96,4%
21317	65	65	25	65	96,6%	98,3%	99,2%	99,2%	96,6%

21318	65	65	25	70	96,6%	98,3%	99,3%	99,3%	96,8%
21319	65	65	25	75	96,7%	98,2%	99,3%	99,3%	97,0%
21320	65	65	25	80	96,7%	98,2%	99,3%	99,4%	97,2%
21321	65	65	30	20	94,9%	98,1%	98,8%	98,5%	93,6%
21322	65	65	30	25	95,0%	98,1%	98,9%	98,6%	94,0%
21323	65	65	30	30	95,1%	98,0%	98,9%	98,7%	94,4%
21324	65	65	30	35	95,2%	98,0%	98,9%	98,8%	94,8%
21325	65	65	30	40	95,3%	98,0%	99,0%	98,9%	95,1%
21326	65	65	30	45	95,3%	98,0%	99,0%	98,9%	95,4%
21327	65	65	30	50	95,4%	97,9%	99,0%	99,0%	95,7%
21328	65	65	30	55	95,5%	97,9%	99,1%	99,1%	96,0%
21329	65	65	30	60	95,6%	97,9%	99,1%	99,1%	96,2%
21330	65	65	30	65	95,6%	97,8%	99,1%	99,2%	96,5%
21331	65	65	30	70	95,7%	97,8%	99,2%	99,2%	96,7%
21332	65	65	30	75	95,8%	97,8%	99,2%	99,3%	96,9%
21333	65	65	30	80	95,9%	97,8%	99,2%	99,3%	97,1%
21334	65	65	35	20	93,6%	97,6%	98,7%	98,4%	93,4%
21335	65	65	35	25	93,7%	97,6%	98,7%	98,5%	93,8%
21336	65	65	35	30	93,8%	97,5%	98,7%	98,6%	94,2%
21337	65	65	35	35	93,9%	97,5%	98,8%	98,7%	94,6%
21338	65	65	35	40	94,0%	97,5%	98,8%	98,8%	94,9%
21339	65	65	35	45	94,1%	97,4%	98,9%	98,9%	95,2%
21340	65	65	35	50	94,2%	97,4%	98,9%	98,9%	95,5%
21341	65	65	35	55	94,3%	97,4%	98,9%	99,0%	95,8%
21342	65	65	35	60	94,4%	97,3%	99,0%	99,1%	96,1%
21343	65	65	35	65	94,5%	97,3%	99,0%	99,1%	96,3%
21344	65	65	35	70	94,6%	97,3%	99,0%	99,2%	96,6%
21345	65	65	35	75	94,7%	97,2%	99,1%	99,2%	96,8%
21346	65	65	35	80	94,8%	97,2%	99,1%	99,3%	97,0%
21347	65	65	40	20	92,0%	97,0%	98,5%	98,3%	93,1%
21348	65	65	40	25	92,1%	96,9%	98,5%	98,4%	93,6%
21349	65	65	40	30	92,2%	96,9%	98,6%	98,5%	94,0%
21350	65	65	40	35	92,3%	96,9%	98,6%	98,6%	94,3%

21351	65	65	40	40	92,5%	96,8%	98,7%	98,7%	94,7%
21352	65	65	40	45	92,6%	96,8%	98,7%	98,8%	95,0%
21353	65	65	40	50	92,7%	96,7%	98,8%	98,9%	95,3%
21354	65	65	40	55	92,8%	96,7%	98,8%	98,9%	95,6%
21355	65	65	40	60	92,9%	96,7%	98,8%	99,0%	95,9%
21356	65	65	40	65	93,1%	96,6%	98,9%	99,1%	96,2%
21357	65	65	40	70	93,2%	96,6%	98,9%	99,1%	96,4%
21358	65	65	40	75	93,3%	96,5%	99,0%	99,2%	96,6%
21359	65	65	40	80	93,4%	96,5%	99,0%	99,3%	96,9%
21360	65	65	45	20	89,9%	96,2%	98,3%	98,2%	92,9%
21361	65	65	45	25	90,1%	96,2%	98,3%	98,3%	93,3%
21362	65	65	45	30	90,2%	96,1%	98,4%	98,4%	93,7%
21363	65	65	45	35	90,4%	96,1%	98,5%	98,5%	94,1%
21364	65	65	45	40	90,6%	96,0%	98,5%	98,6%	94,5%
21365	65	65	45	45	90,7%	96,0%	98,6%	98,7%	94,8%
21366	65	65	45	50	90,9%	95,9%	98,6%	98,8%	95,2%
21367	65	65	45	55	91,0%	95,9%	98,6%	98,9%	95,5%
21368	65	65	45	60	91,1%	95,8%	98,7%	99,0%	95,8%
21369	65	65	45	65	91,3%	95,8%	98,7%	99,0%	96,0%
21370	65	65	45	70	91,4%	95,7%	98,8%	99,1%	96,3%
21371	65	65	45	75	91,6%	95,7%	98,8%	99,1%	96,5%
21372	65	65	45	80	91,7%	95,6%	98,9%	99,2%	96,7%
21373	65	65	50	20	87,5%	95,3%	98,1%	98,1%	92,6%
21374	65	65	50	25	87,7%	95,2%	98,1%	98,2%	93,0%
21375	65	65	50	30	87,9%	95,2%	98,2%	98,3%	93,5%
21376	65	65	50	35	88,0%	95,1%	98,3%	98,4%	93,9%
21377	65	65	50	40	88,2%	95,1%	98,3%	98,5%	94,3%
21378	65	65	50	45	88,4%	95,0%	98,4%	98,6%	94,6%
21379	65	65	50	50	88,6%	94,9%	98,4%	98,7%	95,0%
21380	65	65	50	55	88,8%	94,9%	98,5%	98,8%	95,3%
21381	65	65	50	60	88,9%	94,8%	98,5%	98,9%	95,6%
21382	65	65	50	65	89,1%	94,7%	98,6%	99,0%	95,9%
21383	65	65	50	70	89,3%	94,7%	98,6%	99,0%	96,1%

21384	65	65	50	75	89,4%	94,6%	98,7%	99,1%	96,4%
21385	65	65	50	80	89,6%	94,5%	98,7%	99,2%	96,6%
21386	65	65	55	20	84,5%	94,1%	97,8%	98,0%	92,3%
21387	65	65	55	25	84,7%	94,1%	97,9%	98,1%	92,8%
21388	65	65	55	30	85,0%	94,0%	98,0%	98,2%	93,2%
21389	65	65	55	35	85,2%	93,9%	98,0%	98,3%	93,6%
21390	65	65	55	40	85,4%	93,9%	98,1%	98,4%	94,0%
21391	65	65	55	45	85,6%	93,8%	98,2%	98,6%	94,4%
21392	65	65	55	50	85,8%	93,7%	98,2%	98,6%	94,8%
21393	65	65	55	55	86,1%	93,6%	98,3%	98,7%	95,1%
21394	65	65	55	60	86,3%	93,5%	98,3%	98,8%	95,4%
21395	65	65	55	65	86,5%	93,5%	98,4%	98,9%	95,7%
21396	65	65	55	70	86,7%	93,4%	98,4%	99,0%	96,0%
21397	65	65	55	75	86,9%	93,3%	98,5%	99,0%	96,2%
21398	65	65	55	80	87,1%	93,2%	98,5%	99,1%	96,5%
21399	65	65	60	20	81,0%	92,7%	97,5%	97,8%	92,0%
21400	65	65	60	25	81,3%	92,7%	97,6%	98,0%	92,5%
21401	65	65	60	30	81,5%	92,6%	97,7%	98,1%	93,0%
21402	65	65	60	35	81,8%	92,5%	97,8%	98,2%	93,4%
21403	65	65	60	40	82,1%	92,4%	97,8%	98,4%	93,8%
21404	65	65	60	45	82,3%	92,3%	97,9%	98,5%	94,2%
21405	65	65	60	50	82,6%	92,2%	98,0%	98,6%	94,6%
21406	65	65	60	55	82,8%	92,1%	98,0%	98,7%	94,9%
21407	65	65	60	60	83,1%	92,0%	98,1%	98,7%	95,2%
21408	65	65	60	65	83,3%	91,9%	98,2%	98,8%	95,5%
21409	65	65	60	70	83,6%	91,8%	98,2%	98,9%	95,8%
21410	65	65	60	75	83,8%	91,7%	98,3%	99,0%	96,1%
21411	65	65	60	80	84,0%	91,6%	98,3%	99,0%	96,3%
21412	65	65	65	20	76,9%	91,0%	97,2%	97,7%	91,7%
21413	65	65	65	25	77,2%	90,9%	97,3%	97,9%	92,2%
21414	65	65	65	30	77,5%	90,8%	97,4%	98,0%	92,7%
21415	65	65	65	35	77,8%	90,7%	97,5%	98,1%	93,1%
21416	65	65	65	40	78,1%	90,6%	97,6%	98,3%	93,6%

21417	65	65	65	45	78,4%	90,5%	97,6%	98,4%	94,0%
21418	65	65	65	50	78,7%	90,4%	97,7%	98,5%	94,3%
21419	65	65	65	55	79,0%	90,2%	97,8%	98,6%	94,7%
21420	65	65	65	60	79,3%	90,1%	97,9%	98,7%	95,0%
21421	65	65	65	65	79,6%	90,0%	97,9%	98,8%	95,3%
21422	65	65	65	70	79,9%	89,9%	98,0%	98,8%	95,6%
21423	65	65	65	75	80,2%	89,8%	98,1%	98,9%	95,9%
21424	65	65	65	80	80,4%	89,7%	98,1%	99,0%	96,2%
21425	65	65	70	20	72,2%	88,9%	96,9%	97,6%	91,4%
21426	65	65	70	25	72,6%	88,8%	97,0%	97,7%	91,9%
21427	65	65	70	30	72,9%	88,7%	97,1%	97,9%	92,4%
21428	65	65	70	35	73,3%	88,6%	97,2%	98,0%	92,9%
21429	65	65	70	40	73,6%	88,4%	97,2%	98,1%	93,3%
21430	65	65	70	45	74,0%	88,3%	97,3%	98,3%	93,7%
21431	65	65	70	50	74,3%	88,2%	97,4%	98,4%	94,1%
21432	65	65	70	55	74,6%	88,0%	97,5%	98,5%	94,5%
21433	65	65	70	60	75,0%	87,9%	97,6%	98,6%	94,8%
21434	65	65	70	65	75,3%	87,7%	97,7%	98,7%	95,2%
21435	65	65	70	70	75,6%	87,6%	97,7%	98,8%	95,5%
21436	65	65	70	75	75,9%	87,5%	97,8%	98,8%	95,7%
21437	65	65	70	80	76,3%	87,3%	97,9%	98,9%	96,0%
21438	65	65	75	20	67,0%	86,5%	96,5%	97,4%	91,0%
21439	65	65	75	25	67,4%	86,3%	96,6%	97,6%	91,6%
21440	65	65	75	30	67,8%	86,2%	96,7%	97,7%	92,1%
21441	65	65	75	35	68,2%	86,0%	96,8%	97,9%	92,6%
21442	65	65	75	40	68,5%	85,8%	96,9%	98,0%	93,0%
21443	65	65	75	45	68,9%	85,7%	97,0%	98,2%	93,5%
21444	65	65	75	50	69,3%	85,5%	97,1%	98,3%	93,9%
21445	65	65	75	55	69,7%	85,4%	97,2%	98,4%	94,3%
21446	65	65	75	60	70,0%	85,2%	97,3%	98,5%	94,6%
21447	65	65	75	65	70,4%	85,0%	97,4%	98,6%	95,0%
21448	65	65	75	70	70,8%	84,9%	97,5%	98,7%	95,3%
21449	65	65	75	75	71,1%	84,7%	97,5%	98,8%	95,6%

21450	65	65	75	80	71,5%	84,5%	97,6%	98,9%	95,9%
21451	65	65	80	20	61,3%	83,5%	96,0%	97,3%	90,7%
21452	65	65	80	25	61,8%	83,3%	96,1%	97,4%	91,3%
21453	65	65	80	30	62,2%	83,2%	96,3%	97,6%	91,8%
21454	65	65	80	35	62,6%	83,0%	96,4%	97,8%	92,3%
21455	65	65	80	40	63,0%	82,8%	96,5%	97,9%	92,8%
21456	65	65	80	45	63,4%	82,6%	96,6%	98,0%	93,2%
21457	65	65	80	50	63,8%	82,4%	96,7%	98,2%	93,6%
21458	65	65	80	55	64,2%	82,2%	96,8%	98,3%	94,0%
21459	65	65	80	60	64,6%	82,0%	96,9%	98,4%	94,4%
21460	65	65	80	65	65,0%	81,8%	97,0%	98,5%	94,8%
21461	65	65	80	70	65,4%	81,6%	97,1%	98,6%	95,1%
21462	65	65	80	75	65,8%	81,5%	97,2%	98,7%	95,4%
21463	65	65	80	80	66,2%	81,3%	97,3%	98,8%	95,7%
21464	65	70	20	20	97,1%	98,9%	99,0%	98,6%	93,6%
21465	65	70	20	25	97,2%	98,9%	99,1%	98,7%	94,0%
21466	65	70	20	30	97,2%	98,8%	99,1%	98,7%	94,3%
21467	65	70	20	35	97,3%	98,8%	99,1%	98,8%	94,7%
21468	65	70	20	40	97,3%	98,8%	99,2%	98,9%	95,0%
21469	65	70	20	45	97,3%	98,8%	99,2%	99,0%	95,3%
21470	65	70	20	50	97,4%	98,8%	99,2%	99,0%	95,6%
21471	65	70	20	55	97,4%	98,8%	99,2%	99,1%	95,9%
21472	65	70	20	60	97,5%	98,7%	99,3%	99,2%	96,2%
21473	65	70	20	65	97,5%	98,7%	99,3%	99,2%	96,4%
21474	65	70	20	70	97,6%	98,7%	99,3%	99,3%	96,7%
21475	65	70	20	75	97,6%	98,7%	99,3%	99,3%	96,9%
21476	65	70	20	80	97,6%	98,7%	99,4%	99,4%	97,1%
21477	65	70	25	20	96,3%	98,6%	98,9%	98,5%	93,3%
21478	65	70	25	25	96,4%	98,6%	99,0%	98,6%	93,7%
21479	65	70	25	30	96,4%	98,5%	99,0%	98,7%	94,1%
21480	65	70	25	35	96,5%	98,5%	99,0%	98,8%	94,5%
21481	65	70	25	40	96,6%	98,5%	99,1%	98,8%	94,8%
21482	65	70	25	45	96,6%	98,5%	99,1%	98,9%	95,2%

21483	65	70	25	50	96,7%	98,5%	99,1%	99,0%	95,5%
21484	65	70	25	55	96,7%	98,4%	99,1%	99,1%	95,8%
21485	65	70	25	60	96,8%	98,4%	99,2%	99,1%	96,0%
21486	65	70	25	65	96,8%	98,4%	99,2%	99,2%	96,3%
21487	65	70	25	70	96,9%	98,4%	99,2%	99,2%	96,5%
21488	65	70	25	75	97,0%	98,4%	99,3%	99,3%	96,7%
21489	65	70	25	80	97,0%	98,3%	99,3%	99,3%	96,9%
21490	65	70	30	20	95,3%	98,2%	98,8%	98,4%	93,1%
21491	65	70	30	25	95,4%	98,2%	98,8%	98,5%	93,5%
21492	65	70	30	30	95,5%	98,2%	98,9%	98,6%	93,9%
21493	65	70	30	35	95,6%	98,2%	98,9%	98,7%	94,3%
21494	65	70	30	40	95,6%	98,1%	98,9%	98,8%	94,6%
21495	65	70	30	45	95,7%	98,1%	99,0%	98,8%	95,0%
21496	65	70	30	50	95,8%	98,1%	99,0%	98,9%	95,3%
21497	65	70	30	55	95,9%	98,1%	99,0%	99,0%	95,6%
21498	65	70	30	60	95,9%	98,0%	99,1%	99,1%	95,9%
21499	65	70	30	65	96,0%	98,0%	99,1%	99,1%	96,1%
21500	65	70	30	70	96,1%	98,0%	99,1%	99,2%	96,4%
21501	65	70	30	75	96,1%	98,0%	99,2%	99,2%	96,6%
21502	65	70	30	80	96,2%	97,9%	99,2%	99,3%	96,8%
21503	65	70	35	20	94,1%	97,8%	98,6%	98,3%	92,8%
21504	65	70	35	25	94,2%	97,7%	98,7%	98,4%	93,2%
21505	65	70	35	30	94,3%	97,7%	98,7%	98,5%	93,6%
21506	65	70	35	35	94,4%	97,7%	98,7%	98,6%	94,0%
21507	65	70	35	40	94,5%	97,7%	98,8%	98,7%	94,4%
21508	65	70	35	45	94,6%	97,6%	98,8%	98,8%	94,8%
21509	65	70	35	50	94,7%	97,6%	98,9%	98,9%	95,1%
21510	65	70	35	55	94,8%	97,6%	98,9%	98,9%	95,4%
21511	65	70	35	60	94,9%	97,5%	98,9%	99,0%	95,7%
21512	65	70	35	65	94,9%	97,5%	99,0%	99,1%	96,0%
21513	65	70	35	70	95,0%	97,5%	99,0%	99,1%	96,2%
21514	65	70	35	75	95,1%	97,4%	99,0%	99,2%	96,5%
21515	65	70	35	80	95,2%	97,4%	99,1%	99,2%	96,7%

21516	65	70	40	20	92,6%	97,2%	98,4%	98,2%	92,5%
21517	65	70	40	25	92,7%	97,2%	98,5%	98,3%	93,0%
21518	65	70	40	30	92,8%	97,1%	98,5%	98,4%	93,4%
21519	65	70	40	35	92,9%	97,1%	98,6%	98,5%	93,8%
21520	65	70	40	40	93,1%	97,1%	98,6%	98,6%	94,2%
21521	65	70	40	45	93,2%	97,0%	98,7%	98,7%	94,6%
21522	65	70	40	50	93,3%	97,0%	98,7%	98,8%	94,9%
21523	65	70	40	55	93,4%	96,9%	98,8%	98,9%	95,2%
21524	65	70	40	60	93,5%	96,9%	98,8%	98,9%	95,5%
21525	65	70	40	65	93,6%	96,9%	98,8%	99,0%	95,8%
21526	65	70	40	70	93,7%	96,8%	98,9%	99,1%	96,1%
21527	65	70	40	75	93,8%	96,8%	98,9%	99,1%	96,3%
21528	65	70	40	80	93,9%	96,7%	99,0%	99,2%	96,6%
21529	65	70	45	20	90,7%	96,5%	98,2%	98,1%	92,2%
21530	65	70	45	25	90,9%	96,5%	98,3%	98,2%	92,7%
21531	65	70	45	30	91,0%	96,4%	98,3%	98,3%	93,1%
21532	65	70	45	35	91,1%	96,4%	98,4%	98,4%	93,6%
21533	65	70	45	40	91,3%	96,3%	98,5%	98,5%	94,0%
21534	65	70	45	45	91,4%	96,3%	98,5%	98,6%	94,3%
21535	65	70	45	50	91,6%	96,2%	98,6%	98,7%	94,7%
21536	65	70	45	55	91,7%	96,2%	98,6%	98,8%	95,0%
21537	65	70	45	60	91,8%	96,1%	98,6%	98,9%	95,3%
21538	65	70	45	65	92,0%	96,1%	98,7%	98,9%	95,6%
21539	65	70	45	70	92,1%	96,0%	98,7%	99,0%	95,9%
21540	65	70	45	75	92,2%	96,0%	98,8%	99,1%	96,2%
21541	65	70	45	80	92,3%	95,9%	98,8%	99,1%	96,4%
21542	65	70	50	20	88,4%	95,6%	98,0%	97,9%	91,9%
21543	65	70	50	25	88,6%	95,6%	98,1%	98,1%	92,4%
21544	65	70	50	30	88,8%	95,5%	98,1%	98,2%	92,9%
21545	65	70	50	35	88,9%	95,5%	98,2%	98,3%	93,3%
21546	65	70	50	40	89,1%	95,4%	98,3%	98,4%	93,7%
21547	65	70	50	45	89,3%	95,4%	98,3%	98,5%	94,1%
21548	65	70	50	50	89,4%	95,3%	98,4%	98,6%	94,5%

21549	65	70	50	55	89,6%	95,2%	98,4%	98,7%	94,8%
21550	65	70	50	60	89,8%	95,2%	98,5%	98,8%	95,2%
21551	65	70	50	65	89,9%	95,1%	98,5%	98,9%	95,5%
21552	65	70	50	70	90,1%	95,1%	98,6%	99,0%	95,8%
21553	65	70	50	75	90,3%	95,0%	98,6%	99,0%	96,0%
21554	65	70	50	80	90,4%	94,9%	98,7%	99,1%	96,3%
21555	65	70	55	20	85,6%	94,6%	97,7%	97,8%	91,6%
21556	65	70	55	25	85,8%	94,5%	97,8%	98,0%	92,1%
21557	65	70	55	30	86,1%	94,4%	97,9%	98,1%	92,6%
21558	65	70	55	35	86,3%	94,4%	98,0%	98,2%	93,1%
21559	65	70	55	40	86,5%	94,3%	98,0%	98,3%	93,5%
21560	65	70	55	45	86,7%	94,2%	98,1%	98,4%	93,9%
21561	65	70	55	50	86,9%	94,2%	98,2%	98,5%	94,3%
21562	65	70	55	55	87,1%	94,1%	98,2%	98,6%	94,6%
21563	65	70	55	60	87,3%	94,0%	98,3%	98,7%	95,0%
21564	65	70	55	65	87,5%	93,9%	98,3%	98,8%	95,3%
21565	65	70	55	70	87,7%	93,9%	98,4%	98,9%	95,6%
21566	65	70	55	75	87,9%	93,8%	98,4%	99,0%	95,9%
21567	65	70	55	80	88,0%	93,7%	98,5%	99,0%	96,1%
21568	65	70	60	20	82,3%	93,3%	97,5%	97,7%	91,3%
21569	65	70	60	25	82,6%	93,2%	97,5%	97,8%	91,8%
21570	65	70	60	30	82,8%	93,1%	97,6%	98,0%	92,3%
21571	65	70	60	35	83,1%	93,0%	97,7%	98,1%	92,8%
21572	65	70	60	40	83,3%	92,9%	97,8%	98,2%	93,2%
21573	65	70	60	45	83,6%	92,8%	97,8%	98,3%	93,6%
21574	65	70	60	50	83,8%	92,7%	97,9%	98,5%	94,0%
21575	65	70	60	55	84,0%	92,7%	98,0%	98,6%	94,4%
21576	65	70	60	60	84,3%	92,6%	98,1%	98,7%	94,8%
21577	65	70	60	65	84,5%	92,5%	98,1%	98,7%	95,1%
21578	65	70	60	70	84,7%	92,4%	98,2%	98,8%	95,4%
21579	65	70	60	75	85,0%	92,3%	98,2%	98,9%	95,7%
21580	65	70	60	80	85,2%	92,2%	98,3%	99,0%	96,0%
21581	65	70	65	20	78,4%	91,6%	97,1%	97,5%	90,9%

21582	65	70	65	25	78,7%	91,5%	97,2%	97,7%	91,5%
21583	65	70	65	30	79,0%	91,4%	97,3%	97,8%	92,0%
21584	65	70	65	35	79,3%	91,3%	97,4%	98,0%	92,5%
21585	65	70	65	40	79,6%	91,2%	97,5%	98,1%	93,0%
21586	65	70	65	45	79,9%	91,1%	97,6%	98,2%	93,4%
21587	65	70	65	50	80,2%	91,0%	97,6%	98,4%	93,8%
21588	65	70	65	55	80,4%	90,9%	97,7%	98,5%	94,2%
21589	65	70	65	60	80,7%	90,8%	97,8%	98,6%	94,6%
21590	65	70	65	65	81,0%	90,7%	97,9%	98,7%	94,9%
21591	65	70	65	70	81,3%	90,6%	97,9%	98,7%	95,2%
21592	65	70	65	75	81,5%	90,5%	98,0%	98,8%	95,5%
21593	65	70	65	80	81,8%	90,4%	98,1%	98,9%	95,8%
21594	65	70	70	20	74,0%	89,7%	96,8%	97,4%	90,6%
21595	65	70	70	25	74,3%	89,6%	96,9%	97,6%	91,2%
21596	65	70	70	30	74,6%	89,5%	97,0%	97,7%	91,7%
21597	65	70	70	35	75,0%	89,3%	97,1%	97,9%	92,2%
21598	65	70	70	40	75,3%	89,2%	97,2%	98,0%	92,7%
21599	65	70	70	45	75,6%	89,1%	97,3%	98,1%	93,1%
21600	65	70	70	50	76,0%	89,0%	97,3%	98,3%	93,6%
21601	65	70	70	55	76,3%	88,8%	97,4%	98,4%	94,0%
21602	65	70	70	60	76,6%	88,7%	97,5%	98,5%	94,3%
21603	65	70	70	65	76,9%	88,6%	97,6%	98,6%	94,7%
21604	65	70	70	70	77,2%	88,4%	97,7%	98,7%	95,0%
21605	65	70	70	75	77,5%	88,3%	97,7%	98,8%	95,3%
21606	65	70	70	80	77,8%	88,2%	97,8%	98,8%	95,6%
21607	65	70	75	20	68,9%	87,4%	96,4%	97,2%	90,2%
21608	65	70	75	25	69,3%	87,2%	96,5%	97,4%	90,8%
21609	65	70	75	30	69,7%	87,1%	96,6%	97,6%	91,4%
21610	65	70	75	35	70,1%	86,9%	96,7%	97,7%	91,9%
21611	65	70	75	40	70,4%	86,8%	96,8%	97,9%	92,4%
21612	65	70	75	45	70,8%	86,6%	96,9%	98,0%	92,9%
21613	65	70	75	50	71,2%	86,5%	97,0%	98,1%	93,3%
21614	65	70	75	55	71,5%	86,3%	97,1%	98,3%	93,7%

21615	65	70	75	60	71,9%	86,2%	97,2%	98,4%	94,1%
21616	65	70	75	65	72,2%	86,0%	97,3%	98,5%	94,5%
21617	65	70	75	70	72,6%	85,8%	97,4%	98,6%	94,8%
21618	65	70	75	75	72,9%	85,7%	97,5%	98,7%	95,2%
21619	65	70	75	80	73,3%	85,5%	97,5%	98,8%	95,5%
21620	65	70	80	20	63,4%	84,6%	95,9%	97,1%	89,9%
21621	65	70	80	25	63,8%	84,4%	96,0%	97,2%	90,5%
21622	65	70	80	30	64,2%	84,2%	96,1%	97,4%	91,1%
21623	65	70	80	35	64,6%	84,1%	96,3%	97,6%	91,6%
21624	65	70	80	40	65,0%	83,9%	96,4%	97,8%	92,1%
21625	65	70	80	45	65,4%	83,7%	96,5%	97,9%	92,6%
21626	65	70	80	50	65,8%	83,5%	96,6%	98,0%	93,0%
21627	65	70	80	55	66,2%	83,4%	96,7%	98,2%	93,5%
21628	65	70	80	60	66,6%	83,2%	96,8%	98,3%	93,9%
21629	65	70	80	65	67,0%	83,0%	96,9%	98,4%	94,3%
21630	65	70	80	70	67,4%	82,8%	97,0%	98,5%	94,6%
21631	65	70	80	75	67,8%	82,6%	97,1%	98,6%	95,0%
21632	65	70	80	80	68,2%	82,4%	97,2%	98,7%	95,3%
21633	65	75	20	20	97,3%	99,0%	99,0%	98,5%	93,0%
21634	65	75	20	25	97,4%	98,9%	99,0%	98,6%	93,4%
21635	65	75	20	30	97,4%	98,9%	99,1%	98,7%	93,8%
21636	65	75	20	35	97,5%	98,9%	99,1%	98,7%	94,2%
21637	65	75	20	40	97,5%	98,9%	99,1%	98,8%	94,6%
21638	65	75	20	45	97,6%	98,9%	99,2%	98,9%	94,9%
21639	65	75	20	50	97,6%	98,9%	99,2%	99,0%	95,2%
21640	65	75	20	55	97,6%	98,9%	99,2%	99,0%	95,5%
21641	65	75	20	60	97,7%	98,8%	99,2%	99,1%	95,8%
21642	65	75	20	65	97,7%	98,8%	99,3%	99,2%	96,1%
21643	65	75	20	70	97,8%	98,8%	99,3%	99,2%	96,3%
21644	65	75	20	75	97,8%	98,8%	99,3%	99,3%	96,6%
21645	65	75	20	80	97,8%	98,8%	99,3%	99,3%	96,8%
21646	65	75	25	20	96,6%	98,7%	98,9%	98,4%	92,7%
21647	65	75	25	25	96,7%	98,7%	98,9%	98,5%	93,1%

21648	65	75	25	30	96,7%	98,7%	99,0%	98,6%	93,6%
21649	65	75	25	35	96,8%	98,6%	99,0%	98,7%	94,0%
21650	65	75	25	40	96,8%	98,6%	99,0%	98,7%	94,3%
21651	65	75	25	45	96,9%	98,6%	99,1%	98,8%	94,7%
21652	65	75	25	50	97,0%	98,6%	99,1%	98,9%	95,0%
21653	65	75	25	55	97,0%	98,6%	99,1%	99,0%	95,3%
21654	65	75	25	60	97,1%	98,5%	99,1%	99,0%	95,6%
21655	65	75	25	65	97,1%	98,5%	99,2%	99,1%	95,9%
21656	65	75	25	70	97,2%	98,5%	99,2%	99,2%	96,2%
21657	65	75	25	75	97,2%	98,5%	99,2%	99,2%	96,4%
21658	65	75	25	80	97,3%	98,5%	99,3%	99,3%	96,7%
21659	65	75	30	20	95,7%	98,4%	98,7%	98,3%	92,4%
21660	65	75	30	25	95,8%	98,3%	98,8%	98,4%	92,9%
21661	65	75	30	30	95,9%	98,3%	98,8%	98,5%	93,3%
21662	65	75	30	35	95,9%	98,3%	98,9%	98,6%	93,7%
21663	65	75	30	40	96,0%	98,3%	98,9%	98,7%	94,1%
21664	65	75	30	45	96,1%	98,2%	98,9%	98,8%	94,5%
21665	65	75	30	50	96,1%	98,2%	99,0%	98,8%	94,8%
21666	65	75	30	55	96,2%	98,2%	99,0%	98,9%	95,2%
21667	65	75	30	60	96,3%	98,2%	99,0%	99,0%	95,5%
21668	65	75	30	65	96,3%	98,2%	99,1%	99,1%	95,8%
21669	65	75	30	70	96,4%	98,1%	99,1%	99,1%	96,0%
21670	65	75	30	75	96,4%	98,1%	99,1%	99,2%	96,3%
21671	65	75	30	80	96,5%	98,1%	99,2%	99,2%	96,5%
21672	65	75	35	20	94,6%	97,9%	98,6%	98,1%	92,1%
21673	65	75	35	25	94,7%	97,9%	98,6%	98,3%	92,6%
21674	65	75	35	30	94,8%	97,9%	98,7%	98,4%	93,1%
21675	65	75	35	35	94,9%	97,9%	98,7%	98,5%	93,5%
21676	65	75	35	40	94,9%	97,8%	98,8%	98,6%	93,9%
21677	65	75	35	45	95,0%	97,8%	98,8%	98,7%	94,3%
21678	65	75	35	50	95,1%	97,8%	98,8%	98,8%	94,6%
21679	65	75	35	55	95,2%	97,7%	98,9%	98,8%	95,0%
21680	65	75	35	60	95,3%	97,7%	98,9%	98,9%	95,3%

21681	65	75	35	65	95,3%	97,7%	98,9%	99,0%	95,6%
21682	65	75	35	70	95,4%	97,7%	99,0%	99,1%	95,9%
21683	65	75	35	75	95,5%	97,6%	99,0%	99,1%	96,1%
21684	65	75	35	80	95,6%	97,6%	99,0%	99,2%	96,4%
21685	65	75	40	20	93,2%	97,4%	98,4%	98,0%	91,8%
21686	65	75	40	25	93,3%	97,4%	98,4%	98,2%	92,3%
21687	65	75	40	30	93,4%	97,3%	98,5%	98,3%	92,8%
21688	65	75	40	35	93,5%	97,3%	98,5%	98,4%	93,2%
21689	65	75	40	40	93,6%	97,3%	98,6%	98,5%	93,7%
21690	65	75	40	45	93,7%	97,2%	98,6%	98,6%	94,0%
21691	65	75	40	50	93,8%	97,2%	98,7%	98,7%	94,4%
21692	65	75	40	55	93,9%	97,2%	98,7%	98,8%	94,8%
21693	65	75	40	60	94,0%	97,1%	98,8%	98,9%	95,1%
21694	65	75	40	65	94,1%	97,1%	98,8%	98,9%	95,4%
21695	65	75	40	70	94,2%	97,1%	98,8%	99,0%	95,7%
21696	65	75	40	75	94,3%	97,0%	98,9%	99,1%	96,0%
21697	65	75	40	80	94,4%	97,0%	98,9%	99,1%	96,2%
21698	65	75	45	20	91,4%	96,8%	98,2%	97,9%	91,5%
21699	65	75	45	25	91,6%	96,7%	98,2%	98,1%	92,0%
21700	65	75	45	30	91,7%	96,7%	98,3%	98,2%	92,5%
21701	65	75	45	35	91,8%	96,6%	98,4%	98,3%	93,0%
21702	65	75	45	40	92,0%	96,6%	98,4%	98,4%	93,4%
21703	65	75	45	45	92,1%	96,6%	98,5%	98,5%	93,8%
21704	65	75	45	50	92,2%	96,5%	98,5%	98,6%	94,2%
21705	65	75	45	55	92,3%	96,5%	98,6%	98,7%	94,6%
21706	65	75	45	60	92,5%	96,4%	98,6%	98,8%	94,9%
21707	65	75	45	65	92,6%	96,4%	98,7%	98,9%	95,2%
21708	65	75	45	70	92,7%	96,3%	98,7%	98,9%	95,5%
21709	65	75	45	75	92,8%	96,3%	98,7%	99,0%	95,8%
21710	65	75	45	80	92,9%	96,2%	98,8%	99,1%	96,1%
21711	65	75	50	20	89,3%	96,0%	97,9%	97,8%	91,2%
21712	65	75	50	25	89,5%	95,9%	98,0%	97,9%	91,7%
21713	65	75	50	30	89,6%	95,9%	98,1%	98,1%	92,2%

21714	65	75	50	35	89,8%	95,8%	98,1%	98,2%	92,7%
21715	65	75	50	40	89,9%	95,7%	98,2%	98,3%	93,1%
21716	65	75	50	45	90,1%	95,7%	98,3%	98,4%	93,6%
21717	65	75	50	50	90,3%	95,6%	98,3%	98,5%	94,0%
21718	65	75	50	55	90,4%	95,6%	98,4%	98,6%	94,3%
21719	65	75	50	60	90,6%	95,5%	98,4%	98,7%	94,7%
21720	65	75	50	65	90,7%	95,5%	98,5%	98,8%	95,0%
21721	65	75	50	70	90,9%	95,4%	98,5%	98,9%	95,3%
21722	65	75	50	75	91,0%	95,4%	98,6%	99,0%	95,6%
21723	65	75	50	80	91,1%	95,3%	98,6%	99,0%	95,9%
21724	65	75	55	20	86,7%	95,0%	97,7%	97,7%	90,8%
21725	65	75	55	25	86,9%	94,9%	97,8%	97,8%	91,4%
21726	65	75	55	30	87,1%	94,8%	97,8%	97,9%	91,9%
21727	65	75	55	35	87,3%	94,8%	97,9%	98,1%	92,4%
21728	65	75	55	40	87,5%	94,7%	98,0%	98,2%	92,9%
21729	65	75	55	45	87,7%	94,6%	98,0%	98,3%	93,3%
21730	65	75	55	50	87,9%	94,6%	98,1%	98,4%	93,7%
21731	65	75	55	55	88,0%	94,5%	98,2%	98,5%	94,1%
21732	65	75	55	60	88,2%	94,4%	98,2%	98,6%	94,5%
21733	65	75	55	65	88,4%	94,4%	98,3%	98,7%	94,8%
21734	65	75	55	70	88,6%	94,3%	98,3%	98,8%	95,2%
21735	65	75	55	75	88,8%	94,2%	98,4%	98,9%	95,5%
21736	65	75	55	80	88,9%	94,2%	98,4%	99,0%	95,8%
21737	65	75	60	20	83,6%	93,7%	97,4%	97,5%	90,5%
21738	65	75	60	25	83,8%	93,7%	97,5%	97,7%	91,1%
21739	65	75	60	30	84,1%	93,6%	97,5%	97,8%	91,6%
21740	65	75	60	35	84,3%	93,5%	97,6%	98,0%	92,1%
21741	65	75	60	40	84,5%	93,4%	97,7%	98,1%	92,6%
21742	65	75	60	45	84,7%	93,3%	97,8%	98,2%	93,1%
21743	65	75	60	50	85,0%	93,3%	97,9%	98,3%	93,5%
21744	65	75	60	55	85,2%	93,2%	97,9%	98,4%	93,9%
21745	65	75	60	60	85,4%	93,1%	98,0%	98,5%	94,3%
21746	65	75	60	65	85,6%	93,0%	98,1%	98,6%	94,6%

21747	65	75	60	70	85,9%	92,9%	98,1%	98,7%	95,0%
21748	65	75	60	75	86,1%	92,8%	98,2%	98,8%	95,3%
21749	65	75	60	80	86,3%	92,7%	98,2%	98,9%	95,6%
21750	65	75	65	20	79,9%	92,2%	97,0%	97,4%	90,1%
21751	65	75	65	25	80,2%	92,1%	97,1%	97,5%	90,7%
21752	65	75	65	30	80,5%	92,0%	97,2%	97,7%	91,3%
21753	65	75	65	35	80,7%	91,9%	97,3%	97,8%	91,8%
21754	65	75	65	40	81,0%	91,8%	97,4%	98,0%	92,3%
21755	65	75	65	45	81,3%	91,7%	97,5%	98,1%	92,8%
21756	65	75	65	50	81,5%	91,6%	97,6%	98,2%	93,2%
21757	65	75	65	55	81,8%	91,5%	97,7%	98,4%	93,6%
21758	65	75	65	60	82,1%	91,4%	97,7%	98,5%	94,0%
21759	65	75	65	65	82,3%	91,3%	97,8%	98,6%	94,4%
21760	65	75	65	70	82,6%	91,2%	97,9%	98,7%	94,8%
21761	65	75	65	75	82,8%	91,1%	97,9%	98,7%	95,1%
21762	65	75	65	80	83,1%	91,0%	98,0%	98,8%	95,4%
21763	65	75	70	20	75,6%	90,4%	96,7%	97,2%	89,8%
21764	65	75	70	25	76,0%	90,3%	96,8%	97,4%	90,4%
21765	65	75	70	30	76,3%	90,2%	96,9%	97,5%	90,9%
21766	65	75	70	35	76,6%	90,1%	97,0%	97,7%	91,5%
21767	65	75	70	40	76,9%	89,9%	97,1%	97,9%	92,0%
21768	65	75	70	45	77,2%	89,8%	97,2%	98,0%	92,5%
21769	65	75	70	50	77,5%	89,7%	97,3%	98,1%	93,0%
21770	65	75	70	55	77,8%	89,6%	97,3%	98,2%	93,4%
21771	65	75	70	60	78,1%	89,5%	97,4%	98,4%	93,8%
21772	65	75	70	65	78,4%	89,3%	97,5%	98,5%	94,2%
21773	65	75	70	70	78,7%	89,2%	97,6%	98,6%	94,6%
21774	65	75	70	75	79,0%	89,1%	97,7%	98,7%	94,9%
21775	65	75	70	80	79,3%	89,0%	97,8%	98,8%	95,2%
21776	65	75	75	20	70,8%	88,2%	96,2%	97,0%	89,4%
21777	65	75	75	25	71,2%	88,1%	96,4%	97,2%	90,0%
21778	65	75	75	30	71,5%	87,9%	96,5%	97,4%	90,6%
21779	65	75	75	35	71,9%	87,8%	96,6%	97,6%	91,2%

21780	65	75	75	40	72,2%	87,6%	96,7%	97,7%	91,7%
21781	65	75	75	45	72,6%	87,5%	96,8%	97,9%	92,2%
21782	65	75	75	50	72,9%	87,4%	96,9%	98,0%	92,7%
21783	65	75	75	55	73,3%	87,2%	97,0%	98,1%	93,1%
21784	65	75	75	60	73,6%	87,1%	97,1%	98,3%	93,6%
21785	65	75	75	65	74,0%	86,9%	97,2%	98,4%	94,0%
21786	65	75	75	70	74,3%	86,8%	97,3%	98,5%	94,3%
21787	65	75	75	75	74,6%	86,6%	97,4%	98,6%	94,7%
21788	65	75	75	80	75,0%	86,5%	97,5%	98,7%	95,0%
21789	65	75	80	20	65,5%	85,6%	95,8%	96,8%	89,0%
21790	65	75	80	25	65,9%	85,4%	95,9%	97,0%	89,6%
21791	65	75	80	30	66,2%	85,3%	96,0%	97,2%	90,2%
21792	65	75	80	35	66,6%	85,1%	96,2%	97,4%	90,8%
21793	65	75	80	40	67,0%	84,9%	96,3%	97,6%	91,4%
21794	65	75	80	45	67,4%	84,8%	96,4%	97,7%	91,9%
21795	65	75	80	50	67,8%	84,6%	96,5%	97,9%	92,4%
21796	65	75	80	55	68,2%	84,4%	96,6%	98,0%	92,9%
21797	65	75	80	60	68,6%	84,2%	96,7%	98,2%	93,3%
21798	65	75	80	65	68,9%	84,1%	96,8%	98,3%	93,7%
21799	65	75	80	70	69,3%	83,9%	96,9%	98,4%	94,1%
21800	65	75	80	75	69,7%	83,7%	97,0%	98,5%	94,5%
21801	65	75	80	80	70,1%	83,5%	97,1%	98,6%	94,8%
21802	65	80	20	20	97,6%	99,0%	99,0%	98,3%	92,3%
21803	65	80	20	25	97,6%	99,0%	99,0%	98,4%	92,8%
21804	65	80	20	30	97,6%	99,0%	99,0%	98,5%	93,2%
21805	65	80	20	35	97,7%	99,0%	99,1%	98,6%	93,7%
21806	65	80	20	40	97,7%	99,0%	99,1%	98,7%	94,1%
21807	65	80	20	45	97,8%	99,0%	99,1%	98,8%	94,4%
21808	65	80	20	50	97,8%	99,0%	99,2%	98,9%	94,8%
21809	65	80	20	55	97,8%	98,9%	99,2%	99,0%	95,1%
21810	65	80	20	60	97,9%	98,9%	99,2%	99,0%	95,4%
21811	65	80	20	65	97,9%	98,9%	99,2%	99,1%	95,7%
21812	65	80	20	70	98,0%	98,9%	99,3%	99,2%	96,0%

21813	65	80	20	75	98,0%	98,9%	99,3%	99,2%	96,2%
21814	65	80	20	80	98,0%	98,9%	99,3%	99,3%	96,5%
21815	65	80	25	20	96,9%	98,8%	98,8%	98,2%	92,0%
21816	65	80	25	25	97,0%	98,8%	98,9%	98,4%	92,5%
21817	65	80	25	30	97,0%	98,8%	98,9%	98,5%	93,0%
21818	65	80	25	35	97,1%	98,7%	99,0%	98,6%	93,4%
21819	65	80	25	40	97,1%	98,7%	99,0%	98,7%	93,8%
21820	65	80	25	45	97,2%	98,7%	99,0%	98,7%	94,2%
21821	65	80	25	50	97,2%	98,7%	99,1%	98,8%	94,6%
21822	65	80	25	55	97,3%	98,7%	99,1%	98,9%	94,9%
21823	65	80	25	60	97,3%	98,7%	99,1%	99,0%	95,2%
21824	65	80	25	65	97,3%	98,6%	99,1%	99,0%	95,5%
21825	65	80	25	70	97,4%	98,6%	99,2%	99,1%	95,8%
21826	65	80	25	75	97,4%	98,6%	99,2%	99,2%	96,1%
21827	65	80	25	80	97,5%	98,6%	99,2%	99,2%	96,3%
21828	65	80	30	20	96,1%	98,5%	98,7%	98,1%	91,7%
21829	65	80	30	25	96,1%	98,5%	98,7%	98,2%	92,2%
21830	65	80	30	30	96,2%	98,4%	98,8%	98,4%	92,7%
21831	65	80	30	35	96,3%	98,4%	98,8%	98,5%	93,1%
21832	65	80	30	40	96,3%	98,4%	98,9%	98,6%	93,6%
21833	65	80	30	45	96,4%	98,4%	98,9%	98,7%	94,0%
21834	65	80	30	50	96,5%	98,4%	98,9%	98,8%	94,3%
21835	65	80	30	55	96,5%	98,3%	99,0%	98,8%	94,7%
21836	65	80	30	60	96,6%	98,3%	99,0%	98,9%	95,0%
21837	65	80	30	65	96,6%	98,3%	99,0%	99,0%	95,4%
21838	65	80	30	70	96,7%	98,3%	99,1%	99,1%	95,6%
21839	65	80	30	75	96,7%	98,2%	99,1%	99,1%	95,9%
21840	65	80	30	80	96,8%	98,2%	99,1%	99,2%	96,2%
21841	65	80	35	20	95,0%	98,1%	98,5%	98,0%	91,4%
21842	65	80	35	25	95,1%	98,1%	98,6%	98,1%	91,9%
21843	65	80	35	30	95,2%	98,0%	98,6%	98,3%	92,4%
21844	65	80	35	35	95,3%	98,0%	98,7%	98,4%	92,9%
21845	65	80	35	40	95,3%	98,0%	98,7%	98,5%	93,3%

21846	65	80	35	45	95,4%	98,0%	98,8%	98,6%	93,7%
21847	65	80	35	50	95,5%	97,9%	98,8%	98,7%	94,1%
21848	65	80	35	55	95,6%	97,9%	98,8%	98,8%	94,5%
21849	65	80	35	60	95,7%	97,9%	98,9%	98,8%	94,8%
21850	65	80	35	65	95,7%	97,9%	98,9%	98,9%	95,2%
21851	65	80	35	70	95,8%	97,8%	98,9%	99,0%	95,5%
21852	65	80	35	75	95,9%	97,8%	99,0%	99,1%	95,8%
21853	65	80	35	80	95,9%	97,8%	99,0%	99,1%	96,0%
21854	65	80	40	20	93,7%	97,6%	98,3%	97,9%	91,1%
21855	65	80	40	25	93,8%	97,6%	98,4%	98,0%	91,6%
21856	65	80	40	30	93,9%	97,5%	98,4%	98,2%	92,1%
21857	65	80	40	35	94,0%	97,5%	98,5%	98,3%	92,6%
21858	65	80	40	40	94,1%	97,5%	98,5%	98,4%	93,1%
21859	65	80	40	45	94,2%	97,4%	98,6%	98,5%	93,5%
21860	65	80	40	50	94,3%	97,4%	98,6%	98,6%	93,9%
21861	65	80	40	55	94,4%	97,4%	98,7%	98,7%	94,3%
21862	65	80	40	60	94,5%	97,3%	98,7%	98,8%	94,6%
21863	65	80	40	65	94,6%	97,3%	98,8%	98,9%	95,0%
21864	65	80	40	70	94,7%	97,3%	98,8%	98,9%	95,3%
21865	65	80	40	75	94,8%	97,2%	98,8%	99,0%	95,6%
21866	65	80	40	80	94,9%	97,2%	98,9%	99,1%	95,9%
21867	65	80	45	20	92,1%	97,0%	98,1%	97,8%	90,7%
21868	65	80	45	25	92,2%	97,0%	98,2%	97,9%	91,3%
21869	65	80	45	30	92,4%	96,9%	98,2%	98,0%	91,8%
21870	65	80	45	35	92,5%	96,9%	98,3%	98,2%	92,3%
21871	65	80	45	40	92,6%	96,8%	98,4%	98,3%	92,8%
21872	65	80	45	45	92,7%	96,8%	98,4%	98,4%	93,2%
21873	65	80	45	50	92,8%	96,8%	98,5%	98,5%	93,7%
21874	65	80	45	55	93,0%	96,7%	98,5%	98,6%	94,0%
21875	65	80	45	60	93,1%	96,7%	98,6%	98,7%	94,4%
21876	65	80	45	65	93,2%	96,6%	98,6%	98,8%	94,8%
21877	65	80	45	70	93,3%	96,6%	98,7%	98,9%	95,1%
21878	65	80	45	75	93,4%	96,6%	98,7%	98,9%	95,4%

21879	65	80	45	80	93,5%	96,5%	98,7%	99,0%	95,7%
21880	65	80	50	20	90,1%	96,3%	97,9%	97,6%	90,4%
21881	65	80	50	25	90,3%	96,2%	97,9%	97,8%	91,0%
21882	65	80	50	30	90,4%	96,2%	98,0%	97,9%	91,5%
21883	65	80	50	35	90,6%	96,1%	98,1%	98,1%	92,0%
21884	65	80	50	40	90,7%	96,1%	98,1%	98,2%	92,5%
21885	65	80	50	45	90,9%	96,0%	98,2%	98,3%	93,0%
21886	65	80	50	50	91,0%	96,0%	98,3%	98,4%	93,4%
21887	65	80	50	55	91,2%	95,9%	98,3%	98,5%	93,8%
21888	65	80	50	60	91,3%	95,9%	98,4%	98,6%	94,2%
21889	65	80	50	65	91,4%	95,8%	98,4%	98,7%	94,6%
21890	65	80	50	70	91,6%	95,7%	98,5%	98,8%	94,9%
21891	65	80	50	75	91,7%	95,7%	98,5%	98,9%	95,2%
21892	65	80	50	80	91,8%	95,6%	98,6%	98,9%	95,5%
21893	65	80	55	20	87,7%	95,3%	97,6%	97,5%	90,0%
21894	65	80	55	25	87,9%	95,3%	97,7%	97,6%	90,6%
21895	65	80	55	30	88,1%	95,2%	97,8%	97,8%	91,2%
21896	65	80	55	35	88,2%	95,1%	97,8%	97,9%	91,7%
21897	65	80	55	40	88,4%	95,1%	97,9%	98,1%	92,2%
21898	65	80	55	45	88,6%	95,0%	98,0%	98,2%	92,7%
21899	65	80	55	50	88,8%	95,0%	98,0%	98,3%	93,1%
21900	65	80	55	55	89,0%	94,9%	98,1%	98,4%	93,6%
21901	65	80	55	60	89,1%	94,8%	98,2%	98,5%	94,0%
21902	65	80	55	65	89,3%	94,8%	98,2%	98,6%	94,3%
21903	65	80	55	70	89,5%	94,7%	98,3%	98,7%	94,7%
21904	65	80	55	75	89,6%	94,6%	98,3%	98,8%	95,0%
21905	65	80	55	80	89,8%	94,6%	98,4%	98,9%	95,3%
21906	65	80	60	20	84,8%	94,2%	97,3%	97,3%	89,6%
21907	65	80	60	25	85,0%	94,1%	97,4%	97,5%	90,3%
21908	65	80	60	30	85,2%	94,0%	97,5%	97,7%	90,8%
21909	65	80	60	35	85,4%	94,0%	97,6%	97,8%	91,4%
21910	65	80	60	40	85,6%	93,9%	97,6%	98,0%	91,9%
21911	65	80	60	45	85,9%	93,8%	97,7%	98,1%	92,4%

21912	65	80	60	50	86,1%	93,7%	97,8%	98,2%	92,9%
21913	65	80	60	55	86,3%	93,7%	97,9%	98,3%	93,3%
21914	65	80	60	60	86,5%	93,6%	97,9%	98,4%	93,7%
21915	65	80	60	65	86,7%	93,5%	98,0%	98,5%	94,1%
21916	65	80	60	70	86,9%	93,4%	98,1%	98,6%	94,5%
21917	65	80	60	75	87,1%	93,3%	98,1%	98,7%	94,8%
21918	65	80	60	80	87,3%	93,3%	98,2%	98,8%	95,2%
21919	65	80	65	20	81,3%	92,8%	96,9%	97,2%	89,2%
21920	65	80	65	25	81,5%	92,7%	97,0%	97,3%	89,9%
21921	65	80	65	30	81,8%	92,6%	97,1%	97,5%	90,5%
21922	65	80	65	35	82,1%	92,5%	97,2%	97,7%	91,1%
21923	65	80	65	40	82,3%	92,4%	97,3%	97,8%	91,6%
21924	65	80	65	45	82,6%	92,3%	97,4%	98,0%	92,1%
21925	65	80	65	50	82,8%	92,2%	97,5%	98,1%	92,6%
21926	65	80	65	55	83,1%	92,1%	97,6%	98,2%	93,1%
21927	65	80	65	60	83,3%	92,0%	97,7%	98,3%	93,5%
21928	65	80	65	65	83,6%	91,9%	97,7%	98,5%	93,9%
21929	65	80	65	70	83,8%	91,8%	97,8%	98,6%	94,3%
21930	65	80	65	75	84,1%	91,7%	97,9%	98,6%	94,6%
21931	65	80	65	80	84,3%	91,6%	97,9%	98,7%	95,0%
21932	65	80	70	20	77,2%	91,1%	96,6%	97,0%	88,8%
21933	65	80	70	25	77,5%	91,0%	96,7%	97,2%	89,5%
21934	65	80	70	30	77,8%	90,9%	96,8%	97,4%	90,1%
21935	65	80	70	35	78,2%	90,7%	96,9%	97,5%	90,7%
21936	65	80	70	40	78,5%	90,6%	97,0%	97,7%	91,3%
21937	65	80	70	45	78,7%	90,5%	97,1%	97,8%	91,8%
21938	65	80	70	50	79,0%	90,4%	97,2%	98,0%	92,3%
21939	65	80	70	55	79,3%	90,3%	97,3%	98,1%	92,8%
21940	65	80	70	60	79,6%	90,2%	97,4%	98,2%	93,2%
21941	65	80	70	65	79,9%	90,1%	97,4%	98,4%	93,6%
21942	65	80	70	70	80,2%	89,9%	97,5%	98,5%	94,0%
21943	65	80	70	75	80,5%	89,8%	97,6%	98,6%	94,4%
21944	65	80	70	80	80,7%	89,7%	97,7%	98,7%	94,8%

21945	65	80	75	20	72,6%	89,0%	96,1%	96,8%	88,4%
21946	65	80	75	25	73,0%	88,9%	96,2%	97,0%	89,1%
21947	65	80	75	30	73,3%	88,7%	96,4%	97,2%	89,8%
21948	65	80	75	35	73,6%	88,6%	96,5%	97,4%	90,4%
21949	65	80	75	40	74,0%	88,5%	96,6%	97,6%	90,9%
21950	65	80	75	45	74,3%	88,3%	96,7%	97,7%	91,5%
21951	65	80	75	50	74,7%	88,2%	96,8%	97,9%	92,0%
21952	65	80	75	55	75,0%	88,1%	96,9%	98,0%	92,5%
21953	65	80	75	60	75,3%	87,9%	97,0%	98,1%	93,0%
21954	65	80	75	65	75,6%	87,8%	97,1%	98,3%	93,4%
21955	65	80	75	70	76,0%	87,7%	97,2%	98,4%	93,8%
21956	65	80	75	75	76,3%	87,5%	97,3%	98,5%	94,2%
21957	65	80	75	80	76,6%	87,4%	97,4%	98,6%	94,6%
21958	65	80	80	20	67,4%	86,5%	95,6%	96,6%	88,0%
21959	65	80	80	25	67,8%	86,4%	95,8%	96,8%	88,7%
21960	65	80	80	30	68,2%	86,2%	95,9%	97,0%	89,4%
21961	65	80	80	35	68,6%	86,1%	96,0%	97,2%	90,0%
21962	65	80	80	40	69,0%	85,9%	96,2%	97,4%	90,6%
21963	65	80	80	45	69,3%	85,7%	96,3%	97,6%	91,2%
21964	65	80	80	50	69,7%	85,6%	96,4%	97,7%	91,7%
21965	65	80	80	55	70,1%	85,4%	96,5%	97,9%	92,2%
21966	65	80	80	60	70,5%	85,3%	96,6%	98,0%	92,7%
21967	65	80	80	65	70,8%	85,1%	96,7%	98,1%	93,1%
21968	65	80	80	70	71,2%	84,9%	96,8%	98,3%	93,6%
21969	65	80	80	75	71,5%	84,8%	97,0%	98,4%	94,0%
21970	65	80	80	80	71,9%	84,6%	97,0%	98,5%	94,3%
21971	70	20	20	20	94,6%	98,2%	99,5%	99,5%	98,1%
21972	70	20	20	25	94,7%	98,2%	99,6%	99,6%	98,2%
21973	70	20	20	30	94,8%	98,2%	99,6%	99,6%	98,3%
21974	70	20	20	35	94,9%	98,2%	99,6%	99,6%	98,4%
21975	70	20	20	40	95,0%	98,1%	99,6%	99,6%	98,5%
21976	70	20	20	45	95,1%	98,1%	99,6%	99,7%	98,6%
21977	70	20	20	50	95,2%	98,1%	99,6%	99,7%	98,7%

21978	70	20	20	55	95,2%	98,1%	99,6%	99,7%	98,8%
21979	70	20	20	60	95,3%	98,0%	99,6%	99,7%	98,9%
21980	70	20	20	65	95,4%	98,0%	99,7%	99,8%	99,0%
21981	70	20	20	70	95,5%	98,0%	99,7%	99,8%	99,0%
21982	70	20	20	75	95,5%	98,0%	99,7%	99,8%	99,1%
21983	70	20	20	80	95,6%	97,9%	99,7%	99,8%	99,1%
21984	70	20	25	20	93,2%	97,8%	99,5%	99,5%	98,0%
21985	70	20	25	25	93,4%	97,8%	99,5%	99,5%	98,1%
21986	70	20	25	30	93,5%	97,7%	99,5%	99,6%	98,3%
21987	70	20	25	35	93,6%	97,7%	99,5%	99,6%	98,4%
21988	70	20	25	40	93,7%	97,7%	99,5%	99,6%	98,5%
21989	70	20	25	45	93,8%	97,6%	99,6%	99,7%	98,6%
21990	70	20	25	50	93,9%	97,6%	99,6%	99,7%	98,7%
21991	70	20	25	55	94,0%	97,6%	99,6%	99,7%	98,8%
21992	70	20	25	60	94,1%	97,6%	99,6%	99,7%	98,8%
21993	70	20	25	65	94,2%	97,5%	99,6%	99,7%	98,9%
21994	70	20	25	70	94,3%	97,5%	99,6%	99,8%	99,0%
21995	70	20	25	75	94,4%	97,5%	99,6%	99,8%	99,0%
21996	70	20	25	80	94,5%	97,4%	99,7%	99,8%	99,1%
21997	70	20	30	20	91,5%	97,2%	99,4%	99,5%	97,9%
21998	70	20	30	25	91,6%	97,2%	99,4%	99,5%	98,1%
21999	70	20	30	30	91,8%	97,2%	99,4%	99,5%	98,2%
22000	70	20	30	35	91,9%	97,1%	99,5%	99,6%	98,3%
22001	70	20	30	40	92,0%	97,1%	99,5%	99,6%	98,4%
22002	70	20	30	45	92,2%	97,0%	99,5%	99,6%	98,5%
22003	70	20	30	50	92,3%	97,0%	99,5%	99,7%	98,6%
22004	70	20	30	55	92,4%	97,0%	99,5%	99,7%	98,7%
22005	70	20	30	60	92,5%	96,9%	99,6%	99,7%	98,8%
22006	70	20	30	65	92,7%	96,9%	99,6%	99,7%	98,9%
22007	70	20	30	70	92,8%	96,9%	99,6%	99,7%	98,9%
22008	70	20	30	75	92,9%	96,8%	99,6%	99,8%	99,0%
22009	70	20	30	80	93,0%	96,8%	99,6%	99,8%	99,1%
22010	70	20	35	20	89,4%	96,5%	99,3%	99,4%	97,8%

22011	70	20	35	25	89,5%	96,5%	99,4%	99,5%	98,0%
22012	70	20	35	30	89,7%	96,4%	99,4%	99,5%	98,1%
22013	70	20	35	35	89,9%	96,4%	99,4%	99,5%	98,2%
22014	70	20	35	40	90,0%	96,4%	99,4%	99,6%	98,3%
22015	70	20	35	45	90,2%	96,3%	99,4%	99,6%	98,5%
22016	70	20	35	50	90,3%	96,3%	99,5%	99,6%	98,6%
22017	70	20	35	55	90,5%	96,2%	99,5%	99,7%	98,6%
22018	70	20	35	60	90,6%	96,2%	99,5%	99,7%	98,7%
22019	70	20	35	65	90,8%	96,1%	99,5%	99,7%	98,8%
22020	70	20	35	70	90,9%	96,1%	99,5%	99,7%	98,9%
22021	70	20	35	75	91,1%	96,0%	99,5%	99,7%	99,0%
22022	70	20	35	80	91,2%	96,0%	99,6%	99,8%	99,0%
22023	70	20	40	20	86,8%	95,7%	99,2%	99,4%	97,7%
22024	70	20	40	25	87,0%	95,6%	99,3%	99,4%	97,9%
22025	70	20	40	30	87,2%	95,6%	99,3%	99,5%	98,0%
22026	70	20	40	35	87,4%	95,5%	99,3%	99,5%	98,2%
22027	70	20	40	40	87,6%	95,5%	99,3%	99,6%	98,3%
22028	70	20	40	45	87,8%	95,4%	99,4%	99,6%	98,4%
22029	70	20	40	50	88,0%	95,3%	99,4%	99,6%	98,5%
22030	70	20	40	55	88,2%	95,3%	99,4%	99,6%	98,6%
22031	70	20	40	60	88,3%	95,2%	99,4%	99,7%	98,7%
22032	70	20	40	65	88,5%	95,2%	99,4%	99,7%	98,8%
22033	70	20	40	70	88,7%	95,1%	99,5%	99,7%	98,8%
22034	70	20	40	75	88,9%	95,0%	99,5%	99,7%	98,9%
22035	70	20	40	80	89,0%	95,0%	99,5%	99,7%	99,0%
22036	70	20	45	20	83,7%	94,6%	99,1%	99,4%	97,6%
22037	70	20	45	25	83,9%	94,5%	99,2%	99,4%	97,8%
22038	70	20	45	30	84,2%	94,5%	99,2%	99,5%	97,9%
22039	70	20	45	35	84,4%	94,4%	99,2%	99,5%	98,1%
22040	70	20	45	40	84,6%	94,3%	99,3%	99,5%	98,2%
22041	70	20	45	45	84,9%	94,3%	99,3%	99,6%	98,3%
22042	70	20	45	50	85,1%	94,2%	99,3%	99,6%	98,4%
22043	70	20	45	55	85,3%	94,1%	99,3%	99,6%	98,5%

22044	70	20	45	60	85,5%	94,1%	99,3%	99,6%	98,6%
22045	70	20	45	65	85,8%	94,0%	99,4%	99,7%	98,7%
22046	70	20	45	70	86,0%	93,9%	99,4%	99,7%	98,8%
22047	70	20	45	75	86,2%	93,8%	99,4%	99,7%	98,9%
22048	70	20	45	80	86,4%	93,8%	99,4%	99,7%	99,0%
22049	70	20	50	20	80,1%	93,3%	99,0%	99,3%	97,6%
22050	70	20	50	25	80,3%	93,2%	99,1%	99,4%	97,7%
22051	70	20	50	30	80,6%	93,1%	99,1%	99,4%	97,9%
22052	70	20	50	35	80,9%	93,1%	99,1%	99,5%	98,0%
22053	70	20	50	40	81,2%	93,0%	99,2%	99,5%	98,1%
22054	70	20	50	45	81,4%	92,9%	99,2%	99,5%	98,3%
22055	70	20	50	50	81,7%	92,8%	99,2%	99,6%	98,4%
22056	70	20	50	55	82,0%	92,7%	99,2%	99,6%	98,5%
22057	70	20	50	60	82,2%	92,6%	99,3%	99,6%	98,6%
22058	70	20	50	65	82,5%	92,5%	99,3%	99,6%	98,7%
22059	70	20	50	70	82,7%	92,4%	99,3%	99,7%	98,8%
22060	70	20	50	75	83,0%	92,3%	99,3%	99,7%	98,8%
22061	70	20	50	80	83,2%	92,3%	99,4%	99,7%	98,9%
22062	70	20	55	20	75,8%	91,7%	98,9%	99,3%	97,5%
22063	70	20	55	25	76,1%	91,6%	98,9%	99,3%	97,6%
22064	70	20	55	30	76,5%	91,5%	99,0%	99,4%	97,8%
22065	70	20	55	35	76,8%	91,4%	99,0%	99,4%	97,9%
22066	70	20	55	40	77,1%	91,3%	99,0%	99,5%	98,1%
22067	70	20	55	45	77,4%	91,2%	99,1%	99,5%	98,2%
22068	70	20	55	50	77,7%	91,1%	99,1%	99,5%	98,3%
22069	70	20	55	55	78,0%	91,0%	99,1%	99,6%	98,4%
22070	70	20	55	60	78,3%	90,9%	99,2%	99,6%	98,5%
22071	70	20	55	65	78,6%	90,8%	99,2%	99,6%	98,6%
22072	70	20	55	70	78,9%	90,7%	99,2%	99,6%	98,7%
22073	70	20	55	75	79,2%	90,5%	99,2%	99,7%	98,8%
22074	70	20	55	80	79,5%	90,4%	99,3%	99,7%	98,9%
22075	70	20	60	20	71,0%	89,8%	98,8%	99,2%	97,3%
22076	70	20	60	25	71,4%	89,7%	98,8%	99,3%	97,5%

22077	70	20	60	30	71,7%	89,5%	98,8%	99,3%	97,7%
22078	70	20	60	35	72,1%	89,4%	98,9%	99,4%	97,8%
22079	70	20	60	40	72,4%	89,3%	98,9%	99,4%	98,0%
22080	70	20	60	45	72,8%	89,2%	99,0%	99,5%	98,1%
22081	70	20	60	50	73,1%	89,0%	99,0%	99,5%	98,2%
22082	70	20	60	55	73,5%	88,9%	99,0%	99,5%	98,3%
22083	70	20	60	60	73,8%	88,8%	99,1%	99,6%	98,4%
22084	70	20	60	65	74,2%	88,6%	99,1%	99,6%	98,6%
22085	70	20	60	70	74,5%	88,5%	99,1%	99,6%	98,6%
22086	70	20	60	75	74,8%	88,4%	99,1%	99,6%	98,7%
22087	70	20	60	80	75,2%	88,2%	99,2%	99,7%	98,8%
22088	70	20	65	20	65,7%	87,5%	98,6%	99,2%	97,2%
22089	70	20	65	25	66,1%	87,3%	98,7%	99,3%	97,4%
22090	70	20	65	30	66,5%	87,2%	98,7%	99,3%	97,6%
22091	70	20	65	35	66,9%	87,0%	98,7%	99,4%	97,7%
22092	70	20	65	40	67,3%	86,9%	98,8%	99,4%	97,9%
22093	70	20	65	45	67,6%	86,7%	98,8%	99,4%	98,0%
22094	70	20	65	50	68,0%	86,6%	98,9%	99,5%	98,2%
22095	70	20	65	55	68,4%	86,4%	98,9%	99,5%	98,3%
22096	70	20	65	60	68,8%	86,3%	98,9%	99,5%	98,4%
22097	70	20	65	65	69,2%	86,1%	99,0%	99,6%	98,5%
22098	70	20	65	70	69,5%	85,9%	99,0%	99,6%	98,6%
22099	70	20	65	75	69,9%	85,8%	99,0%	99,6%	98,7%
22100	70	20	65	80	70,3%	85,6%	99,1%	99,6%	98,8%
22101	70	20	70	20	59,9%	84,7%	98,4%	99,2%	97,1%
22102	70	20	70	25	60,3%	84,5%	98,5%	99,2%	97,3%
22103	70	20	70	30	60,8%	84,4%	98,5%	99,3%	97,5%
22104	70	20	70	35	61,2%	84,2%	98,6%	99,3%	97,6%
22105	70	20	70	40	61,6%	84,0%	98,6%	99,4%	97,8%
22106	70	20	70	45	62,0%	83,8%	98,7%	99,4%	97,9%
22107	70	20	70	50	62,4%	83,7%	98,7%	99,4%	98,1%
22108	70	20	70	55	62,8%	83,5%	98,8%	99,5%	98,2%
22109	70	20	70	60	63,3%	83,3%	98,8%	99,5%	98,3%

22110	70	20	70	65	63,7%	83,1%	98,8%	99,5%	98,4%
22111	70	20	70	70	64,1%	82,9%	98,9%	99,6%	98,5%
22112	70	20	70	75	64,5%	82,7%	98,9%	99,6%	98,6%
22113	70	20	70	80	64,9%	82,5%	98,9%	99,6%	98,7%
22114	70	20	75	20	53,9%	81,5%	98,2%	99,1%	97,0%
22115	70	20	75	25	54,3%	81,3%	98,3%	99,2%	97,2%
22116	70	20	75	30	54,8%	81,1%	98,3%	99,2%	97,4%
22117	70	20	75	35	55,2%	80,9%	98,4%	99,3%	97,6%
22118	70	20	75	40	55,6%	80,7%	98,4%	99,3%	97,7%
22119	70	20	75	45	56,1%	80,4%	98,5%	99,4%	97,9%
22120	70	20	75	50	56,5%	80,2%	98,5%	99,4%	98,0%
22121	70	20	75	55	56,9%	80,0%	98,6%	99,4%	98,1%
22122	70	20	75	60	57,4%	79,8%	98,6%	99,5%	98,2%
22123	70	20	75	65	57,8%	79,6%	98,7%	99,5%	98,4%
22124	70	20	75	70	58,2%	79,4%	98,7%	99,5%	98,5%
22125	70	20	75	75	58,6%	79,2%	98,8%	99,6%	98,6%
22126	70	20	75	80	59,1%	79,0%	98,8%	99,6%	98,7%
22127	70	20	80	20	47,7%	77,7%	98,0%	99,0%	96,9%
22128	70	20	80	25	48,2%	77,5%	98,1%	99,1%	97,1%
22129	70	20	80	30	48,6%	77,3%	98,1%	99,2%	97,3%
22130	70	20	80	35	49,0%	77,0%	98,2%	99,2%	97,5%
22131	70	20	80	40	49,5%	76,8%	98,2%	99,3%	97,6%
22132	70	20	80	45	49,9%	76,6%	98,3%	99,3%	97,8%
22133	70	20	80	50	50,4%	76,3%	98,4%	99,4%	97,9%
22134	70	20	80	55	50,8%	76,1%	98,4%	99,4%	98,1%
22135	70	20	80	60	51,2%	75,8%	98,5%	99,4%	98,2%
22136	70	20	80	65	51,7%	75,6%	98,5%	99,5%	98,3%
22137	70	20	80	70	52,1%	75,4%	98,6%	99,5%	98,4%
22138	70	20	80	75	52,6%	75,1%	98,6%	99,5%	98,5%
22139	70	20	80	80	53,0%	74,9%	98,7%	99,6%	98,6%
22140	70	25	20	20	95,1%	98,4%	99,5%	99,5%	97,9%
22141	70	25	20	25	95,2%	98,3%	99,5%	99,5%	98,0%
22142	70	25	20	30	95,2%	98,3%	99,6%	99,6%	98,2%

22143	70	25	20	35	95,3%	98,3%	99,6%	99,6%	98,3%
22144	70	25	20	40	95,4%	98,3%	99,6%	99,6%	98,4%
22145	70	25	20	45	95,5%	98,3%	99,6%	99,6%	98,5%
22146	70	25	20	50	95,5%	98,2%	99,6%	99,7%	98,6%
22147	70	25	20	55	95,6%	98,2%	99,6%	99,7%	98,7%
22148	70	25	20	60	95,7%	98,2%	99,6%	99,7%	98,8%
22149	70	25	20	65	95,8%	98,2%	99,6%	99,7%	98,9%
22150	70	25	20	70	95,8%	98,1%	99,7%	99,7%	98,9%
22151	70	25	20	75	95,9%	98,1%	99,7%	99,8%	99,0%
22152	70	25	20	80	96,0%	98,1%	99,7%	99,8%	99,1%
22153	70	25	25	20	93,8%	98,0%	99,5%	99,5%	97,8%
22154	70	25	25	25	93,9%	97,9%	99,5%	99,5%	97,9%
22155	70	25	25	30	94,0%	97,9%	99,5%	99,5%	98,1%
22156	70	25	25	35	94,1%	97,9%	99,5%	99,6%	98,2%
22157	70	25	25	40	94,2%	97,8%	99,5%	99,6%	98,3%
22158	70	25	25	45	94,3%	97,8%	99,5%	99,6%	98,4%
22159	70	25	25	50	94,4%	97,8%	99,6%	99,6%	98,5%
22160	70	25	25	55	94,5%	97,8%	99,6%	99,7%	98,6%
22161	70	25	25	60	94,6%	97,7%	99,6%	99,7%	98,7%
22162	70	25	25	65	94,6%	97,7%	99,6%	99,7%	98,8%
22163	70	25	25	70	94,7%	97,7%	99,6%	99,7%	98,9%
22164	70	25	25	75	94,8%	97,6%	99,6%	99,8%	99,0%
22165	70	25	25	80	94,9%	97,6%	99,6%	99,8%	99,0%
22166	70	25	30	20	92,2%	97,4%	99,4%	99,4%	97,7%
22167	70	25	30	25	92,3%	97,4%	99,4%	99,5%	97,9%
22168	70	25	30	30	92,4%	97,4%	99,4%	99,5%	98,0%
22169	70	25	30	35	92,5%	97,3%	99,5%	99,5%	98,1%
22170	70	25	30	40	92,7%	97,3%	99,5%	99,6%	98,3%
22171	70	25	30	45	92,8%	97,3%	99,5%	99,6%	98,4%
22172	70	25	30	50	92,9%	97,2%	99,5%	99,6%	98,5%
22173	70	25	30	55	93,0%	97,2%	99,5%	99,7%	98,6%
22174	70	25	30	60	93,1%	97,2%	99,5%	99,7%	98,7%
22175	70	25	30	65	93,2%	97,1%	99,6%	99,7%	98,8%

22176	70	25	30	70	93,4%	97,1%	99,6%	99,7%	98,8%
22177	70	25	30	75	93,5%	97,0%	99,6%	99,7%	98,9%
22178	70	25	30	80	93,6%	97,0%	99,6%	99,8%	99,0%
22179	70	25	35	20	90,2%	96,8%	99,3%	99,4%	97,6%
22180	70	25	35	25	90,4%	96,7%	99,3%	99,4%	97,8%
22181	70	25	35	30	90,5%	96,7%	99,4%	99,5%	97,9%
22182	70	25	35	35	90,7%	96,7%	99,4%	99,5%	98,1%
22183	70	25	35	40	90,8%	96,6%	99,4%	99,5%	98,2%
22184	70	25	35	45	90,9%	96,6%	99,4%	99,6%	98,3%
22185	70	25	35	50	91,1%	96,5%	99,4%	99,6%	98,4%
22186	70	25	35	55	91,2%	96,5%	99,5%	99,6%	98,5%
22187	70	25	35	60	91,4%	96,4%	99,5%	99,7%	98,6%
22188	70	25	35	65	91,5%	96,4%	99,5%	99,7%	98,7%
22189	70	25	35	70	91,6%	96,4%	99,5%	99,7%	98,8%
22190	70	25	35	75	91,8%	96,3%	99,5%	99,7%	98,9%
22191	70	25	35	80	91,9%	96,3%	99,5%	99,7%	98,9%
22192	70	25	40	20	87,8%	96,0%	99,2%	99,4%	97,5%
22193	70	25	40	25	88,0%	95,9%	99,2%	99,4%	97,7%
22194	70	25	40	30	88,2%	95,9%	99,3%	99,4%	97,8%
22195	70	25	40	35	88,3%	95,8%	99,3%	99,5%	98,0%
22196	70	25	40	40	88,5%	95,8%	99,3%	99,5%	98,1%
22197	70	25	40	45	88,7%	95,7%	99,3%	99,5%	98,2%
22198	70	25	40	50	88,9%	95,7%	99,4%	99,6%	98,3%
22199	70	25	40	55	89,0%	95,6%	99,4%	99,6%	98,5%
22200	70	25	40	60	89,2%	95,6%	99,4%	99,6%	98,6%
22201	70	25	40	65	89,4%	95,5%	99,4%	99,7%	98,6%
22202	70	25	40	70	89,6%	95,5%	99,4%	99,7%	98,7%
22203	70	25	40	75	89,7%	95,4%	99,5%	99,7%	98,8%
22204	70	25	40	80	89,9%	95,3%	99,5%	99,7%	98,9%
22205	70	25	45	20	84,9%	95,0%	99,1%	99,3%	97,4%
22206	70	25	45	25	85,1%	94,9%	99,1%	99,4%	97,6%
22207	70	25	45	30	85,3%	94,9%	99,2%	99,4%	97,7%
22208	70	25	45	35	85,5%	94,8%	99,2%	99,5%	97,9%

22209	70	25	45	40	85,8%	94,7%	99,2%	99,5%	98,0%
22210	70	25	45	45	86,0%	94,7%	99,3%	99,5%	98,2%
22211	70	25	45	50	86,2%	94,6%	99,3%	99,6%	98,3%
22212	70	25	45	55	86,4%	94,5%	99,3%	99,6%	98,4%
22213	70	25	45	60	86,6%	94,5%	99,3%	99,6%	98,5%
22214	70	25	45	65	86,8%	94,4%	99,4%	99,6%	98,6%
22215	70	25	45	70	87,0%	94,3%	99,4%	99,7%	98,7%
22216	70	25	45	75	87,2%	94,3%	99,4%	99,7%	98,8%
22217	70	25	45	80	87,4%	94,2%	99,4%	99,7%	98,8%
22218	70	25	50	20	81,4%	93,8%	99,0%	99,3%	97,3%
22219	70	25	50	25	81,7%	93,7%	99,0%	99,3%	97,5%
22220	70	25	50	30	82,0%	93,6%	99,1%	99,4%	97,7%
22221	70	25	50	35	82,2%	93,5%	99,1%	99,4%	97,8%
22222	70	25	50	40	82,5%	93,5%	99,1%	99,5%	97,9%
22223	70	25	50	45	82,7%	93,4%	99,2%	99,5%	98,1%
22224	70	25	50	50	83,0%	93,3%	99,2%	99,5%	98,2%
22225	70	25	50	55	83,2%	93,2%	99,2%	99,6%	98,3%
22226	70	25	50	60	83,5%	93,1%	99,2%	99,6%	98,4%
22227	70	25	50	65	83,7%	93,1%	99,3%	99,6%	98,5%
22228	70	25	50	70	84,0%	93,0%	99,3%	99,6%	98,6%
22229	70	25	50	75	84,2%	92,9%	99,3%	99,7%	98,7%
22230	70	25	50	80	84,4%	92,8%	99,3%	99,7%	98,8%
22231	70	25	55	20	77,4%	92,3%	98,9%	99,2%	97,2%
22232	70	25	55	25	77,7%	92,2%	98,9%	99,3%	97,4%
22233	70	25	55	30	78,0%	92,1%	98,9%	99,3%	97,6%
22234	70	25	55	35	78,3%	92,0%	99,0%	99,4%	97,7%
22235	70	25	55	40	78,6%	91,9%	99,0%	99,4%	97,9%
22236	70	25	55	45	78,9%	91,8%	99,0%	99,5%	98,0%
22237	70	25	55	50	79,2%	91,7%	99,1%	99,5%	98,1%
22238	70	25	55	55	79,5%	91,6%	99,1%	99,5%	98,3%
22239	70	25	55	60	79,8%	91,5%	99,1%	99,6%	98,4%
22240	70	25	55	65	80,1%	91,4%	99,2%	99,6%	98,5%
22241	70	25	55	70	80,3%	91,3%	99,2%	99,6%	98,6%

22242	70	25	55	75	80,6%	91,2%	99,2%	99,6%	98,7%
22243	70	25	55	80	80,9%	91,1%	99,2%	99,7%	98,8%
22244	70	25	60	20	72,8%	90,5%	98,7%	99,2%	97,1%
22245	70	25	60	25	73,2%	90,4%	98,8%	99,2%	97,3%
22246	70	25	60	30	73,5%	90,3%	98,8%	99,3%	97,5%
22247	70	25	60	35	73,8%	90,1%	98,8%	99,3%	97,6%
22248	70	25	60	40	74,2%	90,0%	98,9%	99,4%	97,8%
22249	70	25	60	45	74,5%	89,9%	98,9%	99,4%	97,9%
22250	70	25	60	50	74,8%	89,8%	99,0%	99,5%	98,1%
22251	70	25	60	55	75,2%	89,7%	99,0%	99,5%	98,2%
22252	70	25	60	60	75,5%	89,5%	99,0%	99,5%	98,3%
22253	70	25	60	65	75,8%	89,4%	99,1%	99,6%	98,4%
22254	70	25	60	70	76,2%	89,3%	99,1%	99,6%	98,5%
22255	70	25	60	75	76,5%	89,2%	99,1%	99,6%	98,6%
22256	70	25	60	80	76,8%	89,0%	99,1%	99,6%	98,7%
22257	70	25	65	20	67,7%	88,3%	98,6%	99,1%	97,0%
22258	70	25	65	25	68,0%	88,2%	98,6%	99,2%	97,2%
22259	70	25	65	30	68,4%	88,0%	98,7%	99,3%	97,3%
22260	70	25	65	35	68,8%	87,9%	98,7%	99,3%	97,5%
22261	70	25	65	40	69,2%	87,7%	98,7%	99,3%	97,7%
22262	70	25	65	45	69,6%	87,6%	98,8%	99,4%	97,8%
22263	70	25	65	50	69,9%	87,5%	98,8%	99,4%	98,0%
22264	70	25	65	55	70,3%	87,3%	98,9%	99,5%	98,1%
22265	70	25	65	60	70,7%	87,2%	98,9%	99,5%	98,2%
22266	70	25	65	65	71,0%	87,0%	98,9%	99,5%	98,3%
22267	70	25	65	70	71,4%	86,9%	99,0%	99,6%	98,5%
22268	70	25	65	75	71,7%	86,7%	99,0%	99,6%	98,6%
22269	70	25	65	80	72,1%	86,6%	99,0%	99,6%	98,6%
22270	70	25	70	20	62,0%	85,7%	98,4%	99,1%	96,8%
22271	70	25	70	25	62,4%	85,5%	98,4%	99,1%	97,1%
22272	70	25	70	30	62,9%	85,4%	98,5%	99,2%	97,2%
22273	70	25	70	35	63,3%	85,2%	98,5%	99,3%	97,4%
22274	70	25	70	40	63,7%	85,0%	98,6%	99,3%	97,6%

22275	70	25	70	45	64,1%	84,9%	98,6%	99,4%	97,7%
22276	70	25	70	50	64,5%	84,7%	98,7%	99,4%	97,9%
22277	70	25	70	55	64,9%	84,5%	98,7%	99,4%	98,0%
22278	70	25	70	60	65,3%	84,4%	98,8%	99,5%	98,2%
22279	70	25	70	65	65,7%	84,2%	98,8%	99,5%	98,3%
22280	70	25	70	70	66,1%	84,0%	98,8%	99,5%	98,4%
22281	70	25	70	75	66,5%	83,8%	98,9%	99,6%	98,5%
22282	70	25	70	80	66,9%	83,7%	98,9%	99,6%	98,6%
22283	70	25	75	20	56,1%	82,6%	98,2%	99,0%	96,7%
22284	70	25	75	25	56,5%	82,4%	98,2%	99,1%	96,9%
22285	70	25	75	30	56,9%	82,2%	98,3%	99,2%	97,1%
22286	70	25	75	35	57,4%	82,0%	98,3%	99,2%	97,3%
22287	70	25	75	40	57,8%	81,9%	98,4%	99,3%	97,5%
22288	70	25	75	45	58,2%	81,7%	98,4%	99,3%	97,6%
22289	70	25	75	50	58,7%	81,5%	98,5%	99,4%	97,8%
22290	70	25	75	55	59,1%	81,3%	98,5%	99,4%	97,9%
22291	70	25	75	60	59,5%	81,1%	98,6%	99,4%	98,1%
22292	70	25	75	65	59,9%	80,9%	98,6%	99,5%	98,2%
22293	70	25	75	70	60,4%	80,7%	98,7%	99,5%	98,3%
22294	70	25	75	75	60,8%	80,5%	98,7%	99,5%	98,4%
22295	70	25	75	80	61,2%	80,2%	98,8%	99,6%	98,5%
22296	70	25	80	20	49,9%	79,0%	97,9%	99,0%	96,6%
22297	70	25	80	25	50,4%	78,8%	98,0%	99,0%	96,8%
22298	70	25	80	30	50,8%	78,6%	98,1%	99,1%	97,0%
22299	70	25	80	35	51,3%	78,4%	98,1%	99,2%	97,2%
22300	70	25	80	40	51,7%	78,2%	98,2%	99,2%	97,4%
22301	70	25	80	45	52,1%	77,9%	98,2%	99,3%	97,6%
22302	70	25	80	50	52,6%	77,7%	98,3%	99,3%	97,7%
22303	70	25	80	55	53,0%	77,5%	98,4%	99,4%	97,9%
22304	70	25	80	60	53,4%	77,3%	98,4%	99,4%	98,0%
22305	70	25	80	65	53,9%	77,0%	98,5%	99,4%	98,1%
22306	70	25	80	70	54,3%	76,8%	98,5%	99,5%	98,2%
22307	70	25	80	75	54,8%	76,6%	98,6%	99,5%	98,4%

22308	70	25	80	80	55,2%	76,3%	98,6%	99,5%	98,5%
22309	70	30	20	20	95,5%	98,5%	99,5%	99,5%	97,7%
22310	70	30	20	25	95,5%	98,5%	99,5%	99,5%	97,8%
22311	70	30	20	30	95,6%	98,5%	99,5%	99,5%	98,0%
22312	70	30	20	35	95,7%	98,4%	99,6%	99,6%	98,1%
22313	70	30	20	40	95,8%	98,4%	99,6%	99,6%	98,2%
22314	70	30	20	45	95,8%	98,4%	99,6%	99,6%	98,3%
22315	70	30	20	50	95,9%	98,4%	99,6%	99,6%	98,5%
22316	70	30	20	55	96,0%	98,3%	99,6%	99,7%	98,6%
22317	70	30	20	60	96,0%	98,3%	99,6%	99,7%	98,6%
22318	70	30	20	65	96,1%	98,3%	99,6%	99,7%	98,7%
22319	70	30	20	70	96,2%	98,3%	99,7%	99,7%	98,8%
22320	70	30	20	75	96,2%	98,3%	99,7%	99,7%	98,9%
22321	70	30	20	80	96,3%	98,2%	99,7%	99,8%	99,0%
22322	70	30	25	20	94,3%	98,1%	99,4%	99,4%	97,6%
22323	70	30	25	25	94,4%	98,1%	99,5%	99,5%	97,7%
22324	70	30	25	30	94,5%	98,1%	99,5%	99,5%	97,9%
22325	70	30	25	35	94,6%	98,0%	99,5%	99,5%	98,0%
22326	70	30	25	40	94,6%	98,0%	99,5%	99,6%	98,2%
22327	70	30	25	45	94,7%	98,0%	99,5%	99,6%	98,3%
22328	70	30	25	50	94,8%	98,0%	99,5%	99,6%	98,4%
22329	70	30	25	55	94,9%	97,9%	99,6%	99,6%	98,5%
22330	70	30	25	60	95,0%	97,9%	99,6%	99,7%	98,6%
22331	70	30	25	65	95,1%	97,9%	99,6%	99,7%	98,7%
22332	70	30	25	70	95,2%	97,8%	99,6%	99,7%	98,8%
22333	70	30	25	75	95,2%	97,8%	99,6%	99,7%	98,9%
22334	70	30	25	80	95,3%	97,8%	99,6%	99,8%	98,9%
22335	70	30	30	20	92,8%	97,6%	99,4%	99,4%	97,5%
22336	70	30	30	25	92,9%	97,6%	99,4%	99,4%	97,7%
22337	70	30	30	30	93,0%	97,6%	99,4%	99,5%	97,8%
22338	70	30	30	35	93,1%	97,5%	99,4%	99,5%	97,9%
22339	70	30	30	40	93,2%	97,5%	99,5%	99,5%	98,1%
22340	70	30	30	45	93,4%	97,5%	99,5%	99,6%	98,2%

22341	70	30	30	50	93,5%	97,4%	99,5%	99,6%	98,3%
22342	70	30	30	55	93,6%	97,4%	99,5%	99,6%	98,4%
22343	70	30	30	60	93,7%	97,4%	99,5%	99,7%	98,5%
22344	70	30	30	65	93,8%	97,3%	99,5%	99,7%	98,6%
22345	70	30	30	70	93,9%	97,3%	99,6%	99,7%	98,7%
22346	70	30	30	75	94,0%	97,3%	99,6%	99,7%	98,8%
22347	70	30	30	80	94,1%	97,2%	99,6%	99,7%	98,9%
22348	70	30	35	20	91,0%	97,0%	99,3%	99,4%	97,4%
22349	70	30	35	25	91,1%	97,0%	99,3%	99,4%	97,6%
22350	70	30	35	30	91,2%	96,9%	99,3%	99,4%	97,7%
22351	70	30	35	35	91,4%	96,9%	99,4%	99,5%	97,9%
22352	70	30	35	40	91,5%	96,9%	99,4%	99,5%	98,0%
22353	70	30	35	45	91,7%	96,8%	99,4%	99,5%	98,1%
22354	70	30	35	50	91,8%	96,8%	99,4%	99,6%	98,3%
22355	70	30	35	55	91,9%	96,7%	99,4%	99,6%	98,4%
22356	70	30	35	60	92,0%	96,7%	99,5%	99,6%	98,5%
22357	70	30	35	65	92,2%	96,7%	99,5%	99,7%	98,6%
22358	70	30	35	70	92,3%	96,6%	99,5%	99,7%	98,7%
22359	70	30	35	75	92,4%	96,6%	99,5%	99,7%	98,8%
22360	70	30	35	80	92,5%	96,5%	99,5%	99,7%	98,8%
22361	70	30	40	20	88,7%	96,3%	99,2%	99,3%	97,3%
22362	70	30	40	25	88,9%	96,2%	99,2%	99,4%	97,5%
22363	70	30	40	30	89,1%	96,2%	99,2%	99,4%	97,6%
22364	70	30	40	35	89,2%	96,1%	99,3%	99,4%	97,8%
22365	70	30	40	40	89,4%	96,1%	99,3%	99,5%	97,9%
22366	70	30	40	45	89,6%	96,0%	99,3%	99,5%	98,1%
22367	70	30	40	50	89,7%	96,0%	99,3%	99,5%	98,2%
22368	70	30	40	55	89,9%	95,9%	99,4%	99,6%	98,3%
22369	70	30	40	60	90,0%	95,9%	99,4%	99,6%	98,4%
22370	70	30	40	65	90,2%	95,8%	99,4%	99,6%	98,5%
22371	70	30	40	70	90,4%	95,8%	99,4%	99,7%	98,6%
22372	70	30	40	75	90,5%	95,7%	99,4%	99,7%	98,7%
22373	70	30	40	80	90,7%	95,7%	99,5%	99,7%	98,8%

22374	70	30	45	20	86,0%	95,4%	99,1%	99,3%	97,2%
22375	70	30	45	25	86,2%	95,3%	99,1%	99,3%	97,4%
22376	70	30	45	30	86,4%	95,2%	99,2%	99,4%	97,5%
22377	70	30	45	35	86,6%	95,2%	99,2%	99,4%	97,7%
22378	70	30	45	40	86,8%	95,1%	99,2%	99,4%	97,8%
22379	70	30	45	45	87,0%	95,1%	99,2%	99,5%	98,0%
22380	70	30	45	50	87,2%	95,0%	99,3%	99,5%	98,1%
22381	70	30	45	55	87,4%	94,9%	99,3%	99,6%	98,2%
22382	70	30	45	60	87,6%	94,9%	99,3%	99,6%	98,3%
22383	70	30	45	65	87,8%	94,8%	99,3%	99,6%	98,5%
22384	70	30	45	70	88,0%	94,7%	99,4%	99,6%	98,6%
22385	70	30	45	75	88,2%	94,7%	99,4%	99,7%	98,6%
22386	70	30	45	80	88,3%	94,6%	99,4%	99,7%	98,7%
22387	70	30	50	20	82,7%	94,2%	99,0%	99,2%	97,1%
22388	70	30	50	25	83,0%	94,2%	99,0%	99,3%	97,2%
22389	70	30	50	30	83,2%	94,1%	99,0%	99,3%	97,4%
22390	70	30	50	35	83,5%	94,0%	99,1%	99,4%	97,6%
22391	70	30	50	40	83,7%	93,9%	99,1%	99,4%	97,7%
22392	70	30	50	45	84,0%	93,9%	99,1%	99,5%	97,9%
22393	70	30	50	50	84,2%	93,8%	99,2%	99,5%	98,0%
22394	70	30	50	55	84,4%	93,7%	99,2%	99,5%	98,2%
22395	70	30	50	60	84,7%	93,6%	99,2%	99,6%	98,3%
22396	70	30	50	65	84,9%	93,6%	99,2%	99,6%	98,4%
22397	70	30	50	70	85,1%	93,5%	99,3%	99,6%	98,5%
22398	70	30	50	75	85,3%	93,4%	99,3%	99,6%	98,6%
22399	70	30	50	80	85,6%	93,3%	99,3%	99,7%	98,7%
22400	70	30	55	20	78,9%	92,8%	98,8%	99,2%	96,9%
22401	70	30	55	25	79,2%	92,7%	98,9%	99,2%	97,1%
22402	70	30	55	30	79,5%	92,7%	98,9%	99,3%	97,3%
22403	70	30	55	35	79,8%	92,6%	98,9%	99,3%	97,5%
22404	70	30	55	40	80,1%	92,5%	99,0%	99,4%	97,7%
22405	70	30	55	45	80,4%	92,4%	99,0%	99,4%	97,8%
22406	70	30	55	50	80,6%	92,3%	99,1%	99,5%	97,9%

22407	70	30	55	55	80,9%	92,2%	99,1%	99,5%	98,1%
22408	70	30	55	60	81,2%	92,1%	99,1%	99,5%	98,2%
22409	70	30	55	65	81,4%	92,0%	99,1%	99,6%	98,3%
22410	70	30	55	70	81,7%	91,9%	99,2%	99,6%	98,4%
22411	70	30	55	75	82,0%	91,8%	99,2%	99,6%	98,5%
22412	70	30	55	80	82,2%	91,7%	99,2%	99,6%	98,6%
22413	70	30	60	20	74,5%	91,1%	98,7%	99,1%	96,8%
22414	70	30	60	25	74,9%	91,0%	98,7%	99,2%	97,0%
22415	70	30	60	30	75,2%	90,9%	98,8%	99,2%	97,2%
22416	70	30	60	35	75,5%	90,8%	98,8%	99,3%	97,4%
22417	70	30	60	40	75,8%	90,7%	98,9%	99,3%	97,6%
22418	70	30	60	45	76,2%	90,6%	98,9%	99,4%	97,7%
22419	70	30	60	50	76,5%	90,5%	98,9%	99,4%	97,9%
22420	70	30	60	55	76,8%	90,4%	99,0%	99,5%	98,0%
22421	70	30	60	60	77,1%	90,3%	99,0%	99,5%	98,1%
22422	70	30	60	65	77,4%	90,1%	99,0%	99,5%	98,3%
22423	70	30	60	70	77,7%	90,0%	99,1%	99,6%	98,4%
22424	70	30	60	75	78,0%	89,9%	99,1%	99,6%	98,5%
22425	70	30	60	80	78,3%	89,8%	99,1%	99,6%	98,6%
22426	70	30	65	20	69,6%	89,1%	98,5%	99,1%	96,7%
22427	70	30	65	25	69,9%	89,0%	98,6%	99,1%	96,9%
22428	70	30	65	30	70,3%	88,8%	98,6%	99,2%	97,1%
22429	70	30	65	35	70,7%	88,7%	98,7%	99,2%	97,3%
22430	70	30	65	40	71,0%	88,6%	98,7%	99,3%	97,5%
22431	70	30	65	45	71,4%	88,4%	98,7%	99,3%	97,6%
22432	70	30	65	50	71,8%	88,3%	98,8%	99,4%	97,8%
22433	70	30	65	55	72,1%	88,2%	98,8%	99,4%	97,9%
22434	70	30	65	60	72,5%	88,0%	98,9%	99,5%	98,1%
22435	70	30	65	65	72,8%	87,9%	98,9%	99,5%	98,2%
22436	70	30	65	70	73,2%	87,7%	98,9%	99,5%	98,3%
22437	70	30	65	75	73,5%	87,6%	99,0%	99,6%	98,4%
22438	70	30	65	80	73,8%	87,5%	99,0%	99,6%	98,5%
22439	70	30	70	20	64,1%	86,6%	98,3%	99,0%	96,5%

22440	70	30	70	25	64,5%	86,5%	98,4%	99,1%	96,8%
22441	70	30	70	30	64,9%	86,3%	98,4%	99,1%	97,0%
22442	70	30	70	35	65,3%	86,2%	98,5%	99,2%	97,2%
22443	70	30	70	40	65,7%	86,0%	98,5%	99,3%	97,3%
22444	70	30	70	45	66,1%	85,9%	98,6%	99,3%	97,5%
22445	70	30	70	50	66,5%	85,7%	98,6%	99,3%	97,7%
22446	70	30	70	55	66,9%	85,5%	98,7%	99,4%	97,8%
22447	70	30	70	60	67,3%	85,4%	98,7%	99,4%	98,0%
22448	70	30	70	65	67,7%	85,2%	98,8%	99,5%	98,1%
22449	70	30	70	70	68,0%	85,0%	98,8%	99,5%	98,2%
22450	70	30	70	75	68,4%	84,9%	98,8%	99,5%	98,3%
22451	70	30	70	80	68,8%	84,7%	98,9%	99,6%	98,5%
22452	70	30	75	20	58,2%	83,7%	98,1%	99,0%	96,4%
22453	70	30	75	25	58,7%	83,5%	98,2%	99,0%	96,6%
22454	70	30	75	30	59,1%	83,4%	98,2%	99,1%	96,8%
22455	70	30	75	35	59,5%	83,2%	98,3%	99,2%	97,1%
22456	70	30	75	40	59,9%	83,0%	98,3%	99,2%	97,2%
22457	70	30	75	45	60,4%	82,8%	98,4%	99,3%	97,4%
22458	70	30	75	50	60,8%	82,6%	98,4%	99,3%	97,6%
22459	70	30	75	55	61,2%	82,4%	98,5%	99,4%	97,7%
22460	70	30	75	60	61,6%	82,2%	98,5%	99,4%	97,9%
22461	70	30	75	65	62,0%	82,1%	98,6%	99,4%	98,0%
22462	70	30	75	70	62,5%	81,9%	98,6%	99,5%	98,2%
22463	70	30	75	75	62,9%	81,7%	98,7%	99,5%	98,3%
22464	70	30	75	80	63,3%	81,5%	98,7%	99,5%	98,4%
22465	70	30	80	20	52,1%	80,3%	97,9%	98,9%	96,3%
22466	70	30	80	25	52,6%	80,1%	97,9%	99,0%	96,5%
22467	70	30	80	30	53,0%	79,9%	98,0%	99,0%	96,7%
22468	70	30	80	35	53,5%	79,7%	98,1%	99,1%	96,9%
22469	70	30	80	40	53,9%	79,5%	98,1%	99,2%	97,1%
22470	70	30	80	45	54,3%	79,3%	98,2%	99,2%	97,3%
22471	70	30	80	50	54,8%	79,1%	98,2%	99,3%	97,5%
22472	70	30	80	55	55,2%	78,8%	98,3%	99,3%	97,6%

22473	70	30	80	60	55,6%	78,6%	98,4%	99,4%	97,8%
22474	70	30	80	65	56,1%	78,4%	98,4%	99,4%	97,9%
22475	70	30	80	70	56,5%	78,2%	98,5%	99,4%	98,1%
22476	70	30	80	75	56,9%	77,9%	98,5%	99,5%	98,2%
22477	70	30	80	80	57,4%	77,7%	98,6%	99,5%	98,3%
22478	70	35	20	20	95,8%	98,6%	99,5%	99,4%	97,5%
22479	70	35	20	25	95,9%	98,6%	99,5%	99,5%	97,6%
22480	70	35	20	30	96,0%	98,6%	99,5%	99,5%	97,8%
22481	70	35	20	35	96,0%	98,5%	99,5%	99,5%	97,9%
22482	70	35	20	40	96,1%	98,5%	99,6%	99,6%	98,1%
22483	70	35	20	45	96,2%	98,5%	99,6%	99,6%	98,2%
22484	70	35	20	50	96,2%	98,5%	99,6%	99,6%	98,3%
22485	70	35	20	55	96,3%	98,5%	99,6%	99,6%	98,4%
22486	70	35	20	60	96,4%	98,5%	99,6%	99,7%	98,5%
22487	70	35	20	65	96,4%	98,4%	99,6%	99,7%	98,6%
22488	70	35	20	70	96,5%	98,4%	99,6%	99,7%	98,7%
22489	70	35	20	75	96,5%	98,4%	99,7%	99,7%	98,8%
22490	70	35	20	80	96,6%	98,4%	99,7%	99,7%	98,9%
22491	70	35	25	20	94,7%	98,2%	99,4%	99,4%	97,4%
22492	70	35	25	25	94,8%	98,2%	99,4%	99,4%	97,5%
22493	70	35	25	30	94,9%	98,2%	99,5%	99,5%	97,7%
22494	70	35	25	35	95,0%	98,2%	99,5%	99,5%	97,8%
22495	70	35	25	40	95,1%	98,2%	99,5%	99,5%	98,0%
22496	70	35	25	45	95,2%	98,1%	99,5%	99,6%	98,1%
22497	70	35	25	50	95,2%	98,1%	99,5%	99,6%	98,2%
22498	70	35	25	55	95,3%	98,1%	99,5%	99,6%	98,3%
22499	70	35	25	60	95,4%	98,1%	99,6%	99,6%	98,5%
22500	70	35	25	65	95,5%	98,0%	99,6%	99,7%	98,6%
22501	70	35	25	70	95,6%	98,0%	99,6%	99,7%	98,6%
22502	70	35	25	75	95,6%	98,0%	99,6%	99,7%	98,7%
22503	70	35	25	80	95,7%	98,0%	99,6%	99,7%	98,8%
22504	70	35	30	20	93,4%	97,8%	99,4%	99,3%	97,2%
22505	70	35	30	25	93,5%	97,8%	99,4%	99,4%	97,4%

22506	70	35	30	30	93,6%	97,7%	99,4%	99,4%	97,6%
22507	70	35	30	35	93,7%	97,7%	99,4%	99,5%	97,7%
22508	70	35	30	40	93,8%	97,7%	99,4%	99,5%	97,9%
22509	70	35	30	45	93,9%	97,7%	99,5%	99,5%	98,0%
22510	70	35	30	50	94,0%	97,6%	99,5%	99,6%	98,2%
22511	70	35	30	55	94,1%	97,6%	99,5%	99,6%	98,3%
22512	70	35	30	60	94,2%	97,6%	99,5%	99,6%	98,4%
22513	70	35	30	65	94,3%	97,5%	99,5%	99,6%	98,5%
22514	70	35	30	70	94,4%	97,5%	99,5%	99,7%	98,6%
22515	70	35	30	75	94,5%	97,5%	99,6%	99,7%	98,7%
22516	70	35	30	80	94,6%	97,4%	99,6%	99,7%	98,8%
22517	70	35	35	20	91,7%	97,2%	99,3%	99,3%	97,1%
22518	70	35	35	25	91,8%	97,2%	99,3%	99,4%	97,3%
22519	70	35	35	30	91,9%	97,2%	99,3%	99,4%	97,5%
22520	70	35	35	35	92,1%	97,1%	99,3%	99,4%	97,7%
22521	70	35	35	40	92,2%	97,1%	99,4%	99,5%	97,8%
22522	70	35	35	45	92,3%	97,1%	99,4%	99,5%	97,9%
22523	70	35	35	50	92,4%	97,0%	99,4%	99,5%	98,1%
22524	70	35	35	55	92,6%	97,0%	99,4%	99,6%	98,2%
22525	70	35	35	60	92,7%	97,0%	99,4%	99,6%	98,3%
22526	70	35	35	65	92,8%	96,9%	99,5%	99,6%	98,4%
22527	70	35	35	70	92,9%	96,9%	99,5%	99,7%	98,5%
22528	70	35	35	75	93,0%	96,8%	99,5%	99,7%	98,6%
22529	70	35	35	80	93,1%	96,8%	99,5%	99,7%	98,7%
22530	70	35	40	20	89,6%	96,6%	99,2%	99,3%	97,0%
22531	70	35	40	25	89,7%	96,5%	99,2%	99,3%	97,2%
22532	70	35	40	30	89,9%	96,5%	99,2%	99,4%	97,4%
22533	70	35	40	35	90,0%	96,4%	99,3%	99,4%	97,6%
22534	70	35	40	40	90,2%	96,4%	99,3%	99,4%	97,7%
22535	70	35	40	45	90,4%	96,3%	99,3%	99,5%	97,9%
22536	70	35	40	50	90,5%	96,3%	99,3%	99,5%	98,0%
22537	70	35	40	55	90,7%	96,2%	99,3%	99,5%	98,1%
22538	70	35	40	60	90,8%	96,2%	99,4%	99,6%	98,3%

22539	70	35	40	65	91,0%	96,1%	99,4%	99,6%	98,4%
22540	70	35	40	70	91,1%	96,1%	99,4%	99,6%	98,5%
22541	70	35	40	75	91,2%	96,0%	99,4%	99,7%	98,6%
22542	70	35	40	80	91,4%	96,0%	99,4%	99,7%	98,7%
22543	70	35	45	20	87,0%	95,7%	99,1%	99,2%	96,9%
22544	70	35	45	25	87,2%	95,6%	99,1%	99,3%	97,1%
22545	70	35	45	30	87,4%	95,6%	99,1%	99,3%	97,3%
22546	70	35	45	35	87,6%	95,5%	99,2%	99,4%	97,5%
22547	70	35	45	40	87,8%	95,5%	99,2%	99,4%	97,6%
22548	70	35	45	45	88,0%	95,4%	99,2%	99,4%	97,8%
22549	70	35	45	50	88,2%	95,4%	99,2%	99,5%	97,9%
22550	70	35	45	55	88,4%	95,3%	99,3%	99,5%	98,1%
22551	70	35	45	60	88,5%	95,2%	99,3%	99,5%	98,2%
22552	70	35	45	65	88,7%	95,2%	99,3%	99,6%	98,3%
22553	70	35	45	70	88,9%	95,1%	99,3%	99,6%	98,4%
22554	70	35	45	75	89,1%	95,1%	99,4%	99,6%	98,5%
22555	70	35	45	80	89,2%	95,0%	99,4%	99,7%	98,6%
22556	70	35	50	20	84,0%	94,6%	98,9%	99,2%	96,8%
22557	70	35	50	25	84,2%	94,6%	99,0%	99,2%	97,0%
22558	70	35	50	30	84,4%	94,5%	99,0%	99,3%	97,2%
22559	70	35	50	35	84,7%	94,4%	99,0%	99,3%	97,4%
22560	70	35	50	40	84,9%	94,4%	99,1%	99,4%	97,5%
22561	70	35	50	45	85,1%	94,3%	99,1%	99,4%	97,7%
22562	70	35	50	50	85,3%	94,2%	99,1%	99,4%	97,8%
22563	70	35	50	55	85,6%	94,2%	99,2%	99,5%	98,0%
22564	70	35	50	60	85,8%	94,1%	99,2%	99,5%	98,1%
22565	70	35	50	65	86,0%	94,0%	99,2%	99,6%	98,2%
22566	70	35	50	70	86,2%	93,9%	99,2%	99,6%	98,3%
22567	70	35	50	75	86,4%	93,9%	99,3%	99,6%	98,5%
22568	70	35	50	80	86,6%	93,8%	99,3%	99,6%	98,6%
22569	70	35	55	20	80,4%	93,3%	98,8%	99,1%	96,6%
22570	70	35	55	25	80,6%	93,3%	98,8%	99,2%	96,9%
22571	70	35	55	30	80,9%	93,2%	98,9%	99,2%	97,1%

22572	70	35	55	35	81,2%	93,1%	98,9%	99,3%	97,2%
22573	70	35	55	40	81,5%	93,0%	99,0%	99,3%	97,4%
22574	70	35	55	45	81,7%	92,9%	99,0%	99,4%	97,6%
22575	70	35	55	50	82,0%	92,8%	99,0%	99,4%	97,7%
22576	70	35	55	55	82,2%	92,7%	99,1%	99,5%	97,9%
22577	70	35	55	60	82,5%	92,7%	99,1%	99,5%	98,0%
22578	70	35	55	65	82,7%	92,6%	99,1%	99,5%	98,2%
22579	70	35	55	70	83,0%	92,5%	99,1%	99,6%	98,3%
22580	70	35	55	75	83,2%	92,4%	99,2%	99,6%	98,4%
22581	70	35	55	80	83,5%	92,3%	99,2%	99,6%	98,5%
22582	70	35	60	20	76,2%	91,8%	98,6%	99,1%	96,5%
22583	70	35	60	25	76,5%	91,7%	98,7%	99,1%	96,7%
22584	70	35	60	30	76,8%	91,6%	98,7%	99,2%	96,9%
22585	70	35	60	35	77,1%	91,5%	98,8%	99,2%	97,1%
22586	70	35	60	40	77,4%	91,3%	98,8%	99,3%	97,3%
22587	70	35	60	45	77,7%	91,2%	98,9%	99,3%	97,5%
22588	70	35	60	50	78,0%	91,1%	98,9%	99,4%	97,7%
22589	70	35	60	55	78,3%	91,0%	98,9%	99,4%	97,8%
22590	70	35	60	60	78,6%	90,9%	99,0%	99,5%	97,9%
22591	70	35	60	65	78,9%	90,8%	99,0%	99,5%	98,1%
22592	70	35	60	70	79,2%	90,7%	99,0%	99,5%	98,2%
22593	70	35	60	75	79,5%	90,6%	99,1%	99,6%	98,3%
22594	70	35	60	80	79,8%	90,5%	99,1%	99,6%	98,4%
22595	70	35	65	20	71,4%	89,8%	98,5%	99,0%	96,4%
22596	70	35	65	25	71,8%	89,7%	98,5%	99,1%	96,6%
22597	70	35	65	30	72,1%	89,6%	98,6%	99,1%	96,8%
22598	70	35	65	35	72,5%	89,5%	98,6%	99,2%	97,0%
22599	70	35	65	40	72,8%	89,3%	98,7%	99,2%	97,2%
22600	70	35	65	45	73,2%	89,2%	98,7%	99,3%	97,4%
22601	70	35	65	50	73,5%	89,1%	98,7%	99,3%	97,6%
22602	70	35	65	55	73,9%	89,0%	98,8%	99,4%	97,7%
22603	70	35	65	60	74,2%	88,8%	98,8%	99,4%	97,9%
22604	70	35	65	65	74,5%	88,7%	98,9%	99,5%	98,0%

22605	70	35	65	70	74,9%	88,6%	98,9%	99,5%	98,1%
22606	70	35	65	75	75,2%	88,4%	98,9%	99,5%	98,3%
22607	70	35	65	80	75,5%	88,3%	99,0%	99,6%	98,4%
22608	70	35	70	20	66,1%	87,5%	98,3%	98,9%	96,2%
22609	70	35	70	25	66,5%	87,4%	98,3%	99,0%	96,5%
22610	70	35	70	30	66,9%	87,2%	98,4%	99,1%	96,7%
22611	70	35	70	35	67,3%	87,1%	98,4%	99,1%	96,9%
22612	70	35	70	40	67,7%	86,9%	98,5%	99,2%	97,1%
22613	70	35	70	45	68,1%	86,8%	98,5%	99,2%	97,3%
22614	70	35	70	50	68,4%	86,6%	98,6%	99,3%	97,5%
22615	70	35	70	55	68,8%	86,5%	98,6%	99,3%	97,6%
22616	70	35	70	60	69,2%	86,3%	98,7%	99,4%	97,8%
22617	70	35	70	65	69,6%	86,2%	98,7%	99,4%	97,9%
22618	70	35	70	70	69,9%	86,0%	98,8%	99,5%	98,1%
22619	70	35	70	75	70,3%	85,9%	98,8%	99,5%	98,2%
22620	70	35	70	80	70,7%	85,7%	98,8%	99,5%	98,3%
22621	70	35	75	20	60,4%	84,8%	98,0%	98,9%	96,1%
22622	70	35	75	25	60,8%	84,6%	98,1%	99,0%	96,3%
22623	70	35	75	30	61,2%	84,4%	98,2%	99,0%	96,5%
22624	70	35	75	35	61,6%	84,3%	98,2%	99,1%	96,8%
22625	70	35	75	40	62,1%	84,1%	98,3%	99,1%	97,0%
22626	70	35	75	45	62,5%	83,9%	98,3%	99,2%	97,2%
22627	70	35	75	50	62,9%	83,7%	98,4%	99,3%	97,4%
22628	70	35	75	55	63,3%	83,5%	98,5%	99,3%	97,5%
22629	70	35	75	60	63,7%	83,4%	98,5%	99,4%	97,7%
22630	70	35	75	65	64,1%	83,2%	98,6%	99,4%	97,8%
22631	70	35	75	70	64,5%	83,0%	98,6%	99,4%	98,0%
22632	70	35	75	75	64,9%	82,8%	98,6%	99,5%	98,1%
22633	70	35	75	80	65,3%	82,6%	98,7%	99,5%	98,2%
22634	70	35	80	20	54,4%	81,5%	97,8%	98,8%	95,9%
22635	70	35	80	25	54,8%	81,3%	97,9%	98,9%	96,2%
22636	70	35	80	30	55,2%	81,1%	97,9%	99,0%	96,4%
22637	70	35	80	35	55,7%	80,9%	98,0%	99,0%	96,6%

22638	70	35	80	40	56,1%	80,7%	98,1%	99,1%	96,8%
22639	70	35	80	45	56,5%	80,5%	98,1%	99,2%	97,1%
22640	70	35	80	50	57,0%	80,3%	98,2%	99,2%	97,2%
22641	70	35	80	55	57,4%	80,1%	98,3%	99,3%	97,4%
22642	70	35	80	60	57,8%	79,9%	98,3%	99,3%	97,6%
22643	70	35	80	65	58,3%	79,7%	98,4%	99,4%	97,7%
22644	70	35	80	70	58,7%	79,5%	98,4%	99,4%	97,9%
22645	70	35	80	75	59,1%	79,3%	98,5%	99,4%	98,0%
22646	70	35	80	80	59,5%	79,1%	98,5%	99,5%	98,2%
22647	70	40	20	20	96,2%	98,7%	99,5%	99,4%	97,2%
22648	70	40	20	25	96,2%	98,7%	99,5%	99,4%	97,4%
22649	70	40	20	30	96,3%	98,7%	99,5%	99,5%	97,6%
22650	70	40	20	35	96,4%	98,7%	99,5%	99,5%	97,7%
22651	70	40	20	40	96,4%	98,6%	99,5%	99,5%	97,9%
22652	70	40	20	45	96,5%	98,6%	99,6%	99,6%	98,0%
22653	70	40	20	50	96,6%	98,6%	99,6%	99,6%	98,1%
22654	70	40	20	55	96,6%	98,6%	99,6%	99,6%	98,3%
22655	70	40	20	60	96,7%	98,6%	99,6%	99,6%	98,4%
22656	70	40	20	65	96,7%	98,5%	99,6%	99,7%	98,5%
22657	70	40	20	70	96,8%	98,5%	99,6%	99,7%	98,6%
22658	70	40	20	75	96,8%	98,5%	99,6%	99,7%	98,7%
22659	70	40	20	80	96,9%	98,5%	99,7%	99,7%	98,8%
22660	70	40	25	20	95,2%	98,4%	99,4%	99,3%	97,1%
22661	70	40	25	25	95,2%	98,4%	99,4%	99,4%	97,3%
22662	70	40	25	30	95,3%	98,3%	99,4%	99,4%	97,5%
22663	70	40	25	35	95,4%	98,3%	99,5%	99,5%	97,6%
22664	70	40	25	40	95,5%	98,3%	99,5%	99,5%	97,8%
22665	70	40	25	45	95,6%	98,3%	99,5%	99,5%	97,9%
22666	70	40	25	50	95,6%	98,2%	99,5%	99,6%	98,1%
22667	70	40	25	55	95,7%	98,2%	99,5%	99,6%	98,2%
22668	70	40	25	60	95,8%	98,2%	99,5%	99,6%	98,3%
22669	70	40	25	65	95,8%	98,2%	99,6%	99,6%	98,4%
22670	70	40	25	70	95,9%	98,2%	99,6%	99,7%	98,5%

22671	70	40	25	75	96,0%	98,1%	99,6%	99,7%	98,6%
22672	70	40	25	80	96,0%	98,1%	99,6%	99,7%	98,7%
22673	70	40	30	20	93,9%	98,0%	99,3%	99,3%	97,0%
22674	70	40	30	25	94,0%	97,9%	99,4%	99,3%	97,2%
22675	70	40	30	30	94,1%	97,9%	99,4%	99,4%	97,4%
22676	70	40	30	35	94,2%	97,9%	99,4%	99,4%	97,5%
22677	70	40	30	40	94,3%	97,9%	99,4%	99,5%	97,7%
22678	70	40	30	45	94,4%	97,8%	99,4%	99,5%	97,8%
22679	70	40	30	50	94,5%	97,8%	99,5%	99,5%	98,0%
22680	70	40	30	55	94,6%	97,8%	99,5%	99,6%	98,1%
22681	70	40	30	60	94,7%	97,7%	99,5%	99,6%	98,2%
22682	70	40	30	65	94,7%	97,7%	99,5%	99,6%	98,3%
22683	70	40	30	70	94,8%	97,7%	99,5%	99,6%	98,5%
22684	70	40	30	75	94,9%	97,7%	99,5%	99,7%	98,6%
22685	70	40	30	80	95,0%	97,6%	99,6%	99,7%	98,6%
22686	70	40	35	20	92,3%	97,4%	99,2%	99,3%	96,9%
22687	70	40	35	25	92,4%	97,4%	99,3%	99,3%	97,1%
22688	70	40	35	30	92,6%	97,4%	99,3%	99,4%	97,2%
22689	70	40	35	35	92,7%	97,3%	99,3%	99,4%	97,4%
22690	70	40	35	40	92,8%	97,3%	99,3%	99,4%	97,6%
22691	70	40	35	45	92,9%	97,3%	99,4%	99,5%	97,7%
22692	70	40	35	50	93,0%	97,2%	99,4%	99,5%	97,9%
22693	70	40	35	55	93,1%	97,2%	99,4%	99,5%	98,0%
22694	70	40	35	60	93,3%	97,2%	99,4%	99,6%	98,2%
22695	70	40	35	65	93,4%	97,1%	99,4%	99,6%	98,3%
22696	70	40	35	70	93,5%	97,1%	99,5%	99,6%	98,4%
22697	70	40	35	75	93,6%	97,1%	99,5%	99,6%	98,5%
22698	70	40	35	80	93,7%	97,0%	99,5%	99,7%	98,6%
22699	70	40	40	20	90,4%	96,8%	99,1%	99,2%	96,7%
22700	70	40	40	25	90,5%	96,8%	99,2%	99,3%	96,9%
22701	70	40	40	30	90,7%	96,7%	99,2%	99,3%	97,1%
22702	70	40	40	35	90,8%	96,7%	99,2%	99,4%	97,3%
22703	70	40	40	40	91,0%	96,6%	99,3%	99,4%	97,5%

22704	70	40	40	45	91,1%	96,6%	99,3%	99,4%	97,7%
22705	70	40	40	50	91,2%	96,6%	99,3%	99,5%	97,8%
22706	70	40	40	55	91,4%	96,5%	99,3%	99,5%	97,9%
22707	70	40	40	60	91,5%	96,5%	99,3%	99,5%	98,1%
22708	70	40	40	65	91,7%	96,4%	99,4%	99,6%	98,2%
22709	70	40	40	70	91,8%	96,4%	99,4%	99,6%	98,3%
22710	70	40	40	75	91,9%	96,3%	99,4%	99,6%	98,4%
22711	70	40	40	80	92,1%	96,3%	99,4%	99,7%	98,5%
22712	70	40	45	20	88,0%	96,0%	99,0%	99,2%	96,6%
22713	70	40	45	25	88,2%	96,0%	99,1%	99,2%	96,8%
22714	70	40	45	30	88,4%	95,9%	99,1%	99,3%	97,0%
22715	70	40	45	35	88,5%	95,9%	99,1%	99,3%	97,2%
22716	70	40	45	40	88,7%	95,8%	99,2%	99,4%	97,4%
22717	70	40	45	45	88,9%	95,8%	99,2%	99,4%	97,6%
22718	70	40	45	50	89,1%	95,7%	99,2%	99,4%	97,7%
22719	70	40	45	55	89,2%	95,6%	99,2%	99,5%	97,9%
22720	70	40	45	60	89,4%	95,6%	99,3%	99,5%	98,0%
22721	70	40	45	65	89,6%	95,5%	99,3%	99,5%	98,1%
22722	70	40	45	70	89,7%	95,5%	99,3%	99,6%	98,3%
22723	70	40	45	75	89,9%	95,4%	99,3%	99,6%	98,4%
22724	70	40	45	80	90,1%	95,4%	99,4%	99,6%	98,5%
22725	70	40	50	20	85,1%	95,0%	98,9%	99,1%	96,5%
22726	70	40	50	25	85,4%	95,0%	98,9%	99,2%	96,7%
22727	70	40	50	30	85,6%	94,9%	99,0%	99,2%	96,9%
22728	70	40	50	35	85,8%	94,8%	99,0%	99,3%	97,1%
22729	70	40	50	40	86,0%	94,8%	99,0%	99,3%	97,3%
22730	70	40	50	45	86,2%	94,7%	99,1%	99,4%	97,5%
22731	70	40	50	50	86,4%	94,6%	99,1%	99,4%	97,6%
22732	70	40	50	55	86,6%	94,6%	99,1%	99,4%	97,8%
22733	70	40	50	60	86,8%	94,5%	99,2%	99,5%	97,9%
22734	70	40	50	65	87,0%	94,4%	99,2%	99,5%	98,1%
22735	70	40	50	70	87,2%	94,4%	99,2%	99,5%	98,2%
22736	70	40	50	75	87,4%	94,3%	99,2%	99,6%	98,3%

22737	70	40	50	80	87,6%	94,2%	99,3%	99,6%	98,4%
22738	70	40	55	20	81,7%	93,8%	98,8%	99,1%	96,3%
22739	70	40	55	25	82,0%	93,7%	98,8%	99,1%	96,5%
22740	70	40	55	30	82,2%	93,7%	98,8%	99,2%	96,8%
22741	70	40	55	35	82,5%	93,6%	98,9%	99,2%	97,0%
22742	70	40	55	40	82,8%	93,5%	98,9%	99,3%	97,2%
22743	70	40	55	45	83,0%	93,4%	99,0%	99,3%	97,4%
22744	70	40	55	50	83,3%	93,3%	99,0%	99,4%	97,5%
22745	70	40	55	55	83,5%	93,3%	99,0%	99,4%	97,7%
22746	70	40	55	60	83,7%	93,2%	99,1%	99,5%	97,8%
22747	70	40	55	65	84,0%	93,1%	99,1%	99,5%	98,0%
22748	70	40	55	70	84,2%	93,0%	99,1%	99,5%	98,1%
22749	70	40	55	75	84,4%	92,9%	99,1%	99,6%	98,2%
22750	70	40	55	80	84,7%	92,8%	99,2%	99,6%	98,3%
22751	70	40	60	20	77,7%	92,3%	98,6%	99,0%	96,2%
22752	70	40	60	25	78,0%	92,2%	98,6%	99,1%	96,4%
22753	70	40	60	30	78,3%	92,1%	98,7%	99,1%	96,6%
22754	70	40	60	35	78,6%	92,0%	98,7%	99,2%	96,9%
22755	70	40	60	40	78,9%	91,9%	98,8%	99,2%	97,1%
22756	70	40	60	45	79,2%	91,9%	98,8%	99,3%	97,2%
22757	70	40	60	50	79,5%	91,8%	98,9%	99,3%	97,4%
22758	70	40	60	55	79,8%	91,7%	98,9%	99,4%	97,6%
22759	70	40	60	60	80,1%	91,6%	98,9%	99,4%	97,7%
22760	70	40	60	65	80,4%	91,5%	99,0%	99,5%	97,9%
22761	70	40	60	70	80,6%	91,3%	99,0%	99,5%	98,0%
22762	70	40	60	75	80,9%	91,2%	99,0%	99,5%	98,2%
22763	70	40	60	80	81,2%	91,1%	99,1%	99,6%	98,3%
22764	70	40	65	20	73,2%	90,5%	98,4%	98,9%	96,0%
22765	70	40	65	25	73,5%	90,4%	98,5%	99,0%	96,3%
22766	70	40	65	30	73,9%	90,3%	98,5%	99,1%	96,5%
22767	70	40	65	35	74,2%	90,2%	98,6%	99,1%	96,7%
22768	70	40	65	40	74,5%	90,1%	98,6%	99,2%	96,9%
22769	70	40	65	45	74,9%	89,9%	98,7%	99,2%	97,1%

22770	70	40	65	50	75,2%	89,8%	98,7%	99,3%	97,3%
22771	70	40	65	55	75,5%	89,7%	98,7%	99,3%	97,5%
22772	70	40	65	60	75,9%	89,6%	98,8%	99,4%	97,7%
22773	70	40	65	65	76,2%	89,5%	98,8%	99,4%	97,8%
22774	70	40	65	70	76,5%	89,3%	98,9%	99,5%	97,9%
22775	70	40	65	75	76,8%	89,2%	98,9%	99,5%	98,1%
22776	70	40	65	80	77,1%	89,1%	98,9%	99,5%	98,2%
22777	70	40	70	20	68,1%	88,3%	98,2%	98,9%	95,8%
22778	70	40	70	25	68,5%	88,2%	98,3%	98,9%	96,1%
22779	70	40	70	30	68,8%	88,1%	98,3%	99,0%	96,4%
22780	70	40	70	35	69,2%	87,9%	98,4%	99,1%	96,6%
22781	70	40	70	40	69,6%	87,8%	98,4%	99,1%	96,8%
22782	70	40	70	45	70,0%	87,7%	98,5%	99,2%	97,0%
22783	70	40	70	50	70,3%	87,5%	98,5%	99,2%	97,2%
22784	70	40	70	55	70,7%	87,4%	98,6%	99,3%	97,4%
22785	70	40	70	60	71,1%	87,2%	98,6%	99,3%	97,6%
22786	70	40	70	65	71,4%	87,1%	98,7%	99,4%	97,7%
22787	70	40	70	70	71,8%	86,9%	98,7%	99,4%	97,9%
22788	70	40	70	75	72,1%	86,8%	98,8%	99,5%	98,0%
22789	70	40	70	80	72,5%	86,6%	98,8%	99,5%	98,1%
22790	70	40	75	20	62,5%	85,8%	98,0%	98,8%	95,7%
22791	70	40	75	25	62,9%	85,6%	98,0%	98,9%	96,0%
22792	70	40	75	30	63,3%	85,4%	98,1%	98,9%	96,2%
22793	70	40	75	35	63,7%	85,3%	98,2%	99,0%	96,5%
22794	70	40	75	40	64,1%	85,1%	98,2%	99,1%	96,7%
22795	70	40	75	45	64,5%	84,9%	98,3%	99,1%	96,9%
22796	70	40	75	50	64,9%	84,8%	98,3%	99,2%	97,1%
22797	70	40	75	55	65,3%	84,6%	98,4%	99,3%	97,3%
22798	70	40	75	60	65,7%	84,4%	98,5%	99,3%	97,5%
22799	70	40	75	65	66,1%	84,3%	98,5%	99,3%	97,6%
22800	70	40	75	70	66,5%	84,1%	98,6%	99,4%	97,8%
22801	70	40	75	75	66,9%	83,9%	98,6%	99,4%	97,9%
22802	70	40	75	80	67,3%	83,7%	98,6%	99,5%	98,1%

22803	70	40	80	20	56,5%	82,7%	97,7%	98,7%	95,5%
22804	70	40	80	25	57,0%	82,5%	97,8%	98,8%	95,8%
22805	70	40	80	30	57,4%	82,3%	97,9%	98,9%	96,1%
22806	70	40	80	35	57,8%	82,1%	97,9%	99,0%	96,3%
22807	70	40	80	40	58,3%	81,9%	98,0%	99,0%	96,5%
22808	70	40	80	45	58,7%	81,7%	98,1%	99,1%	96,8%
22809	70	40	80	50	59,1%	81,5%	98,1%	99,1%	97,0%
22810	70	40	80	55	59,5%	81,3%	98,2%	99,2%	97,2%
22811	70	40	80	60	60,0%	81,1%	98,3%	99,3%	97,4%
22812	70	40	80	65	60,4%	80,9%	98,3%	99,3%	97,5%
22813	70	40	80	70	60,8%	80,7%	98,4%	99,4%	97,7%
22814	70	40	80	75	61,2%	80,5%	98,4%	99,4%	97,8%
22815	70	40	80	80	61,7%	80,3%	98,5%	99,4%	98,0%
22816	70	45	20	20	96,5%	98,8%	99,5%	99,3%	96,9%
22817	70	45	20	25	96,6%	98,8%	99,5%	99,4%	97,1%
22818	70	45	20	30	96,6%	98,8%	99,5%	99,4%	97,3%
22819	70	45	20	35	96,7%	98,8%	99,5%	99,5%	97,5%
22820	70	45	20	40	96,7%	98,7%	99,5%	99,5%	97,7%
22821	70	45	20	45	96,8%	98,7%	99,5%	99,5%	97,8%
22822	70	45	20	50	96,8%	98,7%	99,6%	99,6%	98,0%
22823	70	45	20	55	96,9%	98,7%	99,6%	99,6%	98,1%
22824	70	45	20	60	96,9%	98,7%	99,6%	99,6%	98,2%
22825	70	45	20	65	97,0%	98,7%	99,6%	99,6%	98,3%
22826	70	45	20	70	97,0%	98,6%	99,6%	99,7%	98,4%
22827	70	45	20	75	97,1%	98,6%	99,6%	99,7%	98,5%
22828	70	45	20	80	97,1%	98,6%	99,6%	99,7%	98,6%
22829	70	45	25	20	95,6%	98,5%	99,4%	99,3%	96,8%
22830	70	45	25	25	95,6%	98,5%	99,4%	99,3%	97,0%
22831	70	45	25	30	95,7%	98,5%	99,4%	99,4%	97,2%
22832	70	45	25	35	95,8%	98,4%	99,4%	99,4%	97,4%
22833	70	45	25	40	95,8%	98,4%	99,5%	99,5%	97,6%
22834	70	45	25	45	95,9%	98,4%	99,5%	99,5%	97,7%
22835	70	45	25	50	96,0%	98,4%	99,5%	99,5%	97,9%

22836	70	45	25	55	96,1%	98,4%	99,5%	99,6%	98,0%
22837	70	45	25	60	96,1%	98,3%	99,5%	99,6%	98,1%
22838	70	45	25	65	96,2%	98,3%	99,6%	99,6%	98,3%
22839	70	45	25	70	96,2%	98,3%	99,6%	99,6%	98,4%
22840	70	45	25	75	96,3%	98,3%	99,6%	99,7%	98,5%
22841	70	45	25	80	96,4%	98,2%	99,6%	99,7%	98,6%
22842	70	45	30	20	94,4%	98,1%	99,3%	99,2%	96,7%
22843	70	45	30	25	94,5%	98,1%	99,3%	99,3%	96,9%
22844	70	45	30	30	94,6%	98,1%	99,4%	99,3%	97,1%
22845	70	45	30	35	94,7%	98,0%	99,4%	99,4%	97,3%
22846	70	45	30	40	94,7%	98,0%	99,4%	99,4%	97,5%
22847	70	45	30	45	94,8%	98,0%	99,4%	99,5%	97,6%
22848	70	45	30	50	94,9%	98,0%	99,4%	99,5%	97,8%
22849	70	45	30	55	95,0%	97,9%	99,5%	99,5%	97,9%
22850	70	45	30	60	95,1%	97,9%	99,5%	99,6%	98,1%
22851	70	45	30	65	95,2%	97,9%	99,5%	99,6%	98,2%
22852	70	45	30	70	95,2%	97,9%	99,5%	99,6%	98,3%
22853	70	45	30	75	95,3%	97,8%	99,5%	99,6%	98,4%
22854	70	45	30	80	95,4%	97,8%	99,5%	99,7%	98,5%
22855	70	45	35	20	92,9%	97,6%	99,2%	99,2%	96,6%
22856	70	45	35	25	93,0%	97,6%	99,2%	99,3%	96,8%
22857	70	45	35	30	93,1%	97,6%	99,3%	99,3%	97,0%
22858	70	45	35	35	93,3%	97,5%	99,3%	99,3%	97,2%
22859	70	45	35	40	93,4%	97,5%	99,3%	99,4%	97,4%
22860	70	45	35	45	93,5%	97,5%	99,3%	99,4%	97,5%
22861	70	45	35	50	93,6%	97,4%	99,4%	99,5%	97,7%
22862	70	45	35	55	93,7%	97,4%	99,4%	99,5%	97,8%
22863	70	45	35	60	93,8%	97,4%	99,4%	99,5%	98,0%
22864	70	45	35	65	93,9%	97,3%	99,4%	99,6%	98,1%
22865	70	45	35	70	94,0%	97,3%	99,4%	99,6%	98,2%
22866	70	45	35	75	94,1%	97,3%	99,5%	99,6%	98,3%
22867	70	45	35	80	94,2%	97,2%	99,5%	99,6%	98,5%
22868	70	45	40	20	91,1%	97,0%	99,1%	99,1%	96,4%

22869	70	45	40	25	91,3%	97,0%	99,1%	99,2%	96,6%
22870	70	45	40	30	91,4%	97,0%	99,2%	99,3%	96,9%
22871	70	45	40	35	91,5%	96,9%	99,2%	99,3%	97,1%
22872	70	45	40	40	91,7%	96,9%	99,2%	99,4%	97,2%
22873	70	45	40	45	91,8%	96,8%	99,3%	99,4%	97,4%
22874	70	45	40	50	91,9%	96,8%	99,3%	99,4%	97,6%
22875	70	45	40	55	92,1%	96,8%	99,3%	99,5%	97,7%
22876	70	45	40	60	92,2%	96,7%	99,3%	99,5%	97,9%
22877	70	45	40	65	92,3%	96,7%	99,3%	99,5%	98,0%
22878	70	45	40	70	92,4%	96,6%	99,4%	99,6%	98,2%
22879	70	45	40	75	92,6%	96,6%	99,4%	99,6%	98,3%
22880	70	45	40	80	92,7%	96,6%	99,4%	99,6%	98,4%
22881	70	45	45	20	88,9%	96,3%	99,0%	99,1%	96,3%
22882	70	45	45	25	89,1%	96,3%	99,0%	99,2%	96,5%
22883	70	45	45	30	89,2%	96,2%	99,1%	99,2%	96,7%
22884	70	45	45	35	89,4%	96,2%	99,1%	99,3%	96,9%
22885	70	45	45	40	89,6%	96,1%	99,1%	99,3%	97,1%
22886	70	45	45	45	89,7%	96,1%	99,2%	99,4%	97,3%
22887	70	45	45	50	89,9%	96,0%	99,2%	99,4%	97,5%
22888	70	45	45	55	90,1%	96,0%	99,2%	99,4%	97,7%
22889	70	45	45	60	90,2%	95,9%	99,2%	99,5%	97,8%
22890	70	45	45	65	90,4%	95,9%	99,3%	99,5%	97,9%
22891	70	45	45	70	90,5%	95,8%	99,3%	99,5%	98,1%
22892	70	45	45	75	90,7%	95,8%	99,3%	99,6%	98,2%
22893	70	45	45	80	90,8%	95,7%	99,3%	99,6%	98,3%
22894	70	45	50	20	86,2%	95,4%	98,9%	99,0%	96,1%
22895	70	45	50	25	86,4%	95,3%	98,9%	99,1%	96,4%
22896	70	45	50	30	86,6%	95,3%	98,9%	99,2%	96,6%
22897	70	45	50	35	86,8%	95,2%	99,0%	99,2%	96,8%
22898	70	45	50	40	87,0%	95,1%	99,0%	99,3%	97,0%
22899	70	45	50	45	87,2%	95,1%	99,0%	99,3%	97,2%
22900	70	45	50	50	87,4%	95,0%	99,1%	99,4%	97,4%
22901	70	45	50	55	87,6%	95,0%	99,1%	99,4%	97,6%

22902	70	45	50	60	87,8%	94,9%	99,1%	99,4%	97,7%
22903	70	45	50	65	88,0%	94,8%	99,2%	99,5%	97,9%
22904	70	45	50	70	88,2%	94,8%	99,2%	99,5%	98,0%
22905	70	45	50	75	88,4%	94,7%	99,2%	99,5%	98,1%
22906	70	45	50	80	88,5%	94,6%	99,2%	99,6%	98,3%
22907	70	45	55	20	83,0%	94,3%	98,7%	99,0%	96,0%
22908	70	45	55	25	83,3%	94,2%	98,8%	99,0%	96,2%
22909	70	45	55	30	83,5%	94,1%	98,8%	99,1%	96,5%
22910	70	45	55	35	83,7%	94,0%	98,8%	99,2%	96,7%
22911	70	45	55	40	84,0%	94,0%	98,9%	99,2%	96,9%
22912	70	45	55	45	84,2%	93,9%	98,9%	99,3%	97,1%
22913	70	45	55	50	84,5%	93,8%	99,0%	99,3%	97,3%
22914	70	45	55	55	84,7%	93,7%	99,0%	99,4%	97,5%
22915	70	45	55	60	84,9%	93,7%	99,0%	99,4%	97,6%
22916	70	45	55	65	85,1%	93,6%	99,1%	99,4%	97,8%
22917	70	45	55	70	85,4%	93,5%	99,1%	99,5%	97,9%
22918	70	45	55	75	85,6%	93,4%	99,1%	99,5%	98,1%
22919	70	45	55	80	85,8%	93,3%	99,1%	99,6%	98,2%
22920	70	45	60	20	79,2%	92,9%	98,6%	98,9%	95,8%
22921	70	45	60	25	79,5%	92,8%	98,6%	99,0%	96,1%
22922	70	45	60	30	79,8%	92,7%	98,7%	99,1%	96,3%
22923	70	45	60	35	80,1%	92,6%	98,7%	99,1%	96,5%
22924	70	45	60	40	80,4%	92,5%	98,7%	99,2%	96,8%
22925	70	45	60	45	80,7%	92,4%	98,8%	99,2%	97,0%
22926	70	45	60	50	80,9%	92,3%	98,8%	99,3%	97,2%
22927	70	45	60	55	81,2%	92,2%	98,9%	99,3%	97,4%
22928	70	45	60	60	81,5%	92,1%	98,9%	99,4%	97,5%
22929	70	45	60	65	81,7%	92,0%	98,9%	99,4%	97,7%
22930	70	45	60	70	82,0%	92,0%	99,0%	99,5%	97,8%
22931	70	45	60	75	82,3%	91,9%	99,0%	99,5%	98,0%
22932	70	45	60	80	82,5%	91,8%	99,0%	99,5%	98,1%
22933	70	45	65	20	74,9%	91,2%	98,4%	98,8%	95,6%
22934	70	45	65	25	75,2%	91,1%	98,4%	98,9%	95,9%

22935	70	45	65	30	75,5%	91,0%	98,5%	99,0%	96,2%
22936	70	45	65	35	75,9%	90,9%	98,5%	99,1%	96,4%
22937	70	45	65	40	76,2%	90,7%	98,6%	99,1%	96,6%
22938	70	45	65	45	76,5%	90,6%	98,6%	99,2%	96,9%
22939	70	45	65	50	76,8%	90,5%	98,7%	99,2%	97,1%
22940	70	45	65	55	77,1%	90,4%	98,7%	99,3%	97,2%
22941	70	45	65	60	77,4%	90,3%	98,8%	99,3%	97,4%
22942	70	45	65	65	77,8%	90,2%	98,8%	99,4%	97,6%
22943	70	45	65	70	78,1%	90,1%	98,8%	99,4%	97,7%
22944	70	45	65	75	78,4%	90,0%	98,9%	99,5%	97,9%
22945	70	45	65	80	78,7%	89,8%	98,9%	99,5%	98,0%
22946	70	45	70	20	70,0%	89,1%	98,2%	98,8%	95,4%
22947	70	45	70	25	70,3%	89,0%	98,2%	98,9%	95,7%
22948	70	45	70	30	70,7%	88,9%	98,3%	98,9%	96,0%
22949	70	45	70	35	71,1%	88,8%	98,3%	99,0%	96,3%
22950	70	45	70	40	71,4%	88,6%	98,4%	99,1%	96,5%
22951	70	45	70	45	71,8%	88,5%	98,4%	99,1%	96,7%
22952	70	45	70	50	72,1%	88,4%	98,5%	99,2%	96,9%
22953	70	45	70	55	72,5%	88,2%	98,5%	99,2%	97,1%
22954	70	45	70	60	72,8%	88,1%	98,6%	99,3%	97,3%
22955	70	45	70	65	73,2%	87,9%	98,6%	99,3%	97,5%
22956	70	45	70	70	73,5%	87,8%	98,7%	99,4%	97,7%
22957	70	45	70	75	73,9%	87,7%	98,7%	99,4%	97,8%
22958	70	45	70	80	74,2%	87,5%	98,8%	99,5%	97,9%
22959	70	45	75	20	64,5%	86,7%	97,9%	98,7%	95,3%
22960	70	45	75	25	64,9%	86,5%	98,0%	98,8%	95,6%
22961	70	45	75	30	65,3%	86,4%	98,1%	98,9%	95,8%
22962	70	45	75	35	65,7%	86,2%	98,1%	98,9%	96,1%
22963	70	45	75	40	66,1%	86,1%	98,2%	99,0%	96,4%
22964	70	45	75	45	66,5%	85,9%	98,2%	99,1%	96,6%
22965	70	45	75	50	66,9%	85,8%	98,3%	99,1%	96,8%
22966	70	45	75	55	67,3%	85,6%	98,4%	99,2%	97,0%
22967	70	45	75	60	67,7%	85,4%	98,4%	99,2%	97,2%

22968	70	45	75	65	68,1%	85,3%	98,5%	99,3%	97,4%
22969	70	45	75	70	68,5%	85,1%	98,5%	99,3%	97,6%
22970	70	45	75	75	68,8%	84,9%	98,6%	99,4%	97,7%
22971	70	45	75	80	69,2%	84,8%	98,6%	99,4%	97,9%
22972	70	45	80	20	58,7%	83,8%	97,7%	98,6%	95,1%
22973	70	45	80	25	59,1%	83,6%	97,7%	98,7%	95,4%
22974	70	45	80	30	59,6%	83,4%	97,8%	98,8%	95,7%
22975	70	45	80	35	60,0%	83,3%	97,9%	98,9%	96,0%
22976	70	45	80	40	60,4%	83,1%	97,9%	98,9%	96,2%
22977	70	45	80	45	60,8%	82,9%	98,0%	99,0%	96,5%
22978	70	45	80	50	61,2%	82,7%	98,1%	99,1%	96,7%
22979	70	45	80	55	61,7%	82,5%	98,1%	99,1%	96,9%
22980	70	45	80	60	62,1%	82,3%	98,2%	99,2%	97,1%
22981	70	45	80	65	62,5%	82,1%	98,3%	99,3%	97,3%
22982	70	45	80	70	62,9%	81,9%	98,3%	99,3%	97,5%
22983	70	45	80	75	63,3%	81,7%	98,4%	99,3%	97,6%
22984	70	45	80	80	63,7%	81,5%	98,4%	99,4%	97,8%
22985	70	50	20	20	96,8%	98,9%	99,4%	99,3%	96,6%
22986	70	50	20	25	96,8%	98,9%	99,5%	99,3%	96,9%
22987	70	50	20	30	96,9%	98,9%	99,5%	99,4%	97,1%
22988	70	50	20	35	96,9%	98,8%	99,5%	99,4%	97,2%
22989	70	50	20	40	97,0%	98,8%	99,5%	99,5%	97,4%
22990	70	50	20	45	97,0%	98,8%	99,5%	99,5%	97,6%
22991	70	50	20	50	97,1%	98,8%	99,5%	99,5%	97,7%
22992	70	50	20	55	97,1%	98,8%	99,6%	99,6%	97,9%
22993	70	50	20	60	97,2%	98,8%	99,6%	99,6%	98,0%
22994	70	50	20	65	97,2%	98,8%	99,6%	99,6%	98,2%
22995	70	50	20	70	97,3%	98,7%	99,6%	99,6%	98,3%
22996	70	50	20	75	97,3%	98,7%	99,6%	99,7%	98,4%
22997	70	50	20	80	97,4%	98,7%	99,6%	99,7%	98,5%
22998	70	50	25	20	95,9%	98,6%	99,4%	99,2%	96,5%
22999	70	50	25	25	96,0%	98,6%	99,4%	99,3%	96,7%
23000	70	50	25	30	96,1%	98,6%	99,4%	99,3%	96,9%

23001	70	50	25	35	96,1%	98,6%	99,4%	99,4%	97,1%
23002	70	50	25	40	96,2%	98,5%	99,5%	99,4%	97,3%
23003	70	50	25	45	96,2%	98,5%	99,5%	99,5%	97,5%
23004	70	50	25	50	96,3%	98,5%	99,5%	99,5%	97,7%
23005	70	50	25	55	96,4%	98,5%	99,5%	99,5%	97,8%
23006	70	50	25	60	96,4%	98,5%	99,5%	99,6%	98,0%
23007	70	50	25	65	96,5%	98,4%	99,5%	99,6%	98,1%
23008	70	50	25	70	96,6%	98,4%	99,6%	99,6%	98,2%
23009	70	50	25	75	96,6%	98,4%	99,6%	99,6%	98,3%
23010	70	50	25	80	96,7%	98,4%	99,6%	99,7%	98,4%
23011	70	50	30	20	94,8%	98,3%	99,3%	99,2%	96,4%
23012	70	50	30	25	94,9%	98,2%	99,3%	99,2%	96,6%
23013	70	50	30	30	95,0%	98,2%	99,3%	99,3%	96,8%
23014	70	50	30	35	95,1%	98,2%	99,4%	99,3%	97,0%
23015	70	50	30	40	95,2%	98,2%	99,4%	99,4%	97,2%
23016	70	50	30	45	95,2%	98,1%	99,4%	99,4%	97,4%
23017	70	50	30	50	95,3%	98,1%	99,4%	99,5%	97,6%
23018	70	50	30	55	95,4%	98,1%	99,4%	99,5%	97,7%
23019	70	50	30	60	95,5%	98,1%	99,5%	99,5%	97,9%
23020	70	50	30	65	95,6%	98,0%	99,5%	99,6%	98,0%
23021	70	50	30	70	95,6%	98,0%	99,5%	99,6%	98,1%
23022	70	50	30	75	95,7%	98,0%	99,5%	99,6%	98,3%
23023	70	50	30	80	95,8%	98,0%	99,5%	99,6%	98,4%
23024	70	50	35	20	93,5%	97,8%	99,2%	99,1%	96,2%
23025	70	50	35	25	93,6%	97,8%	99,2%	99,2%	96,5%
23026	70	50	35	30	93,7%	97,8%	99,2%	99,2%	96,7%
23027	70	50	35	35	93,8%	97,7%	99,3%	99,3%	96,9%
23028	70	50	35	40	93,9%	97,7%	99,3%	99,3%	97,1%
23029	70	50	35	45	94,0%	97,7%	99,3%	99,4%	97,3%
23030	70	50	35	50	94,1%	97,6%	99,3%	99,4%	97,5%
23031	70	50	35	55	94,2%	97,6%	99,4%	99,5%	97,6%
23032	70	50	35	60	94,3%	97,6%	99,4%	99,5%	97,8%
23033	70	50	35	65	94,4%	97,5%	99,4%	99,5%	97,9%

23034	70	50	35	70	94,5%	97,5%	99,4%	99,6%	98,1%
23035	70	50	35	75	94,6%	97,5%	99,4%	99,6%	98,2%
23036	70	50	35	80	94,7%	97,4%	99,5%	99,6%	98,3%
23037	70	50	40	20	91,8%	97,3%	99,1%	99,1%	96,1%
23038	70	50	40	25	91,9%	97,2%	99,1%	99,1%	96,3%
23039	70	50	40	30	92,1%	97,2%	99,1%	99,2%	96,6%
23040	70	50	40	35	92,2%	97,2%	99,2%	99,3%	96,8%
23041	70	50	40	40	92,3%	97,1%	99,2%	99,3%	97,0%
23042	70	50	40	45	92,4%	97,1%	99,2%	99,3%	97,2%
23043	70	50	40	50	92,6%	97,0%	99,3%	99,4%	97,4%
23044	70	50	40	55	92,7%	97,0%	99,3%	99,4%	97,5%
23045	70	50	40	60	92,8%	97,0%	99,3%	99,5%	97,7%
23046	70	50	40	65	92,9%	96,9%	99,3%	99,5%	97,8%
23047	70	50	40	70	93,0%	96,9%	99,3%	99,5%	98,0%
23048	70	50	40	75	93,1%	96,8%	99,4%	99,6%	98,1%
23049	70	50	40	80	93,3%	96,8%	99,4%	99,6%	98,2%
23050	70	50	45	20	89,7%	96,6%	99,0%	99,0%	95,9%
23051	70	50	45	25	89,9%	96,5%	99,0%	99,1%	96,2%
23052	70	50	45	30	90,1%	96,5%	99,0%	99,2%	96,4%
23053	70	50	45	35	90,2%	96,4%	99,1%	99,2%	96,6%
23054	70	50	45	40	90,4%	96,4%	99,1%	99,3%	96,9%
23055	70	50	45	45	90,5%	96,3%	99,1%	99,3%	97,1%
23056	70	50	45	50	90,7%	96,3%	99,2%	99,4%	97,2%
23057	70	50	45	55	90,8%	96,3%	99,2%	99,4%	97,4%
23058	70	50	45	60	91,0%	96,2%	99,2%	99,4%	97,6%
23059	70	50	45	65	91,1%	96,2%	99,2%	99,5%	97,7%
23060	70	50	45	70	91,3%	96,1%	99,3%	99,5%	97,9%
23061	70	50	45	75	91,4%	96,1%	99,3%	99,5%	98,0%
23062	70	50	45	80	91,5%	96,0%	99,3%	99,6%	98,2%
23063	70	50	50	20	87,2%	95,7%	98,8%	99,0%	95,7%
23064	70	50	50	25	87,4%	95,7%	98,9%	99,0%	96,0%
23065	70	50	50	30	87,6%	95,6%	98,9%	99,1%	96,3%
23066	70	50	50	35	87,8%	95,6%	98,9%	99,2%	96,5%

23067	70	50	50	40	88,0%	95,5%	99,0%	99,2%	96,7%
23068	70	50	50	45	88,2%	95,4%	99,0%	99,3%	96,9%
23069	70	50	50	50	88,4%	95,4%	99,0%	99,3%	97,1%
23070	70	50	50	55	88,5%	95,3%	99,1%	99,4%	97,3%
23071	70	50	50	60	88,7%	95,3%	99,1%	99,4%	97,5%
23072	70	50	50	65	88,9%	95,2%	99,1%	99,4%	97,7%
23073	70	50	50	70	89,1%	95,1%	99,2%	99,5%	97,8%
23074	70	50	50	75	89,2%	95,1%	99,2%	99,5%	97,9%
23075	70	50	50	80	89,4%	95,0%	99,2%	99,5%	98,1%
23076	70	50	55	20	84,2%	94,7%	98,7%	98,9%	95,6%
23077	70	50	55	25	84,5%	94,6%	98,7%	99,0%	95,9%
23078	70	50	55	30	84,7%	94,5%	98,8%	99,0%	96,1%
23079	70	50	55	35	84,9%	94,5%	98,8%	99,1%	96,4%
23080	70	50	55	40	85,1%	94,4%	98,8%	99,2%	96,6%
23081	70	50	55	45	85,4%	94,3%	98,9%	99,2%	96,8%
23082	70	50	55	50	85,6%	94,3%	98,9%	99,3%	97,0%
23083	70	50	55	55	85,8%	94,2%	99,0%	99,3%	97,2%
23084	70	50	55	60	86,0%	94,1%	99,0%	99,4%	97,4%
23085	70	50	55	65	86,2%	94,0%	99,0%	99,4%	97,6%
23086	70	50	55	70	86,4%	94,0%	99,1%	99,4%	97,7%
23087	70	50	55	75	86,6%	93,9%	99,1%	99,5%	97,9%
23088	70	50	55	80	86,8%	93,8%	99,1%	99,5%	98,0%
23089	70	50	60	20	80,7%	93,4%	98,5%	98,8%	95,4%
23090	70	50	60	25	80,9%	93,3%	98,6%	98,9%	95,7%
23091	70	50	60	30	81,2%	93,2%	98,6%	99,0%	96,0%
23092	70	50	60	35	81,5%	93,1%	98,7%	99,0%	96,2%
23093	70	50	60	40	81,7%	93,0%	98,7%	99,1%	96,5%
23094	70	50	60	45	82,0%	93,0%	98,7%	99,2%	96,7%
23095	70	50	60	50	82,3%	92,9%	98,8%	99,2%	96,9%
23096	70	50	60	55	82,5%	92,8%	98,8%	99,3%	97,1%
23097	70	50	60	60	82,8%	92,7%	98,9%	99,3%	97,3%
23098	70	50	60	65	83,0%	92,6%	98,9%	99,4%	97,5%
23099	70	50	60	70	83,3%	92,5%	98,9%	99,4%	97,6%

23100	70	50	60	75	83,5%	92,4%	99,0%	99,5%	97,8%
23101	70	50	60	80	83,8%	92,3%	99,0%	99,5%	97,9%
23102	70	50	65	20	76,5%	91,8%	98,3%	98,8%	95,2%
23103	70	50	65	25	76,8%	91,7%	98,4%	98,8%	95,5%
23104	70	50	65	30	77,1%	91,6%	98,4%	98,9%	95,8%
23105	70	50	65	35	77,5%	91,5%	98,5%	99,0%	96,1%
23106	70	50	65	40	77,8%	91,4%	98,5%	99,1%	96,3%
23107	70	50	65	45	78,1%	91,3%	98,6%	99,1%	96,5%
23108	70	50	65	50	78,4%	91,2%	98,6%	99,2%	96,8%
23109	70	50	65	55	78,7%	91,1%	98,7%	99,2%	97,0%
23110	70	50	65	60	79,0%	91,0%	98,7%	99,3%	97,2%
23111	70	50	65	65	79,3%	90,9%	98,8%	99,3%	97,4%
23112	70	50	65	70	79,5%	90,8%	98,8%	99,4%	97,5%
23113	70	50	65	75	79,8%	90,6%	98,8%	99,4%	97,7%
23114	70	50	65	80	80,1%	90,5%	98,9%	99,5%	97,8%
23115	70	50	70	20	71,8%	89,9%	98,1%	98,7%	95,0%
23116	70	50	70	25	72,2%	89,8%	98,2%	98,8%	95,3%
23117	70	50	70	30	72,5%	89,6%	98,2%	98,9%	95,6%
23118	70	50	70	35	72,9%	89,5%	98,3%	98,9%	95,9%
23119	70	50	70	40	73,2%	89,4%	98,3%	99,0%	96,2%
23120	70	50	70	45	73,5%	89,3%	98,4%	99,1%	96,4%
23121	70	50	70	50	73,9%	89,1%	98,4%	99,1%	96,6%
23122	70	50	70	55	74,2%	89,0%	98,5%	99,2%	96,9%
23123	70	50	70	60	74,6%	88,9%	98,5%	99,2%	97,1%
23124	70	50	70	65	74,9%	88,8%	98,6%	99,3%	97,2%
23125	70	50	70	70	75,2%	88,6%	98,6%	99,3%	97,4%
23126	70	50	70	75	75,6%	88,5%	98,7%	99,4%	97,6%
23127	70	50	70	80	75,9%	88,4%	98,7%	99,4%	97,7%
23128	70	50	75	20	66,5%	87,6%	97,9%	98,6%	94,8%
23129	70	50	75	25	66,9%	87,4%	97,9%	98,7%	95,1%
23130	70	50	75	30	67,3%	87,3%	98,0%	98,8%	95,5%
23131	70	50	75	35	67,7%	87,1%	98,1%	98,9%	95,7%
23132	70	50	75	40	68,1%	87,0%	98,1%	98,9%	96,0%

23133	70	50	75	45	68,5%	86,8%	98,2%	99,0%	96,3%
23134	70	50	75	50	68,9%	86,7%	98,2%	99,1%	96,5%
23135	70	50	75	55	69,2%	86,5%	98,3%	99,1%	96,7%
23136	70	50	75	60	69,6%	86,4%	98,4%	99,2%	96,9%
23137	70	50	75	65	70,0%	86,2%	98,4%	99,2%	97,1%
23138	70	50	75	70	70,3%	86,1%	98,5%	99,3%	97,3%
23139	70	50	75	75	70,7%	85,9%	98,5%	99,3%	97,5%
23140	70	50	75	80	71,1%	85,8%	98,6%	99,4%	97,7%
23141	70	50	80	20	60,8%	84,8%	97,6%	98,5%	94,6%
23142	70	50	80	25	61,3%	84,7%	97,7%	98,6%	94,9%
23143	70	50	80	30	61,7%	84,5%	97,7%	98,7%	95,3%
23144	70	50	80	35	62,1%	84,3%	97,8%	98,8%	95,6%
23145	70	50	80	40	62,5%	84,1%	97,9%	98,9%	95,8%
23146	70	50	80	45	62,9%	84,0%	97,9%	98,9%	96,1%
23147	70	50	80	50	63,3%	83,8%	98,0%	99,0%	96,4%
23148	70	50	80	55	63,7%	83,6%	98,1%	99,1%	96,6%
23149	70	50	80	60	64,1%	83,4%	98,1%	99,1%	96,8%
23150	70	50	80	65	64,6%	83,3%	98,2%	99,2%	97,0%
23151	70	50	80	70	65,0%	83,1%	98,3%	99,2%	97,2%
23152	70	50	80	75	65,4%	82,9%	98,3%	99,3%	97,4%
23153	70	50	80	80	65,8%	82,7%	98,4%	99,3%	97,6%
23154	70	55	20	20	97,0%	99,0%	99,4%	99,2%	96,3%
23155	70	55	20	25	97,1%	99,0%	99,4%	99,3%	96,6%
23156	70	55	20	30	97,1%	98,9%	99,5%	99,3%	96,8%
23157	70	55	20	35	97,2%	98,9%	99,5%	99,4%	97,0%
23158	70	55	20	40	97,2%	98,9%	99,5%	99,4%	97,2%
23159	70	55	20	45	97,3%	98,9%	99,5%	99,5%	97,4%
23160	70	55	20	50	97,3%	98,9%	99,5%	99,5%	97,5%
23161	70	55	20	55	97,4%	98,9%	99,5%	99,5%	97,7%
23162	70	55	20	60	97,4%	98,9%	99,6%	99,6%	97,8%
23163	70	55	20	65	97,5%	98,9%	99,6%	99,6%	98,0%
23164	70	55	20	70	97,5%	98,8%	99,6%	99,6%	98,1%
23165	70	55	20	75	97,6%	98,8%	99,6%	99,6%	98,2%

23166	70	55	20	80	97,6%	98,8%	99,6%	99,7%	98,3%
23167	70	55	25	20	96,3%	98,7%	99,4%	99,2%	96,2%
23168	70	55	25	25	96,3%	98,7%	99,4%	99,2%	96,4%
23169	70	55	25	30	96,4%	98,7%	99,4%	99,3%	96,6%
23170	70	55	25	35	96,4%	98,7%	99,4%	99,3%	96,9%
23171	70	55	25	40	96,5%	98,6%	99,4%	99,4%	97,1%
23172	70	55	25	45	96,6%	98,6%	99,5%	99,4%	97,3%
23173	70	55	25	50	96,6%	98,6%	99,5%	99,5%	97,4%
23174	70	55	25	55	96,7%	98,6%	99,5%	99,5%	97,6%
23175	70	55	25	60	96,7%	98,6%	99,5%	99,5%	97,7%
23176	70	55	25	65	96,8%	98,6%	99,5%	99,6%	97,9%
23177	70	55	25	70	96,8%	98,5%	99,5%	99,6%	98,0%
23178	70	55	25	75	96,9%	98,5%	99,6%	99,6%	98,2%
23179	70	55	25	80	96,9%	98,5%	99,6%	99,6%	98,3%
23180	70	55	30	20	95,3%	98,4%	99,3%	99,1%	96,0%
23181	70	55	30	25	95,3%	98,4%	99,3%	99,2%	96,3%
23182	70	55	30	30	95,4%	98,3%	99,3%	99,2%	96,5%
23183	70	55	30	35	95,5%	98,3%	99,3%	99,3%	96,7%
23184	70	55	30	40	95,6%	98,3%	99,4%	99,3%	96,9%
23185	70	55	30	45	95,6%	98,3%	99,4%	99,4%	97,1%
23186	70	55	30	50	95,7%	98,3%	99,4%	99,4%	97,3%
23187	70	55	30	55	95,8%	98,2%	99,4%	99,5%	97,5%
23188	70	55	30	60	95,8%	98,2%	99,4%	99,5%	97,7%
23189	70	55	30	65	95,9%	98,2%	99,5%	99,5%	97,8%
23190	70	55	30	70	96,0%	98,2%	99,5%	99,6%	98,0%
23191	70	55	30	75	96,1%	98,1%	99,5%	99,6%	98,1%
23192	70	55	30	80	96,1%	98,1%	99,5%	99,6%	98,2%
23193	70	55	35	20	94,0%	98,0%	99,2%	99,1%	95,9%
23194	70	55	35	25	94,1%	97,9%	99,2%	99,1%	96,1%
23195	70	55	35	30	94,2%	97,9%	99,2%	99,2%	96,4%
23196	70	55	35	35	94,3%	97,9%	99,2%	99,2%	96,6%
23197	70	55	35	40	94,4%	97,9%	99,3%	99,3%	96,8%
23198	70	55	35	45	94,5%	97,8%	99,3%	99,3%	97,0%

23199	70	55	35	50	94,6%	97,8%	99,3%	99,4%	97,2%
23200	70	55	35	55	94,7%	97,8%	99,3%	99,4%	97,4%
23201	70	55	35	60	94,7%	97,8%	99,4%	99,5%	97,6%
23202	70	55	35	65	94,8%	97,7%	99,4%	99,5%	97,7%
23203	70	55	35	70	94,9%	97,7%	99,4%	99,5%	97,9%
23204	70	55	35	75	95,0%	97,7%	99,4%	99,6%	98,0%
23205	70	55	35	80	95,1%	97,6%	99,4%	99,6%	98,1%
23206	70	55	40	20	92,4%	97,5%	99,1%	99,0%	95,7%
23207	70	55	40	25	92,6%	97,4%	99,1%	99,1%	96,0%
23208	70	55	40	30	92,7%	97,4%	99,1%	99,1%	96,2%
23209	70	55	40	35	92,8%	97,4%	99,2%	99,2%	96,5%
23210	70	55	40	40	92,9%	97,3%	99,2%	99,2%	96,7%
23211	70	55	40	45	93,0%	97,3%	99,2%	99,3%	96,9%
23212	70	55	40	50	93,2%	97,3%	99,2%	99,3%	97,1%
23213	70	55	40	55	93,3%	97,2%	99,3%	99,4%	97,3%
23214	70	55	40	60	93,4%	97,2%	99,3%	99,4%	97,5%
23215	70	55	40	65	93,5%	97,2%	99,3%	99,5%	97,6%
23216	70	55	40	70	93,6%	97,1%	99,3%	99,5%	97,8%
23217	70	55	40	75	93,7%	97,1%	99,4%	99,5%	97,9%
23218	70	55	40	80	93,8%	97,0%	99,4%	99,6%	98,1%
23219	70	55	45	20	90,5%	96,8%	98,9%	99,0%	95,5%
23220	70	55	45	25	90,7%	96,8%	99,0%	99,0%	95,8%
23221	70	55	45	30	90,8%	96,7%	99,0%	99,1%	96,1%
23222	70	55	45	35	91,0%	96,7%	99,0%	99,1%	96,3%
23223	70	55	45	40	91,1%	96,7%	99,1%	99,2%	96,6%
23224	70	55	45	45	91,3%	96,6%	99,1%	99,3%	96,8%
23225	70	55	45	50	91,4%	96,6%	99,1%	99,3%	97,0%
23226	70	55	45	55	91,5%	96,5%	99,2%	99,4%	97,2%
23227	70	55	45	60	91,7%	96,5%	99,2%	99,4%	97,4%
23228	70	55	45	65	91,8%	96,4%	99,2%	99,4%	97,5%
23229	70	55	45	70	91,9%	96,4%	99,2%	99,5%	97,7%
23230	70	55	45	75	92,1%	96,3%	99,3%	99,5%	97,8%
23231	70	55	45	80	92,2%	96,3%	99,3%	99,5%	98,0%

23232	70	55	50	20	88,2%	96,0%	98,8%	98,9%	95,3%
23233	70	55	50	25	88,4%	96,0%	98,8%	99,0%	95,6%
23234	70	55	50	30	88,6%	95,9%	98,9%	99,0%	95,9%
23235	70	55	50	35	88,7%	95,9%	98,9%	99,1%	96,2%
23236	70	55	50	40	88,9%	95,8%	98,9%	99,2%	96,4%
23237	70	55	50	45	89,1%	95,8%	99,0%	99,2%	96,6%
23238	70	55	50	50	89,3%	95,7%	99,0%	99,3%	96,9%
23239	70	55	50	55	89,4%	95,7%	99,0%	99,3%	97,1%
23240	70	55	50	60	89,6%	95,6%	99,1%	99,4%	97,2%
23241	70	55	50	65	89,7%	95,6%	99,1%	99,4%	97,4%
23242	70	55	50	70	89,9%	95,5%	99,1%	99,4%	97,6%
23243	70	55	50	75	90,1%	95,4%	99,2%	99,5%	97,7%
23244	70	55	50	80	90,2%	95,4%	99,2%	99,5%	97,9%
23245	70	55	55	20	85,4%	95,1%	98,6%	98,8%	95,1%
23246	70	55	55	25	85,6%	95,0%	98,7%	98,9%	95,5%
23247	70	55	55	30	85,8%	94,9%	98,7%	99,0%	95,7%
23248	70	55	55	35	86,0%	94,9%	98,8%	99,0%	96,0%
23249	70	55	55	40	86,2%	94,8%	98,8%	99,1%	96,3%
23250	70	55	55	45	86,4%	94,7%	98,9%	99,2%	96,5%
23251	70	55	55	50	86,6%	94,7%	98,9%	99,2%	96,7%
23252	70	55	55	55	86,8%	94,6%	98,9%	99,3%	96,9%
23253	70	55	55	60	87,0%	94,5%	99,0%	99,3%	97,1%
23254	70	55	55	65	87,2%	94,5%	99,0%	99,4%	97,3%
23255	70	55	55	70	87,4%	94,4%	99,0%	99,4%	97,5%
23256	70	55	55	75	87,6%	94,3%	99,1%	99,4%	97,7%
23257	70	55	55	80	87,8%	94,3%	99,1%	99,5%	97,8%
23258	70	55	60	20	82,0%	93,8%	98,5%	98,7%	95,0%
23259	70	55	60	25	82,3%	93,8%	98,5%	98,8%	95,3%
23260	70	55	60	30	82,5%	93,7%	98,6%	98,9%	95,6%
23261	70	55	60	35	82,8%	93,6%	98,6%	99,0%	95,9%
23262	70	55	60	40	83,0%	93,5%	98,7%	99,0%	96,1%
23263	70	55	60	45	83,3%	93,5%	98,7%	99,1%	96,4%
23264	70	55	60	50	83,5%	93,4%	98,7%	99,2%	96,6%

23265	70	55	60	55	83,8%	93,3%	98,8%	99,2%	96,8%
23266	70	55	60	60	84,0%	93,2%	98,8%	99,3%	97,0%
23267	70	55	60	65	84,2%	93,1%	98,9%	99,3%	97,2%
23268	70	55	60	70	84,5%	93,0%	98,9%	99,4%	97,4%
23269	70	55	60	75	84,7%	93,0%	98,9%	99,4%	97,6%
23270	70	55	60	80	84,9%	92,9%	99,0%	99,4%	97,7%
23271	70	55	65	20	78,1%	92,4%	98,3%	98,7%	94,8%
23272	70	55	65	25	78,4%	92,3%	98,3%	98,8%	95,1%
23273	70	55	65	30	78,7%	92,2%	98,4%	98,8%	95,4%
23274	70	55	65	35	79,0%	92,1%	98,4%	98,9%	95,7%
23275	70	55	65	40	79,3%	92,0%	98,5%	99,0%	96,0%
23276	70	55	65	45	79,5%	91,9%	98,5%	99,1%	96,2%
23277	70	55	65	50	79,8%	91,8%	98,6%	99,1%	96,5%
23278	70	55	65	55	80,1%	91,7%	98,6%	99,2%	96,7%
23279	70	55	65	60	80,4%	91,6%	98,7%	99,2%	96,9%
23280	70	55	65	65	80,7%	91,5%	98,7%	99,3%	97,1%
23281	70	55	65	70	80,9%	91,4%	98,8%	99,3%	97,3%
23282	70	55	65	75	81,2%	91,3%	98,8%	99,4%	97,5%
23283	70	55	65	80	81,5%	91,2%	98,8%	99,4%	97,6%
23284	70	55	70	20	73,6%	90,6%	98,0%	98,6%	94,5%
23285	70	55	70	25	73,9%	90,5%	98,1%	98,7%	94,9%
23286	70	55	70	30	74,2%	90,3%	98,2%	98,8%	95,2%
23287	70	55	70	35	74,6%	90,2%	98,2%	98,8%	95,5%
23288	70	55	70	40	74,9%	90,1%	98,3%	98,9%	95,8%
23289	70	55	70	45	75,2%	90,0%	98,3%	99,0%	96,1%
23290	70	55	70	50	75,6%	89,9%	98,4%	99,1%	96,3%
23291	70	55	70	55	75,9%	89,8%	98,4%	99,1%	96,6%
23292	70	55	70	60	76,2%	89,6%	98,5%	99,2%	96,8%
23293	70	55	70	65	76,5%	89,5%	98,5%	99,2%	97,0%
23294	70	55	70	70	76,8%	89,4%	98,6%	99,3%	97,2%
23295	70	55	70	75	77,2%	89,3%	98,6%	99,3%	97,4%
23296	70	55	70	80	77,5%	89,1%	98,7%	99,4%	97,5%
23297	70	55	75	20	68,5%	88,4%	97,8%	98,5%	94,3%

23298	70	55	75	25	68,9%	88,3%	97,9%	98,6%	94,7%
23299	70	55	75	30	69,2%	88,1%	97,9%	98,7%	95,0%
23300	70	55	75	35	69,6%	88,0%	98,0%	98,8%	95,3%
23301	70	55	75	40	70,0%	87,9%	98,1%	98,9%	95,6%
23302	70	55	75	45	70,4%	87,7%	98,1%	98,9%	95,9%
23303	70	55	75	50	70,7%	87,6%	98,2%	99,0%	96,2%
23304	70	55	75	55	71,1%	87,4%	98,2%	99,1%	96,4%
23305	70	55	75	60	71,5%	87,3%	98,3%	99,1%	96,6%
23306	70	55	75	65	71,8%	87,1%	98,4%	99,2%	96,9%
23307	70	55	75	70	72,2%	87,0%	98,4%	99,2%	97,1%
23308	70	55	75	75	72,5%	86,8%	98,5%	99,3%	97,2%
23309	70	55	75	80	72,9%	86,7%	98,5%	99,3%	97,4%
23310	70	55	80	20	62,9%	85,8%	97,5%	98,4%	94,1%
23311	70	55	80	25	63,3%	85,7%	97,6%	98,5%	94,5%
23312	70	55	80	30	63,8%	85,5%	97,7%	98,6%	94,8%
23313	70	55	80	35	64,2%	85,3%	97,7%	98,7%	95,1%
23314	70	55	80	40	64,6%	85,2%	97,8%	98,8%	95,5%
23315	70	55	80	45	65,0%	85,0%	97,9%	98,9%	95,7%
23316	70	55	80	50	65,4%	84,8%	98,0%	98,9%	96,0%
23317	70	55	80	55	65,8%	84,7%	98,0%	99,0%	96,3%
23318	70	55	80	60	66,2%	84,5%	98,1%	99,1%	96,5%
23319	70	55	80	65	66,6%	84,3%	98,1%	99,1%	96,7%
23320	70	55	80	70	66,9%	84,2%	98,2%	99,2%	96,9%
23321	70	55	80	75	67,3%	84,0%	98,3%	99,2%	97,1%
23322	70	55	80	80	67,7%	83,8%	98,3%	99,3%	97,3%
23323	70	60	20	20	97,3%	99,1%	99,4%	99,2%	96,0%
23324	70	60	20	25	97,3%	99,0%	99,4%	99,2%	96,2%
23325	70	60	20	30	97,4%	99,0%	99,4%	99,3%	96,5%
23326	70	60	20	35	97,4%	99,0%	99,5%	99,3%	96,7%
23327	70	60	20	40	97,5%	99,0%	99,5%	99,4%	96,9%
23328	70	60	20	45	97,5%	99,0%	99,5%	99,4%	97,1%
23329	70	60	20	50	97,6%	99,0%	99,5%	99,4%	97,3%
23330	70	60	20	55	97,6%	99,0%	99,5%	99,5%	97,5%

23331	70	60	20	60	97,6%	98,9%	99,5%	99,5%	97,6%
23332	70	60	20	65	97,7%	98,9%	99,6%	99,6%	97,8%
23333	70	60	20	70	97,7%	98,9%	99,6%	99,6%	97,9%
23334	70	60	20	75	97,8%	98,9%	99,6%	99,6%	98,1%
23335	70	60	20	80	97,8%	98,9%	99,6%	99,6%	98,2%
23336	70	60	25	20	96,6%	98,8%	99,3%	99,1%	95,8%
23337	70	60	25	25	96,6%	98,8%	99,4%	99,2%	96,1%
23338	70	60	25	30	96,7%	98,8%	99,4%	99,2%	96,3%
23339	70	60	25	35	96,7%	98,8%	99,4%	99,3%	96,6%
23340	70	60	25	40	96,8%	98,7%	99,4%	99,3%	96,8%
23341	70	60	25	45	96,8%	98,7%	99,4%	99,4%	97,0%
23342	70	60	25	50	96,9%	98,7%	99,5%	99,4%	97,2%
23343	70	60	25	55	96,9%	98,7%	99,5%	99,5%	97,4%
23344	70	60	25	60	97,0%	98,7%	99,5%	99,5%	97,5%
23345	70	60	25	65	97,0%	98,7%	99,5%	99,5%	97,7%
23346	70	60	25	70	97,1%	98,6%	99,5%	99,6%	97,8%
23347	70	60	25	75	97,1%	98,6%	99,5%	99,6%	98,0%
23348	70	60	25	80	97,2%	98,6%	99,6%	99,6%	98,1%
23349	70	60	30	20	95,6%	98,5%	99,2%	99,1%	95,6%
23350	70	60	30	25	95,7%	98,5%	99,3%	99,1%	95,9%
23351	70	60	30	30	95,8%	98,5%	99,3%	99,2%	96,2%
23352	70	60	30	35	95,9%	98,4%	99,3%	99,2%	96,4%
23353	70	60	30	40	95,9%	98,4%	99,3%	99,3%	96,6%
23354	70	60	30	45	96,0%	98,4%	99,4%	99,3%	96,9%
23355	70	60	30	50	96,1%	98,4%	99,4%	99,4%	97,1%
23356	70	60	30	55	96,1%	98,4%	99,4%	99,4%	97,3%
23357	70	60	30	60	96,2%	98,3%	99,4%	99,5%	97,4%
23358	70	60	30	65	96,3%	98,3%	99,4%	99,5%	97,6%
23359	70	60	30	70	96,3%	98,3%	99,5%	99,5%	97,8%
23360	70	60	30	75	96,4%	98,3%	99,5%	99,6%	97,9%
23361	70	60	30	80	96,4%	98,3%	99,5%	99,6%	98,0%
23362	70	60	35	20	94,5%	98,1%	99,1%	99,0%	95,5%
23363	70	60	35	25	94,6%	98,1%	99,2%	99,1%	95,7%

23364	70	60	35	30	94,7%	98,1%	99,2%	99,1%	96,0%
23365	70	60	35	35	94,8%	98,1%	99,2%	99,2%	96,3%
23366	70	60	35	40	94,8%	98,0%	99,3%	99,2%	96,5%
23367	70	60	35	45	94,9%	98,0%	99,3%	99,3%	96,7%
23368	70	60	35	50	95,0%	98,0%	99,3%	99,3%	96,9%
23369	70	60	35	55	95,1%	97,9%	99,3%	99,4%	97,1%
23370	70	60	35	60	95,2%	97,9%	99,3%	99,4%	97,3%
23371	70	60	35	65	95,3%	97,9%	99,4%	99,5%	97,5%
23372	70	60	35	70	95,3%	97,9%	99,4%	99,5%	97,7%
23373	70	60	35	75	95,4%	97,8%	99,4%	99,5%	97,8%
23374	70	60	35	80	95,5%	97,8%	99,4%	99,6%	98,0%
23375	70	60	40	20	93,0%	97,6%	99,0%	98,9%	95,3%
23376	70	60	40	25	93,2%	97,6%	99,1%	99,0%	95,6%
23377	70	60	40	30	93,3%	97,6%	99,1%	99,1%	95,9%
23378	70	60	40	35	93,4%	97,6%	99,1%	99,1%	96,1%
23379	70	60	40	40	93,5%	97,5%	99,2%	99,2%	96,4%
23380	70	60	40	45	93,6%	97,5%	99,2%	99,2%	96,6%
23381	70	60	40	50	93,7%	97,5%	99,2%	99,3%	96,8%
23382	70	60	40	55	93,8%	97,4%	99,2%	99,3%	97,0%
23383	70	60	40	60	93,9%	97,4%	99,3%	99,4%	97,2%
23384	70	60	40	65	94,0%	97,4%	99,3%	99,4%	97,4%
23385	70	60	40	70	94,1%	97,3%	99,3%	99,5%	97,6%
23386	70	60	40	75	94,2%	97,3%	99,3%	99,5%	97,7%
23387	70	60	40	80	94,3%	97,3%	99,4%	99,5%	97,9%
23388	70	60	45	20	91,3%	97,1%	98,9%	98,9%	95,1%
23389	70	60	45	25	91,4%	97,0%	98,9%	98,9%	95,4%
23390	70	60	45	30	91,5%	97,0%	99,0%	99,0%	95,7%
23391	70	60	45	35	91,7%	96,9%	99,0%	99,1%	96,0%
23392	70	60	45	40	91,8%	96,9%	99,0%	99,1%	96,2%
23393	70	60	45	45	91,9%	96,9%	99,1%	99,2%	96,5%
23394	70	60	45	50	92,1%	96,8%	99,1%	99,3%	96,7%
23395	70	60	45	55	92,2%	96,8%	99,1%	99,3%	96,9%
23396	70	60	45	60	92,3%	96,7%	99,2%	99,3%	97,1%

23397	70	60	45	65	92,5%	96,7%	99,2%	99,4%	97,3%
23398	70	60	45	70	92,6%	96,7%	99,2%	99,4%	97,5%
23399	70	60	45	75	92,7%	96,6%	99,2%	99,5%	97,6%
23400	70	60	45	80	92,8%	96,6%	99,3%	99,5%	97,8%
23401	70	60	50	20	89,1%	96,3%	98,8%	98,8%	94,9%
23402	70	60	50	25	89,3%	96,3%	98,8%	98,9%	95,2%
23403	70	60	50	30	89,4%	96,2%	98,8%	99,0%	95,5%
23404	70	60	50	35	89,6%	96,2%	98,9%	99,0%	95,8%
23405	70	60	50	40	89,8%	96,1%	98,9%	99,1%	96,1%
23406	70	60	50	45	89,9%	96,1%	99,0%	99,1%	96,3%
23407	70	60	50	50	90,1%	96,0%	99,0%	99,2%	96,6%
23408	70	60	50	55	90,2%	96,0%	99,0%	99,3%	96,8%
23409	70	60	50	60	90,4%	95,9%	99,1%	99,3%	97,0%
23410	70	60	50	65	90,5%	95,9%	99,1%	99,4%	97,2%
23411	70	60	50	70	90,7%	95,8%	99,1%	99,4%	97,4%
23412	70	60	50	75	90,8%	95,8%	99,1%	99,4%	97,5%
23413	70	60	50	80	91,0%	95,7%	99,2%	99,5%	97,7%
23414	70	60	55	20	86,4%	95,4%	98,6%	98,7%	94,7%
23415	70	60	55	25	86,6%	95,4%	98,6%	98,8%	95,0%
23416	70	60	55	30	86,9%	95,3%	98,7%	98,9%	95,3%
23417	70	60	55	35	87,1%	95,2%	98,7%	99,0%	95,6%
23418	70	60	55	40	87,2%	95,2%	98,8%	99,0%	95,9%
23419	70	60	55	45	87,4%	95,1%	98,8%	99,1%	96,2%
23420	70	60	55	50	87,6%	95,1%	98,9%	99,2%	96,4%
23421	70	60	55	55	87,8%	95,0%	98,9%	99,2%	96,6%
23422	70	60	55	60	88,0%	94,9%	98,9%	99,3%	96,9%
23423	70	60	55	65	88,2%	94,9%	99,0%	99,3%	97,1%
23424	70	60	55	70	88,4%	94,8%	99,0%	99,4%	97,2%
23425	70	60	55	75	88,6%	94,7%	99,0%	99,4%	97,4%
23426	70	60	55	80	88,7%	94,7%	99,1%	99,4%	97,6%
23427	70	60	60	20	83,3%	94,3%	98,4%	98,6%	94,5%
23428	70	60	60	25	83,5%	94,2%	98,5%	98,7%	94,8%
23429	70	60	60	30	83,8%	94,1%	98,5%	98,8%	95,1%

23430	70	60	60	35	84,0%	94,1%	98,6%	98,9%	95,5%
23431	70	60	60	40	84,2%	94,0%	98,6%	99,0%	95,7%
23432	70	60	60	45	84,5%	93,9%	98,7%	99,0%	96,0%
23433	70	60	60	50	84,7%	93,8%	98,7%	99,1%	96,3%
23434	70	60	60	55	84,9%	93,8%	98,7%	99,2%	96,5%
23435	70	60	60	60	85,2%	93,7%	98,8%	99,2%	96,7%
23436	70	60	60	65	85,4%	93,6%	98,8%	99,3%	96,9%
23437	70	60	60	70	85,6%	93,5%	98,9%	99,3%	97,1%
23438	70	60	60	75	85,8%	93,5%	98,9%	99,4%	97,3%
23439	70	60	60	80	86,0%	93,4%	98,9%	99,4%	97,5%
23440	70	60	65	20	79,6%	92,9%	98,2%	98,6%	94,3%
23441	70	60	65	25	79,8%	92,8%	98,3%	98,7%	94,6%
23442	70	60	65	30	80,1%	92,7%	98,3%	98,7%	95,0%
23443	70	60	65	35	80,4%	92,6%	98,4%	98,8%	95,3%
23444	70	60	65	40	80,7%	92,6%	98,4%	98,9%	95,6%
23445	70	60	65	45	81,0%	92,5%	98,5%	99,0%	95,9%
23446	70	60	65	50	81,2%	92,4%	98,5%	99,0%	96,1%
23447	70	60	65	55	81,5%	92,3%	98,6%	99,1%	96,4%
23448	70	60	65	60	81,8%	92,2%	98,6%	99,2%	96,6%
23449	70	60	65	65	82,0%	92,1%	98,7%	99,2%	96,8%
23450	70	60	65	70	82,3%	92,0%	98,7%	99,3%	97,0%
23451	70	60	65	75	82,5%	91,9%	98,8%	99,3%	97,2%
23452	70	60	65	80	82,8%	91,8%	98,8%	99,4%	97,4%
23453	70	60	70	20	75,2%	91,2%	98,0%	98,5%	94,0%
23454	70	60	70	25	75,6%	91,1%	98,0%	98,6%	94,4%
23455	70	60	70	30	75,9%	91,0%	98,1%	98,7%	94,8%
23456	70	60	70	35	76,2%	90,9%	98,2%	98,8%	95,1%
23457	70	60	70	40	76,5%	90,8%	98,2%	98,8%	95,4%
23458	70	60	70	45	76,9%	90,7%	98,3%	98,9%	95,7%
23459	70	60	70	50	77,2%	90,6%	98,3%	99,0%	96,0%
23460	70	60	70	55	77,5%	90,5%	98,4%	99,1%	96,2%
23461	70	60	70	60	77,8%	90,3%	98,5%	99,1%	96,5%
23462	70	60	70	65	78,1%	90,2%	98,5%	99,2%	96,7%

23463	70	60	70	70	78,4%	90,1%	98,6%	99,2%	96,9%
23464	70	60	70	75	78,7%	90,0%	98,6%	99,3%	97,1%
23465	70	60	70	80	79,0%	89,9%	98,6%	99,3%	97,3%
23466	70	60	75	20	70,4%	89,2%	97,7%	98,4%	93,8%
23467	70	60	75	25	70,7%	89,1%	97,8%	98,5%	94,2%
23468	70	60	75	30	71,1%	88,9%	97,9%	98,6%	94,5%
23469	70	60	75	35	71,5%	88,8%	97,9%	98,7%	94,9%
23470	70	60	75	40	71,8%	88,7%	98,0%	98,8%	95,2%
23471	70	60	75	45	72,2%	88,5%	98,1%	98,8%	95,5%
23472	70	60	75	50	72,5%	88,4%	98,1%	98,9%	95,8%
23473	70	60	75	55	72,9%	88,3%	98,2%	99,0%	96,1%
23474	70	60	75	60	73,2%	88,1%	98,2%	99,1%	96,3%
23475	70	60	75	65	73,6%	88,0%	98,3%	99,1%	96,6%
23476	70	60	75	70	73,9%	87,9%	98,4%	99,2%	96,8%
23477	70	60	75	75	74,2%	87,7%	98,4%	99,2%	97,0%
23478	70	60	75	80	74,6%	87,6%	98,5%	99,3%	97,2%
23479	70	60	80	20	65,0%	86,8%	97,4%	98,3%	93,5%
23480	70	60	80	25	65,4%	86,6%	97,5%	98,4%	94,0%
23481	70	60	80	30	65,8%	86,4%	97,6%	98,5%	94,3%
23482	70	60	80	35	66,2%	86,3%	97,7%	98,6%	94,7%
23483	70	60	80	40	66,6%	86,1%	97,7%	98,7%	95,0%
23484	70	60	80	45	67,0%	86,0%	97,8%	98,8%	95,3%
23485	70	60	80	50	67,3%	85,8%	97,9%	98,9%	95,6%
23486	70	60	80	55	67,7%	85,7%	98,0%	98,9%	95,9%
23487	70	60	80	60	68,1%	85,5%	98,0%	99,0%	96,2%
23488	70	60	80	65	68,5%	85,3%	98,1%	99,1%	96,4%
23489	70	60	80	70	68,9%	85,2%	98,2%	99,1%	96,6%
23490	70	60	80	75	69,3%	85,0%	98,2%	99,2%	96,9%
23491	70	60	80	80	69,6%	84,8%	98,3%	99,2%	97,1%
23492	70	65	20	20	97,5%	99,1%	99,4%	99,1%	95,6%
23493	70	65	20	25	97,6%	99,1%	99,4%	99,2%	95,9%
23494	70	65	20	30	97,6%	99,1%	99,4%	99,2%	96,1%
23495	70	65	20	35	97,6%	99,1%	99,4%	99,3%	96,4%

23496	70	65	20	40	97,7%	99,1%	99,5%	99,3%	96,6%
23497	70	65	20	45	97,7%	99,1%	99,5%	99,4%	96,8%
23498	70	65	20	50	97,8%	99,1%	99,5%	99,4%	97,0%
23499	70	65	20	55	97,8%	99,0%	99,5%	99,4%	97,2%
23500	70	65	20	60	97,8%	99,0%	99,5%	99,5%	97,4%
23501	70	65	20	65	97,9%	99,0%	99,5%	99,5%	97,6%
23502	70	65	20	70	97,9%	99,0%	99,6%	99,5%	97,7%
23503	70	65	20	75	97,9%	99,0%	99,6%	99,6%	97,9%
23504	70	65	20	80	98,0%	99,0%	99,6%	99,6%	98,0%
23505	70	65	25	20	96,8%	98,9%	99,3%	99,0%	95,4%
23506	70	65	25	25	96,9%	98,9%	99,3%	99,1%	95,7%
23507	70	65	25	30	96,9%	98,9%	99,4%	99,2%	96,0%
23508	70	65	25	35	97,0%	98,9%	99,4%	99,2%	96,2%
23509	70	65	25	40	97,0%	98,8%	99,4%	99,3%	96,5%
23510	70	65	25	45	97,1%	98,8%	99,4%	99,3%	96,7%
23511	70	65	25	50	97,1%	98,8%	99,4%	99,4%	96,9%
23512	70	65	25	55	97,2%	98,8%	99,5%	99,4%	97,1%
23513	70	65	25	60	97,2%	98,8%	99,5%	99,4%	97,3%
23514	70	65	25	65	97,3%	98,8%	99,5%	99,5%	97,5%
23515	70	65	25	70	97,3%	98,7%	99,5%	99,5%	97,6%
23516	70	65	25	75	97,4%	98,7%	99,5%	99,6%	97,8%
23517	70	65	25	80	97,4%	98,7%	99,5%	99,6%	97,9%
23518	70	65	30	20	96,0%	98,6%	99,2%	99,0%	95,2%
23519	70	65	30	25	96,1%	98,6%	99,2%	99,1%	95,5%
23520	70	65	30	30	96,1%	98,6%	99,3%	99,1%	95,8%
23521	70	65	30	35	96,2%	98,6%	99,3%	99,2%	96,1%
23522	70	65	30	40	96,3%	98,5%	99,3%	99,2%	96,3%
23523	70	65	30	45	96,3%	98,5%	99,3%	99,3%	96,6%
23524	70	65	30	50	96,4%	98,5%	99,4%	99,3%	96,8%
23525	70	65	30	55	96,4%	98,5%	99,4%	99,4%	97,0%
23526	70	65	30	60	96,5%	98,5%	99,4%	99,4%	97,2%
23527	70	65	30	65	96,6%	98,4%	99,4%	99,5%	97,4%
23528	70	65	30	70	96,6%	98,4%	99,4%	99,5%	97,5%

23529	70	65	30	75	96,7%	98,4%	99,5%	99,5%	97,7%
23530	70	65	30	80	96,7%	98,4%	99,5%	99,6%	97,8%
23531	70	65	35	20	94,9%	98,3%	99,1%	98,9%	95,0%
23532	70	65	35	25	95,0%	98,2%	99,1%	99,0%	95,3%
23533	70	65	35	30	95,1%	98,2%	99,2%	99,1%	95,6%
23534	70	65	35	35	95,2%	98,2%	99,2%	99,1%	95,9%
23535	70	65	35	40	95,3%	98,2%	99,2%	99,2%	96,2%
23536	70	65	35	45	95,3%	98,1%	99,3%	99,2%	96,4%
23537	70	65	35	50	95,4%	98,1%	99,3%	99,3%	96,6%
23538	70	65	35	55	95,5%	98,1%	99,3%	99,3%	96,9%
23539	70	65	35	60	95,6%	98,1%	99,3%	99,4%	97,1%
23540	70	65	35	65	95,6%	98,1%	99,3%	99,4%	97,3%
23541	70	65	35	70	95,7%	98,0%	99,4%	99,5%	97,4%
23542	70	65	35	75	95,8%	98,0%	99,4%	99,5%	97,6%
23543	70	65	35	80	95,9%	98,0%	99,4%	99,5%	97,8%
23544	70	65	40	20	93,6%	97,8%	99,0%	98,9%	94,8%
23545	70	65	40	25	93,7%	97,8%	99,0%	98,9%	95,2%
23546	70	65	40	30	93,8%	97,8%	99,1%	99,0%	95,5%
23547	70	65	40	35	93,9%	97,7%	99,1%	99,1%	95,7%
23548	70	65	40	40	94,0%	97,7%	99,1%	99,1%	96,0%
23549	70	65	40	45	94,1%	97,7%	99,2%	99,2%	96,3%
23550	70	65	40	50	94,2%	97,6%	99,2%	99,2%	96,5%
23551	70	65	40	55	94,3%	97,6%	99,2%	99,3%	96,7%
23552	70	65	40	60	94,4%	97,6%	99,2%	99,3%	96,9%
23553	70	65	40	65	94,5%	97,6%	99,3%	99,4%	97,1%
23554	70	65	40	70	94,6%	97,5%	99,3%	99,4%	97,3%
23555	70	65	40	75	94,7%	97,5%	99,3%	99,5%	97,5%
23556	70	65	40	80	94,8%	97,5%	99,3%	99,5%	97,7%
23557	70	65	45	20	91,9%	97,3%	98,9%	98,8%	94,6%
23558	70	65	45	25	92,1%	97,2%	98,9%	98,9%	95,0%
23559	70	65	45	30	92,2%	97,2%	98,9%	98,9%	95,3%
23560	70	65	45	35	92,3%	97,2%	99,0%	99,0%	95,6%
23561	70	65	45	40	92,5%	97,1%	99,0%	99,1%	95,9%

23562	70	65	45	45	92,6%	97,1%	99,0%	99,1%	96,1%
23563	70	65	45	50	92,7%	97,1%	99,1%	99,2%	96,4%
23564	70	65	45	55	92,8%	97,0%	99,1%	99,2%	96,6%
23565	70	65	45	60	92,9%	97,0%	99,1%	99,3%	96,8%
23566	70	65	45	65	93,0%	96,9%	99,2%	99,3%	97,0%
23567	70	65	45	70	93,2%	96,9%	99,2%	99,4%	97,2%
23568	70	65	45	75	93,3%	96,9%	99,2%	99,4%	97,4%
23569	70	65	45	80	93,4%	96,8%	99,2%	99,5%	97,6%
23570	70	65	50	20	89,9%	96,6%	98,7%	98,7%	94,4%
23571	70	65	50	25	90,1%	96,5%	98,8%	98,8%	94,8%
23572	70	65	50	30	90,2%	96,5%	98,8%	98,9%	95,1%
23573	70	65	50	35	90,4%	96,5%	98,8%	98,9%	95,4%
23574	70	65	50	40	90,5%	96,4%	98,9%	99,0%	95,7%
23575	70	65	50	45	90,7%	96,4%	98,9%	99,1%	96,0%
23576	70	65	50	50	90,8%	96,3%	99,0%	99,1%	96,2%
23577	70	65	50	55	91,0%	96,3%	99,0%	99,2%	96,5%
23578	70	65	50	60	91,1%	96,2%	99,0%	99,3%	96,7%
23579	70	65	50	65	91,3%	96,2%	99,1%	99,3%	96,9%
23580	70	65	50	70	91,4%	96,1%	99,1%	99,3%	97,1%
23581	70	65	50	75	91,5%	96,1%	99,1%	99,4%	97,3%
23582	70	65	50	80	91,7%	96,0%	99,1%	99,4%	97,5%
23583	70	65	55	20	87,5%	95,7%	98,6%	98,6%	94,2%
23584	70	65	55	25	87,6%	95,7%	98,6%	98,7%	94,5%
23585	70	65	55	30	87,8%	95,6%	98,6%	98,8%	94,9%
23586	70	65	55	35	88,0%	95,6%	98,7%	98,9%	95,2%
23587	70	65	55	40	88,2%	95,5%	98,7%	99,0%	95,5%
23588	70	65	55	45	88,4%	95,5%	98,8%	99,0%	95,8%
23589	70	65	55	50	88,6%	95,4%	98,8%	99,1%	96,1%
23590	70	65	55	55	88,7%	95,4%	98,9%	99,2%	96,3%
23591	70	65	55	60	88,9%	95,3%	98,9%	99,2%	96,6%
23592	70	65	55	65	89,1%	95,2%	98,9%	99,3%	96,8%
23593	70	65	55	70	89,3%	95,2%	99,0%	99,3%	97,0%
23594	70	65	55	75	89,4%	95,1%	99,0%	99,4%	97,2%

23595	70	65	55	80	89,6%	95,1%	99,0%	99,4%	97,4%
23596	70	65	60	20	84,5%	94,7%	98,4%	98,5%	94,0%
23597	70	65	60	25	84,7%	94,6%	98,4%	98,6%	94,3%
23598	70	65	60	30	84,9%	94,6%	98,5%	98,7%	94,7%
23599	70	65	60	35	85,2%	94,5%	98,5%	98,8%	95,0%
23600	70	65	60	40	85,4%	94,4%	98,6%	98,9%	95,3%
23601	70	65	60	45	85,6%	94,4%	98,6%	99,0%	95,6%
23602	70	65	60	50	85,8%	94,3%	98,7%	99,0%	95,9%
23603	70	65	60	55	86,0%	94,2%	98,7%	99,1%	96,2%
23604	70	65	60	60	86,2%	94,1%	98,7%	99,2%	96,4%
23605	70	65	60	65	86,4%	94,1%	98,8%	99,2%	96,6%
23606	70	65	60	70	86,7%	94,0%	98,8%	99,3%	96,9%
23607	70	65	60	75	86,9%	93,9%	98,9%	99,3%	97,1%
23608	70	65	60	80	87,1%	93,8%	98,9%	99,4%	97,3%
23609	70	65	65	20	81,0%	93,4%	98,2%	98,5%	93,7%
23610	70	65	65	25	81,2%	93,3%	98,2%	98,6%	94,1%
23611	70	65	65	30	81,5%	93,2%	98,3%	98,7%	94,5%
23612	70	65	65	35	81,8%	93,2%	98,3%	98,7%	94,8%
23613	70	65	65	40	82,0%	93,1%	98,4%	98,8%	95,2%
23614	70	65	65	45	82,3%	93,0%	98,4%	98,9%	95,5%
23615	70	65	65	50	82,5%	92,9%	98,5%	99,0%	95,7%
23616	70	65	65	55	82,8%	92,8%	98,5%	99,0%	96,0%
23617	70	65	65	60	83,0%	92,7%	98,6%	99,1%	96,3%
23618	70	65	65	65	83,3%	92,6%	98,6%	99,2%	96,5%
23619	70	65	65	70	83,5%	92,6%	98,7%	99,2%	96,7%
23620	70	65	65	75	83,8%	92,5%	98,7%	99,3%	96,9%
23621	70	65	65	80	84,0%	92,4%	98,8%	99,3%	97,1%
23622	70	65	70	20	76,9%	91,8%	97,9%	98,4%	93,5%
23623	70	65	70	25	77,2%	91,7%	98,0%	98,5%	93,9%
23624	70	65	70	30	77,5%	91,6%	98,0%	98,6%	94,3%
23625	70	65	70	35	77,8%	91,5%	98,1%	98,7%	94,6%
23626	70	65	70	40	78,1%	91,4%	98,2%	98,8%	95,0%
23627	70	65	70	45	78,4%	91,3%	98,2%	98,8%	95,3%

23628	70	65	70	50	78,7%	91,2%	98,3%	98,9%	95,6%
23629	70	65	70	55	79,0%	91,1%	98,3%	99,0%	95,9%
23630	70	65	70	60	79,3%	91,0%	98,4%	99,0%	96,1%
23631	70	65	70	65	79,6%	90,9%	98,5%	99,1%	96,4%
23632	70	65	70	70	79,9%	90,8%	98,5%	99,2%	96,6%
23633	70	65	70	75	80,1%	90,7%	98,6%	99,2%	96,8%
23634	70	65	70	80	80,4%	90,6%	98,6%	99,3%	97,0%
23635	70	65	75	20	72,2%	89,9%	97,6%	98,3%	93,2%
23636	70	65	75	25	72,5%	89,8%	97,7%	98,4%	93,6%
23637	70	65	75	30	72,9%	89,7%	97,8%	98,5%	94,0%
23638	70	65	75	35	73,2%	89,6%	97,9%	98,6%	94,4%
23639	70	65	75	40	73,6%	89,4%	97,9%	98,7%	94,8%
23640	70	65	75	45	73,9%	89,3%	98,0%	98,8%	95,1%
23641	70	65	75	50	74,3%	89,2%	98,1%	98,8%	95,4%
23642	70	65	75	55	74,6%	89,1%	98,1%	98,9%	95,7%
23643	70	65	75	60	74,9%	88,9%	98,2%	99,0%	96,0%
23644	70	65	75	65	75,3%	88,8%	98,3%	99,1%	96,2%
23645	70	65	75	70	75,6%	88,7%	98,3%	99,1%	96,5%
23646	70	65	75	75	75,9%	88,5%	98,4%	99,2%	96,7%
23647	70	65	75	80	76,2%	88,4%	98,4%	99,2%	96,9%
23648	70	65	80	20	67,0%	87,6%	97,3%	98,2%	92,9%
23649	70	65	80	25	67,4%	87,5%	97,4%	98,3%	93,4%
23650	70	65	80	30	67,7%	87,3%	97,5%	98,4%	93,8%
23651	70	65	80	35	68,1%	87,2%	97,6%	98,5%	94,2%
23652	70	65	80	40	68,5%	87,1%	97,7%	98,6%	94,5%
23653	70	65	80	45	68,9%	86,9%	97,7%	98,7%	94,9%
23654	70	65	80	50	69,3%	86,8%	97,8%	98,8%	95,2%
23655	70	65	80	55	69,6%	86,6%	97,9%	98,9%	95,5%
23656	70	65	80	60	70,0%	86,5%	98,0%	98,9%	95,8%
23657	70	65	80	65	70,4%	86,3%	98,0%	99,0%	96,1%
23658	70	65	80	70	70,7%	86,1%	98,1%	99,1%	96,3%
23659	70	65	80	75	71,1%	86,0%	98,2%	99,1%	96,6%
23660	70	65	80	80	71,5%	85,8%	98,2%	99,2%	96,8%

23661	70	70	20	20	97,7%	99,2%	99,4%	99,0%	95,2%
23662	70	70	20	25	97,8%	99,2%	99,4%	99,1%	95,5%
23663	70	70	20	30	97,8%	99,2%	99,4%	99,2%	95,8%
23664	70	70	20	35	97,8%	99,2%	99,4%	99,2%	96,0%
23665	70	70	20	40	97,9%	99,1%	99,4%	99,3%	96,3%
23666	70	70	20	45	97,9%	99,1%	99,5%	99,3%	96,5%
23667	70	70	20	50	97,9%	99,1%	99,5%	99,4%	96,7%
23668	70	70	20	55	98,0%	99,1%	99,5%	99,4%	96,9%
23669	70	70	20	60	98,0%	99,1%	99,5%	99,4%	97,1%
23670	70	70	20	65	98,0%	99,1%	99,5%	99,5%	97,3%
23671	70	70	20	70	98,1%	99,1%	99,5%	99,5%	97,5%
23672	70	70	20	75	98,1%	99,1%	99,6%	99,5%	97,7%
23673	70	70	20	80	98,1%	99,1%	99,6%	99,6%	97,8%
23674	70	70	25	20	97,1%	99,0%	99,3%	99,0%	95,0%
23675	70	70	25	25	97,2%	99,0%	99,3%	99,0%	95,3%
23676	70	70	25	30	97,2%	99,0%	99,3%	99,1%	95,6%
23677	70	70	25	35	97,2%	98,9%	99,4%	99,2%	95,9%
23678	70	70	25	40	97,3%	98,9%	99,4%	99,2%	96,1%
23679	70	70	25	45	97,3%	98,9%	99,4%	99,3%	96,4%
23680	70	70	25	50	97,4%	98,9%	99,4%	99,3%	96,6%
23681	70	70	25	55	97,4%	98,9%	99,4%	99,4%	96,8%
23682	70	70	25	60	97,5%	98,9%	99,5%	99,4%	97,0%
23683	70	70	25	65	97,5%	98,9%	99,5%	99,4%	97,2%
23684	70	70	25	70	97,6%	98,8%	99,5%	99,5%	97,4%
23685	70	70	25	75	97,6%	98,8%	99,5%	99,5%	97,6%
23686	70	70	25	80	97,6%	98,8%	99,5%	99,5%	97,7%
23687	70	70	30	20	96,3%	98,7%	99,2%	98,9%	94,8%
23688	70	70	30	25	96,4%	98,7%	99,2%	99,0%	95,1%
23689	70	70	30	30	96,4%	98,7%	99,2%	99,0%	95,4%
23690	70	70	30	35	96,5%	98,7%	99,3%	99,1%	95,7%
23691	70	70	30	40	96,6%	98,7%	99,3%	99,2%	96,0%
23692	70	70	30	45	96,6%	98,6%	99,3%	99,2%	96,2%
23693	70	70	30	50	96,7%	98,6%	99,3%	99,3%	96,5%

23694	70	70	30	55	96,7%	98,6%	99,4%	99,3%	96,7%
23695	70	70	30	60	96,8%	98,6%	99,4%	99,4%	96,9%
23696	70	70	30	65	96,8%	98,6%	99,4%	99,4%	97,1%
23697	70	70	30	70	96,9%	98,5%	99,4%	99,5%	97,3%
23698	70	70	30	75	96,9%	98,5%	99,4%	99,5%	97,5%
23699	70	70	30	80	97,0%	98,5%	99,5%	99,5%	97,6%
23700	70	70	35	20	95,3%	98,4%	99,1%	98,8%	94,6%
23701	70	70	35	25	95,4%	98,4%	99,1%	98,9%	94,9%
23702	70	70	35	30	95,5%	98,4%	99,1%	99,0%	95,2%
23703	70	70	35	35	95,6%	98,3%	99,2%	99,1%	95,5%
23704	70	70	35	40	95,6%	98,3%	99,2%	99,1%	95,8%
23705	70	70	35	45	95,7%	98,3%	99,2%	99,2%	96,1%
23706	70	70	35	50	95,8%	98,3%	99,3%	99,2%	96,3%
23707	70	70	35	55	95,9%	98,2%	99,3%	99,3%	96,6%
23708	70	70	35	60	95,9%	98,2%	99,3%	99,3%	96,8%
23709	70	70	35	65	96,0%	98,2%	99,3%	99,4%	97,0%
23710	70	70	35	70	96,1%	98,2%	99,3%	99,4%	97,2%
23711	70	70	35	75	96,1%	98,1%	99,4%	99,5%	97,4%
23712	70	70	35	80	96,2%	98,1%	99,4%	99,5%	97,5%
23713	70	70	40	20	94,1%	98,0%	99,0%	98,8%	94,3%
23714	70	70	40	25	94,2%	98,0%	99,0%	98,9%	94,7%
23715	70	70	40	30	94,3%	97,9%	99,0%	98,9%	95,0%
23716	70	70	40	35	94,4%	97,9%	99,1%	99,0%	95,3%
23717	70	70	40	40	94,5%	97,9%	99,1%	99,1%	95,6%
23718	70	70	40	45	94,6%	97,9%	99,1%	99,1%	95,9%
23719	70	70	40	50	94,7%	97,8%	99,2%	99,2%	96,2%
23720	70	70	40	55	94,8%	97,8%	99,2%	99,2%	96,4%
23721	70	70	40	60	94,8%	97,8%	99,2%	99,3%	96,6%
23722	70	70	40	65	94,9%	97,7%	99,2%	99,3%	96,9%
23723	70	70	40	70	95,0%	97,7%	99,3%	99,4%	97,1%
23724	70	70	40	75	95,1%	97,7%	99,3%	99,4%	97,3%
23725	70	70	40	80	95,2%	97,7%	99,3%	99,5%	97,4%
23726	70	70	45	20	92,6%	97,5%	98,8%	98,7%	94,1%

23727	70	70	45	25	92,7%	97,4%	98,9%	98,8%	94,5%
23728	70	70	45	30	92,8%	97,4%	98,9%	98,9%	94,8%
23729	70	70	45	35	92,9%	97,4%	98,9%	98,9%	95,2%
23730	70	70	45	40	93,1%	97,3%	99,0%	99,0%	95,5%
23731	70	70	45	45	93,2%	97,3%	99,0%	99,1%	95,7%
23732	70	70	45	50	93,3%	97,3%	99,0%	99,1%	96,0%
23733	70	70	45	55	93,4%	97,2%	99,1%	99,2%	96,3%
23734	70	70	45	60	93,5%	97,2%	99,1%	99,2%	96,5%
23735	70	70	45	65	93,6%	97,2%	99,1%	99,3%	96,7%
23736	70	70	45	70	93,7%	97,1%	99,2%	99,3%	96,9%
23737	70	70	45	75	93,8%	97,1%	99,2%	99,4%	97,1%
23738	70	70	45	80	93,9%	97,1%	99,2%	99,4%	97,3%
23739	70	70	50	20	90,7%	96,8%	98,7%	98,6%	93,9%
23740	70	70	50	25	90,8%	96,8%	98,7%	98,7%	94,3%
23741	70	70	50	30	91,0%	96,8%	98,8%	98,8%	94,6%
23742	70	70	50	35	91,1%	96,7%	98,8%	98,9%	95,0%
23743	70	70	50	40	91,3%	96,7%	98,8%	98,9%	95,3%
23744	70	70	50	45	91,4%	96,6%	98,9%	99,0%	95,6%
23745	70	70	50	50	91,6%	96,6%	98,9%	99,1%	95,9%
23746	70	70	50	55	91,7%	96,5%	99,0%	99,1%	96,1%
23747	70	70	50	60	91,8%	96,5%	99,0%	99,2%	96,4%
23748	70	70	50	65	92,0%	96,5%	99,0%	99,3%	96,6%
23749	70	70	50	70	92,1%	96,4%	99,1%	99,3%	96,8%
23750	70	70	50	75	92,2%	96,4%	99,1%	99,3%	97,0%
23751	70	70	50	80	92,3%	96,3%	99,1%	99,4%	97,2%
23752	70	70	55	20	88,4%	96,1%	98,5%	98,5%	93,6%
23753	70	70	55	25	88,6%	96,0%	98,6%	98,6%	94,0%
23754	70	70	55	30	88,7%	96,0%	98,6%	98,7%	94,4%
23755	70	70	55	35	88,9%	95,9%	98,6%	98,8%	94,8%
23756	70	70	55	40	89,1%	95,8%	98,7%	98,9%	95,1%
23757	70	70	55	45	89,3%	95,8%	98,7%	99,0%	95,4%
23758	70	70	55	50	89,4%	95,7%	98,8%	99,0%	95,7%
23759	70	70	55	55	89,6%	95,7%	98,8%	99,1%	96,0%

23760	70	70	55	60	89,8%	95,6%	98,9%	99,1%	96,2%
23761	70	70	55	65	89,9%	95,6%	98,9%	99,2%	96,5%
23762	70	70	55	70	90,1%	95,5%	98,9%	99,3%	96,7%
23763	70	70	55	75	90,2%	95,5%	99,0%	99,3%	96,9%
23764	70	70	55	80	90,4%	95,4%	99,0%	99,4%	97,1%
23765	70	70	60	20	85,6%	95,1%	98,3%	98,4%	93,4%
23766	70	70	60	25	85,8%	95,0%	98,4%	98,5%	93,8%
23767	70	70	60	30	86,0%	95,0%	98,4%	98,6%	94,2%
23768	70	70	60	35	86,2%	94,9%	98,5%	98,7%	94,6%
23769	70	70	60	40	86,5%	94,8%	98,5%	98,8%	94,9%
23770	70	70	60	45	86,7%	94,8%	98,6%	98,9%	95,2%
23771	70	70	60	50	86,9%	94,7%	98,6%	99,0%	95,5%
23772	70	70	60	55	87,1%	94,6%	98,7%	99,0%	95,8%
23773	70	70	60	60	87,3%	94,6%	98,7%	99,1%	96,1%
23774	70	70	60	65	87,5%	94,5%	98,8%	99,2%	96,3%
23775	70	70	60	70	87,6%	94,4%	98,8%	99,2%	96,6%
23776	70	70	60	75	87,8%	94,4%	98,8%	99,3%	96,8%
23777	70	70	60	80	88,0%	94,3%	98,9%	99,3%	97,0%
23778	70	70	65	20	82,3%	93,9%	98,1%	98,3%	93,1%
23779	70	70	65	25	82,5%	93,8%	98,2%	98,4%	93,6%
23780	70	70	65	30	82,8%	93,7%	98,2%	98,6%	94,0%
23781	70	70	65	35	83,1%	93,6%	98,3%	98,6%	94,3%
23782	70	70	65	40	83,3%	93,6%	98,3%	98,7%	94,7%
23783	70	70	65	45	83,5%	93,5%	98,4%	98,8%	95,0%
23784	70	70	65	50	83,8%	93,4%	98,4%	98,9%	95,3%
23785	70	70	65	55	84,0%	93,3%	98,5%	99,0%	95,6%
23786	70	70	65	60	84,3%	93,2%	98,5%	99,0%	95,9%
23787	70	70	65	65	84,5%	93,2%	98,6%	99,1%	96,2%
23788	70	70	65	70	84,7%	93,1%	98,6%	99,2%	96,4%
23789	70	70	65	75	84,9%	93,0%	98,7%	99,2%	96,6%
23790	70	70	65	80	85,2%	92,9%	98,7%	99,3%	96,9%
23791	70	70	70	20	78,4%	92,4%	97,8%	98,2%	92,9%
23792	70	70	70	25	78,7%	92,3%	97,9%	98,4%	93,3%

23793	70	70	70	30	79,0%	92,2%	98,0%	98,5%	93,7%
23794	70	70	70	35	79,3%	92,1%	98,1%	98,6%	94,1%
23795	70	70	70	40	79,6%	92,0%	98,1%	98,7%	94,5%
23796	70	70	70	45	79,9%	91,9%	98,2%	98,7%	94,8%
23797	70	70	70	50	80,1%	91,8%	98,2%	98,8%	95,2%
23798	70	70	70	55	80,4%	91,7%	98,3%	98,9%	95,5%
23799	70	70	70	60	80,7%	91,6%	98,4%	99,0%	95,7%
23800	70	70	70	65	81,0%	91,5%	98,4%	99,0%	96,0%
23801	70	70	70	70	81,2%	91,4%	98,5%	99,1%	96,3%
23802	70	70	70	75	81,5%	91,3%	98,5%	99,2%	96,5%
23803	70	70	70	80	81,8%	91,2%	98,6%	99,2%	96,7%
23804	70	70	75	20	73,9%	90,6%	97,6%	98,1%	92,6%
23805	70	70	75	25	74,3%	90,5%	97,6%	98,3%	93,0%
23806	70	70	75	30	74,6%	90,4%	97,7%	98,4%	93,5%
23807	70	70	75	35	74,9%	90,3%	97,8%	98,5%	93,9%
23808	70	70	75	40	75,3%	90,2%	97,9%	98,6%	94,3%
23809	70	70	75	45	75,6%	90,0%	97,9%	98,7%	94,6%
23810	70	70	75	50	75,9%	89,9%	98,0%	98,8%	95,0%
23811	70	70	75	55	76,2%	89,8%	98,1%	98,8%	95,3%
23812	70	70	75	60	76,6%	89,7%	98,1%	98,9%	95,6%
23813	70	70	75	65	76,9%	89,6%	98,2%	99,0%	95,9%
23814	70	70	75	70	77,2%	89,4%	98,3%	99,1%	96,1%
23815	70	70	75	75	77,5%	89,3%	98,3%	99,1%	96,4%
23816	70	70	75	80	77,8%	89,2%	98,4%	99,2%	96,6%
23817	70	70	80	20	68,9%	88,5%	97,3%	98,0%	92,3%
23818	70	70	80	25	69,3%	88,3%	97,3%	98,1%	92,8%
23819	70	70	80	30	69,7%	88,2%	97,4%	98,3%	93,2%
23820	70	70	80	35	70,0%	88,1%	97,5%	98,4%	93,6%
23821	70	70	80	40	70,4%	87,9%	97,6%	98,5%	94,0%
23822	70	70	80	45	70,8%	87,8%	97,7%	98,6%	94,4%
23823	70	70	80	50	71,1%	87,6%	97,8%	98,7%	94,8%
23824	70	70	80	55	71,5%	87,5%	97,8%	98,8%	95,1%
23825	70	70	80	60	71,8%	87,3%	97,9%	98,8%	95,4%

23826	70	70	80	65	72,2%	87,2%	98,0%	98,9%	95,7%
23827	70	70	80	70	72,5%	87,1%	98,0%	99,0%	96,0%
23828	70	70	80	75	72,9%	86,9%	98,1%	99,1%	96,2%
23829	70	70	80	80	73,2%	86,8%	98,2%	99,1%	96,5%
23830	70	75	20	20	97,9%	99,3%	99,3%	99,0%	94,7%
23831	70	75	20	25	97,9%	99,2%	99,4%	99,0%	95,0%
23832	70	75	20	30	98,0%	99,2%	99,4%	99,1%	95,3%
23833	70	75	20	35	98,0%	99,2%	99,4%	99,2%	95,6%
23834	70	75	20	40	98,1%	99,2%	99,4%	99,2%	95,9%
23835	70	75	20	45	98,1%	99,2%	99,4%	99,3%	96,2%
23836	70	75	20	50	98,1%	99,2%	99,5%	99,3%	96,4%
23837	70	75	20	55	98,1%	99,2%	99,5%	99,4%	96,7%
23838	70	75	20	60	98,2%	99,2%	99,5%	99,4%	96,9%
23839	70	75	20	65	98,2%	99,2%	99,5%	99,4%	97,1%
23840	70	75	20	70	98,2%	99,1%	99,5%	99,5%	97,3%
23841	70	75	20	75	98,3%	99,1%	99,6%	99,5%	97,4%
23842	70	75	20	80	98,3%	99,1%	99,6%	99,5%	97,6%
23843	70	75	25	20	97,3%	99,1%	99,3%	98,9%	94,5%
23844	70	75	25	25	97,4%	99,0%	99,3%	99,0%	94,8%
23845	70	75	25	30	97,4%	99,0%	99,3%	99,0%	95,2%
23846	70	75	25	35	97,5%	99,0%	99,3%	99,1%	95,5%
23847	70	75	25	40	97,5%	99,0%	99,4%	99,2%	95,8%
23848	70	75	25	45	97,6%	99,0%	99,4%	99,2%	96,0%
23849	70	75	25	50	97,6%	99,0%	99,4%	99,3%	96,3%
23850	70	75	25	55	97,6%	99,0%	99,4%	99,3%	96,5%
23851	70	75	25	60	97,7%	99,0%	99,4%	99,4%	96,7%
23852	70	75	25	65	97,7%	98,9%	99,5%	99,4%	96,9%
23853	70	75	25	70	97,8%	98,9%	99,5%	99,4%	97,1%
23854	70	75	25	75	97,8%	98,9%	99,5%	99,5%	97,3%
23855	70	75	25	80	97,8%	98,9%	99,5%	99,5%	97,5%
23856	70	75	30	20	96,6%	98,8%	99,2%	98,8%	94,3%
23857	70	75	30	25	96,7%	98,8%	99,2%	98,9%	94,6%
23858	70	75	30	30	96,7%	98,8%	99,2%	99,0%	95,0%

23859	70	75	30	35	96,8%	98,8%	99,2%	99,0%	95,3%
23860	70	75	30	40	96,8%	98,8%	99,3%	99,1%	95,6%
23861	70	75	30	45	96,9%	98,7%	99,3%	99,2%	95,9%
23862	70	75	30	50	97,0%	98,7%	99,3%	99,2%	96,1%
23863	70	75	30	55	97,0%	98,7%	99,3%	99,3%	96,4%
23864	70	75	30	60	97,1%	98,7%	99,4%	99,3%	96,6%
23865	70	75	30	65	97,1%	98,7%	99,4%	99,4%	96,8%
23866	70	75	30	70	97,2%	98,7%	99,4%	99,4%	97,0%
23867	70	75	30	75	97,2%	98,6%	99,4%	99,4%	97,2%
23868	70	75	30	80	97,2%	98,6%	99,4%	99,5%	97,4%
23869	70	75	35	20	95,7%	98,5%	99,1%	98,8%	94,0%
23870	70	75	35	25	95,8%	98,5%	99,1%	98,8%	94,4%
23871	70	75	35	30	95,9%	98,5%	99,1%	98,9%	94,8%
23872	70	75	35	35	95,9%	98,5%	99,1%	99,0%	95,1%
23873	70	75	35	40	96,0%	98,4%	99,2%	99,1%	95,4%
23874	70	75	35	45	96,1%	98,4%	99,2%	99,1%	95,7%
23875	70	75	35	50	96,1%	98,4%	99,2%	99,2%	96,0%
23876	70	75	35	55	96,2%	98,4%	99,3%	99,2%	96,2%
23877	70	75	35	60	96,3%	98,4%	99,3%	99,3%	96,5%
23878	70	75	35	65	96,3%	98,3%	99,3%	99,3%	96,7%
23879	70	75	35	70	96,4%	98,3%	99,3%	99,4%	96,9%
23880	70	75	35	75	96,4%	98,3%	99,3%	99,4%	97,1%
23881	70	75	35	80	96,5%	98,3%	99,4%	99,5%	97,3%
23882	70	75	40	20	94,6%	98,1%	98,9%	98,7%	93,8%
23883	70	75	40	25	94,7%	98,1%	99,0%	98,8%	94,2%
23884	70	75	40	30	94,8%	98,1%	99,0%	98,8%	94,6%
23885	70	75	40	35	94,8%	98,1%	99,0%	98,9%	94,9%
23886	70	75	40	40	94,9%	98,0%	99,1%	99,0%	95,2%
23887	70	75	40	45	95,0%	98,0%	99,1%	99,1%	95,5%
23888	70	75	40	50	95,1%	98,0%	99,1%	99,1%	95,8%
23889	70	75	40	55	95,2%	98,0%	99,2%	99,2%	96,1%
23890	70	75	40	60	95,3%	97,9%	99,2%	99,2%	96,3%
23891	70	75	40	65	95,3%	97,9%	99,2%	99,3%	96,6%

23892	70	75	40	70	95,4%	97,9%	99,2%	99,3%	96,8%
23893	70	75	40	75	95,5%	97,9%	99,3%	99,4%	97,0%
23894	70	75	40	80	95,6%	97,8%	99,3%	99,4%	97,2%
23895	70	75	45	20	93,2%	97,7%	98,8%	98,6%	93,6%
23896	70	75	45	25	93,3%	97,6%	98,8%	98,7%	94,0%
23897	70	75	45	30	93,4%	97,6%	98,9%	98,8%	94,3%
23898	70	75	45	35	93,5%	97,6%	98,9%	98,9%	94,7%
23899	70	75	45	40	93,6%	97,5%	98,9%	98,9%	95,0%
23900	70	75	45	45	93,7%	97,5%	99,0%	99,0%	95,3%
23901	70	75	45	50	93,8%	97,5%	99,0%	99,1%	95,6%
23902	70	75	45	55	93,9%	97,4%	99,0%	99,1%	95,9%
23903	70	75	45	60	94,0%	97,4%	99,1%	99,2%	96,2%
23904	70	75	45	65	94,1%	97,4%	99,1%	99,2%	96,4%
23905	70	75	45	70	94,2%	97,3%	99,1%	99,3%	96,6%
23906	70	75	45	75	94,3%	97,3%	99,2%	99,3%	96,9%
23907	70	75	45	80	94,4%	97,3%	99,2%	99,4%	97,1%
23908	70	75	50	20	91,4%	97,1%	98,6%	98,5%	93,3%
23909	70	75	50	25	91,6%	97,0%	98,7%	98,6%	93,7%
23910	70	75	50	30	91,7%	97,0%	98,7%	98,7%	94,1%
23911	70	75	50	35	91,8%	97,0%	98,8%	98,8%	94,5%
23912	70	75	50	40	92,0%	96,9%	98,8%	98,9%	94,8%
23913	70	75	50	45	92,1%	96,9%	98,8%	98,9%	95,2%
23914	70	75	50	50	92,2%	96,8%	98,9%	99,0%	95,5%
23915	70	75	50	55	92,3%	96,8%	98,9%	99,1%	95,7%
23916	70	75	50	60	92,5%	96,8%	99,0%	99,1%	96,0%
23917	70	75	50	65	92,6%	96,7%	99,0%	99,2%	96,3%
23918	70	75	50	70	92,7%	96,7%	99,0%	99,2%	96,5%
23919	70	75	50	75	92,8%	96,6%	99,1%	99,3%	96,7%
23920	70	75	50	80	92,9%	96,6%	99,1%	99,3%	96,9%
23921	70	75	55	20	89,3%	96,3%	98,5%	98,4%	93,0%
23922	70	75	55	25	89,4%	96,3%	98,5%	98,5%	93,5%
23923	70	75	55	30	89,6%	96,2%	98,6%	98,6%	93,9%
23924	70	75	55	35	89,8%	96,2%	98,6%	98,7%	94,3%

23925	70	75	55	40	89,9%	96,1%	98,7%	98,8%	94,6%
23926	70	75	55	45	90,1%	96,1%	98,7%	98,9%	95,0%
23927	70	75	55	50	90,2%	96,1%	98,7%	98,9%	95,3%
23928	70	75	55	55	90,4%	96,0%	98,8%	99,0%	95,6%
23929	70	75	55	60	90,5%	96,0%	98,8%	99,1%	95,9%
23930	70	75	55	65	90,7%	95,9%	98,9%	99,1%	96,1%
23931	70	75	55	70	90,8%	95,8%	98,9%	99,2%	96,4%
23932	70	75	55	75	91,0%	95,8%	98,9%	99,3%	96,6%
23933	70	75	55	80	91,1%	95,7%	99,0%	99,3%	96,8%
23934	70	75	60	20	86,7%	95,4%	98,3%	98,3%	92,8%
23935	70	75	60	25	86,9%	95,4%	98,3%	98,4%	93,2%
23936	70	75	60	30	87,1%	95,3%	98,4%	98,5%	93,6%
23937	70	75	60	35	87,3%	95,3%	98,4%	98,6%	94,0%
23938	70	75	60	40	87,5%	95,2%	98,5%	98,7%	94,4%
23939	70	75	60	45	87,7%	95,1%	98,5%	98,8%	94,8%
23940	70	75	60	50	87,8%	95,1%	98,6%	98,9%	95,1%
23941	70	75	60	55	88,0%	95,0%	98,6%	99,0%	95,4%
23942	70	75	60	60	88,2%	95,0%	98,7%	99,0%	95,7%
23943	70	75	60	65	88,4%	94,9%	98,7%	99,1%	96,0%
23944	70	75	60	70	88,6%	94,8%	98,8%	99,1%	96,2%
23945	70	75	60	75	88,8%	94,8%	98,8%	99,2%	96,5%
23946	70	75	60	80	88,9%	94,7%	98,8%	99,3%	96,7%
23947	70	75	65	20	83,5%	94,3%	98,0%	98,2%	92,5%
23948	70	75	65	25	83,8%	94,2%	98,1%	98,3%	93,0%
23949	70	75	65	30	84,0%	94,2%	98,2%	98,4%	93,4%
23950	70	75	65	35	84,3%	94,1%	98,2%	98,5%	93,8%
23951	70	75	65	40	84,5%	94,0%	98,3%	98,6%	94,2%
23952	70	75	65	45	84,7%	94,0%	98,3%	98,7%	94,6%
23953	70	75	65	50	85,0%	93,9%	98,4%	98,8%	94,9%
23954	70	75	65	55	85,2%	93,8%	98,4%	98,9%	95,2%
23955	70	75	65	60	85,4%	93,7%	98,5%	99,0%	95,5%
23956	70	75	65	65	85,6%	93,6%	98,5%	99,0%	95,8%
23957	70	75	65	70	85,8%	93,6%	98,6%	99,1%	96,1%

23958	70	75	65	75	86,0%	93,5%	98,6%	99,2%	96,3%
23959	70	75	65	80	86,3%	93,4%	98,7%	99,2%	96,6%
23960	70	75	70	20	79,9%	92,9%	97,8%	98,1%	92,2%
23961	70	75	70	25	80,2%	92,9%	97,9%	98,2%	92,7%
23962	70	75	70	30	80,4%	92,8%	97,9%	98,3%	93,1%
23963	70	75	70	35	80,7%	92,7%	98,0%	98,5%	93,6%
23964	70	75	70	40	81,0%	92,6%	98,1%	98,6%	94,0%
23965	70	75	70	45	81,2%	92,5%	98,1%	98,7%	94,3%
23966	70	75	70	50	81,5%	92,4%	98,2%	98,7%	94,7%
23967	70	75	70	55	81,8%	92,3%	98,2%	98,8%	95,0%
23968	70	75	70	60	82,0%	92,2%	98,3%	98,9%	95,3%
23969	70	75	70	65	82,3%	92,1%	98,4%	99,0%	95,6%
23970	70	75	70	70	82,6%	92,0%	98,4%	99,0%	95,9%
23971	70	75	70	75	82,8%	91,9%	98,5%	99,1%	96,2%
23972	70	75	70	80	83,1%	91,8%	98,5%	99,2%	96,4%
23973	70	75	75	20	75,6%	91,3%	97,5%	98,0%	91,9%
23974	70	75	75	25	75,9%	91,2%	97,6%	98,1%	92,4%
23975	70	75	75	30	76,3%	91,1%	97,7%	98,2%	92,9%
23976	70	75	75	35	76,6%	90,9%	97,7%	98,4%	93,3%
23977	70	75	75	40	76,9%	90,8%	97,8%	98,5%	93,7%
23978	70	75	75	45	77,2%	90,7%	97,9%	98,6%	94,1%
23979	70	75	75	50	77,5%	90,6%	97,9%	98,7%	94,5%
23980	70	75	75	55	77,8%	90,5%	98,0%	98,7%	94,8%
23981	70	75	75	60	78,1%	90,4%	98,1%	98,8%	95,2%
23982	70	75	75	65	78,4%	90,3%	98,1%	98,9%	95,5%
23983	70	75	75	70	78,7%	90,2%	98,2%	99,0%	95,7%
23984	70	75	75	75	79,0%	90,0%	98,3%	99,0%	96,0%
23985	70	75	75	80	79,3%	89,9%	98,3%	99,1%	96,3%
23986	70	75	80	20	70,8%	89,2%	97,2%	97,9%	91,6%
23987	70	75	80	25	71,1%	89,1%	97,3%	98,0%	92,1%
23988	70	75	80	30	71,5%	89,0%	97,4%	98,1%	92,6%
23989	70	75	80	35	71,9%	88,9%	97,4%	98,3%	93,0%
23990	70	75	80	40	72,2%	88,7%	97,5%	98,4%	93,5%

23991	70	75	80	45	72,6%	88,6%	97,6%	98,5%	93,9%
23992	70	75	80	50	72,9%	88,5%	97,7%	98,6%	94,3%
23993	70	75	80	55	73,3%	88,3%	97,8%	98,7%	94,6%
23994	70	75	80	60	73,6%	88,2%	97,8%	98,8%	95,0%
23995	70	75	80	65	73,9%	88,1%	97,9%	98,8%	95,3%
23996	70	75	80	70	74,3%	87,9%	98,0%	98,9%	95,6%
23997	70	75	80	75	74,6%	87,8%	98,0%	99,0%	95,9%
23998	70	75	80	80	74,9%	87,6%	98,1%	99,1%	96,1%
23999	70	80	20	20	98,1%	99,3%	99,3%	98,9%	94,2%
24000	70	80	20	25	98,1%	99,3%	99,4%	99,0%	94,6%
24001	70	80	20	30	98,1%	99,3%	99,4%	99,0%	94,9%
24002	70	80	20	35	98,2%	99,3%	99,4%	99,1%	95,2%
24003	70	80	20	40	98,2%	99,3%	99,4%	99,1%	95,5%
24004	70	80	20	45	98,2%	99,3%	99,4%	99,2%	95,8%
24005	70	80	20	50	98,3%	99,3%	99,5%	99,3%	96,1%
24006	70	80	20	55	98,3%	99,2%	99,5%	99,3%	96,3%
24007	70	80	20	60	98,3%	99,2%	99,5%	99,4%	96,6%
24008	70	80	20	65	98,4%	99,2%	99,5%	99,4%	96,8%
24009	70	80	20	70	98,4%	99,2%	99,5%	99,4%	97,0%
24010	70	80	20	75	98,4%	99,2%	99,5%	99,5%	97,2%
24011	70	80	20	80	98,4%	99,2%	99,6%	99,5%	97,4%
24012	70	80	25	20	97,6%	99,1%	99,2%	98,8%	94,0%
24013	70	80	25	25	97,6%	99,1%	99,3%	98,9%	94,3%
24014	70	80	25	30	97,6%	99,1%	99,3%	99,0%	94,7%
24015	70	80	25	35	97,7%	99,1%	99,3%	99,0%	95,0%
24016	70	80	25	40	97,7%	99,1%	99,3%	99,1%	95,3%
24017	70	80	25	45	97,8%	99,1%	99,4%	99,2%	95,6%
24018	70	80	25	50	97,8%	99,1%	99,4%	99,2%	95,9%
24019	70	80	25	55	97,8%	99,0%	99,4%	99,3%	96,2%
24020	70	80	25	60	97,9%	99,0%	99,4%	99,3%	96,4%
24021	70	80	25	65	97,9%	99,0%	99,4%	99,4%	96,7%
24022	70	80	25	70	97,9%	99,0%	99,5%	99,4%	96,9%
24023	70	80	25	75	98,0%	99,0%	99,5%	99,4%	97,1%

24024	70	80	25	80	98,0%	99,0%	99,5%	99,5%	97,3%
24025	70	80	30	20	96,9%	98,9%	99,1%	98,7%	93,7%
24026	70	80	30	25	97,0%	98,9%	99,2%	98,8%	94,1%
24027	70	80	30	30	97,0%	98,9%	99,2%	98,9%	94,5%
24028	70	80	30	35	97,1%	98,9%	99,2%	99,0%	94,8%
24029	70	80	30	40	97,1%	98,8%	99,2%	99,0%	95,2%
24030	70	80	30	45	97,2%	98,8%	99,3%	99,1%	95,5%
24031	70	80	30	50	97,2%	98,8%	99,3%	99,2%	95,8%
24032	70	80	30	55	97,2%	98,8%	99,3%	99,2%	96,0%
24033	70	80	30	60	97,3%	98,8%	99,3%	99,3%	96,3%
24034	70	80	30	65	97,3%	98,8%	99,4%	99,3%	96,5%
24035	70	80	30	70	97,4%	98,8%	99,4%	99,4%	96,7%
24036	70	80	30	75	97,4%	98,7%	99,4%	99,4%	96,9%
24037	70	80	30	80	97,5%	98,7%	99,4%	99,4%	97,1%
24038	70	80	35	20	96,1%	98,6%	99,0%	98,7%	93,5%
24039	70	80	35	25	96,1%	98,6%	99,1%	98,7%	93,9%
24040	70	80	35	30	96,2%	98,6%	99,1%	98,8%	94,3%
24041	70	80	35	35	96,3%	98,6%	99,1%	98,9%	94,6%
24042	70	80	35	40	96,3%	98,6%	99,1%	99,0%	95,0%
24043	70	80	35	45	96,4%	98,5%	99,2%	99,0%	95,3%
24044	70	80	35	50	96,4%	98,5%	99,2%	99,1%	95,6%
24045	70	80	35	55	96,5%	98,5%	99,2%	99,2%	95,9%
24046	70	80	35	60	96,6%	98,5%	99,3%	99,2%	96,1%
24047	70	80	35	65	96,6%	98,5%	99,3%	99,3%	96,4%
24048	70	80	35	70	96,7%	98,4%	99,3%	99,3%	96,6%
24049	70	80	35	75	96,7%	98,4%	99,3%	99,4%	96,8%
24050	70	80	35	80	96,8%	98,4%	99,4%	99,4%	97,0%
24051	70	80	40	20	95,0%	98,3%	98,9%	98,6%	93,2%
24052	70	80	40	25	95,1%	98,3%	98,9%	98,7%	93,6%
24053	70	80	40	30	95,2%	98,2%	99,0%	98,8%	94,0%
24054	70	80	40	35	95,3%	98,2%	99,0%	98,8%	94,4%
24055	70	80	40	40	95,3%	98,2%	99,0%	98,9%	94,8%
24056	70	80	40	45	95,4%	98,2%	99,1%	99,0%	95,1%

24057	70	80	40	50	95,5%	98,1%	99,1%	99,1%	95,4%
24058	70	80	40	55	95,6%	98,1%	99,1%	99,1%	95,7%
24059	70	80	40	60	95,6%	98,1%	99,2%	99,2%	96,0%
24060	70	80	40	65	95,7%	98,1%	99,2%	99,2%	96,2%
24061	70	80	40	70	95,8%	98,0%	99,2%	99,3%	96,5%
24062	70	80	40	75	95,9%	98,0%	99,2%	99,3%	96,7%
24063	70	80	40	80	95,9%	98,0%	99,3%	99,4%	96,9%
24064	70	80	45	20	93,7%	97,8%	98,8%	98,5%	93,0%
24065	70	80	45	25	93,8%	97,8%	98,8%	98,6%	93,4%
24066	70	80	45	30	93,9%	97,8%	98,8%	98,7%	93,8%
24067	70	80	45	35	94,0%	97,8%	98,9%	98,8%	94,2%
24068	70	80	45	40	94,1%	97,7%	98,9%	98,8%	94,6%
24069	70	80	45	45	94,2%	97,7%	98,9%	98,9%	94,9%
24070	70	80	45	50	94,3%	97,7%	99,0%	99,0%	95,2%
24071	70	80	45	55	94,4%	97,6%	99,0%	99,1%	95,5%
24072	70	80	45	60	94,5%	97,6%	99,0%	99,1%	95,8%
24073	70	80	45	65	94,6%	97,6%	99,1%	99,2%	96,1%
24074	70	80	45	70	94,7%	97,5%	99,1%	99,2%	96,3%
24075	70	80	45	75	94,8%	97,5%	99,1%	99,3%	96,6%
24076	70	80	45	80	94,8%	97,5%	99,2%	99,3%	96,8%
24077	70	80	50	20	92,1%	97,3%	98,6%	98,4%	92,7%
24078	70	80	50	25	92,2%	97,3%	98,6%	98,5%	93,1%
24079	70	80	50	30	92,3%	97,2%	98,7%	98,6%	93,6%
24080	70	80	50	35	92,5%	97,2%	98,7%	98,7%	94,0%
24081	70	80	50	40	92,6%	97,1%	98,8%	98,8%	94,3%
24082	70	80	50	45	92,7%	97,1%	98,8%	98,9%	94,7%
24083	70	80	50	50	92,8%	97,1%	98,9%	98,9%	95,0%
24084	70	80	50	55	92,9%	97,0%	98,9%	99,0%	95,3%
24085	70	80	50	60	93,1%	97,0%	98,9%	99,1%	95,6%
24086	70	80	50	65	93,2%	97,0%	99,0%	99,1%	95,9%
24087	70	80	50	70	93,3%	96,9%	99,0%	99,2%	96,2%
24088	70	80	50	75	93,4%	96,9%	99,0%	99,2%	96,4%
24089	70	80	50	80	93,5%	96,8%	99,1%	99,3%	96,6%

24090	70	80	55	20	90,1%	96,6%	98,4%	98,3%	92,4%
24091	70	80	55	25	90,2%	96,6%	98,5%	98,4%	92,9%
24092	70	80	55	30	90,4%	96,5%	98,5%	98,5%	93,3%
24093	70	80	55	35	90,6%	96,5%	98,6%	98,6%	93,7%
24094	70	80	55	40	90,7%	96,4%	98,6%	98,7%	94,1%
24095	70	80	55	45	90,9%	96,4%	98,7%	98,8%	94,5%
24096	70	80	55	50	91,0%	96,3%	98,7%	98,9%	94,8%
24097	70	80	55	55	91,1%	96,3%	98,7%	98,9%	95,2%
24098	70	80	55	60	91,3%	96,2%	98,8%	99,0%	95,5%
24099	70	80	55	65	91,4%	96,2%	98,8%	99,1%	95,8%
24100	70	80	55	70	91,6%	96,2%	98,9%	99,1%	96,0%
24101	70	80	55	75	91,7%	96,1%	98,9%	99,2%	96,3%
24102	70	80	55	80	91,8%	96,1%	98,9%	99,2%	96,5%
24103	70	80	60	20	87,7%	95,8%	98,2%	98,2%	92,1%
24104	70	80	60	25	87,8%	95,7%	98,3%	98,3%	92,6%
24105	70	80	60	30	88,0%	95,7%	98,3%	98,4%	93,0%
24106	70	80	60	35	88,2%	95,6%	98,4%	98,5%	93,5%
24107	70	80	60	40	88,4%	95,5%	98,4%	98,6%	93,9%
24108	70	80	60	45	88,6%	95,5%	98,5%	98,7%	94,3%
24109	70	80	60	50	88,8%	95,4%	98,5%	98,8%	94,6%
24110	70	80	60	55	88,9%	95,4%	98,6%	98,9%	95,0%
24111	70	80	60	60	89,1%	95,3%	98,6%	99,0%	95,3%
24112	70	80	60	65	89,3%	95,3%	98,7%	99,0%	95,6%
24113	70	80	60	70	89,4%	95,2%	98,7%	99,1%	95,9%
24114	70	80	60	75	89,6%	95,1%	98,8%	99,1%	96,1%
24115	70	80	60	80	89,8%	95,1%	98,8%	99,2%	96,4%
24116	70	80	65	20	84,7%	94,7%	98,0%	98,1%	91,8%
24117	70	80	65	25	85,0%	94,7%	98,0%	98,2%	92,3%
24118	70	80	65	30	85,2%	94,6%	98,1%	98,3%	92,8%
24119	70	80	65	35	85,4%	94,5%	98,2%	98,4%	93,2%
24120	70	80	65	40	85,6%	94,5%	98,2%	98,5%	93,6%
24121	70	80	65	45	85,8%	94,4%	98,3%	98,6%	94,0%
24122	70	80	65	50	86,1%	94,3%	98,3%	98,7%	94,4%

24123	70	80	65	55	86,3%	94,2%	98,4%	98,8%	94,8%
24124	70	80	65	60	86,5%	94,2%	98,4%	98,9%	95,1%
24125	70	80	65	65	86,7%	94,1%	98,5%	99,0%	95,4%
24126	70	80	65	70	86,9%	94,0%	98,5%	99,0%	95,7%
24127	70	80	65	75	87,1%	94,0%	98,6%	99,1%	96,0%
24128	70	80	65	80	87,3%	93,9%	98,6%	99,2%	96,2%
24129	70	80	70	20	81,3%	93,4%	97,7%	98,0%	91,5%
24130	70	80	70	25	81,5%	93,4%	97,8%	98,1%	92,0%
24131	70	80	70	30	81,8%	93,3%	97,9%	98,2%	92,5%
24132	70	80	70	35	82,1%	93,2%	97,9%	98,3%	93,0%
24133	70	80	70	40	82,3%	93,1%	98,0%	98,4%	93,4%
24134	70	80	70	45	82,6%	93,0%	98,1%	98,5%	93,8%
24135	70	80	70	50	82,8%	92,9%	98,1%	98,6%	94,2%
24136	70	80	70	55	83,1%	92,9%	98,2%	98,7%	94,6%
24137	70	80	70	60	83,3%	92,8%	98,2%	98,8%	94,9%
24138	70	80	70	65	83,6%	92,7%	98,3%	98,9%	95,2%
24139	70	80	70	70	83,8%	92,6%	98,4%	99,0%	95,5%
24140	70	80	70	75	84,0%	92,5%	98,4%	99,0%	95,8%
24141	70	80	70	80	84,3%	92,4%	98,5%	99,1%	96,1%
24142	70	80	75	20	77,2%	91,9%	97,4%	97,8%	91,2%
24143	70	80	75	25	77,5%	91,8%	97,5%	98,0%	91,7%
24144	70	80	75	30	77,8%	91,7%	97,6%	98,1%	92,2%
24145	70	80	75	35	78,1%	91,6%	97,7%	98,2%	92,7%
24146	70	80	75	40	78,4%	91,5%	97,7%	98,3%	93,1%
24147	70	80	75	45	78,7%	91,4%	97,8%	98,5%	93,6%
24148	70	80	75	50	79,0%	91,3%	97,9%	98,6%	94,0%
24149	70	80	75	55	79,3%	91,2%	98,0%	98,7%	94,3%
24150	70	80	75	60	79,6%	91,1%	98,0%	98,7%	94,7%
24151	70	80	75	65	79,9%	90,9%	98,1%	98,8%	95,0%
24152	70	80	75	70	80,2%	90,8%	98,1%	98,9%	95,3%
24153	70	80	75	75	80,4%	90,7%	98,2%	99,0%	95,6%
24154	70	80	75	80	80,7%	90,6%	98,3%	99,0%	95,9%
24155	70	80	80	20	72,6%	90,0%	97,1%	97,7%	90,8%

24156	70	80	80	25	72,9%	89,9%	97,2%	97,9%	91,4%
24157	70	80	80	30	73,3%	89,7%	97,3%	98,0%	91,9%
24158	70	80	80	35	73,6%	89,6%	97,4%	98,1%	92,4%
24159	70	80	80	40	74,0%	89,5%	97,4%	98,2%	92,9%
24160	70	80	80	45	74,3%	89,4%	97,5%	98,4%	93,3%
24161	70	80	80	50	74,6%	89,2%	97,6%	98,5%	93,7%
24162	70	80	80	55	75,0%	89,1%	97,7%	98,6%	94,1%
24163	70	80	80	60	75,3%	89,0%	97,8%	98,7%	94,5%
24164	70	80	80	65	75,6%	88,9%	97,8%	98,8%	94,8%
24165	70	80	80	70	75,9%	88,7%	97,9%	98,8%	95,2%
24166	70	80	80	75	76,3%	88,6%	98,0%	98,9%	95,5%
24167	70	80	80	80	76,6%	88,5%	98,0%	99,0%	95,7%
24168	75	20	20	20	95,8%	98,7%	99,7%	99,7%	98,6%
24169	75	20	20	25	95,8%	98,7%	99,7%	99,7%	98,7%
24170	75	20	20	30	95,9%	98,7%	99,7%	99,7%	98,8%
24171	75	20	20	35	96,0%	98,7%	99,7%	99,7%	98,8%
24172	75	20	20	40	96,0%	98,7%	99,7%	99,8%	98,9%
24173	75	20	20	45	96,1%	98,6%	99,7%	99,8%	99,0%
24174	75	20	20	50	96,2%	98,6%	99,8%	99,8%	99,0%
24175	75	20	20	55	96,2%	98,6%	99,8%	99,8%	99,1%
24176	75	20	20	60	96,3%	98,6%	99,8%	99,8%	99,2%
24177	75	20	20	65	96,4%	98,6%	99,8%	99,8%	99,2%
24178	75	20	20	70	96,4%	98,6%	99,8%	99,8%	99,3%
24179	75	20	20	75	96,5%	98,5%	99,8%	99,9%	99,3%
24180	75	20	20	80	96,5%	98,5%	99,8%	99,9%	99,4%
24181	75	20	25	20	94,6%	98,4%	99,7%	99,7%	98,5%
24182	75	20	25	25	94,7%	98,4%	99,7%	99,7%	98,6%
24183	75	20	25	30	94,8%	98,4%	99,7%	99,7%	98,7%
24184	75	20	25	35	94,9%	98,3%	99,7%	99,7%	98,8%
24185	75	20	25	40	95,0%	98,3%	99,7%	99,8%	98,9%
24186	75	20	25	45	95,1%	98,3%	99,7%	99,8%	98,9%
24187	75	20	25	50	95,1%	98,3%	99,7%	99,8%	99,0%
24188	75	20	25	55	95,2%	98,3%	99,7%	99,8%	99,1%

24189	75	20	25	60	95,3%	98,2%	99,7%	99,8%	99,1%
24190	75	20	25	65	95,4%	98,2%	99,7%	99,8%	99,2%
24191	75	20	25	70	95,5%	98,2%	99,8%	99,8%	99,2%
24192	75	20	25	75	95,5%	98,2%	99,8%	99,8%	99,3%
24193	75	20	25	80	95,6%	98,1%	99,8%	99,9%	99,3%
24194	75	20	30	20	93,2%	98,0%	99,6%	99,7%	98,4%
24195	75	20	30	25	93,3%	98,0%	99,6%	99,7%	98,6%
24196	75	20	30	30	93,4%	98,0%	99,6%	99,7%	98,6%
24197	75	20	30	35	93,6%	97,9%	99,6%	99,7%	98,7%
24198	75	20	30	40	93,7%	97,9%	99,7%	99,7%	98,8%
24199	75	20	30	45	93,8%	97,9%	99,7%	99,8%	98,9%
24200	75	20	30	50	93,9%	97,8%	99,7%	99,8%	99,0%
24201	75	20	30	55	94,0%	97,8%	99,7%	99,8%	99,0%
24202	75	20	30	60	94,1%	97,8%	99,7%	99,8%	99,1%
24203	75	20	30	65	94,2%	97,8%	99,7%	99,8%	99,2%
24204	75	20	30	70	94,3%	97,7%	99,7%	99,8%	99,2%
24205	75	20	30	75	94,4%	97,7%	99,7%	99,8%	99,3%
24206	75	20	30	80	94,4%	97,7%	99,7%	99,8%	99,3%
24207	75	20	35	20	91,5%	97,5%	99,6%	99,6%	98,4%
24208	75	20	35	25	91,6%	97,5%	99,6%	99,7%	98,5%
24209	75	20	35	30	91,8%	97,4%	99,6%	99,7%	98,6%
24210	75	20	35	35	91,9%	97,4%	99,6%	99,7%	98,7%
24211	75	20	35	40	92,0%	97,4%	99,6%	99,7%	98,8%
24212	75	20	35	45	92,2%	97,3%	99,6%	99,7%	98,8%
24213	75	20	35	50	92,3%	97,3%	99,6%	99,8%	98,9%
24214	75	20	35	55	92,4%	97,3%	99,7%	99,8%	99,0%
24215	75	20	35	60	92,5%	97,2%	99,7%	99,8%	99,1%
24216	75	20	35	65	92,7%	97,2%	99,7%	99,8%	99,1%
24217	75	20	35	70	92,8%	97,2%	99,7%	99,8%	99,2%
24218	75	20	35	75	92,9%	97,1%	99,7%	99,8%	99,2%
24219	75	20	35	80	93,0%	97,1%	99,7%	99,8%	99,3%
24220	75	20	40	20	89,4%	96,9%	99,5%	99,6%	98,3%
24221	75	20	40	25	89,5%	96,8%	99,5%	99,6%	98,4%

24222	75	20	40	30	89,7%	96,8%	99,5%	99,7%	98,5%
24223	75	20	40	35	89,9%	96,7%	99,6%	99,7%	98,6%
24224	75	20	40	40	90,0%	96,7%	99,6%	99,7%	98,7%
24225	75	20	40	45	90,2%	96,7%	99,6%	99,7%	98,8%
24226	75	20	40	50	90,3%	96,6%	99,6%	99,7%	98,9%
24227	75	20	40	55	90,5%	96,6%	99,6%	99,8%	99,0%
24228	75	20	40	60	90,6%	96,5%	99,6%	99,8%	99,0%
24229	75	20	40	65	90,8%	96,5%	99,6%	99,8%	99,1%
24230	75	20	40	70	90,9%	96,4%	99,6%	99,8%	99,1%
24231	75	20	40	75	91,1%	96,4%	99,7%	99,8%	99,2%
24232	75	20	40	80	91,2%	96,4%	99,7%	99,8%	99,3%
24233	75	20	45	20	86,8%	96,1%	99,4%	99,6%	98,2%
24234	75	20	45	25	87,0%	96,0%	99,5%	99,6%	98,4%
24235	75	20	45	30	87,2%	96,0%	99,5%	99,6%	98,5%
24236	75	20	45	35	87,4%	95,9%	99,5%	99,7%	98,6%
24237	75	20	45	40	87,6%	95,9%	99,5%	99,7%	98,7%
24238	75	20	45	45	87,8%	95,8%	99,5%	99,7%	98,8%
24239	75	20	45	50	88,0%	95,8%	99,5%	99,7%	98,8%
24240	75	20	45	55	88,1%	95,7%	99,6%	99,7%	98,9%
24241	75	20	45	60	88,3%	95,7%	99,6%	99,8%	99,0%
24242	75	20	45	65	88,5%	95,6%	99,6%	99,8%	99,0%
24243	75	20	45	70	88,7%	95,6%	99,6%	99,8%	99,1%
24244	75	20	45	75	88,9%	95,5%	99,6%	99,8%	99,2%
24245	75	20	45	80	89,0%	95,4%	99,6%	99,8%	99,2%
24246	75	20	50	20	83,7%	95,1%	99,4%	99,6%	98,2%
24247	75	20	50	25	83,9%	95,1%	99,4%	99,6%	98,3%
24248	75	20	50	30	84,2%	95,0%	99,4%	99,6%	98,4%
24249	75	20	50	35	84,4%	94,9%	99,4%	99,6%	98,5%
24250	75	20	50	40	84,6%	94,9%	99,4%	99,7%	98,6%
24251	75	20	50	45	84,9%	94,8%	99,5%	99,7%	98,7%
24252	75	20	50	50	85,1%	94,7%	99,5%	99,7%	98,8%
24253	75	20	50	55	85,3%	94,7%	99,5%	99,7%	98,9%
24254	75	20	50	60	85,5%	94,6%	99,5%	99,7%	98,9%

24255	75	20	50	65	85,7%	94,5%	99,5%	99,8%	99,0%
24256	75	20	50	70	86,0%	94,5%	99,5%	99,8%	99,1%
24257	75	20	50	75	86,2%	94,4%	99,6%	99,8%	99,1%
24258	75	20	50	80	86,4%	94,3%	99,6%	99,8%	99,2%
24259	75	20	55	20	80,0%	93,9%	99,3%	99,5%	98,1%
24260	75	20	55	25	80,3%	93,8%	99,3%	99,6%	98,2%
24261	75	20	55	30	80,6%	93,8%	99,3%	99,6%	98,3%
24262	75	20	55	35	80,9%	93,7%	99,3%	99,6%	98,4%
24263	75	20	55	40	81,1%	93,6%	99,4%	99,6%	98,5%
24264	75	20	55	45	81,4%	93,5%	99,4%	99,7%	98,6%
24265	75	20	55	50	81,7%	93,5%	99,4%	99,7%	98,7%
24266	75	20	55	55	81,9%	93,4%	99,4%	99,7%	98,8%
24267	75	20	55	60	82,2%	93,3%	99,5%	99,7%	98,9%
24268	75	20	55	65	82,4%	93,2%	99,5%	99,7%	99,0%
24269	75	20	55	70	82,7%	93,1%	99,5%	99,8%	99,0%
24270	75	20	55	75	82,9%	93,0%	99,5%	99,8%	99,1%
24271	75	20	55	80	83,2%	93,0%	99,5%	99,8%	99,2%
24272	75	20	60	20	75,8%	92,5%	99,2%	99,5%	98,0%
24273	75	20	60	25	76,1%	92,4%	99,2%	99,5%	98,2%
24274	75	20	60	30	76,4%	92,3%	99,2%	99,6%	98,3%
24275	75	20	60	35	76,7%	92,2%	99,3%	99,6%	98,4%
24276	75	20	60	40	77,1%	92,1%	99,3%	99,6%	98,5%
24277	75	20	60	45	77,4%	92,0%	99,3%	99,6%	98,6%
24278	75	20	60	50	77,7%	91,9%	99,3%	99,7%	98,7%
24279	75	20	60	55	78,0%	91,8%	99,4%	99,7%	98,8%
24280	75	20	60	60	78,3%	91,7%	99,4%	99,7%	98,8%
24281	75	20	60	65	78,6%	91,6%	99,4%	99,7%	98,9%
24282	75	20	60	70	78,9%	91,5%	99,4%	99,7%	99,0%
24283	75	20	60	75	79,2%	91,4%	99,4%	99,8%	99,1%
24284	75	20	60	80	79,5%	91,3%	99,5%	99,8%	99,1%
24285	75	20	65	20	71,0%	90,7%	99,1%	99,5%	97,9%
24286	75	20	65	25	71,3%	90,6%	99,1%	99,5%	98,1%
24287	75	20	65	30	71,7%	90,5%	99,1%	99,5%	98,2%

24288	75	20	65	35	72,1%	90,4%	99,2%	99,6%	98,3%
24289	75	20	65	40	72,4%	90,2%	99,2%	99,6%	98,4%
24290	75	20	65	45	72,8%	90,1%	99,2%	99,6%	98,5%
24291	75	20	65	50	73,1%	90,0%	99,2%	99,6%	98,6%
24292	75	20	65	55	73,5%	89,9%	99,3%	99,7%	98,7%
24293	75	20	65	60	73,8%	89,8%	99,3%	99,7%	98,8%
24294	75	20	65	65	74,1%	89,6%	99,3%	99,7%	98,9%
24295	75	20	65	70	74,5%	89,5%	99,3%	99,7%	99,0%
24296	75	20	65	75	74,8%	89,4%	99,4%	99,7%	99,0%
24297	75	20	65	80	75,1%	89,3%	99,4%	99,8%	99,1%
24298	75	20	70	20	65,6%	88,5%	99,0%	99,4%	97,9%
24299	75	20	70	25	66,0%	88,4%	99,0%	99,5%	98,0%
24300	75	20	70	30	66,4%	88,3%	99,0%	99,5%	98,1%
24301	75	20	70	35	66,8%	88,1%	99,1%	99,5%	98,2%
24302	75	20	70	40	67,2%	88,0%	99,1%	99,6%	98,4%
24303	75	20	70	45	67,6%	87,9%	99,1%	99,6%	98,5%
24304	75	20	70	50	68,0%	87,7%	99,1%	99,6%	98,6%
24305	75	20	70	55	68,4%	87,6%	99,2%	99,6%	98,7%
24306	75	20	70	60	68,8%	87,4%	99,2%	99,7%	98,7%
24307	75	20	70	65	69,1%	87,3%	99,2%	99,7%	98,8%
24308	75	20	70	70	69,5%	87,2%	99,3%	99,7%	98,9%
24309	75	20	70	75	69,9%	87,0%	99,3%	99,7%	99,0%
24310	75	20	70	80	70,2%	86,9%	99,3%	99,8%	99,0%
24311	75	20	75	20	59,9%	86,0%	98,8%	99,4%	97,8%
24312	75	20	75	25	60,3%	85,8%	98,9%	99,4%	97,9%
24313	75	20	75	30	60,7%	85,7%	98,9%	99,5%	98,0%
24314	75	20	75	35	61,1%	85,5%	98,9%	99,5%	98,2%
24315	75	20	75	40	61,6%	85,3%	99,0%	99,5%	98,3%
24316	75	20	75	45	62,0%	85,2%	99,0%	99,6%	98,4%
24317	75	20	75	50	62,4%	85,0%	99,0%	99,6%	98,5%
24318	75	20	75	55	62,8%	84,8%	99,1%	99,6%	98,6%
24319	75	20	75	60	63,2%	84,7%	99,1%	99,7%	98,7%
24320	75	20	75	65	63,6%	84,5%	99,1%	99,7%	98,8%

24321	75	20	75	70	64,0%	84,3%	99,2%	99,7%	98,9%
24322	75	20	75	75	64,4%	84,2%	99,2%	99,7%	98,9%
24323	75	20	75	80	64,8%	84,0%	99,2%	99,7%	99,0%
24324	75	20	80	20	53,8%	83,0%	98,7%	99,4%	97,7%
24325	75	20	80	25	54,3%	82,8%	98,7%	99,4%	97,8%
24326	75	20	80	30	54,7%	82,6%	98,8%	99,4%	98,0%
24327	75	20	80	35	55,1%	82,4%	98,8%	99,5%	98,1%
24328	75	20	80	40	55,6%	82,2%	98,8%	99,5%	98,2%
24329	75	20	80	45	56,0%	82,0%	98,9%	99,5%	98,3%
24330	75	20	80	50	56,4%	81,8%	98,9%	99,6%	98,4%
24331	75	20	80	55	56,9%	81,6%	98,9%	99,6%	98,5%
24332	75	20	80	60	57,3%	81,4%	99,0%	99,6%	98,6%
24333	75	20	80	65	57,7%	81,2%	99,0%	99,7%	98,7%
24334	75	20	80	70	58,2%	81,0%	99,0%	99,7%	98,8%
24335	75	20	80	75	58,6%	80,8%	99,1%	99,7%	98,9%
24336	75	20	80	80	59,0%	80,6%	99,1%	99,7%	99,0%
24337	75	25	20	20	96,1%	98,8%	99,7%	99,7%	98,4%
24338	75	25	20	25	96,2%	98,8%	99,7%	99,7%	98,5%
24339	75	25	20	30	96,2%	98,8%	99,7%	99,7%	98,6%
24340	75	25	20	35	96,3%	98,8%	99,7%	99,7%	98,7%
24341	75	25	20	40	96,4%	98,8%	99,7%	99,7%	98,8%
24342	75	25	20	45	96,4%	98,7%	99,7%	99,8%	98,9%
24343	75	25	20	50	96,5%	98,7%	99,7%	99,8%	99,0%
24344	75	25	20	55	96,5%	98,7%	99,8%	99,8%	99,0%
24345	75	25	20	60	96,6%	98,7%	99,8%	99,8%	99,1%
24346	75	25	20	65	96,7%	98,7%	99,8%	99,8%	99,1%
24347	75	25	20	70	96,7%	98,7%	99,8%	99,8%	99,2%
24348	75	25	20	75	96,8%	98,6%	99,8%	99,8%	99,3%
24349	75	25	20	80	96,8%	98,6%	99,8%	99,9%	99,3%
24350	75	25	25	20	95,1%	98,5%	99,6%	99,6%	98,4%
24351	75	25	25	25	95,1%	98,5%	99,7%	99,7%	98,5%
24352	75	25	25	30	95,2%	98,5%	99,7%	99,7%	98,6%
24353	75	25	25	35	95,3%	98,5%	99,7%	99,7%	98,7%

24354	75	25	25	40	95,4%	98,4%	99,7%	99,7%	98,8%
24355	75	25	25	45	95,5%	98,4%	99,7%	99,7%	98,8%
24356	75	25	25	50	95,5%	98,4%	99,7%	99,8%	98,9%
24357	75	25	25	55	95,6%	98,4%	99,7%	99,8%	99,0%
24358	75	25	25	60	95,7%	98,4%	99,7%	99,8%	99,0%
24359	75	25	25	65	95,8%	98,3%	99,7%	99,8%	99,1%
24360	75	25	25	70	95,8%	98,3%	99,7%	99,8%	99,2%
24361	75	25	25	75	95,9%	98,3%	99,8%	99,8%	99,2%
24362	75	25	25	80	96,0%	98,3%	99,8%	99,8%	99,3%
24363	75	25	30	20	93,8%	98,2%	99,6%	99,6%	98,3%
24364	75	25	30	25	93,9%	98,1%	99,6%	99,6%	98,4%
24365	75	25	30	30	94,0%	98,1%	99,6%	99,7%	98,5%
24366	75	25	30	35	94,1%	98,1%	99,6%	99,7%	98,6%
24367	75	25	30	40	94,2%	98,1%	99,6%	99,7%	98,7%
24368	75	25	30	45	94,3%	98,0%	99,7%	99,7%	98,8%
24369	75	25	30	50	94,4%	98,0%	99,7%	99,8%	98,9%
24370	75	25	30	55	94,5%	98,0%	99,7%	99,8%	98,9%
24371	75	25	30	60	94,5%	98,0%	99,7%	99,8%	99,0%
24372	75	25	30	65	94,6%	97,9%	99,7%	99,8%	99,1%
24373	75	25	30	70	94,7%	97,9%	99,7%	99,8%	99,1%
24374	75	25	30	75	94,8%	97,9%	99,7%	99,8%	99,2%
24375	75	25	30	80	94,9%	97,8%	99,7%	99,8%	99,2%
24376	75	25	35	20	92,2%	97,7%	99,5%	99,6%	98,2%
24377	75	25	35	25	92,3%	97,7%	99,6%	99,6%	98,3%
24378	75	25	35	30	92,4%	97,6%	99,6%	99,7%	98,5%
24379	75	25	35	35	92,5%	97,6%	99,6%	99,7%	98,6%
24380	75	25	35	40	92,7%	97,6%	99,6%	99,7%	98,6%
24381	75	25	35	45	92,8%	97,5%	99,6%	99,7%	98,7%
24382	75	25	35	50	92,9%	97,5%	99,6%	99,7%	98,8%
24383	75	25	35	55	93,0%	97,5%	99,6%	99,8%	98,9%
24384	75	25	35	60	93,1%	97,4%	99,7%	99,8%	99,0%
24385	75	25	35	65	93,2%	97,4%	99,7%	99,8%	99,0%
24386	75	25	35	70	93,3%	97,4%	99,7%	99,8%	99,1%

24387	75	25	35	75	93,5%	97,3%	99,7%	99,8%	99,2%
24388	75	25	35	80	93,6%	97,3%	99,7%	99,8%	99,2%
24389	75	25	40	20	90,2%	97,1%	99,5%	99,6%	98,2%
24390	75	25	40	25	90,3%	97,1%	99,5%	99,6%	98,3%
24391	75	25	40	30	90,5%	97,0%	99,5%	99,6%	98,4%
24392	75	25	40	35	90,6%	97,0%	99,5%	99,7%	98,5%
24393	75	25	40	40	90,8%	96,9%	99,6%	99,7%	98,6%
24394	75	25	40	45	90,9%	96,9%	99,6%	99,7%	98,7%
24395	75	25	40	50	91,1%	96,9%	99,6%	99,7%	98,8%
24396	75	25	40	55	91,2%	96,8%	99,6%	99,7%	98,8%
24397	75	25	40	60	91,4%	96,8%	99,6%	99,8%	98,9%
24398	75	25	40	65	91,5%	96,7%	99,6%	99,8%	99,0%
24399	75	25	40	70	91,6%	96,7%	99,6%	99,8%	99,1%
24400	75	25	40	75	91,8%	96,7%	99,6%	99,8%	99,1%
24401	75	25	40	80	91,9%	96,6%	99,7%	99,8%	99,2%
24402	75	25	45	20	87,8%	96,4%	99,4%	99,5%	98,1%
24403	75	25	45	25	88,0%	96,3%	99,4%	99,6%	98,2%
24404	75	25	45	30	88,1%	96,3%	99,5%	99,6%	98,3%
24405	75	25	45	35	88,3%	96,2%	99,5%	99,6%	98,4%
24406	75	25	45	40	88,5%	96,2%	99,5%	99,7%	98,5%
24407	75	25	45	45	88,7%	96,1%	99,5%	99,7%	98,6%
24408	75	25	45	50	88,9%	96,1%	99,5%	99,7%	98,7%
24409	75	25	45	55	89,0%	96,0%	99,5%	99,7%	98,8%
24410	75	25	45	60	89,2%	96,0%	99,6%	99,7%	98,9%
24411	75	25	45	65	89,4%	95,9%	99,6%	99,8%	99,0%
24412	75	25	45	70	89,5%	95,9%	99,6%	99,8%	99,0%
24413	75	25	45	75	89,7%	95,8%	99,6%	99,8%	99,1%
24414	75	25	45	80	89,9%	95,8%	99,6%	99,8%	99,1%
24415	75	25	50	20	84,9%	95,5%	99,3%	99,5%	98,0%
24416	75	25	50	25	85,1%	95,4%	99,4%	99,6%	98,1%
24417	75	25	50	30	85,3%	95,4%	99,4%	99,6%	98,2%
24418	75	25	50	35	85,5%	95,3%	99,4%	99,6%	98,4%
24419	75	25	50	40	85,7%	95,2%	99,4%	99,6%	98,5%

24420	75	25	50	45	86,0%	95,2%	99,4%	99,7%	98,6%
24421	75	25	50	50	86,2%	95,1%	99,5%	99,7%	98,7%
24422	75	25	50	55	86,4%	95,1%	99,5%	99,7%	98,8%
24423	75	25	50	60	86,6%	95,0%	99,5%	99,7%	98,8%
24424	75	25	50	65	86,8%	94,9%	99,5%	99,7%	98,9%
24425	75	25	50	70	87,0%	94,9%	99,5%	99,8%	99,0%
24426	75	25	50	75	87,2%	94,8%	99,5%	99,8%	99,0%
24427	75	25	50	80	87,4%	94,7%	99,6%	99,8%	99,1%
24428	75	25	55	20	81,4%	94,4%	99,3%	99,5%	97,9%
24429	75	25	55	25	81,7%	94,3%	99,3%	99,5%	98,1%
24430	75	25	55	30	81,9%	94,2%	99,3%	99,6%	98,2%
24431	75	25	55	35	82,2%	94,1%	99,3%	99,6%	98,3%
24432	75	25	55	40	82,5%	94,1%	99,4%	99,6%	98,4%
24433	75	25	55	45	82,7%	94,0%	99,4%	99,6%	98,5%
24434	75	25	55	50	83,0%	93,9%	99,4%	99,7%	98,6%
24435	75	25	55	55	83,2%	93,9%	99,4%	99,7%	98,7%
24436	75	25	55	60	83,5%	93,8%	99,4%	99,7%	98,8%
24437	75	25	55	65	83,7%	93,7%	99,5%	99,7%	98,9%
24438	75	25	55	70	83,9%	93,6%	99,5%	99,7%	98,9%
24439	75	25	55	75	84,2%	93,5%	99,5%	99,8%	99,0%
24440	75	25	55	80	84,4%	93,5%	99,5%	99,8%	99,1%
24441	75	25	60	20	77,4%	93,0%	99,2%	99,5%	97,8%
24442	75	25	60	25	77,7%	92,9%	99,2%	99,5%	98,0%
24443	75	25	60	30	78,0%	92,8%	99,2%	99,5%	98,1%
24444	75	25	60	35	78,3%	92,7%	99,2%	99,6%	98,2%
24445	75	25	60	40	78,6%	92,6%	99,3%	99,6%	98,3%
24446	75	25	60	45	78,9%	92,6%	99,3%	99,6%	98,4%
24447	75	25	60	50	79,2%	92,5%	99,3%	99,6%	98,6%
24448	75	25	60	55	79,5%	92,4%	99,3%	99,7%	98,6%
24449	75	25	60	60	79,8%	92,3%	99,4%	99,7%	98,7%
24450	75	25	60	65	80,0%	92,2%	99,4%	99,7%	98,8%
24451	75	25	60	70	80,3%	92,1%	99,4%	99,7%	98,9%
24452	75	25	60	75	80,6%	92,0%	99,4%	99,7%	99,0%

24453	75	25	60	80	80,9%	91,9%	99,4%	99,8%	99,0%
24454	75	25	65	20	72,8%	91,3%	99,0%	99,4%	97,7%
24455	75	25	65	25	73,1%	91,2%	99,1%	99,5%	97,9%
24456	75	25	65	30	73,5%	91,1%	99,1%	99,5%	98,0%
24457	75	25	65	35	73,8%	91,0%	99,1%	99,5%	98,2%
24458	75	25	65	40	74,1%	90,9%	99,2%	99,6%	98,3%
24459	75	25	65	45	74,5%	90,8%	99,2%	99,6%	98,4%
24460	75	25	65	50	74,8%	90,7%	99,2%	99,6%	98,5%
24461	75	25	65	55	75,1%	90,6%	99,2%	99,6%	98,6%
24462	75	25	65	60	75,5%	90,5%	99,3%	99,7%	98,7%
24463	75	25	65	65	75,8%	90,4%	99,3%	99,7%	98,8%
24464	75	25	65	70	76,1%	90,2%	99,3%	99,7%	98,8%
24465	75	25	65	75	76,4%	90,1%	99,3%	99,7%	98,9%
24466	75	25	65	80	76,8%	90,0%	99,4%	99,7%	99,0%
24467	75	25	70	20	67,6%	89,3%	98,9%	99,4%	97,6%
24468	75	25	70	25	68,0%	89,2%	99,0%	99,4%	97,8%
24469	75	25	70	30	68,4%	89,1%	99,0%	99,5%	97,9%
24470	75	25	70	35	68,8%	88,9%	99,0%	99,5%	98,1%
24471	75	25	70	40	69,1%	88,8%	99,1%	99,5%	98,2%
24472	75	25	70	45	69,5%	88,7%	99,1%	99,6%	98,3%
24473	75	25	70	50	69,9%	88,6%	99,1%	99,6%	98,4%
24474	75	25	70	55	70,3%	88,4%	99,2%	99,6%	98,5%
24475	75	25	70	60	70,6%	88,3%	99,2%	99,6%	98,6%
24476	75	25	70	65	71,0%	88,1%	99,2%	99,7%	98,7%
24477	75	25	70	70	71,4%	88,0%	99,2%	99,7%	98,8%
24478	75	25	70	75	71,7%	87,9%	99,3%	99,7%	98,9%
24479	75	25	70	80	72,1%	87,7%	99,3%	99,7%	99,0%
24480	75	25	75	20	62,0%	86,9%	98,8%	99,3%	97,6%
24481	75	25	75	25	62,4%	86,8%	98,8%	99,4%	97,7%
24482	75	25	75	30	62,8%	86,6%	98,9%	99,4%	97,9%
24483	75	25	75	35	63,2%	86,5%	98,9%	99,5%	98,0%
24484	75	25	75	40	63,6%	86,3%	98,9%	99,5%	98,1%
24485	75	25	75	45	64,0%	86,1%	99,0%	99,5%	98,2%

24486	75	25	75	50	64,5%	86,0%	99,0%	99,6%	98,4%
24487	75	25	75	55	64,9%	85,8%	99,0%	99,6%	98,5%
24488	75	25	75	60	65,3%	85,7%	99,1%	99,6%	98,6%
24489	75	25	75	65	65,7%	85,5%	99,1%	99,6%	98,7%
24490	75	25	75	70	66,1%	85,4%	99,1%	99,7%	98,7%
24491	75	25	75	75	66,4%	85,2%	99,2%	99,7%	98,8%
24492	75	25	75	80	66,8%	85,0%	99,2%	99,7%	98,9%
24493	75	25	80	20	56,0%	84,1%	98,6%	99,3%	97,4%
24494	75	25	80	25	56,5%	83,9%	98,7%	99,4%	97,6%
24495	75	25	80	30	56,9%	83,7%	98,7%	99,4%	97,8%
24496	75	25	80	35	57,3%	83,5%	98,8%	99,4%	97,9%
24497	75	25	80	40	57,8%	83,3%	98,8%	99,5%	98,0%
24498	75	25	80	45	58,2%	83,2%	98,8%	99,5%	98,2%
24499	75	25	80	50	58,6%	83,0%	98,9%	99,5%	98,3%
24500	75	25	80	55	59,0%	82,8%	98,9%	99,6%	98,4%
24501	75	25	80	60	59,5%	82,6%	98,9%	99,6%	98,5%
24502	75	25	80	65	59,9%	82,4%	99,0%	99,6%	98,6%
24503	75	25	80	70	60,3%	82,2%	99,0%	99,7%	98,7%
24504	75	25	80	75	60,7%	82,0%	99,0%	99,7%	98,8%
24505	75	25	80	80	61,2%	81,8%	99,1%	99,7%	98,9%
24506	75	30	20	20	96,4%	98,9%	99,7%	99,6%	98,3%
24507	75	30	20	25	96,5%	98,9%	99,7%	99,7%	98,4%
24508	75	30	20	30	96,5%	98,9%	99,7%	99,7%	98,5%
24509	75	30	20	35	96,6%	98,9%	99,7%	99,7%	98,6%
24510	75	30	20	40	96,7%	98,9%	99,7%	99,7%	98,7%
24511	75	30	20	45	96,7%	98,8%	99,7%	99,7%	98,8%
24512	75	30	20	50	96,8%	98,8%	99,7%	99,8%	98,8%
24513	75	30	20	55	96,8%	98,8%	99,7%	99,8%	98,9%
24514	75	30	20	60	96,9%	98,8%	99,8%	99,8%	99,0%
24515	75	30	20	65	96,9%	98,8%	99,8%	99,8%	99,1%
24516	75	30	20	70	97,0%	98,8%	99,8%	99,8%	99,1%
24517	75	30	20	75	97,0%	98,7%	99,8%	99,8%	99,2%
24518	75	30	20	80	97,1%	98,7%	99,8%	99,8%	99,2%

24519	75	30	25	20	95,5%	98,6%	99,6%	99,6%	98,2%
24520	75	30	25	25	95,5%	98,6%	99,6%	99,6%	98,3%
24521	75	30	25	30	95,6%	98,6%	99,7%	99,7%	98,4%
24522	75	30	25	35	95,7%	98,6%	99,7%	99,7%	98,5%
24523	75	30	25	40	95,8%	98,6%	99,7%	99,7%	98,6%
24524	75	30	25	45	95,8%	98,5%	99,7%	99,7%	98,7%
24525	75	30	25	50	95,9%	98,5%	99,7%	99,7%	98,8%
24526	75	30	25	55	96,0%	98,5%	99,7%	99,8%	98,9%
24527	75	30	25	60	96,0%	98,5%	99,7%	99,8%	99,0%
24528	75	30	25	65	96,1%	98,5%	99,7%	99,8%	99,0%
24529	75	30	25	70	96,2%	98,4%	99,7%	99,8%	99,1%
24530	75	30	25	75	96,2%	98,4%	99,7%	99,8%	99,1%
24531	75	30	25	80	96,3%	98,4%	99,8%	99,8%	99,2%
24532	75	30	30	20	94,3%	98,3%	99,6%	99,6%	98,1%
24533	75	30	30	25	94,4%	98,3%	99,6%	99,6%	98,3%
24534	75	30	30	30	94,5%	98,2%	99,6%	99,6%	98,4%
24535	75	30	30	35	94,5%	98,2%	99,6%	99,7%	98,5%
24536	75	30	30	40	94,6%	98,2%	99,6%	99,7%	98,6%
24537	75	30	30	45	94,7%	98,2%	99,7%	99,7%	98,7%
24538	75	30	30	50	94,8%	98,2%	99,7%	99,7%	98,8%
24539	75	30	30	55	94,9%	98,1%	99,7%	99,7%	98,8%
24540	75	30	30	60	95,0%	98,1%	99,7%	99,8%	98,9%
24541	75	30	30	65	95,1%	98,1%	99,7%	99,8%	99,0%
24542	75	30	30	70	95,1%	98,1%	99,7%	99,8%	99,0%
24543	75	30	30	75	95,2%	98,0%	99,7%	99,8%	99,1%
24544	75	30	30	80	95,3%	98,0%	99,7%	99,8%	99,2%
24545	75	30	35	20	92,8%	97,9%	99,5%	99,6%	98,1%
24546	75	30	35	25	92,9%	97,8%	99,5%	99,6%	98,2%
24547	75	30	35	30	93,0%	97,8%	99,6%	99,6%	98,3%
24548	75	30	35	35	93,1%	97,8%	99,6%	99,6%	98,4%
24549	75	30	35	40	93,2%	97,7%	99,6%	99,7%	98,5%
24550	75	30	35	45	93,3%	97,7%	99,6%	99,7%	98,6%
24551	75	30	35	50	93,5%	97,7%	99,6%	99,7%	98,7%

24552	75	30	35	55	93,6%	97,7%	99,6%	99,7%	98,8%
24553	75	30	35	60	93,7%	97,6%	99,6%	99,8%	98,9%
24554	75	30	35	65	93,8%	97,6%	99,7%	99,8%	98,9%
24555	75	30	35	70	93,9%	97,6%	99,7%	99,8%	99,0%
24556	75	30	35	75	94,0%	97,5%	99,7%	99,8%	99,1%
24557	75	30	35	80	94,1%	97,5%	99,7%	99,8%	99,1%
24558	75	30	40	20	90,9%	97,3%	99,5%	99,5%	98,0%
24559	75	30	40	25	91,1%	97,3%	99,5%	99,6%	98,1%
24560	75	30	40	30	91,2%	97,2%	99,5%	99,6%	98,2%
24561	75	30	40	35	91,4%	97,2%	99,5%	99,6%	98,3%
24562	75	30	40	40	91,5%	97,2%	99,5%	99,7%	98,5%
24563	75	30	40	45	91,6%	97,1%	99,6%	99,7%	98,6%
24564	75	30	40	50	91,8%	97,1%	99,6%	99,7%	98,6%
24565	75	30	40	55	91,9%	97,1%	99,6%	99,7%	98,7%
24566	75	30	40	60	92,0%	97,0%	99,6%	99,7%	98,8%
24567	75	30	40	65	92,2%	97,0%	99,6%	99,8%	98,9%
24568	75	30	40	70	92,3%	96,9%	99,6%	99,8%	99,0%
24569	75	30	40	75	92,4%	96,9%	99,6%	99,8%	99,0%
24570	75	30	40	80	92,5%	96,9%	99,6%	99,8%	99,1%
24571	75	30	45	20	88,7%	96,6%	99,4%	99,5%	97,9%
24572	75	30	45	25	88,9%	96,6%	99,4%	99,5%	98,0%
24573	75	30	45	30	89,0%	96,5%	99,4%	99,6%	98,2%
24574	75	30	45	35	89,2%	96,5%	99,5%	99,6%	98,3%
24575	75	30	45	40	89,4%	96,5%	99,5%	99,6%	98,4%
24576	75	30	45	45	89,5%	96,4%	99,5%	99,7%	98,5%
24577	75	30	45	50	89,7%	96,4%	99,5%	99,7%	98,6%
24578	75	30	45	55	89,9%	96,3%	99,5%	99,7%	98,7%
24579	75	30	45	60	90,0%	96,3%	99,5%	99,7%	98,8%
24580	75	30	45	65	90,2%	96,2%	99,6%	99,7%	98,8%
24581	75	30	45	70	90,3%	96,2%	99,6%	99,8%	98,9%
24582	75	30	45	75	90,5%	96,1%	99,6%	99,8%	99,0%
24583	75	30	45	80	90,6%	96,1%	99,6%	99,8%	99,1%
24584	75	30	50	20	86,0%	95,8%	99,3%	99,5%	97,8%

24585	75	30	50	25	86,2%	95,7%	99,3%	99,5%	97,9%
24586	75	30	50	30	86,4%	95,7%	99,4%	99,5%	98,1%
24587	75	30	50	35	86,6%	95,6%	99,4%	99,6%	98,2%
24588	75	30	50	40	86,8%	95,6%	99,4%	99,6%	98,3%
24589	75	30	50	45	87,0%	95,5%	99,4%	99,6%	98,4%
24590	75	30	50	50	87,2%	95,5%	99,4%	99,7%	98,5%
24591	75	30	50	55	87,4%	95,4%	99,5%	99,7%	98,6%
24592	75	30	50	60	87,6%	95,4%	99,5%	99,7%	98,7%
24593	75	30	50	65	87,8%	95,3%	99,5%	99,7%	98,8%
24594	75	30	50	70	88,0%	95,2%	99,5%	99,7%	98,9%
24595	75	30	50	75	88,1%	95,2%	99,5%	99,8%	99,0%
24596	75	30	50	80	88,3%	95,1%	99,5%	99,8%	99,0%
24597	75	30	55	20	82,7%	94,8%	99,2%	99,5%	97,7%
24598	75	30	55	25	83,0%	94,7%	99,3%	99,5%	97,9%
24599	75	30	55	30	83,2%	94,6%	99,3%	99,5%	98,0%
24600	75	30	55	35	83,5%	94,6%	99,3%	99,6%	98,1%
24601	75	30	55	40	83,7%	94,5%	99,3%	99,6%	98,2%
24602	75	30	55	45	83,9%	94,4%	99,4%	99,6%	98,4%
24603	75	30	55	50	84,2%	94,4%	99,4%	99,6%	98,5%
24604	75	30	55	55	84,4%	94,3%	99,4%	99,7%	98,6%
24605	75	30	55	60	84,6%	94,2%	99,4%	99,7%	98,7%
24606	75	30	55	65	84,9%	94,1%	99,4%	99,7%	98,8%
24607	75	30	55	70	85,1%	94,1%	99,5%	99,7%	98,8%
24608	75	30	55	75	85,3%	94,0%	99,5%	99,7%	98,9%
24609	75	30	55	80	85,5%	93,9%	99,5%	99,8%	99,0%
24610	75	30	60	20	78,9%	93,5%	99,1%	99,4%	97,6%
24611	75	30	60	25	79,2%	93,4%	99,2%	99,5%	97,8%
24612	75	30	60	30	79,5%	93,3%	99,2%	99,5%	97,9%
24613	75	30	60	35	79,8%	93,2%	99,2%	99,5%	98,1%
24614	75	30	60	40	80,0%	93,2%	99,2%	99,6%	98,2%
24615	75	30	60	45	80,3%	93,1%	99,3%	99,6%	98,3%
24616	75	30	60	50	80,6%	93,0%	99,3%	99,6%	98,4%
24617	75	30	60	55	80,9%	92,9%	99,3%	99,6%	98,5%

24618	75	30	60	60	81,1%	92,8%	99,3%	99,7%	98,6%
24619	75	30	60	65	81,4%	92,7%	99,4%	99,7%	98,7%
24620	75	30	60	70	81,7%	92,6%	99,4%	99,7%	98,8%
24621	75	30	60	75	81,9%	92,6%	99,4%	99,7%	98,9%
24622	75	30	60	80	82,2%	92,5%	99,4%	99,7%	98,9%
24623	75	30	65	20	74,5%	91,9%	99,0%	99,4%	97,5%
24624	75	30	65	25	74,8%	91,8%	99,1%	99,4%	97,7%
24625	75	30	65	30	75,2%	91,7%	99,1%	99,5%	97,8%
24626	75	30	65	35	75,5%	91,6%	99,1%	99,5%	98,0%
24627	75	30	65	40	75,8%	91,5%	99,1%	99,5%	98,1%
24628	75	30	65	45	76,1%	91,4%	99,2%	99,6%	98,2%
24629	75	30	65	50	76,5%	91,3%	99,2%	99,6%	98,3%
24630	75	30	65	55	76,8%	91,2%	99,2%	99,6%	98,4%
24631	75	30	65	60	77,1%	91,1%	99,3%	99,6%	98,6%
24632	75	30	65	65	77,4%	91,0%	99,3%	99,7%	98,6%
24633	75	30	65	70	77,7%	90,9%	99,3%	99,7%	98,7%
24634	75	30	65	75	78,0%	90,8%	99,3%	99,7%	98,8%
24635	75	30	65	80	78,3%	90,7%	99,3%	99,7%	98,9%
24636	75	30	70	20	69,5%	90,1%	98,9%	99,3%	97,4%
24637	75	30	70	25	69,9%	89,9%	98,9%	99,4%	97,6%
24638	75	30	70	30	70,3%	89,8%	99,0%	99,4%	97,7%
24639	75	30	70	35	70,6%	89,7%	99,0%	99,5%	97,9%
24640	75	30	70	40	71,0%	89,6%	99,0%	99,5%	98,0%
24641	75	30	70	45	71,4%	89,4%	99,1%	99,5%	98,2%
24642	75	30	70	50	71,7%	89,3%	99,1%	99,6%	98,3%
24643	75	30	70	55	72,1%	89,2%	99,1%	99,6%	98,4%
24644	75	30	70	60	72,4%	89,1%	99,2%	99,6%	98,5%
24645	75	30	70	65	72,8%	88,9%	99,2%	99,6%	98,6%
24646	75	30	70	70	73,1%	88,8%	99,2%	99,7%	98,7%
24647	75	30	70	75	73,5%	88,7%	99,2%	99,7%	98,8%
24648	75	30	70	80	73,8%	88,6%	99,3%	99,7%	98,8%
24649	75	30	75	20	64,1%	87,8%	98,7%	99,3%	97,3%
24650	75	30	75	25	64,5%	87,6%	98,8%	99,3%	97,5%

24651	75	30	75	30	64,9%	87,5%	98,8%	99,4%	97,6%
24652	75	30	75	35	65,3%	87,4%	98,9%	99,4%	97,8%
24653	75	30	75	40	65,7%	87,2%	98,9%	99,5%	97,9%
24654	75	30	75	45	66,1%	87,1%	98,9%	99,5%	98,1%
24655	75	30	75	50	66,5%	86,9%	99,0%	99,5%	98,2%
24656	75	30	75	55	66,8%	86,8%	99,0%	99,6%	98,3%
24657	75	30	75	60	67,2%	86,6%	99,0%	99,6%	98,4%
24658	75	30	75	65	67,6%	86,5%	99,1%	99,6%	98,5%
24659	75	30	75	70	68,0%	86,3%	99,1%	99,6%	98,6%
24660	75	30	75	75	68,4%	86,2%	99,1%	99,7%	98,7%
24661	75	30	75	80	68,8%	86,0%	99,2%	99,7%	98,8%
24662	75	30	80	20	58,2%	85,1%	98,6%	99,3%	97,2%
24663	75	30	80	25	58,6%	84,9%	98,6%	99,3%	97,4%
24664	75	30	80	30	59,1%	84,7%	98,7%	99,4%	97,6%
24665	75	30	80	35	59,5%	84,6%	98,7%	99,4%	97,7%
24666	75	30	80	40	59,9%	84,4%	98,8%	99,4%	97,9%
24667	75	30	80	45	60,3%	84,2%	98,8%	99,5%	98,0%
24668	75	30	80	50	60,8%	84,1%	98,8%	99,5%	98,1%
24669	75	30	80	55	61,2%	83,9%	98,9%	99,5%	98,2%
24670	75	30	80	60	61,6%	83,7%	98,9%	99,6%	98,4%
24671	75	30	80	65	62,0%	83,5%	99,0%	99,6%	98,5%
24672	75	30	80	70	62,4%	83,3%	99,0%	99,6%	98,6%
24673	75	30	80	75	62,8%	83,2%	99,0%	99,7%	98,7%
24674	75	30	80	80	63,2%	83,0%	99,1%	99,7%	98,8%
24675	75	35	20	20	96,7%	99,0%	99,7%	99,6%	98,1%
24676	75	35	20	25	96,8%	99,0%	99,7%	99,6%	98,2%
24677	75	35	20	30	96,8%	99,0%	99,7%	99,7%	98,3%
24678	75	35	20	35	96,9%	99,0%	99,7%	99,7%	98,5%
24679	75	35	20	40	96,9%	98,9%	99,7%	99,7%	98,6%
24680	75	35	20	45	97,0%	98,9%	99,7%	99,7%	98,6%
24681	75	35	20	50	97,0%	98,9%	99,7%	99,7%	98,7%
24682	75	35	20	55	97,1%	98,9%	99,7%	99,8%	98,8%
24683	75	35	20	60	97,1%	98,9%	99,7%	99,8%	98,9%

24684	75	35	20	65	97,2%	98,9%	99,8%	99,8%	99,0%
24685	75	35	20	70	97,2%	98,9%	99,8%	99,8%	99,0%
24686	75	35	20	75	97,3%	98,8%	99,8%	99,8%	99,1%
24687	75	35	20	80	97,3%	98,8%	99,8%	99,8%	99,2%
24688	75	35	25	20	95,8%	98,7%	99,6%	99,6%	98,0%
24689	75	35	25	25	95,9%	98,7%	99,6%	99,6%	98,2%
24690	75	35	25	30	96,0%	98,7%	99,6%	99,6%	98,3%
24691	75	35	25	35	96,0%	98,7%	99,7%	99,7%	98,4%
24692	75	35	25	40	96,1%	98,7%	99,7%	99,7%	98,5%
24693	75	35	25	45	96,2%	98,7%	99,7%	99,7%	98,6%
24694	75	35	25	50	96,2%	98,6%	99,7%	99,7%	98,7%
24695	75	35	25	55	96,3%	98,6%	99,7%	99,7%	98,8%
24696	75	35	25	60	96,4%	98,6%	99,7%	99,8%	98,9%
24697	75	35	25	65	96,4%	98,6%	99,7%	99,8%	98,9%
24698	75	35	25	70	96,5%	98,6%	99,7%	99,8%	99,0%
24699	75	35	25	75	96,5%	98,5%	99,7%	99,8%	99,1%
24700	75	35	25	80	96,6%	98,5%	99,7%	99,8%	99,1%
24701	75	35	30	20	94,7%	98,4%	99,6%	99,6%	97,9%
24702	75	35	30	25	94,8%	98,4%	99,6%	99,6%	98,1%
24703	75	35	30	30	94,9%	98,4%	99,6%	99,6%	98,2%
24704	75	35	30	35	95,0%	98,4%	99,6%	99,6%	98,3%
24705	75	35	30	40	95,1%	98,3%	99,6%	99,7%	98,4%
24706	75	35	30	45	95,2%	98,3%	99,6%	99,7%	98,5%
24707	75	35	30	50	95,2%	98,3%	99,7%	99,7%	98,6%
24708	75	35	30	55	95,3%	98,3%	99,7%	99,7%	98,7%
24709	75	35	30	60	95,4%	98,2%	99,7%	99,7%	98,8%
24710	75	35	30	65	95,5%	98,2%	99,7%	99,8%	98,9%
24711	75	35	30	70	95,5%	98,2%	99,7%	99,8%	99,0%
24712	75	35	30	75	95,6%	98,2%	99,7%	99,8%	99,0%
24713	75	35	30	80	95,7%	98,2%	99,7%	99,8%	99,1%
24714	75	35	35	20	93,4%	98,0%	99,5%	99,5%	97,9%
24715	75	35	35	25	93,5%	98,0%	99,5%	99,6%	98,0%
24716	75	35	35	30	93,6%	98,0%	99,5%	99,6%	98,1%

24717	75	35	35	35	93,7%	97,9%	99,6%	99,6%	98,3%
24718	75	35	35	40	93,8%	97,9%	99,6%	99,6%	98,4%
24719	75	35	35	45	93,9%	97,9%	99,6%	99,7%	98,5%
24720	75	35	35	50	94,0%	97,9%	99,6%	99,7%	98,6%
24721	75	35	35	55	94,1%	97,8%	99,6%	99,7%	98,7%
24722	75	35	35	60	94,2%	97,8%	99,6%	99,7%	98,8%
24723	75	35	35	65	94,3%	97,8%	99,6%	99,8%	98,8%
24724	75	35	35	70	94,4%	97,7%	99,7%	99,8%	98,9%
24725	75	35	35	75	94,5%	97,7%	99,7%	99,8%	99,0%
24726	75	35	35	80	94,5%	97,7%	99,7%	99,8%	99,0%
24727	75	35	40	20	91,6%	97,5%	99,5%	99,5%	97,8%
24728	75	35	40	25	91,8%	97,5%	99,5%	99,5%	97,9%
24729	75	35	40	30	91,9%	97,4%	99,5%	99,6%	98,1%
24730	75	35	40	35	92,0%	97,4%	99,5%	99,6%	98,2%
24731	75	35	40	40	92,2%	97,4%	99,5%	99,6%	98,3%
24732	75	35	40	45	92,3%	97,3%	99,5%	99,7%	98,4%
24733	75	35	40	50	92,4%	97,3%	99,6%	99,7%	98,5%
24734	75	35	40	55	92,5%	97,3%	99,6%	99,7%	98,6%
24735	75	35	40	60	92,7%	97,2%	99,6%	99,7%	98,7%
24736	75	35	40	65	92,8%	97,2%	99,6%	99,7%	98,8%
24737	75	35	40	70	92,9%	97,2%	99,6%	99,8%	98,9%
24738	75	35	40	75	93,0%	97,1%	99,6%	99,8%	98,9%
24739	75	35	40	80	93,1%	97,1%	99,6%	99,8%	99,0%
24740	75	35	45	20	89,5%	96,9%	99,4%	99,5%	97,7%
24741	75	35	45	25	89,7%	96,8%	99,4%	99,5%	97,8%
24742	75	35	45	30	89,9%	96,8%	99,4%	99,5%	98,0%
24743	75	35	45	35	90,0%	96,8%	99,4%	99,6%	98,1%
24744	75	35	45	40	90,2%	96,7%	99,5%	99,6%	98,2%
24745	75	35	45	45	90,3%	96,7%	99,5%	99,6%	98,3%
24746	75	35	45	50	90,5%	96,6%	99,5%	99,7%	98,5%
24747	75	35	45	55	90,6%	96,6%	99,5%	99,7%	98,6%
24748	75	35	45	60	90,8%	96,5%	99,5%	99,7%	98,6%
24749	75	35	45	65	90,9%	96,5%	99,5%	99,7%	98,7%

24750	75	35	45	70	91,1%	96,5%	99,6%	99,7%	98,8%
24751	75	35	45	75	91,2%	96,4%	99,6%	99,8%	98,9%
24752	75	35	45	80	91,4%	96,4%	99,6%	99,8%	99,0%
24753	75	35	50	20	87,0%	96,1%	99,3%	99,4%	97,6%
24754	75	35	50	25	87,2%	96,1%	99,3%	99,5%	97,7%
24755	75	35	50	30	87,4%	96,0%	99,3%	99,5%	97,9%
24756	75	35	50	35	87,6%	96,0%	99,4%	99,5%	98,0%
24757	75	35	50	40	87,8%	95,9%	99,4%	99,6%	98,2%
24758	75	35	50	45	88,0%	95,9%	99,4%	99,6%	98,3%
24759	75	35	50	50	88,2%	95,8%	99,4%	99,6%	98,4%
24760	75	35	50	55	88,3%	95,7%	99,4%	99,7%	98,5%
24761	75	35	50	60	88,5%	95,7%	99,5%	99,7%	98,6%
24762	75	35	50	65	88,7%	95,6%	99,5%	99,7%	98,7%
24763	75	35	50	70	88,9%	95,6%	99,5%	99,7%	98,8%
24764	75	35	50	75	89,0%	95,5%	99,5%	99,7%	98,8%
24765	75	35	50	80	89,2%	95,5%	99,5%	99,8%	98,9%
24766	75	35	55	20	83,9%	95,1%	99,2%	99,4%	97,5%
24767	75	35	55	25	84,2%	95,1%	99,2%	99,4%	97,6%
24768	75	35	55	30	84,4%	95,0%	99,3%	99,5%	97,8%
24769	75	35	55	35	84,6%	95,0%	99,3%	99,5%	97,9%
24770	75	35	55	40	84,9%	94,9%	99,3%	99,6%	98,1%
24771	75	35	55	45	85,1%	94,8%	99,3%	99,6%	98,2%
24772	75	35	55	50	85,3%	94,8%	99,4%	99,6%	98,3%
24773	75	35	55	55	85,5%	94,7%	99,4%	99,6%	98,4%
24774	75	35	55	60	85,8%	94,6%	99,4%	99,7%	98,5%
24775	75	35	55	65	86,0%	94,6%	99,4%	99,7%	98,6%
24776	75	35	55	70	86,2%	94,5%	99,4%	99,7%	98,7%
24777	75	35	55	75	86,4%	94,4%	99,5%	99,7%	98,8%
24778	75	35	55	80	86,6%	94,4%	99,5%	99,7%	98,9%
24779	75	35	60	20	80,3%	94,0%	99,1%	99,4%	97,4%
24780	75	35	60	25	80,6%	93,9%	99,1%	99,4%	97,6%
24781	75	35	60	30	80,9%	93,8%	99,2%	99,5%	97,7%
24782	75	35	60	35	81,2%	93,7%	99,2%	99,5%	97,9%

24783	75	35	60	40	81,4%	93,7%	99,2%	99,5%	98,0%
24784	75	35	60	45	81,7%	93,6%	99,2%	99,6%	98,1%
24785	75	35	60	50	82,0%	93,5%	99,3%	99,6%	98,3%
24786	75	35	60	55	82,2%	93,4%	99,3%	99,6%	98,4%
24787	75	35	60	60	82,5%	93,3%	99,3%	99,6%	98,5%
24788	75	35	60	65	82,7%	93,3%	99,3%	99,7%	98,6%
24789	75	35	60	70	83,0%	93,2%	99,4%	99,7%	98,7%
24790	75	35	60	75	83,2%	93,1%	99,4%	99,7%	98,8%
24791	75	35	60	80	83,5%	93,0%	99,4%	99,7%	98,8%
24792	75	35	65	20	76,1%	92,5%	99,0%	99,3%	97,3%
24793	75	35	65	25	76,5%	92,4%	99,0%	99,4%	97,5%
24794	75	35	65	30	76,8%	92,3%	99,1%	99,4%	97,6%
24795	75	35	65	35	77,1%	92,2%	99,1%	99,5%	97,8%
24796	75	35	65	40	77,4%	92,1%	99,1%	99,5%	97,9%
24797	75	35	65	45	77,7%	92,0%	99,1%	99,5%	98,1%
24798	75	35	65	50	78,0%	91,9%	99,2%	99,6%	98,2%
24799	75	35	65	55	78,3%	91,8%	99,2%	99,6%	98,3%
24800	75	35	65	60	78,6%	91,7%	99,2%	99,6%	98,4%
24801	75	35	65	65	78,9%	91,6%	99,3%	99,6%	98,5%
24802	75	35	65	70	79,2%	91,5%	99,3%	99,7%	98,6%
24803	75	35	65	75	79,5%	91,4%	99,3%	99,7%	98,7%
24804	75	35	65	80	79,8%	91,3%	99,3%	99,7%	98,8%
24805	75	35	70	20	71,4%	90,7%	98,9%	99,3%	97,2%
24806	75	35	70	25	71,7%	90,6%	98,9%	99,3%	97,3%
24807	75	35	70	30	72,1%	90,5%	98,9%	99,4%	97,5%
24808	75	35	70	35	72,4%	90,4%	99,0%	99,4%	97,7%
24809	75	35	70	40	72,8%	90,3%	99,0%	99,5%	97,8%
24810	75	35	70	45	73,1%	90,2%	99,0%	99,5%	98,0%
24811	75	35	70	50	73,5%	90,1%	99,1%	99,5%	98,1%
24812	75	35	70	55	73,8%	89,9%	99,1%	99,6%	98,2%
24813	75	35	70	60	74,2%	89,8%	99,1%	99,6%	98,3%
24814	75	35	70	65	74,5%	89,7%	99,2%	99,6%	98,4%
24815	75	35	70	70	74,8%	89,6%	99,2%	99,6%	98,6%

24816	75	35	70	75	75,2%	89,5%	99,2%	99,7%	98,6%
24817	75	35	70	80	75,5%	89,3%	99,2%	99,7%	98,7%
24818	75	35	75	20	66,1%	88,6%	98,7%	99,2%	97,1%
24819	75	35	75	25	66,5%	88,5%	98,7%	99,3%	97,2%
24820	75	35	75	30	66,9%	88,3%	98,8%	99,3%	97,4%
24821	75	35	75	35	67,3%	88,2%	98,8%	99,4%	97,6%
24822	75	35	75	40	67,6%	88,1%	98,9%	99,4%	97,7%
24823	75	35	75	45	68,0%	87,9%	98,9%	99,5%	97,9%
24824	75	35	75	50	68,4%	87,8%	98,9%	99,5%	98,0%
24825	75	35	75	55	68,8%	87,6%	99,0%	99,5%	98,2%
24826	75	35	75	60	69,2%	87,5%	99,0%	99,6%	98,3%
24827	75	35	75	65	69,5%	87,4%	99,0%	99,6%	98,4%
24828	75	35	75	70	69,9%	87,2%	99,1%	99,6%	98,5%
24829	75	35	75	75	70,3%	87,1%	99,1%	99,6%	98,6%
24830	75	35	75	80	70,6%	86,9%	99,1%	99,7%	98,7%
24831	75	35	80	20	60,3%	86,1%	98,5%	99,2%	96,9%
24832	75	35	80	25	60,8%	85,9%	98,6%	99,3%	97,1%
24833	75	35	80	30	61,2%	85,7%	98,6%	99,3%	97,3%
24834	75	35	80	35	61,6%	85,6%	98,7%	99,3%	97,5%
24835	75	35	80	40	62,0%	85,4%	98,7%	99,4%	97,6%
24836	75	35	80	45	62,4%	85,2%	98,8%	99,4%	97,8%
24837	75	35	80	50	62,8%	85,1%	98,8%	99,5%	97,9%
24838	75	35	80	55	63,3%	84,9%	98,8%	99,5%	98,1%
24839	75	35	80	60	63,7%	84,7%	98,9%	99,5%	98,2%
24840	75	35	80	65	64,1%	84,6%	98,9%	99,6%	98,3%
24841	75	35	80	70	64,5%	84,4%	99,0%	99,6%	98,4%
24842	75	35	80	75	64,9%	84,2%	99,0%	99,6%	98,5%
24843	75	35	80	80	65,3%	84,1%	99,0%	99,6%	98,6%
24844	75	40	20	20	97,0%	99,1%	99,7%	99,6%	97,9%
24845	75	40	20	25	97,0%	99,1%	99,7%	99,6%	98,1%
24846	75	40	20	30	97,1%	99,0%	99,7%	99,6%	98,2%
24847	75	40	20	35	97,1%	99,0%	99,7%	99,7%	98,3%
24848	75	40	20	40	97,2%	99,0%	99,7%	99,7%	98,4%

24849	75	40	20	45	97,2%	99,0%	99,7%	99,7%	98,5%
24850	75	40	20	50	97,3%	99,0%	99,7%	99,7%	98,6%
24851	75	40	20	55	97,3%	99,0%	99,7%	99,7%	98,7%
24852	75	40	20	60	97,4%	99,0%	99,7%	99,8%	98,8%
24853	75	40	20	65	97,4%	99,0%	99,7%	99,8%	98,9%
24854	75	40	20	70	97,5%	98,9%	99,8%	99,8%	98,9%
24855	75	40	20	75	97,5%	98,9%	99,8%	99,8%	99,0%
24856	75	40	20	80	97,5%	98,9%	99,8%	99,8%	99,1%
24857	75	40	25	20	96,2%	98,8%	99,6%	99,6%	97,8%
24858	75	40	25	25	96,2%	98,8%	99,6%	99,6%	98,0%
24859	75	40	25	30	96,3%	98,8%	99,6%	99,6%	98,1%
24860	75	40	25	35	96,4%	98,8%	99,6%	99,6%	98,2%
24861	75	40	25	40	96,4%	98,8%	99,7%	99,7%	98,3%
24862	75	40	25	45	96,5%	98,8%	99,7%	99,7%	98,5%
24863	75	40	25	50	96,5%	98,7%	99,7%	99,7%	98,6%
24864	75	40	25	55	96,6%	98,7%	99,7%	99,7%	98,6%
24865	75	40	25	60	96,7%	98,7%	99,7%	99,7%	98,7%
24866	75	40	25	65	96,7%	98,7%	99,7%	99,8%	98,8%
24867	75	40	25	70	96,8%	98,7%	99,7%	99,8%	98,9%
24868	75	40	25	75	96,8%	98,7%	99,7%	99,8%	99,0%
24869	75	40	25	80	96,9%	98,6%	99,7%	99,8%	99,0%
24870	75	40	30	20	95,2%	98,5%	99,6%	99,5%	97,7%
24871	75	40	30	25	95,2%	98,5%	99,6%	99,6%	97,9%
24872	75	40	30	30	95,3%	98,5%	99,6%	99,6%	98,0%
24873	75	40	30	35	95,4%	98,5%	99,6%	99,6%	98,2%
24874	75	40	30	40	95,5%	98,5%	99,6%	99,6%	98,3%
24875	75	40	30	45	95,5%	98,4%	99,6%	99,7%	98,4%
24876	75	40	30	50	95,6%	98,4%	99,6%	99,7%	98,5%
24877	75	40	30	55	95,7%	98,4%	99,7%	99,7%	98,6%
24878	75	40	30	60	95,8%	98,4%	99,7%	99,7%	98,7%
24879	75	40	30	65	95,8%	98,4%	99,7%	99,7%	98,8%
24880	75	40	30	70	95,9%	98,3%	99,7%	99,8%	98,9%
24881	75	40	30	75	96,0%	98,3%	99,7%	99,8%	98,9%

24882	75	40	30	80	96,0%	98,3%	99,7%	99,8%	99,0%
24883	75	40	35	20	93,9%	98,2%	99,5%	99,5%	97,7%
24884	75	40	35	25	94,0%	98,1%	99,5%	99,5%	97,8%
24885	75	40	35	30	94,1%	98,1%	99,5%	99,6%	97,9%
24886	75	40	35	35	94,2%	98,1%	99,5%	99,6%	98,1%
24887	75	40	35	40	94,3%	98,1%	99,6%	99,6%	98,2%
24888	75	40	35	45	94,4%	98,0%	99,6%	99,6%	98,3%
24889	75	40	35	50	94,5%	98,0%	99,6%	99,7%	98,4%
24890	75	40	35	55	94,6%	98,0%	99,6%	99,7%	98,5%
24891	75	40	35	60	94,6%	98,0%	99,6%	99,7%	98,6%
24892	75	40	35	65	94,7%	97,9%	99,6%	99,7%	98,7%
24893	75	40	35	70	94,8%	97,9%	99,6%	99,7%	98,8%
24894	75	40	35	75	94,9%	97,9%	99,7%	99,8%	98,9%
24895	75	40	35	80	95,0%	97,9%	99,7%	99,8%	99,0%
24896	75	40	40	20	92,3%	97,7%	99,4%	99,5%	97,6%
24897	75	40	40	25	92,4%	97,7%	99,5%	99,5%	97,7%
24898	75	40	40	30	92,5%	97,6%	99,5%	99,5%	97,9%
24899	75	40	40	35	92,7%	97,6%	99,5%	99,6%	98,0%
24900	75	40	40	40	92,8%	97,6%	99,5%	99,6%	98,1%
24901	75	40	40	45	92,9%	97,5%	99,5%	99,6%	98,3%
24902	75	40	40	50	93,0%	97,5%	99,5%	99,6%	98,4%
24903	75	40	40	55	93,1%	97,5%	99,6%	99,7%	98,5%
24904	75	40	40	60	93,2%	97,4%	99,6%	99,7%	98,6%
24905	75	40	40	65	93,4%	97,4%	99,6%	99,7%	98,7%
24906	75	40	40	70	93,5%	97,4%	99,6%	99,7%	98,8%
24907	75	40	40	75	93,6%	97,3%	99,6%	99,8%	98,8%
24908	75	40	40	80	93,7%	97,3%	99,6%	99,8%	98,9%
24909	75	40	45	20	90,4%	97,1%	99,4%	99,4%	97,5%
24910	75	40	45	25	90,5%	97,1%	99,4%	99,5%	97,6%
24911	75	40	45	30	90,7%	97,0%	99,4%	99,5%	97,8%
24912	75	40	45	35	90,8%	97,0%	99,4%	99,5%	97,9%
24913	75	40	45	40	90,9%	97,0%	99,4%	99,6%	98,1%
24914	75	40	45	45	91,1%	96,9%	99,5%	99,6%	98,2%

24915	75	40	45	50	91,2%	96,9%	99,5%	99,6%	98,3%
24916	75	40	45	55	91,4%	96,8%	99,5%	99,7%	98,4%
24917	75	40	45	60	91,5%	96,8%	99,5%	99,7%	98,5%
24918	75	40	45	65	91,6%	96,8%	99,5%	99,7%	98,6%
24919	75	40	45	70	91,8%	96,7%	99,5%	99,7%	98,7%
24920	75	40	45	75	91,9%	96,7%	99,6%	99,7%	98,8%
24921	75	40	45	80	92,0%	96,6%	99,6%	99,8%	98,9%
24922	75	40	50	20	88,0%	96,4%	99,3%	99,4%	97,4%
24923	75	40	50	25	88,2%	96,3%	99,3%	99,4%	97,5%
24924	75	40	50	30	88,3%	96,3%	99,3%	99,5%	97,7%
24925	75	40	50	35	88,5%	96,2%	99,3%	99,5%	97,8%
24926	75	40	50	40	88,7%	96,2%	99,4%	99,5%	98,0%
24927	75	40	50	45	88,9%	96,2%	99,4%	99,6%	98,1%
24928	75	40	50	50	89,0%	96,1%	99,4%	99,6%	98,2%
24929	75	40	50	55	89,2%	96,1%	99,4%	99,6%	98,3%
24930	75	40	50	60	89,4%	96,0%	99,4%	99,7%	98,5%
24931	75	40	50	65	89,6%	96,0%	99,5%	99,7%	98,6%
24932	75	40	50	70	89,7%	95,9%	99,5%	99,7%	98,6%
24933	75	40	50	75	89,9%	95,9%	99,5%	99,7%	98,7%
24934	75	40	50	80	90,0%	95,8%	99,5%	99,7%	98,8%
24935	75	40	55	20	85,1%	95,5%	99,2%	99,4%	97,2%
24936	75	40	55	25	85,3%	95,4%	99,2%	99,4%	97,4%
24937	75	40	55	30	85,5%	95,4%	99,2%	99,4%	97,6%
24938	75	40	55	35	85,8%	95,3%	99,3%	99,5%	97,7%
24939	75	40	55	40	86,0%	95,3%	99,3%	99,5%	97,9%
24940	75	40	55	45	86,2%	95,2%	99,3%	99,5%	98,0%
24941	75	40	55	50	86,4%	95,1%	99,3%	99,6%	98,2%
24942	75	40	55	55	86,6%	95,1%	99,4%	99,6%	98,3%
24943	75	40	55	60	86,8%	95,0%	99,4%	99,6%	98,4%
24944	75	40	55	65	87,0%	95,0%	99,4%	99,7%	98,5%
24945	75	40	55	70	87,2%	94,9%	99,4%	99,7%	98,6%
24946	75	40	55	75	87,4%	94,8%	99,4%	99,7%	98,7%
24947	75	40	55	80	87,6%	94,8%	99,5%	99,7%	98,8%

24948	75	40	60	20	81,7%	94,4%	99,1%	99,3%	97,1%
24949	75	40	60	25	82,0%	94,3%	99,1%	99,4%	97,3%
24950	75	40	60	30	82,2%	94,2%	99,1%	99,4%	97,5%
24951	75	40	60	35	82,5%	94,2%	99,2%	99,4%	97,7%
24952	75	40	60	40	82,7%	94,1%	99,2%	99,5%	97,8%
24953	75	40	60	45	83,0%	94,0%	99,2%	99,5%	97,9%
24954	75	40	60	50	83,2%	94,0%	99,2%	99,6%	98,1%
24955	75	40	60	55	83,5%	93,9%	99,3%	99,6%	98,2%
24956	75	40	60	60	83,7%	93,8%	99,3%	99,6%	98,3%
24957	75	40	60	65	84,0%	93,7%	99,3%	99,6%	98,4%
24958	75	40	60	70	84,2%	93,7%	99,3%	99,7%	98,5%
24959	75	40	60	75	84,4%	93,6%	99,4%	99,7%	98,6%
24960	75	40	60	80	84,7%	93,5%	99,4%	99,7%	98,7%
24961	75	40	65	20	77,7%	93,0%	99,0%	99,3%	97,0%
24962	75	40	65	25	78,0%	92,9%	99,0%	99,3%	97,2%
24963	75	40	65	30	78,3%	92,9%	99,0%	99,4%	97,4%
24964	75	40	65	35	78,6%	92,8%	99,1%	99,4%	97,6%
24965	75	40	65	40	78,9%	92,7%	99,1%	99,5%	97,7%
24966	75	40	65	45	79,2%	92,6%	99,1%	99,5%	97,9%
24967	75	40	65	50	79,5%	92,5%	99,1%	99,5%	98,0%
24968	75	40	65	55	79,8%	92,4%	99,2%	99,6%	98,1%
24969	75	40	65	60	80,1%	92,3%	99,2%	99,6%	98,3%
24970	75	40	65	65	80,3%	92,2%	99,2%	99,6%	98,4%
24971	75	40	65	70	80,6%	92,1%	99,3%	99,6%	98,5%
24972	75	40	65	75	80,9%	92,0%	99,3%	99,7%	98,6%
24973	75	40	65	80	81,2%	91,9%	99,3%	99,7%	98,7%
24974	75	40	70	20	73,2%	91,4%	98,8%	99,2%	96,9%
24975	75	40	70	25	73,5%	91,3%	98,9%	99,3%	97,1%
24976	75	40	70	30	73,8%	91,2%	98,9%	99,3%	97,3%
24977	75	40	70	35	74,2%	91,1%	98,9%	99,4%	97,5%
24978	75	40	70	40	74,5%	91,0%	99,0%	99,4%	97,6%
24979	75	40	70	45	74,8%	90,8%	99,0%	99,5%	97,8%
24980	75	40	70	50	75,2%	90,7%	99,0%	99,5%	97,9%

24981	75	40	70	55	75,5%	90,6%	99,1%	99,5%	98,1%
24982	75	40	70	60	75,8%	90,5%	99,1%	99,6%	98,2%
24983	75	40	70	65	76,2%	90,4%	99,1%	99,6%	98,3%
24984	75	40	70	70	76,5%	90,3%	99,2%	99,6%	98,4%
24985	75	40	70	75	76,8%	90,2%	99,2%	99,6%	98,5%
24986	75	40	70	80	77,1%	90,1%	99,2%	99,7%	98,6%
24987	75	40	75	20	68,0%	89,4%	98,7%	99,2%	96,8%
24988	75	40	75	25	68,4%	89,2%	98,7%	99,2%	97,0%
24989	75	40	75	30	68,8%	89,1%	98,8%	99,3%	97,2%
24990	75	40	75	35	69,2%	89,0%	98,8%	99,3%	97,3%
24991	75	40	75	40	69,5%	88,9%	98,8%	99,4%	97,5%
24992	75	40	75	45	69,9%	88,7%	98,9%	99,4%	97,7%
24993	75	40	75	50	70,3%	88,6%	98,9%	99,5%	97,8%
24994	75	40	75	55	70,7%	88,5%	98,9%	99,5%	98,0%
24995	75	40	75	60	71,0%	88,3%	99,0%	99,5%	98,1%
24996	75	40	75	65	71,4%	88,2%	99,0%	99,6%	98,2%
24997	75	40	75	70	71,7%	88,1%	99,0%	99,6%	98,3%
24998	75	40	75	75	72,1%	87,9%	99,1%	99,6%	98,5%
24999	75	40	75	80	72,5%	87,8%	99,1%	99,6%	98,6%
25000	75	40	80	20	62,4%	87,0%	98,5%	99,1%	96,6%
25001	75	40	80	25	62,9%	86,8%	98,5%	99,2%	96,8%
25002	75	40	80	30	63,3%	86,7%	98,6%	99,2%	97,1%
25003	75	40	80	35	63,7%	86,5%	98,6%	99,3%	97,2%
25004	75	40	80	40	64,1%	86,4%	98,7%	99,3%	97,4%
25005	75	40	80	45	64,5%	86,2%	98,7%	99,4%	97,6%
25006	75	40	80	50	64,9%	86,1%	98,8%	99,4%	97,7%
25007	75	40	80	55	65,3%	85,9%	98,8%	99,5%	97,9%
25008	75	40	80	60	65,7%	85,7%	98,8%	99,5%	98,0%
25009	75	40	80	65	66,1%	85,6%	98,9%	99,5%	98,2%
25010	75	40	80	70	66,5%	85,4%	98,9%	99,6%	98,3%
25011	75	40	80	75	66,9%	85,3%	99,0%	99,6%	98,4%
25012	75	40	80	80	67,3%	85,1%	99,0%	99,6%	98,5%
25013	75	45	20	20	97,2%	99,1%	99,6%	99,6%	97,7%

25014	75	45	20	25	97,3%	99,1%	99,7%	99,6%	97,9%
25015	75	45	20	30	97,3%	99,1%	99,7%	99,6%	98,0%
25016	75	45	20	35	97,4%	99,1%	99,7%	99,6%	98,1%
25017	75	45	20	40	97,4%	99,1%	99,7%	99,7%	98,3%
25018	75	45	20	45	97,5%	99,1%	99,7%	99,7%	98,4%
25019	75	45	20	50	97,5%	99,1%	99,7%	99,7%	98,5%
25020	75	45	20	55	97,5%	99,1%	99,7%	99,7%	98,6%
25021	75	45	20	60	97,6%	99,0%	99,7%	99,7%	98,7%
25022	75	45	20	65	97,6%	99,0%	99,7%	99,8%	98,8%
25023	75	45	20	70	97,7%	99,0%	99,7%	99,8%	98,8%
25024	75	45	20	75	97,7%	99,0%	99,8%	99,8%	98,9%
25025	75	45	20	80	97,8%	99,0%	99,8%	99,8%	99,0%
25026	75	45	25	20	96,5%	98,9%	99,6%	99,5%	97,6%
25027	75	45	25	25	96,5%	98,9%	99,6%	99,6%	97,8%
25028	75	45	25	30	96,6%	98,9%	99,6%	99,6%	97,9%
25029	75	45	25	35	96,7%	98,9%	99,6%	99,6%	98,1%
25030	75	45	25	40	96,7%	98,9%	99,6%	99,6%	98,2%
25031	75	45	25	45	96,8%	98,8%	99,7%	99,7%	98,3%
25032	75	45	25	50	96,8%	98,8%	99,7%	99,7%	98,4%
25033	75	45	25	55	96,9%	98,8%	99,7%	99,7%	98,5%
25034	75	45	25	60	96,9%	98,8%	99,7%	99,7%	98,6%
25035	75	45	25	65	97,0%	98,8%	99,7%	99,7%	98,7%
25036	75	45	25	70	97,0%	98,8%	99,7%	99,8%	98,8%
25037	75	45	25	75	97,1%	98,8%	99,7%	99,8%	98,9%
25038	75	45	25	80	97,1%	98,7%	99,7%	99,8%	98,9%
25039	75	45	30	20	95,5%	98,6%	99,5%	99,5%	97,5%
25040	75	45	30	25	95,6%	98,6%	99,6%	99,5%	97,7%
25041	75	45	30	30	95,7%	98,6%	99,6%	99,6%	97,8%
25042	75	45	30	35	95,8%	98,6%	99,6%	99,6%	98,0%
25043	75	45	30	40	95,8%	98,6%	99,6%	99,6%	98,1%
25044	75	45	30	45	95,9%	98,6%	99,6%	99,6%	98,2%
25045	75	45	30	50	96,0%	98,5%	99,6%	99,7%	98,3%
25046	75	45	30	55	96,0%	98,5%	99,6%	99,7%	98,5%

25047	75	45	30	60	96,1%	98,5%	99,7%	99,7%	98,6%
25048	75	45	30	65	96,2%	98,5%	99,7%	99,7%	98,6%
25049	75	45	30	70	96,2%	98,5%	99,7%	99,7%	98,7%
25050	75	45	30	75	96,3%	98,4%	99,7%	99,8%	98,8%
25051	75	45	30	80	96,4%	98,4%	99,7%	99,8%	98,9%
25052	75	45	35	20	94,4%	98,3%	99,5%	99,5%	97,4%
25053	75	45	35	25	94,5%	98,3%	99,5%	99,5%	97,6%
25054	75	45	35	30	94,6%	98,3%	99,5%	99,5%	97,7%
25055	75	45	35	35	94,6%	98,2%	99,5%	99,6%	97,9%
25056	75	45	35	40	94,7%	98,2%	99,6%	99,6%	98,0%
25057	75	45	35	45	94,8%	98,2%	99,6%	99,6%	98,2%
25058	75	45	35	50	94,9%	98,2%	99,6%	99,6%	98,3%
25059	75	45	35	55	95,0%	98,1%	99,6%	99,7%	98,4%
25060	75	45	35	60	95,1%	98,1%	99,6%	99,7%	98,5%
25061	75	45	35	65	95,2%	98,1%	99,6%	99,7%	98,6%
25062	75	45	35	70	95,2%	98,1%	99,6%	99,7%	98,7%
25063	75	45	35	75	95,3%	98,0%	99,6%	99,7%	98,8%
25064	75	45	35	80	95,4%	98,0%	99,7%	99,8%	98,9%
25065	75	45	40	20	92,9%	97,9%	99,4%	99,4%	97,3%
25066	75	45	40	25	93,0%	97,8%	99,4%	99,5%	97,5%
25067	75	45	40	30	93,1%	97,8%	99,5%	99,5%	97,7%
25068	75	45	40	35	93,2%	97,8%	99,5%	99,5%	97,8%
25069	75	45	40	40	93,4%	97,8%	99,5%	99,6%	97,9%
25070	75	45	40	45	93,5%	97,7%	99,5%	99,6%	98,1%
25071	75	45	40	50	93,6%	97,7%	99,5%	99,6%	98,2%
25072	75	45	40	55	93,7%	97,7%	99,5%	99,6%	98,3%
25073	75	45	40	60	93,8%	97,6%	99,6%	99,7%	98,4%
25074	75	45	40	65	93,9%	97,6%	99,6%	99,7%	98,5%
25075	75	45	40	70	94,0%	97,6%	99,6%	99,7%	98,6%
25076	75	45	40	75	94,1%	97,5%	99,6%	99,7%	98,7%
25077	75	45	40	80	94,2%	97,5%	99,6%	99,7%	98,8%
25078	75	45	45	20	91,1%	97,3%	99,3%	99,4%	97,2%
25079	75	45	45	25	91,2%	97,3%	99,4%	99,4%	97,4%

25080	75	45	45	30	91,4%	97,3%	99,4%	99,5%	97,6%
25081	75	45	45	35	91,5%	97,2%	99,4%	99,5%	97,7%
25082	75	45	45	40	91,7%	97,2%	99,4%	99,5%	97,9%
25083	75	45	45	45	91,8%	97,1%	99,4%	99,6%	98,0%
25084	75	45	45	50	91,9%	97,1%	99,5%	99,6%	98,1%
25085	75	45	45	55	92,0%	97,1%	99,5%	99,6%	98,3%
25086	75	45	45	60	92,2%	97,0%	99,5%	99,6%	98,4%
25087	75	45	45	65	92,3%	97,0%	99,5%	99,7%	98,5%
25088	75	45	45	70	92,4%	97,0%	99,5%	99,7%	98,6%
25089	75	45	45	75	92,5%	96,9%	99,5%	99,7%	98,7%
25090	75	45	45	80	92,7%	96,9%	99,6%	99,7%	98,8%
25091	75	45	50	20	88,9%	96,7%	99,3%	99,4%	97,1%
25092	75	45	50	25	89,1%	96,6%	99,3%	99,4%	97,3%
25093	75	45	50	30	89,2%	96,6%	99,3%	99,4%	97,5%
25094	75	45	50	35	89,4%	96,5%	99,3%	99,5%	97,6%
25095	75	45	50	40	89,6%	96,5%	99,3%	99,5%	97,8%
25096	75	45	50	45	89,7%	96,4%	99,4%	99,5%	97,9%
25097	75	45	50	50	89,9%	96,4%	99,4%	99,6%	98,1%
25098	75	45	50	55	90,0%	96,3%	99,4%	99,6%	98,2%
25099	75	45	50	60	90,2%	96,3%	99,4%	99,6%	98,3%
25100	75	45	50	65	90,4%	96,2%	99,4%	99,7%	98,4%
25101	75	45	50	70	90,5%	96,2%	99,5%	99,7%	98,5%
25102	75	45	50	75	90,7%	96,2%	99,5%	99,7%	98,6%
25103	75	45	50	80	90,8%	96,1%	99,5%	99,7%	98,7%
25104	75	45	55	20	86,2%	95,8%	99,2%	99,3%	97,0%
25105	75	45	55	25	86,4%	95,8%	99,2%	99,4%	97,2%
25106	75	45	55	30	86,6%	95,7%	99,2%	99,4%	97,4%
25107	75	45	55	35	86,8%	95,7%	99,2%	99,4%	97,5%
25108	75	45	55	40	87,0%	95,6%	99,3%	99,5%	97,7%
25109	75	45	55	45	87,2%	95,5%	99,3%	99,5%	97,8%
25110	75	45	55	50	87,4%	95,5%	99,3%	99,5%	98,0%
25111	75	45	55	55	87,6%	95,4%	99,3%	99,6%	98,1%
25112	75	45	55	60	87,8%	95,4%	99,4%	99,6%	98,2%

25113	75	45	55	65	88,0%	95,3%	99,4%	99,6%	98,3%
25114	75	45	55	70	88,2%	95,3%	99,4%	99,7%	98,5%
25115	75	45	55	75	88,3%	95,2%	99,4%	99,7%	98,6%
25116	75	45	55	80	88,5%	95,1%	99,4%	99,7%	98,6%
25117	75	45	60	20	83,0%	94,8%	99,0%	99,3%	96,9%
25118	75	45	60	25	83,2%	94,7%	99,1%	99,3%	97,1%
25119	75	45	60	30	83,5%	94,7%	99,1%	99,4%	97,2%
25120	75	45	60	35	83,7%	94,6%	99,1%	99,4%	97,4%
25121	75	45	60	40	84,0%	94,5%	99,2%	99,4%	97,6%
25122	75	45	60	45	84,2%	94,5%	99,2%	99,5%	97,7%
25123	75	45	60	50	84,4%	94,4%	99,2%	99,5%	97,9%
25124	75	45	60	55	84,7%	94,3%	99,2%	99,5%	98,0%
25125	75	45	60	60	84,9%	94,2%	99,3%	99,6%	98,2%
25126	75	45	60	65	85,1%	94,2%	99,3%	99,6%	98,3%
25127	75	45	60	70	85,3%	94,1%	99,3%	99,6%	98,4%
25128	75	45	60	75	85,6%	94,0%	99,3%	99,7%	98,5%
25129	75	45	60	80	85,8%	94,0%	99,4%	99,7%	98,6%
25130	75	45	65	20	79,2%	93,5%	98,9%	99,2%	96,7%
25131	75	45	65	25	79,5%	93,4%	99,0%	99,3%	96,9%
25132	75	45	65	30	79,8%	93,4%	99,0%	99,3%	97,1%
25133	75	45	65	35	80,1%	93,3%	99,0%	99,4%	97,3%
25134	75	45	65	40	80,4%	93,2%	99,1%	99,4%	97,5%
25135	75	45	65	45	80,6%	93,1%	99,1%	99,5%	97,7%
25136	75	45	65	50	80,9%	93,0%	99,1%	99,5%	97,8%
25137	75	45	65	55	81,2%	92,9%	99,1%	99,5%	97,9%
25138	75	45	65	60	81,4%	92,9%	99,2%	99,6%	98,1%
25139	75	45	65	65	81,7%	92,8%	99,2%	99,6%	98,2%
25140	75	45	65	70	82,0%	92,7%	99,2%	99,6%	98,3%
25141	75	45	65	75	82,2%	92,6%	99,3%	99,6%	98,4%
25142	75	45	65	80	82,5%	92,5%	99,3%	99,7%	98,5%
25143	75	45	70	20	74,9%	92,0%	98,8%	99,2%	96,6%
25144	75	45	70	25	75,2%	91,9%	98,8%	99,2%	96,8%
25145	75	45	70	30	75,5%	91,8%	98,9%	99,3%	97,0%

25146	75	45	70	35	75,8%	91,7%	98,9%	99,3%	97,2%
25147	75	45	70	40	76,2%	91,6%	98,9%	99,4%	97,4%
25148	75	45	70	45	76,5%	91,5%	99,0%	99,4%	97,6%
25149	75	45	70	50	76,8%	91,4%	99,0%	99,5%	97,7%
25150	75	45	70	55	77,1%	91,3%	99,0%	99,5%	97,9%
25151	75	45	70	60	77,4%	91,2%	99,1%	99,5%	98,0%
25152	75	45	70	65	77,7%	91,1%	99,1%	99,6%	98,1%
25153	75	45	70	70	78,0%	91,0%	99,1%	99,6%	98,3%
25154	75	45	70	75	78,3%	90,8%	99,2%	99,6%	98,4%
25155	75	45	70	80	78,6%	90,7%	99,2%	99,6%	98,5%
25156	75	45	75	20	69,9%	90,1%	98,6%	99,1%	96,5%
25157	75	45	75	25	70,3%	90,0%	98,7%	99,2%	96,7%
25158	75	45	75	30	70,7%	89,9%	98,7%	99,2%	96,9%
25159	75	45	75	35	71,0%	89,7%	98,8%	99,3%	97,1%
25160	75	45	75	40	71,4%	89,6%	98,8%	99,3%	97,3%
25161	75	45	75	45	71,8%	89,5%	98,8%	99,4%	97,5%
25162	75	45	75	50	72,1%	89,4%	98,9%	99,4%	97,6%
25163	75	45	75	55	72,5%	89,3%	98,9%	99,5%	97,8%
25164	75	45	75	60	72,8%	89,1%	98,9%	99,5%	97,9%
25165	75	45	75	65	73,2%	89,0%	99,0%	99,5%	98,1%
25166	75	45	75	70	73,5%	88,9%	99,0%	99,6%	98,2%
25167	75	45	75	75	73,8%	88,7%	99,0%	99,6%	98,3%
25168	75	45	75	80	74,2%	88,6%	99,1%	99,6%	98,4%
25169	75	45	80	20	64,5%	87,8%	98,4%	99,1%	96,3%
25170	75	45	80	25	64,9%	87,7%	98,5%	99,1%	96,5%
25171	75	45	80	30	65,3%	87,6%	98,5%	99,2%	96,8%
25172	75	45	80	35	65,7%	87,4%	98,6%	99,2%	97,0%
25173	75	45	80	40	66,1%	87,3%	98,6%	99,3%	97,2%
25174	75	45	80	45	66,5%	87,1%	98,7%	99,3%	97,3%
25175	75	45	80	50	66,9%	87,0%	98,7%	99,4%	97,5%
25176	75	45	80	55	67,3%	86,8%	98,8%	99,4%	97,7%
25177	75	45	80	60	67,7%	86,7%	98,8%	99,5%	97,8%
25178	75	45	80	65	68,0%	86,5%	98,8%	99,5%	98,0%

25179	75	45	80	70	68,4%	86,4%	98,9%	99,5%	98,1%
25180	75	45	80	75	68,8%	86,2%	98,9%	99,6%	98,2%
25181	75	45	80	80	69,2%	86,1%	99,0%	99,6%	98,3%
25182	75	50	20	20	97,5%	99,2%	99,6%	99,5%	97,5%
25183	75	50	20	25	97,5%	99,2%	99,6%	99,5%	97,7%
25184	75	50	20	30	97,5%	99,2%	99,7%	99,6%	97,8%
25185	75	50	20	35	97,6%	99,2%	99,7%	99,6%	97,9%
25186	75	50	20	40	97,6%	99,2%	99,7%	99,6%	98,1%
25187	75	50	20	45	97,7%	99,2%	99,7%	99,7%	98,2%
25188	75	50	20	50	97,7%	99,1%	99,7%	99,7%	98,3%
25189	75	50	20	55	97,8%	99,1%	99,7%	99,7%	98,4%
25190	75	50	20	60	97,8%	99,1%	99,7%	99,7%	98,5%
25191	75	50	20	65	97,8%	99,1%	99,7%	99,7%	98,6%
25192	75	50	20	70	97,9%	99,1%	99,7%	99,8%	98,7%
25193	75	50	20	75	97,9%	99,1%	99,7%	99,8%	98,8%
25194	75	50	20	80	97,9%	99,1%	99,8%	99,8%	98,9%
25195	75	50	25	20	96,8%	99,0%	99,6%	99,5%	97,4%
25196	75	50	25	25	96,8%	99,0%	99,6%	99,5%	97,6%
25197	75	50	25	30	96,9%	99,0%	99,6%	99,6%	97,7%
25198	75	50	25	35	96,9%	99,0%	99,6%	99,6%	97,9%
25199	75	50	25	40	97,0%	98,9%	99,6%	99,6%	98,0%
25200	75	50	25	45	97,0%	98,9%	99,6%	99,6%	98,1%
25201	75	50	25	50	97,1%	98,9%	99,7%	99,7%	98,3%
25202	75	50	25	55	97,1%	98,9%	99,7%	99,7%	98,4%
25203	75	50	25	60	97,2%	98,9%	99,7%	99,7%	98,5%
25204	75	50	25	65	97,2%	98,9%	99,7%	99,7%	98,6%
25205	75	50	25	70	97,3%	98,9%	99,7%	99,7%	98,7%
25206	75	50	25	75	97,3%	98,8%	99,7%	99,8%	98,8%
25207	75	50	25	80	97,4%	98,8%	99,7%	99,8%	98,8%
25208	75	50	30	20	95,9%	98,7%	99,5%	99,5%	97,3%
25209	75	50	30	25	96,0%	98,7%	99,5%	99,5%	97,5%
25210	75	50	30	30	96,0%	98,7%	99,6%	99,5%	97,6%
25211	75	50	30	35	96,1%	98,7%	99,6%	99,6%	97,8%

25212	75	50	30	40	96,2%	98,7%	99,6%	99,6%	97,9%
25213	75	50	30	45	96,2%	98,7%	99,6%	99,6%	98,1%
25214	75	50	30	50	96,3%	98,6%	99,6%	99,6%	98,2%
25215	75	50	30	55	96,4%	98,6%	99,6%	99,7%	98,3%
25216	75	50	30	60	96,4%	98,6%	99,6%	99,7%	98,4%
25217	75	50	30	65	96,5%	98,6%	99,7%	99,7%	98,5%
25218	75	50	30	70	96,5%	98,6%	99,7%	99,7%	98,6%
25219	75	50	30	75	96,6%	98,6%	99,7%	99,7%	98,7%
25220	75	50	30	80	96,7%	98,5%	99,7%	99,8%	98,8%
25221	75	50	35	20	94,8%	98,4%	99,5%	99,4%	97,2%
25222	75	50	35	25	94,9%	98,4%	99,5%	99,5%	97,4%
25223	75	50	35	30	95,0%	98,4%	99,5%	99,5%	97,5%
25224	75	50	35	35	95,1%	98,4%	99,5%	99,5%	97,7%
25225	75	50	35	40	95,2%	98,3%	99,5%	99,6%	97,8%
25226	75	50	35	45	95,2%	98,3%	99,6%	99,6%	98,0%
25227	75	50	35	50	95,3%	98,3%	99,6%	99,6%	98,1%
25228	75	50	35	55	95,4%	98,3%	99,6%	99,6%	98,2%
25229	75	50	35	60	95,5%	98,3%	99,6%	99,7%	98,3%
25230	75	50	35	65	95,6%	98,2%	99,6%	99,7%	98,5%
25231	75	50	35	70	95,6%	98,2%	99,6%	99,7%	98,6%
25232	75	50	35	75	95,7%	98,2%	99,6%	99,7%	98,6%
25233	75	50	35	80	95,8%	98,2%	99,6%	99,7%	98,7%
25234	75	50	40	20	93,5%	98,0%	99,4%	99,4%	97,1%
25235	75	50	40	25	93,6%	98,0%	99,4%	99,4%	97,2%
25236	75	50	40	30	93,7%	98,0%	99,4%	99,5%	97,4%
25237	75	50	40	35	93,8%	97,9%	99,5%	99,5%	97,6%
25238	75	50	40	40	93,9%	97,9%	99,5%	99,5%	97,7%
25239	75	50	40	45	94,0%	97,9%	99,5%	99,6%	97,9%
25240	75	50	40	50	94,1%	97,9%	99,5%	99,6%	98,0%
25241	75	50	40	55	94,2%	97,8%	99,5%	99,6%	98,2%
25242	75	50	40	60	94,3%	97,8%	99,5%	99,6%	98,3%
25243	75	50	40	65	94,4%	97,8%	99,6%	99,7%	98,4%
25244	75	50	40	70	94,5%	97,8%	99,6%	99,7%	98,5%

25245	75	50	40	75	94,6%	97,7%	99,6%	99,7%	98,6%
25246	75	50	40	80	94,6%	97,7%	99,6%	99,7%	98,7%
25247	75	50	45	20	91,8%	97,5%	99,3%	99,3%	96,9%
25248	75	50	45	25	91,9%	97,5%	99,3%	99,4%	97,1%
25249	75	50	45	30	92,1%	97,5%	99,4%	99,4%	97,3%
25250	75	50	45	35	92,2%	97,4%	99,4%	99,5%	97,5%
25251	75	50	45	40	92,3%	97,4%	99,4%	99,5%	97,7%
25252	75	50	45	45	92,4%	97,4%	99,4%	99,5%	97,8%
25253	75	50	45	50	92,6%	97,3%	99,4%	99,6%	97,9%
25254	75	50	45	55	92,7%	97,3%	99,5%	99,6%	98,1%
25255	75	50	45	60	92,8%	97,3%	99,5%	99,6%	98,2%
25256	75	50	45	65	92,9%	97,2%	99,5%	99,6%	98,3%
25257	75	50	45	70	93,0%	97,2%	99,5%	99,7%	98,4%
25258	75	50	45	75	93,1%	97,1%	99,5%	99,7%	98,5%
25259	75	50	45	80	93,3%	97,1%	99,5%	99,7%	98,6%
25260	75	50	50	20	89,7%	96,9%	99,2%	99,3%	96,8%
25261	75	50	50	25	89,9%	96,9%	99,3%	99,4%	97,0%
25262	75	50	50	30	90,0%	96,8%	99,3%	99,4%	97,2%
25263	75	50	50	35	90,2%	96,8%	99,3%	99,4%	97,4%
25264	75	50	50	40	90,4%	96,7%	99,3%	99,5%	97,6%
25265	75	50	50	45	90,5%	96,7%	99,4%	99,5%	97,7%
25266	75	50	50	50	90,7%	96,7%	99,4%	99,5%	97,9%
25267	75	50	50	55	90,8%	96,6%	99,4%	99,6%	98,0%
25268	75	50	50	60	91,0%	96,6%	99,4%	99,6%	98,1%
25269	75	50	50	65	91,1%	96,5%	99,4%	99,6%	98,3%
25270	75	50	50	70	91,2%	96,5%	99,5%	99,7%	98,4%
25271	75	50	50	75	91,4%	96,4%	99,5%	99,7%	98,5%
25272	75	50	50	80	91,5%	96,4%	99,5%	99,7%	98,6%
25273	75	50	55	20	87,2%	96,1%	99,1%	99,3%	96,7%
25274	75	50	55	25	87,4%	96,1%	99,2%	99,3%	96,9%
25275	75	50	55	30	87,6%	96,0%	99,2%	99,4%	97,1%
25276	75	50	55	35	87,8%	96,0%	99,2%	99,4%	97,3%
25277	75	50	55	40	88,0%	95,9%	99,2%	99,4%	97,5%

25278	75	50	55	45	88,2%	95,9%	99,3%	99,5%	97,6%
25279	75	50	55	50	88,4%	95,8%	99,3%	99,5%	97,8%
25280	75	50	55	55	88,5%	95,8%	99,3%	99,5%	97,9%
25281	75	50	55	60	88,7%	95,7%	99,3%	99,6%	98,1%
25282	75	50	55	65	88,9%	95,7%	99,4%	99,6%	98,2%
25283	75	50	55	70	89,1%	95,6%	99,4%	99,6%	98,3%
25284	75	50	55	75	89,2%	95,6%	99,4%	99,7%	98,4%
25285	75	50	55	80	89,4%	95,5%	99,4%	99,7%	98,5%
25286	75	50	60	20	84,2%	95,2%	99,0%	99,2%	96,5%
25287	75	50	60	25	84,4%	95,1%	99,0%	99,3%	96,8%
25288	75	50	60	30	84,7%	95,0%	99,1%	99,3%	97,0%
25289	75	50	60	35	84,9%	95,0%	99,1%	99,4%	97,2%
25290	75	50	60	40	85,1%	94,9%	99,1%	99,4%	97,4%
25291	75	50	60	45	85,3%	94,9%	99,2%	99,4%	97,5%
25292	75	50	60	50	85,6%	94,8%	99,2%	99,5%	97,7%
25293	75	50	60	55	85,8%	94,7%	99,2%	99,5%	97,8%
25294	75	50	60	60	86,0%	94,7%	99,2%	99,5%	98,0%
25295	75	50	60	65	86,2%	94,6%	99,3%	99,6%	98,1%
25296	75	50	60	70	86,4%	94,5%	99,3%	99,6%	98,2%
25297	75	50	60	75	86,6%	94,5%	99,3%	99,6%	98,3%
25298	75	50	60	80	86,8%	94,4%	99,3%	99,7%	98,5%
25299	75	50	65	20	80,6%	94,0%	98,9%	99,2%	96,4%
25300	75	50	65	25	80,9%	93,9%	98,9%	99,2%	96,6%
25301	75	50	65	30	81,2%	93,8%	99,0%	99,3%	96,9%
25302	75	50	65	35	81,5%	93,8%	99,0%	99,3%	97,1%
25303	75	50	65	40	81,7%	93,7%	99,0%	99,4%	97,2%
25304	75	50	65	45	82,0%	93,6%	99,1%	99,4%	97,4%
25305	75	50	65	50	82,2%	93,5%	99,1%	99,4%	97,6%
25306	75	50	65	55	82,5%	93,4%	99,1%	99,5%	97,7%
25307	75	50	65	60	82,7%	93,4%	99,1%	99,5%	97,9%
25308	75	50	65	65	83,0%	93,3%	99,2%	99,6%	98,0%
25309	75	50	65	70	83,2%	93,2%	99,2%	99,6%	98,2%
25310	75	50	65	75	83,5%	93,1%	99,2%	99,6%	98,3%

25311	75	50	65	80	83,7%	93,0%	99,3%	99,6%	98,4%
25312	75	50	70	20	76,5%	92,5%	98,7%	99,1%	96,3%
25313	75	50	70	25	76,8%	92,4%	98,8%	99,2%	96,5%
25314	75	50	70	30	77,1%	92,4%	98,8%	99,2%	96,7%
25315	75	50	70	35	77,4%	92,3%	98,9%	99,3%	96,9%
25316	75	50	70	40	77,7%	92,2%	98,9%	99,3%	97,1%
25317	75	50	70	45	78,0%	92,1%	98,9%	99,4%	97,3%
25318	75	50	70	50	78,3%	92,0%	99,0%	99,4%	97,5%
25319	75	50	70	55	78,6%	91,9%	99,0%	99,5%	97,7%
25320	75	50	70	60	78,9%	91,8%	99,0%	99,5%	97,8%
25321	75	50	70	65	79,2%	91,7%	99,1%	99,5%	97,9%
25322	75	50	70	70	79,5%	91,6%	99,1%	99,6%	98,1%
25323	75	50	70	75	79,8%	91,5%	99,1%	99,6%	98,2%
25324	75	50	70	80	80,1%	91,4%	99,2%	99,6%	98,3%
25325	75	50	75	20	71,8%	90,8%	98,6%	99,1%	96,1%
25326	75	50	75	25	72,1%	90,7%	98,6%	99,1%	96,4%
25327	75	50	75	30	72,5%	90,6%	98,7%	99,2%	96,6%
25328	75	50	75	35	72,8%	90,4%	98,7%	99,2%	96,8%
25329	75	50	75	40	73,2%	90,3%	98,8%	99,3%	97,0%
25330	75	50	75	45	73,5%	90,2%	98,8%	99,3%	97,2%
25331	75	50	75	50	73,9%	90,1%	98,8%	99,4%	97,4%
25332	75	50	75	55	74,2%	90,0%	98,9%	99,4%	97,6%
25333	75	50	75	60	74,5%	89,9%	98,9%	99,5%	97,7%
25334	75	50	75	65	74,9%	89,7%	98,9%	99,5%	97,9%
25335	75	50	75	70	75,2%	89,6%	99,0%	99,5%	98,0%
25336	75	50	75	75	75,5%	89,5%	99,0%	99,6%	98,1%
25337	75	50	75	80	75,8%	89,4%	99,0%	99,6%	98,3%
25338	75	50	80	20	66,5%	88,7%	98,4%	99,0%	96,0%
25339	75	50	80	25	66,9%	88,5%	98,4%	99,1%	96,2%
25340	75	50	80	30	67,3%	88,4%	98,5%	99,1%	96,5%
25341	75	50	80	35	67,7%	88,3%	98,5%	99,2%	96,7%
25342	75	50	80	40	68,1%	88,1%	98,6%	99,2%	96,9%
25343	75	50	80	45	68,4%	88,0%	98,6%	99,3%	97,1%

25344	75	50	80	50	68,8%	87,8%	98,7%	99,3%	97,3%
25345	75	50	80	55	69,2%	87,7%	98,7%	99,4%	97,5%
25346	75	50	80	60	69,6%	87,6%	98,8%	99,4%	97,6%
25347	75	50	80	65	69,9%	87,4%	98,8%	99,5%	97,8%
25348	75	50	80	70	70,3%	87,3%	98,9%	99,5%	97,9%
25349	75	50	80	75	70,7%	87,1%	98,9%	99,5%	98,1%
25350	75	50	80	80	71,0%	87,0%	98,9%	99,6%	98,2%
25351	75	55	20	20	97,7%	99,3%	99,6%	99,5%	97,2%
25352	75	55	20	25	97,7%	99,3%	99,6%	99,5%	97,4%
25353	75	55	20	30	97,8%	99,2%	99,6%	99,5%	97,6%
25354	75	55	20	35	97,8%	99,2%	99,7%	99,6%	97,7%
25355	75	55	20	40	97,8%	99,2%	99,7%	99,6%	97,9%
25356	75	55	20	45	97,9%	99,2%	99,7%	99,6%	98,0%
25357	75	55	20	50	97,9%	99,2%	99,7%	99,7%	98,2%
25358	75	55	20	55	97,9%	99,2%	99,7%	99,7%	98,3%
25359	75	55	20	60	98,0%	99,2%	99,7%	99,7%	98,4%
25360	75	55	20	65	98,0%	99,2%	99,7%	99,7%	98,5%
25361	75	55	20	70	98,0%	99,2%	99,7%	99,7%	98,6%
25362	75	55	20	75	98,1%	99,2%	99,7%	99,8%	98,7%
25363	75	55	20	80	98,1%	99,1%	99,7%	99,8%	98,8%
25364	75	55	25	20	97,0%	99,1%	99,6%	99,4%	97,1%
25365	75	55	25	25	97,1%	99,1%	99,6%	99,5%	97,3%
25366	75	55	25	30	97,1%	99,1%	99,6%	99,5%	97,5%
25367	75	55	25	35	97,2%	99,0%	99,6%	99,6%	97,7%
25368	75	55	25	40	97,2%	99,0%	99,6%	99,6%	97,8%
25369	75	55	25	45	97,3%	99,0%	99,6%	99,6%	97,9%
25370	75	55	25	50	97,3%	99,0%	99,7%	99,6%	98,1%
25371	75	55	25	55	97,4%	99,0%	99,7%	99,7%	98,2%
25372	75	55	25	60	97,4%	99,0%	99,7%	99,7%	98,3%
25373	75	55	25	65	97,5%	99,0%	99,7%	99,7%	98,4%
25374	75	55	25	70	97,5%	98,9%	99,7%	99,7%	98,5%
25375	75	55	25	75	97,6%	98,9%	99,7%	99,7%	98,6%
25376	75	55	25	80	97,6%	98,9%	99,7%	99,8%	98,7%

25377	75	55	30	20	96,2%	98,8%	99,5%	99,4%	97,0%
25378	75	55	30	25	96,3%	98,8%	99,5%	99,5%	97,2%
25379	75	55	30	30	96,4%	98,8%	99,5%	99,5%	97,4%
25380	75	55	30	35	96,4%	98,8%	99,6%	99,5%	97,6%
25381	75	55	30	40	96,5%	98,8%	99,6%	99,6%	97,7%
25382	75	55	30	45	96,6%	98,8%	99,6%	99,6%	97,9%
25383	75	55	30	50	96,6%	98,7%	99,6%	99,6%	98,0%
25384	75	55	30	55	96,7%	98,7%	99,6%	99,6%	98,1%
25385	75	55	30	60	96,7%	98,7%	99,6%	99,7%	98,3%
25386	75	55	30	65	96,8%	98,7%	99,6%	99,7%	98,4%
25387	75	55	30	70	96,8%	98,7%	99,7%	99,7%	98,5%
25388	75	55	30	75	96,9%	98,7%	99,7%	99,7%	98,6%
25389	75	55	30	80	96,9%	98,6%	99,7%	99,7%	98,7%
25390	75	55	35	20	95,2%	98,5%	99,5%	99,4%	96,9%
25391	75	55	35	25	95,3%	98,5%	99,5%	99,4%	97,1%
25392	75	55	35	30	95,4%	98,5%	99,5%	99,5%	97,3%
25393	75	55	35	35	95,5%	98,5%	99,5%	99,5%	97,5%
25394	75	55	35	40	95,6%	98,5%	99,5%	99,5%	97,6%
25395	75	55	35	45	95,6%	98,4%	99,5%	99,6%	97,8%
25396	75	55	35	50	95,7%	98,4%	99,6%	99,6%	97,9%
25397	75	55	35	55	95,8%	98,4%	99,6%	99,6%	98,1%
25398	75	55	35	60	95,8%	98,4%	99,6%	99,6%	98,2%
25399	75	55	35	65	95,9%	98,4%	99,6%	99,7%	98,3%
25400	75	55	35	70	96,0%	98,3%	99,6%	99,7%	98,4%
25401	75	55	35	75	96,0%	98,3%	99,6%	99,7%	98,5%
25402	75	55	35	80	96,1%	98,3%	99,6%	99,7%	98,6%
25403	75	55	40	20	94,0%	98,2%	99,4%	99,3%	96,8%
25404	75	55	40	25	94,1%	98,1%	99,4%	99,4%	97,0%
25405	75	55	40	30	94,2%	98,1%	99,4%	99,4%	97,2%
25406	75	55	40	35	94,3%	98,1%	99,4%	99,5%	97,4%
25407	75	55	40	40	94,4%	98,1%	99,5%	99,5%	97,5%
25408	75	55	40	45	94,5%	98,0%	99,5%	99,5%	97,7%
25409	75	55	40	50	94,6%	98,0%	99,5%	99,6%	97,8%

25410	75	55	40	55	94,7%	98,0%	99,5%	99,6%	98,0%
25411	75	55	40	60	94,7%	98,0%	99,5%	99,6%	98,1%
25412	75	55	40	65	94,8%	97,9%	99,5%	99,6%	98,2%
25413	75	55	40	70	94,9%	97,9%	99,6%	99,7%	98,3%
25414	75	55	40	75	95,0%	97,9%	99,6%	99,7%	98,5%
25415	75	55	40	80	95,1%	97,9%	99,6%	99,7%	98,6%
25416	75	55	45	20	92,4%	97,7%	99,3%	99,3%	96,6%
25417	75	55	45	25	92,6%	97,7%	99,3%	99,3%	96,9%
25418	75	55	45	30	92,7%	97,6%	99,3%	99,4%	97,1%
25419	75	55	45	35	92,8%	97,6%	99,4%	99,4%	97,2%
25420	75	55	45	40	92,9%	97,6%	99,4%	99,5%	97,4%
25421	75	55	45	45	93,0%	97,6%	99,4%	99,5%	97,6%
25422	75	55	45	50	93,1%	97,5%	99,4%	99,5%	97,7%
25423	75	55	45	55	93,3%	97,5%	99,4%	99,6%	97,9%
25424	75	55	45	60	93,4%	97,5%	99,5%	99,6%	98,0%
25425	75	55	45	65	93,5%	97,4%	99,5%	99,6%	98,2%
25426	75	55	45	70	93,6%	97,4%	99,5%	99,6%	98,3%
25427	75	55	45	75	93,7%	97,4%	99,5%	99,7%	98,4%
25428	75	55	45	80	93,8%	97,3%	99,5%	99,7%	98,5%
25429	75	55	50	20	90,5%	97,1%	99,2%	99,3%	96,5%
25430	75	55	50	25	90,7%	97,1%	99,2%	99,3%	96,7%
25431	75	55	50	30	90,8%	97,1%	99,3%	99,3%	96,9%
25432	75	55	50	35	91,0%	97,0%	99,3%	99,4%	97,1%
25433	75	55	50	40	91,1%	97,0%	99,3%	99,4%	97,3%
25434	75	55	50	45	91,2%	96,9%	99,3%	99,5%	97,5%
25435	75	55	50	50	91,4%	96,9%	99,4%	99,5%	97,7%
25436	75	55	50	55	91,5%	96,9%	99,4%	99,5%	97,8%
25437	75	55	50	60	91,7%	96,8%	99,4%	99,6%	97,9%
25438	75	55	50	65	91,8%	96,8%	99,4%	99,6%	98,1%
25439	75	55	50	70	91,9%	96,7%	99,4%	99,6%	98,2%
25440	75	55	50	75	92,1%	96,7%	99,5%	99,6%	98,3%
25441	75	55	50	80	92,2%	96,7%	99,5%	99,7%	98,4%
25442	75	55	55	20	88,2%	96,4%	99,1%	99,2%	96,4%

25443	75	55	55	25	88,4%	96,4%	99,1%	99,3%	96,6%
25444	75	55	55	30	88,5%	96,3%	99,2%	99,3%	96,8%
25445	75	55	55	35	88,7%	96,3%	99,2%	99,4%	97,0%
25446	75	55	55	40	88,9%	96,2%	99,2%	99,4%	97,2%
25447	75	55	55	45	89,1%	96,2%	99,2%	99,4%	97,4%
25448	75	55	55	50	89,2%	96,1%	99,3%	99,5%	97,6%
25449	75	55	55	55	89,4%	96,1%	99,3%	99,5%	97,7%
25450	75	55	55	60	89,6%	96,0%	99,3%	99,5%	97,9%
25451	75	55	55	65	89,7%	96,0%	99,3%	99,6%	98,0%
25452	75	55	55	70	89,9%	95,9%	99,4%	99,6%	98,1%
25453	75	55	55	75	90,1%	95,9%	99,4%	99,6%	98,3%
25454	75	55	55	80	90,2%	95,8%	99,4%	99,7%	98,4%
25455	75	55	60	20	85,3%	95,5%	99,0%	99,2%	96,2%
25456	75	55	60	25	85,6%	95,5%	99,0%	99,2%	96,5%
25457	75	55	60	30	85,8%	95,4%	99,1%	99,3%	96,7%
25458	75	55	60	35	86,0%	95,3%	99,1%	99,3%	96,9%
25459	75	55	60	40	86,2%	95,3%	99,1%	99,4%	97,1%
25460	75	55	60	45	86,4%	95,2%	99,1%	99,4%	97,3%
25461	75	55	60	50	86,6%	95,2%	99,2%	99,4%	97,5%
25462	75	55	60	55	86,8%	95,1%	99,2%	99,5%	97,6%
25463	75	55	60	60	87,0%	95,0%	99,2%	99,5%	97,8%
25464	75	55	60	65	87,2%	95,0%	99,2%	99,5%	97,9%
25465	75	55	60	70	87,4%	94,9%	99,3%	99,6%	98,1%
25466	75	55	60	75	87,6%	94,9%	99,3%	99,6%	98,2%
25467	75	55	60	80	87,8%	94,8%	99,3%	99,6%	98,3%
25468	75	55	65	20	82,0%	94,4%	98,9%	99,1%	96,1%
25469	75	55	65	25	82,2%	94,3%	98,9%	99,2%	96,3%
25470	75	55	65	30	82,5%	94,3%	98,9%	99,2%	96,5%
25471	75	55	65	35	82,8%	94,2%	99,0%	99,3%	96,8%
25472	75	55	65	40	83,0%	94,1%	99,0%	99,3%	97,0%
25473	75	55	65	45	83,3%	94,1%	99,0%	99,4%	97,2%
25474	75	55	65	50	83,5%	94,0%	99,1%	99,4%	97,4%
25475	75	55	65	55	83,7%	93,9%	99,1%	99,4%	97,5%

25476	75	55	65	60	84,0%	93,8%	99,1%	99,5%	97,7%
25477	75	55	65	65	84,2%	93,8%	99,2%	99,5%	97,8%
25478	75	55	65	70	84,4%	93,7%	99,2%	99,5%	98,0%
25479	75	55	65	75	84,7%	93,6%	99,2%	99,6%	98,1%
25480	75	55	65	80	84,9%	93,5%	99,2%	99,6%	98,2%
25481	75	55	70	20	78,0%	93,1%	98,7%	99,0%	95,9%
25482	75	55	70	25	78,3%	93,0%	98,7%	99,1%	96,2%
25483	75	55	70	30	78,6%	92,9%	98,8%	99,2%	96,4%
25484	75	55	70	35	78,9%	92,8%	98,8%	99,2%	96,6%
25485	75	55	70	40	79,2%	92,7%	98,9%	99,3%	96,9%
25486	75	55	70	45	79,5%	92,6%	98,9%	99,3%	97,1%
25487	75	55	70	50	79,8%	92,5%	98,9%	99,4%	97,2%
25488	75	55	70	55	80,1%	92,4%	99,0%	99,4%	97,4%
25489	75	55	70	60	80,4%	92,4%	99,0%	99,5%	97,6%
25490	75	55	70	65	80,6%	92,3%	99,0%	99,5%	97,7%
25491	75	55	70	70	80,9%	92,2%	99,1%	99,5%	97,9%
25492	75	55	70	75	81,2%	92,1%	99,1%	99,6%	98,0%
25493	75	55	70	80	81,5%	92,0%	99,1%	99,6%	98,2%
25494	75	55	75	20	73,5%	91,4%	98,5%	99,0%	95,7%
25495	75	55	75	25	73,9%	91,3%	98,6%	99,1%	96,0%
25496	75	55	75	30	74,2%	91,2%	98,6%	99,1%	96,3%
25497	75	55	75	35	74,5%	91,1%	98,7%	99,2%	96,5%
25498	75	55	75	40	74,9%	91,0%	98,7%	99,2%	96,7%
25499	75	55	75	45	75,2%	90,9%	98,8%	99,3%	96,9%
25500	75	55	75	50	75,5%	90,8%	98,8%	99,3%	97,1%
25501	75	55	75	55	75,9%	90,7%	98,8%	99,4%	97,3%
25502	75	55	75	60	76,2%	90,6%	98,9%	99,4%	97,5%
25503	75	55	75	65	76,5%	90,4%	98,9%	99,5%	97,7%
25504	75	55	75	70	76,8%	90,3%	99,0%	99,5%	97,8%
25505	75	55	75	75	77,1%	90,2%	99,0%	99,5%	97,9%
25506	75	55	75	80	77,4%	90,1%	99,0%	99,6%	98,1%
25507	75	55	80	20	68,5%	89,4%	98,3%	98,9%	95,6%
25508	75	55	80	25	68,8%	89,3%	98,4%	99,0%	95,8%

25509	75	55	80	30	69,2%	89,2%	98,4%	99,1%	96,1%
25510	75	55	80	35	69,6%	89,0%	98,5%	99,1%	96,4%
25511	75	55	80	40	70,0%	88,9%	98,5%	99,2%	96,6%
25512	75	55	80	45	70,3%	88,8%	98,6%	99,2%	96,8%
25513	75	55	80	50	70,7%	88,7%	98,6%	99,3%	97,0%
25514	75	55	80	55	71,1%	88,5%	98,7%	99,3%	97,2%
25515	75	55	80	60	71,4%	88,4%	98,7%	99,4%	97,4%
25516	75	55	80	65	71,8%	88,3%	98,8%	99,4%	97,6%
25517	75	55	80	70	72,1%	88,1%	98,8%	99,5%	97,7%
25518	75	55	80	75	72,5%	88,0%	98,9%	99,5%	97,9%
25519	75	55	80	80	72,8%	87,8%	98,9%	99,5%	98,0%
25520	75	60	20	20	97,9%	99,3%	99,6%	99,4%	97,0%
25521	75	60	20	25	97,9%	99,3%	99,6%	99,5%	97,2%
25522	75	60	20	30	97,9%	99,3%	99,6%	99,5%	97,4%
25523	75	60	20	35	98,0%	99,3%	99,6%	99,5%	97,5%
25524	75	60	20	40	98,0%	99,3%	99,7%	99,6%	97,7%
25525	75	60	20	45	98,0%	99,3%	99,7%	99,6%	97,8%
25526	75	60	20	50	98,1%	99,3%	99,7%	99,6%	98,0%
25527	75	60	20	55	98,1%	99,3%	99,7%	99,7%	98,1%
25528	75	60	20	60	98,1%	99,2%	99,7%	99,7%	98,2%
25529	75	60	20	65	98,2%	99,2%	99,7%	99,7%	98,3%
25530	75	60	20	70	98,2%	99,2%	99,7%	99,7%	98,5%
25531	75	60	20	75	98,2%	99,2%	99,7%	99,7%	98,6%
25532	75	60	20	80	98,3%	99,2%	99,7%	99,8%	98,7%
25533	75	60	25	20	97,3%	99,1%	99,6%	99,4%	96,9%
25534	75	60	25	25	97,3%	99,1%	99,6%	99,4%	97,1%
25535	75	60	25	30	97,4%	99,1%	99,6%	99,5%	97,2%
25536	75	60	25	35	97,4%	99,1%	99,6%	99,5%	97,4%
25537	75	60	25	40	97,5%	99,1%	99,6%	99,5%	97,6%
25538	75	60	25	45	97,5%	99,1%	99,6%	99,6%	97,7%
25539	75	60	25	50	97,6%	99,1%	99,6%	99,6%	97,9%
25540	75	60	25	55	97,6%	99,1%	99,7%	99,6%	98,0%
25541	75	60	25	60	97,6%	99,1%	99,7%	99,7%	98,2%

25542	75	60	25	65	97,7%	99,0%	99,7%	99,7%	98,3%
25543	75	60	25	70	97,7%	99,0%	99,7%	99,7%	98,4%
25544	75	60	25	75	97,8%	99,0%	99,7%	99,7%	98,5%
25545	75	60	25	80	97,8%	99,0%	99,7%	99,7%	98,6%
25546	75	60	30	20	96,6%	98,9%	99,5%	99,4%	96,7%
25547	75	60	30	25	96,6%	98,9%	99,5%	99,4%	96,9%
25548	75	60	30	30	96,7%	98,9%	99,5%	99,4%	97,1%
25549	75	60	30	35	96,7%	98,9%	99,5%	99,5%	97,3%
25550	75	60	30	40	96,8%	98,9%	99,6%	99,5%	97,5%
25551	75	60	30	45	96,8%	98,9%	99,6%	99,6%	97,7%
25552	75	60	30	50	96,9%	98,8%	99,6%	99,6%	97,8%
25553	75	60	30	55	96,9%	98,8%	99,6%	99,6%	98,0%
25554	75	60	30	60	97,0%	98,8%	99,6%	99,6%	98,1%
25555	75	60	30	65	97,0%	98,8%	99,6%	99,7%	98,2%
25556	75	60	30	70	97,1%	98,8%	99,6%	99,7%	98,3%
25557	75	60	30	75	97,1%	98,8%	99,7%	99,7%	98,4%
25558	75	60	30	80	97,2%	98,7%	99,7%	99,7%	98,5%
25559	75	60	35	20	95,6%	98,7%	99,4%	99,3%	96,6%
25560	75	60	35	25	95,7%	98,6%	99,5%	99,4%	96,8%
25561	75	60	35	30	95,8%	98,6%	99,5%	99,4%	97,0%
25562	75	60	35	35	95,8%	98,6%	99,5%	99,5%	97,2%
25563	75	60	35	40	95,9%	98,6%	99,5%	99,5%	97,4%
25564	75	60	35	45	96,0%	98,6%	99,5%	99,5%	97,6%
25565	75	60	35	50	96,1%	98,5%	99,5%	99,6%	97,7%
25566	75	60	35	55	96,1%	98,5%	99,6%	99,6%	97,9%
25567	75	60	35	60	96,2%	98,5%	99,6%	99,6%	98,0%
25568	75	60	35	65	96,2%	98,5%	99,6%	99,6%	98,1%
25569	75	60	35	70	96,3%	98,5%	99,6%	99,7%	98,3%
25570	75	60	35	75	96,4%	98,4%	99,6%	99,7%	98,4%
25571	75	60	35	80	96,4%	98,4%	99,6%	99,7%	98,5%
25572	75	60	40	20	94,5%	98,3%	99,4%	99,3%	96,5%
25573	75	60	40	25	94,6%	98,3%	99,4%	99,3%	96,7%
25574	75	60	40	30	94,7%	98,3%	99,4%	99,4%	96,9%

25575	75	60	40	35	94,7%	98,2%	99,4%	99,4%	97,1%
25576	75	60	40	40	94,8%	98,2%	99,4%	99,5%	97,3%
25577	75	60	40	45	94,9%	98,2%	99,5%	99,5%	97,5%
25578	75	60	40	50	95,0%	98,2%	99,5%	99,5%	97,6%
25579	75	60	40	55	95,1%	98,1%	99,5%	99,6%	97,8%
25580	75	60	40	60	95,2%	98,1%	99,5%	99,6%	97,9%
25581	75	60	40	65	95,2%	98,1%	99,5%	99,6%	98,1%
25582	75	60	40	70	95,3%	98,1%	99,5%	99,6%	98,2%
25583	75	60	40	75	95,4%	98,0%	99,6%	99,7%	98,3%
25584	75	60	40	80	95,5%	98,0%	99,6%	99,7%	98,4%
25585	75	60	45	20	93,0%	97,9%	99,3%	99,2%	96,3%
25586	75	60	45	25	93,1%	97,8%	99,3%	99,3%	96,6%
25587	75	60	45	30	93,3%	97,8%	99,3%	99,3%	96,8%
25588	75	60	45	35	93,4%	97,8%	99,3%	99,4%	97,0%
25589	75	60	45	40	93,5%	97,8%	99,4%	99,4%	97,2%
25590	75	60	45	45	93,6%	97,7%	99,4%	99,5%	97,4%
25591	75	60	45	50	93,7%	97,7%	99,4%	99,5%	97,5%
25592	75	60	45	55	93,8%	97,7%	99,4%	99,5%	97,7%
25593	75	60	45	60	93,9%	97,6%	99,4%	99,6%	97,8%
25594	75	60	45	65	94,0%	97,6%	99,5%	99,6%	98,0%
25595	75	60	45	70	94,1%	97,6%	99,5%	99,6%	98,1%
25596	75	60	45	75	94,2%	97,6%	99,5%	99,6%	98,2%
25597	75	60	45	80	94,3%	97,5%	99,5%	99,7%	98,3%
25598	75	60	50	20	91,3%	97,3%	99,2%	99,2%	96,2%
25599	75	60	50	25	91,4%	97,3%	99,2%	99,2%	96,4%
25600	75	60	50	30	91,5%	97,3%	99,2%	99,3%	96,6%
25601	75	60	50	35	91,7%	97,2%	99,3%	99,3%	96,9%
25602	75	60	50	40	91,8%	97,2%	99,3%	99,4%	97,1%
25603	75	60	50	45	91,9%	97,2%	99,3%	99,4%	97,2%
25604	75	60	50	50	92,1%	97,1%	99,3%	99,5%	97,4%
25605	75	60	50	55	92,2%	97,1%	99,4%	99,5%	97,6%
25606	75	60	50	60	92,3%	97,1%	99,4%	99,5%	97,7%
25607	75	60	50	65	92,4%	97,0%	99,4%	99,6%	97,9%

25608	75	60	50	70	92,6%	97,0%	99,4%	99,6%	98,0%
25609	75	60	50	75	92,7%	96,9%	99,4%	99,6%	98,2%
25610	75	60	50	80	92,8%	96,9%	99,5%	99,6%	98,3%
25611	75	60	55	20	89,1%	96,7%	99,1%	99,1%	96,0%
25612	75	60	55	25	89,2%	96,6%	99,1%	99,2%	96,3%
25613	75	60	55	30	89,4%	96,6%	99,1%	99,3%	96,5%
25614	75	60	55	35	89,6%	96,5%	99,2%	99,3%	96,7%
25615	75	60	55	40	89,7%	96,5%	99,2%	99,4%	96,9%
25616	75	60	55	45	89,9%	96,5%	99,2%	99,4%	97,1%
25617	75	60	55	50	90,1%	96,4%	99,2%	99,4%	97,3%
25618	75	60	55	55	90,2%	96,4%	99,3%	99,5%	97,5%
25619	75	60	55	60	90,4%	96,3%	99,3%	99,5%	97,7%
25620	75	60	55	65	90,5%	96,3%	99,3%	99,5%	97,8%
25621	75	60	55	70	90,7%	96,2%	99,3%	99,6%	97,9%
25622	75	60	55	75	90,8%	96,2%	99,4%	99,6%	98,1%
25623	75	60	55	80	91,0%	96,1%	99,4%	99,6%	98,2%
25624	75	60	60	20	86,4%	95,8%	99,0%	99,1%	95,9%
25625	75	60	60	25	86,6%	95,8%	99,0%	99,2%	96,1%
25626	75	60	60	30	86,8%	95,7%	99,0%	99,2%	96,4%
25627	75	60	60	35	87,0%	95,7%	99,1%	99,3%	96,6%
25628	75	60	60	40	87,2%	95,6%	99,1%	99,3%	96,8%
25629	75	60	60	45	87,4%	95,6%	99,1%	99,4%	97,0%
25630	75	60	60	50	87,6%	95,5%	99,1%	99,4%	97,2%
25631	75	60	60	55	87,8%	95,5%	99,2%	99,4%	97,4%
25632	75	60	60	60	88,0%	95,4%	99,2%	99,5%	97,6%
25633	75	60	60	65	88,2%	95,3%	99,2%	99,5%	97,7%
25634	75	60	60	70	88,4%	95,3%	99,3%	99,5%	97,9%
25635	75	60	60	75	88,5%	95,2%	99,3%	99,6%	98,0%
25636	75	60	60	80	88,7%	95,2%	99,3%	99,6%	98,1%
25637	75	60	65	20	83,3%	94,8%	98,8%	99,0%	95,7%
25638	75	60	65	25	83,5%	94,8%	98,9%	99,1%	96,0%
25639	75	60	65	30	83,7%	94,7%	98,9%	99,2%	96,2%
25640	75	60	65	35	84,0%	94,6%	98,9%	99,2%	96,5%

25641	75	60	65	40	84,2%	94,6%	99,0%	99,3%	96,7%
25642	75	60	65	45	84,5%	94,5%	99,0%	99,3%	96,9%
25643	75	60	65	50	84,7%	94,4%	99,0%	99,4%	97,1%
25644	75	60	65	55	84,9%	94,3%	99,1%	99,4%	97,3%
25645	75	60	65	60	85,1%	94,3%	99,1%	99,4%	97,5%
25646	75	60	65	65	85,4%	94,2%	99,1%	99,5%	97,6%
25647	75	60	65	70	85,6%	94,1%	99,2%	99,5%	97,8%
25648	75	60	65	75	85,8%	94,1%	99,2%	99,5%	97,9%
25649	75	60	65	80	86,0%	94,0%	99,2%	99,6%	98,1%
25650	75	60	70	20	79,5%	93,6%	98,7%	99,0%	95,5%
25651	75	60	70	25	79,8%	93,5%	98,7%	99,0%	95,8%
25652	75	60	70	30	80,1%	93,4%	98,7%	99,1%	96,1%
25653	75	60	70	35	80,4%	93,3%	98,8%	99,2%	96,3%
25654	75	60	70	40	80,7%	93,2%	98,8%	99,2%	96,5%
25655	75	60	70	45	80,9%	93,2%	98,9%	99,3%	96,8%
25656	75	60	70	50	81,2%	93,1%	98,9%	99,3%	97,0%
25657	75	60	70	55	81,5%	93,0%	98,9%	99,4%	97,2%
25658	75	60	70	60	81,7%	92,9%	99,0%	99,4%	97,4%
25659	75	60	70	65	82,0%	92,8%	99,0%	99,4%	97,5%
25660	75	60	70	70	82,3%	92,7%	99,0%	99,5%	97,7%
25661	75	60	70	75	82,5%	92,6%	99,1%	99,5%	97,8%
25662	75	60	70	80	82,8%	92,5%	99,1%	99,6%	98,0%
25663	75	60	75	20	75,2%	92,0%	98,5%	98,9%	95,3%
25664	75	60	75	25	75,5%	91,9%	98,5%	99,0%	95,6%
25665	75	60	75	30	75,9%	91,8%	98,6%	99,1%	95,9%
25666	75	60	75	35	76,2%	91,7%	98,6%	99,1%	96,2%
25667	75	60	75	40	76,5%	91,6%	98,7%	99,2%	96,4%
25668	75	60	75	45	76,8%	91,5%	98,7%	99,2%	96,6%
25669	75	60	75	50	77,1%	91,4%	98,8%	99,3%	96,9%
25670	75	60	75	55	77,4%	91,3%	98,8%	99,3%	97,1%
25671	75	60	75	60	77,8%	91,2%	98,8%	99,4%	97,2%
25672	75	60	75	65	78,1%	91,1%	98,9%	99,4%	97,4%
25673	75	60	75	70	78,4%	91,0%	98,9%	99,5%	97,6%

25674	75	60	75	75	78,7%	90,9%	99,0%	99,5%	97,7%
25675	75	60	75	80	78,9%	90,8%	99,0%	99,5%	97,9%
25676	75	60	80	20	70,3%	90,1%	98,3%	98,8%	95,1%
25677	75	60	80	25	70,7%	90,0%	98,3%	98,9%	95,4%
25678	75	60	80	30	71,1%	89,9%	98,4%	99,0%	95,7%
25679	75	60	80	35	71,4%	89,8%	98,5%	99,1%	96,0%
25680	75	60	80	40	71,8%	89,7%	98,5%	99,1%	96,3%
25681	75	60	80	45	72,1%	89,5%	98,6%	99,2%	96,5%
25682	75	60	80	50	72,5%	89,4%	98,6%	99,2%	96,7%
25683	75	60	80	55	72,8%	89,3%	98,6%	99,3%	96,9%
25684	75	60	80	60	73,2%	89,2%	98,7%	99,3%	97,1%
25685	75	60	80	65	73,5%	89,0%	98,7%	99,4%	97,3%
25686	75	60	80	70	73,9%	88,9%	98,8%	99,4%	97,5%
25687	75	60	80	75	74,2%	88,8%	98,8%	99,5%	97,7%
25688	75	60	80	80	74,6%	88,7%	98,9%	99,5%	97,8%
25689	75	65	20	20	98,0%	99,4%	99,6%	99,4%	96,7%
25690	75	65	20	25	98,1%	99,4%	99,6%	99,4%	96,9%
25691	75	65	20	30	98,1%	99,4%	99,6%	99,5%	97,1%
25692	75	65	20	35	98,1%	99,3%	99,6%	99,5%	97,3%
25693	75	65	20	40	98,2%	99,3%	99,6%	99,5%	97,5%
25694	75	65	20	45	98,2%	99,3%	99,7%	99,6%	97,6%
25695	75	65	20	50	98,2%	99,3%	99,7%	99,6%	97,8%
25696	75	65	20	55	98,3%	99,3%	99,7%	99,6%	97,9%
25697	75	65	20	60	98,3%	99,3%	99,7%	99,7%	98,1%
25698	75	65	20	65	98,3%	99,3%	99,7%	99,7%	98,2%
25699	75	65	20	70	98,4%	99,3%	99,7%	99,7%	98,3%
25700	75	65	20	75	98,4%	99,3%	99,7%	99,7%	98,4%
25701	75	65	20	80	98,4%	99,3%	99,7%	99,7%	98,5%
25702	75	65	25	20	97,5%	99,2%	99,5%	99,4%	96,6%
25703	75	65	25	25	97,6%	99,2%	99,6%	99,4%	96,8%
25704	75	65	25	30	97,6%	99,2%	99,6%	99,4%	97,0%
25705	75	65	25	35	97,6%	99,2%	99,6%	99,5%	97,2%
25706	75	65	25	40	97,7%	99,2%	99,6%	99,5%	97,4%

25707	75	65	25	45	97,7%	99,2%	99,6%	99,5%	97,5%
25708	75	65	25	50	97,8%	99,1%	99,6%	99,6%	97,7%
25709	75	65	25	55	97,8%	99,1%	99,6%	99,6%	97,8%
25710	75	65	25	60	97,8%	99,1%	99,7%	99,6%	98,0%
25711	75	65	25	65	97,9%	99,1%	99,7%	99,7%	98,1%
25712	75	65	25	70	97,9%	99,1%	99,7%	99,7%	98,2%
25713	75	65	25	75	97,9%	99,1%	99,7%	99,7%	98,3%
25714	75	65	25	80	98,0%	99,1%	99,7%	99,7%	98,5%
25715	75	65	30	20	96,8%	99,0%	99,5%	99,3%	96,4%
25716	75	65	30	25	96,9%	99,0%	99,5%	99,4%	96,6%
25717	75	65	30	30	96,9%	99,0%	99,5%	99,4%	96,9%
25718	75	65	30	35	97,0%	99,0%	99,5%	99,4%	97,1%
25719	75	65	30	40	97,0%	99,0%	99,5%	99,5%	97,2%
25720	75	65	30	45	97,1%	98,9%	99,6%	99,5%	97,4%
25721	75	65	30	50	97,1%	98,9%	99,6%	99,5%	97,6%
25722	75	65	30	55	97,2%	98,9%	99,6%	99,6%	97,7%
25723	75	65	30	60	97,2%	98,9%	99,6%	99,6%	97,9%
25724	75	65	30	65	97,3%	98,9%	99,6%	99,6%	98,0%
25725	75	65	30	70	97,3%	98,9%	99,6%	99,7%	98,2%
25726	75	65	30	75	97,4%	98,9%	99,6%	99,7%	98,3%
25727	75	65	30	80	97,4%	98,8%	99,7%	99,7%	98,4%
25728	75	65	35	20	96,0%	98,8%	99,4%	99,3%	96,3%
25729	75	65	35	25	96,1%	98,7%	99,4%	99,3%	96,5%
25730	75	65	35	30	96,1%	98,7%	99,5%	99,4%	96,7%
25731	75	65	35	35	96,2%	98,7%	99,5%	99,4%	96,9%
25732	75	65	35	40	96,2%	98,7%	99,5%	99,5%	97,1%
25733	75	65	35	45	96,3%	98,7%	99,5%	99,5%	97,3%
25734	75	65	35	50	96,4%	98,7%	99,5%	99,5%	97,5%
25735	75	65	35	55	96,4%	98,6%	99,5%	99,6%	97,7%
25736	75	65	35	60	96,5%	98,6%	99,6%	99,6%	97,8%
25737	75	65	35	65	96,6%	98,6%	99,6%	99,6%	98,0%
25738	75	65	35	70	96,6%	98,6%	99,6%	99,6%	98,1%
25739	75	65	35	75	96,7%	98,6%	99,6%	99,7%	98,2%

25740	75	65	35	80	96,7%	98,5%	99,6%	99,7%	98,3%
25741	75	65	40	20	94,9%	98,4%	99,3%	99,2%	96,1%
25742	75	65	40	25	95,0%	98,4%	99,4%	99,3%	96,4%
25743	75	65	40	30	95,1%	98,4%	99,4%	99,3%	96,6%
25744	75	65	40	35	95,2%	98,4%	99,4%	99,4%	96,8%
25745	75	65	40	40	95,2%	98,3%	99,4%	99,4%	97,0%
25746	75	65	40	45	95,3%	98,3%	99,4%	99,5%	97,2%
25747	75	65	40	50	95,4%	98,3%	99,5%	99,5%	97,4%
25748	75	65	40	55	95,5%	98,3%	99,5%	99,5%	97,6%
25749	75	65	40	60	95,6%	98,3%	99,5%	99,6%	97,7%
25750	75	65	40	65	95,6%	98,2%	99,5%	99,6%	97,9%
25751	75	65	40	70	95,7%	98,2%	99,5%	99,6%	98,0%
25752	75	65	40	75	95,8%	98,2%	99,5%	99,6%	98,1%
25753	75	65	40	80	95,8%	98,2%	99,6%	99,7%	98,3%
25754	75	65	45	20	93,6%	98,0%	99,3%	99,2%	96,0%
25755	75	65	45	25	93,7%	98,0%	99,3%	99,2%	96,2%
25756	75	65	45	30	93,8%	98,0%	99,3%	99,3%	96,5%
25757	75	65	45	35	93,9%	98,0%	99,3%	99,3%	96,7%
25758	75	65	45	40	94,0%	97,9%	99,3%	99,4%	96,9%
25759	75	65	45	45	94,1%	97,9%	99,4%	99,4%	97,1%
25760	75	65	45	50	94,2%	97,9%	99,4%	99,5%	97,3%
25761	75	65	45	55	94,3%	97,8%	99,4%	99,5%	97,5%
25762	75	65	45	60	94,4%	97,8%	99,4%	99,5%	97,6%
25763	75	65	45	65	94,5%	97,8%	99,4%	99,6%	97,8%
25764	75	65	45	70	94,6%	97,8%	99,5%	99,6%	97,9%
25765	75	65	45	75	94,7%	97,7%	99,5%	99,6%	98,1%
25766	75	65	45	80	94,7%	97,7%	99,5%	99,6%	98,2%
25767	75	65	50	20	91,9%	97,5%	99,2%	99,1%	95,8%
25768	75	65	50	25	92,1%	97,5%	99,2%	99,2%	96,1%
25769	75	65	50	30	92,2%	97,5%	99,2%	99,2%	96,3%
25770	75	65	50	35	92,3%	97,4%	99,2%	99,3%	96,6%
25771	75	65	50	40	92,4%	97,4%	99,3%	99,3%	96,8%
25772	75	65	50	45	92,6%	97,4%	99,3%	99,4%	97,0%

25773	75	65	50	50	92,7%	97,3%	99,3%	99,4%	97,2%
25774	75	65	50	55	92,8%	97,3%	99,3%	99,5%	97,4%
25775	75	65	50	60	92,9%	97,3%	99,4%	99,5%	97,5%
25776	75	65	50	65	93,0%	97,2%	99,4%	99,5%	97,7%
25777	75	65	50	70	93,1%	97,2%	99,4%	99,6%	97,8%
25778	75	65	50	75	93,3%	97,2%	99,4%	99,6%	98,0%
25779	75	65	50	80	93,4%	97,1%	99,4%	99,6%	98,1%
25780	75	65	55	20	89,9%	96,9%	99,0%	99,1%	95,6%
25781	75	65	55	25	90,1%	96,9%	99,1%	99,1%	95,9%
25782	75	65	55	30	90,2%	96,8%	99,1%	99,2%	96,2%
25783	75	65	55	35	90,4%	96,8%	99,1%	99,3%	96,4%
25784	75	65	55	40	90,5%	96,8%	99,2%	99,3%	96,6%
25785	75	65	55	45	90,7%	96,7%	99,2%	99,3%	96,9%
25786	75	65	55	50	90,8%	96,7%	99,2%	99,4%	97,1%
25787	75	65	55	55	91,0%	96,6%	99,2%	99,4%	97,2%
25788	75	65	55	60	91,1%	96,6%	99,3%	99,5%	97,4%
25789	75	65	55	65	91,3%	96,5%	99,3%	99,5%	97,6%
25790	75	65	55	70	91,4%	96,5%	99,3%	99,5%	97,7%
25791	75	65	55	75	91,5%	96,5%	99,3%	99,6%	97,9%
25792	75	65	55	80	91,7%	96,4%	99,4%	99,6%	98,0%
25793	75	65	60	20	87,4%	96,1%	98,9%	99,0%	95,5%
25794	75	65	60	25	87,6%	96,1%	99,0%	99,1%	95,7%
25795	75	65	60	30	87,8%	96,0%	99,0%	99,1%	96,0%
25796	75	65	60	35	88,0%	96,0%	99,0%	99,2%	96,3%
25797	75	65	60	40	88,2%	95,9%	99,1%	99,3%	96,5%
25798	75	65	60	45	88,4%	95,9%	99,1%	99,3%	96,7%
25799	75	65	60	50	88,5%	95,8%	99,1%	99,4%	96,9%
25800	75	65	60	55	88,7%	95,8%	99,1%	99,4%	97,1%
25801	75	65	60	60	88,9%	95,7%	99,2%	99,4%	97,3%
25802	75	65	60	65	89,1%	95,7%	99,2%	99,5%	97,5%
25803	75	65	60	70	89,2%	95,6%	99,2%	99,5%	97,7%
25804	75	65	60	75	89,4%	95,6%	99,3%	99,5%	97,8%
25805	75	65	60	80	89,6%	95,5%	99,3%	99,6%	97,9%

25806	75	65	65	20	84,5%	95,2%	98,8%	99,0%	95,3%
25807	75	65	65	25	84,7%	95,1%	98,8%	99,0%	95,6%
25808	75	65	65	30	84,9%	95,1%	98,9%	99,1%	95,9%
25809	75	65	65	35	85,1%	95,0%	98,9%	99,2%	96,1%
25810	75	65	65	40	85,4%	94,9%	98,9%	99,2%	96,4%
25811	75	65	65	45	85,6%	94,9%	99,0%	99,3%	96,6%
25812	75	65	65	50	85,8%	94,8%	99,0%	99,3%	96,8%
25813	75	65	65	55	86,0%	94,8%	99,0%	99,4%	97,0%
25814	75	65	65	60	86,2%	94,7%	99,1%	99,4%	97,2%
25815	75	65	65	65	86,4%	94,6%	99,1%	99,4%	97,4%
25816	75	65	65	70	86,6%	94,6%	99,1%	99,5%	97,6%
25817	75	65	65	75	86,8%	94,5%	99,2%	99,5%	97,7%
25818	75	65	65	80	87,0%	94,4%	99,2%	99,5%	97,9%
25819	75	65	70	20	80,9%	94,0%	98,6%	98,9%	95,1%
25820	75	65	70	25	81,2%	93,9%	98,7%	99,0%	95,4%
25821	75	65	70	30	81,5%	93,9%	98,7%	99,0%	95,7%
25822	75	65	70	35	81,7%	93,8%	98,7%	99,1%	96,0%
25823	75	65	70	40	82,0%	93,7%	98,8%	99,2%	96,2%
25824	75	65	70	45	82,3%	93,6%	98,8%	99,2%	96,5%
25825	75	65	70	50	82,5%	93,6%	98,9%	99,3%	96,7%
25826	75	65	70	55	82,8%	93,5%	98,9%	99,3%	96,9%
25827	75	65	70	60	83,0%	93,4%	98,9%	99,4%	97,1%
25828	75	65	70	65	83,3%	93,3%	99,0%	99,4%	97,3%
25829	75	65	70	70	83,5%	93,2%	99,0%	99,4%	97,5%
25830	75	65	70	75	83,8%	93,2%	99,0%	99,5%	97,6%
25831	75	65	70	80	84,0%	93,1%	99,1%	99,5%	97,8%
25832	75	65	75	20	76,8%	92,6%	98,4%	98,8%	94,9%
25833	75	65	75	25	77,1%	92,5%	98,5%	98,9%	95,2%
25834	75	65	75	30	77,5%	92,4%	98,5%	99,0%	95,5%
25835	75	65	75	35	77,8%	92,3%	98,6%	99,0%	95,8%
25836	75	65	75	40	78,1%	92,2%	98,6%	99,1%	96,1%
25837	75	65	75	45	78,4%	92,1%	98,7%	99,2%	96,3%
25838	75	65	75	50	78,7%	92,0%	98,7%	99,2%	96,5%

25839	75	65	75	55	79,0%	91,9%	98,8%	99,3%	96,8%
25840	75	65	75	60	79,3%	91,8%	98,8%	99,3%	97,0%
25841	75	65	75	65	79,5%	91,7%	98,8%	99,4%	97,2%
25842	75	65	75	70	79,8%	91,6%	98,9%	99,4%	97,4%
25843	75	65	75	75	80,1%	91,5%	98,9%	99,4%	97,5%
25844	75	65	75	80	80,4%	91,4%	99,0%	99,5%	97,7%
25845	75	65	80	20	72,2%	90,8%	98,2%	98,8%	94,7%
25846	75	65	80	25	72,5%	90,7%	98,3%	98,8%	95,0%
25847	75	65	80	30	72,9%	90,6%	98,3%	98,9%	95,3%
25848	75	65	80	35	73,2%	90,5%	98,4%	99,0%	95,6%
25849	75	65	80	40	73,5%	90,4%	98,5%	99,1%	95,9%
25850	75	65	80	45	73,9%	90,3%	98,5%	99,1%	96,2%
25851	75	65	80	50	74,2%	90,1%	98,6%	99,2%	96,4%
25852	75	65	80	55	74,6%	90,0%	98,6%	99,2%	96,6%
25853	75	65	80	60	74,9%	89,9%	98,6%	99,3%	96,9%
25854	75	65	80	65	75,2%	89,8%	98,7%	99,3%	97,1%
25855	75	65	80	70	75,6%	89,7%	98,7%	99,4%	97,2%
25856	75	65	80	75	75,9%	89,6%	98,8%	99,4%	97,4%
25857	75	65	80	80	76,2%	89,4%	98,8%	99,5%	97,6%
25858	75	70	20	20	98,2%	99,4%	99,6%	99,4%	96,4%
25859	75	70	20	25	98,2%	99,4%	99,6%	99,4%	96,6%
25860	75	70	20	30	98,3%	99,4%	99,6%	99,4%	96,8%
25861	75	70	20	35	98,3%	99,4%	99,6%	99,5%	97,0%
25862	75	70	20	40	98,3%	99,4%	99,6%	99,5%	97,2%
25863	75	70	20	45	98,4%	99,4%	99,6%	99,5%	97,4%
25864	75	70	20	50	98,4%	99,4%	99,7%	99,6%	97,6%
25865	75	70	20	55	98,4%	99,4%	99,7%	99,6%	97,7%
25866	75	70	20	60	98,4%	99,4%	99,7%	99,6%	97,9%
25867	75	70	20	65	98,5%	99,3%	99,7%	99,7%	98,0%
25868	75	70	20	70	98,5%	99,3%	99,7%	99,7%	98,1%
25869	75	70	20	75	98,5%	99,3%	99,7%	99,7%	98,3%
25870	75	70	20	80	98,5%	99,3%	99,7%	99,7%	98,4%
25871	75	70	25	20	97,7%	99,3%	99,5%	99,3%	96,2%

25872	75	70	25	25	97,8%	99,3%	99,5%	99,4%	96,5%
25873	75	70	25	30	97,8%	99,2%	99,6%	99,4%	96,7%
25874	75	70	25	35	97,8%	99,2%	99,6%	99,4%	96,9%
25875	75	70	25	40	97,9%	99,2%	99,6%	99,5%	97,1%
25876	75	70	25	45	97,9%	99,2%	99,6%	99,5%	97,3%
25877	75	70	25	50	97,9%	99,2%	99,6%	99,5%	97,5%
25878	75	70	25	55	98,0%	99,2%	99,6%	99,6%	97,6%
25879	75	70	25	60	98,0%	99,2%	99,6%	99,6%	97,8%
25880	75	70	25	65	98,0%	99,2%	99,7%	99,6%	97,9%
25881	75	70	25	70	98,1%	99,2%	99,7%	99,7%	98,1%
25882	75	70	25	75	98,1%	99,2%	99,7%	99,7%	98,2%
25883	75	70	25	80	98,1%	99,1%	99,7%	99,7%	98,3%
25884	75	70	30	20	97,1%	99,1%	99,5%	99,3%	96,1%
25885	75	70	30	25	97,1%	99,1%	99,5%	99,3%	96,3%
25886	75	70	30	30	97,2%	99,1%	99,5%	99,4%	96,6%
25887	75	70	30	35	97,2%	99,0%	99,5%	99,4%	96,8%
25888	75	70	30	40	97,3%	99,0%	99,5%	99,4%	97,0%
25889	75	70	30	45	97,3%	99,0%	99,6%	99,5%	97,2%
25890	75	70	30	50	97,4%	99,0%	99,6%	99,5%	97,4%
25891	75	70	30	55	97,4%	99,0%	99,6%	99,5%	97,5%
25892	75	70	30	60	97,5%	99,0%	99,6%	99,6%	97,7%
25893	75	70	30	65	97,5%	99,0%	99,6%	99,6%	97,8%
25894	75	70	30	70	97,6%	99,0%	99,6%	99,6%	98,0%
25895	75	70	30	75	97,6%	98,9%	99,6%	99,7%	98,1%
25896	75	70	30	80	97,6%	98,9%	99,6%	99,7%	98,2%
25897	75	70	35	20	96,3%	98,8%	99,4%	99,2%	95,9%
25898	75	70	35	25	96,4%	98,8%	99,4%	99,3%	96,2%
25899	75	70	35	30	96,4%	98,8%	99,4%	99,3%	96,4%
25900	75	70	35	35	96,5%	98,8%	99,5%	99,4%	96,6%
25901	75	70	35	40	96,6%	98,8%	99,5%	99,4%	96,9%
25902	75	70	35	45	96,6%	98,8%	99,5%	99,4%	97,1%
25903	75	70	35	50	96,7%	98,8%	99,5%	99,5%	97,3%
25904	75	70	35	55	96,7%	98,7%	99,5%	99,5%	97,4%

25905	75	70	35	60	96,8%	98,7%	99,5%	99,6%	97,6%
25906	75	70	35	65	96,8%	98,7%	99,6%	99,6%	97,7%
25907	75	70	35	70	96,9%	98,7%	99,6%	99,6%	97,9%
25908	75	70	35	75	96,9%	98,7%	99,6%	99,6%	98,0%
25909	75	70	35	80	97,0%	98,7%	99,6%	99,7%	98,2%
25910	75	70	40	20	95,3%	98,5%	99,3%	99,2%	95,7%
25911	75	70	40	25	95,4%	98,5%	99,3%	99,2%	96,0%
25912	75	70	40	30	95,5%	98,5%	99,4%	99,3%	96,3%
25913	75	70	40	35	95,6%	98,5%	99,4%	99,3%	96,5%
25914	75	70	40	40	95,6%	98,5%	99,4%	99,4%	96,7%
25915	75	70	40	45	95,7%	98,5%	99,4%	99,4%	96,9%
25916	75	70	40	50	95,8%	98,4%	99,4%	99,5%	97,1%
25917	75	70	40	55	95,8%	98,4%	99,5%	99,5%	97,3%
25918	75	70	40	60	95,9%	98,4%	99,5%	99,5%	97,5%
25919	75	70	40	65	96,0%	98,4%	99,5%	99,6%	97,7%
25920	75	70	40	70	96,1%	98,3%	99,5%	99,6%	97,8%
25921	75	70	40	75	96,1%	98,3%	99,5%	99,6%	98,0%
25922	75	70	40	80	96,2%	98,3%	99,5%	99,6%	98,1%
25923	75	70	45	20	94,1%	98,2%	99,2%	99,1%	95,6%
25924	75	70	45	25	94,2%	98,2%	99,3%	99,2%	95,9%
25925	75	70	45	30	94,3%	98,1%	99,3%	99,2%	96,1%
25926	75	70	45	35	94,4%	98,1%	99,3%	99,3%	96,4%
25927	75	70	45	40	94,5%	98,1%	99,3%	99,3%	96,6%
25928	75	70	45	45	94,6%	98,1%	99,3%	99,4%	96,8%
25929	75	70	45	50	94,7%	98,0%	99,4%	99,4%	97,0%
25930	75	70	45	55	94,7%	98,0%	99,4%	99,5%	97,2%
25931	75	70	45	60	94,8%	98,0%	99,4%	99,5%	97,4%
25932	75	70	45	65	94,9%	98,0%	99,4%	99,5%	97,6%
25933	75	70	45	70	95,0%	97,9%	99,4%	99,6%	97,7%
25934	75	70	45	75	95,1%	97,9%	99,5%	99,6%	97,9%
25935	75	70	45	80	95,2%	97,9%	99,5%	99,6%	98,0%
25936	75	70	50	20	92,6%	97,7%	99,1%	99,1%	95,4%
25937	75	70	50	25	92,7%	97,7%	99,2%	99,1%	95,7%

25938	75	70	50	30	92,8%	97,7%	99,2%	99,2%	96,0%
25939	75	70	50	35	92,9%	97,6%	99,2%	99,2%	96,2%
25940	75	70	50	40	93,0%	97,6%	99,2%	99,3%	96,5%
25941	75	70	50	45	93,2%	97,6%	99,3%	99,3%	96,7%
25942	75	70	50	50	93,3%	97,5%	99,3%	99,4%	96,9%
25943	75	70	50	55	93,4%	97,5%	99,3%	99,4%	97,1%
25944	75	70	50	60	93,5%	97,5%	99,3%	99,5%	97,3%
25945	75	70	50	65	93,6%	97,4%	99,4%	99,5%	97,5%
25946	75	70	50	70	93,7%	97,4%	99,4%	99,5%	97,6%
25947	75	70	50	75	93,8%	97,4%	99,4%	99,6%	97,8%
25948	75	70	50	80	93,9%	97,3%	99,4%	99,6%	97,9%
25949	75	70	55	20	90,7%	97,1%	99,0%	99,0%	95,2%
25950	75	70	55	25	90,8%	97,1%	99,0%	99,1%	95,5%
25951	75	70	55	30	91,0%	97,1%	99,1%	99,1%	95,8%
25952	75	70	55	35	91,1%	97,0%	99,1%	99,2%	96,1%
25953	75	70	55	40	91,3%	97,0%	99,1%	99,2%	96,3%
25954	75	70	55	45	91,4%	97,0%	99,2%	99,3%	96,6%
25955	75	70	55	50	91,5%	96,9%	99,2%	99,3%	96,8%
25956	75	70	55	55	91,7%	96,9%	99,2%	99,4%	97,0%
25957	75	70	55	60	91,8%	96,8%	99,2%	99,4%	97,2%
25958	75	70	55	65	91,9%	96,8%	99,3%	99,5%	97,4%
25959	75	70	55	70	92,1%	96,8%	99,3%	99,5%	97,5%
25960	75	70	55	75	92,2%	96,7%	99,3%	99,5%	97,7%
25961	75	70	55	80	92,3%	96,7%	99,3%	99,6%	97,8%
25962	75	70	60	20	88,4%	96,4%	98,9%	98,9%	95,0%
25963	75	70	60	25	88,6%	96,4%	98,9%	99,0%	95,3%
25964	75	70	60	30	88,7%	96,3%	99,0%	99,1%	95,6%
25965	75	70	60	35	88,9%	96,3%	99,0%	99,1%	95,9%
25966	75	70	60	40	89,1%	96,2%	99,0%	99,2%	96,2%
25967	75	70	60	45	89,3%	96,2%	99,1%	99,3%	96,4%
25968	75	70	60	50	89,4%	96,1%	99,1%	99,3%	96,6%
25969	75	70	60	55	89,6%	96,1%	99,1%	99,3%	96,9%
25970	75	70	60	60	89,7%	96,0%	99,1%	99,4%	97,1%

25971	75	70	60	65	89,9%	96,0%	99,2%	99,4%	97,2%
25972	75	70	60	70	90,1%	95,9%	99,2%	99,5%	97,4%
25973	75	70	60	75	90,2%	95,9%	99,2%	99,5%	97,6%
25974	75	70	60	80	90,4%	95,8%	99,3%	99,5%	97,7%
25975	75	70	65	20	85,6%	95,5%	98,7%	98,9%	94,8%
25976	75	70	65	25	85,8%	95,5%	98,8%	99,0%	95,1%
25977	75	70	65	30	86,0%	95,4%	98,8%	99,0%	95,5%
25978	75	70	65	35	86,2%	95,4%	98,9%	99,1%	95,7%
25979	75	70	65	40	86,4%	95,3%	98,9%	99,2%	96,0%
25980	75	70	65	45	86,6%	95,3%	98,9%	99,2%	96,3%
25981	75	70	65	50	86,8%	95,2%	99,0%	99,3%	96,5%
25982	75	70	65	55	87,0%	95,1%	99,0%	99,3%	96,7%
25983	75	70	65	60	87,2%	95,1%	99,0%	99,4%	96,9%
25984	75	70	65	65	87,4%	95,0%	99,1%	99,4%	97,1%
25985	75	70	65	70	87,6%	94,9%	99,1%	99,4%	97,3%
25986	75	70	65	75	87,8%	94,9%	99,1%	99,5%	97,5%
25987	75	70	65	80	88,0%	94,8%	99,2%	99,5%	97,7%
25988	75	70	70	20	82,3%	94,4%	98,6%	98,8%	94,6%
25989	75	70	70	25	82,5%	94,4%	98,6%	98,9%	95,0%
25990	75	70	70	30	82,8%	94,3%	98,7%	99,0%	95,3%
25991	75	70	70	35	83,0%	94,2%	98,7%	99,0%	95,6%
25992	75	70	70	40	83,3%	94,2%	98,8%	99,1%	95,9%
25993	75	70	70	45	83,5%	94,1%	98,8%	99,2%	96,1%
25994	75	70	70	50	83,8%	94,0%	98,8%	99,2%	96,4%
25995	75	70	70	55	84,0%	93,9%	98,9%	99,3%	96,6%
25996	75	70	70	60	84,2%	93,9%	98,9%	99,3%	96,8%
25997	75	70	70	65	84,5%	93,8%	98,9%	99,4%	97,0%
25998	75	70	70	70	84,7%	93,7%	99,0%	99,4%	97,2%
25999	75	70	70	75	84,9%	93,6%	99,0%	99,4%	97,4%
26000	75	70	70	80	85,1%	93,6%	99,0%	99,5%	97,6%
26001	75	70	75	20	78,4%	93,1%	98,4%	98,7%	94,4%
26002	75	70	75	25	78,7%	93,0%	98,4%	98,8%	94,8%
26003	75	70	75	30	79,0%	92,9%	98,5%	98,9%	95,1%

26004	75	70	75	35	79,3%	92,8%	98,5%	99,0%	95,4%
26005	75	70	75	40	79,5%	92,8%	98,6%	99,0%	95,7%
26006	75	70	75	45	79,8%	92,7%	98,6%	99,1%	96,0%
26007	75	70	75	50	80,1%	92,6%	98,7%	99,2%	96,2%
26008	75	70	75	55	80,4%	92,5%	98,7%	99,2%	96,5%
26009	75	70	75	60	80,7%	92,4%	98,8%	99,3%	96,7%
26010	75	70	75	65	80,9%	92,3%	98,8%	99,3%	96,9%
26011	75	70	75	70	81,2%	92,2%	98,8%	99,4%	97,1%
26012	75	70	75	75	81,5%	92,1%	98,9%	99,4%	97,3%
26013	75	70	75	80	81,7%	92,0%	98,9%	99,4%	97,5%
26014	75	70	80	20	73,9%	91,5%	98,2%	98,7%	94,2%
26015	75	70	80	25	74,2%	91,4%	98,2%	98,8%	94,5%
26016	75	70	80	30	74,6%	91,3%	98,3%	98,8%	94,9%
26017	75	70	80	35	74,9%	91,1%	98,4%	98,9%	95,2%
26018	75	70	80	40	75,2%	91,0%	98,4%	99,0%	95,5%
26019	75	70	80	45	75,6%	90,9%	98,5%	99,0%	95,8%
26020	75	70	80	50	75,9%	90,8%	98,5%	99,1%	96,1%
26021	75	70	80	55	76,2%	90,7%	98,6%	99,2%	96,3%
26022	75	70	80	60	76,5%	90,6%	98,6%	99,2%	96,6%
26023	75	70	80	65	76,8%	90,5%	98,7%	99,3%	96,8%
26024	75	70	80	70	77,2%	90,4%	98,7%	99,3%	97,0%
26025	75	70	80	75	77,5%	90,3%	98,7%	99,4%	97,2%
26026	75	70	80	80	77,8%	90,2%	98,8%	99,4%	97,4%
26027	75	75	20	20	98,4%	99,5%	99,6%	99,3%	96,0%
26028	75	75	20	25	98,4%	99,5%	99,6%	99,3%	96,3%
26029	75	75	20	30	98,4%	99,4%	99,6%	99,4%	96,5%
26030	75	75	20	35	98,4%	99,4%	99,6%	99,4%	96,7%
26031	75	75	20	40	98,5%	99,4%	99,6%	99,5%	96,9%
26032	75	75	20	45	98,5%	99,4%	99,6%	99,5%	97,1%
26033	75	75	20	50	98,5%	99,4%	99,6%	99,5%	97,3%
26034	75	75	20	55	98,5%	99,4%	99,7%	99,6%	97,5%
26035	75	75	20	60	98,6%	99,4%	99,7%	99,6%	97,7%
26036	75	75	20	65	98,6%	99,4%	99,7%	99,6%	97,8%

26037	75	75	20	70	98,6%	99,4%	99,7%	99,6%	98,0%
26038	75	75	20	75	98,6%	99,4%	99,7%	99,7%	98,1%
26039	75	75	20	80	98,7%	99,4%	99,7%	99,7%	98,2%
26040	75	75	25	20	97,9%	99,3%	99,5%	99,3%	95,9%
26041	75	75	25	25	97,9%	99,3%	99,5%	99,3%	96,1%
26042	75	75	25	30	98,0%	99,3%	99,5%	99,4%	96,4%
26043	75	75	25	35	98,0%	99,3%	99,6%	99,4%	96,6%
26044	75	75	25	40	98,0%	99,3%	99,6%	99,4%	96,8%
26045	75	75	25	45	98,1%	99,3%	99,6%	99,5%	97,0%
26046	75	75	25	50	98,1%	99,3%	99,6%	99,5%	97,2%
26047	75	75	25	55	98,1%	99,3%	99,6%	99,5%	97,4%
26048	75	75	25	60	98,2%	99,3%	99,6%	99,6%	97,6%
26049	75	75	25	65	98,2%	99,2%	99,6%	99,6%	97,7%
26050	75	75	25	70	98,2%	99,2%	99,7%	99,6%	97,9%
26051	75	75	25	75	98,3%	99,2%	99,7%	99,7%	98,0%
26052	75	75	25	80	98,3%	99,2%	99,7%	99,7%	98,1%
26053	75	75	30	20	97,3%	99,2%	99,5%	99,2%	95,7%
26054	75	75	30	25	97,4%	99,1%	99,5%	99,3%	96,0%
26055	75	75	30	30	97,4%	99,1%	99,5%	99,3%	96,2%
26056	75	75	30	35	97,5%	99,1%	99,5%	99,4%	96,5%
26057	75	75	30	40	97,5%	99,1%	99,5%	99,4%	96,7%
26058	75	75	30	45	97,6%	99,1%	99,5%	99,4%	96,9%
26059	75	75	30	50	97,6%	99,1%	99,6%	99,5%	97,1%
26060	75	75	30	55	97,6%	99,1%	99,6%	99,5%	97,3%
26061	75	75	30	60	97,7%	99,1%	99,6%	99,5%	97,5%
26062	75	75	30	65	97,7%	99,0%	99,6%	99,6%	97,6%
26063	75	75	30	70	97,8%	99,0%	99,6%	99,6%	97,8%
26064	75	75	30	75	97,8%	99,0%	99,6%	99,6%	97,9%
26065	75	75	30	80	97,8%	99,0%	99,6%	99,7%	98,1%
26066	75	75	35	20	96,6%	98,9%	99,4%	99,2%	95,5%
26067	75	75	35	25	96,7%	98,9%	99,4%	99,2%	95,8%
26068	75	75	35	30	96,7%	98,9%	99,4%	99,3%	96,1%
26069	75	75	35	35	96,8%	98,9%	99,4%	99,3%	96,3%

26070	75	75	35	40	96,8%	98,9%	99,5%	99,4%	96,6%
26071	75	75	35	45	96,9%	98,9%	99,5%	99,4%	96,8%
26072	75	75	35	50	96,9%	98,8%	99,5%	99,4%	97,0%
26073	75	75	35	55	97,0%	98,8%	99,5%	99,5%	97,2%
26074	75	75	35	60	97,0%	98,8%	99,5%	99,5%	97,4%
26075	75	75	35	65	97,1%	98,8%	99,5%	99,5%	97,5%
26076	75	75	35	70	97,1%	98,8%	99,6%	99,6%	97,7%
26077	75	75	35	75	97,2%	98,8%	99,6%	99,6%	97,8%
26078	75	75	35	80	97,2%	98,8%	99,6%	99,6%	98,0%
26079	75	75	40	20	95,7%	98,7%	99,3%	99,1%	95,3%
26080	75	75	40	25	95,8%	98,6%	99,3%	99,2%	95,6%
26081	75	75	40	30	95,9%	98,6%	99,3%	99,2%	95,9%
26082	75	75	40	35	95,9%	98,6%	99,4%	99,3%	96,2%
26083	75	75	40	40	96,0%	98,6%	99,4%	99,3%	96,4%
26084	75	75	40	45	96,1%	98,6%	99,4%	99,4%	96,6%
26085	75	75	40	50	96,1%	98,5%	99,4%	99,4%	96,9%
26086	75	75	40	55	96,2%	98,5%	99,4%	99,5%	97,1%
26087	75	75	40	60	96,3%	98,5%	99,5%	99,5%	97,3%
26088	75	75	40	65	96,3%	98,5%	99,5%	99,5%	97,4%
26089	75	75	40	70	96,4%	98,5%	99,5%	99,6%	97,6%
26090	75	75	40	75	96,4%	98,5%	99,5%	99,6%	97,8%
26091	75	75	40	80	96,5%	98,4%	99,5%	99,6%	97,9%
26092	75	75	45	20	94,6%	98,3%	99,2%	99,1%	95,2%
26093	75	75	45	25	94,7%	98,3%	99,2%	99,1%	95,5%
26094	75	75	45	30	94,8%	98,3%	99,3%	99,2%	95,7%
26095	75	75	45	35	94,8%	98,2%	99,3%	99,2%	96,0%
26096	75	75	45	40	94,9%	98,2%	99,3%	99,3%	96,3%
26097	75	75	45	45	95,0%	98,2%	99,3%	99,3%	96,5%
26098	75	75	45	50	95,1%	98,2%	99,3%	99,4%	96,7%
26099	75	75	45	55	95,2%	98,2%	99,4%	99,4%	96,9%
26100	75	75	45	60	95,3%	98,1%	99,4%	99,5%	97,1%
26101	75	75	45	65	95,3%	98,1%	99,4%	99,5%	97,3%
26102	75	75	45	70	95,4%	98,1%	99,4%	99,5%	97,5%

26103	75	75	45	75	95,5%	98,1%	99,5%	99,6%	97,7%
26104	75	75	45	80	95,6%	98,0%	99,5%	99,6%	97,8%
26105	75	75	50	20	93,2%	97,9%	99,1%	99,0%	95,0%
26106	75	75	50	25	93,3%	97,9%	99,1%	99,1%	95,3%
26107	75	75	50	30	93,4%	97,8%	99,2%	99,1%	95,6%
26108	75	75	50	35	93,5%	97,8%	99,2%	99,2%	95,9%
26109	75	75	50	40	93,6%	97,8%	99,2%	99,2%	96,1%
26110	75	75	50	45	93,7%	97,7%	99,2%	99,3%	96,4%
26111	75	75	50	50	93,8%	97,7%	99,3%	99,3%	96,6%
26112	75	75	50	55	93,9%	97,7%	99,3%	99,4%	96,8%
26113	75	75	50	60	94,0%	97,7%	99,3%	99,4%	97,0%
26114	75	75	50	65	94,1%	97,6%	99,3%	99,5%	97,2%
26115	75	75	50	70	94,2%	97,6%	99,4%	99,5%	97,4%
26116	75	75	50	75	94,3%	97,6%	99,4%	99,5%	97,6%
26117	75	75	50	80	94,4%	97,5%	99,4%	99,6%	97,7%
26118	75	75	55	20	91,4%	97,4%	99,0%	98,9%	94,8%
26119	75	75	55	25	91,5%	97,3%	99,0%	99,0%	95,1%
26120	75	75	55	30	91,7%	97,3%	99,0%	99,1%	95,4%
26121	75	75	55	35	91,8%	97,2%	99,1%	99,1%	95,7%
26122	75	75	55	40	91,9%	97,2%	99,1%	99,2%	96,0%
26123	75	75	55	45	92,1%	97,2%	99,1%	99,2%	96,2%
26124	75	75	55	50	92,2%	97,1%	99,2%	99,3%	96,5%
26125	75	75	55	55	92,3%	97,1%	99,2%	99,3%	96,7%
26126	75	75	55	60	92,5%	97,1%	99,2%	99,4%	96,9%
26127	75	75	55	65	92,6%	97,0%	99,2%	99,4%	97,1%
26128	75	75	55	70	92,7%	97,0%	99,3%	99,5%	97,3%
26129	75	75	55	75	92,8%	97,0%	99,3%	99,5%	97,5%
26130	75	75	55	80	92,9%	96,9%	99,3%	99,5%	97,6%
26131	75	75	60	20	89,3%	96,7%	98,8%	98,9%	94,5%
26132	75	75	60	25	89,4%	96,6%	98,9%	98,9%	94,9%
26133	75	75	60	30	89,6%	96,6%	98,9%	99,0%	95,2%
26134	75	75	60	35	89,8%	96,6%	99,0%	99,1%	95,5%
26135	75	75	60	40	89,9%	96,5%	99,0%	99,1%	95,8%

26136	75	75	60	45	90,1%	96,5%	99,0%	99,2%	96,1%
26137	75	75	60	50	90,2%	96,4%	99,1%	99,2%	96,3%
26138	75	75	60	55	90,4%	96,4%	99,1%	99,3%	96,6%
26139	75	75	60	60	90,5%	96,3%	99,1%	99,3%	96,8%
26140	75	75	60	65	90,7%	96,3%	99,1%	99,4%	97,0%
26141	75	75	60	70	90,8%	96,2%	99,2%	99,4%	97,2%
26142	75	75	60	75	91,0%	96,2%	99,2%	99,5%	97,4%
26143	75	75	60	80	91,1%	96,1%	99,2%	99,5%	97,5%
26144	75	75	65	20	86,6%	95,9%	98,7%	98,8%	94,3%
26145	75	75	65	25	86,9%	95,8%	98,7%	98,9%	94,7%
26146	75	75	65	30	87,1%	95,8%	98,8%	99,0%	95,0%
26147	75	75	65	35	87,2%	95,7%	98,8%	99,0%	95,3%
26148	75	75	65	40	87,4%	95,6%	98,9%	99,1%	95,6%
26149	75	75	65	45	87,6%	95,6%	98,9%	99,1%	95,9%
26150	75	75	65	50	87,8%	95,5%	98,9%	99,2%	96,2%
26151	75	75	65	55	88,0%	95,5%	99,0%	99,3%	96,4%
26152	75	75	65	60	88,2%	95,4%	99,0%	99,3%	96,6%
26153	75	75	65	65	88,4%	95,4%	99,0%	99,4%	96,9%
26154	75	75	65	70	88,6%	95,3%	99,1%	99,4%	97,1%
26155	75	75	65	75	88,7%	95,3%	99,1%	99,4%	97,2%
26156	75	75	65	80	88,9%	95,2%	99,1%	99,5%	97,4%
26157	75	75	70	20	83,5%	94,8%	98,5%	98,7%	94,1%
26158	75	75	70	25	83,8%	94,8%	98,6%	98,8%	94,5%
26159	75	75	70	30	84,0%	94,7%	98,6%	98,9%	94,8%
26160	75	75	70	35	84,2%	94,6%	98,7%	99,0%	95,1%
26161	75	75	70	40	84,5%	94,6%	98,7%	99,0%	95,5%
26162	75	75	70	45	84,7%	94,5%	98,8%	99,1%	95,7%
26163	75	75	70	50	84,9%	94,4%	98,8%	99,2%	96,0%
26164	75	75	70	55	85,2%	94,4%	98,8%	99,2%	96,3%
26165	75	75	70	60	85,4%	94,3%	98,9%	99,3%	96,5%
26166	75	75	70	65	85,6%	94,2%	98,9%	99,3%	96,7%
26167	75	75	70	70	85,8%	94,2%	98,9%	99,4%	96,9%
26168	75	75	70	75	86,0%	94,1%	99,0%	99,4%	97,1%

26169	75	75	70	80	86,2%	94,0%	99,0%	99,4%	97,3%
26170	75	75	75	20	79,8%	93,6%	98,3%	98,6%	93,9%
26171	75	75	75	25	80,1%	93,5%	98,4%	98,7%	94,3%
26172	75	75	75	30	80,4%	93,4%	98,4%	98,8%	94,6%
26173	75	75	75	35	80,7%	93,3%	98,5%	98,9%	95,0%
26174	75	75	75	40	81,0%	93,3%	98,5%	99,0%	95,3%
26175	75	75	75	45	81,2%	93,2%	98,6%	99,0%	95,6%
26176	75	75	75	50	81,5%	93,1%	98,6%	99,1%	95,9%
26177	75	75	75	55	81,8%	93,0%	98,7%	99,2%	96,1%
26178	75	75	75	60	82,0%	92,9%	98,7%	99,2%	96,4%
26179	75	75	75	65	82,3%	92,8%	98,8%	99,3%	96,6%
26180	75	75	75	70	82,5%	92,8%	98,8%	99,3%	96,8%
26181	75	75	75	75	82,8%	92,7%	98,8%	99,4%	97,0%
26182	75	75	75	80	83,0%	92,6%	98,9%	99,4%	97,2%
26183	75	75	80	20	75,6%	92,1%	98,1%	98,6%	93,6%
26184	75	75	80	25	75,9%	92,0%	98,2%	98,7%	94,0%
26185	75	75	80	30	76,2%	91,9%	98,2%	98,7%	94,4%
26186	75	75	80	35	76,5%	91,8%	98,3%	98,8%	94,8%
26187	75	75	80	40	76,9%	91,7%	98,4%	98,9%	95,1%
26188	75	75	80	45	77,2%	91,6%	98,4%	99,0%	95,4%
26189	75	75	80	50	77,5%	91,5%	98,5%	99,0%	95,7%
26190	75	75	80	55	77,8%	91,4%	98,5%	99,1%	96,0%
26191	75	75	80	60	78,1%	91,3%	98,6%	99,2%	96,2%
26192	75	75	80	65	78,4%	91,1%	98,6%	99,2%	96,5%
26193	75	75	80	70	78,7%	91,0%	98,7%	99,3%	96,7%
26194	75	75	80	75	79,0%	90,9%	98,7%	99,3%	96,9%
26195	75	75	80	80	79,3%	90,8%	98,7%	99,4%	97,1%
26196	75	80	20	20	98,5%	99,5%	99,6%	99,2%	95,6%
26197	75	80	20	25	98,5%	99,5%	99,6%	99,3%	95,9%
26198	75	80	20	30	98,5%	99,5%	99,6%	99,3%	96,2%
26199	75	80	20	35	98,6%	99,5%	99,6%	99,4%	96,4%
26200	75	80	20	40	98,6%	99,5%	99,6%	99,4%	96,6%
26201	75	80	20	45	98,6%	99,5%	99,6%	99,5%	96,9%

26202	75	80	20	50	98,6%	99,5%	99,6%	99,5%	97,1%
26203	75	80	20	55	98,7%	99,5%	99,7%	99,5%	97,3%
26204	75	80	20	60	98,7%	99,4%	99,7%	99,6%	97,4%
26205	75	80	20	65	98,7%	99,4%	99,7%	99,6%	97,6%
26206	75	80	20	70	98,7%	99,4%	99,7%	99,6%	97,8%
26207	75	80	20	75	98,8%	99,4%	99,7%	99,6%	97,9%
26208	75	80	20	80	98,8%	99,4%	99,7%	99,7%	98,0%
26209	75	80	25	20	98,1%	99,4%	99,5%	99,2%	95,5%
26210	75	80	25	25	98,1%	99,4%	99,5%	99,3%	95,8%
26211	75	80	25	30	98,1%	99,4%	99,5%	99,3%	96,0%
26212	75	80	25	35	98,2%	99,4%	99,5%	99,4%	96,3%
26213	75	80	25	40	98,2%	99,3%	99,6%	99,4%	96,5%
26214	75	80	25	45	98,2%	99,3%	99,6%	99,4%	96,7%
26215	75	80	25	50	98,3%	99,3%	99,6%	99,5%	96,9%
26216	75	80	25	55	98,3%	99,3%	99,6%	99,5%	97,1%
26217	75	80	25	60	98,3%	99,3%	99,6%	99,5%	97,3%
26218	75	80	25	65	98,4%	99,3%	99,6%	99,6%	97,5%
26219	75	80	25	70	98,4%	99,3%	99,6%	99,6%	97,7%
26220	75	80	25	75	98,4%	99,3%	99,7%	99,6%	97,8%
26221	75	80	25	80	98,4%	99,3%	99,7%	99,7%	98,0%
26222	75	80	30	20	97,6%	99,2%	99,4%	99,2%	95,3%
26223	75	80	30	25	97,6%	99,2%	99,5%	99,2%	95,6%
26224	75	80	30	30	97,6%	99,2%	99,5%	99,3%	95,9%
26225	75	80	30	35	97,7%	99,2%	99,5%	99,3%	96,1%
26226	75	80	30	40	97,7%	99,2%	99,5%	99,4%	96,4%
26227	75	80	30	45	97,8%	99,2%	99,5%	99,4%	96,6%
26228	75	80	30	50	97,8%	99,2%	99,5%	99,4%	96,8%
26229	75	80	30	55	97,8%	99,1%	99,6%	99,5%	97,0%
26230	75	80	30	60	97,9%	99,1%	99,6%	99,5%	97,2%
26231	75	80	30	65	97,9%	99,1%	99,6%	99,5%	97,4%
26232	75	80	30	70	97,9%	99,1%	99,6%	99,6%	97,6%
26233	75	80	30	75	98,0%	99,1%	99,6%	99,6%	97,7%
26234	75	80	30	80	98,0%	99,1%	99,6%	99,6%	97,9%

26235	75	80	35	20	96,9%	99,0%	99,4%	99,1%	95,1%
26236	75	80	35	25	96,9%	99,0%	99,4%	99,2%	95,4%
26237	75	80	35	30	97,0%	99,0%	99,4%	99,2%	95,7%
26238	75	80	35	35	97,0%	99,0%	99,4%	99,3%	96,0%
26239	75	80	35	40	97,1%	99,0%	99,4%	99,3%	96,2%
26240	75	80	35	45	97,1%	98,9%	99,5%	99,4%	96,5%
26241	75	80	35	50	97,2%	98,9%	99,5%	99,4%	96,7%
26242	75	80	35	55	97,2%	98,9%	99,5%	99,4%	96,9%
26243	75	80	35	60	97,3%	98,9%	99,5%	99,5%	97,1%
26244	75	80	35	65	97,3%	98,9%	99,5%	99,5%	97,3%
26245	75	80	35	70	97,4%	98,9%	99,5%	99,5%	97,5%
26246	75	80	35	75	97,4%	98,9%	99,6%	99,6%	97,6%
26247	75	80	35	80	97,5%	98,8%	99,6%	99,6%	97,8%
26248	75	80	40	20	96,1%	98,8%	99,3%	99,0%	94,9%
26249	75	80	40	25	96,1%	98,7%	99,3%	99,1%	95,2%
26250	75	80	40	30	96,2%	98,7%	99,3%	99,2%	95,5%
26251	75	80	40	35	96,3%	98,7%	99,3%	99,2%	95,8%
26252	75	80	40	40	96,3%	98,7%	99,4%	99,3%	96,1%
26253	75	80	40	45	96,4%	98,7%	99,4%	99,3%	96,3%
26254	75	80	40	50	96,4%	98,7%	99,4%	99,4%	96,6%
26255	75	80	40	55	96,5%	98,6%	99,4%	99,4%	96,8%
26256	75	80	40	60	96,6%	98,6%	99,4%	99,4%	97,0%
26257	75	80	40	65	96,6%	98,6%	99,5%	99,5%	97,2%
26258	75	80	40	70	96,7%	98,6%	99,5%	99,5%	97,4%
26259	75	80	40	75	96,7%	98,6%	99,5%	99,6%	97,5%
26260	75	80	40	80	96,8%	98,6%	99,5%	99,6%	97,7%
26261	75	80	45	20	95,0%	98,4%	99,2%	99,0%	94,7%
26262	75	80	45	25	95,1%	98,4%	99,2%	99,1%	95,0%
26263	75	80	45	30	95,2%	98,4%	99,2%	99,1%	95,3%
26264	75	80	45	35	95,3%	98,4%	99,3%	99,2%	95,6%
26265	75	80	45	40	95,3%	98,4%	99,3%	99,2%	95,9%
26266	75	80	45	45	95,4%	98,3%	99,3%	99,3%	96,2%
26267	75	80	45	50	95,5%	98,3%	99,3%	99,3%	96,4%

26268	75	80	45	55	95,6%	98,3%	99,4%	99,4%	96,6%
26269	75	80	45	60	95,6%	98,3%	99,4%	99,4%	96,9%
26270	75	80	45	65	95,7%	98,2%	99,4%	99,5%	97,1%
26271	75	80	45	70	95,8%	98,2%	99,4%	99,5%	97,3%
26272	75	80	45	75	95,9%	98,2%	99,4%	99,5%	97,4%
26273	75	80	45	80	95,9%	98,2%	99,5%	99,6%	97,6%
26274	75	80	50	20	93,7%	98,0%	99,1%	98,9%	94,5%
26275	75	80	50	25	93,8%	98,0%	99,1%	99,0%	94,8%
26276	75	80	50	30	93,9%	98,0%	99,1%	99,1%	95,2%
26277	75	80	50	35	94,0%	98,0%	99,2%	99,1%	95,5%
26278	75	80	50	40	94,1%	97,9%	99,2%	99,2%	95,7%
26279	75	80	50	45	94,2%	97,9%	99,2%	99,2%	96,0%
26280	75	80	50	50	94,3%	97,9%	99,2%	99,3%	96,3%
26281	75	80	50	55	94,4%	97,9%	99,3%	99,3%	96,5%
26282	75	80	50	60	94,5%	97,8%	99,3%	99,4%	96,7%
26283	75	80	50	65	94,6%	97,8%	99,3%	99,4%	96,9%
26284	75	80	50	70	94,7%	97,8%	99,3%	99,5%	97,1%
26285	75	80	50	75	94,8%	97,7%	99,4%	99,5%	97,3%
26286	75	80	50	80	94,8%	97,7%	99,4%	99,5%	97,5%
26287	75	80	55	20	92,1%	97,5%	98,9%	98,9%	94,3%
26288	75	80	55	25	92,2%	97,5%	99,0%	98,9%	94,6%
26289	75	80	55	30	92,3%	97,5%	99,0%	99,0%	95,0%
26290	75	80	55	35	92,5%	97,5%	99,0%	99,1%	95,3%
26291	75	80	55	40	92,6%	97,4%	99,1%	99,1%	95,6%
26292	75	80	55	45	92,7%	97,4%	99,1%	99,2%	95,9%
26293	75	80	55	50	92,8%	97,4%	99,1%	99,2%	96,1%
26294	75	80	55	55	92,9%	97,3%	99,2%	99,3%	96,4%
26295	75	80	55	60	93,0%	97,3%	99,2%	99,3%	96,6%
26296	75	80	55	65	93,2%	97,2%	99,2%	99,4%	96,8%
26297	75	80	55	70	93,3%	97,2%	99,2%	99,4%	97,0%
26298	75	80	55	75	93,4%	97,2%	99,3%	99,5%	97,2%
26299	75	80	55	80	93,5%	97,1%	99,3%	99,5%	97,4%
26300	75	80	60	20	90,1%	96,9%	98,8%	98,8%	94,0%

26301	75	80	60	25	90,2%	96,9%	98,9%	98,9%	94,4%
26302	75	80	60	30	90,4%	96,9%	98,9%	98,9%	94,8%
26303	75	80	60	35	90,5%	96,8%	98,9%	99,0%	95,1%
26304	75	80	60	40	90,7%	96,8%	99,0%	99,1%	95,4%
26305	75	80	60	45	90,8%	96,7%	99,0%	99,1%	95,7%
26306	75	80	60	50	91,0%	96,7%	99,0%	99,2%	96,0%
26307	75	80	60	55	91,1%	96,6%	99,1%	99,2%	96,2%
26308	75	80	60	60	91,3%	96,6%	99,1%	99,3%	96,5%
26309	75	80	60	65	91,4%	96,6%	99,1%	99,3%	96,7%
26310	75	80	60	70	91,5%	96,5%	99,2%	99,4%	96,9%
26311	75	80	60	75	91,7%	96,5%	99,2%	99,4%	97,1%
26312	75	80	60	80	91,8%	96,4%	99,2%	99,5%	97,3%
26313	75	80	65	20	87,6%	96,2%	98,7%	98,7%	93,8%
26314	75	80	65	25	87,8%	96,1%	98,7%	98,8%	94,2%
26315	75	80	65	30	88,0%	96,1%	98,7%	98,9%	94,5%
26316	75	80	65	35	88,2%	96,0%	98,8%	98,9%	94,9%
26317	75	80	65	40	88,4%	96,0%	98,8%	99,0%	95,2%
26318	75	80	65	45	88,6%	95,9%	98,9%	99,1%	95,5%
26319	75	80	65	50	88,7%	95,9%	98,9%	99,1%	95,8%
26320	75	80	65	55	88,9%	95,8%	98,9%	99,2%	96,1%
26321	75	80	65	60	89,1%	95,8%	99,0%	99,3%	96,3%
26322	75	80	65	65	89,3%	95,7%	99,0%	99,3%	96,6%
26323	75	80	65	70	89,4%	95,7%	99,0%	99,3%	96,8%
26324	75	80	65	75	89,6%	95,6%	99,1%	99,4%	97,0%
26325	75	80	65	80	89,8%	95,5%	99,1%	99,4%	97,2%
26326	75	80	70	20	84,7%	95,2%	98,5%	98,6%	93,6%
26327	75	80	70	25	84,9%	95,2%	98,5%	98,7%	94,0%
26328	75	80	70	30	85,2%	95,1%	98,6%	98,8%	94,3%
26329	75	80	70	35	85,4%	95,0%	98,6%	98,9%	94,7%
26330	75	80	70	40	85,6%	95,0%	98,7%	99,0%	95,0%
26331	75	80	70	45	85,8%	94,9%	98,7%	99,0%	95,3%
26332	75	80	70	50	86,0%	94,8%	98,8%	99,1%	95,6%
26333	75	80	70	55	86,2%	94,8%	98,8%	99,1%	95,9%

26334	75	80	70	60	86,4%	94,7%	98,8%	99,2%	96,2%
26335	75	80	70	65	86,7%	94,6%	98,9%	99,3%	96,4%
26336	75	80	70	70	86,9%	94,6%	98,9%	99,3%	96,6%
26337	75	80	70	75	87,1%	94,5%	98,9%	99,4%	96,9%
26338	75	80	70	80	87,3%	94,4%	99,0%	99,4%	97,1%
26339	75	80	75	20	81,2%	94,0%	98,3%	98,5%	93,3%
26340	75	80	75	25	81,5%	94,0%	98,3%	98,6%	93,7%
26341	75	80	75	30	81,8%	93,9%	98,4%	98,7%	94,1%
26342	75	80	75	35	82,0%	93,8%	98,4%	98,8%	94,5%
26343	75	80	75	40	82,3%	93,7%	98,5%	98,9%	94,8%
26344	75	80	75	45	82,5%	93,7%	98,5%	99,0%	95,1%
26345	75	80	75	50	82,8%	93,6%	98,6%	99,0%	95,5%
26346	75	80	75	55	83,0%	93,5%	98,6%	99,1%	95,7%
26347	75	80	75	60	83,3%	93,4%	98,7%	99,2%	96,0%
26348	75	80	75	65	83,5%	93,4%	98,7%	99,2%	96,3%
26349	75	80	75	70	83,8%	93,3%	98,8%	99,3%	96,5%
26350	75	80	75	75	84,0%	93,2%	98,8%	99,3%	96,7%
26351	75	80	75	80	84,2%	93,1%	98,9%	99,4%	96,9%
26352	75	80	80	20	77,2%	92,6%	98,1%	98,5%	93,0%
26353	75	80	80	25	77,5%	92,5%	98,1%	98,6%	93,5%
26354	75	80	80	30	77,8%	92,4%	98,2%	98,6%	93,9%
26355	75	80	80	35	78,1%	92,3%	98,2%	98,7%	94,3%
26356	75	80	80	40	78,4%	92,2%	98,3%	98,8%	94,6%
26357	75	80	80	45	78,7%	92,2%	98,4%	98,9%	95,0%
26358	75	80	80	50	79,0%	92,1%	98,4%	99,0%	95,3%
26359	75	80	80	55	79,3%	92,0%	98,5%	99,0%	95,6%
26360	75	80	80	60	79,6%	91,9%	98,5%	99,1%	95,9%
26361	75	80	80	65	79,9%	91,8%	98,6%	99,2%	96,1%
26362	75	80	80	70	80,1%	91,7%	98,6%	99,2%	96,4%
26363	75	80	80	75	80,4%	91,6%	98,7%	99,3%	96,6%
26364	75	80	80	80	80,7%	91,5%	98,7%	99,3%	96,8%
26365	80	20	20	20	96,6%	99,1%	99,8%	99,8%	98,9%
26366	80	20	20	25	96,7%	99,1%	99,8%	99,8%	99,0%

26367	80	20	20	30	96,8%	99,1%	99,8%	99,8%	99,1%
26368	80	20	20	35	96,8%	99,1%	99,8%	99,8%	99,1%
26369	80	20	20	40	96,9%	99,0%	99,8%	99,8%	99,2%
26370	80	20	20	45	96,9%	99,0%	99,8%	99,9%	99,2%
26371	80	20	20	50	97,0%	99,0%	99,8%	99,9%	99,3%
26372	80	20	20	55	97,0%	99,0%	99,8%	99,9%	99,3%
26373	80	20	20	60	97,1%	99,0%	99,8%	99,9%	99,4%
26374	80	20	20	65	97,1%	99,0%	99,9%	99,9%	99,4%
26375	80	20	20	70	97,2%	99,0%	99,9%	99,9%	99,5%
26376	80	20	20	75	97,2%	98,9%	99,9%	99,9%	99,5%
26377	80	20	20	80	97,3%	98,9%	99,9%	99,9%	99,5%
26378	80	20	25	20	95,8%	98,9%	99,8%	99,8%	98,9%
26379	80	20	25	25	95,8%	98,8%	99,8%	99,8%	99,0%
26380	80	20	25	30	95,9%	98,8%	99,8%	99,8%	99,0%
26381	80	20	25	35	96,0%	98,8%	99,8%	99,8%	99,1%
26382	80	20	25	40	96,0%	98,8%	99,8%	99,8%	99,2%
26383	80	20	25	45	96,1%	98,8%	99,8%	99,8%	99,2%
26384	80	20	25	50	96,2%	98,8%	99,8%	99,9%	99,3%
26385	80	20	25	55	96,2%	98,7%	99,8%	99,9%	99,3%
26386	80	20	25	60	96,3%	98,7%	99,8%	99,9%	99,4%
26387	80	20	25	65	96,4%	98,7%	99,8%	99,9%	99,4%
26388	80	20	25	70	96,4%	98,7%	99,8%	99,9%	99,4%
26389	80	20	25	75	96,5%	98,7%	99,8%	99,9%	99,5%
26390	80	20	25	80	96,5%	98,7%	99,8%	99,9%	99,5%
26391	80	20	30	20	94,6%	98,6%	99,7%	99,8%	98,8%
26392	80	20	30	25	94,7%	98,5%	99,8%	99,8%	98,9%
26393	80	20	30	30	94,8%	98,5%	99,8%	99,8%	99,0%
26394	80	20	30	35	94,9%	98,5%	99,8%	99,8%	99,1%
26395	80	20	30	40	95,0%	98,5%	99,8%	99,8%	99,1%
26396	80	20	30	45	95,1%	98,5%	99,8%	99,8%	99,2%
26397	80	20	30	50	95,1%	98,4%	99,8%	99,8%	99,2%
26398	80	20	30	55	95,2%	98,4%	99,8%	99,9%	99,3%
26399	80	20	30	60	95,3%	98,4%	99,8%	99,9%	99,3%

26400	80	20	30	65	95,4%	98,4%	99,8%	99,9%	99,4%
26401	80	20	30	70	95,5%	98,4%	99,8%	99,9%	99,4%
26402	80	20	30	75	95,5%	98,3%	99,8%	99,9%	99,5%
26403	80	20	30	80	95,6%	98,3%	99,8%	99,9%	99,5%
26404	80	20	35	20	93,2%	98,2%	99,7%	99,8%	98,8%
26405	80	20	35	25	93,3%	98,2%	99,7%	99,8%	98,9%
26406	80	20	35	30	93,4%	98,1%	99,7%	99,8%	99,0%
26407	80	20	35	35	93,5%	98,1%	99,7%	99,8%	99,0%
26408	80	20	35	40	93,7%	98,1%	99,7%	99,8%	99,1%
26409	80	20	35	45	93,8%	98,1%	99,8%	99,8%	99,1%
26410	80	20	35	50	93,9%	98,1%	99,8%	99,8%	99,2%
26411	80	20	35	55	94,0%	98,0%	99,8%	99,8%	99,3%
26412	80	20	35	60	94,1%	98,0%	99,8%	99,9%	99,3%
26413	80	20	35	65	94,2%	98,0%	99,8%	99,9%	99,3%
26414	80	20	35	70	94,3%	97,9%	99,8%	99,9%	99,4%
26415	80	20	35	75	94,3%	97,9%	99,8%	99,9%	99,4%
26416	80	20	35	80	94,4%	97,9%	99,8%	99,9%	99,5%
26417	80	20	40	20	91,5%	97,7%	99,7%	99,7%	98,7%
26418	80	20	40	25	91,6%	97,7%	99,7%	99,8%	98,8%
26419	80	20	40	30	91,8%	97,7%	99,7%	99,8%	98,9%
26420	80	20	40	35	91,9%	97,6%	99,7%	99,8%	99,0%
26421	80	20	40	40	92,0%	97,6%	99,7%	99,8%	99,0%
26422	80	20	40	45	92,1%	97,6%	99,7%	99,8%	99,1%
26423	80	20	40	50	92,3%	97,6%	99,7%	99,8%	99,2%
26424	80	20	40	55	92,4%	97,5%	99,7%	99,8%	99,2%
26425	80	20	40	60	92,5%	97,5%	99,8%	99,8%	99,3%
26426	80	20	40	65	92,6%	97,5%	99,8%	99,9%	99,3%
26427	80	20	40	70	92,8%	97,4%	99,8%	99,9%	99,4%
26428	80	20	40	75	92,9%	97,4%	99,8%	99,9%	99,4%
26429	80	20	40	80	93,0%	97,4%	99,8%	99,9%	99,4%
26430	80	20	45	20	89,4%	97,2%	99,6%	99,7%	98,7%
26431	80	20	45	25	89,5%	97,1%	99,6%	99,7%	98,8%
26432	80	20	45	30	89,7%	97,1%	99,7%	99,8%	98,9%

26433	80	20	45	35	89,8%	97,1%	99,7%	99,8%	98,9%
26434	80	20	45	40	90,0%	97,0%	99,7%	99,8%	99,0%
26435	80	20	45	45	90,2%	97,0%	99,7%	99,8%	99,1%
26436	80	20	45	50	90,3%	96,9%	99,7%	99,8%	99,1%
26437	80	20	45	55	90,5%	96,9%	99,7%	99,8%	99,2%
26438	80	20	45	60	90,6%	96,9%	99,7%	99,8%	99,2%
26439	80	20	45	65	90,8%	96,8%	99,7%	99,8%	99,3%
26440	80	20	45	70	90,9%	96,8%	99,7%	99,9%	99,3%
26441	80	20	45	75	91,1%	96,7%	99,7%	99,9%	99,4%
26442	80	20	45	80	91,2%	96,7%	99,8%	99,9%	99,4%
26443	80	20	50	20	86,8%	96,5%	99,6%	99,7%	98,6%
26444	80	20	50	25	87,0%	96,4%	99,6%	99,7%	98,7%
26445	80	20	50	30	87,2%	96,4%	99,6%	99,7%	98,8%
26446	80	20	50	35	87,4%	96,3%	99,6%	99,8%	98,9%
26447	80	20	50	40	87,6%	96,3%	99,6%	99,8%	99,0%
26448	80	20	50	45	87,7%	96,2%	99,6%	99,8%	99,0%
26449	80	20	50	50	87,9%	96,2%	99,7%	99,8%	99,1%
26450	80	20	50	55	88,1%	96,1%	99,7%	99,8%	99,2%
26451	80	20	50	60	88,3%	96,1%	99,7%	99,8%	99,2%
26452	80	20	50	65	88,5%	96,0%	99,7%	99,8%	99,3%
26453	80	20	50	70	88,7%	96,0%	99,7%	99,8%	99,3%
26454	80	20	50	75	88,8%	95,9%	99,7%	99,9%	99,4%
26455	80	20	50	80	89,0%	95,9%	99,7%	99,9%	99,4%
26456	80	20	55	20	83,7%	95,6%	99,5%	99,7%	98,6%
26457	80	20	55	25	83,9%	95,5%	99,5%	99,7%	98,7%
26458	80	20	55	30	84,1%	95,5%	99,6%	99,7%	98,8%
26459	80	20	55	35	84,4%	95,4%	99,6%	99,7%	98,8%
26460	80	20	55	40	84,6%	95,3%	99,6%	99,8%	98,9%
26461	80	20	55	45	84,8%	95,3%	99,6%	99,8%	99,0%
26462	80	20	55	50	85,1%	95,2%	99,6%	99,8%	99,1%
26463	80	20	55	55	85,3%	95,2%	99,6%	99,8%	99,1%
26464	80	20	55	60	85,5%	95,1%	99,6%	99,8%	99,2%
26465	80	20	55	65	85,7%	95,0%	99,6%	99,8%	99,2%

26466	80	20	55	70	85,9%	95,0%	99,7%	99,8%	99,3%
26467	80	20	55	75	86,1%	94,9%	99,7%	99,9%	99,3%
26468	80	20	55	80	86,4%	94,9%	99,7%	99,9%	99,4%
26469	80	20	60	20	80,0%	94,5%	99,5%	99,7%	98,5%
26470	80	20	60	25	80,3%	94,4%	99,5%	99,7%	98,6%
26471	80	20	60	30	80,6%	94,4%	99,5%	99,7%	98,7%
26472	80	20	60	35	80,8%	94,3%	99,5%	99,7%	98,8%
26473	80	20	60	40	81,1%	94,2%	99,5%	99,7%	98,9%
26474	80	20	60	45	81,4%	94,1%	99,5%	99,8%	99,0%
26475	80	20	60	50	81,6%	94,1%	99,6%	99,8%	99,0%
26476	80	20	60	55	81,9%	94,0%	99,6%	99,8%	99,1%
26477	80	20	60	60	82,2%	93,9%	99,6%	99,8%	99,1%
26478	80	20	60	65	82,4%	93,8%	99,6%	99,8%	99,2%
26479	80	20	60	70	82,7%	93,8%	99,6%	99,8%	99,3%
26480	80	20	60	75	82,9%	93,7%	99,6%	99,8%	99,3%
26481	80	20	60	80	83,2%	93,6%	99,6%	99,9%	99,3%
26482	80	20	65	20	75,8%	93,2%	99,4%	99,6%	98,5%
26483	80	20	65	25	76,1%	93,1%	99,4%	99,7%	98,6%
26484	80	20	65	30	76,4%	93,0%	99,4%	99,7%	98,7%
26485	80	20	65	35	76,7%	92,9%	99,5%	99,7%	98,7%
26486	80	20	65	40	77,0%	92,8%	99,5%	99,7%	98,8%
26487	80	20	65	45	77,3%	92,7%	99,5%	99,7%	98,9%
26488	80	20	65	50	77,6%	92,6%	99,5%	99,8%	99,0%
26489	80	20	65	55	78,0%	92,5%	99,5%	99,8%	99,0%
26490	80	20	65	60	78,3%	92,5%	99,5%	99,8%	99,1%
26491	80	20	65	65	78,6%	92,4%	99,6%	99,8%	99,2%
26492	80	20	65	70	78,8%	92,3%	99,6%	99,8%	99,2%
26493	80	20	65	75	79,1%	92,2%	99,6%	99,8%	99,3%
26494	80	20	65	80	79,4%	92,1%	99,6%	99,8%	99,3%
26495	80	20	70	20	70,9%	91,5%	99,3%	99,6%	98,4%
26496	80	20	70	25	71,3%	91,4%	99,3%	99,6%	98,5%
26497	80	20	70	30	71,7%	91,3%	99,4%	99,7%	98,6%
26498	80	20	70	35	72,0%	91,2%	99,4%	99,7%	98,7%

26499	80	20	70	40	72,4%	91,1%	99,4%	99,7%	98,8%
26500	80	20	70	45	72,7%	91,0%	99,4%	99,7%	98,9%
26501	80	20	70	50	73,1%	90,9%	99,4%	99,7%	98,9%
26502	80	20	70	55	73,4%	90,8%	99,5%	99,8%	99,0%
26503	80	20	70	60	73,8%	90,7%	99,5%	99,8%	99,1%
26504	80	20	70	65	74,1%	90,6%	99,5%	99,8%	99,1%
26505	80	20	70	70	74,4%	90,5%	99,5%	99,8%	99,2%
26506	80	20	70	75	74,8%	90,3%	99,5%	99,8%	99,2%
26507	80	20	70	80	75,1%	90,2%	99,5%	99,8%	99,3%
26508	80	20	75	20	65,6%	89,6%	99,2%	99,6%	98,3%
26509	80	20	75	25	66,0%	89,4%	99,2%	99,6%	98,4%
26510	80	20	75	30	66,4%	89,3%	99,3%	99,6%	98,5%
26511	80	20	75	35	66,8%	89,2%	99,3%	99,7%	98,6%
26512	80	20	75	40	67,2%	89,1%	99,3%	99,7%	98,7%
26513	80	20	75	45	67,6%	88,9%	99,3%	99,7%	98,8%
26514	80	20	75	50	68,0%	88,8%	99,4%	99,7%	98,9%
26515	80	20	75	55	68,3%	88,7%	99,4%	99,7%	99,0%
26516	80	20	75	60	68,7%	88,5%	99,4%	99,8%	99,0%
26517	80	20	75	65	69,1%	88,4%	99,4%	99,8%	99,1%
26518	80	20	75	70	69,5%	88,3%	99,4%	99,8%	99,2%
26519	80	20	75	75	69,8%	88,1%	99,5%	99,8%	99,2%
26520	80	20	75	80	70,2%	88,0%	99,5%	99,8%	99,3%
26521	80	20	80	20	59,8%	87,2%	99,1%	99,6%	98,3%
26522	80	20	80	25	60,3%	87,0%	99,1%	99,6%	98,4%
26523	80	20	80	30	60,7%	86,9%	99,2%	99,6%	98,5%
26524	80	20	80	35	61,1%	86,7%	99,2%	99,7%	98,6%
26525	80	20	80	40	61,5%	86,6%	99,2%	99,7%	98,7%
26526	80	20	80	45	61,9%	86,4%	99,3%	99,7%	98,8%
26527	80	20	80	50	62,4%	86,3%	99,3%	99,7%	98,8%
26528	80	20	80	55	62,8%	86,1%	99,3%	99,7%	98,9%
26529	80	20	80	60	63,2%	86,0%	99,3%	99,8%	99,0%
26530	80	20	80	65	63,6%	85,8%	99,4%	99,8%	99,1%
26531	80	20	80	70	64,0%	85,7%	99,4%	99,8%	99,1%

26532	80	20	80	75	64,4%	85,5%	99,4%	99,8%	99,2%
26533	80	20	80	80	64,8%	85,3%	99,4%	99,8%	99,2%
26534	80	25	20	20	96,9%	99,2%	99,8%	99,8%	98,8%
26535	80	25	20	25	97,0%	99,1%	99,8%	99,8%	98,9%
26536	80	25	20	30	97,0%	99,1%	99,8%	99,8%	99,0%
26537	80	25	20	35	97,1%	99,1%	99,8%	99,8%	99,0%
26538	80	25	20	40	97,1%	99,1%	99,8%	99,8%	99,1%
26539	80	25	20	45	97,2%	99,1%	99,8%	99,8%	99,2%
26540	80	25	20	50	97,2%	99,1%	99,8%	99,9%	99,2%
26541	80	25	20	55	97,3%	99,1%	99,8%	99,9%	99,3%
26542	80	25	20	60	97,3%	99,1%	99,8%	99,9%	99,3%
26543	80	25	20	65	97,4%	99,1%	99,8%	99,9%	99,4%
26544	80	25	20	70	97,4%	99,0%	99,9%	99,9%	99,4%
26545	80	25	20	75	97,5%	99,0%	99,9%	99,9%	99,4%
26546	80	25	20	80	97,5%	99,0%	99,9%	99,9%	99,5%
26547	80	25	25	20	96,1%	98,9%	99,8%	99,8%	98,8%
26548	80	25	25	25	96,2%	98,9%	99,8%	99,8%	98,9%
26549	80	25	25	30	96,2%	98,9%	99,8%	99,8%	98,9%
26550	80	25	25	35	96,3%	98,9%	99,8%	99,8%	99,0%
26551	80	25	25	40	96,4%	98,9%	99,8%	99,8%	99,1%
26552	80	25	25	45	96,4%	98,9%	99,8%	99,8%	99,1%
26553	80	25	25	50	96,5%	98,9%	99,8%	99,8%	99,2%
26554	80	25	25	55	96,5%	98,8%	99,8%	99,9%	99,2%
26555	80	25	25	60	96,6%	98,8%	99,8%	99,9%	99,3%
26556	80	25	25	65	96,7%	98,8%	99,8%	99,9%	99,3%
26557	80	25	25	70	96,7%	98,8%	99,8%	99,9%	99,4%
26558	80	25	25	75	96,8%	98,8%	99,8%	99,9%	99,4%
26559	80	25	25	80	96,8%	98,8%	99,8%	99,9%	99,5%
26560	80	25	30	20	95,1%	98,7%	99,7%	99,7%	98,7%
26561	80	25	30	25	95,1%	98,7%	99,7%	99,8%	98,8%
26562	80	25	30	30	95,2%	98,6%	99,8%	99,8%	98,9%
26563	80	25	30	35	95,3%	98,6%	99,8%	99,8%	99,0%
26564	80	25	30	40	95,4%	98,6%	99,8%	99,8%	99,0%

26565	80	25	30	45	95,5%	98,6%	99,8%	99,8%	99,1%
26566	80	25	30	50	95,5%	98,6%	99,8%	99,8%	99,2%
26567	80	25	30	55	95,6%	98,5%	99,8%	99,8%	99,2%
26568	80	25	30	60	95,7%	98,5%	99,8%	99,9%	99,3%
26569	80	25	30	65	95,8%	98,5%	99,8%	99,9%	99,3%
26570	80	25	30	70	95,8%	98,5%	99,8%	99,9%	99,4%
26571	80	25	30	75	95,9%	98,5%	99,8%	99,9%	99,4%
26572	80	25	30	80	96,0%	98,4%	99,8%	99,9%	99,4%
26573	80	25	35	20	93,8%	98,3%	99,7%	99,7%	98,7%
26574	80	25	35	25	93,9%	98,3%	99,7%	99,7%	98,8%
26575	80	25	35	30	94,0%	98,3%	99,7%	99,8%	98,8%
26576	80	25	35	35	94,1%	98,3%	99,7%	99,8%	98,9%
26577	80	25	35	40	94,2%	98,2%	99,7%	99,8%	99,0%
26578	80	25	35	45	94,3%	98,2%	99,7%	99,8%	99,1%
26579	80	25	35	50	94,4%	98,2%	99,8%	99,8%	99,1%
26580	80	25	35	55	94,4%	98,2%	99,8%	99,8%	99,2%
26581	80	25	35	60	94,5%	98,1%	99,8%	99,8%	99,2%
26582	80	25	35	65	94,6%	98,1%	99,8%	99,9%	99,3%
26583	80	25	35	70	94,7%	98,1%	99,8%	99,9%	99,3%
26584	80	25	35	75	94,8%	98,1%	99,8%	99,9%	99,4%
26585	80	25	35	80	94,9%	98,1%	99,8%	99,9%	99,4%
26586	80	25	40	20	92,1%	97,9%	99,7%	99,7%	98,6%
26587	80	25	40	25	92,3%	97,9%	99,7%	99,7%	98,7%
26588	80	25	40	30	92,4%	97,8%	99,7%	99,8%	98,8%
26589	80	25	40	35	92,5%	97,8%	99,7%	99,8%	98,9%
26590	80	25	40	40	92,6%	97,8%	99,7%	99,8%	99,0%
26591	80	25	40	45	92,8%	97,8%	99,7%	99,8%	99,0%
26592	80	25	40	50	92,9%	97,7%	99,7%	99,8%	99,1%
26593	80	25	40	55	93,0%	97,7%	99,7%	99,8%	99,1%
26594	80	25	40	60	93,1%	97,7%	99,7%	99,8%	99,2%
26595	80	25	40	65	93,2%	97,6%	99,8%	99,8%	99,3%
26596	80	25	40	70	93,3%	97,6%	99,8%	99,9%	99,3%
26597	80	25	40	75	93,4%	97,6%	99,8%	99,9%	99,3%

26598	80	25	40	80	93,5%	97,6%	99,8%	99,9%	99,4%
26599	80	25	45	20	90,2%	97,4%	99,6%	99,7%	98,6%
26600	80	25	45	25	90,3%	97,3%	99,6%	99,7%	98,7%
26601	80	25	45	30	90,5%	97,3%	99,6%	99,7%	98,7%
26602	80	25	45	35	90,6%	97,3%	99,7%	99,8%	98,8%
26603	80	25	45	40	90,8%	97,2%	99,7%	99,8%	98,9%
26604	80	25	45	45	90,9%	97,2%	99,7%	99,8%	99,0%
26605	80	25	45	50	91,1%	97,2%	99,7%	99,8%	99,0%
26606	80	25	45	55	91,2%	97,1%	99,7%	99,8%	99,1%
26607	80	25	45	60	91,3%	97,1%	99,7%	99,8%	99,2%
26608	80	25	45	65	91,5%	97,1%	99,7%	99,8%	99,2%
26609	80	25	45	70	91,6%	97,0%	99,7%	99,8%	99,3%
26610	80	25	45	75	91,8%	97,0%	99,7%	99,9%	99,3%
26611	80	25	45	80	91,9%	96,9%	99,7%	99,9%	99,4%
26612	80	25	50	20	87,8%	96,7%	99,6%	99,7%	98,5%
26613	80	25	50	25	87,9%	96,7%	99,6%	99,7%	98,6%
26614	80	25	50	30	88,1%	96,6%	99,6%	99,7%	98,7%
26615	80	25	50	35	88,3%	96,6%	99,6%	99,7%	98,8%
26616	80	25	50	40	88,5%	96,5%	99,6%	99,8%	98,9%
26617	80	25	50	45	88,7%	96,5%	99,6%	99,8%	98,9%
26618	80	25	50	50	88,8%	96,5%	99,6%	99,8%	99,0%
26619	80	25	50	55	89,0%	96,4%	99,7%	99,8%	99,1%
26620	80	25	50	60	89,2%	96,4%	99,7%	99,8%	99,1%
26621	80	25	50	65	89,4%	96,3%	99,7%	99,8%	99,2%
26622	80	25	50	70	89,5%	96,3%	99,7%	99,8%	99,2%
26623	80	25	50	75	89,7%	96,2%	99,7%	99,8%	99,3%
26624	80	25	50	80	89,8%	96,2%	99,7%	99,9%	99,3%
26625	80	25	55	20	84,8%	95,9%	99,5%	99,7%	98,4%
26626	80	25	55	25	85,1%	95,8%	99,5%	99,7%	98,5%
26627	80	25	55	30	85,3%	95,8%	99,5%	99,7%	98,6%
26628	80	25	55	35	85,5%	95,7%	99,6%	99,7%	98,7%
26629	80	25	55	40	85,7%	95,7%	99,6%	99,7%	98,8%
26630	80	25	55	45	85,9%	95,6%	99,6%	99,8%	98,9%

26631	80	25	55	50	86,2%	95,6%	99,6%	99,8%	99,0%
26632	80	25	55	55	86,4%	95,5%	99,6%	99,8%	99,0%
26633	80	25	55	60	86,6%	95,5%	99,6%	99,8%	99,1%
26634	80	25	55	65	86,8%	95,4%	99,6%	99,8%	99,2%
26635	80	25	55	70	87,0%	95,3%	99,7%	99,8%	99,2%
26636	80	25	55	75	87,2%	95,3%	99,7%	99,8%	99,3%
26637	80	25	55	80	87,4%	95,2%	99,7%	99,9%	99,3%
26638	80	25	60	20	81,4%	94,9%	99,4%	99,6%	98,4%
26639	80	25	60	25	81,7%	94,8%	99,5%	99,7%	98,5%
26640	80	25	60	30	81,9%	94,8%	99,5%	99,7%	98,6%
26641	80	25	60	35	82,2%	94,7%	99,5%	99,7%	98,7%
26642	80	25	60	40	82,4%	94,6%	99,5%	99,7%	98,8%
26643	80	25	60	45	82,7%	94,6%	99,5%	99,7%	98,8%
26644	80	25	60	50	82,9%	94,5%	99,5%	99,8%	98,9%
26645	80	25	60	55	83,2%	94,4%	99,6%	99,8%	99,0%
26646	80	25	60	60	83,4%	94,4%	99,6%	99,8%	99,1%
26647	80	25	60	65	83,7%	94,3%	99,6%	99,8%	99,1%
26648	80	25	60	70	83,9%	94,2%	99,6%	99,8%	99,2%
26649	80	25	60	75	84,1%	94,1%	99,6%	99,8%	99,2%
26650	80	25	60	80	84,4%	94,1%	99,6%	99,8%	99,3%
26651	80	25	65	20	77,4%	93,6%	99,4%	99,6%	98,3%
26652	80	25	65	25	77,7%	93,6%	99,4%	99,6%	98,4%
26653	80	25	65	30	78,0%	93,5%	99,4%	99,7%	98,5%
26654	80	25	65	35	78,3%	93,4%	99,4%	99,7%	98,6%
26655	80	25	65	40	78,6%	93,3%	99,5%	99,7%	98,7%
26656	80	25	65	45	78,9%	93,2%	99,5%	99,7%	98,8%
26657	80	25	65	50	79,2%	93,2%	99,5%	99,7%	98,9%
26658	80	25	65	55	79,4%	93,1%	99,5%	99,8%	99,0%
26659	80	25	65	60	79,7%	93,0%	99,5%	99,8%	99,0%
26660	80	25	65	65	80,0%	92,9%	99,5%	99,8%	99,1%
26661	80	25	65	70	80,3%	92,8%	99,6%	99,8%	99,1%
26662	80	25	65	75	80,6%	92,7%	99,6%	99,8%	99,2%
26663	80	25	65	80	80,8%	92,6%	99,6%	99,8%	99,3%

26664	80	25	70	20	72,7%	92,1%	99,3%	99,6%	98,2%
26665	80	25	70	25	73,1%	92,0%	99,3%	99,6%	98,4%
26666	80	25	70	30	73,4%	91,9%	99,3%	99,6%	98,5%
26667	80	25	70	35	73,8%	91,8%	99,4%	99,7%	98,6%
26668	80	25	70	40	74,1%	91,7%	99,4%	99,7%	98,7%
26669	80	25	70	45	74,5%	91,6%	99,4%	99,7%	98,7%
26670	80	25	70	50	74,8%	91,5%	99,4%	99,7%	98,8%
26671	80	25	70	55	75,1%	91,4%	99,4%	99,7%	98,9%
26672	80	25	70	60	75,4%	91,3%	99,5%	99,8%	99,0%
26673	80	25	70	65	75,8%	91,2%	99,5%	99,8%	99,0%
26674	80	25	70	70	76,1%	91,1%	99,5%	99,8%	99,1%
26675	80	25	70	75	76,4%	91,0%	99,5%	99,8%	99,2%
26676	80	25	70	80	76,7%	90,9%	99,5%	99,8%	99,2%
26677	80	25	75	20	67,6%	90,3%	99,2%	99,6%	98,2%
26678	80	25	75	25	68,0%	90,2%	99,2%	99,6%	98,3%
26679	80	25	75	30	68,3%	90,0%	99,2%	99,6%	98,4%
26680	80	25	75	35	68,7%	89,9%	99,3%	99,6%	98,5%
26681	80	25	75	40	69,1%	89,8%	99,3%	99,7%	98,6%
26682	80	25	75	45	69,5%	89,7%	99,3%	99,7%	98,7%
26683	80	25	75	50	69,9%	89,6%	99,3%	99,7%	98,8%
26684	80	25	75	55	70,2%	89,4%	99,4%	99,7%	98,9%
26685	80	25	75	60	70,6%	89,3%	99,4%	99,7%	98,9%
26686	80	25	75	65	71,0%	89,2%	99,4%	99,8%	99,0%
26687	80	25	75	70	71,3%	89,1%	99,4%	99,8%	99,1%
26688	80	25	75	75	71,7%	88,9%	99,4%	99,8%	99,1%
26689	80	25	75	80	72,0%	88,8%	99,5%	99,8%	99,2%
26690	80	25	80	20	62,0%	88,0%	99,1%	99,5%	98,1%
26691	80	25	80	25	62,4%	87,9%	99,1%	99,6%	98,2%
26692	80	25	80	30	62,8%	87,8%	99,2%	99,6%	98,3%
26693	80	25	80	35	63,2%	87,6%	99,2%	99,6%	98,4%
26694	80	25	80	40	63,6%	87,5%	99,2%	99,6%	98,5%
26695	80	25	80	45	64,0%	87,3%	99,2%	99,7%	98,6%
26696	80	25	80	50	64,4%	87,2%	99,3%	99,7%	98,7%

26697	80	25	80	55	64,8%	87,0%	99,3%	99,7%	98,8%
26698	80	25	80	60	65,2%	86,9%	99,3%	99,7%	98,9%
26699	80	25	80	65	65,6%	86,7%	99,3%	99,8%	99,0%
26700	80	25	80	70	66,0%	86,6%	99,4%	99,8%	99,0%
26701	80	25	80	75	66,4%	86,4%	99,4%	99,8%	99,1%
26702	80	25	80	80	66,8%	86,3%	99,4%	99,8%	99,2%
26703	80	30	20	20	97,2%	99,2%	99,8%	99,8%	98,7%
26704	80	30	20	25	97,2%	99,2%	99,8%	99,8%	98,8%
26705	80	30	20	30	97,3%	99,2%	99,8%	99,8%	98,9%
26706	80	30	20	35	97,3%	99,2%	99,8%	99,8%	99,0%
26707	80	30	20	40	97,4%	99,2%	99,8%	99,8%	99,0%
26708	80	30	20	45	97,4%	99,2%	99,8%	99,8%	99,1%
26709	80	30	20	50	97,5%	99,2%	99,8%	99,8%	99,1%
26710	80	30	20	55	97,5%	99,1%	99,8%	99,9%	99,2%
26711	80	30	20	60	97,5%	99,1%	99,8%	99,9%	99,3%
26712	80	30	20	65	97,6%	99,1%	99,8%	99,9%	99,3%
26713	80	30	20	70	97,6%	99,1%	99,8%	99,9%	99,3%
26714	80	30	20	75	97,7%	99,1%	99,9%	99,9%	99,4%
26715	80	30	20	80	97,7%	99,1%	99,9%	99,9%	99,4%
26716	80	30	25	20	96,4%	99,0%	99,8%	99,7%	98,7%
26717	80	30	25	25	96,5%	99,0%	99,8%	99,8%	98,8%
26718	80	30	25	30	96,5%	99,0%	99,8%	99,8%	98,8%
26719	80	30	25	35	96,6%	99,0%	99,8%	99,8%	98,9%
26720	80	30	25	40	96,7%	99,0%	99,8%	99,8%	99,0%
26721	80	30	25	45	96,7%	99,0%	99,8%	99,8%	99,0%
26722	80	30	25	50	96,8%	98,9%	99,8%	99,8%	99,1%
26723	80	30	25	55	96,8%	98,9%	99,8%	99,8%	99,2%
26724	80	30	25	60	96,9%	98,9%	99,8%	99,9%	99,2%
26725	80	30	25	65	96,9%	98,9%	99,8%	99,9%	99,3%
26726	80	30	25	70	97,0%	98,9%	99,8%	99,9%	99,3%
26727	80	30	25	75	97,0%	98,9%	99,8%	99,9%	99,4%
26728	80	30	25	80	97,1%	98,9%	99,8%	99,9%	99,4%
26729	80	30	30	20	95,5%	98,8%	99,7%	99,7%	98,6%

26730	80	30	30	25	95,5%	98,8%	99,7%	99,7%	98,7%
26731	80	30	30	30	95,6%	98,7%	99,7%	99,8%	98,8%
26732	80	30	30	35	95,7%	98,7%	99,8%	99,8%	98,9%
26733	80	30	30	40	95,8%	98,7%	99,8%	99,8%	98,9%
26734	80	30	30	45	95,8%	98,7%	99,8%	99,8%	99,0%
26735	80	30	30	50	95,9%	98,7%	99,8%	99,8%	99,1%
26736	80	30	30	55	96,0%	98,7%	99,8%	99,8%	99,1%
26737	80	30	30	60	96,0%	98,6%	99,8%	99,8%	99,2%
26738	80	30	30	65	96,1%	98,6%	99,8%	99,9%	99,2%
26739	80	30	30	70	96,2%	98,6%	99,8%	99,9%	99,3%
26740	80	30	30	75	96,2%	98,6%	99,8%	99,9%	99,3%
26741	80	30	30	80	96,3%	98,6%	99,8%	99,9%	99,4%
26742	80	30	35	20	94,3%	98,5%	99,7%	99,7%	98,6%
26743	80	30	35	25	94,4%	98,4%	99,7%	99,7%	98,6%
26744	80	30	35	30	94,4%	98,4%	99,7%	99,7%	98,7%
26745	80	30	35	35	94,5%	98,4%	99,7%	99,8%	98,8%
26746	80	30	35	40	94,6%	98,4%	99,7%	99,8%	98,9%
26747	80	30	35	45	94,7%	98,4%	99,7%	99,8%	99,0%
26748	80	30	35	50	94,8%	98,3%	99,7%	99,8%	99,0%
26749	80	30	35	55	94,9%	98,3%	99,8%	99,8%	99,1%
26750	80	30	35	60	95,0%	98,3%	99,8%	99,8%	99,2%
26751	80	30	35	65	95,1%	98,3%	99,8%	99,8%	99,2%
26752	80	30	35	70	95,1%	98,2%	99,8%	99,9%	99,3%
26753	80	30	35	75	95,2%	98,2%	99,8%	99,9%	99,3%
26754	80	30	35	80	95,3%	98,2%	99,8%	99,9%	99,4%
26755	80	30	40	20	92,8%	98,1%	99,7%	99,7%	98,5%
26756	80	30	40	25	92,9%	98,0%	99,7%	99,7%	98,6%
26757	80	30	40	30	93,0%	98,0%	99,7%	99,7%	98,7%
26758	80	30	40	35	93,1%	98,0%	99,7%	99,8%	98,8%
26759	80	30	40	40	93,2%	98,0%	99,7%	99,8%	98,8%
26760	80	30	40	45	93,3%	97,9%	99,7%	99,8%	98,9%
26761	80	30	40	50	93,4%	97,9%	99,7%	99,8%	99,0%
26762	80	30	40	55	93,6%	97,9%	99,7%	99,8%	99,1%

26763	80	30	40	60	93,7%	97,9%	99,7%	99,8%	99,1%
26764	80	30	40	65	93,8%	97,8%	99,7%	99,8%	99,2%
26765	80	30	40	70	93,9%	97,8%	99,8%	99,8%	99,2%
26766	80	30	40	75	94,0%	97,8%	99,8%	99,9%	99,3%
26767	80	30	40	80	94,1%	97,7%	99,8%	99,9%	99,3%
26768	80	30	45	20	90,9%	97,6%	99,6%	99,7%	98,4%
26769	80	30	45	25	91,1%	97,5%	99,6%	99,7%	98,5%
26770	80	30	45	30	91,2%	97,5%	99,6%	99,7%	98,6%
26771	80	30	45	35	91,4%	97,5%	99,6%	99,7%	98,7%
26772	80	30	45	40	91,5%	97,4%	99,7%	99,8%	98,8%
26773	80	30	45	45	91,6%	97,4%	99,7%	99,8%	98,9%
26774	80	30	45	50	91,8%	97,4%	99,7%	99,8%	99,0%
26775	80	30	45	55	91,9%	97,3%	99,7%	99,8%	99,0%
26776	80	30	45	60	92,0%	97,3%	99,7%	99,8%	99,1%
26777	80	30	45	65	92,2%	97,3%	99,7%	99,8%	99,1%
26778	80	30	45	70	92,3%	97,2%	99,7%	99,8%	99,2%
26779	80	30	45	75	92,4%	97,2%	99,7%	99,8%	99,3%
26780	80	30	45	80	92,5%	97,2%	99,7%	99,9%	99,3%
26781	80	30	50	20	88,7%	97,0%	99,6%	99,7%	98,4%
26782	80	30	50	25	88,8%	96,9%	99,6%	99,7%	98,5%
26783	80	30	50	30	89,0%	96,9%	99,6%	99,7%	98,6%
26784	80	30	50	35	89,2%	96,8%	99,6%	99,7%	98,7%
26785	80	30	50	40	89,4%	96,8%	99,6%	99,7%	98,8%
26786	80	30	50	45	89,5%	96,8%	99,6%	99,8%	98,8%
26787	80	30	50	50	89,7%	96,7%	99,6%	99,8%	98,9%
26788	80	30	50	55	89,9%	96,7%	99,6%	99,8%	99,0%
26789	80	30	50	60	90,0%	96,6%	99,7%	99,8%	99,0%
26790	80	30	50	65	90,2%	96,6%	99,7%	99,8%	99,1%
26791	80	30	50	70	90,3%	96,5%	99,7%	99,8%	99,2%
26792	80	30	50	75	90,5%	96,5%	99,7%	99,8%	99,2%
26793	80	30	50	80	90,6%	96,5%	99,7%	99,8%	99,3%
26794	80	30	55	20	85,9%	96,2%	99,5%	99,6%	98,3%
26795	80	30	55	25	86,2%	96,1%	99,5%	99,7%	98,4%

26796	80	30	55	30	86,4%	96,1%	99,5%	99,7%	98,5%
26797	80	30	55	35	86,6%	96,0%	99,5%	99,7%	98,6%
26798	80	30	55	40	86,8%	96,0%	99,6%	99,7%	98,7%
26799	80	30	55	45	87,0%	95,9%	99,6%	99,7%	98,8%
26800	80	30	55	50	87,2%	95,9%	99,6%	99,8%	98,9%
26801	80	30	55	55	87,4%	95,8%	99,6%	99,8%	98,9%
26802	80	30	55	60	87,6%	95,8%	99,6%	99,8%	99,0%
26803	80	30	55	65	87,8%	95,7%	99,6%	99,8%	99,1%
26804	80	30	55	70	87,9%	95,7%	99,6%	99,8%	99,1%
26805	80	30	55	75	88,1%	95,6%	99,7%	99,8%	99,2%
26806	80	30	55	80	88,3%	95,6%	99,7%	99,8%	99,2%
26807	80	30	60	20	82,7%	95,3%	99,4%	99,6%	98,2%
26808	80	30	60	25	82,9%	95,2%	99,4%	99,6%	98,3%
26809	80	30	60	30	83,2%	95,1%	99,5%	99,7%	98,4%
26810	80	30	60	35	83,4%	95,1%	99,5%	99,7%	98,5%
26811	80	30	60	40	83,7%	95,0%	99,5%	99,7%	98,6%
26812	80	30	60	45	83,9%	94,9%	99,5%	99,7%	98,7%
26813	80	30	60	50	84,2%	94,9%	99,5%	99,7%	98,8%
26814	80	30	60	55	84,4%	94,8%	99,5%	99,8%	98,9%
26815	80	30	60	60	84,6%	94,8%	99,6%	99,8%	99,0%
26816	80	30	60	65	84,8%	94,7%	99,6%	99,8%	99,0%
26817	80	30	60	70	85,1%	94,6%	99,6%	99,8%	99,1%
26818	80	30	60	75	85,3%	94,6%	99,6%	99,8%	99,2%
26819	80	30	60	80	85,5%	94,5%	99,6%	99,8%	99,2%
26820	80	30	65	20	78,9%	94,1%	99,4%	99,6%	98,2%
26821	80	30	65	25	79,2%	94,0%	99,4%	99,6%	98,3%
26822	80	30	65	30	79,5%	93,9%	99,4%	99,6%	98,4%
26823	80	30	65	35	79,7%	93,9%	99,4%	99,7%	98,5%
26824	80	30	65	40	80,0%	93,8%	99,4%	99,7%	98,6%
26825	80	30	65	45	80,3%	93,7%	99,5%	99,7%	98,7%
26826	80	30	65	50	80,6%	93,6%	99,5%	99,7%	98,8%
26827	80	30	65	55	80,9%	93,6%	99,5%	99,7%	98,8%
26828	80	30	65	60	81,1%	93,5%	99,5%	99,8%	98,9%

26829	80	30	65	65	81,4%	93,4%	99,5%	99,8%	99,0%
26830	80	30	65	70	81,7%	93,3%	99,5%	99,8%	99,1%
26831	80	30	65	75	81,9%	93,2%	99,6%	99,8%	99,1%
26832	80	30	65	80	82,2%	93,2%	99,6%	99,8%	99,2%
26833	80	30	70	20	74,5%	92,7%	99,3%	99,6%	98,1%
26834	80	30	70	25	74,8%	92,6%	99,3%	99,6%	98,2%
26835	80	30	70	30	75,1%	92,5%	99,3%	99,6%	98,3%
26836	80	30	70	35	75,5%	92,4%	99,3%	99,6%	98,4%
26837	80	30	70	40	75,8%	92,3%	99,4%	99,7%	98,5%
26838	80	30	70	45	76,1%	92,2%	99,4%	99,7%	98,6%
26839	80	30	70	50	76,4%	92,1%	99,4%	99,7%	98,7%
26840	80	30	70	55	76,7%	92,0%	99,4%	99,7%	98,8%
26841	80	30	70	60	77,1%	91,9%	99,4%	99,7%	98,9%
26842	80	30	70	65	77,4%	91,8%	99,5%	99,8%	99,0%
26843	80	30	70	70	77,7%	91,7%	99,5%	99,8%	99,0%
26844	80	30	70	75	78,0%	91,6%	99,5%	99,8%	99,1%
26845	80	30	70	80	78,3%	91,5%	99,5%	99,8%	99,1%
26846	80	30	75	20	69,5%	90,9%	99,2%	99,5%	98,0%
26847	80	30	75	25	69,9%	90,8%	99,2%	99,6%	98,1%
26848	80	30	75	30	70,2%	90,7%	99,2%	99,6%	98,2%
26849	80	30	75	35	70,6%	90,6%	99,3%	99,6%	98,4%
26850	80	30	75	40	71,0%	90,5%	99,3%	99,6%	98,5%
26851	80	30	75	45	71,3%	90,4%	99,3%	99,7%	98,6%
26852	80	30	75	50	71,7%	90,3%	99,3%	99,7%	98,7%
26853	80	30	75	55	72,0%	90,2%	99,3%	99,7%	98,7%
26854	80	30	75	60	72,4%	90,0%	99,4%	99,7%	98,8%
26855	80	30	75	65	72,7%	89,9%	99,4%	99,7%	98,9%
26856	80	30	75	70	73,1%	89,8%	99,4%	99,8%	99,0%
26857	80	30	75	75	73,4%	89,7%	99,4%	99,8%	99,0%
26858	80	30	75	80	73,8%	89,6%	99,4%	99,8%	99,1%
26859	80	30	80	20	64,0%	88,8%	99,1%	99,5%	97,9%
26860	80	30	80	25	64,4%	88,7%	99,1%	99,5%	98,0%
26861	80	30	80	30	64,8%	88,6%	99,1%	99,6%	98,2%

26862	80	30	80	35	65,2%	88,5%	99,2%	99,6%	98,3%
26863	80	30	80	40	65,6%	88,3%	99,2%	99,6%	98,4%
26864	80	30	80	45	66,0%	88,2%	99,2%	99,6%	98,5%
26865	80	30	80	50	66,4%	88,0%	99,2%	99,7%	98,6%
26866	80	30	80	55	66,8%	87,9%	99,3%	99,7%	98,7%
26867	80	30	80	60	67,2%	87,8%	99,3%	99,7%	98,8%
26868	80	30	80	65	67,6%	87,6%	99,3%	99,7%	98,9%
26869	80	30	80	70	68,0%	87,5%	99,3%	99,7%	98,9%
26870	80	30	80	75	68,4%	87,3%	99,4%	99,8%	99,0%
26871	80	30	80	80	68,7%	87,2%	99,4%	99,8%	99,1%
26872	80	35	20	20	97,4%	99,3%	99,8%	99,7%	98,6%
26873	80	35	20	25	97,5%	99,3%	99,8%	99,8%	98,7%
26874	80	35	20	30	97,5%	99,3%	99,8%	99,8%	98,8%
26875	80	35	20	35	97,5%	99,3%	99,8%	99,8%	98,8%
26876	80	35	20	40	97,6%	99,2%	99,8%	99,8%	98,9%
26877	80	35	20	45	97,6%	99,2%	99,8%	99,8%	99,0%
26878	80	35	20	50	97,7%	99,2%	99,8%	99,8%	99,1%
26879	80	35	20	55	97,7%	99,2%	99,8%	99,8%	99,1%
26880	80	35	20	60	97,7%	99,2%	99,8%	99,9%	99,2%
26881	80	35	20	65	97,8%	99,2%	99,8%	99,9%	99,2%
26882	80	35	20	70	97,8%	99,2%	99,8%	99,9%	99,3%
26883	80	35	20	75	97,9%	99,2%	99,8%	99,9%	99,3%
26884	80	35	20	80	97,9%	99,2%	99,9%	99,9%	99,4%
26885	80	35	25	20	96,7%	99,1%	99,8%	99,7%	98,5%
26886	80	35	25	25	96,8%	99,1%	99,8%	99,7%	98,6%
26887	80	35	25	30	96,8%	99,1%	99,8%	99,8%	98,7%
26888	80	35	25	35	96,9%	99,1%	99,8%	99,8%	98,8%
26889	80	35	25	40	96,9%	99,0%	99,8%	99,8%	98,9%
26890	80	35	25	45	97,0%	99,0%	99,8%	99,8%	99,0%
26891	80	35	25	50	97,0%	99,0%	99,8%	99,8%	99,0%
26892	80	35	25	55	97,1%	99,0%	99,8%	99,8%	99,1%
26893	80	35	25	60	97,1%	99,0%	99,8%	99,8%	99,1%
26894	80	35	25	65	97,2%	99,0%	99,8%	99,9%	99,2%

26895	80	35	25	70	97,2%	99,0%	99,8%	99,9%	99,3%
26896	80	35	25	75	97,3%	99,0%	99,8%	99,9%	99,3%
26897	80	35	25	80	97,3%	98,9%	99,8%	99,9%	99,3%
26898	80	35	30	20	95,8%	98,9%	99,7%	99,7%	98,5%
26899	80	35	30	25	95,9%	98,8%	99,7%	99,7%	98,6%
26900	80	35	30	30	96,0%	98,8%	99,7%	99,7%	98,7%
26901	80	35	30	35	96,0%	98,8%	99,7%	99,8%	98,8%
26902	80	35	30	40	96,1%	98,8%	99,8%	99,8%	98,8%
26903	80	35	30	45	96,2%	98,8%	99,8%	99,8%	98,9%
26904	80	35	30	50	96,2%	98,8%	99,8%	99,8%	99,0%
26905	80	35	30	55	96,3%	98,8%	99,8%	99,8%	99,0%
26906	80	35	30	60	96,4%	98,7%	99,8%	99,8%	99,1%
26907	80	35	30	65	96,4%	98,7%	99,8%	99,8%	99,2%
26908	80	35	30	70	96,5%	98,7%	99,8%	99,9%	99,2%
26909	80	35	30	75	96,5%	98,7%	99,8%	99,9%	99,3%
26910	80	35	30	80	96,6%	98,7%	99,8%	99,9%	99,3%
26911	80	35	35	20	94,7%	98,6%	99,7%	99,7%	98,4%
26912	80	35	35	25	94,8%	98,6%	99,7%	99,7%	98,5%
26913	80	35	35	30	94,9%	98,5%	99,7%	99,7%	98,6%
26914	80	35	35	35	95,0%	98,5%	99,7%	99,7%	98,7%
26915	80	35	35	40	95,1%	98,5%	99,7%	99,8%	98,8%
26916	80	35	35	45	95,1%	98,5%	99,7%	99,8%	98,9%
26917	80	35	35	50	95,2%	98,5%	99,7%	99,8%	98,9%
26918	80	35	35	55	95,3%	98,4%	99,7%	99,8%	99,0%
26919	80	35	35	60	95,4%	98,4%	99,8%	99,8%	99,1%
26920	80	35	35	65	95,5%	98,4%	99,8%	99,8%	99,1%
26921	80	35	35	70	95,5%	98,4%	99,8%	99,8%	99,2%
26922	80	35	35	75	95,6%	98,4%	99,8%	99,9%	99,2%
26923	80	35	35	80	95,7%	98,3%	99,8%	99,9%	99,3%
26924	80	35	40	20	93,3%	98,2%	99,6%	99,7%	98,3%
26925	80	35	40	25	93,4%	98,2%	99,7%	99,7%	98,4%
26926	80	35	40	30	93,6%	98,2%	99,7%	99,7%	98,6%
26927	80	35	40	35	93,7%	98,1%	99,7%	99,7%	98,6%

26928	80	35	40	40	93,8%	98,1%	99,7%	99,7%	98,7%
26929	80	35	40	45	93,9%	98,1%	99,7%	99,8%	98,8%
26930	80	35	40	50	94,0%	98,1%	99,7%	99,8%	98,9%
26931	80	35	40	55	94,1%	98,0%	99,7%	99,8%	99,0%
26932	80	35	40	60	94,2%	98,0%	99,7%	99,8%	99,0%
26933	80	35	40	65	94,3%	98,0%	99,7%	99,8%	99,1%
26934	80	35	40	70	94,4%	98,0%	99,7%	99,8%	99,2%
26935	80	35	40	75	94,4%	97,9%	99,8%	99,8%	99,2%
26936	80	35	40	80	94,5%	97,9%	99,8%	99,9%	99,3%
26937	80	35	45	20	91,6%	97,7%	99,6%	99,6%	98,3%
26938	80	35	45	25	91,8%	97,7%	99,6%	99,7%	98,4%
26939	80	35	45	30	91,9%	97,7%	99,6%	99,7%	98,5%
26940	80	35	45	35	92,0%	97,7%	99,6%	99,7%	98,6%
26941	80	35	45	40	92,2%	97,6%	99,6%	99,7%	98,7%
26942	80	35	45	45	92,3%	97,6%	99,7%	99,8%	98,8%
26943	80	35	45	50	92,4%	97,6%	99,7%	99,8%	98,8%
26944	80	35	45	55	92,5%	97,5%	99,7%	99,8%	98,9%
26945	80	35	45	60	92,7%	97,5%	99,7%	99,8%	99,0%
26946	80	35	45	65	92,8%	97,5%	99,7%	99,8%	99,1%
26947	80	35	45	70	92,9%	97,4%	99,7%	99,8%	99,1%
26948	80	35	45	75	93,0%	97,4%	99,7%	99,8%	99,2%
26949	80	35	45	80	93,1%	97,4%	99,7%	99,8%	99,2%
26950	80	35	50	20	89,5%	97,2%	99,5%	99,6%	98,2%
26951	80	35	50	25	89,7%	97,1%	99,6%	99,7%	98,3%
26952	80	35	50	30	89,9%	97,1%	99,6%	99,7%	98,4%
26953	80	35	50	35	90,0%	97,1%	99,6%	99,7%	98,5%
26954	80	35	50	40	90,2%	97,0%	99,6%	99,7%	98,6%
26955	80	35	50	45	90,3%	97,0%	99,6%	99,7%	98,7%
26956	80	35	50	50	90,5%	97,0%	99,6%	99,8%	98,8%
26957	80	35	50	55	90,6%	96,9%	99,6%	99,8%	98,9%
26958	80	35	50	60	90,8%	96,9%	99,6%	99,8%	99,0%
26959	80	35	50	65	90,9%	96,8%	99,7%	99,8%	99,0%
26960	80	35	50	70	91,1%	96,8%	99,7%	99,8%	99,1%

26961	80	35	50	75	91,2%	96,8%	99,7%	99,8%	99,1%
26962	80	35	50	80	91,4%	96,7%	99,7%	99,8%	99,2%
26963	80	35	55	20	87,0%	96,5%	99,5%	99,6%	98,1%
26964	80	35	55	25	87,2%	96,4%	99,5%	99,6%	98,2%
26965	80	35	55	30	87,4%	96,4%	99,5%	99,7%	98,4%
26966	80	35	55	35	87,6%	96,3%	99,5%	99,7%	98,5%
26967	80	35	55	40	87,8%	96,3%	99,5%	99,7%	98,6%
26968	80	35	55	45	88,0%	96,2%	99,6%	99,7%	98,7%
26969	80	35	55	50	88,1%	96,2%	99,6%	99,7%	98,8%
26970	80	35	55	55	88,3%	96,1%	99,6%	99,8%	98,8%
26971	80	35	55	60	88,5%	96,1%	99,6%	99,8%	98,9%
26972	80	35	55	65	88,7%	96,0%	99,6%	99,8%	99,0%
26973	80	35	55	70	88,9%	96,0%	99,6%	99,8%	99,0%
26974	80	35	55	75	89,0%	95,9%	99,6%	99,8%	99,1%
26975	80	35	55	80	89,2%	95,9%	99,7%	99,8%	99,2%
26976	80	35	60	20	83,9%	95,6%	99,4%	99,6%	98,0%
26977	80	35	60	25	84,2%	95,5%	99,4%	99,6%	98,2%
26978	80	35	60	30	84,4%	95,5%	99,4%	99,6%	98,3%
26979	80	35	60	35	84,6%	95,4%	99,5%	99,7%	98,4%
26980	80	35	60	40	84,9%	95,4%	99,5%	99,7%	98,5%
26981	80	35	60	45	85,1%	95,3%	99,5%	99,7%	98,6%
26982	80	35	60	50	85,3%	95,3%	99,5%	99,7%	98,7%
26983	80	35	60	55	85,5%	95,2%	99,5%	99,7%	98,8%
26984	80	35	60	60	85,7%	95,1%	99,5%	99,8%	98,9%
26985	80	35	60	65	86,0%	95,1%	99,6%	99,8%	98,9%
26986	80	35	60	70	86,2%	95,0%	99,6%	99,8%	99,0%
26987	80	35	60	75	86,4%	95,0%	99,6%	99,8%	99,1%
26988	80	35	60	80	86,6%	94,9%	99,6%	99,8%	99,1%
26989	80	35	65	20	80,3%	94,5%	99,3%	99,6%	98,0%
26990	80	35	65	25	80,6%	94,4%	99,4%	99,6%	98,1%
26991	80	35	65	30	80,9%	94,4%	99,4%	99,6%	98,2%
26992	80	35	65	35	81,1%	94,3%	99,4%	99,6%	98,3%
26993	80	35	65	40	81,4%	94,2%	99,4%	99,7%	98,4%

26994	80	35	65	45	81,7%	94,2%	99,4%	99,7%	98,5%
26995	80	35	65	50	81,9%	94,1%	99,5%	99,7%	98,6%
26996	80	35	65	55	82,2%	94,0%	99,5%	99,7%	98,7%
26997	80	35	65	60	82,4%	93,9%	99,5%	99,7%	98,8%
26998	80	35	65	65	82,7%	93,9%	99,5%	99,8%	98,9%
26999	80	35	65	70	82,9%	93,8%	99,5%	99,8%	99,0%
27000	80	35	65	75	83,2%	93,7%	99,5%	99,8%	99,0%
27001	80	35	65	80	83,4%	93,6%	99,6%	99,8%	99,1%
27002	80	35	70	20	76,1%	93,2%	99,2%	99,5%	97,9%
27003	80	35	70	25	76,4%	93,1%	99,3%	99,6%	98,0%
27004	80	35	70	30	76,7%	93,0%	99,3%	99,6%	98,2%
27005	80	35	70	35	77,1%	92,9%	99,3%	99,6%	98,3%
27006	80	35	70	40	77,4%	92,8%	99,3%	99,6%	98,4%
27007	80	35	70	45	77,7%	92,8%	99,4%	99,7%	98,5%
27008	80	35	70	50	78,0%	92,7%	99,4%	99,7%	98,6%
27009	80	35	70	55	78,3%	92,6%	99,4%	99,7%	98,7%
27010	80	35	70	60	78,6%	92,5%	99,4%	99,7%	98,8%
27011	80	35	70	65	78,9%	92,4%	99,4%	99,7%	98,8%
27012	80	35	70	70	79,2%	92,3%	99,5%	99,8%	98,9%
27013	80	35	70	75	79,5%	92,2%	99,5%	99,8%	99,0%
27014	80	35	70	80	79,7%	92,1%	99,5%	99,8%	99,1%
27015	80	35	75	20	71,3%	91,6%	99,1%	99,5%	97,8%
27016	80	35	75	25	71,7%	91,5%	99,2%	99,5%	97,9%
27017	80	35	75	30	72,1%	91,4%	99,2%	99,6%	98,1%
27018	80	35	75	35	72,4%	91,3%	99,2%	99,6%	98,2%
27019	80	35	75	40	72,8%	91,2%	99,3%	99,6%	98,3%
27020	80	35	75	45	73,1%	91,0%	99,3%	99,6%	98,4%
27021	80	35	75	50	73,5%	90,9%	99,3%	99,7%	98,5%
27022	80	35	75	55	73,8%	90,8%	99,3%	99,7%	98,6%
27023	80	35	75	60	74,1%	90,7%	99,3%	99,7%	98,7%
27024	80	35	75	65	74,5%	90,6%	99,4%	99,7%	98,8%
27025	80	35	75	70	74,8%	90,5%	99,4%	99,7%	98,9%
27026	80	35	75	75	75,1%	90,4%	99,4%	99,8%	99,0%

27027	80	35	75	80	75,5%	90,3%	99,4%	99,8%	99,0%
27028	80	35	80	20	66,0%	89,6%	99,0%	99,5%	97,7%
27029	80	35	80	25	66,4%	89,5%	99,1%	99,5%	97,9%
27030	80	35	80	30	66,8%	89,4%	99,1%	99,5%	98,0%
27031	80	35	80	35	67,2%	89,2%	99,1%	99,6%	98,1%
27032	80	35	80	40	67,6%	89,1%	99,2%	99,6%	98,2%
27033	80	35	80	45	68,0%	89,0%	99,2%	99,6%	98,4%
27034	80	35	80	50	68,4%	88,9%	99,2%	99,6%	98,5%
27035	80	35	80	55	68,8%	88,7%	99,2%	99,7%	98,6%
27036	80	35	80	60	69,1%	88,6%	99,3%	99,7%	98,7%
27037	80	35	80	65	69,5%	88,5%	99,3%	99,7%	98,7%
27038	80	35	80	70	69,9%	88,3%	99,3%	99,7%	98,8%
27039	80	35	80	75	70,2%	88,2%	99,3%	99,7%	98,9%
27040	80	35	80	80	70,6%	88,0%	99,4%	99,8%	99,0%
27041	80	40	20	20	97,6%	99,3%	99,8%	99,7%	98,5%
27042	80	40	20	25	97,7%	99,3%	99,8%	99,7%	98,6%
27043	80	40	20	30	97,7%	99,3%	99,8%	99,8%	98,6%
27044	80	40	20	35	97,7%	99,3%	99,8%	99,8%	98,7%
27045	80	40	20	40	97,8%	99,3%	99,8%	99,8%	98,8%
27046	80	40	20	45	97,8%	99,3%	99,8%	99,8%	98,9%
27047	80	40	20	50	97,9%	99,3%	99,8%	99,8%	99,0%
27048	80	40	20	55	97,9%	99,3%	99,8%	99,8%	99,0%
27049	80	40	20	60	97,9%	99,3%	99,8%	99,8%	99,1%
27050	80	40	20	65	98,0%	99,3%	99,8%	99,9%	99,2%
27051	80	40	20	70	98,0%	99,2%	99,8%	99,9%	99,2%
27052	80	40	20	75	98,0%	99,2%	99,8%	99,9%	99,3%
27053	80	40	20	80	98,1%	99,2%	99,8%	99,9%	99,3%
27054	80	40	25	20	97,0%	99,2%	99,7%	99,7%	98,4%
27055	80	40	25	25	97,0%	99,2%	99,8%	99,7%	98,5%
27056	80	40	25	30	97,1%	99,1%	99,8%	99,7%	98,6%
27057	80	40	25	35	97,1%	99,1%	99,8%	99,8%	98,7%
27058	80	40	25	40	97,2%	99,1%	99,8%	99,8%	98,8%
27059	80	40	25	45	97,2%	99,1%	99,8%	99,8%	98,8%

27060	80	40	25	50	97,3%	99,1%	99,8%	99,8%	98,9%
27061	80	40	25	55	97,3%	99,1%	99,8%	99,8%	99,0%
27062	80	40	25	60	97,4%	99,1%	99,8%	99,8%	99,1%
27063	80	40	25	65	97,4%	99,1%	99,8%	99,8%	99,1%
27064	80	40	25	70	97,5%	99,0%	99,8%	99,9%	99,2%
27065	80	40	25	75	97,5%	99,0%	99,8%	99,9%	99,2%
27066	80	40	25	80	97,5%	99,0%	99,8%	99,9%	99,3%
27067	80	40	30	20	96,2%	98,9%	99,7%	99,7%	98,3%
27068	80	40	30	25	96,2%	98,9%	99,7%	99,7%	98,4%
27069	80	40	30	30	96,3%	98,9%	99,7%	99,7%	98,5%
27070	80	40	30	35	96,4%	98,9%	99,7%	99,7%	98,6%
27071	80	40	30	40	96,4%	98,9%	99,7%	99,8%	98,7%
27072	80	40	30	45	96,5%	98,9%	99,8%	99,8%	98,8%
27073	80	40	30	50	96,5%	98,9%	99,8%	99,8%	98,9%
27074	80	40	30	55	96,6%	98,8%	99,8%	99,8%	99,0%
27075	80	40	30	60	96,7%	98,8%	99,8%	99,8%	99,0%
27076	80	40	30	65	96,7%	98,8%	99,8%	99,8%	99,1%
27077	80	40	30	70	96,8%	98,8%	99,8%	99,8%	99,1%
27078	80	40	30	75	96,8%	98,8%	99,8%	99,9%	99,2%
27079	80	40	30	80	96,9%	98,8%	99,8%	99,9%	99,3%
27080	80	40	35	20	95,1%	98,7%	99,7%	99,7%	98,3%
27081	80	40	35	25	95,2%	98,7%	99,7%	99,7%	98,4%
27082	80	40	35	30	95,3%	98,6%	99,7%	99,7%	98,5%
27083	80	40	35	35	95,4%	98,6%	99,7%	99,7%	98,6%
27084	80	40	35	40	95,5%	98,6%	99,7%	99,7%	98,7%
27085	80	40	35	45	95,5%	98,6%	99,7%	99,8%	98,8%
27086	80	40	35	50	95,6%	98,6%	99,7%	99,8%	98,8%
27087	80	40	35	55	95,7%	98,6%	99,7%	99,8%	98,9%
27088	80	40	35	60	95,8%	98,5%	99,7%	99,8%	99,0%
27089	80	40	35	65	95,8%	98,5%	99,8%	99,8%	99,0%
27090	80	40	35	70	95,9%	98,5%	99,8%	99,8%	99,1%
27091	80	40	35	75	96,0%	98,5%	99,8%	99,8%	99,2%
27092	80	40	35	80	96,0%	98,5%	99,8%	99,9%	99,2%

27093	80	40	40	20	93,9%	98,3%	99,6%	99,6%	98,2%
27094	80	40	40	25	94,0%	98,3%	99,6%	99,7%	98,3%
27095	80	40	40	30	94,1%	98,3%	99,7%	99,7%	98,4%
27096	80	40	40	35	94,2%	98,3%	99,7%	99,7%	98,5%
27097	80	40	40	40	94,3%	98,3%	99,7%	99,7%	98,6%
27098	80	40	40	45	94,4%	98,2%	99,7%	99,7%	98,7%
27099	80	40	40	50	94,5%	98,2%	99,7%	99,8%	98,8%
27100	80	40	40	55	94,5%	98,2%	99,7%	99,8%	98,9%
27101	80	40	40	60	94,6%	98,2%	99,7%	99,8%	98,9%
27102	80	40	40	65	94,7%	98,1%	99,7%	99,8%	99,0%
27103	80	40	40	70	94,8%	98,1%	99,7%	99,8%	99,1%
27104	80	40	40	75	94,9%	98,1%	99,7%	99,8%	99,1%
27105	80	40	40	80	95,0%	98,1%	99,8%	99,8%	99,2%
27106	80	40	45	20	92,3%	97,9%	99,6%	99,6%	98,1%
27107	80	40	45	25	92,4%	97,9%	99,6%	99,6%	98,2%
27108	80	40	45	30	92,5%	97,9%	99,6%	99,7%	98,3%
27109	80	40	45	35	92,7%	97,8%	99,6%	99,7%	98,5%
27110	80	40	45	40	92,8%	97,8%	99,6%	99,7%	98,6%
27111	80	40	45	45	92,9%	97,8%	99,6%	99,7%	98,6%
27112	80	40	45	50	93,0%	97,7%	99,7%	99,8%	98,7%
27113	80	40	45	55	93,1%	97,7%	99,7%	99,8%	98,8%
27114	80	40	45	60	93,2%	97,7%	99,7%	99,8%	98,9%
27115	80	40	45	65	93,3%	97,7%	99,7%	99,8%	99,0%
27116	80	40	45	70	93,5%	97,6%	99,7%	99,8%	99,0%
27117	80	40	45	75	93,6%	97,6%	99,7%	99,8%	99,1%
27118	80	40	45	80	93,7%	97,6%	99,7%	99,8%	99,2%
27119	80	40	50	20	90,3%	97,4%	99,5%	99,6%	98,0%
27120	80	40	50	25	90,5%	97,4%	99,5%	99,6%	98,2%
27121	80	40	50	30	90,6%	97,3%	99,6%	99,7%	98,3%
27122	80	40	50	35	90,8%	97,3%	99,6%	99,7%	98,4%
27123	80	40	50	40	90,9%	97,3%	99,6%	99,7%	98,5%
27124	80	40	50	45	91,1%	97,2%	99,6%	99,7%	98,6%
27125	80	40	50	50	91,2%	97,2%	99,6%	99,7%	98,7%

27126	80	40	50	55	91,4%	97,1%	99,6%	99,8%	98,8%
27127	80	40	50	60	91,5%	97,1%	99,6%	99,8%	98,8%
27128	80	40	50	65	91,6%	97,1%	99,6%	99,8%	98,9%
27129	80	40	50	70	91,8%	97,0%	99,7%	99,8%	99,0%
27130	80	40	50	75	91,9%	97,0%	99,7%	99,8%	99,1%
27131	80	40	50	80	92,0%	97,0%	99,7%	99,8%	99,1%
27132	80	40	55	20	88,0%	96,7%	99,5%	99,6%	97,9%
27133	80	40	55	25	88,1%	96,7%	99,5%	99,6%	98,1%
27134	80	40	55	30	88,3%	96,6%	99,5%	99,6%	98,2%
27135	80	40	55	35	88,5%	96,6%	99,5%	99,7%	98,3%
27136	80	40	55	40	88,7%	96,6%	99,5%	99,7%	98,4%
27137	80	40	55	45	88,9%	96,5%	99,5%	99,7%	98,5%
27138	80	40	55	50	89,0%	96,5%	99,6%	99,7%	98,6%
27139	80	40	55	55	89,2%	96,4%	99,6%	99,7%	98,7%
27140	80	40	55	60	89,4%	96,4%	99,6%	99,8%	98,8%
27141	80	40	55	65	89,5%	96,3%	99,6%	99,8%	98,9%
27142	80	40	55	70	89,7%	96,3%	99,6%	99,8%	99,0%
27143	80	40	55	75	89,9%	96,2%	99,6%	99,8%	99,0%
27144	80	40	55	80	90,0%	96,2%	99,6%	99,8%	99,1%
27145	80	40	60	20	85,1%	95,9%	99,4%	99,5%	97,9%
27146	80	40	60	25	85,3%	95,9%	99,4%	99,6%	98,0%
27147	80	40	60	30	85,5%	95,8%	99,4%	99,6%	98,1%
27148	80	40	60	35	85,7%	95,8%	99,4%	99,6%	98,2%
27149	80	40	60	40	86,0%	95,7%	99,5%	99,7%	98,4%
27150	80	40	60	45	86,2%	95,7%	99,5%	99,7%	98,5%
27151	80	40	60	50	86,4%	95,6%	99,5%	99,7%	98,6%
27152	80	40	60	55	86,6%	95,5%	99,5%	99,7%	98,7%
27153	80	40	60	60	86,8%	95,5%	99,5%	99,7%	98,8%
27154	80	40	60	65	87,0%	95,4%	99,6%	99,8%	98,8%
27155	80	40	60	70	87,2%	95,4%	99,6%	99,8%	98,9%
27156	80	40	60	75	87,4%	95,3%	99,6%	99,8%	99,0%
27157	80	40	60	80	87,6%	95,3%	99,6%	99,8%	99,0%
27158	80	40	65	20	81,7%	94,9%	99,3%	99,5%	97,8%

27159	80	40	65	25	81,9%	94,8%	99,3%	99,6%	97,9%
27160	80	40	65	30	82,2%	94,8%	99,4%	99,6%	98,1%
27161	80	40	65	35	82,5%	94,7%	99,4%	99,6%	98,2%
27162	80	40	65	40	82,7%	94,7%	99,4%	99,6%	98,3%
27163	80	40	65	45	83,0%	94,6%	99,4%	99,7%	98,4%
27164	80	40	65	50	83,2%	94,5%	99,4%	99,7%	98,5%
27165	80	40	65	55	83,5%	94,4%	99,5%	99,7%	98,6%
27166	80	40	65	60	83,7%	94,4%	99,5%	99,7%	98,7%
27167	80	40	65	65	83,9%	94,3%	99,5%	99,7%	98,8%
27168	80	40	65	70	84,2%	94,2%	99,5%	99,8%	98,9%
27169	80	40	65	75	84,4%	94,2%	99,5%	99,8%	98,9%
27170	80	40	65	80	84,6%	94,1%	99,5%	99,8%	99,0%
27171	80	40	70	20	77,7%	93,7%	99,2%	99,5%	97,7%
27172	80	40	70	25	78,0%	93,6%	99,2%	99,5%	97,8%
27173	80	40	70	30	78,3%	93,5%	99,3%	99,6%	98,0%
27174	80	40	70	35	78,6%	93,4%	99,3%	99,6%	98,1%
27175	80	40	70	40	78,9%	93,4%	99,3%	99,6%	98,2%
27176	80	40	70	45	79,2%	93,3%	99,3%	99,6%	98,3%
27177	80	40	70	50	79,5%	93,2%	99,4%	99,7%	98,4%
27178	80	40	70	55	79,8%	93,1%	99,4%	99,7%	98,6%
27179	80	40	70	60	80,0%	93,0%	99,4%	99,7%	98,6%
27180	80	40	70	65	80,3%	92,9%	99,4%	99,7%	98,7%
27181	80	40	70	70	80,6%	92,9%	99,4%	99,7%	98,8%
27182	80	40	70	75	80,9%	92,8%	99,5%	99,8%	98,9%
27183	80	40	70	80	81,1%	92,7%	99,5%	99,8%	99,0%
27184	80	40	75	20	73,1%	92,2%	99,1%	99,5%	97,6%
27185	80	40	75	25	73,5%	92,1%	99,1%	99,5%	97,7%
27186	80	40	75	30	73,8%	92,0%	99,2%	99,5%	97,9%
27187	80	40	75	35	74,1%	91,9%	99,2%	99,6%	98,0%
27188	80	40	75	40	74,5%	91,8%	99,2%	99,6%	98,2%
27189	80	40	75	45	74,8%	91,7%	99,3%	99,6%	98,3%
27190	80	40	75	50	75,1%	91,6%	99,3%	99,6%	98,4%
27191	80	40	75	55	75,5%	91,5%	99,3%	99,7%	98,5%

27192	80	40	75	60	75,8%	91,4%	99,3%	99,7%	98,6%
27193	80	40	75	65	76,1%	91,3%	99,3%	99,7%	98,7%
27194	80	40	75	70	76,4%	91,2%	99,4%	99,7%	98,8%
27195	80	40	75	75	76,8%	91,1%	99,4%	99,7%	98,8%
27196	80	40	75	80	77,1%	90,9%	99,4%	99,8%	98,9%
27197	80	40	80	20	68,0%	90,3%	99,0%	99,4%	97,5%
27198	80	40	80	25	68,4%	90,2%	99,0%	99,5%	97,6%
27199	80	40	80	30	68,8%	90,1%	99,1%	99,5%	97,8%
27200	80	40	80	35	69,1%	90,0%	99,1%	99,5%	97,9%
27201	80	40	80	40	69,5%	89,8%	99,1%	99,6%	98,1%
27202	80	40	80	45	69,9%	89,7%	99,2%	99,6%	98,2%
27203	80	40	80	50	70,3%	89,6%	99,2%	99,6%	98,3%
27204	80	40	80	55	70,6%	89,5%	99,2%	99,6%	98,4%
27205	80	40	80	60	71,0%	89,4%	99,2%	99,7%	98,5%
27206	80	40	80	65	71,3%	89,2%	99,3%	99,7%	98,6%
27207	80	40	80	70	71,7%	89,1%	99,3%	99,7%	98,7%
27208	80	40	80	75	72,1%	89,0%	99,3%	99,7%	98,8%
27209	80	40	80	80	72,4%	88,9%	99,3%	99,7%	98,9%
27210	80	45	20	20	97,8%	99,4%	99,8%	99,7%	98,3%
27211	80	45	20	25	97,9%	99,4%	99,8%	99,7%	98,4%
27212	80	45	20	30	97,9%	99,4%	99,8%	99,7%	98,5%
27213	80	45	20	35	97,9%	99,4%	99,8%	99,8%	98,6%
27214	80	45	20	40	98,0%	99,4%	99,8%	99,8%	98,7%
27215	80	45	20	45	98,0%	99,3%	99,8%	99,8%	98,8%
27216	80	45	20	50	98,0%	99,3%	99,8%	99,8%	98,9%
27217	80	45	20	55	98,1%	99,3%	99,8%	99,8%	98,9%
27218	80	45	20	60	98,1%	99,3%	99,8%	99,8%	99,0%
27219	80	45	20	65	98,1%	99,3%	99,8%	99,8%	99,1%
27220	80	45	20	70	98,2%	99,3%	99,8%	99,8%	99,1%
27221	80	45	20	75	98,2%	99,3%	99,8%	99,9%	99,2%
27222	80	45	20	80	98,2%	99,3%	99,8%	99,9%	99,2%
27223	80	45	25	20	97,2%	99,2%	99,7%	99,7%	98,2%
27224	80	45	25	25	97,3%	99,2%	99,7%	99,7%	98,3%

27225	80	45	25	30	97,3%	99,2%	99,8%	99,7%	98,5%
27226	80	45	25	35	97,4%	99,2%	99,8%	99,7%	98,6%
27227	80	45	25	40	97,4%	99,2%	99,8%	99,8%	98,6%
27228	80	45	25	45	97,5%	99,2%	99,8%	99,8%	98,7%
27229	80	45	25	50	97,5%	99,2%	99,8%	99,8%	98,8%
27230	80	45	25	55	97,5%	99,2%	99,8%	99,8%	98,9%
27231	80	45	25	60	97,6%	99,1%	99,8%	99,8%	99,0%
27232	80	45	25	65	97,6%	99,1%	99,8%	99,8%	99,0%
27233	80	45	25	70	97,7%	99,1%	99,8%	99,8%	99,1%
27234	80	45	25	75	97,7%	99,1%	99,8%	99,9%	99,2%
27235	80	45	25	80	97,7%	99,1%	99,8%	99,9%	99,2%
27236	80	45	30	20	96,5%	99,0%	99,7%	99,7%	98,2%
27237	80	45	30	25	96,5%	99,0%	99,7%	99,7%	98,3%
27238	80	45	30	30	96,6%	99,0%	99,7%	99,7%	98,4%
27239	80	45	30	35	96,7%	99,0%	99,7%	99,7%	98,5%
27240	80	45	30	40	96,7%	99,0%	99,7%	99,7%	98,6%
27241	80	45	30	45	96,8%	99,0%	99,7%	99,8%	98,7%
27242	80	45	30	50	96,8%	98,9%	99,8%	99,8%	98,8%
27243	80	45	30	55	96,9%	98,9%	99,8%	99,8%	98,8%
27244	80	45	30	60	96,9%	98,9%	99,8%	99,8%	98,9%
27245	80	45	30	65	97,0%	98,9%	99,8%	99,8%	99,0%
27246	80	45	30	70	97,0%	98,9%	99,8%	99,8%	99,1%
27247	80	45	30	75	97,1%	98,9%	99,8%	99,8%	99,1%
27248	80	45	30	80	97,1%	98,9%	99,8%	99,9%	99,2%
27249	80	45	35	20	95,5%	98,8%	99,7%	99,6%	98,1%
27250	80	45	35	25	95,6%	98,8%	99,7%	99,7%	98,2%
27251	80	45	35	30	95,7%	98,7%	99,7%	99,7%	98,3%
27252	80	45	35	35	95,8%	98,7%	99,7%	99,7%	98,4%
27253	80	45	35	40	95,8%	98,7%	99,7%	99,7%	98,5%
27254	80	45	35	45	95,9%	98,7%	99,7%	99,7%	98,6%
27255	80	45	35	50	96,0%	98,7%	99,7%	99,8%	98,7%
27256	80	45	35	55	96,0%	98,7%	99,7%	99,8%	98,8%
27257	80	45	35	60	96,1%	98,6%	99,7%	99,8%	98,9%

27258	80	45	35	65	96,2%	98,6%	99,7%	99,8%	99,0%
27259	80	45	35	70	96,2%	98,6%	99,8%	99,8%	99,0%
27260	80	45	35	75	96,3%	98,6%	99,8%	99,8%	99,1%
27261	80	45	35	80	96,4%	98,6%	99,8%	99,8%	99,1%
27262	80	45	40	20	94,4%	98,5%	99,6%	99,6%	98,0%
27263	80	45	40	25	94,5%	98,4%	99,6%	99,6%	98,1%
27264	80	45	40	30	94,5%	98,4%	99,6%	99,7%	98,3%
27265	80	45	40	35	94,6%	98,4%	99,7%	99,7%	98,4%
27266	80	45	40	40	94,7%	98,4%	99,7%	99,7%	98,5%
27267	80	45	40	45	94,8%	98,4%	99,7%	99,7%	98,6%
27268	80	45	40	50	94,9%	98,3%	99,7%	99,7%	98,7%
27269	80	45	40	55	95,0%	98,3%	99,7%	99,8%	98,8%
27270	80	45	40	60	95,1%	98,3%	99,7%	99,8%	98,8%
27271	80	45	40	65	95,1%	98,3%	99,7%	99,8%	98,9%
27272	80	45	40	70	95,2%	98,3%	99,7%	99,8%	99,0%
27273	80	45	40	75	95,3%	98,2%	99,7%	99,8%	99,0%
27274	80	45	40	80	95,4%	98,2%	99,7%	99,8%	99,1%
27275	80	45	45	20	92,9%	98,1%	99,6%	99,6%	97,9%
27276	80	45	45	25	93,0%	98,0%	99,6%	99,6%	98,1%
27277	80	45	45	30	93,1%	98,0%	99,6%	99,6%	98,2%
27278	80	45	45	35	93,2%	98,0%	99,6%	99,7%	98,3%
27279	80	45	45	40	93,3%	98,0%	99,6%	99,7%	98,4%
27280	80	45	45	45	93,5%	97,9%	99,6%	99,7%	98,5%
27281	80	45	45	50	93,6%	97,9%	99,6%	99,7%	98,6%
27282	80	45	45	55	93,7%	97,9%	99,7%	99,7%	98,7%
27283	80	45	45	60	93,8%	97,9%	99,7%	99,8%	98,8%
27284	80	45	45	65	93,9%	97,8%	99,7%	99,8%	98,9%
27285	80	45	45	70	94,0%	97,8%	99,7%	99,8%	98,9%
27286	80	45	45	75	94,1%	97,8%	99,7%	99,8%	99,0%
27287	80	45	45	80	94,2%	97,7%	99,7%	99,8%	99,1%
27288	80	45	50	20	91,1%	97,6%	99,5%	99,6%	97,8%
27289	80	45	50	25	91,2%	97,5%	99,5%	99,6%	98,0%
27290	80	45	50	30	91,4%	97,5%	99,5%	99,6%	98,1%

27291	80	45	50	35	91,5%	97,5%	99,6%	99,6%	98,2%
27292	80	45	50	40	91,6%	97,5%	99,6%	99,7%	98,3%
27293	80	45	50	45	91,8%	97,4%	99,6%	99,7%	98,5%
27294	80	45	50	50	91,9%	97,4%	99,6%	99,7%	98,6%
27295	80	45	50	55	92,0%	97,4%	99,6%	99,7%	98,6%
27296	80	45	50	60	92,2%	97,3%	99,6%	99,8%	98,7%
27297	80	45	50	65	92,3%	97,3%	99,6%	99,8%	98,8%
27298	80	45	50	70	92,4%	97,3%	99,6%	99,8%	98,9%
27299	80	45	50	75	92,5%	97,2%	99,7%	99,8%	99,0%
27300	80	45	50	80	92,7%	97,2%	99,7%	99,8%	99,0%
27301	80	45	55	20	88,9%	97,0%	99,4%	99,5%	97,7%
27302	80	45	55	25	89,0%	96,9%	99,5%	99,6%	97,9%
27303	80	45	55	30	89,2%	96,9%	99,5%	99,6%	98,0%
27304	80	45	55	35	89,4%	96,9%	99,5%	99,6%	98,2%
27305	80	45	55	40	89,5%	96,8%	99,5%	99,7%	98,3%
27306	80	45	55	45	89,7%	96,8%	99,5%	99,7%	98,4%
27307	80	45	55	50	89,9%	96,7%	99,5%	99,7%	98,5%
27308	80	45	55	55	90,0%	96,7%	99,6%	99,7%	98,6%
27309	80	45	55	60	90,2%	96,6%	99,6%	99,7%	98,7%
27310	80	45	55	65	90,3%	96,6%	99,6%	99,8%	98,8%
27311	80	45	55	70	90,5%	96,6%	99,6%	99,8%	98,8%
27312	80	45	55	75	90,6%	96,5%	99,6%	99,8%	98,9%
27313	80	45	55	80	90,8%	96,5%	99,6%	99,8%	99,0%
27314	80	45	60	20	86,2%	96,2%	99,4%	99,5%	97,6%
27315	80	45	60	25	86,4%	96,2%	99,4%	99,5%	97,8%
27316	80	45	60	30	86,6%	96,1%	99,4%	99,6%	97,9%
27317	80	45	60	35	86,8%	96,1%	99,4%	99,6%	98,1%
27318	80	45	60	40	87,0%	96,0%	99,5%	99,6%	98,2%
27319	80	45	60	45	87,2%	96,0%	99,5%	99,7%	98,3%
27320	80	45	60	50	87,4%	95,9%	99,5%	99,7%	98,4%
27321	80	45	60	55	87,6%	95,9%	99,5%	99,7%	98,5%
27322	80	45	60	60	87,8%	95,8%	99,5%	99,7%	98,6%
27323	80	45	60	65	88,0%	95,8%	99,5%	99,7%	98,7%

27324	80	45	60	70	88,1%	95,7%	99,6%	99,8%	98,8%
27325	80	45	60	75	88,3%	95,7%	99,6%	99,8%	98,9%
27326	80	45	60	80	88,5%	95,6%	99,6%	99,8%	99,0%
27327	80	45	65	20	83,0%	95,3%	99,3%	99,5%	97,6%
27328	80	45	65	25	83,2%	95,2%	99,3%	99,5%	97,7%
27329	80	45	65	30	83,5%	95,2%	99,3%	99,5%	97,9%
27330	80	45	65	35	83,7%	95,1%	99,4%	99,6%	98,0%
27331	80	45	65	40	83,9%	95,0%	99,4%	99,6%	98,1%
27332	80	45	65	45	84,2%	95,0%	99,4%	99,6%	98,2%
27333	80	45	65	50	84,4%	94,9%	99,4%	99,7%	98,4%
27334	80	45	65	55	84,6%	94,8%	99,4%	99,7%	98,5%
27335	80	45	65	60	84,9%	94,8%	99,5%	99,7%	98,6%
27336	80	45	65	65	85,1%	94,7%	99,5%	99,7%	98,7%
27337	80	45	65	70	85,3%	94,7%	99,5%	99,7%	98,8%
27338	80	45	65	75	85,5%	94,6%	99,5%	99,8%	98,8%
27339	80	45	65	80	85,8%	94,5%	99,5%	99,8%	98,9%
27340	80	45	70	20	79,2%	94,1%	99,2%	99,4%	97,5%
27341	80	45	70	25	79,5%	94,1%	99,2%	99,5%	97,6%
27342	80	45	70	30	79,8%	94,0%	99,2%	99,5%	97,8%
27343	80	45	70	35	80,0%	93,9%	99,3%	99,6%	97,9%
27344	80	45	70	40	80,3%	93,8%	99,3%	99,6%	98,1%
27345	80	45	70	45	80,6%	93,8%	99,3%	99,6%	98,2%
27346	80	45	70	50	80,9%	93,7%	99,3%	99,6%	98,3%
27347	80	45	70	55	81,1%	93,6%	99,4%	99,7%	98,4%
27348	80	45	70	60	81,4%	93,5%	99,4%	99,7%	98,5%
27349	80	45	70	65	81,7%	93,4%	99,4%	99,7%	98,6%
27350	80	45	70	70	81,9%	93,4%	99,4%	99,7%	98,7%
27351	80	45	70	75	82,2%	93,3%	99,4%	99,7%	98,8%
27352	80	45	70	80	82,5%	93,2%	99,5%	99,8%	98,9%
27353	80	45	75	20	74,8%	92,7%	99,1%	99,4%	97,3%
27354	80	45	75	25	75,2%	92,6%	99,1%	99,5%	97,5%
27355	80	45	75	30	75,5%	92,5%	99,1%	99,5%	97,7%
27356	80	45	75	35	75,8%	92,4%	99,2%	99,5%	97,8%

27357	80	45	75	40	76,1%	92,3%	99,2%	99,6%	98,0%
27358	80	45	75	45	76,5%	92,3%	99,2%	99,6%	98,1%
27359	80	45	75	50	76,8%	92,2%	99,3%	99,6%	98,2%
27360	80	45	75	55	77,1%	92,1%	99,3%	99,6%	98,3%
27361	80	45	75	60	77,4%	92,0%	99,3%	99,7%	98,4%
27362	80	45	75	65	77,7%	91,9%	99,3%	99,7%	98,6%
27363	80	45	75	70	78,0%	91,8%	99,3%	99,7%	98,6%
27364	80	45	75	75	78,3%	91,7%	99,4%	99,7%	98,7%
27365	80	45	75	80	78,6%	91,6%	99,4%	99,7%	98,8%
27366	80	45	80	20	69,9%	91,0%	99,0%	99,4%	97,2%
27367	80	45	80	25	70,3%	90,9%	99,0%	99,4%	97,4%
27368	80	45	80	30	70,6%	90,8%	99,0%	99,5%	97,6%
27369	80	45	80	35	71,0%	90,7%	99,1%	99,5%	97,7%
27370	80	45	80	40	71,4%	90,5%	99,1%	99,5%	97,9%
27371	80	45	80	45	71,7%	90,4%	99,1%	99,6%	98,0%
27372	80	45	80	50	72,1%	90,3%	99,2%	99,6%	98,2%
27373	80	45	80	55	72,4%	90,2%	99,2%	99,6%	98,3%
27374	80	45	80	60	72,8%	90,1%	99,2%	99,6%	98,4%
27375	80	45	80	65	73,1%	90,0%	99,2%	99,7%	98,5%
27376	80	45	80	70	73,5%	89,9%	99,3%	99,7%	98,6%
27377	80	45	80	75	73,8%	89,7%	99,3%	99,7%	98,7%
27378	80	45	80	80	74,2%	89,6%	99,3%	99,7%	98,8%
27379	80	50	20	20	98,0%	99,4%	99,8%	99,7%	98,1%
27380	80	50	20	25	98,0%	99,4%	99,8%	99,7%	98,3%
27381	80	50	20	30	98,1%	99,4%	99,8%	99,7%	98,4%
27382	80	50	20	35	98,1%	99,4%	99,8%	99,7%	98,5%
27383	80	50	20	40	98,1%	99,4%	99,8%	99,8%	98,6%
27384	80	50	20	45	98,2%	99,4%	99,8%	99,8%	98,7%
27385	80	50	20	50	98,2%	99,4%	99,8%	99,8%	98,8%
27386	80	50	20	55	98,2%	99,4%	99,8%	99,8%	98,8%
27387	80	50	20	60	98,3%	99,4%	99,8%	99,8%	98,9%
27388	80	50	20	65	98,3%	99,4%	99,8%	99,8%	99,0%
27389	80	50	20	70	98,3%	99,4%	99,8%	99,8%	99,0%

27390	80	50	20	75	98,4%	99,3%	99,8%	99,8%	99,1%
27391	80	50	20	80	98,4%	99,3%	99,8%	99,9%	99,2%
27392	80	50	25	20	97,5%	99,3%	99,7%	99,7%	98,1%
27393	80	50	25	25	97,5%	99,3%	99,7%	99,7%	98,2%
27394	80	50	25	30	97,5%	99,3%	99,7%	99,7%	98,3%
27395	80	50	25	35	97,6%	99,3%	99,8%	99,7%	98,4%
27396	80	50	25	40	97,6%	99,2%	99,8%	99,7%	98,5%
27397	80	50	25	45	97,7%	99,2%	99,8%	99,8%	98,6%
27398	80	50	25	50	97,7%	99,2%	99,8%	99,8%	98,7%
27399	80	50	25	55	97,7%	99,2%	99,8%	99,8%	98,8%
27400	80	50	25	60	97,8%	99,2%	99,8%	99,8%	98,9%
27401	80	50	25	65	97,8%	99,2%	99,8%	99,8%	98,9%
27402	80	50	25	70	97,9%	99,2%	99,8%	99,8%	99,0%
27403	80	50	25	75	97,9%	99,2%	99,8%	99,8%	99,1%
27404	80	50	25	80	97,9%	99,2%	99,8%	99,8%	99,1%
27405	80	50	30	20	96,8%	99,1%	99,7%	99,6%	98,0%
27406	80	50	30	25	96,8%	99,1%	99,7%	99,7%	98,1%
27407	80	50	30	30	96,9%	99,1%	99,7%	99,7%	98,2%
27408	80	50	30	35	96,9%	99,1%	99,7%	99,7%	98,3%
27409	80	50	30	40	97,0%	99,1%	99,7%	99,7%	98,5%
27410	80	50	30	45	97,0%	99,0%	99,7%	99,7%	98,6%
27411	80	50	30	50	97,1%	99,0%	99,7%	99,8%	98,6%
27412	80	50	30	55	97,1%	99,0%	99,8%	99,8%	98,7%
27413	80	50	30	60	97,2%	99,0%	99,8%	99,8%	98,8%
27414	80	50	30	65	97,2%	99,0%	99,8%	99,8%	98,9%
27415	80	50	30	70	97,3%	99,0%	99,8%	99,8%	99,0%
27416	80	50	30	75	97,3%	99,0%	99,8%	99,8%	99,0%
27417	80	50	30	80	97,4%	98,9%	99,8%	99,8%	99,1%
27418	80	50	35	20	95,9%	98,9%	99,6%	99,6%	97,9%
27419	80	50	35	25	96,0%	98,9%	99,7%	99,6%	98,0%
27420	80	50	35	30	96,0%	98,8%	99,7%	99,7%	98,2%
27421	80	50	35	35	96,1%	98,8%	99,7%	99,7%	98,3%
27422	80	50	35	40	96,2%	98,8%	99,7%	99,7%	98,4%

27423	80	50	35	45	96,2%	98,8%	99,7%	99,7%	98,5%
27424	80	50	35	50	96,3%	98,8%	99,7%	99,7%	98,6%
27425	80	50	35	55	96,4%	98,8%	99,7%	99,8%	98,7%
27426	80	50	35	60	96,4%	98,7%	99,7%	99,8%	98,8%
27427	80	50	35	65	96,5%	98,7%	99,7%	99,8%	98,8%
27428	80	50	35	70	96,5%	98,7%	99,8%	99,8%	98,9%
27429	80	50	35	75	96,6%	98,7%	99,8%	99,8%	99,0%
27430	80	50	35	80	96,7%	98,7%	99,8%	99,8%	99,1%
27431	80	50	40	20	94,8%	98,6%	99,6%	99,6%	97,8%
27432	80	50	40	25	94,9%	98,6%	99,6%	99,6%	97,9%
27433	80	50	40	30	95,0%	98,5%	99,6%	99,6%	98,1%
27434	80	50	40	35	95,1%	98,5%	99,6%	99,7%	98,2%
27435	80	50	40	40	95,2%	98,5%	99,7%	99,7%	98,3%
27436	80	50	40	45	95,2%	98,5%	99,7%	99,7%	98,4%
27437	80	50	40	50	95,3%	98,5%	99,7%	99,7%	98,5%
27438	80	50	40	55	95,4%	98,4%	99,7%	99,7%	98,6%
27439	80	50	40	60	95,5%	98,4%	99,7%	99,8%	98,7%
27440	80	50	40	65	95,5%	98,4%	99,7%	99,8%	98,8%
27441	80	50	40	70	95,6%	98,4%	99,7%	99,8%	98,9%
27442	80	50	40	75	95,7%	98,4%	99,7%	99,8%	99,0%
27443	80	50	40	80	95,8%	98,3%	99,7%	99,8%	99,0%
27444	80	50	45	20	93,5%	98,2%	99,6%	99,6%	97,7%
27445	80	50	45	25	93,6%	98,2%	99,6%	99,6%	97,9%
27446	80	50	45	30	93,7%	98,2%	99,6%	99,6%	98,0%
27447	80	50	45	35	93,8%	98,1%	99,6%	99,6%	98,1%
27448	80	50	45	40	93,9%	98,1%	99,6%	99,7%	98,3%
27449	80	50	45	45	94,0%	98,1%	99,6%	99,7%	98,4%
27450	80	50	45	50	94,1%	98,1%	99,6%	99,7%	98,5%
27451	80	50	45	55	94,2%	98,0%	99,6%	99,7%	98,6%
27452	80	50	45	60	94,3%	98,0%	99,7%	99,7%	98,7%
27453	80	50	45	65	94,4%	98,0%	99,7%	99,8%	98,8%
27454	80	50	45	70	94,5%	98,0%	99,7%	99,8%	98,8%
27455	80	50	45	75	94,5%	97,9%	99,7%	99,8%	98,9%

27456	80	50	45	80	94,6%	97,9%	99,7%	99,8%	99,0%
27457	80	50	50	20	91,8%	97,8%	99,5%	99,5%	97,6%
27458	80	50	50	25	91,9%	97,7%	99,5%	99,6%	97,8%
27459	80	50	50	30	92,0%	97,7%	99,5%	99,6%	97,9%
27460	80	50	50	35	92,2%	97,7%	99,5%	99,6%	98,1%
27461	80	50	50	40	92,3%	97,6%	99,6%	99,6%	98,2%
27462	80	50	50	45	92,4%	97,6%	99,6%	99,7%	98,3%
27463	80	50	50	50	92,5%	97,6%	99,6%	99,7%	98,4%
27464	80	50	50	55	92,7%	97,6%	99,6%	99,7%	98,5%
27465	80	50	50	60	92,8%	97,5%	99,6%	99,7%	98,6%
27466	80	50	50	65	92,9%	97,5%	99,6%	99,7%	98,7%
27467	80	50	50	70	93,0%	97,5%	99,6%	99,8%	98,8%
27468	80	50	50	75	93,1%	97,4%	99,7%	99,8%	98,9%
27469	80	50	50	80	93,2%	97,4%	99,7%	99,8%	98,9%
27470	80	50	55	20	89,7%	97,2%	99,4%	99,5%	97,5%
27471	80	50	55	25	89,9%	97,2%	99,4%	99,5%	97,7%
27472	80	50	55	30	90,0%	97,1%	99,5%	99,6%	97,8%
27473	80	50	55	35	90,2%	97,1%	99,5%	99,6%	98,0%
27474	80	50	55	40	90,3%	97,0%	99,5%	99,6%	98,1%
27475	80	50	55	45	90,5%	97,0%	99,5%	99,6%	98,2%
27476	80	50	55	50	90,6%	97,0%	99,5%	99,7%	98,3%
27477	80	50	55	55	90,8%	96,9%	99,5%	99,7%	98,5%
27478	80	50	55	60	90,9%	96,9%	99,6%	99,7%	98,6%
27479	80	50	55	65	91,1%	96,9%	99,6%	99,7%	98,6%
27480	80	50	55	70	91,2%	96,8%	99,6%	99,8%	98,7%
27481	80	50	55	75	91,4%	96,8%	99,6%	99,8%	98,8%
27482	80	50	55	80	91,5%	96,7%	99,6%	99,8%	98,9%
27483	80	50	60	20	87,2%	96,5%	99,4%	99,5%	97,4%
27484	80	50	60	25	87,4%	96,4%	99,4%	99,5%	97,6%
27485	80	50	60	30	87,6%	96,4%	99,4%	99,5%	97,7%
27486	80	50	60	35	87,8%	96,4%	99,4%	99,6%	97,9%
27487	80	50	60	40	88,0%	96,3%	99,4%	99,6%	98,0%
27488	80	50	60	45	88,2%	96,3%	99,5%	99,6%	98,2%

27489	80	50	60	50	88,3%	96,2%	99,5%	99,7%	98,3%
27490	80	50	60	55	88,5%	96,2%	99,5%	99,7%	98,4%
27491	80	50	60	60	88,7%	96,1%	99,5%	99,7%	98,5%
27492	80	50	60	65	88,9%	96,1%	99,5%	99,7%	98,6%
27493	80	50	60	70	89,0%	96,0%	99,5%	99,7%	98,7%
27494	80	50	60	75	89,2%	96,0%	99,6%	99,8%	98,8%
27495	80	50	60	80	89,4%	95,9%	99,6%	99,8%	98,8%
27496	80	50	65	20	84,2%	95,6%	99,3%	99,4%	97,3%
27497	80	50	65	25	84,4%	95,6%	99,3%	99,5%	97,5%
27498	80	50	65	30	84,6%	95,5%	99,3%	99,5%	97,6%
27499	80	50	65	35	84,9%	95,5%	99,3%	99,5%	97,8%
27500	80	50	65	40	85,1%	95,4%	99,4%	99,6%	97,9%
27501	80	50	65	45	85,3%	95,3%	99,4%	99,6%	98,1%
27502	80	50	65	50	85,5%	95,3%	99,4%	99,6%	98,2%
27503	80	50	65	55	85,8%	95,2%	99,4%	99,7%	98,3%
27504	80	50	65	60	86,0%	95,2%	99,4%	99,7%	98,4%
27505	80	50	65	65	86,2%	95,1%	99,5%	99,7%	98,5%
27506	80	50	65	70	86,4%	95,0%	99,5%	99,7%	98,6%
27507	80	50	65	75	86,6%	95,0%	99,5%	99,7%	98,7%
27508	80	50	65	80	86,8%	94,9%	99,5%	99,8%	98,8%
27509	80	50	70	20	80,6%	94,5%	99,2%	99,4%	97,2%
27510	80	50	70	25	80,9%	94,5%	99,2%	99,4%	97,4%
27511	80	50	70	30	81,2%	94,4%	99,2%	99,5%	97,6%
27512	80	50	70	35	81,4%	94,3%	99,2%	99,5%	97,7%
27513	80	50	70	40	81,7%	94,3%	99,3%	99,5%	97,9%
27514	80	50	70	45	82,0%	94,2%	99,3%	99,6%	98,0%
27515	80	50	70	50	82,2%	94,1%	99,3%	99,6%	98,1%
27516	80	50	70	55	82,5%	94,1%	99,3%	99,6%	98,3%
27517	80	50	70	60	82,7%	94,0%	99,4%	99,7%	98,4%
27518	80	50	70	65	83,0%	93,9%	99,4%	99,7%	98,5%
27519	80	50	70	70	83,2%	93,8%	99,4%	99,7%	98,6%
27520	80	50	70	75	83,5%	93,8%	99,4%	99,7%	98,7%
27521	80	50	70	80	83,7%	93,7%	99,4%	99,7%	98,8%

27522	80	50	75	20	76,5%	93,2%	99,1%	99,4%	97,1%
27523	80	50	75	25	76,8%	93,1%	99,1%	99,4%	97,3%
27524	80	50	75	30	77,1%	93,1%	99,1%	99,5%	97,5%
27525	80	50	75	35	77,4%	93,0%	99,2%	99,5%	97,6%
27526	80	50	75	40	77,7%	92,9%	99,2%	99,5%	97,8%
27527	80	50	75	45	78,0%	92,8%	99,2%	99,6%	97,9%
27528	80	50	75	50	78,3%	92,7%	99,2%	99,6%	98,1%
27529	80	50	75	55	78,6%	92,6%	99,3%	99,6%	98,2%
27530	80	50	75	60	78,9%	92,5%	99,3%	99,6%	98,3%
27531	80	50	75	65	79,2%	92,4%	99,3%	99,7%	98,4%
27532	80	50	75	70	79,5%	92,3%	99,3%	99,7%	98,5%
27533	80	50	75	75	79,8%	92,3%	99,4%	99,7%	98,6%
27534	80	50	75	80	80,1%	92,2%	99,4%	99,7%	98,7%
27535	80	50	80	20	71,7%	91,6%	98,9%	99,3%	97,0%
27536	80	50	80	25	72,1%	91,5%	99,0%	99,4%	97,2%
27537	80	50	80	30	72,4%	91,4%	99,0%	99,4%	97,3%
27538	80	50	80	35	72,8%	91,3%	99,0%	99,5%	97,5%
27539	80	50	80	40	73,1%	91,2%	99,1%	99,5%	97,7%
27540	80	50	80	45	73,5%	91,1%	99,1%	99,5%	97,8%
27541	80	50	80	50	73,8%	91,0%	99,1%	99,6%	98,0%
27542	80	50	80	55	74,2%	90,9%	99,2%	99,6%	98,1%
27543	80	50	80	60	74,5%	90,8%	99,2%	99,6%	98,2%
27544	80	50	80	65	74,8%	90,7%	99,2%	99,6%	98,3%
27545	80	50	80	70	75,2%	90,5%	99,2%	99,7%	98,4%
27546	80	50	80	75	75,5%	90,4%	99,3%	99,7%	98,6%
27547	80	50	80	80	75,8%	90,3%	99,3%	99,7%	98,6%
27548	80	55	20	20	98,2%	99,5%	99,8%	99,7%	97,9%
27549	80	55	20	25	98,2%	99,5%	99,8%	99,7%	98,1%
27550	80	55	20	30	98,2%	99,5%	99,8%	99,7%	98,2%
27551	80	55	20	35	98,3%	99,5%	99,8%	99,7%	98,3%
27552	80	55	20	40	98,3%	99,4%	99,8%	99,7%	98,4%
27553	80	55	20	45	98,3%	99,4%	99,8%	99,8%	98,5%
27554	80	55	20	50	98,4%	99,4%	99,8%	99,8%	98,6%

27555	80	55	20	55	98,4%	99,4%	99,8%	99,8%	98,7%
27556	80	55	20	60	98,4%	99,4%	99,8%	99,8%	98,8%
27557	80	55	20	65	98,4%	99,4%	99,8%	99,8%	98,9%
27558	80	55	20	70	98,5%	99,4%	99,8%	99,8%	99,0%
27559	80	55	20	75	98,5%	99,4%	99,8%	99,8%	99,0%
27560	80	55	20	80	98,5%	99,4%	99,8%	99,8%	99,1%
27561	80	55	25	20	97,7%	99,3%	99,7%	99,6%	97,9%
27562	80	55	25	25	97,7%	99,3%	99,7%	99,7%	98,0%
27563	80	55	25	30	97,7%	99,3%	99,7%	99,7%	98,1%
27564	80	55	25	35	97,8%	99,3%	99,7%	99,7%	98,3%
27565	80	55	25	40	97,8%	99,3%	99,8%	99,7%	98,4%
27566	80	55	25	45	97,9%	99,3%	99,8%	99,7%	98,5%
27567	80	55	25	50	97,9%	99,3%	99,8%	99,8%	98,6%
27568	80	55	25	55	97,9%	99,3%	99,8%	99,8%	98,7%
27569	80	55	25	60	98,0%	99,3%	99,8%	99,8%	98,8%
27570	80	55	25	65	98,0%	99,3%	99,8%	99,8%	98,8%
27571	80	55	25	70	98,0%	99,2%	99,8%	99,8%	98,9%
27572	80	55	25	75	98,1%	99,2%	99,8%	99,8%	99,0%
27573	80	55	25	80	98,1%	99,2%	99,8%	99,8%	99,0%
27574	80	55	30	20	97,0%	99,2%	99,7%	99,6%	97,8%
27575	80	55	30	25	97,1%	99,2%	99,7%	99,6%	97,9%
27576	80	55	30	30	97,1%	99,1%	99,7%	99,7%	98,1%
27577	80	55	30	35	97,2%	99,1%	99,7%	99,7%	98,2%
27578	80	55	30	40	97,2%	99,1%	99,7%	99,7%	98,3%
27579	80	55	30	45	97,3%	99,1%	99,7%	99,7%	98,4%
27580	80	55	30	50	97,3%	99,1%	99,7%	99,7%	98,5%
27581	80	55	30	55	97,4%	99,1%	99,7%	99,8%	98,6%
27582	80	55	30	60	97,4%	99,1%	99,8%	99,8%	98,7%
27583	80	55	30	65	97,5%	99,1%	99,8%	99,8%	98,8%
27584	80	55	30	70	97,5%	99,1%	99,8%	99,8%	98,9%
27585	80	55	30	75	97,5%	99,0%	99,8%	99,8%	98,9%
27586	80	55	30	80	97,6%	99,0%	99,8%	99,8%	99,0%
27587	80	55	35	20	96,2%	99,0%	99,6%	99,6%	97,7%

27588	80	55	35	25	96,3%	98,9%	99,7%	99,6%	97,8%
27589	80	55	35	30	96,4%	98,9%	99,7%	99,6%	98,0%
27590	80	55	35	35	96,4%	98,9%	99,7%	99,7%	98,1%
27591	80	55	35	40	96,5%	98,9%	99,7%	99,7%	98,2%
27592	80	55	35	45	96,5%	98,9%	99,7%	99,7%	98,3%
27593	80	55	35	50	96,6%	98,9%	99,7%	99,7%	98,5%
27594	80	55	35	55	96,7%	98,9%	99,7%	99,7%	98,6%
27595	80	55	35	60	96,7%	98,8%	99,7%	99,8%	98,6%
27596	80	55	35	65	96,8%	98,8%	99,7%	99,8%	98,7%
27597	80	55	35	70	96,8%	98,8%	99,7%	99,8%	98,8%
27598	80	55	35	75	96,9%	98,8%	99,8%	99,8%	98,9%
27599	80	55	35	80	96,9%	98,8%	99,8%	99,8%	99,0%
27600	80	55	40	20	95,2%	98,7%	99,6%	99,6%	97,6%
27601	80	55	40	25	95,3%	98,7%	99,6%	99,6%	97,7%
27602	80	55	40	30	95,4%	98,6%	99,6%	99,6%	97,9%
27603	80	55	40	35	95,5%	98,6%	99,6%	99,6%	98,0%
27604	80	55	40	40	95,5%	98,6%	99,6%	99,7%	98,2%
27605	80	55	40	45	95,6%	98,6%	99,7%	99,7%	98,3%
27606	80	55	40	50	95,7%	98,6%	99,7%	99,7%	98,4%
27607	80	55	40	55	95,8%	98,6%	99,7%	99,7%	98,5%
27608	80	55	40	60	95,8%	98,5%	99,7%	99,7%	98,6%
27609	80	55	40	65	95,9%	98,5%	99,7%	99,8%	98,7%
27610	80	55	40	70	96,0%	98,5%	99,7%	99,8%	98,8%
27611	80	55	40	75	96,0%	98,5%	99,7%	99,8%	98,9%
27612	80	55	40	80	96,1%	98,5%	99,7%	99,8%	98,9%
27613	80	55	45	20	94,0%	98,3%	99,5%	99,5%	97,5%
27614	80	55	45	25	94,1%	98,3%	99,6%	99,6%	97,7%
27615	80	55	45	30	94,2%	98,3%	99,6%	99,6%	97,8%
27616	80	55	45	35	94,3%	98,3%	99,6%	99,6%	97,9%
27617	80	55	45	40	94,4%	98,3%	99,6%	99,6%	98,1%
27618	80	55	45	45	94,5%	98,2%	99,6%	99,7%	98,2%
27619	80	55	45	50	94,6%	98,2%	99,6%	99,7%	98,3%
27620	80	55	45	55	94,6%	98,2%	99,6%	99,7%	98,4%

27621	80	55	45	60	94,7%	98,2%	99,6%	99,7%	98,5%
27622	80	55	45	65	94,8%	98,1%	99,7%	99,7%	98,6%
27623	80	55	45	70	94,9%	98,1%	99,7%	99,8%	98,7%
27624	80	55	45	75	95,0%	98,1%	99,7%	99,8%	98,8%
27625	80	55	45	80	95,1%	98,1%	99,7%	99,8%	98,9%
27626	80	55	50	20	92,4%	97,9%	99,5%	99,5%	97,4%
27627	80	55	50	25	92,5%	97,9%	99,5%	99,5%	97,6%
27628	80	55	50	30	92,7%	97,9%	99,5%	99,6%	97,7%
27629	80	55	50	35	92,8%	97,8%	99,5%	99,6%	97,9%
27630	80	55	50	40	92,9%	97,8%	99,5%	99,6%	98,0%
27631	80	55	50	45	93,0%	97,8%	99,6%	99,6%	98,1%
27632	80	55	50	50	93,1%	97,8%	99,6%	99,7%	98,3%
27633	80	55	50	55	93,2%	97,7%	99,6%	99,7%	98,4%
27634	80	55	50	60	93,4%	97,7%	99,6%	99,7%	98,5%
27635	80	55	50	65	93,5%	97,7%	99,6%	99,7%	98,6%
27636	80	55	50	70	93,6%	97,6%	99,6%	99,7%	98,7%
27637	80	55	50	75	93,7%	97,6%	99,6%	99,8%	98,8%
27638	80	55	50	80	93,8%	97,6%	99,7%	99,8%	98,8%
27639	80	55	55	20	90,5%	97,4%	99,4%	99,5%	97,3%
27640	80	55	55	25	90,7%	97,4%	99,4%	99,5%	97,5%
27641	80	55	55	30	90,8%	97,3%	99,4%	99,5%	97,6%
27642	80	55	55	35	90,9%	97,3%	99,5%	99,6%	97,8%
27643	80	55	55	40	91,1%	97,3%	99,5%	99,6%	97,9%
27644	80	55	55	45	91,2%	97,2%	99,5%	99,6%	98,1%
27645	80	55	55	50	91,4%	97,2%	99,5%	99,6%	98,2%
27646	80	55	55	55	91,5%	97,2%	99,5%	99,7%	98,3%
27647	80	55	55	60	91,6%	97,1%	99,5%	99,7%	98,4%
27648	80	55	55	65	91,8%	97,1%	99,6%	99,7%	98,5%
27649	80	55	55	70	91,9%	97,0%	99,6%	99,7%	98,6%
27650	80	55	55	75	92,0%	97,0%	99,6%	99,8%	98,7%
27651	80	55	55	80	92,2%	97,0%	99,6%	99,8%	98,8%
27652	80	55	60	20	88,2%	96,7%	99,3%	99,4%	97,2%
27653	80	55	60	25	88,3%	96,7%	99,4%	99,5%	97,4%

27654	80	55	60	30	88,5%	96,7%	99,4%	99,5%	97,5%
27655	80	55	60	35	88,7%	96,6%	99,4%	99,5%	97,7%
27656	80	55	60	40	88,9%	96,6%	99,4%	99,6%	97,8%
27657	80	55	60	45	89,0%	96,5%	99,4%	99,6%	98,0%
27658	80	55	60	50	89,2%	96,5%	99,5%	99,6%	98,1%
27659	80	55	60	55	89,4%	96,4%	99,5%	99,7%	98,2%
27660	80	55	60	60	89,6%	96,4%	99,5%	99,7%	98,3%
27661	80	55	60	65	89,7%	96,4%	99,5%	99,7%	98,5%
27662	80	55	60	70	89,9%	96,3%	99,5%	99,7%	98,6%
27663	80	55	60	75	90,0%	96,3%	99,5%	99,7%	98,6%
27664	80	55	60	80	90,2%	96,2%	99,6%	99,8%	98,7%
27665	80	55	65	20	85,3%	95,9%	99,2%	99,4%	97,1%
27666	80	55	65	25	85,5%	95,9%	99,3%	99,4%	97,2%
27667	80	55	65	30	85,8%	95,8%	99,3%	99,5%	97,4%
27668	80	55	65	35	86,0%	95,8%	99,3%	99,5%	97,6%
27669	80	55	65	40	86,2%	95,7%	99,3%	99,5%	97,7%
27670	80	55	65	45	86,4%	95,7%	99,4%	99,6%	97,9%
27671	80	55	65	50	86,6%	95,6%	99,4%	99,6%	98,0%
27672	80	55	65	55	86,8%	95,6%	99,4%	99,6%	98,2%
27673	80	55	65	60	87,0%	95,5%	99,4%	99,7%	98,3%
27674	80	55	65	65	87,2%	95,5%	99,4%	99,7%	98,4%
27675	80	55	65	70	87,4%	95,4%	99,5%	99,7%	98,5%
27676	80	55	65	75	87,6%	95,3%	99,5%	99,7%	98,6%
27677	80	55	65	80	87,8%	95,3%	99,5%	99,7%	98,7%
27678	80	55	70	20	82,0%	94,9%	99,1%	99,4%	96,9%
27679	80	55	70	25	82,2%	94,9%	99,2%	99,4%	97,1%
27680	80	55	70	30	82,5%	94,8%	99,2%	99,4%	97,3%
27681	80	55	70	35	82,7%	94,7%	99,2%	99,5%	97,5%
27682	80	55	70	40	83,0%	94,7%	99,3%	99,5%	97,7%
27683	80	55	70	45	83,2%	94,6%	99,3%	99,5%	97,8%
27684	80	55	70	50	83,5%	94,5%	99,3%	99,6%	97,9%
27685	80	55	70	55	83,7%	94,5%	99,3%	99,6%	98,1%
27686	80	55	70	60	84,0%	94,4%	99,3%	99,6%	98,2%

27687	80	55	70	65	84,2%	94,3%	99,4%	99,7%	98,3%
27688	80	55	70	70	84,4%	94,3%	99,4%	99,7%	98,4%
27689	80	55	70	75	84,7%	94,2%	99,4%	99,7%	98,5%
27690	80	55	70	80	84,9%	94,1%	99,4%	99,7%	98,6%
27691	80	55	75	20	78,0%	93,7%	99,0%	99,3%	96,8%
27692	80	55	75	25	78,3%	93,6%	99,1%	99,4%	97,0%
27693	80	55	75	30	78,6%	93,5%	99,1%	99,4%	97,2%
27694	80	55	75	35	78,9%	93,5%	99,1%	99,4%	97,4%
27695	80	55	75	40	79,2%	93,4%	99,2%	99,5%	97,6%
27696	80	55	75	45	79,5%	93,3%	99,2%	99,5%	97,7%
27697	80	55	75	50	79,8%	93,2%	99,2%	99,6%	97,9%
27698	80	55	75	55	80,1%	93,1%	99,2%	99,6%	98,0%
27699	80	55	75	60	80,3%	93,1%	99,3%	99,6%	98,1%
27700	80	55	75	65	80,6%	93,0%	99,3%	99,6%	98,3%
27701	80	55	75	70	80,9%	92,9%	99,3%	99,7%	98,4%
27702	80	55	75	75	81,2%	92,8%	99,3%	99,7%	98,5%
27703	80	55	75	80	81,4%	92,7%	99,4%	99,7%	98,6%
27704	80	55	80	20	73,5%	92,2%	98,9%	99,3%	96,7%
27705	80	55	80	25	73,8%	92,1%	98,9%	99,3%	96,9%
27706	80	55	80	30	74,2%	92,0%	99,0%	99,4%	97,1%
27707	80	55	80	35	74,5%	91,9%	99,0%	99,4%	97,3%
27708	80	55	80	40	74,8%	91,8%	99,0%	99,5%	97,5%
27709	80	55	80	45	75,2%	91,7%	99,1%	99,5%	97,6%
27710	80	55	80	50	75,5%	91,6%	99,1%	99,5%	97,8%
27711	80	55	80	55	75,8%	91,5%	99,1%	99,6%	97,9%
27712	80	55	80	60	76,2%	91,4%	99,2%	99,6%	98,1%
27713	80	55	80	65	76,5%	91,3%	99,2%	99,6%	98,2%
27714	80	55	80	70	76,8%	91,2%	99,2%	99,6%	98,3%
27715	80	55	80	75	77,1%	91,1%	99,2%	99,7%	98,4%
27716	80	55	80	80	77,4%	91,0%	99,3%	99,7%	98,5%
27717	80	60	20	20	98,3%	99,5%	99,7%	99,6%	97,7%
27718	80	60	20	25	98,4%	99,5%	99,8%	99,7%	97,9%
27719	80	60	20	30	98,4%	99,5%	99,8%	99,7%	98,0%

27720	80	60	20	35	98,4%	99,5%	99,8%	99,7%	98,2%
27721	80	60	20	40	98,4%	99,5%	99,8%	99,7%	98,3%
27722	80	60	20	45	98,5%	99,5%	99,8%	99,7%	98,4%
27723	80	60	20	50	98,5%	99,5%	99,8%	99,8%	98,5%
27724	80	60	20	55	98,5%	99,5%	99,8%	99,8%	98,6%
27725	80	60	20	60	98,5%	99,5%	99,8%	99,8%	98,7%
27726	80	60	20	65	98,6%	99,5%	99,8%	99,8%	98,8%
27727	80	60	20	70	98,6%	99,4%	99,8%	99,8%	98,9%
27728	80	60	20	75	98,6%	99,4%	99,8%	99,8%	98,9%
27729	80	60	20	80	98,6%	99,4%	99,8%	99,8%	99,0%
27730	80	60	25	20	97,9%	99,4%	99,7%	99,6%	97,7%
27731	80	60	25	25	97,9%	99,4%	99,7%	99,6%	97,8%
27732	80	60	25	30	97,9%	99,4%	99,7%	99,7%	97,9%
27733	80	60	25	35	98,0%	99,4%	99,7%	99,7%	98,1%
27734	80	60	25	40	98,0%	99,4%	99,7%	99,7%	98,2%
27735	80	60	25	45	98,0%	99,3%	99,8%	99,7%	98,3%
27736	80	60	25	50	98,1%	99,3%	99,8%	99,7%	98,4%
27737	80	60	25	55	98,1%	99,3%	99,8%	99,8%	98,5%
27738	80	60	25	60	98,1%	99,3%	99,8%	99,8%	98,6%
27739	80	60	25	65	98,2%	99,3%	99,8%	99,8%	98,7%
27740	80	60	25	70	98,2%	99,3%	99,8%	99,8%	98,8%
27741	80	60	25	75	98,2%	99,3%	99,8%	99,8%	98,9%
27742	80	60	25	80	98,3%	99,3%	99,8%	99,8%	99,0%
27743	80	60	30	20	97,3%	99,2%	99,7%	99,6%	97,6%
27744	80	60	30	25	97,3%	99,2%	99,7%	99,6%	97,7%
27745	80	60	30	30	97,4%	99,2%	99,7%	99,6%	97,9%
27746	80	60	30	35	97,4%	99,2%	99,7%	99,7%	98,0%
27747	80	60	30	40	97,5%	99,2%	99,7%	99,7%	98,1%
27748	80	60	30	45	97,5%	99,2%	99,7%	99,7%	98,3%
27749	80	60	30	50	97,5%	99,2%	99,7%	99,7%	98,4%
27750	80	60	30	55	97,6%	99,2%	99,7%	99,7%	98,5%
27751	80	60	30	60	97,6%	99,1%	99,7%	99,8%	98,6%
27752	80	60	30	65	97,7%	99,1%	99,8%	99,8%	98,7%

27753	80	60	30	70	97,7%	99,1%	99,8%	99,8%	98,8%
27754	80	60	30	75	97,8%	99,1%	99,8%	99,8%	98,8%
27755	80	60	30	80	97,8%	99,1%	99,8%	99,8%	98,9%
27756	80	60	35	20	96,5%	99,0%	99,6%	99,6%	97,5%
27757	80	60	35	25	96,6%	99,0%	99,6%	99,6%	97,6%
27758	80	60	35	30	96,7%	99,0%	99,7%	99,6%	97,8%
27759	80	60	35	35	96,7%	99,0%	99,7%	99,6%	97,9%
27760	80	60	35	40	96,8%	99,0%	99,7%	99,7%	98,1%
27761	80	60	35	45	96,8%	99,0%	99,7%	99,7%	98,2%
27762	80	60	35	50	96,9%	99,0%	99,7%	99,7%	98,3%
27763	80	60	35	55	96,9%	98,9%	99,7%	99,7%	98,4%
27764	80	60	35	60	97,0%	98,9%	99,7%	99,7%	98,5%
27765	80	60	35	65	97,0%	98,9%	99,7%	99,8%	98,6%
27766	80	60	35	70	97,1%	98,9%	99,7%	99,8%	98,7%
27767	80	60	35	75	97,1%	98,9%	99,7%	99,8%	98,8%
27768	80	60	35	80	97,2%	98,9%	99,8%	99,8%	98,9%
27769	80	60	40	20	95,6%	98,8%	99,6%	99,5%	97,4%
27770	80	60	40	25	95,7%	98,8%	99,6%	99,6%	97,5%
27771	80	60	40	30	95,8%	98,7%	99,6%	99,6%	97,7%
27772	80	60	40	35	95,8%	98,7%	99,6%	99,6%	97,8%
27773	80	60	40	40	95,9%	98,7%	99,6%	99,6%	98,0%
27774	80	60	40	45	96,0%	98,7%	99,6%	99,7%	98,1%
27775	80	60	40	50	96,0%	98,7%	99,7%	99,7%	98,2%
27776	80	60	40	55	96,1%	98,7%	99,7%	99,7%	98,3%
27777	80	60	40	60	96,2%	98,6%	99,7%	99,7%	98,5%
27778	80	60	40	65	96,2%	98,6%	99,7%	99,7%	98,6%
27779	80	60	40	70	96,3%	98,6%	99,7%	99,8%	98,6%
27780	80	60	40	75	96,4%	98,6%	99,7%	99,8%	98,7%
27781	80	60	40	80	96,4%	98,6%	99,7%	99,8%	98,8%
27782	80	60	45	20	94,5%	98,5%	99,5%	99,5%	97,2%
27783	80	60	45	25	94,6%	98,5%	99,5%	99,5%	97,4%
27784	80	60	45	30	94,6%	98,4%	99,6%	99,6%	97,6%
27785	80	60	45	35	94,7%	98,4%	99,6%	99,6%	97,7%

27786	80	60	45	40	94,8%	98,4%	99,6%	99,6%	97,9%
27787	80	60	45	45	94,9%	98,4%	99,6%	99,6%	98,0%
27788	80	60	45	50	95,0%	98,3%	99,6%	99,7%	98,2%
27789	80	60	45	55	95,1%	98,3%	99,6%	99,7%	98,3%
27790	80	60	45	60	95,2%	98,3%	99,6%	99,7%	98,4%
27791	80	60	45	65	95,2%	98,3%	99,6%	99,7%	98,5%
27792	80	60	45	70	95,3%	98,3%	99,7%	99,7%	98,6%
27793	80	60	45	75	95,4%	98,2%	99,7%	99,8%	98,7%
27794	80	60	45	80	95,5%	98,2%	99,7%	99,8%	98,8%
27795	80	60	50	20	93,0%	98,1%	99,5%	99,5%	97,1%
27796	80	60	50	25	93,1%	98,1%	99,5%	99,5%	97,3%
27797	80	60	50	30	93,2%	98,0%	99,5%	99,5%	97,5%
27798	80	60	50	35	93,4%	98,0%	99,5%	99,6%	97,7%
27799	80	60	50	40	93,5%	98,0%	99,5%	99,6%	97,8%
27800	80	60	50	45	93,6%	98,0%	99,5%	99,6%	97,9%
27801	80	60	50	50	93,7%	97,9%	99,6%	99,6%	98,1%
27802	80	60	50	55	93,8%	97,9%	99,6%	99,7%	98,2%
27803	80	60	50	60	93,9%	97,9%	99,6%	99,7%	98,3%
27804	80	60	50	65	94,0%	97,8%	99,6%	99,7%	98,4%
27805	80	60	50	70	94,1%	97,8%	99,6%	99,7%	98,5%
27806	80	60	50	75	94,2%	97,8%	99,6%	99,7%	98,6%
27807	80	60	50	80	94,3%	97,8%	99,6%	99,8%	98,7%
27808	80	60	55	20	91,2%	97,6%	99,4%	99,4%	97,0%
27809	80	60	55	25	91,4%	97,6%	99,4%	99,5%	97,2%
27810	80	60	55	30	91,5%	97,5%	99,4%	99,5%	97,4%
27811	80	60	55	35	91,7%	97,5%	99,4%	99,5%	97,6%
27812	80	60	55	40	91,8%	97,5%	99,5%	99,6%	97,7%
27813	80	60	55	45	91,9%	97,4%	99,5%	99,6%	97,9%
27814	80	60	55	50	92,0%	97,4%	99,5%	99,6%	98,0%
27815	80	60	55	55	92,2%	97,4%	99,5%	99,6%	98,1%
27816	80	60	55	60	92,3%	97,3%	99,5%	99,7%	98,3%
27817	80	60	55	65	92,4%	97,3%	99,5%	99,7%	98,4%
27818	80	60	55	70	92,5%	97,3%	99,6%	99,7%	98,5%

27819	80	60	55	75	92,7%	97,2%	99,6%	99,7%	98,6%
27820	80	60	55	80	92,8%	97,2%	99,6%	99,7%	98,7%
27821	80	60	60	20	89,1%	97,0%	99,3%	99,4%	96,9%
27822	80	60	60	25	89,2%	96,9%	99,3%	99,4%	97,1%
27823	80	60	60	30	89,4%	96,9%	99,4%	99,5%	97,3%
27824	80	60	60	35	89,6%	96,9%	99,4%	99,5%	97,5%
27825	80	60	60	40	89,7%	96,8%	99,4%	99,5%	97,6%
27826	80	60	60	45	89,9%	96,8%	99,4%	99,6%	97,8%
27827	80	60	60	50	90,0%	96,7%	99,4%	99,6%	97,9%
27828	80	60	60	55	90,2%	96,7%	99,5%	99,6%	98,1%
27829	80	60	60	60	90,4%	96,7%	99,5%	99,6%	98,2%
27830	80	60	60	65	90,5%	96,6%	99,5%	99,7%	98,3%
27831	80	60	60	70	90,7%	96,6%	99,5%	99,7%	98,4%
27832	80	60	60	75	90,8%	96,5%	99,5%	99,7%	98,5%
27833	80	60	60	80	91,0%	96,5%	99,5%	99,7%	98,6%
27834	80	60	65	20	86,4%	96,2%	99,2%	99,4%	96,8%
27835	80	60	65	25	86,6%	96,2%	99,2%	99,4%	97,0%
27836	80	60	65	30	86,8%	96,1%	99,3%	99,4%	97,2%
27837	80	60	65	35	87,0%	96,1%	99,3%	99,5%	97,4%
27838	80	60	65	40	87,2%	96,0%	99,3%	99,5%	97,5%
27839	80	60	65	45	87,4%	96,0%	99,3%	99,5%	97,7%
27840	80	60	65	50	87,6%	95,9%	99,4%	99,6%	97,8%
27841	80	60	65	55	87,8%	95,9%	99,4%	99,6%	98,0%
27842	80	60	65	60	88,0%	95,8%	99,4%	99,6%	98,1%
27843	80	60	65	65	88,2%	95,8%	99,4%	99,7%	98,2%
27844	80	60	65	70	88,3%	95,7%	99,4%	99,7%	98,3%
27845	80	60	65	75	88,5%	95,7%	99,5%	99,7%	98,5%
27846	80	60	65	80	88,7%	95,6%	99,5%	99,7%	98,6%
27847	80	60	70	20	83,2%	95,3%	99,1%	99,3%	96,6%
27848	80	60	70	25	83,5%	95,2%	99,1%	99,4%	96,9%
27849	80	60	70	30	83,7%	95,2%	99,2%	99,4%	97,1%
27850	80	60	70	35	84,0%	95,1%	99,2%	99,4%	97,2%
27851	80	60	70	40	84,2%	95,1%	99,2%	99,5%	97,4%

27852	80	60	70	45	84,4%	95,0%	99,3%	99,5%	97,6%
27853	80	60	70	50	84,7%	94,9%	99,3%	99,5%	97,7%
27854	80	60	70	55	84,9%	94,9%	99,3%	99,6%	97,9%
27855	80	60	70	60	85,1%	94,8%	99,3%	99,6%	98,0%
27856	80	60	70	65	85,3%	94,7%	99,3%	99,6%	98,2%
27857	80	60	70	70	85,6%	94,7%	99,4%	99,7%	98,3%
27858	80	60	70	75	85,8%	94,6%	99,4%	99,7%	98,4%
27859	80	60	70	80	86,0%	94,5%	99,4%	99,7%	98,5%
27860	80	60	75	20	79,5%	94,2%	99,0%	99,3%	96,5%
27861	80	60	75	25	79,8%	94,1%	99,0%	99,3%	96,7%
27862	80	60	75	30	80,1%	94,0%	99,1%	99,4%	96,9%
27863	80	60	75	35	80,4%	93,9%	99,1%	99,4%	97,1%
27864	80	60	75	40	80,6%	93,9%	99,1%	99,4%	97,3%
27865	80	60	75	45	80,9%	93,8%	99,2%	99,5%	97,5%
27866	80	60	75	50	81,2%	93,7%	99,2%	99,5%	97,7%
27867	80	60	75	55	81,4%	93,6%	99,2%	99,5%	97,8%
27868	80	60	75	60	81,7%	93,5%	99,2%	99,6%	97,9%
27869	80	60	75	65	82,0%	93,5%	99,3%	99,6%	98,1%
27870	80	60	75	70	82,2%	93,4%	99,3%	99,6%	98,2%
27871	80	60	75	75	82,5%	93,3%	99,3%	99,7%	98,3%
27872	80	60	75	80	82,7%	93,2%	99,3%	99,7%	98,4%
27873	80	60	80	20	75,2%	92,7%	98,9%	99,2%	96,4%
27874	80	60	80	25	75,5%	92,7%	98,9%	99,3%	96,6%
27875	80	60	80	30	75,8%	92,6%	98,9%	99,3%	96,8%
27876	80	60	80	35	76,2%	92,5%	99,0%	99,4%	97,0%
27877	80	60	80	40	76,5%	92,4%	99,0%	99,4%	97,2%
27878	80	60	80	45	76,8%	92,3%	99,0%	99,4%	97,4%
27879	80	60	80	50	77,1%	92,2%	99,1%	99,5%	97,6%
27880	80	60	80	55	77,4%	92,1%	99,1%	99,5%	97,7%
27881	80	60	80	60	77,7%	92,0%	99,1%	99,6%	97,9%
27882	80	60	80	65	78,0%	91,9%	99,2%	99,6%	98,0%
27883	80	60	80	70	78,3%	91,8%	99,2%	99,6%	98,1%
27884	80	60	80	75	78,6%	91,7%	99,2%	99,6%	98,3%

27885	80	60	80	80	78,9%	91,6%	99,2%	99,7%	98,4%
27886	80	65	20	20	98,5%	99,5%	99,7%	99,6%	97,5%
27887	80	65	20	25	98,5%	99,5%	99,7%	99,6%	97,7%
27888	80	65	20	30	98,5%	99,5%	99,8%	99,6%	97,8%
27889	80	65	20	35	98,5%	99,5%	99,8%	99,7%	98,0%
27890	80	65	20	40	98,6%	99,5%	99,8%	99,7%	98,1%
27891	80	65	20	45	98,6%	99,5%	99,8%	99,7%	98,2%
27892	80	65	20	50	98,6%	99,5%	99,8%	99,7%	98,3%
27893	80	65	20	55	98,6%	99,5%	99,8%	99,8%	98,5%
27894	80	65	20	60	98,7%	99,5%	99,8%	99,8%	98,6%
27895	80	65	20	65	98,7%	99,5%	99,8%	99,8%	98,6%
27896	80	65	20	70	98,7%	99,5%	99,8%	99,8%	98,7%
27897	80	65	20	75	98,7%	99,5%	99,8%	99,8%	98,8%
27898	80	65	20	80	98,8%	99,5%	99,8%	99,8%	98,9%
27899	80	65	25	20	98,0%	99,4%	99,7%	99,6%	97,4%
27900	80	65	25	25	98,1%	99,4%	99,7%	99,6%	97,6%
27901	80	65	25	30	98,1%	99,4%	99,7%	99,6%	97,7%
27902	80	65	25	35	98,1%	99,4%	99,7%	99,7%	97,9%
27903	80	65	25	40	98,2%	99,4%	99,7%	99,7%	98,0%
27904	80	65	25	45	98,2%	99,4%	99,7%	99,7%	98,2%
27905	80	65	25	50	98,2%	99,4%	99,8%	99,7%	98,3%
27906	80	65	25	55	98,3%	99,4%	99,8%	99,7%	98,4%
27907	80	65	25	60	98,3%	99,4%	99,8%	99,8%	98,5%
27908	80	65	25	65	98,3%	99,4%	99,8%	99,8%	98,6%
27909	80	65	25	70	98,4%	99,4%	99,8%	99,8%	98,7%
27910	80	65	25	75	98,4%	99,3%	99,8%	99,8%	98,8%
27911	80	65	25	80	98,4%	99,3%	99,8%	99,8%	98,9%
27912	80	65	30	20	97,5%	99,3%	99,7%	99,5%	97,3%
27913	80	65	30	25	97,5%	99,3%	99,7%	99,6%	97,5%
27914	80	65	30	30	97,6%	99,3%	99,7%	99,6%	97,7%
27915	80	65	30	35	97,6%	99,3%	99,7%	99,6%	97,8%
27916	80	65	30	40	97,7%	99,2%	99,7%	99,7%	97,9%
27917	80	65	30	45	97,7%	99,2%	99,7%	99,7%	98,1%

27918	80	65	30	50	97,8%	99,2%	99,7%	99,7%	98,2%
27919	80	65	30	55	97,8%	99,2%	99,7%	99,7%	98,3%
27920	80	65	30	60	97,8%	99,2%	99,7%	99,7%	98,4%
27921	80	65	30	65	97,9%	99,2%	99,7%	99,8%	98,5%
27922	80	65	30	70	97,9%	99,2%	99,8%	99,8%	98,6%
27923	80	65	30	75	97,9%	99,2%	99,8%	99,8%	98,7%
27924	80	65	30	80	98,0%	99,2%	99,8%	99,8%	98,8%
27925	80	65	35	20	96,8%	99,1%	99,6%	99,5%	97,2%
27926	80	65	35	25	96,9%	99,1%	99,6%	99,5%	97,4%
27927	80	65	35	30	96,9%	99,1%	99,6%	99,6%	97,6%
27928	80	65	35	35	97,0%	99,1%	99,7%	99,6%	97,7%
27929	80	65	35	40	97,0%	99,1%	99,7%	99,6%	97,9%
27930	80	65	35	45	97,1%	99,0%	99,7%	99,7%	98,0%
27931	80	65	35	50	97,1%	99,0%	99,7%	99,7%	98,1%
27932	80	65	35	55	97,2%	99,0%	99,7%	99,7%	98,3%
27933	80	65	35	60	97,2%	99,0%	99,7%	99,7%	98,4%
27934	80	65	35	65	97,3%	99,0%	99,7%	99,7%	98,5%
27935	80	65	35	70	97,3%	99,0%	99,7%	99,8%	98,6%
27936	80	65	35	75	97,4%	99,0%	99,7%	99,8%	98,7%
27937	80	65	35	80	97,4%	99,0%	99,7%	99,8%	98,8%
27938	80	65	40	20	96,0%	98,9%	99,6%	99,5%	97,1%
27939	80	65	40	25	96,0%	98,9%	99,6%	99,5%	97,3%
27940	80	65	40	30	96,1%	98,8%	99,6%	99,6%	97,5%
27941	80	65	40	35	96,2%	98,8%	99,6%	99,6%	97,6%
27942	80	65	40	40	96,2%	98,8%	99,6%	99,6%	97,8%
27943	80	65	40	45	96,3%	98,8%	99,6%	99,6%	97,9%
[truncated: 44,495 more chars]
