# Supplementary figures and images for: Associations between the probabilities of frequency-specific hearing loss and unaided APHAB scores
Source: Eur Arch Otorhinolaryngol. 2016 Nov 17;274(3):1345–9. doi: 10.1007/s00405-016-4385-7 (PMC5309283; doi:10.1007/s00405-016-4385-7)

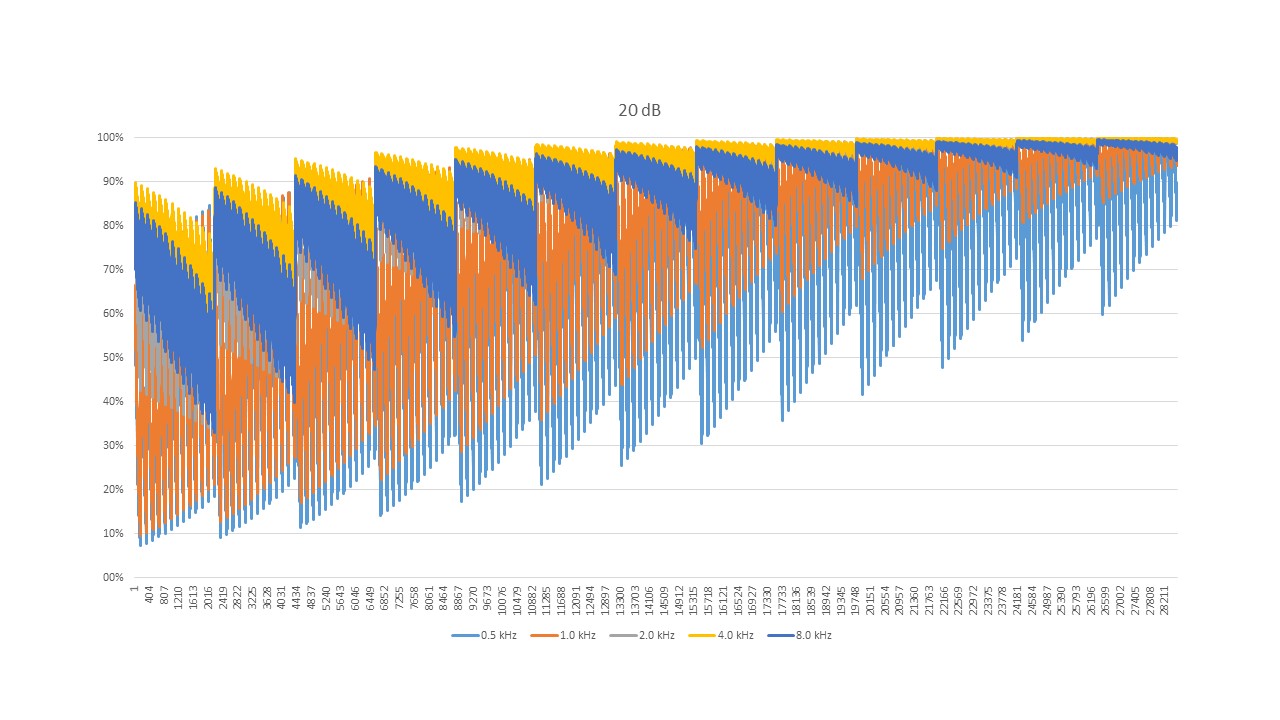

Supplement: Supplementary file 13 — Supplementary material 13 (JPEG 149 kb) [file 405_2016_4385_MOESM13_ESM.jpg]

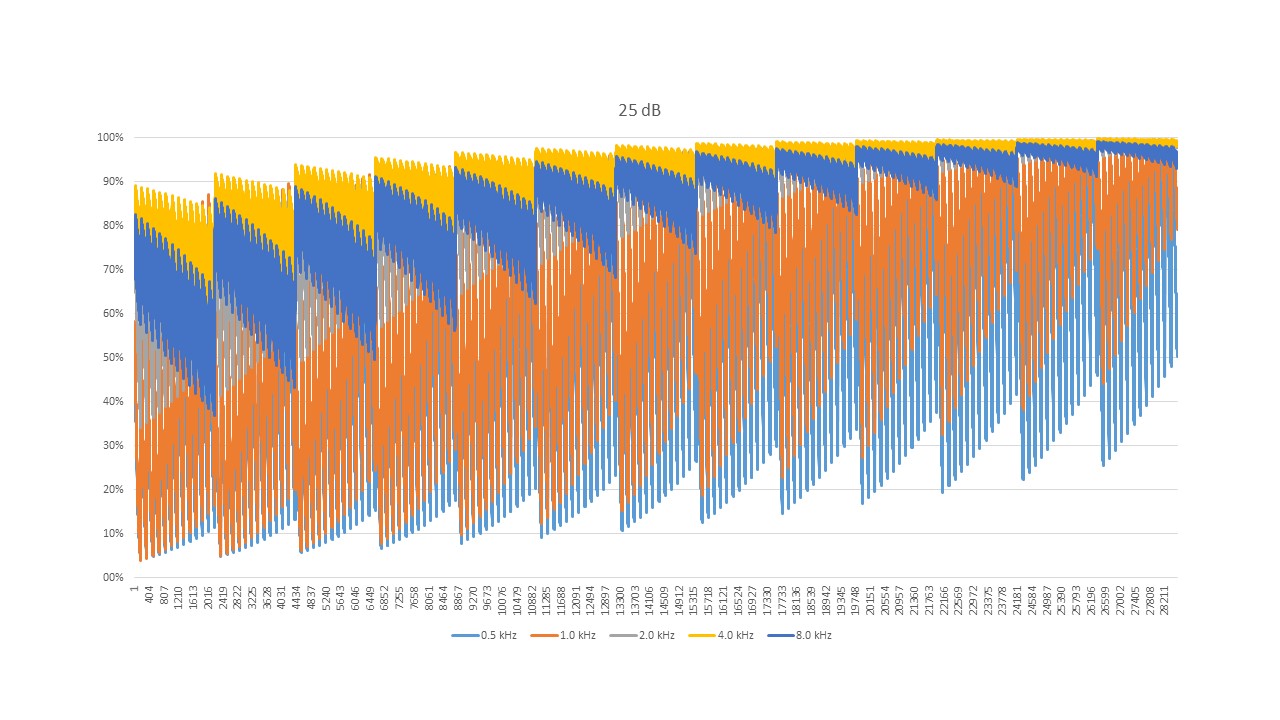

Supplement: Supplementary file 14 — Supplementary material 14 (JPEG 170 kb) [file 405_2016_4385_MOESM14_ESM.jpg]

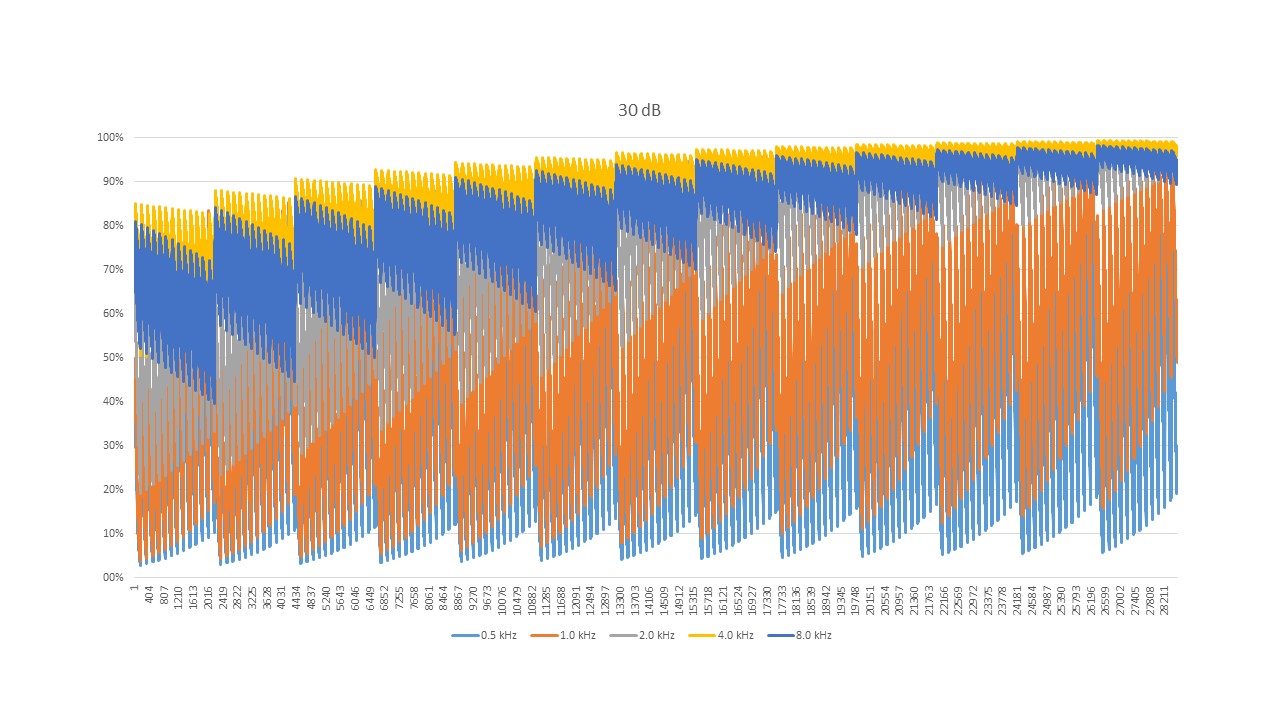

Supplement: Supplementary file 15 — Supplementary material 15 (JPEG 179 kb) [file 405_2016_4385_MOESM15_ESM.jpg]

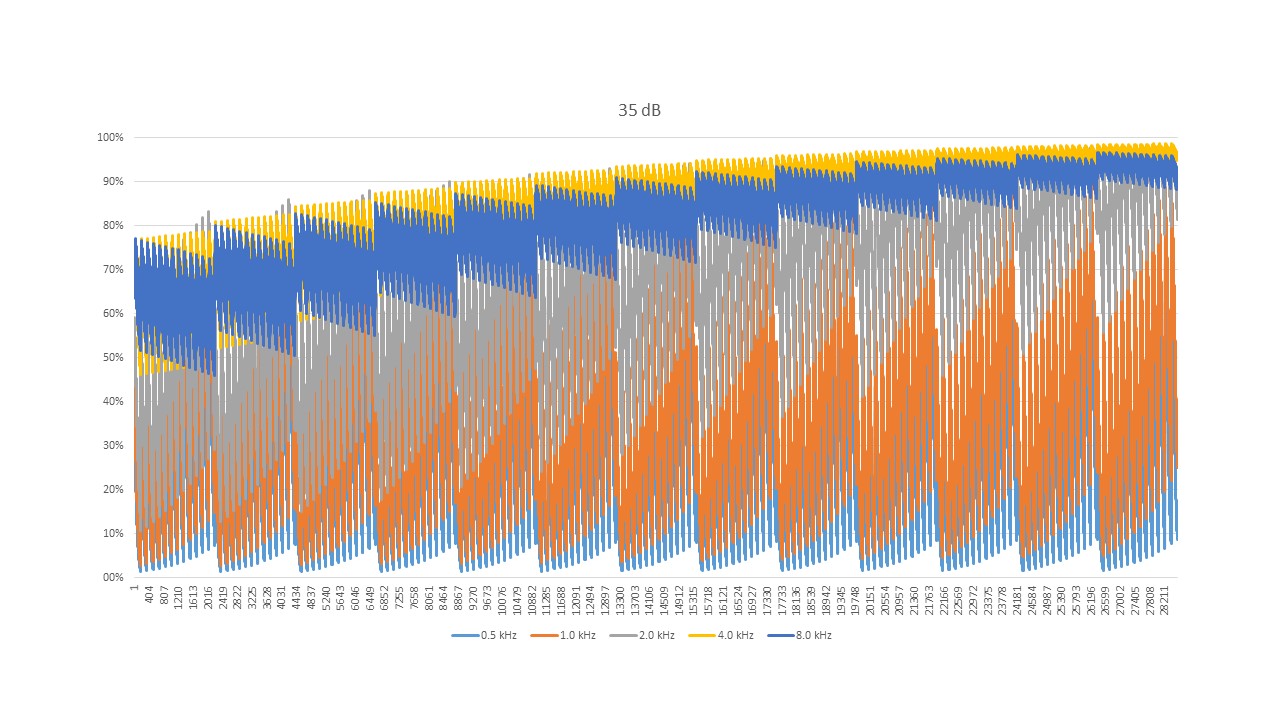

Supplement: Supplementary file 16 — Supplementary material 16 (JPEG 186 kb) [file 405_2016_4385_MOESM16_ESM.jpg]

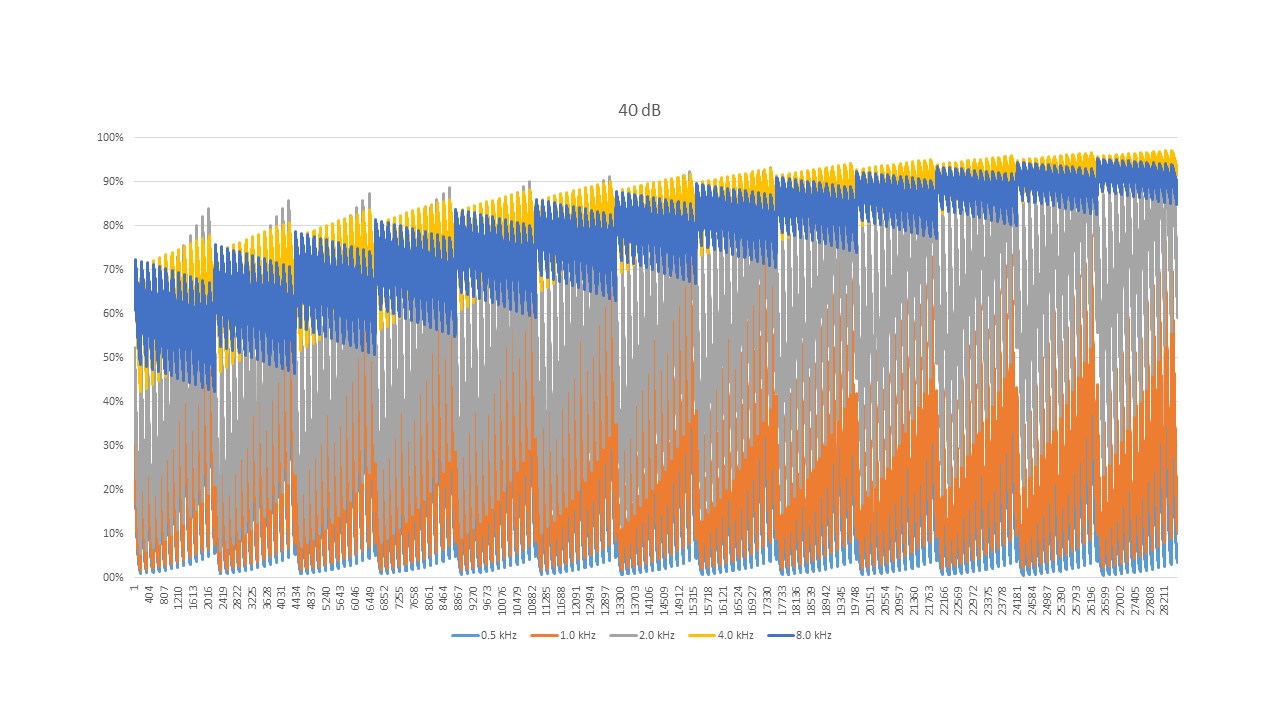

Supplement: Supplementary file 17 — Supplementary material 17 (JPEG 177 kb) [file 405_2016_4385_MOESM17_ESM.jpg]

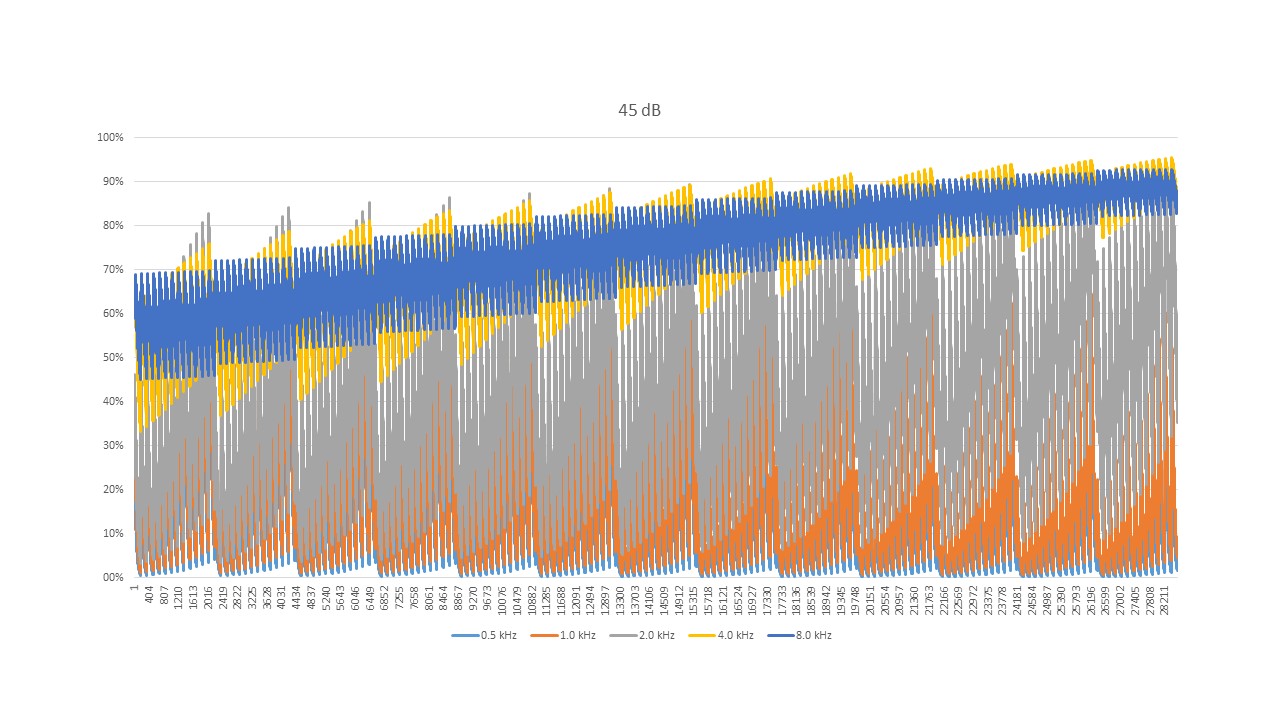

Supplement: Supplementary file 18 — Supplementary material 18 (JPEG 174 kb) [file 405_2016_4385_MOESM18_ESM.jpg]

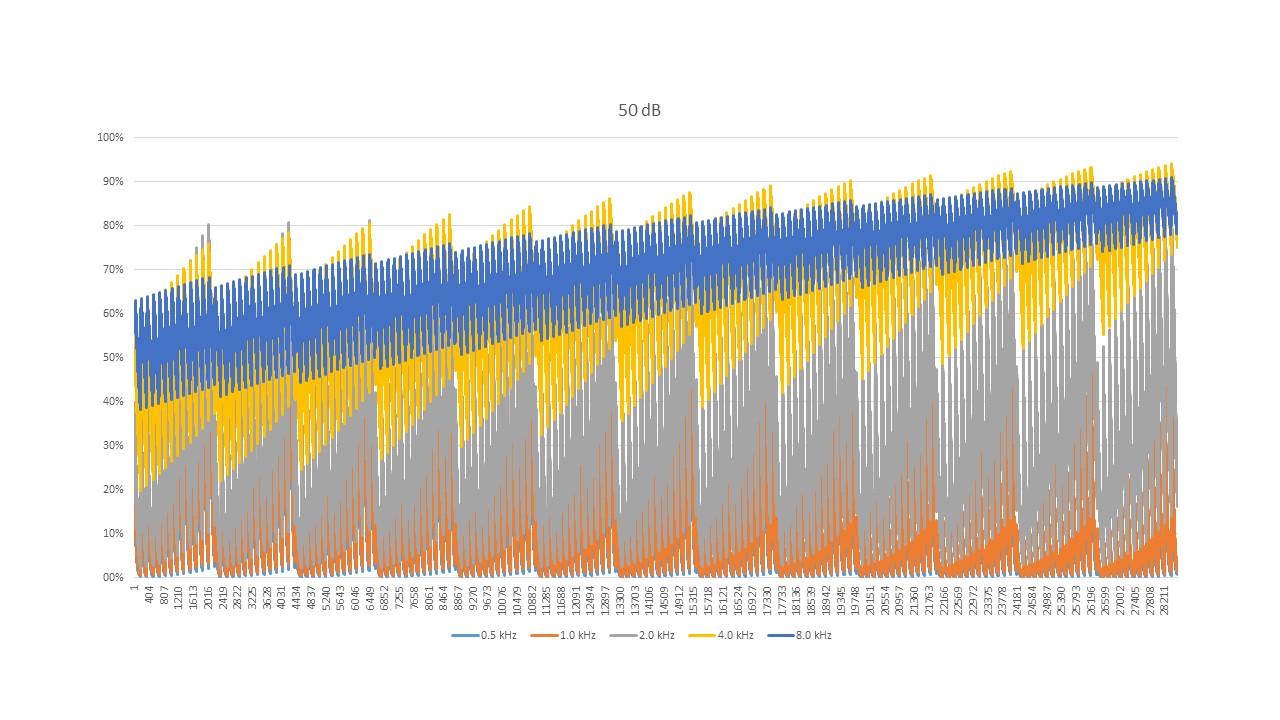

Supplement: Supplementary file 19 — Supplementary material 19 (JPEG 179 kb) [file 405_2016_4385_MOESM19_ESM.jpg]

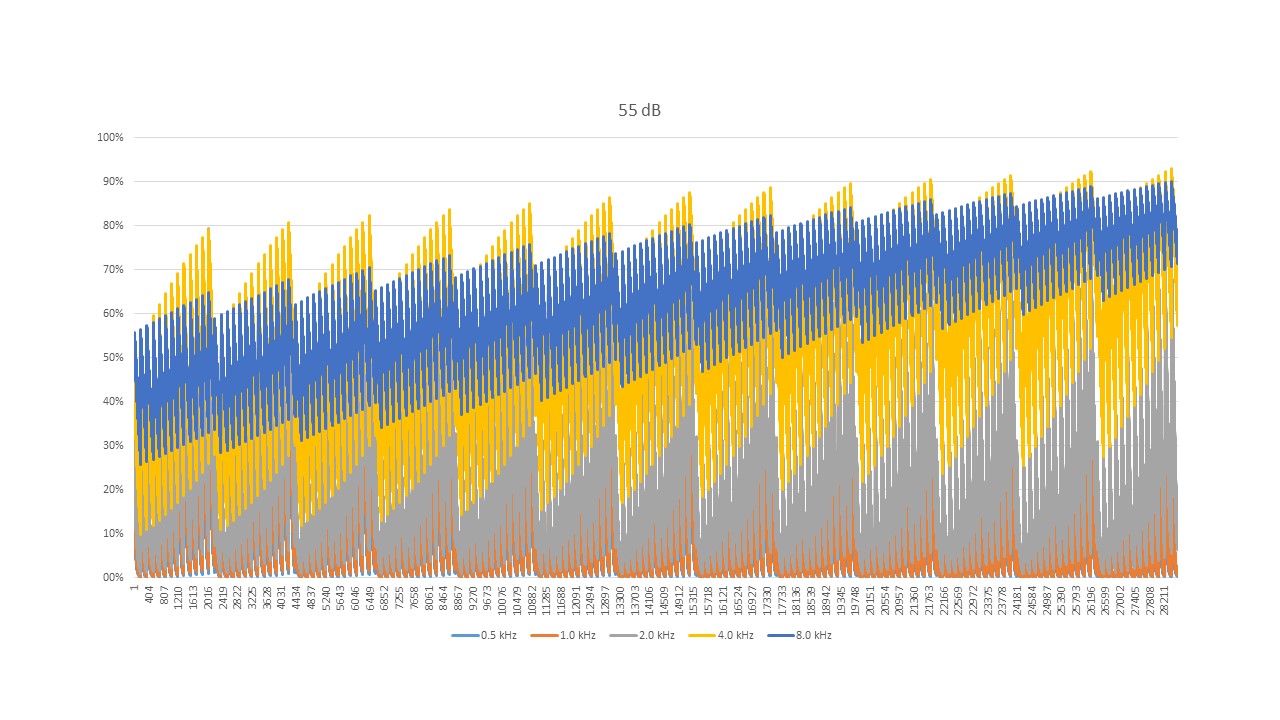

Supplement: Supplementary file 20 — Supplementary material 20 (JPEG 179 kb) [file 405_2016_4385_MOESM20_ESM.jpg]

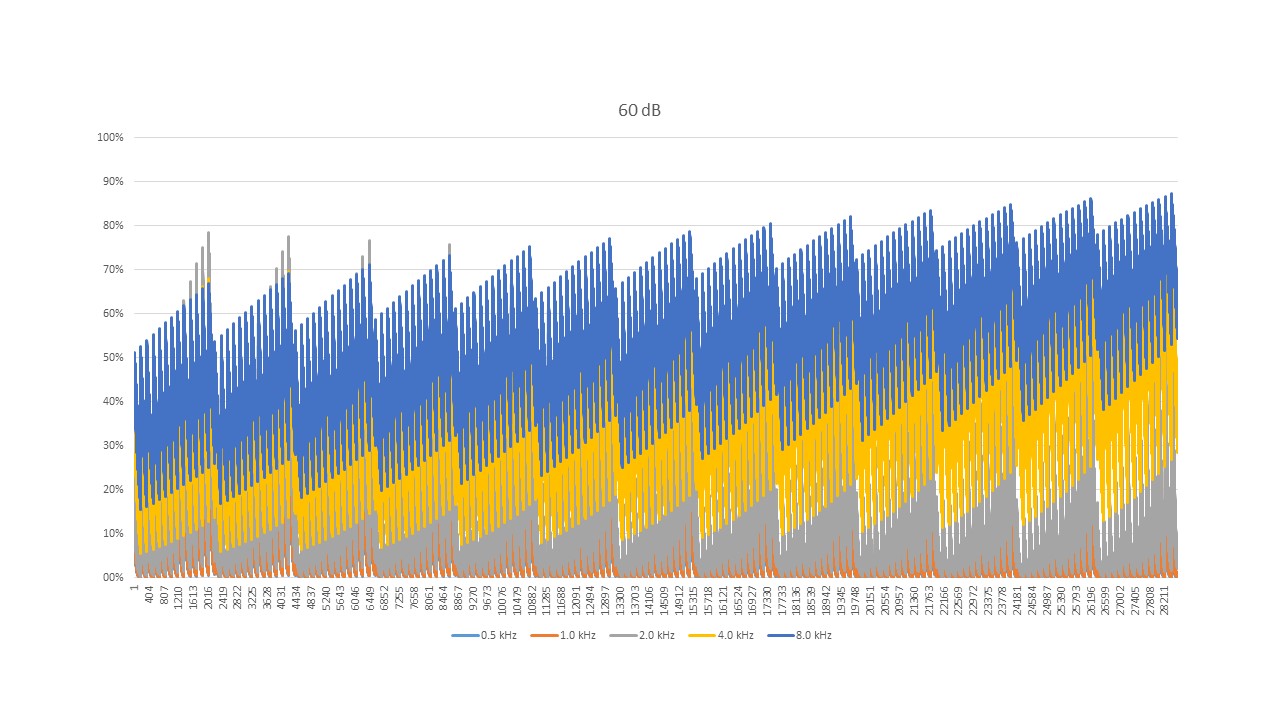

Supplement: Supplementary file 21 — Supplementary material 21 (JPEG 162 kb) [file 405_2016_4385_MOESM21_ESM.jpg]

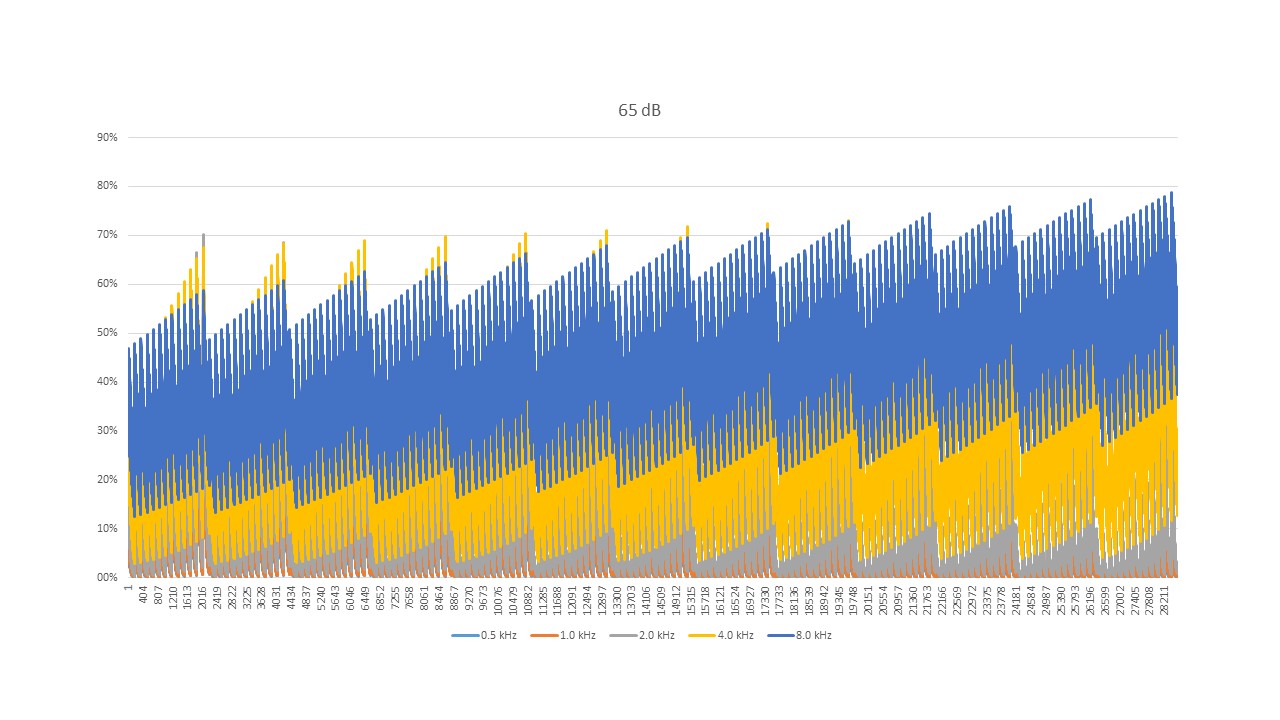

Supplement: Supplementary file 22 — Supplementary material 22 (JPEG 150 kb) [file 405_2016_4385_MOESM22_ESM.jpg]

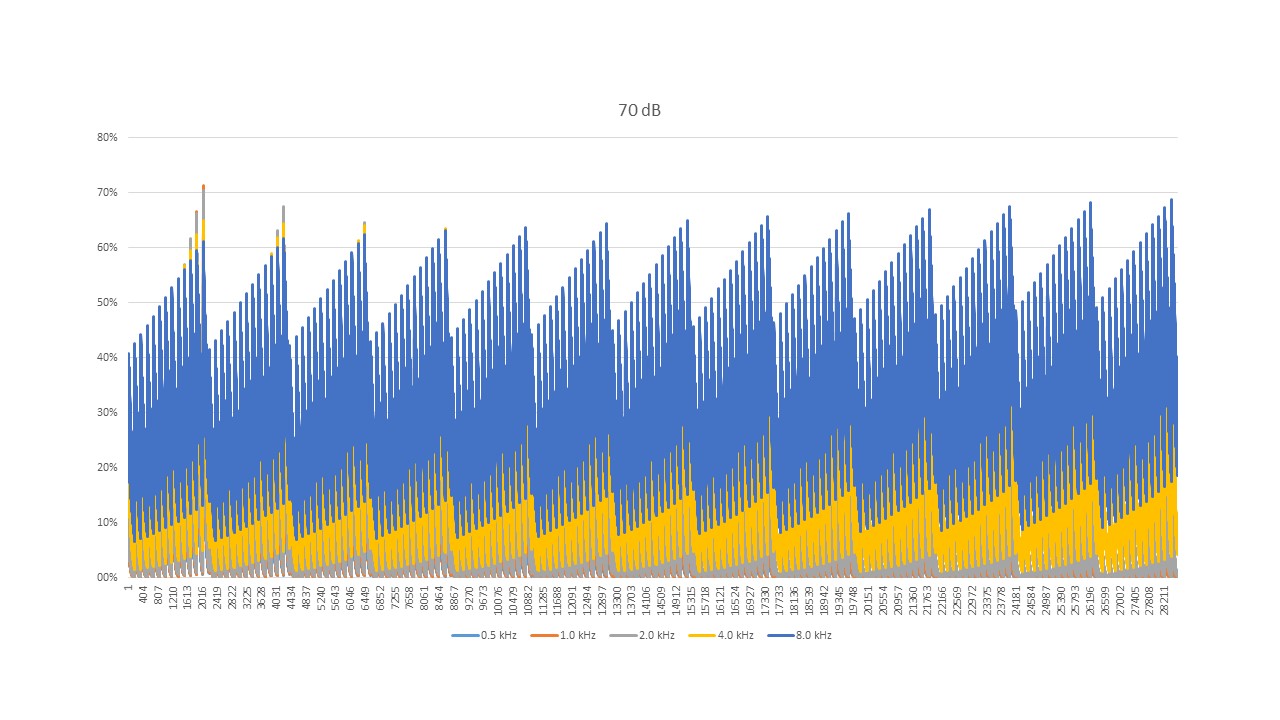

Supplement: Supplementary file 23 — Supplementary material 23 (JPEG 149 kb) [file 405_2016_4385_MOESM23_ESM.jpg]

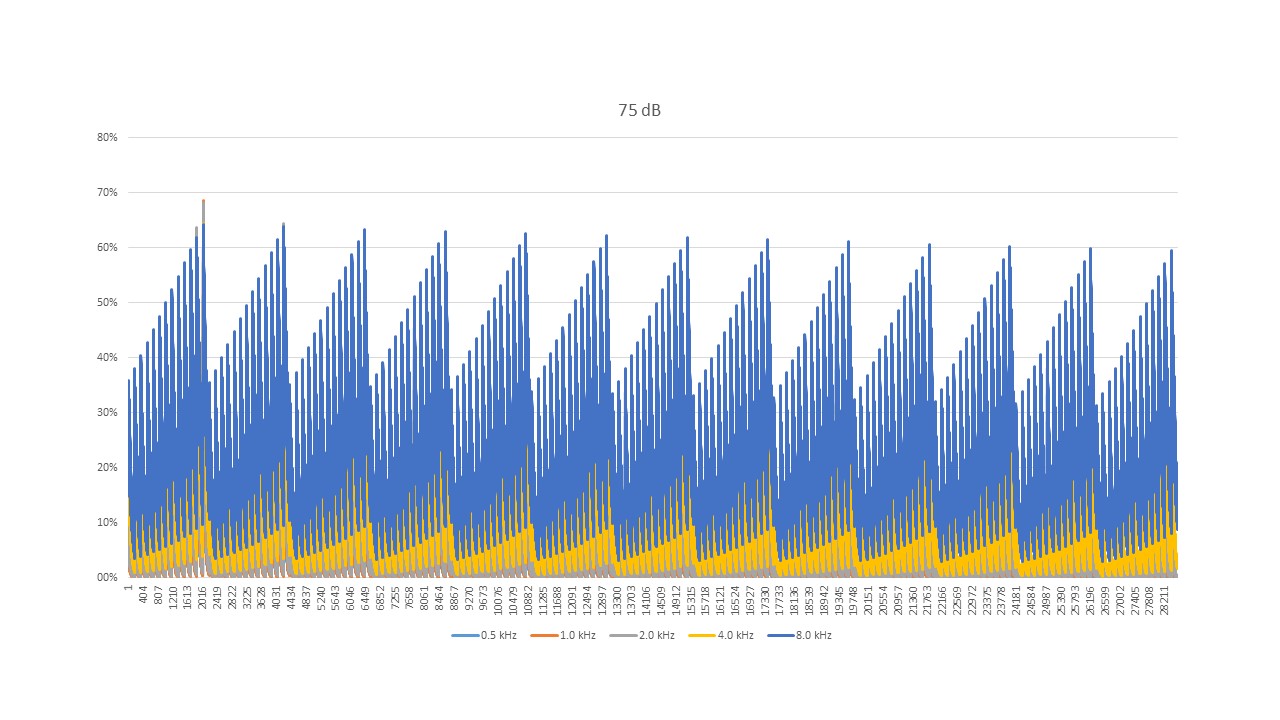

Supplement: Supplementary file 24 — Supplementary material 24 (JPEG 149 kb) [file 405_2016_4385_MOESM24_ESM.jpg]
